# A Bibliometric Analysis of Scientific Publications on Eating Disorder Prevention in the Past Three Decades

**DOI:** 10.3390/nu16081111

**Published:** 2024-04-10

**Authors:** Zhenxin Liao, Martina Scaltritti, Zhihan Xu, Thu Ngoc Xuan Dinh, Jiahe Chen, Ata Ghaderi

**Affiliations:** 1 Department of Medical Epidemiology and Biostatistics, Karolinska Institutet, Nobels vag 12A, 17177 Stockholm, Sweden; zhenxin.liao@stud.ki.se; 2 Department of General Psychology, University of Padua, Via Venezia, 12, 35131 Padua, Italy; martina.scaltritti@studenti.unipd.it; 3 Division of Network and Systems Engineering (NSE), KTH School of Electrical Engineering and Computer Science, Teknikringen 33, 10044 Stockholm, Sweden; zhihanx@kth.se; 4 Department of Learning, Informatics, Management and Ethics (LIME), Karolinska Institute, Nobels vag 6, 17177 Stockholm, Sweden; ngoc.xuan.thu.dinh@stud.ki.se (T.N.X.D.); jiahe.chen@stud.ki.se (J.C.); 5 Division of Psychology, Department of Clinical Neuroscience, Karolinska Institutet, Nobels vag 9, 17177 Stockholm, Sweden

**Keywords:** prevention, eating disorders, bibliometric analysis

## Abstract

Background: Eating disorders (EDs) present a growing concern due to their widespread occurrence and chronic course, the low access to evidence-based treatment, and the significant burden they place on the patients and society. This picture justifies intensive focus on the prevention of EDs. The current study provides the first bibliometric analysis of research on the prevention of EDs, focusing on trends and contributions, to prompt further prevention research. Methods: We conducted a bibliometric analysis of publications on the prevention of EDs using the Web of Science database, from 1993 to 2023. Focusing on universal and selective prevention strategies, our study involved a rigorous selection process, narrowing down from 10,546 to 383 relevant papers through manual screening. The analysis utilized the “bibliometrix” R package (version 4.2.2) and Python (version 3.9.6) for data processing, with VOSviewer employed for mapping collaboration networks. Results: Our analysis revealed a consistent annual growth rate of 10.85% in ED prevention research publications, with significant contributions from the “International Journal of Eating Disorders” and some notable authors. The United States emerged as the dominant contributor. The analysis also highlighted key trends, including a surge in publications between 2010 and 2017, and the role of major institutions in advancing research in this field. Discussion: The increasing rate of publications on the prevention of EDs is encouraging. However, the actual number of studies on the prevention of EDs are limited, and the majority of this work is performed by a few research groups. Given the high concentration of publications within a few countries and research groups, increased funding, facilitation of prevention research on a wider scale, and engagement of more researchers and further collaboration are called for.

## 1. Background

Eating disorders (EDs) are prevalent, complex conditions that cause significant suffering and are difficult and costly to treat [1]. In rigorously conducted community-based studies, estimates of the combined lifetime prevalence of all DSM-5 EDs among adolescent and young adult women ranged from 5.5 to 17.9% [2]. Globally, the prevalence of EDs has increased by 25% [3], and the economic burden of EDs is substantial [4]. Another important fact is that the majority of patients with EDs do not seek professional help; only about 20% of those afflicted present for treatment [3], and of those who seek such treatment, only a minority receive evidence-based treatment [5,6]. One potential way to lower the prevalence of EDs and the suffering caused by these conditions and to reduce the costs to society is prevention [7,8,9].

Preventive interventions within the mental health field target risk factors and/or promote protective factors among individuals with various levels of risk [10]. Such interventions could target a whole population regardless of individual risk (universal prevention), a subpopulation known to be at increased risk of mental disorders (selective prevention), or individuals already showing subthreshold clinical manifestations (indicated prevention) [10].

The present study focuses on universal and selective prevention. Because indicated prevention often involves aspects of treatment, it is often challenging to distinguish between preventative measures and treatments. Therefore, the present study focuses on universal and selective prevention. 

Despite the potential of prevention efforts to make a significant contribution through the reduction in onset of EDs [11,12], or at least a significant reduction in risk factors for EDs [13,14,15,16], research on ED prevention is limited. One way of stimulating such research is to provide a bibliometric study of prevention trials [17]. To our knowledge, although there are a few bibliometric studies on EDs [18,19], their trends [18], and binge eating [20], there are no specific bibliometric analyses of the prevention of EDs. 

Thus, we conducted a bibliometric analysis to obtain a meta-perspective on publication rates, the trends related to ED prevention, and the framework of collaboration among authors. More specifically, we used a citation analysis to evaluate research performance [21], to highlight productive authorship, and to map the co-occurrence of institutes, countries, and authors with potential to significantly shape the ED prevention research landscape [22]. Furthermore, by highlighting patterns of collaboration and networking among experts, a bibliometric analysis can facilitate more effective partnerships, ultimately leading to research with a more substantial impact [22].

## 2. Methods

### 2.1. Study Design

This study presents a bibliometric analysis focused on the universal and selective prevention of EDs. A comparison was also made between manually screened and automatically screened sources to investigate potential differences in outcome.

### 2.2. Bibliometric Search Strategy

On 13 April 2023, we performed a search using the Web of Science (WoS) Core Collection database, employing the following search strategy:

(((((((((((TS = (eating disorder)) OR TS = (bulimia)) OR TS = (anorexia)) OR TS = (binge eating)) OR TS = (disordered eating)) OR TS = (unhealthy eating)) OR TS = (unhealthy diet)) OR TS = (compulsive eating)) OR TS = (problematic eating)) AND ((((TS = (prevent*)) OR TS = (onset)) OR TS = (protective factor)) OR TS = (incidence)))

We searched for papers published between 13 April 1993 and 16 January 2024.

### 2.3. Inclusion Criteria

In addition to original prevention research, we also included review papers with a focus on universal or selective prevention of EDs, and reviews discussing the strategies and programs (interventions) aimed at preventing EDs. 

Papers that primarily focused on the diagnosis or treatment of EDs or aimed at identifying risk factors for EDs were excluded.

### 2.4. Data Processing

A total of 10,546 abstracts were found and imported into Rayyan for review. To ensure thorough evaluation, the first and second author, as well as the fourth and fifth, teamed up in two separate pairs. The first pair reviewed the first 5256 papers, while the second reviewed the remaining 5290 papers. The initial review was conducted independently within each pair, followed by a discussion with the blind mode in Rayyan turned off to reach a consensus. In cases of persistent conflicts, the last author was consulted as the final arbiter. Following the review process, a total of 385 papers met the inclusion criteria and were included for further analysis.

A bibliometric analysis typically relies on automated methods rather than manual screening. However, thorough scrutiny of studies after a completed search usually reveals a marked number of irrelevant studies, which are difficult to exclude through the refinement of the search strategy. Such studies might cause bias to the result [23]. The bias might include topic bias, citation bias, and co-operation bias [24]. Consequently, in the present study, we have opted to manually screen all of the papers to ensure the inclusion of only the relevant literature pertaining to ED prevention. By combining the rigor of manual screening with the power of bibliometric analysis, we aim to provide a comprehensive and reliable assessment of the research landscape in ED prevention.

### 2.5. Bibliometric Analysis

The bibliometric analysis was primarily performed using R Studio (version 4.2.2) and the “bibliometrix” package [25]. Python (version 3.9.6) was used for data preprocessing, specifically to adjust the data frame for R compatibility and to calculate the total number of citations of authors, institutions, and countries. The “bibliometrix” package provided analysis of journal statistics and citation characteristics, while VOSviewer was utilized for mapping the collaboration network among authors [26].

A comprehensive analysis was conducted to examine various aspects of the data, including the sources of the papers, authorship details, affiliations, keywords, trend evaluation, and collaboration between countries. In addition, we compared the results generated from 383 manually selected papers (Supplementary File S1) versus 10,546 automatically generated papers (Supplementary File S2) to obtain an overview of the similarities and differences between the two methods. 

## 3. Results

We found a total of 383 publications from 126 distinct sources (journals and books) (Table 1). These papers span a publication timeframe from 1993 to 2023. Notably, we observed a consistent annual growth rate of 10.85% throughout this period.

Particularly striking is the period from 2010 to 2017, during which the number of published papers surged from 4 to 30 annually. Subsequently, the production rate remained remarkably stable, maintaining an annual average of around 30 papers from 2018 onwards. The composition of these papers includes 315 original studies, 50 reviews, and 9 editorial papers. 

### 3.1. Sources

**Figure 1:**
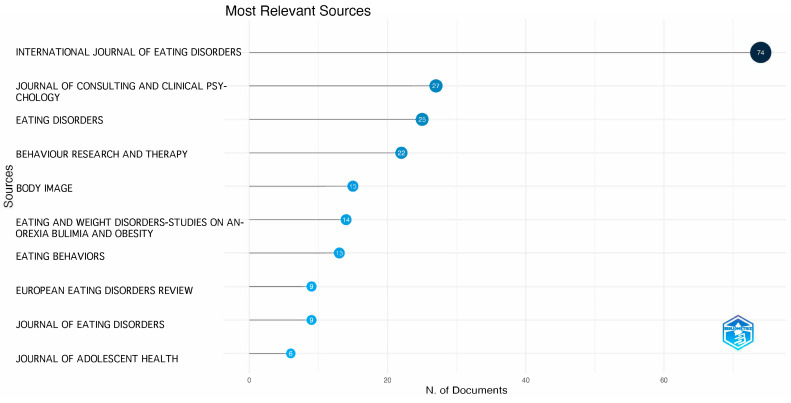
Peer-reviewed journals in their order of published research on the prevention of EDs.

A significant proportion of the papers (19.32%) have been published in the “International Journal of Eating Disorders” (Figure 1). Notably, this journal has experienced a substantial increase in its impact factor over the past decade, climbing from an initial IF of 2.877 to its current standing at 5.791. This upward trajectory underscores its growing influence in the field.

Following closely behind in terms of publication count are the “Journal of Consulting and Clinical Psychology”, with 27 papers, “Eating Disorders”, with 25 papers, and “Behaviour Research and Therapy”, with 22 papers. According to Bradford’s law [27], these four journals can be considered the core sources in the field of universal and selective eating disorder prevention.

**Figure 2:**
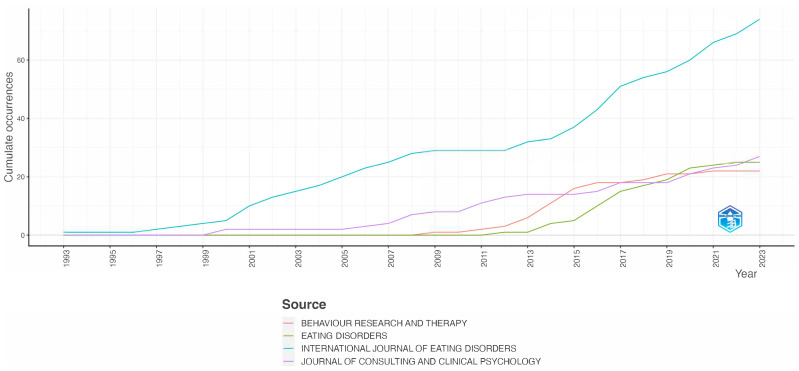
The publication trend in journals over time.

Furthermore, there is a clear upward trend in the volume of publications regarding the prevention of EDs in these journals over time (Figure 2). This trend highlights the increasing research activity and interest in the field of ED prevention.

### 3.2. Authors, Affiliations, and Countries

**Figure 3:**
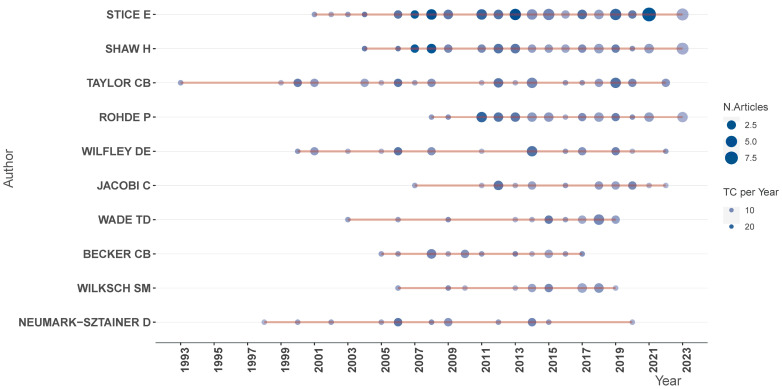
The authors’ production over time.

In our study, Eric Stice, who previously worked at the Oregon Research Institute and is currently affiliated with Stanford University, stands out as the most prolific author, with a total of 66 publications on ED prevention out of the 383 included papers in the current study. Eric Stice’s contributions to this field, with focus on dissonance-based prevention, based on the current search, dates back to 2001, and he continues to actively publish in this area (e.g., in 2021, he published nine papers (Figure 3)).

Heather Shaw, from the Oregon Research Center, has made significant contributions, with a total of 40 publications in the field of ED prevention. Her publishing journey, based on the search in the current study with a focus on prevention, began in 2004. Similarly, the late Dr. Barr Taylor, affiliated with Stanford University, has authored 35 papers of the 383 included papers.

Turning our attention to affiliations, Stanford University holds the top position, with 62 publications and 2653 total citations (Table 2), followed closely by the Oregon Research Institute, with 53 papers and 2586 citations, and Harvard University, with 32 papers and 1042 citations.

Analysis of the leading countries publishing eating disorder prevention research reveals that the United States is at the forefront (the country of the corresponding authors). It has contributed a significant number of papers in this search (*n* = 206). This figure represents 53.79% of the total number of publications in this field based on this search, thus highlighting the United States’ dominant role. These papers have garnered a total of 9313 citations, indicating their impact and relevance in the research community.

Following the United States, Australia is the second most contributing country, with 46 papers and 1896 citations. Germany also has a notable presence with 34 papers. Spain contributes 16 papers to this field, with 291 citations. Both Canada and the United Kingdom have 14 publications each.

### 3.3. Collaboration Networks

**Figure 4:**
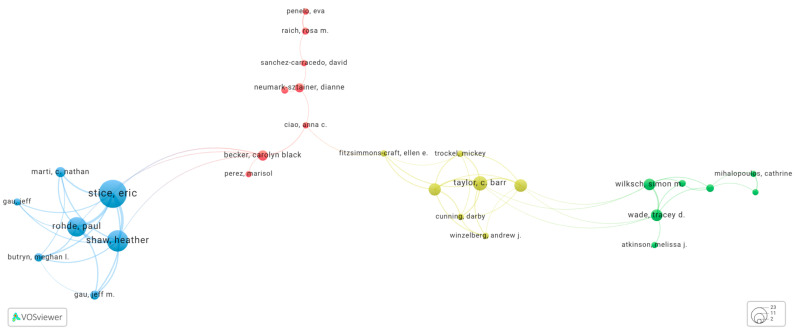
The connections and publication orders among authors (no. of papers ≥ 5) (http://tinyurl.com/24wttzxw, accessed on 2 April 2024).

Figure 4 illustrates the connections and publication orders among authors in our study. We identified 39 authors who have been involved in at least 5 or more papers. To increase readability in depicting author connections, we omitted the names of authors with no or weak connections (*n* = 10 authors). The resulting network encompasses 29 authors, grouped into 4 distinct clusters. 

One of these clusters, led by Eric Stice, primarily focuses on selected prevention strategies. They have developed a prevention project known as the “Body Project”, which is a dissonance-based body-acceptance program. This initiative aims to assist high school girls and college-aged women in resisting societal pressures to conform to unrealistic beauty standards while reducing the pursuit of unattainable body ideals. It is worth noting that the “Body Project” has garnered substantial research support and has been proven to reduce the onset of EDs [12]. 

In another cluster, represented by the late Dr. Taylor, the primary focus is on developing prevention programs for EDs and obesity in college-aged women and older adolescents. The program developed by Dr. Taylor and his group, The StudentBodies, has received good empirical support for its efficacy [28]. The third cluster, led by Simon Wilksch, is dedicated to the development of ‘Media Smart Schools,’ a school-based program aimed at reducing the risk of EDs. The fourth cluster, led by Dianne Neumark Sztainer, centers around her primary prevention program based on “Project EAT” [29], to help prevent and reduce the public health impact of weight-related problems.

Furthermore, we also identified authors who have published 10 or more papers in collaboration, resulting in two distinct groups. The first group consists of Eric Stice, Heather Shaw, Paul Rohde, C. Nathan Marti, and Carolyn Black Becker. These authors have collaborated on the development of dissonance-based interventions for ED prevention. In contrast, the second group, comprising Corinna Jacobi, Tracey D. Wade, C. Barr Taylor, Denise E. Wilfley, and Simon M. Wilksch, is primarily focused on a computer-mediated ED intervention program.

The results were re-run using all of the publications obtained through the bibliometric search, without manual screening and control of the papers. In the total pool of obtained publications, all studies that are somewhat relevant to the prevention of EDs were included. Bibliometric analyses of these studies (*n* = 10,546) are presented in Supplementary File S3. Although some trends are similar, a series of differences do appear compared to the narrow selection of studies on the prevention of EDs. As an example, while the International Journal of Eating Disorders remains the top journal in the broad search, the rest of the top journals are completely different to this journal based on the search with fewer studies. One of the most productive researchers in the field of EDs (Professor Cynthia Bulik) emerges as the second most productive author in the prevention of EDs list in the broad search given her focus on many aspects of EDs that are relevant to the prevention of EDs in the long-run, but there are few prevention trials on her list of publications. Thus, the collaboration network around Professor Bulik also becomes more salient in the broad search, as does the network of Professor Wilfley. 

## 4. Discussion

A bibliometric analysis offers extensive insights into the landscape of academic research, encompassing sources (e.g., journals), institutions, authors, countries, and collaborations. In our study, this approach not only provided a comprehensive historical perspective in the field of ED prevention but also highlighted current trends and pivotal contributors. This analysis has been instrumental in identifying key authors and journals publishing this line of research.

Our bibliometric analysis, spanning three decades, has charted a clear trajectory of growth in ED prevention research publications. This trend is exemplified by a 10.85% annual increase in publications and a fair range of contributing sources and authors. The period from 2010 to 2017 was particularly notable, with publications increasing from 4 to 30 annually. This trend reflects a heightened focus on ED prevention, possibly linked to evolving societal attitudes and academic interest in mental health. This is a very positive trend that by far surpasses the 4% compound annual growth in global publication output from 2010 to 2020. As availability of funding is a central factor in all research, including research on the prevention of EDs, potential trends in funding, particularly the occurrence of special calls for prevention research, merit investigation in relation to publication trends and productivity in this field. 

The dominance of the International Journal of Eating Disorders in this field, with a significant increase in its impact factor, underscores the journal’s pivotal role. As shown by Soh and colleagues [30], the International Journal of Eating Disorders publishes the highest number of papers on EDs compared to general psychiatry journals. However, the psychiatry journals have a higher impact factor in general and each paper on EDs in such journals receives more citations than in the specialized journals. Nevertheless, the top five journals publishing ED prevention papers are all either within the domain of psychology or are specialized journals. Interestingly, not all of the specialized ED journals belong to the top five journals publishing ED prevention research. More active support and systematic initiatives (e.g., calls for Special Issues) from these journals might help boost the number of publications. If systematic efforts are put in place to stimulate research and publications on the prevention of EDs, they should be guided by the current state of knowledge about the efficacy and effectiveness of prevention programs, as well as the need for innovation and scalability of programs. Systematic reviews of the research on the prevention of EDs suggest that prevention efforts based on cognitive dissonance, cognitive behavior therapy, or media literacy are promising in terms of reducing risk factors for EDs or disordered eating behavior [31]. A review of randomized controlled trials has confirmed these findings [32]. Media literacy received the best support among universal interventions, while dissonance-based intervention (Body Project) was the most efficacious selective intervention. In addition, cognitive behavior therapy, another dissonance-based intervention called Healthy Weight, media literacy, and psychoeducation were also effective when applied as selective intervention [32]. In terms of actual reduction in the future onset of EDs, only healthy lifestyle modification prevention programs and dissonance-based interventions emerged as efficacious when independent replications were included as criteria for evidence [33]. 

Likewise, while the prolific contributions of a few authors in the field highlight key individuals driving this research forward, this also points to the need for more researchers to enter this field. The pre-eminence of the United States in ED prevention research, contributing more than half of the total number of publications, indicates a concentrated effort in this region. Institutions like Stanford University and the Oregon Research Institute emerge as key centers of research, reflecting the importance of institutional support in advancing this field. On the other hand, this picture might simply be a reflection of the affiliation of some prominent researchers within this field and not that of focused and planned institutional support to promote research on ED prevention. Nevertheless, as it is often the case, the interaction between the support of the institutions and the interest and skills of the researchers, together with the availability of funding, might be the recipe for successful research on the prevention of EDs. The dominance of United States in ED prevention research publications is probably reflective of its dominance in (1) research and research output in general and (2) more availability of funding. Future research should examine the ED prevention research output from different countries in relation to their overall research output, the proportion of funding devoted to research in relation to their gross national income, and what portion of the research funding goes to ED research, and more specifically, to research on the prevention of EDs. Current investigations indicate that a very small fraction of research funding goes to ED research [34], despite anorexia nervosa being one of the psychiatric conditions with highest mortality rate [35,36]. This picture is probably universal and even more extreme in non-Western countries. 

Barriers to publication is another factor that should be investigated. When bibliometric findings are considered from a global perspective, several factors should be taken into account. When the literature search is conducted in English and only on papers written in English, a potentially significant portion of the other relevant literature will be automatically excluded. Given the dominance of English as the main research language, many researchers in countries where English is not the main or one of the official languages might fall behind in the publication rate in English journals. This will lead to a skewed picture of the current state of publications within any area of science. For many highly skilled researchers in non-English-speaking countries, English often becomes a barrier, and the probability of publishing their research in renowned English journals is generally low. Not all German, French, Russian, Chinese, Spanish, Japanese, or Iranian researchers, to mention a few nationalities, are fluent in English and proficient with the writing style required by English journals to successfully publish their work in such journals. Although some editors consciously work to be more inclusive and supportive to make the journal that they present into a truly international outlet of research, the gap is still very large. Future bibliometric studies using various languages to capture publication volume, contributions, collaborations, and trends are necessary for an accurate global picture and to ensure that important research findings, not always in English, are not ignored or marginalized. 

Finally, the discrepancies in the results of the broad search compared to the papers selected after manual screening point to the importance of the precision of the keywords and their combination for the outcome of the search, and the necessity of screening the papers before running the analyses. A comparison of the narrow versus wide literature search shows clearly that although a few prominent researchers emerge as the main figures in each cluster, and different ones based on the type of search, collaboration is the central feature, and nobody’s prominence occurs in isolation from other researchers. 

### Limitation

The present bibliographic analysis only covers the last three decades. It could have been extended to cover a longer period of time. However, given the low overall number of publications on the prevention of EDs before the 1990s, we considered a three-decade frame more informative. The key to all bibliographic studies is the use of adequate keywords and their combination, as well as the language of focus. We chose keywords in a way that would provide high sensitivity in the results, at the cost of specificity. However, the lack of specificity was compensated for by manual screening. The current search does not include gray literature, publications in other languages, and research papers not indexed in the Web of Science. Systematic reviews should use multiple databases, because different databases catalogue different literature sources [37]. However, this is rarely considered in bibliometric reviews [38]. One of the reasons is the technical challenges encountered to smoothly merge overlapping content [39], as different databases also structure their content differently. We used the Web of Science Core Collection for the search, which is the premier resource on the platform and the world’s most trusted citation index for scientific and scholarly research [40]. It has 4 indexes and a curated collection of over 21,000 peer-reviewed, scholarly journals of high quality (including open access journals) in over 250 science, social sciences, and humanities disciplines [41].

## 5. Conclusions

Our bibliometric analysis has provided some insights into the evolving landscape of ED prevention research. Despite some limitations, it informs us of a significant growth in the number of publications, particularly from 2010 to 2017, which may reflect an increasing academic emphasis on the prevention of EDs. The substantial contributions from key journals, authors, and institutions, especially within the United States, highlight the concentrated efforts in this field. Based on the current bibliographic analysis of publications on the prevention of EDs, it might be plausible to aim for a larger number of researchers and thus more research on the topic, as well as more collaboration between researchers to push the field forward. To achieve this, strategic investment in prevention research (funding), institutional support for prevention science, and strategic work on behalf of scientific journals to attract more publications on the prevention of EDs are called for. 

## Figures and Tables

**Table 1:** A basic description of the included papers.

Description	Results			
**Main information about data**				
Timespan	1993:2003	2004:2013	2014:2023	1993:2023
Sources (Journals, Books)	28	44	83	126
Documents	45	104	234	383
Annual Growth Rate %	21.48	8.84	1.06	10.85
Document Average Age	24.2	15.2	5.34	10.2
Average citations per doc	58.91	65.22	18.47	35.92
References	1269	2530	6815	9417
**Authors**				
Authors (involved in included publication)	126	232	729	1005
Authors of single-authored docs	6	8	8	20
**Authors collaboration**				
Single-authored papers	7	9	12	28
Co-Authors per paper	3.51	3.85	4.86	4.43
International co-authorships %	2.222	12.5	26.5	19.84
**Document types**				
Original research	38	90	195	323
Review	6	12	33	51
Editorial papers	1	2	6	9

**Table 2:** Authors, countries, and institutes with the highest number of published papers on ED prevention and their total number of citations.

Article Characteristics		No. of Articles	Total No. of Citations
Author	Stice E	66	3255
	Shaw H	40	1946
	Taylor CB	34	1284
	Rohde P	34	1603
	Wilfley DE	21	720
Country	USA	206	9313
	Australia	46	1896
	Germany	34	645
	Spain	16	291
	Canada	14	396
	United Kingdom	14	370
Institute	Stanford University	62	2653
	Oregon Research Institute	53	2586
	Harvard University	32	1863
	Washington University (Wustl)	30	1108
	Flinders University South Australia	24	1088

## Data Availability

Data are provided in Supplementary Materials.

## Supplementary Materials

The following supporting information can be downloaded at: https://www.mdpi.com/article/10.3390/nu16081111/s1, File S1: List of manually selected papers.; File S2: List of automatically generated papers.; File S3: Biometric analyses based on automatically selected papers. Figure S1: Peer-reviewed journals, in their order of publishing research on prevention of ED (*n* = 10,546). Figure S2: The publications trend in journals over time (*n* = 10,546). Figure S3: The authors’ production over time. Figure S4: The connections and publication orders among authors (*n* = 10,546).
