# Supplementary material for: A Bibliometric Analysis of Scientific Publications on Eating Disorder Prevention in the Past Three Decades
Source: Nutrients. 2024 Apr 10;16(8):1111. doi: 10.3390/nu16081111 (PMC11054308; doi:10.3390/nu16081111)
Supplement: Supplementary file 1 [file nutrients-16-01111-s001.zip › File S1.pdf]

1. Simpson Courtney, Boutte Rachel, Burnette C., Weinstock Madison, Goel Neha, Mazzeo Suzanne Cultural adaptation of an integrated eating disorders prevention and healthy weight management program. JOURNAL OF EATING DISORDERS. 2023;11(1):.

2. Jones Christina, Read Rebecca, O'Donnell Nicola, Wakelin Katherine, John Mary, Skene Simon, Stewart Rose, Hale Lucy, Cooke Debbie, Kanumakala Shankar, Satherley Rose-Marie PRIORITY Trial: Results from a feasibility randomised controlled trial of a psychoeducational intervention for parents to prevent disordered eating in children and young people with type 1 diabetes. DIABETIC MEDICINE. 2023;():.

3. Stice Eric, Wisting Line, Desjardins Christopher, Hood Korey, Hanes Sarah, Rubino Laura, Shaw Heather Evaluation of a novel eating disorder prevention program for young women with type 1 diabetes: A preliminary randomized trial. DIABETES RESEARCH AND CLINICAL PRACTICE. 2023;206():.

4. Perez-Vazquez Jorge, Gonzalez-Roz Alba, Amigo-Vazquez Isaac Effectiveness of an e-Health Quasi-Randomized Controlled Universal Prevention Program for Eating Disorders in Spanish Adolescents. JOURNAL OF PREVENTION. 2024;45(1):87-105.

5. Hilling Jonathan, Robertson Claire A review of the nutritional guidance for athletes to prevent eating disorders. EUROPEAN EATING DISORDERS REVIEW. 2024;32(1):116-129.

6. Rohde Paul, Bearman Sarah, Pauling Sydney, Gau Jeff, Shaw Heather, Stice Eric Setting and Provider Predictors of Implementation Success for an Eating Disorder Prevention Program Delivered by College Peer Educators. ADMINISTRATION AND POLICY IN MENTAL HEALTH AND MENTAL HEALTH SERVICES RESEARCH. 2023;50(6):912-925.

7. Alva Patricio, Ghaderi Ata, Andersson Gerhard, Feldman Inna, Sampaio Filipa The cost-effectiveness of a virtual intervention to prevent eating disorders in young women in Sweden. INTERNATIONAL JOURNAL OF EATING DISORDERS. 2023;56(10):1887-1897.

8. Nordin-Bates Sanna, Lundstrom Petra, Melin Anna, Sundgot-Borgen Jorunn, Edlund Klara Evaluation of DancExcellent, a Combined CBT and Nutrition Education Intervention. MEDICAL PROBLEMS OF PERFORMING ARTISTS. 2023;38(2):71-79.

9. Unikel Santoncini Claudia, Barajas Marquez Miriam, Leon Vazquez Concepcion, Parra Carriedo Alicia, Rivera Marquez Jose, Morcelle Gladys, Diaz Gutierrez Maricarmen Sex and Body Mass Index differences after one-year follow-up of an eating disorders risk factors

universal prevention intervention in university students in Mexico City. SALUD MENTAL. 2023;46(3):147-154.

10. Hage Trine, Nilsen Jan-Vegard, Karlsen Katrine, Lyslid Martine, Wennersberg Anne, Wisting Line ``I am not alone{''}. A qualitative feasibility study of eating disorders prevention groups for young females with type 1 diabetes. JOURNAL OF EATING DISORDERS. 2023;11(1):.

11. Koreshe Eyza, Paxton Susan, Miskovic-Wheatley Jane, Bryant Emma, Le Anvi, Maloney Danielle, Touyz Stephen, Maguire Sarah, Consortium Natl Prevention and early intervention in eating disorders: findings from a rapid review. JOURNAL OF EATING DISORDERS. 2023;11(1):.

12. Stice Eric, Rohde Paul, Gau Jeff, Bearman Sarah, Shaw Heather An Experimental Test of Increasing Implementation Support for College Peer Educators Delivering an Evidence-Based Prevention Program. JOURNAL OF CONSULTING AND CLINICAL PSYCHOLOGY. 2023;91(4):208-220.

13. Levinson Cheri, Trombley Christopher, Brosof Leigh, Williams Brenna, Hunt Rowan Binge Eating, Purging, and Restriction Symptoms: Increasing Accuracy of Prediction Using Machine Learning. BEHAVIOR THERAPY. 2023;54(2):247-259.

14. Schleider Jessica, Smith Arielle, Ahuvia Isaac Realizing the untapped promise of single-session interventions for eating disorders. INTERNATIONAL JOURNAL OF EATING DISORDERS. 2023;56(5):853-863.

15. Stice Eric, Rohde Paul, Gau Jeff, Shaw Heather Implementation Factors That Predict Larger Effects From a Peer Educator Delivered Eating Disorder Prevention Program at Universities. JOURNAL OF CONSULTING AND CLINICAL PSYCHOLOGY. 2023;91(2):60-70.

16. Dunker Karin, Carvalho Pedro, Amaral Ana Eating disorders prevention programs in Latin American countries: A systematic review. INTERNATIONAL JOURNAL OF EATING DISORDERS. 2023;56(4, SI):691-707.

17. Rutsztein Guillermina, Scappatura Maria, Elizathe Luciana, Leonardelli Eduardo, Murawski Brenda, Lievendag Leonora, Sanday Julieta, Falivelli Maria, Bidacovich German, Keegan Eduardo Efficacy of an integrated program (PIA-2) to reduce the risk for problems related to eating, weight and body image in female adolescents from Argentina.

INTERNATIONAL JOURNAL OF EATING DISORDERS. 2023;56(4, SI):758-769.

18. Stice Eric, Bohon Cara, Shaw Heather, Desjardins Christopher Efficacy of Virtual Delivery of a Dissonance-Based Eating Disorder Prevention Program and Evaluation of a Donation Model to Support Sustained Implementation. JOURNAL OF CONSULTING AND CLINICAL PSYCHOLOGY. 2023;91(3):139-149.

19. Stice Eric, Rohde Paul, Butryn Meghan, Desjardins Christopher, Shaw Heather Enhancing Efficacy of a Brief Obesity and Eating Disorder Prevention Program: Long-Term Results from an Experimental Therapeutics Trial. NUTRIENTS. 2023;15(4):.

20. Levine Michael, Sadeh-Sharvit Shiri Preventing eating disorders and disordered eating in genetically vulnerable, high-risk families. INTERNATIONAL JOURNAL OF EATING DISORDERS. 2023;56(3):523-534.

21. Pursey Kirrilly, Hart Melissa, Hure Alexis, Cheung Hei, Ong Liting, Burrows Tracy, Yager Zali The Needs of School Professionals for Eating Disorder Prevention in Australian Schools: A Mixed-Methods Survey. CHILDREN-BASEL. 2022;9(12):.

22. Long Michael, Ward Zachary, Wright Davene, Rodriguez Patricia, Tefft Nathan, Austin S. Cost-Effectiveness of 5 Public Health Approaches to Prevent Eating Disorders. AMERICAN JOURNAL OF PREVENTIVE MEDICINE. 2022;63(6):935-943.

23. Tobin Leah, Sears Christopher, Ranson Kristin Two Eating Disorder Preventive Interventions Reduce Attentional Biases in Body-Dissatisfied University Women: A Cluster Randomized Controlled Trial. JOURNAL OF CONSULTING AND CLINICAL PSYCHOLOGY. 2022;90(12):911-924.

24. Fararova Adela, Papezova Hana, Gricova Jana, Stepankova Tereza, Capek Vaclav, Reedtz Charlotte, Lauritzen Camilla, Doesum Karin ChildTalks plus : a study protocol of a pre-post controlled, paired design study on the use of preventive intervention for children of parents with a mental illness with focus on eating disorders. BMC PSYCHIATRY. 2022;22(1):.

25. Katcher Julia, Suminski Richard, Pacanowski Carly Impact of an Intuitive Eating Intervention on Disordered Eating Risk Factors in Female-Identifying Undergraduates: A Randomized Waitlist-Controlled Trial. INTERNATIONAL JOURNAL OF ENVIRONMENTAL RESEARCH AND PUBLIC HEALTH. 2022;19(19):.

26. Selenius Sofia, Birgegard Andreas, Mantilla Emma Preliminary evaluation of the online course ``I Care{''} targeting eating disorder knowledge and attitudes among sports coaches and fitness instructors. JOURNAL OF EATING DISORDERS. 2022;10(1):.

27. Blomquist Kerstin, Pate Sarah, Hock Amanda, Austin S. Evidence-based policy solutions to prevent eating disorders: Do disclaimer labels on fashion advertisements mitigate negative impact on adult women?. BODY IMAGE. 2022;43():180-192.

28. Royen Annelies, Malderen Eva, Desmeta Maurane, Goossens Lien, Verbeken Sandra, Kemps Eva Go or no-go? An assessment of inhibitory control training using the GO/NO-GO task in adolescents. APPETITE. 2022;179():.

29. Le Long, Tan Eng, Perez Joahna, Chiotelis Oxana, Hay Phillipa, Ananthapavan Jaithri, Lee Yong, Mihalopoulos Cathrine Prevention of high body mass index and eating disorders: a systematic review and meta-analysis. EATING AND WEIGHT DISORDERS-STUDIES ON ANOREXIA BULIMIA AND OBESITY. 2022;27(8):2989-3003.

30. Lepe-Salazar Francisco, Salgado-Torres Sarita Multiple Composite Scenarios: A Game-Based Methodology for the Prevention of Mental Disorders. ENTERTAINMENT COMPUTING. 2023;44():.

31. Estey Esther, Roff Chelsea, Kozlowski Michael, Rovig Stephanie, Guyker Wendy, Cook-Cottone Catherine Efficacy of Eat Breathe Thrive: A randomized controlled trial of a yoga-based program. BODY IMAGE. 2022;42():427-439.

32. Trojanowski Paige, Frietchen Rachel, Harvie Blair, Mehlenbeck Robyn, Fischer Sarah Internet-delivered eating disorders prevention program for adolescent girls with type 1 diabetes: Acceptable and feasible. PEDIATRIC DIABETES. 2022;23(7):1122-1132.

33. Peat Christine, Feltner Cynthia Addressing eating disorders in primary care: Understanding screening recommendations and opportunities to improve care. INTERNATIONAL JOURNAL OF EATING DISORDERS. 2022;55(9, SI):1202-1207.

34. Kristoffersen Mhairi, Johnson Catherine, Atkinson Melissa Feasibility and acceptability of video-based microinterventions for eating disorder prevention among adolescents in secondary schools. INTERNATIONAL JOURNAL OF EATING DISORDERS. 2022;55(11):1496-1505.

35. Lloyd Naomi Mental health problems and eating disorders: a student teacher's perception of current challenges facing pastoral care in education. PASTORAL CARE IN EDUCATION. 2022;40(3, SI):297-309.

36. Pellegrini Danielle, Grennan Laura, Bhatnagar Neera, McVey Gail, Couturier Jennifer Virtual prevention of eating disorders in children, adolescents, and emerging adults: a scoping review. JOURNAL OF EATING DISORDERS. 2022;10(1):.

37. Nicolaou Patrisia, Merwin Rhonda, Karekla Maria Acceptability and feasibility of a gamified digital eating disorder early-intervention program (<i>AcceptME</i>) based on Acceptance and Commitment Therapy (ACT). JOURNAL OF CONTEXTUAL BEHAVIORAL SCIENCE. 2022;25():26-34.

38. Jacobi Corinna, Vollert Bianka, Huetter Kristian, Bloh Paula, Eiterich Nadine, Goerlich Dennis, Taylor C. Indicated Web-Based Prevention for Women With Anorexia Nervosa Symptoms: Randomized Controlled Efficacy Trial. JOURNAL OF MEDICAL INTERNET RESEARCH. 2022;24(6):.

39. Sick Kelsey, Sabiston Catherine, Maharaj Aryel, Pila Eva Body image and disordered eating prevention in girls' sport: A partner-driven and stakeholder-informed scoping review of interventions. PSYCHOLOGY OF SPORT AND EXERCISE. 2022;61():.

40. Fiskum Charlotte, Riiber Ashild, Eik-Nes Trine Prevention of Unhealthy Weight, Disordered Eating, and Poor Body Image in Children. Perspectives From Norwegian Parents and Healthcare Professionals. FRONTIERS IN PSYCHIATRY. 2022;13():.

41. LaMarre Andrea, Levine Michael, Holmes Su, Malson Helen An open invitation to productive conversations about feminism and the spectrum of eating disorders (part 2): Potential contributions to the science of diagnosis, treatment, and prevention. JOURNAL OF EATING DISORDERS. 2022;10(1):.

42. Karekla Maria, Nikolaou Patrisia, Merwin Rhonda Randomized Clinical Trial Evaluating <i>AcceptME</i>-A Digital Gamified Acceptance and Commitment Early Intervention Program for Individuals at High Risk for Eating Disorders. JOURNAL OF CLINICAL MEDICINE. 2022;11(7):.

43. Levine Michael Prevention of eating disorders: 2021 in review. EATING DISORDERS. 2022;30(2):121-143.

44. Ali Kathina, Fassnacht Daniel, Farrer Louise, Rieger Elizabeth, Moessner Markus, Bauer Stephanie, Griffiths Kathleen Recruitment, adherence and attrition challenges in internet-based indicated prevention programs for eating disorders: lessons learned from a randomised controlled trial of <i>ProYouth OZ</i>. JOURNAL OF EATING DISORDERS. 2022;10(1):.

45. Fitzsimmons-Craft Ellen, Chan William, Smith Arielle, Firebaugh Marie-Laure, Fowler Lauren, Topooco Naira, DePietro Bianca, Wilfley Denise, Taylor C., Jacobson Nicholas Effectiveness of a chatbot for eating disorders prevention: A randomized clinical trial. INTERNATIONAL JOURNAL OF EATING DISORDERS. 2022;55(3):343-353.

46. Haslam Rebecca, Clarke Erin, Gray Scarlett, Gearon Rachel, Pursey Kirrilly Findings from a web content analysis of resources targeting sporting coaches aimed at educating or upskilling on eating disorders and disordered eating in athletes. JOURNAL OF EATING DISORDERS. 2021;9(1):.

47. Stice Eric, Onipede Z., Shaw Heather, Rohde Paul, Gau Jeff Effectiveness of the Body Project Eating Disorder Prevention Program for Different Racial and Ethnic Groups and an Evaluation of the Potential Benefits of Ethnic Matching. JOURNAL OF CONSULTING AND CLINICAL PSYCHOLOGY. 2021;89(12):1007-1019.

48. Carrard Isabelle, Della Torre Sophie A study protocol for a preliminary randomised controlled trial assessing the acceptability and effectiveness of two eating disorders prevention interventions in Switzerland: The HEIDI BP-HW project. PLOS ONE. 2021;16(11):.

49. Jones Christina, O'Donnell Nicola, John Mary, Cooke Debbie, Stewart Rose, Hale Lucy, Skene Simon, Kanumakala Shankar, Harrington Megan, Satherley Rose-Marie PaRent InterventiOn to pRevent dIsordered eating in children with TYpe 1 diabetes (PRIORITY): Study protocol for a feasibility randomised controlled trial. DIABETIC MEDICINE. 2022;39(4):.

50. Haderlein Taona, Tomiyama A. Effects of internet-delivered eating disorder prevention on reward-based eating drive: A randomized controlled trial. EATING BEHAVIORS. 2021;43():.

51. Stice Eric, Rohde Paul, Gau Jeff, Butryn Meghan, Shaw Heather, Cloud Kasie, D'Adamo Laura Enhancing Efficacy of a Dissonance-Based Obesity and Eating Disorder Prevention Program: Experimental Therapeutics. JOURNAL OF CONSULTING AND CLINICAL PSYCHOLOGY. 2021;89(10):793-804.

52. Wolter Vanessa, Hammerle Florian, Buerger Arne, Ernst Verena Prevention of eating disorders-Efficacy and cost-benefit of a school-based program ({''}MaiStep{''}) in a randomized controlled trial (RCT). INTERNATIONAL JOURNAL OF EATING DISORDERS. 2021;54(10):1855-1864.

53. Vila-Marti Anna, Elio Inaki, Sumalla-Cano Sandra Eating Behavior during First-Year College Students, including Eating Disorders-RUVIC-RUNEAT-TCA Project. Protocol of an Observational Multicentric Study. INTERNATIONAL JOURNAL OF ENVIRONMENTAL RESEARCH AND PUBLIC HEALTH. 2021;18(18):.

54. Linardon Jake, Messer Mariel, Shatte Adrian, Skvarc David, Rosato John, Rathgen April, Fuller-Tyszkiewicz Matthew Targeting dietary restraint to reduce binge eating: a randomised controlled trial of a blended internet- and smartphone app-based intervention. PSYCHOLOGICAL MEDICINE. 2023;53(4):1277-1287.

55. Pursey Kirrilly, Burrows Tracy, Barker Daniel, Hart Melissa, Paxton Susan Disordered eating, body image concerns, and weight control behaviors in primary school aged children: A systematic review and meta-analysis of universal-selective prevention interventions. INTERNATIONAL JOURNAL OF EATING DISORDERS. 2021;54(10):1730-1765.

56. Wisting Line, Haugvik Severina, Wennersberg Anne, Hage Trine, Stice Eric, Olmsted Marion, Ghaderi Ata, Brunborg Cathrine, Skrivarhaug Torild, Dahl-Jorgensen Knut, Ro Oyvind Feasibility of a virtually delivered eating disorder prevention program for young females with type 1 diabetes. INTERNATIONAL JOURNAL OF EATING DISORDERS. 2021;54(9):1696-1706.

57. Nacke Barbara, Zeiler Michael, Kuso Stefanie, Klesges Lisa, Jacobi Corinna, Waldherr Karin A systematic review of reach, adoption, implementation and maintenance of Internet-based interventions to prevent eating disorders in adults. EUROPEAN JOURNAL OF PUBLIC HEALTH. 2021;31(1):29-37.

58. Akers Laura, Rohde Paul, Shaw Heather, Stice Eric Cost-Effectiveness Comparison of Delivery Modalities for a Dissonance-Based Eating Disorder Prevention Program over 4-

Year Follow-Up. PREVENTION SCIENCE. 2021;22(8):1086-1095.

59. Oliveira Resende Thaina, Almeida Mauricio, Alvarenga Marle, Brown Tiffany, Carvalho Pedro Dissonance-based eating disorder prevention improves intuitive eating: a randomized controlled trial for Brazilian women with body dissatisfaction. EATING AND WEIGHT DISORDERS-STUDIES ON ANOREXIA BULIMIA AND OBESITY. 2022;27(3):1099-1112.

60. Raith Anna-Marie, Haemmerling Marie, Klein Sabrina, Peitz Diana, Knaevelsrud Christine, Zagorscak Pavle Promotion of self-esteem in the universal prevention of eating disorders. Pilot study of an internet-based intervention in a sample of students. PSYCHOTHERAPEUT. 2021;66(4, SI):275-281.

61. Stice Eric, Onipede Z., Marti C. A meta-analytic review of trials that tested whether eating disorder prevention programs prevent eating disorder onset. CLINICAL PSYCHOLOGY REVIEW. 2021;87():.

62. Balciuniene Vaiva, Jankauskiene Rasa, Baceviciene Migle Effect of an education and mindfulness-based physical activity intervention for the promotion of positive body image in Lithuanian female students. EATING AND WEIGHT DISORDERS-STUDIES ON ANOREXIA BULIMIA AND OBESITY. 2022;27(2):563-577.

63. Call Christine, D'Adamo Laura, Butryn Meghan, Stice Eric Examining weight suppression as a predictor and moderator of intervention outcomes in an eating disorder and obesity prevention trial: A replication and extension study. BEHAVIOUR RESEARCH AND THERAPY. 2021;141():.

64. Jarman Hannah, Treneman-Evans Georgia, Halliwell Emma ``I didn't want to say something and them to go outside and tell everyone{''}: The acceptability of a dissonance-based body image intervention among adolescent girls in the UK. BODY IMAGE. 2021;38():80-84.

65. Hudson Tassiana, Soares Amaral Ana, Stice Eric, Gau Jeff, Caputo Ferreira Maria Dissonance-based eating disorder prevention among Brazilian young women: A randomized efficacy trial of the<i> Body</i><i> Project</i>. BODY IMAGE. 2021;38():1-9.

66. Nina Kalindjian, France Hirot, Anne-Claire Stona, Caroline Huas, Nathalie Godart Early detection of eating disorders: a scoping review. EATING AND WEIGHT DISORDERS-

STUDIES ON ANOREXIA BULIMIA AND OBESITY. 2022;27(1):21-68.

67. Oldham-Cooper Rosie, Semple Claire Prevention and early help for eating disorders in young people with type 1 diabetes. CLINICAL CHILD PSYCHOLOGY AND PSYCHIATRY. 2021;26(3):656-668.

68. Atkinson Melissa, Diedrichs Phillippa Examining the efficacy of video-based microinterventions for improving risk and protective factors for disordered eating among young adult women. INTERNATIONAL JOURNAL OF EATING DISORDERS. 2021;54(5):708-720.

69. Doley Joanna, McLean Sian, Griffiths Scott, Yager Zali Designing Body Image and Eating Disorder Prevention Programs for Boys and Men: Theoretical, Practical, and Logistical Considerations From Boys, Parents, Teachers, and Experts. PSYCHOLOGY OF MEN \& MASCULINITIES. 2021;22(1):124-134.

70. Luo Yi-Jun, Jackson Todd, Stice Eric, Chen Hong Effectiveness of an Internet Dissonance-Based Eating Disorder Prevention Intervention Among Body-Dissatisfied Young Chinese Women. BEHAVIOR THERAPY. 2021;52(1):221-233.

71. Kwag Kyung, Han Soo, Cho Ji-Yeoun, Ko Myeong, Park Eun, Kim Youl-Ri A school-based eating disorder prevention program (Me, You \& Us) for young adolescents in Korea: A 3-year follow-up study. INTERNATIONAL JOURNAL OF EATING DISORDERS. 2021;54(2, SI):168-173.

72. Almeida Mauricio, Brown Tiffany, Campos Priscila, Amaral Ana, Carvalho Pedro Dissonance-based eating disorder prevention delivered in-person after an online training: A randomized controlled trial for Brazilian men with body dissatisfaction. INTERNATIONAL JOURNAL OF EATING DISORDERS. 2021;54(3):293-304.

73. Yang Jiwon, Han Kuem A rational emotive behavior therapy-based intervention for binge eating behavior management among female students: a quasi-experimental study. JOURNAL OF EATING DISORDERS. 2020;8(1):.

74. Kumar Maya, Argo Taylor, Chang Jane, Cifra Nicole, Docter Alicia, Galagali Preeti, Kapphahn Cynthia, Key Janice, Pitt Paulette, Weiss Amy Preventing Nutritional Disorders in Adolescents by Encouraging a Healthy Relationship With Food. JOURNAL OF ADOLESCENT

HEALTH. 2020;67(6):875-879.

75. Vanderkruik Rachel, Gist Darcy, Dimidjian Sona Preventing Eating Disorders in Young Women: An RCT and Mixed-Methods Evaluation of the Peer-Delivered Body Project. JOURNAL OF CONSULTING AND CLINICAL PSYCHOLOGY. 2020;88(12):1105-1118.

76. Linardon Jake, Messer Mariel, Lee Sohee, Rosato John Perspectives of e-health interventions for treating and preventing eating disorders: descriptive study of perceived advantages and barriers, help-seeking intentions, and preferred functionality. EATING AND WEIGHT DISORDERS-STUDIES ON ANOREXIA BULIMIA AND OBESITY. 2021;26(4):1097-1109.

77. Vanderkruik Rachel, Conte Isabella, Dimidjian Sona Fat talk frequency in high school women: Changes associated with participation in the Body Project. BODY IMAGE. 2020;34():196-200.

78. Burke Natasha, Neyland M., Young Jami, Wilfley Denise, Tanofsky-Kraff Marian Interpersonal psychotherapy for the prevention of binge-eating disorder and adult obesity in an African American adolescent military dependent boy. EATING BEHAVIORS. 2020;38():.

79. Borden Ashlye, Cook-Cottone Catherine Yoga and eating disorder prevention and treatment: A comprehensive review and meta-analysis. EATING DISORDERS. 2020;28(4, SI):400-437.

80. Pacanowski C., Diers L., Crosby R., Mackenzie M., Neumark-Sztainer D. Yoga's impact on risk and protective factors for disordered eating: a pilot prevention trial. EATING DISORDERS. 2020;28(4, SI):513-541.

81. Schoen Eva, Clougher Kelly, Wiese Joanna Developing an Eating Disorder Peer Advocate Program on Campus: A Report on the Eating Disorder Awareness and Advocacy Program (EDAAP). JOURNAL OF COLLEGE STUDENT PSYCHOTHERAPY. 2020;34(3):211-227.

82. Ghaderi Ata, Stice Eric, Andersson Gerhard, Persson Johanna, Allzen Elin A Randomized Controlled Trial of the Effectiveness of Virtually Delivered Body Project (vBP) Groups to Prevent Eating Disorders. JOURNAL OF CONSULTING AND CLINICAL PSYCHOLOGY. 2020;88(7):643-656.

83. Burnette C., Mazzeo Suzanne An uncontrolled pilot feasibility trial of an intuitive eating intervention for college women with disordered eating delivered through group and guided self-help modalities. INTERNATIONAL JOURNAL OF EATING DISORDERS. 2020;53(9):1405-1417.

84. Simpson Courtney, Burnette C., Mazzeo Suzanne Integrating eating disorder and weight gain prevention: a pilot and feasibility trial of INSPIRE. EATING AND WEIGHT DISORDERS-STUDIES ON ANOREXIA BULIMIA AND OBESITY. 2020;25(3):761-775.

85. Hirsch Katherine, Blomquist Kerstin Community-Based Prevention Programs for Disordered Eating and Obesity: Updates and Current Limitations. CURRENT OBESITY REPORTS. 2020;9(2):81-97.

86. Stice Eric, Rohde Paul, Shaw Heather, Gau Jeff Clinician-Led, Peer-Led, and Internet-Delivered Dissonance-Based Eating Disorder Prevention Programs: Effectiveness of These Delivery Modalities Through 4-Year Follow-Up. JOURNAL OF CONSULTING AND CLINICAL PSYCHOLOGY. 2020;88(5):481-494.

87. Vollert Bianka, Bloh Paula, Eiterich Nadine, Beintner Ina, Huetter Kristian, Taylor Craig, Jacobi Corinna Recruiting participants to an Internet-based eating disorder prevention trial: Impact of the recruitment strategy on symptom severity and program utilization. INTERNATIONAL JOURNAL OF EATING DISORDERS. 2020;53(5):476-484.

88. Wilson Rebecca, Marshall Rachel, Murakami Jessica, Latner Janet Brief non-dieting intervention increases intuitive eating and reduces dieting intention, body image dissatisfaction, and anti-fat attitudes: A randomized controlled trial. APPETITE. 2020;148():.

89. Blackstone Sarah, Sangiorgio Celeste, Johnson Aimee Peer Recognition of Disordered Eating Behaviors: Implications for Improving Awareness through Health Education. AMERICAN JOURNAL OF HEALTH EDUCATION. 2020;51(3):142-150.

90. Muehleck Julia, Borse Sigrid, Wunderer Eva, Strauss Bernhard, Berger Uwe Online survey on the awareness of offers for information, prevention, counselling, and aftercare for eating disorders. PRAVENTION UND GESUNDHEITSFORDERUNG. 2020;15(1):73-79.

91. Harrer Mathias, Adam Sophia, Messner Eva-Maria, Baumeister Harald, Cuijpers Pim, Bruffaerts Ronny, Auerbach Randy, Kessler Ronald, Jacobi Corinna, Taylor Craig, Ebert

David Prevention of eating disorders at universities: A systematic review and meta-analysis. INTERNATIONAL JOURNAL OF EATING DISORDERS. 2020;53(6):813-833.

92. Levine Michael Prevention of eating disorders: 2019 in review. EATING DISORDERS. 2020;28(1):6-20.

93. Kramer Rachel, Cuccolo Kelly Yoga Practice in a College Sample: Associated Changes in Eating Disorder, Body Image, and Related Factors Over Time. EATING DISORDERS. 2020;28(4, SI):494-512.

94. Moessner Markus, Bilic Sally, Bauer Stephanie The Importance of Social Exchange for Internet-based Eating Disorder Prevention. PRAXIS DER KINDERPSYCHOLOGIE UND KINDERPSYCHIATRIE. 2019;68(8):728-741.

95. Kant R., Wong-Chung Agnes, Evans Elizabeth, Stanton Elaine, Boothroyd Lynda The Impact of a Dissonance-Based Eating Disorders Intervention on Implicit Attitudes to Thinness in Women of Diverse Sexual Orientations. FRONTIERS IN PSYCHOLOGY. 2019;10():.

96. Chua Joelle, Tam Wilson, Shorey Shefaly Research Review: Effectiveness of universal eating disorder prevention interventions in improving body image among children: a systematic review and meta-analysis. JOURNAL OF CHILD PSYCHOLOGY AND PSYCHIATRY. 2020;61(5):522-535.

97. Kremer Michael, Kremer Kristen, Kremer Theodore School health class associated with reduced odds of eating disorder symptoms in American adolescents. INTERNATIONAL JOURNAL OF EATING DISORDERS. 2020;53(3):383-390.

98. Fitzsimmons-Craft Ellen, Balantekin Katherine, Eichen Dawn, Graham Andrea, Monterubio Grace, Sadeh-Sharvit Shiri, Goel Neha, Flatt Rachael, Saffran Kristina, Karam Anna, Firebaugh Marie-Laure, Trockel Mickey, Taylor C., Wilfley Denise Screening and offering online programs for eating disorders: Reach, pathology, and differences across eating disorder status groups at 28 US universities. INTERNATIONAL JOURNAL OF EATING DISORDERS. 2019;52(10, SI):1125-1136.

99. Castillo Irais, Solano Santos, Sepulveda Ana A controlled study of an integrated prevention program for improving disordered eating and body image among Mexican university students: A 3-month follow-up. EUROPEAN EATING DISORDERS REVIEW.

2019;27(5):541-556.

100. Christian Caroline, Brosof Leigh, Vanzhula Irina, Williams Brenna, Ram Shruti, Levinson Cheri Implementation of a dissonance-based, eating disorder prevention program in Southern, all-female high schools. BODY IMAGE. 2019;30():26-34.

101. Hall Peter Brain Stimulation as a Method for Understanding, Treating, and Preventing Disorders of Indulgent Food Consumption. CURRENT ADDICTION REPORTS. 2019;6(3):266-272.

102. Hinojo-Lucena Francisco-Javier, Aznar-Diaz Inmaculada, Caceres-Reche Maria-Pilar, Trujillo-Torres Juan-Manuel, Romero-Rodriguez Jose-Maria Problematic Internet Use as a Predictor of Eating Disorders in Students: A Systematic Review and Meta-Analysis Study. NUTRIENTS. 2019;11(9):.

103. Shu Chloe, Watson Hunna, Anderson Rebecca, Wade Tracey, Kane Robert, Egan Sarah A randomized controlled trial of unguided internet cognitive behaviour therapy for perfectionism in adolescents: Impact on risk for eating disorders. BEHAVIOUR RESEARCH AND THERAPY. 2019;120():.

104. Beintner Ina, Emmerich Olivia, Vollert Bianka, Taylor C., Jacobi Corinna Promoting positive body image and intuitive eating in women with overweight and obesity via an online intervention: Results from a pilot feasibility study. EATING BEHAVIORS. 2019;34():.

105. Dakanalis Antonios, Clerici Massimo, Stice Eric Prevention of eating disorders: current evidence-base for dissonance-based programmes and future directions. EATING AND WEIGHT DISORDERS-STUDIES ON ANOREXIA BULIMIA AND OBESITY. 2019;24(4):597-603.

106. Casasnovas Arielle, Huryk Kathryn, Levinson Devorah, Markowitz Sara, Friedman Shoshana, Stice Eric, Loeb Katharine Cognitive dissonance-based eating disorder prevention: pilot study of a cultural adaptation for the Orthodox Jewish community. EATING DISORDERS. 2021;29(2):192-204.

107. Stice Eric, Desjardins Christopher, Shaw Heather, Rohde Paul Moderators of two dual eating disorder and obesity prevention programs. BEHAVIOUR RESEARCH AND THERAPY. 2019;118():77-86.

108. Soares Amaral Ana, Stice Eric, Caputo Ferreira Maria A controlled trial of a dissonance-based eating disorders prevention program with Brazilian girls. PSICOLOGIA-REFLEXAO E CRITICA. 2019;32():.

109. Buerger Arne, Ernst Verena, Wolter Vanessa, Huss Michael, Kaess Michael, Hammerle Florian Treating eating disorders in the real world - MaiStep: A skill-based universal prevention for schools. PREVENTIVE MEDICINE. 2019;123():324-332.

110. Stice Eric, Marti C., Shaw Heather, Rohde Paul Meta-analytic review of dissonance-based eating disorder prevention programs: Intervention, participant, and facilitator features that predict larger effects. CLINICAL PSYCHOLOGY REVIEW. 2019;70():91-107.

111. Stice Eric, Johnson Sarah, Turgon Roxane Eating Disorder Prevention. PSYCHIATRIC CLINICS OF NORTH AMERICA. 2019;42(2, 2):309+.

112. Fitzsimmons-Craft Ellen, Firebaugh Marie-Laure, Graham Andrea, Eichen Dawn, Monterubio Grace, Balantekin Katherine, Karam Anna, Seal Annie, Funk Burkhardt, Taylor C., Wilfley Denise State-Wide University Implementation of an Online Platform for Eating Disorders Screening and Intervention. PSYCHOLOGICAL SERVICES. 2019;16(2, SI):239-249.

113. Nacke Barbara, Beintner Ina, Goerlich Dennis, Vollert Bianka, Schmidt-Hantke Juliane, Huetter Kristian, Taylor C., Jacobi Corinna everyBody-Tailored online health promotion and eating disorder prevention for women: Study protocol of a dissemination trial. INTERNET INTERVENTIONS-THE APPLICATION OF INFORMATION TECHNOLOGY IN MENTAL AND BEHAVIOURAL HEALTH. 2019;16(SI):20-25.

114. Unikel-Santoncini Claudia, Leon-Vazquez Concepcion, Rivera-Marquez Jose, Bojorquez-Chapela Ietza, Mendez-Rios Enrique Dissonance-based Program for Eating Disorders Prevention in Mexican University Students. PSYCHOSOCIAL INTERVENTION. 2019;28(1):29-35.

115. Rodgers Rachel, Donovan Elizabeth, Cousineau Tara, McGowan Kayla, Yates Kayla, Cook Elizabeth, Lowy Alice, Franko Debra Ethnic and racial diversity in eating disorder prevention trials. EATING DISORDERS. 2019;27(2, SI):168-182.

116. Voelker Dana, Petrie Trent, Huang Qiushi, Chandran Avinash Bodies in Motion: An empirical evaluation of a program to support positive body image in female collegiate

athletes. BODY IMAGE. 2019;28():149-158.

117. Leme Ana, Philippi Sonia, Thompson Debbe, Nicklas Theresa, Baranowski Tom ``Healthy Habits, Healthy Girls-Brazil{''}: an obesity prevention program with added focus on eating disorders. EATING AND WEIGHT DISORDERS-STUDIES ON ANOREXIA BULIMIA AND OBESITY. 2019;24(1):107-119.

118. Wilksch Simon, O'Shea Anne, Wade Tracey Depressive symptoms, alcohol and other drug use, and suicide risk: Prevention and treatment effects from a two-country online eating disorder risk reduction trial. INTERNATIONAL JOURNAL OF EATING DISORDERS. 2019;52(2):132-141.

119. Bauer Stephanie, Bilic Sally, Reetz Christina, Ozer Fikret, Becker Katja, Eschenbeck Heike, Kaess Michael, Rummel-Kluge Christine, Salize Hans-Joachim, Diestelkamp Silke, Moessner Markus, Thomasius Rainer, Bertsch Katja, Brunner Romuald, Feldhege Johannes, Gallinat Christina, Herpertz Sabine, Koenig Julian, Lustig Sophia, Parzer Peter, Resch Franz, Ritter Sabrina, Spinner Jens, Wille Kristina, Baldofski Sabrina, Kohls Elisabeth, Peter Lina-Jolien, Gille Vera, Hofmann Hanna, Lehner Laya, Voss Elke, Pfeiffer Jens, Samel Alisa, Consortium ProHEAD Efficacy and cost-effectiveness of Internet-based selective eating disorder prevention: study protocol for a randomized controlled trial within the ProHEAD Consortium. TRIALS. 2019;20():.

120. Breithaupt Lauren, Eickman Laura, Byrne Catherine, Fischer Sarah REbeL peer education: A model of a voluntary, after-school program for eating disorder prevention. EATING BEHAVIORS. 2019;32():111-116.

121. Levine Michael Prevention of eating disorders: 2018 in review. EATING DISORDERS. 2019;27(1):18-33.

122. Linardon Jake, Gleeson John, Yap Keong, Murphy Kylie, Brennan Leah Meta-analysis of the effects of third-wave behavioural interventions on disordered eating and body image concerns: implications for eating disorder prevention. COGNITIVE BEHAVIOUR THERAPY. 2019;48(1):15-38.

123. Munsch Simone Eating disorders - new challenges. ZEITSCHRIFT FUR PSYCHIATRIE PSYCHOLOGIE UND PSYCHOTHERAPIE. 2019;67(1):5-8.

124. Pickhardt Mara, Adametz Luise, Richter Felicitas, Strauss Bernhard, Berger Uwe German Prevention Programs for Eating Disorders A Systematic Review. PSYCHOTHERAPIE PSYCHOSOMATIK MEDIZINISCHE PSYCHOLOGIE. 2019;69(1):10-19.

125. Jacobi Corinna, Huetter Kristian, Voelker Ulrike, Moebius Katharina, Richter Robert, Trockel Mickey, Bell Megan, Lock James, Taylor C. Efficacy of a Parent-Based, Indicated Prevention for Anorexia Nervosa: Randomized Controlled Trial. JOURNAL OF MEDICAL INTERNET RESEARCH. 2018;20(12):.

126. Golan Moria, Abu Ahmad Wiessam School-based versus after-school delivery of a universal wellness programme - A randomized controlled multi-arm trial. EATING BEHAVIORS. 2018;31():41-47.

127. Green M., Kroska A., Herrick A., Bryant B., Sage E., Miles L., Ravet M., Powers M., Whitegoat W., Linkhart R., King B. A preliminary trial of an online dissonance-based eating disorder intervention. EATING BEHAVIORS. 2018;31():88-98.

128. Saucedo-Molina Teresita, Villarreal Castillo Martin, Oliva Macias Luz, Unikel Santoncini Claudia, Guzman Saldana Rebeca Disordered eating behaviours and sedentary lifestyle prevention among young Mexicans: A pilot study. HEALTH EDUCATION JOURNAL. 2018;77(8):872-883.

129. Stinson Emma, Perez Marisol, Ohrt Tara, Von Schell Anna, Bruening Amanda The association between program credibility, expectancy, and acceptability with baseline pathology and outcome for a body acceptance prevention program. JOURNAL OF CLINICAL PSYCHOLOGY. 2018;74(12):2161-2172.

130. Wade Tracey, Wilksch Simon Internet eating disorder prevention. CURRENT OPINION IN PSYCHIATRY. 2018;31(6):456-461.

131. Lee Gi, Park Eun, Kim Youl-Ri, Kwag Kyung, Park Jin, An So, Lee Ji, Sim Jeong, Treasure Janet Feasibility and acceptability of a prevention program for eating disorders (Me, You and Us) adapted for young adolescents in Korea. EATING AND WEIGHT DISORDERS-STUDIES ON ANOREXIA BULIMIA AND OBESITY. 2018;23(5):673-683.

132. Lee Yong, Le Long, Stockings Emily, Hay Phillipa, Whiteford Harvey, Barendregt Jan, Mihalopoulos Cathrine Estimation of a Relative Risk Effect Size when Using Continuous Outcomes Data: An Application of Methods in the Prevention of Major Depression and

Eating Disorders. MEDICAL DECISION MAKING. 2018;38(7):866-880.

133. Wilksch Simon, O'Shea Anne, Taylor C., Wilfley Denise, Jacobi Corinna, Wade Tracey Online prevention of disordered eating in at-risk young-adult women: a two-country pragmatic randomized controlled trial. PSYCHOLOGICAL MEDICINE. 2018;48(12):2034-2044.

134. Shaw Heather, Rohde Paul, Stice Eric Using participant feedback to improve two selective eating disorder and obesity prevention programs. EATING BEHAVIORS. 2018;30():93-97.

135. Rohde Paul, Desjardins Christopher, Arigo Danielle, Shaw Heather, Stice Eric Mediators of two selective prevention interventions targeting both obesity and eating disorders. BEHAVIOUR RESEARCH AND THERAPY. 2018;106():8-17.

136. Rodgers Rachel, Sonneville Kendrin Research for leveraging food policy in universal eating disorder prevention. INTERNATIONAL JOURNAL OF EATING DISORDERS. 2018;51(6):503-506.

137. Warschburger Petra, Zitzmann Jana The Efficacy of a Universal School-Based Prevention Program for Eating Disorders among German Adolescents: Results from a Randomized-Controlled Trial. JOURNAL OF YOUTH AND ADOLESCENCE. 2018;47(6, SI):1317-1331.

138. Pennesi Jamie-Lee, Wade Tracey Imagery rescripting and cognitive dissonance: A randomized controlled trial of two brief online interventions for women at risk of developing an eating disorder. INTERNATIONAL JOURNAL OF EATING DISORDERS. 2018;51(5):439-448.

139. Le Long, Hay Phillipa, Mihalopoulos Cathrine A systematic review of cost-effectiveness studies of prevention and treatment for eating disorders. AUSTRALIAN AND NEW ZEALAND JOURNAL OF PSYCHIATRY. 2018;52(4):328-338.

140. Leme Ana, Thompson Debbe, Dunker Karin, Nicklas Theresa, Philippi Sonia, Lopez Tabbetha, Vezina-Im Lydi-Anne, Baranowski Tom Obesity and eating disorders in integrative prevention programmes for adolescents: protocol for a systematic review and meta-analysis. BMJ OPEN. 2018;8(4):.

141. Stice E., Rohde P., Shaw H., Gau J. An experimental therapeutics test of whether adding dissonance-induction activities improves the effectiveness of a selective obesity and eating disorder prevention program. INTERNATIONAL JOURNAL OF OBESITY. 2018;42(3):462-468.

142. Wilksch Simon, O'Shea Anne, Wade Tracey Media Smart-Targeted: Diagnostic outcomes from a two-country pragmatic online eating disorder risk reduction trial for young adults. INTERNATIONAL JOURNAL OF EATING DISORDERS. 2018;51(3):270-274.

143. Eickman Laura, Betts Jessica, Pollack Lauren, Bozsik Frances, Beauchamp Marshall, Lundgren Jennifer Randomized controlled trial of REbeL: A peer education program to promote positive body image, healthy eating behavior, and empowerment in teens. EATING DISORDERS. 2018;26(2):127-142.

144. Green Emalee, Venta Amanda Lack of implementation of eating disorder education and prevention programs in high schools: Data from incoming college freshmen. EATING DISORDERS. 2018;26(5):430-447.

145. Podina Ioana, Fodor Liviu, Cosmoiu Ana, Boian Rares An evidence-based gamified mHealth intervention for overweight young adults with maladaptive eating habits: study protocol for a randomized controlled trial. TRIALS. 2017;18():.

146. Green M., Willis M., Fernandez-Kong K., Reyes S., Linkhart R., Johnson M., Thorne T., Lindberg J., Kroska E., Woodward H. A Controlled Randomized Preliminary Trial of a Modified Dissonance-Based Eating Disorder Intervention Program. JOURNAL OF CLINICAL PSYCHOLOGY. 2017;73(12):1612-1628.

147. Hermans Roel, Bruin Hanneke, Larsen Junilla, Mensink Frederike, Hoek Annet Adolescents' Responses to a School-Based Prevention Program Promoting Healthy Eating at School. FRONTIERS IN PUBLIC HEALTH. 2017;5():.

148. Kollei Ines, Lukas Christian, Loeber Sabine, Berking Matthias An App-Based Blended Intervention to Reduce Body Dissatisfaction: A Randomized Controlled Pilot Study. JOURNAL OF CONSULTING AND CLINICAL PSYCHOLOGY. 2017;85(11):1104-1108.

149. Chithambo Taona, Huey Stanley Internet-delivered eating disorder prevention: A randomized controlled trial of dissonance-based and cognitive-behavioral interventions.

INTERNATIONAL JOURNAL OF EATING DISORDERS. 2017;50(10):1142-1151.

150. Kindermann Sally, Moessner Markus, Ozer Fikret, Bauer Stephanie Associations between eating disorder related symptoms and participants' utilization of an individualized Internet-based prevention and early intervention program. INTERNATIONAL JOURNAL OF EATING DISORDERS. 2017;50(10):1215-1221.

151. Bauer Stephanie, Kindermann Sally, Moessner Markus Prevention of eating disorder: a review. ZEITSCHRIFT FUR KINDER-UND JUGENDPSYCHIATRIE UND PSYCHOTHERAPIE. 2017;45(5):403-411.

152. Burke Natasha, Shomaker Lauren, Brady Sheila, Reynolds James, Young Jami, Wilfley Denise, Sbrocco Tracy, Stephens Mark, Olsen Cara, Yanovski Jack, Tanofsky-Kraff Marian Impact of Age and Race on Outcomes of a Program to Prevent Excess Weight Gain and Disordered Eating in Adolescent Girls. NUTRIENTS. 2017;9(9):.

153. Stice Eric, Rohde Paul, Shaw Heather, Gau Jeff Clinician-Led, Peer-Led, and Internet-Delivered Dissonance-Based Eating Disorder Prevention Programs: Acute Effectiveness of These Delivery Modalities. JOURNAL OF CONSULTING AND CLINICAL PSYCHOLOGY. 2017;85(9):883-895.

154. Gumz Antje, Weigel Angelika, Daubmann Anne, Wegscheider Karl, Romer Georg, Loewe Bernd Efficacy of a prevention program for eating disorders in schools: a cluster-randomized controlled trial. BMC PSYCHIATRY. 2017;17():.

155. Becker Carolyn, Stice Eric From Efficacy to Effectiveness to Broad Implementation: Evolution of the Body Project. JOURNAL OF CONSULTING AND CLINICAL PSYCHOLOGY. 2017;85(8):767-782.

156. Brown Tiffany, Forney K., Pinner Dennis, Keel Pamela A randomized controlled trial of <i>The Body Project</i>: <i>More Than Muscles</i> for men with body dissatisfaction. INTERNATIONAL JOURNAL OF EATING DISORDERS. 2017;50(8):873-883.

157. Calzo Jerel, Blashill Aaron, Brown Tiffany, Argenal Russell Eating Disorders and Disordered Weight and Shape Control Behaviors in Sexual Minority Populations. CURRENT PSYCHIATRY REPORTS. 2017;19(8):.

158. Le Long, Barendregt Jan, Hay Phillipa, Sawyer Susan, Paxton Susan, Mihalopoulos Cathrine The modelled cost-effectiveness of cognitive dissonance for the prevention of anorexia nervosa and bulimia nervosa in adolescent girls in Australia. INTERNATIONAL JOURNAL OF EATING DISORDERS. 2017;50(7):834-841.

159. McLean Sian, Wertheim Eleanor, Masters Jennifer, Paxton Susan A pilot evaluation of a social media literacy intervention to reduce risk factors for eating disorders. INTERNATIONAL JOURNAL OF EATING DISORDERS. 2017;50(7):847-851.

160. Wade Tracey, Wilksch Simon, Paxton Susan, Byrne Susan, Austin S. Do universal media literacy programs have an effect on weight and shape concern by influencing media internalization?. INTERNATIONAL JOURNAL OF EATING DISORDERS. 2017;50(7):731-738.

161. Akers Laura, Rohde Paul, Stice Eric, Butryn Meghan, Shaw Heather Cost-effectiveness of achieving clinical improvement with a dissonance-based eating disorder prevention program. EATING DISORDERS. 2017;25(3):263-272.

162. Mills Jennifer, Vu Nicole, Manley Ron, Tse Shasha Adolescent and young adult women's opinions of common eating disorder prevention messages. EATING DISORDERS. 2017;25(3):246-262.

163. Watson Hunna, Goodman Erica, McLagan Nicole, Joyce Tara, French Elizabeth, Willan Vivienne, Egan Sarah Quality of randomized controlled trials in eating disorder prevention. INTERNATIONAL JOURNAL OF EATING DISORDERS. 2017;50(5):459-470.

164. Adametz Luise, Richter Felicitas, Strauss Bernhard, Walther Mario, Wick Katharina, Berger Uwe Long-term effectiveness of a school-based primary prevention program for anorexia nervosa: A 7-to 8-year follow-up. EATING BEHAVIORS. 2017;25(SI):42-50.

165. Breithaupt Lauren, Eickman Laura, Byrne Catherine, Fischer Sarah Enhancing empowerment in eating disorder prevention: Another examination of the REbeL peer education model. EATING BEHAVIORS. 2017;25(SI):38-41.

166. Lipson Sarah, Jones J., Taylor C., Wilfley Denise, Eichen Dawn, Fitzsimmons-Craft Ellen, Eisenberg Daniel Understanding and promoting treatment-seeking for eating disorders and body image concerns on college campuses through online screening, prevention and intervention. EATING BEHAVIORS. 2017;25(SI):68-73.

167. Le Long, Barendregt Jan, Hay Phillipa, Mihalopoulos Cathrine Prevention of eating disorders: A systematic review and meta-analysis. CLINICAL PSYCHOLOGY REVIEW. 2017;53():46-58.

168. Mora Marisol, Penelo Eva, Roses Rocio, Gonzalez Marcela, Espinoza Paola, Devi Josep, Raich Rosa Pilot assessment of two disordered eating prevention programs. Preliminary findings on maladaptive beliefs related to eating disorders. EATING BEHAVIORS. 2017;25(SI):51-57.

169. Sanchez-Carracedo David, Carretero Cristina, Conesa Alfons Roundtable on the Prevention of Eating Disorders: The Catalan public policy initiative. EATING BEHAVIORS. 2017;25(SI):15-17.

170. Wilksch Simon How can we improve dissemination of universal eating disorder risk reduction programs?. EATING BEHAVIORS. 2017;25(SI):58-61.

171. Bar Rachel, Cassin Stephanie, Dionne Michelle The long-term impact of an eating disorder prevention program for professional ballet school students: A 15-year follow-up study. EATING DISORDERS. 2017;25(5):375-387.

172. Cook-Cottone Catherine, Talebkhah Kellie, Guyker Wendy, Keddie Emily A controlled trial of a yoga-based prevention program targeting eating disorder risk factors among middle school females. EATING DISORDERS. 2017;25(5):392-405.

173. Piran Niva On synchronicity, passing the torch, and the task of prevention. EATING DISORDERS. 2017;25(5):388-391.

174. Wilksch Simon, Paxton Susan, Byrne Susan, Austin S., O'Shea Anne, Wade Tracey Outcomes of three universal eating disorder risk reduction programs by participants with higher and lower baseline shape and weight concern. INTERNATIONAL JOURNAL OF EATING DISORDERS. 2017;50(1):66-75.

175. Shaw Heather, Rohde Paul, Stice Eric Participant feedback from peer-led, clinician-led, and internet-delivered eating disorder prevention interventions. INTERNATIONAL JOURNAL OF EATING DISORDERS. 2016;49(12):1087-1092.

176. Ishak Sharifah, Chin Yit, Taib Mohd., Shariff Zalilah School-based intervention to prevent overweight and disordered eating in secondary school Malaysian adolescents: a study protocol. BMC PUBLIC HEALTH. 2016;16():.

177. Perez Marisol, Ohrt Tara, Bruening Amanda The effects of different recruitment and incentive strategies for body acceptance programs on college women. EATING DISORDERS. 2016;24(5):383-392.

178. Watson Hunna, Joyce Tara, French Elizabeth, Willan Vivienne, Kane Robert, Tanner-Smith Emily, McCormack Julie, Dawkins Hayley, Hoiles Kimberley, Egan Sarah Prevention of Eating Disorders: A Systematic Review of Randomized, Controlled Trials. INTERNATIONAL JOURNAL OF EATING DISORDERS. 2016;49(9):833-862.

179. Atkinson Melissa, Wade Tracey Does mindfulness have potential in eating disorders prevention? A preliminary controlled trial with young adult women. EARLY INTERVENTION IN PSYCHIATRY. 2016;10(3):234-245.

180. Johnson Catherine, Burke Christine, Brinkman Sally, Wade Tracey Effectiveness of a school-based mindfulness program for transdiagnostic prevention in young adolescents. BEHAVIOUR RESEARCH AND THERAPY. 2016;81():1-11.

181. Kilpela Lisa, Blomquist Kerstin, Verzijl Christina, Wilfred Salome, Beyl Robbie, Becker Carolyn The body project 4 all: A pilot randomized controlled trial of a mixed-gender dissonance-based body image program. INTERNATIONAL JOURNAL OF EATING DISORDERS. 2016;49(6):591-602.

182. Hart Laura, Damiano Stephanie, Paxton Susan Confident Body, Confident Child: A Randomized Controlled Trial Evaluation of a Parenting Resource for Promoting Healthy Body Image and Eating Patterns in 2-to 6-Year-Old Children. INTERNATIONAL JOURNAL OF EATING DISORDERS. 2016;49(5):458-472.

183. Taylor C., Kass Andrea, Trockel Mickey, Cunning Darby, Weisman Hannah, Bailey Jakki, Sinton Meghan, Aspen Vandana, Schecthman Kenneth, Jacobi Corinna, Wilfley Denise Reducing Eating Disorder Onset in a Very High Risk Sample With Significant Comorbid Depression: A Randomized Controlled Trial. JOURNAL OF CONSULTING AND CLINICAL PSYCHOLOGY. 2016;84(5):402-414.

184. Bar Rachel, Cassin Stephanie, Dionne Michelle Eating disorder prevention initiatives for athletes: A review. EUROPEAN JOURNAL OF SPORT SCIENCE. 2016;16(3):325-335.

185. Castillo Irais, Solano Santos, Sepulveda Ana Prevention program for disordered eating and obesity among Mexican university students. BEHAVIORAL PSYCHOLOGY-PSICOLOGIA CONDUCTUAL. 2016;24(1):5-28.

186. Tirlea Loredana, Truby Helen, Haines Terry Pragmatic, Randomized Controlled Trials of the Girls on the Go! Program to Improve Self-Esteem in Girls. AMERICAN JOURNAL OF HEALTH PROMOTION. 2016;30(4):231-241.

187. Fitzsimmons-Craft Ellen, Ciao Anna, Accurso Erin A naturalistic examination of social comparisons and disordered eating thoughts, urges, and behaviors in college women. INTERNATIONAL JOURNAL OF EATING DISORDERS. 2016;49(2):143-152.

188. Sanchez-Carracedo David, Fauquet Jordi, Lopez-Guimera Gemma, Leiva David, Punti Joaquim, Trepat Esther, Pamias Montserrat, Palao Diego The MABIC project: An effectiveness trial for reducing risk factors for eating disorders. BEHAVIOUR RESEARCH AND THERAPY. 2016;77():23-33.

189. Cohn Leigh, Murray Stuart, Walen Andrew, Wooldridge Tom Including the excluded: Males and gender minorities in eating disorder prevention. EATING DISORDERS. 2016;24(1, SI):114-120.

190. Corning Alexandra, Heibel Haley Re-thinking eating disorder prevention: The case for prioritizing the promotion of healthy identity development. EATING DISORDERS. 2016;24(1, SI):106-113.

191. McVey Gail Building partnerships with prevention experts targeting other mental health problems. EATING DISORDERS. 2016;24(1, SI):63-70.

192. Melioli Tiffany, Bauer Stephanie, Franko Debra, Moessner Markus, Ozer Fikret, Chabrol Henri, Rodgers Rachel Reducing Eating Disorder Symptoms and Risk Factors Using the Internet: A Meta-Analytic Review. INTERNATIONAL JOURNAL OF EATING DISORDERS. 2016;49(1):19-31.

193. Shaw Heather, Stice Eric The implementation of evidence-based eating disorder prevention programs. EATING DISORDERS. 2016;24(1, SI):71-78.

194. Stice Eric, Yokum Sonja, Waters Allison Dissonance-Based Eating Disorder Prevention Program Reduces Reward Region Response to Thin Models; How Actions Shape Valuation. PLOS ONE. 2015;10(12):.

195. Greif Rebecca, Becker Carolyn, Hildebrandt Tom Reducing Eating Disorder Risk Factors: A Pilot Effectiveness Trial of a Train-the-Trainer Approach to Dissemination and Implementation. INTERNATIONAL JOURNAL OF EATING DISORDERS. 2015;48(8):1122-1131.

196. Linville Deanna, Cobb Erin, Lenee-Bluhm Tracy, Lopez-Zeron Gabriela, Gau Jeff, Stice Eric Effectiveness of an eating disorder preventative intervention in primary care medical settings. BEHAVIOUR RESEARCH AND THERAPY. 2015;75():32-39.

197. Atkinson Melissa, Wade Tracey Mindfulness-based prevention for eating disorders: A school-based cluster randomized controlled study. INTERNATIONAL JOURNAL OF EATING DISORDERS. 2015;48(7):1024-1037.

198. Brown Tiffany, Keel Pamela A randomized controlled trial of a peer co-led dissonance-based eating disorder prevention program for gay men. BEHAVIOUR RESEARCH AND THERAPY. 2015;74():1-10.

199. Diedrichs Phillippa, Atkinson Melissa, Steer Rebecca, Garbett Kirsty, Rumsey Nichola, Halliwell Emma Effectiveness of a brief school-based body image intervention `<i>Dove Confident Me: Single Session</i>' when delivered by teachers and researchers: Results from a cluster randomised controlled trial. BEHAVIOUR RESEARCH AND THERAPY. 2015;74():94-104.

200. Goldschmidt Andrea, Wall Melanie, Loth Katie, Neumark-Sztainer Dianne Risk Factors for Disordered Eating in Overweight Adolescents and Young Adults. JOURNAL OF PEDIATRIC PSYCHOLOGY. 2015;40(10):1048-1055.

201. Ciao Anna, Latner Janet, Brown Krista, Ebneter Daria, Becker Carolyn Effectiveness of a peer-delivered dissonance-based program in reducing eating disorder risk factors in high school girls. INTERNATIONAL JOURNAL OF EATING DISORDERS. 2015;48(6):779-784.

202. Rohde Paul, Shaw Heather, Butryn Meghan, Stice Eric Assessing program sustainability in an eating disorder prevention effectiveness trial delivered by college clinicians. BEHAVIOUR RESEARCH AND THERAPY. 2015;72():1-8.

203. Stice Eric, Rohde Paul, Butryn Meghan, Shaw Heather, Marti C. Effectiveness trial of a selective dissonance-based eating disorder prevention program with female college students: Effects at 2-and 3-year follow-up. BEHAVIOUR RESEARCH AND THERAPY. 2015;71():20-26.

204. Weigel Angelika, Gumz Antje, Kaestner Denise, Romer Georg, Wegscheider Karl, Loewe Bernd Prevention and Treatment of Eating Disorders: The Health Care Network Anorexia and Bulimia nervosa. PSYCHIATRISCHE PRAXIS. 2015;42(1):S30-S34.

205. Wilksch S., Paxton S., Byrne S., Austin S., McLean S., Thompson K., Dorairaj K., Wade T. Prevention Across the Spectrum: a randomized controlled trial of three programs to reduce risk factors for both eating disorders and obesity. PSYCHOLOGICAL MEDICINE. 2015;45(9):1811-1823.

206. Gonzalez Marcela, Mora Marisol, Penelo Eva, Goddard Elizabeth, Treasure Janet, Raich Rosa Gender differences found in a qualitative study of a disordered eating prevention programme: What do boys have to say?. JOURNAL OF HEALTH PSYCHOLOGY. 2015;20(6, SI):858-874.

207. Horney Audra, Stice Eric, Rohde Paul An Examination of Participants Who Develop an Eating Disorder Despite Completing an Eating Disorder Prevention Program: Implications for Improving the Yield of Prevention Efforts. PREVENTION SCIENCE. 2015;16(4):518-526.

208. Martinsen Marianne, Sherman Roberta, Thompson Ron, Sundgot-Borgen Jorunn Coaches' Knowledge and Management of Eating Disorders: A Randomized Controlled Trial. MEDICINE AND SCIENCE IN SPORTS AND EXERCISE. 2015;47(5):1070-1078.

209. Rodgers R., Franko D. Eating disorder prevention on college campuses: Recruitment challenges. EUROPEAN REVIEW OF APPLIED PSYCHOLOGY-REVUE EUROPEENNE DE PSYCHOLOGIE APPLIQUEE. 2015;65(3):125-131.

210. Hart Laura, Cornell Chelsea, Damiano Stephanie, Paxton Susan Parents and Prevention: A Systematic Review of Interventions Involving Parents that Aim to Prevent Body Dissatisfaction or Eating Disorders. INTERNATIONAL JOURNAL OF EATING DISORDERS.

2015;48(2):157-169.

211. Weigel Angelika, Gumz Antje, Uhlenbusch Natalie, Wegscheider Karl, Romer Georg, Loewe Bernd Preventing eating disorders with an interactive gender-adapted intervention program in schools: Study protocol of a randomized controlled trial. BMC PSYCHIATRY. 2015;15():.

212. Wilksch Simon School-based eating disorder prevention: a pilot effectiveness trial of teacher-delivered <i>Media Smart</i>. EARLY INTERVENTION IN PSYCHIATRY. 2015;9(1):21-28.

213. Kroshus Emily, Kubzansky Laura, Goldman Roberta, Austin S. Anti-Dieting Advice From Teammates: A Pilot Study of the Experience of Female Collegiate Cross Country Runners. EATING DISORDERS. 2015;23(1):31-44.

214. Lindenberg Katajun, Kordy Hans Efficacy of an Internet-Delivered Tiered Strategy for Eating Disorder Prevention in High School Students. KINDHEIT UND ENTWICKLUNG. 2015;24(1):55-63.

215. Kass Andrea, Trockel Mickey, Safer Debra, Sinton Meghan, Cunning Darby, Rizk Marianne, Genkin Brooke, Weisman Hannah, Bailey Jakki, Jacobi Corinna, Wilfley Denise, Taylor C. Internet-based preventive intervention for reducing eating disorder risk: A randomized controlled trial comparing guided with unguided self-help. BEHAVIOUR RESEARCH AND THERAPY. 2014;63():90-98.

216. Stice Eric, Durant Shelley, Rohde Paul, Shaw Heather Effects of a Prototype Internet Dissonance-Based Eating Disorder Prevention Program at 1-and 2-Year Follow-Up. HEALTH PSYCHOLOGY. 2014;33(12):1558-1567.

217. Voelker Ulrike, Jacobi Corinna, Trockel Mickey, Taylor C. Moderators and mediators of outcome in Internet-based indicated prevention for eating disorders. BEHAVIOUR RESEARCH AND THERAPY. 2014;63():114-121.

218. Stewart T., Plasencia M., Han H., Jackson H., Becker C. Moderators and predictors of response to eating disorder risk factor reduction programs in collegiate female athletes. PSYCHOLOGY OF SPORT AND EXERCISE. 2014;15(6, SI):713-720.

219. Butryn Meghan, Rohde Paul, Marti C., Stice Eric Do participant, facilitator, or group factors moderate effectiveness of the <i>Body Project</i>? Implications for dissemination. BEHAVIOUR RESEARCH AND THERAPY. 2014;61():142-149.

220. Tanofsky-Kraff Marian, Shomaker Lauren, Wilfley Denise, Young Jami, Sbrocco Tracy, Stephens Mark, Ranzenhofer Lisa, Elliott Camden, Brady Sheila, Radin Rachel, Vannucci Anna, Bryant Edny, Osborn Robyn, Berger Sarah, Olsen Cara, Kozlosky Merel, Reynolds James, Yanovski Jack Targeted prevention of excess weight gain and eating disorders in high-risk adolescent girls: a randomized controlled trial. AMERICAN JOURNAL OF CLINICAL NUTRITION. 2014;100(4):1010-1018.

221. Berger U., Schaefer J., Wick K., Brix C., Bormann B., Sowa M., Schwartze D., Strauss B. Effectiveness of Reducing the Risk of Eating-Related Problems Using the German School-Based Intervention Program, ``Torera{''}, for Preadolescent Boys and Girls. PREVENTION SCIENCE. 2014;15(4):557-569.

222. Ciao Anna, Loth Katie, Neumark-Sztainer Dianne Preventing Eating Disorder Pathology: Common and Unique Features of Successful Eating Disorders Prevention Programs. CURRENT PSYCHIATRY REPORTS. 2014;16(7):.

223. Jones Megan, Kass Andrea, Trockel Mickey, Glass Alan, Wilfley Denise, Taylor C. A Population-Wide Screening and Tailored Intervention Platform for Eating Disorders on College Campuses: The <i>Healthy Body Imag</i>e Program. JOURNAL OF AMERICAN COLLEGE HEALTH. 2014;62(5):351-356.

224. Rohde Paul, Auslander Beth, Shaw Heather, Raineri Kate, Gau Jeff, Stice Eric Dissonance-based Prevention of Eating Disorder Risk Factors in Middle School Girls: Results from Two Pilot Trials. INTERNATIONAL JOURNAL OF EATING DISORDERS. 2014;47(5):483-494.

225. Goldschmidt Andrea, Wall Melanie, Loth Katie, Bucchianeri Michaela, Neumark-Sztainer Dianne The Course of Binge Eating From Adolescence to Young Adulthood. HEALTH PSYCHOLOGY. 2014;33(5):457-460.

226. Musiat Peter, Conrod Patricia, Treasure Janet, Tylee Andre, Williams Chris, Schmidt Ulrike Targeted Prevention of Common Mental Health Disorders in University Students: Randomised Controlled Trial of a Transdiagnostic Trait-Focused Web-Based Intervention. PLOS ONE. 2014;9(4):.

227. Stice Eric, Marti C., Cheng Zhen Effectiveness of a dissonance-based eating disorder prevention program for ethnic groups in two randomized controlled trials. BEHAVIOUR RESEARCH AND THERAPY. 2014;55():54-64.

228. Martinsen Marianne, Bahr Roald, Borresen Runi, Holme Ingar, Pensgaard Anne, Sundgot-Borgen Jorunn Preventing Eating Disorders among Young Elite Athletes: A Randomized Controlled Trial. MEDICINE AND SCIENCE IN SPORTS AND EXERCISE. 2014;46(3):435-447.

229. Jones Megan, Lynch Katherine, Kass Andrea, Burrows Amanda, Williams Joanne, Wilfley Denise, Taylor C. Healthy Weight Regulation and Eating Disorder Prevention in High School Students: A Universal and Targeted Web-Based Intervention. JOURNAL OF MEDICAL INTERNET RESEARCH. 2014;16(2):.

230. DeBate Rita, Severson Herbert, Cragun Deborah, Bleck Jennifer, Gau Jeff, Merrell Laura, Cantwell Carley, Christiansen Steve, Koerber Anne, Tomar Scott, Brown Kelli, Tedesco Lisa, Hendricson William, Taris Mark Randomized Trial of Two e-Learning Programs for Oral Health Students on Secondary Prevention of Eating Disorders. JOURNAL OF DENTAL EDUCATION. 2014;78(1):5-15.

231. Runfola Cristin The Body Project: A Dissonance-Based Eating Disorder Prevention Intervention, Updated Edition-Programs ThatWork. EATING DISORDERS. 2014;22(3):275-277.

232. Serdar Kasey, Kelly Nichole, Palmberg Allison, Lydecker Janet, Thornton Laura, Tully Carrie, Mazzeo Suzanne Comparing Online and Face-to-Face Dissonance-Based Eating Disorder Prevention. EATING DISORDERS. 2014;22(3):244-260.

233. Wilksch Simon, Wade Tracey Depression as a moderator of benefit from Media Smart: A school-based eating disorder prevention program. BEHAVIOUR RESEARCH AND THERAPY. 2014;52():64-71.

234. Wilksch Simon Where Did Universal Eating Disorder Prevention Go?. EATING DISORDERS. 2014;22(2):184-192.

235. Stice Eric, Butryn Meghan, Rohde Paul, Shaw Heather, Marti C. An effectiveness trial of a new enhanced dissonance eating disorder prevention program among female college

students. BEHAVIOUR RESEARCH AND THERAPY. 2013;51(12):862-871.

236. Sanchez-Carracedo David, Lopez-Guimera Gemma, Fauquet Jordi, Ramon Barrada Juan, Pamias Montserrat, Punti Joaquim, Querol Mireia, Trepat Esther A school-based program implemented by community providers previously trained for the prevention of eating and weight-related problems in secondary-school adolescents: the MABIC study protocol. BMC PUBLIC HEALTH. 2013;13():.

237. Wilksch Simon, Starkey Karina, Gannoni Anne, Kelly Tania, Wade Tracey Interactive programme to enhance protective factors for eating disorders in girls with type 1 diabetes. EARLY INTERVENTION IN PSYCHIATRY. 2013;7(3):315-321.

238. Bauer Stephanie, Moessner Markus Harnessing the power of technology for the treatment and prevention of eating disorders. INTERNATIONAL JOURNAL OF EATING DISORDERS. 2013;46(5, SI):508-515.

239. Bulik Cynthia Are we really paddling as fast as we can? reflections on why eating disorders treatment and research always seem to be one step behind: Commentary on hay, mitchell, and stice \& becker: Prevention and treatment. INTERNATIONAL JOURNAL OF EATING DISORDERS. 2013;46(5, SI):489-491.

240. Ohlmer Ricarda, Jacobi Corinna, Taylor Craig Preventing Symptom Progression in Women at Risk for AN: Results of a Pilot Study. EUROPEAN EATING DISORDERS REVIEW. 2013;21(4):323-329.

241. Stice Eric, Becker Carolyn, Yokum Sonja Eating disorder prevention: Current evidence-base and future directions. INTERNATIONAL JOURNAL OF EATING DISORDERS. 2013;46(5, SI):478-485.

242. Varnado-Sullivan Paula, Parr Francoise, O'Grady Megan, Savoy Sarah Educators' views of eating disorder prevention programs. EATING AND WEIGHT DISORDERS-STUDIES ON ANOREXIA BULIMIA AND OBESITY. 2013;18(2):143-150.

243. Yager Zali, Diedrichs Phillippa, Ricciardelli Lina, Halliwell Emma What works in secondary schools? A systematic review of classroom-based body image programs. BODY IMAGE. 2013;10(3):271-281.

244. Stice Eric, Rohde Paul, Durant Shelley, Shaw Heather, Wade Emily Effectiveness of peer-led dissonance-based eating disorder prevention groups: Results from two randomized pilot trials. BEHAVIOUR RESEARCH AND THERAPY. 2013;51(4-5):197-206.

245. Gonzalez Marcela, Mora Marisol, Penelo Eva, Goddard Elizabeth, Treasure Janet, Raich Rosa Qualitative findings in a long-term disordered eating prevention programme follow-up with school-going girls. JOURNAL OF HEALTH PSYCHOLOGY. 2013;18(4):587-598.

246. Espinoza Paola, Penelo Eva, Raich Rosa Prevention programme for eating disturbances in adolescents. Is their effect on body image maintained at 30 months later?. BODY IMAGE. 2013;10(2):175-181.

247. Mueller Sina, Stice Eric Moderators of the intervention effects for a dissonance-based eating disorder prevention program; results from an amalgam of three randomized trials. BEHAVIOUR RESEARCH AND THERAPY. 2013;51(3):128-133.

248. Stice Eric, Rohde Paul, Shaw Heather, Marti C. Efficacy Trial of a Selective Prevention Program Targeting Both Eating Disorders and Obesity Among Female College Students: 1- and 2-Year Follow-Up Effects. JOURNAL OF CONSULTING AND CLINICAL PSYCHOLOGY. 2013;81(1):183-189.

249. Van Diest Ashley, Perez Marisol Exploring the integration of thin-ideal internalization and self-objectification in the prevention of eating disorders. BODY IMAGE. 2013;10(1):16-25.

250. Sanchez-Carracedo David, Neumark-Sztainer Dianne, Lopez-Guimera Gemma Integrated prevention of obesity and eating disorders: barriers, developments and opportunities. PUBLIC HEALTH NUTRITION. 2012;15(12):2295-2309.

251. Nicholls Dasha, Yi Irene Early intervention in eating disorders: a parent group approach. EARLY INTERVENTION IN PSYCHIATRY. 2012;6(4):357-367.

252. Austin S. A public health approach to eating disorders prevention: It's time for public health professionals to take a seat at the table. BMC PUBLIC HEALTH. 2012;12():.

253. Stice Eric, Rohde Paul, Durant Shelley, Shaw Heather A Preliminary Trial of a Prototype Internet Dissonance-Based Eating Disorder Prevention Program for Young Women With

Body Image Concerns. JOURNAL OF CONSULTING AND CLINICAL PSYCHOLOGY. 2012;80(5):907-916.

254. Sonneville K., Calzo J., Horton N., Haines J., Austin S., Field A. Body satisfaction, weight gain and binge eating among overweight adolescent girls. INTERNATIONAL JOURNAL OF OBESITY. 2012;36(7):944-949.

255. Ramirez Ana, Perez Marisol, Taylor Aaron Preliminary examination of a couple-based eating disorder prevention program. BODY IMAGE. 2012;9(3):324-333.

256. Jones Megan, Voelker Ulrike, Lock James, Taylor C., Jacobi Corinna Family-based Early Intervention for Anorexia Nervosa. EUROPEAN EATING DISORDERS REVIEW. 2012;20(3):e137-e143.

257. Stice Eric, Rohde Paul, Gau Jeff, Shaw Heather Effect of a Dissonance-Based Prevention Program on Risk for Eating Disorder Onset in the Context of Eating Disorder Risk Factors. PREVENTION SCIENCE. 2012;13(2):129-139.

258. Jacobi Corinna, Voelker Ulrike, Trockel Mickey, Taylor Craig Effects of an Internet-based intervention for subthreshold eating disorders: A randomized controlled trial. BEHAVIOUR RESEARCH AND THERAPY. 2012;50(2):93-99.

259. Stice Eric, Rohde Paul, Shaw Heather, Marti C. Efficacy Trial of a Selective Prevention Program Targeting Both Eating Disorder Symptoms and Unhealthy Weight Gain Among Female College Students. JOURNAL OF CONSULTING AND CLINICAL PSYCHOLOGY. 2012;80(1):164-170.

260. Beintner Ina, Jacobi Corinna, Taylor Craig Effects of an Internet-based Prevention Programme for Eating Disorders in the USA and Germany u A Meta-analytic Review. EUROPEAN EATING DISORDERS REVIEW. 2012;20(1):1-8.

261. Contreras Jordan Onofre, Gil Madrona Pedro, Garcia Lopez Luis, Fernandez Bustos Juan, Pastor Vicedo Juan Changes in Body Image Perception Brought about by a Physical Education Program. REVISTA DE EDUCACION. 2012;(357):281-303.

262. Thompson Carmen, Russell-Mayhew Shelly, Saraceni Reana Evaluating the Effects of a Peer-Support Model: Reducing Negative Body Esteem and Disordered Eating Attitudes and

Behaviours in Grade Eight Girls. EATING DISORDERS. 2012;20(2):113-126.

263. Voelker U., Jacobi C., Taylor C. Adaptation and evaluation of an Internet-based prevention program for eating disorders in a sample of women with subclinical eating disorder symptoms: A pilot study. EATING AND WEIGHT DISORDERS-STUDIES ON ANOREXIA BULIMIA AND OBESITY. 2011;16(4):E270-E273.

264. Aspen V., Stein R., Cooperberg J., Manwaring J., Barch D., Wilfley D. Selective processing of body image words in women at-risk for developing an eating disorder: A preliminary study. EATING AND WEIGHT DISORDERS-STUDIES ON ANOREXIA BULIMIA AND OBESITY. 2011;16(3):E199-E203.

265. Stice Eric, Rohde Paul, Shaw Heather, Gau Jeff An Effectiveness Trial of a Selected Dissonance-Based Eating Disorder Prevention Program for Female High School Students: Long-Term Effects. JOURNAL OF CONSULTING AND CLINICAL PSYCHOLOGY. 2011;79(4):500-508.

266. Gonzalez Marcela, Penelo Eva, Gutierrez Teresa, Raich Rosa Disordered Eating Prevention Programme in Schools: A 30-Month Follow-up. EUROPEAN EATING DISORDERS REVIEW. 2011;19(4):349-356.

267. Stice Eric, Marti C., Rohde Paul, Shaw Heather Testing Mediators Hypothesized to Account for the Effects of a Dissonance-Based Eating Disorder Prevention Program Over Longer Term Follow-Up. JOURNAL OF CONSULTING AND CLINICAL PSYCHOLOGY. 2011;79(3):398-405.

268. Torres-McGehee Toni, Green James, Leaver-Dunn Deidre, Leeper James, Bishop Phillip, Richardson Mark ATTITUDE AND KNOWLEDGE CHANGES IN COLLEGIATE DANCERS FOLLOWING A SHORT-TERM, TEAM-CENTERED PREVENTION PROGRAM ON EATING DISORDERS. PERCEPTUAL AND MOTOR SKILLS. 2011;112(3):711-725.

269. Lopez-Guimera Gemma, Sanchez-Carracedo David, Fauquet Jordi, Portell Mariona, Raich Rosa Impact of a School-Based Disordered Eating Prevention Program in Adolescent Girls: General and Specific Effects Depending on Adherence to the Interactive Activities. SPANISH JOURNAL OF PSYCHOLOGY. 2011;14(1):293-303.

270. McMillan Whitney, Stice Eric, Rohde Paul High- and Low-Level Dissonance-Based Eating Disorder Prevention Programs With Young Women With Body Image Concerns: An

Experimental Trial. JOURNAL OF CONSULTING AND CLINICAL PSYCHOLOGY. 2011;79(1):129-134.

271. Marchand Erica, Stice Eric, Rohde Paul, Becker Carolyn Moving from efficacy to effectiveness trials in prevention research. BEHAVIOUR RESEARCH AND THERAPY. 2011;49(1):32-41.

272. Yager Zali, O'Dea Jennifer A controlled intervention to promote a healthy body image, reduce eating disorder risk and prevent excessive exercise among trainee health education and physical education teachers. HEALTH EDUCATION RESEARCH. 2010;25(5):841-852.

273. Becker Carolyn, Wilson Chantale, Williams Allison, Kelly Mackenzie, McDaniel Leda, Elmquist Joanna Peer-facilitated cognitive dissonance versus healthy weight eating disorders prevention: A randomized comparison. BODY IMAGE. 2010;7(4):280-288.

274. Perez Marisol, Becker Carolyn, Ramirez Ana Transportability of an empirically supported dissonance-based prevention program for eating disorders. BODY IMAGE. 2010;7(3):179-186.

275. Wilksch Simon Universal school-based eating disorder prevention: Benefits to both high- and low-risk participants on the core cognitive feature of eating disorders. CLINICAL PSYCHOLOGIST. 2010;14(2):62-69.

276. Stice Eric, Rohde Paul, Gau Jeff, Shaw Heather An Effectiveness Trial of a Dissonance-Based Eating Disorder Prevention Program for High-Risk Adolescent Girls. JOURNAL OF CONSULTING AND CLINICAL PSYCHOLOGY. 2009;77(5):825-834.

277. Seidel Anke, Presnell Katherine, Rosenfield David Mediators in the dissonance eating disorder prevention program. BEHAVIOUR RESEARCH AND THERAPY. 2009;47(8):645-653.

278. Presnell Katherine, Stice Eric, Seidel Anke, Madeley Mary Depression and Eating Pathology: Prospective Reciprocal Relations in Adolescents. CLINICAL PSYCHOLOGY \& PSYCHOTHERAPY. 2009;16(4, SI):357-365.

279. Canals J., Sancho C., Arija M. Influence of parent's eating attitudes on eating disorders in school adolescents. EUROPEAN CHILD \& ADOLESCENT PSYCHIATRY. 2009;18(6):353-359.

280. Wilksch Simon, Wade Tracey Reduction of Shape and Weight Concern in Young Adolescents: A 30-Month Controlled Evaluation of a Media Literacy Program. JOURNAL OF THE AMERICAN ACADEMY OF CHILD AND ADOLESCENT PSYCHIATRY. 2009;48(6):652-661.

281. Langmesser Lisa, Verscheure Susan Are Eating Disorder Prevention Programs Effective?. JOURNAL OF ATHLETIC TRAINING. 2009;44(3):304-305.

282. Loth Katie, Neumark-Sztainer Dianne, Croll Jillian Informing Family Approaches to Eating Disorder Prevention: Perspectives of Those Who Have Been There. INTERNATIONAL JOURNAL OF EATING DISORDERS. 2009;42(2):146-152.

283. Neumark-Sztainer Dianne Preventing Obesity and Eating Disorders in Adolescents: What Can Health Care Providers Do?. JOURNAL OF ADOLESCENT HEALTH. 2009;44(3):206-213.

284. Levine Michael, Murnen Sarah ``EVERYBODY KNOWS THAT MASS MEDIA ARE/ARE NOT {[}<i>pick</i> <i>one</i>] A CAUSE OF EATING DISORDERS{''}: A CRITICAL REVIEW OF EVIDENCE FOR A CAUSAL LINK BETWEEN MEDIA, NEGATIVE BODY IMAGE, AND DISORDERED EATING IN FEMALES. JOURNAL OF SOCIAL AND CLINICAL PSYCHOLOGY. 2009;28(1):9-42.

285. Shaw Heather, Stice Eric, Becker Carolyn Preventing Eating Disorders. CHILD AND ADOLESCENT PSYCHIATRIC CLINICS OF NORTH AMERICA. 2009;18(1):199+.

286. Franko Debra, George Jessica A Pilot Intervention to Reduce Eating Disorder Risk in Latina Women. EUROPEAN EATING DISORDERS REVIEW. 2008;16(6):436-441.

287. Rodriguez Rosalia, Marchand Erica, Ng Janet, Stice Eric Effects of a Cognitive Dissonance-Based Eating Disorder Prevention Program Are Similar for Asian American, Hispanic, and White Participants. INTERNATIONAL JOURNAL OF EATING DISORDERS. 2008;41(7):618-625.

288. Stice Eric, Marti Nathan, Shaw Heather, O'Neil Kelly General and Program-Specific Moderators of Two Eating Disorder Prevention Programs. INTERNATIONAL JOURNAL OF EATING DISORDERS. 2008;41(7):611-617.

289. Leon M., Diaz J., Ruiz E. A pilot study of the clinical and statistical significance of a program to reduce eating disorder risk factors in children. EATING AND WEIGHT DISORDERS-STUDIES ON ANOREXIA BULIMIA AND OBESITY. 2008;13(3):111-118.

290. Doyle Angela, Goldschmidt Andrea, Huang Christina, Winzelberg Andrew, Taylor C., Wilfley Denise Reduction of overweight and eating disorder symptoms via the Internet in adolescents: A randomized controlled trial. JOURNAL OF ADOLESCENT HEALTH. 2008;43(2):172-179.

291. Stice Eric, Shaw Heather, Becker Carolyn, Rohde Paul Dissonance-based interventions for the prevention of eating disorders: Using persuasion principles to promote health. PREVENTION SCIENCE. 2008;9(2):114-128.

292. Yager Zali, O'Dea Jennifer Prevention programs for body image and eating disorders on University campuses: a review of large, controlled interventions. HEALTH PROMOTION INTERNATIONAL. 2008;23(2):173-189.

293. Berger Uwe, Sowa Melanie, Bormann Bianca, Brix Christina, Strauss Bernhard Primary prevention of eating disorders: Characteristics of effective programmes and how to bring them to broader dissemination. EUROPEAN EATING DISORDERS REVIEW. 2008;16(3):173-183.

294. Becker Carolyn, Bull Stephanie, Schaumberg Katherine, Cauble Adele, Franco Amanda Effectiveness of peer-led eating disorders prevention: A replication trial. JOURNAL OF CONSULTING AND CLINICAL PSYCHOLOGY. 2008;76(2):347-354.

295. Manwaring Jamie, Bryson Susan, Goldschmidt Andrea, Winzelberg Andrew, Luce Kristine, Cunning Darby, Wilfley Denise, Taylor C. Do adherence variables predict outcome in an online program for the prevention of eating disorders?. JOURNAL OF CONSULTING AND CLINICAL PSYCHOLOGY. 2008;76(2):341-346.

296. Stice Eric, Marti C., Spoor Sonja, Presnell Katherine, Shaw Heather Dissonance and healthy weight eating disorder prevention programs: Long-term effects from a randomized efficacy trial. JOURNAL OF CONSULTING AND CLINICAL PSYCHOLOGY. 2008;76(2):329-

340.

297. Scime Melinda, Cook-Cottone Catherine Primary prevention of eating disorders: A constructivist integration of mind and body strategies. INTERNATIONAL JOURNAL OF EATING DISORDERS. 2008;41(2):134-142.

298. Becker Carolyn, Ciao Anna, Smith Lisa Moving from efficacy to effectiveness in eating disorders prevention: The sorority body image program. COGNITIVE AND BEHAVIORAL PRACTICE. 2008;15(1):18-27.

299. Neumark-Sztainer Dianne, Eisenberg Marla, Fulkerson Jayne, Story Mary, Larson Nicole Family meals and disordered eating in adolescents - Longitudinal findings from project EAT. ARCHIVES OF PEDIATRICS \& ADOLESCENT MEDICINE. 2008;162(1):17-22.

300. Raabe K. Girl-specific prevention of Eating Disorder. PSYCHOTHERAPEUT. 2008;53(1):71-72.

301. Smith Ariane, Petrie Trent Reducing the Risk of Disordered Eating Among Female Athletes: A Test of Alternative Interventions. JOURNAL OF APPLIED SPORT PSYCHOLOGY. 2008;20(4):392-407.

302. Stock Suzanne, Miranda Charmaine, Evans Stacey, Plessis Suzanne, Ridley Julia, Yeh Sophia, Chanoine Jean-Pierre Healthy Buddies: A novel, peer-led health promotion program for the prevention of obesity and eating disorders in children in elementary school. PEDIATRICS. 2007;120(4):e1059-E1068.

303. Sepulveda A., Carrobles J., Gandarillas A., Poveda J., Pastor V. Prevention program for disturbed eating and body dissatisfaction in a Spanish university population: A pilot study. BODY IMAGE. 2007;4(3):317-328.

304. Berger Uwe, Joseph Andrea, Sowa Melanie, Strauss Bernhard The Barbie-matrix: Effectiveness of a school based German program for the primary prevention of anorexia nervosa developed for girls up to the age of 12. PSYCHOTHERAPIE PSYCHOSOMATIK MEDIZINISCHE PSYCHOLOGIE. 2007;57(6):248-255.

305. McVey Gail, Tweed Stacey, Blackmore Elizabeth Healthy Schools-Healthy Kids: A controlled evaluation of a comprehensive universal eating disorder prevention program.

BODY IMAGE. 2007;4(2):115-136.

306. Zerbe Kathryn Eating disorders in the 21st century: identification, management, and prevention in obstetrics and gynecology. BEST PRACTICE \& RESEARCH CLINICAL OBSTETRICS \& GYNAECOLOGY. 2007;21(2):331-343.

307. Jacobi Corinna, Morris Lisette, Beckers Christina, Bronisch-Holtze Janina, Winter Jana, Winzelberg Andrew, Taylor Craig Maintenance of Internet-based prevention: A randomized controlled trial. INTERNATIONAL JOURNAL OF EATING DISORDERS. 2007;40(2):114-119.

308. Schwartz Marlene, Thomas Jennifer, Bohan Kristin, Vartanian Lenny Intended and unintended effects of an eating disorder educational program: Impact of presenter identity. INTERNATIONAL JOURNAL OF EATING DISORDERS. 2007;40(2):187-192.

309. Stice Eric, Presnell Katherine, Gau Jeff, Shaw Heather Testing mediators of intervention effects in randomized controlled trials: An evaluation of two eating disorder prevention programs. JOURNAL OF CONSULTING AND CLINICAL PSYCHOLOGY. 2007;75(1):20-32.

310. Stice Eric, Shaw Heather, Marti C. A meta-analytic review of eating disorder prevention programs: Encouraging findings. ANNUAL REVIEW OF CLINICAL PSYCHOLOGY. 2007;3():207-231.

311. Haines Jess, Neumark-Sztainer Dianne Prevention of obesity and eating disorders: a consideration of shared risk factors. HEALTH EDUCATION RESEARCH. 2006;21(6):770-782.

312. Haines Jess, Neumark-Sztainer Dianne, Perry Cheryl, Hannan Peter, Levine Michael VIK (Very Important Kids): a school-based program designed to reduce teasing and unhealthy weight-control behaviors. HEALTH EDUCATION RESEARCH. 2006;21(6):884-895.

313. Pokrajac-Bulian A., Zivcic-Becirevic I., Calugi S., Dalle Grave R. School prevention program for eating disorders in Croatia: A controlled study with six months of follow-up. EATING AND WEIGHT DISORDERS-STUDIES ON ANOREXIA BULIMIA AND OBESITY. 2006;11(4):171-178.

314. Becker Carolyn, Smith Lisa, Ciao Anna Peer-facilitated eating disorder prevention: A randomized effectiveness trial of cognitive dissonance and media advocacy. JOURNAL OF

COUNSELING PSYCHOLOGY. 2006;53(4):550-555.

315. Taylor C., Bryson Susan, Luce Kristine, Cunning Darby, Doyle Angela, Abascal Liana, Rockwell Roxanne, Dev Pavarti, Winzelberg Andrew, Wilfley Denise Prevention of eating disorders in at-risk college-age women. ARCHIVES OF GENERAL PSYCHIATRY. 2006;63(8):881-888.

316. Taylor C., Bryson Susan, Doyle Angela, Luce Kristine, Cunning Darby, Abascal Liana, Rockwell Roxanne, Field Alison, Striegel-Moore Ruth, Winzelberg Andrew, Wilfley Denise The adverse effect of negative comments about weight and shape from family and siblings on women at high risk for eating disorders. PEDIATRICS. 2006;118(2):731-738.

317. Wilksch Simon, Tiggemann Marika, Wade Tracey Impact of interactive school-based media literacy lessons for reducing internalization of media ideals in young adolescent girls and boys. INTERNATIONAL JOURNAL OF EATING DISORDERS. 2006;39(5):385-393.

318. Pineda Garcia Gisela, Gomez-Peresmitre Gilda Eating disorders prevention program:: A pilot study based on cognitive dissonance theory. REVISTA MEXICANA DE PSICOLOGIA. 2006;23(1):87-95.

319. Varnado-Sullivan Paula, Horton Rachael Acceptability of programs for the prevention of eating Disorders. JOURNAL OF CLINICAL PSYCHOLOGY. 2006;62(6):687-703.

320. Berger Uwe Primary prevention of eating disorders. PSYCHOTHERAPEUT. 2006;51(3):187+.

321. Stice E, Orjada K, Tristan J Trial of a psychoeducational eating disturbance intervention for college women: A replication and extension. INTERNATIONAL JOURNAL OF EATING DISORDERS. 2006;39(3):233-239.

322. Stice Eric, Shaw Heather, Burton Emily, Wade Emily Dissonance and healthy weight eating disorder prevention programs: A randomized efficacy trial. JOURNAL OF CONSULTING AND CLINICAL PSYCHOLOGY. 2006;74(2):263-275.

323. Cook-Cottone C The attuned representation model for the primary prevention of eating disorders: An overview for school psychologists. PSYCHOLOGY IN THE SCHOOLS.

2006;43(2):223-230.

324. Elliot DL, Moe EL, Goldberg L, DeFrancesco CA, Durham MB, Hix-Small H Definition and outcome of a curriculum to prevent disordered eating and body-shaping drug use. JOURNAL OF SCHOOL HEALTH. 2006;76(2):67-73.

325. Roehrig M, Thompson JK, Brannick M, Berg P Dissonance-based eating disorder prevention program: A preliminary dismantling investigation. INTERNATIONAL JOURNAL OF EATING DISORDERS. 2006;39(1):1-10.

326. Durkin SJ, Paxton SJ, Wertheim EH How do adolescent girls evaluate body dissatisfaction prevention messages?. JOURNAL OF ADOLESCENT HEALTH. 2005;37(5):381-390.

327. Franko DL, Mintz LB, Villapiano M, Green TC, Mainelli D, Folensbee L, Butler SF, Davidson MM, Hamilton E, Little D, Kearns M, Budman SH Food, mood, and attitude: Reducing risk for eating disorders in college women. HEALTH PSYCHOLOGY. 2005;24(6):567-578.

328. Neumark-Sztainer D Can we simultaneously work toward the prevention of obesity and eating disorders in children and adolescents?. INTERNATIONAL JOURNAL OF EATING DISORDERS. 2005;38(3):220-227.

329. Favaro A, Zanetti T, Huon G, Santonastaso P Engaging teachers in an eating disorder preventive intervention. INTERNATIONAL JOURNAL OF EATING DISORDERS. 2005;38(1):73-77.

330. Becker CB, Smith LM, Ciao AC Reducing eating disorder risk factors in sorority members: A randomized trial. BEHAVIOR THERAPY. 2005;36(3):245-253.

331. Luce KH, Osborne MI, Winzelberg AJ, Das S, Abascal LB, Celio AA, Wilfley DE, Stevenson D, Dev P, Taylor CB Application of an algorithm-driven protocol to simultaneously provide universal and targeted prevention programs. INTERNATIONAL JOURNAL OF EATING DISORDERS. 2005;37(3):220-226.

332. Matusek JA, Wendt SJ, Wiseman CV Dissonance thin-ideal and didactic healthy behavior eating disorder prevention programs: Results from a controlled trial.

INTERNATIONAL JOURNAL OF EATING DISORDERS. 2004;36(4):376-388.

333. Elliot DL, Goldberg L, Moe EL, DeFrancesco CA, Durham MB, Hix-Small H Preventing substance use and disordered eating - Initial outcomes of the ATHENA (Athletes Targeting Healthy Exercise and Nutrition Alternatives) Program. ARCHIVES OF PEDIATRICS \& ADOLESCENT MEDICINE. 2004;158(11):1043-1049.

334. Varnado-Sullivan PJ, Zucker N The body logic program for adolescents - A treatment manual for the prevention of eating disorders. BEHAVIOR MODIFICATION. 2004;28(6):854-875.

335. Brown JB, Winzelberg AJ, Abascal LB, Taylor CB An evaluation of an Internet-delivered eating disorder prevention program for adolescents and their parents. JOURNAL OF ADOLESCENT HEALTH. 2004;35(4):290-296.

336. Stice E, Shaw H Eating disorder prevention programs: A meta-analytic review. PSYCHOLOGICAL BULLETIN. 2004;130(2):206-227.

337. Abascal L, Brown JB, Winzelberg AJ, Dev P, Taylor CB Combining universal and targeted prevention for school-based eating disorder programs. INTERNATIONAL JOURNAL OF EATING DISORDERS. 2004;35(1):1-9.

338. Steck EL, Abrams LM, Phelps L Positive psychology in the prevention of eating disorders. PSYCHOLOGY IN THE SCHOOLS. 2004;41(1):111-117.

339. Sanderson CA, Holloway RM Who benefits from what? Drive for thinness as a moderator of responsiveness to different eating disorder prevention messages. JOURNAL OF APPLIED SOCIAL PSYCHOLOGY. 2003;33(9):1837-1861.

340. Wade TD, Davidson S, O'Dea JA A preliminary controlled evaluation of a school-based media literacy program and self-esteem program for reducing eating disorder risk factors. INTERNATIONAL JOURNAL OF EATING DISORDERS. 2003;33(4):371-383.

341. Zabinski MF, Celio AA, Jacobs MJ, Manwaring J, Wilfley DE Internet-based prevention of eating disorders. EUROPEAN EATING DISORDERS REVIEW. 2003;11(3):183-197.

342. Luce KH, Winzelberg AJ, Zabinski MF, Osborne MI Internet-delivered psychological interventions for body image dissatisfaction and disordered eating. PSYCHOTHERAPY. 2003;40(1-2, SI):148-154.

343. Ben-Tovim DI Eating disorders: outcome, prevention and treatment of eating disorders. CURRENT OPINION IN PSYCHIATRY. 2003;16(1):65-69.

344. Dalle Grave R School-based prevention programs for eating disorders - Achievements and opportunities. DISEASE MANAGEMENT \& HEALTH OUTCOMES. 2003;11(9):579-593.

345. Stice E, Trost A, Chase A Healthy weight control and dissonance-based eating disorder prevention programs: results from a controlled trial. INTERNATIONAL JOURNAL OF EATING DISORDERS. 2003;33(1):10-21.

346. Olmsted MP, Daneman D, Rydall AC, Lawson ML, Rodin G The effects of psychoeducation on disturbed eating attitudes and behavior in young women with type 1 diabetes mellitus. INTERNATIONAL JOURNAL OF EATING DISORDERS. 2002;32(2):230-239.

347. Pearson J, Goldklang D, Striegel-Moore RH Prevention of eating disorders: Challenges and opportunities. INTERNATIONAL JOURNAL OF EATING DISORDERS. 2002;31(3):233-239.

348. Irving LM, Neumark-Sztainer D Integrating the prevention, of eating disorders and obesity: Feasible or futile?. PREVENTIVE MEDICINE. 2002;34(3):299-309.

349. Stice E, Ragan J A preliminary controlled evaluation of an eating disturbance psychoeducational intervention for college students. INTERNATIONAL JOURNAL OF EATING DISORDERS. 2002;31(2):159-171.

350. Rocco PL, Ciano RP, Balestrieri M Psychoeducation in the prevention of eating disorders: An experimental approach in adolescent schoolgirls. BRITISH JOURNAL OF MEDICAL PSYCHOLOGY. 2001;74(3):351-358.

351. Zabinski MF, Wilfley DE, Pung MA, Winzelberg AJ, Eldredge K, Taylor CB An interactive Internet-based intervention for women at risk of eating disorders: A pilot study.

INTERNATIONAL JOURNAL OF EATING DISORDERS. 2001;30(2):129-137.

352. Franko DL Rethinking prevention efforts in eating disorders. COGNITIVE AND BEHAVIORAL PRACTICE. 2001;8(3):265-270.

353. Varnado-Sullivan PJ, Zucker N, Williamson DA, Reas D, Thaw J, Netemeyer SB Development and implementation of the body logic program for adolescents: A two-stage prevention program for eating disorders. COGNITIVE AND BEHAVIORAL PRACTICE. 2001;8(3):248-259.

354. Zabinski MF, Pung MA, Wilfley DE, Eppstein DL, Winzelberg AJ, Celio A, Taylor CB Reducing risk factors for eating disorders: Targeting at-risk women with a computerized psychoeducational program. INTERNATIONAL JOURNAL OF EATING DISORDERS. 2001;29(4):401-408.

355. Stice E, Chase A, Stormer S, Appel A A randomized trial of a dissonance-based eating disorder prevention program. INTERNATIONAL JOURNAL OF EATING DISORDERS. 2001;29(3):247-262.

356. Austin SB Population-based prevention of eating disorders: An application of the Rose prevention model. PREVENTIVE MEDICINE. 2001;32(3):268-283.

357. Baranowski MJ, Hetherington MM Testing the efficacy of an eating disorder prevention program. INTERNATIONAL JOURNAL OF EATING DISORDERS. 2001;29(2):119-124.

358. Stewart DA, Carter JC, Drinkwater J, Hainsworth J, Fairburn CG Modification of eating attitudes and behavior in adolescent girls: A controlled study. INTERNATIONAL JOURNAL OF EATING DISORDERS. 2001;29(2):107-118.

359. Neumark-Sztainer D, Sherwood NE, Coller T, Hannan PJ Primary prevention of disordered eating among preadolescent girls: Feasibility and short-term effect of a community-based intervention. JOURNAL OF THE AMERICAN DIETETIC ASSOCIATION. 2000;100(12):1466-1473.

360. Austin SB Prevention research in eating disorders: theory and new directions. PSYCHOLOGICAL MEDICINE. 2000;30(6):1249-1262.

361. Mussell MP, Binford RB, Fulkerson JA Eating disorders: Summary of risk factors, prevention programming, and prevention research. COUNSELING PSYCHOLOGIST. 2000;28(6):764-796.

362. Phelps L, Sapia J, Nathanson D, Nelson L An empirically supported eating disorder prevention program. PSYCHOLOGY IN THE SCHOOLS. 2000;37(5):443-452.

363. Celio AA, Winzelberg AJ, Wilfley DE, Eppstein-Herald D, Springer EA, Dev P, Taylor CB Reducing risk factors for eating disorders: Comparison of an Internet- and a classroom-delivered psychoeducational program. JOURNAL OF CONSULTING AND CLINICAL PSYCHOLOGY. 2000;68(4):650-657.

364. O'Dea JA, Abraham S Improving the body image, eating attitudes, and behaviors of young male and female adolescents: A new educational approach that focuses on self-esteem. INTERNATIONAL JOURNAL OF EATING DISORDERS. 2000;28(1):43-57.

365. Abood DA, Black DR Health education prevention for eating disorders among college female athletes. AMERICAN JOURNAL OF HEALTH BEHAVIOR. 2000;24(3):209-219.

366. Ghaderi A, Scott B Coping in dieting and eating disorders - A population-based study. JOURNAL OF NERVOUS AND MENTAL DISEASE. 2000;188(5):273-279.

367. Winzelberg AJ, Eppstein D, Eldredge KL, Wilfley D, Dasmahapatra R, Dev P, Taylor CB Effectiveness of an Internet-based program for reducing risk factors for eating disorders. JOURNAL OF CONSULTING AND CLINICAL PSYCHOLOGY. 2000;68(2):346-350.

368. Springer EA, Winzelberg AJ, Perkins R, Taylor CB Effects of a body image curriculum for college students on improved body image. INTERNATIONAL JOURNAL OF EATING DISORDERS. 1999;26(1):13-20.

369. Martz DM, Bazzini DG Eating disorders prevention programming may be failing: Evaluation of 2 one-shot programs. JOURNAL OF COLLEGE STUDENT DEVELOPMENT. 1999;40(1):32-42.

370. Santonastaso P, Zanetti T, Ferrara S, Olivotto MC, Magnavita N, Favaro A A preventive intervention program in adolescent schoolgirls: A longitudinal study. PSYCHOTHERAPY

AND PSYCHOSOMATICS. 1999;68(1):46-50.

371. Santoncini CU, Ríos JM, Peresmitré GG Perception of obesity among adolescents and its relation to abnormal eating behaviors. REVISTA INTERAMERICANA DE PSICOLOGIA. 1999;33(1):11-29.

372. Franko DL, Orosan-Weine P The prevention of eating disorders: Empirical, methodological, and conceptual considerations. CLINICAL PSYCHOLOGY-SCIENCE AND PRACTICE. 1998;5(4):459-477.

373. Rosen DS, Neumark-Sztainer D Review of options for primary prevention of eating disturbances among adolescents. JOURNAL OF ADOLESCENT HEALTH. 1998;23(6):354-363.

374. Huon GF, Braganza C, Brown LB, Ritchie JE, Roncolato WG Reflections on prevention in dieting-induced disorders. INTERNATIONAL JOURNAL OF EATING DISORDERS. 1998;23(4):455-458.

375. Friedman SS Girls in the 90s: A gender-based model for eating disorder prevention. PATIENT EDUCATION AND COUNSELING. 1998;33(3):217-224.

376. Carter JC, Stewart DA, Dunn VJ, Fairburn CG Primary prevention of eating disorders: Might it do more harm than good?. INTERNATIONAL JOURNAL OF EATING DISORDERS. 1997;22(2):167-172.

377. Mann T, NolenHoeksema S, Huang K, Burgard D, Wright A, Hanson K Are two interventions worse than none? Joint primary and secondary prevention of eating disorders in college females. HEALTH PSYCHOLOGY. 1997;16(3):215-225.

378. Joy E, Clark N, Ireland ML, Martire J, Nattiv A, Varechok S Team management of the female athlete triad .2. Optimal treatment and prevention tactics - Roundtable. PHYSICIAN AND SPORTSMEDICINE. 1997;25(4):55-\&.

379. Battle EK, Brownell KD Confronting a rising tide of eating disorders and obesity: Treatment vs prevention and policy. ADDICTIVE BEHAVIORS. 1996;21(6):755-765.

380. NeumarkSztainer D School-based programs for preventing eating disturbances. JOURNAL OF SCHOOL HEALTH. 1996;66(2):64-71.

381. REAVIS PA, ESPTEIN BA, PIOTROWICZ LM PARENTS GUIDE TO EATING DISORDERS - PREVENTION AND TREATMENT OF ANOREXIA AND BULIMIA - VALETTE,B. PSYCHIATRIC SERVICES. 1995;46(12):1299.

382. NEUMARKSZTAINER D, BUTLER R, PALTI H EATING DISTURBANCES AMONG ADOLESCENT GIRLS - EVALUATION OF A SCHOOL-BASED PRIMARY PREVENTION PROGRAM. JOURNAL OF NUTRITION EDUCATION. 1995;27(1):24-31.

383. KILLEN JD, TAYLOR CB, HAMMER LD, LITT I, WILSON DM, RICH T, HAYWARD C, SIMMONDS B, KRAEMER H, VARADY A AN ATTEMPT TO MODIFY UNHEALTHFUL EATING ATTITUDES AND WEIGHT REGULATION PRACTICES OF YOUNG ADOLESCENT GIRLS. INTERNATIONAL JOURNAL OF EATING DISORDERS. 1993;13(4):369-384.
