# Supplementary material for: A Bibliometric Analysis of Scientific Publications on Eating Disorder Prevention in the Past Three Decades
Source: Nutrients. 2024 Apr 10;16(8):1111. doi: 10.3390/nu16081111 (PMC11054308; doi:10.3390/nu16081111)
Supplement: Supplementary file 1 [file nutrients-16-01111-s001.zip › File S2.pdf]

1. RHEUMATOLOGY ADVANCES IN PRACTICE Volume 7 Supplement 2, October 2023 Case-based Conference 2023 Abstracts. Rheumatology Advances in Practice. 2023;7():1.

2. Abbate-Daga G., Taverna A., Martini M. The oracle of Delphi 2.0: considering artificial intelligence as a challenging tool for the treatment of eating disorders. Eating and Weight Disorders-Studies on Anorexia Bulimia and Obesity. 2023;28(1):5.

3. Abu Libdeh A., Alkalbani L., Freedman D., Quezada J., Nyp S. Considerations in the Management of Functional Neurological Disorders in Patients with Hearing Loss. Journal of Developmental and Behavioral Pediatrics. 2023;44(4):e333-e335.

4. Abuqwider J., Di Porzio A., Barrella V., Gatto C., Sequino G., De Filippis F., Crescenzo R., Spagnuolo M., Cigliano L., Mauriello G., Iossa S., Mazzoli A. <i>Limosilactobacillus reuteri</i> DSM 17938 reverses gut metabolic dysfunction induced by Western diet in adult rats. Frontiers in Nutrition. 2023;10():13.

5. Ackerman K., Rogers M., Heikura I., Burke L., Stellingwerff T., Hackney A., Verhagen E., Schley S., Saville G., Mountjoy M., Holtzman B. Methodology for studying Relative Energy Deficiency in Sport (REDs): a narrative review by a subgroup of the International Olympic Committee (IOC) consensus on REDs. British Journal of Sports Medicine. 2023;57(17):1136-+.

6. Adigun A., Adebile T., Okoye C., Ogundipe T., Ajekigbe O., Mbaezue R., Okobi O. Causes and Prevention of Early-Onset Colorectal Cancer. Cureus Journal of Medical Science. 2023;15(9):11.

7. Affaticati L., Buoli M., Vaccaro N., Manzo F., Scalia A., Coloccini S., Zuliani T., La Tegola D., Capuzzi E., Nicastro M., Colmegna F., Clerici M., Dakanalis A., Caldiroli A. The Impact of Clinical Factors, Vitamin B12 and Total Cholesterol on Severity of Anorexia Nervosa: A Multicentric Cross-Sectional Study. Nutrients. 2023;15(23):15.

8. Aguiar A., Önal F., Hendricks G., Blanchard L., Romanenko E., Fismen A., Nwosu E., Herstad S., Savona N., Harbron J., Knai C., Samdal O., Rutter H., Lien N., Jalali M., Kopainsky B. Understanding the dynamics emerging from the interplay among poor mental wellbeing, energy balance-related behaviors, and obesity prevalence in adolescents: A simulation-based study. Obesity Reviews. 2023;24():15.

9. Al-Tamimi A., Paric M., Groot W., Pavlova M. Yemeni refugees' health literacy and experience with the Dutch healthcare system: a qualitative study. Bmc Public Health. 2023;23(1):9.

10. Alghanami B., El Keshky M. The Relationship between the Family Environment and Eating Disorder Symptoms in a Saudi Non-Clinical Sample of Students: A Moderated Mediated Model of Automatic Thoughts and Gender. Behavioral Sciences. 2023;13(10):11.

11. Ali S., Mahfouz M., Hakami R., Altubayqi T., Alhazmi N., Adawi N., Khormi R., Yaqoub W., Maghfori G., Mujarribi M., Dighriri I. Prevalence and Associated Factors of Eating Disorders Among Female Students at Jazan University, Kingdom of Saudi Arabia: A Survey Study. Cureus Journal of Medical Science. 2023;15(8):13.

12. Aljuraiban G., Alfhili M., Aldhwayan M., Aljazairy E., Al-Musharaf S. Shared and Distinct Gut Microbial Profiles in Saudi Women with Metabolically Healthy and Unhealthy Obesity. Microorganisms. 2023;11(6):14.

13. Alloy L., Chat I., Grehl M., Stephenson A., Adogli Z., Olino T., Ellman L., Miller G., Nusslo R. Reward and Immune Systems in Emotion (RISE) prospective longitudinal study: Protocol overview of an integrative reward-inflammation model of first onset of major depression in adolescence. Brain, Behavior, & Immunity - Health. 2023;30():13.

14. AlMuammar S., Algarni A., Badroun F., Mushaeb H., Abu Sadi R., Algarni A. Meal Timing Habits among Adults in Saudi Arabia: A Cross-Sectional Study. Advances in Public Health. 2023;2023():8.

15. Alomran S., Estrella E. Effect of Dietary Regimen on the Development of Polycystic Ovary Syndrome: A Narrative Review. Cureus Journal of Medical Science. 2023;15(10):11.

16. Ambrosecchia M., Ardizzi M., Russo E., Ditaranto F., Speciale M., Vinai P., Todisco P., Maestro S., Gallese V. Bodily self-recognition and body size overestimation in restrictive anorexia nervosa: implicit and explicit mechanisms. Frontiers in Psychology. 2023;14():12.

17. Ameneh P., Mohtasham G., Sakineh R., Yadollah M., Ali R. School-based peer-led diabetes intervention among female adolescents: a cluster randomized trial. Bmc Public Health. 2023;23(1):11.

18. Amianto F., Arletti L., Vesco S., Davico C., Vitiello B. Therapeutic outcome and long-term naturalistic follow-up of female adolescent outpatients with AN: clinical, personality and psychopathology evolution, process indicators and outcome predictors. Bmc Psychiatry. 2023;23(1):13.

19. Aouad P., Ahmed M., Nassar N., Miskovic-Wheatley J., Touyz S., Maguire S., Cunich M. Appraisal of the costs, health effects, and cost-effectiveness of screening, prevention, treatment and policy-indicated evidence-based interventions for eating disorders: a systematic review protocol. Journal of Eating Disorders. 2023;11(1):7.

20. Arafat S., Monira S., Lily S. Filicide in Bangladesh: A case indicating the need for psychosocial support among mothers during peripartum. Journal of General and Family Medicine. 2023;24(4):254-256.

21. Arai H., Maeda K., Wakabayashi H., Naito T., Konishi M., Assantachai P., Auyeung W., Chalermsri C., Chen W., Chew J., Chou M., Hsu C., Hum A., Hwang I., Kaido T., Kang L., Kamaruzzaman S., Kim M., Lee J., Lee W., Liang C., Lim W., Lim J., Lim Y., Lo R., Ong T., Pan W., Peng L., Pramyothin P., Razalli N., Saitoh M., Shahar S., Shi H., Tung H., Uezono Y., Haehling S., Won C., Woo J., Chen L. Diagnosis and outcomes of cachexia in Asia: Working Consensus Report from the Asian Working Group for Cachexia. Journal of Cachexia Sarcopenia and Muscle. 2023;():10.

22. Araki T., Hayashi K., Sonoda Y., Honda T., Imamura Y., Koide Y., Hamada H., Nakao K. Dabigatran-induced esophagitis with full circumferential blue pigmentation. Den Open. 2023;4(1):4.

23. Arbour S., Paul S., Rice M. Reduction of adverse events in a psychiatric inpatient eating disorder unit during the COVID-19 pandemic. Journal of Child and Adolescent Psychiatric Nursing. 2023;():6.

24. Arslan S., Avcu G., Bal Z., Arslan A., Ozkinay F., Kurugol Z. Evaluation of post-COVID symptoms of the SARS-CoV-2 Delta and Omicron variants in children: a prospective study. European Journal of Pediatrics. 2023;182(10):4565-4571.

25. Aston S., Caffo B., Bhasin H., Moran T., Tamashiro K. Timing matters: The contribution of running during different periods of the to to anorexia in rats. Physiology & Behavior. 2023;271():9.

26. Athanasaki D., Lakoumentas J., Milani G., Agostoni C., Berghea F., Ionescu M., Vassilopoulou E. Maternal Bonding as a Protective Factor for Orthorexia Nervosa Risk in Dietetics Students. Nutrients. 2023;15(16):16.

27. Atkinson M., Parnell J., Diedrichs P. Task shifting eating disorders prevention: A pilot study of selective interventions adapted for teacher-led universal delivery in secondary schools. International Journal of Eating Disorders. 2023;():14.

28. Augimeri G., Bonofiglio D. Promising Effects of <i>N</i>-Docosahexaenoyl Ethanolamine in Breast Cancer: Molecular and Cellular Insights. Molecules. 2023;28(9):13.

29. Avila-Ribeiro P., Lopes A., Martins-Martinho J., Nogueira E., Antunes J., Romeu J., Cruz-Machado A., Vieira-Sousa E. Secukinumab-induced systemic lupus erythematosus in psoriatic arthritis. Arp Rheumatology. 2023;2(3):265-268.

30. Ayrolles A., Bargiacchi A., Clarke J., Michel M., Baillin F., Trebossen V., Kalifa H., Guilmin-Crepon S., Delorme R., Godart N., Stordeur C. Comparison between continued inpatient treatment versus day patient treatment after short inpatient care in early onset anorexia nervosa (COTIDEA trial): a study protocol for a non-inferiority randomised controlled trial. Bmc Psychiatry. 2023;23(1):9.

31. Ayton A., Ibrahim A., Downs J., Baker S., Kumar A., Virgo H., Breen G. From awareness to action: an urgent call to reduce mortality and improve outcomes in eating disorders. British Journal of Psychiatry. 2023;():3.

32. Babbott K., Consedine N., Roberts M. Eating behaviour, body image, and mental health: updated estimates of adolescent health, well-being, and positive functioning in Aotearoa New Zealand. Journal of Primary Health Care. 2023;():11.

33. Baceviciene M., Jankauskiene R., Rutkauskaite R. The Comparison of Disordered Eating, Body Image, Sociocultural and Coach-Related Pressures in Athletes across Age Groups and Groups of Different Weight Sensitivity in Sports. Nutrients. 2023;15(12):18.

34. Baharuddin S., Shah Nnak, Yazan L., Abd Rashed A., Kadota K., Al-Awaadh A., Yusof Y. Optimization of<i> Pluchea</i><i> indica</i> (L.) leaf extract using ultrasound-assisted extraction and its cytotoxicity on the HT-29 colorectal cancer cell line. Ultrasonics Sonochemistry. 2023;101():11.

35. Baker S., Maiano C., Houle S., Nadon L., Aim é A., Morin A. Profiles of body image concerns and their associations with disordered eating behaviors. Appetite. 2023;191():11.

36. Balestrieri P., Cicala M., Ribolsi M. Psychological distress in inflammatory bowel disease. Expert Review of Gastroenterology & Hepatology. 2023;17(6):539-553.

37. Barlow I., Lee E., Sailing L. Orthorexia nervosa versus healthy orthorexia: Anxiety, perfectionism, and mindfulness as risk and preventative factors of distress. European Eating Disorders Review. 2023;():18.

38. Barnhart W., Cui T., Zhang H., Cui S., Zhao Y., Lu Y., He J. Examining an integrated sociocultural and objectification model of thinness- and muscularity-oriented disordered eating in Chinese older men and women. International Journal of Eating Disorders. 2023;56(10):1875-1886.

39. Basheikh Z., Jumbe T., Kulwa K. Perception and attitudes of street food vendors toward the healthiness of meals prepared and sold in Dodoma. Food Science & Nutrition. 2023;11(7):3885-3897.

40. Beckers D., Burk W., Larsen J., Cillessen A. The bidirectional associations between self-esteem and problematic eating behaviors in adolescents. International Journal of Eating Disorders. 2023;():12.

41. Beckers D., Larsen J., Burk W. Self-esteem and negative affectivity as mediators of the prospective links between adolescent interpersonal peer problems and disordered eating behaviors. Appetite. 2023;186():11.

42. Bell S., Berg M., Liboro R. Employing Dissonance-Based Interventions to Promote Health Equity Utilizing a Community-Based Participatory Research Approach and Social Network Analysis. Social Sciences-Basel. 2023;12(10):17.

43. Beltrán-Garrayo L., Solar M., Blanco M., Graell M., Sepúlveda A. Examining associations between obesity and mental health disorders from childhood to adolescence: A case-control prospective study. Psychiatry Research. 2023;326():9.

44. Ben-Shachar M., Daniel T., Wollman A., Govindaraj S., Aviel-Ronen S., Pinhasov A., Rosenzweig T. Inherited stress resiliency prevents the development of metabolic alterations

in diet-induced obese mice. Obesity. 2023;31(8):2043-2056.

45. Benedet P., Safikhan N., Pereira M., Lum B., Botezelli J., Kuo C., Wu H., Craddock B., Miller W., Eriksson J., Yue J., Conway E. CD248 promotes insulin resistance by binding to the insulin receptor and dampening its insulin-induced autophosphorylation. Ebiomedicine. 2024;99():23.

46. Berengüí R., Castejón M. Personality Traits and Risk of Eating Disorders in Men: A Cross-Sectional Study. Healthcare. 2023;11(21):11.

47. Bergen C., Lomas M., Ryan M., McCabe R. Gatekeeping and factors underlying decisions not to refer to mental health services after self-harm: Triangulating video-recordings of consultations, interviews, medical records and discharge letters. Ssm-Qualitative Research in Health. 2023;4():14.

48. Bhat R., Vellekkat F., Goutama I., Gill P., Kakar G., Jabeen H., Gireesh K., Sanker V., Gupta U. A case of trichotillomania and bulimia nervosa in a patient with adult-onset attention-deficit/hyperactivity disorder (ADHD). Clinical Case Reports. 2023;11(8):6.

49. Bianchi D., Schinelli A., Fatta L., Lonigro A., Lucidi F., Laghi F. Body image impact on quality of life and adolescents' binge eating: the indirect role of body image coping strategies. Eating and Weight Disorders-Studies on Anorexia Bulimia and Obesity. 2023;28(1):10.

50. Bianco V., Kratky D. Glycoprotein Non-Metastatic Protein B (GPNMB): The Missing Link Between Lysosomes and Obesity. Experimental and Clinical Endocrinology & Diabetes. 2023;():7.

51. Biderman C., Bianchini G., Bodell L. The role of negative evaluation fears on associations between societal appearance pressures and disordered eating in university students. Journal of American College Health. 2023;():9.

52. Biswas T., Sinha A., Abhijita B., Mishra S., Padhy S. Pediatric Acute Onset Neuropsychiatric Syndrome Presenting with Atypical Eating Disorder: A Case Report. Journal of Indian Association for Child and Adolescent Mental Health. 2022;18(4):364-366.

53. Blaak E., Goossens G. Metabolic phenotyping in people living with obesity: Implications for dietary prevention. Reviews in Endocrine & Metabolic Disorders. 2023;24(5):825-838.

54. Bodega P., Cos-Gandoy A., Fernández-Alvira J., Fernandez-Jiménez R., Moreno L., Santos-Beneit G. Body image and dietary habits in adolescents: a systematic review. Nutrition Reviews. 2023;():24.

55. Bohrer B., Chen Y., Christensen K., Forbush K., Thomeczek M., Richson B., Chapa D., Jarmolowicz D., Gould S., Negi S., Perko V., Morgan R. A pilot multiple-baseline study of a mobile cognitive behavioral therapy for the treatment of eating disorders in university students. International Journal of Eating Disorders. 2023;():14.

56. Borner T., Doebley S., Furst C., Pataro A., Halas J., Gao X., Choi G., Ramadan S., Chow A., De Jonghe B. Screening study of anti-emetics to improve GDF15-induced malaise and anorexia: Implications for emesis control. Physiology & Behavior. 2023;267():7.

57. Borsarini B., Pappaianni E., Micali N. Locked down with my eating disorder: a retrospective study on the impact of COVID-19 lockdown on adolescents with eating disorders. Journal of Eating Disorders. 2023;11(1):8.

58. Bourke M., Pila E. Body-related self-conscious emotions, self-compassion, and dietary restraint in Canadian undergraduate university students: A multilevel mediation and moderation analysis. International Journal of Eating Disorders. 2023;56(11):2131-2141.

59. Bourne L., Bryant-Waugh R., Mandy W., Solmi F. Investigating the prevalence and risk factors of picky eating in a birth cohort study. Eating Behaviors. 2023;50():9.

60. Bracké K., Dieleman G., Dias L., Steegers C., Dremmen M., Vernooij M., White T. RESTING-STATE FUNCTIONAL CONNECTIVITY IN FEMALE ADOLESCENTS WITH FIRST-ONSET ANOREXIA NERVOSA COMPARED TO HEALTHY CONTROLS. Journal of the American Academy of Child and Adolescent Psychiatry. 2023;62(10):S167-S167.

61. Breiner C., Miller M., Sanzari C., Perry T., Hormes J. Peer Ethnicity as a Mediator in the Relationship Between Ethnic Identity and Body Appreciation in Black College-Aged Women. Journal of Black Psychology. 2023;49(6):814-834.

62. Brewerton T., Gavidia I., Suro G., Perlman M. Associations between major depressive and bipolar disorders and eating disorder, PTSD, and comorbid symptom severity in eating disorder patients. European Eating Disorders Review. 2023;():13.

63. Brizzi G., Sansoni M., Di Lernia D., Frisone F., Tuena C., Riva G. The multisensory mind: a systematic review of multisensory integration processing in Anorexia and Bulimia Nervosa. Journal of Eating Disorders. 2023;11(1):31.

64. Brosig L., Düplois D., Hiemisch A., Kiess W., Hilbert A., Schlensog-Schuster F., Schmidt R. Birth-related, medical, and diagnostic characteristics in younger versus older children with avoidant/restrictive food intake disorder (ARFID). Journal of Eating Disorders. 2023;11(1):11.

65. Bruno R., Varbiro S., Pucci G., Nemcsik J., Lonnebakken M., Kublickiene K., Schluchter H., Park C., Mozos I., Guala A., Hametner B., Seeland U., Boutouyrie P., Sex Gender VascAgeNet Expert Grp Vascular function in hypertension: does gender dimension matter?. Journal of Human Hypertension. 2023;37(8):634-643.

66. Bryant E., Spielman K., Burton A., Ong S., Livney J., Corry S., Maguire S. Identifying eating disorders at the earliest opportunity: Testing the reliability of an online eating disorder screener (IOI-S) in primary care and youth mental health settings. Early Intervention in Psychiatry. 2023;():9.

67. Bugaeva P., Arkusha I., Bikaev R., Kamenskiy I., Pokrovskaya A., El-Taravi Y., Caso V., Avedisova A., Chu D., Genuneit J., Torbahn G., Nicholson T., Baimukhambetova D., Mursalova A., Kolotilina A., Gadetskaya S., Kondrikova E., Zinchuk M., Akzhigitov R., Boyle R., Guekht A., Munblit D. Association of breastfeeding with mental disorders in mother and child: a systematic review and meta-analysis. Bmc Medicine. 2023;21(1):19.

68. Burgon R., Beard J., Waller G. Body image concerns across different sports and sporting levels: A systematic review and meta-analysis. Body Image. 2023;46():9-31.

69. Burgos A., Ortega I., Fontes F., Valdés V., Camaño-Cacó E., Ríos-Castillo I. Malnutrition and unhealthy eating habits in Panamanian firefighters. Revista Chilena De Nutricion. 2023;50(4):413-423.

70. Cacciatore C., Cangiano B., Carbone E., Spagnoli S., Ramirez M., Polli N., Bonomi M., Persani L. Body weight variation is not an independent factor in the determination of

functional hypothalamic amenorrhea in anorexia nervosa. Journal of Endocrinological Investigation. 2023;():9.

71. Calcaterra V., Cena H., Rossi V., Santero S., Bianchi A., Zuccotti G. Ultra-Processed Food, Reward System and Childhood Obesity. Children-Basel. 2023;10(5):25.

72. Calcaterra V., Magenes V., Siccardo F., Hruby C., Basso M., Conte V., Maggioni G., Fabiano V., Russo S., Veggiotti P., Zuccotti G. Thyroid dysfunction in children and adolescents affected by undernourished and overnourished eating disorders. Frontiers in Nutrition. 2023;10():11.

73. Caldiroli A., La Tegola D., Manzo F., Scalia A., Affaticati L., Capuzzi E., Colmegna F., Argyrides M., Giaginis C., Mendolicchio L., Buoli M., Clerici M., Dakanalis A. The Impact of the COVID-19 Pandemic on Binge Eating Disorder: A Systematic Review. Nutrients. 2023;15(17):16.

74. Carbone E., Aloi M., Rania M., Filippis R., Quirino D., Fiorentino T., Segura-Garcia C. The relationship of food addiction with binge eating disorder and obesity: A network analysis study. Appetite. 2023;190():8.

75. Carrillo-Ruiz J., Carrillo-Márquez J., Beltrán J., Jiménez-Ponce F., García-Muñoz L., Navarro-Olvera J., Márquez-Franco R., Velasco F. Innovative perspectives in limbic surgery using deep brain stimulation. Frontiers in Neuroscience. 2023;17():9.

76. Carrino E., Flatt R., Pawar P., Sanzari C., Tregarthen J., Argue S., Thornton L., Bulik C., Watson H. Sociodemographic and clinical characteristics of treated and untreated adults with bulimia nervosa or binge-eating disorder recruited for a large-scale research study. Journal of Eating Disorders. 2023;11(1):19.

77. Carvalho F., Lahlou R., Pires P., Salgado M., Silva L. Natural Functional Beverages as an Approach to Manage Diabetes. International Journal of Molecular Sciences. 2023;24(23):22.

78. Cascino G., Monteleone A. Early traumatic experiences and the hypothalamus-pituitary-adrenal axis in people with eating disorders: A narrative review. Psychoneuroendocrinology. 2024;159():8.

79. Castellini G., Cassioli E., Rossi E., Marchesoni G., Cerini G., Pastore E., Cavalcabo N., Rotella F., Mezzani B., Alterini B., Lucarelli S., Magazzini S., Corazzesi P., Caini S., Ricca V. Use and misuse of the emergency room by patients with eating disorders in a matched-cohort analysis: What can we learn from it?. Psychiatry Research. 2023;328():8.

80. Castro-Cuesta J., Montoro-García S., Sánchez-Macarro M., Martínez M., Marenco I., Pérez-Camacho A., Martínez-Pastor A., Abellán-Alemán J. Adherence to the Mediterranean diet in first-year university students and its association with lifestyle-related factors: A cross-sectional study. Hipertension Y Riesgo Vascular. 2023;40(2):65-74.

81. Cavaliere G., Cimmino F., Trinchese G., Catapano A., Petrella L., D'Angelo M., Lucchin L., Mollica M. From Obesity-Induced Low-Grade Inflammation to Lipotoxicity and Mitochondrial Dysfunction: Altered Multi-Crosstalk between Adipose Tissue and Metabolically Active Organs. Antioxidants. 2023;12(6):23.

82. Chacón A., Wang C., Waqar D., Syeda S., Kumar R., Meghana D. Long-Term Usage of Oral Glucocorticoids Leading to Adrenal Insufficiency: A Comprehensive Review of the Literature. Cureus Journal of Medical Science. 2023;15(5):9.

83. Chan K., Sawyer A., Taylor A. Understanding early risk factors for eating disorder symptoms in adolescence: the role of body dissatisfaction, negative emotional reactivity and self-esteem at age 10-11 years. Australian Journal of Psychology. 2023;75(1):11.

84. Chancharoenthana W., Kamolratanakul S., Leelahavanichkul A., Ariyanon W., Chinpraditsuk S., Saelim R., Vadcharavivad S., Phumratanaprapin W., Wilairatana P. Gastrointestinal manifestations of long-term effects after COVID-19 infection in patients with dialysis or kidney transplantation: An observational cohort study. World Journal of Gastroenterology. 2023;29(19):3013-3026.

85. Charrat J., Massoubre C., Germain N., Gay A., Galusca B. Systematic review of prospective studies assessing risk factors to predict anorexia nervosa onset. Journal of Eating Disorders. 2023;11(1):16.

86. Chen W., Zhang S., Hu X., Chen F., Li D. A Review of Healthy Dietary Choices for Cardiovascular Disease: From Individual Nutrients and Foods to Dietary Patterns. Nutrients. 2023;15(23):36.

87. Chen X., Li W., Liu Y., Xiao M., Chen H. Altered effective connectivity between reward and inhibitory control networks in people with binge eating episodes: A spectral dynamic causal modeling study. Appetite. 2023;188():8.

88. Chen Y. A study on intervention effect of eating disorder prevention program for junior high school students in Taiwan. European Journal of Psychology Open. 2023;82():82-82.

89. Cheng C., Chu J., Ganson K., Trompeter N., Testa A., Jackson D., He J., Glidden D., Baker F., Nagata J. Cyberbullying and eating disorder symptoms in US early adolescents. International Journal of Eating Disorders. 2023;():7.

90. Chmiel J., Gladka A., Leszek J. The Effect of Transcranial Direct Current Stimulation (tDCS) on Anorexia Nervosa: A Narrative Review. Nutrients. 2023;15(20):18.

91. Choksawat O., Sripichyakan K., Sriarporn P., Baosoun C. Challenges of Avoiding Congenital Disorders: Experiences of Pregnant Women in Northeastern Thailand. Pacific Rim International Journal of Nursing Research. 2023;27(4):640-653.

92. Chua S., Craddock N., Rodtanaporn W., Or F., Austin S. Social media, traditional media, and other body image influences and disordered eating and cosmetic procedures in Malaysia, Singapore, Thailand, and Hong Kong. Body Image. 2023;45():265-272.

93. Chung C., Lee H., Seo M., Lee S., Kim K., Nazim K., Song J., Bae D., Rhee M., Kwon O., Kwak D. Molecular Detection and Genotyping of <i>Theileria</i> spp. in Deer (Cervidae) in Korea. Microorganisms. 2023;11(11):11.

94. Ciciulla D., Soriano V., McWilliam V., Koplin J., Peters R. Systematic Review of the Incidence and/or Prevalence of Eating Disorders in Individuals With Food Allergies. Journal of Allergy and Clinical Immunology-in Practice. 2023;11(7):25.

95. Ciurez B., Cobilinschi O., Luca A., Tincu I., Plesca D. Risk Factors Related to Eating Disorders in a Romanian Children Population. Nutrients. 2023;15(13):10.

96. Clemente-Suárez V., Ramírez-Goerke M., Redondo-Flórez L., Beltrán-Velasco A., Martín-Rodríguez A., Ramos-Campo D., Navarro-Jiménez E., Yáñez-Sepúlveda R., Tornero-Aguilera J. The Impact of Anorexia Nervosa and the Basis for Non-Pharmacological Interventions.

Nutrients. 2023;15(11):33.

97. Collantoni E., Alberti F., Dahmen B., Polier G., Konrad K., Herpertz-Dahlmann B., Favaro A., Seitz J. Intra-individual cortical networks in Anorexia Nervosa: Evidence from a longitudinal dataset. European Eating Disorders Review. 2023;():12.

98. Colton E., Wilson K., Chong T., Verdejo-Garcia A. Dysfunctional decision-making in binge-eating disorder: A meta-analysis and systematic review. Neuroscience and Biobehavioral Reviews. 2023;152():23.

99. Cooney R., Tang D., Barrett K., Russell R. Children and Young Adults With Inflammatory Bowel Disease Have an Increased Incidence and Risk of Developing Mental Health Conditions: A UK Population-Based Cohort Study. Inflammatory Bowel Diseases. 2023;():10.

100. Corsello A., Trovato C., Dipasquale V., Bolasco G., Labriola F., Gottrand F., Verduci E., Diamanti A., Romano C. Refeeding Syndrome in Pediatric Age, An Unknown Disease: A Narrative Review. Journal of Pediatric Gastroenterology and Nutrition. 2023;77(6):E75-E83.

101. Costa D., Charvin I., Da Fonseca D., Bat-Pitault F. Day hospital program for anorexia nervosa in children and adolescents: Assessment, management and specific focus on early onset anorexia nervosa. Encephale-Revue De Psychiatrie Clinique Biologique Et Therapeutique. 2023;49(6):557-563.

102. Cucinotta U., Romano C., Dipasquale V. A Systematic Review to Manage Avoidant/Restrictive Food Intake Disorders in Pediatric Gastroenterological Practice. Healthcare. 2023;11(16):12.

103. D'Adamo L., Linardon J., Manasse S., Juarascio A. Trajectories of therapeutic skills use and their dynamic relations to symptom change during cognitive-behavioral therapy for bulimia nervosa. International Journal of Eating Disorders. 2023;():11.

104. D'Adamo L., Sonnenblick R., Juarascio A., Manasse S. Relations between forms of dietary restraint, restriction, and loss-of-control eating among adolescents seeking weight control: An ecological momentary assessment study. Eating Behaviors. 2023;50():6.

105. d'Oplinter A., Verce M., Huwart S., Lessard-Lord J., Depommier C., Van Hul M., Desjardins Y., Cani P., Everard A. Obese-associated gut microbes and derived phenolic metabolite as mediators of excessive motivation for food reward. Microbiome. 2023;11(1):21.

106. D'Souza N., Downing K., Zheng M., Abbott G., Lioret S., Campbell K., Hesketh K. Cross-sectional and prospective associations between behavioural patterns and adiposity in school-aged children. Public Health Nutrition. 2023;():10.

107. Dadamo L., Smolar L., Balantekin K., Taylor C., Wilfley D., Fitzsimmons-Craft E. Prevalence, characteristics, and correlates of probable avoidant/restrictive food intake disorder among adult respondents to the National Eating Disorders Association online screen: a cross-sectional study. Journal of Eating Disorders. 2023;11(1):11.

108. Dalibalta S., Ghader N., Rabah L., Shaban S., Al Mheiri N. Impact of COVID-19 restrictions on health and well-being in the United Arab Emirates. Frontiers in Psychology. 2023;14():9.

109. Damigou E., Kouvari M., Chrysohoou C., Barkas F., Kravvariti E., Dalmyras D., Koutsogianni A., Tsioufis C., Pitsavos C., Liberopoulos E., Sfikakis P., Panagiotakos D. Diet Quality and Consumption of Healthy and Unhealthy Foods Measured via the Global Diet Quality Score in Relation to Cardiometabolic Outcomes in Apparently Healthy Adults from the Mediterranean Region: The ATTICA Epidemiological Cohort Study (2002-2022). Nutrients. 2023;15(20):13.

110. Daniel L., Haile D., Egata G. Disordered eating behaviours and body shape dissatisfaction among adolescents with type 1 diabetes: a cross sectional study. Journal of Eating Disorders. 2023;11(1):10.

111. Danielli S., Ashrafian H., Darzi A. Healthy city: global systematic scoping review of city initiatives to improve health with policy recommendations. Bmc Public Health. 2023;23(1):15.

112. Das U., Kar N. Prevalence and risk factor of diabetes among the elderly people in West Bengal: evidence-based LASI 1st wave. Bmc Endocrine Disorders. 2023;23(1):10.

113. Davis H., Smith Z., Smith G. Longitudinal transactions between negative urgency and fasting predict binge eating. Appetite. 2024;192():8.

114. Alva P., Ghaderi A., Andersson G., Feldman I., Sampaio F. The cost-effectiveness of a virtual intervention to prevent eating disorders in young women in Sweden. International Journal of Eating Disorders. 2023;56(10):1887-1897.

115. Connor A., Joshi V., Raffard S. When helping becomes hurting: An understanding of the implications of family accommodation on the effectiveness of psychological interventions in OCD. Annales Medico-Psychologiques. 2023;181(6):521-529.

116. Crom T., Steur M., Ikram M., Ikram M., Voortman T. Plant-based dietary patterns and the risk of dementia: a population-based study. Age and Ageing. 2023;52(9):8.

117. Hoz I., Osman A., Ryad R., Li W., Shah S., Notman D., Isea L., Tambunan D. A Rare Presentation of Sarcoidosis in a Young Male With Acute Renal Failure: A Case Report and Literature Review. Cureus Journal of Medical Science. 2023;15(11):7.

118. León S., Jiménez J., Peña-Quintana L., González-Martín J., Nóvoa-Medina Y. The Healthy Lifestyle Habits Screening Questionnaire: A pilot study in the Canary Islands. Endocrinologia Diabetes Y Nutricion. 2023;70(5):335-346.

119. Macedo P., Brito E., Cunha C., Araújo M., Martins P., Santana M. Weight stigma and disordered eating behaviors during the COVID-19 pandemic: the mediating role of weight gain concern and psychological distress. Eating and Weight Disorders-Studies on Anorexia Bulimia and Obesity. 2023;28(1):11.

120. Macedo P., Brito E., Cunha C., Costa P., Araújo M., Martins P., Santana M. Weight stigma is a predictor of disordered eating in Brazilian college students during the COVID-19 pandemic: A 16-month cohort follow-up. Appetite. 2024;192():9.

121. Declercq D., Kafle E., Peters J., Raby S., Chawner D., Blease J., Foye U. "Finding light in the darkness": exploring comedy as an intervention for eating disorder recovery. Mental Health Review Journal. 2024;():17.

122. Dehghan M., Tantbirojn D., Harrison J., Stewart C., Johnson N., Tolley E., Zhang Y. Oral Health and Behavior Patterns of Women with Eating Disorders-A Clinical Pilot Study. Life-

Basel. 2023;13(12):12.

123. Delcoigne B., Horne A., Askling J., Askling J. Risk of Psychiatric Disorders in Juvenile Idiopathic Arthritis: Population- and Sibling-Controlled Cohort and Cross-Sectional Analyses. Acr Open Rheumatology. 2023;5(5):277-284.

124. Dell'Osso L., Carpita B., Nardi B., Benedetti F., Dell'Oste V., Massimetti G., Cremone I., Barlati S., Castellini G., Luciano M., Bossini L., Rocchetti M., Signorelli M., Ricca V., Aguglia E., Fagiolini A., Vita A., Politi P., Maj M. Autistic traits distribution in different psychiatric conditions: A cluster analysis on the basis of the Adult Autism Subthreshold Spectrum (AdAS Spectrum) questionnaire. Psychiatry Research. 2023;326():9.

125. Deng Y., He J. A Comparison Between Before and During the Pandemic in Nonsuicidal Self-Injury Behaviors of Bipolar Disorder. Journal of Nervous and Mental Disease. 2023;211(12):896-901.

126. Dillberger J., Tobias J., Labelle P. A duodenal diverticulum in a deerhound. Veterinary Record Case Reports. 2023;():6.

127. Dimitropoulos G. CHANGES IN THE INCIDENCE OF NEW-ONSET ANOREXIA NERVOSA AND ATYPICAL ANOREXIA NERVOSA AMONG YOUTH DURING THE COVID-19 PANDEMIC IN CANADA. Journal of the American Academy of Child and Adolescent Psychiatry. 2023;62(10):S327-S327.

128. Dingemans A., Veldhuis J., Lähde I. Motives for using social networking sites: a uses & gratifications perspective amongst people with eating disorder symptoms. Journal of Eating Disorders. 2023;11(1):12.

129. Dolapoglu N., Ozcan D., Tulaci R. Is Orthorexia Nervosa a Non-specific Eating Disorder or a Disease in the Spectrum of Obsessive-Compulsive Disorder?. Cureus Journal of Medical Science. 2023;15(5):8.

130. Dolly A., Pötgens S., Thibaut M., Neyrinck A., Castro G., Galbert C., Lefevre C., Wyart E., Gomes S., Gonçalves D., Lanthier N., Baldin P., Huot J., Bonetto A., Seelaender M., Delzenne N., Sokol H., Bindels L. Impairment of aryl hydrocarbon receptor signalling promotes hepatic disorders in cancer cachexia. Journal of Cachexia Sarcopenia and Muscle. 2023;14(3):1569-1582.

131. Dominguez L., Donat-Vargas C., Sayon-Orea C., Barberia-Latasa M., Veronese N., Rey-Garcia J., Rodríguez-Artalejo F., Guallar-Castillón P., Martínez-González M., Barbagallo M. Rationale of the association between Mediterranean diet and the risk of frailty in older adults and systematic review and meta-analysis. Experimental Gerontology. 2023;177():11.

132. Dominiak H., Hasselsteen S., Nielsen S., Andersen J., Herrstedt J. Prevention of Taste Alterations in Patients with Cancer Receiving Paclitaxel- or Oxaliplatin-Based Chemotherapy-A Pilot Trial of Cannabidiol. Nutrients. 2023;15(13):10.

133. Donofre G., Campos Jadb, Santos P., Maroco J., Campos L., Silva W. Social Appearance Anxiety Scale: a psychometric investigation and evaluation of the influence of individual characteristics on social appearance anxiety in Brazilian adults who practice physical exercise. Frontiers in Psychology. 2023;14():12.

134. Dougherty E., Bottera A., Haedt-Matt A., Wildes J. Reconceptualizing emotion regulation and coping strategy usage in eating disorders research: The utility of a regulatory flexibility framework. International Journal of Eating Disorders. 2023;56(10):1835-1841.

135. Du Z., Huang J., Xia R., Ermakov P., Xu X. Obese people are more likely to exhibit unhealthy food decisions when sated. Food Quality and Preference. 2023;112():12.

136. Duan H., Zhou D., Xu N., Yang T., Wu Q., Wang Z., Sun Y., Li Z., Li W., Ma F., Chen Y., Du Y., Zhang M., Yan J., Sun C., Wang G., Huang G. Association of Unhealthy Lifestyle and Genetic Risk Factors With Mild Cognitive Impairment in Chinese Older Adults. Jama Network Open. 2023;6(7):11.

137. Duan J., Li H., Wang Y., Ji Y., Chen C., Feng C., Zhang W. Benzo a pyrene and a high-fat diet induce aortic injury and promote low-density lipoprotein accumulation in the endothelium. Ecotoxicology and Environmental Safety. 2023;259():11.

138. Duan W., Ding Q., Peng S., Kang Q., Guo L., Zhang L., Wei Y., Xiao Z., Fan J., Chen J. Chinese university students showed less disordered eating during the COVID-19 campus lockdown. Eating and Weight Disorders-Studies on Anorexia Bulimia and Obesity. 2023;28(1):10.

139. Dufils B., Galmiche M., Déchelotte P. The role of the dispensary pharmacist in the screening and referring patients with eating disorders. Nutrition Clinique Et Metabolisme.

2023;37(4):201-213.

140. Dufour R., Breton E., Morin A., Coté S., Dubois L., Vitaro F., Boivin M., Tremblay R., Booij L. Childhood hyperactivity, eating behaviours, and executive functions: Their association with the development of eating-disorder symptoms in adolescence. Journal of Eating Disorders. 2023;11(1):11.

141. Düplois D., Brosig L., Hiemisch A., Kiess W., Hilbert A., Schlensog-Schuster F., Schmidt R. Distribution and clinical comparison of restrictive feeding and eating disorders using ICD-10 and ICD-11 criteria. International Journal of Eating Disorders. 2023;():13.

142. Dworschak C., Polack R., Winschel J., Joormann J., Kober H. Emotion regulation and disordered eating behaviour in youths: Two daily-diary studies. European Eating Disorders Review. 2023;31(5):655-669.

143. Echouffo-Tcheugui J., Perreault L., Ji L., Dagogo-Jack S. Diagnosis and Management of Prediabetes: A Review. Obstetrical & Gynecological Survey. 2023;78(7):408-409.

144. Eddy K., Plessow F., Breithaupt L., Becker K., Slattery M., Mancuso C., Izquierdo A., De Water A., Kahn D., Dreier M., Ebrahimi S., Deckersbach T., Thomas J., Holsen L., Misra M., Lawson E. Neural activation of regions involved in food reward and cognitive control in young females with anorexia nervosa and atypical anorexia nervosa versus healthy controls. Translational Psychiatry. 2023;13(1):10.

145. Egan S., Neal J., Ure S., Callaghan T., Ho P., Shafran R., Wade T. The development of co-designed parent-supported cognitive behaviour therapy for perfectionism in adolescents with eating disorders: initial feasibility and acceptability. Journal of Eating Disorders. 2023;11(1):12.

146. Elahi A., Ali A., Khan A., Samad Z., Shahab H., Aziz N., Almas A. Challenges of managing hypertension in Pakistan-a review. Clinical Hypertension. 2023;29(1):14.

147. Elbeltagi R., Al-Beltagi M., Saeed N., Bediwy A. COVID-19-induced gastrointestinal autonomic dysfunction: A systematic review. World Journal of Clinical Cases. 2023;11(22):5252-5272.

148. Eppe J., Bayrou C., Casalta H., Cassart D., Gille L., Stipulanti M., Versyp J., Sartelet A. Oak Acorn Poisoning in Cattle during Autumn 2022: A Case Series and Review of the Current Knowledge. Animals. 2023;13(16):11.

149. Esmaeeli S., Rahimi Z., Saeed F., Shoib S. COVID-19 induced anorexia nervosa: A case series and brief review of literature. Clinical Case Reports. 2023;11(6):8.

150. Fabry E., Fassnacht D., Ford R., Burns N., O'Shea A., Ali K. The role of self-reliance and denial in the help-seeking process for eating disorders among university students. European Eating Disorders Review. 2023;():8.

151. Faller J., Perez J., Mihalopoulos C., Chatterton M., Engel L., Lee Y., Le P., Le L. Economic evidence for prevention and treatment of eating disorders: An updated systematic review. International Journal of Eating Disorders. 2023;():21.

152. Fantini L., Gostoli S., Artin M., Rafanelli C. An intervention based on Well-Being Therapy to prevent alcohol use and other unhealthy lifestyle behaviors among students: a three-arm cluster randomized controlled trial. Psychology Health & Medicine. 2023;():21.

153. Fekih-Romdhane F., Hallit R., Malaeb D., Sakr F., Dabbous M., Sawma T., Obeid S., Hallit S. Psychometric properties of an Arabic translation of the Nine Item Avoidant/Restrictive Food Intake Disorder Screen (NIAS) in a community sample of adults. Journal of Eating Disorders. 2023;11(1):10.

154. Fernández P., Labra J., Méndez M., González C., Coballes S., Souto-Gestal A. The Moderating Effect of Sex and Age on the Pattern of Body Image by Pre-Adolescents and Adolescents and Its Relationship with the Time They Spend Doing Sports. Sustainability. 2023;15(13):20.

155. Figueira R., Xavier M., Tomasi E., Demarco F., Gonzalez M., Bielemann R. Validation of dysphagia perception to predict the risk for dysphagia in non-institutionalized older adults. Clinical Nutrition Espen. 2023;57():358-363.

156. Font M., Busquets-Córtes C., Ramírez-Manent J., Tomás-Gil P., Paublini H., López-González A. Influence of Sociodemographic Variables and Healthy Habits on the Values of Insulin Resistance Indicators in 386,924 Spanish Workers. Nutrients. 2023;15(24):15.

157. Forbes J., Paxton S., Yager Z. Independent pragmatic replication of the Dove Confident Me body image program in an Australian Girls Independent Secondary School. Body Image. 2023;46():152-167.

158. Forester G., Johnson J., Reilly E., Lloyd E., Johnson E., Schaefer L. Back to the future: Progressing memory research in eating disorders. International Journal of Eating Disorders. 2023;56(11):2032-2048.

159. Foster L., Lundh L., Daukantaite D. Disordered eating in a 10-year perspective from adolescence to young adulthood: Stability, change, and body dissatisfaction as a predictor. Scandinavian Journal of Psychology. 2023;():10.

160. Franco L., Nakano E., Raposo A., Alturki H., Alarifi S., Chaves C., Teixeira-Lemos E., Romao B. Eating Attitudes of Patients with Celiac Disease in Brazil: A Nationwide Assessment with the EAT-26 Instrument. Nutrients. 2023;15(22):14.

161. Franco-Paredes K., Díaz-Reséndiz F., Peláez-Fernández M., Bautista-Díaz M. Variables that explain disordered eating behaviors among women: the mediating role of body dissatisfaction. Eating and Weight Disorders-Studies on Anorexia Bulimia and Obesity. 2024;29(1):8.

162. Freire G., Martinez B., Chacon R., Naranjo G. EDUCATIONAL PROGRAM FOR PATIENTS WITH GESTATIONAL DIABETES CARE AT HOSPITAL GENERAL RIOBAMBA IESS. Revista Conrado. 2023;19():385-392.

163. Ganderats-Fuentes M., Morgan S. Front-of-Package Nutrition Labeling and Its Impact on Food Industry Practices: A Systematic Review of the Evidence. Nutrients. 2023;15(11):24.

164. Ganson K. Association Between Psychological Flexibility and Eating Disorder Symptoms Among College Students. Journal of the Society for Social Work and Research. 2023;():9.

165. Gao D., Wang H., Wang Y., Ma S., Zou Z. Association between Diet Quality and Stroke among Chinese Adults: Results from China Health and Nutrition Survey 2011. Nutrients. 2023;15(14):11.

166. Gao X., Tang Y., Kong L., Fan Y., Wang C., Wang R. Treg cell: Critical role of regulatory T-cells in depression. Pharmacological Research. 2023;195():18.

167. García J., Livio J., Matto C., Dutra F., Scioli V., Giannitti F., Langston J., Poppenga R., Cantón G., Uzal F. Pollen beetle (<i>Astylus atromaculatus</i>)-associated gastroenteric disease in cattle: report of 6 natural outbreaks. Journal of Veterinary Diagnostic Investigation. 2023;():8.

168. García-Fernández S., Fernández-Morán E., López-Martínez C., Vivanco-Allende B., Costales-Alvarez C., Ordóñez-Alvarez F. Tubulointerstitial nephritis and uveitis syndrome and SARS-CoV-2 infection in an adolescent: just a coincidence in time?. Pediatric Nephrology. 2023;38(12):4203-4207.

169. Gardini V., Ruini C., Tossani E., Grandi S., Tomba E. Protocol for a Randomized Controlled Trial Testing the Efficacy of a Transdiagnostic Virtual Reality-Based Intervention for the Reduction of Unhealthy Lifestyles and Behaviors in the General Population. Journal of Clinical Medicine. 2023;12(23):14.

170. Garutti M., Noto C., Pastò B., Cucciniello L., Alajmo M., Casirati A., Pedrazzoli P., Caccialanza R., Puglisi F. Nutritional Management of Oncological Symptoms: A Comprehensive Review. Nutrients. 2023;15(24):23.

171. Gerend M., Zetrenne S., Sutin A., Naar S., Maner J. Weight Discrimination and Health Risk Behavior in Racial, Ethnic, and Sexual Minority Adults. Annals of Behavioral Medicine. 2023;57(7):571-581.

172. Gerges S., Obeid S., Malaeb D., El Dine A., Hallit R., Soufia M., Fekih-Romdhane F., Hallit S. Validation of an Arabic version of the eating disorder inventory's body dissatisfaction subscale among adolescents, adults, and pregnant women. Journal of Eating Disorders. 2023;11(1):12.

173. Gergov V., Prevendar T., Vousoura E., Ulberg R., Dahl H., Feller C., Jacobsen C., Karain A., Milic B., Poznyak E., Sacco R., Tulbure B., Camilleri N., Liakea I., Podina I., Saliba A., Torres S., Poulsen S. Sociodemographic Predictors and Moderators of Treatment Outcomes of Psychotherapeutic Interventions for Young People with Mental Disorders: A Systematic Review. Adolescent Research Review. 2023;():23.

174. Ghazzawi H., Nimer L., Sweidan D., Alhaj O., Abulawi D., Amawi A., Levine M., Jahrami H. The global prevalence of screen-based disordered eating and associated risk factors among high school students: systematic review, meta-analysis, and meta-regression. Journal of Eating Disorders. 2023;11(1):23.

175. Gibson A., Gale J., Stamatakis E., Lindley R., Fontana L., Cistulli P., Nassar N. Impact of lifestyle risk factors on admission to nursing home care: a cohort study of 127 108 people aged 60 years and over. Journal of Epidemiology and Community Health. 2023;77(11):744-751.

176. Giles E., Purcell S., Olson J., Vrieling A., Hirko K., Woodruff K., Playdon M., Thomas G., Gilmore L., Moberly H., Newell-Fugate A. Trends in Diet and Cancer Research: A Bibliometric and Visualization Analysis. Cancers. 2023;15(15):18.

177. Golzarand M., Moslehi N., Mirmiran P., Azizi F. Adherence to the DASH, MeDi, and MIND diet scores and the incidence of metabolically unhealthy phenotypes. Obesity Research & Clinical Practice. 2023;17(3):226-232.

178. González-Gómez A., Fernández-Golfín C., Hinojar R., Monteagudo J., García A., García-Sebástian C., García-Lunar I., Sánchez-Recalde A., Salido L., Pardo A., Zamorano J. The 4A classification for patients with tricuspid regurgitation. Revista Espanola De Cardiologia. 2023;76(11):845-851.

179. Gordon A., Beccia A., Egan N., Lipson S. Intersecting gender identity and racial/ethnic inequities in eating disorder risk factors, symptoms, and diagnosis among US college students: An intersectional multilevel analysis of individual heterogeneity and discriminatory accuracy. International Journal of Eating Disorders. 2023;():16.

180. Gordon A., Egan K., Wang M., Ziyadeh N., Kenney E., Rosario M., Austin S. Weight-based discrimination and disordered eating behaviors in a cohort of US sexual minority young adults. International Journal of Eating Disorders. 2023;56(10):1983-1990.

181. Gordon C., Gasbarro A., Wendell V., Fischer S., Hardin R., Marino J. Assessing Exposure and Response Prevention Readiness for Clients With Obsessive Compulsive Disorder and Co-Occurring Conditions: A Decision-Making Model and Case Example. Professional Psychology-Research and Practice. 2023;54(4):305-313.

182. Gorosito C., Jahn A., Cueto V. Incidence of beak deformities in austral thrushes (<i>Turdus falcklandii</i>) increases with urbanization and flocking behavior. Integrative Zoology. 2023;():10.

183. Gravina D., Keeler J., Akkese M., Bektas S., Fina P., Tweed C., Willmund G., Treasure J., Himmerich H. Randomized Controlled Trials to Treat Obesity in Military Populations: A Systematic Review and Meta-Analysis. Nutrients. 2023;15(22):25.

184. Gronhaug G., Joubert L., Saeterbakken A., Drum S., Nelson M. Top of the podium, at what cost? injuries in female international elite climbers. Frontiers in Sports and Active Living. 2023;5():7.

185. Gu D., Ou S., Liu G. Assessing the causal association of trauma with subsequent psychiatric disorders by a Mendelian randomization study trauma and common psychiatric disorders. Frontiers in Psychiatry. 2023;14():9.

186. Guimaraes J., Pauzé E., Kent M., Barquera S., Jáuregui A., Sacks G., Vanderlee L., Hammond D. The relationship between parent's self-reported exposure to food marketing and child and parental purchasing and consumption outcomes in five countries: findings from the International Food Policy Study. Journal of Nutritional Science. 2023;12():12.

187. Gumz A., Reuter L., Löwe B., Voderholzer U., Schwennen B., Fehrs H., Wünsch-Leiteritz W., Brunner R., Kästner D., Zapf A., Weigel A. Factors influencing the duration of untreated illness among patients with anorexia nervosa: A multicenter and multi-informant study. International Journal of Eating Disorders. 2023;():13.

188. Günes M., Demirer B. The effect of social media use on eating behaviors and physical activity among university students. Journal of Public Health-Heidelberg. 2023;():8.

189. Haake M., Haack B., Schäfer T., Harter P., Mattavelli G., Eiring P., Vashist N., Wedekink F., Genssler S., Fischer B., Dahlhoff J., Mokhtari F., Kuzkina A., Welters M., Benz T., Sorger L., Thiemann V., Almanzar G., Selle M., Thein K., Späth J., Gonzalez M., Reitinger C., Ipsen-Escobedo A., Wistuba-Hamprecht K., Eichler K., Filipski K., Zeiner P., Beschorner R., Goedemans R., Gogolla F., Hackl H., Rooswinkel R., Thiem A., Roche P., Joshi H., Pühringer D., Wöckel A., Diessner J., Rüdiger M., Leo E., Cheng P., Levesque M., Goebeler M., Sauer M., Nimmerjahn F., Schuberth-Wagner C., Felten S., Mittelbronn M., Mehling M., Beilhack A., Burg S., Riedel A., Weide B., Dummer R., Wischhusen J. Tumor-derived GDF-15 blocks LFA-1 dependent T cell recruitment and suppresses responses to anti-PD-1 treatment. Nature

Communications. 2023;14(1):19.

190. Haas A., Laboe A., McGinnis C., Firebaugh M., Shah J., Bardone-Cone A., Pike K., Taylor C., Wilfley D., Fitzsimmons-Craft E. Adapting a mobile app to support patients with anorexia nervosa following post-acute care: perspectives from eating disorder treatment center stakeholders. Frontiers in Digital Health. 2023;5():10.

191. Haghish E., Nes R., Obaidi M., Qin P., Staenicke L., Bekkhus M., Laeng B., Czajkowski N. Unveiling Adolescent Suicidality: Holistic Analysis of Protective and Risk Factors Using Multiple Machine Learning Algorithms. Journal of Youth and Adolescence. 2023;():19.

192. Hallward L., Feng O., Duncan L. An exploration and comparison of #BodyPositivity and #BodyNeutrality content on TikTok. Eating Behaviors. 2023;50():7.

193. Hamed M., Alamoudi D. Recurrent COVID-19 Infection in a Refractory/Classical Hodgkin's Lymphoma Patient Undergoing Autologous Stem Cell Transplantation: A Case Report. Cureus Journal of Medical Science. 2023;15(10):11.

194. Hamel J., Logigian E. Clinical Spectrum and Prognosis in Patients With Acute Nutritional Axonal Neuropathy. Neurology. 2023;100(20):E2134-E2140.

195. Hamid N. Internet-based cognitive behaviour therapy for the prevention, treatment and relapse prevention of eating disorders: A systematic review and meta-analysis. Psych Journal. 2023;():14.

196. Hao M., Yang J., Xu S., Yan W., Yu H., Wang Q. The relationship between body dissatisfaction, lifestyle, and nutritional status among university students in Southern China. Bmc Psychiatry. 2023;23(1):8.

197. Harris C., Benjamin K., Miao Z., Fantuzzi J., Averill M. Gender differences in factors related to eating competence in college students: Weight-and-body shame and guilt, weight satisfaction, weight loss effort, and eating disorder risk. Eating Behaviors. 2023;51():6.

198. Hasegawa T., Nishikawa K., Ohjino Y., Sano C., Ohta R. A Case of Late-Onset Systemic Lupus Erythematosus With Systemic Symptoms Leading to Multiple Organ Failure. Cureus Journal of Medical Science. 2023;15(10):6.

199. Hazzard V., Loth K., Fertig A., Trofholz A., Brito J., Doering A., Berge J. Household food insecurity is associated with greater prevalence and 18-month incidence of a range of disordered eating behaviors in a racially and ethnically diverse sample of parents. Eating Behaviors. 2023;49():7.

200. He Y., Zheng J., Ye B., Dai Y., Nie K. Chemotherapy-induced gastrointestinal toxicity: Pathogenesis and current management. Biochemical Pharmacology. 2023;216():25.

201. Hebebrand J., Antel J., Piechowski L., Kiewert C., Stueve B., Gradl-Dietsch G. Case report: Rapid improvements of anorexia nervosa and probable myalgic encephalomyelitis/chronic fatigue syndrome upon metreleptin treatment during two dosing episodes. Frontiers in Psychiatry. 2023;14():8.

202. Hendricks E., Jenkinson E., Falconer L., Griffiths C. How effective are psychosocial interventions at improving body image and reducing disordered eating in adult men? A systematic review. Body Image. 2023;47():14.

203. Hewlings S. Eating Disorders and Dietary Supplements: A Review of the Science. Nutrients. 2023;15(9):11.

204. Hilling J., Robertson C. A review of the nutritional guidance for athletes to prevent eating disorders. European Eating Disorders Review. 2023;():14.

205. Hirvelä L., Keski-Rahkonen A., Sipilä P. Associations of broad eating disorder symptoms with later alcohol problems in Finnish adult twins: A nationwide 10-year follow-up. International Journal of Eating Disorders. 2023;56(10):1854-1865.

206. Hoeeg D., Frohlich K., Christensen U., Grabowski D. Mechanisms of Stigmatization in Family-Based Prevention and Treatment of Childhood Overweight and Obesity. Children-Basel. 2023;10(10):13.

207. Hogg J., Cameron J., Cramb S., Baade P., Mengersen K. Mapping the prevalence of cancer risk factors at the small area level in Australia. International Journal of Health Geographics. 2023;22(1):19.

208. Holmes S. 'It's always that idea that everyone's trying to<i> look</i><i> like</i> something': Revisioning sociocultural factors in eating disorders through Photovoice.

Womens Studies International Forum. 2023;99():13.

209. Hooper L., Puhl R., Eisenberg M., Berge J., Neumark-Sztainer D. Can Family and Parenting Factors Modify the Impact of Weight Stigma on Disordered Eating in Young People? A Population-Based Longitudinal Study. Journal of Adolescent Health. 2023;73(1):44-52.

210. Hooper S., Espinoza S., Marshall V., Kilpela L. The Clinical Phenotype of Binge Eating Disorder among Postmenopausal Women: A Pilot Study. Nutrients. 2023;15(9):12.

211. Huang J., Chan S., Ko S., Lok V., Zhang L., Lin X., Lucero-Prisno III, Xu W., Zheng Z., Elcarte E., Withers M., Wong M. Global Incidence, Mortality, Risk Factors and Trends of Melanoma: A Systematic Analysis of Registries. American Journal of Clinical Dermatology. 2023;24(6):965-975.

212. Huh J., Lee J., Song J. Efficacy and safety of combination therapy with pirfenidone and nintedanib in patients with idiopathic pulmonary fibrosis. Frontiers in Pharmacology. 2023;14():8.

213. Hussain S., Saeed M., Saeed S., Faridi S. Investigating the Link Between Abdominal Pain, Weight Loss, and IgG4-Related Pancreatitis: A Case Report. Cureus Journal of Medical Science. 2023;15(7):5.

214. Huttunen-Lenz M., Raben A., Adam T., Macdonald I., Taylor M., Stratton G., Mackintosh K., Martinez J., Handjieva-Darlenska T., Bogdanov G., Poppitt S., Silvestre M., Fogelholm M., Jalo E., Brand-Miller J., Muirhead R., Schlicht W. Socio-economic factors, mood, primary care utilization, and quality of life as predictors of intervention cessation and chronic stress in a type 2 diabetes prevention intervention (PREVIEW Study). Bmc Public Health. 2023;23(1):20.

215. Ibáñez-Caparrós A., Sánchez I., Granero R., Jiménez-Murcia S., Rosinska M., Thiel A., Zipfel S., Pablo J., Camacho-Barcia L., Fernandez-Aranda F. Athletes with Eating Disorders: Analysis of Their Clinical Characteristics, Psychopathology and Response to Treatment. Nutrients. 2023;15(13):16.

216. Ip E., Doroudgar S., Salehi A., Salehi F., Najmi M. Diabulimia: A Risky Trend Among Adults with Type 1 Diabetes Mellitus. Endocrine Practice. 2023;29(11):849-854.

217. Iqbal H., West J., McEachan R., Haith-Cooper M. Identifying the Health Concerns of Pregnant British Pakistani Women Living in Deprived Areas: A Qualitative Study. Maternal and Child Health Journal. 2023;():7.

218. Ishak Sizs, Chin Y., Tay C., Syah M., Sufyan D. BMI-for-age z-score and psychological distress associated with disordered eating: A comparative study among Indonesian and Malaysian urban female adolescents. Human Nutrition & Metabolism. 2023;33():7.

219. Islam R., Ahmed M., Ullah W., Tahir Y., Gul S., Hussain N., Islam H., Anjum M. Effect of Caffeine in Hypertension. Current Problems in Cardiology. 2023;48(11):24.

220. Jaramillo A., Castells J., Ibrahimli S., Jaramillo L., Andriuoli R., Moncada D., Revilla J. Time-Restricted Feeding and Intermittent Fasting as Preventive Therapeutics: A Systematic Review of the Literature. Cureus Journal of Medical Science. 2023;15(7):7.

221. Jayasinghe S., Hills A. Strategies to Improve Physical Activity and Nutrition Behaviours in Children and Adolescents: A Review. Nutrients. 2023;15(15):15.

222. Jensen P., Engdahl B., Gustavson K., Lund I., Pettersen J., Madsen C., Hauge L., Knudsen A., Reneflot A., Brandlistuen R., Ask H., Nesvag R. Incidence rates of treated mental disorders before and during the COVID-19 pandemic-a nationwide study comparing trends in the period 2015 to 2021. Bmc Psychiatry. 2023;23(1):13.

223. Jha H., Baveja C., Kamal V., Agarwal P., Saxena S., Dhakad M., Sharma D. Comparative Diagnostic of Cervical Tuberculous Lymphadenitis: PCR is a Fast, Efficient, and Improved Diagnostic Approach. Canadian Journal of Infectious Diseases & Medical Microbiology. 2023;2023():6.

224. Jia C., Zeng Y., Huang X., Yang H., Qu Y., Hu Y., Chen W., Yang X. Lifestyle patterns, genetic susceptibility, and risk of valvular heart disease: a prospective cohort study based on the UK Biobank. European Journal of Preventive Cardiology. 2023;30(15):1665-1673.

225. Jiang X., Pestoni G., Vinci L., Suter F., Lorez M., Rohrmann S., Karavasiloglou N. Cancer cases attributable to modifiable lifestyle risk factors in Switzerland between 2015 and 2019. International Journal of Cancer. 2023;():14.

226. Jiao H., Fan Y., Gong A., Li T., Fu X., Yan Z. Xiaoyaosan ameliorates CUMS-induced depressive-like and anorexia behaviors in mice via necroptosis related cellular senescence in hypothalamus. Journal of Ethnopharmacology. 2024;318():20.

227. Johnson M., Gicking J., Keys D. Evaluation of red blood cell distribution width, neutrophil-to-lymphocyte ratio, and other hematologic parameters in canine acute pancreatitis. Journal of Veterinary Emergency and Critical Care. 2023;33(5):587-597.

228. Jones C., Read R., O'Donnell N., Wakelin K., John M., Skene S., Stewart R., Hale L., Cooke D., Kanumakala S., Satherley R. PRIORITY Trial: Results from a feasibility randomised controlled trial of a psychoeducational intervention for parents to prevent disordered eating in children and young people with type 1 diabetes. Diabetic Medicine. 2023;():11.

229. Ju A., Wiltink L., Walker J., White K., Rutherford C. Supportive care interventions for managing gastrointestinal symptoms following treatment for colorectal cancer: a systematic review. Journal of Cancer Survivorship. 2023;():8.

230. Kahlon S., Gjestad R., Lindner P., Nordgreen T. Perfectionism as a predictor of change in digital self-guided interventions for public speaking anxiety in adolescents: A secondary analysis of a four-armed randomized controlled trial. Cognitive Behaviour Therapy. 2023;():19.

231. Kaji N., Iwaoka K., Nakamura S., Tsukamoto A. Fuzapladib reduces postsurgical inflammation in the intestinal muscularis externa. Journal of Veterinary Medical Science. 2023;85(11):1151-1156.

232. Kakar V., Fardouly J., Rapee R., Guo M., Arman S., Niazi E. Exploring the tripartite influence model of body image and disordered eating among adolescent girls living in Australia, China, India, and Iran. Body Image. 2023;47():16.

233. Kamra N., Jagadeesan S., Singh R. Scrub Typhus Presenting As Acute Febrile Illness With Splenic Infarct: A Rare Manifestation. Cureus Journal of Medical Science. 2023;15(9):3.

234. Kapsomenakis A., Kasselimis D., Vaniotis E., Bougea A., Koros C., Simitsi A., Stefanis L., Potagas C. Frequency of Impulsive-Compulsive Behavior and Associated Psychological Factors in Parkinson's Disease: Lack of Control or Too Much of It?. Medicina-Lithuania. 2023;59(11):15.

235. Karam J., Bouteen C., Mahmoud Y., Tur J., Bouzas C. The Relationship between Social Media Use and Body Image in Lebanese University Students. Nutrients. 2023;15(18):11.

236. Karlsson E., Alricsson M., Melin A. Symptoms of eating disorders and low energy availability in recreational active female runners. Bmj Open Sport & Exercise Medicine. 2023;9(3):6.

237. Kasumi E., Chiba M., Kuzumaki Y., Kuzuoka H., Sato N., Takahashi B. Development and Characterization of a Cancer Cachexia Rat Model Transplanted with Cells of the Rat Lung Adenocarcinoma Cell Line Sato Lung Cancer (SLC). Biomedicines. 2023;11(10):16.

238. Kawai K., Tachimori H., Yamamoto Y., Nakatani Y., Iwasaki S., Sekiguchi A., Kim Y., Tamura N. Trends in the effect of COVID-19 on consultations for persons with clinical and subclinical eating disorders. Biopsychosocial Medicine. 2023;17(1):9.

239. Kawashima Y., Ishimoto O., Miyauchi E., Sakakibara T., Harada T., Usui K., Inoue A., Sugawara S. Phase II trial of daily S-1 combined with weekly irinotecan in previously treated patients with advanced or recurrent squamous cell lung cancer: North Japan lung cancer group 1101. Thoracic Cancer. 2023;14(27):2804-2810.

240. Kaza M., Tsentidis C., Vlachopapadopoulou E., Karanasios S., Sakou II, Paltoglou G., Mastorakos G., Karavanaki K. The impact of physical activity, quality of life and eating habits on cardiometabolic profile and adipokines in youth with T1D. Endocrine. 2023;80(3):541-551.

241. Kerby M., Tobias K., Monto T., Morandi F. Epiploic foramen entrapment in a dog. Veterinary Surgery. 2023;():8.

242. Keyes K., Platt J. Annual Research Review: Sex, gender, and internalizing conditions among adolescents in the 21st century - trends, causes, consequences. Journal of Child Psychology and Psychiatry. 2023;():24.

243. Khadem A., Shiraseb F., Mirzababaei A., Noori S., Mirzaei K. Association of Mediterranean-DASH Intervention for Neurodegenerative Delay (MIND) diet and metabolically unhealthy overweight/obesity phenotypes among Iranian women: a cross sectional study. Bmc Endocrine Disorders. 2023;23(1):10.

244. Khaltaev N., Axelrod S. Decline of the chronic respiratory disease mortality in the WHO Western Pacific Region. Journal of Thoracic Disease. 2023;():10.

245. Khemka S., Reddy A., Garcia R., Jacobs M., Reddy R., Roghani A., Pattoor V., Basu T., Sehar U., Reddy P. Role of diet and exercise in aging, Alzheimer's disease, and other chronic diseases. Ageing Research Reviews. 2023;91():19.

246. Khrais A., Mathew A., Kahlam A., Le Alxd, Mittal A., Verma S. Investigating the Correlation Between Clostridioides difficile Infection and Vitamin D Deficiency. Cureus Journal of Medical Science. 2023;15(6):5.

247. Kim H., Park K., Jo H., Shin Y., Chung G., Ko S., Jin Y., Kim W. The effect of ginger extract on cisplatin-induced acute anorexia in rats. Frontiers in Pharmacology. 2023;14():12.

248. Kingsbury M., Arim R. Cybervictimization and mental health among Canadian youth. Health Reports. 2023;34(9):3-13.

249. Kleppe M., Kessler U., Rekkedal G., Skjakodegard H., Danielsen Y. Differences in sleep patterns between patients with anorexia nervosa and healthy controls: a cross-sectional study. Journal of Eating Disorders. 2023;11(1):8.

250. Knight R., Preston C. Exploring the effects of gender and sexual orientation on disordered eating: an EFA to CFA study of the Eating Disorder Examination Questionnaire. Journal of Eating Disorders. 2023;11(1):14.

251. Kocabas S., Sanlier N. Exploring the intricacies of food disgust: Unveiling links between gender, healthy eating obsession, and disgust propensity. Food Quality and Preference. 2024;113():7.

252. Konstantinov V., Reznik A., Isralowitz R. Depression and Quality of Life among Ukrainian Adults Relocated to Russia. Journal of Loss & Trauma. 2023;28(6):493-503.

253. Kontele I., Vassilakou T., Psychountaki M., Reel J., Donti O. Development and Validation of the Greek Version of Weight Pressures in Sport-Females Questionnaire. Journal of Clinical Sport Psychology. 2023;():18.

254. Kovács D., Eitmann S., Berta G., Kormos V., Gaszner B., Pétervári E., Balaskó M. Aging Changes the Efficacy of Central Urocortin 2 to Induce Weight Loss in Rats. International Journal of Molecular Sciences. 2023;24(10):15.

255. Krishna G., Singh M., Gill B., Kumar V., Aggarwal V., Singh I. Bilateral sixth nerve palsy with subdural hematoma: a unique presentation of B12 deficiency. Childs Nervous System. 2023;39(9):2533-2536.

256. Kudamatsu H., Kawashiri T., Mine K., Mori K., Inoue M., Ishida H., Uchida M., Tsuchiya T., Kobayashi D., Shimazoe T. Ameliorating effects of cystine and theanine in a cancer cachexia mouse model. Journal of Pharmacological Sciences. 2023;152(3):163-166.

257. Kudoh M., Kakiuchi T., Yoshiura M., Esaki M., Matsuo M. Fecal calprotectin measurement to detect recurrence of solitary juvenile polyps: A case report. Medicine. 2023;102(43):4.

258. Kumar M. Eating Disorders in Youth with Chronic Health Conditions: Clinical Strategies for Early Recognition and Prevention. Nutrients. 2023;15(17):15.

259. Kurdak H., Tiyekli E., Özcan S., Özer Z., Topuz A. Eating disorders, primary care, and stigma: an analysis of research trends and patterns. Frontiers in Psychiatry. 2023;14():13.

260. Kuriki S., Nishida T., Chang L., Hosokawa K., Fujii Y., Osugi N., Nakamatsu D., Matsumoto K., Yamamoto M., Morimura O., Abe K., Okauchi Y., Iwahashi H., Inada M. Gastrointestinal symptoms in 609 Japanese patients with COVID-19: a single-center retrospective study. Scandinavian Journal of Gastroenterology. 2023;58(10):1139-1144.

261. Kuriyama T., Murata Y., Ohtani R., Yahara R., Nakashima S., Mori M., Ohe K., Mine K., Enjoji M. Modified activity-based anorexia paradigm dampens chronic food restriction-induced hyperadiponectinemia in adolescent female mice. Plos One. 2023;18(7):18.

262. Kussman A., Choo H. Mental Health and Disordered Eating in Athletes. Clinics in Sports Medicine. 2024;43(1):71-91.

263. La Spina M., Caruso M., Gulizia C., Comella M., Soma R., Meli M., Samperi P., Bertuna G., Di Cataldo A., Russo G. Diencephalic Syndrome: Misleading Clinical Onset of Low-Grade

Glioma. Current Oncology. 2023;30(9):8401-8410.

264. Labossière S., Couture S., Laurier C., Lemieux A., Boudreault V. The progression and mechanisms of mental illness symptoms in university student-athletes during the COVID-19 pandemic. Stress and Health. 2023;():18.

265. Lacroix E., Wilson S., McGue M., Iacono W., Ranson K. Trajectories and Personality Predictors of Eating-Pathology Development in Girls From Preadolescence to Adulthood. Clinical Psychological Science. 2023;():19.

266. Lahham E., Albandak M., Ayyad M., AlQadi M. Abdominal wall necrotizing fasciitis as a complication of strangulated hernia-an ominous consequence of a preventable scenario. Journal of Surgical Case Reports. 2023;2023(7):3.

267. Lakshminarayanan S., Giriyappa D., Kar S. The hidden imbalance in neighbourhood nutrition environment: a perspective from lower-middle-income country setting. Journal of Public Health Policy. 2023;44(3):415-434.

268. Lalani E., Menon R., Mufti M., Kumfa C., Raji M. Mirtazapine: A One-Stop Strategy for Treatment of Opioid Withdrawal Symptoms. Cureus Journal of Medical Science. 2023;15(8):6.

269. Latzer I., Richmond T., Zhang B., Pearl P. Eating disorders occur at high rates in adolescents with epilepsy and are associated with psychiatric comorbidities and suicidality. Epilepsia. 2023;():11.

270. Law S., Dong S., Zhou F., Zheng D., Wang C., Dong Z. Bariatric surgery and mental health outcomes: an umbrella review. Frontiers in Endocrinology. 2023;14():12.

271. Lay T., Nurchasanah F., Wanda D., Wardhany II, Agustin R., Haresaku S., Wimardhani Y., Mandasari M. Awareness, Attitudes, and Perceptions of Oral Healthcare among First Year Dental, Medical, and Nursing Students. Dentistry Journal. 2023;11(7):9.

272. Lazzeri M., Mastorci F., Piaggi P., Doveri C., Marinaro I., Trivellini G., Casu A., Devine C., Ait-Ali L., Vassalle C., Pingitore A. A Close Association between Body Weight, Health-Related Quality of Life, and Risk Behaviors in a Sample of Italian High School Students. Nutrients.

2023;15(24):13.

273. Lee K., Quek K., Ramadas A. Dietary and Lifestyle Risk Factors of Obesity Among Young Adults: A Scoping Review of Observational Studies. Current Nutrition Reports. 2023;():11.

274. Leth-Moller K., Hebebrand J., Strandberg-Larsen K., Baker J., Jensen B. Childhood body mass index and the subsequent risk of anorexia nervosa and bulimia nervosa among women: A large Danish population-based study. International Journal of Eating Disorders. 2023;():9.

275. Leutner M., Dervic E., Bellach L., Klimek P., Thurner S., Kautzky A. Obesity as pleiotropic risk state for metabolic and mental health throughout life. Translational Psychiatry. 2023;13(1):12.

276. Levine M., Tavernier R., Conlon R., Grace J., Sweeny G., Wang B., Cheng Y. Loss of control eating during pregnancy is associated with excessive gestational weight gain among individuals with overweight and obesity. Bmc Pregnancy and Childbirth. 2023;23(1):11.

277. Lewandowska K., Klinkosz W., Styk W., Kowalczyk M. Diversity of Binge-Eating Disorder Symptoms Is Associated with Anxiety about Getting Fat Rather Than Body Image: A Clinical Study of Women in Poland. Nutrients. 2023;15(21):10.

278. Lewis-Smith H., Pegram G., White P., Ward L., Diedrichs P. A short-form drama series created for the digital media environment: A randomised controlled trial exploring effects on girls' body satisfaction, acceptance of appearance diversity, and appearance-related internalised racism. Body Image. 2023;47():14.

279. Li J., Zhang Q., Xu C., Zhang Y., Lu Y., Ai M., Tan X. Differences in clinical characteristics and liver injury between patients diagnosed with the Omicron subvariant BA.5.2 and the prototype of SARS-CoV-2: a single center retrospective study. Bmc Gastroenterology. 2023;23(1):9.

280. Li M., Gao X., Miao T., Sun H. Identification of biomarkers of acne based on transcriptome analysis and combined with network pharmacology to explore the therapeutic mechanism of Jinhuang ointment. Medicine. 2023;102(44):16.

281. Li R., Li R., Xie J., Chen J., Liu S., Pan A., Liu G. Associations of socioeconomic status and healthy lifestyle with incident early-onset and late-onset dementia: a prospective cohort study. Lancet Healthy Longevity. 2023;4(12):693-702.

282. Li X., Cao X., Zhang J., Fu J., Mohedaner M., Danzengzhuoga B., Sun X., Yang G., Yang Z., Kuo C., Chen X., Cohen A., Liu Z. Accelerated aging mediates the associations of unhealthy lifestyles with cardiovascular disease, cancer, and mortality. Journal of the American Geriatrics Society. 2023;():13.

283. Li Y., Ding Q., Li X. The efficacy of psychological treatments on adolescent psychopathology: a narrative review. Translational Pediatrics. 2023;12(6):1225-+.

284. Li Z., Jia J., Hao H., Qiao S., Zhang Q., Zhang X., Qi Y., Sun X., Wang K., Gu R., Kang L., Xu B. Anti-Diabetic Drugs Inhibit Bulimia Induced Obesity. Frontiers in Bioscience-Landmark. 2023;28(5):11.

285. Liang G., Cheng Y., Barnhart W., Song J., Lu T., He J. A network analysis of disordered eating symptoms, big-five personality traits, and psychological distress in Chinese adults. International Journal of Eating Disorders. 2023;56(10):1842-1853.

286. Liang K., Li X., Ma J., Yang H., Shi X., Fan Y., Yang D., Guo D., Liu C., Dong L., Chang Q., Gu Q., Chen S., Li D. Predictors of dopamine dysregulation syndrome in patients with early Parkinson's disease. Neurological Sciences. 2023;():10.

287. Liang X., He X., Liu Q., Ren Y., Xu S., Chen L., Wang F., Bi Y., Peng Z. The impact of dietary and sleep rhythms on blood pressure in children and adolescents: a cross-sectional study. Hypertension Research. 2023;():14.

288. Liao J., Hu W., Wang W., Wang X., Yu S., Niu X., Zhu W., Zhou B., Song Y., Zeng W., Lu Z., Chen J. Plasma metabonomics of classical swine fever virus-infected pigs. Frontiers in Veterinary Science. 2023;10():26.

289. Lieberman A., Robison M., Wonderlich S., Crosby R., Mitchell J., Crow S., Peterson C., Le Grange D., Bardone-Cone A., Kolden G., Joiner T. Self-hate, dissociation, and suicidal behavior in bulimia nervosa. Journal of Affective Disorders. 2023;335():44-48.

290. Lim M., Nam Y. Gut microbiome in healthy aging versus those associated with frailty. Gut Microbes. 2023;15(2):18.

291. Lindekilde N., Diaz L., Lasgaard M., Henriksen J., Scheuer S., Andersen G., Rubin K., Pouwer F. Elevated risk of developing type 2 diabetes in people with a psychiatric disorder: What is the role of health behaviors and psychotropic medication?. Journal of Diabetes and Its Complications. 2023;37(11):9.

292. Lindgreen P., Willaing I., Clausen L., Ismail K., Gronbæk H., Andersen C., Persson F., Cleal B. "I Haven't Told Anyone but You": Experiences and Biopsychosocial Support Needs of People With Type 2 Diabetes and Binge Eating. Qualitative Health Research. 2024;():14.

293. Liu C., Dai M., Tian K., Zhou S., Luo L., Zeng Z., Yan X., Xiao Y., Wang Y., Deng R., Lei X., Liu T. Association of remnant cholesterol with CVD incidence: a general population cohort study in Southwest China. Frontiers in Cardiovascular Medicine. 2023;10():10.

294. Loftus I., Umana E., Scholtz I., McElwee D. Mackler's Triad: An Evolving Case of Boerhaave Syndrome in the Emergency Department. Cureus Journal of Medical Science. 2023;15(4):6.

295. Lok C., Wong M., Yip K., Ching W., Choi E. Validation of the traditional Chinese version of the diabetes eating problem survey-revised and study of the prevalence of disordered eating patterns in Chinese patients with type 1 DM. Bmc Psychiatry. 2023;23(1):17.

296. López M., Marchena-Giráldez C., Bernabéu-Brotons E. Nutrient intake, alcohol consumption, emotional eating and anxiety in women nursing students. Heliyon. 2023;9(12):16.

297. Lopez-Barreiro J., Garcia-Soidan J., Alvarez-Sabucedo L., Santos-Gago J. Practical Approach to Designing and Implementing a Recommendation System for Healthy Challenges. Applied Sciences-Basel. 2023;13(17):18.

298. López-Gómez L., Alcorta A., Abalo R. Probiotics and Probiotic-like Agents against Chemotherapy-Induced Intestinal Mucositis: A Narrative Review. Journal of Personalized Medicine. 2023;13(10):34.

299. Lydecker J., Winschel J., Gilbert K., Cotter E. School absenteeism and impairment associated with weight bullying. Journal of Adolescence. 2023;95(7):1478-1487.

300. Ma R., Cheah C., Buchanan N., Barman S. An examination of individual, relational, and cultural risk for disordered eating in Asian American college students. Journal of American College Health. 2023;():12.

301. Maas J., Simeunovic-Ostojic M., Bodde N. Is a dissonance-based group intervention targeting thin-ideal internalization a successful potential add-on for specialized eating disorder care? A randomized feasibility and acceptability pilot study. Journal of Eating Disorders. 2023;11(1):18.

302. Macit-Çelebi M., Özata-Uyar G., Yildiran H., Köksal E. Is Adherence to the Mediterranean Diet Associated with Eating Behaviour and Emotional Appetite in Young Women?. Revista Espanola De Nutricion Humana Y Dietetica. 2022;27(2):115-124.

303. Mack R., Kelleher K., Bhattarai J., Spence T. Individuals with Eating Disorders' Perspectives on a Meal Preparation Intervention. Occupational Therapy in Mental Health. 2023;():20.

304. Maia B., Oliveira-Cardoso E., Santos M. Eating disorders during the COVID-19 pandemic: scoping review of psychosocial impact. Middle East Current Psychiatry-Mecpsych. 2023;30(1):14.

305. Malekpour P., Hasanzadeh R., Masroor M., Chaman R., Motaghi Z. Effectiveness of a mixed lifestyle program in couples undergoing assisted reproductive technology: a study protocol. Reproductive Health. 2023;20(1):8.

306. Mallaram G., Sharma P., Kattula D., Singh S., Pavuluru P. Body image perception, eating disorder behavior, self-esteem and quality of life: a cross-sectional study among female medical students. Journal of Eating Disorders. 2023;11(1):9.

307. Mani R., Dogra N., Katare D. The Connection between Chronic Liver Damage and Sporadic Alzheimer's Disease: Evidence and Insights from a Rat Model. Brain Sciences. 2023;13(10):25.

308. Mansour M., Nassar K., Masri M., Kanas M., Aldrea F., Alzaylaa Y., Salloum H., Ahmad Y., Samha R., Kanjawi O. A massive primary hydatid splenic cyst was successfully managed through open total splenectomy: a case report and review article. Annals of Medicine and Surgery. 2023;85(10):5208-5213.

309. Mardani P., Kamran H., Ghaderpanah R., Geramizadeh B., Fouladi D., Shahriarirad R., Amirian A. A massive immature mediastinal teratoma treated with chemotherapy and surgical resection: a case report. Journal of Cardiothoracic Surgery. 2023;18(1):7.

310. Marek S., Forbes G., Avery R., Zanganeh T., Davidson S., Decarlo E., Kumar P., Hammersmith K. Potential blindness from nutritional xerophthalmia in autistic patients. Journal of Aapos. 2023;27(4):4.

311. Mares L., Davenport R., Kiropoulos L. Adverse Childhood Experiences and Depression, Anxiety, and Eating Disorders: The Mediating Role of Intolerance of Uncertainty and Emotion Regulation Difficulty. Traumatology. 2023;():13.

312. Martin R., Davis A., Pigott A., Cremona A. A scoping review exploring the role of the dietitian in the identification and management of eating disorders and disordered eating in adolescents and adults with type 1 diabetes mellitus. Clinical Nutrition Espen. 2023;58():375-387.

313. Martin-Wagar C., Attaway S., Melcher K. Differences among feminist and non-feminist women on weight bias internalization, body image, and disordered eating. Journal of Eating Disorders. 2023;11(1):11.

314. Maunder K., Molloy E., Jenkins E., Hayden J., Adamis D., McNicholas F. Anorexia Nervosa in vivo cytokine production: a systematic review. Psychoneuroendocrinology. 2023;158():17.

315. Mazza E., Troiano E., Mazza S., Ferro Y., Abbinante A., Agneta M., Montalcini T., Pujia A. The impact of endometriosis on dietary choices and activities of everyday life: a cross-sectional study. Frontiers in Nutrition. 2023;10():11.

316. Mazzolani B., Smaira F., Esteves G., Santini M., Leitao A., Santo André H., Gualano B., Roschel H. Disordered Eating Attitudes and Food Choice Motives Among Individuals Who Follow a Vegan Diet in Brazil. Jama Network Open. 2023;6(6):12.

317. McElroy S., Coloma P., Berger B., Guerdjikova A., Joyce J., Liebowitz M., Pain S., Rabasa C. Efficacy, safety, and tolerability of nivasorexant in adults with binge eating disorder: A randomized, Phase II proof of concept trial. International Journal of Eating Disorders. 2023;56(11):2120-2130.

318. Mehanovic E., Rosso G., Cuomo G., Diecidue R., Maina G., Costa G., Vigna-Taglianti F. Risk Factors for Suicide Reattempt among Adolescents and Young Adults: The Role of Psychiatric Disorders. Psychiatric Quarterly. 2023;():16.

319. Melinda C., Brigitta S., Péter T., Andrea L., Antal D., Aron C., Nikoletta V., Bea P., Attila S., Irena S. The role of cognitive emotion regulation in disordered eating among chronically ill adolescents. Orvosi Hetilap. 2023;164(48):1895-1903.

320. Melisse B., Blankers M., Berg E., Jonge M., Lommerse N., Furth E., Dekker J., Beurs E. Economic evaluation of web-based guided self-help cognitive behavioral therapy-enhanced for binge-eating disorder compared to a waiting list: A randomized controlled trial. International Journal of Eating Disorders. 2023;():13.

321. Melles H., Duijvis S., Jansen A. Inhibitory Learning during Exposure Treatment in Anorexia Nervosa: A Practical Guide. Behavioral Sciences. 2023;13(5):18.

322. Mendia J., Zumeta L., Pascual A., Conejero S., Caicedo-Moreno A., Díaz V. Body and appearance-related self-conscious emotions, emotional regulation strategies, and disordered eating in adult men. Current Psychology. 2023;():18.

323. Mendoza R., Convertino A., Blashill A. A longitudinal study of potentially traumatic events and binge-purge eating disorder onset in children. Appetite. 2024;193():5.

324. Meneguzzo P., Ceccato E., Sala A., Santonastaso P. When time worsens framing: a longitudinal analysis of the psychological effects of the COVID-19 pandemic in women with an eating disorder and their healthy sisters. Archives of Womens Mental Health. 2023;():9.

325. Micai M., Fatta L., Gila L., Caruso A., Salvitti T., Fulceri F., Ciaramella A., D'Amico R., Del Giovane C., Bertelli M., Romano G., Schünemann H., Scattoni M. Prevalence of co-occurring conditions in children and adults with autism spectrum disorder: A systematic review and meta-analysis. Neuroscience and Biobehavioral Reviews. 2023;155():10.

326. Miletta M., Horvath T. Construction of Activity-based Anorexia Mouse Models. Bio-Protocol. 2023;13(15):8.

327. Minor R., Huckins G., Hawkins S., Townsend A., Sample S., Tolliver S., Loeber S., Brandao J., Doss G. Diagnosis and surgical correction of gastrointestinal obstruction secondary to an intestinal trichophytobezoar in a red-necked wallaby<i> (Notamacropus</i> rufogriseus). Journal of Exotic Pet Medicine. 2023;46():1-4.

328. Miranda C., Garcia A., Watts C., Timko C. BODY EMPOWERMENT PROJECT: EATING DISORDER PREVENTION IN YOUTH. Journal of the American Academy of Child and Adolescent Psychiatry. 2023;62(10):S299-S299.

329. Miranda-Olivos R., Agüera Z., Granero R., Jiménez-Murcia S., Puig-Llobet M., Lluch-Canut M., Gearhardt A., Fernández-Aranda F. The Role of Food Addiction and Lifetime Substance Use on Eating Disorder Treatment Outcomes. Nutrients. 2023;15(13):12.

330. Miskovic-Wheatley J., Bryant E., Ong S., Vatter S., Le A., Touyz S., Maguire S., Natl Eating Disorder Res Consortium Eating disorder outcomes: findings from a rapid review of over a decade of research. Journal of Eating Disorders. 2023;11(1):25.

331. Miyachi Y., Sakiko K., Yokoi T., Kaido T. Rapidly progressing severe coagulopathy and thrombocytopenia in extreme anorexia nervosa patient with small bowel strangulation: A case report. International Journal of Surgery Case Reports. 2023;112():5.

332. Mizuno A., Kaneko H., Suzuki Y., Okada A., Takeda N., Morita H., Fujiu K., Node K., Yasunaga H., Komuro I. Enduring Relevance of the Stages of Change Model for Transforming Lifestyle Behaviors. Circulation Journal. 2023;87(8):1138-1142.

333. Mol T., Thompson J., Fuller-Tyszkiewicz M. A meta-analytic review of impact of measurement choice on RCTs to reduce appearance internalization. International Journal of Eating Disorders. 2023;():22.

334. Momeñe J., Estévez A., Herrero M., Griffiths M., Olave L., Iruarrizaga I. Emotional regulation and body dissatisfaction: the mediating role of anger in young adult women. Frontiers in Psychiatry. 2023;14():10.

335. Monteleone A., Barone E., Cascino G., Schmidt U., Gorwood P., Volpe U., Abbate-Daga G., Castellini G., Marsá M., Favaro A., Fukutomi A., Guillaume S., Minarík P., Pacheco J., Panero M., Papezová H., Ricca V., Segura-Garcia C., Scanferla E., Tyszkiewicz-Nwafor M., Fernandez-Aranda F., Voderholzer U., Treasure J., Monteleone P. Pathways to eating disorder care: A European multicenter study. European Psychiatry. 2023;66(1):9.

336. Moraes J., Sproesser G., Alvarenga M. Food healthiness judgements among Brazilian and German lay adults. Nutrition Bulletin. 2023;():18.

337. Morales-Suárez-Varela M., Amezcua-Prieto C., Llopis-Gonzalez A., Perez C., Mateos-Campos R., Hernández-Segura N., Ortiz-Moncada R., Almaraz A., Alguacil J., Rodríguez M., Abellán G., Alonso-Molero J., Martínez-Ruiz V., Santana-Garcia I., Cancela J., Juan L., Martín-Peláez S., Fernández-Villa T., Grp Invest UniHcos Prevalence of Depression and Fish Consumption among First Year Spanish University Students: UniHcos Project. Nutrients. 2023;15(12):17.

338. Moreau C., Tran L., Ayrolles A., Bonicel R., Bergeret P., Traut N., Stordeur C., Thompson P., Bourgeron T., Delorme R. Brain Abnormalities in Children With Early-Onset Anorexia. Biological Psychiatry. 2023;93(9):S90-S90.

339. Moslehi N., Golzarand M., Mirmiran P., Hosseinpanah F., Azizi F. Macronutrient quality and the incidence of metabolically unhealthy phenotypes in adults with normal weight and overweight/obesity. Obesity Research & Clinical Practice. 2023;17(5):369-377.

340. Mosuka E., Murugan A., Thakral A., Ngomo M., Budhiraja S., St Victor R. Clinical Outcomes of Refeeding Syndrome: A Systematic Review of High vs. Low-Calorie Diets for the Treatment of Anorexia Nervosa and Related Eating Disorders in Children and Adolescents. Cureus Journal of Medical Science. 2023;15(5):10.

341. Moubayed D., Chadi N. An innovative inpatient protocol for alcohol withdrawal prevention in a 16-year-old adolescent: a case report. Journal of Medical Case Reports. 2023;17(1):7.

342. Muhammad A., Xiao Z., Lin W., Zhang Y., Meng T., Ning J., Xu H., Tang R., Xiao X. Acute interstitial nephritis caused by ANCA-associated vasculitis: a case based review. Clinical Rheumatology. 2023;():18.

343. Muir X., Preece D., Becerra R. Alexithymia and eating disorder symptoms: the mediating role of emotion regulation. Australian Psychologist. 2023;():11.

344. Munoz-Mireles G., Mantzios M., Schellinger J., Messiah S., Marroquin E. Mindful Eating as a Tool for Diabetes Prevention and Management: A Review of Potential Mechanisms of Action. Mindfulness. 2023;():17.

345. Nagata J., Smith-Russack Z., Paul A., Saldana G., Shao I., Al-Shoaibi A., Chaphekar A., Downey A., He J., Murray S., Baker F., Ganson K. The social epidemiology of binge-eating disorder and behaviors in early adolescents. Journal of Eating Disorders. 2023;11(1):9.

346. Najafi F., Mohseni P., Pasdar Y., Niknam M., Izadi N. The association between dietary amino acid profile and the risk of type 2 diabetes: Ravansar non-communicable disease cohort study. Bmc Public Health. 2023;23(1):11.

347. Nakamura H., Kawazoe A., Okunaka M., Demachi K., Kotani D., Shitara K. Safety and Efficacy of Trifluridine/Tipiracil Administered After Anti-PD-1 Therapies for Advanced Gastric Cancer. In Vivo. 2023;37(4):1729-1734.

348. Napp A., Kaman A., Erhart M., Westenhöfer J., Ravens-Sieberer U. Eating disorder symptoms among children and adolescents in Germany before and after the onset of the COVID-19 pandemic. Frontiers in Psychiatry. 2023;14():13.

349. Newberry-Dupé J., Chu W., Craig S., Borschmann R., O'Reilly G., Yates P., Melvin G., King K., Hiscock H. Adult Mental Health Presentations to Emergency Departments in Victoria, Australia between January 2018 and October 2020: Changes Associated with COVID-19 Pandemic Public Health Restrictions. Psychiatric Quarterly. 2023;():20.

350. Nguyen C., Phan H., Lee C., Do L. Assessing Awareness of Colorectal Cancer Symptoms among Outpatients: A Cross-Sectional Study at a Hospital in Vietnam. Healthcare. 2023;11(23):11.

351. Nguyen T., Rokhman M., Stiensma I., Hanifa R., Ong T., Postma M., Schans J. Cost-effectiveness of non-communicable disease prevention in Southeast Asia: a scoping review. Frontiers in Public Health. 2023;11():27.

352. Nicanor-Carreón J., Seyedsadjadi N., Rowitz B., Pepino M. Weight Regain and Ingestive Behavior in Women after Metabolic Surgery. Nutrients. 2023;15(17):14.

353. Nindenshuti P., Caire-Juvera G. Changes in Diet, Physical Activity, Alcohol Consumption, and Tobacco Use in Adults During the COVID-19 Pandemic: A Systematic Review. Inquiry-the Journal of Health Care Organization Provision and Financing. 2023;60():13.

354. Niu J., Xu D., Huang Y., You J., Zhang J., Li J., Su D., Lin S., Suo L., Ma J., Wu S. Sex-related association of modifiable risk factors with hypertension: A national cross-sectional study of NHANES 2007-2018. Clinical Cardiology. 2023;():8.

355. Noone J., Rooney M., Karavyraki M., Yates A., O'Sullivan S., Porter R. Cancer-Cachexia-Induced Human Skeletal Muscle Myotube Degeneration Is Prevented via Cannabinoid Receptor 2 Agonism In Vitro. Pharmaceuticals. 2023;16(11):15.

356. Nordin-Bates S., Lundström P., Melin A., Sundgot-Borgen J., Edlund K. Evaluation of DancExcellent, a Combined CBT and Nutrition Education Intervention. Medical Problems of Performing Artists. 2023;38(2):71-79.

357. Norton L., Parkinson J., MacGuinness M., Harris N., Hart L. Examining the feasibility of a brief parent intervention designed to promote positive food communication with infants. Pilot and Feasibility Studies. 2023;9(1):12.

358. Nymberg V., Nymberg P., Pikkemaat M., Calling S., Stenman E., Grundberg A., Smith J., Sundquist K. Lifestyle and cardiovascular risk factors in a Swedish primary care population with self-reported psychiatric symptoms. Preventive Medicine Reports. 2024;37():8.

359. O'Connor L., Downs C., Langan J., Chinnadurai S., Adkesson M., Ivancic M., Aitken-Palmer C. Perioperative management of umbilical herniorrhaphy with ileal resection and anastomosis in a pygmy hippopotamus (<i>Choeropsis liberiensis</i>). Veterinary Record Case Reports. 2023;():7.

360. Öcalan S., Kovanci M., Hiçdurmaz D. 'It is not a mannequin disease': A lived experience narrative of living with bulimia nervosa. Journal of Psychiatric and Mental Health Nursing. 2023;():7.

361. Ohashi Y., Wang S., Shingleton R., Nock M. Body dissatisfaction, ideals, and identity in the development of disordered eating among adolescent ballet dancers. International Journal of Eating Disorders. 2023;():9.

362. Ohrt T., Perez M., Iida M., Luecken L., Lemery-Chalfant K., Liew J. The Interdependent Nature of Mother's and Children's Temperament and Eating Behaviors on Weight. Maternal and Child Health Journal. 2023;27(8):1382-1391.

363. Ohta H., Horii T., Yasu T. Adverse metabolic effects on glucose in patients receiving anamorelin using a Japanese claims database. Oncology. 2023;():11.

364. Olson E., Miro E., Roche M., Mehta S., Sainani K., Kraus E. Mental Health Matters: A Cross-Sectional Survey on Depression and Anxiety Symptoms and the Female and Male Athlete Triad. Clinical Journal of Sport Medicine. 2023;33(4):368-375.

365. Önal S., Sachadyn-Król M., Kostecka M. A Review of the Nutritional Approach and the Role of Dietary Components in Children with Autism Spectrum Disorders in Light of the Latest Scientific Research. Nutrients. 2023;15(23):22.

366. Osadchuk M., Tikhonova Y., Krivetskaya M. The issue of vaccine refusal: the study of a risky behavior. Clinical and Experimental Vaccine Research. 2023;12(3):216-223.

367. Osam C., Hope H., Ashcroft D., Abel K., Pierce M. Maternal mental illness and child atopy: a UK population-based, primary care cohort study. British Journal of General Practice. 2023;():8.

368. Oudkerk J., Grenade C., Davarpanah A., Vanheusden A., Vandenput S., Mainjot A. Risk factors of tooth wear in permanent dentition: A scoping review. Journal of Oral Rehabilitation. 2023;50(10):1110-1165.

369. Oyabu M., Ono Y., Fujimaki S., Yoshioka K., Fujimaki J., Kawaguchi R., Hatazawa Y., Miura S., Kamei Y. Loss of FOXOs in skeletal muscle prevents the onset of cancer cachexia-induced muscle atrophy. Annals of Nutrition and Metabolism. 2023;79():292-292.

370. Özbey H., Bayat M., Kalkan I., Hatipoglu N. Diabetes-specific eating disorder and social exclusion in adolescents with type 1 diabetes. International Journal of Diabetes in

Developing Countries. 2023;():6.

371. Ozsvar J., Gissler M., Lavebratt C., Nilsson I. Exposures during pregnancy and at birth are associated with the risk of offspring eating disorders. International Journal of Eating Disorders. 2023;():18.

372. Öztürk PÇ, Ouyaba A. Prevalence and related factors of eating disorders in pregnancy: a systematic review and meta-analysis. Archives of Gynecology and Obstetrics. 2023;():15.

373. Pacanowski C., Dominick G., Crosby R., Engel S., Cao L., Linde J. Daily self-weighing compared with an active control causes greater negative affective lability in emerging adult women: A randomized trial. Applied Psychology-Health and Well Being. 2023;15(4):1695-1713.

374. Palermo M., Rancourt D. Anxiety, body dissatisfaction, and exercise identity: Differentiating between adaptive and compulsive exercise. Eating Behaviors. 2023;49():4.

375. Panyod S., Wu W., Hu M., Huang H., Chen R., Chen Y., Shen T., Ho C., Liu C., Chuang H., Huang C., Wu M., Sheen L. Healthy diet intervention reverses the progression of NASH through gut microbiota modulation. Microbiology Spectrum. 2023;():20.

376. Park C., Choi E., Han K., Ahn H., Kwon S., Lee S., Oh S., Lip G. Increased cardiovascular events in young patients with mental disorders: a nationwide cohort study. European Journal of Preventive Cardiology. 2023;30(15):1582-1592.

377. Parpia R., Spettigue W., Norris M. Approach to anorexia nervosa and atypical anorexia nervosa in adolescents. Canadian Family Physician. 2023;69(6):387-391.

378. Parsons M. Autism diagnosis in females by eating disorder professionals. Journal of Eating Disorders. 2023;11(1):7.

379. Paszynska E., Hernik A., Rangé H., Amaechi B., Gross G., Pawinska M. Diet Traps during Eating Disorders among Dentate Patients at an Oral Health Glance. Nutrients. 2023;15(20):18.

380. Pearce E., Birken M., Pais S., Tamworth M., Ng Y., Wang J., Chipp B., Crane E., Schlief M., Yang J., Stamos A., Cheng L., Condon M., Lloyd-Evans B., Kirkbride J., Osborn D., Pitman A.,

Johnson S. Associations between constructs related to social relationships and mental health conditions and symptoms: an umbrella review. Bmc Psychiatry. 2023;23(1):31.

381. Pegu S., Sonowal J., Deb R., Das P., Sengar G., Rajkhowa S., Gupta V. Clinicopathological and ultrastructural study of African swine fever outbreak in North-East India. Microbial Pathogenesis. 2023;185():8.

382. Peláez T., López-Carrillero R., Subirana-Mirete J., Ochoa S., Osma J. Applying the Unified Protocol for Transdiagnostic Treatment of Emotional Disorders in a Case of Ultra-High Risk for Psychosis With Emotional Comorbidity. Clinical Case Studies. 2023;():21.

383. Pelczynska M., Moszak M., Wesolek A., Bogdanski P. The Preventive Mechanisms of Bioactive Food Compounds against Obesity-Induced Inflammation. Antioxidants. 2023;12(6):30.

384. Pellizzer M., Wade T. Developing a definition of body neutrality and strategies for an intervention. Body Image. 2023;46():434-442.

385. Pena-Ramos J., Finch N., Sanchez-Redondo S. Suspected robenacoxib-induced liver injury in a dog. Veterinary Record Case Reports. 2023;():6.

386. Perez M., Hayes T., Frazier L. Beyond generalized anxiety: the association of anxiety sensitivity with disordered eating. Journal of Eating Disorders. 2023;11(1):15.

387. Pérez-Vázquez J., González-Roz A., Amigo-Vázquez I. Effectiveness of an e-Health Quasi-Randomized Controlled Universal Prevention Program for Eating Disorders in Spanish Adolescents. Journal of Prevention. 2023;():19.

388. Perrin E., Ravi H., Borra G., South A. Prevalence and risk factors of disordered eating behavior in youth with hypertension disorders. Pediatric Nephrology. 2023;38(11):3779-3789.

389. Philip B., Poku O., Attoh-Okine N., Presskreischer R. The need for epidemiological research on eating disorders in Africa and the Caribbean. International Journal of Eating Disorders. 2023;():6.

390. Pi S., Li Q., Li J., Long H., Xiao B. Clinical Reasoning: A 14-Year-Old Girl With Reversible Peripheral Neuropathy and Encephalopathy. Neurology. 2023;101(6):E665-E671.

391. Pitirut I., Swami V., Poama-Neagra T., Enea V. Appearance-based rejection sensitivity mediates the relationship between Instagram addiction and dysmorphic concerns in young adult women. Scandinavian Journal of Psychology. 2023;():9.

392. Pledger S., Ahmadizar F. Gene-environment interactions and the effect on obesity risk in low and middle-income countries: a scoping review. Frontiers in Endocrinology. 2023;14():14.

393. Podgórski R., Galiniak S., Mazur A., Podgórska D., Domin A. Serum Levels of Hormones Regulating Appetite in Patients with Fetal Alcohol Spectrum Disorders. Nutrients. 2023;15(19):15.

394. Poiani-Cordella C., Toh W., Phillipou A. Eating behaviours and personality characteristics of clinicians and researchers working in eating disorders. Eating Disorders. 2023;():17.

395. Pötgens S., Lecop S., Havelange V., Li F., Neyrinck A., Neveux N., Maertens J., Walter J., Schoemans H., Delzenne N., Bindels L. Gut microbiota alterations induced by intensive chemotherapy in acute myeloid leukaemia patients are associated with gut barrier dysfunction and body weight loss. Clinical Nutrition. 2023;42(11):2214-2228.

396. Poudel S., Sharma S., Gurung P., Kandel K., Aryal S., Maharjan S. Takayasu arteritis in a young male patient: a case report and review of literature. Annals of Medicine and Surgery. 2023;85(6):3133-3136.

397. Propper-Lewinsohn T., Gillon-Keren M., Shalitin S., Elran-Barak R., Yackobovitch-Gavan M., Fayman G., David M., Liberman A., Phillip M., Oron T. Disordered eating behaviours in adolescents with type 1 diabetes can be influenced by their weight at diagnosis and rapid weight gain subsequently. Diabetic Medicine. 2023;40(11):9.

398. Pruccoli J., Guardi G., La Tempa A., Valeriani B., Chiavarino F., Parmeggiani A. Food and Development: Children and Adolescents with Neurodevelopmental and Comorbid Eating Disorders-A Case Series. Behavioral Sciences. 2023;13(6):16.

399. Puchner E., Platzer M., Dalkner N., Schwalsberger K., Lenger M., Fellendorf F., Unterrainer H., Schwerdtfeger A., Reininghaus B., Reininghaus E. Effects of Metabolic Syndrome and Sex on Stress Coping Strategies in Individuals with Depressive Disorder. Metabolites. 2023;13(5):13.

400. Quadflieg N., Naab S., Schlegl S., Bauman T., Voderholzer U. Inpatient Treatment Outcome in a Large Sample of Adolescents with Anorexia Nervosa. Nutrients. 2023;15(19):14.

401. Raffoul A., Turner S., Salvia M., Austin S. Population-level policy recommendations for the prevention of disordered weight control behaviors: A scoping review. International Journal of Eating Disorders. 2023;():17.

402. Rahimabadi P., Nazari A., Kamyabi M., Mosavari N. Serological and Bacteriological Surveillance of Glanders Among Horses in Central Region of Iran. Journal of Equine Veterinary Science. 2023;127():7.

403. Ralph-Nearman C., Sandoval-Araujo L., Karem A., Cusack C., Glatt S., Hooper M., Pena C., Cohen D., Allen S., Cash E., Welch K., Levinson C. Using machine learning with passive wearable sensors to pilot the detection of eating disorder behaviors in everyday life. Psychological Medicine. 2023;():7.

404. Ram J., Shelke S. Understanding Eating Disorders in Children and Adolescent Population. Journal of Indian Association for Child and Adolescent Mental Health. 2023;19(1):60-69.

405. Ramos R., Vaz A., Rodrigues T., Baenas I., Fernández-Aranda F., Machado P. Exploring the relationship between emotion regulation, inhibitory control, and eating psychopathology in a non-clinical sample. European Eating Disorders Review. 2023;():14.

406. Rando-Cueto D., Heras-pedrosa C., Paniagua-Rojano F. Health Communication Strategies via TikTok for the Prevention of Eating Disorders. Systems. 2023;11(6):17.

407. Rane R., Musial M., Beck A., Rapp M., Schlagenhauf F., Banaschewski T., Bokde A., Martinot M., Artiges E., Nees F., Lemaitre H., Hohmann S., Schumann G., Walter H., Heinz A., Ritter K., Consortium Imagen Uncontrolled eating and sensation-seeking partially explain the prediction of future binge drinking from adolescent brain structure. Neuroimage-

Clinical. 2023;40():9.

408. Rantala E., Vanhatalo S., Perez-Cueto F., Pihlajamäki J., Poutanen K., Karhunen L., Absetz P. Acceptability of workplace choice architecture modification for healthy behaviours. Bmc Public Health. 2023;23(1):22.

409. Rao V., Shankar R., Rao G. Acute Vascular Events: Cellular and Molecular Mechanisms. International Journal of Biomedicine. 2023;13(3):9-16.

410. Ravanfar P., Rushmore R., Lyall A., Cropley V., Makris N., Desmond P., Velakoulis D., Shenton M., Bush A., Rossell S., Pantelis C., Syeda W., Phillipou A. Investigation of brain iron in anorexia nervosa, a quantitative susceptibility mapping study. Journal of Eating Disorders. 2023;11(1):10.

411. Ravanfar P., Rushmore R., Lyall A., Cropley V., Makris N., Desmond P., Velakoulis D., Shenton M., Bush A., Rossell S., Pantelis C., Syeda W., Phillipou A. Investigation of brain iron in anorexia nervosa, a quantitative susceptibility mapping study. Journal of Eating Disorders. 2023;11(1):10.

412. Ren W., Qi Y., Liu Y., Yan Y., Zheng X., Jin S., Chang Y. Evaluation of risk factors for impulse control disorder in Parkinson's disease in northern China. Frontiers in Aging Neuroscience. 2023;15():10.

413. Requena-Ocaña N., Flores-López M., García-Marchena N., Pavón-Morón F., Pedraza C., Wallace A., Castilla-Ortega E., Fonseca F., Serrano A., Araos P. Plasma Lysophosphatidic Acid Concentrations in Sex Differences and Psychiatric Comorbidity in Patients with Cocaine Use Disorder. International Journal of Molecular Sciences. 2023;24(21):19.

414. Rios J., Berg M., Gearhardt A. Evaluating Bidirectional Predictive Pathways between Dietary Restraint and Food Addiction in Adolescents. Nutrients. 2023;15(13):10.

415. Ritz L., Mauny N., Leconte P., Margas N. French validation of the Compensatory Eating and Behaviors in Response to Alcohol Consumption Scale (CEBRACS) in a university student sample. Eating and Weight Disorders-Studies on Anorexia Bulimia and Obesity. 2023;28(1):11.

416. Rives C., Martin C., Evariste L., Polizzi A., Huillet M., Lasserre F., Alquier-Bacquie V., Perrier P., Gomez J., Lippi Y., Naylies C., Levade T., Sabourdy F., Remignon H., Fafournoux P., Chassaing B., Loiseau N., Guillou H., Ellero-Simatos S., Gamet-Payrastre L., Fougerat A. Dietary Amino Acid Source Elicits Sex-Specific Metabolic Response to Diet-Induced NAFLD in Mice. Molecular Nutrition & Food Research. 2023;():15.

417. Robinson S., Granic A., Cruz-Jentoft A., Sayer A. The role of nutrition in the prevention of sarcopenia. American Journal of Clinical Nutrition. 2023;118(5):852-864.

418. Rohde P., Bearman S., Pauling S., Gau J., Shaw H., Stice E. Setting and Provider Predictors of Implementation Success for an Eating Disorder Prevention Program Delivered by College Peer Educators. Administration and Policy in Mental Health and Mental Health Services Research. 2023;50(6):912-925.

419. Rohrbach P., Fokkema M., Spinhoven P., Van Furth E., Dingemans A. Predictors and moderators of three online interventions for eating disorder symptoms in a randomized controlled trial. International Journal of Eating Disorders. 2023;56(10):1909-1918.

420. Rojo M., Beltrán-Garrayo L., Blanco-Barredo M., Sepúlveda A. Spanish validation of two social media appearance-related constructs associated with disordered eating in adolescents: The Appearance-related Social Media Consciousness scale (ASMC) and the Critical Thinking about Media Messages scale (CTMM). Body Image. 2023;45():401-413.

421. Rolle C., Ng G., Nho Y., Barbosa D., Shivacharan R., Gold J., Bassett D., Halpern C., Buch V. Accumbens connectivity during deep-brain stimulation differentiates loss of control from physiologic behavioral states. Brain Stimulation. 2023;16(5):1384-1391.

422. Rometsch C., Guthoff M., Zipfel S., Stengel A. Renal failure following insulin purging in atypical anorexia nervosa and type 1 diabetes mellitus. Frontiers in Psychiatry. 2023;14():5.

423. Rondanelli M., Gasparri C., Razza C., Ferraris C., Perna S., Ferrarotti I., Corsico A. Practical dietary advices for subjects with alpha-1 antitrypsin deficiency. Biomedicine & Pharmacotherapy. 2023;163():9.

424. Rozgonjuk D., Ignell J., Mech F., Rothermund E., Gündel H., Montag C. Smartphone and Instagram use, body dissatisfaction, and eating disorders: investigating the associations using self-report and tracked data. Journal of Eating Disorders. 2023;11(1):11.

425. Ruan H., Sun J., Zhao K., He M., Yuan C., Fu L., Shen N. Subjective Toxicity Profiles of Children With Cancer During Treatment. Cancer Nursing. 2024;47(1):E1-E9.

426. Ruffieux Y., Wettstein A., Maartens G., Folb N., Mesa-Vieira C., Didden C., Tlali M., Williams C., Cornell M., Schomaker M., Johnson L., Joska J., Egger M., Haas A. Life years lost associated with mental illness: A cohort study of beneficiaries of a South African medical insurance scheme. Journal of Affective Disorders. 2023;340():204-212.

427. Russell J., Hunt G. Oxytocin and eating disorders: Knowledge gaps and future directions. Psychoneuroendocrinology. 2023;154():7.

428. Sadiq I. Lifestyle medicine as a modality for prevention and management of chronic diseases. Journal of Taibah University Medical Sciences. 2023;18(5):1115-1117.

429. Sakamoto N., Hamada S., Takahashi H., Satou R., Suzuki M., Maeno T. Improvement of Intestinal Pseudo-Obstruction by Total Parenteral Nutrition in a Young Woman With Mitochondrial Myopathy, Encephalopathy, Lactic Acidosis, and Stroke-Like Episodes: A Case Report. Cureus Journal of Medical Science. 2023;15(12):10.

430. Sakouhi M., Matmour D., Belakhdar K., Kraroubi A. Cross-Sectional Study on Adverse Effects of Metformin Hydrochloride on 130 Patients Type 2 Diabetic Admitted to Medical Center and Diabetes Home of Sidi Bel-Abbes. Annales Pharmaceutiques Francaises. 2023;81(4):684-695.

431. Sala M., Levinson C., Kober H., Roos C. A Pilot Open Trial of a Digital Mindfulness-Based Intervention for Anorexia Nervosa. Behavior Therapy. 2023;54(4):637-651.

432. Sambu W., Picchioni F., Stevano S., Codjoe E., Nkegbe P., Turner C. Food systems thinking unpacked: a scoping review on industrial diets among adolescents in Ghana. Food Security. 2023;():36.

433. Sandgren S., Haycraft E., Arcelus J., Plateau C. An intervention mapping adaptation framework to develop a self-help intervention for athletes with eating disorder symptoms. International Journal of Eating Disorders. 2023;56(11):2022-2031.

434. Sanjuán-Navarro P., Agudelo-Suárez A., Mora-Cárdenas A., Angarita-Navarro M., Valdés-Payares L., Martínez-Gómez M., Salazar-González C. Frequency of symptoms and the

associated factors of eating disorders in a group of dental students in Medellin, Colombia. Dental and Medical Problems. 2023;60(3):401-411.

435. Santoncini C., Márquez M., Vazquez C., Carriedo A., Márquez J., Morcelle G., Gutiérrez M. Sex and Body Mass Index differences after one-year follow-up of an eating disorders risk factors universal prevention intervention in university students in Mexico City. Salud Mental. 2023;46(3):147-154.

436. Sawalha K., Asad R., Habash F., López-Candales A. Profiling Cardiometabolic Health in Jordan: A Call to Action to Improve Cardiovascular Health. Cureus Journal of Medical Science. 2023;15(5):8.

437. Scaglia E., Reggi S., Canala B., Frazzini S., Dell'Anno M., Hejna M., Rossi L. The Effects of Milk Replacer Supplemented with <i>Ascophyllum nodosum</i> as a Novel Ingredient to Prevent Neonatal Diarrhea in Dairy Calves and Improve Their Health Status. Veterinary Sciences. 2023;10(10):15.

438. Schaefer L., Forester G., Dvorak R., Steinglass J., Wonderlich S. Integrating aspects of affect, reward, and cognition to develop more comprehensive models of binge-eating pathology. International Journal of Eating Disorders. 2023;():9.

439. Schankweiler P., Raddatz D., Ellrott T., Cirkel C. Correlates of Food Addiction and Eating Behaviours in Patients with Morbid Obesity. Obesity Facts. 2023;16(5):465-474.

440. Schell S., Racine S. Reconsidering the role of interpersonal stress in eating pathology: Sensitivity to rejection might be more important than actual experiences of peer stress. Appetite. 2023;187():12.

441. Schienle A., Unger I. Open-label placebo treatment for reducing overeating in children: A study protocol for a randomized clinical trial with an app-assisted approach. Contemporary Clinical Trials Communications. 2023;34():5.

442. Schlapfer L., Gettis M., Dutreuil V., Cherven B. Hospitalization patterns for adolescents with eating disorders during COVID-19. Journal of Eating Disorders. 2023;11(1):9.

443. Schmerler J., Chiu A., Agarwal A., Kreulen R., Srikumaran U., Best M. Increased prevalence of lower extremity soft tissue injuries and surgeries in patients with anorexia

nervosa and bulimia nervosa. Physician and Sportsmedicine. 2023;():7.

444. Schmidt R., Wandrer H., Boutelle K., Kiess W., Hilbert A. Associations between eating in the absence of hunger and executive functions in adolescents with binge-eating disorder: An experimental study. Appetite. 2023;186():9.

445. Schneidergruber T., Blechert J., Arzt S., Pannicke B., Reichenberger J., Arend A., Ginzinger S. Predicting food craving in everyday life through smartphone-derived sensor and usage data. Frontiers in Digital Health. 2023;5():10.

446. Schnupp C., Sattel H., Morawa E., Kronester M., Sack M., Schaeflein E. Autopilot Functionality and Self-Destructive Behavior in Patients With Complex Dissociative Disorders-A Qualitative Study. Psychological Trauma-Theory Research Practice and Policy. 2023;():8.

447. Scimeca R., Reichard M. Differential gene expression response to acute and chronic<i> Cytauzxoon</i><i> felis</i> infection in domestic cats<i> (Felis</i><i> catus)</i>. Ticks and Tick-Borne Diseases. 2023;14(6):7.

448. Sciscent B., Hallan D., Rizk E. Mental health and substance use disorders in young adults with spina bifida. Journal of Neurosurgery-Pediatrics. 2023;31(6):633-639.

449. Seemi T., Sharif H., Sharif S., Naeem H., Naeem F., Fatima Z. Anxiety levels among school-going adolescents in peri-urban areas of Karachi, Pakistan. Plos One. 2023;18(10):14.

450. Seminog O., Thakrar D., James A., Goldacre M. Low risk of some common cancers in women with anorexia nervosa: Evidence from a national record-linkage study. Acta Psychiatrica Scandinavica. 2023;148(1):71-80.

451. Sempere-Ferre F., Escrivá D., Caplliure-Llopis J., Benet I., Barrios C. Eating Disorder Attitudes and Body Self-Image of Urban Mediterranean Adolescents. Children-Basel. 2023;10(6):11.

452. Sescu D., Chansiriwongs A., Minta K., Vasudevan J., Kaliaperumal C. Early Preventive Strategies and CNS Meningioma - Is This Feasible? A Comprehensive Review of the

Literature. World Neurosurgery. 2023;180():123-133.

453. Setiawan A., Budiarto A., Indriyanti R. Eating behavior of adolescent girls in countries with a high prevalence of stunting under five: a systematic review. Frontiers in Psychology. 2023;14():20.

454. Shaheen N., Shamim A., Choudhury S., Sarwar S., Ashraf M., Bahar N., Al Mamun M., Sobhan S., Abedin M., Karim M., Amin M., Alim A. Commonly consumed processed packaged foods in Bangladesh are unhealthy and their nutrient contents are not in conformity with the label declaration. Food Science & Nutrition. 2023;():13.

455. Shahmohamadi E., Sedaghat M., Rahmani A., Larti F., Geraiely B. "Recognition of heart attack symptoms and treatment-seeking behaviors: a multi-center survey in Tehran, Iran". Bmc Public Health. 2023;23(1):11.

456. Sharma A., Vidal C. A scoping literature review of the associations between highly visual social media use and eating disorders and disordered eating: a changing landscape. Journal of Eating Disorders. 2023;11(1):11.

457. Sharma N., Bhattarai M., Baral K., Poudel S., Hassan N., Dhakal T., Baral R. Vitamin B12 deficiency in an infant with neurological and hematological findings: A case report. Clinical Case Reports. 2023;11(8):4.

458. Shekhar K., Pathak M., Pisulkar G. Diet and Lifestyle Impact on Rheumatoid Arthritis: A Comprehensive Review. Cureus Journal of Medical Science. 2023;15(11):11.

459. Sherry D., Mondal A., McGill M., Gmuca S. Pediatric Complex Regional Pain Syndrome With and Without a History of Prior Physical Trauma at Onset. Clinical Journal of Pain. 2023;39(9):437-441.

460. Shinsugi C., Takimoto H. Trends in Mean Energy and Nutrient Intakes in Japanese Children and Adolescents: The National Health and Nutrition Survey, 1995-2019. Nutrients. 2023;15(15):24.

461. Simons E., Noteboom F., Furth E. Pro-anorexia coaches prey on individuals with eating disorders. International Journal of Eating Disorders. 2023;():8.

462. Simpson C., Boutte R., Burnette C., Weinstock M., Goel N., Mazzeo S. Cultural adaptation of an integrated eating disorders prevention and healthy weight management program. Journal of Eating Disorders. 2023;11(1):13.

463. Singh S., Contrucci A. Superior mesenteric artery syndrome and anorexia nervosa: a case report. Journal of Medical Case Reports. 2023;17(1):4.

464. Snits A., Rozner L., Shoshan S., Peles E. Exposure and addiction rates to substances and behaviours among Israeli university students. Heroin Addiction and Related Clinical Problems. 2023;25(4):23-32.

465. Snyder H., Pai N., Meaney B., Birbeck C., Whitney R., Johnson N., Rosato L., Jones K. Significant vomiting and weight loss in a pediatric epilepsy patient secondary to vagus nerve stimulation: A case report and review of the literature. Epilepsy & Behavior Reports. 2023;24():5.

466. Solmi M., Seitidis G., Mavridis D., Correll C., Dragioti E., Guimond S., Tuominen L., Dargél A., Carvalho A., Fornaro M., Maes M., Monaco F., Song M., Shin J., Cortese S. Incidence, prevalence, and global burden of schizophrenia-data, with critical appraisal, from the Global Burden of Disease (GBD) 2019. Molecular Psychiatry. 2023;():9.

467. Soroceanu R., Soroceanu A., Timofte D., Timofeiov S., Tanase A., Iordache A., Ambrosie L., Miler A., Azoicai D. From Pleasure to Pathology: Understanding the Neural Basis of Food Addiction in the Context of Obesity. Chirurgia. 2023;118(4):348-357.

468. Soullane S., Israel M., Steiger H., Chadi N., Low N., Dewar R., Ayoub A., Auger N. Association of hospitalization for suicide attempts in adolescent girls with subsequent hospitalization for eating disorders. International Journal of Eating Disorders. 2023;():9.

469. Soyer J., Gabet A., Grave C., Piffaretti C., Verdot C., Salanave B., Deschamps V., Fosse-Edorh S., Carcaillon-Bentata L., Olié V. Need for improvement of cardiovascular health: a clustering method to identify cardiovascular health profiles. From epidemiological surveillance to identification of targeted audiences for prevention campaigns. European Journal of Public Health. 2023;33(4):732-737.

470. Spielmann M., Krolo-Wicovsky F., Tiede A., Krause K., Baumann S., Siewert-Markus U., John U., Freyer-Adam J. Patient motivation and preferences in changing co-occurring health

risk behaviors in general hospital patients. Patient Education and Counseling. 2023;114():6.

471. Spinelli A., Censi L., Mandolini D., Ciardullo S., Salvatore M., Mazzarella G., Nardone P., Grp O. Inequalities in Childhood Nutrition, Physical Activity, Sedentary Behaviour and Obesity in Italy. Nutrients. 2023;15(18):15.

472. Stackpole R., Greene D., Bills E., Egan S. The association between eating disorders and perfectionism in adults: A systematic review and meta-analysis. Eating Behaviors. 2023;50():12.

473. Staller K., Abber S., Murray H. The intersection between eating disorders and gastrointestinal disorders: a narrative review and practical guide. Lancet Gastroenterology & Hepatology. 2023;8(6):565-578.

474. Stanmyre J., Nower L., Malkin M. Problem Gambling and Sexual Minority Individuals: Evaluating Influence of Age and Comorbid Mental Health and Substance Use Problems. Journal of Gambling Studies. 2023;():13.

475. Stice E., Wisting L., Desjardins C., Hood K., Hanes S., Rubino L., Shaw H. Evaluation of a novel eating disorder prevention program for young women with type 1 diabetes: A preliminary randomized trial. Diabetes Research and Clinical Practice. 2023;206():7.

476. Stojic S., Eriks-Hoogland I., Gamba M., Valido E., Minder B., Chatelan A., Karagounis L., Ballesteros M., Díaz C., Brach M., Stoyanov J., Diviani N., Rubinelli S., Perret C., Glisic M. Mapping of Dietary Interventions Beneficial in the Prevention of Secondary Health Conditions in Spinal Cord Injured Population: A Systematic Review. Journal of Nutrition Health & Aging. 2023;27(7):524-541.

477. Stordeur C., Ayrolles A., Trebossen V., Barret S., Baillin F., Poncet-Kalifa H., Meslot C., Clarke J., Bargiacchi A., Peyre H., Delorme R. Early-onset restrictive food intake disorders in children: a latent class analysis. European Child & Adolescent Psychiatry. 2023;():7.

478. Strelnikov K., Debladis J., Salles J., Valette M., Cortadellas J., Tauber M., Barone P. Amygdala hyperactivation relates to eating behaviour: a potential indicator of food addiction in Prader-Willi syndrome. Brain Communications. 2023;5(3):13.

479. Sun M., Dai Y. Late-onset cobalamin C deficiency type in adult with cognitive and behavioral disturbances and significant cortical atrophy and cerebellar damage in the MRI: a case report. Frontiers in Neurology. 2023;14():7.

480. Sun W., Matsuoka T., Imai A., Narumoto J. Relationship between eating problems and the risk of dementia: A retrospective study. Psychogeriatrics. 2023;():8.

481. Sun Y., Ferguson M., Reeves M., Kelly J. Maternal Dietary Patterns and Risk of Postpartum Depression: A Systematic Review. Maternal and Child Health Journal. 2023;27(12):2077-2090.

482. Swaminath S., Sistad R., Simons R., Simons J. The role of thought suppression and negative urgency in eating disorder symptoms and alcohol-related problems among survivors of childhood maltreatment. Clinical Psychologist. 2023;27(3):316-327.

483. Tajaddini A., Fallahi M., Haghshenas H., Nourmohammadi S., Ghahramani L., Shahriarirad R. Primary abdominal cocoon syndrome manifesting with Chilaiditi syndrome and intestinal obstruction: A case report. Clinical Case Reports. 2024;12(1):5.

484. Tamaishi Y., Tanaka H., Hirai T., Hiramatsu S., Takakura S., Magawa S., Tanaka K., Kondo E., Iwamoto T., Ikeda T. Safety and dose-finding trial of tadalafil administered for fetus in labor: A phase I clinical study. Journal of Obstetrics and Gynaecology Research. 2024;():8.

485. Tanaka T., Nagasu S., Furuta T., Gobaru M., Suzuki H., Shimotsuura Y., Akiba J., Nomura M., Fujita F., Kawaguchi T., Miwa K. Case report: A case of fulminant type 1 diabetes mellitus after COVID-19 vaccination during treatment of advanced gastric cancer: pitfall in managing immune-related adverse events. Frontiers in Oncology. 2023;13():6.

486. Tanofsky-Kraff M., Schvey N. Parents of children with high weight and disordered eating: Commentary on Wilksch (2023). International Journal of Eating Disorders. 2023;56(7):1293-1296.

487. Taylor K., Beeken R., Fisher A., Lally P. Did the COVID-19 pandemic impact the dietary intake of individuals living with and beyond breast, prostate, and colorectal cancer and who were most likely to experience change?. Supportive Care in Cancer. 2023;31(10):10.

488. Tayyem R., Zakarneh S., Al-Jayyousi G. Investigating the association between dietary patterns and glycemic control among children and adolescents with T1DM. Open Life Sciences. 2023;18(1):8.

489. Tebbe E., Simone M., Greene M. Challenges of Deep Learning in Cancers. International Journal of Mental Health Nursing. 2023;32(4):1148-1159.

490. Temple N. A Proposed Strategy against Obesity: How Government Policy Can Counter the Obesogenic Environment. Nutrients. 2023;15(13):10.

491. Thompson D., Mirabile Y., Islam N., Callender C., Musaad S., Miranda J., Moreno J., Dave J., Baranowski T. Diet Quality among Pre-Adolescent African American Girls in a Randomized Controlled Obesity Prevention Intervention Trial. Nutrients. 2023;15(12):14.

492. Thompson K., Bauman V., Sunderland K., Thornton J., Schvey N., Moyer R., Sekyere N., Funk W., Pav V., Brydum R., Klein D., Lavender J., Tanofsky-Kraff M. Incidence and prevalence of eating disorders among active duty US military-dependent youth from 2016 to 2021. International Journal of Eating Disorders. 2023;56(10):1973-1982.

493. Thorp J., Gerring Z., Derks E. Machine learning drives genetic discovery for binge eating disorder. Nature Genetics. 2023;55(9):1424-1425.

494. Thriemer K., Commons R., Rajasekhar M., Degaga T., Chand K., Chau N., Assefa A., Naddim M., Pasaribu A., Rahim A., Sutanto I., Hien T., Hailu A., Hasanzai M., Ekawati L., Woyessa A., Teferi T., Waithira N., Taylor W., Ley B., Dondorp A., Baird J., White N., Day N., Price R., Simpson J., Seidlein L. The heterogeneity of symptom reporting across study sites: a secondary analysis of a randomised placebo-controlled multicentre antimalarial trial. Bmc Medical Research Methodology. 2023;23(1):10.

495. Tie B., Xu Y., Cui S., He J. Gay Dating Apps Usage, Body Dissatisfaction, and Disordered Eating in Chinese Young Gay Men. Journal of Homosexuality. 2023;():21.

496. Tokumitsu K., Sugawara N., Adachi N., Kubota Y., Watanabe Y., Miki K., Azekawa T., Edagawa K., Katsumoto E., Hongo S., Goto E., Ueda H., Kato M., Yoshimura R., Nakagawa A., Kikuchi T., Tsuboi T., Watanabe K., Yasui-Furukori N. Real-world predictors of divorce among outpatients with bipolar disorder: sex differences and clinical implications. Annals of General Psychiatry. 2023;22(1):12.

497. Toorani Z., Radhi A., Hassan M., Aloraibi A. A Rare Concurrence of Myelodysplastic Neoplasia and Tetrasomy 8 in a 3-Year-Old Bahraini Male. Cureus Journal of Medical Science. 2023;15(7):10.

498. Torres-McGehee T., Uriegas N., Hauge M., Monsma E., Emerson D., Smith A. Eating Disorder Risk and Pathogenic Behaviors Among Collegiate Student-Athletes. Journal of Athletic Training. 2023;58(10):803-812.

499. Torstveit M., Ackerman K., Constantini N., Holtzman B., Koehler K., Mountjoy M., Sundgot-Borgen J., Melin A. Primary, secondary and tertiary prevention of Relative Energy Deficiency in Sport (REDs): a narrative review by a subgroup of the IOC consensus on REDs. British Journal of Sports Medicine. 2023;57(17):1119-+.

500. Tos M., Grazynska A., Antoniuk S., Siuda J. Impulse Control Disorders in the Polish Population of Patients with Parkinson's Disease. Medicina-Lithuania. 2023;59(8):12.

501. Toumpakari Z., Valerino-Perea S., Willis K., Adams J., White M., Vasiljevic M., Ternent L., Brown J., Kelly M., Bonell C., Cummins S., Majeed A., Anderson S., Robinson T., Araujo-Soares V., Watson J., Soulsby I., Green D., Sniehotta F., Jago R. Exploring views of members of the public and policymakers on the acceptability of population level dietary and active-travel policies: a qualitative study. International Journal of Behavioral Nutrition and Physical Activity. 2023;20(1):14.

502. Trethewey E., Evans S., McIver S., O'Brien J., Lowndes J., Pepin G., O'Shea M. One size may not fit all: A mapping review of yoga-based interventions for the treatment of eating disorders. Complementary Therapies in Clinical Practice. 2023;53():21.

503. Trovini G., Amici E., Bauco P., Matrone M., Lombardozzi G., Giovanetti V., Kotzalidis G., De Filippis S. A comprehensive evaluation of adverse childhood experiences, social-emotional impairments, and neurodevelopmental disorders in cannabis-use disorder: Implications for clinical practice. European Psychiatry. 2023;66(1):9.

504. Tsai H., Huang C., Chen Y., Su W., Chang T., Chen P., Li C., Chang Y., Wang J. Real-World Outcomes of First-Line FOLFIRI Plus Bevacizumab with Irinotecan Dose Escalation versus FOLFOXIRI Plus Bevacizumab in <i>BRAF^{V600E}</i>-Mutant Metastatic Colorectal Cancer: The Preliminary Data from a Single-Center Observational Study. Medicina-Lithuania. 2023;59(12):14.

505. Tsubota-Utsugi M., Satoh M., Watanabe J., Takebayashi J., Oki T., Tatsumi Y., Asayama K., Kikuya M., Murakami T., Hirose T., Metoki H., Hara A., Nomura K., Hozawa A., Tsubono Y., Imai Y., Ohkubo T. Association between an Antioxidant-Rich Japanese Diet and Chronic Kidney Disease: The Ohasama Study. Journal of Atherosclerosis and Thrombosis. 2023;():17.

506. Tsuji D., Nakagaki S., Yonezawa I., Suzuki K., Yokokawa T., Kawasaki Y., Yamaguchi T., Kawaguchi T., Hatori M., Matsumoto T., Sakata Y., Yamamoto K., Nishimura T., Kogure Y., Hayashi T., Osawa M., Itoh K., Watanabe M. A multicenter phase II trial of the triplet antiemetic therapy with palonosetron, aprepitant, and olanzapine for a cisplatin-containing regimen. - PATROL-I. Investigational New Drugs. 2023;():9.

507. Uchida M., Ishida S., Mochizuki E., Ozawa N., Yonemitsu H., Ochiai H., Nakamura H., Kawashiri T., Kato K., Egashira N., Akashi K., Ieiri I. Improvement of Medication Guidance Sheet for Total Body Irradiation/Cyclophosphamide Followed by Allogeneic Hematopoietic Stem Cell Transplantation Based on Real Monitoring Data. Anticancer Research. 2023;43(9):4067-4075.

508. Uher R., Pavlova B., Radua J., Provenzani U., Najafi S., Fortea L., Ortuño M., Nazarova A., Perroud N., Palaniyappan L., Domschke K., Cortese S., Arnold P., Austin J., Vanyukov M., Weissman M., Young A., Hillegers M., Danese A., Nordentoft M., Murray R., Fusar-Poli P. Transdiagnostic risk of mental disorders in offspring of affected parents: a meta-analysis of family high-risk and registry studies. World Psychiatry. 2023;22(3):433-448.

509. Uttam S., Kumar S., Singh S. A Rare Case of Rapunzel Syndrome Presenting with Perforation Peritonitis. Cureus Journal of Medical Science. 2023;15(7):7.

510. Valenta S., Stecchi M., Perazza F., Nuccitelli C., Villanova N., Pironi L., Atti A., Petroni M. Liraglutide 3.0 mg and mental health: can psychiatric symptoms be associated to adherence to therapy? Insights from a clinical audit. Eating and Weight Disorders-Studies on Anorexia Bulimia and Obesity. 2023;28(1):10.

511. Van Dyne A., Washington N., Villodas M., Cronan T. Racial/ethnic differences in anorexia and bulimia diagnoses among US college students. Eating Behaviors. 2023;50():4.

512. Eeden A., Hoeken D., Hendriksen J., Hoek H. Increase in incidence of anorexia nervosa among 10-to 14-year-old girls: A nationwide study in the Netherlands over four decades. International Journal of Eating Disorders. 2023;():9.

513. Hooijdonk K., Simons S., Noorden T., Geurts S., Vink J. Prevalence and clustering of health behaviours and the association with socio-demographics and mental well-being in Dutch university students. Preventive Medicine Reports. 2023;35():10.

514. Van Malderen E., Goossens L., Claes L., Wilderjans T., Kemps E., Verbeken S. Self-regulation profiles in addictive behaviors among adolescents: A transdiagnostic approach. Appetite. 2024;192():13.

515. Tetering E., Muskens J., Deenik J., Pillen S., Cahn W., Rosenstiel I., Oomen M., Rommelse N., Staal W., Klip H. The short and long-term effects of a lifestyle intervention in children with mental illnesses: a randomized controlled trial (Movementss study). Bmc Psychiatry. 2023;23(1):15.

516. Vasiliu O. At the Crossroads between Eating Disorders and Body Dysmorphic Disorders-The Case of Bigorexia Nervosa. Brain Sciences. 2023;13(9):14.

517. Vasiliu O. Current trends and perspectives in the exploration of anorexia athletica-clinical challenges and therapeutic considerations. Frontiers in Nutrition. 2023;10():7.

518. Verde L., Pagano A., Leo M., Vetrani C., Ambretti A., Lucania L., Babudieri S., De Chiara A., Colao A., Corsi M., Muscogiuri G., Barrea L. Diet-Related Risk Factors for Chronic Noncommunicable Diseases in Italian Prisoners: BACI (Benessere All'interno delle Carceri Italiane, Well-Being Inside the Italian Prisons) Project by the Italian Society of Penitentiary Medicine and Public Health (SIMSPe. Società Italiana di Medicina e Sanità Penitenziaria). Current Nutrition Reports. 2023;():12.

519. Via E., Contreras-Rodríguez O. Binge-Eating Precursors in Children and Adolescents: Neurodevelopment, and the Potential Contribution of Ultra-Processed Foods. Nutrients. 2023;15(13):20.

520. Vijayan M., Deshpande K., Anand S., Deshpande P. Risk Amplifiers for Vascular Disease and CKD in South Asians When Intrinsic β-Cell Dysfunction Meets a High-Carbohydrate Diet. Clinical Journal of the American Society of Nephrology. 2023;18(5):681-688.

521. Viroli G., Kalmpourtzidou A., Cena H. Exploring Benefits and Barriers of Plant-Based Diets: Health, Environmental Impact, Food Accessibility and Acceptability. Nutrients. 2023;15(22):15.

522. Volpp K., Berkowitz S., Sharma S., Anderson C., Brewer L., Elkind M., Gardner C., Gervis J., Harrington R., Herrero M., Lichtenstein A., McClellan M., Muse J., Roberto C., Zachariah J., American Heart Assoc Food Is Medicine: A Presidential Advisory From the American Heart Association. Circulation. 2023;148(18):1417-1439.

523. Waite E., Jenkinson E., Kershaw S., Guest E. Psychosocial Interventions for Children and Young People With Visible Differences Resulting From Appearance-Altering Conditions, Injury, or Treatment Effects: An Updated Systematic Review. Journal of Pediatric Psychology. 2023;():12.

524. Wakelin K., Read R., O'Donnell N., Baker M., Satherley R., Stewart R., Jones C. Integrating conversations about disordered eating in children and young people into routine type 1 diabetes care: a practical guide. Practical Diabetes. 2023;40(4):11-17.

525. Wald E., Eickhoff J., Flood G., Heinz M., Liu D., Agrawal A., Morse R., Raney V., Veerapandiyan A., Madan J. Estimate of the incidence of PANDAS and PANS in 3 primary care populations. Frontiers in Pediatrics. 2023;11():8.

526. Wallace I., Toya C., Muñoz M., Meyer J., Busby T., Reynolds A., Martinez J., Thompson T., Miller-Moore M., Harris A., Rios R., Martinez A., Jashashvili T., Ruff C. Effects of the energy balance transition on bone mass and strength. Scientific Reports. 2023;13(1):13.

527. Wang D., He Q., Xia B., Zheng J., Cao W., Su S., Hu F., Li J., Zhang Y., Ren Z., Li X., Wu X., Huang Y., Tang Y., Wei F., Zou H., Jiang H., Huang J., Meng W., Bai M., Yang K., Yuan J., Chrimac A protocol of Chinese expert consensuses for the management of health risk in the general public. Frontiers in Public Health. 2023;11():7.

528. Wang H., Zhang H., Zou Z. Changing profiles of cardiovascular disease and risk factors in China: a secondary analysis for the Global Burden of Disease Study 2019. Chinese Medical Journal. 2023;136(20):2431-2441.

529. Wang M., Yu X., Shi Y., Gao Y. Risk Investigation and Analysis of Risk Factors for Malnutrition in Patients with Advanced Kidney Cancer: A Single-Centre Retrospective Study. Archivos Espanoles De Urologia. 2023;76(5):328-334.

530. Wang Y., Ma W., Mehta R., Nguyen L., Song M., Drew D., Asnicar F., Huttenhower C., Segata N., Wolf J., Spector T., Berry S., Staller K., Chan A. Diet and gut microbial associations

in irritable bowel syndrome according to disease subtype. Gut Microbes. 2023;15(2):13.

531. Wang Z., Chen L., Sun L., Cai F., Yang Q., Hu X., Fu Q., Chen W., Li P., Li W. Prophylactic cranial irradiation for extensive stage small cell lung cancer: a meta-analysis of randomized controlled trials. Frontiers in Oncology. 2023;13():10.

532. Wani M., Chakole S., Agrawal S., Gupta A., Chavada J., Pathade A., Yelne S. Unveiling Skin Manifestations: Exploring Cutaneous Signs of Malnutrition in Eating Disorders. Cureus Journal of Medical Science. 2023;15(9):15.

533. Wardill H., Ferreira A., Kumar H., Bateman E., Cross C., Bowen J., Havinga R., Harmsen H., Knol J., Dorresteijn B., Dijk M., Bergenhenegouwen J., Tissing W. Whey-based diet containing medium chain triglycerides modulates the gut microbiota and protects the intestinal mucosa from chemotherapy while maintaining therapy efficacy. Cell Death & Disease. 2023;14(5):11.

534. Weckström T., Elovainio M., Pulkki-Råback L., Suokas K., Komulainen K., Mullola S., Böckerman P., Hakulinen C. School achievement in adolescence and the risk of mental disorders in early adulthood: a Finnish nationwide register study. Molecular Psychiatry. 2023;28(7):3104-3110.

535. Welling M., Kleinendorst L., Haelst M., Akker E. The Narrative of a Patient with Leptin Receptor Deficiency: Personalized Medicine for a Rare Genetic Obesity Disorder. Obesity Facts. 2023;16(5):514-518.

536. Wheaton N., Alston E., Versace V., Field M., Shee A., Jacobs J., Backholer K., Allender S., Nichols M., Needham C., Bolton K., Blake M., Stewart F., Close E., Alston L., Busetto L. Diet-Related Disease Prevention in a Rural Australian Setting: Understanding Barriers, Enablers, and the Role of Rural Health Services in Supporting Changes in Local Rural Food Environments. Nutrients. 2023;15(23):15.

537. Wilczynska D., Hryniewicz A., Jaroch-Lidzbarska M., Hryniewicz K., Lipowski M. Gender and Work Experience as Moderators of Relations between Management Level, Physical Activity, Eating Attitudes, and Social Skills of Managers during the COVID-19 Pandemic. Nutrients. 2023;15(19):24.

538. Willson E., Buono S., Kerr G., Stirling A. Maltreatment experiences and mental health indicators among elite athletes. Psychology of Sport and Exercise. 2023;69():9.

539. Winkens L., Braver N., Mackenbach J., Visser M., De Vet E. Can mindful eating buffer against the influence of neighbourhood fast food exposure on unhealthy food intake?. Health & Place. 2023;83():9.

540. Winter H., Rolnik D., Mol B., Torkel S., Alesi S., Mousa A., Habibi N., Silva T., Cheung T., Tay C., Quinteros A., Grieger J., Moran L. Can Dietary Patterns Impact Fertility Outcomes? A Systematic Review and Meta-Analysis. Nutrients. 2023;15(11):20.

541. Wójcik M., Alvarez-Pitti J., Koziol-Kozakowska A., Brzezinski M., Gabbianelli R., Herceg-Cavrak V., Wühl E., Lucas I., Radovanovic D., Melk A., Lopez-Valcarcel B., Fernández-Aranda F., Mazur A., Lurbe E., Borghi C., Drozdz D. Psychosocial and environmental risk factors of obesity and hypertension in children and adolescents-a literature overview. Frontiers in Cardiovascular Medicine. 2023;10():14.

542. Wolff B., Franco V., Magiati I., Pestell C., Glasson E. Psychosocial and neurocognitive correlates of suicidal thoughts and behaviours amongst siblings of persons with and without neurodevelopmental conditions. Research in Developmental Disabilities. 2023;139():18.

543. Wong A., Aslanidou A., Malbec M., Pittig A., Wieser M., Andreatta M. A Systematic Review of the Inter-individual Differences in Avoidance Learning. Collabra-Psychology. 2023;9(1):33.

544. Woolf B., Cronjé H., Zagkos L., Burgess S., Gill D., Larsson S. Appraising the causal relationship between plasma caffeine levels and neuropsychiatric disorders through Mendelian randomization. Bmc Medicine. 2023;21(1):8.

545. Wu M., Li S., Lv Y., Liu K., Wang Y., Cui Z., Wang X., Meng H. Associations between the inflammatory potential of diets with adherence to plant-based dietary patterns and the risk of new-onset cardiometabolic diseases in Chinese adults: findings from a nation-wide prospective cohort study. Food & Function. 2023;14(19):9018-9034.

546. Wu R., Guang Z., Wang Y., Xue B., Zhang A., Dawa Y., Guo C., Tong X., Wang S., Lu C. Eating disorders symptoms and depressive symptoms in Chinese Tibetan University

students: a network analysis. Bmc Psychiatry. 2023;23(1):12.

547. Wu S., Wang J., Xu Y., Zhang Z., Jin X., Liang Y., Ge Y., Zhan H., Peng L., Luo D., Li M., Bi W., Guan Q., He Z. Energy deficiency promotes rhythmic foraging behavior by activating neurons in paraventricular hypothalamic nucleus. Frontiers in Nutrition. 2023;10():12.

548. Wu S., Ou C., Lee M., Hsin I., Kang Y., Jan M., Ko J. Polysaccharide of Ganoderma lucidum Ameliorates Cachectic Myopathy Induced by the Combination Cisplatin plus Docetaxel in Mice. Microbiology Spectrum. 2023;11(3):12.

549. Wu Y., Hou C., Weng S., Lee J. Lifestyle and Diet as Risk Factors for Urinary Stone Formation: A Study in a Taiwanese Population. Medicina-Lithuania. 2023;59(11):8.

550. Xia X., He S., Zhang X., Wang D., He Q., Xiao Q., Yang Y. The causality between gut microbiome and anorexia nervosa: a Mendelian randomization analysis. Frontiers in Microbiology. 2023;14():10.

551. Xiang W., Liu A., Xu C., Zhang D., Li W., Ni Y. Bile Acid Alterations Characterize the Early Onset and Progression of Nonalcoholic Fatty Liver Disease in Young Mice Fed with High Fat and Fructose Diet. Molecular Nutrition & Food Research. 2023;67(11):9.

552. Xiao Q., Huang W., Wu Q., Xu H., Zhang Y., Yang J., Bian S., Tan H., Nie S. The effects of pectin on the gut microbiota and serum metabolites in mice fed with a high fat diet and exposed to low-dose antibiotics. Food & Function. 2023;14(10):4752-4762.

553. Xu W., Liu J., Zhang J., Lu J., Guo J. Tumor microenvironment crosstalk between tumors and the nervous system in pancreatic cancer: Molecular mechanisms and clinical perspectives. Biochimica Et Biophysica Acta-Reviews on Cancer. 2024;1879(1):9.

554. Yang C., Yang Y., Zhang F., Wang D., Bian Q., Zhou M., Zhou M., Yang X. Congenital leukemia: A case report and review of literature. World Journal of Clinical Cases. 2023;11(29):7227-+.

555. Yang C., Li X., Ma X. Idiopathic Isolated Adrenocorticotropic Hormone Deficiency: A Single-Center Retrospective Study. Experimental and Clinical Endocrinology & Diabetes. 2023;():9.

556. Yang H., Zhou X., Xie L., Sun J. The effect of emotion regulation on emotional eating among undergraduate students in China: The chain mediating role of impulsivity and depressive symptoms. Plos One. 2023;18(6):16.

557. Yang T., Yan C., Han T., Huai K., Hu J., Wu Y., Jin Y., Jiang J., Xue X. Efficacy and safety of zonisamide as the first additional treatment in Chinese patients with focal or secondary bilateral tonic-clonic seizures: An observational ,prospective study. Heliyon. 2023;9(7):9.

558. Yang W., Niu G., Shi X., Song K., Zhang Y., Yuan Z. Negative family body talk and negative emotional eating among Chinese adolescent girls and young adult women: The role of body dissatisfaction and feminism consciousness. Appetite. 2023;188():9.

559. Yilmaz B., Sirbu A., Basar H., Goksen G., Chabi I., Kumagai H., Ozogul F. Potential roles of cereal bioactive compounds in the prevention and treatment of type 2 diabetes: A review of the current knowledge. Critical Reviews in Food Science and Nutrition. 2023;():18.

560. Yokoyama N., Kinoshita R., Ohta H., Okada K., Shimbo G., Sasaoka K., Nagata N., Sasaki N., Morishita K., Nakamura K., Kagawa Y., Takiguchi M. Successful treatment of sclerosing encapsulating peritonitis in a cat using bioresorbable hyaluronate-carboxymethylcellulose membrane after surgical adhesiolysis and long-term prednisolone. Journal of Feline Medicine and Surgery Open Reports. 2023;9(2):7.

561. Yoon C., Mai D., Kinariwala K., Ledoux T., Betts R., Johnston C. Sex and ethnic/racial differences in disordered eating behaviors and intuitive eating among college student. Frontiers in Psychology. 2023;14():12.

562. You Z., Shan L., Cheng S., Xia Y., Zhao Y., Zhang H., Zhao Z. Dietary intake patterns during pregnancy and excessive gestational weight gain: a systematic review and meta-analysis. Food & Function. 2023;14(13):5910-5920.

563. Yuan Y., Tian W., Jin Z., Wang L., Zhu S. Cholecystitis in older patients following hip fracture: a case series and literature review. Bmc Geriatrics. 2023;23(1):10.

564. Yuki M., Taira H., Inden T. Development of acute pancreatitis after oral administering a praziquantel, pyrantel pamoate, and febantel combination in a dog: A case report. Heliyon. 2023;9(6):5.

565. Yüksel S., Gencer F., Alptekin F., Saglam N. Disordered Eating in Young Women with Polycystic Ovary Syndrome. Reproductive Sciences. 2023;():8.

566. Zhang B., Qing J., Yan Z., Shi Y., Wang Z., Chen J., Li J., Li S., Wu W., Hu X., Li Y., Zhang X., Wu L., Zhu S., Yan Z., Wang Y., Guo X., Yu L., Li X. Investigation and analysis of porcine epidemic diarrhea cases and evaluation of different immunization strategies in the large-scale swine farming system. Porcine Health Management. 2023;9(1):9.

567. Zhang C., Zaleski G., Kailley J., Teng K., English M., Riminchan A., Robillard J. Debate: Social media content moderation may do more harm than good for youth mental health. Child and Adolescent Mental Health. 2023;():3.

568. Zhang C., Yu Z., Gao S., Ma M., Gou L., Wang C., Wang L., Li J., Zhong L., Zhou Y., Wang W., Song H. Efficacy and safety of thalidomide in children with monogenic autoinflammatory diseases: a single-center, real-world-evidence study. Pediatric Rheumatology. 2023;21(1):10.

569. Zhang F., Wu H., Shi H., Yu Z., Zhuang C. Sarcopenia and malignancies: epidemiology, clinical classification and implications. Ageing Research Reviews. 2023;91():11.

570. Zhang L., Wang Y., Sun Y., Zhang X. Intermittent Fasting and Physical Exercise for Preventing Metabolic Disorders through Interaction with Gut Microbiota: A Review. Nutrients. 2023;15(10):13.

571. Zhang M., Chen Y., Wu W., Jin F., Li Y., Long J., Luo X., Gong X., Chen X., Liu L., Tang H., Wang Z. A prospective phase II randomized study of docetaxel combined with lobaplatin versus TPF regimen induction chemotherapy followed by concurrent chemoradiotherapy for locally advanced head and neck squamous cell carcinoma. Journal of Cancer Research and Clinical Oncology. 2023;():11.

572. Zhang M., Guo Q., Zhang T., Fu M., Bi H., Zhang J., Zou K. Efficacy and safety of Aprepitant-containing triple therapy for the prevention and treatment of chemotherapy-induced nausea and vomiting: A meta-analysis. Medicine. 2023;102(47):10.

573. Zhang W., Peng Y., Kang X., Wang C., Chen F., He Y., Li W. Healthy and Unhealthy Plant-Based Diets and Glioma in the Chinese Population. Brain Sciences. 2023;13(10):12.

574. Zhao J., Fan B., Huang J., Cowling B., Yeung Slra, Baccarelli A., Leung G., Schooling C., Lee S. Environment- and epigenome-wide association study of obesity in 'Children of 1997' birth cohort. Elife. 2023;12():22.

575. Zhao J., Huang H., Gong H., Zhao Q., Wu H. Ovarian cyst torsion in Prader-Willi Syndrome. Bmc Pediatrics. 2023;23(1):7.

576. Zhao Y., Li Y., Wang W., Song Z., Zhuang Z., Li D., Qi L., Huang T. Low-carbohydrate diets, low-fat diets, and mortality in middle-aged and older people: A prospective cohort study. Journal of Internal Medicine. 2023;294(2):203-215.

577. Zhou P., Li Y., Lau P., Yan L., Song H., Shi T. Effectiveness of parent-based electronic health<i> (eHealth)</i> intervention on physical activity, dietary behaviors, and sleep in preschoolers: A systematic review. Journal of Exercise Science & Fitness. 2024;22(1):1-13.

578. Zhu R., Huttunen-Lenz M., Stratton G., Handjieva-Darlenska T., Handjiev S., Sundvall J., Silvestre M., Jalo E., Pietiläinen K., Adam T., Drummen M., Simpson E., Taylor M., Poppitt S., Navas-Carretero S., Martinez J., Schlicht W., Fogelholm M., Brand-Miller J., Raben A. Associations of obesity phenotypes with weight change, cardiometabolic benefits, and type 2 diabetes incidence during a lifestyle intervention: results from the PREVIEW study. International Journal of Obesity. 2023;47(9):833-840.

579. Zielinski-Gussen I., Herpertz-Dahlmann B., Dahmen B. Involuntary Treatment for Child and Adolescent Anorexia Nervosa-A Narrative Review and Possible Advances to Move Away from Coercion. Healthcare. 2023;11(24):15.

580. Zuo H., Li M. Ankylosing spondylitis and psychiatric disorders in European population: a Mendelian randomization study. Frontiers in Immunology. 2023;14():11.

581. Levallius Johanna, Monell Elin, Birgegard Andreas, Clinton David, Forsen Mantilla Emma Binge Eating and Addictive-Like Behaviours in Males and Females. PSYCHOLOGICAL REPORTS. 2022;125(1):148-166.

582. Holland Lauren, Brown Tiffany, Keel Pamela Defining features of unhealthy exercise associated with disordered eating and eating disorder diagnoses. PSYCHOLOGY OF SPORT AND EXERCISE. 2014;15(1):116-123.

583. Levinson Cheri, Trombley Christopher, Brosof Leigh, Williams Brenna, Hunt Rowan Binge Eating, Purging, and Restriction Symptoms: Increasing Accuracy of Prediction Using Machine Learning. BEHAVIOR THERAPY. 2023;54(2):247-259.

584. Goldschmidt Andrea, Wall Melanie, Loth Katie, Neumark-Sztainer Dianne Risk Factors for Disordered Eating in Overweight Adolescents and Young Adults. JOURNAL OF PEDIATRIC PSYCHOLOGY. 2015;40(10):1048-1055.

585. Stice Eric, Marti C., Rohde Paul Prevalence, Incidence, Impairment, and Course of the Proposed DSM-5 Eating Disorder Diagnoses in an 8-Year Prospective Community Study of Young Women. JOURNAL OF ABNORMAL PSYCHOLOGY. 2013;122(2):445-457.

586. Saunders R Compulsive eating and gastric bypass surgery: What does hunger have to do with it?. OBESITY SURGERY. 2001;11(6):757-761.

587. Chen Ximei, Gao Xiao, Qin Jingmin, Wang Chuan, Xiao Mingyue, Tian Yun, Luo Yi-jun, Qiu Jiang, Feng Tingyong, He Qinghua, Lei Xu, Chen Hong Resting-state functional network connectivity underlying eating disorder symptoms in healthy young adults. NEUROIMAGE-CLINICAL. 2021;30():.

588. Fear JL, Bulik CM, Sullivan PF The prevalence of disordered eating behaviours and attitudes in adolescent girls. NEW ZEALAND JOURNAL OF PSYCHOLOGY. 1996;25(1):7-12.

589. Kontic Olga, Vasiljevic Nadja, Jorga Jagoda, Jasovic-Gasic Miroslava, Lakic Aneta, Arsic Aleksandra Presence of Different Forms of Compensatory Behaviours among Eating Disordered Patients. SRPSKI ARHIV ZA CELOKUPNO LEKARSTVO. 2010;138(5-6):328-332.

590. Mason Tyler, Lewis Robin Clustered Patterns of Behavioral and Health-Related Variables Among Young Lesbian Women. BEHAVIOR THERAPY. 2019;50(4):683-695.

591. Herpertz-Dahlmann B., Hagenah U. Eating disorders in childhood and adolescence. MONATSSCHRIFT KINDERHEILKUNDE. 2015;163(7):688+.

592. Butler Rachel, Heimberg Richard Exposure therapy for eating disorders: A systematic review. CLINICAL PSYCHOLOGY REVIEW. 2020;78():.

593. Larsen Pernille, Strandberg-Larsen Katrine, Micali Nadia, Andersen Anne-Marie Parental and Child Characteristics Related to Early-Onset Disordered Eating: A Systematic Review. HARVARD REVIEW OF PSYCHIATRY. 2015;23(6):395-412.

594. Allison Kelly, Wu Jingwei, Spitzer Jacqueline, McCuen-Wurst Courtney, Ashare Rebecca, Tewksbury Colleen, LaGrotte Caitlin, Wadden Thomas, Williams Noel, Sarwer David Changes in Eating Behaviors and Their Relation to Weight Change 6 and 12 Months After Bariatric Surgery. OBESITY SURGERY. 2023;33(3):733-742.

595. Escriva-Martinez Tamara, Herrero Rocio, Molinari Guadalupe, Rodriguez-Arias Marta, Verdejo-Garcia Antonio, Banos Rosa Binge Eating and Binge Drinking: A Two-Way Road? An Integrative Review. CURRENT PHARMACEUTICAL DESIGN. 2020;26(20):2402-2415.

596. Monteleone P Eating disorders and obsessive-compulsive disorder: phenomenological and biological similarities. CURRENT OPINION IN PSYCHIATRY. 2000;13(6):665-671.

597. McDonald Caity, Rossell Susan, Phillipou Andrea The comorbidity of eating disorders in bipolar disorder and associated clinical correlates characterised by emotion dysregulation and impulsivity: A systematic review. JOURNAL OF AFFECTIVE DISORDERS. 2019;259():228-243.

598. Rohde Paul, Arigo Danielle, Shaw Heather, Stice Eric Relation of Self-Weighing to Future Weight Gain and Onset of Disordered Eating Symptoms. JOURNAL OF CONSULTING AND CLINICAL PSYCHOLOGY. 2018;86(8):677-687.

599. Bristow Claire, Allen Kelly-Ann, Simmonds Janette, Snell Tristan, McLean Louise Anti-obesity public health advertisements increase risk factors for the development of eating disorders. HEALTH PROMOTION INTERNATIONAL. 2022;37(2):.

600. Pawar Pratiksha, Thornton Laura, Flatt Rachael, Sanzari Christina, Carrino Emily, Tregarthen Jenna, Argue Stuart, Bulik Cynthia, Watson Hunna Binge-eating disorder with and without lifetime anorexia nervosa: A comparison of sociodemographic and clinical features. INTERNATIONAL JOURNAL OF EATING DISORDERS. 2022;():.

601. Gori Alessio, Topino Eleonora, Griffiths Mark Protective and Risk Factors in Exercise Addiction: A Series of Moderated Mediation Analyses. INTERNATIONAL JOURNAL OF ENVIRONMENTAL RESEARCH AND PUBLIC HEALTH. 2021;18(18):.

602. Lyons MA The phenomenon of compulsive overeating in a selected group of professional women. JOURNAL OF ADVANCED NURSING. 1998;27(6):1158-1164.

603. McClure Zoe, Messer Mariel, Anderson Cleo, Liu Claudia, Linardon Jake Which dimensions of emotion dysregulation predict the onset and persistence of eating disorder behaviours? A prospective study. JOURNAL OF AFFECTIVE DISORDERS. 2022;310():123-128.

604. Neumark-Sztainer Dianne, Wall Melanie, Story Mary, Sherwood Nancy Five-Year Longitudinal Predictive Factors for Disordered Eating in a Population-Based Sample of Overweight Adolescents: Implications for Prevention and Treatment. INTERNATIONAL JOURNAL OF EATING DISORDERS. 2009;42(7):664-672.

605. Eisenberg Marla, Berge Jerica, Neumark-Sztainer Dianne Dieting and Encouragement to Diet by Significant Others: Associations With Disordered Eating in Young Adults. AMERICAN JOURNAL OF HEALTH PROMOTION. 2013;27(6):370-377.

606. Treasure Janet, Claudino Angelica, Zucker Nancy Eating disorders. LANCET. 2010;375(9714):583-593.

607. Veeraraghavan Vishnupriya Obesogenic Behavior and Binge Eating Disorder in an Elderly Female with Schizophrenia. JOURNAL OF OBESITY \& METABOLIC SYNDROME. 2021;30(2):184-187.

608. Fernandez-Aranda Fernando, Poyastro Pinheiro Andrea, Thornton Laura, Berrettini Wade, Crow Scott, Fichter Manfred, Halmi Katherine, Kaplan Allan, Keel Pamela, Mitchell James, Rotondo Alessandro, Strober Michael, Woodside D., Kaye Walter, Bulik Cynthia Impulse control disorders in women with eating disorders. PSYCHIATRY RESEARCH. 2008;157(1-3):147-157.

609. Stice Eric, Gau Jeff, Rohde Paul, Shaw Heather Risk Factors That Predict Future Onset of Each DSM-5 Eating Disorder: Predictive Specificity in High-Risk Adolescent Females. JOURNAL OF ABNORMAL PSYCHOLOGY. 2017;126(1):38-51.

610. Neumark-Sztainer Dianne, Wall Melanie, Haines Jess, Story Mary, Sherwood Nancy, Berg Patricia Shared risk and protective factors for overweight and disordered eating in adolescents. AMERICAN JOURNAL OF PREVENTIVE MEDICINE. 2007;33(5):359-369.

611. Luz Felipe, Swinbourne Jessica, Sainsbury Amanda, Touyz Stephen, Palavras Marly, Claudino Angelica, Hay Phillipa HAPIFED: a Healthy APproach to weight management and Food in Eating Disorders: a case series and manual development. JOURNAL OF EATING DISORDERS. 2017;5():.

612. Cattaneo Camilla, Mambrini Sara, Gilardini Luisa, Scacchi Massimo, Castelnuovo Gianluca, Pagliarini Ella, Bertoli Simona The phenomenon of abnormal eating and taste perception: What's the link in subjects with obesity and eating disorders?. FOOD QUALITY AND PREFERENCE. 2023;104():.

613. Villagomez L, Cortes J, Barrera E, Saucedo D, Alcocer L Obesity comorbidity with eating disorders. REVISTA DE INVESTIGACION CLINICA-CLINICAL AND TRANSLATIONAL INVESTIGATION. 2003;55(5):535-545.

614. Lydecker Janet, Grilo Carlos Food insecurity and bulimia nervosa in the United States. INTERNATIONAL JOURNAL OF EATING DISORDERS. 2019;52(6):735-739.

615. Stabouli Stella, Erdine Serap, Suurorg Lagle, Jankauskiene Augustina, Lurbe Empar Obesity and Eating Disorders in Children and Adolescents: The Bidirectional Link. NUTRIENTS. 2021;13(12):.

616. Haynos Ann, Roberto Christina The effects of restaurant menu calorie labeling on hypothetical meal choices of females with disordered eating. INTERNATIONAL JOURNAL OF EATING DISORDERS. 2017;50(3, SI):275-283.

617. Ma Ruofan, Mikhail Megan, Fowler Natasha, Culbert Kristen, Klump Kelly The Role of Puberty and Ovarian Hormones in the Genetic Diathesis of Eating Disorders in Females. CHILD AND ADOLESCENT PSYCHIATRIC CLINICS OF NORTH AMERICA. 2019;28(4):617+.

618. Goldschmidt Andrea, Aspen Vandana, Sinton Meghan, Tanofsky-Kraff Marian, Wilfley Denise Disordered eating attitudes and behaviors in overweight youth. OBESITY. 2008;16(2):257-264.

619. Ivezaj Valentina, Carr Meagan, Brode Cassie, Devlin Michael, Heinberg Leslie, Kalarchian Melissa, Sysko Robyn, Williams-Kerver Gail, Mitchell James Disordered eating following bariatric surgery: a review of measurement and conceptual considerations. SURGERY FOR OBESITY AND RELATED DISEASES. 2021;17(8):1510-1520.

620. Goldschmidt Andrea, Le Grange Daniel, Powers Pauline, Crow Scott, Hill Laura, Peterson Carol, Crosby Ross, Mitchell Jim Eating Disorder Symptomatology in Normal-Weight vs. Obese Individuals With Binge Eating Disorder. OBESITY. 2011;19(7):1515-1518.

621. Hooper Laura, Mason Susan, Telke Susan, Larson Nicole, Neumark-Sztainer Dianne Experiencing Household Food Insecurity During Adolescence Predicts Disordered Eating and Elevated Body Mass Index 8 Years Later. JOURNAL OF ADOLESCENT HEALTH. 2022;70(5):788-795.

622. Goodwin Huw, Haycraft Emma, Meyer Caroline Disordered Eating, Compulsive Exercise, and Sport Participation in a UK Adolescent Sample. EUROPEAN EATING DISORDERS REVIEW. 2016;24(4):304-309.

623. Eiber R, Friedman S Relationships between eating disorders and sleep disturbances. ENCEPHALE-REVUE DE PSYCHIATRIE CLINIQUE BIOLOGIQUE ET THERAPEUTIQUE. 2001;27(5):429-434.

624. Smith Fiona, Latchford Gary, Hall Richard, Dickson Robert Do chronic medical conditions increase the risk of eating disorder? A cross-sectional investigation of eating pathology in adolescent females with scoliosis and diabetes. JOURNAL OF ADOLESCENT HEALTH. 2008;42(1):58-63.

625. Neumark-Sztainer D, Wall M, Story M, Fulkerson JA Are family meal patterns associated with disordered eating behaviors among adolescents?. JOURNAL OF ADOLESCENT HEALTH. 2004;35(5):350-359.

626. Kremer Michael, Kremer Kristen, Kremer Theodore School health class associated with reduced odds of eating disorder symptoms in American adolescents. INTERNATIONAL JOURNAL OF EATING DISORDERS. 2020;53(3):383-390.

627. Farstad Sarah, McGeown Laura, Ranson Kristin Eating disorders and personality, 2004-2016: A systematic review and meta-analysis. CLINICAL PSYCHOLOGY REVIEW. 2016;46():91-105.

628. Stunkard AJ, Allison KC Two forms of disordered eating in obesity: binge eating and night eating. INTERNATIONAL JOURNAL OF OBESITY. 2003;27(1):1-12.

629. Mussell MP, Mitchell JE, deZwaan M, Crosby RD, Seim HC, Crow SJ Clinical characteristics associated with binge eating in obese females: A descriptive study. INTERNATIONAL JOURNAL OF OBESITY. 1996;20(4):324-331.

630. Purkiewicz Aleksandra, Kamelska-Sadowska Anna, Ciborska Joanna, Mikulska Julia, Pietrzak-Fiecko Renata Risk Factors for Eating Disorders and Perception of Body in Young Adults Associated with Sex. NUTRIENTS. 2021;13(8):.

631. Becker Kendra, Plessow Franziska, Coniglio Kathryn, Tabri Nassim, Franko Debra, Zayas Lazaro, Germine Laura, Thomas Jennifer, Eddy Kamryn Global/local processing style: Explaining the relationship between trait anxiety and binge eating. INTERNATIONAL JOURNAL OF EATING DISORDERS. 2017;50(11):1264-1272.

632. Neumark-Sztainer Dianne, Eisenberg Marla, Fulkerson Jayne, Story Mary, Larson Nicole Family meals and disordered eating in adolescents - Longitudinal findings from project EAT. ARCHIVES OF PEDIATRICS \& ADOLESCENT MEDICINE. 2008;162(1):17-22.

633. Quick Virginia, Byrd-Bredbenner Carol, Neumark-Sztainers Dianne Chronic Illness and Disordered Eating: A Discussion of the Literature. ADVANCES IN NUTRITION. 2013;4(3):277-286.

634. Tanofsky-Kraff M, Yanovski SZ Eating disorder or disordered eating? Non-normative eating patterns in obese individuals. OBESITY RESEARCH. 2004;12(9):1361-1366.

635. Langlet Billy, Nyberg Maria, Wendin Karin, Zandian Modjtaba The clinicians' view of food-related obstacles for treating eating disorders: A qualitative study. FOOD \& NUTRITION RESEARCH. 2023;67():.

636. Kolar David, Mebarak Moises An update on the epidemiology of eating disorders in Latin America: current findings and future challenges. CURRENT OPINION IN PSYCHIATRY. 2022;35(6):385-389.

637. Berkman Nancy, Lohr Kathleen, Bulik Cynthia Outcomes of eating disorders: A systematic review of the literature. INTERNATIONAL JOURNAL OF EATING DISORDERS. 2007;40(4):293-309.

638. Fitzsimmons-Craft Ellen, Ciao Anna, Accurso Erin A naturalistic examination of social comparisons and disordered eating thoughts, urges, and behaviors in college women. INTERNATIONAL JOURNAL OF EATING DISORDERS. 2016;49(2):143-152.

639. Fischer Sophia, Munsch Simone Self-Regulation in Eating Disorders and Obesity - Implications for the Treatment. VERHALTENSTHERAPIE. 2012;22(3):158-164.

640. Hilbert Anja, Czaja Julia Binge eating and obesity in children. PSYCHOTHERAPIE PSYCHOSOMATIK MEDIZINISCHE PSYCHOLOGIE. 2007;57(11):413-419.

641. Ptacek Radek, Stefano George, Weissenberger Simon, Akotia Devang, Raboch Jiri, Papezova Hana, Domkarova Lucie, Stepankova Tereza, Goetz Michal Attention deficit hyperactivity disorder and disordered eating behaviors: links, risks, and challenges faced. NEUROPSYCHIATRIC DISEASE AND TREATMENT. 2016;12():571-579.

642. CRAIGHEAD LW, ALLEN HN APPETITE AWARENESS TRAINING - A COGNITIVE-BEHAVIORAL INTERVENTION FOR BINGE-EATING. COGNITIVE AND BEHAVIORAL PRACTICE. 1995;2(2):249-270.

643. Bulik CM Anxiety disorders and eating disorders: A review of their relationship. NEW ZEALAND JOURNAL OF PSYCHOLOGY. 1995;24(2):51-62.

644. Jacobi C, Hayward C, Zwaan M, Kraemer HC, Agras WS Coming to terms with risk factors for eating disorders: Application of risk terminology and suggestions for a general taxonomy. PSYCHOLOGICAL BULLETIN. 2004;130(1):19-65.

645. Bulik CM, Sullivan PF, Carter FA, Joyce PR Initial manifestations of disordered eating behavior: Dieting versus binging. INTERNATIONAL JOURNAL OF EATING DISORDERS. 1997;22(2):195-201.

646. Combs Jessica, Pearson Carolyn, Smith Gregory A Risk Model for Preadolescent Disordered Eating. INTERNATIONAL JOURNAL OF EATING DISORDERS. 2011;44(7):596-604.

647. Solmi Francesca, Bulik Cynthia, De Stavola Bianca, Dalman Christina, Khandaker Golam, Lewis Glyn Longitudinal associations between circulating interleukin-6 and C-reactive protein in childhood, and eating disorders and disordered eating in adolescence.

BRAIN BEHAVIOR AND IMMUNITY. 2020;89():491-500.

648. Sampedro-Piquero Patricia, Zancada-Menendez Clara, Bernabeu-Brotons Elena, Moreno-Fernandez Roman The Relationship between Binge Drinking and Binge Eating in Adolescence and Youth: A Systematic Review and Meta-Analysis. INTERNATIONAL JOURNAL OF ENVIRONMENTAL RESEARCH AND PUBLIC HEALTH. 2023;20(1):.

649. Krabbenborg Manon, Danner Unna, Larsen Junilla, Veer Nienke, Elburg Annemarie, Ridder Denise, Evers Catharine, Stice Eric, Engels Rutger The Eating Disorder Diagnostic Scale: Psychometric Features Within a Clinical Population and a Cut-off Point to Differentiate Clinical Patients from Healthy Controls. EUROPEAN EATING DISORDERS REVIEW. 2012;20(4):315-320.

650. Seamoore D., Buckroyd J., Stott D. Changes in eating behaviour following group therapy for women who binge eat: a pilot study. JOURNAL OF PSYCHIATRIC AND MENTAL HEALTH NURSING. 2006;13(3):337-346.

651. Flaudias Valentin, Iceta Sylvain, Zerhouni Oulmann, Rodgers Rachel, Billieux Joel, Llorca Pierre-Michel, Boudesseul Jordane, De Chazeron Ingrid, Romo Lucia, Maurage Pierre, Samalin Ludovic, Begue Laurent, Naassila Mickael, Brousse Georges, Guillaume Sebastien COVID-19 pandemic lockdown and problematic eating behaviors in a student population. JOURNAL OF BEHAVIORAL ADDICTIONS. 2020;9(3):826-835.

652. Lydecker Janet, Grilo Carlos Fathers and mothers with eating-disorder psychopathology: Associations with child eating-disorder behaviors. JOURNAL OF PSYCHOSOMATIC RESEARCH. 2016;86():63-69.

653. Hagan Kelsey, Forbush Kelsie, Chen Po-Yi Is Dietary Restraint a Unitary or Multi-Faceted Construct?. PSYCHOLOGICAL ASSESSMENT. 2017;29(10):1249-1260.

654. Henry Beverly, Ozier Amy Position of the American Dietetic Association: Nutrition intervention in the treatment of anorexia nervosa, bulimia nervosa, and other eating disorders. JOURNAL OF THE AMERICAN DIETETIC ASSOCIATION. 2006;106(12):2073-2082.

655. Brode Cassie, Mitchell James Problematic Eating Behaviors and Eating Disorders Associated with Bariatric Surgery. PSYCHIATRIC CLINICS OF NORTH AMERICA. 2019;42(2,

2):287+.

656. Brosof Leigh, Levinson Cheri Social appearance anxiety and dietary restraint as mediators between perfectionism and binge eating: A six month three wave longitudinal study. APPETITE. 2017;108():335-342.

657. Haiman C, Devlin MJ Binge eating before the onset of dieting: A distinct subgroup of bulimia nervosa?. INTERNATIONAL JOURNAL OF EATING DISORDERS. 1999;25(2):151-157.

658. Allen Karina, Byrne Susan, Crosby Ross Distinguishing Between Risk Factors for Bulimia Nervosa, Binge Eating Disorder, and Purging Disorder. JOURNAL OF YOUTH AND ADOLESCENCE. 2015;44(8, SI):1580-1591.

659. Hughes Elizabeth, Kerr Jessica, Patton George, Sawyer Susan, Wake Melissa, Le Grange Daniel, Azzopardi Peter Eating disorder symptoms across the weight spectrum in Australian adolescents. INTERNATIONAL JOURNAL OF EATING DISORDERS. 2019;52(8):885-894.

660. Mason Susan, Frazier Patricia, Austin S., Harlow Bernard, Jackson Benita, Raymond Nancy, Rich-Edwards Janet Posttraumatic Stress Disorder Symptoms and Problematic Overeating Behaviors in Young Men and Women. ANNALS OF BEHAVIORAL MEDICINE. 2017;51(6):822-832.

661. Cleland Lana, Kennedy Hannah, Pettie Michaela, Kennedy Martin, Bulik Cynthia, Jordan Jennifer Eating disorders, disordered eating, and body image research in New Zealand: a scoping review. JOURNAL OF EATING DISORDERS. 2023;11(1):.

662. Davidsen Annika, Hoyt William, Poulsen Stig, Waaddegaard Mette, Lau Marianne Eating disorder severity and functional impairment: moderating effects of illness duration in a clinical sample. EATING AND WEIGHT DISORDERS-STUDIES ON ANOREXIA BULIMIA AND OBESITY. 2017;22(3):499-507.

663. Rush Christina, Curry John, Looney John Alcohol expectancies and drinking behaviors among college students with disordered eating. JOURNAL OF AMERICAN COLLEGE HEALTH. 2016;64(3):195-204.

664. Hagan MM, Wauford PK, Chandler PC, Jarrett LA, Rybak RJ, Blackburn K A new animal model of binge eating: Key synergistic role of past caloric restriction and stress. PHYSIOLOGY \& BEHAVIOR. 2002;77(1):45-54.

665. Wonderlich S, Mitchell JE The role of personality in the onset of eating disorders and treatment implications. PSYCHIATRIC CLINICS OF NORTH AMERICA. 2001;24(2):249+.

666. Messer Mariel, Tylka Tracy, Fuller-Tyszkiewicz Matthew, Linardon Jake Does body appreciation predict decreases in eating pathology via intuitive eating? A longitudinal mediation analysis. BODY IMAGE. 2022;43():107-111.

667. Hinojo-Lucena Francisco-Javier, Aznar-Diaz Inmaculada, Caceres-Reche Maria-Pilar, Trujillo-Torres Juan-Manuel, Romero-Rodriguez Jose-Maria Problematic Internet Use as a Predictor of Eating Disorders in Students: A Systematic Review and Meta-Analysis Study. NUTRIENTS. 2019;11(9):.

668. Marcus MD, Moulton MM, Greeno CG Binge eating onset in obese patients with binge eating disorder. ADDICTIVE BEHAVIORS. 1995;20(6):747-755.

669. Boswell Rebecca, Lydecker Janet Double trouble? Associations of parental substance use and eating behaviors with pediatric disordered eating. ADDICTIVE BEHAVIORS. 2021;123():.

670. Yanovski SZ Binge eating disorder and obesity in 2003: Could treating an eating disorder have a positive effect on the obesity epidemic?. INTERNATIONAL JOURNAL OF EATING DISORDERS. 2003;34(S):S117-S120.

671. Blackstone Sarah, Sangiorgio Celeste, Johnson Aimee Peer Recognition of Disordered Eating Behaviors: Implications for Improving Awareness through Health Education. AMERICAN JOURNAL OF HEALTH EDUCATION. 2020;51(3):142-150.

672. Neumark-Sztainer Dianne, Bauer Katherine, Friend Sarah, Hannan Peter, Story Mary, Berge Jerica Family Weight Talk and Dieting: How Much Do They Matter for Body Dissatisfaction and Disordered Eating Behaviors in Adolescent Girls?. JOURNAL OF ADOLESCENT HEALTH. 2010;47(3):270-276.

673. Handford Charlotte, Rapee Ronald, Fardouly Jasmine The influence of maternal modeling on body image concerns and eating disturbances in preadolescent girls. BEHAVIOUR RESEARCH AND THERAPY. 2018;100():17-23.

674. Jones JM, Bennett S, Olmsted MP, Lawson ML, Rodin G Disordered eating attitudes and behaviours in teenaged girls: a school-based study. CANADIAN MEDICAL ASSOCIATION JOURNAL. 2001;165(5):547-552.

675. Vogeltanz-Holm ND, Wonderlich SA, Lewis BA, Wilsnack SC, Harris TR, Wilsnack RW, Kristjanson AF Longitudinal predictors of binge eating, intense dieting, and weight concerns in a national sample of women. BEHAVIOR THERAPY. 2000;31(2):221-235.

676. Smink Frederique, Hoeken Daphne, Hoek Hans Epidemiology of Eating Disorders: Incidence, Prevalence and Mortality Rates. CURRENT PSYCHIATRY REPORTS. 2012;14(4):406-414.

677. Combs Jessica, Pearson Carolyn, Zapolski Tamika, Smith Gregory Preadolescent Disordered Eating Predicts Subsequent Eating Dysfunction. JOURNAL OF PEDIATRIC PSYCHOLOGY. 2013;38(1):41-49.

678. Rivera-Iniguez Ingrid, Panduro Arturo, Villasenor-Bayardo Sergio, Sepulveda-Villegas Maricruz, Ojeda-Granados Claudia, Roman Sonia Influence of a Nutrigenetic Intervention on Self-Efficacy, Emotions, and Rewarding Behaviors in Unhealthy Eating among Mexicans: An Exploratory Pilot Study. NUTRIENTS. 2022;14(1):.

679. Eisenberg Marla, Neumark-Sztainer Dianne Friends' Dieting and Disordered Eating Behaviors Among Adolescents Five Years Later: Findings From Project EAT. JOURNAL OF ADOLESCENT HEALTH. 2010;47(1):67-73.

680. Goldschmidt Andrea, Tanofsky-Kraff Marian, Goossens Lien, Eddy Kamryn, Ringham Rebecca, Yanovski Susan, Braet Caroline, Marcus Marsha, Wilfley Denise, Yanovski Jack Subtyping children and adolescents with loss of control eating by negative affect and dietary restraint. BEHAVIOUR RESEARCH AND THERAPY. 2008;46(7):777-787.

681. Jacobi Corinna, Voelker Ulrike, Trockel Mickey, Taylor Craig Effects of an Internet-based intervention for subthreshold eating disorders: A randomized controlled trial. BEHAVIOUR RESEARCH AND THERAPY. 2012;50(2):93-99.

682. Harrell Zaje, Jackson Benita Thinking fat and feeling blue: Eating behaviors, ruminative coping, and depressive symptoms in college women. SEX ROLES. 2008;58(9-10):658-665.

683. Grilo CM, Masheb RM Onset of dieting vs binge eating in outpatients with binge eating disorder. INTERNATIONAL JOURNAL OF OBESITY. 2000;24(4):404-409.

684. Yamamiya Yuko, Desjardins Christopher, Stice Eric Sequencing of symptom emergence in anorexia nervosa, bulimia nervosa, binge eating disorder, and purging disorder in adolescent girls and relations of prodromal symptoms to future onset of these eating disorders. PSYCHOLOGICAL MEDICINE. 2022;():.

685. Tanofsky-Kraff Marian, Wilfley Denise, Young Jami, Mufson Laura, Yanovski Susan, Glasofer Deborah, Salaita Christine Preventing excessive weight gain in adolescents: Interpersonal psychotherapy for binge eating. OBESITY. 2007;15(6):1345-1355.

686. Stice Eric, Yokum Sonja, Rohde Paul, Shaw Heather, Gau Jeff, Johnson Sarah, Johns Aviva Randomized Trial of a Dissonance-Based Transdiagnostic Group Treatment for Eating Disorders: An Evaluation of Target Engagement. JOURNAL OF CONSULTING AND CLINICAL PSYCHOLOGY. 2019;87(9):772-786.

687. Neumark-Sztainer Dianne, Wall Melanie, Larson Nicole, Eisenberg Marla, Loth Katie Dieting and Disordered Eating Behaviors from Adolescence to Young Adulthood: Findings from a 10-Year Longitudinal Study. JOURNAL OF THE AMERICAN DIETETIC ASSOCIATION. 2011;111(7):1004-1011.

688. Ramklint Mia, Jeansson Mats, Holmgren Sven, Ghaderi Ata Assessing personality disorders in eating disordered patients using the SCID-II: Influence of measures and timing on prevalence rate. PERSONALITY AND INDIVIDUAL DIFFERENCES. 2010;48(2):218-223.

689. Matthews-Ewald Molly, Zullig Keith, Ward Rose Sexual orientation and disordered eating behaviors among self-identified male and female college students. EATING BEHAVIORS. 2014;15(3):441-444.

690. Toni Giada, Berioli Maria, Cerquiglini Laura, Ceccarini Giulia, Grohmann Ursula, Principi Nicola, Esposito Susanna Eating Disorders and Disordered Eating Symptoms in Adolescents with Type 1 Diabetes. NUTRIENTS. 2017;9(8):.

691. Haines J, Neumark-Sztainer D, Eisenberg ME, Hannan PJ Weight teasing and disordered eating behaviors in adolescents: Longitudinal findings from Project EAT (Eating Among Teens). PEDIATRICS. 2006;117(2):E209-E215.

692. Hilbert A Binge eating disorder in children and adolescents. KINDHEIT UND ENTWICKLUNG. 2005;14(4):209-221.

693. Darby A., Hay P., Mond J., Rodgers B., Owen C. Disordered eating behaviours and cognitions in young women with obesity: relationship with psychological status. INTERNATIONAL JOURNAL OF OBESITY. 2007;31(5):876-882.

694. Klein David, Sylvester Jillian, Schvey Natasha Eating Disorders in Primary Care: Diagnosis and Management. AMERICAN FAMILY PHYSICIAN. 2021;103(1):22-32.

695. Hay Phillipa, Mitchison Deborah Eating Disorders and Obesity: The Challenge for Our Times. NUTRIENTS. 2019;11(5):.

696. Derks Ivonne, Harris Holly, Staats Soundry, Gaillard Romy, Dieleman Gwen, Llewellyn Clare, Swanson Sonja, Jansen Pauline Subclinical binge eating symptoms in early adolescence and its preceding and concurrent factors: a population-based study. JOURNAL OF EATING DISORDERS. 2022;10(1):.

697. Tseng MC, Lee MB, Chen SY, Lee YJ, Lin KH, Chen PR, Lai JS Response of Taiwanese obese binge eaters to a hospital-based weight reduction program. JOURNAL OF PSYCHOSOMATIC RESEARCH. 2004;57(3):279-285.

698. Perpina Conxa, Borra Cristina Body-esteem and teasing in female binge-eaters: developing a negative body image. BEHAVIORAL PSYCHOLOGY-PSICOLOGIA CONDUCTUAL. 2010;18(1):91-104.

699. Field Alison, Javaras Kristin, Aneja Parul, Kitos Nicole, Camargo Carlos, Taylor C., Laird Nan Family, peer, and media predictors of becoming eating disordered. ARCHIVES OF PEDIATRICS \& ADOLESCENT MEDICINE. 2008;162(6):574-579.

700. Conceicao Eva, Mitchell James, Engel Scott, Machado Paulo, Lancaster Kathryn, Wonderlich Stephen What is ``grazing{''}? Reviewing its definition, frequency, clinical characteristics, and impact on bariatric surgery outcomes, and proposing a standardized

definition. SURGERY FOR OBESITY AND RELATED DISEASES. 2014;10(5):973-982.

701. Jordan Jennifer, Joyce Peter, Carter Frances, Horn Jacqueline, McIntosh Virginia, Luty Suzanne, McKenzie Janice, Frampton Christopher, Mulder Roger, Bulik Cynthia Specific and nonspecific Comorbidity in anorexia nervosa. INTERNATIONAL JOURNAL OF EATING DISORDERS. 2008;41(1):47-56.

702. Jeffers Amy, Benotsch Eric Non-medical use of prescription stimulants for weight loss, disordered eating, and body image. EATING BEHAVIORS. 2014;15(3):414-418.

703. Tzischinsky O, Latzer Y Nocturnal eating: Prevalence, features and night sleep among binge eating disorder and bulimia nervosa patients in Israel. EUROPEAN EATING DISORDERS REVIEW. 2004;12(2):101-109.

704. Hilbert Anja, Pike Kathleen, Goldschmidt Andrea, Wilfley Denise, Fairburn Christopher, Dohm Faith-Anne, Walsh B., Weissman Ruth Risk factors across the eating disorders. PSYCHIATRY RESEARCH. 2014;220(1-2):500-506.

705. Freedman Julie, Hage Sally, Quatromoni Paula Eating Disorders in Male Athletes: Factors Associated With Onset and Maintenance. JOURNAL OF CLINICAL SPORT PSYCHOLOGY. 2021;15(3):227-248.

706. Ali Kathina, Fassnacht Daniel, Farrer Louise, Rieger Elizabeth, Feldhege Johannes, Moessner Markus, Griffiths Kathleen, Bauer Stephanie What prevents young adults from seeking help? Barriers toward help-seeking for eating disorder symptomatology. INTERNATIONAL JOURNAL OF EATING DISORDERS. 2020;53(6):894-906.

707. Conviser Jenny, Fisher Sheehan, McColley Susanna Are children with chronic illnesses requiring dietary therapy at risk for disordered eating or eating disorders? A systematic review. INTERNATIONAL JOURNAL OF EATING DISORDERS. 2018;51(3):187-213.

708. Gearhardt Ashley, White Marney, Masheb Robin, Grilo Carlos An examination of food addiction in a racially diverse sample of obese patients with binge eating disorder in primary care settings. COMPREHENSIVE PSYCHIATRY. 2013;54(5):500-505.

709. Grilo CM Subtyping female adolescent psychiatric inpatients with features of eating disorders along dietary restraint and negative affect dimensions. BEHAVIOUR RESEARCH

AND THERAPY. 2004;42(1):67-78.

710. Ambwani Suman, Sellinger Gina, Rose Kelsey, Richmond Tracy, Sonneville Kendrin ``It's Healthy Because It's Natural.{''} Perceptions of ``Clean{''} Eating among US Adolescents and Emerging Adults. NUTRIENTS. 2020;12(6):.

711. Higgins Neyland M., Shank Lisa, Burke Natasha, Schvey Natasha, Pine Abigail, Quattlebaum Mary, Leu William, Gillmore Dakota, Morettini Alexandria, Wilfley Denise, Stephens Mark, Sbrocco Tracy, Yanovski Jack, Jorgensen Sarah, Klein David, Olsen Cara, Quinlan Jeffrey, Tanofsky-Kraff Marian Parental deployment and distress, and adolescent disordered eating in prevention-seeking military dependents. INTERNATIONAL JOURNAL OF EATING DISORDERS. 2020;53(2):201-209.

712. Davis C Eating disorders and hyperactivity: A psychobiological perspective. CANADIAN JOURNAL OF PSYCHIATRY-REVUE CANADIENNE DE PSYCHIATRIE. 1997;42(2):168-175.

713. Blomquist Kerstin, Milsom Vanessa, Barnes Rachel, Boeka Abbe, White Marney, Masheb Robin, Grilo Carlos Metabolic syndrome in obese men and women with binge eating disorder: developmental trajectories of eating and weight-related behaviors. COMPREHENSIVE PSYCHIATRY. 2012;53(7):1021-1027.

714. Napolitano Melissa, Himes Susan Race, weight, and correlates of binge eating in female college students. EATING BEHAVIORS. 2011;12(1):29-36.

715. THACKWRAY DE, SMITH MC, BODFISH JW, MEYERS AW A COMPARISON OF BEHAVIORAL AND COGNITIVE-BEHAVIORAL INTERVENTIONS FOR BULIMIA-NERVOSA. JOURNAL OF CONSULTING AND CLINICAL PSYCHOLOGY. 1993;61(4):639-645.

716. Verbeek Dorothe, Petermann Franz Eating Disorders. KINDHEIT UND ENTWICKLUNG. 2019;28(4):191-196.

717. Goodwin Huw, Haycraft Emma, Meyer Caroline Psychological risk factors for compulsive exercise: A longitudinal investigation of adolescent boys and girls. PERSONALITY AND INDIVIDUAL DIFFERENCES. 2014;68():83-86.

718. Neumark-Sztainer Dianne, Berg Patricia, Hannan Peter, Story Mary Self-weighing in adolescents: Helpful or harmful? Longitudinal associations with body weight changes and

disordered eating. JOURNAL OF ADOLESCENT HEALTH. 2006;39(6):811-818.

719. Saltzman Jaclyn, Liechty Janet Family correlates of childhood binge eating: A systematic review. EATING BEHAVIORS. 2016;22():62-71.

720. Gamero-Villarroel Carmen, Rodriguez-Lopez Raquel, Jimenez Mercedes, Carrillo Juan, Garcia-Herraiz Angustias, Albuquerque David, Flores Isalud, Gervasini Guillermo Melanocortin-4 receptor gene variants are not associated with binge-eating behavior in nonobese patients with eating disorders. PSYCHIATRIC GENETICS. 2015;25(1):35-38.

721. Ishak Sharifah, Chin Yit, Taib Mohd., Shariff Zalilah School-based intervention to prevent overweight and disordered eating in secondary school Malaysian adolescents: a study protocol. BMC PUBLIC HEALTH. 2016;16():.

722. Brewerton Timothy, Rance Samantha, Dansky Bonnie, O'Neil Patrick, Kilpatrick Dean A Comparison of Women with Child-Adolescent Versus Adult Onset Binge Eating: Results from the National Women's Study. INTERNATIONAL JOURNAL OF EATING DISORDERS. 2014;47(7, SI):836-843.

723. Mason Tyler, Do Bridgette, Chu Daniel, Belcher Britni, Dunton Genevieve, Lopez Nanette Associations among affect, diet, and activity and binge-eating severity using ecological momentary assessment in a non-clinical sample of middle-aged fathers. EATING AND WEIGHT DISORDERS-STUDIES ON ANOREXIA BULIMIA AND OBESITY. 2022;27(2):543-551.

724. Mond J., Hay P., Darby A., Paxton S., Quirk F., Buttner P., Owen C., Rodgers B. Women With Bulimic Eating Disorders: When Do They Receive Treatment for an Eating Problem?. JOURNAL OF CONSULTING AND CLINICAL PSYCHOLOGY. 2009;77(5):835-844.

725. Leslie Monica, Leppanen Jenni, Paloyelis Yannis, Treasure Janet The influence of oxytocin on eating behaviours and stress in women with bulimia nervosa and binge eating disorder. MOLECULAR AND CELLULAR ENDOCRINOLOGY. 2019;497():.

726. Grigg M, Bowman J, Redman S Disordered eating and unhealthy weight reduction practices among adolescent females. PREVENTIVE MEDICINE. 1996;25(6):748-756.

727. Romano Kelly, Heron Kristin, Ferguson Giselle, Scott Stacey Emotion word use patterns and eating disorder symptoms: Considering the circumplex model of affect and basic emotions theory. INTERNATIONAL JOURNAL OF EATING DISORDERS. 2022;():.

728. NeumarkSztainer D, Story M, French SA Covariations of unhealthy weight loss behaviors and other high-risk behaviors among adolescents. ARCHIVES OF PEDIATRICS \& ADOLESCENT MEDICINE. 1996;150(3):304-308.

729. Feltner Cynthia, Peat Christine, Reddy Shivani, Riley Sean, Berkman Nancy, Middleton Jennifer, Balio Casey, Coker-Schwimmer Manny, Jonas Daniel Screening for Eating Disorders in Adolescents and Adults Evidence Report and Systematic Review for the US Preventive Services Task Force. JAMA-JOURNAL OF THE AMERICAN MEDICAL ASSOCIATION. 2022;327(11):1068-1082.

730. Follansbee-Junger Katherine, Janicke David, Sallinen Bethany The Influence of a Behavioral Weight Management Program on Disordered Eating Attitudes and Behaviors in Children with Overweight. JOURNAL OF THE AMERICAN DIETETIC ASSOCIATION. 2010;110(11):1653-1659.

731. Carter JC, Bewell C, Blackmore E, Woodside DB The impact of childhood sexual abuse in anorexia nervosa. CHILD ABUSE \& NEGLECT. 2006;30(3):257-269.

732. Kells Meredith, Kelly-Weeder Susan Binge eating behavior among a cohort of normal weight college women. JOURNAL OF THE AMERICAN ASSOCIATION OF NURSE PRACTITIONERS. 2019;31(12):741-746.

733. Neyland M., Shank Lisa, Lavender Jason, Rice Alexander, Schindler Rachel, Hennigan Kathrin, Solomon Senait, Kroke Phillip, Schvey Natasha, Sbrocco Tracy, Wilfley Denise, Jorgensen Sarah, Yanovski Jack, Olsen Cara, Haigney Mark, Klein David, Quinlan Jeffrey, Tanofsky-Kraff Marian Permanent change of station moves and disordered-eating attitudes and behaviors in prevention-seeking adolescent military-dependents. EATING BEHAVIORS. 2021;40():.

734. Allen Karina, Byrne Susan, Oddy Wendy, Schmidt Ulrike, Crosby Ross Risk Factors for Binge Eating and Purging Eating Disorders: Differences Based on Age of Onset. INTERNATIONAL JOURNAL OF EATING DISORDERS. 2014;47(7, SI):802-812.

735. Simone Melissa, Telke Susan, Anderson Lisa, Eisenberg Marla, Neumark-Sztainer Dianne Ethnic/racial and gender differences in disordered eating behavior prevalence trajectories among women and men from adolescence into adulthood. SOCIAL SCIENCE \& MEDICINE. 2022;294():.

736. Striegel-Moore Ruth, Dohm Faith-Anne, Kraemer Helena, Schreiber George, Taylor C., Daniels Stephen Risk factors for binge-eating disorders: An exploratory study. INTERNATIONAL JOURNAL OF EATING DISORDERS. 2007;40(6):481-487.

737. Gagne Danielle, Von Holle Ann, Brownley Kimberly, Runfola Cristin, Hofmeier Sara, Branch Kateland, Bulik Cynthia Eating disorder symptoms and weight and shape concerns in a large web-based convenience sample of women ages 50 and above: Results of the gender and body image (GABI) study. INTERNATIONAL JOURNAL OF EATING DISORDERS. 2012;45(7):832-844.

738. Kass Andrea, Wilfley Denise, Eddy Kamryn, Boutelle Kerri, Zucker Nancy, Peterson Carol, Le Grange Daniel, Celio-Doyle Angela, Goldschmidt Andrea Secretive eating among youth with overweight or obesity. APPETITE. 2017;114():275-281.

739. Nagata Jason, Thurston Idia, Karazsia Bryan, Woolridge Daniel, Buckelew Sara, Murray Stuart, Calzo Jerel Self-reported eating disorders and sleep disturbances in young adults: a prospective cohort study. EATING AND WEIGHT DISORDERS-STUDIES ON ANOREXIA BULIMIA AND OBESITY. 2021;26(2):695-702.

740. Frank Guido, Shott Megan, DeGuzman Marisa The Neurobiology of Eating Disorders. CHILD AND ADOLESCENT PSYCHIATRIC CLINICS OF NORTH AMERICA. 2019;28(4):629+.

741. Kroshus Emily, Goldman Roberta, Kubzansky Laura Team-Level Approaches to Addressing Disordered Eating: A Qualitative Study of Two Female Collegiate Cross Country Running Teams. EATING DISORDERS. 2014;22(2):136-151.

742. Freidlich Cassandra, Covarrubias Andrea, Park Hyoungjin, Murray Stuart Updates in the treatment of Eating Disorders in 2022: a year in review in Eating Disorders: The Journal of Treatment \& Prevention. EATING DISORDERS. 2023;31(2):128-138.

743. Wells LA, Sadowski CA Bulimia nervosa: an update and treatment recommendations. CURRENT OPINION IN PEDIATRICS. 2001;13(6):591-597.

744. Albayrak Ozgur, Pott Wilfried, Hebebrand Johannes, Zwaan Martina, Pauli-Pott Ursula Baseline Dietary Restraint Predicts Negative Treatment Outcomes after 12 Months in Children and Adolescents with Obesity Participating in a Lifestyle Intervention. OBESITY FACTS. 2019;12(2):179-189.

745. Dingemans Alexandra, Furth Eric Binge eating disorder psychopathology in normal weight and obese individuals. INTERNATIONAL JOURNAL OF EATING DISORDERS. 2012;45(1):135-138.

746. Elran-Barak Roni, Sztainer Maya, Goldschmidt Andrea, Crow Scott, Peterson Carol, Hill Laura, Crosby Ross, Powers Pauline, Mitchell James, Le Grange Daniel Dietary Restriction Behaviors and Binge Eating in Anorexia Nervosa, Bulimia Nervosa and Binge Eating Disorder: Trans-diagnostic Examination of the Restraint Model. EATING BEHAVIORS. 2015;18():192-196.

747. Romano Kelly, Heron Kristin, Amerson Rachel, Howard Lindsay, MacIntyre Rachel, Mason Tyler Changes in disordered eating behaviors over 10 or more years: A meta-analysis. INTERNATIONAL JOURNAL OF EATING DISORDERS. 2020;53(7):1034-1055.

748. Pearson Carolyn, Smith Gregory Bulimic Symptom Onset in Young Girls: A Longitudinal Trajectory Analysis. JOURNAL OF ABNORMAL PSYCHOLOGY. 2015;124(4):1003-1013.

749. Mazzeo Suzanne, Lydecker Janet, Harney Megan, Palmberg Allison, Kelly Nichole, Gow Rachel, Bean Melanie, Thornton Laura, Tanofsky-Kraff Marian, Bulik Cynthia, Latzer Yael, Stern Marilyn Development and preliminary effectiveness of an innovative treatment for binge eating in racially diverse adolescent girls. EATING BEHAVIORS. 2016;22():199-205.

750. Hudson James, Hiripi Eva, Pope Harrison, Kessler Ronald The prevalence and correlates of eating disorders in the national comorbidity survey replication. BIOLOGICAL PSYCHIATRY. 2007;61(3):348-358.

751. Cassidy E, Allsopp M, Williams T Obsessive compulsive symptoms at initial presentation of adolescent eating disorders. EUROPEAN CHILD \& ADOLESCENT PSYCHIATRY. 1999;8(3):193-199.

752. Hunt Tyler, Forbush Kelsie Is ``drunkorexia{''} an eating disorder, substance use disorder, or both?. EATING BEHAVIORS. 2016;22():40-45.

753. Horta Cristina, Pimenta Filipa, Leal Isabel, Maroco Joao FACTORS FOR THE BEGINNING AND MAINTENANCE OF OBESITY/ EXCESS WEIGHT: A COMPARISON BETWEEN ADULTS WITH BINGE EATING AND NO BINGE EATING. RBONE-REVISTA BRASILEIRA DE OBESIDADE NUTRICAO E EMAGRECIMENTO. 2018;12(69):3-16.

754. Linardon Jake, Gleeson John, Yap Keong, Murphy Kylie, Brennan Leah Meta-analysis of the effects of third-wave behavioural interventions on disordered eating and body image concerns: implications for eating disorder prevention. COGNITIVE BEHAVIOUR THERAPY. 2019;48(1):15-38.

755. Santonastaso P, Ferrara S, Favaro A Differences between binge eating disorder and nonpurging bulimia nervosa. INTERNATIONAL JOURNAL OF EATING DISORDERS. 1999;25(2):215-218.

756. Butryn Meghan, Juarascio Adrienne, Lowe Michael The Relation of Weight Suppression and BMI to Bulimic Symptoms. INTERNATIONAL JOURNAL OF EATING DISORDERS. 2011;44(7):612-617.

757. Stice E, Killen JD, Hayward C, Taylor CB Age of onset for binge eating and purging during late adolescence: A 4-year survival analysis. JOURNAL OF ABNORMAL PSYCHOLOGY. 1998;107(4):671-675.

758. Matherne Camden, Tanofsky-Kraff Marian, Altschul Anne, Shank Lisa, Schvey Natasha, Brady Sheila, Galescu Ovidiu, Demidowich Andrew, Yanovski Susan, Yanovski Jack A preliminary examination of Loss of Control Eating Disorder (LOC-ED) in middle childhood. EATING BEHAVIORS. 2015;18():57-61.

759. Kass Andrea, Jones Megan, Kolko Rachel, Altman Myra, Fitzsimmons-Craft Ellen, Eichen Dawn, Balantekin Katherine, Trockel Mickey, Taylor C., Wilfley Denise Universal prevention efforts should address eating disorder pathology across the weight spectrum: Implications for screening and intervention on college campuses. EATING BEHAVIORS. 2017;25(SI):74-80.

760. Keski-Rahkonen Anna, Mustelin Linda Epidemiology of eating disorders in Europe: prevalence, incidence, comorbidity, course, consequences, and risk factors. CURRENT OPINION IN PSYCHIATRY. 2016;29(6):340-345.

761. Pearson Carolyn, Miller Jonathan, Ackard Diann, Loth Katie, Wall Melanie, Haynos Ann, Neumark-Sztainer Dianne Stability and change in patterns of eating disorder symptoms from adolescence to young adulthood. INTERNATIONAL JOURNAL OF EATING DISORDERS. 2017;50(7):748-757.

762. Hagan Kelsey, Bohon Cara Subcortical brain volume and cortical thickness in adolescent girls and women with binge eating. INTERNATIONAL JOURNAL OF EATING DISORDERS. 2021;54(8):1527-1536.

763. Dakanalis Antonios, Zanetti Maria, Colmegna Fabrizia, Riva Giuseppe, Clerici Massimo Classifying binge eating-disordered adolescents based on severity levels. JOURNAL OF ADOLESCENCE. 2018;62():47-54.

764. Luca Antonina, Luca Maria, Calandra Carmela Eating Disorders in Late-life. AGING AND DISEASE. 2015;6(1):48-55.

765. Kulshreshtha Monika, Babu Nandita, Goel Neha, Chandel Shivani Disordered eating attitudes and body shape concerns among North Indian Kathak dancers. INTERNATIONAL JOURNAL OF EATING DISORDERS. 2021;54(2, SI):148-154.

766. Schaumberg Katherine, Zerwas Stephanie, Goodman Erica, Yilmaz Zeynep, Bulik Cynthia, Micali Nadia Anxiety disorder symptoms at age 10 predict eating disorder symptoms and diagnoses in adolescence. JOURNAL OF CHILD PSYCHOLOGY AND PSYCHIATRY. 2019;60(6):686-696.

767. Tanofsky-Kraff M, Faden D, Yanovski SZ, Wilfley DE, Yanovski JA The perceived onset of dieting and loss of control eating behaviors in overweight children. INTERNATIONAL JOURNAL OF EATING DISORDERS. 2005;38(2):112-122.

768. Filbey Francesca, Myers Ursula, DeWitt Samuel Reward circuit function in high BMI individuals with compulsive overeating: Similarities with addiction. NEUROIMAGE. 2012;63(4):1800-1806.

769. Carwell Micaela, Spatz Diane Eating Disorders \& Breastfeeding. MCN-THE AMERICAN JOURNAL OF MATERNAL-CHILD NURSING. 2011;36(2):112-117.

770. Glazer Kimberly, Ziobrowski Hannah, Horton Nicholas, Calzo Jerel, Field Alison The Course of Weight/Shape Concerns and Disordered Eating Symptoms Among Adolescent and Young Adult Males. JOURNAL OF ADOLESCENT HEALTH. 2021;69(4):615-621.

771. Liechty Janet, Lee Meng-Jung Longitudinal Predictors of Dieting and Disordered Eating Among Young Adults in the US. INTERNATIONAL JOURNAL OF EATING DISORDERS. 2013;46(8):790-800.

772. Jones Megan, Luce Kristine, Osborne Megan, Taylor Katherine, Cunning Darby, Doyle Angela, Wilfley Denise, Taylor C. Randomized, controlled trial of an internet-facilitated intervention for reducing binge eating and overweight in adolescents. PEDIATRICS. 2008;121(3):453-462.

773. Monteleone Palmiero, Maj Mario Genetic susceptibility to eating disorders: associated polymorphisms and pharmacogenetic suggestions. PHARMACOGENOMICS. 2008;9(10):1487-1520.

774. Bulik Cynthia, Coleman Jonathan, Hardaway J., Breithaupt Lauren, Watson Hunna, Bryant Camron, Breen Gerome Genetics and neurobiology of eating disorders. NATURE NEUROSCIENCE. 2022;25(5):543-554.

775. Meyer C., Blissett J., Alberry R., Sykes A. Beliefs about exercise: Relationship to eating psychopathology and core beliefs among young female exercisers. EATING BEHAVIORS. 2013;14(1):79-82.

776. Coimbra Maria, Ferreira Claudia Making the leap from healthy to disordered eating: the role of intuitive and inflexible eating attitudes in orthorexic behaviours among women. EATING AND WEIGHT DISORDERS-STUDIES ON ANOREXIA BULIMIA AND OBESITY. 2021;26(6):1793-1800.

777. Stoyel Hannah, Delderfield Russell, Shanmuganathan-Felton Vaithehy, Stoyel Alex, Serpell Lucy A Qualitative Exploration of Sport and Social Pressures on Elite Athletes in Relation to Disordered Eating. FRONTIERS IN PSYCHOLOGY. 2021;12():.

778. Borden Ashlye, Cook-Cottone Catherine Yoga and eating disorder prevention and treatment: A comprehensive review and meta-analysis. EATING DISORDERS. 2020;28(4, SI):400-437.

779. Stice Eric, Desjardins Christopher, Rohde Paul, Shaw Heather Sequencing of Symptom Emergence in Anorexia Nervosa, Bulimia Nervosa, Binge Eating Disorder, and Purging Disorder and Relations of Prodromal Symptoms to Future Onset of These Disorders. JOURNAL OF ABNORMAL PSYCHOLOGY. 2021;130(4):377-387.

780. Sharpe Helen, Griffiths Scott, Choo Tse-Hwei, Eisenberg Marla, Mitchison Deborah, Wall Melanie, Neumark-Sztainer Dianne The relative importance of dissatisfaction, overvaluation and preoccupation with weight and shape for predicting onset of disordered eating behaviors and depressive symptoms over 15 years. INTERNATIONAL JOURNAL OF EATING DISORDERS. 2018;51(10):1168-1175.

781. Zaider TI, Johnson JG, Cockell SJ Psychiatric disorders associated with the onset and persistence of bulimia nervosa and binge eating disorder during adolescence. JOURNAL OF YOUTH AND ADOLESCENCE. 2002;31(5):319-329.

782. Alboni Silvia, Di Bonaventura Maria, Benatti Cristina, Giusepponi Maria, Brunello Nicoletta, Cifani Carlo Hypothalamic expression of inflammatory mediators in an animal model of binge eating. BEHAVIOURAL BRAIN RESEARCH. 2017;320():420-430.

783. Sahlan Reza, Williams Brenna, Forrest Lauren, Saunders Jessica, Fitzsimmons-Craft Ellen, Levinson Cheri Disordered eating,self-esteem, and depression symptoms in Iranian adolescents and young adults: A network analysis. INTERNATIONAL JOURNAL OF EATING DISORDERS. 2021;54(2, SI):132-147.

784. Lowe Michael, Thomas J., Safer Debra, Butryn Meghan The relationship of weight suppression and dietary restraint to binge eating in bulimia nervosa. INTERNATIONAL JOURNAL OF EATING DISORDERS. 2007;40(7):640-644.

785. Goossens Lien, Braet Caroline, Verbeken Sandra, Decaluwe Veerle, Bosmans Guy Long-Term Outcome of Pediatric Eating Pathology and Predictors for the Onset of Loss of Control over Eating Following Weight-Loss Treatment. INTERNATIONAL JOURNAL OF EATING DISORDERS. 2011;44(5):397-405.

786. Stein Karen, Corte Colleen, Ronis David Personal identities and disordered eating behaviors in Mexican American women. EATING BEHAVIORS. 2010;11(3):197-200.

787. Au Emily, Cosh Suzanne Social media and eating disorder recovery: An exploration of Instagram recovery community users and their reasons for engagement. EATING

BEHAVIORS. 2022;46():.

788. Sehm Marie, Warschburger Petra Prospective Associations Between Binge Eating and Psychological Risk Factors in Adolescence. JOURNAL OF CLINICAL CHILD AND ADOLESCENT PSYCHOLOGY. 2018;47(5):770-784.

789. MacNeil Laura, Esposito-Smythers Christianne, Mehlenbeck Robyn, Weismoore Julie The effects of avoidance coping and coping self-efficacy on eating disorder attitudes and behaviors: A stress-diathesis model. EATING BEHAVIORS. 2012;13(4):293-296.

790. Penas-Lledo E., Fernandez-Aranda F., Jimenez-Murcia S., Granero R., Penelo E., Soto A., Gunnard K., Menchon J. Subtyping eating disordered patients along drive for thinness and depression. BEHAVIOUR RESEARCH AND THERAPY. 2009;47(6):513-519.

791. Gaviria David, Ammerman Alice Eating disorders and disordered eating in servicemen and women: A narrative review. JOURNAL OF CLINICAL PSYCHOLOGY. 2023;79(2):316-373.

792. Linardon Jake, Mitchell Sarah Rigid dietary control, flexible dietary control, and intuitive eating: Evidence for their differential relationship to disordered eating and body image concerns. EATING BEHAVIORS. 2017;26():16-22.

793. Degortes Daniela, Santonastaso Paolo, Zanetti Tatiana, Tenconi Elena, Veronese Angela, Favaro Angela Stressful Life Events and Binge Eating Disorder. EUROPEAN EATING DISORDERS REVIEW. 2014;22(5):378-382.

794. Bosch Amanda, Miltenberger Raymond, Gross Amy, Knudson Peter, Breitwieser Carrie Evaluation of extinction as a functional treatment for binge eating. BEHAVIOR MODIFICATION. 2008;32(4):556-576.

795. Hoek HW, Hoeken D Review of the prevalence and incidence of eating disorders. INTERNATIONAL JOURNAL OF EATING DISORDERS. 2003;34(4):383-396.

796. Racicka Ewa, Brynska Anita Eating Disorders in children and adolescents with type 1 and type 2 diabetes - prevalence, risk factors, warning signs. PSYCHIATRIA POLSKA. 2015;49(5):1017-1024.

797. Swenne Ingemar Evaluation of the Compulsive Exercise Test (CET) in Adolescents with Eating Disorders: Factor Structure and Relation to Eating Disordered Psychopathology. EUROPEAN EATING DISORDERS REVIEW. 2016;24(4):334-340.

798. Solomon Senait, Shank Lisa, Lavender Jason, Neyland M., Gallager-Teske Julia, Markos Bethelhem, Haynes Hannah, Repke Hannah, Rice Alexander, Sbrocco Tracy, Wilfley Denise, Schvey Natasha, Jorgensen Sarah, Ford Brian, Ford Caitlin, Haigney Mark, Klein David, Quinlan Jeffrey, Tanofsky-Kraff Marian The relationship between anxiety, coping, and disordered-eating attitudes in adolescent military-dependents at high-risk for excess weight gain. MILITARY PSYCHOLOGY. 2023;35(2):95-106.

799. Keel PK, Mitchell JE, Miller KB, Davis TL, Crow SJ Long-term outcome of bulimia nervosa. ARCHIVES OF GENERAL PSYCHIATRY. 1999;56(1):63-69.

800. Ertl Melissa, Pazienza Rena, Cannon Margeaux, Tineo Yajaira, Fresquez Cara, McDonough Alicia, Bozek Dana, Ozmat Evan, Ladouceur Guy, Planz Emily, Martin Jessica Associations between Impulsivity and Exercise Addiction, Disordered Eating, and Alcohol Use Behaviors: A Latent Profile Analysis. SUBSTANCE USE \& MISUSE. 2022;57(6):886-896.

801. Thomas J., Butryn Meghan, Stice Eric, Lowe Michael A Prospective Test of the Relation Between Weight Change and Risk for Bulimia Nervosa. INTERNATIONAL JOURNAL OF EATING DISORDERS. 2011;44(4):295-303.

802. Tobin DL, Griffing AS Coping, sexual abuse, and compensatory behavior. INTERNATIONAL JOURNAL OF EATING DISORDERS. 1996;20(2):143-148.

803. Shuttlesworth Mary, Zotter Deanne Disordered Eating in African American and Caucasian Women: The Role of Ethnic Identity. JOURNAL OF BLACK STUDIES. 2011;42(6):906-922.

804. Striegel-Moore RH, Franko DL, Thompson D, Barton B, Schreiber GB, Daniels SR Caffeine intake in eating disorders. INTERNATIONAL JOURNAL OF EATING DISORDERS. 2006;39(2):162-165.

805. Walsh BT, Devlin MJ Eating disorders: Progress and problems. SCIENCE. 1998;280(5368):1387-1390.

806. Linardon Jake, Fuller-Tyszkiewicz Matthew, Garcia Xochitl, Messer Mariel, Brennan Leah Do body checking and avoidance behaviours explain variance in disordered eating beyond attitudinal measures of body image?. EATING BEHAVIORS. 2019;32():7-11.

807. Ruhl U, Jacobi C Cognitive-behavioral psychotherapy for adolescents with eating disorders. PRAXIS DER KINDERPSYCHOLOGIE UND KINDERPSYCHIATRIE. 2005;54(4):286-302.

808. Haycraft Emma, Powell Faye, Meyer Caroline Activity-Related Parenting Practices: Development of the Parenting Related to Activity Measure (PRAM) and Links with Mothers' Eating Psychopathology and Compulsive Exercise Beliefs. EUROPEAN EATING DISORDERS REVIEW. 2015;23(1):51-61.

809. Vannucci Anna, Tanofsky-Kraff Marian, Ranzenhofer Lisa, Kelly Nichole, Hannallah Louise, Pickworth C., Grygorenko Mariya, Brady Sheila, Condarco Tania, Kozlosky Merel, Demidowich Andrew, Yanovski Susan, Shomaker Lauren, Yanovski Jack Puberty and the Manifestations of Loss of Control Eating in Children and Adolescents. INTERNATIONAL JOURNAL OF EATING DISORDERS. 2014;47(7, SI):738-747.

810. Corcos M, Taieb O, Benoit-Lamy S, Paterniti S, Jeammet P, Flament MF Suicide attempts in women with bulimia nervosa: frequency and characteristics. ACTA PSYCHIATRICA SCANDINAVICA. 2002;106(5):381-386.

811. Giles Sarah, Toohey Madeline, Hughes Elizabeth, Fuller-Tyszkiewicz Matthew, Krug Isabel Do orthorexia and intolerance of uncertainty mediate the relationship between autism spectrum traits and disordered eating symptoms?. EATING AND WEIGHT DISORDERS-STUDIES ON ANOREXIA BULIMIA AND OBESITY. 2021;26(7):2309-2316.

812. Neale Alice, Abraham Suzanne, Russell Janice ``Ice{''} Use and Eating Disorders: A Report of Three Cases. INTERNATIONAL JOURNAL OF EATING DISORDERS. 2009;42(2):188-191.

813. Brauhardt Anne, Zwaan Martina, Hilbert Anja The psychotherapeutic process in the treatment of binge eating. ZEITSCHRIFT FUR PSYCHIATRIE PSYCHOLOGIE UND PSYCHOTHERAPIE. 2014;62(1):9-17.

814. Leigh Sarah-Jane, Morris Margaret The role of reward circuitry and food addiction in the obesity epidemic: An update. BIOLOGICAL PSYCHOLOGY. 2018;131(SI):31-42.

815. Wiederman MW, Pryor T Multi-impulsivity among women with bulimia nervosa. INTERNATIONAL JOURNAL OF EATING DISORDERS. 1996;20(4):359-365.

816. Burton Amy, Abbott Maree Conceptualising Binge Eating: A Review of the Theoretical and Empirical Literature. BEHAVIOUR CHANGE. 2017;34(3):168-198.

817. Scott Stephanie, Elamin Wafa, Giles Emma, Hillier-Brown Frances, Byrnes Kate, Connor Natalie, Newbury-Birch Dorothy, Ells Louisa Socio-Ecological Influences on Adolescent (Aged 10-17) Alcohol Use and Unhealthy Eating Behaviours: A Systematic Review and Synthesis of Qualitative Studies. NUTRIENTS. 2019;11(8):.

818. Mitchison Deborah, Hay Phillipa, Slewa-Younan Shameran, Mond Jonathan Time Trends in Population Prevalence of Eating Disorder Behaviors and Their Relationship to Quality of Life. PLOS ONE. 2012;7(11):.

819. Shaw Ruth, Cassidy Tony Self-Compassion, Mindful Eating, Eating Attitudes and Wellbeing Among Emerging Adults. JOURNAL OF PSYCHOLOGY. 2022;156(1):33-47.

820. Stice Eric Interactive and Mediational Etiologic Models of Eating Disorder Onset: Evidence from Prospective Studies. . 2016;12():359-381.

821. De Panfilis Chiara, Cero Sara, Torre Mariateresa, Salvatore Paola, Dall'Aglio Elisabetta, Adorni Aristodemo, Maggini Carlo Changes in body image disturbance in morbidly obese patients 1 year after laparoscopic adjustable gastric banding. OBESITY SURGERY. 2007;17(6):792-799.

822. Pike Kathleen, Hoek Hans, Dunne Patricia Cultural trends and eating disorders. CURRENT OPINION IN PSYCHIATRY. 2014;27(6):436-442.

823. Fernandez-Aranda F, Solano R, Badia A, Jimenez-Murcia S Binge eating disorder onset by unusual parasitic intestinal disease: A case-report. INTERNATIONAL JOURNAL OF EATING DISORDERS. 2001;30(1):107-109.

824. Koritar Priscila, Philippi Sonia, Alvarenga Marie Attitudes toward health and taste of food among women with bulimia nervosa and women of a non-clinical sample. APPETITE. 2017;113():172-177.

825. Reijonen JH, Pratt HD, Patel DR, Greydanus DE Eating disorders in the adolescent population: An overview. JOURNAL OF ADOLESCENT RESEARCH. 2003;18(3):209-222.

826. Hildebrandt Britny, Fisher Hayley, LaPalombara Zoe, Young Michael, Ahmari Susanne Corticostriatal dynamics underlying components of binge-like consumption of palatable food in mice. APPETITE. 2023;183():.

827. Rodgers Rachel, Watts Allison, Austin S., Haines Jess, Neumark-Sztainer Dianne Disordered eating in ethnic minority adolescents with overweight. INTERNATIONAL JOURNAL OF EATING DISORDERS. 2017;50(6):665-671.

828. Udo Tomoko, Grilo Carlos Epidemiology of eating disorders among US adults. CURRENT OPINION IN PSYCHIATRY. 2022;35(6):372-378.

829. Dyck Zoe, Schulz Andre, Blechert Jens, Herbert Beate, Lutz Annika, Vogele Claus Gastric interoception and gastric myoelectrical activity in bulimia nervosa and binge-eating disorder. INTERNATIONAL JOURNAL OF EATING DISORDERS. 2021;54(7):1106-1115.

830. Neumark-Sztainer D, Wall M, Guo J, Story M, Haines J, Eisenberg M Obesity, disordered eating, and eating disorders in a longitudinal study of adolescents: How do dieters fare 5 years later?. JOURNAL OF THE AMERICAN DIETETIC ASSOCIATION. 2006;106(4):559-568.

831. Blomquist Kerstin, Masheb Robin, White Marney, Grilo Carlos Parental substance use history of overweight men and women with binge eating disorder is associated with distinct developmental trajectories and comorbid mood disorder. COMPREHENSIVE PSYCHIATRY. 2011;52(6):693-700.

832. Glasofer Deborah, Tanofsky-Kraff Marian, Eddy Kamryn, Yanovski Susan, Theim Kelly, Mirch Margaret, Ghorbani Samareh, Ranzenhofer Lisa, Haaga David, Yanovski Jack Binge eating in overweight treatment-seeking adolescents. JOURNAL OF PEDIATRIC PSYCHOLOGY. 2007;32(1):95-105.

833. Ambwani Suman, Shippe Meghan, Gao Ziting, Austin S. Is \#cleaneating a healthy or harmful dietary strategy? Perceptions of clean eating and associations with disordered eating among young adults. JOURNAL OF EATING DISORDERS. 2019;7():.

834. Murray Sidney, Holton Kathleen Post-traumatic stress disorder may set the neurobiological stage for eating disorders: A focus on glutamatergic dysfunction. APPETITE. 2021;167():.

835. Stice Eric, Rohde Paul, Shaw Heather, Desjardins Chris Weight suppression increases odds for future onset of anorexia nervosa, bulimia nervosa, and purging disorder, but not binge eating disorder. AMERICAN JOURNAL OF CLINICAL NUTRITION. 2020;112(4):941-947.

836. Lease Haidee, Doley Joanna, Bond Malcolm My mother told me: the roles of maternal messages, body image, and disordered eating in maladaptive exercise. EATING AND WEIGHT DISORDERS-STUDIES ON ANOREXIA BULIMIA AND OBESITY. 2016;21(3):469-476.

837. Brandsma Lynn Eating disorders across the life span. JOURNAL OF WOMEN \& AGING. 2007;19(1-2):155-172.

838. Macias LG, Unikel C, Cruz C, Caballero A Eating disorders and personality.. SALUD MENTAL. 2003;26(3):1-8.

839. Hagan MM, Shuman ES, Oswald KD, Corcoran KJ, Profitt JH, Blackburn K, Schwiebert MW, Chandler PC, Birbaum C Incidence of chaotic eating behaviors in binge-eating disorder: Contributing factors. BEHAVIORAL MEDICINE. 2002;28(3):99-105.

840. Wagner Allison, Vitousek Kelly Personality Variables and Eating Pathology. PSYCHIATRIC CLINICS OF NORTH AMERICA. 2019;42(1, 1):105+.

841. Vannucci Anna, Tanofsky-Kraff Marian, Crosby Ross, Ranzenhofer Lisa, Shomaker Lauren, Field Sara, Mooreville Mira, Reina Samantha, Kozlosky Merel, Yanovski Susan, Yanovski Jack Latent Profile Analysis to Determine the Typology of Disinhibited Eating Behaviors in Children and Adolescents. JOURNAL OF CONSULTING AND CLINICAL PSYCHOLOGY. 2013;81(3):494-507.

842. Sanchez-Carracedo David, Neumark-Sztainer Dianne, Lopez-Guimera Gemma Integrated prevention of obesity and eating disorders: barriers, developments and opportunities. PUBLIC HEALTH NUTRITION. 2012;15(12):2295-2309.

843. Gerontidis Alexandros, Grammatikopoulou Maria, Tzimos Christos, Gkiouras Konstantinos, Taousani Eleftheria, Athanasiadis Loukas, Goulis Dimitrios Effectors of Pregorexia and Emesis among Pregnant Women: A Pilot Study. NUTRIENTS. 2022;14(24):.

844. Christensen Stephanie, Bentz Mette, Clemmensen Lars, Strandberg-Larsen Katrine, Olsen Else Disordered eating behaviours and autistic traits-Are there any associations in nonclinical populations? A systematic review. EUROPEAN EATING DISORDERS REVIEW. 2019;27(1):8-23.

845. RAYMOND NC, MUSSELL MP, MITCHELL JE, DEZWAAN M, CROSBY RD AN AGE-MATCHED COMPARISON OF SUBJECTS WITH BINGE-EATING DISORDER AND BULIMIA-NERVOSA. INTERNATIONAL JOURNAL OF EATING DISORDERS. 1995;18(2):135-143.

846. Zwaan M Binge eating disorder (BED) and obesity. VERHALTENSTHERAPIE. 2002;12(4):288-295.

847. Voelker Dana, Gould Dan, Reel Justine Prevalence and correlates of disordered eating in female figure skaters. PSYCHOLOGY OF SPORT AND EXERCISE. 2014;15(6, SI):696-704.

848. McGarrity Larissa, Shepardson Robyn, Carey Kate, Carey Michael Sexual assault predicts unhealthy weight management among college women: A longitudinal, prospective study. JOURNAL OF AMERICAN COLLEGE HEALTH. 2022;():.

849. Gallardo Laura, Plumed-Domingo J., Rojo-Moreno Luis Weight-Teasing and Eating Disorders-A Comparative Study in Adolescent and Adult Samples. CHILDREN-BASEL. 2022;9(11):.

850. McElroy Susan, Frye Mark, Hellemann Gerhard, Altshuler Lori, Leverich Gabriele, Suppes Trisha, Keck Paul, Nolen Willem, Kupka Ralph, Post Robert Prevalence and correlates of eating disorders in 875 patients with bipolar disorder. JOURNAL OF AFFECTIVE DISORDERS. 2011;128(3):191-198.

851. Spurrell EB, Wilfley DE, Tanofsky MB, Brownell KD Age of onset for binge eating: Are there different pathways to binge eating?. INTERNATIONAL JOURNAL OF EATING DISORDERS. 1997;21(1):55-65.

852. Dunn Catherine, Riley Joan, Hawkins Kirsten, Tercyak Kenneth Factors Associated With Disordered Eating Behavior Among Adolescent Girls: Screening and Education. JOURNAL OF PRIMARY CARE AND COMMUNITY HEALTH. 2022;13():.

853. Reese Elizabeth, Pollert Garrett, Veilleux Jennifer Self-regulatory predictors of eating disorder symptoms: Understanding the contributions of action control and willpower beliefs. EATING BEHAVIORS. 2016;20():64-69.

854. Pine Abigail, Shank Lisa, Burke Natasha, Neyland M., Schvey Natasha, Quattlebaum Mary, Leu William, Wilfley Denise, Stephens Mark, Jorgensen Sarah, Olsen Cara, Sbrocco Tracy, Yanovski Jack, Klein David, Quinlan Jeffrey, Tanofsky-Kraff Marian Examination of the Interpersonal Model With Adolescent Military Dependents at High Risk for Adult Obesity. AMERICAN JOURNAL OF PSYCHOTHERAPY. 2020;73(2):43-49.

855. Binford RB, Mussell MP, Peterson CB, Crow SJ, Mitchell JE Relation of binge eating age of onset to functional aspects of binge eating in binge eating disorder. INTERNATIONAL JOURNAL OF EATING DISORDERS. 2004;35(3):286-292.

856. Castellini Giovanni, Zagaglioni Alice, Godini Lucia, Monami Francesca, Dini Carla, Faravelli Carlo, Ricca Valdo Religion orientations and eating disorders. RIVISTA DI PSICHIATRIA. 2014;49(3):140-144.

857. Jackson TD, Grilo CM, Masheb RM Teasing history, onset of obesity, current eating disorder psychopathology, body dissatisfaction, and psychological functioning in binge eating disorder. OBESITY RESEARCH. 2000;8(6):451-458.

858. Herle Moritz, De Stavola Bianca, Hubel Christopher, Abdulkadir Mohamed, Ferreira Diana, Loos Ruth, Bryant-Waugh Rachel, Bulik Cynthia, Micali Nadia A longitudinal study of eating behaviours in childhood and later eating disorder behaviours and diagnoses. BRITISH JOURNAL OF PSYCHIATRY. 2020;216(2):113-119.

859. Altun Hatice Association of eating attitude with anxiety and depression levels in children and adolescents with obsessive-compulsive disorder. PSYCHIATRY AND CLINICAL

PSYCHOPHARMACOLOGY. 2019;29(2):130-136.

860. Hudson James, Javaras Kristin, Pope Harrison The Challenges of Metabolic Syndrome in Eating Disorders. PSYCHIATRIC ANNALS. 2020;50(8):346-350.

861. Thiebaut S., Godart N., Radon L., Courtet P., Guillaume S. Crossed prevalence results between subtypes of eating disorder and bipolar disorder: A systematic review of the literature. ENCEPHALE-REVUE DE PSYCHIATRIE CLINIQUE BIOLOGIQUE ET THERAPEUTIQUE. 2019;45(1):60-73.

862. Corwin Rebecca, Wojnicki Francis, Zimmer Derek, Babbs R., McGrath Lauren, Olivos Diana, Mietlicki-Baase Elizabeth, Hayes Matthew Binge-Type Eating Disrupts Dopaminergic and GABAergic Signaling in the Prefrontal Cortex and Ventral Tegmental Area. OBESITY. 2016;24(10):2118-2125.

863. Leme Ana, Thompson Debbe, Dunker Karin, Nicklas Theresa, Philippi Sonia, Lopez Tabbetha, Vezina-Im Lydi-Anne, Baranowski Tom Obesity and eating disorders in integrative prevention programmes for adolescents: protocol for a systematic review and meta-analysis. BMJ OPEN. 2018;8(4):.

864. Toufexis Megan, Hommer Rebecca, Gerardi Diana, Grant Paul, Rothschild Leah, D'Souza Precilla, Williams Kyle, Leckman James, Swedo Susan, Murphy Tanya Disordered Eating and Food Restrictions in Children with PANDAS/PANS. JOURNAL OF CHILD AND ADOLESCENT PSYCHOPHARMACOLOGY. 2015;25(1, SI):48-56.

865. Brewerton TD, Dansky BS, Kilpatrick DG, O'Neil PM Which comes first in the pathogenesis of bulimia nervosa: Dieting or bingeing. INTERNATIONAL JOURNAL OF EATING DISORDERS. 2000;28(3):259-264.

866. Feldman Matthew, Meyer Ilan Comorbidity and age of onset of eating disorders in gay men, lesbians, and bisexuals. PSYCHIATRY RESEARCH. 2010;180(2-3):126-131.

867. Karam Anna, Fitzsimmons-Craft Ellen, Tanofsky-Kraff Marian, Wilfley Denise Interpersonal Psychotherapy and the Treatment of Eating Disorders. PSYCHIATRIC CLINICS OF NORTH AMERICA. 2019;42(2, 2):205+.

868. Hauck Carolin, Cook Brian, Ellrott Thomas Food addiction, eating addiction and eating disorders. PROCEEDINGS OF THE NUTRITION SOCIETY. 2020;79(1):103-112.

869. Henderson NJ, Huon GF Negative affect and binge eating in overweight women. BRITISH JOURNAL OF HEALTH PSYCHOLOGY. 2002;7(1):77-87.

870. Peterson CB, Mitchell JE Psychosocial and pharmacological treatment of eating disorders: A review of research findings. JOURNAL OF CLINICAL PSYCHOLOGY. 1999;55(6):685-697.

871. Lantz Elin, Gillberg Christopher, Rastam Maria, Wentz Elisabet, Lowe Michael Premorbid BMI predicts binge-purge symptomatology among individuals with anorexia nervosa. INTERNATIONAL JOURNAL OF EATING DISORDERS. 2017;50(7):852-855.

872. Saunders Jessica, Frazier Leslie, Nichols-Lopez Kristin Self-esteem, diet self-efficacy, body mass index, and eating disorders: modeling effects in an ethnically diverse sample. EATING AND WEIGHT DISORDERS-STUDIES ON ANOREXIA BULIMIA AND OBESITY. 2016;21(3):459-468.

873. Carrard I., Kruseman M., Di Capua D., Suringar V., Weber C. Face validity of a French questionnaire to screen binge eating disorder in adolescents. ARCHIVES DE PEDIATRIE. 2013;20(10):1105-1112.

874. Yanase Maya, Sugihara Genichi, Murai Toshiya, Noma Shun'ichi Shoplifting and eating disorders: an anonymous self-administered survey. EATING AND WEIGHT DISORDERS-STUDIES ON ANOREXIA BULIMIA AND OBESITY. 2018;23(6):753-759.

875. Kachi Yuko Latent structure of dieting among female high-school students in Japan. PERSONALITY AND INDIVIDUAL DIFFERENCES. 2010;48(1):11-15.

876. Miller-Matero Lisa, Hamann Aaron, LaLonde Leah, Martens Kellie, Son John, Clark-Sienkiewicz Shannon, Sata Monika, Coleman Joseph, Hecht Leah, Braciszewski Jordan, Carlin Arthur Predictors of Alcohol Use after Bariatric Surgery. JOURNAL OF CLINICAL PSYCHOLOGY IN MEDICAL SETTINGS. 2021;28(3):596-602.

877. Allen Karina, Byrne Susan, Oddy Wendy, Crosby Ross Early Onset Binge Eating and Purging Eating Disorders: Course and Outcome in a Population-Based Study of Adolescents.

JOURNAL OF ABNORMAL CHILD PSYCHOLOGY. 2013;41(7):1083-1096.

878. Godoy-Izquierdo Debora, Ramirez Maria, Diaz Isabel, Lopez-Mora Clara A Systematic Review on Exercise Addiction and the Disordered Eating-Eating Disorders Continuum in the Competitive Sport Context. INTERNATIONAL JOURNAL OF MENTAL HEALTH AND ADDICTION. 2023;21(1):529-561.

879. Marcus MD, Kalarchian MA Binge eating in children and adolescents. INTERNATIONAL JOURNAL OF EATING DISORDERS. 2003;34(S):S47-S57.

880. Jenkins Paul, Price Tom Eating pathology in midlife women: Similar or different to younger counterparts?. INTERNATIONAL JOURNAL OF EATING DISORDERS. 2018;51(1):3-9.

881. Mustelin Linda, Raevuori Anu, Hoek Hans, Kaprio Jaakko, Keski-Rahkonen Anna Incidence and Weight Trajectories of Binge Eating Disorder among Young Women in the Community. INTERNATIONAL JOURNAL OF EATING DISORDERS. 2015;48(8):1106-1112.

882. Wiederman MW, Pryor T A comparison of ever-married and never-married women with anorexia nervosa or bulimia nervosa. INTERNATIONAL JOURNAL OF EATING DISORDERS. 1997;22(4):395-401.

883. Hamilton Amber, Mitchison Deborah, Basten Christopher, Byrne Susan, Goldstein Mandy, Hay Phillipa, Heruc Gabriella, Thornton Christopher, Touyz Stephen Understanding treatment delay: Perceived barriers preventing treatment-seeking for eating disorders. AUSTRALIAN AND NEW ZEALAND JOURNAL OF PSYCHIATRY. 2022;56(3):248-259.

884. Yim See, Schmidt Ulrike Self-Help Treatment of Eating Disorders. PSYCHIATRIC CLINICS OF NORTH AMERICA. 2019;42(2, 2):231+.

885. Goossens Lien, Braet Caroline, Decaluwe Veerle Loss of control over eating in obese youngsters. BEHAVIOUR RESEARCH AND THERAPY. 2007;45(1):1-9.

886. White Marney, Masheb Robin, Grilo Carlos Regimented and Lifestyle Restraint in Binge Eating Disorder. INTERNATIONAL JOURNAL OF EATING DISORDERS. 2009;42(4):326-331.

887. Kaye WH, Bulik CM, Thornton L, Barbarich N, Masters K, Grp Price Comorbidity of anxiety disorders with anorexia and bulimia nervosa. AMERICAN JOURNAL OF PSYCHIATRY. 2004;161(12):2215-2221.

888. Ramacciotti CE, Paoli RA, Marcacci G, Piccinni A, Burgalassi A, Dell'Osso L, Garfinkel PE Relationship between bipolar illness and binge-eating disorders. PSYCHIATRY RESEARCH. 2005;135(2):165-170.

889. Bell Katie, Coulthard Helen, Wildbur Diane Self-Disgust within Eating Disordered Groups: Associations with Anxiety, Disgust Sensitivity and Sensory Processing. EUROPEAN EATING DISORDERS REVIEW. 2017;25(5):373-380.

890. Scott Charlotte, Haycraft Emma, Plateau Carolyn Teammate influences and relationship quality are associated with eating and exercise psychopathology in athletes. APPETITE. 2019;143():.

891. Haycraft Emma, Goodwin Huw, Meyer Caroline Adolescents' Level of Eating Psychopathology Is Related to Perceptions of Their Parents' Current Feeding Practices. JOURNAL OF ADOLESCENT HEALTH. 2014;54(2):204-208.

892. Stice E, Fisher M, Martinez E Eating Disorder Diagnostic Scale: Additional evidence of reliability and validity. PSYCHOLOGICAL ASSESSMENT. 2004;16(1):60-71.

893. Drewnowski A Metabolic determinants of binge eating. ADDICTIVE BEHAVIORS. 1995;20(6):733-745.

894. Anderson Laura, Chacko Thomas Bariatric Outcomes: Self-Management for Sustained Surgical Success: A Multicomponent Treatment for Dysregulated Overeating in Bariatric Surgery Patients. JOURNAL OF ADDICTIONS NURSING. 2018;29(1):32-42.

895. Hilbert Anja Binge-Eating Disorder. PSYCHIATRIC CLINICS OF NORTH AMERICA. 2019;42(1, 1):33+.

896. AGRAS WS, TELCH CF, ARNOW B, ELDREDGE K, DETZER MJ, HENDERSON J, MARNELL M DOES INTERPERSONAL THERAPY HELP PATIENTS WITH BINGE-EATING DISORDER WHO FAIL TO RESPOND TO COGNITIVE-BEHAVIORAL THERAPY. JOURNAL OF

CONSULTING AND CLINICAL PSYCHOLOGY. 1995;63(3):356-360.

897. Holmes Millicent, Fuller-Tyszkiewicz Matthew, Skouteris Helen, Broadbent Jaclyn Tests of an extension of the dual pathway model of bulimic symptoms to the state-based level. EATING BEHAVIORS. 2014;15(2):280-285.

898. Mitchell J., Zwaan M., Roerig J. Drug Therapy for Patients with Eating Disorders. CNS \& NEUROLOGICAL DISORDERS-DRUG TARGETS. 2003;2(1):17-29.

899. Paganini Chiara, Peterson Gregory, Stavropoulos Vasilis, Krug Isabel The Overlap between Binge Eating Behaviors and Polycystic Ovarian Syndrome: An Etiological Integrative Model. CURRENT PHARMACEUTICAL DESIGN. 2018;24(9):999-1006.

900. Goldschmidt Andrea, Crosby Ross, Engel Scott, Crow Scott, Cao Li, Peterson Carol, Durkin Nora Affect and eating behavior in obese adults with and without elevated depression symptoms. INTERNATIONAL JOURNAL OF EATING DISORDERS. 2014;47(3):281-286.

901. Omiwole Michael, Richardson Candice, Huniewicz Paulina, Dettmer Elizabeth, Paslakis Georgios Review of Mindfulness-Related Interventions to Modify Eating Behaviors in Adolescents. NUTRIENTS. 2019;11(12):.

902. Dingemans AE, Bruna MJ, Furth EF Binge eating disorder: a review. INTERNATIONAL JOURNAL OF OBESITY. 2002;26(3):299-307.

903. De Young Kyle, Lavender Jason, Crosby Ross, Wonderlich Stephen, Engel Scott, Mitchell James, Crow Scott, Peterson Carol, Le Grange Daniel Bidirectional associations between binge eating and restriction in anorexia nervosa. An ecological momentary assessment study. APPETITE. 2014;83():69-74.

904. Moghimi Elnaz, Davis Caroline, Bonder Revi, Knyahnytska Yuliya, Quilty Lena Exploring women's experiences of treatment for binge eating disorder: Methylphenidate vs. cognitive behavioural therapy. PROGRESS IN NEURO-PSYCHOPHARMACOLOGY \& BIOLOGICAL PSYCHIATRY. 2022;114():.

905. Khanna Shivam, Talwar Dhruv, Kumar Sunil, Madaan Sparsh, Goyal Aditi Bulimia Nervosa Leading to Squamous Cell Carcinoma of the Esophagus in a Young Adult. CUREUS.

2021;13(6):.

906. Woods Amanda, Racine Sarah, Klump Kelly Examining the relationship between dietary restraint and binge eating: Differential effects of major and minor stressors. EATING BEHAVIORS. 2010;11(4):276-280.

907. Bryant-Waugh Rachel Feeding and Eating Disorders in Children. PSYCHIATRIC CLINICS OF NORTH AMERICA. 2019;42(1, 1):157+.

908. Stice Eric, Van Ryzin Mark A Prospective Test of the Temporal Sequencing of Risk Factor Emergence in the Dual Pathway Model of Eating Disorders. JOURNAL OF ABNORMAL PSYCHOLOGY. 2019;128(2):119-128.

909. Rohde Paul, Stice Eric, Gau Jeff Predicting persistence of eating disorder compensatory weight control behaviors. INTERNATIONAL JOURNAL OF EATING DISORDERS. 2017;50(5):561-568.

910. Blomquist Kerstin, Grilo Carlos Family histories of anxiety in overweight men and women with binge eating disorder: A preliminary investigation. COMPREHENSIVE PSYCHIATRY. 2015;62():161-169.

911. Forney K., Schwendler Teresa, Ward Rose Examining similarities in eating pathology, negative affect, and perfectionism among peers: A social network analysis. APPETITE. 2019;137():236-243.

912. Rutkowska Magdalena, Czajkowska Mariola, Nowakowska Iwona, Kowalczyk Anna, Krol Tomasz, Dabrowska-Galas Magdalena, Skrzypulec-Plinta Violetta Eating Behaviours in Sportswomen from the Silesian Training in Different Sports Disciplines. INTERNATIONAL JOURNAL OF ENVIRONMENTAL RESEARCH AND PUBLIC HEALTH. 2022;19(24):.

913. Howard CE, Porzelius LK The role of dieting in binge eating disorder: Etiology and treatment implications. CLINICAL PSYCHOLOGY REVIEW. 1999;19(1):25-44.

914. Carter Frances, Jansen Anita Improving psychological treatment for obesity. Which eating behaviours should we target?. APPETITE. 2012;58(3):1063-1069.

915. McElroy Susan, Crow Scott, Blom Thomas, Biernacka Joanna, Winham Stacey, Geske Jennifer, Cuellar-Barboza Alfredo, Bobo William, Prieto Miguel, Veldic Marin, Mori Nicole, Seymour Lisa, Bond David, Frye Mark Prevalence and correlates of DSM-5 eating disorders in patients with bipolar disorder. JOURNAL OF AFFECTIVE DISORDERS. 2016;191():216-221.

916. Nutley Sara, Falise Alyssa, Henderson Rebecca, Apostolou Vasiliki, Mathews Carol, Striley Catherine Impact of the COVID-19 Pandemic on Disordered Eating Behavior: Qualitative Analysis of Social Media Posts. JMIR MENTAL HEALTH. 2021;8(1):.

917. Luz F., Sainsbury A., Mannan H., Touyz S., Mitchison D., Hay P. Prevalence of obesity and comorbid eating disorder behaviors in South Australia from 1995 to 2015. INTERNATIONAL JOURNAL OF OBESITY. 2017;41(7):1148-1153.

918. Carnier June, Lofrano Mara, Prado Wagner, Caranti Danielle, Piano Aline, Tock Lian, Nascimento Claudia, Oyama Lila, Mello Marco, Tufik Sergio, Damaso Ana Hormonal alteration in obese adolescents with eating disorder: Effects of multidisciplinary therapy. HORMONE RESEARCH. 2008;70(2):79-84.

919. STEEL ZP, FARAG PA, BLASZCZYNSKI AP INTERRUPTING THE BINGE-PURGE CYCLE IN BULIMIA - THE USE OF PLANNED BINGES. INTERNATIONAL JOURNAL OF EATING DISORDERS. 1995;18(3):199-208.

920. Stice Eric, Desjardins Christopher Interactions between risk factors in the prediction of onset of eating disorders: Exploratory hypothesis generating analyses. BEHAVIOUR RESEARCH AND THERAPY. 2018;105():52-62.

921. Zhang Zuo, Robinson Lauren, Jia Tianye, Quinlan Erin, Tay Nicole, Chu Congying, Barker Edward, Banaschewski Tobias, Barker Gareth, Bokde Arun, Flor Herta, Grigis Antoine, Garavan Hugh, Gowland Penny, Heinz Andreas, Ittermann Bernd, Martinot Jean-Luc, Stringaris Argyris, Penttila Jani, Noort Betteke, Grimmer Yvonne, Martinot Marie-Laure, Isensee Corinna, Becker Andreas, Nees Frauke, Orfanos Dimitri, Paus Tomas, Poustka Luise, Hohmann Sarah, Froehner Juliane, Smolka Michael, Walter Henrik, Whelan Robert, Schumann Gunter, Schmidt Ulrike, Desrivieres Sylvane Development of Disordered Eating Behaviors and Comorbid Depressive Symptoms in Adolescence: Neural and Psychopathological Predictors. BIOLOGICAL PSYCHIATRY. 2021;90(12):853-862.

922. Mond Jonathan, Arrighi Anais Perceived acceptability of anorexia and bulimia in women with and without eating disorder symptoms. AUSTRALIAN JOURNAL OF

PSYCHOLOGY. 2012;64(2):108-117.

923. Welch SL, Doll HA, Fairburn CG Life events and the onset of bulimia nervosa: A controlled study. PSYCHOLOGICAL MEDICINE. 1997;27(3):515-522.

924. Cooper Jane, Wade Tracey The Relationship Between Memory and Interpretation Biases, Difficulties with Emotion Regulation, and Disordered Eating in Young Women. COGNITIVE THERAPY AND RESEARCH. 2015;39(6):853-862.

925. Paans Nadine, Bot Mariska, Strien Tatjana, Brouwer Ingeborg, Visser Marjolein, Penninx Brenda Eating styles in major depressive disorder: Results from a large-scale study. JOURNAL OF PSYCHIATRIC RESEARCH. 2018;97():38-46.

926. Bentley Caroline, Monda Jonathan, Rodgers Bryan Sex differences in psychosocial impairment associated with eating-disordered behavior: What if there aren't any?. EATING BEHAVIORS. 2014;15(4):609-614.

927. Robinson Lauren, Zhang Zuo, Jia Tianye, Bobou Marina, Roach Anna, Campbell Iain, Irish Madeleine, Quinlan Erin, Tay Nicole, Barker Edward, Banaschewski Tobias, Bokde Arun, Grigis Antoine, Garavan Hugh, Heinz Andreas, Ittermann Bernd, Martinot Jean-Luc, Stringaris Argyris, Penttilae Jani, Noort Betteke, Grimmer Yvonne, Martinot Marie-Laure, Insensee Corinna, Becker Andreas, Nees Frauke, Orfanos Dimitri, Paus Tomas, Poustka Luise, Hohmann Sarah, Froehner Juliane, Smolka Michael, Walter Henrik, Whelan Robert, Schumann Gunter, Schmidt Ulrike, Desrivieres Sylvane, Consortium IMAGEN Association of Genetic and Phenotypic Assessments With Onset of Disordered Eating Behaviors and Comorbid Mental Health Problems Among Adolescents. JAMA NETWORK OPEN. 2020;3(12):.

928. Reas Deborah, Grilo Carlos Timing and sequence of the onset of overweight, dieting, and binge eating in overweight patients with binge eating disorder. INTERNATIONAL JOURNAL OF EATING DISORDERS. 2007;40(2):165-170.

929. Steinhausen Hans-Christoph Outcome of Eating Disorders. CHILD AND ADOLESCENT PSYCHIATRIC CLINICS OF NORTH AMERICA. 2009;18(1):225+.

930. Scott Charlotte, Haycraft Emma, Plateau Carolyn The influence of social networks within sports teams on athletes' eating and exercise psychopathology: A longitudinal study.

PSYCHOLOGY OF SPORT AND EXERCISE. 2021;52():.

931. Mutiso Victoria, Ndetei David, Muia Esther, Alietsi Rita, Onsinyo Lydia, Kameti Frida, Masake Monicah, Musyimi Christine, Mamah Daniel The prevalance of binge eating disorder and associated psychiatric and substance use disorders in a student population in Kenya - towards a public health approach. BMC PSYCHIATRY. 2022;22(1):.

932. Thompson-Brenner Heather Relationship-Focused Therapy for Bulimia and Binge Eating: Introduction to the Special Section. PSYCHOTHERAPY. 2016;53(2):185-187.

933. Danner Unna, Sternheim Lot, Oppen Patricia, Hendriks Gert-Jan, Balkom Ton, Cath Danielle The relationship between eating disorders and OCD symptom dimensions: An explorative study in a large sample of patients with OCD. JOURNAL OF OBSESSIVE-COMPULSIVE AND RELATED DISORDERS. 2022;35():.

934. Fichter MM, Kruger R, Rief W, Holland R, Dohne J Fluvoxamine in prevention of relapse in bulimia nervosa: Effects on eating-specific psychopathology. JOURNAL OF CLINICAL PSYCHOPHARMACOLOGY. 1996;16(1):9-18.

935. Field Alison, Inge Thomas, Belle Steven, Johnson Geoffrey, Wahed Abdus, Pories Walter, Spaniolas Konstantinos, Mitchell James, Pomp Alfons, Dakin Gregory, Wolfe Bruce, Courcoulas Anita Association of Obesity Subtypes in the Longitudinal Assessment of Bariatric Surgery Study and 3-Year Postoperative Weight Change. OBESITY. 2018;26(12):1931-1937.

936. Takii M, Komaki G, Uchigata Y, Maeda M, Omori Y, Kubo C Differences between bulimia nervosa and binge-eating disorder in females with type 1 diabetes: The important role of insulin omission. JOURNAL OF PSYCHOSOMATIC RESEARCH. 1999;47(3):221-231.

937. Grissett NI, Fitzgibbon ML The clinical significance of binge eating in an obese population: Support for BED and questions regarding its criteria. ADDICTIVE BEHAVIORS. 1996;21(1):57-66.

938. Wiseman CV, Sunday SR, Halligan P, Korn S, Brown C, Halmi KA Substance dependence and eating disorders: Impact of sequence on comorbidity. COMPREHENSIVE PSYCHIATRY. 1999;40(5):332-336.

939. Craven Michael, Fekete Erin Weight-related shame and guilt, intuitive eating, and binge eating in female college students. EATING BEHAVIORS. 2019;33():44-48.

940. Benjet Corina, Mendez Enrique, Borges Guilherme, Elena Medina-Moral Maria Epidemiology of eating disorders in a representative sample of adolescents. SALUD MENTAL. 2012;35(6):483-490.

941. NEUMARKSZTAINER D, STORY M, RESNICK MD, GARWICK A, BLUM RW BODY DISSATISFACTION AND UNHEALTHY WEIGHT-CONTROL PRACTICES AMONG ADOLESCENTS WITH AND WITHOUT CHRONIC ILLNESS - A POPULATION-BASED STUDY. ARCHIVES OF PEDIATRICS \& ADOLESCENT MEDICINE. 1995;149(12):1330-1335.

942. Bakalar Jennifer, Shank Lisa, Vannucci Anna, Radin Rachel, Tanofsky-Kraff Marian Recent Advances in Developmental and Risk Factor Research on Eating Disorders. CURRENT PSYCHIATRY REPORTS. 2015;17(6):.

943. Pike Kathleen, Wilfley Denise, Hilbert Anja, Fairburn Christopher, Dohm Faith-Anne, Striegel-Moore Ruth Antecedent life events of binge-eating disorder. PSYCHIATRY RESEARCH. 2006;142(1):19-29.

944. MUSSELL MP, MITCHELL JE, WELLER CL, RAYMOND NC, CROW SJ, CROSBY RD ONSET OF BINGE-EATING, DIETING, OBESITY, AND MOOD DISORDERS AMONG SUBJECTS SEEKING TREATMENT FOR BINGE-EATING DISORDER. INTERNATIONAL JOURNAL OF EATING DISORDERS. 1995;17(4):395-401.

945. Lowe Michael, Steenburgh Jason, Ochner Christopher, Coletta Maria Neural correlates of individual differences related to appetite. PHYSIOLOGY \& BEHAVIOR. 2009;97(5, SI):561-571.

946. Wade TD, Bergin JL, Tiggemann M, Bulik CM, Fairburn CG Prevalence and long-term course of lifetime eating disorders in an adult Australian twin cohort. AUSTRALIAN AND NEW ZEALAND JOURNAL OF PSYCHIATRY. 2006;40(2):121-128.

947. Ackard Diann, Fulkerson Jayne, Neumark-Sztainer Dianne Psychological and Behavioral Risk Profiles as they Relate to Eating Disorder Diagnoses and Symptomatology among a School-Based Sample of Youth. INTERNATIONAL JOURNAL OF EATING DISORDERS. 2011;44(5):440-446.

948. Abbott DW, Zwaan M, Mussell MP, Raymond NC, Seim HC, Crow SJ, Crosby RD, Mitchell JE Onset of binge eating and dieting in overweight women: Implications for etiology, associated features and treatment. JOURNAL OF PSYCHOSOMATIC RESEARCH. 1998;44(3-4):367-374.

949. Wildman P, Lilenfeld LRR, Marcus MD Axis I comorbidity onset and parasuicide in women with eating disorders. INTERNATIONAL JOURNAL OF EATING DISORDERS. 2004;35(2):190-197.

950. Leme Ana, Philippi Sonia, Thompson Debbe, Nicklas Theresa, Baranowski Tom ``Healthy Habits, Healthy Girls-Brazil{''}: an obesity prevention program with added focus on eating disorders. EATING AND WEIGHT DISORDERS-STUDIES ON ANOREXIA BULIMIA AND OBESITY. 2019;24(1):107-119.

951. Berner Laura, Winter Samantha, Matheson Brittany, Benson Leora, Lowe Michael Behind binge eating: A review of food-specific adaptations of neurocognitive and neuroimaging tasks. PHYSIOLOGY \& BEHAVIOR. 2017;176(SI):59-70.

952. FORNARI V, KENT J, KABO L, GOODMAN B ANOREXIA-NERVOSA - 30 SOMETHING. JOURNAL OF SUBSTANCE ABUSE TREATMENT. 1994;11(1):45-54.

953. Muzi Laura, Tieghi Laura, Franco Anna, Rugo Michele, Lingiardi Vittorio The Mediator Effect of Personality on the Relationship Between Symptomatic Impairment and Treatment Outcome in Eating Disorders. FRONTIERS IN PSYCHOLOGY. 2021;12():.

954. Jimerson DC, Wolfe BE, Metzger ED, Finkelstein DM, Cooper TB, Levine JM Decreased serotonin function in bulimia nervosa. ARCHIVES OF GENERAL PSYCHIATRY. 1997;54(6):529-534.

955. Kelly Nichole, Shank Lisa, Bakalar Jennifer, Tanofsky-Kraff Marian Pediatric Feeding and Eating Disorders: Current State of Diagnosis and Treatment. CURRENT PSYCHIATRY REPORTS. 2014;16(5):.

956. Scharmer Christina, Gorrell Sasha, Schaumberg Katherine, Anderson Drew Compulsive exercise or exercise dependence? Clarifying conceptualizations of exercise in the context of eating disorder pathology. PSYCHOLOGY OF SPORT AND EXERCISE. 2020;46():.

957. Lee Tien-Jui, Kinzig Kimberly Repeated adolescent activity-based anorexia influences central estrogen signaling and adulthood anxiety-like behaviors in rats. PHYSIOLOGY \& BEHAVIOR. 2017;171():199-206.

958. Berg Cecilie, Torgersen Leila, Von Holle Ann, Hamer Robert, Bulik Cynthia, Reichborn-Kjennerud Ted Factors Associated with Binge Eating Disorder in Pregnancy. INTERNATIONAL JOURNAL OF EATING DISORDERS. 2011;44(2):124-133.

959. Knightsmith Pooky, Treasure Janet, Schmidt Ulrike We don't know how to help: an online survey of school staff. CHILD AND ADOLESCENT MENTAL HEALTH. 2014;19(3):208-214.

960. Bundros Joanna, Clifford Dawn, Silliman Kathryn, Morris Michelle Prevalence of Orthorexia nervosa among college students based on Bratman's test and associated tendencies. APPETITE. 2016;101():86-94.

961. Fu Emily, Neubert Sarah, Chang Angela, Smith J., Graham Andrea Characterizing behavior change techniques used in the self-management of binge eating and weight: Applying a user-centered design approach. EATING BEHAVIORS. 2022;44():.

962. Allen Karina, Byrne Susan, Crosby Ross, Stice Eric Testing for interactive and non-linear effects of risk factors for binge eating and purging eating disorders. BEHAVIOUR RESEARCH AND THERAPY. 2016;87():40-47.

963. Preti Antonio, Girolamo Giovanni, Vilagut Gemma, Alonso Jordi, Graaf Ron, Bruffaerts Ronny, Demyttenaere Koen, Pinto-Meza Alejandra, Haro Josep, Morosini Piero, Investigators ESEMeD-WMH The epidemiology of eating disorders in six European countries: Results of the ESEMeD-WMH project. JOURNAL OF PSYCHIATRIC RESEARCH. 2009;43(14):1125-1132.

964. Strober M, Freeman R, Bower S, Rigali J Binge eating in anorexia nervosa predicts later onset of substance use disorder: A ten-year prospective, longitudinal follow-up of 95 adolescents. JOURNAL OF YOUTH AND ADOLESCENCE. 1996;25(4):519-532.

965. Day Jemma, Schmidt Ulrike, Collier David, Perkins Sarah, Eynde Frederique, Treasure Janet, Yi Irene, Winn Suzanne, Robinson Paul, Murphy Rebecca, Keville Saskia, Johnson-Sabine Eric, Jenkins Mari, Frost Susie, Dodge Liz, Berelowitz Mark, Eisler Ivan Risk Factors, Correlates, and Markers in Early-Onset Bulimia Nervosa and EDNOS. INTERNATIONAL

JOURNAL OF EATING DISORDERS. 2011;44(4):287-294.

966. Yilmaz Zeynep, Quattlebaum Mary, Pawar Pratiksha, Thornton Laura, Bulik Cynthia, Javaras Kristin, Yao Shuyang, Lichtenstein Paul, Larsson Henrik, Baker Jessica Associations Between Attention Deficit Hyperactivity Disorder Symptom Dimensions and Disordered Eating Symptoms in Adolescence: A Population-Based Twin Study. BEHAVIOR GENETICS. 2023;53(2):143-153.

967. Silen Yasmina, Keski-Rahkonen Anna Worldwide prevalence of DSM-5 eating disorders among young people. CURRENT OPINION IN PSYCHIATRY. 2022;35(6):362-371.

968. Cifani Carlo, Polidori Carlo, Melotto Sergio, Ciccocioppo Roberto, Massi Maurizio A preclinical model of binge eating elicited by yo-yo dieting and stressful exposure to food: effect of sibutramine, fluoxetine, topiramate, and midazolam. PSYCHOPHARMACOLOGY. 2009;204(1):113-125.

969. Bohon Cara Binge Eating Disorder in Children and Adolescents. CHILD AND ADOLESCENT PSYCHIATRIC CLINICS OF NORTH AMERICA. 2019;28(4):549+.

970. Karjalainen Louise, Gillberg Christopher, Rastam Maria, Wentz Elisabet Eating disorders and eating pathology in young adult and adult patients with ESSENCE. COMPREHENSIVE PSYCHIATRY. 2016;66():79-86.

971. Baltruschat N, Geissner E, Bents H Exposure in the treatment of bulimia nervosa: Evaluation of an intensive cognitive-behavioral inpatient treatment approach. ZEITSCHRIFT FUR KLINISCHE PSYCHOLOGIE UND PSYCHOTHERAPIE. 2006;35(1):1-11.

972. House Eve, Gow Megan, Lister Natalie, Baur Louise, Garnett Sarah, Paxton Susan, Jebeile Hiba Pediatric weight management, dietary restraint, dieting, and eating disorder risk: a systematic review. NUTRITION REVIEWS. 2021;79(10):1114-1133.

973. Engler Patricia, Crowther Janis, Dalton Ginnie, Sanftner Jennifer Predicting eating disorder group membership: An examination and extension of the sociocultural model. BEHAVIOR THERAPY. 2006;37(1):69-79.

974. Churruca Kate, Ussher Jane, Perz Janette Just Desserts? Exploring Constructions of Food in Women's Experiences of Bulimia. QUALITATIVE HEALTH RESEARCH. 2017;27(10,

SI):1491-1506.

975. Bulik CM, Klump KL, Thornton L, Kaplan AS, Devlin B, Fichter MM, Halmi KA, Strober M, Woodside B, Crow S, Mitchell JE, Rotondo A, Mauri M, Cassano GB, Keel PK, Berrettini WH, Kaye WH Alcohol use disorder comorbidity in eating disorders: A multicenter study. JOURNAL OF CLINICAL PSYCHIATRY. 2004;65(7):1000-1006.

976. Ferrer-Garcia Marta, Gutierrez-Maldonado Jose, Pla-Sanjuanelo Joana, Vilalta-Abella Ferran, Riva Giuseppe, Clerici Massimo, Ribas-Sabate Joan, Andreu-Gracia Alexis, Fernandez-Aranda Fernando, Forcano Laura, Riesco Nadine, Sanchez Isabel, Escandon-Nagel Neli, Gomez-Tricio Osane, Tena Virginia, Dakanalis Antonios A Randomised Controlled Comparison of Second-Level Treatment Approaches for Treatment-Resistant Adults with Bulimia Nervosa and Binge Eating Disorder: Assessing the Benefits of Virtual Reality Cue Exposure Therapy. EUROPEAN EATING DISORDERS REVIEW. 2017;25(6):479-490.

977. Reyes-Rodriguez Mae, Gulisano Monica, Silva Yormeri, Pivarunas Bernadette, Luna-Reyes Kiara, Bulik Cynthia ``Las penas con pan duelen menos{''}: The role Latinas with disordered eating behaviors. APPETITE. 2016;100():102-109.

978. Grogan Katie, MacGarry Diarmuid, Bramham Jessica, Scriven Mary, Maher Caroline, Fitzgerald Amanda Family-related non-abuse adverse life experiences occurring for adults diagnosed with eating disorders: a systematic review. JOURNAL OF EATING DISORDERS. 2020;8(1):.

979. Osoro Andrea, Villalobos Dolores, Antonio Tamayo Jose Efficacy of emotion-focused therapy in the treatment of eating disorders: A systematic review. CLINICAL PSYCHOLOGY \& PSYCHOTHERAPY. 2022;29(3):815-836.

980. Mehr Jacqueline, Mitchison Deborah, Bowrey Hannah, James Morgan Sleep dysregulation in binge eating disorder and ``food addiction{''}: the orexin (hypocretin) system as a potential neurobiological link. NEUROPSYCHOPHARMACOLOGY. 2021;46(12):2051-2061.

981. Wilfley DE, Cohen LR Psychological treatment of bulimia nervosa and binge eating disorder. PSYCHOPHARMACOLOGY BULLETIN. 1997;33(3):437-454.

982. Conceicao Eva, Mitchell James, Vaz Ana, Bastos Ana, Ramalho Sofia, Silva Catia, Cao Li, Brandao Isabel, Machado Paulo The presence of maladaptive eating behaviors after bariatric surgery in a cross sectional study: Importance of picking or nibbling on weight regain. EATING BEHAVIORS. 2014;15(4):558-562.

983. Lo Sauro Carolina, Ravaldi Claudia, Cabras Pier, Faravelli Carlo, Ricca Valdo Stress, hypothalamic-pituitary-adrenal axis and eating disorders. NEUROPSYCHOBIOLOGY. 2008;57(3):95-115.

984. Tanofsky-Kraff Marian Psychosocial preventive interventions for obesity and eating disorders in youths. INTERNATIONAL REVIEW OF PSYCHIATRY. 2012;24(3):262-270.

985. Hay Phillipa, Bacaltchuk Josue, Stefano Sergio, Kashyap Priyanka Psychological treatments for bulimia nervosa and binging. COCHRANE DATABASE OF SYSTEMATIC REVIEWS. 2009;(4):.

986. Slane Jennifer, Burt S., Klump Kelly Genetic and Environmental Influences on Disordered Eating and Depressive Symptoms. INTERNATIONAL JOURNAL OF EATING DISORDERS. 2011;44(7):605-611.

987. Neumark-Sztainer D, Wall MM, Story M, Perry CL Correlates of unhealthy weight-control behaviors among adolescents: Implications for prevention programs. HEALTH PSYCHOLOGY. 2003;22(1):88-98.

988. Swinbourne Jessica, Hunt Caroline, Abbott Maree, Russell Janice, St Clare Tamsen, Touyz Stephen The comorbidity between eating disorders and anxiety disorders: Prevalence in an eating disorder sample and anxiety disorder sample. AUSTRALIAN AND NEW ZEALAND JOURNAL OF PSYCHIATRY. 2012;46(2):118-131.

989. Manwaring JL, Hilbert A, Wilfley DE, Pike KM, Fairburn CG, Dohm FA, Striegel-Moore RH Risk factors and patterns of onset in binge eating disorder. INTERNATIONAL JOURNAL OF EATING DISORDERS. 2006;39(2):101-107.

990. Borges MBF, Jorge MR, Morgan CM, Silveira DX, Custodio O Binge-eating disorder in Brazilian women on a weight-loss program. OBESITY RESEARCH. 2002;10(11):1127-1134.

991. Kessler R., Shahly V., Hudson J., Supina D., Berglund P., Chiu W., Gruber M., Aguilar-Gaxiola S., Alonso J., Andrade L., Benjet C., Bruffaerts R., Girolamo G., Graaf R., Florescu S., Haro J., Murphy S., Posada-Villa J., Scott K., Xavier M. A comparative analysis of role attainment and impairment in binge-eating disorder and bulimia nervosa: results from the WHO World Mental Health Surveys. EPIDEMIOLOGY AND PSYCHIATRIC SCIENCES. 2014;23(1):27-41.

992. Lopez-Guimera Gemma, Neumark-Sztainer Dianne, Hannan Peter, Fauquet Jordi, Loth Katie, Sanchez-Carracedo David Unhealthy Weight-control Behaviours, Dieting and Weight Status: A Cross-cultural Comparison between North American and Spanish Adolescents. EUROPEAN EATING DISORDERS REVIEW. 2013;21(4):276-283.

993. Pokrajac-Bulian Alessandra, Tkalcic Mladenka, Kardum Igor, Sajina Sarlota, Kukic Miljana Perfectionism, Private Self-Consciousness, Negative Affect and Avoidance as Determinants of Binge Eating. DRUSTVENA ISTRAZIVANJA. 2009;18(1-2):111-128.

994. Larsen Pernille, Andersen Anne-Marie, Olsen Else, Andersen Per, Micali Nadia, Strandberg-Larsen Katrine Weight trajectories and disordered eating behaviours in 11-to 12-year-olds: A longitudinal study within the Danish National Birth Cohort. EUROPEAN EATING DISORDERS REVIEW. 2019;27(4):436-444.

995. Treasure Janet, Bektas Sevgi, Mutwalli Hiba, Dhopatkar Namrata, Himmerich Hubertus Novel approaches to tackling emotional loss of control of eating across the weight spectrum. PROCEEDINGS OF THE NUTRITION SOCIETY. 2022;():.

996. Sala Margarita, Levinson Cheri The longitudinal relationship between worry and disordered eating: Is worry a precursor or consequence of disordered eating?. EATING BEHAVIORS. 2016;23():28-32.

997. Friend Sarah, Bauer Katherine, Madden Tracy, Neumark-Sztainer Dianne Self-Weighing among Adolescents: Associations with Body Mass Index, Body Satisfaction, Weight Control Behaviors, and Binge Eating. JOURNAL OF THE ACADEMY OF NUTRITION AND DIETETICS. 2012;112(1):99-103.

998. Warschburger Petra, Zitzmann Jana The Efficacy of a Universal School-Based Prevention Program for Eating Disorders among German Adolescents: Results from a Randomized-Controlled Trial. JOURNAL OF YOUTH AND ADOLESCENCE. 2018;47(6, SI):1317-1331.

999. Tanis Jorg, Vroling Maartje, Martijn Carolien, Heijningen Leila, Maas Joyce, Keijsers Ger Effects of implementation intentions on subthreshold binge eating. EATING DISORDERS. 2022;30(4):370-384.

1000. Hsu LKG Epidemiology of the eating disorders. PSYCHIATRIC CLINICS OF NORTH AMERICA. 1996;19(4):681+.

1001. Melles Hanna, Jansen Anita Transdiagnostic fears and avoidance behaviors in self-reported eating disorders. JOURNAL OF EATING DISORDERS. 2023;11(1):.

1002. Striegel-Moore RH, Franko DL, Thompson D, Barton B, Schreiber GB, Daniels SR Changes in weight and body image over time in women with eating disorders. INTERNATIONAL JOURNAL OF EATING DISORDERS. 2004;36(3):315-327.

1003. Ricca Valdo, Castellini Giovanni, Mannucci Edoardo, Lo Sauro Carolina, Ravaldi Claudia, Rotella Carlo, Faravelli Carlo Comparison of individual and group cognitive behavioral therapy for binge eating disorder. A randomized, three-year follow-up study. APPETITE. 2010;55(3):656-665.

1004. Moreno Silvia, Warren Cortney, Rodriguez Sonia, Carmen Fernandez M., Cepeda-Benito Antonio Food cravings discriminate between anorexia and bulimia nervosa. Implications for ``success{''} versus ``failure{''} in dietary restriction. APPETITE. 2009;52(3):588-594.

1005. Marzilli Eleonora, Cerniglia Luca, Cimino Silvia A narrative review of binge eating disorder in adolescence: prevalence, impact, and psychological treatment strategies. ADOLESCENT HEALTH MEDICINE AND THERAPEUTICS. 2018;9():17-30.

1006. Whitehead Jacqui, Slater Gary, Wright Hattie, Martin Lisa, O'Connor Helen, Mitchell Lachlan Disordered eating behaviours in female physique athletes. EUROPEAN JOURNAL OF SPORT SCIENCE. 2020;20(9):1206-1214.

1007. Treasure Janet, Stein Daniel, Maguire Sarah Has the time come for a staging model to map the course of eating disorders from high risk to severe enduring illness? An examination of the evidence. EARLY INTERVENTION IN PSYCHIATRY. 2015;9(3):173-184.

1008. Verri A, Nappi RE, Cecchini AP, Vallero E, Luzi S, Zara C Eating disorders and Axis I psychiatric comorbidity in amenorrheic women. INTERNATIONAL JOURNAL OF EATING DISORDERS. 1998;24(2):137-146.

1009. Mitchison Deborah, Bussey Kay, Touyz Stephen, Gonzalez-Chica David, Musker Michael, Stocks Nigel, Licinio Julio, Hay Phillipa Shared associations between histories of victimisation among people with eating disorder symptoms and higher weight. AUSTRALIAN AND NEW ZEALAND JOURNAL OF PSYCHIATRY. 2019;53(6):540-549.

1010. Kim Eun, Ebesutani Chad, Wall David, Olatunji Bunmi Depression mediates the relationship between obsessive-compulsive symptoms and eating disorder symptoms in an inpatient sample. JOURNAL OF OBSESSIVE-COMPULSIVE AND RELATED DISORDERS. 2012;1(1):62-68.

1011. Backe J The prevalence of eating disorders in patients of a primary care gynaecologist. GEBURTSHILFE UND FRAUENHEILKUNDE. 2001;61(6):408-413.

1012. Kroshus Emily, Kubzansky Laura, Goldman Roberta, Austin S. Anti-Dieting Advice From Teammates: A Pilot Study of the Experience of Female Collegiate Cross Country Runners. EATING DISORDERS. 2015;23(1):31-44.

1013. Safer DL, Lively TJ, Telch CF, Agras WS Predictors of relapse following successful dialectical behavior therapy for binge eating disorder. INTERNATIONAL JOURNAL OF EATING DISORDERS. 2002;32(2):155-163.

1014. Dahl J., Eriksen L., Vedul-Kjelsas E., Strommen M., Kulseng B., Marvik R., Holen A. Prevalence of all relevant eating disorders in patients waiting for bariatric surgery: A comparison between patients with and without eating disorders. EATING AND WEIGHT DISORDERS-STUDIES ON ANOREXIA BULIMIA AND OBESITY. 2010;15(4):E247-E255.

1015. Lydecker Janet, Grilo Carlos Comparing men and women with binge-eating disorder and co-morbid obesity. INTERNATIONAL JOURNAL OF EATING DISORDERS. 2018;51(5):411-417.

1016. Cusack Claire, Christian Caroline, Drake Jordan, Levinson Cheri A network analysis of eating disorder symptoms and co-occurring alcohol misuse among heterosexual and sexual minority college women. ADDICTIVE BEHAVIORS. 2021;118():.

1017. Sahlan Reza, Taravatrooy Fatemeh, Quick Virginia, Mond Jonathan Eating-disordered behavior among male and female college students in Iran. EATING BEHAVIORS. 2020;37():.

1018. Drutschinin Katherine, Fuller-Tyszkiewicz Matthew, De Paoli Tara, Lewis Vivienne, Krug Isabel The Daily Frequency, Type, and Effects of Appearance Comparisons on Disordered Eating. PSYCHOLOGY OF WOMEN QUARTERLY. 2018;42(2):151-161.

1019. Stice Eric, Bohon Cara, Gau Jeff, Rohde Paul Factors that predict persistence versus non-persistence of eating disorder Symptoms: A prospective study of high-risk young women{*}. BEHAVIOUR RESEARCH AND THERAPY. 2021;144():.

1020. Swinbourne Jessica, Touyz Stephen The co-morbidity of eating disorders and anxiety disorders: A review. EUROPEAN EATING DISORDERS REVIEW. 2007;15(4):253-274.

1021. Castelao-Naval Olga, Blanco-Fernandez Ascension, Marina Meseguer-Barros Carmen, Thuissard-Vasallo Israel, Cerda Begona, Larrosa Mar Life style and risk of atypical eating disorders in university students: Reality versus perception. ENFERMERIA CLINICA. 2019;29(5):280-290.

1022. Milano Walter, Capasso Anna Neuroendocrine and Metabolic Disorders in Bulimia Nervosa. ENDOCRINE METABOLIC \& IMMUNE DISORDERS-DRUG TARGETS. 2018;18(4):297-305.

1023. RASTAM M, GILLBERG IC, GILLBERG C ANOREXIA-NERVOSA 6 YEARS AFTER ONSET .2. COMORBID PSYCHIATRIC-PROBLEMS. COMPREHENSIVE PSYCHIATRY. 1995;36(1):70-76.

1024. Gendall KA, Joyce PR, Carter FA, McIntosh VV, Bulik CM Childhood gastrointestinal complaints in women with bulimia nervosa. INTERNATIONAL JOURNAL OF EATING DISORDERS. 2005;37(3):256-260.

1025. Kessler Ronald, Berglund Patricia, Chiu Wai, Deitz Anne, Hudson James, Shahly Victoria, Aguilar-Gaxiola Sergio, Alonso Jordi, Angermeyer Matthias, Benjet Corina, Bruffaerts Ronny, Girolamo Giovanni, Graaf Ron, Maria Haro Josep, Kovess-Masfety Viviane, O'Neill Siobhan, Posada-Villa Jose, Sasu Carmen, Scott Kate, Viana Maria, Xavier Miguel The Prevalence and Correlates of Binge Eating Disorder in the World Health Organization World Mental Health Surveys. BIOLOGICAL PSYCHIATRY. 2013;73(9):904-914.

1026. Hubel Christopher, Marzi Sarah, Bernn Gerome, Bulik Cynthia Epigenetics in eating disorders: a systematic review. MOLECULAR PSYCHIATRY. 2019;24(6):901-915.

1027. Hilbert Anja Cognitive-behavioral therapy for binge eating disorder in adolescents: study protocol for a randomized controlled trial. TRIALS. 2013;14():.

1028. Garcia-Garcia Isabel, Morys Filip, Michaud Andreanne, Dagher Alain Food Addiction, Skating on Thin Ice: a Critical Overview of Neuroimaging Findings. CURRENT ADDICTION REPORTS. 2020;7(1):20-29.

1029. Silen Yasmina, Sipila Pyry, Raevuori Anu, Mustelin Linda, Marttunen Mauri, Kaprio Jaakko, Keski-Rahkonen Anna DSM-5 eating disorders among adolescents and young adults in Finland: A public health concern. INTERNATIONAL JOURNAL OF EATING DISORDERS. 2020;53(5):520-531.

1030. Keshen Aaron, Hilbert Anja, Taylor Victoria, Harris Anastasia, Trappenberg Nami, Sadek Joseph, Frank Guido, Murray Stuart Effect of stimulant medication on loss of control eating in youth with attention deficit/hyperactivity disorder: a prospective, observational case series study protocol. JOURNAL OF EATING DISORDERS. 2022;10(1):.

1031. Papathomas A, Lavallee D A life history analysis of a male athlete with an eating disorder. JOURNAL OF LOSS \& TRAUMA. 2006;11(2):143-179.

1032. Braun Tosca, Park Crystal, Gorin Amy Self-compassion, body image, and disordered eating: A review of the literature. BODY IMAGE. 2016;17():117-131.

1033. Vickers Steven, Goddard Simon, Brammer Richard, Hutson Peter, Heal David Investigation of impulsivity in binge-eating rats in a delay-discounting task and its prevention by the d-amphetamine prodrug, lisdexamfetamine. JOURNAL OF PSYCHOPHARMACOLOGY. 2017;31(6):784-797.

1034. Bush Hannah, Rossy Lynn, Mintz Laurie, Schopp Laura Eat for Life: A Work Site Feasibility Study of a Novel Mindfulness-Based Intuitive Eating Intervention. AMERICAN JOURNAL OF HEALTH PROMOTION. 2014;28(6):380-388.

1035. Davidson Karina, Barry Michael, Mangione Carol, Cabana Michael, Chelmow David, Coker Tumaini, Davis Esa, Donahue Katrina, Jaen Carlos, Kubik Martha, Li Li, Ogedegbe

Gbenga, Pbert Lori, Ruiz John, Silverstein Michael, Stevermer James, Wong John, Force US Screening for Eating Disorders in Adolescents and Adults US Preventive Services Task Force Recommendation Statement. JAMA-JOURNAL OF THE AMERICAN MEDICAL ASSOCIATION. 2022;327(11):1061-1067.

1036. Van Alsten Sarah, Duncan Alexis Lifetime patterns of comorbidity in eating disorders: An approach using sequence analysis. EUROPEAN EATING DISORDERS REVIEW. 2020;28(6):709-723.

1037. Rosen JC Body image assessment and treatment in controlled studies of eating disorders. INTERNATIONAL JOURNAL OF EATING DISORDERS. 1996;20(4):331-343.

1038. Ricca V, Mannucci E, Zucchi T, Rotella CM, Faravelli C Cognitive-behavioural therapy for bulimia nervosa and binge eating disorder - A review. PSYCHOTHERAPY AND PSYCHOSOMATICS. 2000;69(6):287-295.

1039. Jauregui-Lobera Ignacio Neuroimaging in eating disorders. NEUROPSYCHIATRIC DISEASE AND TREATMENT. 2011;7():577-584.

1040. Goldschmidt Andrea, Wall Melanie, Loth Katie, Le Grange Daniel, Neumark-Sztainer Dianne Which Dieters Are at Risk for the Onset of Binge Eating? A Prospective Study of Adolescents and Young Adults. JOURNAL OF ADOLESCENT HEALTH. 2012;51(1):86-92.

1041. Bleck Jennifer, DeBate Rita Exploring the co-morbidity of attention-deficit/hyperactivity disorder with eating disorders and disordered eating behaviors in a nationally representative community-based sample. EATING BEHAVIORS. 2013;14(3):390-393.

1042. Thomsen Kristine, Callesen Mette, Hesse Morten, Kvamme Timo, Pedersen Michael, Pedersen Mads, Voon Valerie Impulsivity traits and addiction-related behaviors in youth. JOURNAL OF BEHAVIORAL ADDICTIONS. 2018;7(2):317-330.

1043. Bodell Lindsay, Keel Pamela Weight Suppression in Bulimia Nervosa: Associations With Biology and Behavior. JOURNAL OF ABNORMAL PSYCHOLOGY. 2015;124(4):994-1002.

1044. Dorard G., Khorramian-Pour M. Binge eating disorder: Links with personality and emotionality. ENCEPHALE-REVUE DE PSYCHIATRIE CLINIQUE BIOLOGIQUE ET THERAPEUTIQUE. 2017;43(2):114-119.

1045. Kass Andrea, Wang Annie, Kolko Rachel, Holland Jodi, Altman Myra, Trockel Mickey, Taylor C., Wilfley Denise Identification as overweight by medical professionals: Relation to eating disorder diagnosis and risk. EATING BEHAVIORS. 2015;17():62-68.

1046. Micali Nadia, Martini Maria, Thomas Jennifer, Eddy Kamryn, Kothari Radha, Russell Ellie, Bulik Cynthia, Treasure Janet Lifetime and 12-month prevalence of eating disorders amongst women in mid-life: a population-based study of diagnoses and risk factors. BMC MEDICINE. 2017;15():.

1047. Berner Laura, Marsh Rachel Frontostriatal circuits and the development of bulimia nervosa. FRONTIERS IN BEHAVIORAL NEUROSCIENCE. 2014;8():.

1048. Stice Eric, Marti C., Shaw Heather, Jaconis Maryanne An 8-Year Longitudinal Study of the Natural History of Threshold, Subthreshold, and Partial Eating Disorders From a Community Sample of Adolescents. JOURNAL OF ABNORMAL PSYCHOLOGY. 2009;118(3):587-597.

1049. Luz F., Hay P., Gibson A., Touyz S., Swinbourne J., Roekenes J., Sainsbury A. Does severe dietary energy restriction increase binge eating in overweight or obese individuals? A systematic review. OBESITY REVIEWS. 2015;16(8):652-665.

1050. Okumus F., Berk H., Yucel Basak Body image, depression and eating behaviour: a comparative study in eating disordered women and healthy controls. PSYCHIATRY AND CLINICAL PSYCHOPHARMACOLOGY. 2019;29(4):774-780.

1051. Kim Sun, Kim Seong, Sun Kyung, Park Yongjin A Case of Gastric Atony on Bulimia Nervosa. IRANIAN RED CRESCENT MEDICAL JOURNAL. 2017;19(10):.

1052. Lumley Mark, Neely Lynn, Burger Amanda The assessment of alexithymia in medical settings: Implications for understanding and treating health problems. JOURNAL OF PERSONALITY ASSESSMENT. 2007;89(3):230-246.

1053. Goethals Ingeborg, Vervaet Myriam, Audenaert Kurt., JacobS Hip, Ham Hamphrey, Van Heeringen Cees Does regional brain perfusion correlate with eating disorder symptoms in anorexia and bulimia nervosa patients?. JOURNAL OF PSYCHIATRIC RESEARCH. 2007;41(12):1005-1011.

1054. Raymond NC, Zwaan M, Mitchell JE, Ackard D, Thuras P Effect of a very low calorie diet on the diagnostic category of individuals with binge eating disorder. INTERNATIONAL JOURNAL OF EATING DISORDERS. 2002;31(1):49-56.

1055. Root T., Pisetsky E., Thornton L., Lichtenstein P., Pedersen N., Bulik C. Patterns of co-morbidity of eating disorders and substance use in Swedish females. PSYCHOLOGICAL MEDICINE. 2010;40(1):105-115.

1056. Baker Jessica, Peterson Claire, Thornton Laura, Brownley Kimberly, Bulik Cynthia, Girdler Susan, Marcus Marsha, Bromberger Joyce Reproductive and Appetite Hormones and Bulimic Symptoms during Midlife. EUROPEAN EATING DISORDERS REVIEW. 2017;25(3):188-194.

1057. Lampard Amy, Byrne Susan, McLean Neil, Fursland Anthea An evaluation of the enhanced cognitive-behavioural model of bulimia nervosa. BEHAVIOUR RESEARCH AND THERAPY. 2011;49(9):529-535.

1058. Ackard DM, Croll JK, Kearney-Cooke A Dieting frequency among college females: Association with disordered eating, body image, and related psychological problems. JOURNAL OF PSYCHOSOMATIC RESEARCH. 2002;52(3):129-136.

1059. Sinclair-McBride Keneisha, Cole David Prospective Relations between Overeating, Loss of Control Eating, Binge Eating, and Depressive Symptoms in a School-Based Sample of Adolescents. JOURNAL OF ABNORMAL CHILD PSYCHOLOGY. 2017;45(4):693-703.

1060. Keel Pamela, Forney K., Brown Tiffany, Heatherton Todd Influence of College Peers on Disordered Eating in Women and Men at 10-Year Follow-Up. JOURNAL OF ABNORMAL PSYCHOLOGY. 2013;122(1):105-110.

1061. Bogusz Krzysztof, Kopera Maciej, Jakubczyk Andrzej, Trucco Elisa, Kucharska Katarzyna, Walenda Anna, Wojnar Marcin Prevalence of alcohol use disorder among individuals who binge eat: a systematic review and meta-analysis. ADDICTION.

2021;116(1):18-31.

1062. Sonneville K., Calzo J., Horton N., Field A., Crosby R., Solmi F., Micali N. Childhood hyperactivity/inattention and eating disturbances predict binge eating in adolescence. PSYCHOLOGICAL MEDICINE. 2015;45(12):2511-2520.

1063. Haynos Ann, Pearson Carolyn, Utzinger Linsey, Wonderlich Stephen, Crosby Ross, Mitchell James, Crow Scott, Peterson Carol Empirically derived personality subtyping for predicting clinical symptoms and treatment response in bulimia nervosa. INTERNATIONAL JOURNAL OF EATING DISORDERS. 2017;50(5):506-514.

1064. Raevuori Anu, Suokas Jaana, Haukka Jari, Gissler Mika, Linna Milla, Grainger Marjut, Suvisaari Jaana Highly increased risk of type 2 diabetes in patients with binge eating disorder and bulimia nervosa. INTERNATIONAL JOURNAL OF EATING DISORDERS. 2015;48(6):555-562.

1065. Verstuyf Joke, Van Petegem Stijn, Vansteenkiste Maarten, Soenens Bart, Boone Liesbet The Body Perfect Ideal and Eating Regulation Goals: Investigating the Role of Adolescents' Identity Styles. JOURNAL OF YOUTH AND ADOLESCENCE. 2014;43(2):284-297.

1066. Wagner A, Barbarich-Marsteller NC, Frank GK, Bailer UF, Wonderlich SA, Crosby RD, Henry SE, Vogel V, Plotnicov K, McConaha C, Kaye WH Personality traits after recovery from eating disorders: Do subtypes differ?. INTERNATIONAL JOURNAL OF EATING DISORDERS. 2006;39(4):276-284.

1067. Izydorczyk Bernadetta, Sitnik-Warchulska Katarzyna, Lizinczyk Sebastian, Lipiarz Adrianna Psychological Predictors of Unhealthy Eating Attitudes in Young Adults. FRONTIERS IN PSYCHOLOGY. 2019;10():.

1068. Rosenbaum Diane, White Kamila Does cognitive avoidance mediate the relation of anxiety and binge eating?. EATING AND WEIGHT DISORDERS-STUDIES ON ANOREXIA BULIMIA AND OBESITY. 2016;21(4):653-659.

1069. Yang Jiwon, Han Kuem A rational emotive behavior therapy-based intervention for binge eating behavior management among female students: a quasi-experimental study. JOURNAL OF EATING DISORDERS. 2020;8(1):.

1070. Serpell L, Livingstone A, Neiderman M, Lask B Anorexia nervosa: Obsessive-compulsive disorder, obsessive-compulsive personality disorder, or neither?. CLINICAL PSYCHOLOGY REVIEW. 2002;22(5):647-669.

1071. Fernandez-Aranda F, Amor A, Jimenez-Murcia S, Gimenez-Martinez L, Turon-Gil V, Vallejo-Ruiloba J Bulimia nervosa and misuse of orlistat: Two case reports. INTERNATIONAL JOURNAL OF EATING DISORDERS. 2001;30(4):458-461.

1072. Solmi Marco, Radua Joaquim, Stubbs Brendon, Ricca Valdo, Moretti Davide, Busatta Daniele, Carvalho Andre, Dragioti Elena, Favaro Angela, Monteleone Alessio, Shin Jae, Fusar-Poli Paolo, Castellini Giovanni Risk factors for eating disorders: an umbrella review of published meta-analyses. BRAZILIAN JOURNAL OF PSYCHIATRY. 2021;43(3):314-323.

1073. Carter JC, Fairburn CG Cognitive-behavioral self-help for binge eating disorder: A controlled effectiveness study. JOURNAL OF CONSULTING AND CLINICAL PSYCHOLOGY. 1998;66(4):616-623.

1074. Boffano Paolo, Cavarra Francesco, Tricarico Gerardo, Melle Andrea, Ruslin Muhammad, Rocchetti Vincenzo Bulimia nervosa and oral cavity: a review of the literature. DENTAL CADMOS. 2022;90(1):14-20.

1075. Vanderlinden Johan, Schoevaerts Katrien, Simons Annik, Van Den Eede Ursula, Bruffaerts Ronny, Serra Riccardo, Van Roie Elke, Vervaet Myriam, Janssens Nelle, Vrieze Elske Sociodemographic and clinical characteristics of eating disorder patients treated in the specialized residential settings in Belgium. EATING AND WEIGHT DISORDERS-STUDIES ON ANOREXIA BULIMIA AND OBESITY. 2021;26(2):475-481.

1076. Lampard Amy, Sharbanee Jason The Cognitive-Behavioural Theory and Treatment of Bulimia Nervosa: An Examination of Treatment Mechanisms and Future Directions. AUSTRALIAN PSYCHOLOGIST. 2015;50(1):6-13.

1077. Byrne SM, McLean NJ The cognitive-behavioral model of bulimia nervosa: A direct evaluation. INTERNATIONAL JOURNAL OF EATING DISORDERS. 2002;31(1):17-31.

1078. Harris Samantha, Carrillo Maritza, Fujioka Ken Binge-Eating Disorder and Type 2 Diabetes: A Review. ENDOCRINE PRACTICE. 2021;27(2):158-164.

1079. Costa JL, Brennen MB, Hochgeschwender U The human genetics of eating disorders - Lessons from the leptin/melanocortin system. CHILD AND ADOLESCENT PSYCHIATRIC CLINICS OF NORTH AMERICA. 2002;11(2):387+.

1080. DAVIS C, KENNEDY SH, RAVELSKI E, DIONNE M THE ROLE OF PHYSICAL-ACTIVITY IN THE DEVELOPMENT AND MAINTENANCE OF EATING DISORDERS. PSYCHOLOGICAL MEDICINE. 1994;24(4):957-967.

1081. Franca Giovanny, Gigante Denise, Anselmo Olinto Maria Binge eating in adults: prevalence and association with obesity, poor self-rated health status and body dissatisfaction. PUBLIC HEALTH NUTRITION. 2014;17(4):932-938.

1082. Le Grange Daniel, Crosby Ross, Lock James Predictors and moderators of outcome in family-based treatment for adolescent bulimia nervosa. JOURNAL OF THE AMERICAN ACADEMY OF CHILD AND ADOLESCENT PSYCHIATRY. 2008;47(4):464-470.

1083. Claus Line, Braet Caroline, Decaluwe Veerle Dieting history in obese youngsters with and without disordered eating. INTERNATIONAL JOURNAL OF EATING DISORDERS. 2006;39(8):721-728.

1084. Godfrey Kathryn, Butryn Meghan, Forman Evan, Martinez Monica, Roberts Savannah, Sherwood Nancy Depressive symptoms, psychological flexibility, and binge eating in individuals seeking behavioral weight loss treatment. JOURNAL OF CONTEXTUAL BEHAVIORAL SCIENCE. 2019;14():50-54.

1085. Shomaker Lauren, Tanofsky-Kraff Marian, Matherne Camden, Mehari Rim, Olsen Cara, Marwitz Shannon, Bakalar Jennifer, Ranzenhofer Lisa, Kelly Nichole, Schvey Natasha, Burke Natasha, Cassidy Omni, Brady Sheila, Dietz Laura, Wilfley Denise, Yanovski Susan, Yanovski Jack A randomized, comparative pilot trial of family-based interpersonal psychotherapy for reducing psychosocial symptoms, disordered-eating, and excess weight gain in at-risk preadolescents with loss-of-control-eating. INTERNATIONAL JOURNAL OF EATING DISORDERS. 2017;50(9):1084-1094.

1086. Brewerton Timothy Food addiction as a proxy for eating disorder and obesity severity, trauma history, PTSD symptoms, and comorbidity. EATING AND WEIGHT DISORDERS-STUDIES ON ANOREXIA BULIMIA AND OBESITY. 2017;22(2):241-247.

1087. Salvia Meg, Ritholz Marilyn, Craigen Katherine, Quatromoni Paula Managing type 2 diabetes or prediabetes and binge eating disorder: a qualitative study of patients' perceptions and lived experiences. JOURNAL OF EATING DISORDERS. 2022;10(1):.

1088. Williams-Kerver Gail, Steffen Kristine, Mitchell James Eating Pathology After Bariatric Surgery: an Updated Review of the Recent Literature. CURRENT PSYCHIATRY REPORTS. 2019;21(9):.

1089. Scott Charlotte, Haycraft Emma, Plateau Carolyn A Prospective Study of Teammate Factors on Athletes' Well-Being, Disordered Eating, and Compulsive Exercise. SPORT EXERCISE AND PERFORMANCE PSYCHOLOGY. 2022;11(3):290-304.

1090. Bulik Cynthia, Von Holle Ann, Hamer Robert, Berg Cecilie, Torgersen Leila, Magnus Per, Stoltenberg Camilla, Siega-Riz Anna, Sullivan Patrick, Reichborn-Kjennerud Ted Patterns of remission, continuation and incidence of broadly defined eating disorders during early pregnancy in the Norwegian Mother and Child Cohort Study (MoBa). PSYCHOLOGICAL MEDICINE. 2007;37(8):1109-1118.

1091. Anderson L, Shaw JM, McCargar L Physiological effects of bulimia nervosa on the gastrointestinal tract. CANADIAN JOURNAL OF GASTROENTEROLOGY. 1997;11(5):451-459.

1092. Fichter Manfred, Quadflieg Norbert Mortality in eating disorders - results of a large prospective clinical longitudinal study. INTERNATIONAL JOURNAL OF EATING DISORDERS. 2016;49(4):391-401.

1093. Lie Selma, Bulik Cynthia, Andreassen Ole, Ro Oyvind, Bang Lasse The association between bullying and eating disorders: A case-control study. INTERNATIONAL JOURNAL OF EATING DISORDERS. 2021;54(8):1405-1414.

1094. Ross HE, Ivis F Binge eating and substance use among male and female adolescents. INTERNATIONAL JOURNAL OF EATING DISORDERS. 1999;26(3):245-260.

1095. Linardon Jake, Messer Mariel, Helms Eric, McLean Courtney, Incerti Lisa, Fuller-Tyszkiewicz Matthew Interactions between different eating patterns on recurrent binge eating behavior: A machine learning approach. INTERNATIONAL JOURNAL OF EATING DISORDERS. 2020;53(4):533-540.

1096. Kinkel-Ram Shruti, Williams Brenna, Ortiz Shelby, Forrest Lauren, Magee Joshua, Smith April, Levinson Cheri Testing intrusive thoughts as illness pathways between eating disorders and obsessive-compulsive disorder symptoms: a network analysis. EATING DISORDERS. 2022;30(6):647-669.

1097. Brown Melanie, Hochman Ayelet, Micali Nadia Emotional instability as a trait risk factor for eating disorder behaviors in adolescents: Sex differences in a large-scale prospective study. PSYCHOLOGICAL MEDICINE. 2020;50(11):1783-1794.

1098. Jackson TD, Grilo CM, Masheb RM Teasing history and eating disorder features: An age- and body mass index-matched comparison of bulimia nervosa and binge-eating disorder. COMPREHENSIVE PSYCHIATRY. 2002;43(2):108-113.

1099. Rosenvinge JH, Borgen JS, Borresen R The prevalence and psychological correlates of anorexia nervosa, bulimia nervosa and binge eating among 15-year-old students: A controlled epidemiological study. EUROPEAN EATING DISORDERS REVIEW. 1999;7(5):382-391.

1100. Neumark-Sztainer D, Story M, Hannan PJ, Perry CL, Irving LM Weight-related concerns and behaviors among overweight and nonoverweight adolescents - Implications for preventing weight-related disorders. ARCHIVES OF PEDIATRICS \& ADOLESCENT MEDICINE. 2002;156(2):171-178.

1101. Romano Kelly, Heron Kristin, Ebener Deborah Associations among weight suppression, self-acceptance, negative body image, and eating disorder behaviors among women with eating disorder symptoms. WOMEN \& HEALTH. 2021;61(8):791-799.

1102. Chen Ximei, Li Wei, Qin Jingmin, Gao Xiao, Liu Yong, Song Shiqing, Huang Yufei, Chen Hong Gray matter volume and functional connectivity underlying binge eating in healthy children. EATING AND WEIGHT DISORDERS-STUDIES ON ANOREXIA BULIMIA AND OBESITY. 2022;27(8):3469-3478.

1103. Agras WS Pharmacotherapy of bulimia nervosa and binge eating disorder: Longer-term outcomes. PSYCHOPHARMACOLOGY BULLETIN. 1997;33(3):433-436.

1104. Piccoli Laura, Di Bonaventura Maria, Cifani Carlo, Costantini Vivian, Massagrande Mario, Montanari Dino, Martinelli Prisca, Antolini Marinella, Ciccocioppo Roberto, Massi Maurizio, Merlo-Pich Emilio, Di Fabio Romano, Corsi Mauro Role of Orexin-1 Receptor

Mechanisms on Compulsive Food Consumption in a Model of Binge Eating in Female Rats. NEUROPSYCHOPHARMACOLOGY. 2012;37(9):1999-2011.

1105. Hughes Elizabeth, Goldschmidt Andrea, Labuschagne Zandre, Loeb Katharine, Sawyer Susan, Le Grange Daniel Eating Disorders with and without Comorbid Depression and Anxiety: Similarities and Differences in a Clinical Sample of Children and Adolescents. EUROPEAN EATING DISORDERS REVIEW. 2013;21(5):386-394.

1106. Watson Ryan, Adjei Jones, Saewyc Elizabeth, Homma Yuko, Goodenow Carol Trends and disparities in disordered eating among heterosexual and sexual minority adolescents. INTERNATIONAL JOURNAL OF EATING DISORDERS. 2017;50(1):22-31.

1107. Carpine Laureline, Charvin Isabelle, Da Fonseca David, Bat-pitault Flora Clinical features of children and adolescents with anorexia nervosa and problematic physical activity. EATING AND WEIGHT DISORDERS-STUDIES ON ANOREXIA BULIMIA AND OBESITY. 2022;27(1):119-129.

1108. Harrington Ellen, Crowther Janis, Shipherd Jillian Trauma, Binge Eating, and the ``Strong Black Woman{''}. JOURNAL OF CONSULTING AND CLINICAL PSYCHOLOGY. 2010;78(4):469-479.

1109. Tanofsky-Kraff Marian, Schvey Natasha, Grilo Carlos A Developmental Framework of Binge-Eating Disorder Based on Pediatric Loss of Control Eating. AMERICAN PSYCHOLOGIST. 2020;75(2, SI):189-203.

1110. Goossens Lien, Braet Caroline, Bosmans Guy Relations of dietary restraint and depressive symptomatology to loss of control over eating in overweight youngsters. EUROPEAN CHILD \& ADOLESCENT PSYCHIATRY. 2010;19(7):587-596.

1111. Zwaan M., Friederich H. Binge eating disorder. THERAPEUTISCHE UMSCHAU. 2006;63(8):529-533.

1112. Muratore Alexandra, Lowe Michael Why is premorbid BMI consistently elevated in clinical samples, but not in risk factor samples, of individuals with eating disorders?. INTERNATIONAL JOURNAL OF EATING DISORDERS. 2019;52(2):117-120.

1113. Teixeira Paula, Costa Roberto, Matsudo Sandra, Cordas Taki Physical exercises in patients with eating disorders. REVISTA DE PSIQUIATRIA CLINICA. 2009;36(4):138-145.

1114. Nickel Kathrin, Maier Simon, Endres Dominique, Joos Andreas, Maier Viktoria, Elst Ludger, Zeeck Almut Systematic Review: Overlap Between Eating, Autism Spectrum, and Attention-Deficit/Hyperactivity Disorder. FRONTIERS IN PSYCHIATRY. 2019;10():.

1115. Patterson Megan, Goodson Patricia Using Social Network Analysis to Better Understand Compulsive Exercise Behavior Among a Sample of Sorority Members. JOURNAL OF PHYSICAL ACTIVITY \& HEALTH. 2017;14(5):360-367.

1116. Jennings Karen, Wildes Jennifer, Coccaro Emil Intermittent explosive disorder and eating disorders: Analysis of national comorbidity and research samples. COMPREHENSIVE PSYCHIATRY. 2017;75():62-67.

1117. Meule Adrian A Critical Examination of the Practical Implications Derived from the Food Addiction Concept. CURRENT OBESITY REPORTS. 2019;8(1):11-17.

1118. DuBois Russell, Rodgers Rachel, Franko Debra, Eddy Kamryn, Thomas Jennifer A network analysis investigation of the cognitive-behavioral theory of eating disorders. BEHAVIOUR RESEARCH AND THERAPY. 2017;97():213-221.

1119. Simpson H., Wetterneck Chad, Cahill Shawn, Steinglass Joanna, Franklin Martin, Leonard Rachel, Weltzin Theodore, Riemann Bradley Treatment of Obsessive-Compulsive Disorder Complicated by Comorbid Eating Disorders. COGNITIVE BEHAVIOUR THERAPY. 2013;42(1):64-76.

1120. Chapa Danielle, Kite Benjamin, Forbush Kelsie, Tregarthen Jenna, Argue Stuart Eating-disorder psychopathology and driven exercise change models: A latent change score analysis. INTERNATIONAL JOURNAL OF EATING DISORDERS. 2020;53(12):2013-2025.

1121. Burke Natasha, Neyland M., Young Jami, Wilfley Denise, Tanofsky-Kraff Marian Interpersonal psychotherapy for the prevention of binge-eating disorder and adult obesity in an African American adolescent military dependent boy. EATING BEHAVIORS. 2020;38():.

1122. Bulik CM, Sullivan PF, Fear JL, Joyce PR Eating disorders and antecedent anxiety disorders: A controlled study. ACTA PSYCHIATRICA SCANDINAVICA. 1997;96(2):101-107.

1123. Weintraub Daniel, Koester Juergen, Potenza Marc, Siderowf Andrew, Stacy Mark, Voon Valerie, Whetteckey Jacqueline, Wunderlich Glen, Lang Anthony Impulse Control Disorders in Parkinson Disease A Cross-Sectional Study of 3090 Patients. ARCHIVES OF NEUROLOGY. 2010;67(5):589-595.

1124. Beintner Ina, Emmerich Olivia, Vollert Bianka, Taylor C., Jacobi Corinna Promoting positive body image and intuitive eating in women with overweight and obesity via an online intervention: Results from a pilot feasibility study. EATING BEHAVIORS. 2019;34():.

1125. Aguera Zaida, Lozano-Madrid Maria, Mallorqui-Bague Nuria, Jimenez-Murcia Susana, Menchon Jose, Fernandez-Aranda Fernando A review of binge eating disorder and obesity. NEUROPSYCHIATRIE. 2021;35(2):57-67.

1126. Rossi Alessandro, Pietrabissa Giada, Tagliagambe Andrea, Scuderi Anna, Montecchiani Lorenzo, Castelnuovo Gianluca, Mannarini Stefania, Dalla Ragione Laura Many Facets of Eating Disorders: Profiling Key Psychological Features of Anorexia Nervosa and Binge Eating Disorder. BEHAVIORAL SCIENCES. 2023;13(3):.

1127. Bianchi Dora, Baiocco Roberto, Pompili Sara, Lonigro Antonia, Di Norcia Anna, Cannoni Eleonora, Longobardi Emiddia, Zammuto Marta, Di Tata Daniele, Laghi Fiorenzo Binge Eating and Binge Drinking in Emerging Adults During COVID-19 Lockdown in Italy: An Examination of Protective and Risk Factors. EMERGING ADULTHOOD. 2022;10(1, SI):291-303.

1128. Vannucci Anna, Kass Andrea, Sinton Meghan, Aspen Vandana, Weisman Hannah, Bailey Jakki, Wilfley Denise, Taylor C. An examination of the Clinical Impairment Assessment among women at high risk for eating disorder onset. BEHAVIOUR RESEARCH AND THERAPY. 2012;50(6):407-414.

1129. Hildebrandt Britny, Fisher Hayley, Ahmari Susanne Examination of Onset Trajectories and Persistence of Binge-Like Eating Behavior in Mice After Intermittent Palatable Food Exposure. BEHAVIORAL NEUROSCIENCE. 2023;():.

1130. Baker Jessica, Runfola Cristin Eating disorders in midlife women: A perimenopausal eating disorder?. MATURITAS. 2016;85():112-116.

1131. Konstantakopoulos George, Varsou Eleytheria, Dikeos Dimitris, Ioannidi Nikoleta, Gonidakis Fragiskos, Papadimitriou George, Oulis Panagiotis Delusionality of body image beliefs in eating disorders. PSYCHIATRY RESEARCH. 2012;200(2-3):482-488.

1132. Pompanin Sara, Jelcic Nela, Cecchin Diego, Cagnin Annachiara Impulse control disorders in frontotemporal dementia: spectrum of symptoms and response to treatment. GENERAL HOSPITAL PSYCHIATRY. 2014;36(6):.

1133. Santonastaso P, Zambenedetti M, Favaro A, Favaron C, Pavan T Family psychiatric morbidity in eating disorders. EUROPEAN EATING DISORDERS REVIEW. 1997;5(1):3-10.

1134. Cachelin FM, Schug RA, Juarez LC, Monreal TK Sexual abuse and eating disorders in a community sample of Mexican American women. HISPANIC JOURNAL OF BEHAVIORAL SCIENCES. 2005;27(4):533-546.

1135. Almenara Carlos, Umemura Tomo, Macek Petr PARENT-DAUGHTER RELATIONSHIPS AND DISORDERED EATING AMONG EMERGING ADULT WOMEN FROM THE CZECH REPUBLIC. STUDIA PSYCHOLOGICA. 2016;58(3):216-230.

1136. Tetzlaff Anne, Schmidt Ricarda, Brauhardt Anne, Hilbert Anja Family Functioning in Adolescents with Binge-Eating Disorder. EUROPEAN EATING DISORDERS REVIEW. 2016;24(5):430-433.

1137. Donnelly Brooke, Touyz Stephen, Hay Phillipa, Burton Amy, Russell Janice, Caterson Ian Neuroimaging in bulimia nervosa and binge eating disorder: a systematic review. JOURNAL OF EATING DISORDERS. 2018;6():.

1138. Swanson Sonja, Saito Naomi, Borges Guilherme, Benjet Corina, Aguilar-Gaxiola Sergio, Medina-Mora Maria, Breslau Joshua Change in binge eating and binge eating disorder associated with migration from Mexico to the US. JOURNAL OF PSYCHIATRIC RESEARCH. 2012;46(1):31-37.

1139. Stunkard Albert Eating Disorders and Obesity. PSYCHIATRIC CLINICS OF NORTH AMERICA. 2011;34(4):765+.

1140. Hricova Lucia, Orosova O., Bacikova-Sleskova M. Disordered eating in the context of Self-determination theory. CURRENT PSYCHOLOGY. 2020;39(2):608-617.

1141. Robertson DN, Palmer RL The prevalence and correlates of binge eating in a British community sample of women with a history of obesity. INTERNATIONAL JOURNAL OF EATING DISORDERS. 1997;22(3):323-327.

1142. Anderson DA, Maloney KC The efficacy of cognitive-behavioral therapy on the core symptoms of bulimia nervosa. CLINICAL PSYCHOLOGY REVIEW. 2001;21(7):971-988.

1143. Sahlan Reza, Sala Margaret Eating disorder psychopathology and resilience in Iranian college students: A network analysis. JOURNAL OF CLINICAL PSYCHOLOGY. 2023;79(2):497-513.

1144. Yan Wan-Sen, Zheng Dan-Hui, Liu Meng-Meng Trait Impulsivity and Choice Impulsivity in Young Adult Students With Probable Binge Eating Disorder. FRONTIERS IN PSYCHIATRY. 2022;13():.

1145. Starkey Karina, Wade Tracey Disordered eating in girls with Type 1 diabetes: Examining directions for prevention. CLINICAL PSYCHOLOGIST. 2010;14(1):2-9.

1146. Tanofsky-Kraff M, Yanovski SZ, Wilfley DE, Marmarosh C, Morgan CM, Yanovski JA Eating-disordered behaviors, body fat, and psychopathology in overweight and normal-weight children. JOURNAL OF CONSULTING AND CLINICAL PSYCHOLOGY. 2004;72(1):53-61.

1147. Ferrer-Garcia Marta, Pla-Sanjuanelo Joana, Dakanalis Antonios, Vilalta-Abella Ferran, Riva Giuseppe, Fernandez-Aranda Fernando, Forcano Laura, Riesco Nadine, Sanchez Isabel, Clerici Massimo, Ribas-Sabate Joan, Andreu-Gracia Alexis, Escandon-Nagel Neli, Gomez-Tricio Osane, Tena Virginia, Gutierrez-Maldonado Jose A Randomized Trial of Virtual Reality-Based Cue Exposure Second-Level Therapy and Cognitive Behavior Second-Level Therapy for Bulimia Nervosa and Binge-Eating Disorder: Outcome at Six-Month Followup. CYBERPSYCHOLOGY BEHAVIOR AND SOCIAL NETWORKING. 2019;22(1, SI):60-68.

1148. Biby EL The relationship between body dysmorphic disorder and depression, self-esteem, somatization, and obsessive-compulsive disorder. JOURNAL OF CLINICAL PSYCHOLOGY. 1998;54(4):489-499.

1149. Pattinson Andrea, Nassar Natasha, Luz Felipe, Hay Phillipa, Touyz Stephen, Sainsbury Amanda The Real Happy Study: Protocol for a Prospective Assessment of the Real-World Effectiveness of the HAPIFED Program-a Healthy APproach to weIght management and Food in Eating Disorders. BEHAVIORAL SCIENCES. 2019;9(7):.

1150. Quesnel Danika, Cook Brian, Murray Kellen, Zamudio Jessica Inspiration or Thinspiration: the Association Among Problematic Internet Use, Exercise Dependence, and Eating Disorder Risk. INTERNATIONAL JOURNAL OF MENTAL HEALTH AND ADDICTION. 2018;16(5):1113-1124.

1151. Laghi Fiorenzo, Bianchi Dora, Pompili Sara, Lonigro Antonia, Baiocco Roberto Metacognition, emotional functioning and binge eating in adolescence: the moderation role of need to control thoughts. EATING AND WEIGHT DISORDERS-STUDIES ON ANOREXIA BULIMIA AND OBESITY. 2018;23(6):861-869.

1152. Troop N. Helplessness, mastery and the development of eating disorders: Exploring the links between vulnerability and precipitating factors. EATING AND WEIGHT DISORDERS-STUDIES ON ANOREXIA BULIMIA AND OBESITY. 2012;17(4):E274-E281.

1153. Altman Sarah, Shankman Stewart What is the association between obsessive-compulsive disorder and eating disorders?. CLINICAL PSYCHOLOGY REVIEW. 2009;29(7):638-646.

1154. Donovan Caroline, Uhlmann Laura Looking at me, looking at you: The mediating roles of body surveillance and social comparison in the relationship between fit ideal internalisation and body dissatisfaction. EATING BEHAVIORS. 2022;47():.

1155. Briguglio Matteo, Vitale Jacopo, Galentino Roberta, Banfi Giuseppe, Dina Carlotta, Bona Alberto, Panzica Giancarlo, Porta Mauro, Dell'Osso Bernardo, Glick Ira Healthy Eating, Physical Activity, and Sleep Hygiene (HEPAS) as the Winning Triad for Sustaining Physical and Mental Health in Patients at Risk for or with Neuropsychiatric Disorders: Considerations for Clinical Practice. NEUROPSYCHIATRIC DISEASE AND TREATMENT. 2020;16():55-70.

1156. Schmidt UH, Troop NA, Treasure JL Events and the onset of eating disorders: Correcting an ``age old{''} myth. INTERNATIONAL JOURNAL OF EATING DISORDERS. 1999;25(1):83-88.

1157. Hilbert Anja Psychotherapy of eating disorders. ZEITSCHRIFT FUR PSYCHIATRIE PSYCHOLOGIE UND PSYCHOTHERAPIE. 2014;62(1):5-7.

1158. Hernandez-Escalante V, Trava-Garcia M, Bastarrachea-Sosa R, Laviada-Molina H Metabolic disorders in bulimia nervosa and binge eating.. SALUD MENTAL. 2003;26(3):9-15.

1159. Jacobson Isabel, Smith Tyler, Smith Besa, Keel Pamela, Amoroso Paul, Wells Timothy, Bathalon Gaston, Boyko Edward, Ryan Margaret, Team Millennium Disordered Eating and Weight Changes After Deployment: Longitudinal Assessment of a Large US Military Cohort. AMERICAN JOURNAL OF EPIDEMIOLOGY. 2009;169(4):415-427.

1160. Bodell Lindsay, Wildes Jennifer, Cheng Yu, Goldschmidt Andrea, Keenan Kate, Hipwell Alison, Stepp Stephanie Associations between Race and Eating Disorder Symptom Trajectories in Black and White Girls. JOURNAL OF ABNORMAL CHILD PSYCHOLOGY. 2018;46(3):625-638.

1161. Dagan Yael, Yager Joel Severe bupropion XR abuse in a patient with long-standing bulimia nervosa and complex PTSD. INTERNATIONAL JOURNAL OF EATING DISORDERS. 2018;51(10):1207-1209.

1162. Halmi Katherine, Bellace Dara, Berthod Samantha, Ghosh Samiran, Berrettini Wade, Brandt Harry, Bulik Cynthia, Crawford Steve, Fichter Manfred, Johnson Craig, Kaplan Allan, Kaye Walter, Thornton Laura, Treasure Janet, Woodside D., Strober Michael An examination of early childhood perfectionism across anorexia nervosa subtypes. INTERNATIONAL JOURNAL OF EATING DISORDERS. 2012;45(6):800-807.

1163. Bandini S., Antonelli G., Moretti P., Pampanelli S., Quartesan R., Perriello G. Factors affecting dropout in outpatient eating disorder treatment. EATING AND WEIGHT DISORDERS-STUDIES ON ANOREXIA BULIMIA AND OBESITY. 2006;11(4):179-184.

1164. Lichtenstein Mia, Griffiths Mark, Hemmingsen Simone, Stoving Rene Exercise addiction in adolescents and emerging adults - Validation of a youth version of the Exercise

Addiction Inventory. JOURNAL OF BEHAVIORAL ADDICTIONS. 2018;7(1):117-125.

1165. Goldschmidt Andrea, Wall Melanie, Loth Katie, Bucchianeri Michaela, Neumark-Sztainer Dianne The Course of Binge Eating From Adolescence to Young Adulthood. HEALTH PSYCHOLOGY. 2014;33(5):457-460.

1166. MacDonald Danielle, Trottier Kathryn, Olmsted Marion Rapid improvements in emotion regulation predict intensive treatment outcome for patients with bulimia nervosa and purging disorder. INTERNATIONAL JOURNAL OF EATING DISORDERS. 2017;50(10):1152-1161.

1167. Selenius Sofia, Birgegard Andreas, Mantilla Emma Preliminary evaluation of the online course ``I Care{''} targeting eating disorder knowledge and attitudes among sports coaches and fitness instructors. JOURNAL OF EATING DISORDERS. 2022;10(1):.

1168. Prefit Alice-Beatrice, Candea Diana, Szentagotai-Tatar Aurora Emotion regulation across eating pathology: A meta-analysis. APPETITE. 2019;143():.

1169. Keel Pamela Purging disorder: recent advances and future challenges. CURRENT OPINION IN PSYCHIATRY. 2019;32(6):518-524.

1170. Field Alison, Sonneville Kendrin, Micali Nadia, Crosby Ross, Swanson Sonja, Laird Nan, Treasure Janet, Solmi Francesca, Horton Nicholas Prospective Association of Common Eating Disorders and Adverse Outcomes. PEDIATRICS. 2012;130(2):E289-E295.

1171. Chen Joanna, Singh Simar, Lowe Michael The food restriction wars: Proposed resolution of a primary battle. PHYSIOLOGY \& BEHAVIOR. 2021;240():.

1172. Kelly Nichole, Bulik Cynthia, Mazzeo Suzanne Executive functioning and behavioral impulsivity of young women who binge eat. INTERNATIONAL JOURNAL OF EATING DISORDERS. 2013;46(2):127-139.

1173. Buckner Julia, Silgado Jose, Lewinsohn Peter Delineation of differential temporal relations between specific eating and anxiety disorders. JOURNAL OF PSYCHIATRIC RESEARCH. 2010;44(12):781-787.

1174. Qadir Shwan, Muhsin Salwa Administration of Psychoeducational Treatment Programs Improves Depression, Eating Disorders, and Associated Psychological Traits. INTERNATIONAL JOURNAL OF MEDICAL RESEARCH \& HEALTH SCIENCES. 2020;9(2):32-42.

1175. Magson Natasha, Handford Charlotte, Norberg Melissa The Empirical Status of Cue Exposure and Response Prevention Treatment for Binge Eating: A Systematic Review. BEHAVIOR THERAPY. 2021;52(2, SI):442-454.

1176. Navarro-Tapia Elisabet, Almeida-Toledano Laura, Sebastiani Giorgia, Serra-Delgado Mariona, Garcia-Algar Oscar, Andreu-Fernandez Vicente Effects of Microbiota Imbalance in Anxiety and Eating Disorders: Probiotics as Novel Therapeutic Approaches. INTERNATIONAL JOURNAL OF MOLECULAR SCIENCES. 2021;22(5):.

1177. Stice E Modeling of eating pathology and social reinforcement of the thin-ideal predict onset of bulimic symptoms. BEHAVIOUR RESEARCH AND THERAPY. 1998;36(10):931-944.

1178. Goldschmidt Andrea, Wilfley Denise, Eddy Kamryn, Boutelle Kerri, Zucker Nancy, Peterson Carol, Celio-Doyle Angela, Le Grange Daniel Overvaluation of shape and weight among overweight children and adolescents with loss of control eating. BEHAVIOUR RESEARCH AND THERAPY. 2011;49(10):682-688.

1179. Mustelin Linda, Lehtokari Vilma-Lotta, Keski-Rahkonen Anna Other specified and unspecified feeding or eating disorders among women in the community. INTERNATIONAL JOURNAL OF EATING DISORDERS. 2016;49(11):1010-1017.

1180. French SA, Jeffery RW, Sherwood NE, Neumark-Sztainer D Prevalence and correlates of binge eating in a nonclinical sample of women enrolled in a weight gain prevention program. INTERNATIONAL JOURNAL OF OBESITY. 1999;23(6):576-585.

1181. Wang Shirley, Pisetsky Emily, Skutch Julie, Fruzzetti Alan, Haynos Ann Restrictive eating and nonsuicidal self-injury in a nonclinical sample: Co-occurrence and associations with emotion dysregulation and interpersonal problems. COMPREHENSIVE PSYCHIATRY. 2018;82():128-132.

1182. Beintner Ina, Jacobi Corinna Internet-based follow-up care for bulimia nervosa. PSYCHOTHERAPEUT. 2011;56(6):516-521.

1183. Sari Norma, Noviasty Reny, Ifroh Riza Dietary Behavior and the Risk of Developing Eating Disorders among Adolescent Girls who Participate in Modern Dance. JURNAL GIZI DAN PANGAN. 2021;16(1):81-88.

1184. Podfigurna-Stopa Agnieszka, Czyzyk Adam, Katulski Krzysztof, Smolarczyk Roman, Grymowicz Monika, Maciejewska-Jeske Marzena, Meczekalski Blazej Eating disorders in older women. MATURITAS. 2015;82(2):146-152.

1185. Pagoto Sherry, Bodenlos Jamie, Kantor Lyle, Gitkind Mitchell, Curtin Carol, Ma Yunsheng Association of major depression and binge eating disorder with weight loss in a clinical setting. OBESITY. 2007;15(11):2557-2559.

1186. Stice Eric, Durant Shelley, Rohde Paul, Shaw Heather Effects of a Prototype Internet Dissonance-Based Eating Disorder Prevention Program at 1-and 2-Year Follow-Up. HEALTH PSYCHOLOGY. 2014;33(12):1558-1567.

1187. Stice E, Presnell K, Bearman SK Relation of early menarche to depression, eating disorders, substance abuse, and comorbid psychopathology among adolescent girls. DEVELOPMENTAL PSYCHOLOGY. 2001;37(5):608-619.

1188. Peterson CB, Miller KB, Crow SJ, Thuras P, Mitchell JE Subtypes of binge eating disorder based on psychiatric history. INTERNATIONAL JOURNAL OF EATING DISORDERS. 2005;38(3):273-276.

1189. Holm-Denoma Jill, Hankin Benjamin, Young Jami Developmental trends of eating disorder symptoms and comorbid internalizing symptoms in children and adolescents. EATING BEHAVIORS. 2014;15(2):275-279.

1190. Clarine Lauren, Kittleson Kimberly, Fujioka Ken Incidence of Prediabetes in Patients with Binge Eating Disorder Seeking Weight Management. DIABETES. 2017;66(1):A217.

1191. FAHY TA, OSACAR A, MARKS I HISTORY OF EATING DISORDERS IN FEMALE-PATIENTS WITH OBSESSIVE-COMPULSIVE DISORDER. INTERNATIONAL JOURNAL OF EATING DISORDERS. 1993;14(4):439-443.

1192. Didie Elizabeth, Reinecke Mark, Phillips Katharine Case Conceptualization and Treatment of Comorbid Body Dysmorphic Disorder and Bulimia Nervosa. COGNITIVE AND

BEHAVIORAL PRACTICE. 2010;17(3):259-269.

1193. Keel Pamela, Brown Tiffany Update on Course and Outcome in Eating Disorders. INTERNATIONAL JOURNAL OF EATING DISORDERS. 2010;43(3):195-204.

1194. Kenny Bridget, Fuller-Tyszkiewicz Matthew, Moodie Marj, Brown Vicki, Williams Joanne Bi-directional associations between depressive symptoms and eating disorder symptoms in early adolescence. BODY IMAGE. 2022;42():246-256.

1195. Constant Aymery, Gautier Yentl, Coquery Nicolas, Thibault Ronan, Moirand Romain, Val-Laillet David Emotional overeating is common and negatively associated with alcohol use in normal-weight female university students. APPETITE. 2018;129():186-191.

1196. Ranson KM, Kaye WH, Weltzin TE, Rao R, Matsunaga H Obsessive-compulsive disorder symptoms before and after recovery from bulimia nervosa. AMERICAN JOURNAL OF PSYCHIATRY. 1999;156(11):1703-1708.

1197. Masheb Robin, Grilo Carlos Examination of Predictors and Moderators for Self-Help Treatments of Binge-Eating Disorder. JOURNAL OF CONSULTING AND CLINICAL PSYCHOLOGY. 2008;76(5):900-904.

1198. Pluhar Emily, Abdullah Syidah, Burton E. Endorsement of Binge Eating Symptoms in a Sample of Predominantly Non-Hispanic Black Adolescents. CLINICAL PEDIATRICS. 2020;59(8):766-772.

1199. Werner Antonia, Beutel Manfred, Ernst Mareike, Braehler Elmar, Berger Uwe, Strauss Bernhard, Tibubos Ana Child Maltreatment and Eating Disturbances - The Role of Self-Regulation. ZEITSCHRIFT FUR PSYCHIATRIE PSYCHOLOGIE UND PSYCHOTHERAPIE. 2019;67(2):112-124.

1200. Stice E, Mazotti L, Krebs M, Martin S Predictors of adolescent dieting behaviors: A longitudinal study. PSYCHOLOGY OF ADDICTIVE BEHAVIORS. 1998;12(3):195-205.

1201. Martinez-Gonzalez Leticia, Fernandez Villa Tania, Torre Antonio, Ayan Perez Carlos, Bueno Cavanillas Aurora, Capelo Alvarez Rocio, Mateos Campos Ramona, Martin Sanchez Vicente PREVALENCE OF EATING DISORDERS IN COLLEGE STUDENTS AND ASSOCIATED

FACTORS: UNHICOS PROJECT. NUTRICION HOSPITALARIA. 2014;30(4):927-934.

1202. Fichter M., Quadflieg N., Nisslmueller K., Lindner S., Voderholzer U., Wuensch-Leiteritz W., Osen B., Huber T., Zahn S., Meermann R., Irrgang V., Bleichner F. Internet-based approaches in the therapy of eating disorders. NERVENARZT. 2011;82(9):1107+.

1203. Shapiro Jennifer, Woolson Sandra, Hamer Robert, Kalarchian Melissa, Marcus Marsha, Bulik Cynthia Evaluating binge eating disorder in children: Development of the Children's Binge Eating Disorder Scale (C-BEDS). INTERNATIONAL JOURNAL OF EATING DISORDERS. 2007;40(1):82-89.

1204. Hannon-Engel Sandra, Filin Evgeniy, Wolfe Barbara CCK response in bulimia nervosa and following remission. PHYSIOLOGY \& BEHAVIOR. 2013;122(SI):56-61.

1205. Becker Daniel, Grilo Carlos Comorbidity of mood and substance use disorders in patients with binge-eating disorder: Associations with personality disorder and eating disorder pathology. JOURNAL OF PSYCHOSOMATIC RESEARCH. 2015;79(2):159-164.

1206. Dolan Sarah, Reilly Erin, Brown Tiffany, Shott Megan, Frank Guido Anticipatory and consummatory pleasure in eating disorders. JOURNAL OF EATING DISORDERS. 2022;10(1):.

1207. Johnson JG, Cohen P, Kasen S, Brook JS Personality and risk disorder traits evident by early adulthood for eating and weight problems during middle adulthood. INTERNATIONAL JOURNAL OF EATING DISORDERS. 2006;39(3):184-192.

1208. Kass Andrea, Taylor C., Wilfley Denise Relation Between Weight Status and Disordered Eating Pathology in College-Age Women at High-Risk For Eating Disorder Onset. OBESITY. 2011;19(1):S141.

1209. Udo Tomoko, White Marney, Lydecker Janet, Barnes Rachel, Genao Inginia, Garcia Rina, Masheb Robin, Grilo Carlos Biopsychosocial Correlates of Binge Eating Disorder in Caucasian and African American Women with Obesity in Primary Care Settings. EUROPEAN EATING DISORDERS REVIEW. 2016;24(3):181-186.

1210. Yan Wan-Sen, Zhang Ran-Ran, Lan Yan, Li Zhi-Ming, Li Yong-Hui Questionnaire-Based Maladaptive Decision-Coping Patterns Involved in Binge Eating Among 1013 College

Students. FRONTIERS IN PSYCHOLOGY. 2018;9():.

1211. Brauhardt Anne, Zwaan Martina, Hilbert Anja The Therapeutic Process in Psychological Treatments for Eating Disorders: A Systematic Review. INTERNATIONAL JOURNAL OF EATING DISORDERS. 2014;47(6):565-584.

1212. Mond Jonathan, Hay Phillipa, Rodgers Bryan, Owen Cathy Self-recognition of disordered eating among women with bulimic-type eating disorders: A community-based study. INTERNATIONAL JOURNAL OF EATING DISORDERS. 2006;39(8):747-753.

1213. Tor Phern, Lee Ee Treatment Emergent Mania Responding to Valproate in a Chinese Female Adolescent Population with Eating Disorders: A Case Series. EUROPEAN EATING DISORDERS REVIEW. 2008;16(6):421-426.

1214. Franklin Elizabeth, Simpson Vanessa, Berthet-Miron Michelle, Gupta Olga, Barlow Sarah A Pilot Study Evaluating a Binge-Eating Screener in Children: Development of the Children's Brief Binge-Eating Questionnaire in a Pediatric Obesity Clinic. CLINICAL PEDIATRICS. 2019;58(10):1063-1071.

1215. Tong Jenny, D'Alessio David Eating disorders and gastrointestinal peptides. CURRENT OPINION IN ENDOCRINOLOGY DIABETES AND OBESITY. 2011;18(1):42-49.

1216. Brelet Lisa, Flaudias Valentin, Desert Michel, Guillaume Sebastien, Llorca Pierre-Michel, Boirie Yves Stigmatization toward People with Anorexia Nervosa, Bulimia Nervosa, and Binge Eating Disorder: A Scoping Review. NUTRIENTS. 2021;13(8):.

1217. Le Long, Hay Phillipa, Mihalopoulos Cathrine A systematic review of cost-effectiveness studies of prevention and treatment for eating disorders. AUSTRALIAN AND NEW ZEALAND JOURNAL OF PSYCHIATRY. 2018;52(4):328-338.

1218. Varchol Lisa, Cooper Henrietta Psychotherapy approaches for adolescents with eating disorders. CURRENT OPINION IN PEDIATRICS. 2009;21(4):457-464.

1219. Ivezaj Valentina, Kalebjian Roushig, Grilo Carlos, Barnes Rachel Comparing weight gain in the year prior to treatment for overweight and obese patients with and without binge eating disorder in primary care. JOURNAL OF PSYCHOSOMATIC RESEARCH.

2014;77(2):151-154.

1220. Cimino Silvia, Simonelli Alessandra, Parolin Micol, Ballarotto Giulia, Carbone Paola, Cerniglia Luca A Theoretical and Empirical Linkage between Road Accidents and Binge Eating Behaviors in Adolescence. INTERNATIONAL JOURNAL OF ENVIRONMENTAL RESEARCH AND PUBLIC HEALTH. 2018;15(2):.

1221. Yilmaz Zeynep, Gottfredson Nisha, Zerwas Stephanie, Bulik Cynthia, Micali Nadia Developmental Premorbid Body Mass Index Trajectories of Adolescents With Eating Disorders in a Longitudinal Population Cohort. JOURNAL OF THE AMERICAN ACADEMY OF CHILD AND ADOLESCENT PSYCHIATRY. 2019;58(2):191-199.

1222. Dubosc Auberi, Capitaine Maud, Franko Debra, Bui Eric, Brunet Alain, Chabrol Henri, Rodgers Rachel Early adult sexual assault and disordered eating: The mediating role of posttraumatic stress symptoms. JOURNAL OF TRAUMATIC STRESS. 2012;25(1):50-56.

1223. Berg Kelly, Wonderlich Stephen Emerging Psychological Treatments in the Field of Eating Disorders. CURRENT PSYCHIATRY REPORTS. 2013;15(11):.

1224. Goode Rachel, Webster C., Gwira Rebecca A Review of Binge-Eating Disorder in Black Women: Treatment Recommendations and Implications for Healthcare Providers. CURRENT PSYCHIATRY REPORTS. 2022;24(12):757-766.

1225. Feinson Marjorie, Hornik-Lurie Tzipi Binge eating \& childhood emotional abuse: The mediating role of anger. APPETITE. 2016;105():487-493.

1226. Sim Leslie, Lebow Jocelyn, Weiss Karen, Harrison Tracy, Bruce Barbara Eating Disorders in Adolescents With Chronic Pain. JOURNAL OF PEDIATRIC HEALTH CARE. 2017;31(1):67-74.

1227. Simpson Courtney, Mazzeo Suzanne Attitudes toward orthorexia nervosa relative to DSM-5 eating disorders. INTERNATIONAL JOURNAL OF EATING DISORDERS. 2017;50(7):781-792.

1228. Masheb RM, Grilo CM On the relation of flexible and rigid control of eating to body mass index and overeating in patients with binge eating disorder. INTERNATIONAL

JOURNAL OF EATING DISORDERS. 2002;31(1):82-91.

1229. French SA, Peterson CB, Story M, Anderson N, Mussell MP, Mitchell JE Agreement between survey and interview measures of weight control practices in adolescents. INTERNATIONAL JOURNAL OF EATING DISORDERS. 1998;23(1):45-56.

1230. Lee-Winn Angela, Reinblatt Shauna, Mojtabai Ramin, Mendelson Tamar Gender and racial/ethnic differences in binge eating symptoms in a nationally representative sample of adolescents in the United States. EATING BEHAVIORS. 2016;22():27-33.

1231. Park Emma, Waller Glenn, Gannon Kenneth Early Improvement in Eating Attitudes during Cognitive Behavioural Therapy for Eating Disorders: The Impact of Personality Disorder Cognitions. BEHAVIOURAL AND COGNITIVE PSYCHOTHERAPY. 2014;42(2):224-237.

1232. Davis Heather, Smith Gregory An Integrative Model of Risk for High School Disordered Eating. JOURNAL OF ABNORMAL PSYCHOLOGY. 2018;127(6):559-570.

1233. Dobrescu Sandra, Dinkler Lisa, Gillberg Carina, Rastam Maria, Gillberg Christopher, Wentz Elisabet Anorexia nervosa: 30-year outcome. BRITISH JOURNAL OF PSYCHIATRY. 2020;216(2):97-104.

1234. Garfinkel PE, Lin E, Goering P, Spegg C, Goldbloom DS, Kennedy S, Kaplan AS, Woodside DB Purging and nonpurging forms of bulimia nervosa in a community sample. INTERNATIONAL JOURNAL OF EATING DISORDERS. 1996;20(3):231-238.

1235. Guidi Jenny, Pender Maribeth, Hollon Steven, Zisook Sidney, Schwartz Faye, Pedrelli Paola, Farabaugh Amy, Fava Maurizio, Petersen Timothy The prevalence of compulsive eating and exercise among college students: An exploratory study. PSYCHIATRY RESEARCH. 2009;165(1-2):154-162.

1236. Zehr Julia, Culbert Kristen, Sisk Cheryl, Kiump Kelly An association of early puberty with disordered eating and anxiety in a population of undergraduate women and men. HORMONES AND BEHAVIOR. 2007;52(4):427-435.

1237. Pearson Carolyn, Combs Jessica, Zapolslci Tamika, Smith Gregory A Longitudinal Transactional Risk Model for Early Eating Disorder Onset. JOURNAL OF ABNORMAL

PSYCHOLOGY. 2012;121(3):707-718.

1238. Kass Andrea, Kolko Rachel, Wilfley Denise Psychological treatments for eating disorders. CURRENT OPINION IN PSYCHIATRY. 2013;26(6):549-555.

1239. Nagata T, Kawarada Y, Kiriike N, Iketani T Multi-impulsivity of Japanese patients with eating disorders: primary and secondary impulsivity. PSYCHIATRY RESEARCH. 2000;94(3):239-250.

1240. Treasure J. The time is right to launch large-scale controlled treatment effectiveness studies of early-onset binge eating disorders and bulimia nervosa in student populations. EPIDEMIOLOGY AND PSYCHIATRIC SCIENCES. 2014;23(1):47-49.

1241. Barry DT, Grilo CM, Masheb RM Gender differences in patients with binge eating disorder. INTERNATIONAL JOURNAL OF EATING DISORDERS. 2002;31(1):63-70.

1242. Fahrenholtz Ida, Melin Anna, Wasserfurth Paulina, Stenling Andreas, Logue Danielle, Garthe Ina, Koehler Karsten, Grafnings Maria, Lichtenstein Mia, Madigan Sharon, Torstveit Monica Risk of Low Energy Availability, Disordered Eating, Exercise Addiction, and Food Intolerances in Female Endurance Athletes. FRONTIERS IN SPORTS AND ACTIVE LIVING. 2022;4():.

1243. Larson Nicole, Loth Katie, Eisenberg Marla, Hazzard Vivienne, Neumark-Sztainer Dianne Body dissatisfaction and disordered eating are prevalent problems among US young people from diverse socioeconomic backgrounds: Findings from the EAT 2010-2018 study. EATING BEHAVIORS. 2021;42():.

1244. Norberg Melissa, Handford Charlotte, Magson Natasha, Basten Christopher Reevaluating Cue Exposure and Response Prevention in a Pilot Study: An Updated Treatment for Binge Eating Disorder. BEHAVIOR THERAPY. 2021;52(1):195-207.

1245. Huke Vanessa, Turk Jeremy, Saeidi Saeideh, Kent Andy, Morgan John. Autism Spectrum Disorders in Eating Disorder Populations: A Systematic Review. EUROPEAN EATING DISORDERS REVIEW. 2013;21(5):345-351.

1246. Wade TD, Bulik CM, Sullivan PF, Neale MC, Kendler KS The relation between risk factors for binge eating and bulimia nervosa: A population-based female twin study.

HEALTH PSYCHOLOGY. 2000;19(2):115-123.

1247. Elran-Barak Roni, Bromberg Michal, Shimony Tal, Dichtiar Rita, Mery Nisim, Nitsan Lesley, Keinan-Boker Lital Disordered eating among Arab and Jewish youth in Israel: the role of eating dinner with the family. ISRAEL JOURNAL OF HEALTH POLICY RESEARCH. 2020;9(1):.

1248. Mandelli Laura, Arminio Angelo, Atti Anna-Rita, De Ronchi Diana Suicide attempts in eating disorder subtypes: a meta-analysis of the literature employing DSM-IV, DSM-5, or ICD-10 diagnostic criteria. PSYCHOLOGICAL MEDICINE. 2019;49(8):1237-1249.

1249. GARFINKEL PE, LIN E, GOERING P, SPEGG C, GOLDBLOOM DS, KENNEDY S, KAPLAN AS, WOODSIDE DB BULIMIA-NERVOSA IN A CANADIAN COMMUNITY SAMPLE - PREVALENCE AND COMPARISON OF SUBGROUPS. AMERICAN JOURNAL OF PSYCHIATRY. 1995;152(7):1052-1058.

1250. Wilfley Denise, Crow Scott, Hudson James, Mitchell James, Berkowitz Robert, Blakesley Vicky, Walsh B., Grp Sibutramine Efficacy of sibutramine for the treatment of binge eating disorder: A randomized multicenter placebo-controlled double-blind study. AMERICAN JOURNAL OF PSYCHIATRY. 2008;165(1):51-58.

1251. Atalayer Deniz, Gibson Charlisa, Konopacka Alexandra, Geliebter Allan Ghrelin and eating disorders. PROGRESS IN NEURO-PSYCHOPHARMACOLOGY \& BIOLOGICAL PSYCHIATRY. 2013;40():70-82.

1252. Dingemans Alexandra, Danner Unna, Parks Melissa Emotion Regulation in Binge Eating Disorder: A Review. NUTRIENTS. 2017;9(11):.

1253. Goldschmidt Andrea, Crosby Ross, Cao Li, Moessner Markus, Forbush Kelsie, Accurso Erin, Le Grange Daniel Network Analysis of Pediatric Eating Disorder Symptoms in a Treatment-Seeking, Transdiagnostic Sample. JOURNAL OF ABNORMAL PSYCHOLOGY. 2018;127(2):251-264.

1254. Taylor Jacquelyn, Caldwell Cleopatra, Baser Raymond, Faison Nakesha, Jackson James Prevalence of eating disorders among Blacks in the national survey of American life. INTERNATIONAL JOURNAL OF EATING DISORDERS. 2007;40(S):S10-S14.

1255. Blesa MPG, Lopez-Torres JD, Garcia YD, Honrubia MCG, Rabadan FE, Ramirez MM Detection of bulimia nervosa in primary health care consultations. ACTAS ESPANOLAS DE PSIQUIATRIA. 2003;31(3):129-132.

1256. Nieto-Martinez Ramfis, Gonzalez-Rivas Juan, Medina-Inojosa Jose, Florez Hermes Are Eating Disorders Risk Factors for Type 2 Diabetes? A Systematic Review and Meta-analysis. CURRENT DIABETES REPORTS. 2017;17(12):.

1257. Garcia-Rubio Mauricio, Otero-Cerdeira Maria, Toledo-Lozano Christian, Alcaraz-Estrada Sofia, Suarez-Cuenca Juan, Coral-Vazquez Ramon, Mondragon-Teran Paul, Pineda-Juarez Juan, Diaz-Lopez Luis, Garcia Silvia Analysis of Impulse Control Disorders (ICDs) and Factors Associated with Their Development in a Parkinson's Disease Population. HEALTHCARE. 2021;9(10):.

1258. Cassin SE, Ranson KM Personality and eating disorders: A decade in review. CLINICAL PSYCHOLOGY REVIEW. 2005;25(7):895-916.

1259. Linardon Jake, Messer Mariel, Shatte Adrian, Skvarc David, Rosato John, Rathgen April, Fuller-Tyszkiewicz Matthew Targeting dietary restraint to reduce binge eating: a randomised controlled trial of a blended internet- and smartphone app-based intervention. PSYCHOLOGICAL MEDICINE. 2023;53(4):1277-1287.

1260. Lydecker Janet, Grilo Carlos Different Yet Similar: Examining Race and Ethnicity in Treatment-Seeking Adults With Binge Eating Disorder. JOURNAL OF CONSULTING AND CLINICAL PSYCHOLOGY. 2016;84(1):88-94.

1261. Efthymiou Dimitris, Kokokiris Lampros, Mesiari Christina, Vassilopoulou Emilia Perceived ideal body weight exacerbates bulimia and dieting in Bodybuilding Athletes. TOXICOLOGY REPORTS. 2021;8():1777-1782.

1262. Watson H., Von Holle A., Hamer R., Berg C., Torgersen L., Magnus P., Stoltenberg C., Sullivan P., Reichborn-Kjennerud T., Bulik C. Remission, continuation and incidence of eating disorders during early pregnancy: a validation study in a population-based birth cohort. PSYCHOLOGICAL MEDICINE. 2013;43(8):1723-1734.

1263. Wu Ya-Ke, Brownley Kimberly, Bardone-Cone Anna, Bulik Cynthia, Baker Jessica Associations of Stress and Appetite Hormones with Binge Eating in Females with Anorexia Nervosa after Weight Restoration: A Longitudinal Study. JOURNAL OF PERSONALIZED

MEDICINE. 2021;11(10):.

1264. Goncalves Sonia, Machado Barbara, Silva Catia, Crosby Ross, Lavender Jason, Cao Li, Machado Paulo The Moderating Role of Purging Behaviour in the Relationship Between Sexual/Physical Abuse and Nonsuicidal Self-Injury in Eating Disorder Patients. EUROPEAN EATING DISORDERS REVIEW. 2016;24(2):164-168.

1265. Arthur-Cameselle Jessyca, Quatromoni Paula A Qualitative Analysis of Female Collegiate Athletes' Eating Disorder Recovery Experiences. SPORT PSYCHOLOGIST. 2014;28(4):334-346.

1266. Troop NA, Bifulco A Childhood social arena and cognitive sets in eating disorders. BRITISH JOURNAL OF CLINICAL PSYCHOLOGY. 2002;41(2):205-211.

1267. Holzer Sarah, Uppala Saritha, Wonderlich Stephen, Crosby Ross, Simonich Heather Mediational significance of PTSD in the relationship of sexual trauma and eating disorders. CHILD ABUSE \& NEGLECT. 2008;32(5):561-566.

1268. VANDERHAM T, VANSTRIEN DC, VANENGELAND H A 4-YEAR PROSPECTIVE FOLLOW-UP-STUDY OF 49 EATING-DISORDERED ADOLESCENTS - DIFFERENCES IN COURSE OF ILLNESS. ACTA PSYCHIATRICA SCANDINAVICA. 1994;90(3):229-235.

1269. Sonneville Kendrin, Grilo Carlos, Richmond Tracy, Thurston Idia, Jernigan Maryam, Gianini Loren, Field Alison Prospective Association Between Overvaluation of Weight and Binge Eating Among Overweight Adolescent Girls. JOURNAL OF ADOLESCENT HEALTH. 2015;56(1):25-29.

1270. Michalska Aneta, Szejko Natalia, Jakubczyk Andrzej, Wojnar Marcin Nonspecific eating disorders - a subjective review. PSYCHIATRIA POLSKA. 2016;50(3):497-507.

1271. Anstine D, Grinenko D Rapid screening for disordered eating in college-aged females in the primary care setting. JOURNAL OF ADOLESCENT HEALTH. 2000;26(5):338-342.

1272. Peric Mia, Zenic Natasa, Sekulic Damir, Kondric Miran, Zaletel Petra DISORDERED EATING, AMENORRHEA, AND SUBSTANCE USE AND MISUSE AMONG PROFESSIONAL BALLET DANCERS: PRELIMINARY ANALYSIS. MEDYCYNA PRACY. 2016;67(1):21-27.

1273. SCHENCK CH, HURWITZ TD, OCONNOR KA, MAHOWALD MW ADDITIONAL CATEGORIES OF SLEEP-RELATED EATING DISORDERS AND THE CURRENT STATUS OF TREATMENT. SLEEP. 1993;16(5):457-466.

1274. Knightsmith Pooky, Sharpe Helen, Breen Olivia, Treasure Janet, Schmidt Ulrike `My teacher saved my life' versus `Teachers don't have a clue': an online survey of pupils' experiences of eating disorders. CHILD AND ADOLESCENT MENTAL HEALTH. 2014;19(2):131-137.

1275. Aman Marya, Coelho Jennifer, Lin Boyee, Lu Cynthia, Westwell-Roper Clara, Best John, Stewart S. Prevalence of pediatric acute-onset neuropsychiatric syndrome (PANS) in children and adolescents with eating disorders. JOURNAL OF EATING DISORDERS. 2022;10(1):.

1276. Tannous Wadad, Hay Phillipa, Girosi Federico, Heriseanu Andreea, Ahmed Moin, Touyz Stephen The economic cost of bulimia nervosa and binge eating disorder: a population-based study. PSYCHOLOGICAL MEDICINE. 2022;52(16):3924-3938.

1277. Hilbert Anja Childhood Eating and Feeding Disturbances. NUTRIENTS. 2020;12(4):.

1278. Wang Youfa, Jahns Lisa, Tussing-Humphreys Lisa, Xie Bin, Rockett Helaine, Liang Huifang, Johnson Luann Dietary Intake Patterns of Low-Income Urban African-American Adolescents. JOURNAL OF THE AMERICAN DIETETIC ASSOCIATION. 2010;110(9):1340-1345.

1279. Staples Cody, Rancourt Diana Testing the interaction of thinness/restriction and negative affect reduction expectancies on disordered eating behavior. EATING BEHAVIORS. 2022;47():.

1280. Guarino Roberta, Pellai Alberto, Bassoli Luca, Cozzi Mario, Di Sanzo Maria, Campra Daniela, Guala Andrea Overweight, thinness, body self-image and eating strategies of 2,121 Italian teenagers. THESCIENTIFICWORLDJOURNAL. 2005;5():812-819.

1281. Nobles Carrie, Thomas Jennifer, Valentine Sarah, Gerber Monica, Vaewsorn Adin, Marques Luana Association of premenstrual syndrome and premenstrual dysphoric disorder with bulimia nervosa and binge-eating disorder in a nationally representative epidemiological sample. INTERNATIONAL JOURNAL OF EATING DISORDERS.

2016;49(7):641-650.

1282. Zwaan Martina Should we Use New Media in the Treatment of Eating Disorders?. PSYCHOTHERAPIE PSYCHOSOMATIK MEDIZINISCHE PSYCHOLOGIE. 2015;65(1):30-32.

1283. Godart NT, Perdereau F, Jeammet P, Flament MF Comorbidity and time of occurrence of anxiety disorders in eating disorders. ANNALES MEDICO-PSYCHOLOGIQUES. 2003;161(7):498-503.

1284. Kauffman Brooke, Garey Lorra, Zvolensky Michael Psychological inflexibility in terms of eating behaviors among individuals seeking treatment for comorbid affective vulnerabilities and weight-related behaviors. JOURNAL OF CONTEXTUAL BEHAVIORAL SCIENCE. 2022;26():168-172.

1285. Schienle Anne, Schaefer Axel, Hermann Andrea, Vaitl Dieter Binge-Eating Disorder: Reward Sensitivity and Brain Activation to Images of Food. BIOLOGICAL PSYCHIATRY. 2009;65(8):654-661.

1286. Toselli AL, Villani S, Ferro AM, Verri A, Cucurullo L, Marinoni A Eating disorders and their correlates in high school adolescents of Northern Italy. EPIDEMIOLOGIA E PSICHIATRIA SOCIALE. 2005;14(2):91-99.

1287. Blachno Magda, Brynska Anita Comorbidity and characteristic of obsessive-compulsive symptoms in anorexia nervosa. PSYCHIATRIA POLSKA. 2012;46(6):1019-1028.

1288. Angst J, Gamma A, Endrass J, Goodwin R, Ajdacic V, Eich D, Rossler W Obsessive-compulsive severity spectrum in the community: prevalence, comorbidity, and course. EUROPEAN ARCHIVES OF PSYCHIATRY AND CLINICAL NEUROSCIENCE. 2004;254(3):156-164.

1289. Grilo CM, Masheb RM Childhood psychological, physical, and sexual maltreatment in outpatients with binge eating disorder: Frequency and associations with gender, obesity, and eating-related psychopathology. OBESITY RESEARCH. 2001;9(5):320-325.

1290. Hilbert Anja, Brauhardt Anne Childhood Loss of Control Eating Over Five-Year Follow-Up. INTERNATIONAL JOURNAL OF EATING DISORDERS. 2014;47(7, SI):758-761.

1291. Leon GR, Fulkerson JA, Perry CL, Keel PK, Klump KL Three to four year prospective evaluation of personality and behavioral risk factors for later disordered eating in adolescent girls and boys. JOURNAL OF YOUTH AND ADOLESCENCE. 1999;28(2):181-196.

1292. Bauer Stephanie, Kindermann Sally, Moessner Markus Prevention of eating disorder: a review. ZEITSCHRIFT FUR KINDER-UND JUGENDPSYCHIATRIE UND PSYCHOTHERAPIE. 2017;45(5):403-411.

1293. Spielmans Glen, Benish Steven, Marin Catherine, Bowman Wesley, Menster Maria, Wheeler Anthony Specificity of psychological treatments for bulimia nervosa and binge eating disorder? A meta-analysis of direct comparisons. CLINICAL PSYCHOLOGY REVIEW. 2013;33(3):460-469.

1294. Flynn Michaela, Austin Amelia, Lang Katie, Allen Karina, Bassi Ranjeet, Brady Gabrielle, Brown Amy, Connan Frances, Franklin-Smith Mary, Glennon Danielle, Grant Nina, Jones William, Kali Kuda, Koskina Antonia, Mahony Kate, Mountford Victoria, Nunes Nicole, Schelhase Monique, Serpell Lucy, Schmidt Ulrike Assessing the impact of First Episode Rapid Early Intervention for Eating Disorders on duration of untreated eating disorder: A multi-centre quasi-experimental study. EUROPEAN EATING DISORDERS REVIEW. 2021;29(3, SI):458-471.

1295. Vervaet M, Heeringen C, Audenaert K Personality-related characteristics in restricting versus binging and purging eating disordered patients. COMPREHENSIVE PSYCHIATRY. 2004;45(1):37-43.

1296. Mond JM, Hay PJ, Rodgers B, Owen C, Mitchell JE Correlates of self-induced vomiting and laxative misuse in a community sample of women. JOURNAL OF NERVOUS AND MENTAL DISEASE. 2006;194(1):40-46.

1297. Ben Othman Rym, Berriche Olfa, Gamoudi Amel, Mizouri Ramla, Jerab Donia, Ben Amor Nadia, Mahjoub Faten, Jamoussi Henda Cross sectional study about nutritional risk factors of metabolically unhealthy obesity. ROMANIAN JOURNAL OF INTERNAL MEDICINE. 2023;61(1):53-62.

1298. Mumtaz Sara, Farhat Syeda, Saeed Rida, Younis Sidra, Ali Mahwish Binge eating disorder during COVID-19. OPEN LIFE SCIENCES. 2022;17(1):321-322.

1299. Schag Kathrin, Leehr Elisabeth, Skoda Eva-Maria, Becker Sandra, Zipfel Stephan, Giel Katrin Impulsivity-focused Group Intervention to reduce Binge Eating Episodes in Patients with Binge Eating Disorder - A Group Training Program. PSYCHOTHERAPIE PSYCHOSOMATIK MEDIZINISCHE PSYCHOLOGIE. 2016;66(11):449-454.

1300. Berg Kelly, Cao Li, Crosby Ross, Engel Scott, Peterson Carol, Crow Scott, Le Grange Daniel, Mitchell James, Lavender Jason, Durkin Nora, Wonderlich Stephen Negative affect and binge eating: Reconciling differences between two analytic approaches in ecological momentary assessment research. INTERNATIONAL JOURNAL OF EATING DISORDERS. 2017;50(10):1222-1230.

1301. Royen Annelies, Malderen Eva, Desmeta Maurane, Goossens Lien, Verbeken Sandra, Kemps Eva Go or no-go? An assessment of inhibitory control training using the GO/NO-GO task in adolescents. APPETITE. 2022;179():.

1302. Daniele A., Guarini A., Summa S., Dellino M., Lerario G., Ciavarella S., Ditonno P., Paradiso A., Divella R., Casamassima P., Savino E., Carbonara M., Minoia C. Body Composition Change, Unhealthy Lifestyles and Steroid Treatment as Predictor of Metabolic Risk in Non-Hodgkin's Lymphoma Survivors. JOURNAL OF PERSONALIZED MEDICINE. 2021;11(3):.

1303. Cyr Marilyn, Wang Zhishun, Tau Gregory, Zhao Guihu, Friedl Eve, Stefan Mihaela, Terranova Kate, Marsh Rachel Reward-Based Spatial Learning in Teens With Bulimia Nervosa. JOURNAL OF THE AMERICAN ACADEMY OF CHILD AND ADOLESCENT PSYCHIATRY. 2016;55(11):962-971.

1304. Scott Charlotte, Plateau Carolyn, Haycraft Emma Teammate influences, psychological well-being, and athletes' eating and exercise psychopathology: A moderated mediation analysis. INTERNATIONAL JOURNAL OF EATING DISORDERS. 2020;53(4):564-573.

1305. Santonastaso Paolo, Zanetti Tatiana, De Antoni Chiara, Tenconi Elena, Favaro Angela Anorexia nervosa patients with a prior history of bulimia nervosa. COMPREHENSIVE PSYCHIATRY. 2006;47(6):519-522.

1306. Pennesi Jamie-Lee, Wade Tracey Imagery rescripting and cognitive dissonance: A randomized controlled trial of two brief online interventions for women at risk of developing an eating disorder. INTERNATIONAL JOURNAL OF EATING DISORDERS. 2018;51(5):439-448.

1307. Mousavi Asl Esmaeil, Abdi Leila, Sadegh Amirali, Behrouzian Forouzan The mediating role of self-compassion in the relationship between positive reactivity, negative reactivity, and perfectionism with disordered eating. JOURNAL OF EDUCATION AND HEALTH PROMOTION. 2021;10(1):.

1308. Fitzgerald Amanda, Heary Caroline, Kelly Colette, Nixon Elizabeth, Shevlin Mark Self-efficacy for healthy eating and peer support for unhealthy eating are associated with adolescents' food intake patterns. APPETITE. 2013;63():48-58.

1309. Pryor T, Wiederman MW, McGilley B Clinical correlates of anorexia nervosa subtypes. INTERNATIONAL JOURNAL OF EATING DISORDERS. 1996;19(4):371-379.

1310. Hirte Teresa, Fellendorf Frederike, Unterrainer Human-Friedrich, Dalkner Nina, Bengesser Susanne, Birner Armin, Platzer Martina, Queissner Robert, Holasek Sandra, Schwerdtfeger Andreas, Reininghaus Eva Eating Habits and Eating Disorder Associated Behavior in Bipolar Disorder. JOURNAL OF NERVOUS AND MENTAL DISEASE. 2022;210(12):930-934.

1311. Lydecker Janet, Grilo Carlos Children of parents with BED have more eating behavior disturbance than children of parents with obesity or healthy weight. INTERNATIONAL JOURNAL OF EATING DISORDERS. 2017;50(6):648-656.

1312. Watson Hunna, Zerwas Stephanie, Torgersen Leila, Gustavson Kristin, Diemer Elizabeth, Knudsen Gun, Reichborn-Kjennerud Ted, Bulik Cynthia Maternal Eating Disorders and Perinatal Outcomes: A Three-Generation Study in the Norwegian Mother and Child Cohort Study. JOURNAL OF ABNORMAL PSYCHOLOGY. 2017;126(5):552-564.

1313. Maillard A., Hippolyte L., Rodriguez-Herreros B., Chawner S., Dremmel D., Aguera Z., Fagundo A., Pain A., Martin-Brevet S., Hilbert A., Kurz S., Etienne R., Draganski B., Jimenez-Murcia S., Mannik K., Metspalu A., Reigo A., Isidor B., Le Caignec C., David A., Mignot C., Keren B., Bree M., Munsch S., Fernandez-Aranda F., Beckmann J., Reymond A., Jacquemont S., 2 European Consortium 16p11.2 Locus modulates response to satiety before the onset of obesity. INTERNATIONAL JOURNAL OF OBESITY. 2016;40(5):870-876.

1314. Mirch Margaret, McDuffie Jennifer, Yanovski Susan, Schollnberger Merel, Tanofsky-Kraff Marian, Theim Kelly, Krakoff Jonathan, Yanovski Jack Effects of binge eating on satiation, satiety, and energy intake of overweight children. AMERICAN JOURNAL OF CLINICAL NUTRITION. 2006;84(4):732-738.

1315. Berger U., Schaefer J., Wick K., Brix C., Bormann B., Sowa M., Schwartze D., Strauss B. Effectiveness of Reducing the Risk of Eating-Related Problems Using the German School-Based Intervention Program, ``Torera{''}, for Preadolescent Boys and Girls. PREVENTION SCIENCE. 2014;15(4):557-569.

1316. Grilo CM, Masheb RM, Brody M, Burke-Martindale CH, Rothschild BS Binge eating and self-esteem predict body image dissatisfaction among obese men and women seeking bariatric surgery. INTERNATIONAL JOURNAL OF EATING DISORDERS. 2005;37(4):347-351.

1317. O' Reardon JP, Peshek A, Allison KC Night eating syndrome - Diagnosis, epidemiology and management. CNS DRUGS. 2005;19(12):997-1008.

1318. Fichter MM, Quadflieg N Six-year course and outcome of anorexia nervosa. INTERNATIONAL JOURNAL OF EATING DISORDERS. 1999;26(4):359-385.

1319. Linardon Jake, Messer Mariel Reciprocal Associations Between Self-compassion and Eating Disorder Symptoms: an 8-month Longitudinal Study. MINDFULNESS. 2023;14(1):141-147.

1320. Godart NT, Flament MF, Lecrubier Y, Jeammet P Anxiety disorders in anorexia nervosa and bulimia nervosa: co-morbidity and chronology of appearance. EUROPEAN PSYCHIATRY. 2000;15(1):38-45.

1321. Lynch Frances, Striegel-Moore Ruth, Dickerson John, Perrin Nancy, DeBar Lynn, Wilson G., Kraemer Helena Cost-Effectiveness of Guided Self-Help Treatment for Recurrent Binge Eating. JOURNAL OF CONSULTING AND CLINICAL PSYCHOLOGY. 2010;78(3):322-333.

1322. Lie Selma, Ro Oyvind, Bang Lasse Is bullying and teasing associated with eating disorders? A systematic review and meta-analysis. INTERNATIONAL JOURNAL OF EATING DISORDERS. 2019;52(5):497-514.

1323. Davis Caroline, Levitan Robert, Carter Jacqueline, Kaplan Allan, Reid Caroline, Curtis Claire, Patte Karen, Kennedy James Personality and eating behaviors: A case-control study of binge eating disorder. INTERNATIONAL JOURNAL OF EATING DISORDERS. 2008;41(3):243-250.

1324. Guzel Amine, Mutlu Naz, Molendijk Marc COVID-19-related changes in eating disorder pathology, emotional and binge eating and need for care: a systematic review with frequentist and Bayesian meta-analyses. EATING AND WEIGHT DISORDERS-STUDIES ON ANOREXIA BULIMIA AND OBESITY. 2023;28(1):.

1325. Keel PK, Dorer DJ, Franko DL, Jackson SC, Herzog DB Postremission predictors of relapse in women with eating disorders. AMERICAN JOURNAL OF PSYCHIATRY. 2005;162(12):2263-2268.

1326. Bowden David, Kilburn-Toppin Fleur, Scoffings Daniel Radiology of Eating Disorders: A Pictorial Review. RADIOGRAPHICS. 2013;33(4):1171-1193.

1327. Cororve MB, Gleaves DH Body dysmorphic disorder: A review of conceptualizations, assessment, and treatment strategies. CLINICAL PSYCHOLOGY REVIEW. 2001;21(6):949-970.

1328. Pederson KJ, Roerig JL, Mitchell JE Towards the pharmacotherapy of eating disorders. EXPERT OPINION ON PHARMACOTHERAPY. 2003;4(10):1659-1678.

1329. Rayworth BB, Wise LA, Harlow BL Childhood abuse and risk of eating disorders in women. EPIDEMIOLOGY. 2004;15(3):271-278.

1330. Gleaves DH, Brown JD, Warren CS The continuity/discontinuity models of eating disorders - A review of the literature and implications for assessment, treatment, and prevention. BEHAVIOR MODIFICATION. 2004;28(6):739-762.

1331. Emami Ashley, Woodcock Anna, Swanson Heidi, Kapphahn Teresa, Pulvers Kim Distress tolerance is linked to unhealthy eating through pain catastrophizing. APPETITE. 2016;107():454-459.

1332. Niviere P., Fonseca D., Deruelle C., Bat-Pitault F. Use of virtual reality in eating disorders. ENCEPHALE-REVUE DE PSYCHIATRIE CLINIQUE BIOLOGIQUE ET THERAPEUTIQUE. 2021;47(3):263-269.

1333. HEEBINK DM, SUNDAY SR, HALMI KA ANOREXIA-NERVOSA AND BULIMIA-NERVOSA IN ADOLESCENCE - EFFECTS OF AGE AND MENSTRUAL STATUS ON PSYCHOLOGICAL VARIABLES. JOURNAL OF THE AMERICAN ACADEMY OF CHILD AND ADOLESCENT

PSYCHIATRY. 1995;34(3):378-382.

1334. Parylak Sarah, Koob George, Zorrilla Eric The dark side of food addiction. PHYSIOLOGY \& BEHAVIOR. 2011;104(1, SI):149-156.

1335. Masheb RM, Grilo CM On the relation of attempting to lose weight, restraint, and binge eating in outpatients with binge eating disorder. OBESITY RESEARCH. 2000;8(9):638-645.

1336. Paans Nadine, Bot Mariska, Brouwer Ingeborg, Visser Marjolein, Roca Miguel, Kohls Elisabeth, Watkins Ed, Penninx Brenda The association between depression and eating styles in four European countries: The MooDFOOD prevention study. JOURNAL OF PSYCHOSOMATIC RESEARCH. 2018;108():85-92.

1337. Howard Lindsay, Heron Kristin, Cramer Robert The Deliberate Denial of Disordered Eating Behaviors Scale: Development and Initial Validation in Young Women with Subclinical Disordered Eating. JOURNAL OF PSYCHOPATHOLOGY AND BEHAVIORAL ASSESSMENT. 2020;42(4):774-786.

1338. Wilksch Simon, Starkey Karina, Gannoni Anne, Kelly Tania, Wade Tracey Interactive programme to enhance protective factors for eating disorders in girls with type 1 diabetes. EARLY INTERVENTION IN PSYCHIATRY. 2013;7(3):315-321.

1339. Chaitoff Alexander, Swetlik Carol, Ituarte Catherine, Pfoh Elizabeth, Lee Ling-Ling, Heinberg Leslie, Rothberg Michael Associations Between Unhealthy Weight-Loss Strategies and Depressive Symptoms. AMERICAN JOURNAL OF PREVENTIVE MEDICINE. 2019;56(2):241-250.

1340. Prud'homme Julie, Hofer Marlise, Ames Megan, Turner Brianna Disparities in the prevalence, frequency and trajectories of substance use and disordered eating across first-year university in sexual minority undergraduates. JOURNAL OF AMERICAN COLLEGE HEALTH. 2022;():.

1341. Lazaro L, Toro J, Canalda G, Castro J, Martinez E, Puig J Clinical, psychological and biological variables in a group of 108 adolescent patients with anorexia nervosa. MEDICINA CLINICA. 1996;107(5):169-174.

1342. Volpe Umberto, Monteleone Alessio, Ricca Valdo, Corsi Elisa, Favaro Angela, Santonastaso Paolo, De Giorgi Serafino, Renna Caterina, Daga Giovanni, Amianto Federico, Balestrieri Matteo, Luxardi Gian, Clerici Massimo, Alamia Alberto, Segura-Garcia Cristina, Rania Marianna, Monteleone Palmiero, Maj Mario Pathways to specialist care for eating disorders: An Italian multicentre study. EUROPEAN EATING DISORDERS REVIEW. 2019;27(3):274-282.

1343. Mason Tyler, Smith Kathryn, Anderson Lisa, Hazzard Vivienne Anhedonia, positive affect dysregulation, and risk and maintenance of binge-eating disorder. INTERNATIONAL JOURNAL OF EATING DISORDERS. 2021;54(3):287-292.

1344. Bar Rachel, Cassin Stephanie, Dionne Michelle The long-term impact of an eating disorder prevention program for professional ballet school students: A 15-year follow-up study. EATING DISORDERS. 2017;25(5):375-387.

1345. Kent A, Waller G, Dagnan D A greater role of emotional than physical or sexual abuse in predicting disordered eating attitudes: The role of mediating variables. INTERNATIONAL JOURNAL OF EATING DISORDERS. 1999;25(2):159-167.

1346. Tanofsky-Kraff M, Cohen ML, Yanovski SZ, Cox C, Theim KR, Keil M, Reynolds JC, Yanovski JA A prospective study of psychological predictors of body fat gain among children at high risk for adult obesity. PEDIATRICS. 2006;117(4):1203-1209.

1347. Godart N, Flament M, Curt F, Perdereau E, Lang F, Venisse JL, Halfon O, Bizourad P, Loas G, Corcos M, Jeammet P, Fermanian J Anxiety disorders in subjects seeking treatment for eating disorders. A DSM-IV controlled study. ANNALES DE MEDECINE INTERNE. 2003;154(4):209-218.

1348. Lazzeri Maria, Mastorci Francesca, Piaggi Paolo, Doveri Cristina, Casu Anselmo, Trivellini Gabriele, Marinaro Irene, Bardelli Andrea, Pingitore Alessandro The Impact of Unhealthy Behaviors on Personalized Well-Being Index in a Sample of School Dropout Adolescents. CHILDREN-BASEL. 2022;9(8):.

1349. Guerdjikova Anna, Blom Thomas, Mori Nicole, Casuto Leah, Keck Paul, McElroy Susan Gender Differences in Binge Eating Disorder: A Pooled Analysis of Eleven Pharmacotherapy Trials from One Research Group. JOURNAL OF MENS HEALTH. 2014;11(4):183-188.

1350. Chapa Danielle, Bohrer Brittany, Forbush Kelsie Is the diagnostic threshold for bulimia nervosa clinically meaningful?. EATING BEHAVIORS. 2018;28():16-19.

1351. Mond JM, Hay PJ, Rodgers B, Owen C, Beumont PJV Relationships between exercise behaviour, eating-disordered behaviour and quality of life in a community sample of women: When is exercise `excessive'?. EUROPEAN EATING DISORDERS REVIEW. 2004;12(4):265-272.

1352. Schwitzer AM, Rodriguez LE, Thomas C, Salimi L The eating disorders NOS diagnostic profile among college women. JOURNAL OF AMERICAN COLLEGE HEALTH. 2001;49(4):157-166.

1353. Flament Martine, Bissada Hany, Spettigue Wendy Evidence-based pharmacotherapy of eating disorders. INTERNATIONAL JOURNAL OF NEUROPSYCHOPHARMACOLOGY. 2012;15(2):189-207.

1354. Schlegl Sandra, Buerger Carolina, Schmidt Luise, Herbst Nirmal, Voderholzer Ulrich The Potential of Technology-Based Psychological Interventions for Anorexia and Bulimia Nervosa: A Systematic Review and Recommendations for Future Research. JOURNAL OF MEDICAL INTERNET RESEARCH. 2015;17(3):.

1355. Vincent MA, McCabe MP, Ricciardelli LA Factorial validity of the Bulimia Test-Revised in adolescent boys and girls. BEHAVIOUR RESEARCH AND THERAPY. 1999;37(11):1129-1140.

1356. Neziroglu F, Hsia C, Yaryura-Tobias JA Behavioral, cognitive, and family therapy for obsessive-compulsive and related disorders. PSYCHIATRIC CLINICS OF NORTH AMERICA. 2000;23(3):657+.

1357. Neumark-Sztainer Dianne, Wall Melanie, Eisenberg Marla, Story Mary, Hannan Peter Overweight status and weight control behaviors in adolescents: Longitudinal and secular trends from 1999 to 2004. PREVENTIVE MEDICINE. 2006;43(1):52-59.

1358. Amrtavarshini R., Ghosh Sreyoshi, Seshadri Shekar Anorexia nervosa - Binge - eating/purging type in an adolescent boy: A case report. JOURNAL OF INDIAN ASSOCIATION FOR CHILD AND ADOLESCENT MENTAL HEALTH. 2021;17(1):130-136.

1359. Udo Tomoko, Grilo Carlos Prevalence and Correlates of DSM-5-Defined Eating Disorders in a Nationally Representative Sample of US Adults. BIOLOGICAL PSYCHIATRY. 2018;84(5):345-354.

1360. Morgante Francesca, Fasano Alfonso, Ginevrino Monia, Petrucci Simona, Ricciardi Lucia, Bove Francesco, Criscuolo Chiara, Moccia Marcello, De Rosa Anna, Sorbera Chiara, Bentivoglio Anna, Barone Paolo, De Michele Giuseppe, Pellecchia Maria, Valente Enza Impulsive-compulsive behaviors in parkin-associated Parkinson disease. NEUROLOGY. 2016;87(14):1436-1441.

1361. Fitzsimmons-Craft Ellen, Eichen Dawn, Kass Andrea, Trockel Mickey, Crosby Ross, Taylor C., Wilfley Denise Reciprocal longitudinal relations between weight/shape concern and comorbid pathology among women at very high risk for eating disorder onset. EATING AND WEIGHT DISORDERS-STUDIES ON ANOREXIA BULIMIA AND OBESITY. 2019;24(6):1189-1198.

1362. Brunault Paul, Frammery Julie, Montaudon Pauline, De Luca Arnaud, Hankard Regis, Ducluzeau Pierre, Cortese Samuele, Ballon Nicolas Adulthood and childhood ADHD in patients consulting for obesity is associated with food addiction and binge eating, but not sleep apnea syndrome. APPETITE. 2019;136():25-32.

1363. Baer RA, Fischer S, Huss DB Mindfulness-based cognitive therapy applied to binge eating: A case study. COGNITIVE AND BEHAVIORAL PRACTICE. 2005;12(3):351-358.

1364. Mond JM, Hay PJ, Rodgers B, Owen C, Beumont PJV Beliefs of women concerning the severity and prevalence of bulimia nervosa. SOCIAL PSYCHIATRY AND PSYCHIATRIC EPIDEMIOLOGY. 2004;39(4):299-304.

1365. Miniati Mario, Callari Antonio, Maglio Alessandra, Calugi Simona Interpersonal psychotherapy for eating disorders: current perspectives. PSYCHOLOGY RESEARCH AND BEHAVIOR MANAGEMENT. 2018;11():353-369.

1366. Koskina Antonia, Campbell Iain, Schmidt Ulrike Exposure therapy in eating disorders revisited. NEUROSCIENCE AND BIOBEHAVIORAL REVIEWS. 2013;37(2):193-208.

1367. Leenaerts Nicolas, Jongen Danielle, Ceccarini Jenny, Van Oudenhove Lukas, Vrieze Elske The neurobiological reward system and binge eating: A critical systematic review of neuroimaging studies. INTERNATIONAL JOURNAL OF EATING DISORDERS.

2022;55(11):1421-1458.

1368. Dunne Julie, Shindul-Rothschild Judith, White Laura, Lee Christopher, Wolfe Barbara Mindfulness in persons with anorexia nervosa and the relationships between eating disorder symptomology, anxiety and pain. EATING DISORDERS. 2021;29(5):497-508.

1369. Sonneville Kendrin, Horton Nicholas, Micali Nadia, Crosby Ross, Swanson Sonja, Solmi Francesca, Field Alison Longitudinal Associations Between Binge Eating and Overeating and Adverse Outcomes Among Adolescents and Young Adults Does Loss of Control Matter?. JAMA PEDIATRICS. 2013;167(2):149-155.

1370. Rienecke Renee, Johnson Craig, Mehler Philip, Le Grange Daniel, Manwaring Jamie, Duffy Alan, McClanahan Susan, Blalock Dan Adverse childhood experiences among a treatment-seeking sample of adults with eating disorders. EUROPEAN EATING DISORDERS REVIEW. 2022;30(2):156-167.

1371. Chapman James, Woodman Tim Disordered eating in male athletes: a meta-analysis. JOURNAL OF SPORTS SCIENCES. 2016;34(2):101-109.

1372. Ralph-Nearman Christina, Achee Margaret, Lapidus Rachel, Stewart Jennifer, Filik Ruth A systematic and methodological review of attentional biases in eating disorders: Food, body, and perfectionism. BRAIN AND BEHAVIOR. 2019;9(12):.

1373. Brodin Ulf, Zandian Modjtaba, Langlet Billy, Sodersten Per, Anvret Anna, Sjoberg Jennie, Bergh Cecilia A Computer-Based Platform for Aiding Clinicians in Eating Disorder Analysis and Diagnosis. JOVE-JOURNAL OF VISUALIZED EXPERIMENTS. 2022;(183):.

1374. Mussell MP, Mitchell JE, Fenna CJ, Crosby RD, Miller JP, Hoberman HM A comparison of onset of binge eating versus dieting in the development of bulimia nervosa. INTERNATIONAL JOURNAL OF EATING DISORDERS. 1997;21(4):353-360.

1375. Schell Sarah, Brassard Sarah, Racine Sarah Extending the Acquired Preparedness model of binge eating: Testing the indirect effects of high-risk personality traits on binge eating via positive and negative reinforcement expectancies. APPETITE. 2019;140():206-212.

1376. Rasmusson Grace, Lydecker Janet, Coffino Jaime, White Marney, Grilo Carlos Household food insecurity is associated with binge-eating disorder and obesity. INTERNATIONAL JOURNAL OF EATING DISORDERS. 2019;52(1):28-35.

1377. Simone Melissa, Scodes Jennifer, Mason Tyler, Loth Katie, Wall Melanie, Neumark-Sztainer Dianne Shared and non-shared risk and protective factors of binge eating and binge drinking from adolescence to young adulthood. JOURNAL OF HEALTH PSYCHOLOGY. 2021;26(6):805-817.

1378. Nagata T, Kaye WH, Kiriike N, Rao R, McConaha C, Plotnicov KH Physical and sexual abuse histories in patients with eating disorders: A comparison of Japanese and American patients. PSYCHIATRY AND CLINICAL NEUROSCIENCES. 2001;55(4):333-340.

1379. Hautala Lea, Helenius Hans, Karukivi Max, Maunula Aija-Mari, Nieminen Jutta, Aromaa Minna, Liuksila Pirjo-Riitta, Raiha Hannele, Valimaki Maritta, Saarijarvi Simo The role of gender, affectivity and parenting in the course of disordered eating: A 4-year prospective case-control study among adolescents. INTERNATIONAL JOURNAL OF NURSING STUDIES. 2011;48(8):959-972.

1380. Strand Mattias, Gustafsson Sanna Mukbang and Disordered Eating: A Netnographic Analysis of Online Eating Broadcasts. CULTURE MEDICINE AND PSYCHIATRY. 2020;44(4):586-609.

1381. Williamson DA, White MA, York-Crowe E, Stewart TM Cognitive-behavioral theories of eating disorders. BEHAVIOR MODIFICATION. 2004;28(6):711-738.

1382. Stinson Emma, Perez Marisol, Ohrt Tara, Von Schell Anna, Bruening Amanda The association between program credibility, expectancy, and acceptability with baseline pathology and outcome for a body acceptance prevention program. JOURNAL OF CLINICAL PSYCHOLOGY. 2018;74(12):2161-2172.

1383. Scott Charlotte, Haycraft Emma, Plateau Carolyn The impact of critical comments from teammates on athletes' eating and exercise psychopathology. BODY IMAGE. 2022;43():170-179.

1384. Horney Audra, Stice Eric, Rohde Paul An Examination of Participants Who Develop an Eating Disorder Despite Completing an Eating Disorder Prevention Program: Implications

for Improving the Yield of Prevention Efforts. PREVENTION SCIENCE. 2015;16(4):518-526.

1385. NeumarkSztainer D, Butler R, Palti H Personal and socioenvironmental predictors of disordered eating among adolescent females. JOURNAL OF NUTRITION EDUCATION. 1996;28(4):195-201.

1386. Goldschmidt Andrea, Wall Melanie, Choo Tse-Hwei, Evans E., Jelalian Elissa, Larson Nicole, Neumark-Sztainer Dianne Fifteen-year Weight and Disordered Eating Patterns Among Community-based Adolescents. AMERICAN JOURNAL OF PREVENTIVE MEDICINE. 2018;54(1):E19-E27.

1387. Field Alison Is set-shifting a risk factor for anorexia nervosa or a broader range of disordered eating?. INTERNATIONAL JOURNAL OF EATING DISORDERS. 2022;55(3):415-417.

1388. Grilo CM, Masheb RM Correlates of body image dissatisfaction in treatment-seeking men and women with binge eating disorder. INTERNATIONAL JOURNAL OF EATING DISORDERS. 2005;38(2):162-166.

1389. Blomquist Kerstin, Ansell Emily, White Marney, Masheb Robin, Grilo Carlos Interpersonal problems and developmental trajectories of binge eating disorder. COMPREHENSIVE PSYCHIATRY. 2012;53(8):1088-1095.

1390. Hilbert Anja, Hartmann Andrea, Czaja Julia, Schoebi Dominik Natural Course of Preadolescent Loss of Control Eating. JOURNAL OF ABNORMAL PSYCHOLOGY. 2013;122(3):684-693.

1391. Andres Ana, Saldana Carmina Body dissatisfaction and dietary restraint influence binge eating behavior. NUTRITION RESEARCH. 2014;34(11):944-950.

1392. Conceicao Costa Daniel, Assuncao Melissa, Ferrao Ygor, Conrado Luciana, Gonzalez Christina, Fontenelle Leonardo, Fossaluza Victor, Miguel Euripedes, Torres Albina, Shavitt Roseli BODY DYSMORPHIC DISORDER IN PATIENTS WITH OBSESSIVE-COMPULSIVE DISORDER: PREVALENCE AND CLINICAL CORRELATES. DEPRESSION AND ANXIETY. 2012;29(11):966-975.

1393. Brachel Ruth, Hoetzel Katrin, Hirschfeld Gerrit, Rieger Elizabeth, Schmidt Ulrike, Kosfelder Joachim, Hechler Tanja, Schulte Dietmar, Vocks Silja Internet-Based Motivation Program for Women With Eating Disorders: Eating Disorder Pathology and Depressive Mood Predict Dropout. JOURNAL OF MEDICAL INTERNET RESEARCH. 2014;16(3):145-157.

1394. Koposov Roman, Stickley Andrew, Ruchkin Vladislav Bulimia Symptoms in Russian Youth: Prevalence and Association With Internalizing Problems. FRONTIERS IN PSYCHIATRY. 2022;12():.

1395. Carrard I., Fernandez-Aranda F., Lam T., Nevonen L., Liwowsky I., Volkart A., Rouget P., Golay A., Linden M., Norring C. Evaluation of a Guided Internet Self-Treatment Programme for Bulimia Nervosa in Several European Countries. EUROPEAN EATING DISORDERS REVIEW. 2011;19(2):138-149.

1396. Fairburn Christopher, Murphy Rebecca Treating eating disorders using the internet. CURRENT OPINION IN PSYCHIATRY. 2015;28(6):461-467.

1397. Moosavi Mandana, Kreisman Stuart, Hall Lacresha Intentional Hypoglycemia to Control Bingeing in a Patient with Type 1 Diabetes and Bulimia Nervosa. CANADIAN JOURNAL OF DIABETES. 2015;39(1):16-17.

1398. Channa Sunita, Lavis Anna, Connor Charlotte, Palmer Colin, Leung Newman, Birchwood Max Overlaps and Disjunctures: A Cultural Case Study of a British Indian Young Woman's Experiences of Bulimia Nervosa. CULTURE MEDICINE AND PSYCHIATRY. 2019;43(3):361-386.

1399. Roehrig Megan, Masheb Robin, White Marney, Grilo Carlos Dieting Frequency in Obese Patients With Binge Eating Disorder: Behavioral and Metabolic Correlates. OBESITY. 2009;17(4):689-697.

1400. Krug Isabel, King Ross, Youssef George, Sorabji Anisha, Wertheim Eleanor, Le Grange Daniel, Hughes Elizabeth, Letcher Primrose, Olsson Craig The effect of low parental warmth and low monitoring on disordered eating in mid-adolescence: Findings from the Australian Temperament Project. APPETITE. 2016;105():232-241.

1401. Marks Kevin, Thastum Mikael, Jensen Morten, Kristensen Lene, Mose Anne, Pouwer Frans, Birkebaek Niels Overeating, binge eating, quality of life, emotional difficulties, and HbA(1c) in adolescents with type 1 diabetes: A Danish national survey. DIABETES

RESEARCH AND CLINICAL PRACTICE. 2021;182():.

1402. O'Sullivan Sean, Poyares Dalva Augmentation and impulsivity in restless legs syndrome patients: A complex interaction. NEUROLOGY. 2016;87(1):15-16.

1403. Kania Alan, Szlaga Agata, Sambak Patryk, Gugula Anna, Blasiak Ewa, Di Bonaventura Maria, Hossain Mohammad, Cifani Carlo, Hess Grzegorz, Gundlach Andrew, Blasiak Anna RLN3/RXFP3 Signaling in the PVN Inhibits Magnocellular Neurons via M-like Current Activation and Contributes to Binge Eating Behavior. JOURNAL OF NEUROSCIENCE. 2020;40(28):5362-5375.

1404. West Caroline, Goldschmidt Andrea, Mason Susan, Neumark-Sztainer Dianne Differences in risk factors for binge eating by socioeconomic status in a community-based sample of adolescents: Findings from Project EAT. INTERNATIONAL JOURNAL OF EATING DISORDERS. 2019;52(6):659-668.

1405. Mushquash Aislin, McGeown Laura, Mushquash Christopher, McGrath Daniel Which came first? Exploring the reciprocal relations between impulsivity and binge eating. PERSONALITY AND INDIVIDUAL DIFFERENCES. 2019;151():.

1406. Zhou Yuan, Pennesi Jamie-Lee, Wade Tracey Online imagery rescripting among young women at risk of developing an eating disorder: A randomized controlled trial. INTERNATIONAL JOURNAL OF EATING DISORDERS. 2020;53(12):1906-1917.

1407. Boggiano Mary, Wenger Lowell, Mrug Sylvie, Burgess Emilee, Morgan Phillip The Kids-Palatable Eating Motives Scale: Relation to BMI and binge eating traits. EATING BEHAVIORS. 2015;17():69-73.

1408. Nevonen L, Broberg AG The emergence of eating disorders: An exploratory study. EUROPEAN EATING DISORDERS REVIEW. 2000;8(4):279-292.

1409. Hampshire Chloe, Mahoney Berenice, Davis Sarah Parenting Styles and Disordered Eating Among Youths: A Rapid Scoping Review. FRONTIERS IN PSYCHOLOGY. 2022;12():.

1410. Pinhas Leora, Heinmaa Margus, Bryden Pier, Bradley Susan, Toner Brenda Disordered eating in Jewish adolescent girls. CANADIAN JOURNAL OF PSYCHIATRY-REVUE

CANADIENNE DE PSYCHIATRIE. 2008;53(9):601-608.

1411. Mehak Adrienne, Friedman Aliza, Cassin Stephanie Self-objectification, weight bias internalization, and binge eating in young women: Testing a mediational model. BODY IMAGE. 2018;24():111-115.

1412. Kim Hyoun, Hodgins David, Torres Albina, Fontenelle Leonardo, Rosario Maria, Mathis Maria, Ferrao Ygor, Miguel Euripedes, Tavares Hermano Dual diagnosis of obsessive compulsive and compulsive buying disorders: Demographic, clinical, and psychiatric correlates. COMPREHENSIVE PSYCHIATRY. 2018;86():67-73.

1413. Guerrero-Hreins Eva, Foldi Claire, Oldfield Brian, Stefanidis Aneta, Sumithran Priya, Brown Robyn Gut-brain mechanisms underlying changes in disordered eating behaviour after bariatric surgery: a review. REVIEWS IN ENDOCRINE \& METABOLIC DISORDERS. 2022;23(4, SI):733-751.

1414. WALLIN GV, NORRING C, HOLMGREN S SELECTIVE DIETING PATTERNS AMONG ANORECTICS AND BULIMICS AT THE ONSET OF EATING DISORDER. EUROPEAN EATING DISORDERS REVIEW. 1994;2(4):221-232.

1415. Silva Vixey, Schukow Casey, Restini Carolina Striae distensae as a diagnostic indicator for eating disorder pathologies. INTERNATIONAL JOURNAL OF DERMATOLOGY. 2022;():.

1416. Aardoom Jiska, Dingemans Alexandra, Spinhoven Philip, Van Furth Eric Treating eating disorders over the internet: A systematic review and future research directions. INTERNATIONAL JOURNAL OF EATING DISORDERS. 2013;46(6):539-552.

1417. Dakanalis Antonios, Timko Alix, Serino Silvia, Riva Giuseppe, Clerici Massimo, Carra Giuseppe Prospective Psychosocial Predictors of Onset and Cessation of Eating Pathology amongst College Women. EUROPEAN EATING DISORDERS REVIEW. 2016;24(3):251-256.

1418. Escriva-Martinez Tamara, Galiana Laura, Herrero Rocio, Rodriguez-Arias Marta, Banos Rosa Understanding the Influence of Eating Patterns on Binge Drinking: A Mediation Model. INTERNATIONAL JOURNAL OF ENVIRONMENTAL RESEARCH AND PUBLIC HEALTH. 2020;17(24):.

1419. Johnson WG, Tsoh JY, Varnado PJ Eating disorders: Efficacy of pharmacological and psychological interventions. CLINICAL PSYCHOLOGY REVIEW. 1996;16(6):457-478.

1420. Chapa Danielle, Hagan Kelsey, Forbush Kelsie, Perko Victoria, Sorokina Daria, Alasmar Ahmed, Becker Carolyn, Sherman Roberta, Thompson Ron, Farrell Jennifer, Stewart Tiffany The Athletes' Relationships with Training scale (ART): A self-report measure of unhealthy training behaviors associated with eating disorders. INTERNATIONAL JOURNAL OF EATING DISORDERS. 2018;51(9):1080-1089.

1421. Baker Jessica, Thornton Laura, Lichtenstein Paul, Bulik Cynthia Pubertal development predicts eating behaviors in adolescence. INTERNATIONAL JOURNAL OF EATING DISORDERS. 2012;45(7):819-826.

1422. Rousset I, Kipman A, Ades P, Gorwood P Personality, temperament and anorexia nervosa. ANNALES MEDICO-PSYCHOLOGIQUES. 2004;162(3):180-188.

1423. Oliva Rossella, Morys Filip, Horstmann Annette, Castiello Umberto, Begliomini Chiara Characterizing impulsivity and resting-state functional connectivity in normal-weight binge eaters. INTERNATIONAL JOURNAL OF EATING DISORDERS. 2020;53(3):478-488.

1424. Stice E., Rohde P., Shaw H., Gau J. An experimental therapeutics test of whether adding dissonance-induction activities improves the effectiveness of a selective obesity and eating disorder prevention program. INTERNATIONAL JOURNAL OF OBESITY. 2018;42(3):462-468.

1425. Lim Michele, Parsons Sam, Goglio Alessia, Fox Elaine Anxiety, stress, and binge eating tendencies in adolescence: a prospective approach. JOURNAL OF EATING DISORDERS. 2021;9(1):.

1426. Wade T., Treloar S., Martin N. Shared and unique risk factors between lifetime purging and objective binge eating: a twin study. PSYCHOLOGICAL MEDICINE. 2008;38(10):1455-1464.

1427. AlHadi Ahmad, Almeharish Amani, Bilal Lisa, Al-Habeeb Abdulhameed, Al-Subaie Abdullah, Naseem Mohammad, Altwaijri Yasmin The prevalence and correlates of bulimia nervosa, binge-eating disorder, and anorexia nervosa: The Saudi National Mental Health Survey. INTERNATIONAL JOURNAL OF EATING DISORDERS. 2022;55(11):1541-1552.

1428. Smith Kathryn, Mason Tyler, Anderson Nicholas, Lavender Jason Unpacking cognitive emotion regulation in eating disorder psychopathology: The differential relationships between rumination, thought suppression, and eating disorder symptoms among men and women. EATING BEHAVIORS. 2019;32():95-100.

1429. Rania Marianna, Aloi Matteo, Filippis Renato, Carbone Elvira, Caroleo Mariarita, De Fazio Pasquale, Segura-Garcia Cristina Executive functions and depressive symptoms interplay in binge eating disorder: A structural equation model analysis. EUROPEAN EATING DISORDERS REVIEW. 2021;29(5):811-819.

1430. Brunner R., Resch F. Eating disorders - an increasing problem in children and adolescents?. THERAPEUTISCHE UMSCHAU. 2006;63(8):545-549.

1431. Ham T, Strien DC, Engeland H Personality characteristics predict outcome of eating disorders in adolescents: A 4-year prospective study. EUROPEAN CHILD \& ADOLESCENT PSYCHIATRY. 1998;7(2):79-84.

1432. Giel Katrin, Bulik Cynthia, Fernandez-Aranda Fernando, Hay Phillipa, Keski-Rahkonen Anna, Schag Kathrin, Schmidt Ulrike, Zipfel Stephan Binge eating disorder. NATURE REVIEWS DISEASE PRIMERS. 2022;8(1):.

1433. Reed Zoe, Micali Nadia, Bulik Cynthia, Smith George, Wade Kaitlin Assessing the causal role of adiposity on disordered eating in childhood, adolescence, and adulthood: a Mendelian randomization analysis. AMERICAN JOURNAL OF CLINICAL NUTRITION. 2017;106(3):764-772.

1434. Dougherty Elizabeth, Johnson Nicole, Badillo Krystal, Alissa A., Haedt-Matt Sleep reactivity is associated with social anxiety and disordered-eating behaviors in college students. JOURNAL OF AMERICAN COLLEGE HEALTH. 2021;():.

1435. Grilo CM, Masheb RM, Salant SL Cognitive behavioral therapy guided self-help and orlistat for the treatment of binge eating disorder: A randomized, double-blind, placebo-controlled trial. BIOLOGICAL PSYCHIATRY. 2005;57(10):1193-1201.

1436. Carbone Elvira, D'Amato Pasquale, Vicchio Giuseppe, De Fazio Pasquale, Segura-Garcia Cristina A systematic review on the role of microbiota in the pathogenesis and treatment of eating disorders. EUROPEAN PSYCHIATRY. 2020;64(1):.

1437. Gill Simerpal, Kaplan Allan A retrospective chart review study of symptom onset, diagnosis, comorbidities, and treatment in patients with binge eating disorder in Canadian clinical practice. EATING AND WEIGHT DISORDERS-STUDIES ON ANOREXIA BULIMIA AND OBESITY. 2021;26(4):1233-1242.

1438. Kong Seong Impact of Eating Psychopathology, Obsessive-Compulsion and Depression on Self-Harm Behavior in Patients with Eating Disorders. JOURNAL OF KOREAN ACADEMY OF NURSING. 2009;39(4):459-468.

1439. Guerdjikova Anna, O'Melia Anne, Mori Nicole, McCoy Jessica, McElroy Susan Binge eating disorder in elderly individuals. INTERNATIONAL JOURNAL OF EATING DISORDERS. 2012;45(7):905-908.

1440. Broadley Melanie, Bishop Tenile, White Melanie, Andrew Brooke The relationship between attentional bias to food and disordered eating in females with type 1 diabetes. APPETITE. 2019;140():269-276.

1441. Albohn-Kuehne Christina, Rief Winfried Shame, Guilt and Social Anxiety in Obesity with Binge-Eating Disorder. PSYCHOTHERAPIE PSYCHOSOMATIK MEDIZINISCHE PSYCHOLOGIE. 2011;61(9-10):412-417.

1442. Mason Tyler, Lavender Jason, Wonderlich Stephen, Crosby Ross, Engel Scott, Mitchell James, Crow Scott, Le Grange Daniel, Peterson Carol Examining a momentary mediation model of appearance-related stress, anxiety, and eating disorder behaviors in adult anorexia nervosa. EATING AND WEIGHT DISORDERS-STUDIES ON ANOREXIA BULIMIA AND OBESITY. 2018;23(5):637-644.

1443. Rigaud Daniel, Brayer Veronique, Biton-Jelic Violaine, Pais Vanessa, Pennacchio Helene, Brun Jean-Marcel Nasogastric tube feeding in bulimia - Controlled study with follow-up at 3 months. PRESSE MEDICALE. 2007;36(10, 1):1354-1363.

1444. Calzo Jerel, Austin S., Micali Nadia Sexual orientation disparities in eating disorder symptoms among adolescent boys and girls in the UK. EUROPEAN CHILD \& ADOLESCENT PSYCHIATRY. 2018;27(11):1483-1490.

1445. KURTH CL, KRAHN DD, NAIRN K, DREWNOWSKI A THE SEVERITY OF DIETING AND BINGEING BEHAVIORS IN COLLEGE-WOMEN - INTERVIEW VALIDATION OF SURVEY DATA.

JOURNAL OF PSYCHIATRIC RESEARCH. 1995;29(3):211-225.

1446. Nilsson K, Hagglof B Long-term follow-up of adolescent onset anorexia nervosa in northern Sweden. EUROPEAN EATING DISORDERS REVIEW. 2005;13(2):89-100.

1447. Gibbs Elise, Kass Andrea, Eichen Dawn, Fitzsimmons-Craft Ellen, Trockel Mickey, Wilfley Denise, Taylor C. Attention-deficit/hyperactivity disorder-specific stimulant misuse, mood, anxiety, and stress in college-age women at high risk for or with eating disorders. JOURNAL OF AMERICAN COLLEGE HEALTH. 2016;64(4):300-308.

1448. Behar R Eating disorders in adolescents: Epidemiological and clinical aspects. REVISTA MEDICA DE CHILE. 1998;126(9):1085-1092.

1449. Blythin Suzanne, Nicholson Hannah, Macintyre Vanessa, Dickson Joanne, Fox John, Taylor Peter Experiences of shame and guilt in anorexia and bulimia nervosa: A systematic review. PSYCHOLOGY AND PSYCHOTHERAPY-THEORY RESEARCH AND PRACTICE. 2020;93(1):134-159.

1450. Carter Jacqueline, Bewell-Weiss Carmen Nonfat Phobic Anorexia Nervosa: Clinical Characteristics and Response to Inpatient Treatment. INTERNATIONAL JOURNAL OF EATING DISORDERS. 2011;44(3):220-224.

1451. Wheaton Michael, Timpano Kiara, LaSalle-Ricci V., Murphy Dennis Characterizing the hoarding phenotype in individuals with OCD: Associations with comorbidity, severity and gender. JOURNAL OF ANXIETY DISORDERS. 2008;22(2):243-252.

1452. Arnone Jacqueline, Conti Richard Kleine-Levin Syndrome An Overview and Relevance to Nursing Practice. JOURNAL OF PSYCHOSOCIAL NURSING AND MENTAL HEALTH SERVICES. 2016;54(3):41-47.

1453. Kontic Olga, Vasiljevic Nadja, Trisovic Marija, Jorga Jagoda, Lakic Aneta, Gasic Miroslava Eating Disorders. SRPSKI ARHIV ZA CELOKUPNO LEKARSTVO. 2012;140(9-10):673-678.

1454. Mansour Salma, Rozenblat Vanja, Fuller-Tyszkiewicz Matthew, Paganini Chiara, Treasure Janet, Krug Isabel Emotions mediate the relationship between autistic traits and disordered eating: A new autistic-emotional model for eating pathology. PSYCHIATRY

RESEARCH. 2016;245():119-126.

1455. Hayes S., Napolitano M. Examination of weight control practices in a non-clinical sample of college women. EATING AND WEIGHT DISORDERS-STUDIES ON ANOREXIA BULIMIA AND OBESITY. 2012;17(3):E157-E163.

1456. Chamay-Weber Catherine, Combescure Christophe, Lanza Lydia, Carrard Isabelle, Haller Dagmar Screening Obese Adolescents for Binge Eating Disorder in Primary Care: The Adolescent Binge Eating Scale. JOURNAL OF PEDIATRICS. 2017;185():68+.

1457. Reas DL, Masheb RM, Grilo CM Appearance vs. health reasons for seeking treatment among obese patients with binge eating disorder. OBESITY RESEARCH. 2004;12(5):758-760.

1458. Wade Kaitlin, Kramer Michael, Oken Emily, Timpson Nicholas, Skugarevsky Oleg, Patel Rita, Bogdanovich Natalia, Vilchuck Konstantin, Smith George, Thompson Jennifer, Martin Richard Prospective associations between problematic eating attitudes in midchildhood and the future onset of adolescent obesity and high blood pressure. AMERICAN JOURNAL OF CLINICAL NUTRITION. 2017;105(2):306-312.

1459. Culbert K., Burt S., Sisk C., Nigg J., Klump K. The effects of circulating testosterone and pubertal maturation on risk for disordered eating symptoms in adolescent males. PSYCHOLOGICAL MEDICINE. 2014;44(11):2271-2286.

1460. Zaragoza-Marti Ana, Sanchez-SanSegundo Miriam, Ferrer-Cascales Rosario Is the binge eating disorder related to the adherence to Mediterranean diet in university students?. NUTRICION HOSPITALARIA. 2016;33(6):1385-1390.

1461. Sachs-Ericsson Natalie, Keel Pamela, Holland Lauren, Selby Edward, Verona Edelyn, Cougle Jesse, Palmer Emily Parental disorders, childhood abuse, and binge eating in a large community sample. INTERNATIONAL JOURNAL OF EATING DISORDERS. 2012;45(3):316-325.

1462. Rosa Sepulveda Ana, Moreno-Encinas Alba, Nova Esther, Gomez-Martinez Sonia, Marcos A., Antonio Carrobles Jose, Graell Montserrat BIOLOGICAL, PSYCHOLOGICAL AND FAMILIAL SPECIFIC CORRELATES IN EATING DISORDERS AT ONSET: A CONTROL-CASE STUDY PROTOCOL (ANOBAS). ACTAS ESPANOLAS DE PSIQUIATRIA. 2022;50(2):92-105.

1463. Hay P Quality of life and bulimic eating disorder behaviors: Findings from a community-based sample. INTERNATIONAL JOURNAL OF EATING DISORDERS. 2003;33(4):434-442.

1464. Steeves Elizabeth, Jones-Smith Jessica, Hopkins Laura, Gittelsohn Joel Perceived Social Support From Friends and Parents for Eating Behavior and Diet Quality Among Low-Income, Urban, Minority Youth. JOURNAL OF NUTRITION EDUCATION AND BEHAVIOR. 2016;48(5):304+.

1465. Mueller A., Zwaan M. Treatment of compulsive buying. FORTSCHRITTE DER NEUROLOGIE PSYCHIATRIE. 2008;76(8):478-483.

1466. Boggiano Mary Palatable Eating Motives Scale in a college population: Distribution of scores and scores associated with greater BMI and binge-eating. EATING BEHAVIORS. 2016;21():95-98.

1467. Samdal Gro, Furset Ole, Nysaether Marte, Abildsnes Eirik, Mildestvedt Thomas, Meland Eivind Healthy and unhealthy eating after a behaviour change intervention in primary care. PRIMARY HEALTH CARE RESEARCH AND DEVELOPMENT. 2022;23():.

1468. Jarvinen Anna, Leine Merja, Tikkanen Roope, Castren Maija Beneficial Effects of GLP-1 Agonist in a Male With Compulsive Food-Related Behavior Associated With Autism. FRONTIERS IN PSYCHIATRY. 2019;10():.

1469. Robinson Athena, Safer Debra Moderators of dialectical behavior therapy for binge eating disorder: Results from a randomized controlled trial. INTERNATIONAL JOURNAL OF EATING DISORDERS. 2012;45(4):597-602.

1470. Pataky Z., Gasteyger C., Ziegler O., Rissanen A., Hanotin C., Golay A. Efficacy of Rimonabant in Obese Patients with Binge Eating Disorder. EXPERIMENTAL AND CLINICAL ENDOCRINOLOGY \& DIABETES. 2013;121(1):20-26.

1471. Krafchek Jennifer, Kronborg Leonie The Changing Role of Academic Achievement Before the Onset of Disordered Eating in Academically High-Achieving Females. JOURNAL FOR THE EDUCATION OF THE GIFTED. 2019;42(2):135-163.

1472. Ribases M, Gratacos M, Fernandez-Aranda F, Bellodi L, Boni C, Anderluh M, Cavallini MC, Cellini E, Di Bella D, Erzegovesi S, Foulon C, Gabrovsek M, Gorwood P, Hebebrand J, Hinney A, Holliday J, Hu X, Karwautz A, Kipman A, Komel R, Nacmias B, Remschmidt H, Ricca V, Sorbi S, Wagner G, Treasure J, Collier DA, Estivill X Association of BDNF with anorexia, bulimia and age of onset of weight loss in six European populations. HUMAN MOLECULAR GENETICS. 2004;13(12):1205-1212.

1473. Brooks Samantha, Dahlberg Linda, Swenne Ingemar, Aronsson Marianne, Zarei Sanaz, Lundberg Lina, Jacobsson Josefin, Rask-Andersen Mathias, Salonen-Ros Helena, Rosling Agneta, Larsson Elna-Marie, Schioth Helgi Obsessive-compulsivity and working memory are associated with differential prefrontal cortex and insula activation in adolescents with a recent diagnosis of an eating disorder. PSYCHIATRY RESEARCH-NEUROIMAGING. 2014;224(3):246-253.

1474. Udo Tomoko, Bitley Sarah, Grilo Carlos Suicide attempts in US adults with lifetime DSM-5 eating disorders. BMC MEDICINE. 2019;17():.

1475. Barata-Santos Mafalda, Marta-Simoes Joana, Ferreira Claudia Body compassion safeguards against the impact of major life events on binge eating. APPETITE. 2019;134():34-39.

1476. Peveler RC, Bryden KS, Neil HAW, Fairburn CG, Mayou RA, Dunger DB, Turner HM The relationship of disordered eating habits and attitudes to clinical outcomes in young adult females with type 1 diabetes. DIABETES CARE. 2005;28(1):84-88.

1477. Hart Laura, Damiano Stephanie, Chittleborough Philip, Paxton Susan, Jorm Anthony Parenting to prevent body dissatisfaction and unhealthy eating patterns in preschool children: A Delphi consensus study. BODY IMAGE. 2014;11(4):418-425.

1478. Stoyel Hannah, Shanmuganathan-Felton Vaithehy, Meyer Caroline, Serpell Lucy Psychological risk indicators of disordered eating in athletes. PLOS ONE. 2020;15(5):.

1479. Valls M., Callahan S., Rousseau A., Chabrol H. Eating disorders and depressive symptoms: An epidemiological study in a male population. ENCEPHALE-REVUE DE PSYCHIATRIE CLINIQUE BIOLOGIQUE ET THERAPEUTIQUE. 2014;40(3):223-230.

1480. Sala Margaret, Keshishian Ani, Song Sarah, Moskowitz Rivka, Bulik Cynthia, Roos Corey, Levinson Cheri Predictors of relapse in eating disorders: A meta-analysis. JOURNAL

OF PSYCHIATRIC RESEARCH. 2023;158():281-299.

1481. Faris PL, Kim SW, Meller WH, Goodale RL, Oakman SA, Hofbauer RD, Marshall AM, Daughters RS, Banerjee-Stevens D, Eckert ED, Hartman BK Effect of decreasing afferent vagal activity with ondansetron on symptoms of bulimia nervosa: a randomised, double-blind trial. LANCET. 2000;355(9206):792-797.

1482. Fornaro Michele, Daray Federico, Hunter Fernando, Anastasia Annalisa, Stubbs Brendon, De Berardis Domenico, Shin Jae, Husain Muhammad, Dragioti Elena, Fusar-Poli Paolo, Solmi Marco, Berk Michael, Vieta Eduard, Carvalho Andre The prevalence, odds and predictors of lifespan comorbid eating disorder among people with a primary diagnosis of bipolar disorders, and vice-versa: Systematic review and meta-analysis. JOURNAL OF AFFECTIVE DISORDERS. 2021;280(A):409-431.

1483. Autran Teixeira Amanda, Silva Amanda, Alves Sandymara, Pessa Rosane, Maniglia Fabiola, Manochio-Pina Marina Associated personality disorder and depression to eating disorders. RBONE-REVISTA BRASILEIRA DE OBESIDADE NUTRICAO E EMAGRECIMENTO. 2022;16(101):282-292.

1484. Bennett Sarah, Dodge Tonya Ethnic-racial differences in feelings of embarrassment associated with binge eating and fear of losing control. INTERNATIONAL JOURNAL OF EATING DISORDERS. 2007;40(5):454-459.

1485. Herpertz-Dahlmann B, Muller B, Herpertz S, Heussen N, Hebebrand J, Remschmidt H Prospective 10-year follow-up in adolescent anorexia nervosa - Course, outcome, psychiatric comorbidity, and psychosocial adaptation. JOURNAL OF CHILD PSYCHOLOGY AND PSYCHIATRY. 2001;42(5):603-612.

1486. Thompson-Memmer Carly, Glassman Tavis, Diehr Aaron Drunkorexia: A new term and diagnostic criteria. JOURNAL OF AMERICAN COLLEGE HEALTH. 2019;67(7):620-626.

1487. Brauhardt Anne, Rudolph Almut, Hilbert Anja Implicit cognitive processes in binge-eating disorder and obesity. JOURNAL OF BEHAVIOR THERAPY AND EXPERIMENTAL PSYCHIATRY. 2014;45(2):285-290.

1488. Winzelberg AJ, Tayor CB, Sharpe T, Eldredge KL, Dev P, Constantinou PS Evaluation of a computer-mediated eating disorder intervention program. INTERNATIONAL JOURNAL OF

EATING DISORDERS. 1998;24(4):339-349.

1489. Hancock SD, Menard JL, Olmstead MC Variations in maternal care influence vulnerability to stress-induced binge eating in female rats. PHYSIOLOGY \& BEHAVIOR. 2005;85(4):430-439.

1490. Ibrahim Mohamad, Ismail Wan, Jaafar Nik, Mokhtaruddin Ummi, Ong Hooi, Abu Bakar Nur, Sahimi Hajar Depression and Its Association With Self-Esteem and Lifestyle Factors Among School-Going Adolescents in Kuala Lumpur, Malaysia. FRONTIERS IN PSYCHIATRY. 2022;13():.

1491. Coimbra Maria, Ferreira Claudia Understanding the omnivore, vegetarian, vegan, and paleo dietary patterns: How they differ in disordered eating indicators. REVISTA PORTUGUESA DE INVESTIGACAO COMPORTAMENTAL E SOCIAL. 2021;7(1):41-51.

1492. Neumark-Sztainer D, Patterson J, Mellin A, Ackard DM, Utter J, Story M, Sockalosky J Weight control practices and disordered eating behaviors among adolescent females and males with type 1 diabetes - Associations with sociodemographics, weight concerns, familial factors, and metabolic outcomes. DIABETES CARE. 2002;25(8):1289-1296.

1493. Hecht Leah, Schwartz Natalie, Miller-Matero Lisa, Braciszewski Jordan, Haedt-Matt Alissa Eating pathology and depressive symptoms as predictors of excessive weight gain during pregnancy. JOURNAL OF HEALTH PSYCHOLOGY. 2021;26(13):2414-2423.

1494. Ciarma Jessica, Mathew Jaya Social anxiety and disordered eating: The influence of stress reactivity and self-esteem. EATING BEHAVIORS. 2017;26():177-181.

1495. Rouleau Codie, Ranson Kristin Potential risks of pro-eating disorder websites. CLINICAL PSYCHOLOGY REVIEW. 2011;31(4):525-531.

1496. Strober M, Freeman R, Morrell W Atypical anorexia nervosa: Separation from typical cases in course and outcome in a long-term prospective study. INTERNATIONAL JOURNAL OF EATING DISORDERS. 1999;25(2):135-142.

1497. Parker Lacie, Harriger Jennifer Eating disorders and disordered eating behaviors in the LGBT population: a review of the literature. JOURNAL OF EATING DISORDERS.

2020;8(1):.

1498. Le Long, Mihalopoulos Cathrine, Engel Lidia, Touyz Stephen, Gonzalez-Chica David, Stocks Nigel, Hay Phillipa Burden and health state utility values of eating disorders: results from a population-based survey. PSYCHOLOGICAL MEDICINE. 2021;51(1):130-137.

1499. Dahlgren Camilla, Stedal Kristin, Wisting Line A systematic review of eating disorder prevalence in the Nordic countries: 1994-2016. NORDIC PSYCHOLOGY. 2018;70(3):209-227.

1500. Mikhail Megan Affect Dysregulation in Context: Implications and Future Directions of Experience Sampling Research on Affect Regulation Models of Loss of Control Eating. FRONTIERS IN PSYCHIATRY. 2021;12():.

1501. Heyne Andrea, Kiesselbach Christoph, Sahun Ignasi, McDonald Jerome, Gaiffi Monica, Dierssen Mara, Wolffgramm Jochen An animal model of compulsive food-taking behaviour. ADDICTION BIOLOGY. 2009;14(4):373-383.

1502. Williams Brenna, Levinson Cheri Intolerance of uncertainty and maladaptive perfectionism as maintenance factors for eating disorders and obsessive-compulsive disorder symptoms. EUROPEAN EATING DISORDERS REVIEW. 2021;29(1):101-111.

1503. Fontenelle LE, Mendlowicz MV, Versiani M Impulse control disorders in patients with obsessive-compulsive disorder. PSYCHIATRY AND CLINICAL NEUROSCIENCES. 2005;59(1):30-37.

1504. Gaudio Santino, Di Ciommo Vincenzo Prevalence of Personality Disorders and Their Clinical Correlates in Outpatient Adolescents With Anorexia Nervosa. PSYCHOSOMATIC MEDICINE. 2011;73(9):769-774.

1505. NEUMARKSZTAINER D, BUTLER R, PALTI H EATING DISTURBANCES AMONG ADOLESCENT GIRLS - EVALUATION OF A SCHOOL-BASED PRIMARY PREVENTION PROGRAM. JOURNAL OF NUTRITION EDUCATION. 1995;27(1):24-31.

1506. Raevuori Anu, Haukka Jari, Vaarala Outi, Suvisaari Jaana, Gissler Mika, Grainger Marjut, Linna Milla, Suokas Jaana The Increased Risk for Autoimmune Diseases in Patients

with Eating Disorders. PLOS ONE. 2014;9(8):.

1507. Najjar Rana, Jacob Eufemia, Evangelista Lorraine Eating Behaviors, Weight Bias, and Psychological Functioning in Multi-ethnic Low-income Adolescents. JOURNAL OF PEDIATRIC NURSING-NURSING CARE OF CHILDREN \& FAMILIES. 2018;38():81-87.

1508. Dakanalis Antonios, Colmegna Fabrizia, Riva Giuseppe, Clerici Massimo Validity and utility of the DSM-5 severity specifier for binge-eating disorder. INTERNATIONAL JOURNAL OF EATING DISORDERS. 2017;50(8):917-923.

1509. Hove O Prevalence of eating disorders in adults with mental retardation living in the community. AMERICAN JOURNAL ON MENTAL RETARDATION. 2004;109(6):501-506.

1510. OROURKE DA, WURTMAN JJ, WURTMAN RJ, TSAY R, GLEASON R, BAER L, JENIKE MA ABERRANT SNACKING PATTERNS AND EATING DISORDERS IN PATIENTS WITH OBSESSIVE-COMPULSIVE DISORDER. JOURNAL OF CLINICAL PSYCHIATRY. 1994;55(10):445-447.

1511. Smith Kathryn, Luo Shan, Mason Tyler A systematic review of neural correlates of dysregulated eating associated with obesity risk in youth. NEUROSCIENCE AND BIOBEHAVIORAL REVIEWS. 2021;124():245-266.

1512. Davies Helena, Hubel Christopher, Herle Moritz, Kakar Saakshi, Mundy Jessica, Peel Alicia, Kuile Abigail, Zvrskovec Johan, Monssen Dina, Lim Kai, Davies Molly, Palmos Alish, Lin Yuhao, Kalsi Gursharan, Rogers Henry, Bristow Shannon, Glen Kiran, Malouf Chelsea, Kelly Emily, Purves Kirstin, Young Katherine, Hotopf Matthew, Armour Cherie, McIntosh Andrew, Eley Thalia, Treasure Janet, Breen Gerome Risk and protective factors for new-onset binge eating, low weight, and self-harm symptoms in >35,000 individuals in the UK during the COVID-19 pandemic. INTERNATIONAL JOURNAL OF EATING DISORDERS. 2023;56(1, SI):91-107.

1513. Zhou Yuan, Wade Tracey The impact of COVID-19 on body-dissatisfied female university students. INTERNATIONAL JOURNAL OF EATING DISORDERS. 2021;54(7):1283-1288.

1514. Agras W., Fitzsimmons-Craft Ellen, Wilfley Denise Evolution of cognitive-behavioral therapy for eating disorders. BEHAVIOUR RESEARCH AND THERAPY. 2017;88():26-36.

1515. Bottera Angeline, Kambanis P., De Young Kyle The differential associations of shame and guilt with eating disorder behaviors. EATING BEHAVIORS. 2020;39():.

1516. Faravelli C., Ravaldi C., Truglia E., Zucchi T., Cosci F., Ricca V. Clinical epidemiology of eating disorders: Results from the Sesto Fiorentino Study. PSYCHOTHERAPY AND PSYCHOSOMATICS. 2006;75(6):376-383.

1517. Haines Jess, Ziyadeh Najat, Franko Debra, McDonald Julia, Mond Jonathan, Austin S. Screening High School Students for Eating Disorders: Validity of Brief Behavioral and Attitudinal Measures. JOURNAL OF SCHOOL HEALTH. 2011;81(9):530-535.

1518. Stice Eric, Shaw Heather, Burton Emily, Wade Emily Dissonance and healthy weight eating disorder prevention programs: A randomized efficacy trial. JOURNAL OF CONSULTING AND CLINICAL PSYCHOLOGY. 2006;74(2):263-275.

1519. Mason Tyler, Smith Kathryn, Lavender Jason, Leventhal Adam Longitudinal Prospective Association between Hedonic Hunger and Unhealthy Food and Drink Intake in Adolescents. INTERNATIONAL JOURNAL OF ENVIRONMENTAL RESEARCH AND PUBLIC HEALTH. 2020;17(24):.

1520. Borja Celina, Holtzman Bryan, McCall Lauren, Carson Traci, Moretti Laura, Farnsworth Nicole, Ackerman Kathryn Specific dietary practices in female athletes and their association with positive screening for disordered eating. JOURNAL OF EATING DISORDERS. 2021;9(1):.

1521. Mukherjee Ashmita, DiBrog Adrianne, Mietlicki-Baase Elizabeth The impact of binge-like palatable food intake on the endogenous glucagon-like peptide-1 system in female rats. BEHAVIOURAL BRAIN RESEARCH. 2022;428():.

1522. Aznar Diaz Inmaculada, Kopecky Kamil, Romero Rodriguez Jose, Caceres Reche Maria, Trujillo Torres Juan Pathologies associated with Problematic Internet Use. A systematic review and meta-analysis in WoS and Scopus. INVESTIGACION BIBLIOTECOLOGICA. 2020;34(82):229-253.

1523. Talarico G., Canevelli M., Tosto G., Piscopo P., Confaloni A., Galimberti D., Fenoglio C., Scarpini E., Gasparini M., Bruno G. Binge eating and fast cognitive worsening in an early-onset bvFTD patient carrying C9ORF72 expansion. NEUROCASE. 2015;21(5):543-547.

1524. Ogutlu Hakan, McNicholas Fiona Stigma and Treatment of Eating Disorders in School Counselors in Turkey (STED-SCIT). PSYCHIATRY AND BEHAVIORAL SCIENCES. 2021;11(1):8-17.

1525. Goode Rachel, Kalarchian Melissa, Craighead Linda, Conroy Molly, Wallace John, Eack Shaun, Burke Lora The feasibility of a binge eating intervention in Black women with obesity. EATING BEHAVIORS. 2018;29():83-90.

1526. Wu Ya-Ke, Liu Yi Weight-related stigmatization and binge eating among overweight adults in Southern Taiwan. ASIA PACIFIC JOURNAL OF CLINICAL NUTRITION. 2015;24(1):118-127.

1527. Williams Brenna, Brown Mackenzie, Sandoval-Araujo Luis, Russell Street, Levinson Cheri Psychiatric Comorbidity Among Eating Disorders and Obsessive-Compulsive Disorder and Underlying Shared Mechanisms and Features: An Updated Review. JOURNAL OF COGNITIVE PSYCHOTHERAPY. 2022;36(3, SI):226-246.

1528. Wildes Jennifer, Marcus Marsha, Fagiolini Andrea Obesity in patients with bipolar disorder: A biopsychosocial-behavioral model. JOURNAL OF CLINICAL PSYCHIATRY. 2006;67(6):904-915.

1529. Kerr G, Berman E, De Souza MJ Disordered eating in women's gymnastics: Perspectives of athletes, coaches, parents, and judges. JOURNAL OF APPLIED SPORT PSYCHOLOGY. 2006;18(1):28-43.

1530. Yoon Cynthia, Mason Susan, Hooper Laura, Eisenberg Marla, Neumark-Sztainer Dianne Disordered Eating Behaviors and 15-year Trajectories in Body Mass Index: Findings From Project Eating and Activity in Teens and Young Adults (EAT). JOURNAL OF ADOLESCENT HEALTH. 2020;66(2):181-188.

1531. Bell Megan, Zeiler Michael, Herrero Rocio, Kuso Stefanie, Nitsch Martina, Etchemendy Ernestina, Fonseca-Baeza Sara, Oliver Elia, Adamcik Tanja, Karwautz Andreas, Wagner Gudrun, Banos Rosa, Botella Cristina, Goerlich Dennis, Jacobi Corinna, Waldherr Karin Healthy Teens @ School: Evaluating and disseminating transdiagnostic preventive interventions for eating disorders and obesity for adolescents in school settings. INTERNET INTERVENTIONS-THE APPLICATION OF INFORMATION TECHNOLOGY IN MENTAL AND BEHAVIOURAL HEALTH. 2019;16(SI):65-75.

1532. Stice Eric, Rohde Paul, Shaw Heather, Marti C. Efficacy Trial of a Selective Prevention Program Targeting Both Eating Disorder Symptoms and Unhealthy Weight Gain Among Female College Students. JOURNAL OF CONSULTING AND CLINICAL PSYCHOLOGY. 2012;80(1):164-170.

1533. Kristjansdottir Hafrun, Sigurdardottir Petra, Jonsdottir Sigurlaug, Thorsteinsdottir Gudlaug, Saavedra Jose Body Image Concern and Eating Disorder Symptoms Among Elite Icelandic Athletes. INTERNATIONAL JOURNAL OF ENVIRONMENTAL RESEARCH AND PUBLIC HEALTH. 2019;16(15):.

1534. Cao Zhangqi, Cini Erica, Pellegrini Dario, Fragkos Kostantinos The association between sexual orientation and eating disorders-related eating behaviours in adolescents: A systematic review and meta-analysis. EUROPEAN EATING DISORDERS REVIEW. 2023;31(1):46-64.

1535. Santonastaso P, Zanetti T, Ferrara S, Olivotto MC, Magnavita N, Favaro A A preventive intervention program in adolescent schoolgirls: A longitudinal study. PSYCHOTHERAPY AND PSYCHOSOMATICS. 1999;68(1):46-50.

1536. Beccia Ariel, Ruf Andrea, Druker Susan, Ludwig Vera, Brewer Judson Women's Experiences with a Mindful Eating Program for Binge and Emotional Eating: A Qualitative Investigation into the Process of Change. JOURNAL OF ALTERNATIVE AND COMPLEMENTARY MEDICINE. 2020;26(10):937-944.

1537. DeVille Danielle, Erchull Mindy, Mailloux Jennifer Intuitive eating mediates the relationship between interoceptive accuracy and eating disorder risk. EATING BEHAVIORS. 2021;41():.

1538. Rancourt Diana, McCullough Mary Overlap in Eating Disorders and Obesity in Adolescence. CURRENT DIABETES REPORTS. 2015;15(10):.

1539. Mond J., Marks P., Hay P., Rodgers B., Kelly C., Owen C., Paxton S. Mental health literacy and eating-disordered behavior: Beliefs of adolescent girls concerning the treatment of and treatment-seeking for bulimia nervosa. JOURNAL OF YOUTH AND ADOLESCENCE. 2007;36(6):753-762.

1540. Turel Tacibaht, Jameson Molly, Gitimu Priscilla, Rowlands Zara, Mincher Jeanine, Pohle-Krauza Rachael Disordered eating: Influence of body image, sociocultural attitudes,

appearance anxiety and depression - a focus on college males and a gender comparison. COGENT PSYCHOLOGY. 2018;5(1):.

1541. Kodama K, Noda S, Murakami A, Azuma Y, Takeda N, Yamanouchi N, Okada S, Komatsu N, Sato T, Miyazawa Y, Kawamura I Depressive disorders as psychiatric complications after obesity surgery. PSYCHIATRY AND CLINICAL NEUROSCIENCES. 1998;52(5):471-476.

1542. Ma Ruofan, Capobianco Kevin, Buchanan NiCole, Hu Zhiyuan, Oakman Jonathan Etiologic and treatment conceptualizations of disordered eating symptoms among mainland Chinese therapists. INTERNATIONAL JOURNAL OF EATING DISORDERS. 2020;53(3):391-403.

1543. Peterson Carol, Becker Carolyn, Treasure Janet, Shafran Roz, Bryant-Waugh Rachel The three-legged stool of evidence-based practice in eating disorder treatment: research, clinical, and patient perspectives. BMC MEDICINE. 2016;14():.

1544. SUNDGOTBORGEN J RISK AND TRIGGER FACTORS FOR THE DEVELOPMENT OF EATING DISORDERS IN FEMALE ELITE ATHLETES. MEDICINE AND SCIENCE IN SPORTS AND EXERCISE. 1994;26(4):414-419.

1545. Joy Elizabeth, Kussman Andrea, Nattiv Aurelia 2016 update on eating disorders in athletes: A comprehensive narrative review with a focus on clinical assessment and management. BRITISH JOURNAL OF SPORTS MEDICINE. 2016;50(3):154+.

1546. McIntosh V., Carter F., Bulik C., Frampton C., Joyce P. Five-year outcome of cognitive behavioral therapy and exposure with response prevention for bulimia nervosa. PSYCHOLOGICAL MEDICINE. 2011;41(5):1061-1071.

1547. Littleton HL, Ollendick T Negative body image and disordered eating behavior in children and adolescents: What places youth at risk and how can these problems be prevented?. CLINICAL CHILD AND FAMILY PSYCHOLOGY REVIEW. 2003;6(1):51-66.

1548. Froreich Franzisca, Vartanian Lenny, Zawadzki Matthew, Grisham Jessica, Touyz Stephen Psychological need satisfaction, control, and disordered eating. BRITISH JOURNAL OF CLINICAL PSYCHOLOGY. 2017;56(1):53-68.

1549. Ketata W., Aloulou J., Charfi N., Abid M., Amami O. Binge eating disorder and obesity: Epidemiological, clinical and psychopathological aspects. A study of an obese population in Sfax (Tunisia). ANNALES D ENDOCRINOLOGIE. 2009;70(6):462-467.

1550. Forrest Lauren, Zuromski Kelly, Dodd Dorian, Smith April Suicidality in adolescents and adults with binge-eating disorder: Results from the national comorbidity survey replication and adolescent supplement. INTERNATIONAL JOURNAL OF EATING DISORDERS. 2017;50(1):40-49.

1551. Legenbauer Tanja, Martin Franziska, Blaschke Ariane, Schwenzfeier Anne, Blechert Jens, Schnicker Katja Two sides of the same coin? A new instrument to assess body checking and avoidance behaviors in eating disorders. BODY IMAGE. 2017;21():39-46.

1552. Livingston Whitney, Fargo Jamison, Blais Rebecca Depression symptoms as a potential mediator of the association between disordered eating symptoms and sexual function in women service members and veterans. MILITARY PSYCHOLOGY. 2022;34(6):687-696.

1553. Bonfa F., Cabrini S., Aanzi M., Bettinardi O., Spotti R., Uber E. Treatment dropout in drug-addicted women: Are eating disorders implicated?. EATING AND WEIGHT DISORDERS-STUDIES ON ANOREXIA BULIMIA AND OBESITY. 2008;13(2):81-86.

1554. TOBIN DL, MOLTENI AL, ELIN MR EARLY TRAUMA, DISSOCIATION, AND LATE-ONSET IN THE EATING DISORDERS. INTERNATIONAL JOURNAL OF EATING DISORDERS. 1995;17(3):305-308.

1555. Halvorsen I, Andersen A, Heyerdahl S Good outcome of adolescent onset anorexia nervosa after systematic treatment - Intermediate to long-term follow-up of a representative county-sample. EUROPEAN CHILD \& ADOLESCENT PSYCHIATRY. 2004;13(5):295-306.

1556. Talen Mary, Mann Misty Obesity and Mental Health. PRIMARY CARE. 2009;36(2):287+.

1557. Rivera Heidi, Christiansen Kelly, Sullivan Elinor The role of maternal obesity in the risk of neuropsychiatric disorders. FRONTIERS IN NEUROSCIENCE. 2015;9():.

1558. Manaboriboon Boonying, In-iw Supinya, Winijkul Gornmigar, Choowong Tidarat, Suwanwaree Jedsada, Apinuntavech Suporn EATING DISORDER SUSCEPTIBILITY AMONG UNIVERSITY STUDENTS IN THAILAND. SOUTHEAST ASIAN JOURNAL OF TROPICAL MEDICINE AND PUBLIC HEALTH. 2017;48(2):19-28.

1559. Zarychta Karolina, Chan Carina, Kruk Magdalena, Luszczynska Aleksandra Body satisfaction and body weight in under- and healthy-weight adolescents: mediating effects of restrictive dieting, healthy and unhealthy food intake. EATING AND WEIGHT DISORDERS-STUDIES ON ANOREXIA BULIMIA AND OBESITY. 2020;25(1):41-50.

1560. Pullmer Rachelle, Zaitsoff Shannon, Coelho Jennifer Cognitive and behavioral correlates of depressive symptoms in a community sample of adolescents. CLINICAL CHILD PSYCHOLOGY AND PSYCHIATRY. 2020;25(1):98-105.

1561. Kamody Rebecca, Yonkers Kimberly, Pluhar Emily, Olezeski Christy Disordered Eating Among Trans-Masculine Youth: Considerations Through a Developmental Lens. LGBT HEALTH. 2020;7(4):170-173.

1562. Francisco Rita, Narciso Isabel, Alarcao Madalena Individual and relational risk factors for the development of eating disorders in adolescent aesthetic athletes and general adolescents. EATING AND WEIGHT DISORDERS-STUDIES ON ANOREXIA BULIMIA AND OBESITY. 2013;18(4):403-411.

1563. Petersson BH Bulimia nervosa.. UGESKRIFT FOR LAEGER. 2001;163(25):3465-3468.

1564. Aschenbrenner K, Aschenbrenner F, Kirchmann H, Strauss B Disturbed eating behaviour among high school and university students. PSYCHOTHERAPIE PSYCHOSOMATIK MEDIZINISCHE PSYCHOLOGIE. 2004;54(6):259-263.

1565. Gan Wan, Mohamad Normasliana, Law Leh Factors Associated with Binge Eating Behavior among Malaysian Adolescents. NUTRIENTS. 2018;10(1):.

1566. Vollert Bianka, Beintner Ina, Musiat Peter, Gordon Gemma, Goerlich Dennis, Nacke Barbara, Schmidt-Hantke Juliane, Potterton Rachel, Spencer Lucy, Grant Nina, Schmidt Ulrike, Jacobi Corinna Using internet-based self-help to bridge waiting time for face-to-face outpatient treatment for Bulimia Nervosa, Binge Eating Disorder and related disorders: Study protocol of a randomized controlled trial. INTERNET INTERVENTIONS-THE APPLICATION OF INFORMATION TECHNOLOGY IN MENTAL AND BEHAVIOURAL HEALTH.

2019;16(SI):26-34.

1567. Goldschmidt Andrea, Wall Melanie, Choo Tse-Hwei, Becker Carolyn, Neumark-Sztainer Dianne Shared Risk Factors for Mood-, Eating-, and Weight-Related Health Outcomes. HEALTH PSYCHOLOGY. 2016;35(3):245-252.

1568. Quick Virginia, Loth Katie, MacLehose Richard, Linde Jennifer, Neumark-Sztainer Dianne Prevalence of Adolescents' Self-Weighing Behaviors and Associations With Weight-Related Behaviors and Psychological Well-Being. JOURNAL OF ADOLESCENT HEALTH. 2013;52(6):738-744.

1569. Martinussen Monica, Friborg Oddgeir, Schmierer Phoebe, Kaiser Sabine, Overgard Karl, Neunhoeffer Anna-Lena, Martinsen Egil, Rosenvinge Jan The comorbidity of personality disorders in eating disorders: a meta-analysis. EATING AND WEIGHT DISORDERS-STUDIES ON ANOREXIA BULIMIA AND OBESITY. 2017;22(2):201-209.

1570. Becker K, Jennen-Steinmetz C, Holtmann M, El-Faddagh M, Schmidt H Comorbidity of obsessive-compulsive disorders in childhood and adolescence. ZEITSCHRIFT FUR KINDER- UND JUGENDPSYCHIATRIE UND PSYCHOTHERAPIE. 2003;31(3):175-185.

1571. Fuller-Tyszkiewicz Matthew, Rodgers Rachel, Maiano Christophe, Mellor David, Sicilia Alvaro, Markey Charlotte, Aime Annie, Dion Jacinthe, Pietrabissa Giada, Lo Coco Gianluca, Caltabiano Marie, Strodl Esben, Alcaraz-Ibanez Manuel, Begin Catherine, Blackburn Marie-Eve, Castelnuovo Gianluca, Granero-Gallegos Antonio, Gullo Salvatore, Hayami-Chisuwa Naomi, He Qiqiang, Manzonie Gian, Probst Michel, McCabe Marita Testing of a model for risk factors for eating disorders and higher weight among emerging adults: Baseline evaluation. BODY IMAGE. 2022;40():322-339.

1572. Forrester-Knauss Christine, Stutz Elisabeth Gender differences in disordered eating and weight dissatisfaction in Swiss adults: Which factors matter?. BMC PUBLIC HEALTH. 2012;12():.

1573. NeumarkSztainer D School-based programs for preventing eating disturbances. JOURNAL OF SCHOOL HEALTH. 1996;66(2):64-71.

1574. GILLBERG IC, RASTAM M, GILLBERG C ANOREXIA-NERVOSA 6 YEARS AFTER ONSET .1. PERSONALITY-DISORDERS. COMPREHENSIVE PSYCHIATRY. 1995;36(1):61-69.

1575. Maglia Marilena, Corello Graziana, Caponnetto Pasquale Evaluation of the Effects of Telepsychotherapy in the Treatment and Prevention of Eating Disorders in Adolescents. INTERNATIONAL JOURNAL OF ENVIRONMENTAL RESEARCH AND PUBLIC HEALTH. 2021;18(23):.

1576. Eldredge KL, Agras WS Burned out binge eaters: A preliminary investigation. INTERNATIONAL JOURNAL OF EATING DISORDERS. 1996;19(4):411-414.

1577. Cottone Pietro, Sabino Valentina, Steardo Luca, Zorrilla Eric Opioid-dependent anticipatory negative contrast and binge-like eating in rats with limited access to highly preferred food. NEUROPSYCHOPHARMACOLOGY. 2008;33(3):524-535.

1578. Tremblay Line, Lariviere Michel The influence of puberty onset, Body Mass Index, and pressure to be thin on disordered eating behaviors in children and adolescents. EATING BEHAVIORS. 2009;10(2):75-83.

1579. Hershko Shirley, Cortese Samuele, Ert Eyal, Aronis Anna, Maeir Adina, Pollak Yehuda The influence of attractiveness and convenience cues on food appeal in adults with and without ADHD. APPETITE. 2020;150():.

1580. Gianni Angela, Donatis Domenico, Valente Stefano, Ronchi Diana, Atti Anna Eating disorders: Do PET and SPECT have a role? A systematic review of the literature.. PSYCHIATRY RESEARCH-NEUROIMAGING. 2020;300():.

1581. Mischoulon David, Eddy Kamryn, Keshaviah Aparna, Dinescu Diana, Ross Stephanie, Kass Andrea, Franko Debra, Herzog David Depression and eating disorders: Treatment and course. JOURNAL OF AFFECTIVE DISORDERS. 2011;130(3):470-477.

1582. Nogueira Sofia, Garcez Fernanda, Sa Susana, Moutinho Luis, Cardoso Armando, Soares Raquel, Fonseca Bruno, Leal Sandra Early unhealthy eating habits underlie morpho-functional changes in the liver and adipose tissue in male rats. HISTOCHEMISTRY AND CELL BIOLOGY. 2022;157(6):657-669.

1583. Bacalhau Silvia, Moleiro Pascoal EATING DISORDERS IN ADOLESCENTS What To Look For?. ACTA MEDICA PORTUGUESA. 2010;23(5):777-784.

1584. Nakai Y, Hamagaki S, Kato S, Seino Y, Takagi R, Kurimoto F Leptin in women with eating disorders. METABOLISM-CLINICAL AND EXPERIMENTAL. 1999;48(2):217-220.

1585. Tanofsky-Kraff Marian, Goossens Lien, Eddy Kamryn, Ringham Rebecca, Goldschmidt Andrea, Yanovski Susan, Braet Caroline, Marcus Marsha, Wilfley Denise, Olsen Cara, Yanovski Jack A multisite investigation of binge eating behaviors in children and adolescents. JOURNAL OF CONSULTING AND CLINICAL PSYCHOLOGY. 2007;75(6):901-913.

1586. Jones Michelle, Crowther Janis Predicting the onset of inappropriate compensatory behaviors in undergraduate college women. EATING BEHAVIORS. 2013;14(1):17-20.

1587. Ebneter Daria, Latner Janet Stigmatizing Attitudes Differ Across Mental Health Disorders A Comparison of Stigma Across Eating Disorders, Obesity, and Major Depressive Disorder. JOURNAL OF NERVOUS AND MENTAL DISEASE. 2013;201(4):281-285.

1588. Hoerster Katherine, Jakupcak Matthew, Hanson Robert, McFall Miles, Reiber Gayle, Hall Katherine, Nelson Karin PTSD and depression symptoms are associated with binge eating among US Iraq and Afghanistan veterans. EATING BEHAVIORS. 2015;17():115-118.

1589. White Marney Smoking for weight control and its associations with eating disorder symptomatology. COMPREHENSIVE PSYCHIATRY. 2012;53(4):403-407.

1590. Kong Peiling, Harris Lynne The Sporting Body: Body Image and Eating Disorder Symptomatology Among Female Athletes from Leanness Focused and Nonleanness Focused Sports. JOURNAL OF PSYCHOLOGY. 2015;149(2):141-160.

1591. Roehrig Megan, Masheb Robin, White Marney, Grilo Carlos The Metabolic Syndrome and Behavioral Correlates in Obese Patients With Binge Eating Disorder. OBESITY. 2009;17(3):481-486.

1592. Fiechtner Lauren, Fonte Maria, Castro Ines, Gerber Monica, Horan Chrissy, Sharifi Mona, Cena Hellas, Taveras Elsie Determinants of Binge Eating Symptoms in Children with Overweight/Obesity. CHILDHOOD OBESITY. 2018;14(8):510-517.

1593. Loth Katie, Wall Melanie, Larson Nicole, Neumark-Sztainer Dianne Disordered Eating and Psychological Well-Being in Overweight and Nonoverweight Adolescents: Secular Trends from 1999 to 2010. INTERNATIONAL JOURNAL OF EATING DISORDERS.

2015;48(3):323-327.

1594. Sultson Hedvig, Kukk Katrin, Akkermann Kirsti Positive and negative emotional eating have different associations with overeating and binge eating: Construction and validation of the Positive-Negative Emotional Eating Scale. APPETITE. 2017;116():423-430.

1595. Monteleone P, Fabrazzo M, Martiadis V, Fuschino A, Serritella C, Milici N, Maj M Opposite changes in circulating adiponectin in women with bulimia nervosa or binge eating disorder. JOURNAL OF CLINICAL ENDOCRINOLOGY \& METABOLISM. 2003;88(11):5387-5391.

1596. Dokhani Abolfazl, Dehghan Mahlagha, Rayani Masoud, Maazallahi Mahboobeh, Forouzi Mansooreh Factors associated with disordered feeding among high school students in Kerman City, Iran. JOURNAL OF EATING DISORDERS. 2022;10(1):.

1597. Oliveira Coelho Gabriela, Soares Eliane, Ribeiro Beatriz Are female athletes at increased risk for disordered eating and its complications?. APPETITE. 2010;55(3):379-387.

1598. Rosello Rocio, Gledhill Julia, Yi Irene, Watkins Beth, Harvey Lucy, Hosking Alexandra, Nicholls Dasha Recognition and duration of illness in adolescent eating disorders: Parental perceptions of symptom onset. EARLY INTERVENTION IN PSYCHIATRY. 2022;16(8):854-861.

1599. Ramirez Ana, Perez Marisol, Taylor Aaron Preliminary examination of a couple-based eating disorder prevention program. BODY IMAGE. 2012;9(3):324-333.

1600. Cuzzocrea Francesca, Costa Sebastiano, Larcan Rosalba, Toffle Mary Differences between adolescents exhibiting moderate binging and non-binging eating behaviors. SPRINGERPLUS. 2015;4():.

1601. Hughes Elizabeth Comorbid depression and anxiety in childhood and adolescent anorexia nervosa: Prevalence and implications for outcome. CLINICAL PSYCHOLOGIST. 2012;16(1, SI):15-24.

1602. Martenstyn Jordan, Jeacocke Nikki, Pittman Jana, Touyz Stephen, Maguire Sarah Treatment Considerations for Compulsive Exercise in High-Performance Athletes with an

Eating Disorder. SPORTS MEDICINE-OPEN. 2022;8(1):.

1603. Calzo Jerel, Blashill Aaron, Brown Tiffany, Argenal Russell Eating Disorders and Disordered Weight and Shape Control Behaviors in Sexual Minority Populations. CURRENT PSYCHIATRY REPORTS. 2017;19(8):.

1604. Austin SB Population-based prevention of eating disorders: An application of the Rose prevention model. PREVENTIVE MEDICINE. 2001;32(3):268-283.

1605. Molendijk M., Hoek H., Brewerton T., Elzinga B. Childhood maltreatment and eating disorder pathology: a systematic review and dose-response meta-analysis. PSYCHOLOGICAL MEDICINE. 2017;47(8):1402-1416.

1606. Roosen M., Safer D., Adler S., Cebolla A., Strien T. Group dialectical behavior therapy adapted for obese emotional eaters; a pilot study. NUTRICION HOSPITALARIA. 2012;27(4):1141-1147.

1607. Kass Andrea, Trockel Mickey, Safer Debra, Sinton Meghan, Cunning Darby, Rizk Marianne, Genkin Brooke, Weisman Hannah, Bailey Jakki, Jacobi Corinna, Wilfley Denise, Taylor C. Internet-based preventive intervention for reducing eating disorder risk: A randomized controlled trial comparing guided with unguided self-help. BEHAVIOUR RESEARCH AND THERAPY. 2014;63():90-98.

1608. Stice Eric, Davis Kendra, Miller Nicole, Marti C. Fasting Increases Risk for Onset of Binge Eating and Bulimic Pathology: A 5-Year Prospective Study. JOURNAL OF ABNORMAL PSYCHOLOGY. 2008;117(4):941-946.

1609. McNicholas Fiona, Dooley Barbara, McNamara Niamh, Lennon Ruth The Impact of Self-Reported Pubertal Status and Pubertal Timing on Disordered Eating in Irish Adolescents. EUROPEAN EATING DISORDERS REVIEW. 2012;20(5):355-362.

1610. Lowe Michael, Arigo Danielle, Butryn Meghan, Gilbert Jennifer, Sarwer David, Stice Eric Hedonic Hunger Prospectively Predicts Onset and Maintenance of Loss of Control Eating Among College Women. HEALTH PSYCHOLOGY. 2016;35(3):238-244.

1611. Hautala Lea, Junnila Jouni, Helenius Hans, Vaananen Aija-Mari, Liuksila Pirjo-Riitta, Raiha Hannele, Valimaki Maritta, Saarijarvi Simo Towards understanding gender

differences in disordered eating among adolescents. JOURNAL OF CLINICAL NURSING. 2008;17(13):1803-1813.

1612. Saruco Elodie, Pleger Burkhard A Systematic Review of Obesity and Binge Eating Associated Impairment of the Cognitive Inhibition System. FRONTIERS IN NUTRITION. 2021;8():.

1613. Fergerson Ava, Brausch Amy Resilience Mediates the Relationship Between PTSD Symptoms and Disordered Eating in College Women Who Have Experienced Sexual Victimization. JOURNAL OF INTERPERSONAL VIOLENCE. 2022;37(1-2):NP1013-NP1030.

1614. Loprinzi Paul, Lee I-Min, Andersen Ross, Crespo Carlos, Smit Ellen Association of Concurrent Healthy Eating and Regular Physical Activity With Cardiovascular Disease Risk Factors in US Youth. AMERICAN JOURNAL OF HEALTH PROMOTION. 2015;30(1):2-8.

1615. Ivezaj Valentina, Fu Emily, Lydecker Janet, Duffy Andrew, Grilo Carlos Racial Comparisons of Postoperative Weight Loss and Eating-Disorder Psychopathology Among Patients Following Sleeve Gastrectomy Surgery. OBESITY. 2019;27(5):740-745.

1616. Laguna-Camacho Antonio, Booth David Meals described as healthy or unhealthy match public health education in England. APPETITE. 2015;87():283-287.

1617. Ganson Kyle, Lisi Nicole, O'Connor Julia, Nagata Jason Association between binge eating and physical violence perpetration among US college students. JOURNAL OF EATING DISORDERS. 2022;10(1):.

1618. Mancini Giacomo, Biolcati Roberta, Pupi Virginia, Andrei Federica, La Grutta Sabina, Lo Baido Rosa, Trombini Elena Eating disorders in males: an overview of research over the period 2007-2017. RIVISTA DI PSICHIATRIA. 2018;53(4):177-191.

1619. Bolanos-Rios P., Jauregui-Lobera I. Overweight and obesity in eating disorders. REVISTA ESPANOLA DE NUTRICION COMUNITARIA-SPANISH JOURNAL OF COMMUNITY NUTRITION. 2010;16(2):83-89.

1620. Berchio Cristina, Cambi Susanne, Pappaianni Edoardo, Micali Nadia EEG Biomarkers in Children and Adolescents With Feeding and Eating Disorders: Current Evidence and

Future Directions. FRONTIERS IN PSYCHIATRY. 2022;13():.

1621. Thomas M., Lovell A. Anxiety and compulsion patterns in the maintenance of bingeing/purging behaviours by individuals with bulimia nervosa. JOURNAL OF PSYCHIATRIC AND MENTAL HEALTH NURSING. 2015;22(1):20-29.

1622. Siqueira KS, Appolinario JC, Sichieri R Relationship between binge-eating episodes and self-perception of body weight in a nonclinical sample of five Brazilian cities. REVISTA BRASILEIRA DE PSIQUIATRIA. 2005;27(4):290-294.

1623. Squires Claire, Lalanne Christophe, Murday Nasha, Simoglou Vassiliki, Vaivre-Douret Laurence The influence of eating disorders on mothers' sensitivity and adaptation during feeding: a longitudinal observational study. BMC PREGNANCY AND CHILDBIRTH. 2014;14():.

1624. Modrzejewska Renata Comorbidity in adolescence simultaneous declaration of depressive, eating, obsessive-compulsive symptoms and use of psychoactive substances m the general population of 17 year old students in a big city. PSYCHIATRIA POLSKA. 2010;44(5):651-663.

1625. Pokrajac-Bulian Alessandra, Tkalcic Mladenka, Ambrosi-Randic Neala Binge eating as a determinant of emotional state in overweight and obese males with cardiovascular disease. MATURITAS. 2013;74(4):352-356.

1626. Dubovi Abigail, Li Yue, Martin Jessica Breaking the Silence: Disordered Eating and Big Five Traits in College Men. AMERICAN JOURNAL OF MENS HEALTH. 2016;10(6):N118-N126.

1627. Laczkovics Clarissa, Czernin Klara, Carlitscheck Jessica, Zeiler Michael, Schlund Pauline, Wunram Heidrun, Lehmkuhl Gerd, Krischer Maya Personality Disorder in Adolescent Patients with Anorexia Nervosa. PSYCHOPATHOLOGY. 2022;():.

1628. Puglisi Francesco, Antonucci Nicola, Capuano Palma, Zavoianni Leonardo, Lobascio Pierluigi, Martines Gennaro, Lograno Giuseppe, Memeo Vincenzo Intragastric balloon and binge eating. OBESITY SURGERY. 2007;17(4):504-509.

1629. Thompson Alexandra, Petrie Trent, Balcom Kayla, Tackett Bailey, Watkins Jr Edward Psychosocial predictors of eating classification in female athletes: From collegiate sport to retirement. INTERNATIONAL JOURNAL OF EATING DISORDERS. 2021;54(4):646-651.

1630. Luce Kristine, Crowther Janis, Pole Michele Eating Disorder Examination Questionnaire (EDE-Q): Norms for undergraduate women. INTERNATIONAL JOURNAL OF EATING DISORDERS. 2008;41(3):273-276.

1631. Lavender Jason, Brown Tiffany, Murray Stuart Men, Muscles, and Eating Disorders: an Overview of Traditional and Muscularity-Oriented Disordered Eating. CURRENT PSYCHIATRY REPORTS. 2017;19(6):.

1632. Stoving Rene, Andries Alin, Brixen Kim, Bilenberg Niels, Horder Kirsten Gender differences in outcome of eating disorders: A retrospective cohort study. PSYCHIATRY RESEARCH. 2011;186(2-3):362-366.

1633. Tohid H., Ooi C., Leong Y., Ngasri Mohamad, Ismail M., Alwi Mohamad, Jafri N., Monoto Md Meal Skipping among Patients with Type 2 Diabetes Mellitus (T2DM) and Its Associations with Glycaemic Control, Eating Out of Home and Binge Eating. MEDICINE AND HEALTH. 2020;15(2):246-261.

1634. Dicker SL, Craighead LW Appetite-focused cognitive-behavioral therapy in the treatment of binge eating with purging. COGNITIVE AND BEHAVIORAL PRACTICE. 2004;11(2):213-221.

1635. Lister Natalie, Baur Louise, Paxton Susan, Garnett Sarah, Ahern Amy, Wilfley Denise, Maguire Sarah, Sainsbury Amanda, Steinbeck Katharine, Braet Caroline, Hill Andrew, Nicholls Dasha, Jones Rebecca, Dammery Genevieve, Grunseit Alicia, Cooper Kelly, Kyle Theodore, Heeren Faith, Hunter Kylie, McMaster Caitlin, Johnson Brittany, Seidler Anna, Jebeile Hiba Eating Disorders In weight-related Therapy (EDIT) Collaboration: rationale and study design. NUTRITION RESEARCH REVIEWS. 2023;():.

1636. Zaitsoff Shannon, Pullmer Rachelle, Coelho Jennifer A longitudinal examination of body-checking behaviors and eating disorder pathology in a community sample of adolescent males and females. INTERNATIONAL JOURNAL OF EATING DISORDERS. 2020;53(11):1836-1843.

1637. Murray Stuart, Duval Christina, Balkchyan Ane, Cabeen Ryan, Nagata Jason, Toga Arthur, Siegel Steven, Jann Kay Regional gray matter abnormalities in pre-adolescent binge eating disorder: A voxel-based morphometry study. PSYCHIATRY RESEARCH. 2022;310():.

1638. Wilson GT Treatment of bulimia nervosa: When CBT fails. BEHAVIOUR RESEARCH AND THERAPY. 1996;34(3):197-212.

1639. Samuels Karen, Maine Margo, Tantillo Mary Disordered Eating, Eating Disorders, and Body Image in Midlife and Older Women. CURRENT PSYCHIATRY REPORTS. 2019;21(8):.

1640. Stice Eric, Sysko Robyn, Roberto Christina, Allison Shelley Are dietary restraint scales valid measures of dietary restriction? Additional objective behavioral and biological data suggest not. APPETITE. 2010;54(2):331-339.

1641. Schreyer Colleen, Guarda Angela, Pletch Allisyn, Redgrave Graham, Salwen-Deremer Jessica, Coughlin Janelle A modified inpatient eating disorders treatment protocol for postbariatric surgery patients: patient characteristics and treatment response. SURGERY FOR OBESITY AND RELATED DISEASES. 2019;15(9):1612-1619.

1642. Erford Bradley, Richards Taryn, Peacock Elizabeth, Voith Karen, McGair Heather, Muller Brooke, Duncan Kelly, Chang Catherine Counseling and Guided Self-Help Outcomes for Clients With Bulimia Nervosa: A Meta-Analysis of Clinical Trials From 1980 to 2010. JOURNAL OF COUNSELING AND DEVELOPMENT. 2013;91(2):152-172.

1643. Wang Shirley, Borders Ashley The unique effects of angry and depressive rumination on eating-disorder psychopathology and the mediating role of impulsivity. EATING BEHAVIORS. 2018;29():41-47.

1644. Pike K., Hilbert A., Wilfley D., Fairburn C., Dohms F., Walsh B., Striegel-Moores R. Toward an understanding of risk factors for anorexia nervosa: a case-control study. PSYCHOLOGICAL MEDICINE. 2008;38(10):1443-1453.

1645. Lynch Wesley, Heil Daniel, Wagner Elise, Havens Michael Body dissatisfaction mediates the association between body mass index and risky weight control behaviors among White and Native American adolescent girls. APPETITE. 2008;51(1):210-213.

1646. Nunes Maria, Olinto Maria, Camey Suzi, Morgan Christina, Mari Jair Abnormal eating behaviors in adolescent and young adult women from southern Brazil: Reassessment after four years. SOCIAL PSYCHIATRY AND PSYCHIATRIC EPIDEMIOLOGY. 2006;41(12):951-956.

1647. Al-Kloub Manal, Al-Khawaldeh Omar, ALBashtawy Mohammed, Batiha Abdul-Monim, Al-Haliq Mahmoud Disordered eating in Jordanian adolescents. INTERNATIONAL JOURNAL OF NURSING PRACTICE. 2019;25(1):.

1648. Streatfeild Jared, Hickson Josiah, Austin S., Hutcheson Rebecca, Kandel Johanna, Lampert Jillian, Myers Elissa, Richmond Tracy, Samnaliev Mihail, Velasquez Katrina, Weissman Ruth, Pezzullo Lynne Social and economic cost of eating disorders in the United States: Evidence to inform policy action. INTERNATIONAL JOURNAL OF EATING DISORDERS. 2021;54(5):851-868.

1649. Ellis Jordan, Schenk Rebecca, Galloway Amy, Zickgraf Hana, Webb Rose, Martz Denise A multidimensional approach to understanding the potential risk factors and covariates of adult picky eating. APPETITE. 2018;125():1-9.

1650. Maraldo Toni, Zhou Wanni, Dowling Jessica, Wal Jillon Replication and extension of the dual pathway model of disordered eating: The role of fear of negative evaluation, suggestibility, rumination, and self-compassion. EATING BEHAVIORS. 2016;23():187-194.

1651. Meyer Lene, Waaddegaard Mette, Lau Marianne, Tjornhoj-Thomsen Tine (Dis-)solving the Weight Problem in Binge-Eating Disorder: Systemic Insights From Three Treatment Contexts With Weight Stability, Weight Loss, and Weight Acceptance. QUALITATIVE HEALTH RESEARCH. 2019;29(4):597-608.

1652. Hazzard Vivienne, Yoon Cynthia, Emery Rebecca, Mason Susan, Crosby Ross, Wonderlich Stephen, Neumark-Sztainer Dianne Adverse childhood experiences in relation to mood-, weight-, and eating-related outcomes in emerging adulthood: Does self-compassion play a buffering role?. CHILD ABUSE \& NEGLECT. 2021;122():.

1653. Beck D, Casper R, Andersen A Truly late onset of eating disorders: A study of 11 cases averaging 60 years of age at presentation. INTERNATIONAL JOURNAL OF EATING DISORDERS. 1996;20(4):389-395.

1654. Chapa Danielle, Johnson Sarah, Richson Brianne, Bjorlie Kayla, Won Ying, Nelson Sarah, Ayres Joseph, Jun Daiil, Forbush Kelsie, Christensen Kara, Perko Victoria Eating-

disorder psychopathology in female athletes and non-athletes: A meta-analysis. INTERNATIONAL JOURNAL OF EATING DISORDERS. 2022;55(7):861-885.

1655. Feibelman Jennifer, Turner Lisa Relationships Between Eating Disorder Symptomology and Forgiveness Among College Students. CURRENT PSYCHOLOGY. 2015;34(1):121-129.

1656. Conti Chiara, Lanzara Roberta, Scipioni Mattia, Iasenza Marzia, Guagnano Maria, Fulcheri Mario The Relationship between Binge Eating Disorder and Suicidality: A Systematic Review. FRONTIERS IN PSYCHOLOGY. 2017;8():.

1657. Cuesta-Zamora Cristina, Parra Marta, Toledano-Gonzalez Abel, Ricarte Jorge, Plateau Carolyn Exploring the Link Between Self-compassion and Compulsive Exercise Amongst Women. MINDFULNESS. 2022;13(7):1679-1691.

1658. Nakai Yoshikatsu, Noma Shun'ichi, Fukusima Mitsuo, Taniguchi Ataru, Teramukai Satoshi Serum Lipid Levels in Patients with Eating Disorders. INTERNAL MEDICINE. 2016;55(14):1853-1857.

1659. Eisenberg ME, Neumark-Sztainer D, Story M, Perry C The role of social norms and friends' influences on unhealthy weight-control behaviors among adolescent girls. SOCIAL SCIENCE \& MEDICINE. 2005;60(6):1165-1173.

1660. Bryden KS, Neil A, Mayou RA, Peveler RC, Fairburn CG, Dunger DB Eating habits, body weight and insulin misuse - A longitudinal study of teenagers and young adults with type 1 diabetes. DIABETES CARE. 1999;22(12):1956-1960.

1661. Watson Hunna, Steele Anna, Bergin Jacqueline, Fursland Anthea, Wade Tracey Bulimic symptomatology: The role of adaptive perfectionism, shape and weight concern, and self-esteem. BEHAVIOUR RESEARCH AND THERAPY. 2011;49(9):565-572.

1662. Arthur-Cameselle Jessyca, Quatromoni Paula Factors Related to the Onset of Eating Disorders Reported by Female Collegiate Athletes. SPORT PSYCHOLOGIST. 2011;25(1):1-17.

1663. Romano Adele, Di Bonaventura Maria, Gallelli Cristina, Koczwara Justyna, Smeets Dorien, Giusepponi Maria, De Ceglia Marialuisa, Friuli Marzia, Di Bonaventura Emanuela,

Scuderi Caterina, Vitalone Annabella, Tramutola Antonella, Altieri Fabio, Lutz Thomas, Giudetti Anna, Cassano Tommaso, Cifani Carlo, Gaetani Silvana Oleoylethanolamide decreases frustration stress-induced binge-like eating in female rats: a novel potential treatment for binge eating disorder. NEUROPSYCHOPHARMACOLOGY. 2020;45(11):1931-1941.

1664. Hebebrand J, Fichter M, Gerber G, Gorg T, Hermann H, Geller F, Schafer H, Remschmidt H, Hinney A Genetic predisposition to obesity in bulimia nervosa: a mutation screen of the melanocortin-4 receptor gene. MOLECULAR PSYCHIATRY. 2002;7(6):647-651.

1665. Daeie-Farshbaf Lida, Ebrahimi-Mameghani Mehrangiz, Sarbakhsh Parvin, Roshanravan Neda, Tarighat-Esfanjani Ali Age at menarche, eating disorders, and their relationships with some parameters in female adolescents in Iran. BMC RESEARCH NOTES. 2021;14(1):.

1666. Egbert Amy, Wilfley Denise, Eddy Kamryn, Boutelle Kerri, Zucker Nancy, Peterson Carol, Doyle Angela, Le Grange Daniel, Goldschmidt Andrea Attention-Deficit/Hyperactivity Disorder Symptoms Are Associated with Overeating with and without Loss of Control in Youth with Overweight/Obesity. CHILDHOOD OBESITY. 2018;14(1):50-57.

1667. Lee Warren An overview of pediatric obesity. PEDIATRIC DIABETES. 2007;8(9):76-87.

1668. Hilbert A., Voegele C., Himmelmann U. Cue reactivity in male restrained eaters: The role of negative cognitions as predictors of food intake. EATING AND WEIGHT DISORDERS-STUDIES ON ANOREXIA BULIMIA AND OBESITY. 2007;12(1):27-34.

1669. Bachner-Melman R., Zohar A., Elizur Y., Kremer I., Golan M., Ebstein R. Protective self-presentation style: Association with disordered eating and anorexia nervosa mediated by sociocultural attitudes towards appearance. EATING AND WEIGHT DISORDERS-STUDIES ON ANOREXIA BULIMIA AND OBESITY. 2009;14(1):1-12.

1670. Vansteenkiste M, Soenens B, Vandereycken W Motivation to change in eating disorder patients: A conceptual clarification on the basis of self-determination theory. INTERNATIONAL JOURNAL OF EATING DISORDERS. 2005;37(3):207-219.

1671. De Pasquale Concetta, Morando Martina, Platania Silvia, Sciacca Federica, Hichy Zira, Di Nuovo Santo, Quattropani Maria The Roles of Anxiety and Self-Esteem in the Risk of

Eating Disorders and Compulsive Buying Behavior. INTERNATIONAL JOURNAL OF ENVIRONMENTAL RESEARCH AND PUBLIC HEALTH. 2022;19(23):.

1672. Hahn Samantha, Burnette C., Borton Kelley, Carpenter Lisa, Sonneville Kendrin, Bailey Beth Eating disorder risk in rural US adolescents: What do we know and where do we go?. INTERNATIONAL JOURNAL OF EATING DISORDERS. 2022;():.

1673. Nagata Jason, Murray Stuart Updates in the treatment of eating disorders in 2020: a year in review in eating disorders: the journal of treatment \& prevention. EATING DISORDERS. 2021;29(2):123-133.

1674. Artoni P., Chierici M., Arnone F., Cigarini C., De Bernardis E., Galeazzi G., Minneci D., Scita F., Turrini G., De Bernardis M., Pingani L. Body perception treatment, a possible way to treat body image disturbance in eating disorders: a case-control efficacy study. EATING AND WEIGHT DISORDERS-STUDIES ON ANOREXIA BULIMIA AND OBESITY. 2021;26(2):499-514.

1675. Neumark-Sztainer D, Story M, Dixon LB, Murray DM Adolescents engaging in unhealthy weight control behaviors: Are they at risk for other health-compromising behaviors?. AMERICAN JOURNAL OF PUBLIC HEALTH. 1998;88(6):952-955.

1676. Bornioli Anna, Lewis-Smith Helena, Smith Andrew, Slater Amy, Bray Isabelle Adolescent body dissatisfaction and disordered eating: Predictors of later risky health behaviours. SOCIAL SCIENCE \& MEDICINE. 2019;238():.

1677. Stice Eric, Rohde Paul, Shaw Heather, Gau Jeff Randomized Trial of a Dissonance-Based Group Treatment for Eating Disorders Versus a Supportive Mindfulness Group Treatment. JOURNAL OF CONSULTING AND CLINICAL PSYCHOLOGY. 2019;87(1):79-90.

1678. Armour Cherie, Mullerova Jana, Fletcher Shelley, Lagdon Susan, Burns Carol, Robinson Martin, Robinson Jake Assessing childhood maltreatment and mental health correlates of disordered eating profiles in a nationally representative sample of English females. SOCIAL PSYCHIATRY AND PSYCHIATRIC EPIDEMIOLOGY. 2016;51(3):383-393.

1679. Hudson Tassiana, Soares Amaral Ana, Stice Eric, Gau Jeff, Caputo Ferreira Maria Dissonance-based eating disorder prevention among Brazilian young women: A randomized efficacy trial of the Body Project. BODY IMAGE. 2021;38():1-9.

1680. Krug Isabel, Casasnovas Carolina, Granero Roser, Martinez Cristina, Jimenez-Murcia Susana, Bulik Cynthia, Fernandez-Aranda Fernando Comparison study of full and subthreshold bulimia nervosa: Personality, clinical characteristics, and short-term response to therapy. PSYCHOTHERAPY RESEARCH. 2008;18(1):37-47.

1681. Scherag Susann, Hebebrand Johannes, Hinney Anke Eating disorders: the current status of molecular genetic research. EUROPEAN CHILD \& ADOLESCENT PSYCHIATRY. 2010;19(3, SI):211-226.

1682. Son Gabrielle, Hoeken Daphne, Furth Eric, Donker Ge, Hoek Hans Course and Outcome of Eating Disorders in a Primary Care-Based Cohort. INTERNATIONAL JOURNAL OF EATING DISORDERS. 2010;43(2):130-138.

1683. Aspen Vandana, Weisman Hannah, Vannucci Anna, Nafiz Najia, Gredysa Dana, Kass Andrea, Trockel Mickey, Jacobi Corinna, Wilfley Denise, Taylor C. Psychiatric co-morbidity in women presenting across the continuum of disordered eating. EATING BEHAVIORS. 2014;15(4):686-693.

1684. Ackard DM, Neumark-Sztainer D, Story M, Perry C Overeating among adolescents: Prevalence and associations with weight-related characteristics and psychological health. PEDIATRICS. 2003;111(1):67-74.

1685. Quattlebaum Mary, Burke Natasha, Neyland M., Leu William, Schvey Natasha, Pine Abigail, Morettini Alexandria, LeMay-Russell Sarah, Wilfley Denise, Stephens Mark, Sbrocco Tracy, Yanovski Jack, Jorgensen Sarah, Olsen Cara, Klein David, Quinlan Jeffrey, Tanofsky-Kraff Marian Sex differences in eating related behaviors and psychopathology among adolescent military dependents at risk for adult obesity and eating disorders. EATING BEHAVIORS. 2019;33():73-77.

1686. Kamody Rebecca, Thurston Idia, Burton E. Acceptance-based skill acquisition and cognitive reappraisal in a culturally responsive treatment for binge eating in adolescence. EATING DISORDERS. 2020;28(2, SI):184-201.

1687. Klatzkin Rebecca, Gaffney Sierra, Cyrus Kathryn, Bigus Elizabeth, Brownley Kimberly Stress-induced eating in women with binge-eating disorder and obesity. BIOLOGICAL PSYCHOLOGY. 2018;131(SI):96-106.

1688. Luethcke Cynthia, McDaniel Leda, Becker Carolyn A comparison of mindfulness, nonjudgmental, and cognitive dissonance-based approaches to mirror exposure. BODY IMAGE. 2011;8(3):251-258.

1689. Fornaro M., Perugi G., Gabrielli F., Prestia D., Mattei C., Vinciguerra V., Fornaro P. Lifetime co-morbidity with different subtypes of eating disorders in 148 females with bipolar disorders. JOURNAL OF AFFECTIVE DISORDERS. 2010;121(1-2):147-151.

1690. Devlin MJ Binge-eating disorder and obesity - A combined treatment approach. PSYCHIATRIC CLINICS OF NORTH AMERICA. 2001;24(2):325+.

1691. Price Amanda, Brehm Victoria, Hommel Jonathan, Anastasio Noelle, Cunningham Kathryn Pimavanserin and Lorcaserin Attenuate Measures of Binge Eating in Male Sprague-Dawley Rats. FRONTIERS IN PHARMACOLOGY. 2018;9():.

1692. Buehren K., Schwarte R., Fluck F., Timmesfeld N., Krei M., Egberts K., Pfeiffer E., Fleischhaker C., Wewetzer C., Herpertz-Dahlmann B. Comorbid Psychiatric Disorders in Female Adolescents with First-Onset Anorexia Nervosa. EUROPEAN EATING DISORDERS REVIEW. 2014;22(1):39-44.

1693. Reinehr Thomas, Dieris Barbara, Galler Angela, Teufel Martin, Berger Gabriele, Stachow Rainer, Golembowski Sven, Ohlenschlager Ute, Holder Martin, Hummel Michael, Holl Reinhard, Prinz Nicole Worse Metabolic Control and Dynamics of Weight Status in Adolescent Girls Point to Eating Disorders in the First Years after Manifestation of Type 1 Diabetes Mellitus: Findings from the Diabetes Patienten Verlaufsdokumentation Registry. JOURNAL OF PEDIATRICS. 2019;207():205-212.

1694. Hunt Tyler, Forbush Kelsie, Hagan Kelsey, Chapa Danielle Do emotion regulation difficulties when upset influence the association between dietary restraint and weight gain among college students?. APPETITE. 2017;114():101-109.

1695. Pereira Peixoto Maciel Ana, Santos Arianny, Silva Emerson, Umena Messias Cristhiane Night eating and binge eating disorder in employees of an academic institution. MUNDO DA SAUDE. 2018;42(1):142-157.

1696. Calzo Jerel, Turner Blair, Marro Rachel, Phillips Gregory Alcohol Use and Disordered Eating in a US Sample of Heterosexual and Sexual Minority Adolescents. JOURNAL OF THE

AMERICAN ACADEMY OF CHILD AND ADOLESCENT PSYCHIATRY. 2019;58(2):200-210.

1697. Yu Zhiping, Tan Michael Disordered Eating Behaviors and Food Addiction among Nutrition Major College Students. NUTRIENTS. 2016;8(11):.

1698. Evans Brittney, Felton Julia, Lagacey Madeline, Manasse Stephanie, Lejuez Carl, Juarascio Adrienne Impulsivity and affect reactivity prospectively predict disordered eating attitudes in adolescents: a 6-year longitudinal study. EUROPEAN CHILD \& ADOLESCENT PSYCHIATRY. 2019;28(9):1193-1202.

1699. Dell'Osso L., Carpita B., Gesi C., Cremone I., Corsi M., Massimetti E., Muti D., Calderani E., Castellini G., Luciano M., Ricca V., Carmassi C., Maj M. Subthreshold autism spectrum disorder in patients with eating disorders. COMPREHENSIVE PSYCHIATRY. 2018;81():66-72.

1700. Linardon Jake Positive body image, intuitive eating, and self-compassion protect against the onset of the core symptoms of eating disorders: A prospective study. INTERNATIONAL JOURNAL OF EATING DISORDERS. 2021;54(11):1967-1977.

1701. Kennedy Samantha, Kovan Jeffrey, Werner Emily, Mancine Ryley, Gusfa Donald, Kleiman Heather Initial validation of a screening tool for disordered eating in adolescent athletes. JOURNAL OF EATING DISORDERS. 2021;9(1):.

1702. Hincapie Cesar, Cassidy J. Disordered Eating, Menstrual Disturbances, and Low Bone Mineral Density in Dancers: A Systematic Review. ARCHIVES OF PHYSICAL MEDICINE AND REHABILITATION. 2010;91(11):1777-1789.

1703. Ziauddeen H., Chamberlain S., Nathan P., Koch A., Maltby K., Bush M., Tao W., Napolitano A., Skeggs A., Brooke A., Cheke L., Clayton N., Farooqi I., O'Rahilly S., Waterworth D., Song K., Hosking L., Richards D., Fletcher P., Bullmore E. Effects of the mu-opioid receptor antagonist GSK1521498 on hedonic and consummatory eating behaviour: a proof of mechanism study in binge-eating obese subjects. MOLECULAR PSYCHIATRY. 2013;18(12):1287-1293.

1704. Bartholdy Savani, O'Daly Owen, Campbell Iain, Banaschewski Tobias, Barker Gareth, Bokde Arun, Bromberg Uli, Buechel Christian, Quinlan Erin, Desrivieres Sylvane, Flor Herta, Frouin Vincent, Garavan Hugh, Gowland Penny, Heinz Andreas, Ittermann Bernd, Martinot Jean-Luc, Martinot Marie-Laure, Nees Frauke, Orfanos Dimitri, Poustka Luise, Hohmann

Sarah, Froehner Juliane, Smolka Michael, Walter Henrik, Whelan Robert, Schumann Gunter, Schmidt Ulrike, Rapp Michael, Artiges Eric, Schneider Sophia, Bach Christine, Paus Tomas, Barbot Alexis, Bokde Arun, Vetter Nora, Cattrell Anna, Constant Patrick, Crombag Hans, Czech Katharina, Dalley Jeffrey, Decideur Benjamin, Spranger Tade, Ripley Tamzin, Heym Nadja, Sommer Wolfgang, Fuchs Birgit, Gallinat Juegen, Spanagel Rainer, Kaviani Mehri, Heinrichs Bert, Subramaniam Naresh, Jia Tianye, Ihlenfeld Albrecht, Ireland James, Conrod Patricia, Klaassen Arno, Lanzerath Dirk, Lawrence Claire, Lemaitre Herve, Mallik Catherine, Mann Karl, Mar Adam, Martinez-Medina Lourdes, Mennigen Eva, Carvahlo Fabiana, Schwartz Yannick, Bruehl Ruediger, Mueller Kathrin, Nymberg Charlotte, Lathrop Mark, Robbins Trevor, Pausova Zdenka, Pentilla Jani, Biondo Francesca, Poline Jean-Baptiste, Millenet Sabina, Smolka Michael, Froehner Juliane, Struve Maren, Williams Steve, Huebner Thomas, Aydin Semiha, Rogers John, Romanowski Alexander, Schmael Christine, Schmidt Dirk, Ripke Stephan, Arroyo Mercedes, Schubert Florian, Pena-Oliver Yolanda, Fauth-Buehler Mira, Mignon Xavier, Speiser Claudia, Fadai Tahmine, Stephens Dai, Stroehle Andreas, Paillere Marie-Laure, Strache Nicole, Theobald David, Jurk Sarah, Vulser Helene, Miranda Ruben, Yacubian Juliana, Genauck Alexander, Parchetka Caroline, Gemmeke Isabel, Kruschwitz Johann, Weiss Katharina, Feng Jianfeng, Filippi Irina, Ing Alex, Ruggeri Barbara, Xu Bing, Macare Christine, Chu Congying, Hanratty Eanna, Robert Gabriel, Yu Tao, Ziesch Veronika, Stedman Alicia, Consortium IMAGEN Neural Correlates of Failed Inhibitory Control as an Early Marker of Disordered Eating in Adolescents. BIOLOGICAL PSYCHIATRY. 2019;85(11):956-965.

1705. Legenbauer Tanja, Preuss Hanna Improving Impulse and Emotion Regulation in Binge Eating Disorder: Possible Application and First Results of the ImpulsE Manual. KINDHEIT UND ENTWICKLUNG. 2019;28(4):210-219.

1706. Sokol MS, Gray NS Case study: An infection-triggered, autoimmune subtype of anorexia nervosa. JOURNAL OF THE AMERICAN ACADEMY OF CHILD AND ADOLESCENT PSYCHIATRY. 1997;36(8):1128-1133.

1707. Duarte Cristiana, Ferreira Claudia, Trindade Ines, Pinto-Gouveia Jose Normative body dissatisfaction and eating psychopathology in teenage girls: the impact of inflexible eating rules. EATING AND WEIGHT DISORDERS-STUDIES ON ANOREXIA BULIMIA AND OBESITY. 2016;21(1):41-48.

1708. Webb Jennifer, Applegate Katherine, Grant John A comparative analysis of Type 2 diabetes and binge eating disorder in a bariatric sample. EATING BEHAVIORS. 2011;12(3):175-181.

1709. Rojo-Moreno Luis, Arribas Pilar, Plumed Javier, Gimeno Natalia, Garcia-Blanco Ana, Vaz-Leal Francisco, Luisa Vila Maria, Livianos Lorenzo Prevalence and comorbidity of eating disorders among a community sample of adolescents: 2-year follow-up. PSYCHIATRY RESEARCH. 2015;227(1):52-57.

1710. Croll J, Neumark-Sztainer D, Story M, Ireland M Prevalence and risk and protective factors related to disordered eating behaviors among adolescents: Relationship to gender and ethnicity. JOURNAL OF ADOLESCENT HEALTH. 2002;31(2):166-175.

1711. Cavicchioli Marco, Ramella Pietro, Vassena Giulia, Simone Giulia, Prudenziati Francesca, Sirtori Federica, Movalli Mariagrazia, Maffei Cesare Dialectical behaviour therapy skills training for the treatment of addictive behaviours among individuals with alcohol use disorder: the effect of emotion regulation and experiential avoidance. AMERICAN JOURNAL OF DRUG AND ALCOHOL ABUSE. 2020;46(3):368-384.

1712. Baylan Gonul, Erol Atila, Kilicoglu Alev Predictors of Anorectic and Bulimic Symptoms in Adolescent Girls. KLINIK PSIKOFARMAKOLOJI BULTENI-BULLETIN OF CLINICAL PSYCHOPHARMACOLOGY. 2009;19(4):407-413.

1713. Scott Charlotte, Haycraft Emma, Plateau Carolyn Teammate influences on the eating attitudes and behaviours of athletes: A systematic review. PSYCHOLOGY OF SPORT AND EXERCISE. 2019;43():183-194.

1714. Borg Dana, Hall Kate, Youssef George, Sloan Elise, Graeme Liam, Moulding Richard Examining the role of brooding, distress, and negative urgency in dysregulated behaviors: A cross-sectional study in treatment-seeking young people. JOURNAL OF CLINICAL PSYCHOLOGY. 2022;78(12):2538-2563.

1715. GERACIOTI TD, LOOSEN PT, EBERT MH, SCHMIDT D, EKHATOR NN FASTING AND POSTPRANDIAL CEREBROSPINAL-FLUID GLUCOSE-CONCENTRATIONS IN HEALTHY WOMEN AND IN AN OBESE BINGE EATER. INTERNATIONAL JOURNAL OF EATING DISORDERS. 1995;18(4):365-369.

1716. Howell Michael, Schenck Carlos, Crow Scott A review of nighttime eating disorders. SLEEP MEDICINE REVIEWS. 2009;13(1):23-34.

1717. Eli Karin Striving for liminality: Eating disorders and social suffering. TRANSCULTURAL PSYCHIATRY. 2018;55(4, SI):475-494.

1718. Guo Lei, Gu Lian, Peng Yihua, Gao Yiming, Mei Li, Kang Qing, Chen Chen, Hu Yanran, Xu Wenyan, Chen Jue Online media exposure and weight and fitness management app use correlate with disordered eating symptoms: evidence from the mainland of China. JOURNAL OF EATING DISORDERS. 2022;10(1):.

1719. Monell Elin, Clinton David, Birgegard Andreas Emotion dysregulation and eating disordersAssociations with diagnostic presentation and key symptoms. INTERNATIONAL JOURNAL OF EATING DISORDERS. 2018;51(8):921-930.

1720. Khosravi Maryam, Sotoudeh Gity, Majdzadeh Reza, Nejati Somayeh, Darabi Samaneh, Raisi Firoozeh, Esmaillzadeh Ahmad, Sorayani Maryam Healthy and Unhealthy Dietary Patterns Are Related to Depression: A Case-Control Study. PSYCHIATRY INVESTIGATION. 2015;12(4):434-442.

1721. Hodgins David, Ranson Kristin, Montpetit Carlie Problem Drinking, Gambling and Eating Among Undergraduate University Students. What are the Links?. INTERNATIONAL JOURNAL OF MENTAL HEALTH AND ADDICTION. 2016;14(2):181-199.

1722. Murray Stuart, Alba Celina, Duval Christina, Nagata Jason, Cabeen Ryan, Lee Darrin, Toga Arthur, Siegel Steven, Jann Kay Aberrant functional connectivity between reward and inhibitory control networks in pre-adolescent binge eating disorder. PSYCHOLOGICAL MEDICINE. 2022;():.

1723. Aoun Carla, Nassar Lynn, Soumi Stephanie, El Osta Nada, Papazian Tatiana, Khabbaz Lydia The Cognitive, Behavioral, and Emotional Aspects of Eating Habits and Association With Impulsivity, Chronotype, Anxiety, and Depression: A Cross-Sectional Study. FRONTIERS IN BEHAVIORAL NEUROSCIENCE. 2019;13():.

1724. Corsica Joyce, Pelchat Marcia Food addiction: true or false?. CURRENT OPINION IN GASTROENTEROLOGY. 2010;26(2):165-169.

1725. Tanofsky-Kraff Marian, Yanovski Susan, Schvey Natasha, Olsen Cara, Gustafson Jennifer, Yanovski Jack A Prospective Study of Loss of Control Eating for Body Weight Gain in Children at High Risk for Adult Obesity. INTERNATIONAL JOURNAL OF EATING

DISORDERS. 2009;42(1):26-30.

1726. Murakami Jessica, Essayli Jamal, Latner Janet The relative stigmatization of eating disorders and obesity in males and females. APPETITE. 2016;102():77-82.

1727. Forghieri M., Monzani D., Mackinnon A., Ferrari S., Gherpelli C., Galeazzi G. Posturographic destabilization in eating disorders in female patients exposed to body image related phobic stimuli. NEUROSCIENCE LETTERS. 2016;629():155-159.

1728. Beltran-Carrillo Vicente, Megias Angel, Gonzalez-Cutre David, Jimenez-Loaisa Alejandro Elements behind sedentary lifestyles and unhealthy eating habits in individuals with severe obesity. INTERNATIONAL JOURNAL OF QUALITATIVE STUDIES ON HEALTH AND WELL-BEING. 2022;17(1):.

1729. Baker Jessica, Thornton Laura, Strober Michael, Brandt Harry, Crawford Steve, Fichter Manfred, Halmi Katherine, Johnson Craig, Jones Ian, Kaplan Allan, Klump Kelly, Mitchell James, Treasure Janet, Woodside D., Berrettini Wade, Kaye Walter, Bulik Cynthia Temporal sequence of comorbid alcohol use disorder and anorexia nervosa. ADDICTIVE BEHAVIORS. 2013;38(3):1704-1709.

1730. Morales Pernalete Adaucio, Gordillo Gutierrez Carlos, Perez Alvarado Carlos, Marcano Flores Daniela, Perez Perez Francisco, Flores Navas Heymir, Perez Navea Jenifer, Perez Linarez Mayela, Melendez Flores Patricia Risk factors for binge eating disorders and its association with obesity in adolescents. GACETA MEDICA DE MEXICO. 2014;150(1):125-131.

1731. Bauer Anika, Schneider Silvia, Waldorf Manuel, Braks Karsten, Huber Thomas, Adolph Dirk, Vocks Silja Selective Visual Attention Towards Oneself and Associated State Body Satisfaction: an Eye-Tracking Study in Adolescents with Different Types of Eating Disorders. JOURNAL OF ABNORMAL CHILD PSYCHOLOGY. 2017;45(8, SI):1647-1661.

1732. Zabinski MF, Pung MA, Wilfley DE, Eppstein DL, Winzelberg AJ, Celio A, Taylor CB Reducing risk factors for eating disorders: Targeting at-risk women with a computerized psychoeducational program. INTERNATIONAL JOURNAL OF EATING DISORDERS. 2001;29(4):401-408.

1733. Godart NT, Perdereau F, Curt F, Rein Z, Lang F, Venisse JL, Halfon O, Bizouard P, Loas G, Corcos M, Jeammet P, Flament MF Is major depressive episode related to anxiety

disorders in anorexics and bulimics?. COMPREHENSIVE PSYCHIATRY. 2006;47(2):91-98.

1734. Mond JM, Hay PJ, Rodgers B, Owen C An update on the definition of ``excessive exercise{''} in eating disorders research. INTERNATIONAL JOURNAL OF EATING DISORDERS. 2006;39(2):147-153.

1735. Wakeling A Epidemiology of anorexia nervosa. PSYCHIATRY RESEARCH. 1996;62(1):3-9.

1736. Schaefer Lauren, Burke Natasha, Thompson J. Thin-ideal internalization: How much is too much?. EATING AND WEIGHT DISORDERS-STUDIES ON ANOREXIA BULIMIA AND OBESITY. 2019;24(5):933-937.

1737. Arigo Danielle, Schumacher Leah, Martin Lindsay Upward Appearance Comparison and the Development of Eating Pathology in College Women. INTERNATIONAL JOURNAL OF EATING DISORDERS. 2014;47(5):467-470.

1738. Presnell Katherine, Stice Eric, Seidel Anke, Madeley Mary Depression and Eating Pathology: Prospective Reciprocal Relations in Adolescents. CLINICAL PSYCHOLOGY \& PSYCHOTHERAPY. 2009;16(4, SI):357-365.

1739. Agras W., Bryson Susan, Hammer Lawrence, Kraemer Helena Childhood risk factors for thin body preoccupation and social pressure to be thin. JOURNAL OF THE AMERICAN ACADEMY OF CHILD AND ADOLESCENT PSYCHIATRY. 2007;46(2):171-178.

1740. Mangweth-Matzek Barbara, Rupp Claudia, Hausmann Armand, Kemmler Georg, Biebl Wilfried Menarche, puberty, and first sexual activities in eating-disordered patients as compared with a psychiatric and a nonpsychiatric control group. INTERNATIONAL JOURNAL OF EATING DISORDERS. 2007;40(8):705-710.

1741. Hazzard Vivienne, Hahn Samantha, Sonneville Kendrin Weight misperception and disordered weight control behaviors among US high school students with overweight and obesity: Associations and trends, 1999-2013. EATING BEHAVIORS. 2017;26():189-195.

1742. Yanovski S Sugar and fat: Cravings and aversions. JOURNAL OF NUTRITION. 2003;133(3):835S-837S.

1743. Muehleck Julia, Borse Sigrid, Wunderer Eva, Strauss Bernhard, Berger Uwe Online survey on the awareness of offers for information, prevention, counselling, and aftercare for eating disorders. PRAVENTION UND GESUNDHEITSFORDERUNG. 2020;15(1):73-79.

1744. Wu Hemmings, Adler Sarah, Azagury Dan, Bohon Cara, Safer Debra, Barbosa Daniel, Bhati Mahendra, Williams Nolan, Dunn Laura, Tass Peter, Knutson Brian, Yutsis Maya, Fraser Ayesha, Cunningham Tricia, Richardson Kara, Skarpaas Tara, Tcheng Thomas, Morrell Martha, Roberts Laura, Malenka Robert, Lock James, Halpern Casey Brain-Responsive Neurostimulation for Loss of Control Eating: Early Feasibility Study. NEUROSURGERY. 2020;87(6):1277-1288.

1745. Sonneville K., Calzo J., Horton N., Haines J., Austin S., Field A. Body satisfaction, weight gain and binge eating among overweight adolescent girls. INTERNATIONAL JOURNAL OF OBESITY. 2012;36(7):944-949.

1746. Solmi Marco, Radua Joaquim, Olivola Miriam, Croce Enrico, Soardo Livia, Pablo Gonzalo, Shin Jae, Kirkbride James, Jones Peter, Kim Jae, Kim Jong, Carvalho Andre, Seeman Mary, Correll Christoph, Fusar-Poli Paolo Age at onset of mental disorders worldwide: large-scale meta-analysis of 192 epidemiological studies. MOLECULAR PSYCHIATRY. 2022;27(1):281-295.

1747. Chen Eunice, McCloskey Michael, Keenan Kathryn Subtyping Dietary Restraint and Negative Affect in a Longitudinal Community Sample of Girls. INTERNATIONAL JOURNAL OF EATING DISORDERS. 2009;42(3):275-283.

1748. Rouzitalab Tohid, Gargari Bahram, Amirsasan Ramin, Jafarabadi Mohammad, Naeimi Alireza, Sanoobar Meisam The Relationship of Disordered Eating Attitudes With Body Composition and Anthropometric Indices in Physical Education Students. IRANIAN RED CRESCENT MEDICAL JOURNAL. 2015;17(11):.

1749. Warnick Jennifer, Darling Katherine, Rancourt Diana The association between negative body talk and body shame on disordered eating symptoms among college students. EATING BEHAVIORS. 2022;46():.

1750. Loth Katie, MacLehose Rich, Bucchianeri Michaela, Crow Scott, Neumark-Sztainer Dianne Predictors of Dieting and Disordered Eating Behaviors From Adolescence to Young Adulthood. JOURNAL OF ADOLESCENT HEALTH. 2014;55(5):705-712.

1751. Xu Furong, Cohen S., Lofgren I., Greene G., Delmonico M., Greaney M. RELATIONSHIP BETWEEN DIET QUALITY, PHYSICAL ACTIVITY AND HEALTH-RELATED QUALITY OF LIFE IN OLDER ADULTS: FINDINGS FROM 2007-2014 NATIONAL HEALTH AND NUTRITION EXAMINATION SURVEY. JOURNAL OF NUTRITION HEALTH \& AGING. 2018;22(9):1072-1079.

1752. Ralph-Nearman Christina, Hooper Madison, Filik Ruth An eye-tracking study examining the relationship between males' eating disorder symptomatology, body mass index, and expectations about character behaviour in text male eating disorder symptoms and reading. COGNITION \& EMOTION. 2021;35(8):1543-1558.

1753. Jacobi Corinna, Beintner Ina, Fittig Eike, Trockel Mickey, Braks Karsten, Schade-Brittinger Carmen, Dempfle Astrid Web-Based Aftercare for Women With Bulimia Nervosa Following Inpatient Treatment: Randomized Controlled Efficacy Trial. JOURNAL OF MEDICAL INTERNET RESEARCH. 2017;19(9):.

1754. Mitchell Tarrah, Steele Ric The Effect of Body Mass Index, Negative Affect, and Disordered Eating on Health-Related Quality of Life in Preadolescent Youth. JOURNAL OF PEDIATRIC PSYCHOLOGY. 2016;41(7):768-776.

1755. Hudson James, McElroy Susan, Ferreira-Cornwell Celeste, Radewonuk Jana, Gasior Maria Efficacy of Lisdexamfetamine in Adults With Moderate to Severe Binge-Eating Disorder A Randomized Clinical Trial. JAMA PSYCHIATRY. 2017;74(9):903-910.

1756. Ulukaya Sema, Onur Ozge, Erkiran Murat Night Eating Syndrome Among Euthymic Patients With Bipolar I Disorder May Be Common and Related With Eating Concerns and Atypical Depression: A Cross-Sectional, Clinic-Based Study. PSYCHIATRY AND CLINICAL PSYCHOPHARMACOLOGY. 2020;30(1):38-46.

1757. McShane Johanna, Zirkel Sabrina Dissociation in the Binge-Purge Cycle of Bulimia Nervosa. JOURNAL OF TRAUMA \& DISSOCIATION. 2008;9(4):463-479.

1758. Estanol Elena, Shepherd Caitlin, MacDonald Tiare Mental Skills as Protective Attributes Against Eating Disorder Risk in Dancers. JOURNAL OF APPLIED SPORT PSYCHOLOGY. 2013;25(2):209-222.

1759. Dalle Grave R School-based prevention programs for eating disorders - Achievements and opportunities. DISEASE MANAGEMENT \& HEALTH OUTCOMES. 2003;11(9):579-593.

1760. Rakusin Dori, O'Brien Kate, Murphy Michael Case reports of new-onset eating disorders in older adult cancer survivors. JOURNAL OF EATING DISORDERS. 2021;9(1):.

1761. Hayes Jacqueline, Fitzsimmons-Craft Ellen, Karam Anna, Jakubiak Jessica, Brown Mackenzie, Wilfley Denise Disordered Eating Attitudes and Behaviors in Youth with Overweight and Obesity: Implications for Treatment. CURRENT OBESITY REPORTS. 2018;7(3):235-246.

1762. Manni R, Ratti MT, Tartara A Nocturnal eating: Prevalence and features in 120 insomniac referrals. SLEEP. 1997;20(9):734-738.

1763. Yashkov Yury, Bekuzarov Dmitry Effectiveness of Biliopancreatic diversion in the patients with bulimia nervosa. OBESITY SURGERY. 2006;16(11):1433-1439.

1764. Galante Marina, Ward Rose, Weinberg Robert Comparing Weight-Conscious Drinking Among Athletes and Nonathletes. JOURNAL OF CLINICAL SPORT PSYCHOLOGY. 2017;11(4):273-286.

1765. Posluszny Hannah, Quick Virginia, Worobey John Disordered eating in college women: associations with the mother-daughter relationship and family weight-related conversations. EATING AND WEIGHT DISORDERS-STUDIES ON ANOREXIA BULIMIA AND OBESITY. 2022;27(1):243-251.

1766. Levitan RD, Kaplan AS, Rockert W Characterization of the ``seasonal'' bulimic patient. INTERNATIONAL JOURNAL OF EATING DISORDERS. 1996;19(2):187-192.

1767. Stunkard AJ, Faith MS, Allison KC Depression and obesity. BIOLOGICAL PSYCHIATRY. 2003;54(3):330-337.

1768. Velazquez Cayley, Pasch Keryn, Ranjit Nalini, Mirchandani Gita, Hoelscher Deanna Are Adolescents' Perceptions of Dietary Practices Associated with Their Dietary Behaviors?. JOURNAL OF THE AMERICAN DIETETIC ASSOCIATION. 2011;111(11):1735-1740.

1769. Pike Kathleen, So Mirai, Hilbert Anja, Maekawa Hiroko, Shimanouchi Tomoko, Wilfley Denise, Dohm Faith-Anne, Fairburn Christopher, Weissman Ruth Risk factors for anorexia nervosa and bulimia nervosa in Japan and compared to a US sample. INTERNATIONAL JOURNAL OF EATING DISORDERS. 2021;54(2, SI):155-167.

1770. Mabe Annalise, Forney K., Keel Pamela Do You ``Like{''} My Photo? Facebook Use Maintains Eating Disorder Risk. INTERNATIONAL JOURNAL OF EATING DISORDERS. 2014;47(5):516-523.

1771. Han Wenyue, Zheng Zheng, Zhang Ning Three Mediating Pathways of Anxiety and Security in the Relationship between Coping Style and Disordered Eating Behaviors among Chinese Female College Students. NEURAL PLASTICITY. 2021;2021():.

1772. Laghi Fiorenzo, Liga Francesca, Baumgartner Emma, Baiocco Roberto Identity and conformism among Italian adolescents who binge eat and drink. HEALTH RISK \& SOCIETY. 2012;14(4):361-376.

1773. Olejniczak Dominik, Bugajec Dorota, Staniszewska Anna, Panczyk Mariusz, Kielan Aleksandra, Czerw Aleksandra, Manczuk Marta, Juszczyk Grzegorz, Skonieczna Joanna, Brytek-Matera Anna Risk assessment of night-eating syndrome occurrence in women in Poland, considering the obesity factor in particular. NEUROPSYCHIATRIC DISEASE AND TREATMENT. 2018;14():1521-1526.

1774. Timko C., Hormes Julia, Chubski Janice Will the real vegetarian please stand up? An investigation of dietary restraint and eating disorder symptoms in vegetarians versus non-vegetarians. APPETITE. 2012;58(3):982-990.

1775. Hayes Samantha, Linardon Jake, Kim Christina, Mitchison Deborah Understanding the relationship between sexual harassment and eating disorder psychopathology: A systematic review and meta-analysis. INTERNATIONAL JOURNAL OF EATING DISORDERS. 2021;54(5):673-689.

1776. Mond Jonathan, Hay Phillipa, Rodgers Bryan, Owen Cathy Comparing the Health Burden of Eating-Disordered Behavior and Overweight in Women. JOURNAL OF WOMENS HEALTH. 2009;18(7):1081-1089.

1777. Vasiliu Octavian Current Status of Evidence for a New Diagnosis: Food Addiction-A Literature Review. FRONTIERS IN PSYCHIATRY. 2022;12():.

1778. Schaumberg Katherine, Robinson Lauren, Hochman Ayelet, Micali Nadia <p>Prospective Associations Between Driven Exercise and Other Eating Disorder Behaviors in Adolescence: A Longitudinal Cohort Study</p>. JOURNAL OF ADOLESCENT HEALTH. 2022;70(5):781-787.

1779. Steiger H, Lehoux PM, Gauvin L Impulsivity, dietary control and the urge to binge in bulimic syndromes. INTERNATIONAL JOURNAL OF EATING DISORDERS. 1999;26(3):261-274.

1780. Warren Janet, Smith Nicola, Ashwell Margaret A structured literature review on the role of mindfulness, mindful eating and intuitive eating in changing eating behaviours: effectiveness and associated potential mechanisms. NUTRITION RESEARCH REVIEWS. 2017;30(2):272-283.

1781. Ando Tetsuya, Komaki Gen, Nishimura Hiroki, Naruo Tetsuro, Okabe Kenjiro, Kawai Keisuke, Takii Masato, Oka Takakazu, Kodama Naoki, Nakamoto Chiemi, Ishikawa Toshio, Suzuki-Hotta Mari, Minatozaki Kazunori, Yamaguchi Chikara, Nishizono-Maher Aya, Kono Masaki, Kajiwara Sohei, Suematsu Hiroyuki, Tomita Yuichiro, Ebana Shoichi, Okamoto Yuri, Nagata Katsutaro, Nakai Yoshikatsu, Koide Masanori, Kobayashi Nobuyuki, Kurokawa Nobuo, Nagata Toshihiko, Kiriike Nobuo, Takenaka Yoshito, Nagamine Kiyohide, Ookuma Kazuyoshi, Murata Shiho, Eating Japanese A ghrelin gene variant may predict crossover rate from restricting-type anorexia nervosa to other phenotypes of eating disorders: a retrospective survival analysis. PSYCHIATRIC GENETICS. 2010;20(4):153-159.

1782. Doelemeyer Ruth, Tietjen Annemarie, Kersting Anette, Wagner Birgit Internet-based interventions for eating disorders in adults: a systematic review. BMC PSYCHIATRY. 2013;13():.

1783. Jankauskiene Rasa, Baceviciene Migle Media Pressures, Internalization of Appearance Ideals and Disordered Eating among Adolescent Girls and Boys: Testing the Moderating Role of Body Appreciation. NUTRIENTS. 2022;14(11):.

1784. Rania Marianna, Filippis Renato, Caroleo Mariarita, Carbone Elvira, Aloi Matteo, Bratman Steven, Segura-Garcia Cristina Pathways to orthorexia nervosa: a case series discussion. EATING AND WEIGHT DISORDERS-STUDIES ON ANOREXIA BULIMIA AND

OBESITY. 2021;26(5):1675-1683.

1785. Bentley Caroline, Gratwick-Sarll Kassandra, Harrison Carmel, Mond Jonathan Sex differences in psychosocial impairment associated with eating disorder features in adolescents: A school-based study. INTERNATIONAL JOURNAL OF EATING DISORDERS. 2015;48(6):633-640.

1786. Eynde Frederique, Sentuerk Vesile, Naudts Kris Gilles de la Tourette syndrome mimicking an eating disorder. TURK PSIKIYATRI DERGISI. 2007;18(4):375-378.

1787. Novelle Marta, Dieguez Carlos Unravelling the role and mechanism of adipokine and gastrointestinal signals in animal models in the nonhomeostatic control of energy homeostasis: Implications for binge eating disorder. EUROPEAN EATING DISORDERS REVIEW. 2018;26(6):551-568.

1788. Leahey Tricia, Crowther Janis, Ciesla Jeffrey An Ecological Momentary Assessment of the Effects of Weight and Shape Social Comparisons on Women With Eating Pathology, High Body Dissatisfaction, and Low Body Dissatisfaction. BEHAVIOR THERAPY. 2011;42(2):197-210.

1789. Frank GK, Bailer UF, Henry S, Wagner A, Kaye WH Neuroimaging studies in eating disorders. CNS SPECTRUMS. 2004;9(7):539-548.

1790. Hartmann Andrea, Cordes Martin, Hirschfeld Gerrit, Vocks Silja Affect and worry during a checking episode: A comparison of individuals with symptoms of obsessive-compulsive disorder, anorexia nervosa, bulimia nervosa, body dysmorphic disorder, illness anxiety disorder, and panic disorder. PSYCHIATRY RESEARCH. 2019;272():349-358.

1791. Mayne Stephanie, Virudachalam Senbagam, Fiks Alexander Clustering of unhealthy behaviors in a nationally representative sample of US children and adolescents. PREVENTIVE MEDICINE. 2020;130():.

1792. Lenz Dunker Karin, Claudino Angelica Validity and Reliability of the Brazilian Version of the Weight Control Behaviors Scale. JOURNAL OF PEDIATRICS. 2017;189():143+.

1793. VanKim Nicole, Erickson Darin, Eisenberg Marla, Lust Katherine, Rosser B., Laska Melissa Differences in Weight-Related Behavioral Profiles by Sexual Orientation Among

College Men: A Latent Class Analysis. AMERICAN JOURNAL OF HEALTH PROMOTION. 2016;30(8):623-633.

1794. Reynolds Kalli, Plateau Carolyn, Haycraft Emma Sociocultural Influences on Compulsive Exercise in Young People: A Systematic Review. ADOLESCENT RESEARCH REVIEW. 2023;8(2):179-194.

1795. Rocks Tetyana, Pelly Fiona, Slater Gary, Martin Lisa Eating attitudes and behaviours of students enrolled in undergraduate nutrition and dietetics degrees. NUTRITION \& DIETETICS. 2017;74(4):381-387.

1796. Jackson Todd, Chen Hong Predicting changes in eating disorder symptoms among Chinese adolescents: A 9-month prospective study. JOURNAL OF PSYCHOSOMATIC RESEARCH. 2008;64(1):87-95.

1797. Lang T, Hauser R, Schlumpf R, Klaghofer R, Buddeberg C Psychic comorbidity and quality of life in patients with morbid obesity applying for gastric banding. SCHWEIZERISCHE MEDIZINISCHE WOCHENSCHRIFT. 2000;130(20):739-748.

1798. Rosenberger Patricia, Henderson Kathryn, Bell Robert, Grilo Carlos Associations of weight-based teasing history and current eating disorder features and psychological functioning in bariatric surgery patients. OBESITY SURGERY. 2007;17(4):470-477.

1799. Cachelin FM, Rebeck R, Veisel C, Striegel-Moore RH Barriers to treatment for eating disorders among ethnically diverse women. INTERNATIONAL JOURNAL OF EATING DISORDERS. 2001;30(3):269-278.

1800. Miccoli Laura, Martinez-Fiestas Myriam, Delgado-Rodriguez Rafael, Diaz-Ferrer Sandra, Rodriguez-Ruiz Sonia, Carmen Fernandez-Santaella M. Adolescent emotions toward sweet food cues as a function of obesity and risky dieting practices. FOOD QUALITY AND PREFERENCE. 2018;68():205-214.

1801. Wilhelm Leonie, Hartmann Andrea, Becker Julia, Kisi Melahat, Waldorf Manuel, Vocks Silja Body Covering and Body Image: A Comparison of Veiled and Unveiled Muslim Women, Christian Women, and Atheist Women Regarding Body Checking, Body Dissatisfaction, and Eating Disorder Symptoms. JOURNAL OF RELIGION \& HEALTH. 2018;57(5):1808-1828.

1802. Mathis Maria, Rosario Maria, Diniz Juliana, Torres Albina, Shavitt Roseli, Ferrao Ygor, Fossaluza Victor, Braganca Pereira Carlos, Miguel Euripedes Obsessive-compulsive disorder: Influence of age at onset on comorbidity patterns. EUROPEAN PSYCHIATRY. 2008;23(3):187-194.

1803. Djamshidian Atbin, Averbeck Bruno, Lees Andrew, O'Sullivan Sean Clinical aspects of impulsive compulsive behaviours in Parkinson's disease. JOURNAL OF THE NEUROLOGICAL SCIENCES. 2011;310(1-2, SI):183-188.

1804. Ripoli Carlo, Ricciardi Maria, Zuncheddu Ester, Angelo Maria, Pinna Anna, Ripoli Daniela Emotional eating and disordered eating behaviors in children and adolescents with type 1 diabetes. SCIENTIFIC REPORTS. 2022;12(1):.

1805. Isaksson Martina, Ghaderi Ata, Wolf-Arehult Martina, Ramklint Mia Overcontrolled, undercontrolled, and resilient personality styles among patients with eating disorders. JOURNAL OF EATING DISORDERS. 2021;9(1):.

1806. Li Shaojie, Cui Guanghui, Yin Yongtian, Tang Kaixuan, Chen Lei, Liu Xinyao Prospective Association Between Problematic Mobile Phone Use and Eating Disorder Symptoms and the Mediating Effect of Resilience in Chinese College Students: A 1-Year Longitudinal Study. FRONTIERS IN PUBLIC HEALTH. 2022;10():.

1807. Bresin Konrad Toward a unifying theory of dysregulated behaviors. CLINICAL PSYCHOLOGY REVIEW. 2020;80():.

1808. Scardera Sara, Sacco Sabrina, Di Sante Jessica, Booij Linda Body image-related cognitive fusion and disordered eating: the role of self-compassion and sad mood. EATING AND WEIGHT DISORDERS-STUDIES ON ANOREXIA BULIMIA AND OBESITY. 2021;26(2):483-490.

1809. Stein Dan, Aguilar-Gaxiola Sergio, Alonso Jordi, Bruffaerts Ronny, Jonge Peter, Liu Zharoui, Caldas-de-Almeida Jose, O'Neill Siobhan, Viana Maria, Al-Hamzawi Ali, Angermeyer Mattias, Benjet Corina, Graaf Ron, Ferry Finola, Kovess-Masfety Viviane, Levinson Daphna, Girolamo Giovanni, Florescu Silvia, Hu Chiyi, Kawakami Norito, Haro Josep, Piazza Marina, Posada-Villa Jose, Wojtyniak Bogdan, Xavier Miguel, Lim Carmen, Kessler Ronald, Scott Kate Associations between mental disorders and subsequent onset of hypertension. GENERAL HOSPITAL PSYCHIATRY. 2014;36(2):142-149.

1810. Harden K., Kretsch Natalie, Moore Sarah, Mendle Jane Descriptive Review: Hormonal Influences on Risk for Eating Disorder Symptoms During Puberty and Adolescence. INTERNATIONAL JOURNAL OF EATING DISORDERS. 2014;47(7, SI):718-726.

1811. Ghaderi Ata, Stice Eric, Andersson Gerhard, Persson Johanna, Allzen Elin A Randomized Controlled Trial of the Effectiveness of Virtually Delivered Body Project (vBP) Groups to Prevent Eating Disorders. JOURNAL OF CONSULTING AND CLINICAL PSYCHOLOGY. 2020;88(7):643-656.

1812. Zini A., Siani R., Sandri M., Soardo F., Siciliani O. Partial syndromes in eating disorders: A prevalence study on a sample of Italian adolescents. EATING AND WEIGHT DISORDERS-STUDIES ON ANOREXIA BULIMIA AND OBESITY. 2007;12(3):125-131.

1813. Brown Tiffany, Keel Pamela The impact of relationships on the association between sexual orientation and disordered eating in men. INTERNATIONAL JOURNAL OF EATING DISORDERS. 2012;45(6):792-799.

1814. Chang Jun-Jie, Xu Nuo, Song Ling-Ling, Li Yong-Han, Yuan Meng-Yuan, Zhang Ting-Ting, He Yang, Chen Shan-Shan, Wang Geng-Fu, Su Pu-Yu Association between the dietary literacy of children's daily diet providers and school-age children's nutritional status and eating behaviours: a cross-sectional study. BMC PUBLIC HEALTH. 2022;22(1):.

1815. Klatzkin Rebecca, Gaffney Sierra, Cyrus Kathryn, Bigus Elizabeth, Brownley Kimberly Binge eating disorder and obesity: Preliminary evidence for distinct cardiovascular and psychological phenotypes. PHYSIOLOGY \& BEHAVIOR. 2015;142():20-27.

1816. Martinez-Lacoba Roberto, Pardo-Garcia Isabel, Amo-Saus Elisa, Escribano-Sotos Francisco Socioeconomic, demographic and lifestyle-related factors associated with unhealthy diet: a cross-sectional study of university students. BMC PUBLIC HEALTH. 2018;18():.

1817. Elliot DL, Goldberg L, Moe EL, DeFrancesco CA, Durham MB, Hix-Small H Preventing substance use and disordered eating - Initial outcomes of the ATHENA (Athletes Targeting Healthy Exercise and Nutrition Alternatives) Program. ARCHIVES OF PEDIATRICS \& ADOLESCENT MEDICINE. 2004;158(11):1043-1049.

1818. Nilsson EW, Gillberg C, Gillberg IC, Rastam M Ten-year follow-up of adolescent-onset anorexia nervosa: personality disorders. JOURNAL OF THE AMERICAN ACADEMY OF CHILD

AND ADOLESCENT PSYCHIATRY. 1999;38(11):1389-1395.

1819. Hinney A, Remschmidt H, Hebebrand J Candidate gene polymorphisms in eating disorders. EUROPEAN JOURNAL OF PHARMACOLOGY. 2000;410(2-3):147-159.

1820. Jendrzyca Anna, Warschburger Petra Weight stigma and eating behaviours in elementary school children: A prospective population-based study. APPETITE. 2016;102():51-59.

1821. Haynos Ann, Watts Allison, Loth Katie, Pearson Carolyn, Neumark-Stzainer Dianne Factors Predicting an Escalation of Restrictive Eating During Adolescence. JOURNAL OF ADOLESCENT HEALTH. 2016;59(4):391-396.

1822. Albertz Jennifer, Boersma Gretha, Tamashiro Kellie, Moran Timothy The effects of scheduled running wheel access on binge-like eating behavior and its consequences. APPETITE. 2018;126():176-184.

1823. Shapiro Jennifer, Berkman Nancy, Brownley Kimberly, Sedway Jan, Lohr Kathleen, Bulik Cynthia Bulimia nervosa treatment: A systematic review of randomized controlled trials. INTERNATIONAL JOURNAL OF EATING DISORDERS. 2007;40(4):321-336.

1824. Tenconi Elena, Lunardi Noemi, Zanetti Tatiana, Santonastaso Paolo, Favaro Angela Predictors of binge eating in restrictive anorexia nervosa patients in Italy. JOURNAL OF NERVOUS AND MENTAL DISEASE. 2006;194(9):712-715.

1825. Duncan Alexis, Ziobrowski Hannah, Nicol Ginger The Prevalence of Past 12-Month and Lifetime DSM-IV Eating Disorders by BMI Category in US Men and Women. EUROPEAN EATING DISORDERS REVIEW. 2017;25(3):165-171.

1826. Riquin Elise, Raynal Agathe, Mattar Lama, Lalanne Christophe, Hirot France, Huas Caroline, Duclos Jeanne, Berthoz Sylvie, Godart Nathalie, Grp EVHAN Is the Severity of the Clinical Expression of Anorexia Nervosa Influenced by an Anxiety, Depressive, or Obsessive-Compulsive Comorbidity Over a Lifetime?. FRONTIERS IN PSYCHIATRY. 2021;12():.

1827. DAWKINS K GENDER DIFFERENCES IN PSYCHIATRY - EPIDEMIOLOGY AND DRUG RESPONSE. CNS DRUGS. 1995;3(5):393-407.

1828. Perelman Hayley, Reel Justine Using a culturally humble approach to examine and address disordered eating in sport. JOURNAL OF APPLIED SPORT PSYCHOLOGY. 2022;():.

1829. Ferrand Claude, Magnan Claire, Rouveix Mathieu, Filaire Edith Disordered eating, perfectionism and body-esteem of elite synchronized swimmers. EUROPEAN JOURNAL OF SPORT SCIENCE. 2007;7(4):223-230.

1830. Gokalp Cengiz, Yildiz Suleyman, Baltali Oya Eating Behaviors in Early Childhood (1-5 Years) and Their Association With Sociodemographic Characteristics in Turkey. CUREUS JOURNAL OF MEDICAL SCIENCE. 2021;13(8):.

1831. Kong Seongsook, Bernstein Kunsook Childhood trauma as a predictor of eating psychopathology and its mediating variables in patients with eating disorders. JOURNAL OF CLINICAL NURSING. 2009;18(13):1897-1907.

1832. Inoue Yuichi Sleep-related eating disorder and its associated conditions. PSYCHIATRY AND CLINICAL NEUROSCIENCES. 2015;69(6):309-320.

1833. Linville Deanna, Stice Eric, Gau Jeff, O'Neil Maya Predictive Effects of Mother and Peer Influences on Increases in Adolescent Eating Disorder Risk Factors and Symptoms: A 3-Year Longitudinal Study. INTERNATIONAL JOURNAL OF EATING DISORDERS. 2011;44(8):745-751.

1834. Treasure J., Kan C., Stephenson L., Warren E., Smith E., Heller S., Ismail K. Developing a theoretical maintenance model for disordered eating in Type 1 diabetes. DIABETIC MEDICINE. 2015;32(12):1541-1545.

1835. Wang Peiyi, Garcia Esmeralda, Chen Chuansheng, Yim Ilona Does perceived support moderate the link between acculturative stress and problematic eating behaviors? The role of family, significant other, and friend support. INTERNATIONAL JOURNAL OF EATING DISORDERS. 2023;56(6):1075-1086.

1836. Janas-Kozik Malgorzata, Zmijowska Anna, Zasada Ida, Jelonek Ireneusz, Cichon Lena, Siwiec Andrzej, Wilczynski Krzysztof Systematic Review of Literature on Eating Disorders During Pregnancy-Risk and Consequences for Mother and Child. FRONTIERS IN PSYCHIATRY. 2021;12():.

1837. Dakanalis Antonios, Clerici Massimo, Riva Giuseppe, Carra Giuseppe Testing the DSM-5 severity indicator for bulimia nervosa in a treatment-seeking sample. EATING AND WEIGHT DISORDERS-STUDIES ON ANOREXIA BULIMIA AND OBESITY. 2017;22(1):161-167.

1838. Butler Rachel, Williams Brenna, Levinson Cheri An examination of eating disorder fears in imaginal exposure scripts. JOURNAL OF AFFECTIVE DISORDERS. 2023;326():163-167.

1839. Dabone Charles, Delisle Helene, Receveur Olivier Predisposing, facilitating and reinforcing factors of healthy and unhealthy food consumption in schoolchildren: a study in Ouagadougou, Burkina Faso. GLOBAL HEALTH PROMOTION. 2013;20(1):68-77.

1840. Rauof Maryam, Ebrahimi Hossein, Jafarabadi Mohammad, Malek Ayyoub, Kheiroddin Jalil Prevalence of Eating Disorders Among Adolescents in the Northwest of Iran. IRANIAN RED CRESCENT MEDICAL JOURNAL. 2015;17(10):.

1841. Hudson JI, Lalonde JK, Berry JM, Pindyck LJ, Bulik CM, Crow SJ, McElroy SL, Laird NM, Tsuang MT, Walsh BT, Rosenthal NR, Pope HG Binge-eating disorder as a distinct familial phenotype in obese individuals. ARCHIVES OF GENERAL PSYCHIATRY. 2006;63(3):313-319.

1842. Fairweather-Schmidt A., Wade Tracey DSM-5 Eating Disorders and Other Specified Eating and Feeding Disorders: Is There a Meaningful Differentiation?. INTERNATIONAL JOURNAL OF EATING DISORDERS. 2014;47(5):524-533.

1843. McLean Sian, Paxton Susan, Wertheim Eleanor The role of media literacy in body dissatisfaction and disordered eating: A systematic review. BODY IMAGE. 2016;19():9-23.

1844. Peebles Rebecka, Wilson Jenny, Lock James How do children with eating disorders differ from adolescents with eating disorders at initial evaluation?. JOURNAL OF ADOLESCENT HEALTH. 2006;39(6):800-805.

1845. Romero-Mesa Juana, Pelaez-Fernandez Maria, Extremera Natalio Emotional intelligence and eating disorders: a systematic review. EATING AND WEIGHT DISORDERS-STUDIES ON ANOREXIA BULIMIA AND OBESITY. 2021;26(5):1287-1301.

1846. Pursey Kirrilly, Burrows Tracy, Barker Daniel, Hart Melissa, Paxton Susan Disordered eating, body image concerns, and weight control behaviors in primary school aged children: A systematic review and meta-analysis of universal-selective prevention interventions. INTERNATIONAL JOURNAL OF EATING DISORDERS. 2021;54(10):1730-1765.

1847. Kesby Alice, Maguire Sarah, Brownlow Rachel, Grisham Jessica Intolerance of Uncertainty in eating disorders: An update on the field. CLINICAL PSYCHOLOGY REVIEW. 2017;56():94-105.

1848. Uotani Nao, Noma Shun'ichi, Akamine Momoko, Miyawaki Takashi Continuous glucose monitoring for detection of glycemic variability, hypoglycemia, and hyperglycemia in women with eating disorders. BIOPSYCHOSOCIAL MEDICINE. 2022;16(1):.

1849. Sen Demirdogen Ezgi, Algedik Pinar, Kadak Muhammed, Alikasifoglu Mujgan, Okbay Gunes Asli, Demir Turkay The association between separation individuation process and binge eating disorder in adolescents aged 12-18. EATING AND WEIGHT DISORDERS-STUDIES ON ANOREXIA BULIMIA AND OBESITY. 2021;26(4):1089-1096.

1850. Solomon-Moore Emma, Lambert Jeffrey, Grey Elisabeth, Gillison Fiona, Townsend Nick, Busam Betty, Velemis Kyriakos, Millen Christopher, Baber Fran, Griffin Tania Life in lockdown: a longitudinal study investigating the impact of the UK COVID-19 lockdown measures on lifestyle behaviours and mental health. BMC PUBLIC HEALTH. 2022;22(1):.

1851. Cook-Cottone Catherine Incorporating positive body image into the treatment of eating disorders: A model for attunement and mindful self-care. BODY IMAGE. 2015;14():158-167.

1852. Petisco-Rodriguez Cristina, Sanchez-Sanchez Laura, Fernandez-Garcia Ruben, Sanchez-Sanchez Javier, Manuel Garcia-Montes Jose Disordered Eating Attitudes, Anxiety, Self-Esteem and Perfectionism in Young Athletes and Non-Athletes. INTERNATIONAL JOURNAL OF ENVIRONMENTAL RESEARCH AND PUBLIC HEALTH. 2020;17(18):.

1853. Monteleone Palmiero, Tortorella Alfonso, Scognamiglio Pasquale, Serino Ismene, Monteleone Alessio, Maj Mario The Acute Salivary Ghrelin Response to a Psychosocial Stress Is Enhanced in Symptomatic Patients with Bulimia Nervosa: A Pilot Study. NEUROPSYCHOBIOLOGY. 2012;66(4):230-236.

1854. Lavender Jason, Jardin Bianca, Anderson Drew Bulimic symptoms in undergraduate men and women: Contributions of mindfulness and thought suppression. EATING BEHAVIORS. 2009;10(4):228-231.

1855. Murray Matthew, Perelman Hayley, Sandhu Danielle, Quinones Isabel, Haedt-Matt Alissa Overweight preoccupation is associated with eating pathology in male collegiate athletes with body dissatisfaction. EATING AND WEIGHT DISORDERS-STUDIES ON ANOREXIA BULIMIA AND OBESITY. 2022;27(7):2387-2395.

1856. Barnett Michaela, Dripps Weston, Blomquist Kerstin Organivore or organorexic? Examining the relationship between alternative food network engagement, disordered eating, and special diets. APPETITE. 2016;105():713-720.

1857. Puccio Francis, Fuller-Tyszkiewicz Matthew, Ong Deborah, Krug Isabel A Systematic Review and Meta-Analysis on the Longitudinal Relationship Between Eating Pathology and Depression. INTERNATIONAL JOURNAL OF EATING DISORDERS. 2016;49(5):439-454.

1858. Stein Karen, Corte Colleen The identity impairment model - A longitudinal study of self-schemas as predictors of disordered eating behaviors. NURSING RESEARCH. 2008;57(3):182-190.

1859. Domingues Rita, Carmo Claudia Orthorexia nervosa in yoga practitioners: relationship with personality, attitudes about appearance, and yoga engagement. EATING AND WEIGHT DISORDERS-STUDIES ON ANOREXIA BULIMIA AND OBESITY. 2021;26(3):789-795.

1860. Juli Maria, Juli Giada, Juli Rebecca AN EPIDEPIC IN THE PANDEMIC: THE MOST DIFFICULT CHALLENGE FOR YOUNG ADOLESCENTS. PSYCHIATRIA DANUBINA. 2021;33(9):S142-S147.

1861. Cooper PJ, Watkins B, Bryant-Waugh R, Lask B The nosological status of early onset anorexia nervosa. PSYCHOLOGICAL MEDICINE. 2002;32(5):873-880.

1862. Ben-Porath Denise, Duthu Florencia, Luo Tana, Gonidakis Fragiskos, Compte Emilio, Wisniewski Lucene Dialectical behavioral therapy: an update and review of the existing treatment models adapted for adults with eating disorders. EATING DISORDERS. 2020;28(2, SI):101-121.

1863. Munt A., Partridge S., Allman-Farinelli M. The barriers and enablers of healthy eating among young adults: a missing piece of the obesity puzzle: A scoping review. OBESITY REVIEWS. 2017;18(1):1-17.

1864. Shope Megan, Freeman Andrew, Culbert Kristen Elucidating early pubertal timing effects on disordered eating symptoms in young adult women. EATING BEHAVIORS. 2022;45():.

1865. Mond Jonathan, Myers Tricia, Crosby Ross, Hay Phillipa, Mitche James `Excessive exercise' and eating-disordered behaviour in young adult women: Further evidence from a primary care sample. EUROPEAN EATING DISORDERS REVIEW. 2008;16(3):215-221.

1866. Bankoff Sarah, Richards Lauren, Bartlett Brooke, Wolf Erika, Mitchell Karen Examining weight and eating behavior by sexual orientation in a sample of male veterans. COMPREHENSIVE PSYCHIATRY. 2016;68():134-139.

1867. Rothschild-Yakar Lily, Levy-Shiff Rachel, Fridman-Balaban Rachel, Gur Eitan, Stein Daniel Mentalization and Relationships With Parents as Predictors of Eating Disordered Behavior. JOURNAL OF NERVOUS AND MENTAL DISEASE. 2010;198(7):501-507.

1868. Reyes-Rodriguez Mae, Von Holle Ann, Ulman Teresa, Thornton Laura, Klump Kelly, Brandt Harry, Crawford Steve, Fichter Manfred, Halmi Katherine, Huber Thomas, Johnson Craig, Jones Ian, Kaplan Allan, Mitchell James, Strober Michael, Treasure Janet, Woodside D., Berrettini Wade, Kaye Walter, Bulik Cynthia Posttraumatic Stress Disorder in Anorexia Nervosa. PSYCHOSOMATIC MEDICINE. 2011;73(6):491-497.

1869. Corriere C., Michel G., Feart C., Pellay H., Onorato O., Barat P., Thibault H. Relationships between emotional disorders, personality dimensions, and binge eating disorder in French obese adolescents. ARCHIVES DE PEDIATRIE. 2019;26(3):138-144.

1870. Franko Debra, George Jessica A Pilot Intervention to Reduce Eating Disorder Risk in Latina Women. EUROPEAN EATING DISORDERS REVIEW. 2008;16(6):436-441.

1871. Robinson Katherine, Wade Tracey Perfectionism interventions targeting disordered eating: A systematic review and meta-analysis. INTERNATIONAL JOURNAL OF EATING DISORDERS. 2021;54(4):473-487.

1872. Broadley Melanie, White Melanie, Andrew Brooke Executive function is associated with diabetes-specific disordered eating in young adults with type 1 diabetes. JOURNAL OF PSYCHOSOMATIC RESEARCH. 2018;111():1-12.

1873. Udo Tomoko, McKee Sherry, White Marney, Masheb Robin, Barnes Rachel, Grilo Carlos Sex differences in biopsychosocial correlates of binge eating disorder: a study of treatment-seeking obese adults in primary care setting. GENERAL HOSPITAL PSYCHIATRY. 2013;35(6):587-591.

1874. Puls Hans-Christian, Schmidt Ricarda, Herpertz Stephan, Zipfel Stephan, Tuschen-Caffier Brunna, Friederich Hans-Christoph, Gerlach Frauke, Mayr Andreas, Lam Tony, Schade-Brittinger Carmen, Zwaan Martina, Hilbert Anja Adherence as a predictor of dropout in Internet-based guided self-help for adults with binge-eating disorder and overweight or obesity. INTERNATIONAL JOURNAL OF EATING DISORDERS. 2020;53(4):555-563.

1875. Christian Caroline, Martel Michelle, Levinson Cheri Emotion regulation difficulties, but not negative urgency, are associated with attention-deficit/hyperactivity disorder and eating disorder symptoms in undergraduate students. EATING BEHAVIORS. 2020;36():.

1876. Messer Mariel, Anderson Cleo, Linardon Jake Self-compassion explains substantially more variance in eating disorder psychopathology and associated impairment than mindfulness. BODY IMAGE. 2021;36():27-33.

1877. Bartlett SJ, Wadden TA, Vogt RA Psychosocial consequences of weight cycling. JOURNAL OF CONSULTING AND CLINICAL PSYCHOLOGY. 1996;64(3):587-592.

1878. Suarez-Ortiz Josue, Cortes-Salazar Felipe, Malagon-Carrillo Ariadna, Lopez-Alonso Veronica, Mancilla-Diaz Juan, Tejas-Juarez Juan, Escartin-Perez Rodrigo Intra-accumbens Raclopride Administration Prevents Behavioral Changes Induced by Intermittent Access to Sucrose Solution. FRONTIERS IN NEUROSCIENCE. 2018;12():.

1879. Bae Eun, Yoon Ju Unhealthy weight control behaviors and related factors by gender and weight status: Results from a nationally representative sample of Korean adolescents. ARCHIVES OF PSYCHIATRIC NURSING. 2023;42():75-83.

1880. Lydecker Janet, Park Jiwoo, Grilo Carlos Parents Can Experience Impairment Because of Their Children's Weight and Problematic Eating Behaviors. JOURNAL OF ADOLESCENT

HEALTH. 2020;66(2):189-194.

1881. Gonzalez Marcela, Penelo Eva, Espinoza Paola, Francisco Rita, Mora Marisol, Gutierrez Teresa, Raich Rosa BODY DISSATISFACTION AND DISORDERED EATING ATTITUDES AMONG ADOLESCENTS FROM PORTUGAL AND SPAIN. BEHAVIORAL PSYCHOLOGY-PSICOLOGIA CONDUCTUAL. 2018;26(2):323-335.

1882. Murray Cecily, Kidd Martin, Moxley Karis, Jordaan Gerhard Self-Induced Vomiting and Other Impulsive Behaviors in Alcohol Use Disorder: A Cross-sectional Descriptive Study. JOURNAL OF DUAL DIAGNOSIS. 2020;16(4):402-408.

1883. Bayes Adam, Madden Sloane Early onset eating disorders in male adolescents: a series of 10 inpatients. AUSTRALASIAN PSYCHIATRY. 2011;19(6):526-530.

1884. Khubchandani Jagdish, Kandiah Jayanthi, Saiki Diana The COVID-19 Pandemic, Stress, and Eating Practices in the United States. EUROPEAN JOURNAL OF INVESTIGATION IN HEALTH PSYCHOLOGY AND EDUCATION. 2020;10(4):.

1885. Zullig Keith, Matthews-Ewald Molly, Valois Robert Relationship between disordered eating and self-identified sexual minority youth in a sample of public high school adolescents. EATING AND WEIGHT DISORDERS-STUDIES ON ANOREXIA BULIMIA AND OBESITY. 2019;24(3):565-573.

1886. Steinhausen HC, Seidel R, Metzke CW Evaluation of treatment and intermediate and long-term outcome of adolescent eating disorders. PSYCHOLOGICAL MEDICINE. 2000;30(5):1089-1098.

1887. Kinnaird Emma, Norton Caroline, Tchanturia Kate Clinicians' views on treatment adaptations for men with eating disorders: a qualitative study. BMJ OPEN. 2018;8(8):.

1888. Keski-Rahkonen Anna Epidemiology of binge eating disorder: prevalence, course, comorbidity, and risk factors. CURRENT OPINION IN PSYCHIATRY. 2021;34(6):525-531.

1889. Juarascio Adrienne, Lantz Elin, Muratore Alexandra, Lowe Michael Addressing Weight Suppression to Improve Treatment Outcome for Bulimia Nervosa. COGNITIVE AND BEHAVIORAL PRACTICE. 2018;25(3):391-401.

1890. Gonzalez Marcela, Mora Marisol, Penelo Eva, Goddard Elizabeth, Treasure Janet, Raich Rosa Gender differences found in a qualitative study of a disordered eating prevention programme: What do boys have to say?. JOURNAL OF HEALTH PSYCHOLOGY. 2015;20(6, SI):858-874.

1891. Jacobi Corinna, Vollert Bianka, Huetter Kristian, Bloh Paula, Eiterich Nadine, Goerlich Dennis, Taylor C. Indicated Web-Based Prevention for Women With Anorexia Nervosa Symptoms: Randomized Controlled Efficacy Trial. JOURNAL OF MEDICAL INTERNET RESEARCH. 2022;24(6):.

1892. Springer EA, Winzelberg AJ, Perkins R, Taylor CB Effects of a body image curriculum for college students on improved body image. INTERNATIONAL JOURNAL OF EATING DISORDERS. 1999;26(1):13-20.

1893. Reas Deborah, Stedal Kristin Eating disorders in men aged midlife and beyond. MATURITAS. 2015;81(2):248-255.

1894. Butler Rachel, Heimberg Richard Imaginal Exposure for Disordered Eating Related Fears: An Initial Randomized Controlled Trial. BEHAVIOR MODIFICATION. 2023;47(1):46-70.

1895. Evans Elizabeth, Adamson Ashley, Basterfield Laura, Le Couteur Ann, Reilly Jessica, Reilly John, Parkinson Kathryn Risk factors for eating disorder symptoms at 12 years of age: A 6-year longitudinal cohort study. APPETITE. 2017;108():12-20.

1896. Huemer Julia, Haidvogl Maria, Mattejat Fritz, Wagner Gudrun, Nobis Gerald, Fernandez-Aranda Fernando, Collier David, Treasure Janet, Karwautz Andreas Perception of Autonomy and Connectedness Prior to the Onset of Anorexia Nervosa and Bulimia Nervosa A Retrospective Study in Sister Pairs Discordant for an Eating Disorder. ZEITSCHRIFT FUR KINDER-UND JUGENDPSYCHIATRIE UND PSYCHOTHERAPIE. 2012;40(1):61-68.

1897. Lidaka Lasma, Lazdane Gunta, Kivite-Urtane Anda, Gailite Linda, Dzivite-Krisane Iveta, Stokenberga Ieva Health-related quality of life and binge eating among adolescent girls with PCOS. CLINICAL AND EXPERIMENTAL OBSTETRICS \& GYNECOLOGY. 2022;49(3):.

1898. Korczak Daphne, Perruzza Stephanie, Chandrapalan Mathura, Cost Katherine, Cleverley Kristin, Birken Catherine, McCrindle Brian The association of diet and depression:

an analysis of dietary measures in depressed, non-depressed, and healthy youth. NUTRITIONAL NEUROSCIENCE. 2022;25(9):1948-1955.

1899. Haines Jess, Kleinman Ken, Rifas-Shiman Sheryl, Field Alison, Austin S. Examination of Shared Risk and Protective Factors for Overweight and Disordered Eating Among Adolescents. ARCHIVES OF PEDIATRICS \& ADOLESCENT MEDICINE. 2010;164(4):336-343.

1900. Jonge Peter, Alonso Jordi, Stein Dan, Kiejna Andrzej, Aguilar-Gaxiola Sergio, Viana Maria, Liu Zhaorui, O'Neill Siobhan, Bruffaerts Ronny, Caldas-de-Almeida Jose, Lepine Jean-Pierre, Matschinger Herbert, Levinson Daphna, Girolamo Giovanni, Fukao Akira, Bunting Brendan, Maria Haro Josep, Posada-Villa Jose, Al-Hamzawi Ali, Elena Medina-Mora Maria, Piazza Marina, Hu Chiyi, Sasu Carmen, Lim Carmen, Kessler Ronald, Scott Kate Associations between DSM-IV mental disorders and diabetes mellitus: a role for impulse control disorders and depression. DIABETOLOGIA. 2014;57(4):699-709.

1901. Goldschmidt Andrea, Lavender Jason, Hipwell Alison, Stepp Stephanie, Keenan Kate Emotion Regulation and Loss of Control Eating in Community-Based Adolescents. JOURNAL OF ABNORMAL CHILD PSYCHOLOGY. 2017;45(1):183-191.

1902. Bulik Cynthia, Bertoia Monica, Lu Mei, Seeger John, Spalding William Suicidality risk among adults with binge-eating disorder. SUICIDE AND LIFE-THREATENING BEHAVIOR. 2021;51(5):897-906.

1903. Britz B, Siegfried W, Ziegler A, Lamertz C, Herpertz-Dahlmann BM, Remschmidt H, Wittchen HU, Hebebrand J Rates of psychiatric disorders in a clinical study group of adolescents with extreme obesity and in obese adolescents ascertained via a population based study. INTERNATIONAL JOURNAL OF OBESITY. 2000;24(12):1707-1714.

1904. De Moor RJG Eating disorder-induced dental complications: a case report. JOURNAL OF ORAL REHABILITATION. 2004;31(7):725-732.

1905. Berro Jana, Akel Marwan, Hallit Souheil, Obeid Sahar Relationships between inappropriate eating habits and problematic alcohol use, cigarette and waterpipe dependence among male adolescents in Lebanon. BMC PUBLIC HEALTH. 2021;21(1):.

1906. Kothari Radha, Barona Manuela, Treasure Janet, Micali Nadia Social cognition in children at familial high-risk of developing an eating disorder. FRONTIERS IN BEHAVIORAL

NEUROSCIENCE. 2015;9():.

1907. Kelly Nichole, Shomaker Lauren, Radin Rachel, Thompson Katherine, Cassidy Omni, Brady Sheila, Mehari Rim, Courville Amber, Chen Kong, Galescu Ovidiu, Tanofsky-Kraff Marian, Yanovski Jack Associations of sleep duration and quality with disinhibited eating behaviors in adolescent girls at-risk for type 2 diabetes. EATING BEHAVIORS. 2016;22():149-155.

1908. Sanderson CA, Holloway RM Who benefits from what? Drive for thinness as a moderator of responsiveness to different eating disorder prevention messages. JOURNAL OF APPLIED SOCIAL PSYCHOLOGY. 2003;33(9):1837-1861.

1909. Kluck Annette, Carriere Lucille, Dallesasse Starla, Bvunzawabaya Batsirai, English Erin, Cobb Megan, Borges Therese, Zhuzha Kseniya, Fry Daniel Pathways of family influence: Alcohol use and disordered eating in daughters. ADDICTIVE BEHAVIORS. 2014;39(10):1404-1407.

1910. Oldham-Cooper Rosie, Semple Claire Prevention and early help for eating disorders in young people with type 1 diabetes. CLINICAL CHILD PSYCHOLOGY AND PSYCHIATRY. 2021;26(3):656-668.

1911. Teasdale Scott, Ward Philip, Samaras Katherine, Firth Joseph, Stubbs Brendon, Tripodi Elise, Burrows Tracy Dietary intake of people with severe mental illness: systematic review and meta-analysis. BRITISH JOURNAL OF PSYCHIATRY. 2019;214(5):251-259.

1912. Smith Laura, Foster Nicole, Bollepalli Sureka, Fitterman-Harris Hannah, Rancourt Diana An Examination of Sex Differences in a Disease-Specific Model of Disordered Eating Behaviors in Type 1 Diabetes. JOURNAL OF PEDIATRIC PSYCHOLOGY. 2020;45(1):91-100.

1913. Eichen Dawn, Conner Bradley, Daly Brian, Fauber Robert Weight Perception, Substance Use, and Disordered Eating Behaviors: Comparing Normal Weight and Overweight High-School Students. JOURNAL OF YOUTH AND ADOLESCENCE. 2012;41(1, SI):1-13.

1914. Sick Kelsey, Sabiston Catherine, Maharaj Aryel, Pila Eva Body image and disordered eating prevention in girls' sport: A partner-driven and stakeholder-informed scoping review of interventions. PSYCHOLOGY OF SPORT AND EXERCISE. 2022;61():.

1915. Garcia-Burgos David, Maglieri Sabine, Vogele Claus, Munsch Simone How Does Food Taste in Anorexia and Bulimia Nervosa? A Protocol for a Quasi-Experimental, Cross-Sectional Design to Investigate Taste Aversion or Increased Hedonic Valence of Food in Eating Disorders. FRONTIERS IN PSYCHOLOGY. 2018;9():.

1916. Eeden Annelies, Hoeken Daphne, Hoek Hans Incidence, prevalence and mortality of anorexia nervosa and bulimia nervosa. CURRENT OPINION IN PSYCHIATRY. 2021;34(6):515-524.

1917. Schaumberg Katherine, Reilly Erin, Gorrell Sasha, Levinson Cheri, Farrell Nicholas, Brown Tiffany, Smith Kathryn, Schaefer Lauren, Essayli Jamal, Haynos Ann, Anderson Lisa Conceptualizing eating disorder psychopathology using an anxiety disorders framework: Evidence and implications for exposure-based clinical research. CLINICAL PSYCHOLOGY REVIEW. 2021;83():.

1918. Hay Phillipa, Aouad Phillip, Le Anvi, Marks Peta, Maloney Danielle, Touyz Stephen, Maguire Sarah, Consortium Natl Epidemiology of eating disorders: population, prevalence, disease burden and quality of life informing public policy in Australia-a rapid review. JOURNAL OF EATING DISORDERS. 2023;11(1):.

1919. Hildebrandt Tom, Bacow Terri, Markella Mariana, Loeb Katharine Anxiety in anorexia nervosa and its management using family-based treatment. EUROPEAN EATING DISORDERS REVIEW. 2012;20(1):e1-e16.

1920. Annus Agnes, Smith Gregory, Masters Katie Manipulation of thinness and restricting expectancies: Further evidence for a causal role of thinness and restricting expectancies in the etiology of eating disorders. PSYCHOLOGY OF ADDICTIVE BEHAVIORS. 2008;22(2):278-287.

1921. Moreno-Maldonado Concepcion, Ramos Pilar, Moreno Carmen, Rivera Francisco How family socioeconomic status, peer behaviors, and school-based intervention on healthy habits influence adolescent eating behaviors. SCHOOL PSYCHOLOGY INTERNATIONAL. 2018;39(1):92-118.

1922. Teresa Plana Maria, Torres Teresa, Rodriguez Natalia, Boloc Daniel, Gasso Patricia, Moreno Elena, Lafuente Amalia, Castro-Fornieles Josefina, Mas Sergi, Lazaro Luisa Genetic variability in the serotoninergic system and age of onset in anorexia nervosa and obsessive-compulsive disorder. PSYCHIATRY RESEARCH. 2019;271():554-558.

1923. Calacattawi Abdullah, Jastaniah Salma, Almatrafi Sahar, Modhish Moatasem, Kaki Abdulrahman, Alosaimi Rayed, Sumili Ibrahim, Alzain Mohammid, Alrfaai Rakan, Makkawi Doha, Alahmadi Ayad EATING DISORDERS IN CHILDREN. INDO AMERICAN JOURNAL OF PHARMACEUTICAL SCIENCES. 2019;6(1):1504-1508.

1924. Gonzalez Marcela, Mora Marisol, Penelo Eva, Goddard Elizabeth, Treasure Janet, Raich Rosa Qualitative findings in a long-term disordered eating prevention programme follow-up with school-going girls. JOURNAL OF HEALTH PSYCHOLOGY. 2013;18(4):587-598.

1925. Holdsworth Michelle, Pradeilles Rebecca, Tandoh Akua, Green Mark, Wanjohi Milkah, Zotor Francis, Asiki Gershim, Klomegah Senam, Abdul-Haq Zakia, Osei-Kwasi Hibbah, Akparibo Robert, Bricas Nicolas, Auma Carol, Griffiths Paula, Laar Amos Unhealthy eating practices of city-dwelling Africans in deprived neighbourhoods: Evidence for policy action from Ghana and Kenya. GLOBAL FOOD SECURITY-AGRICULTURE POLICY ECONOMICS AND ENVIRONMENT. 2020;26():.

1926. Quick Virginia, Wall Melanie, Larson Nicole, Haines Jess, Neumark-Sztainer Dianne Personal, behavioral and socio-environmental predictors of overweight incidence in young adults: 10-yr longitudinal findings. INTERNATIONAL JOURNAL OF BEHAVIORAL NUTRITION AND PHYSICAL ACTIVITY. 2013;10():.

1927. Gruber NP, Dilsaver SC Bulimia and anorexia nervosa in winter depression: Lifetime rates in a clinical sample. JOURNAL OF PSYCHIATRY \& NEUROSCIENCE. 1996;21(1):9-12.

1928. Manaf Haidzir, Nor Norazmir, Azhari Nurul, Ismut Nur Eating Behaviour among Disabled Athletes in Malaysia. MALAYSIAN JOURNAL OF MEDICAL SCIENCES. 2019;26(3):129-134.

1929. Sinton Meghan, Goldschmidt Andrea, Aspen Vandana, Theim Kelly, Stein Richard, Saelens Brian, Epstein Leonard, Wilfley Denise Psychosocial Correlates of Shape and Weight Concerns in Overweight Pre-Adolescents. JOURNAL OF YOUTH AND ADOLESCENCE. 2012;41(1, SI):67-75.

1930. McAndrew Annamaria, Menna Rosanne Perceptions of disordered eating and associated help seeking in young women. EATING DISORDERS. 2018;26(2):107-126.

1931. Patel DR, Greydanus DE, Pratt HD, Phillips EL Eating disorders in adolescent athletes. JOURNAL OF ADOLESCENT RESEARCH. 2003;18(3):280-296.

1932. Boujut E., Bruchon-Schweitzer M. Eating disorders among freshmen students: A prospective multigroup study. PSYCHOLOGIE FRANCAISE. 2010;55(4):295-307.

1933. Bolton Kristy, Jacka Felice, Allender Steven, Kremer Peter, Gibbs Lisa, Waters Elizabeth, Silva Andrea The association between self-reported diet quality and health-related quality of life in rural and urban Australian adolescents. AUSTRALIAN JOURNAL OF RURAL HEALTH. 2016;24(5):317-325.

1934. Cook-Cottone C The attuned representation model for the primary prevention of eating disorders: An overview for school psychologists. PSYCHOLOGY IN THE SCHOOLS. 2006;43(2):223-230.

1935. De Pasquale Concetta, Sciacca Federica, Conti Daniela, Pistorio Maria, Hichy Zira, Cardullo Rosa, Di Nuovo Santo Relations Between Mood States and Eating Behavior During COVID-19 Pandemic in a Sample of Italian College Students. FRONTIERS IN PSYCHOLOGY. 2021;12():.

1936. BREWERTON TD, LYDIARD RB, HERZOG DB, BROTMAN AW, ONEIL PM, BALLENGER JC COMORBIDITY OF AXIS-I PSYCHIATRIC-DISORDERS IN BULIMIA-NERVOSA. JOURNAL OF CLINICAL PSYCHIATRY. 1995;56(2):77-80.

1937. Black Emma, Mildred Helen Predicting Impulsive Self-Injurious Behavior in a Sample of Adult Women. JOURNAL OF NERVOUS AND MENTAL DISEASE. 2013;201(1):72-75.

1938. Miller Alexia, Racine Sarah Emotion regulation difficulties as common and unique predictors of impulsive behaviors in university students. JOURNAL OF AMERICAN COLLEGE HEALTH. 2022;70(5):1387-1395.

1939. Sparti Claudia, Santomauro Damian, Cruwys Tegan, Burgess Philip, Harris Meredith Disordered eating among Australian adolescents: Prevalence, functioning, and help received. INTERNATIONAL JOURNAL OF EATING DISORDERS. 2019;52(3):246-254.

1940. Murray Stuart, Diaz-Fong Joel, Duval Christina, Balkchyan Ane, Nagata Jason, Lee Darrin, Ganson Kyle, Toga Arthur, Siegel Steven, Jann Kay Sex differences in regional gray

matter density in pre-adolescent binge eating disorder: a voxel-based morphometry study. PSYCHOLOGICAL MEDICINE. 2022;():.

1941. Sreenivasa Nivedita, Rao T., Malini S. Eating disorders: Prevalence in the student population of Mysore, South India. INDIAN JOURNAL OF PSYCHIATRY. 2018;60(4):433-437.

1942. Hooper Laura, Puhl Rebecca, Eisenberg Marla, Crow Scott, Neumark-Sztainer Dianne Weight teasing experienced during adolescence and young adulthood: Cross-sectional and longitudinal associations with disordered eating behaviors in an ethnically/racially and socioeconomically diverse sample. INTERNATIONAL JOURNAL OF EATING DISORDERS. 2021;54(8):1449-1462.

1943. Rodgers R., Franko D. Eating disorder prevention on college campuses: Recruitment challenges. EUROPEAN REVIEW OF APPLIED PSYCHOLOGY-REVUE EUROPEENNE DE PSYCHOLOGIE APPLIQUEE. 2015;65(3):125-131.

1944. Olivo Gaia, Dahlberg Linda, Wiemerslage Lyle, Swenne Ingemar, Zhukovsky Christina, Salonen-Ros Helena, Larsson Elna-Marie, Gaudio Santino, Brooks Samantha, Schioth Helgi Atypical anorexia nervosa is not related to brain structural changes in newly diagnosed adolescent patients. INTERNATIONAL JOURNAL OF EATING DISORDERS. 2018;51(1):39-45.

1945. Nagata Jason, Braudt David, Domingue Benjamin, Bibbins-Domingo Kirsten, Garber Andrea, Griffiths Scott, Murray Stuart Genetic risk, body mass index, and weight control behaviors: Unlocking the triad. INTERNATIONAL JOURNAL OF EATING DISORDERS. 2019;52(7):825-833.

1946. Toro J, Cervera M, Feliu MH, Garriga N, Jou M, Martinez E, Toro E Cue exposure in the treatment of resistant bulimia nervosa. INTERNATIONAL JOURNAL OF EATING DISORDERS. 2003;34(2):227-234.

1947. Leal Greisse, Philippi Sonia, Alvarenga Marle Unhealthy weight control behaviors, disordered eating, and body image dissatisfaction in adolescents from Sao Paulo, Brazil. BRAZILIAN JOURNAL OF PSYCHIATRY. 2020;42(3):264-270.

1948. Lloyd E., Overas Maria, Ro Oyvind, Verplanken Bas, Haase Anne Predicting the restrictive eating, exercise, and weight monitoring compulsions of anorexia nervosa. EATING AND WEIGHT DISORDERS-STUDIES ON ANOREXIA BULIMIA AND OBESITY.

2020;25(3):701-707.

1949. Hofer Patrizia, Wahl Karina, Meyer Andrea, Miche Marcel, Beesdo-Baum Katja, Wong Shiu, Grisham Jessica, Wittchen Hans-Ulrich, Lieb Roselind Obsessive-compulsive disorder and the risk of subsequent mental disorders: A community study of adolescents and young adults. DEPRESSION AND ANXIETY. 2018;35(4):339-345.

1950. Munn-Chernoff Melissa, Grant Julia, Agrawal Arpana, Koren Rachel, Glowinski Anne, Bucholz Kathleen, Madden Pamela, Heath Andrew, Duncan Alexis Are There Common Familial Influences for Major Depressive Disorder and an Overeating-Binge Eating Dimension in Both European American and African American Female Twins?. INTERNATIONAL JOURNAL OF EATING DISORDERS. 2015;48(4):375-382.

1951. Latzer Y, Tzischinsky O, Epstein R, Klein E, Peretz L Naturalistic sleep monitoring in women suffering from bulimia nervosa. INTERNATIONAL JOURNAL OF EATING DISORDERS. 1999;26(3):315-321.

1952. Abraham S Eating and weight controlling behaviours of young ballet dancers. PSYCHOPATHOLOGY. 1996;29(4):218-222.

1953. Rastam M, Gillberg C, Gillberg IC A six-year follow-up study of anorexia nervosa subjects with teenage onset. JOURNAL OF YOUTH AND ADOLESCENCE. 1996;25(4):439-453.

1954. Thiels Cornelia, Paetel Johanna Survey of disordered eating and behaviour in children and adolescents. ZEITSCHRIFT FUR KINDER-UND JUGENDPSYCHIATRIE UND PSYCHOTHERAPIE. 2008;36(4):265-274.

1955. Mendes Ana, Coimbra Maria, Canavarro Maria, Ferreira Claudia The powerful effect of body image inflexibility on the explanation of eating psychopathology severity. EATING AND WEIGHT DISORDERS-STUDIES ON ANOREXIA BULIMIA AND OBESITY. 2022;27(3):961-968.

1956. CASTLE DJ, DEALE A, MARKS IM GENDER DIFFERENCES IN OBSESSIVE-COMPULSIVE DISORDER. AUSTRALIAN AND NEW ZEALAND JOURNAL OF PSYCHIATRY. 1995;29(1):114-117.

1957. Levinson Cheri, Rodebaugh Thomas Social anxiety and eating disorder comorbidity: The role of negative social evaluation fears. EATING BEHAVIORS. 2012;13(1):27-35.

1958. Zaremba Natalie, Robert Glenn, Allan Jacqueline, Harrison Amy, Brown Jennie, Konstantara Emmanouela, Rosenthal Miranda, Pillay Divina, Beckwith Anita, Treasure Janet, Hopkins David, Ismail Khalida, Stadler Marietta Developing a novel intervention for type 1 diabetes and disordered eating using a participatory action design process: Safe management of people with Type 1 diabetes and EAting Disorders studY (STEADY). DIABETIC MEDICINE. 2022;39(4):.

1959. Westwood Heather, Mandy William, Tchanturia Kate Clinical evaluation of autistic symptoms in women with anorexia nervosa. MOLECULAR AUTISM. 2017;8():.

1960. Wildes JE, Simons AD, Marcus MD Bulimic symptoms, cognitions, and body dissatisfaction in women with major depressive disorder. INTERNATIONAL JOURNAL OF EATING DISORDERS. 2005;38(1):9-17.

1961. Beekley Matthew, Byrne Robert, Yavorek Trudy, Kidd Kelli, Wolff Janet, Johnson Michael Incidence, Prevalence, and Risk of Eating Disorder Behaviors in Military Academy Cadets. MILITARY MEDICINE. 2009;174(6):637-641.

1962. Tanofsky-Kraff Marian, Shomaker Lauren, Wilfley Denise, Young Jami, Sbrocco Tracy, Stephens Mark, Ranzenhofer Lisa, Elliott Camden, Brady Sheila, Radin Rachel, Vannucci Anna, Bryant Edny, Osborn Robyn, Berger Sarah, Olsen Cara, Kozlosky Merel, Reynolds James, Yanovski Jack Targeted prevention of excess weight gain and eating disorders in high-risk adolescent girls: a randomized controlled trial. AMERICAN JOURNAL OF CLINICAL NUTRITION. 2014;100(4):1010-1018.

1963. De Berardis Domenico, Fornaro Michele, Orsolini Laura, Valchera Alessandro, Carano Alessandro, Vellante Federica, Perna Giampaolo, Serafini Gianluca, Gonda Xenia, Pompili Maurizio, Martinotti Giovanni, Di Giannantonio Massimo Alexithymia and Suicide Risk in Psychiatric Disorders: A Mini-Review. FRONTIERS IN PSYCHIATRY. 2017;8():.

1964. Obeid Nicole, Flament Martine, Buchholz Annick, Henderson Katherine, Schubert Nick, Tasca Giorgio, Thai Helen, Goldfield Gary Examining Shared Pathways for Eating Disorders and Obesity in a Community Sample of Adolescents: The REAL Study. FRONTIERS IN PSYCHOLOGY. 2022;13():.

1965. Amianto Federico, Martini Matteo, Olandese Francesco, Davico Chiara, Abbate-Daga Giovanni, Fassino Secondo, Vitiello Benedetto Affectionless control: A parenting style associated with obesity and binge eating disorder in adulthood. EUROPEAN EATING DISORDERS REVIEW. 2021;29(2):178-192.

1966. Jaramillo Manuela, Burke Natasha, Shomaker Lauren, Brady Sheila, Kozlosky Merel, Yanovski Jack, Tanofsky-Kraff Marian Perceived Family Functioning in Relation to Energy Intake in Adolescent Girls with Loss of Control Eating. NUTRIENTS. 2018;10(12):.

1967. Munsch Simone, Biedert Esther, Meyer Andrea, Herpertz Stephan, Beglinger Christoph CCK, ghrelin, and PYY responses in individuals with binge eating disorder before and after a cognitive behavioral treatment (CBT). PHYSIOLOGY \& BEHAVIOR. 2009;97(1):14-20.

1968. Pursey Kirrilly, Hart Melissa, Hure Alexis, Cheung Hei, Ong Liting, Burrows Tracy, Yager Zali The Needs of School Professionals for Eating Disorder Prevention in Australian Schools: A Mixed-Methods Survey. CHILDREN-BASEL. 2022;9(12):.

1969. Elmquist JoAnna, Shorey Ryan, Anderson Scott, Stuart Gregory A preliminary investigation of the relationship between dispositional mindfulness and eating disorder symptoms among men in residential substance use treatment. ADDICTION RESEARCH \& THEORY. 2017;25(1):67-73.

1970. Balantekin Katherine, Birch Leann, Savage Jennifer Eating in the absence of hunger during childhood predicts self-reported binge eating in adolescence. EATING BEHAVIORS. 2017;24():7-10.

1971. Imperatori Claudio, Fabbricatore Mariantonietta, Lester David, Manzoni Gian, Castelnuovo Gianluca, Raimondi Giulia, Innamorati Marco Psychometric properties of the modified Yale Food Addiction Scale Version 2.0 in an Italian non-clinical sample. EATING AND WEIGHT DISORDERS-STUDIES ON ANOREXIA BULIMIA AND OBESITY. 2019;24(1):37-45.

1972. Henkel D. A Review of Prevalence Data on Substance Use, Problematic Gambling and Eating Disorders among Social Welfare Recipients (Hartz-IV) in Germany. SUCHTTHERAPIE. 2016;17(3):106-114.

1973. Canals J., Sancho C., Arija M. Influence of parent's eating attitudes on eating disorders in school adolescents. EUROPEAN CHILD \& ADOLESCENT PSYCHIATRY. 2009;18(6):353-359.

1974. Hellstrom PM, Geliebter A, Naslund E, Schmidt PT, Yahav EK, Hashim SA, Yeomans MR Peripheral and central signals in the control of eating in normal, obese and binge-eating human subjects. BRITISH JOURNAL OF NUTRITION. 2004;92(1):S47-S57.

1975. Srivastava Paakhi, Felonis Christina, Clancy Olivia, Wons Olivia, Abber Sophie, Juarascio Adrienne Real-time predictors of body dissatisfaction in females with binge eating: an ecological momentary assessment study. EATING AND WEIGHT DISORDERS-STUDIES ON ANOREXIA BULIMIA AND OBESITY. 2022;27(4):1547-1553.

1976. Quatromoni Paula A Tale of Two Runners: A Case Report of Athletes' Experiences with Eating Disorders in College. JOURNAL OF THE ACADEMY OF NUTRITION AND DIETETICS. 2017;117(1):21-31.

1977. Pejovic-Milovancevic Milica, Popovic-Deusic Smiljka, Draganic-Gajic Saveta, Lecic-Tosevski Dusica Internet Addiction - A Case Report. SRPSKI ARHIV ZA CELOKUPNO LEKARSTVO. 2009;137(1-2):86-90.

1978. Wong Yueching, Chang Yu-Jhen, Tsai Mei-Rong, Liu Tsai-Wei, Lin Wei The Body Image, Weight Satisfaction, and Eating Disorder Tendency of School Children: The 2-Year Follow-up Study. JOURNAL OF THE AMERICAN COLLEGE OF NUTRITION. 2011;30(2):126-133.

1979. Linardon Jake, Incerti Lisa, McLean Courtney Factor structure and psychometric properties of the Inflexible Eating Questionnaire in a sample of adult women. APPETITE. 2019;142():.

1980. Sahlan Reza, Sala Margaret Eating disorder psychopathology and negative affect in Iranian college students: a network analysis. JOURNAL OF EATING DISORDERS. 2022;10(1):.

1981. Henin A, Kendall PC Obsessive-compulsive disorder in childhood and adolescence. . 1997;19():75-131.

1982. Jung Jin-Yi, Kim Kye-Hyun, Woo Hee-Yeon, Shin Dong-Won, Shin Young-Chul, Oh Kang-Seob, Shin Eun-Hee, Lim Se-Won Binge eating is associated with trait anxiety in Korean adolescent girls: a cross sectional study. BMC WOMENS HEALTH. 2017;17():.

1983. Black Donald Obsessive-Compulsive Disorder and Its Potential Subtypes. CNS SPECTRUMS. 2000;5(6, 4):40-46.

1984. Torres-McGehee Toni, Green James, Leaver-Dunn Deidre, Leeper James, Bishop Phillip, Richardson Mark ATTITUDE AND KNOWLEDGE CHANGES IN COLLEGIATE DANCERS FOLLOWING A SHORT-TERM, TEAM-CENTERED PREVENTION PROGRAM ON EATING DISORDERS. PERCEPTUAL AND MOTOR SKILLS. 2011;112(3):711-725.

1985. Black DW Compulsive buying disorder - Definition, assessment, epidemiology and clinical management. CNS DRUGS. 2001;15(1):17-27.

1986. Hellberg Samantha, Ladis Ilana, Shepherd Caitlin Pilot study of a personality-based approach to assessing eating disorder and Obsessive Compulsive Disorder symptom risk in college men and women. JOURNAL OF AMERICAN COLLEGE HEALTH. 2019;67(8):801-816.

1987. Godart NT, Flament MF, Curt F, Perdereau F, Lang F, Venisse JL, Halfon O, Bizouard P, Loas G, Corcos M, Jeammet P, Fermanian J Anxiety disorders in subjects seeking treatment for eating disorders: a DSM-IV controlled study. PSYCHIATRY RESEARCH. 2003;117(3):245-258.

1988. Rodriguez-Ortega Elisa, Alcaraz-Iborra Manuel, Fuente Leticia, Cubero Inmaculada Protective and therapeutic benefits of environmental enrichment on binge-like sucrose intake in C57BL/6J mice. APPETITE. 2019;138():184-189.

1989. Holland Lauren, Bodell Lindsay, Keel Pamela Psychological Factors Predict Eating Disorder Onset and Maintenance at 10-year Follow-up. EUROPEAN EATING DISORDERS REVIEW. 2013;21(5):405-410.

1990. Killen JD, Taylor CB, Hayward C, Haydel KF, Wilson DM, Hammer L, Kraemer H, BlairGreiner A, Strachowski D Weight concerns influence the development of eating disorders: A 4-year prospective study. JOURNAL OF CONSULTING AND CLINICAL PSYCHOLOGY. 1996;64(5):936-940.

1991. Rauh Mitchell, Nichols Jeanne, Barrack Michelle Relationships Among Injury and Disordered Eating, Menstrual Dysfunction, and Low Bone Mineral Density in High School Athletes: A Prospective Study. JOURNAL OF ATHLETIC TRAINING. 2010;45(3):243-252.

1992. Scott Stephanie, Beyer Fiona, Parkinson Kathryn, Muir Cassey, Graye Alice, Kaner Eileen, Stead Martine, Power Christine, Fitzgerald Niamh, Bradley Jen, Wrieden Wendy, Adamson Ashley Non-Pharmacological Interventions to Reduce Unhealthy Eating and Risky Drinking in Young Adults Aged 18-25 Years: A Systematic Review and Meta-Analysis. NUTRIENTS. 2018;10(10):.

1993. Pedrelli Paola, Nyer Maren, Yeung Albert, Zulauf Courtney, Wilens Timothy College Students: Mental Health Problems and Treatment Considerations. ACADEMIC PSYCHIATRY. 2015;39(5):503-511.

1994. Valente Martina, Syurina Elena, Muftugil-Yalcin Seda, Cesuroglu Tomris ``Keep Yourself Alive{''}: From Healthy Eating to Progression to Orthorexia Nervosa A Mixed Methods Study among Young Women in the Netherlands. ECOLOGY OF FOOD AND NUTRITION. 2020;59(6):578-597.

1995. Zerwas Stephanie, Larsen Janne, Petersen Liselotte, Thornton Laura, Mortensen Preben, Bulik Cynthia The incidence of eating disorders in a Danish register study: Associations with suicide risk and mortality. JOURNAL OF PSYCHIATRIC RESEARCH. 2015;65():16-22.

1996. Watson Hunna, McLagan Nicole, Zerwas Stephanie, Crosby Ross, Levine Michele, Runfola Cristin, Peat Christine, Moessner Markus, Zimmer Benjamin, Hofmeier Sara, Hamer Robert, Marcus Marsha, Bulik Cynthia, Crow Scott Cost-Effectiveness of Internet-Based Cognitive-Behavioral Treatment for Bulimia Nervosa: Results of a Randomized Controlled Trial. JOURNAL OF CLINICAL PSYCHIATRY. 2018;79(1):.

1997. Tatlow-Golden Mimi, Hennessy Eilis, Dean Moira, Hollywood Lynsey Young children's food brand knowledge. Early development and associations with television viewing and parent's diet. APPETITE. 2014;80():197-203.

1998. Liberman Tamar, Burke Natasha The development and validation of the Conscious Objectification Questionnaire. INTERNATIONAL JOURNAL OF EATING DISORDERS. 2022;55(8):1162-1168.

1999. Van Son Gabrielle, Meer Paul, Van Furth Eric Correlates and associations between weight suppression and binge eating symptomatology in a population-based sample. EATING BEHAVIORS. 2013;14(2):102-106.

2000. COKER S ONSET OF BULIMIA-NERVOSA IN A 64-YEAR-OLD WOMAN. INTERNATIONAL JOURNAL OF EATING DISORDERS. 1994;16(1):89-91.

2001. Kerbeshian Jacob, Burd Larry Is anorexia nervosa a neuropsychiatric developmental disorder? An illustrative case report. WORLD JOURNAL OF BIOLOGICAL PSYCHIATRY. 2009;10(4, 2):648-657.

2002. FRENCH SA, PERRY CL, LEON GR, FULKERSON JA DIETING BEHAVIORS AND WEIGHT CHANGE HISTORY IN FEMALE ADOLESCENTS. HEALTH PSYCHOLOGY. 1995;14(6):548-555.

2003. Dakanalis Antonios, Clerici Massimo, Caslini Manuela, Gaudio Santino, Serino Silvia, Riva Giuseppe, Carra Giuseppe Predictors of initiation and persistence of recurrent binge eating and inappropriate weight compensatory behaviors in college men. INTERNATIONAL JOURNAL OF EATING DISORDERS. 2016;49(6):581-590.

2004. Lal M., Abraham S. Adolescent development and eating disorder related quality of life in Indian females. EATING AND WEIGHT DISORDERS-STUDIES ON ANOREXIA BULIMIA AND OBESITY. 2011;16(1):E56-E60.

2005. Messer Mariel, McClure Zoe, Norton Bethany, Smart Melanie, Linardon Jake Using an app to count calories: Motives, perceptions, and connections to thinness- and muscularity-oriented disordered eating. EATING BEHAVIORS. 2021;43():.

2006. Santos Amanda, Guerra Benute Glaucia, Santos Niraldo, Yamamoto Nomura Roseli, Lucia Mara, Vieira Francisco Rossana Presence of eating disorders and its relationship to anxiety and depression in pregnant women. MIDWIFERY. 2017;51():12-15.

2007. Eli Karin, Warin Megan Anthropological Perspectives on Eating Disorders: Deciphering Cultural Logics. TRANSCULTURAL PSYCHIATRY. 2018;55(4, SI):443-453.

2008. Rathner G, Rainer B Annual treatment rates and estimated prevalence of eating disorders in Austria. WIENER KLINISCHE WOCHENSCHRIFT. 1997;109(8):275-280.

2009. Castillo Irais, Solano Santos, Sepulveda Ana Prevention program for disordered eating and obesity among Mexican university students. BEHAVIORAL PSYCHOLOGY-PSICOLOGIA CONDUCTUAL. 2016;24(1):5-28.

2010. Lipson Sarah, Sonneville Kendrin Understanding suicide risk and eating disorders in college student populations: Results from a National Study. INTERNATIONAL JOURNAL OF EATING DISORDERS. 2020;53(2):229-238.

2011. Quirk-Baillot D., Flament M., Allen A., Obeid N., Remy B., Falissard B., Godart N. The Attitudes and Patterns of Eating (APE) Questionnaire: Development and factor analysis in a US adolescent community sample. EATING AND WEIGHT DISORDERS-STUDIES ON ANOREXIA BULIMIA AND OBESITY. 2012;17(2):E147-E156.

2012. Beals KA, Manore MM Behavioral, psychological, and physical characteristics of female athletes with subclinical eating disorders. INTERNATIONAL JOURNAL OF SPORT NUTRITION. 2000;10(2):128-143.

2013. Nakaoka Hirotomo, Mogi Masaki, Kan-no Harumi, Tsukuda Kana, Ohshima Kousei, Wang Xiao-Li, Chisaka Toshiyuki, Bai Hui-Yu, Shan Bao-Shuai, Kukida Masayoshi, Iwanami Jun, Horiuchi Masatsugu Angiotensin II type 2 receptor signaling affects dopamine levels in the brain and prevents binge eating disorder. JOURNAL OF THE RENIN-ANGIOTENSIN-ALDOSTERONE SYSTEM. 2015;16(4):749-757.

2014. Lilenfeld LR, Kaye WH The link between alcoholism and eating disorders. ALCOHOL HEALTH \& RESEARCH WORLD. 1996;20(2):94-99.

2015. SCHMIDT U BULIMIA-NERVOSA IN THE CHINESE. INTERNATIONAL JOURNAL OF EATING DISORDERS. 1993;14(4):505-509.

2016. Marcus Marsha, Gandica Rachelle, Grp Today Longitudinal Association of Depressive Symptoms, Binge Eating, and Quality of Life With Cardiovascular Risk Factors in Young Adults With Youth-Onset Type 2 Diabetes: The TODAY2 Study. DIABETES CARE. 2022;45(5):1073-1081.

2017. Shaw Laura-Kate, Homewood Judi The Effect of Eating Disorder Memoirs in Individuals With Self-Identified Eating Pathologies. JOURNAL OF NERVOUS AND MENTAL DISEASE. 2015;203(8):591-595.

2018. Bailer UF, Price JC, Meltzer CC, Mathis CA, Frank GK, Weissfeld L, McConaha CW, Henry SE, Brooks-Achenbach S, Barbarich NC, Kaye WH Altered 5-HT2A receptor binding after recovery from bulimia-type anorexia nervosa: Relationships to harm avoidance and drive for thinness. NEUROPSYCHOPHARMACOLOGY. 2004;29(6):1143-1155.

2019. Werneck Andre, Silva Danilo, Malta Deborah, Gomes Crizian, Souza-Junior Paulo, Azevedo Luiz, Barros Marilisa, Szwarcwald Celia Associations of sedentary behaviours and incidence of unhealthy diet during the COVID-19 quarantine in Brazil. PUBLIC HEALTH NUTRITION. 2021;24(3):422-426.

2020. Salafia Elizabeth, Gondoli Dawn, Corning Alexandra, McEnery Amanda, Grundy Amber Psychological distress as a mediator of the relation between perceived maternal parenting and normative maladaptive eating among adolescent girls. JOURNAL OF COUNSELING PSYCHOLOGY. 2007;54(4):434-446.

2021. Tate Ashley, Liu Shengxin, Zhang Ruyue, Yilmaz Zeynep, Larsen Janne, Petersen Liselotte, Bulik Cynthia, Svensson Ann-Marie, Gudbjornsdottir Soffia, Larsson Henrik, Butwicka Agnieszka, Kuja-Halkola Ralf Association and Familial Coaggregation of Type 1 Diabetes and Eating Disorders: A Register-Based Cohort Study in Denmark and Sweden. DIABETES CARE. 2021;44(5):1143-1150.

2022. Tanofsky-Kraff Marian, Wilfley Denise, Young Jami, Mufson Laura, Yanovski Susan, Glasofer Deborah, Salaita Christine, Schvey Natasha A Pilot Study of Interpersonal Psychotherapy for Preventing Excess Weight Gain in Adolescent Girls at-risk for Obesity. INTERNATIONAL JOURNAL OF EATING DISORDERS. 2010;43(8):701-706.

2023. Vogel Suzan, Bijlenga Denise, Tanke Marjolein, Bron Tannetje, Heijden Kristiaan, Swaab Hanna, Beekman Aartjan, Kooij J. Circadian rhythm disruption as a link between Attention-Deficit/Hyperactivity Disorder and obesity?. JOURNAL OF PSYCHOSOMATIC RESEARCH. 2015;79(5):443-450.

2024. Alciati Alessandra, Caldirola Daniela, Foschi Diego, Perna Giampaolo Psychiatric Disorders and Childhood Parental Loss in Obesity: Relationship with the Mode of Weight Gain. JOURNAL OF LOSS \& TRAUMA. 2016;21(3):213-224.

2025. Rodriguez Rosalia, Marchand Erica, Ng Janet, Stice Eric Effects of a Cognitive Dissonance-Based Eating Disorder Prevention Program Are Similar for Asian American, Hispanic, and White Participants. INTERNATIONAL JOURNAL OF EATING DISORDERS. 2008;41(7):618-625.

2026. Thompson Sharon Characteristics of the Female Athlete Triad in collegiate cross-country runners. JOURNAL OF AMERICAN COLLEGE HEALTH. 2007;56(2):129-136.

2027. Isomaa Rasmus, Isomaa Anna-Lisa, Marttunen Mauri, Kaltiala-Heino Riittakerttu, Bjorkqvist Kaj The Prevalence, Incidence and Development of Eating Disorders in Finnish Adolescents-A Two-step 3-year Follow-up Study. EUROPEAN EATING DISORDERS REVIEW. 2009;17(3):199-207.

2028. Brockdorf Alexandra, Kennedy Grace, Keel Pamela Examining associations among sensitivity to punishment and reward, shame, and eating pathology through tests of mediation. EATING DISORDERS. 2018;26(5):407-417.

2029. Giladi Nir, Weitzman Nina, Schreiber Shaul, Shabtai Herzet, Peretz Chava New onset heightened interest or drive for gambling, shopping, eating or sexual activity in patients with Parkinson's disease: the rote of dopamine agonist treatment and age at motor symptoms onset. JOURNAL OF PSYCHOPHARMACOLOGY. 2007;21(5):501-506.

2030. Fatima Waseem, Ahmad Leena Prevalence of disordered eating attitudes among adolescent girls in Arar City, Kingdom of Saudi Arabia. HEALTH PSYCHOLOGY RESEARCH. 2018;6(1):30-35.

2031. Wagner Gudrun, Karwautz Andreas Eating disorders in adolescents with type 1 diabetes mellitus. CURRENT OPINION IN PSYCHIATRY. 2020;33(6):602-610.

2032. Messer Mariel, Duxson Siahn, Diluvio Paige, McClure Zoe, Linardon Jake The independent contribution of muscularity-oriented disordered eating to functional impairment and emotional distress in adult men and women. EATING DISORDERS. 2023;31(2):161-172.

2033. Branson R, Potoczna N, Kral JG, Lentes K, Hoehe MR, Horber FF Binge eating as a major phenotype of melanocortin 4 receptor gene mutations. NEW ENGLAND JOURNAL OF MEDICINE. 2003;348(12):1096-1103.

2034. Nakajima Kei Unhealthy eating habits around sleep and sleep duration: To eat or fast?. WORLD JOURNAL OF DIABETES. 2018;9(11):190-194.

2035. Grilo Carlos, White Marney Orlistat with behavioral weight loss for obesity with versus without binge eating disorder: Randomized placebo-controlled trial at a community mental health center serving educationally and economically disadvantaged Latino/as. BEHAVIOUR RESEARCH AND THERAPY. 2013;51(3):167-175.

2036. Wertheim EH, Paxton SJ, Schutz HK, Muir SL Why do adolescent girls watch their weight? An interview study examining sociocultural pressures to be thin. JOURNAL OF PSYCHOSOMATIC RESEARCH. 1997;42(4):345-355.

2037. Johnson L., Boyd L., Rainchuso L., Rothman A., Mayer B. Eating disorder professionals' perceptions of oral health knowledge. INTERNATIONAL JOURNAL OF DENTAL HYGIENE. 2017;15(3):164-171.

2038. Buoli Massimiliano, Grassi Silvia, Ciappolino Valentina, Serati Marta, Altamura Alfredo The Use of Zonisamide for the Treatment of Psychiatric Disorders: A Systematic Review. CLINICAL NEUROPHARMACOLOGY. 2017;40(2):85-92.

2039. Sultson Hedvig, Akkermann Kirsti Investigating phenotypes of emotional eating based on weight categories: A latent profile analysis. INTERNATIONAL JOURNAL OF EATING DISORDERS. 2019;52(9):1024-1034.

2040. Holtkamp K, Konrad K, Kaiser N, Ploenes Y, Heussen N, Grzella I, Herpertz-Dahlmann B A retrospective study of SSRI treatment in adolescent anorexia nervosa: insufficient evidence for efficacy. JOURNAL OF PSYCHIATRIC RESEARCH. 2005;39(3):303-310.

2041. Pinhas Leora, McVey Gail, Walker Kathryn, Norris Mark, Katzman Debra, Collier Sarah Trading Health for a Healthy Weight: The Uncharted Side of Healthy Weights Initiatives. EATING DISORDERS. 2013;21(2):109-116.

2042. Bagheri Minoo, Willett Walter, Townsend Mary, Kraft Peter, Ivey Kerry, Rimm Eric, Wilson Kathryn, Costenbader Karen, Karlson Elizabeth, Poole Elizabeth, Zeleznik Oana, Eliassen A. A lipid-related metabolomic pattern of diet quality. AMERICAN JOURNAL OF CLINICAL NUTRITION. 2020;112(6):1613-1630.

2043. Perey Iris, Cook-Cottone Catherine Eating disorders, embodiment, and yoga: a conceptual overview. EATING DISORDERS. 2020;28(4, SI):315-329.

2044. Hollander E, Benzaquen SD The obsessive-compulsive spectrum disorders. INTERNATIONAL REVIEW OF PSYCHIATRY. 1997;9(1):99-109.

2045. Hege M., Stingl K., Kullmann S., Schag K., Giel K., Zipfel S., Preissl H. Attentional impulsivity in binge eating disorder modulates response inhibition performance and frontal brain networks. INTERNATIONAL JOURNAL OF OBESITY. 2015;39(2):353-360.

2046. Ono Y, Berger D, Saito S, Takahashi Y, Kuboki T, Ishikawa Y, Tezuka I, Nakamura K, Suematsu H, Asai M Relationship of childhood abuse to psychiatric distress, social adjustment, and eating disorder severity in Japanese bulimics. EUROPEAN EATING DISORDERS REVIEW. 1996;4(2):121-130.

2047. Bryant Emma, Spielman Karen, Le Anvi, Marks Peta, Touyz Stephen, Maguire Sarah, Consortiu Natl Screening, assessment and diagnosis in the eating disorders: findings from a rapid review. JOURNAL OF EATING DISORDERS. 2022;10(1):.

2048. Perkins AJ, Fritz JJ, Barber CE, Turner JG The prevalence and family correlates of eating disorder tendencies in older women. JOURNAL OF WOMEN \& AGING. 1997;9(3):67-84.

2049. Burnette C., Simpson Courtney, Mazzeo Suzanne Relation of BMI and weight suppression to eating pathology in undergraduates. EATING BEHAVIORS. 2018;30():16-21.

2050. Stice Eric, Butryn Meghan, Rohde Paul, Shaw Heather, Marti C. An effectiveness trial of a new enhanced dissonance eating disorder prevention program among female college students. BEHAVIOUR RESEARCH AND THERAPY. 2013;51(12):862-871.

2051. Solmi Marco, Collantoni Enrico, Meneguzzo Paolo, Tenconi Elena, Favaro Angela Network analysis of specific psychopathology and psychiatric symptoms in patients with anorexia nervosa. EUROPEAN EATING DISORDERS REVIEW. 2019;27(1):24-33.

2052. RAVELLI AM, HELPS BA, DEVANE SP, LASK BD, MILLA PJ NORMAL GASTRIC ANTRAL MYOELECTRICAL ACTIVITY IN EARLY-ONSET ANOREXIA-NERVOSA. ARCHIVES OF

DISEASE IN CHILDHOOD. 1993;69(3):342-346.

2053. Abild Caroline, Jensen Annesofie, Lassen Rikke, Vestergaard Esben, Bruun Jens, Kristensen Kurt, Stoving Rene, Clausen Loa Patients' perspectives on screening for disordered eating among adolescents with type 1 diabetes. EATING AND WEIGHT DISORDERS-STUDIES ON ANOREXIA BULIMIA AND OBESITY. 2023;28(1):.

2054. Alleva Jessica, Paraskeva Nicole, Craddock Nadia, Stuijfzand Bobby, Diedrichs Phillippa A longitudinal study investigating positive body image, eating disorder symptoms, and other related factors among a community sample of men in the UK. BODY IMAGE. 2022;41():384-395.

2055. Wentz E, Gillberg C, Gillberg IC, Rastam M Ten-year follow-up of adolescent-onset anorexia nervosa: Psychiatric disorders and overall functioning scales. JOURNAL OF CHILD PSYCHOLOGY AND PSYCHIATRY. 2001;42(5):613-622.

2056. Tanaka Kenichiro, Wada-Isoe Kenji, Nakashita Satoko, Yamamoto Mikie, Nakashima Kenji Impulsive compulsive behaviors in Japanese Parkinson's disease patients and utility of the Japanese version of the Questionnaire for Impulsive-Compulsive Disorders in Parkinson's disease. JOURNAL OF THE NEUROLOGICAL SCIENCES. 2013;331(1-2):76-80.

2057. Taborelli E., Krug I., Karwautz A., Wagner G., Haidvogl M., Fernandez-Aranda F., Castro R., Jimenez-Murcia S., Anderluh M., Collier D., Treasure J., Micali N. Maternal Anxiety, Overprotection and Anxious Personality as Risk Factors for Eating Disorder: A Sister Pair Study. COGNITIVE THERAPY AND RESEARCH. 2013;37(4):820-828.

2058. Vannucci Anna, Shomaker Lauren, Field Sara, Sbrocco Tracy, Stephens Mark, Kozlosky Merel, Reynolds James, Yanovski Jack, Tanofsky-Kraff Marian History of Weight Control Attempts Among Adolescent Girls With Loss of Control Eating. HEALTH PSYCHOLOGY. 2014;33(5):419-423.

2059. Jankowski Glen, Gough Brendan, Fawkner Helen, Halliwell Emma, Diedrichs Phillippa Young men's minimisation of their body dissatisfaction. PSYCHOLOGY \& HEALTH. 2018;33(11):1343-1363.

2060. Mehl Annette, Rohde Paul, Gau Jeff, Stice Eric Disaggregating the predictive effects of impaired psychosocial functioning on future DSM-5 eating disorder onset in high-risk female adolescents. INTERNATIONAL JOURNAL OF EATING DISORDERS. 2019;52(7):817-

824.

2061. Gusella Joanne, Goodwin Jacqueline, Roosmalen Erica `I want to lose weight': Early risk for disordered eating?. PAEDIATRICS \& CHILD HEALTH. 2008;13(2):105-110.

2062. Mason Tyler, Smith Kathryn, Crosby Ross, Engel Scott, Peterson Carol, Wonderlich Stephen, Jin Haomiao Multi-state modeling of thought-shape fusion using ecological momentary assessment. BODY IMAGE. 2021;39():139-145.

2063. Feinson Marjorie, Meir Adi Disordered Eating and Cultural Distinctions: Exploring Prevalence and Predictors among Women in Israel. ISRAEL JOURNAL OF PSYCHIATRY AND RELATED SCIENCES. 2014;51(2):145-153.

2064. Clayton Heather, Demissie Zewditu, Lowry Richard, Lundeen Elizabeth, Sharma Andrea, Bohm Michele Unhealthy Weight Management Practices and Non-medical Use of Prescription Drugs. AMERICAN JOURNAL OF PREVENTIVE MEDICINE. 2017;52(2):215-219.

2065. Kraeling Svenja, Losekam Stefanie, Goetzky Benjamin, Rief Winfried, Hilbert Anja Impact of Weight-Related Discrimination on Eating Disorder and General Psychopathology in Children and Adolescents with and without Migration Background. PSYCHOTHERAPIE PSYCHOSOMATIK MEDIZINISCHE PSYCHOLOGIE. 2010;60(9-10):397-401.

2066. Jackson Todd, Chen Hong Features of Objectified Body Consciousness and Sociocultural Perspectives as Risk Factors for Disordered Eating Among Late-Adolescent Women and Men. JOURNAL OF COUNSELING PSYCHOLOGY. 2015;62(4):741-752.

2067. Shisslak CM, Renger R, Sharpe T, Crago M, McKnight KM, Gray N, Bryson S, Estes LS, Parnaby OG, Killen J, Taylor CB Development and evaluation of the McKnight Risk Factor Survey for assessing potential risk and protective factors for disordered eating in preadolescent and adolescent girls. INTERNATIONAL JOURNAL OF EATING DISORDERS. 1999;25(2):195-214.

2068. Lapid Maria, Prom Maria, Burton M., McAlpine Donald, Sutor Bruce, Rummans Teresa Eating disorders in the elderly. INTERNATIONAL PSYCHOGERIATRICS. 2010;22(4):523-536.

2069. Barnhart Wesley, Cui Shuqi, Cui Tianxiang, He Jinbo Relationships between weight bias internalization and biopsychosocial health outcomes: A prospective study in Chinese adolescents. INTERNATIONAL JOURNAL OF EATING DISORDERS. 2023;56(5):1021-1033.

2070. Santos Rodrigues Ana, Silveira Erika Super-obesity associated factors in women: binge eating and food intake. RBONE-REVISTA BRASILEIRA DE OBESIDADE NUTRICAO E EMAGRECIMENTO. 2018;12(73):643-654.

2071. Woropay-Hordziejewicz Natalia, Buzniak Aleksandra, Lawendowski Rafal, Atroszko Pawel Compulsive Study Behaviors Are Associated with Eating Disorders and Have Independent Negative Effects on Well-Being: A Structural Equation Model Study among Young Musicians. SUSTAINABILITY. 2022;14(14):.

2072. Mac Neil Brad, Leung Pauline, Montemarano Vanessa Exposure with response prevention (ERP) for body dissatisfaction in a group therapy format: an exploratory study. EATING AND WEIGHT DISORDERS-STUDIES ON ANOREXIA BULIMIA AND OBESITY. 2018;23(2):225-232.

2073. Norton Lyza, Parkinson Joy, Harris Neil, Hart Laura What Factors Predict the Use of Coercive Food Parenting Practices among Mothers of Young Children? An Examination of Food Literacy, Disordered Eating and Parent Demographics. INTERNATIONAL JOURNAL OF ENVIRONMENTAL RESEARCH AND PUBLIC HEALTH. 2021;18(19):.

2074. Utter Jennifer, Denny Simon, Percival Teuila, Crengle Sue, Ameratunga Shanthi, Dixon Robyn, Teevale Tasileta, Hall Anganette Prevalence of weight-related concerns and behaviours among New Zealand young people. JOURNAL OF PAEDIATRICS AND CHILD HEALTH. 2012;48(11):1021-1028.

2075. Berner Laura, Arigo Danielle, Mayer Laurel, Sarwer David, Lowe Michael Examination of central body fat deposition as a risk factor for loss-of-control eating. AMERICAN JOURNAL OF CLINICAL NUTRITION. 2015;102(4):736-744.

2076. Lubrano-Berthelier C, Dubern B, Lacorte JM, Picard F, Shapiro A, Zhang SM, Bertrais S, Hercberg S, Basdevant A, Clement K, Vaisse C Melanocortin 4 receptor mutations in a large cohort of severely obese adults: Prevalence, functional classification, genotype-phenotype relationship, and lack of association with binge eating. JOURNAL OF CLINICAL ENDOCRINOLOGY \& METABOLISM. 2006;91(5):1811-1818.

2077. Goode Rachel, Cowell Mariah, Mazzeo Suzanne, Cooper-Lewter Courtney, Forte Alexandria, Olayia Oona-Ife, Bulik Cynthia Binge eating and binge-eating disorder in Black women: A systematic review. INTERNATIONAL JOURNAL OF EATING DISORDERS. 2020;53(4):491-507.

2078. Kollei Ines, Lukas Christian, Loeber Sabine, Berking Matthias An App-Based Blended Intervention to Reduce Body Dissatisfaction: A Randomized Controlled Pilot Study. JOURNAL OF CONSULTING AND CLINICAL PSYCHOLOGY. 2017;85(11):1104-1108.

2079. Vigna Luisella, Brunani Amelia, Brugnera Agostino, Grossi Enzo, Compare Angelo, Tirelli Amedea, Conti Diana, Agnelli Gianna, Andersen Lars, Buscema Massimo, Riboldi Luciano Determinants of metabolic syndrome in obese workers: gender differences in perceived job-related stress and in psychological characteristics identified using artificial neural networks. EATING AND WEIGHT DISORDERS-STUDIES ON ANOREXIA BULIMIA AND OBESITY. 2019;24(1):73-81.

2080. Manley RS, Rickson H, Standeven B Children and adolescents with eating disorders: Strategies for teachers and school counselors. INTERVENTION IN SCHOOL AND CLINIC. 2000;35(4):228-231.

2081. Gruber Maria, Koenigi Daniel, Holzhaeuser Julika, Castillo Deirdre, Blueml Victor, Jahn Rebecca, Leser Carmen, Werneck-Rohrer Sonja, Werneck Harald Parental feeding practices and the relationship with parents in female adolescents and young adults with eating disorders: A case control study. PLOS ONE. 2020;15(11):.

2082. Goel Neha, Sadeh-Sharvit Shiri, Flatt Rachael, Trockel Mickey, Balantekin Katherine, Fitzsimmons-Craft Ellen, Monterubio Grace, Firebaugh Marie-Laure, Jacobi Corinna, Wilfley Denise, Taylor C. Correlates of suicidal ideation in college women with eating disorders. INTERNATIONAL JOURNAL OF EATING DISORDERS. 2018;51(6):579-584.

2083. Kalucka Sylwia, Kaleta Dorota, Makowiec-Dabrowska Teresa Prevalence of Dietary Behavior and Determinants of Quality of Diet among Beneficiaries of Government Welfare Assistance in Poland. INTERNATIONAL JOURNAL OF ENVIRONMENTAL RESEARCH AND PUBLIC HEALTH. 2019;16(3):.

2084. Vogele Claus, Hilbert Anja, Tuschen-Caffier Brunna Dietary restriction, cardiac autonomic regulation and stress reactivity in bulimic women. PHYSIOLOGY \& BEHAVIOR. 2009;98(1-2):229-234.

2085. Zhou Nan, Cheah Charissa, Li Yan, Liu Junsheng, Sun Shuyan The Role of Maternal and Child Characteristics in Chinese Children's Dietary Intake Across Three Groups. JOURNAL OF PEDIATRIC PSYCHOLOGY. 2018;43(5):503-512.

2086. Hildebrandt Britny, Klump Kelly, Racine Sarah, Sisk Cheryl Differential strain vulnerability to binge eating behaviors in rats. PHYSIOLOGY \& BEHAVIOR. 2014;127():81-86.

2087. Haslam Rebecca, Clarke Erin, Gray Scarlett, Gearon Rachel, Pursey Kirrilly Findings from a web content analysis of resources targeting sporting coaches aimed at educating or upskilling on eating disorders and disordered eating in athletes. JOURNAL OF EATING DISORDERS. 2021;9(1):.

2088. Davis C Excessive exercise and anorexia nervosa: Addictive and compulsive behaviors. PSYCHIATRIC ANNALS. 1999;29(4):221+.

2089. Berg Cecilie, Bulik Cynthia, Von Holle Ann, Torgersen Leila, Hamer Robert, Sullivan Patrick, Reichborn-Kjennerud Ted Psychosocial factors associated with broadly defined bulimia nervosa during early pregnancy: findings from the Norwegian mother and child cohort study. AUSTRALIAN AND NEW ZEALAND JOURNAL OF PSYCHIATRY. 2008;42(5):396-404.

2090. Couturier Jennifer, Pellegrini Danielle, Miller Catherine, Bhatnagar Neera, Boachie Ahmed, Bourret Kerry, Brouwers Melissa, Coelho Jennifer, Dimitropoulos Gina, Findlay Sheri, Ford Catherine, Geller Josie, Grewal Seena, Gusella Joanne, Isserlin Leanna, Jericho Monique, Johnson Natasha, Katzman Debra, Kimber Melissa, Lafrance Adele, Leclerc Anick, Loewen Rachel, Loewen Techiya, McVey Gail, Norris Mark, Pilon David, Preskow Wendy, Spettigue Wendy, Steinegger Cathleen, Waite Elizabeth, Webb Cheryl The COVID-19 pandemic and eating disorders in children, adolescents, and emerging adults: virtual care recommendations from the Canadian consensus panel during COVID-19 and beyond. JOURNAL OF EATING DISORDERS. 2021;9(1):.

2091. Bass M, Turner L, Hunt S Counseling female athletes: Application of the stages of change model to avoid disordered eating, amenorrhea, and osteoporosis. PSYCHOLOGICAL REPORTS. 2001;88(3, 2):1153-1160.

2092. Bancheri Lara, Patrizi Barbara, Kotzalidis Giorgio, Mosticoni Stefano, Gargano Imena, Angrisani Patrizia, Tatarelli Roberto, Girard Paolo Treatment choice and psychometric characteristics: Differences between patients who choose bariatric surgical treatment and

those who do not. OBESITY SURGERY. 2006;16(12):1630-1637.

2093. Siqueira KS, Appolinario JC, Sichieri R Overweight, obesity, and binge eating in a non-clinical sample of five Brazilian cities. OBESITY RESEARCH. 2004;12(12):1921-1924.

2094. Magee Meghan, Jones Margaret, Fields Jennifer, Kresta Julie, Khurelbaatar Chinguun, Dodge Christopher, Merfeld Brandon, Ambrosius Abby, Carpenter Makenna, Jagim Andrew Body Composition, Energy Availability, Risk of Eating Disorder, and Sport Nutrition Knowledge in Young Athletes. NUTRIENTS. 2023;15(6):.

2095. Steinglass Joanna, Albano Anne, Simpson H., Wang Yuanjia, Zou Jingjing, Attia Evelyn, Walsh B. Confronting Fear Using Exposure and Response Prevention for Anorexia Nervosa: A Randomized Controlled Pilot Study. INTERNATIONAL JOURNAL OF EATING DISORDERS. 2014;47(2):174-180.

2096. Vo Phuong, Fowler Natasha, Rolan Emily, Culbert Kristen, Racine Sarah, Burt S., Klump Kelly The effects of puberty on associations between mood/personality factors and disordered eating symptoms in girls. INTERNATIONAL JOURNAL OF EATING DISORDERS. 2021;54(9):1619-1631.

2097. Weiderman MW, Pryor T Substance use and impulsive behaviors among adolescents with eating disorders. ADDICTIVE BEHAVIORS. 1996;21(2):269-272.

2098. Lau David, Douketis James, Morrison Katherine, Hramiak Irene, Sharma Arya, Ur Ehud, Guide Obesity 2006 Canadian clinical practice guidelines on the management and prevention of obesity in adults and children {[}summary]. CANADIAN MEDICAL ASSOCIATION JOURNAL. 2007;176(8):S1-S13.

2099. Ali Kathina, Fassnacht Daniel, Farrer Louise, Rieger Elizabeth, Moessner Markus, Bauer Stephanie, Griffiths Kathleen Recruitment, adherence and attrition challenges in internet-based indicated prevention programs for eating disorders: lessons learned from a randomised controlled trial of ProYouth OZ. JOURNAL OF EATING DISORDERS. 2022;10(1):.

2100. Latzer Yael, Weinberger-Litman Sarah, Spivak-Lavi Zohar, Tzischinsky Orna Disordered Eating Pathology and Body Image Among Adolescent Girls in Israel: The Role of Sense of Coherence. COMMUNITY MENTAL HEALTH JOURNAL. 2019;55(7):1246-1252.

2101. Nagata Jason, Garber Andrea, Tabler Jennifer, Murray Stuart, Bibbins-Domingo Kirsten Differential Risk Factors for Unhealthy Weight Control Behaviors by Sex and Weight Status Among US Adolescents. JOURNAL OF ADOLESCENT HEALTH. 2018;63(3):335-341.

2102. Holtkamp K, Muller B, Heussen N, Remschmidt H, Herpertz-Dahlmann B Depression, anxiety, and obsessionality in long-term recovered patients with adolescent-onset anorexia nervosa. EUROPEAN CHILD \& ADOLESCENT PSYCHIATRY. 2005;14(2):106-110.

2103. Wisting Line, Reas Deborah, Bang Lasse, Skrivarhaug Torild, Dahl-Jorgensen Knut, Ro Oyvind Eating patterns in adolescents with type 1 diabetes: Associations with metabolic control, insulin omission, and eating disorder pathology. APPETITE. 2017;114():226-231.

2104. Mitchell Karen, Mazzeo Suzanne, Rausch Sarah, Cooke Kathryn Innovative interventions for disordered eating: Evaluating dissonance-based and yoga interventions. INTERNATIONAL JOURNAL OF EATING DISORDERS. 2007;40(2):120-128.

2105. Loureiro Lais, Almeida Luciene, Machado Carla, Pessoa Milene, Duarte Maria, Franceschini Sylvia, Ribeiro Andreia Food Consumption and Characteristics Associated in a Brazilian Older Adult Population: A Cluster Analysis. FRONTIERS IN NUTRITION. 2021;8():.

2106. Peters Jacqueline, Parletta Natalie, Lynch John, Campbell Karen A comparison of parental views of their pre-school children's `healthy' versus `unhealthy' diets. A qualitative study. APPETITE. 2014;76():129-136.

2107. Simone Melissa, Long Emily, Lockhart Ginger The Dynamic Relationship between Unhealthy Weight Control and Adolescent Friendships: A Social Network Approach. JOURNAL OF YOUTH AND ADOLESCENCE. 2018;47(7):1373-1384.

2108. Schwebel David Adolescent Tanning, Disordered Eating, and Risk Taking. JOURNAL OF DEVELOPMENTAL AND BEHAVIORAL PEDIATRICS. 2014;35(3):225-227.

2109. Alantar Zeynep, Maner Fulya Eating disorders in the context of attachment theory. ANADOLU PSIKIYATRI DERGISI-ANATOLIAN JOURNAL OF PSYCHIATRY. 2008;9(2):97-104.

2110. Dzombak Jesse, Haynos Ann, Rienecke Renee, Van Huysse Jessica Brief report: Differences in nonsuicidal self-injury according to binge eating and purging status in an

adolescent sample seeking eating disorder treatment. EATING BEHAVIORS. 2020;37():.

2111. Jacobi Corinna, Morris Lisette, Beckers Christina, Bronisch-Holtze Janina, Winter Jana, Winzelberg Andrew, Taylor Craig Maintenance of Internet-based prevention: A randomized controlled trial. INTERNATIONAL JOURNAL OF EATING DISORDERS. 2007;40(2):114-119.

2112. Hoek Hans Incidence, prevalence and mortality of anorexia nervosa and other eating disorders. CURRENT OPINION IN PSYCHIATRY. 2006;19(4):389-394.

2113. Lee Kirsty, Vaillancourt Tracy Longitudinal Associations Among Bullying by Peers, Disordered Eating Behavior, and Symptoms of Depression During Adolescence. JAMA PSYCHIATRY. 2018;75(6):605-612.

2114. Hahn Samantha, Bauer Katherine, Kaciroti Niko, Eisenberg Daniel, Lipson Sarah, Sonneville Kendrin Relationships between patterns of weight-related self-monitoring and eating disorder symptomology among undergraduate and graduate students. INTERNATIONAL JOURNAL OF EATING DISORDERS. 2021;54(4):595-605.

2115. Coleman Paul, Hanson Petra, Rens Thijs, Oyebode Oyinlola A rapid review of the evidence for children's TV and online advertisement restrictions to fight obesity. PREVENTIVE MEDICINE REPORTS. 2022;26():.

2116. Milano Walter, Ambrosio Paola, Carizzone Francesa, De Biasio Valeria, Foggia Giuseppina, Capasso Anna Gender Dysphoria, Eating Disorders and Body Image: An Overview. ENDOCRINE METABOLIC \& IMMUNE DISORDERS-DRUG TARGETS. 2020;20(4):518-524.

2117. Nichols JF, Rauh MJ, Lawson MJ, Ji M, Barkai HS Prevalence of the female athlete triad syndrome among high school athletes. ARCHIVES OF PEDIATRICS \& ADOLESCENT MEDICINE. 2006;160(2):137-142.

2118. Bleck Jennifer, DeBate Rita, Levin Bruce, Baldwin Julie Underlying Mechanisms and Trajectory of Comorbid ADHD and Eating Disorders: Proposing an Innovative Systems Framework for Informing Research. INTERNATIONAL JOURNAL OF MENTAL HEALTH AND ADDICTION. 2016;14(4):449-458.

2119. Le Heuzey MF, Mouren-Simeoni MC Does bulimia nervosa exist in children?. ANNALES MEDICO-PSYCHOLOGIQUES. 1999;157(10):717-720.

2120. Fitzsimmons-Craft Ellen Eating disorder-related social comparison in college women's everyday lives. INTERNATIONAL JOURNAL OF EATING DISORDERS. 2017;50(8):893-905.

2121. Valenciano-Mendoza Eduardo, Fernandez-Aranda Fernando, Granero Roser, Vintro-Alcaraz Cristina, Mora-Maltas Bernat, Valero-Solis Susana, Sanchez Isabel, Toro Jessica, Gomez-Pena Monica, Moragas Laura, Jimenez-Murcia Susana Common and differential risk factors behind suicidal behavior in patients with impulsivity- related disorders: The case of bulimic spectrum eating disorders and gambling disorder. JOURNAL OF BEHAVIORAL ADDICTIONS. 2022;11(4):963-978.

2122. Roehrig Megan, Masheb Robin, White Marney, Rothschild Bruce, Burke-Martindale Carolyn, Grilo Carlos Chronic Dieting Among Extremely Obese Bariatric Surgery Candidates. OBESITY SURGERY. 2009;19(8):1116-1123.

2123. Cibralic Sara, Conti Janet ``I'm More Acknowledged{''}: A Qualitative Exploration of Men's Positioning of their Body Image, Eating, and Exercise Concerns. JOURNAL OF CONSTRUCTIVIST PSYCHOLOGY. 2018;31(2):186-205.

2124. Mubarak Naser, Wynn Austin, Tapasak Brandon, Collins Andrew, Mubarak Norah, Gonzalez Carla, Marino Gustavo, Mayne Stephanie An examination of temporal trends in health behaviors among United States children, adolescents, and adults from 2005 to 2016. JOURNAL OF PUBLIC HEALTH RESEARCH. 2022;11(2):.

2125. Medisauskaite Asta, Kamau Caroline Does occupational distress raise the risk of alcohol use, binge-eating, ill health and sleep problems among medical doctors? A UK cross-sectional study. BMJ OPEN. 2019;9(5):.

2126. Allen Karina, Fursland Anthea, Watson Hunna, Byrne Susan Eating disorder diagnoses in general practice settings: Comparison with structured clinical interview and self-report questionnaires. JOURNAL OF MENTAL HEALTH. 2011;20(3):270-280.

2127. Balodis Iris, Kober Hedy, Worhunsky Patrick, White Marney, Stevens Michael, Pearlson Godfrey, Sinha Rajita, Grilo Carlos, Potenza Marc Monetary Reward Processing in Obese Individuals With and Without Binge Eating Disorder. BIOLOGICAL PSYCHIATRY.

2013;73(9):877-886.

2128. Zutven K., Mond J., Latner J., Rodgers B. Obesity and psychosocial impairment: mediating roles of health status, weight/shape concerns and binge eating in a community sample of women and men. INTERNATIONAL JOURNAL OF OBESITY. 2015;39(2):346-352.

2129. Sanchez-Carracedo David, Lopez-Guimera Gemma, Fauquet Jordi, Ramon Barrada Juan, Pamias Montserrat, Punti Joaquim, Querol Mireia, Trepat Esther A school-based program implemented by community providers previously trained for the prevention of eating and weight-related problems in secondary-school adolescents: the MABIC study protocol. BMC PUBLIC HEALTH. 2013;13():.

2130. Blackwell Donald, Becker Carolyn, Bermudez Ovidio, Berrett Michael, Brooks Gayle, Bunnell Douglas, Cabrera Dena, Costin Carolyn, Hemendinger Nancy, Johnson Craig, Klump Kelly, Levinson Cheri, Lutter Michael, Maine Margo, McAdams Carrie, McGilley Beth, Murray Stuart, Myers Elissa, Ouellette J., Peat Christine, Saffran Kristina, Setliff Stephanie The legacy of hope summit: a consensus-based initiative and report on eating disorders in the US and recommendations for the path forward. JOURNAL OF EATING DISORDERS. 2021;9(1):.

2131. Pascale Esterina, Cimino Silvia, Cerniglia Luca, Bevilacqua Arturo Disordered eating in early childhood: DRD4 and DAT1 gene polymorphisms and quality of mother-child interaction. EATING AND WEIGHT DISORDERS-STUDIES ON ANOREXIA BULIMIA AND OBESITY. 2022;27(7):2605-2616.

2132. Wentz Elisabet, Gillberg I., Anckarsater Henrik, Gillberg Christopher, Rastam Maria Adolescent-onset anorexia nervosa: 18-year outcome. BRITISH JOURNAL OF PSYCHIATRY. 2009;194(2):168-174.

2133. Long Michael, Ward Zachary, Wright Davene, Rodriguez Patricia, Tefft Nathan, Austin S. Cost-Effectiveness of 5 Public Health Approaches to Prevent Eating Disorders. AMERICAN JOURNAL OF PREVENTIVE MEDICINE. 2022;63(6):935-943.

2134. Rohde Paul, Desjardins Christopher, Arigo Danielle, Shaw Heather, Stice Eric Mediators of two selective prevention interventions targeting both obesity and eating disorders. BEHAVIOUR RESEARCH AND THERAPY. 2018;106():8-17.

2135. Cook-Cottone Catherine, Talebkhah Kellie, Guyker Wendy, Keddie Emily A controlled trial of a yoga-based prevention program targeting eating disorder risk factors among

middle school females. EATING DISORDERS. 2017;25(5):392-405.

2136. Numata Noriko, Nakagawa Akiko, Yoshioka Kazuko, Isomura Kayoko, Matsuzawa Daisuke, Setsu Rikukage, Nakazato Michiko, Shimizu Eiji Associations between autism spectrum disorder and eating disorders with and without self-induced vomiting: an empirical study. JOURNAL OF EATING DISORDERS. 2021;9(1):.

2137. Colton P, Olmsted M, Daneman D, Rydall A, Rodin G Disturbed eating disorders behavior and eating disorders in preteen and early teenage girls with type 1 diabetes - A case-controlled study. DIABETES CARE. 2004;27(7):1654-1659.

2138. Giuliano Chiara, Robbins Trevor, Nathan Pradeep, Bullmore Edward, Everitt Barry Inhibition of Opioid Transmission at the mu-Opioid Receptor Prevents Both Food Seeking and Binge-Like Eating. NEUROPSYCHOPHARMACOLOGY. 2012;37(12):2643-2652.

2139. Garriz Miguel, Andres-Perpina Susana, Teresa Plana Maria, Flamarique Itziar, Romero Sonia, Julia Laia, Castro-Fornieles Josefina Personality disorder traits, obsessive ideation and perfectionism 20 years after adolescent-onset anorexia nervosa: a recovered study. EATING AND WEIGHT DISORDERS-STUDIES ON ANOREXIA BULIMIA AND OBESITY. 2021;26(2):667-677.

2140. Carroll-Scott Amy, Gilstad-Hayden Kathryn, Rosenthal Lisa, Peters Susan, McCaslin Catherine, Joyce Rebecca, Ickovics Jeannette Disentangling neighborhood contextual associations with child body mass index, diet, and physical activity: The role of built, socioeconomic, and social environments. SOCIAL SCIENCE \& MEDICINE. 2013;95(SI):106-114.

2141. Rosenberger Patricia, Henderson Kathryn, Grilo Carlos Correlates of body image dissatisfaction in extremely obese female bariatric surgery candidates. OBESITY SURGERY. 2006;16(10):1331-1336.

2142. Tiggemann Marika Objectification Theory: Of relevance for eating disorder researchers and clinicians?. CLINICAL PSYCHOLOGIST. 2013;17(2):35-45.

2143. Weinberger-Litman Sarah, Rabin Laura, Fogel Joshua, Mensinger Janell, Litman Leib Psychosocial Mediators of the Relationship Between Religious Orientation and Eating Disorder Risk Factors in Young Jewish Women. PSYCHOLOGY OF RELIGION AND

SPIRITUALITY. 2016;8(4):265-276.

2144. Osborne Emma, Ainsworth Ben, Chadwick Paul, Atkinson Melissa The role of emotion regulation in the relationship between mindfulness and risk factors for disordered eating: A longitudinal mediation analysis. INTERNATIONAL JOURNAL OF EATING DISORDERS. 2022;():.

2145. Barry Mikayla, Sonneville Kendrin, Leung Cindy Students with Food Insecurity Are More Likely to Screen Positive for an Eating Disorder at a Large, Public University in the Midwest. JOURNAL OF THE ACADEMY OF NUTRITION AND DIETETICS. 2021;121(6):1115-1124.

2146. Dong D., Lei X., Jackson T., Wang Y., Su Y., Chen H. ALTERED REGIONAL HOMOGENEITY AND EFFICIENT RESPONSE INHIBITION IN RESTRAINED EATERS. NEUROSCIENCE. 2014;266():116-126.

2147. Zipfel S, Seibel MJ, Lowe B, Beumont PJ, Kasperk C, Herzog W Osteoporosis in eating disorders: A follow-up study of patients with anorexia and bulimia nervosa. JOURNAL OF CLINICAL ENDOCRINOLOGY \& METABOLISM. 2001;86(11):5227-5233.

2148. Smith Allison, Gay Jennifer, Arent Shawn, Sarzynski Mark, Emerson Dawn, Torres-McGehee Toni Examination of the Prevalence of Female Athlete Triad Components among Competitive Cheerleaders. INTERNATIONAL JOURNAL OF ENVIRONMENTAL RESEARCH AND PUBLIC HEALTH. 2022;19(3):.

2149. Mitchell Anne, Bulik Cynthia Eating disorders and women's health: An update. JOURNAL OF MIDWIFERY \& WOMENS HEALTH. 2006;51(3):193-201.

2150. STEINHAUSEN HC, SEIDEL R CORRESPONDENCE BETWEEN THE CLINICAL-ASSESSMENT OF EATING-DISORDERED PATIENTS AND FINDINGS DERIVED FROM QUESTIONNAIRES AT FOLLOW-UP. INTERNATIONAL JOURNAL OF EATING DISORDERS. 1993;14(3):367-374.

2151. Hart Laura, Damiano Stephanie, Paxton Susan Confident Body, Confident Child: A Randomized Controlled Trial Evaluation of a Parenting Resource for Promoting Healthy Body Image and Eating Patterns in 2-to 6-Year-Old Children. INTERNATIONAL JOURNAL OF EATING DISORDERS. 2016;49(5):458-472.

2152. Voelker Ulrike, Jacobi Corinna, Trockel Mickey, Taylor C. Moderators and mediators of outcome in Internet-based indicated prevention for eating disorders. BEHAVIOUR RESEARCH AND THERAPY. 2014;63():114-121.

2153. Gooding Holly, Cheever Elizabeth, Forman Sara, Hatoun Jonathan, Jooma Farah, Touloumtzis Currie, Vernacchio Louis Implementation and Evaluation of Two Educational Strategies to Improve Screening for Eating Disorders in Pediatric Primary Care. JOURNAL OF ADOLESCENT HEALTH. 2017;60(5):606-611.

2154. Becker CB, Smith LM, Ciao AC Reducing eating disorder risk factors in sorority members: A randomized trial. BEHAVIOR THERAPY. 2005;36(3):245-253.

2155. Erinosho Temitope, Hales Derek, McWilliams Christina, Emunah Josie, Ward Dianne Nutrition Policies at Child-Care Centers and Impact on Role Modeling of Healthy Eating Behaviors of Caregivers. JOURNAL OF THE ACADEMY OF NUTRITION AND DIETETICS. 2012;112(1):119-124.

2156. Chua Sook, Fitzsimmons-Craft Ellen, Austin S., Wilfley Denise, Taylor C. Estimated prevalence of eating disorders in Malaysia based on a diagnostic screen. INTERNATIONAL JOURNAL OF EATING DISORDERS. 2022;55(6):763-775.

2157. Goldschmidt Andrea, Smith Kathryn, Lavender Jason, Engel Scott, Haedt-Matt Alissa Trait-level facets of impulsivity and momentary, naturalistic eating behavior in children and adolescents with overweight/obesity. JOURNAL OF PSYCHIATRIC RESEARCH. 2019;110():24-30.

2158. Black Donald Compulsive buying disorder: A review of the evidence.. CNS SPECTRUMS. 2007;12(2):124-132.

2159. Liu Qing, Guo Xia-Nan, Liu Cai-Yan, Xu Wei-Hai A proposed synergistic effect of CSF1R and NMUR2 variants contributes to binge eating in hereditary diffuse leukoencephalopathy with spheroids. ANNALS OF TRANSLATIONAL MEDICINE. 2020;8(1, SI):.

2160. Landry Alicia, Madson Michael, Mohn Richard, Nicholson Bonnie Development and Psychometric Evaluation of the College Eating and Drinking Behaviors Scale in US College Students. INTERNATIONAL JOURNAL OF MENTAL HEALTH AND ADDICTION. 2017;15(3):485-492.

2161. Yu Zhiping, Indelicato Natalie, Fuglestad Paul, Tan Michael, Bane Lindsay, Stice Caitlin Sex differences in disordered eating and food addiction among college students. APPETITE. 2018;129():12-18.

2162. Jacobs M., Roesch S., Wonderlich S., Crosby R., Thornton L., Wilfley D., Berrettini W., Brandt H., Crawford S., Fichter M., Halmi K., Johnson C., Kaplan A., LaVia M., Mitchell J., Rotondo A., Strober M., Woodside D., Kaye W., Bulik C. Anorexia nervosa trios: behavioral profiles of individuals with anorexia nervosa and their parents. PSYCHOLOGICAL MEDICINE. 2009;39(3):451-461.

2163. Bulik CM, Sullivan PF, Carter FA, Joyce PR Lifetime anxiety disorders in women with bulimia nervosa. COMPREHENSIVE PSYCHIATRY. 1996;37(5):368-374.

2164. Meydanlioglu Ayse, Ergun Ayse The Effect of Nurse-Led Diet and Physical Activity Program for Health on Diet and Physical Activity Behavior of Children. JOURNAL OF PHYSICAL ACTIVITY \& HEALTH. 2019;16(7):504-511.

2165. Langdon-Daly Jasmin, Serpell Lucy Protective factors against disordered eating in family systems: a systematic review of research. JOURNAL OF EATING DISORDERS. 2017;5():.

2166. Nederkoorn C, Smulders F, Havermans R, Jansen A Exposure to bingefood in bulimia nervosa: finger pulse amplitude as a potential measure of urge to eat and predictor of food intake. APPETITE. 2004;42(2):125-130.

2167. Brausch Amy, Boone Shannon Frequency of Nonsuicidal Self-Injury in Adolescents: Differences in Suicide Attempts, Substance Use, and Disordered Eating. SUICIDE AND LIFE-THREATENING BEHAVIOR. 2015;45(5):612-622.

2168. Jeffers Amy, Mason Tyler, Benotsch Eric Psychological eating factors, affect, and ecological momentary assessed diet quality. EATING AND WEIGHT DISORDERS-STUDIES ON ANOREXIA BULIMIA AND OBESITY. 2020;25(5):1151-1159.

2169. Siu Judy, Chan Kara, Lee Albert Adolescents from low-income families in Hong Kong and unhealthy eating behaviours: Implications for health and social care practitioners. HEALTH \& SOCIAL CARE IN THE COMMUNITY. 2019;27(2):366-374.

2170. Huang Li-Chung, Tsai Kuen-Jer, Wang Hao-Kuang, Sung Pi-Shan, Wu Ming-Hsiu, Hung Kuo-Wei, Lin Sheng-Hsiang Prevalence, incidence, and comorbidity of clinically diagnosed obsessive-compulsive disorder in Taiwan: A national population-based study. PSYCHIATRY RESEARCH. 2014;220(1-2):335-341.

2171. Ravi Suvi, Ihalainen Johanna, Taipale-Mikkonen Ritva, Kujala Urho, Waller Benjamin, Mierlahti Laura, Lehto Johanna, Valtonen Maarit Self-Reported Restrictive Eating, Eating Disorders, Menstrual Dysfunction, and Injuries in Athletes Competing at Different Levels and Sports. NUTRIENTS. 2021;13(9):.

2172. KILLEN JD, TAYLOR CB, HAYWARD C, WILSON DM, HAYDEL KF, HAMMER LD, SIMMONDS B, ROBINSON TN, LITT I, VARADY A, KRAEMER H PURSUIT OF THINNESS AND ONSET OF EATING DISORDER SYMPTOMS IN A COMMUNITY SAMPLE OF ADOLESCENT GIRLS - A 3-YEAR PROSPECTIVE ANALYSIS. INTERNATIONAL JOURNAL OF EATING DISORDERS. 1994;16(3):227-238.

2173. SUZUKI K, TAKEDA A, MATSUSHITA S COPREVALENCE OF BULIMIA WITH ALCOHOL-ABUSE AND SMOKING AMONG JAPANESE MALE AND FEMALE HIGH-SCHOOL-STUDENTS. ADDICTION. 1995;90(7):971-975.

2174. Ball K, Lee C Relationships between psychological stress, coping and disordered eating: A review. PSYCHOLOGY \& HEALTH. 1999;14(6):1007-1035.

2175. Berger Sarah, Elliott Camden, Ranzenhofer Lisa, Shomaker Lauren, Hannallah Louise, Field Sara, Young Jami, Sbrocco Tracy, Wilfley Denise, Yanovski Jack, Tanofsky-Kraff Marian Interpersonal problem areas and alexithymia in adolescent girls with loss of control eating. COMPREHENSIVE PSYCHIATRY. 2014;55(1):170-178.

2176. Fernandez ML, Otero MC, Castro YR, Prieto MF Eating disorders in spanish university students. ACTAS ESPANOLAS DE PSIQUIATRIA. 2002;30(6):343-349.

2177. Levitan Robert, Davis Caroline Emotions and Eating Behaviour: Implications for the Current Obesity Epidemic. UNIVERSITY OF TORONTO QUARTERLY. 2010;79(2, SI):783-799.

2178. Sorbara M, Geliebter A Body image disturbance in obese outpatients before and after weight loss in relation to race, gender, binge eating, and age of onset of obesity.

INTERNATIONAL JOURNAL OF EATING DISORDERS. 2002;31(4):416-423.

2179. McGowan Andrea, Barry Mikayla, Sonneville Kendrin, Leung Cindy Greater social adversity associated with more disordered eating attitudes and behaviors among children from Southeast Michigan. INTERNATIONAL JOURNAL OF EATING DISORDERS. 2022;55(12):1670-1677.

2180. Levinson Cheri, Sala Margarita, Murray Stuart, Ma Jackie, Rodebaugh Thomas, Lenze Eric Diagnostic, clinical, and personality correlates of food anxiety during a food exposure in patients diagnosed with an eating disorder. EATING AND WEIGHT DISORDERS-STUDIES ON ANOREXIA BULIMIA AND OBESITY. 2019;24(6):1079-1088.

2181. Landstedt Evelina, Hammarstrom Anne, Fairweather-Schmidt A., Wade Tracey Associations between adolescent risk for restrictive disordered eating and long-term outcomes related to somatic symptoms, body mass index, and poor well-being. BRITISH JOURNAL OF HEALTH PSYCHOLOGY. 2018;23(2):496-518.

2182. Ioannidis Konstantinos, Serfontein Jaco, Mueller Ulrich Bulimia nervosa patient diagnosed with previously unsuspected ADHD in adulthood: Clinical case report, literature review, and diagnostic challenges. INTERNATIONAL JOURNAL OF EATING DISORDERS. 2014;47(4):431-436.

2183. Abraham S Obstetricians and maternal body weight and eating disorders during pregnancy. JOURNAL OF PSYCHOSOMATIC OBSTETRICS \& GYNECOLOGY. 2001;22(3):159-163.

2184. Deckelman MC, Dixon LB, Conley RR Comorbid bulimia nervosa and schizophrenia. INTERNATIONAL JOURNAL OF EATING DISORDERS. 1997;22(1):101-105.

2185. Patton GC, Selzer R, Coffey C, Carlin JB, Wolfe R Onset of adolescent eating disorders: population based cohort study over 3 years. BMJ-BRITISH MEDICAL JOURNAL. 1999;318(7186):765-768.

2186. Gomez-Martinez Carlos, Babio Nancy, Julvez Jordi, Nishi Stephanie, Fernandez-Aranda Fernando, Angel Martinez-Gonzalez Miguel, Cuenca-Royo Aida, Fernandez Rebeca, Jimenez-Murcia Susana, Torre Rafael, Pinto Xavier, Bloemendaal Mirjam, Fito Montse, Corella Dolores, Arias Alejandro, Salas-Salvado Jordi Impulsivity is longitudinally associated with healthy and unhealthy dietary patterns in individuals with overweight or obesity and

metabolic syndrome within the framework of the PREDIMED-Plus trial. INTERNATIONAL JOURNAL OF BEHAVIORAL NUTRITION AND PHYSICAL ACTIVITY. 2022;19(1):.

2187. Penas-Lledo E, Fernandez JD, Waller G Association of anger with bulimic and other impulsive behaviours among non-clinical women and men. EUROPEAN EATING DISORDERS REVIEW. 2004;12(6):392-397.

2188. Smith Kathryn, Mason Tyler Psychiatric comorbidity associated with weight status in 9 to 10 year old children. PEDIATRIC OBESITY. 2022;17(5):.

2189. Hall Katherine, Hoerster Katherine, Yancy William Post-Traumatic Stress Disorder, Physical Activity, and Eating Behaviors. EPIDEMIOLOGIC REVIEWS. 2015;37(1):103-115.

2190. Robson Jacob, Laborda Trevor, Fitzgerald Susan, Andersen Joseph, Peterson Kathryn, O'Gorman Molly, Guthery Stephen, Bennett-Murphy Laura Avoidant/Restrictive Food Intake Disorder in Diet-treated Children With Eosinophilic Esophagitis. JOURNAL OF PEDIATRIC GASTROENTEROLOGY AND NUTRITION. 2019;69(1):57-60.

2191. Monteleone P., Scognamiglio P., Canestrelli B., Serino I., Monteleone A., Maj M. Asymmetry of salivary cortisol and alpha-amylase responses to psychosocial stress in anorexia nervosa but not in bulimia nervosa. PSYCHOLOGICAL MEDICINE. 2011;41(9):1963-1969.

2192. Redondo Iratxe, Luyten Patrick Alexithymia Mediates the Relationship Between Insecure Attachment and Eating Disorder Symptoms. JOURNAL OF RATIONAL-EMOTIVE AND COGNITIVE-BEHAVIOR THERAPY. 2021;39(4):491-508.

2193. Eichen Dawn, Matheson Brittany, Appleton-Knapp Sara, Boutelle Kerri Neurocognitive Treatments for Eating Disorders and Obesity. CURRENT PSYCHIATRY REPORTS. 2017;19(9):.

2194. Shank Lisa, Crosby Ross, Grammer Anne, Shomaker Lauren, Vannucci Anna, Burke Natasha, Stojek Monika, Brady Sheila, Kozlosky Merel, Reynolds James, Yanovski Jack, Tanofsky-Kraff Marian Examination of the interpersonal model of loss of control eating in the laboratory. COMPREHENSIVE PSYCHIATRY. 2017;76():36-44.

2195. Watson Ryan, VanKim Nicole, Rose Hilary, Porta Carolyn, Gahagan Jacqueline, Eisenberg Marla Unhealthy weight control behaviors among youth: Sex of sexual partner is linked to important differences. EATING DISORDERS. 2018;26(5):448-463.

2196. Messer Mariel, Liu Claudia, McClure Zoe, Mond Jasmin, Tiffin Catherine, Linardon Jake Negative body image components as risk factors for orthorexia nervosa: Prospective findings. APPETITE. 2022;178():.

2197. Bueno B., Krug I., Bulik C., Jimenez-Murcia S., Granero R., Thornton L., Penelo E., Menchon J., Sanchez I., Tinahones F., Fernandez-Aranda F. Late Onset Eating Disorders in Spain: Clinical Characteristics and Therapeutic Implications. JOURNAL OF CLINICAL PSYCHOLOGY. 2014;70(1):1-17.

2198. Yaryura-Tobias JA, Grunes MS, Todaro J, McKay D, Neziroglu FA, Stockman R Nosological insert ion of Axis I disorders in the etiology of obsessive-compulsive disorder. JOURNAL OF ANXIETY DISORDERS. 2000;14(1):19-30.

2199. Boggiano M., Wenger L., Turan B., Tatum M., Morgan P., Sylvester M. Eating tasty food to cope. Longitudinal association with BMI. APPETITE. 2015;87():365-370.

2200. WARD A, TROOP N, CACHIA M, WATKINS P, TREASURE J ORIGINAL ARTICLES - DOUBLY DISABLED - DIABETES IN COMBINATION WITH AN EATING DISORDER. POSTGRADUATE MEDICAL JOURNAL. 1995;71(839):546-550.

2201. Akbari Mehdi, Seydavi Mohammad, Zamani Elahe, Griffiths Mark The risk of exercise addiction mediates the relationship between social media use and mental health indices among young Iranians. ADDICTION RESEARCH \& THEORY. 2022;():.

2202. Mandy Will, Tchanturia Kate Do women with eating disorders who have social and flexibility difficulties really have autism? A case series. MOLECULAR AUTISM. 2015;6():.

2203. Stice E, Presnell K, Spangler D Risk factors for binge eating onset in adolescent girls: A 2-year prospective investigation. HEALTH PSYCHOLOGY. 2002;21(2):131-138.

2204. Cena Hellas, Vandoni Matteo, Magenes Vittoria, Di Napoli Ilaria, Marin Luca, Baldassarre Paola, Luzzi Alessia, De Pasquale Francesca, Zuccotti Gianvincenzo, Calcaterra Valeria Benefits of Exercise in Multidisciplinary Treatment of Binge Eating Disorder in

Adolescents with Obesity. INTERNATIONAL JOURNAL OF ENVIRONMENTAL RESEARCH AND PUBLIC HEALTH. 2022;19(14):.

2205. Bodell Lindsay, Joiner Thomas, Ialongo Nicholas Longitudinal Association Between Childhood Impulsivity and Bulimic Symptoms in African American Adolescent Girls. JOURNAL OF CONSULTING AND CLINICAL PSYCHOLOGY. 2012;80(2):313-316.

2206. Areemit Rosawan, Katzman Debra, Pinhas Leora, Kaufman Miriam The Experience of Siblings of Adolescents With Eating Disorders. JOURNAL OF ADOLESCENT HEALTH. 2010;46(6):569-576.

2207. Mills Jessica, Thomas Susan, Larkin Theresa, Pai Nagesh, Deng Chao Problematic eating behaviours, changes in appetite, and weight gain in Major Depressive Disorder: The role of leptin. JOURNAL OF AFFECTIVE DISORDERS. 2018;240():137-145.

2208. Oliveira Tadeu, Goncalves Bruno, Oliveira Bruna, Oliveira Antonio, Reis Helton, Ferreira Claudia, Aguiar Daniele, Miranda Aline, Ribeiro Fabiola, Vieira Erica, Palotas Andras, Vieira Luciene Negative Modulation of the Metabotropic Glutamate Receptor Type 5 as a Potential Therapeutic Strategy in Obesity and Binge-Like Eating Behavior. FRONTIERS IN NEUROSCIENCE. 2021;15():.

2209. SKODOL AE, OLDHAM JM, HYLER SE, KELLMAN HD, DOIDGE N, DAVIES M COMORBIDITY OF DSM-III-R EATING DISORDERS AND PERSONALITY-DISORDERS. INTERNATIONAL JOURNAL OF EATING DISORDERS. 1993;14(4):403-416.

2210. Jackson Todd, Chen Hong Risk Factors for Disordered Eating During Early and Middle Adolescence: A Two Year Longitudinal Study of Mainland Chinese Boys and Girls. JOURNAL OF ABNORMAL CHILD PSYCHOLOGY. 2014;42(5):791-802.

2211. Burnette C., Simpson Courtney, Mazzeo Suzanne Exploring gender differences in the link between weight suppression and eating pathology. EATING BEHAVIORS. 2017;27():17-22.

2212. Olmsted MP, Daneman D, Rydall AC, Lawson ML, Rodin G The effects of psychoeducation on disturbed eating attitudes and behavior in young women with type 1 diabetes mellitus. INTERNATIONAL JOURNAL OF EATING DISORDERS. 2002;32(2):230-239.

2213. Olmsted Marion, Colton Patricia, Daneman Denis, Rydall Anne, Rodin Gary Prediction of the Onset of Disturbed Eating Behavior in Adolescent Girls With Type 1 Diabetes. DIABETES CARE. 2008;31(10):1978-1982.

2214. Lewis Sarah, Katsikitis Mary, Mulgrew Kate Like mother, like daughter? An examination of the emotive responses to food. JOURNAL OF HEALTH PSYCHOLOGY. 2015;20(6, SI):828-838.

2215. Hauck Carolin, Schipfer Melanie, Ellrott Thomas, Cook Brian The relationship between food addiction and patterns of disordered eating with exercise dependence: in amateur endurance athletes. EATING AND WEIGHT DISORDERS-STUDIES ON ANOREXIA BULIMIA AND OBESITY. 2020;25(6):1573-1582.

2216. Linardon Jake, Greenwood Christopher, Macdonald Jacqui, Spry Elizabeth, Wertheim Eleanor, Le Grange Daniel, Letcher Primrose, Olsson Craig Eating and Body Image Disturbances in Adolescence and Substance Use Throughout Young Adulthood: Findings from the Australian Temperament Project. INTERNATIONAL JOURNAL OF MENTAL HEALTH AND ADDICTION. 2023;():.

2217. Erzse Agnes, Rwafa-Ponela Teurai, Kruger Petronell, Wayas Feyisayo, Lambert Estelle, Mapa-Tassou Clarisse, Ngwa Edwin, Goldstein Susan, Foley Louise, Hofman Karen, Teguia Stephanie, Oni Tolu, Assah Felix, Shung-King Maylene, Abdool Karim Safura A Mixed-Methods Participatory Intervention Design Process to Develop Intervention Options in Immediate Food and Built Environments to Support Healthy Eating and Active Living among Children and Adolescents in Cameroon and South Africa. INTERNATIONAL JOURNAL OF ENVIRONMENTAL RESEARCH AND PUBLIC HEALTH. 2022;19(16):.

2218. Alhaj Omar, Fekih-Romdhane Feten, Sweidan Dima, Saif Zahra, Khudhair Mina, Ghazzawi Hadeel, Nadar Mohammed, Alhajeri Saad, Levine Michael, Jahrami Haitham The prevalence and risk factors of screen-based disordered eating among university students: a global systematic review, meta-analysis, and meta-regression. EATING AND WEIGHT DISORDERS-STUDIES ON ANOREXIA BULIMIA AND OBESITY. 2022;27(8):3215-3243.

2219. Richson Brianne, Forbush Kelsie, Chapa Danielle, Gould Sara, Perko Victoria, Johnson Sarah, Christensen Kara, Swanson Trevor, Tregarthen Jenna Measurement invariance of the Eating Pathology Symptoms Inventory (EPSI) in adolescents and adults. EATING BEHAVIORS. 2021;42():.

2220. Patterson-Norrie Tiffany, Ramjan Lucie, Sousa Mariana, Sank Lindy, George Ajesh Eating disorders and oral health: a scoping review on the role of dietitians. JOURNAL OF EATING DISORDERS. 2020;8(1):.

2221. Schroeder Mariana, Jakovcevski Mira, Polacheck Tamar, Lebow Maya, Drori Yonat, Engel Mareen, Ben-Dor Shifra, Chen Alon A Methyl-Balanced Diet Prevents CRF-Induced Prenatal Stress-Triggered Predisposition to Binge Eating-like Phenotype. CELL METABOLISM. 2017;25(6):1269+.

2222. Coutinho Joana, Ramos Ana, Maia Liliana, Castro Liliana, Conceicao Eva, Geliebter Allan, Machado Paulo, Goncalves Oscar, Sampaio Adriana Volumetric Alterations in the Nucleus Accumbens and Caudate Nucleus in Bulimia Nervosa: A Structural Magnetic Resonance Imaging Study. INTERNATIONAL JOURNAL OF EATING DISORDERS. 2015;48(2):206-214.

2223. Goddard Georgia, Oxlad Melissa Caring for individuals with Type 1 Diabetes Mellitus who restrict and omit insulin for weight control: Evidence-based guidance for healthcare professionals. DIABETES RESEARCH AND CLINICAL PRACTICE. 2022;185():.

2224. Santana M., Costa Ribeiro Junior H., Mora Giral M., Raich R. EPIDEMIOLOGY AND RISK FACTORS OF EATING DISORDER IN ADOLESCENCE; A REVIEW. NUTRICION HOSPITALARIA. 2012;27(2):391-401.

2225. Cuenca-Garcia Magdalena, Huybrechts Inge, Ruiz Jonatan, Ortega Francisco, Ottevaere Charlene, Gonzalez-Gross Marcela, Moreno Luis, Vicente-Rodriguez German, Molnar Denes, Polito Angela, Manios Yannis, Plada Maria, Vanhelst Jeremy, Widhalm Kurt, Sjostrom Michael, Kersting Mathilde, Castillo Manuel, Grp HELENA Clustering of Multiple Lifestyle Behaviors and Health-related Fitness in European Adolescents. JOURNAL OF NUTRITION EDUCATION AND BEHAVIOR. 2013;45(6):549-557.

2226. Thompson Carmen, Russell-Mayhew Shelly, Saraceni Reana Evaluating the Effects of a Peer-Support Model: Reducing Negative Body Esteem and Disordered Eating Attitudes and Behaviours in Grade Eight Girls. EATING DISORDERS. 2012;20(2):113-126.

2227. Boutelle Kerri, Peterson Carol, Crosby Ross, Rydell Sarah, Zucker Nancy, Harnack Lisa Overeating phenotypes in overweight and obese children. APPETITE. 2014;76():95-100.

2228. Saunokonoko A., Mars M., Sattmann-Frese W. The significance of the father-daughter relationship to understanding and treating Bulimia Nervosa: a Hermeneutic Phenomenological Study. COGENT PSYCHOLOGY. 2022;9(1):.

2229. Martinez-Cabrera Ileana, Gustavo Sierra-Gonzalez Victoriano, Maria Fajardo-Diaz Esther Gut microbiota and its relationship with opportunistic agents linked to epidemics. ACTA BIOQUIMICA CLINICA LATINOAMERICANA. 2021;55(3):319-345.

2230. CROSBY RD, MITCHELL JE, RAYMOND N, SPECKER S, NUGENT SM, PYLE RL SURVIVAL ANALYSIS OF RESPONSE TO GROUP-PSYCHOTHERAPY IN BULIMIA-NERVOSA. INTERNATIONAL JOURNAL OF EATING DISORDERS. 1993;13(4):359-368.

2231. Hilbert Anja, Staerk Christian, Stromer Annika, Mansfeld Thomas, Sander Johannes, Seyfried Florian, Kaiser Stefan, Dietrich Arne, Mayr Andreas Nonnormative Eating Behaviors and Eating Disorders and Their Associations With Weight Loss and Quality of Life During 6 Years Following Obesity Surgery. JAMA NETWORK OPEN. 2022;5(8):.

2232. Teasdale Scott, Latimer Geogina, Byron Annette, Schuldt Vanessa, Pizzinga Josephine, Plain Janice, Buttenshaw Kerryn, Forsyth Adrienne, Parker Elizabeth, Soh Nerissa Expanding collaborative care: integrating the role of dietitians and nutrition interventions in services for people with mental illness. AUSTRALASIAN PSYCHIATRY. 2018;26(1):47-49.

2233. McLean Sian, Caldwell Belinda, Roberton Michelle Reach Out and Recover: Intentions to seek treatment in individuals using online support for eating disorders. INTERNATIONAL JOURNAL OF EATING DISORDERS. 2019;52(10, SI):1137-1149.

2234. Galvao Patricia, Valente Juliana, Almeida Mireille, Gubert Fabiane, Reboucas Lidiane, Mari Jair, Caetano Sheila, Sanchez Zila Being bullied and using drugs are associate with eating disorder symptoms in Brazilian students. INTERNATIONAL JOURNAL OF EATING DISORDERS. 2021;54(3):445-450.

2235. Luo Yi-Jun, Jackson Todd, Stice Eric, Chen Hong Effectiveness of an Internet Dissonance-Based Eating Disorder Prevention Intervention Among Body-Dissatisfied Young Chinese Women. BEHAVIOR THERAPY. 2021;52(1):221-233.

2236. Cooley E, Toray T Body image and personality predictors of eating disorder symptoms during the college years. INTERNATIONAL JOURNAL OF EATING DISORDERS.

2001;30(1):28-36.

2237. Fulkerson JA, Sherwood NE, Perry CL, Neumark-Sztainer D, Story M Depressive symptoms and adolescent eating and health behaviors: a multifaceted view in a population-based sample. PREVENTIVE MEDICINE. 2004;38(6):865-875.

2238. Lahortiga-Ramos F, De Irala-Estevez J, Cano-Prous A, Gual-Garcia P, Martinez-Gonzalez MA, Cervera-Enguix S Incidence of eating disorders in Navarra (Spain). EUROPEAN PSYCHIATRY. 2005;20(2):179-185.

2239. Luykx Jurjen, Carpay Johannes Nervous system adverse responses to topiramate in the treatment of neuropsychiatric disorders. EXPERT OPINION ON DRUG SAFETY. 2010;9(4):623-631.

2240. Tanahashi Tokusei, Kawai Keisuke, Tatsushima Keita, Saeki Chihiro, Wakabayashi Kunie, Tamura Naho, Ando Tetsuya, Ishikawa Toshio Purging behaviors relate to impaired subjective sleep quality in female patients with anorexia nervosa: a prospective observational study. BIOPSYCHOSOCIAL MEDICINE. 2017;11():.

2241. Rolnik Ashley, Engeln-Maddox Renee, Miller Steven Here's Looking at You: Self-Objectification, Body Image Disturbance, and Sorority Rush. SEX ROLES. 2010;63(1-2):6-17.

2242. Musiat Peter, Moritz Steffen, Jacobi Corinna, Schmidt Ulrike Association Splitting: feasibility study of a novel technique to reduce weight and shape concerns. EATING AND WEIGHT DISORDERS-STUDIES ON ANOREXIA BULIMIA AND OBESITY. 2014;19(2):153-158.

2243. Wade Tracey, Byrne Susan, Bryant-Waugh Rachel The eating disorder examination: Norms and construct validity with young and middle adolescent girls. INTERNATIONAL JOURNAL OF EATING DISORDERS. 2008;41(6):551-558.

2244. Ruwaard Jeroen, Lange Alfred, Broeksteeg Janneke, Renteria-Agirre Aitziber, Schrieken Bart, Dolan Conor, Emmelkamp Paul Online Cognitive-Behavioural Treatment of Bulimic Symptoms: A Randomized Controlled Trial. CLINICAL PSYCHOLOGY \& PSYCHOTHERAPY. 2013;20(4):308-318.

2245. Schulze U, Neudorfl A, Krill A, Warnke A, Remschmidt H, HerpertzDahlmann B Early-onset anorexia nervosa: Course and outcome.. ZEITSCHRIFT FUR KINDER-UND JUGENDPSYCHIATRIE UND PSYCHOTHERAPIE. 1997;25(1):5-16.

2246. Mueller Manfred, Koertzinger Inga, Mast Mareike, Langnaese Kristina, Grund Andreas Physical activity and diet in 5 to 7 years old children. PUBLIC HEALTH NUTRITION. 1999;2(3A, S):443-444.

2247. Klump Kelly, Fowler Natasha, Mayhall Laura, Sisk Cheryl, Culbert K., Burt S. Estrogen Moderates Genetic Influences on Binge Eating During Puberty: Disruption of Normative Processes?. JOURNAL OF ABNORMAL PSYCHOLOGY. 2018;127(5):458-470.

2248. Lunde Anna, Fasmer Ole, Akiskal Kareen, Akiskal Hagop, Oedegaard Ketil The relationship of bulimia and anorexia nervosa with bipolar disorder and its temperamental foundations. JOURNAL OF AFFECTIVE DISORDERS. 2009;115(3):309-314.

2249. McElroy SL, Altshuler LL, Suppes T, Keck PE, Frye MA, Denicoff KD, Nolen WA, Kupka RW, Leverich GS, Rochussen JR, Rush AJ, Post RM Axis I psychiatric comorbidity and its relationship to historical illness variables in 288 patients with bipolar disorder. AMERICAN JOURNAL OF PSYCHIATRY. 2001;158(3):420-426.

2250. Quadflieg Norbert, Strobel Christine, Naab Silke, Voderholzer Ulrich, Fichter Manfred Mortality in males treated for an eating disorder-A large prospective study. INTERNATIONAL JOURNAL OF EATING DISORDERS. 2019;52(12, SI):1365-1369.

2251. Zibordi Federica, Giovanna Zorzi, Miryam Carecchio, Nardo Nardocci CANS: Childhood acute neuropsychiatric syndromes. EUROPEAN JOURNAL OF PAEDIATRIC NEUROLOGY. 2018;22(2, SI):316-320.

2252. Seiffge-Krenke Inge Eating Disorders: Developmental Psychological and Developmental Psychopathological Perspective. KINDHEIT UND ENTWICKLUNG. 2019;28(4):197-209.

2253. Maxwell Millie, Thornton Laura, Root Tammy, Pinheiro Andrea, Strober Michael, Brandt Harry, Crawford Steve, Crow Scott, Fichter Manfred, Halmi Katherine, Johnson Craig, Kaplan Allan, Keel Pamela, Klump Kelly, LaVia Maria, Mitchell James, Plotnicov Kathy, Rotondo Alessandro, Woodside D., Berrettini Wade, Kaye Walter, Bulik Cynthia Life Beyond the Eating Disorder: Education, Relationships, and Reproduction. INTERNATIONAL

JOURNAL OF EATING DISORDERS. 2011;44(3):225-232.

2254. Segura-Garcia Cristina, Aloi Matteo, Rania Marianna, Ciambrone Paola, Palmieri Antonella, Pugliese Valentina, Ruiz Moruno Antonio, De Fazio Pasquale Ability of EDI-2 and EDI-3 to correctly identify patients and subjects at risk for eating disorders. EATING BEHAVIORS. 2015;19():20-23.

2255. Pearson Carolyn, Riley Elizabeth, Davis Heather, Smith Gregory Research Review: Two pathways toward impulsive action: an integrative risk model for bulimic behavior in youth. JOURNAL OF CHILD PSYCHOLOGY AND PSYCHIATRY. 2014;55(8):852-864.

2256. Damiano Stephanie, Hart Laura, Paxton Susan Correlates of parental feeding practices with pre-schoolers: Parental body image and eating knowledge, attitudes, and behaviours. APPETITE. 2016;101():192-198.

2257. Graham Andrea, Trockel Mickey, Weisman Hannah, Fitzsimmons-Craft Ellen, Balantekin Katherine, Wilfley Denise, Taylor C. A screening tool for detecting eating disorder risk and diagnostic symptoms among college-age women. JOURNAL OF AMERICAN COLLEGE HEALTH. 2019;67(4):357-366.

2258. Meule Adrian, Schrambke Dominic, Furst Loredo Artur, Schlegl Sandra, Naab Silke, Voderholzer Ulrich Inpatient treatment of anorexia nervosa in adolescents: A 1-year follow-up study. EUROPEAN EATING DISORDERS REVIEW. 2021;29(2):165-177.

2259. Sierra-Baigrie Susana, Lemos-Giraldez Serafin, Paino Mercedes, Fonseca-Pedrero Eduardo Exploring the Relationship between Coping Strategies and Binge Eating in Nonclinical Adolescents. EUROPEAN EATING DISORDERS REVIEW. 2012;20(1):e63-e69.

2260. Pinto Anthony, Mancebo Maria, Eisen Jane, Pagano Maria, Rasmussen Steve The Brown longitudinal obsessive compulsive study: Clinical features and symptoms of the sample at intake. JOURNAL OF CLINICAL PSYCHIATRY. 2006;67(5):703-711.

2261. Loprinzi Paul, Smit Ellen, Mahoney Sara Physical Activity and Dietary Behavior in US Adults and Their Combined Influence on Health. MAYO CLINIC PROCEEDINGS. 2014;89(2):190-198.

2262. Schlegl Sandra, Hupe Kerstin, Hessler Johannes, Diedrich Alice, Huber Thomas, Rauh Elisabeth, Aita Stephen, Gaertner Thomas, Voderholzer Ulrich Pathways to Care and Duration of Untreated Illness of Inpatients with Anorexia and Bulimia nervosa. PSYCHIATRISCHE PRAXIS. 2019;46(6):342-348.

2263. Keel PK, Heatherton TF, Dorer DJ, Joiner TE, Zalta AK Point prevalence of bulimia nervosa in 1982, 1992, and 2002. PSYCHOLOGICAL MEDICINE. 2006;36(1):119-127.

2264. Miyamoto Etsuko, Okumura Yusuke, Maruo Kazushi, Kitani Seiichi Shoplifting Behavior Among Patients With an Eating Disorder at a Medical Correctional Center in Japan: A Cross-Sectional Study. FRONTIERS IN PSYCHIATRY. 2022;13():.

2265. Lloyd Naomi Mental health problems and eating disorders: a student teacher's perception of current challenges facing pastoral care in education. PASTORAL CARE IN EDUCATION. 2022;40(3, SI):297-309.

2266. ERGUN Y, PAYDAS S, SEYREK N, SEYREK E IS THERE ANY RELATIONSHIP BETWEEN PICA AND LIVER-FAILURE. BRITISH JOURNAL OF CLINICAL PRACTICE. 1993;47(3):147-149.

2267. ANDERSEN AE, WOODWARD PJ, LAFRANCE N BONE-MINERAL DENSITY OF EATING DISORDER SUBGROUPS. INTERNATIONAL JOURNAL OF EATING DISORDERS. 1995;18(4):335-342.

2268. Yates William, Lund Brian, Johnson Craig, Mitchell Jeff, McKee Patrick Attention-Deficit Hyperactivity Symptoms and Disorder in Eating Disorder Inpatients. INTERNATIONAL JOURNAL OF EATING DISORDERS. 2009;42(4):375-378.

2269. Hill Mary, Masuda Akihiko, Latzman Robert Body image flexibility as a protective factor against disordered eating behavior for women with lower body mass index. EATING BEHAVIORS. 2013;14(3):336-341.

2270. Hermanussen M., Tresguerres J. A new anti-obesity drug treatment: First clinical evidence that, antagonising glutamate-gated Ca2+ ion channels with memantine normalises binge-eating disorders. ECONOMICS \& HUMAN BIOLOGY. 2005;3(2):329-337.

2271. Hendricks PS, Thompson JK An integration of cognitive-behavioral therapy and interpersonal psychotherapy for bulimila nervosa: A case study using the case formulation method. INTERNATIONAL JOURNAL OF EATING DISORDERS. 2005;37(2):171-174.

2272. Ferretti Fabrizio Unhealthy Behaviours: An International Comparison. PLOS ONE. 2015;10(10):.

2273. Baigrie Susana, Giraldez Serafin Examining the relationship between binge eating and coping strategies and the definition of binge eating in a sample of Spanish adolescents. SPANISH JOURNAL OF PSYCHOLOGY. 2008;11(1):172-180.

2274. Bizri Maya, Geagea Luna, Kobeissy Firas, Talih Farid Prevalence of Eating Disorders Among Medical Students in a Lebanese Medical School: A Cross-Sectional Study. NEUROPSYCHIATRIC DISEASE AND TREATMENT. 2020;16():1879-1887.

2275. Takii Masato, Uchigata Yasuko, Kishimoto Junji, Morita Chihiro, Hata Tomokazu, Nozaki Takehiro, Kawai Keisuke, Iwamoto Yasuhiko, Sudo Nobuyuki, Kubo Chiharu The relationship between the age of onset of type 1 diabetes and the subsequent development of a severe eating disorder by female patients. PEDIATRIC DIABETES. 2011;12(4, 2):396-401.

2276. Lieb Roselind, Miche Marcel, Gloster Andrew, Beesdo-Baum Katja, Meyer Andrea, Wittchen Hans-Ulrich IMPACT OF SPECIFIC PHOBIA ON THE RISK OF ONSET OF MENTAL DISORDERS: A 10-YEAR PROSPECTIVE-LONGITUDINAL COMMUNITY STUDY OF ADOLESCENTS AND YOUNG ADULTS. DEPRESSION AND ANXIETY. 2016;33(7):667-675.

2277. Mueller Astrid, Mitchell James, Zwaan Martina Compulsive Buying. AMERICAN JOURNAL ON ADDICTIONS. 2015;24(2):132-137.

2278. Goldschmidt Andrea, Cotton Brandi, Mackey Scott, Laurent Jennifer, Bryson William, Bond Dale Prevalence and Correlates of Loss of Control Eating among Adults Presenting for Methadone Maintenance Treatment. INTERNATIONAL JOURNAL OF BEHAVIORAL MEDICINE. 2018;25(6):693-697.

2279. Barrack Michelle, Rauh Mitchell, Barkai Hava-Shoshana, Nichols Jeanne Dietary restraint and low bone mass in female adolescent endurance runners. AMERICAN JOURNAL OF CLINICAL NUTRITION. 2008;87(1):36-43.

2280. Cragun Deborah, Ata Rheanna, DeBate Rita, Thompson J. Gender Comparisons of Unhealthy Weight-control Behaviors Among Sixth-Graders. JOURNAL OF NUTRITION EDUCATION AND BEHAVIOR. 2013;45(5):450-454.

2281. Sproch Laura, Anderson Kimberly A Novel In-Home Relapse Prevention Treatment for Anorexia Nervosa. CLINICAL CASE STUDIES. 2018;17(6):499-514.

2282. Shimshoni Yaara, Shrinivasa Basavaraj, Cherian Anish, Lebowitz Eli Family accommodation in psychopathology: A synthesized review. INDIAN JOURNAL OF PSYCHIATRY. 2019;61(7, 1):S93-S103.

2283. Valdez Alondra, Smith Kathryn, Mason Tyler Impulsivity and reward sensitivity facets as predictors of weight change in children: Differences by binge-eating disorder diagnostic status. PEDIATRIC OBESITY. 2023;18(2):.

2284. O'Dea JA, Abraham S Knowledge, beliefs, attitudes, and behaviors related to weight control, eating disorders, and body image in Australian trainee home economics and physical education teachers. JOURNAL OF NUTRITION EDUCATION. 2001;33(6):332-340.

2285. Fineberg Naomi, Reghunandanan Samar, Kolli Sangeetha, Atmaca Murad Obsessive-compulsive (anankastic) personality disorder: toward the ICD-11 classification. REVISTA BRASILEIRA DE PSIQUIATRIA. 2014;36(1):S40-S50.

2286. Shioiri Toshiki, Shinada Keita, Kuwabara Hideki, Someya Toshiyuki Early prodromal symptoms and diagnoses before first psychotic episode in 219 inpatients with schizophrenia. PSYCHIATRY AND CLINICAL NEUROSCIENCES. 2007;61(4):348-354.

2287. Saunders Jessica, Eaton Asia, Frazier Stacy Disordered Society: Women in Eating Disorder Recovery Advise Policymakers on Change. ADMINISTRATION AND POLICY IN MENTAL HEALTH AND MENTAL HEALTH SERVICES RESEARCH. 2019;46(2):175-187.

2288. Jans Thomas, Wewetzer Christoph, Klampfl Karin, Schulz Eberhard, Herpertz-Dahlmann Beate, Remschmidt Helmut, Warnke Andreas Phenomenology and co-morbidity of childhood onset obsessive compulsive disorder. ZEITSCHRIFT FUR KINDER-UND JUGENDPSYCHIATRIE UND PSYCHOTHERAPIE. 2007;35(1):41-50.

2289. Bogetto F, Venturello S, Albert U, Maina G, Ravizza L Gender-related clinical differences in obsessive-compulsive disorder. EUROPEAN PSYCHIATRY. 1999;14(8):434-441.

2290. Reilly-Harrington Noreen, Feig Emily, Huffman Jeff Bipolar Disorder and Obesity: Contributing Factors, Impact on Clinical Course, and the Role of Bariatric Surgery. CURRENT OBESITY REPORTS. 2018;7(4):294-300.

2291. Compas BE, Haaga DAF, Keefe FJ, Leitenberg H, Williams DA Sampling of empirically supported psychological treatments from health psychology: Smoking, chronic pain, cancer, and bulimia nervosa. JOURNAL OF CONSULTING AND CLINICAL PSYCHOLOGY. 1998;66(1):89-112.

2292. Herpertz-Dahlmann B Outcome in adolescent anorexia nervosa. ACTA NEUROPSYCHIATRICA. 2002;14(2):90-92.

2293. Viscardi Sharon, Quilodran Javier, Escobar Yissel, Salazar Bettsy, Marileo Luis Nutrition education intervention for children with cancer and their parents. REVISTA CHILENA DE NUTRICION. 2021;48(5):782-788.

2294. Giusti Emanuele, Manna Chiara, Scolari Anna, Mestre Jose, Prevendar Tamara, Castelnuovo Gianluca, Pietrabissa Giada The Relationship between Emotional Intelligence, Obesity and Eating Disorder in Children and Adolescents: A Systematic Mapping Review. INTERNATIONAL JOURNAL OF ENVIRONMENTAL RESEARCH AND PUBLIC HEALTH. 2021;18(4):.

2295. Takii M, Uchigata Y, Nozaki T, Nishikata H, Kawai K, Komaki G, Iwamoto Y, Kubok C Classification of type 1 diabetic females with bulimia nervosa into subgroups 4D according to purging behavior. DIABETES CARE. 2002;25(9):1571-1575.

2296. Michels Nathalie, Susi Kriemler, Marques-Vidal Pedro, Nydegger Andreas, Puder Jardena Psychosocial Quality-of-Life, Lifestyle and Adiposity: A Longitudinal Study in Pre-schoolers (Ballabeina Study). INTERNATIONAL JOURNAL OF BEHAVIORAL MEDICINE. 2016;23(3):383-392.

2297. Bohon Cara, Weinbach Noam, Lock James Performance and brain activity during the Wisconsin Card Sorting Test in adolescents with obsessive-compulsive disorder and adolescents with weight-restored anorexia nervosa. EUROPEAN CHILD \& ADOLESCENT

PSYCHIATRY. 2020;29(2):217-226.

2298. Seike Kaoru, Hanazawa Hisashi, Ohtani Toshiyuki, Takamiya Shizuo, Sakuta Ryoichi, Nakazato Michiko A Questionnaire Survey of the Type of Support Required by Yogo Teachers to Effectively Manage Students Suspected of Having an Eating Disorder. BIOPSYCHOSOCIAL MEDICINE. 2016;10():.

2299. Shan Zhilei, Li Yanping, Zong Geng, Guo Yanjun, Li Jun, Manson JoAnn, Hu Frank, Willett Walter, Schernhammer Eva, Bhupathiraju Shilpa Rotating night shift work and adherence to unhealthy lifestyle in predicting risk of type 2 diabetes: results from two large US cohorts of female nurses. BMJ-BRITISH MEDICAL JOURNAL. 2018;363():.

2300. Wise Taylor Programs on a Diet?: An Examination of Athletic Departments' Eating Disorder Policies at NCAA Division I Universities. JOURNAL OF CLINICAL SPORT PSYCHOLOGY. 2018;12(4, SI):658-674.

2301. Avena Nicole Examining the addictive-like properties of binge eating using an animal model of sugar dependence. EXPERIMENTAL AND CLINICAL PSYCHOPHARMACOLOGY. 2007;15(5):481-491.

2302. Mills Jennifer, Vu Nicole, Manley Ron, Tse Shasha Adolescent and young adult women's opinions of common eating disorder prevention messages. EATING DISORDERS. 2017;25(3):246-262.

2303. Chen Eunice, Bocchieri-Ricciardi Lindsey, Munoz Daniel, Fischer Sarah, Katterman Shawn, Roehrig Megan, Dymek-Valentine Maureen, Alverdy John, Le Grange Daniel Depressed mood in class III obesity predicted by weight-related stigma. OBESITY SURGERY. 2007;17(5):669-671.

2304. Atkinson Melissa, Wade Tracey Does mindfulness have potential in eating disorders prevention? A preliminary controlled trial with young adult women. EARLY INTERVENTION IN PSYCHIATRY. 2016;10(3):234-245.

2305. Bonaccio Marialaura, Iacoviello Licia, Gaetano Giovanni The Mediterranean diet: The reasons for a success. THROMBOSIS RESEARCH. 2012;129(3):401-404.

2306. Longo Paola, Bertorello Antonella, Panero Matteo, Abbate-Daga Giovanni, Marzola Enrica Traumatic events and post-traumatic symptoms in anorexia nervosa. EUROPEAN JOURNAL OF PSYCHOTRAUMATOLOGY. 2019;10(1):.

2307. Sala Margarita, Levinson Cheri A Longitudinal Study on the Association Between Facets of Mindfulness and Disinhibited Eating. MINDFULNESS. 2017;8(4):893-902.

2308. Jansen A A learning model of binge eating: Cue reactivity and cue exposure. BEHAVIOUR RESEARCH AND THERAPY. 1998;36(3):257-272.

2309. Lu Donghao, Aleknaviciute Jurate, Kamperman Astrid, Tamimi Rulla, Ludvigsson Jonas, Valdimarsdottir Unnur, Bertone-Johnson Elizabeth Association Between Childhood Body Size and Premenstrual Disorders in Young Adulthood. JAMA NETWORK OPEN. 2022;5(3):.

2310. JOHNSON WG, JARRELL MP, CHUPURDIA KM, WILLIAMSON DA REPEATED BINGE PURGE CYCLES IN BULIMIA-NERVOSA - ROLE OF GLUCOSE AND INSULIN. INTERNATIONAL JOURNAL OF EATING DISORDERS. 1994;15(4):331-341.

2311. Baenas Isabel, Etxandi Mikel, Munguia Lucero, Granero Roser, Mestre-Bach Gemma, Sanchez Isabel, Ortega Emilio, Andreu Alba, Moize Violeta, Fernandez-Real Jose-Manuel, Tinahones Francisco, Dieguez Carlos, Fruhbeck Gema, Le Grange Daniel, Tchanturia Kate, Karwautz Andreas, Zeiler Michael, Imgart Hartmut, Zanko Annika, Favaro Angela, Claes Laurence, Shekriladze Ia, Serrano-Troncoso Eduardo, Cecilia-Costa Raquel, Rangil Teresa, Loran-Meler Maria, Soriano-Pacheco Jose, Carceller-Sindreu Mar, Navarrete Rosa, Lozano Meritxell, Linares Raquel, Gudiol Carlota, Carratala Jordi, Plana Maria, Graell Montserrat, Gonzalez-Parra David, Gomez-del Barrio Jose, Sepulveda Ana, Sanchez-Gonzalez Jessica, Machado Paulo, Hakansson Anders, Tury Ferenc, Paszthy Bea, Stein Daniel, Papezova Hana, Gricova Jana, Bax Brigita, Borisenkov Mikhail, Popov Sergey, Gubin Denis, Petrov Ivan, Isakova Dilara, Mustafina Svetlana, Kim Youl-Ri, Nakazato Michiko, Godart Nathalie, Voren Robert, Ilnytska Tetiana, Chen Jue, Rowlands Katie, Voderholzer Ulrich, Monteleone Alessio, Treasure Janet, Jimenez-Murcia Susana, Fernandez-Aranda Fernando Impact of COVID-19 Lockdown in Eating Disorders: A Multicentre Collaborative International Study. NUTRIENTS. 2022;14(1):.

2312. Romano SJ, Halmi KA, Sarkar NP, Koke SC, Lee JS A placebo-controlled study of fluoxetine in continued treatment of bulimia nervosa after successful acute fluoxetine treatment. AMERICAN JOURNAL OF PSYCHIATRY. 2002;159(1):96-102.

2313. Kolko Rachel, Emery Rebecca, Marcus Marsha, Levine Michele Loss of control over eating before and during early pregnancy among community women with overweight and obesity. INTERNATIONAL JOURNAL OF EATING DISORDERS. 2017;50(5):582-586.

2314. Pinto Anthony, Greene Ashley, Storch Eric, Simpson H. Prevalence of childhood obsessive-compulsive personality traits in adults with obsessive compulsive disorder versus obsessive compulsive personality disorder. JOURNAL OF OBSESSIVE-COMPULSIVE AND RELATED DISORDERS. 2015;4():25-29.

2315. Citrome L. Lisdexamfetamine for binge eating disorder in adults: a systematic review of the efficacy and safety profile for this newly approved indication what is the number needed to treat, number needed to harm and likelihood to be helped or harmed?. INTERNATIONAL JOURNAL OF CLINICAL PRACTICE. 2015;69(4):410-421.

2316. Souza Magella Luiza, Fontes Vieira Patricia, Lima Pereria Priscila, Prote Karoline Evaluation of the Food Consumption of footballers Members of the Nutfut project of the Federal University of Juiz de Fora. REVISTA BRASILEIRA DE FUTSAL E FUTEBOL. 2019;11(42):8-13.

2317. Rosen Lisa, Scott Shannon, Paulman Briana Peer victimization and eating behaviors in college students: the mediating role of perceived stress. JOURNAL OF AGGRESSION CONFLICT AND PEACE RESEARCH. 2023;():.

2318. Wang Xue-Ping, Wei Ming, Xiao Qin A survey of impulse control disorders in Parkinson's disease patients in Shanghai area and literature review. TRANSLATIONAL NEURODEGENERATION. 2016;5():1-5.

2319. Safi Fatima, Aniserowicz Anna, Colquhoun Heather, Stier Jill, Nowrouzi-Kia Behdin Impact of eating disorders on paid or unpaid work participation and performance: a systematic review and meta-analysis protocol. JOURNAL OF EATING DISORDERS. 2022;10(1):.

2320. Simpson K, Noble S Fluoxetine - A review of its use in women's health. CNS DRUGS. 2000;14(4):301-328.

2321. Crucianelli Laura, Cardi Valentina, Treasure Janet, Jenkinson Paul, Fotopoulou Aikaterini The perception of affective touch in anorexia nervosa. PSYCHIATRY RESEARCH.

2016;239():72-78.

2322. Amerzadeh Mohammad, Takian Amirhossein, Pouraram Hamed, Sari Ali, Ostovar Afshin Economic barriers and gaps to reach the desirable consumption of salt, sugar, and fat in Iran: a qualitative study. JOURNAL OF HEALTH POPULATION AND NUTRITION. 2023;42(1):.

2323. Toyoshima Kuniyoshi, Kusumi Ichiro Controlling the laxative abuse of anorexia nervosa patients with the Serigaya Methamphetamine Relapse Prevention Program workbook: a case report. BIOPSYCHOSOCIAL MEDICINE. 2019;13(1):.

2324. Nagl Michaela, Jacobi Corinna, Paul Martin, Beesdo-Baum Katja, Hoefler Michael, Lieb Roselind, Wittchen Hans-Ulrich Prevalence, incidence, and natural course of anorexia and bulimia nervosa among adolescents and young adults. EUROPEAN CHILD \& ADOLESCENT PSYCHIATRY. 2016;25(8):903-918.

2325. Fitzsimmons-Craft Ellen, Firebaugh Marie-Laure, Graham Andrea, Eichen Dawn, Monterubio Grace, Balantekin Katherine, Karam Anna, Seal Annie, Funk Burkhardt, Taylor C., Wilfley Denise State-Wide University Implementation of an Online Platform for Eating Disorders Screening and Intervention. PSYCHOLOGICAL SERVICES. 2019;16(2, SI):239-249.

2326. Tyrka AR, Waldron I, Graber JA, Brooks-Gunn J Prospective predictors of the onset of anorexic and bulimic syndromes. INTERNATIONAL JOURNAL OF EATING DISORDERS. 2002;32(3):282-290.

2327. Werrij Marieke, Jansen Anita, Mulkens Sandra, Elgersma Hermien, Ament Andre, Hospers Hann Adding cognitive therapy to dietetic treatment is associated with less relapse in obesity. JOURNAL OF PSYCHOSOMATIC RESEARCH. 2009;67(4):315-324.

2328. McLean Sian, Wertheim Eleanor, Masters Jennifer, Paxton Susan A pilot evaluation of a social media literacy intervention to reduce risk factors for eating disorders. INTERNATIONAL JOURNAL OF EATING DISORDERS. 2017;50(7):847-851.

2329. Klavina Aija, Veliks Viktors, Zusa-Rodke Anna, Porozovs Juris, Aniscenko Aleksandrs, Bebrisa-Fedotova Luize The Associations Between Problematic Internet Use, Healthy Lifestyle Behaviors and Health Complaints in Adolescents. FRONTIERS IN EDUCATION. 2021;6():.

2330. Demirdogen Ezgi, Algedik Pinar, Demirpence Dilara Psychometric properties of turkish version of dutch eating behavior questionnaire in adolescents aged between 12 and 18 years and assessment of the effect of age and gender on eating behaviours. KLINIK PSIKIYATRI DERGISI-TURKISH JOURNAL OF CLINICAL PSYCHIATRY. 2021;24(4):547-557.

2331. Williams Lauren, Veitch Jenny, Ball Kylie What helps children eat well? A qualitative exploration of resilience among disadvantaged families. HEALTH EDUCATION RESEARCH. 2011;26(2):296-307.

2332. Pearlman Arielle, Schvey Natasha, Neyland M., Solomon Senait, Hennigan Kathrin, Schindler Rachel, Leu William, Gillmore Dakota, Shank Lisa, Lavender Jason, Burke Natasha, Wilfley Denise, Sbrocco Tracy, Stephens Mark, Jorgensen Sarah, Klein David, Quinlan Jeffrey, Tanofsky-Kraff Marian Associations between Family Weight-Based Teasing, Eating Pathology, and Psychosocial Functioning among Adolescent Military Dependents. INTERNATIONAL JOURNAL OF ENVIRONMENTAL RESEARCH AND PUBLIC HEALTH. 2020;17(1):.

2333. Jackson Alexandra, Weaver Raven, Iniguez Anaderi, Lanigan Jane A lifespan perspective of structural and perceived social relationships, food insecurity, and dietary behaviors during the COVID-19 pandemic. APPETITE. 2022;168():.

2334. Mendia Jara, Pascual Aitziber, Conejero Susana, Mayordomo Sonia The Relationship Between Body and Appearance-Related Self-conscious Emotions and Disordered Eating: The Mediating Role of Symptoms of Depression and Anxiety. INTERNATIONAL JOURNAL OF PSYCHOLOGY AND PSYCHOLOGICAL THERAPY. 2021;21(1):93-105.

2335. Cassidy Omni, Eichen Dawn, Burke Natasha, Patmore Jacqueline, Shore Allison, Radin Rachel, Sbrocco Tracy, Shomaker Lauren, Mirza Nazrat, Young Jami, Wilfley Denise, Tanofsky-Kraff Marian Engaging African American Adolescents and Stakeholders to Adapt Interpersonal Psychotherapy for Weight Gain Prevention. JOURNAL OF BLACK PSYCHOLOGY. 2018;44(2):128-161.

2336. Neumark-Sztainer Dianne, Wall Melanie, Chen Chen, Larson Nicole, Christoph Mary, Sherwood Nancy Eating, Activity, and Weight-related Problems From Adolescence to Adulthood. AMERICAN JOURNAL OF PREVENTIVE MEDICINE. 2018;55(2):133-141.

2337. Drach Linda, Maher Julie, Braun Margaret, Murray Stefanie, Sazie Elizabeth Substance Use, Disordered Eating, and Weight Gain: Describing the Prevention and Treatment Needs

of Incarcerated Women. JOURNAL OF CORRECTIONAL HEALTH CARE. 2016;22(2):139-145.

2338. Zou Haowen, Zhou Hongliang, Yan Rui, Yao Zhijian, Lu Qing Chronotype, circadian rhythm, and psychiatric disorders: Recent evidence and potential mechanisms. FRONTIERS IN NEUROSCIENCE. 2022;16():.

2339. Daulatzai Mak ``Boomerang Neuropathology{''} of Late-Onset Alzheimer's Disease is Shrouded in Harmful ``BDDS{''}: Breathing, Diet, Drinking, and Sleep During Aging. NEUROTOXICITY RESEARCH. 2015;28(1):55-93.

2340. Tos Mateusz, Siuda Joanna Impulse control disorders in Parkinson's disease. AKTUALNOSCI NEUROLOGICZNE. 2021;21(1):30-35.

2341. Hasler G, Pine DS, Gamma A, Milos G, Ajdacic V, Eich D, Rossler W, Angst J The associations between psychopathology and being overweight: a 20-year prospective study. PSYCHOLOGICAL MEDICINE. 2004;34(6):1047-1057.

2342. Breiner Courtney, Scharmer Christina, Zon Caitlyn, Anderson Drew The moderating role of self-compassion on the relationship between emotion-focused impulsivity and dietary restraint in a diverse undergraduate sample. EATING BEHAVIORS. 2022;46():.

2343. Seike Kaoru, Nakazato Michiko, Hanazawa Hisashi, Ohtani Toshiyuki, Niitsu Tomihisa, Ishikawa Shin-ichi, Ayabe Atsuko, Otani Ryoko, Kawabe Kentaro, Horiuchi Fumie, Takamiya Shizuo, Sakuta Ryoichi A questionnaire survey regarding the support needed by Yogo teachers to take care of students suspected of having eating disorders (second report). BIOPSYCHOSOCIAL MEDICINE. 2016;10():.

2344. Boni Marco, Acquarini Elena, Montecchi Leonardo Eating disorders in males. An update. JOURNAL OF PSYCHOPATHOLOGY. 2022;28(3):120-126.

2345. Bezerra Ilana, Sichieri Rosely Household food diversity and nutritional status among adults in Brazil. INTERNATIONAL JOURNAL OF BEHAVIORAL NUTRITION AND PHYSICAL ACTIVITY. 2011;8():.

2346. Oliveira Resende Thaina, Almeida Mauricio, Alvarenga Marle, Brown Tiffany, Carvalho Pedro Dissonance-based eating disorder prevention improves intuitive eating: a randomized controlled trial for Brazilian women with body dissatisfaction. EATING AND

WEIGHT DISORDERS-STUDIES ON ANOREXIA BULIMIA AND OBESITY. 2022;27(3):1099-1112.

2347. Sharma Verinder Relationship of bipolar disorder with psychiatric comorbidity in the postpartum period-a scoping review. ARCHIVES OF WOMENS MENTAL HEALTH. 2018;21(2):141-147.

2348. Duckham R., Brooke-Wavell K., Summers G., Cameron N., Peirce N. Stress fracture injury in female endurance athletes in the United Kingdom: A 12-month prospective study. SCANDINAVIAN JOURNAL OF MEDICINE \& SCIENCE IN SPORTS. 2015;25(6):854-859.

2349. Soares Amaral Ana, Stice Eric, Caputo Ferreira Maria A controlled trial of a dissonance-based eating disorders prevention program with Brazilian girls. PSICOLOGIA-REFLEXAO E CRITICA. 2019;32():.

2350. Carrot B., Radon L., Hubert T., Vibert S., Duclos J., Curt F., Godart N. Are lifetime affective disorders predictive of long-term outcome in severe adolescent anorexia nervosa?. EUROPEAN CHILD \& ADOLESCENT PSYCHIATRY. 2017;26(8):969-978.

2351. Hesselmark Eva, Bejerot Susanne Clinical features of paediatric acute-onset neuropsychiatric syndrome: findings from a case- control study. BJPSYCH OPEN. 2019;5(2):.

2352. Kunstman Jonathan, Smith April, Maner Jon OVERPOWERING RESTRICTION: POWER REDUCES RESTRICTION AMONG SELF-CRITICAL PERFECTIONISTS. JOURNAL OF SOCIAL AND CLINICAL PSYCHOLOGY. 2014;33(7):630-652.

2353. Iniesta Sepulveda Marina, Nadeau Joshua, Whelan Megan, Oiler Christina, Ramos Amaya, Riemann Bradley, Storch Eric Intensive family exposure-based cognitive-behavioral treatment for adolescents with anorexia nervosa. PSICOTHEMA. 2017;29(4):433-439.

2354. Scaife Jessica, Eraifej John, Green Alexander, Petric Beth, Aziz Tipu, Park Rebecca Deep Brain Stimulation of the Nucleus Accumbens in Severe Enduring Anorexia Nervosa: A Pilot Study. FRONTIERS IN BEHAVIORAL NEUROSCIENCE. 2022;16():.

2355. Lugli-Rivero Z, Vivas E Eating disorders and personal behavioral control. SALUD PUBLICA DE MEXICO. 2001;43(1):9-16.

2356. Biederman Joseph, Ball Sarah, Monuteaux Michael, Surman Craig, Johnson Jessica, Zeitlin Sarah Are girls with ADHD at risk for eating disorders? Results from a controlled, five-year prospective study. JOURNAL OF DEVELOPMENTAL AND BEHAVIORAL PEDIATRICS. 2007;28(4):302-307.

2357. Kwok Cecilia, Kwok Victor, Lee Huei, Tan Shian Clinical and socio-demographic features in childhood vs adolescent-onset anorexia nervosa in an Asian population. EATING AND WEIGHT DISORDERS-STUDIES ON ANOREXIA BULIMIA AND OBESITY. 2020;25(3):821-826.

2358. Martinsen Marianne, Bahr Roald, Borresen Runi, Holme Ingar, Pensgaard Anne, Sundgot-Borgen Jorunn Preventing Eating Disorders among Young Elite Athletes: A Randomized Controlled Trial. MEDICINE AND SCIENCE IN SPORTS AND EXERCISE. 2014;46(3):435-447.

2359. Currin L, Schmidt U, Treasure J, Jick H Time trends in eating disorder incidence. BRITISH JOURNAL OF PSYCHIATRY. 2005;186():132-135.

2360. Parmar Deepika, Alabaster Amy, Vance Stanley, Weintraub Miranda, Lau Josephine Disordered Eating, Body Image Dissatisfaction, and Associated Healthcare Utilization Patterns for Sexual Minority Youth. JOURNAL OF ADOLESCENT HEALTH. 2021;69(3):470-476.

2361. Eisenberg Marla, Wall Melanie, Shim Jin, Bruening Meg, Loth Katie, Neumark-Sztainer Dianne Associations between friends' disordered eating and muscle-enhancing behaviors. SOCIAL SCIENCE \& MEDICINE. 2012;75(12):2242-2249.

2362. Frank JB, Thomas CD Externalized self-perceptions, self-silencing, and the prediction of eating pathology. CANADIAN JOURNAL OF BEHAVIOURAL SCIENCE-REVUE CANADIENNE DES SCIENCES DU COMPORTEMENT. 2003;35(3):219-228.

2363. Forbush K., Siew C., Vitevitch M. Application of network analysis to identify interactive systems of eating disorder psychopathology. PSYCHOLOGICAL MEDICINE. 2016;46(12):2667-2677.

2364. Beals KA Mirror, mirror on the wall, who is the most muscular one of all? Disordered eating did body image disturbances in male athletes. ACSMS HEALTH \& FITNESS JOURNAL.

2003;7(2):6-11.

2365. Sandgren Sebastian, Haycraft Emma, Arcelus Jon, Plateau Carolyn Evaluating a motivational and psycho-educational self-help intervention for athletes with mild eating disorder symptoms: A mixed methods feasibility study. EUROPEAN EATING DISORDERS REVIEW. 2022;30(3):250-266.

2366. Veisy Fateme, Ahmadi Seyed, Sadeghi Kheirollah, Rezaee Mansour The Psychometric Properties of Body Shape Questionnaire 8C in Women With Eating Disorders. IRANIAN JOURNAL OF PSYCHIATRY AND CLINICAL PSYCHOLOGY. 2018;23(4):480-492.

2367. Yilmazel Gulay, Bozdogan Serpil Limited health literacy increases the risk of orthorexia nervosa among urban schoolteachers. UNIVERSA MEDICINA. 2020;39(3):162-170.

2368. Bigand Teresa, Dietz Jeremy, Gubitz Hannah, Wilson Marian Barriers and Facilitators to Healthy Eating among Adult Food Bank Users. WESTERN JOURNAL OF NURSING RESEARCH. 2021;43(7):660-667.

2369. Makara-Studzinska Marta, Zaborska Anna Obesity and body image. PSYCHIATRIA POLSKA. 2009;43(1):109-114.

2370. Compte Emilio, Murray Stuart, Sepulveda Ana, Schweiger Sofia, Bressan Michelle, Torrente Fernando What position do you play? Eating disorder pathology among rugby players, and the understudied role of player position. INTERNATIONAL JOURNAL OF EATING DISORDERS. 2018;51(8):1015-1019.

2371. Grilo Carlos, Kerrigan Stephanie, Lydecker Janet, White Marney Physical activity changes during behavioral weight loss treatment by Latinx patients with obesity with and without binge eating disorder. OBESITY. 2021;29(12):2026-2034.

2372. Weintraub Daniel, David Anthony, Evans Andrew, Grant Jon, Stacy Mark Clinical Spectrum of Impulse Control Disorders in Parkinson's Disease. MOVEMENT DISORDERS. 2015;30(2):121-127.

2373. Fang Angela, Hofmann Stefan Relationship between social anxiety disorder and body dysmorphic disorder. CLINICAL PSYCHOLOGY REVIEW. 2010;30(8):1040-1048.

2374. Nicholls Dasha, Lynn Richard, Viner Russell Childhood eating disorders: British national surveillance study. BRITISH JOURNAL OF PSYCHIATRY. 2011;198(4):295-301.

2375. Kaye WH Anorexia nervosa, obsessional behavior, and serotonin. PSYCHOPHARMACOLOGY BULLETIN. 1997;33(3):335-344.

2376. Chung Young, Kim Jin, Lee Jung-Hyun, Jung Young-Chul Onset of dieting in childhood and adolescence: implications for personality, psychopathology, eating attitudes and behaviors of women with eating disorder. EATING AND WEIGHT DISORDERS-STUDIES ON ANOREXIA BULIMIA AND OBESITY. 2017;22(3):491-497.

2377. Larranaga Alejandra, Garia-Mayor Ricardo Psychological treatment for obesity. MEDICINA CLINICA. 2007;129(10):387-391.

2378. Pasch Keryn, Nelson Melissa, Lytle Leslie, Moe Stacey, Perry Cheryl Adoption of Risk-Related Factors Through Early Adolescence: Associations with Weight Status and Implications for Causal Mechanisms. JOURNAL OF ADOLESCENT HEALTH. 2008;43(4):387-393.

2379. Marek Ryan, Ben-Porath Denise, Federici Anita, Wisniewski Lucene, Warren Mark Targeting premeal anxiety in eating disordered clients and normal controls: A preliminary investigation into the use of mindful eating vs. distraction during food exposure. INTERNATIONAL JOURNAL OF EATING DISORDERS. 2013;46(6):582-585.

2380. Rosenthal Lisa, Carroll-Scott Amy, Earnshaw Valerie, Santilli Alycia, Ickovics Jeannette The importance of full-time work for urban adults' mental and physical health. SOCIAL SCIENCE \& MEDICINE. 2012;75(9):1692-1696.

2381. Cassidy Omni, Sbrocco Tracy, Vannucci Anna, Nelson Beatrice, Jackson-Bowen Darlene, Heimdal James, Mirza Nazrat, Wilfley Denise, Osborn Robyn, Shomaker Lauren, Young Jami, Waldron Heather, Carter Michele, Tanofsky-Kraff Marian Adapting Interpersonal Psychotherapy for the Prevention of Excessive Weight Gain in Rural African American Girls. JOURNAL OF PEDIATRIC PSYCHOLOGY. 2013;38(9, SI):965-977.

2382. HerpertzDahlmann BM, Wewetzer C, Schulz E, Remschmidt H Course and outcome in adolescent anorexia nervosa. INTERNATIONAL JOURNAL OF EATING DISORDERS. 1996;19(4):335-345.

2383. Cabrini Silvia, Baratti Mario, Bonfa Flavio, Cabri Giulio, Uber Elena, Avanzi Maurizio Preliminary evaluation of the DDS-PC inventory: a new tool to assess impulsive-compulsive behaviours associated to dopamine replacement therapy in Parkinson's disease. NEUROLOGICAL SCIENCES. 2009;30(4):307-313.

2384. Luca Cerniglia, Silvia Cimino, Giulia Ballarotto, Renata Tambelli Do parental traumatic experiences have a role in the psychological functioning of early adolescents with binge eating disorder?. EATING AND WEIGHT DISORDERS-STUDIES ON ANOREXIA BULIMIA AND OBESITY. 2016;21(4):635-644.

2385. Whittington Joyce, Holland Anthony A review of psychiatric conceptions of mental and behavioural disorders in Prader-Willi syndrome. NEUROSCIENCE AND BIOBEHAVIORAL REVIEWS. 2018;95():396-405.

2386. Frieiro Paula, Gonzalez-Rodriguez Ruben, Dominguez-Alonso Jose Self-esteem and socialisation in social networks as determinants in adolescents' eating disorders. HEALTH \& SOCIAL CARE IN THE COMMUNITY. 2022;30(6):E4416-E4424.

2387. Fombonne E Increased rates of psychosocial disorders in youth. EUROPEAN ARCHIVES OF PSYCHIATRY AND CLINICAL NEUROSCIENCE. 1998;248(1):14-21.

2388. Rosen David, Adolescence Comm Clinical Report-Identification and Management of Eating Disorders in Children and Adolescents. PEDIATRICS. 2010;126(6):1240-1253.

2389. Nakai Yoshikatsu, Nin Kazuko, Goel Neha The changing profile of eating disorders and related sociocultural factors in Japan between 1700 and 2020: A systematic scoping review. INTERNATIONAL JOURNAL OF EATING DISORDERS. 2021;54(1, SI):40-53.

2390. Wade Tracey Developing the ``single-session mindset{''} in eating disorder research: Commentary on Schleider et al., 2023 ``Realizing the untapped promise of single-session interventions for eating disorders{''}. INTERNATIONAL JOURNAL OF EATING DISORDERS. 2023;56(5):864-866.

2391. Griffiths JA, McCabe MP The influence of significant others on disordered eating and body dissatisfaction among early adolescent girls. EUROPEAN EATING DISORDERS REVIEW. 2000;8(4):301-314.

2392. Cusack Claire, Iampieri Alan, Galupo M. ``I'm Still Not Sure If the Eating Disorder is a Result of Gender Dysphoria{''}: Trans and Nonbinary Individuals' Descriptions of Their Eating and Body Concerns in Relation to Their Gender. PSYCHOLOGY OF SEXUAL ORIENTATION AND GENDER DIVERSITY. 2022;9(4):422-433.

2393. Levinson Cheri, Byrne Meghan The Fear of Food Measure: A Novel Measure for use in Exposure Therapy for Eating Disorders. INTERNATIONAL JOURNAL OF EATING DISORDERS. 2015;48(3):271-283.

2394. Levine Michael Prevention of eating disorders: 2021 in review. EATING DISORDERS. 2022;30(2):121-143.

2395. Schnitz Samantha, Hill Laura, Lacey Hubert Eating Disorders in Older Women: Does Late Onset Anorexia Nervosa Exist?. INTERNATIONAL JOURNAL OF EATING DISORDERS. 2010;43(5):393-397.

2396. Strober M, Freeman R, Morrell W The long-term course of severe anorexia nervosa in adolescents: Survival analysis of recovery, relapse, and outcome predictors over 10-15 years in a prospective study. INTERNATIONAL JOURNAL OF EATING DISORDERS. 1997;22(4):339-360.

2397. Fairweather-Schmidt A., Wade Tracey Characterizing and Predicting Trajectories of Disordered Eating Over Adolescence. JOURNAL OF ABNORMAL PSYCHOLOGY. 2016;125(3):369-380.

2398. Ciccolo Erica EXPLORING EXPERIENCE OF FAMILY RELATIONS BY PATIENTS WITH ANOREXIA NERVOSA AND BULIMIA NERVOSA USING A PROJECTIVE FAMILY TEST. PSYCHOLOGICAL REPORTS. 2008;103(1):231-242.

2399. Brandenburg B., Andersen A. Unintentional onset of anorexia nervosa. EATING AND WEIGHT DISORDERS-STUDIES ON ANOREXIA BULIMIA AND OBESITY. 2007;12(2):97-100.

2400. Charlton Emma, Kaehkoenen Laila, Sacks Gary, Cameron Adrian Supermarkets and unhealthy food marketing: An international comparison of the content of supermarket catalogues/circulars. PREVENTIVE MEDICINE. 2015;81():168-173.

2401. Godart N., Radon L., Curt F., Duclos J., Perdereau F., Lang F., Venisse J., Halfon O., Bizouard P., Loas G., Corcos M., Jeammet Ph., Flament M. Mood disorders in eating disorder patients: Prevalence and chronology of ONSET. JOURNAL OF AFFECTIVE DISORDERS. 2015;185():115-122.

2402. Verschueren Margaux, Claes Laurence, Bogaerts Annabel, Palmeroni Nina, Gandhi Amarendra, Moons Philip, Luyckx Koen Eating Disorder Symptomatology and Identity Formation in Adolescence: A Cross-Lagged Longitudinal Approach. FRONTIERS IN PSYCHOLOGY. 2018;9():.

2403. Hartmann Andrea, Steenbergen Florian, Vocks Silja, Buesch Dirk, Waldorf Manuel How Healthy is a Desire to be Fit and Strong? Drives for Thinness, Leanness, and Muscularity in Women in Weight Training. JOURNAL OF CLINICAL SPORT PSYCHOLOGY. 2018;12(4, SI):544-561.

2404. Forrest Lauren, Smith April, Swanson Sonja Characteristics of seeking treatment among US adolescents with eating disorders. INTERNATIONAL JOURNAL OF EATING DISORDERS. 2017;50(7):826-833.

2405. Chisuwa Naomi, O'Dea Jennifer Body image and eating disorders amongst Japanese adolescents. A review of the literature. APPETITE. 2010;54(1):5-15.

2406. Yang Shu, Luo Yi, Chiang Chia-Hsun Electronic Health Literacy and Dietary Behaviors in Taiwanese College Students: Cross-Sectional Study. JOURNAL OF MEDICAL INTERNET RESEARCH. 2019;21(11):.

2407. Peralta Robert, Barr Peter Gender orientation and alcohol-related weight control behavior among male and female college students. JOURNAL OF AMERICAN COLLEGE HEALTH. 2017;65(4):229-242.

2408. Mazzeo SE, Espelage DL Association between childhood physical and emotional abuse and disordered eating behaviors in female undergraduates: An investigation of the mediating role of alexithymia and depression. JOURNAL OF COUNSELING PSYCHOLOGY.

2002;49(1):86-100.

2409. Pettorruso Mauro, Martinotti Giovanni, Fasano Alfonso, Loria Giovanna, Di Nicola Marco, De Risio Luisa, Ricciardi Lucia, Conte Gianluigi, Janiri Luigi, Bentivoglio Anna Anhedonia in Parkinson's disease patients with and without pathological gambling: A case-control study. PSYCHIATRY RESEARCH. 2014;215(2):448-452.

2410. Lucas AR, Crowson CS, O'Fallon WM, Melton LJ The ups and downs of anorexia nervosa. INTERNATIONAL JOURNAL OF EATING DISORDERS. 1999;26(4):397-405.

2411. Krug I, Linardon J., Greenwood C., Youssef G., Treasure J., Fernandez-Aranda F., Karwautz A., Wagner G., Collier D., Anderluh M., Tchanturia K., Ricca V, Sorbi S., Nacmias B., Bellodi L., Fuller-Tyszkiewicz M. A proof-of-concept study applying machine learning methods to putative risk factors for eating disorders: results from the multi-centre European project on healthy eating. PSYCHOLOGICAL MEDICINE. 2023;53(7):2913-2922.

2412. Smith Alyssa, Farstad Sarah, Ranson Kristin Self-reported eating disorder psychopathology prevalence in community-based female and male Albertans: Gender and age group comparisons. INTERNATIONAL JOURNAL OF EATING DISORDERS. 2021;54(3):438-444.

2413. Kaye Walter Neurobiology of anorexia and bulimia nervosa. PHYSIOLOGY \& BEHAVIOR. 2008;94(1, SI):121-135.

2414. Spielvogel Ines, Naderer Brigitte, Binder Alice, Matthes Jorg The Forbidden Reward. The Emergence of Parent-Child Conflicts About Food Over Time and the Influence of Parents' Communication Strategies and Feeding Practices. FRONTIERS IN PUBLIC HEALTH. 2021;8():.

2415. Potoczna N, Branson R, Kral YG, Piec G, Steffen R, Ricklin T, Hoehe MR, Lentes KU, Horber FF Gene variants and binge eating as predictors of comorbidity and outcome of treatment in severe obesity. JOURNAL OF GASTROINTESTINAL SURGERY. 2004;8(8):971-981.

2416. Lang Brittany, Rancourt Diana Drive for leanness: potentially less maladaptive compared to drives for thinness and muscularity. EATING AND WEIGHT DISORDERS-STUDIES ON ANOREXIA BULIMIA AND OBESITY. 2020;25(5):1213-1223.

2417. Linnet Jakob, Jensen Esben, Runge Eik, Hansen Marina, Hertz Soren, Mathiasen Kim, Lichtenstein Mia Text based internet intervention of Binge Eating Disorder (BED): Words per message is associated with treatment adherence. INTERNET INTERVENTIONS-THE APPLICATION OF INFORMATION TECHNOLOGY IN MENTAL AND BEHAVIOURAL HEALTH. 2022;28():.

2418. Cesa Gian, Manzoni Gian, Bacchetta Monica, Castelnuovo Gianluca, Conti Sara, Gaggioli Andrea, Mantovani Fabrizia, Molinari Enrico, Cardenas-Lopez Georgina, Riva Giuseppe Virtual Reality for Enhancing the Cognitive Behavioral Treatment of Obesity With Binge Eating Disorder: Randomized Controlled Study With One-Year Follow-up. JOURNAL OF MEDICAL INTERNET RESEARCH. 2013;15(6):.

2419. Levy RL, Linde JA, Feld KA, Crowell MD, Jeffery RW The association of gastrointestinal symptoms with weight, diet, and exercise in weight-loss program participants. CLINICAL GASTROENTEROLOGY AND HEPATOLOGY. 2005;3(10):992-996.

2420. Yanovski SZ, Billington CJ, Epstein LH, Goodwin NJ, Hill JO, Pi-Sunyer FX, Rolls BJ, Stern JS, Wadden TA, Weinsier RL, Wilson GT, Wing RR, Treat Natl Dieting and the development of eating disorders in overweight and obese adults. ARCHIVES OF INTERNAL MEDICINE. 2000;160(17):2581-2589.

2421. Hillege Sharon, Beale Barbara, McMaster Rose The impact of type 1 diabetes and eating disorders: the perspective of individuals. JOURNAL OF CLINICAL NURSING. 2008;17(7B, SI):169-176.

2422. Cecchini Michele, Sassi Franco, Lauer Jeremy, Lee Yong, Guajardo-Barron Veronica, Chisholm Daniel Chronic Diseases: Chronic Diseases and Development 3 Tackling of unhealthy diets, physical inactivity, and obesity: health effects and cost-effectiveness. LANCET. 2010;376(9754):1775-1784.

2423. GREEN MW, MCKENNA FP DEVELOPMENTAL ONSET OF EATING RELATED COLOR-NAMING INTERFERENCE. INTERNATIONAL JOURNAL OF EATING DISORDERS. 1993;13(4):391-397.

2424. Armstrong Bridget, Westen Sarah, Janicke David The Role of Overweight Perception and Depressive Symptoms in Child and Adolescent Unhealthy Weight Control Behaviors: A Mediation Model. JOURNAL OF PEDIATRIC PSYCHOLOGY. 2014;39(3):340-348.

2425. Espinoza Paola, Penelo Eva, Raich Rosa Disordered eating behaviors and body image in a longitudinal pilot study of adolescent girls: What happens 2 years later?. BODY IMAGE. 2010;7(1):70-73.

2426. Cao Xuehong, Xu Pingwen, Oyola Mario, Xia Yan, Yan Xiaofeng, Saito Kenji, Zou Fang, Wang Chunmei, Yang Yongjie, Hinton Antentor, Yan Chunling, Ding Hongfang, Zhu Liangru, Yu Likai, Yang Bin, Feng Yuxin, Clegg Deborah, Khan Sohaib, DiMarchi Richard, Mani Sheila, Tong Qingchun, Xu Yong Estrogens stimulate serotonin neurons to inhibit binge-like eating in mice. JOURNAL OF CLINICAL INVESTIGATION. 2014;124(10):4351-4362.

2427. Gonsalves Diane, Hawk Helen, Goodenow Carol Unhealthy Weight Control Behaviors and Related Risk Factors in Massachusetts Middle and High School Students. MATERNAL AND CHILD HEALTH JOURNAL. 2014;18(8):1803-1813.

2428. Lippi Giuseppe, Franchini Massimo, Favaloro Emmanuel Holiday Thrombosis. SEMINARS IN THROMBOSIS AND HEMOSTASIS. 2011;37(8):868-873.

2429. Gruettner M. Eating Disorders in Female High School Students: Educational and Migration Background, School-Related Stress and Performance-Orientated Classes. GESUNDHEITSWESEN. 2018;80(1):S5-S11.

2430. Schaefer Lauren, Nooner Kate A dimensional examination of eating disorder symptoms in relation to cognitive processing: An event-related potentials study. JOURNAL OF APPLIED BIOBEHAVIORAL RESEARCH. 2018;23(3):.

2431. FELKER KR, STIVERS C THE RELATIONSHIP OF GENDER AND FAMILY ENVIRONMENT TO EATING DISORDER RISK IN ADOLESCENTS. ADOLESCENCE. 1994;29(116):821-834.

2432. Perales Jose, Maldonado Antonio, Lopez-Quirantes Eva, Lopez-Torrecillas Francisca Association patterns of cannabis abuse and dependence with risk of problematic non-substance-related dysregulated and addictive behaviors. PLOS ONE. 2021;16(8):.

2433. Lyzwinski Lynnette, Caffery Liam, Bambling Matthew, Edirippulige Sisira The Relationship Between Stress and Maladaptive Weight-Related Behaviors in College Students: A Review of the Literature. AMERICAN JOURNAL OF HEALTH EDUCATION. 2018;49(3):166-178.

2434. Hall Peter Brain Stimulation as a Method for Understanding, Treating, and Preventing Disorders of Indulgent Food Consumption. CURRENT ADDICTION REPORTS. 2019;6(3):266-272.

2435. Lawrence Jean, Liese Angela, Liu Lenna, Dabelea Dana, Anderson Andrea, Imperatore Giuseppina, Bell Ronny Weight-Loss Practices and Weight-Related Issues Among Youth With Type 1 or Type 2 Diabetes. DIABETES CARE. 2008;31(12):2251-2257.

2436. Zanella Elise, Lee Eunro Integrative review on psychological and social risk and prevention factors of eating disorders including anorexia nervosa and bulimia nervosa: seven major theories. HELIYON. 2022;8(11):.

2437. Fernando Gomez Luis, Fernanda Parra Maria, Ivan Lucumi Diego, Camila Bermudez Laura, Jose Calderon Antonio, Mantilla Juliana, Mora-Plazas Mercedes Relevance of Political Actions Aimed at Guaranteeing a Healthy and Sufficient Diet During and After the COVID-19 Pandemic in the context of Latin America. UNIVERSITAS MEDICA. 2021;62(3):.

2438. Gagliano Antonella, Galati Cecilia, Ingrassia Massimo, Ciuffo Massimo, Alquino Maria, Tanca Marcello, Carucci Sara, Zuddas Alessandro, Grossi Enzo Pediatric Acute-Onset Neuropsychiatric Syndrome: A Data Mining Approach to a Very Specific Constellation of Clinical Variables. JOURNAL OF CHILD AND ADOLESCENT PSYCHOPHARMACOLOGY. 2020;30(8):495-511.

2439. Garcia-Continente X., Allue N., Perez-Gimenez A., Ariza C., Sanchez-Martinez F., Lopez M., Nebot M. Eating habits, sedentary behaviors and overweight and obesity among adolescents in Barcelona. ANALES DE PEDIATRIA. 2015;83(1):3-10.

2440. Zeiler Michael, Waldherr Karin, Philipp Julia, Nitsch Martina, Duer Wolfgang, Karwautz Andreas, Wagner Gudrun Prevalence of Eating Disorder Risk and Associations with Health-related Quality of Life: Results from a Large School-based Population Screening. EUROPEAN EATING DISORDERS REVIEW. 2016;24(1):9-18.

2441. Treasure Janet, Crane Anna, McKnight Rebecca, Buchanan Emmakate, Wolfe Melissa First do no harm: Iatrogenic Maintaining Factors in Anorexia Nervosa. EUROPEAN EATING DISORDERS REVIEW. 2011;19(4):296-302.

2442. Wilson Rebecca, Marshall Rachel, Murakami Jessica, Latner Janet Brief non-dieting intervention increases intuitive eating and reduces dieting intention, body image

dissatisfaction, and anti-fat attitudes: A randomized controlled trial. APPETITE. 2020;148():.

2443. Hargrave Sara, Kinzig Kimberly Repeated gastric distension alters food intake and neuroendocrine profiles in rats. PHYSIOLOGY \& BEHAVIOR. 2012;105(4):975-981.

2444. Latimer Lara, Velazquez Cayley, Pasch Keryn Characteristics and Behaviors of Non-overweight College Students Who are Trying to Lose Weight. JOURNAL OF PRIMARY PREVENTION. 2013;34(4):251-260.

2445. Jacoby GE, Braks K, Kopp W Reports on sexual abuse by eating-disordered women before and after psychotherapy: A comparison of anamnestic and catamnestic data. EUROPEAN EATING DISORDERS REVIEW. 1997;5(3):171-183.

2446. Cezaretto Adriana, Siqueira-Catania Antonela, Barros Camila, Salvador Emanuel, Ferreira Sandra Benefits on quality of life concomitant to metabolic improvement in intervention program for prevention of diabetes mellitus. QUALITY OF LIFE RESEARCH. 2012;21(1):105-113.

2447. Carton Louise, Icick Romain, Weibel Sebastien, Dematteis Maurice, Kammerer Etienne, Batisse Anne, Rolland Benjamin What is the potential for abuse of lisdexamfetamine in adults? A preclinical and clinical literature review and expert opinion. EXPERT REVIEW OF CLINICAL PHARMACOLOGY. 2022;15(8):921-925.

2448. Ganson Kyle, Lavender Jason, Rodgers Rachel, Cunningham Mitchell, Nagata Jason Compulsive exercise and vaping among a sample of US College students aged 18-26 years. EATING AND WEIGHT DISORDERS-STUDIES ON ANOREXIA BULIMIA AND OBESITY. 2022;27(3):1153-1161.

2449. Fernell Elisabeth, Sundin Mikael, Fasth Anders, Dinkler Lisa, Galazka Martyna, Gillberg Christopher, Johnson Mats Paediatric Acute onset Neuropsychiatric Syndrome: Exploratory study finds no evidence of HLA class II association but high rate of autoimmunity in first-degree relatives. ACTA PAEDIATRICA. 2022;111(4):820-824.

2450. Bardone-Cone Anna, Lin Stacy, Butler Rachel Perfectionism and Contingent Self-Worth in Relation to Disordered Eating and Anxiety. BEHAVIOR THERAPY. 2017;48(3):380-390.

2451. Stice Eric, Marti C., Spoor Sonja, Presnell Katherine, Shaw Heather Dissonance and healthy weight eating disorder prevention programs: Long-term effects from a randomized efficacy trial. JOURNAL OF CONSULTING AND CLINICAL PSYCHOLOGY. 2008;76(2):329-340.

2452. Samuels JF, Riddle MA, Greenberg BD, Fyer AJ, McCracken JT, Rauch SL, Murphy DL, Grados MA, Pinto A, Knowles JA, Piacentini J, Cannistraro PA, Cullen B, Bienvenu OJ, Rasmussen SA, Pauls DL, Willour VL, Shugart YY, Liang KY, Hoehn-Saric R, Nestadt G The OCD Collaborative Genetics Study: Methods and sample description. AMERICAN JOURNAL OF MEDICAL GENETICS PART B-NEUROPSYCHIATRIC GENETICS. 2006;141B(3):201-207.

2453. Burnette C., Mazzeo Suzanne An uncontrolled pilot feasibility trial of an intuitive eating intervention for college women with disordered eating delivered through group and guided self-help modalities. INTERNATIONAL JOURNAL OF EATING DISORDERS. 2020;53(9):1405-1417.

2454. Akers Laura, Rohde Paul, Shaw Heather, Stice Eric Cost-Effectiveness Comparison of Delivery Modalities for a Dissonance-Based Eating Disorder Prevention Program over 4-Year Follow-Up. PREVENTION SCIENCE. 2021;22(8):1086-1095.

2455. WILSON GT RELATION OF DIETING AND VOLUNTARY WEIGHT-LOSS TO PSYCHOLOGICAL FUNCTIONING AND BINGE-EATING. ANNALS OF INTERNAL MEDICINE. 1993;119(7, 2):727-730.

2456. Reel Justine, Petrie Trent, SooHoo Sonya, Anderson Carlin Weight pressures in sport: Examining the factor structure and incremental validity of the weight pressures in sport - Females. EATING BEHAVIORS. 2013;14(2):137-144.

2457. Kalarchian Melissa, King Wendy, Devlin Michael, Marcus Marsha, Garcia Luis, Chen Jia-Yuh, Yanovski Susan, Mitchell James Psychiatric Disorders and Weight Change in a Prospective Study of Bariatric Surgery Patients: A 3-Year Follow-Up. PSYCHOSOMATIC MEDICINE. 2016;78(3):373-381.

2458. Troop NA, Treasure JL Psychosocial factors in the onset of eating disorders: Responses to life-events and difficulties. BRITISH JOURNAL OF MEDICAL PSYCHOLOGY. 1997;70(4):373-385.

2459. Corcos M, Flament MF, Giraud MJ, Paterniti S, Ledoux S, Atger F, Jeammet P Early psychopathological signs in bulimia nervosa. A retrospective comparison of the period of puberty in bulimic and control girls. EUROPEAN CHILD \& ADOLESCENT PSYCHIATRY. 2000;9(2):115-121.

2460. Nolen-Hoeksema Susan, Stice Eric, Wade Emily, Bohon Cara Reciprocal relations between rumination and bulimic, substance abuse, and depressive symptoms in female adolescents. JOURNAL OF ABNORMAL PSYCHOLOGY. 2007;116(1):198-207.

2461. Linardon Jake, Greenwood Christopher, Fuller-Tyszkiewicz Matthew, Macdonald Jacqui, Spry Elizabeth, Hutchinson Delyse, Youssef George, Sanson Ann, Wertheim Eleanor, McIntosh Jennifer, Le Grange Daniel, Letcher Primrose, Olsson Craig Young adult mental health sequelae of eating and body image disturbances in adolescence. INTERNATIONAL JOURNAL OF EATING DISORDERS. 2021;54(9):1680-1688.

2462. Greene Amanda, Brownstone Lisa ``Just a place to keep track of myself{''}: eating disorders, social media, and the quantified self. FEMINIST MEDIA STUDIES. 2023;23(2):508-524.

2463. Petroni Maria, Villanova Nicola, Avagnina Sebastiano, Fusco Maria, Fatati Giuseppe, Compare Angelo, Marchesini Giulio, Grp QUOVADIS Psychological distress in morbid obesity in relation to weight history. OBESITY SURGERY. 2007;17(3):391-399.

2464. Mullie P., Clarys P., Hulens M., Vansant G. Dietary patterns and socioeconomic position. EUROPEAN JOURNAL OF CLINICAL NUTRITION. 2010;64(3):231-238.

2465. Bacopoulou Flora, Foskolos Elizabeth, Stefanaki Charikleia, Tsitsami Eleni, Vousoura Eleni Disordered eating attitudes and emotional/behavioral adjustment in Greek adolescents. EATING AND WEIGHT DISORDERS-STUDIES ON ANOREXIA BULIMIA AND OBESITY. 2018;23(5):621-628.

2466. Strumia Renata Eating disorders and the skin. CLINICS IN DERMATOLOGY. 2013;31(1):80-85.

2467. Pasdar Yahya, Moradi Shima, Saedi Saman, Moradinazar Mehdi, Rahmani Negin, Hamzeh Behrooz, Najafi Farid Mediterranean-DASH Intervention for Neurodegenerative Delay (MIND) diet in relation to age-associated poor muscle strength; a cross-sectional

study from the Kurdish cohort study. SCIENTIFIC REPORTS. 2022;12(1):.

2468. Turton P, Hughes P, Bolton H, Sedgwick P Incidence and demographic correlates of eating disorder symptoms in a pregnant population. INTERNATIONAL JOURNAL OF EATING DISORDERS. 1999;26(4):448-452.

2469. Warren MP, Voussoughian F, Geer EB, Hyle EP, Adberg CL, Ramos RH Functional hypothalamic amenorrhea: Hypoleptinemia and disordered eating. JOURNAL OF CLINICAL ENDOCRINOLOGY \& METABOLISM. 1999;84(3):873-877.

2470. Sandoz Emily, Boullion Gina, Mallik Debesh, Hebert Emmie Relative associations of body image avoidance constructs with eating disorder pathology in a large college student sample. BODY IMAGE. 2020;34():242-248.

2471. Cliffe Charlotte, Shetty Hitesh, Himmerich Hubertus, Schmidt Ulrike, Stewart Robert, Dutta Rina Suicide attempts requiring hospitalization in patients with eating disorders: A retrospective cohort study. INTERNATIONAL JOURNAL OF EATING DISORDERS. 2020;53(5):458-465.

2472. Bauer Katherine, Larson Nicole, Nelson Melissa, Story Mary, Neumark-Sztainer Dianne Socio-environmental, personal and behavioural predictors of fast-food intake among adolescents. PUBLIC HEALTH NUTRITION. 2009;12(10):1767-1774.

2473. Tchanturia K, Anderluh MB, Morris RG, Rabe-Hesketh S, Collier DA, Sanchez P, Treasure JL Cognitive flexibility in anorexia nervosa and bulimia nervosa. JOURNAL OF THE INTERNATIONAL NEUROPSYCHOLOGICAL SOCIETY. 2004;10(4):513-520.

2474. Kuehner C. Mental Disorders in Pregnancy and Postpartum Prevalence, Course and clinical Diagnostics. NERVENARZT. 2016;87(9):926-936.

2475. Smiarowska Malgorzata, Bialecka Monika, Korwin-Piotrowska Karolina Molecular mediators in control of food consumption and energy balance in eating disorders. ADVANCES IN CLINICAL AND EXPERIMENTAL MEDICINE. 2007;16(4):569-576.

2476. Voon Valerie, Schoerling Andrea, Wenzel Sascha, Ekanayake Vindhya, Reiff Julia, Trenkwalder Claudia, Sixel-Doering Friederike Frequency of impulse control behaviours associated with dopaminergic therapy in restless legs syndrome. BMC NEUROLOGY.

2011;11():.

2477. Spoor Sonja, Stice Eric, Bekker Marrie, Van Strien Tatjana, Croon Marcel, Van Heck Guus Relations between dietary restraint, depressive symptoms, and binge eating: A longitudinal study. INTERNATIONAL JOURNAL OF EATING DISORDERS. 2006;39(8):700-707.

2478. Olatunji BO, Sawchuk CN Disgust: Characteristic features, social manifestations, and clinical implications. JOURNAL OF SOCIAL AND CLINICAL PSYCHOLOGY. 2005;24(7):932-962.

2479. Franko Debra, Jenkins Amy, Roehrig James, Luce Kristine, Crowther Janis, Rodgers Rachel Psychometric properties of measures of eating disorder risk in latina college women. INTERNATIONAL JOURNAL OF EATING DISORDERS. 2012;45(4):592-596.

2480. Schmidt Jennifer, Martin Alexandra ``Smile away your cravings{''} - Facial feedback modulates cue -induced food cravings. APPETITE. 2017;116():536-543.

2481. Moseley Katrina-Louise From Beveridge Britain to Birds Eye Britain: shaping knowledge about `healthy eating' in the mid-to-late twentieth-century. CONTEMPORARY BRITISH HISTORY. 2021;35(4):515-544.

2482. Liu Xingcun Study on the relationship between diet and environmental exposure-related factors and the incidence of colorectal cancer. INTERNATIONAL JOURNAL OF CLINICAL AND EXPERIMENTAL MEDICINE. 2020;13(10):8037-8043.

2483. Bell Brooke, Spruijt-Metz Donna, Naya Christine, Lane Christianne, Wen Cheng, Davis Jaimie, Weigensberg Marc The mediating role of emotional eating in the relationship between perceived stress and dietary intake quality in Hispanic/Latino adolescents. EATING BEHAVIORS. 2021;42():.

2484. Goode Rachel, Ye Lei, Sereika Susan, Zheng Yaguang, Mattos Meghan, Acharya Sushama, Ewing Linda, Danford Cynthia, Hu Lu, Imes Christopher, Chasens Eileen, Osier Nicole, Mancino Juliet, Burke Lora Socio-demographic, anthropometric, and psychosocial predictors of attrition across behavioral weight-loss trials. EATING BEHAVIORS. 2016;20():27-33.

2485. Fairweather-Schmidt A., Wade Tracey Common genetic architecture and environmental risk factors underpin the anxiety-disordered eating relationship: Findings from an adolescent twin cohort. INTERNATIONAL JOURNAL OF EATING DISORDERS. 2020;53(1):52-60.

2486. Roveda E., Montaruli A., Galasso L., Pesenti C., Bruno E., Pasanisi P., Cortellini M., Rampichini S., Erzegovesi S., Caumo A., Esposito F. Rest-activity circadian rhythm and sleep quality in patients with binge eating disorder. CHRONOBIOLOGY INTERNATIONAL. 2018;35(2):198-207.

2487. Steinhausen Hans-Christoph, Jakobsen Helle Incidence Rates of Treated Mental Disorders in Childhood and Adolescence in a Complete Nationwide Birth Cohort. JOURNAL OF CLINICAL PSYCHIATRY. 2019;80(3):.

2488. Lazarevich Irina, Esther Irigoyen-Camacho Maria, Consuelo Velazquez-Alva Maria Obesity, eating behaviour and mental health among university students in Mexico city. NUTRICION HOSPITALARIA. 2013;28(6):1892-1899.

2489. Blackstone Sarah, Johnson Aimee, Sutton Debra Perceptions of Weight-Conscious Drinking and the Role of Mental Health: A Mixed-Methods Approach. AMERICAN JOURNAL OF HEALTH EDUCATION. 2019;50(4, SI):225-235.

2490. Mancine Ryley, Gusfa Donald, Moshrefi Ali, Kennedy Samantha Prevalence of disordered eating in athletes categorized by emphasis on leanness and activity type - a systematic review. JOURNAL OF EATING DISORDERS. 2020;8(1):.

2491. Hebebrand Johannes, Gearhardt Ashley The concept of ``food addiction{''} helps inform the understanding of overeating and obesity: NO. AMERICAN JOURNAL OF CLINICAL NUTRITION. 2021;113(2):268-273.

2492. Hazzard Vivienne, Ziobrowski Hannah, Borg Skylar, Schaefer Lauren, Mangold Ani, Herting Nicola, Lipson Sarah, Crosby Ross, Wonderlich Stephen Past-Year Abuse and Eating Disorder Symptoms Among US College Students. JOURNAL OF INTERPERSONAL VIOLENCE. 2022;37(15-16):NP13226-NP13244.

2493. Chatelan Angeline, Carrard Isabelle Diet quality in middle-aged and older women with and without body weight dissatisfaction: results from a population-based national

nutrition survey in Switzerland. JOURNAL OF NUTRITIONAL SCIENCE. 2021;10():.

2494. Friars Dara, Walsh Orla, McNicholas Fiona Assessment and management of cardiovascular complications in eating disorders. JOURNAL OF EATING DISORDERS. 2023;11(1):.

2495. Song Weichen, Wang Weidi, Yu Shunying, Lin Guan Dissection of the Genetic Association between Anorexia Nervosa and Obsessive-Compulsive Disorder at the Network and Cellular Levels. GENES. 2021;12(4):.

2496. Bailly-Lambin I, Bailly D Separation anxiety disorder and eating disorders. ENCEPHALE-REVUE DE PSYCHIATRIE CLINIQUE BIOLOGIQUE ET THERAPEUTIQUE. 1999;25(3):226+.

2497. Nacke Barbara, Beintner Ina, Goerlich Dennis, Vollert Bianka, Schmidt-Hantke Juliane, Huetter Kristian, Taylor C., Jacobi Corinna everyBody-Tailored online health promotion and eating disorder prevention for women: Study protocol of a dissemination trial. INTERNET INTERVENTIONS-THE APPLICATION OF INFORMATION TECHNOLOGY IN MENTAL AND BEHAVIOURAL HEALTH. 2019;16(SI):20-25.

2498. Haszard Jillian, Skidmore Paula, Williams Sheila, Taylor Rachael Associations between parental feeding practices, problem food behaviours and dietary intake in New Zealand overweight children aged 4-8 years. PUBLIC HEALTH NUTRITION. 2015;18(6):1036-1043.

2499. Jongenelis Michelle, Pettigrew Simone Body Image and Eating Disturbances in Children: The Role of Self-Objectification. PSYCHOLOGY OF WOMEN QUARTERLY. 2020;44(3):393-402.

2500. Hinney Anke, Volckmar Anna-Lena Genetics of Eating Disorders. CURRENT PSYCHIATRY REPORTS. 2013;15(12):.

2501. Schiros Ashley, Antshel Kevin The relationship between anorexia nervosa and bulimia nervosa, attention deficit/hyperactivity disorder, and suicidality in college students. EUROPEAN EATING DISORDERS REVIEW. 2023;31(3):390-401.

2502. Tabri Nassim, Werner Kaitlyn, Milyayskaya Marina, Wohl Michael Perfectionism Predicts Disordered Gambling Via Financially Focused Self-Concept. JOURNAL OF

GAMBLING ISSUES. 2018;(38, SI):252-267.

2503. Yilmaz Zeynep, Kaplan Allan, Levitan Robert, Zai Clement, Kennedy James Possible association of the DRD4 gene with a history of attention-deficit/hyperactivity disorder in women with bulimia nervosa. INTERNATIONAL JOURNAL OF EATING DISORDERS. 2012;45(4):622-625.

2504. Beintner Ina, Jacobi Corinna, Taylor Craig Effects of an Internet-based Prevention Programme for Eating Disorders in the USA and Germany u A Meta-analytic Review. EUROPEAN EATING DISORDERS REVIEW. 2012;20(1):1-8.

2505. Wade T, Heath AC, Abraham S, Treloar SA, Martin NG, Tiggemann M Assessing the prevalence of eating disorders in an Australian twin population. AUSTRALIAN AND NEW ZEALAND JOURNAL OF PSYCHIATRY. 1996;30(6):845-851.

2506. Lee Kirsty, Vaillancourt Tracy A Four-Year Prospective Study of Bullying, Anxiety, and Disordered Eating Behavior Across Early Adolescence. CHILD PSYCHIATRY \& HUMAN DEVELOPMENT. 2019;50(5):815-825.

2507. Zerwas Stephanie, Von Holle Ann, Watson Hunna, Gottfredson Nisha, Bulik Cynthia Childhood Anxiety Trajectories and Adolescent Disordered Eating: Findings from the NICHD Study of Early Child Care and Youth Development. INTERNATIONAL JOURNAL OF EATING DISORDERS. 2014;47(7, SI):784-792.

2508. Gillikin Lindsay, Manasse Stephanie, Dyk Ilana An examination of emotion regulation as a mechanism underlying eating disorder pathology in lesbian, gay, and bisexual individuals. EATING BEHAVIORS. 2021;41():.

2509. Bulchmann G, Seifert-Klauss V, Backmund H, Gelinghoff M Eating disorders in gynecologic patients. GEBURTSHILFE UND FRAUENHEILKUNDE. 2001;61(8):569-577.

2510. Bjoerk Tabita, Skarberg Kurt, Engstroem Ingemar Eating disorders and anabolic androgenic steroids in males - similarities and differences in self-image and psychiatric symptoms. SUBSTANCE ABUSE TREATMENT PREVENTION AND POLICY. 2013;8():.

2511. Tylka Tracy, Calogero Rachel, Danielsdottir Sigrun Is intuitive eating the same as flexible dietary control? Their links to each other and well-being could provide an answer.

APPETITE. 2015;95():166-175.

2512. Rowe Sarah, Jordan Jennifer, McIntosh Virginia, Carter Frances, Frampton Chris, Bulik Cynthia, Joyce Peter Dimensional measures of personality as a predictor of outcome at 5-year follow-up in women with bulimia nervosa. PSYCHIATRY RESEARCH. 2011;185(3):414-420.

2513. SULLIVAN PF, BULIK CM, CARTER FA, JOYCE PR THE SIGNIFICANCE OF A HISTORY OF CHILDHOOD SEXUAL ABUSE IN BULIMIA-NERVOSA. BRITISH JOURNAL OF PSYCHIATRY. 1995;167():679-682.

2514. Rojo-Moreno Luis, Rubio Teresa, Plumed Javier, Barbera Maria, Serrano Marisa, Gimeno Natalia, Conesa Llanos, Ruiz Elias, Rojo-Bofill Luis, Beato Luis, Livianos Lorenzo Teasing and Disordered Eating Behaviors in Spanish Adolescents. EATING DISORDERS. 2013;21(1):53-69.

2515. Lanfredi Mariangela, Macis Ambra, Ferrari Clarissa, Meloni Serena, Pedrini Laura, Ridolfi Maria, Zonca Valentina, Cattane Nadia, Cattaneo Anna, Rossi Roberta Maladaptive behaviours in adolescence and their associations with personality traits, emotion dysregulation and other clinical features in a sample of Italian students: a cross-sectional study. BORDERLINE PERSONALITY DISORDER AND EMOTION DYSREGULATION. 2021;8(1):.

2516. Leon-Munoz Luz, Garcia-Esquinas Esther, Soler-Vila Hosanna, Guallar-Castillon Pilar, Banegas Jose, Rodriguez-Artalejo Fernando Unhealthy Eating Behaviors and Weight Gain: A Prospective Study in Young and Middle-Age Adults. OBESITY. 2016;24(5):1178-1184.

2517. Jones Candace, Pearce Brad, Barrera Ingrid, Mummert Amanda Fetal programming and eating disorder risk. JOURNAL OF THEORETICAL BIOLOGY. 2017;428():26-33.

2518. Thomas Kai, Williams Marc, Vanderwert Ross Disordered eating and internalizing symptoms in preadolescence. BRAIN AND BEHAVIOR. 2021;11(1):.

2519. Quiles-Marcos Yolanda, Balaguer-Sola Isabel, Pamies-Aubalat Lidia, Jose Quiles-Sebastian Mara, Carlos Marzo-Campos Juan, Rodriguez-Marin Jesus Eating Habits, Physical Activity, Consumption of Substances and Eating Disorders in Adolescents. SPANISH JOURNAL OF PSYCHOLOGY. 2011;14(2):712-723.

2520. Kluck Annette Family influence on disordered eating: The role of body image dissatisfaction. BODY IMAGE. 2010;7(1):8-14.

2521. SMITH C, FELDMAN SS, NASSERBAKHT A, STEINER H PSYCHOLOGICAL CHARACTERISTICS AND DSM-III-R DIAGNOSES AT 6-YEAR FOLLOW-UP OF ADOLESCENT ANOREXIA-NERVOSA. JOURNAL OF THE AMERICAN ACADEMY OF CHILD AND ADOLESCENT PSYCHIATRY. 1993;32(6):1237-1245.

2522. Quevedo Bolivar Paula Malnutrition: Beyond Nutritional Deficiencies. TRABAJO SOCIAL. 2019;21(1):219-239.

2523. Monteleone Palmiero, Di Genio Monica, Monteleone Alessio, Di Filippo Carmela, Maj Mario Investigation of factors associated to crossover from anorexia nervosa restricting type (ANR) and anorexia nervosa binge-purging type (ANBP) to bulimia nervosa and comparison of bulimia nervosa patients with or without previous ANR or ANBP. COMPREHENSIVE PSYCHIATRY. 2011;52(1):56-62.

2524. Richardson Thomas, Stallard Paul, Velleman Sophie Computerised Cognitive Behavioural Therapy for the Prevention and Treatment of Depression and Anxiety in Children and Adolescents: A Systematic Review. CLINICAL CHILD AND FAMILY PSYCHOLOGY REVIEW. 2010;13(3):275-290.

2525. Masheb Robin, White Marney, Grilo Carlos Substantial weight gains are common prior to treatment-seeking in obese patients with binge eating disorder. COMPREHENSIVE PSYCHIATRY. 2013;54(7):880-884.

2526. Nakai Y, Hamagaki S, Kato S, Seino Y, Takagi R, Kurimoto F Role of leptin in women with eating disorders. INTERNATIONAL JOURNAL OF EATING DISORDERS. 1999;26(1):29-35.

2527. Kalarchian Melissa, Marcus Marsha Psychiatric comorbidity of childhood obesity. INTERNATIONAL REVIEW OF PSYCHIATRY. 2012;24(3):241-246.

2528. SIDIBE EH, NDIAYE AT, DIOP G, KANE A, SOW AM, DIOPFASSA H, SARR A, DIOP SN, KACISSE M CHRONIC DIET-RELATED DISORDERS IN AFRICAN ADULTS. SEMAINE DES HOPITAUX. 1995;71(15-16):482-488.

2529. Allen Karina, Byrne Susan, Hii Hilary, Eekelen Anke, Mattes Eugen, Foster Jonathan Neurocognitive functioning in adolescents with eating disorders: A population-based study. COGNITIVE NEUROPSYCHIATRY. 2013;18(5):355-375.

2530. Babbs R., Unger E., Corwin R. 2-Hydroxyestradiol enhances binge onset in female rats and reduces prefrontal cortical dopamine in male rats. HORMONES AND BEHAVIOR. 2013;63(1):88-96.

2531. Ostuzzi Roberto, D'Andrea Giovanni, Francesconi Federica, Musco Francesca Eating disorders and headache: coincidence or consequence?. NEUROLOGICAL SCIENCES. 2008;29(1):S83-S87.

2532. Smith Gregory, Simmons Jean, Flory Kate, Annus Agnes, Hill Kelly Thinness and eating expectancies predict subsequent binge-eating and purging behavior among adolescent girls. JOURNAL OF ABNORMAL PSYCHOLOGY. 2007;116(1):188-197.

2533. Fakra Eric, Belzeaux R., Azorin J., Adida M. Affective disorders and eating disorders. ENCEPHALE-REVUE DE PSYCHIATRIE CLINIQUE BIOLOGIQUE ET THERAPEUTIQUE. 2014;40(3):S46-S50.

2534. Valero Solis Susana, Granero Perez Roser, Sanchez-Carracedo David Frequency of family meals and risk of eating disorders in adolescents in Spain and Peru. REVISTA LATINOAMERICANA DE PSICOLOGIA. 2019;51(1):48-57.

2535. Fichter MM, Leibl C, Kruger R, Rief W Effects of fluvoxamine on depression, anxiety, and other areas of general psychopathology in bulimia nervosa. PHARMACOPSYCHIATRY. 1997;30(3):85-92.

2536. Puhl Rebecca, Neumark-Sztainer Dianne, Austin S., Suh Young, Wakefield Dorothy Policy Actions to Address Weight-Based Bullying and Eating Disorders in Schools: Views of Teachers and School Administrators. JOURNAL OF SCHOOL HEALTH. 2016;86(7):507-515.

2537. Stice Eric, Rohde Paul, Shaw Heather, Gau Jeff An Effectiveness Trial of a Selected Dissonance-Based Eating Disorder Prevention Program for Female High School Students: Long-Term Effects. JOURNAL OF CONSULTING AND CLINICAL PSYCHOLOGY. 2011;79(4):500-508.

2538. Steiner H, Lock J Anorexia nervosa and bulimia nervosa in children and adolescents: A review of the past 10 years. JOURNAL OF THE AMERICAN ACADEMY OF CHILD AND ADOLESCENT PSYCHIATRY. 1998;37(4):352-359.

2539. McVey Gail, Tweed Stacey, Blackmore Elizabeth Healthy Schools-Healthy Kids: A controlled evaluation of a comprehensive universal eating disorder prevention program. BODY IMAGE. 2007;4(2):115-136.

2540. Castro-Fornieles Josefina, Deulofeu Ramon, Martinez-Mallen Esteve, Baeza Immaculada, Fernandez Lorena, Lazaro Luisa, Toro Josep, Vila Montserrat, Bernardo Miquel Plasma homovanillic acid in adolescents with bulimia nervosa. PSYCHIATRY RESEARCH. 2009;170(2-3):241-244.

2541. Herpertz-Dahlmann B., Buehren K., Seitz J. Anorexia nervosa in childhood and adolescence. Course and significance for adulthood. NERVENARZT. 2011;82(9):1093+.

2542. Burger Kyle, Stice Eric Relation of dietary restraint scores to activation of reward-related brain regions in response to food intake, anticipated intake, and food pictures. NEUROIMAGE. 2011;55(1):233-239.

2543. Unikel Claudia, Root Tammy, Vonholle Ann, Ocampo Rene, Bulik Cynthia Disordered Eating and Substance Use Among a Female Sample of Mexican Adolescents. SUBSTANCE USE \& MISUSE. 2011;46(4):523-534.

2544. Altinyazar Vesile, Maner Fulya Eating disorders and psychosis. ANADOLU PSIKIYATRI DERGISI-ANATOLIAN JOURNAL OF PSYCHIATRY. 2014;15(1):84-88.

2545. Vilela Darlene, Fonseca Pamela, Pinto Sonia, Bressan Josefina Influence of dietary patterns on the metabolically healthy obesity phenotype: A systematic review. NUTRITION METABOLISM AND CARDIOVASCULAR DISEASES. 2021;31(10):2779-2791.

2546. Marks Andrea The evolution of our understanding and treatment of eating disorders over the past 50 years. JOURNAL OF CLINICAL PSYCHOLOGY. 2019;75(8, SI):1380-1391.

2547. Musiat Peter, Conrod Patricia, Treasure Janet, Tylee Andre, Williams Chris, Schmidt Ulrike Targeted Prevention of Common Mental Health Disorders in University Students: Randomised Controlled Trial of a Transdiagnostic Trait-Focused Web-Based Intervention.

PLOS ONE. 2014;9(4):.

2548. Desai AM, Perrone R, Dwyer J Eating disorder in a hemodialysis patient: Case report. NUTRITION REVIEWS. 2005;63(12, 1):423-426.

2549. Conceicao Eva, Orcutt Molly, Mitchell James, Engel Scott, LaHaise Kim, Jorgensen Michelle, Woodbury Kara, Hass Naomi, Garcia Luis, Wonderlich Stephen Eating disorders after bariatric surgery: A case series. INTERNATIONAL JOURNAL OF EATING DISORDERS. 2013;46(3):274-279.

2550. Blum Kenneth, Thanos Panayotis, Wang Gene, Bowirrat Abdalla, Gomez Luis, Baron David, Jalali Rehan, Gondre-Lewis Marjorie, Gold Mark Dopaminergic and other genes related to reward induced overeating, Bulimia, Anorexia Nervosa, and Binge eating. EXPERT REVIEW OF PRECISION MEDICINE AND DRUG DEVELOPMENT. 2022;7(1):79-95.

2551. Pinhas Leora, Morris Anne, Crosby Ross, Katzman Debra Incidence and Age-Specific Presentation of Restrictive Eating Disorders in Children A Canadian Paediatric Surveillance Program Study. ARCHIVES OF PEDIATRICS \& ADOLESCENT MEDICINE. 2011;165(10):895-899.

2552. Edmonds LK, Mosley BJ, Admiraal AJ, Olds RJ, Romans SE, Silverstone T, Walsh AES Familial bipolar disorder: preliminary results from the Otago Familial Bipolar Genetic Study. AUSTRALIAN AND NEW ZEALAND JOURNAL OF PSYCHIATRY. 1998;32(6):823-829.

2553. Tripp B, Ludvik B Novel anti-obesity drugs. ACTA MEDICA AUSTRIACA. 2004;31(4):133-138.

2554. Woods S Untreated recovery from eating disorders. ADOLESCENCE. 2004;39(154):361-371.

2555. Linardon Jake, Messer Mariel, Lee Sohee, Rosato John Perspectives of e-health interventions for treating and preventing eating disorders: descriptive study of perceived advantages and barriers, help-seeking intentions, and preferred functionality. EATING AND WEIGHT DISORDERS-STUDIES ON ANOREXIA BULIMIA AND OBESITY. 2021;26(4):1097-1109.

2556. Steinglass Joanna, Sysko Robyn, Glasofer Deborah, Albano Anne, Simpson H., Walsh B. Rationale for the application of Exposure and Response Prevention to the Treatment of Anorexia Nervosa. INTERNATIONAL JOURNAL OF EATING DISORDERS. 2011;44(2):134-141.

2557. Dakanalis Antonios, Timko C., Carra Giuseppe, Clerici Massimo, Zanetti M., Riva Giuseppe, Caccialanza Riccardo Testing the original and the extended dual-pathway model of lack of control over eating in adolescent girls. A two-year longitudinal study. APPETITE. 2014;82():180-193.

2558. Fairburn CG, Welch SL, Doll HA, Davies BA, OConnor ME Risk factors for bulimia nervosa - A community-based case-control study. ARCHIVES OF GENERAL PSYCHIATRY. 1997;54(6):509-517.

2559. Laghi Fiorenzo, Pompili Sara, Baumgartner Emma, Baiocco Roberto The role of sensation seeking and motivations for eating in female and male adolescents who binge eat. EATING BEHAVIORS. 2015;17():119-124.

2560. Donini Lorenzo, Barrada Juan, Barthels Friederike, Dunn Thomas, Babeau Camille, Brytek-Matera Anna, Cena Hellas, Cerolini Silvia, Cho Hye-hyun, Coimbra Maria, Cuzzolaro Massimo, Ferreira Claudia, Galfano Valeria, Grammatikopoulou Maria, Hallit Souheil, Haman Linn, Hay Phillipa, Jimbo Masahito, Lasson Clotilde, Lindgren Eva-Carin, McGregor Renee, Minnetti Marianna, Mocini Edoardo, Obeid Sahar, Oberle Crystal, Onieva-Zafra Maria-Dolores, Opitz Marie-Christine, Parra-Fernandez Maria-Laura, Pietrowsky Reinhard, Plasonja Natalija, Poggiogalle Eleonora, Rigo Adrien, Rodgers Rachel, Roncero Maria, Saldana Carmina, Segura-Garcia Cristina, Setnick Jessica, Shin Ji-Yeon, Spitoni Grazia, Strahler Jana, Stroebele-Benschop Nanette, Todisco Patrizia, Vacca Mariacarolina, Valente Martina, Varga Marta, Zagaria Andrea, Zickgraf Hana, Lombardo Caterina A consensus document on definition and diagnostic criteria for orthorexia nervosa. EATING AND WEIGHT DISORDERS-STUDIES ON ANOREXIA BULIMIA AND OBESITY. 2022;27(8):3695-3711.

2561. LEAL L, WEISE SM, DODD DK THE RELATIONSHIP BETWEEN GENDER, SYMPTOMS OF BULIMIA, AND TOLERANCE FOR STRESS. ADDICTIVE BEHAVIORS. 1995;20(1):105-109.

2562. Bulik CM, Sullivan PF, Joyce PR, Carter FA, McIntosh VV Predictors of 1-year treatment outcome in bulimia nervosa. COMPREHENSIVE PSYCHIATRY. 1998;39(4):206-214.

2563. Green Sarah, Hicks Allison, Hilsendager Chelsea, Bauer Maureen, Frank Guido An adolescent girl with signs and symptoms of anaphylaxis and negative immunologic workup: a case report. JOURNAL OF MEDICAL CASE REPORTS. 2020;14(1):.

2564. Cella Stefania, Iannaccone Mara, Cotrufo Paolo Influence of gender role orientation (masculinity versus femininity) on body satisfaction and eating attitudes in homosexuals, heterosexuals and transsexuals. EATING AND WEIGHT DISORDERS-STUDIES ON ANOREXIA BULIMIA AND OBESITY. 2013;18(2):115-124.

2565. Pullmer Rachelle, Coelho Jennifer, Zaitsoff Shannon Kindness begins with yourself: The role of self-compassion in adolescent body satisfaction and eating pathology. INTERNATIONAL JOURNAL OF EATING DISORDERS. 2019;52(7):809-816.

2566. Kally Zina, Cumella Edward 100 Midlife Women With Eating Disorders: A Phenomenological Analysis of Etiology. JOURNAL OF GENERAL PSYCHOLOGY. 2008;135(4, SI):359-377.

2567. Scardina G., Messina P. Good Oral Health and Diet. JOURNAL OF BIOMEDICINE AND BIOTECHNOLOGY. 2012;():.

2568. Yazkan Gullu, Ugurlu Nezihe The relationship between orthorexia nervosa tendencies and OCD symptoms in healthcare professionals. JOURNAL OF PSYCHIATRIC NURSING. 2022;13(1):49-56.

2569. Hartmann Andrea, Staufenbiel Thomas, Bielefeld Lukas, Buhlmann Ulrike, Heinrichs Nina, Martin Alexandra, Ritter Viktoria, Kollei Ines, Grocholewski Anja An empirically derived recommendation for the classification of body dysmorphic disorder: Findings from structural equation modeling. PLOS ONE. 2020;15(6):.

2570. Uher R, Murphy T, Friederich HC, Dalgleish T, Brammer MJ, Giampietro V, Phillips ML, Andrew CM, Ng VW, Williams SCR, Campbell IC, Treasure J Functional neuroanatomy of body shape perception in healthy and eating-disordered women. BIOLOGICAL PSYCHIATRY. 2005;58(12):990-997.

2571. Brewerton Timothy, Gavidia Ismael, Suro Giulia, Perlman Molly Eating disorder onset during childhood is associated with higher trauma dose, provisional PTSD, and severity of illness in residential treatment. EUROPEAN EATING DISORDERS REVIEW. 2022;30(3):267-

277.

2572. Avila Asuncion, Cardona Xavier, Martin-Baranera Montse, Bello Juan, Sastre Francesc Impulsive and compulsive behaviors in Parkinson's disease: A one-year follow-up study. JOURNAL OF THE NEUROLOGICAL SCIENCES. 2011;310(1-2, SI):197-201.

2573. Fulkerson Jayne, Story Mary, Mellin Alison, Leffert Nancy, Neumark-Sztainer Dianne, French Simone Family dinner meal frequency and adolescent development: Relationships with developmental assets and high-risk behaviors. JOURNAL OF ADOLESCENT HEALTH. 2006;39(3):337-345.

2574. Ximei Chen, Yijun Luo, Hong Chen Friendship quality and adolescents' intuitive eating: A serial mediation model and the gender difference. ACTA PSYCHOLOGICA SINICA. 2020;52(4):485-496.

2575. Asghar Ayesha, Shah Aresha, Hussain Abbas, Tahir Amber, Asghar Hajra Frequency of Pre-obesity and Obesity in Medical Students of Karachi and the Predisposing Lifestyle Habits. CUREUS. 2019;11(1):.

2576. Melissa Rizk, Lama Mattar, Laurence Kern, Sylvie Berthoz, Jeanne Duclos, Odile Viltart, Nathalie Godart Physical Activity in Eating Disorders: A Systematic Review. NUTRIENTS. 2020;12(1):.

2577. D'Addario C., Di Bonaventura M., Pucci M., Romano A., Gaetani S., Ciccocioppo R., Cifani C., Maccarrone M. Endocannabinoid signaling and food addiction. NEUROSCIENCE AND BIOBEHAVIORAL REVIEWS. 2014;47():203-224.

2578. VITOUSEK K, MANKE F PERSONALITY-VARIABLES AND DISORDERS IN ANOREXIA-NERVOSA AND BULIMIA-NERVOSA. JOURNAL OF ABNORMAL PSYCHOLOGY. 1994;103(1):137-147.

2579. Stice E, Agras WS Subtyping bulimic women along dietary restraint and negative affect dimensions. JOURNAL OF CONSULTING AND CLINICAL PSYCHOLOGY. 1999;67(4):460-469.

2580. Perkins S., Schmidt U., Eisler I., Treasure J., Berelowitz M., Dodge E., Frost S., Jenkins M., Johnson-Sabine E., Keville S., Murphy R., Robinson P., Winn S., Yi I. Motivation to change

in recent onset and long-standing bulimia nervosa: Are there differences?. EATING AND WEIGHT DISORDERS-STUDIES ON ANOREXIA BULIMIA AND OBESITY. 2007;12(2):61-69.

2581. Centeno-leguia Dercy, Ango-bedrinana Jimmy, Mejia Christian Sedentary life and unhealthy eating patterns on the anthropometry of peruvian young lives: 2009 - 2016. NUTRICION CLINICA Y DIETETICA HOSPITALARIA. 2022;42(4):52-60.

2582. Bagherniya Mohammad, Darani Firoozeh, Sharma Manoj, Allipour-Birgani Ramesh, Taghipour Ali, Safarian Mohammad Qualitative Study to Determine Stressors Influencing Dietary and Physical Activity Behaviors of Overweight and Obese Adolescents in Iran. INTERNATIONAL JOURNAL OF PREVENTIVE MEDICINE. 2019;10():.

2583. Wigren M, Hansen S ADHD symptoms and insistence on sameness in Prader-Willi syndrome. JOURNAL OF INTELLECTUAL DISABILITY RESEARCH. 2005;49(6):449-456.

2584. Austin S., Yu Kimberly, Tran Alvin, Mayer Beth Research-to-policy translation for prevention of disordered weight and shape control behaviors: A case example targeting dietary supplements sold for weight loss and muscle building. EATING BEHAVIORS. 2017;25(SI):9-14.

2585. Culbert Kristen, Sinclair Elaine, Hildebrandt Britny, Klump Kelly, Sisk Cheryl Perinatal Testosterone Contributes to Mid-to-Post Pubertal Sex Differences in Risk for Binge Eating in Male and Female Rats. JOURNAL OF ABNORMAL PSYCHOLOGY. 2018;127(2):239-250.

2586. Tong J, Miao SJ, Wang J, Zhang JJ, Wu HM, Li T, Hsu LKG Five cases of male eating disorders in central China. INTERNATIONAL JOURNAL OF EATING DISORDERS. 2005;37(1):72-75.

2587. Romano Kelly, Lipson Sarah, Beccia Ariel, Quatromoni Paula, Murgueitio Jose Disparities in eating disorder symptoms and mental healthcare engagement prior to and following the onset of the COVID-19 pandemic: Findings from a national study of US college students. INTERNATIONAL JOURNAL OF EATING DISORDERS. 2023;56(1, SI):203-215.

2588. Hilbert Anja, Tuschen-Caffier Brunna, Karwautz Andreas, Niederhofer Helmut, Munsch Simone Eating disorder examination-questionnaire: Psychometric properties of the German version. DIAGNOSTICA. 2007;53(3):144-154.

2589. Reyes-Rodriguez Mae, Franko Debra, Matos-Lamourt Anguelique, Bulik Cynthia, Von Holle Ann, Camara-Fuentes Luis, Rodriguez-Anglero Dianisa, Cervantes-Lopez Sarah, Suarez-Torres Alba Eating Disorder Symptomatology: Prevalence Among Latino College Freshmen Students. JOURNAL OF CLINICAL PSYCHOLOGY. 2010;66(6):666-679.

2590. Cohen Rachel, Newton-John Toby, Slater Amy `Selfie'-objectification: The role of selfies in self-objectification and disordered eating in young women. COMPUTERS IN HUMAN BEHAVIOR. 2018;79():68-74.

2591. COOKE RA, CHAMBERS JB ANOREXIA-NERVOSA AND THE HEART. BRITISH JOURNAL OF HOSPITAL MEDICINE. 1995;54(7):313-317.

2592. Hartmann Andrea, Czaja Julia, Rief Winfried, Hilbert Anja Psychosocial risk factors of loss of control eating in primary school children: A retrospective case-control study. INTERNATIONAL JOURNAL OF EATING DISORDERS. 2012;45(6):751-758.

2593. Loth Katie, Neumark-Sztainer Dianne, Croll Jillian Informing Family Approaches to Eating Disorder Prevention: Perspectives of Those Who Have Been There. INTERNATIONAL JOURNAL OF EATING DISORDERS. 2009;42(2):146-152.

2594. Holmes Millicent, Fuller-Tyszkiewicz Matthew, Skouteris Helen, Broadbent Jaclyn Improving Prediction of Binge Episodes by Modelling Chronicity of Dietary Restriction. EUROPEAN EATING DISORDERS REVIEW. 2014;22(6):405-411.

2595. Schoenfelder Erin, Kollins Scott Topical Review: ADHD and Health-Risk Behaviors: Toward Prevention and Health Promotion. JOURNAL OF PEDIATRIC PSYCHOLOGY. 2016;41(7):735-740.

2596. Lucas Nicolas, Legrand Romain, Bole-Feysot Christine, Breton Jonathan, Coeffier Moise, Akkermann Kirsti, Jarv Anu, Harro Jaanus, Dechelotte Pierre, Fetissov Serguei Immunoglobulin G modulation of the melanocortin 4 receptor signaling in obesity and eating disorders. TRANSLATIONAL PSYCHIATRY. 2019;9():.

2597. Boutelle Kerri, Braden Abby, Knatz-Peck Stephanie, Anderson Leslie, Rhee Kyung An open trial targeting emotional eating among adolescents with overweight or obesity. EATING DISORDERS. 2018;26(1, SI):79-91.

2598. Klein Kelly, Brown Tiffany, Kennedy Grace, Keel Pamela Examination of parental dieting and comments as risk factors for increased drive for thinness in men and women at 20-year follow-up. INTERNATIONAL JOURNAL OF EATING DISORDERS. 2017;50(5):490-497.

2599. Keck PE, McElroy SL Bipolar disorder, obesity, and pharmacotherapy-associated weight gain. JOURNAL OF CLINICAL PSYCHIATRY. 2003;64(12):1426-1435.

2600. Nastaskin Robyn, Fiocco Alexandra A survey of diet self-efficacy and food intake in students with high and low perceived stress. NUTRITION JOURNAL. 2015;14():.

2601. Lebrun CM, Rumball JS Female athlete triad. SPORTS MEDICINE AND ARTHROSCOPY REVIEW. 2002;10(1):23-32.

2602. Bioulac B., Debre P., Hauw J., Galibert F., Biol-Genetique-Technolo Commission Biomarkers in psychiatric disorders. BULLETIN DE L ACADEMIE NATIONALE DE MEDECINE. 2020;204(5):444-454.

2603. Minian Nadia, deruiter Wayne, Lingam Mathangee, Corrin Tricia, Dragonetti Rosa, Manson Heather, Taylor Valerie, Zawertailo Laurie, Ebnahmady Arezoo, Melamed Osnat, Rodak Terri, Hahn Margaret, Selby Peter The effects of interventions targeting multiple health behaviors on smoking cessation outcomes: a rapid realist review protocol. SYSTEMATIC REVIEWS. 2018;7():.

2604. Mills IH, Park GR, Manara AR, Merriman RJ Treatment of compulsive behaviour in eating disorders with intermittent ketamine infusions. QJM-MONTHLY JOURNAL OF THE ASSOCIATION OF PHYSICIANS. 1998;91(7):493-503.

2605. Ward Zachary, Rodriguez Patricia, Wright Davene, Austin S., Long Michael Estimation of Eating Disorders Prevalence by Age and Associations With Mortality in a Simulated Nationally Representative US Cohort. JAMA NETWORK OPEN. 2019;2(10):.

2606. Domine Frangoise, Berchtold Andre, Akre Christina, Michaud Pierre-Andre, Suris Joan-Carles Disordered Eating Behaviors: What About Boys?. JOURNAL OF ADOLESCENT HEALTH. 2009;44(2):111-117.

2607. Rosa Ana, Diniz Juliana, Fossaluza Victor, Torres Albina, Fontenelle Leonardo, De Mathis Alice, Rosario Maria, Miguel Euripedes, Shavitt Roseli Clinical correlates of social adjustment in patients with obsessive-compulsive disorder. JOURNAL OF PSYCHIATRIC RESEARCH. 2012;46(10):1286-1292.

2608. Bejarano Carolina, Hesse Daryl, Cushing Christopher Hedonic Appetite, Affect, and Loss of Control Eating: Macrotemporal and Microtemporal Associations in Adolescents. JOURNAL OF PEDIATRIC PSYCHOLOGY. 2023;48(5):448-457.

2609. Tengia-Kessy Anna, Killenga Jackline Prevalence of excess body weight and associated factors among secondary school adolescent girls in northern Tanzania: a cross-sectional study. PAN AFRICAN MEDICAL JOURNAL. 2020;37():.

2610. Rabat Y., Berthoz S., Sibon I. Is addictive-like eating an overlooked stroke risk factor? A study case. EATING AND WEIGHT DISORDERS-STUDIES ON ANOREXIA BULIMIA AND OBESITY. 2021;26(8):2801-2806.

2611. Gerlach G., Loeber S., Herpertz S. Personality disorders and obesity: a systematic review. OBESITY REVIEWS. 2016;17(8):691-723.

2612. Chang Cindy, Putukian Margot, Aerni Giselle, Diamond Alex, Hong Eugene, Ingram Yvette, Reardon Claudia, Wolanin Andrew Mental Health Issues and Psychological Factors in Athletes: Detection, Management, Effect on Performance, and Prevention: American Medical Society for Sports Medicine Position Statement. CLINICAL JOURNAL OF SPORT MEDICINE. 2020;30(2):E61-E87.

2613. Lee Jordan, Prabhakaran Sowmya, Wilkie Bruce, Peng Calvin, Thomson Benjamin Massive gastric distension due to bulimia nervosa: a hotpot emergency. JOURNAL OF SURGICAL CASE REPORTS. 2022;2022(8):.

2614. Nestadt G, Addington A, Samuels J, Liang KY, Bienvenu OJ, Riddle M, Grados M, Hoehn-Saric R, Cullen B The identification of OCD-related subgroups based on comorbidity. BIOLOGICAL PSYCHIATRY. 2003;53(10):914-920.

2615. Burns Maree, Gavey Nicola `Healthy weight' at what cost? `Bulimia' and a discourse of weight control. JOURNAL OF HEALTH PSYCHOLOGY. 2004;9(4):549-565.

2616. Herrmann Kristin, Kaluscha Rainer, Liebert Alex, Spohrs Jennifer, Guendel Harald, Wietersheim Joern First onset of treatment of patients with eating disorders and treatment course: Results of data from a German health insurance company. EUROPEAN EATING DISORDERS REVIEW. 2022;30(6):787-796.

2617. Eynde Frederique, Suda Masashi, Broadbent Hannah, Guillaume Sebastien, Eynde Magali, Steiger Howard, Israel Mimi, Berlim Marcelo, Giampietro Vincent, Simmons Andrew, Treasure Janet, Campbell Iain, Schmidt Ulrike Structural Magnetic Resonance Imaging in Eating Disorders: A Systematic Review of Voxel-Based Morphometry Studies. EUROPEAN EATING DISORDERS REVIEW. 2012;20(2):94-105.

2618. Larson Nicole, Davey Cynthia, Caspi Caitlin, Kubik Martha, Nanney Marilyn School-Based Obesity-Prevention Policies and Practices and Weight-Control Behaviors among Adolescents. JOURNAL OF THE ACADEMY OF NUTRITION AND DIETETICS. 2017;117(2):204-213.

2619. Abbate-Daga G., Piero A., Rigardetto R., Gandione M., Gramaglia C., Fassino S. Clinical, psychological and personality features related to age of onset of anorexia nervosa. PSYCHOPATHOLOGY. 2007;40(4):261-268.

2620. Schwitzer AM, Bergholz K, Dore T, Salimi L Eating disorders among college women: Prevention, education, and treatment responses. JOURNAL OF AMERICAN COLLEGE HEALTH. 1998;46(5):199-207.

2621. Molinar-Toribio Eunice, Perez-Jimenez Jara, Ramos-Romero Sara, Gomez Livia, Taltavull Nuria, Rosa Nogues Maria, Adeva Alberto, Jauregui Olga, Joglar Jesus, Clapes Pere, Lluis Torres Josep D-Fagomine attenuates metabolic alterations induced by a high-energy-dense diet in rats. FOOD \& FUNCTION. 2015;6(8):2614-2619.

2622. Mathis Maria, Alvarenga Pedro, Funaro Guilherme, Torresan Ricardo, Moraes Ivanil, Torres Albina, Zilberman Monica, Hounie Ana Gender differences in obsessive-compulsive disorder: a literature review. REVISTA BRASILEIRA DE PSIQUIATRIA. 2011;33(4):390-399.

2623. Pechmann Cornelia, Catlin Jesse, Zheng Yu Facilitating Adolescent Well-Being: A Review of the Challenges and Opportunities and the Beneficial Roles of Parents, Schools, Neighborhoods, and Policymakers. JOURNAL OF CONSUMER PSYCHOLOGY. 2020;30(1):149-177.

2624. STEINHAUSEN HC, SEIDEL R BERLIN FOLLOW-UP-STUDY OF EATING DISORDERS IN ADOLESCENCE .3. EVALUATION AND PROGNOSIS. NERVENARZT. 1994;65(1):35-40.

2625. Huang Cong, Momma Haruki, Cui Yufei, Chujo Masahiko, Otomo Atsushi, Sugiyama Shota, Ren Zhongyu, Niu Kaijun, Nagatomi Ryoichi Independent and combined relationship of habitual unhealthy eating behaviors with depressive symptoms: A prospective study. JOURNAL OF EPIDEMIOLOGY. 2017;27(1):42-47.

2626. Hoover Lindzey, Yu Hayley, Cummings Jenna, Ferguson Stuart, Gearhardt Ashley Co-Occurrence of Food Addiction, Obesity, Problematic Substance Use, and Parental History of Problematic Alcohol Use. PSYCHOLOGY OF ADDICTIVE BEHAVIORS. 2022;():.

2627. Mehler Philip, Anderson Kristin, Bauschka Maryrose, Cost Jeana, Farooq Asma Emergency room presentations of people with anorexia nervosa. JOURNAL OF EATING DISORDERS. 2023;11(1):.

2628. Warren CS, Gleaves DH, Cepeda-Benito A, Fernandez MD Ethnicity as a protective factor against internalization of a thin ideal and body dissatisfaction. INTERNATIONAL JOURNAL OF EATING DISORDERS. 2005;37(3):241-249.

2629. Turnbull S, Ward A, Treasure J, Jick H, Derby L The demand for eating disorder care - An epidemiological study using the general practice research database. BRITISH JOURNAL OF PSYCHIATRY. 1996;169(6):705-712.

2630. Kumar Maya, Argo Taylor, Chang Jane, Cifra Nicole, Docter Alicia, Galagali Preeti, Kapphahn Cynthia, Key Janice, Pitt Paulette, Weiss Amy Preventing Nutritional Disorders in Adolescents by Encouraging a Healthy Relationship With Food. JOURNAL OF ADOLESCENT HEALTH. 2020;67(6):875-879.

2631. Jenkinson Paul, Taylor Lauren, Laws Keith Self-reported interoceptive deficits in eating disorders: A meta-analysis of studies using the eating disorder inventory. JOURNAL OF PSYCHOSOMATIC RESEARCH. 2018;110():38-45.

2632. Strangio Annamaria, Rinaldi Lucio, Monniello Gianluigi, Sisti Leuconoe, Waure Chiara, Janiri Luigi The Effect of Abuse History on Adolescent Patients with Feeding and Eating Disorders Treated through Psychodynamic Therapy: Comorbidities and Outcome. FRONTIERS IN PSYCHIATRY. 2017;8():.

2633. MUNKJORGENSEN P, MOLLERMADSEN S, NIELSEN S, NYSTRUP J INCIDENCE OF EATING DISORDERS IN PSYCHIATRIC-HOSPITALS AND WARDS IN DENMARK, 1970-1993. ACTA PSYCHIATRICA SCANDINAVICA. 1995;92(2):91-96.

2634. Hahn Samantha, Hazzard Vivienne, Larson Nicole, Klein Laura, Loth Katie, Neumark-Sztainer Dianne Correlates of weight-related self-monitoring application use during emerging adulthood in a population-based sample. EATING AND WEIGHT DISORDERS-STUDIES ON ANOREXIA BULIMIA AND OBESITY. 2022;27(6):2107-2119.

2635. Osborne Emma, Atkinson Melissa Effects of Decentering and Non-judgement on Body Dissatisfaction and Negative Affect Among Young Adult Women. MINDFULNESS. 2022;13(3):615-626.

2636. Steinglass Joanna, Albano Anne, Simpson H., Carpenter Kenneth, Schebendach Janet, Attia Evelyn Fear of food as a treatment target: Exposure and response prevention for anorexia nervosa in an open series. INTERNATIONAL JOURNAL OF EATING DISORDERS. 2012;45(4):615-621.

2637. Holle Ann, Pinheiro Andrea, Thornton Laura, Klump Kelly, Berrettini Wade, Brandt Harry, Crawford Steven, Crow Scott, Fichter Manfred, Halmi Katherine, Johnson Craig, Kaplan Allan, Keel Pamela, LaVia Maria, Mitchell James, Strober Michael, Woodside D., Kaye Walter, Bulik Cynthia Temporal patterns of recovery across eating disorder subtypes. AUSTRALIAN AND NEW ZEALAND JOURNAL OF PSYCHIATRY. 2008;42(2):108-117.

2638. Irving LM, Neumark-Sztainer D Integrating the prevention, of eating disorders and obesity: Feasible or futile?. PREVENTIVE MEDICINE. 2002;34(3):299-309.

2639. Griffiths RA, Beumont PJV, Russell J, Schotte D, Thornton C, Touyz S, Varano P Sociocultural attitudes towards appearance in dieting disordered and nondieting disordered subjects. EUROPEAN EATING DISORDERS REVIEW. 1999;7(3):193-203.

2640. Feinson Marjorie, Hornik-Lurie Tzipi Body Dissatisfaction and the Relevance of Religiosity: A Focus on Ultra-Orthodox Jews in a Community Study of Adult Women. CLINICAL SOCIAL WORK JOURNAL. 2016;44(1, SI):87-97.

2641. Xie Weiguang, Lou Hongliang Implementation of Key Technologies for a Healthy Food Culture Recommendation System Using Internet of Things. MOBILE INFORMATION

SYSTEMS. 2022;2022():.

2642. Bjornsson Andri, Didie Elizabeth, Grant Jon, Menard William, Stalker Emily, Phillips Katharine. Age at onset and clinical correlates in body dysmorphic disorder. COMPREHENSIVE PSYCHIATRY. 2013;54(7):893-903.

2643. Neumark-Sztainer Dianne, Cook-Cottone Catherine, Tylka Tracy, Cox Anne Introduction to the special issue on yoga and positive embodiment: a note from the editors on how we got here. EATING DISORDERS. 2020;28(4, SI):309-314.

2644. Troop NA, Allan S, Treasure JL, Katzman M Social comparison and submissive behaviour in eating disorder patients. PSYCHOLOGY AND PSYCHOTHERAPY-THEORY RESEARCH AND PRACTICE. 2003;76(3):237-249.

2645. Stice Eric, Johnson Sarah, Turgon Roxane Eating Disorder Prevention. PSYCHIATRIC CLINICS OF NORTH AMERICA. 2019;42(2, 2):309+.

2646. KOEPP W, SCHILDBACH S, SCHMAGER C, ROHNER R BORDERLINE DIAGNOSIS AND SUBSTANCE-ABUSE IN FEMALE-PATIENTS WITH EATING DISORDERS. INTERNATIONAL JOURNAL OF EATING DISORDERS. 1993;14(1):107-110.

2647. O'Sullivan Sean, Wu Kit, Politis Marios, Lawrence Andrew, Evans Andrew, Bose Subrata, Djamshidian Atbin, Lees Andrew, Piccini Paola Cue-induced striatal dopamine release in Parkinson's disease-associated impulsive-compulsive behaviours. BRAIN. 2011;134(4):969-978.

2648. Sanchez-Ortiz V., Munro C., Stahl D., House J., Startup H., Treasure J., Williams C., Schmidt U. A randomized controlled trial of internet-based cognitive-behavioural therapy for bulimia nervosa or related disorders in a student population. PSYCHOLOGICAL MEDICINE. 2011;41(2):407-417.

2649. Yoder Ruth, MacNeela Padraig, Conway Ronan, Heary Caroline How Do Individuals Develop Alcohol Use Disorder After Bariatric Surgery? A Grounded Theory Exploration. OBESITY SURGERY. 2018;28(3):717-724.

2650. Sundgot-Borgen J., Torstveit M. Aspects of disordered eating continuum in elite high-intensity sports. SCANDINAVIAN JOURNAL OF MEDICINE \& SCIENCE IN SPORTS.

2010;20(2):112-121.

2651. Puttevils Louise, Vanderhasselt Marie-Anne, Horczak Paula, Vervaet Myriam Differences in the use of emotion regulation strategies between anorexia and bulimia nervosa: A systematic review and meta-analysis. COMPREHENSIVE PSYCHIATRY. 2021;109():.

2652. Van Vlierberghe Leen, Braet Caroline, Goossens Lien, Rosseel Yves, Mels Saskia Psychological disorder, symptom severity and weight loss in inpatient adolescent obesity treatment. INTERNATIONAL JOURNAL OF PEDIATRIC OBESITY. 2009;4(1):36-44.

2653. Chang Cindy, Putukian Margot, Aerni Giselle, Diamond Alex, Hong Eugene, Ingram Yvette, Reardon Claudia, Wolanin Andrew American Medical Society for Sports Medicine Position Statement: Mental Health Issues and Psychological Factors in Athletes: Detection, Management, Effect on Performance, and Prevention-Executive Summary. CLINICAL JOURNAL OF SPORT MEDICINE. 2020;30(2):91-95.

2654. Paans Nadine, Bot Mariska, Brouwer Ingeborg, Visser Marjolein, Gili Margalida, Roca Miguel, Hegerl Ulrich, Kohls Elisabeth, Owens Matthew, Watkins Ed, Penninx Brenda, Investig MooDFOOD Effects of food-related behavioral activation therapy on eating styles, diet quality and body weight change: Results from the MooDFOOD Randomized Clinical Trial. JOURNAL OF PSYCHOSOMATIC RESEARCH. 2020;137():.

2655. Burnette C., Davies Alexandria, Mazzeo Suzanne Lessons learned from a pilot intuitive eating intervention for college women delivered through group and guided self-help: qualitative and process data. EATING DISORDERS. 2022;30(4):385-410.

2656. Weintraub Daniel Impulse control disorders in Parkinson's disease: prevalence and possible risk factors. PARKINSONISM \& RELATED DISORDERS. 2009;15(3):S110-S113.

2657. Andreescu Cristina, Pascual-Leone Antonio, Nardone Stephanie Disordered eating is related to deficits in emotional processing: A correlational study with a subclinical sample. JOURNAL OF AFFECTIVE DISORDERS. 2023;325():337-345.

2658. Goldfield Gary, Moore Ceri, Henderson Katherine, Buchholz Annick, Obeid Nicole, Flament Martine The relation between weight-based teasing and psychological adjustment in adolescents. PAEDIATRICS \& CHILD HEALTH. 2010;15(5):283-288.

2659. Russo Jade, Brennan Leah, Walkley Jeff, Fraser Steve, Greenway Kate Psychosocial Predictors of Eating Disorder Risk in Overweight and Obese Treatment-Seeking Adolescents. BEHAVIOUR CHANGE. 2011;28(3):111-127.

2660. Stice E, Agras WS Predicting onset and cessation of bulimic behaviors during adolescence: A longitudinal grouping analysis. BEHAVIOR THERAPY. 1998;29(2):257-276.

2661. Varela-Mato Veronica, Cancela Jose, Ayan Carlos, Martin Vicente, Molina Antonio Lifestyle and Health among Spanish University Students: Differences by Gender and Academic Discipline. INTERNATIONAL JOURNAL OF ENVIRONMENTAL RESEARCH AND PUBLIC HEALTH. 2012;9(8):2728-2741.

2662. Williamson DA, Gleaves DH, Stewart TM Categorical versus dimensional models of eating disorders: An examination of the evidence. INTERNATIONAL JOURNAL OF EATING DISORDERS. 2005;37(1):1-10.

2663. MORENO AC, CALO JJP, ZARAGOZA CL, PINERO MV, HORCAJADAS FA PSYCHOPHARMACOLOGICAL TREATMENT OF BULIMIA-NERVOSA. ACTAS LUSO-ESPANOLAS DE NEUROLOGIA PSIQUIATRIA Y CIENCIAS AFINES. 1993;21(6):211-220.

2664. Martinez-Gonzalez MA, Gual P, Lahortiga F, Alonso Y, Irala-Estevez J, Cervera S Parental factors, mass media influences, and the onset of eating disorders in a prospective population-based cohort. PEDIATRICS. 2003;111(2):315-320.

2665. Hicks Caitlin, DeMarsh Samantha, Singh Harjoat, Gillespie Laura, Worley Sarah, Rome Ellen Knowledge about various contraceptive methods in young women with and without eating disorders. INTERNATIONAL JOURNAL OF EATING DISORDERS. 2013;46(2):171-176.

2666. Atkinson Melissa, Diedrichs Phillippa Examining the efficacy of video-based microinterventions for improving risk and protective factors for disordered eating among young adult women. INTERNATIONAL JOURNAL OF EATING DISORDERS. 2021;54(5):708-720.

2667. Wilksch Simon, O'Shea Anne, Wade Tracey Depressive symptoms, alcohol and other drug use, and suicide risk: Prevention and treatment effects from a two-country online eating disorder risk reduction trial. INTERNATIONAL JOURNAL OF EATING DISORDERS. 2019;52(2):132-141.

2668. FAIRBURN CG, JONES R, PEVELER RC, HOPE RA, OCONNOR M PSYCHOTHERAPY AND BULIMIA-NERVOSA - LONGER-TERM EFFECTS OF INTERPERSONAL PSYCHOTHERAPY, BEHAVIOR-THERAPY, AND COGNITIVE-BEHAVIOR THERAPY. ARCHIVES OF GENERAL PSYCHIATRY. 1993;50(6):419-428.

2669. Passel Boris, Danner Unna, Dingemans Alexandra, Aarts Emmeke, Sternheim Lot, Becker Eni, Elburg Annemarie, Furth Eric, Hendriks Gert-Jan, Cath Danielle Cognitive Remediation Therapy Does Not Enhance Treatment Effect in Obsessive-Compulsive Disorder and Anorexia Nervosa: A Randomized Controlled Trial. PSYCHOTHERAPY AND PSYCHOSOMATICS. 2020;89(4):228-241.

2670. BULIK CM, SULLIVAN PF, MCKEE M, WELTZIN TE, KAYE WH CHARACTERISTICS OF BULIMIC WOMEN WITH AND WITHOUT ALCOHOL-ABUSE. AMERICAN JOURNAL OF DRUG AND ALCOHOL ABUSE. 1994;20(2):273-283.

2671. Guldstrand Marie, Simberg Caroline High-fat diets: healthy or unhealthy?. CLINICAL SCIENCE. 2007;113(9-10):397-399.

2672. Scaglioni Silvia, Arrizza Chiara, Vecchi Fiammetta, Tedeschi Sabrina Determinants of children's eating behavior. AMERICAN JOURNAL OF CLINICAL NUTRITION. 2011;94(6):2006S-2011S.

2673. Jancso Z., Marton H., Simay A., Ujhelyi I., Ilyes I. THE EFFECT OF EATING HABITS ON CARDIOVASCULAR RISK FACTORS AND THE ASSESSED CARDIOVASCULAR RISK. ACTA ALIMENTARIA. 2011;40(2):254-261.

2674. Crow Scott The Economics of Eating Disorder Treatment. CURRENT PSYCHIATRY REPORTS. 2014;16(7):.

2675. Pace Ugo, Cacioppo Marco, Schimmenti Adriano The Moderating Role of Father's Care on the Onset of Binge Eating Symptoms Among Female Late Adolescents with Insecure Attachment. CHILD PSYCHIATRY \& HUMAN DEVELOPMENT. 2012;43(2):282-292.

2676. Steenkamp Maria, Corry Nida, Qian Meng, Li Meng, McMaster Hope, Fairbank John, Stander Valerie, Hollahan Laura, Marmar Charles Prevalence of psychiatric morbidity in United States military spouses: The Millennium Cohort Family Study. DEPRESSION AND ANXIETY. 2018;35(9):815-829.

2677. Francisco Rita, Alarcao Madalena, Narciso Isabel Assesment of risk factors in eatine disorpers. development and validation of portoguese version or Mcknigmy Risk Factor Survey IV. REVISTA IBEROAMERICANA DE DIAGNOSTICO Y EVALUACION-E AVALIACAO PSICOLOGICA. 2011;2(32):143-170.

2678. Phelps L, Sapia J, Nathanson D, Nelson L An empirically supported eating disorder prevention program. PSYCHOLOGY IN THE SCHOOLS. 2000;37(5):443-452.

2679. Preiss K., Brennan L., Clarke D. A systematic review of variables associated with the relationship between obesity and depression. OBESITY REVIEWS. 2013;14(11):906-918.

2680. Franko DL, Dorer DJ, Keel PK, Jackson S, Manzo MP, Herzog DB How do eating disorders and alcohol use disorder influence each other?. INTERNATIONAL JOURNAL OF EATING DISORDERS. 2005;38(3):200-207.

2681. Aouad Phillip, Hay Phillipa, Soh Nerissa, Touyz Stephen, Mannan Haider, Mitchison Deborah Chew and spit (CHSP) in a large adolescent sample: prevalence, impact on health-related quality of life, and relation to other disordered eating features. EATING DISORDERS. 2021;29(5):509-522.

2682. Godart NT, Perdereau F, Jeammet PH, Flament MF Comorbidity between eating disorders and anxiety disorders. First part: methodological review. ENCEPHALE-REVUE DE PSYCHIATRIE CLINIQUE BIOLOGIQUE ET THERAPEUTIQUE. 2005;31(1, 1):44-55.

2683. Gabrovsek M, Brecelj-Anderluh M, Bellodi L, Cellini E, Di Bella D, Estivill X, Fernandez-Aranda F, Freeman B, Geller F, Gratacos M, Haigh R, Hebebrand J, Hinney A, Holliday J, Hu X, Karwautz A, Nacmias B, Ribases M, Remschmidt H, Komel R, Sorbi S, Tomori M, Treasure J, Wagner G, Zhao J, Collier DA Combined family trio and case-control analysis of the COMT Val158Met polymorphism in European patients with anorexia nervosa. AMERICAN JOURNAL OF MEDICAL GENETICS PART B-NEUROPSYCHIATRIC GENETICS. 2004;124B(1):68-72.

2684. Roshandel Azam, Safavi Mahboobeh, Ghasemi Iran Prevalence of Eating Disorders among Female Students of University (Tehran - Iran). LIFE SCIENCE JOURNAL-ACTA ZHENGZHOU UNIVERSITY OVERSEAS EDITION. 2012;9(4):2822-2828.

2685. Conviser Jenny, Tierney Amanda, Nickols Riley Assessment of Athletes With Eating Disorders: Essentials for Best Practice. JOURNAL OF CLINICAL SPORT PSYCHOLOGY.

2018;12(4, SI):480-494.

2686. Diotaiuti Pierluigi, Girelli Laura, Mancone Stefania, Valente Giuseppe, Bellizzi Fernando, Misiti Francesco, Cavicchiolo Elisa Psychometric properties and measurement invariance across gender of the Italian version of the tempest self-regulation questionnaire for eating adapted for young adults. FRONTIERS IN PSYCHOLOGY. 2022;13():.

2687. Krafchek Jennifer, Kronborg Leonie Stressful Life Events Experienced by Academically High-Achieving Females Before the Onset of Disordered Eating. ROEPER REVIEW-A JOURNAL ON GIFTED EDUCATION. 2018;40(4):245-254.

2688. Bahrke U, Arends M, Bandemer-Greulich U, Dreyer B, Ropke H, Fikentscher E The incidence of pathological eating behaviour among schoolchildren in a large urban area. PSYCHOTHERAPIE PSYCHOSOMATIK MEDIZINISCHE PSYCHOLOGIE. 2003;53(1):29-34.

2689. Niederhofer Helmut Report on incidence of right-hand preference in 18 eating disordered patients. PERCEPTUAL AND MOTOR SKILLS. 2007;105(3, 1):705-706.

2690. Mascarenhas Paulo, Furtado Jose, Almeida Silvia, Ferraz Maria, Ferraz Fernando, Oliveira Pedro Pediatric Overweight, Fatness and Risk for Dyslipidemia Are Related to Diet: A Cross-Sectional Study in 9-year-old Children. NUTRIENTS. 2023;15(2):.

2691. Shisslak CM, Mays MZ, Crago M, Jirsak JK, Taltano K, Cagno C Eating and weight control behaviors among middle school girls in relationship to body weight and ethnicity. JOURNAL OF ADOLESCENT HEALTH. 2006;38(5):631-633.

2692. Foley DL, Thacker LR, Aggen SH, Neale MC, Kendler KS Pregnancy and perinatal complications associated with risks for common psychiatric disorders in a population-based sample of female twins. AMERICAN JOURNAL OF MEDICAL GENETICS. 2001;105(5):426-431.

2693. Michels Nathalie, Man Tsun, Vinck Billie, Verbeyst Laura Dietary changes and its psychosocial moderators during the university examination period. EUROPEAN JOURNAL OF NUTRITION. 2020;59(1):273-286.

2694. Mehta A, Viner R, Christie D, Newson T, Dattani MT An unusual case of an atypical eating disorder masquerading as a serious multi-systemic illness. ACTA PAEDIATRICA.

2004;93(5):714-716.

2695. Shah Ravi, Zanarini Mary Comorbidity of Borderline Personality Disorder Current Status and Future Directions. PSYCHIATRIC CLINICS OF NORTH AMERICA. 2018;41(4):583+.

2696. Mangweth-Matzek Barbara, Rupp Claudia, Hausmann Armand, Assmayr Karin, Mariacher Edith, Kemmler Georg, Whitworth Alexandra, Biebl Wilfried Never too old for eating disorders or body dissatisfaction: A community study of elderly women. INTERNATIONAL JOURNAL OF EATING DISORDERS. 2006;39(7):583-586.

2697. Rowe Sarah, Jordan Jennifer, McIntosh Virginia, Carter Frances, Frampton Chris, Bulik Cynthia, Joyce Peter Does Avoidant Personality Disorder Impact on the Outcome of Treatment for Bulimia Nervosa?. INTERNATIONAL JOURNAL OF EATING DISORDERS. 2010;43(5):420-427.

2698. Unikel C, Gomez-Peresmitre G Construct validity of an instrument to search for eating disorder risk factors in Mexican 38 women.. SALUD MENTAL. 2004;27(1):38-49.

2699. Taylor C., Kass Andrea, Trockel Mickey, Cunning Darby, Weisman Hannah, Bailey Jakki, Sinton Meghan, Aspen Vandana, Schecthman Kenneth, Jacobi Corinna, Wilfley Denise Reducing Eating Disorder Onset in a Very High Risk Sample With Significant Comorbid Depression: A Randomized Controlled Trial. JOURNAL OF CONSULTING AND CLINICAL PSYCHOLOGY. 2016;84(5):402-414.

2700. Robertson Kirsten, Thyne Maree, Green James Supporting a sugar tax in New Zealand: Sugar sweetened beverage ('fizzy drink') consumption as a normal behaviour within the obesogenic environment. PEERJ. 2018;6():.

2701. Carruba MO, Cuzzolaro M, Riva L, Bosello O, Liberti S, Castra R, Dalle Grave R, Santonastaso P, Garosi J, Nisoli E Efficacy and tolerability of moclobemide in bulimia nervosa: a placebo-controlled trial. INTERNATIONAL CLINICAL PSYCHOPHARMACOLOGY. 2001;16(1):27-32.

2702. Peat Christine, Peyerl Naomi, Muehlenkamp Jennifer Body Image and Eating Disorders in Older Adults: A Review. JOURNAL OF GENERAL PSYCHOLOGY. 2008;135(4, SI):343-358.

2703. Kostopoulou M., Varsou E., Stalikas A. Thought-shape fusion in bulimia PAPER nervosa: An experimental investigation. EATING AND WEIGHT DISORDERS-STUDIES ON ANOREXIA BULIMIA AND OBESITY. 2011;16(2):E86-E92.

2704. Wilksch Simon, O'Shea Anne, Ho Pheobe, Byrne Sue, Wade Tracey The relationship between social media use and disordered eating in young adolescents. INTERNATIONAL JOURNAL OF EATING DISORDERS. 2020;53(1):96-106.

2705. Neumark-Sztainer D, Croll J, Story M, Hannan PJ, French SA, Perry C Ethnic/racial differences in weight-related concerns and behaviors among adolescent girls and boys - Findings from Project EAT. JOURNAL OF PSYCHOSOMATIC RESEARCH. 2002;53(5):963-974.

2706. Dakanalis Antonios, Timko C., Colmegna Fabrizia, Riva Giuseppe, Clerici Massimo Evaluation of the DSM-5 severity ratings for anorexia nervosa in a clinical sample. PSYCHIATRY RESEARCH. 2018;262():124-128.

2707. McIntyre Roger, Alsuwaidan Mohammad, Goldstein Benjamin, Taylor Valerie, Schaffer Ayal, Beaulieu Serge, Kemp David The Canadian Network for Mood and Anxiety Treatments (CANWAT) task force recommendations for the management of patients with mood disorders and comorbid metabolic disorders. ANNALS OF CLINICAL PSYCHIATRY. 2012;24(1):69-81.

2708. Remschmidt H Evidence concerning the effectiveness of psychotherapies with children and adolescents. CURRENT OPINION IN PSYCHIATRY. 2003;16(4):389-393.

2709. Seward M., Block J., Chatterjee A. Student experiences with traffic-light labels at college cafeterias: a mixed methods study. OBESITY SCIENCE \& PRACTICE. 2018;4(2):159-177.

2710. Bouguettaya Ayoub, Cruwys Tegan, Moulding Richard, King Ross, Bliuc Ana-Maria Evidence That Frame of Reference Effects Can Reduce Socially Prescribed Perfectionism. FRONTIERS IN PSYCHOLOGY. 2019;9():.

2711. Hoyt Crystal, Burnette Jeni, Thomas Fanice, Orvidas Kasey Public Health Messages and Weight-Related Beliefs: Implications for Well-Being and Stigma. FRONTIERS IN PSYCHOLOGY. 2019;10():.

2712. Bryant Judith, Darkes Jack, Rahal Collin College Students' Compensatory Eating and Behaviors in Response to Alcohol Consumption. JOURNAL OF AMERICAN COLLEGE HEALTH. 2012;60(5):350-356.

2713. Vitousek KM, Gray JA, Grubbs KM Caloric restriction for longevity: I. Paradigm, protocols and physiological findings in animal research. EUROPEAN EATING DISORDERS REVIEW. 2004;12(5):279-299.

2714. Kluck Annette, Dallesasse Starla, English Erin Family Relations and Psychopathology: Examining Depressive and Bulimic Symptomatology. CHILD PSYCHIATRY \& HUMAN DEVELOPMENT. 2017;48(5):818-827.

2715. Masih Tasmiah, Dimmock James, Epel Elissa, Guelfi Kyrn Stress-induced eating and the relaxation response as a potential antidote: A review and hypothesis. APPETITE. 2017;118():136-143.

2716. SOUNDY TJ, LUCAS AR, SUMAN VJ, MELTON LJ BULIMIA-NERVOSA IN ROCHESTER, MINNESOTA FROM 1980 TO 1990. PSYCHOLOGICAL MEDICINE. 1995;25(5):1065-1071.

2717. Guillem E., Arbabzadeh-Bouchez S., Vorspan F., Bellivier F. Comorbidity in 207 cannabis users in a specific outpatient setting. ENCEPHALE-REVUE DE PSYCHIATRIE CLINIQUE BIOLOGIQUE ET THERAPEUTIQUE. 2015;41(1):S7-S12.

2718. Veatupu Loma, Puloka Viliami, Smith Moira, McKerchar Christina, Signal Louise Me'akai in Tonga: Exploring the Nature and Context of the Food Tongan Children Eat in Ha'apai Using Wearable Cameras. INTERNATIONAL JOURNAL OF ENVIRONMENTAL RESEARCH AND PUBLIC HEALTH. 2019;16(10):.

2719. Gates Taylor, Stough Cathleen Exploring risk factors of food and alcohol disturbance (FAD) in US college students. EATING AND WEIGHT DISORDERS-STUDIES ON ANOREXIA BULIMIA AND OBESITY. 2022;27(5):1739-1749.

2720. Ackard Diann, Fedio Gregory, Neumark-Sztainer Dianne, Britt Heather Factors associated with disordered eating among sexually active adolescent males: Gender and number of sexual partners. PSYCHOSOMATIC MEDICINE. 2008;70(2):232-238.

2721. Aardoom Jiska, Dingemans Alexandra, Spinhoven Philip, Hakkaart-van Roijen Leona, Van Furth Eric An Internet-based intervention for eating disorders consisting of automated computer-tailored feedback with or without supplemented frequent or infrequent support from a coach: study protocol for a randomized controlled trial. TRIALS. 2013;14():.

2722. Franca Vivian, Azzolini Thairine, Pissaia Ediane, Bortoloti Durcelina, Signorini Taise, Dalla Costa Lediana, Queiroz Souza Marilia, Reis Livero Francislaine, Wietzikoski Lovato Evellyn Diet, Epidemiological Factors and Cognitive Impairment: A Cross-Sectional Study in the Elderly Population. BRAZILIAN ARCHIVES OF BIOLOGY AND TECHNOLOGY. 2018;61():.

2723. Raffi AR, Rondini M, Grandi S, Fava GA Life events and prodromal symptoms in bulimia nervosa. PSYCHOLOGICAL MEDICINE. 2000;30(3):727-731.

2724. Demmler Joanne, Brophy Sinead, Marchant Amanda, John Ann, Tan Jacinta Shining the light on eating disorders, incidence, prognosis and profiling of patients in primary and secondary care: national data linkage study. BRITISH JOURNAL OF PSYCHIATRY. 2020;216(2):105-112.

2725. Wolter Vanessa, Hammerle Florian, Buerger Arne, Ernst Verena Prevention of eating disorders-Efficacy and cost-benefit of a school-based program ({''}MaiStep{''}) in a randomized controlled trial (RCT). INTERNATIONAL JOURNAL OF EATING DISORDERS. 2021;54(10):1855-1864.

2726. Villarejo Cynthia, Fernandez-Aranda Fernando, Jimenez-Murcia Susana, Penas-Lledo Eva, Granero Roser, Penelo Eva, Tinahones Francisco, Sancho Carolina, Vilarrasa Nuria, Bernabe Monica, Casanueva Felipe, Manuel Fernandez-Real Jose, Fruehbeck Gema, Torre Rafael, Treasure Janet, Botella Cristina, Manuel Menchon Jose Lifetime Obesity in Patients with Eating Disorders: Increasing Prevalence, Clinical and Personality Correlates. EUROPEAN EATING DISORDERS REVIEW. 2012;20(3):250-254.

2727. Goddard Georgia, Oxlad Melissa Insulin restriction or omission in Type 1 Diabetes Mellitus: a meta-synthesis of individuals' experiences of diabulimia. HEALTH PSYCHOLOGY REVIEW. 2023;17(2):227-246.

2728. Houeto Jean-Luc, Magnard Robin, Dalley Jeffrey, Belin David, Carnicella Sebastien Trait Impulsivity and Anhedonia: Two Gateways for the Development of Impulse Control Disorders in Parkinson's Disease?. FRONTIERS IN PSYCHIATRY. 2016;7():.

2729. Dalgleish T, Tchanturia K, Serpell L, Hems S, Silva P, Treasure J Perceived control over events in the world in patients with eating disorders: A preliminary study. PERSONALITY AND INDIVIDUAL DIFFERENCES. 2001;31(3):453-460.

2730. Ray Supriya, Khaire Priya, Joping Mukesh, Parshipog Alvina, Gole Prathmesh, George Mekha An exploratory study to assess the eating attitude adolescent children residing in selected areas of Pune City. INTERNATIONAL JOURNAL OF EARLY CHILDHOOD SPECIAL EDUCATION. 2022;14(04):912-916.

2731. Treasure Janet Coherence and other autistic spectrum traits and eating disorders: Building from mechanism to treatment. The Birgit Olsson lecture. NORDIC JOURNAL OF PSYCHIATRY. 2013;67(1):38-42.

2732. Phillips KA, Pagano ME, Menard W, Fay C, Stout RL Predictors of remission from body dysmorphic disorder: A prospective study. JOURNAL OF NERVOUS AND MENTAL DISEASE. 2005;193(8):564-567.

2733. Freeland-Graves Jeanne, Nitzke Susan Position of the Academy of Nutrition and Dietetics: Total Diet Approach to Healthy Eating. JOURNAL OF THE ACADEMY OF NUTRITION AND DIETETICS. 2013;113(2):307-317.

2734. Wade T, Neale MC, Lake RIE, Martin NG A genetic analysis of the eating and attitudes associated with bulimia nervosa: Dealing with the problem of ascertainment in twin studies. BEHAVIOR GENETICS. 1999;29(1):1-10.

2735. Button EJ, SonugaBarke EJS, Davies J, Thompson M A prospective study of self-esteem in the prediction of eating problems in adolescent schoolgirls: Questionnaire findings. BRITISH JOURNAL OF CLINICAL PSYCHOLOGY. 1996;35(2):193-203.

2736. Callesen M., Weintraub D., Damholdt M., Moller A. Impulsive and compulsive behaviors among Danish patients with Parkinson's disease: Prevalence, depression, and personality. PARKINSONISM \& RELATED DISORDERS. 2014;20(1):22-26.

2737. Milan Stephanie, Acker Jenna Early attachment quality moderates eating disorder risk among adolescent girls. PSYCHOLOGY \& HEALTH. 2014;29(8):896-914.

2738. Gulec Hayriye, Moessner Markus, Tury Ferenc, Fiedler Peter, Mezei Agnes, Bauer Stephanie A Randomized Controlled Trial of an Internet-Based Posttreatment Care for Patients with Eating Disorders. TELEMEDICINE AND E-HEALTH. 2014;20(10):916-922.

2739. Baechle Christina, Stahl-Pehe Anna, Rosenbauer Joachim Disordered eating and insulin restriction in youths receiving intensified insulin treatment: Results from a nationwide population-based study. INTERNATIONAL JOURNAL OF EATING DISORDERS. 2016;49(2):193-198.

2740. Hart Susan, Abraham Suzanne, Franklin Richard, Twigg Stephen, Russell Janice Hypoglycaemia following a mixed meal in eating disorder patients. POSTGRADUATE MEDICAL JOURNAL. 2011;87(1028):405-409.

2741. Vacca Mariacarolina, Ballesio Andrea, Lombardo Caterina The relationship between perfectionism and eating-related symptoms in adolescents: A systematic review. EUROPEAN EATING DISORDERS REVIEW. 2021;29(1):32-51.

2742. Ravi Suvi, Valtonen Maarit, Ihalainen Johanna, Holopainen Elina, Kosola Silja, Heinonen Saara, Waller Ben, Kujala Urho, Parkkari Jari Eating behaviours, menstrual history and the athletic career: a retrospective survey from adolescence to adulthood in female endurance athletes. BMJ OPEN SPORT \& EXERCISE MEDICINE. 2023;9(1):.

2743. Quiles Marcos Yolanda, Terol Cantero Maria Coping and eating disorders: A review. REVISTA LATINOAMERICANA DE PSICOLOGIA. 2008;40(2):259-280.

2744. SUZUKI K, HIGUCHI S, YAMADA K, MIZUTANI Y, KONO H YOUNG FEMALE ALCOHOLICS WITH AND WITHOUT EATING DISORDERS - A COMPARATIVE-STUDY IN JAPAN. AMERICAN JOURNAL OF PSYCHIATRY. 1993;150(7):1053-1058.

2745. Roca Miquel, Kohls Elisabeth, Gili Margalida, Watkins Ed, Owens Matthew, Hegerl Ulrich, Grootheest Gerard, Bot Mariska, Cabout Mieke, Brouwer Ingeborg, Visser Marjolein, Penninx Brenda, Trial MooDFOOD Prevention of depression through nutritional strategies in high-risk persons: rationale and design of the MooDFOOD prevention trial. BMC PSYCHIATRY. 2016;16():.

2746. Wiseman CV, Turco RM, Sunday SR, Halmi KA Smoking and body image concerns in adolescent girls. INTERNATIONAL JOURNAL OF EATING DISORDERS. 1998;24(4):429-433.

2747. Gonzalez B, Huerta-Sanchez E, Ortiz-Nieves A, Vazquez-Alvarez T, Kribs-Zaleta C AmI too fat? Bulimia as an epidemic. JOURNAL OF MATHEMATICAL PSYCHOLOGY. 2003;47(5-6):515-526.

2748. Manent Jose, Lomas Samuel, Marcos Loreto, Lopez Gonzalez Angel, Soler Maria, Tarraga Lopez Pedro Analysis of the efficacy of the main dietary patterns in reducing cardiovascular risk. MEDICINA BALEAR. 2023;38(1):153-170.

2749. Manzato E., Gualandi M., Roncarati E. Complete androgen insensitivity syndrome (CAIS) and eating disorders: a case report. EATING AND WEIGHT DISORDERS-STUDIES ON ANOREXIA BULIMIA AND OBESITY. 2021;26(7):2421-2426.

2750. Johansen Sara, Stenhaug Ben, Robakis Thalia, Williams Katherine, Cullen Mark Past Psychiatric Conditions as Risk Factors for Postpartum Depression: A Nationwide Cohort Study. JOURNAL OF CLINICAL PSYCHIATRY. 2020;81(1):.

2751. Honary Mahsa, Bell Beth, Clinch Sarah, Wild Sarah, McNaney Roisin Understanding the Role of Healthy Eating and Fitness Mobile Apps in the Formation of Maladaptive Eating and Exercise Behaviors in Young People. JMIR MHEALTH AND UHEALTH. 2019;7(6):.

2752. Cachelin Fary, Striegel-Moore Ruth, Regan Pamela Factors associated with treatment seeking in a community sample of European American and Mexican American women with eating disorders. EUROPEAN EATING DISORDERS REVIEW. 2006;14(6):422-429.

2753. ATKINS DM, SILBER TJ CLINICAL SPECTRUM OF ANOREXIA-NERVOSA IN CHILDREN. JOURNAL OF DEVELOPMENTAL AND BEHAVIORAL PEDIATRICS. 1993;14(4):211-216.

2754. Santin Julia, Mery Victoria, Elso Maria, Retamal Eva, Torres Catalina, Ivelic Jose, Godoy Jaime Sleep-related eating disorder: a descriptive study in Chilean patients. SLEEP MEDICINE. 2014;15(2):163-167.

2755. Neudeck P, Florin I, Tuschen-Caffier B Food exposure in patients with bulimia nervosa. PSYCHOTHERAPY AND PSYCHOSOMATICS. 2001;70(4):193-200.

2756. Moschis George, Mathur Anil, Shannon Randall Toward Achieving Sustainable Food Consumption: Insights from the Life Course Paradigm. SUSTAINABILITY. 2020;12(13):.

2757. Vieira Ana, Machado Barbara, Machado Paulo, Brandao Isabel, Roma-Torres Antonio, Goncalves Sonia Putative Risk Factors for Non-Suicidal Self-Injury in Eating Disorders. EUROPEAN EATING DISORDERS REVIEW. 2017;25(6):544-550.

2758. POLLOCK M, KOVACS M, CHARRONPROCHOWNIK D EATING DISORDERS AND MALADAPTIVE DIETARY INSULIN MANAGEMENT AMONG YOUTHS WITH CHILDHOOD-ONSET INSULIN-DEPENDENT DIABETES-MELLITUS. JOURNAL OF THE AMERICAN ACADEMY OF CHILD AND ADOLESCENT PSYCHIATRY. 1995;34(3):291-296.

2759. Monfort-Pires Milena, Salvador Emanuel, Folchetti Luciana, Siqueira-Catania Antonela, Barros Camila, Gouvea Ferreira Sandra Diet Quality Is Associated with Leisure-Time Physical Activity in Individuals at Cardiometabolic Risk. JOURNAL OF THE AMERICAN COLLEGE OF NUTRITION. 2014;33(4):297-305.

2760. Fernandez-Aranda Fernando, Pinheiro Andrea, Tozzi Federica, Thornton Laura, Fichter Manfred, Halmi Katherine, Kaplan Allan, Klump Kelly, Strober Michael, Woodside D., Crow Scott, Mitchell James, Rotondo Alessandro, Keel Pamela, Plotnicov Katherine, Berrettini Wade, Kaye Walter, Crawford Steven, Johnson Craig, Brandt Harry, La Via Maria, Bulik Cynthia Symptom profile of major depressive disorder in women with eating disorders. AUSTRALIAN AND NEW ZEALAND JOURNAL OF PSYCHIATRY. 2007;41(1):24-31.

2761. Etxandi Mikel, Baenas Isabel, Munguia Lucero, Mestre-Bach Gemma, Granero Roser, Gomez-Pena Monica, Moragas Laura, Pino-Gutierrez Amparo, Codina Ester, Mora-Maltas Bernat, Valenciano-Mendoza Eduardo, Potenza Marc, Gearhardt Ashley, Fernandez-Aranda Fernando, Jimenez-Murcia Susana Clinical Features of Gambling Disorder Patients with and Without Food Addiction: Gender-Related Considerations. JOURNAL OF GAMBLING STUDIES. 2022;38(3):843-862.

2762. Pickhardt Mara, Adametz Luise, Richter Felicitas, Strauss Bernhard, Berger Uwe German Prevention Programs for Eating Disorders A Systematic Review. PSYCHOTHERAPIE PSYCHOSOMATIK MEDIZINISCHE PSYCHOLOGIE. 2019;69(1):10-19.

2763. Argyrides Marios, Anastasiades Elly, Alexiou Evangelia Risk and Protective Factors of Disordered Eating in Adolescents Based on Gender and Body Mass Index. INTERNATIONAL JOURNAL OF ENVIRONMENTAL RESEARCH AND PUBLIC HEALTH. 2020;17(24):.

2764. Wilksch S., Paxton S., Byrne S., Austin S., McLean S., Thompson K., Dorairaj K., Wade T. Prevention Across the Spectrum: a randomized controlled trial of three programs to reduce risk factors for both eating disorders and obesity. PSYCHOLOGICAL MEDICINE.

2015;45(9):1811-1823.

2765. Hsieh Kuan-Ying, Hsiao Ray, Yang Yi-Hsin, Liu Tai-Ling, Yen Cheng-Fang Predictive Effects of Sex, Age, Depression, and Problematic Behaviors on the Incidence and Remission of Internet Addiction in College Students: A Prospective Study. INTERNATIONAL JOURNAL OF ENVIRONMENTAL RESEARCH AND PUBLIC HEALTH. 2018;15(12):.

2766. Tylka Tracy, Russell Hannah, Neal Ashley Self-compassion as a moderator of thinness-related pressures' associations with thin-ideal internalization and disordered eating. EATING BEHAVIORS. 2015;17():23-26.

2767. Smith April, Hawkeswood Sean, Joiner Thomas The Measure of a Man: Associations between Digit Ratio and Disordered Eating in Males. INTERNATIONAL JOURNAL OF EATING DISORDERS. 2010;43(6):543-548.

2768. Ferreira Tiago, Koszegi Natalia Obsessive-Compulsive Disorder According to the Inference-Based Approach. PSILOGOS. 2019;17(1-2):92-101.

2769. Carter JC, Blackmore E, Sutandar-Pinnock K, Woodside DB Relapse in anorexia nervosa: a survival analysis. PSYCHOLOGICAL MEDICINE. 2004;34(4):671-679.

2770. Reas DL, Williamson DA, Martin CK, Zucker NL Duration of illness predicts outcome for bulimia nervosa: A long-term follow-up study. INTERNATIONAL JOURNAL OF EATING DISORDERS. 2000;27(4):428-434.

2771. Lewis Stephen, Arbuthnott Alexis Searching for Thinspiration: The Nature of Internet Searches for Pro-Eating Disorder Websites. CYBERPSYCHOLOGY BEHAVIOR AND SOCIAL NETWORKING. 2012;15(4):200-204.

2772. Raney T., Thornton Laura, Berrettini Wade, Brandt Harry, Crawford Steven, Fichter Manfred, Halmi Katherine, Johnson Craig, Kaplan Allan, Lavia Maria, Mitchell James, Rotondo Alessandro, Strober Michael, Woodside D., Kaye Walter, Bulik Cynthia Influence of overanxious disorder of childhood on the expression of anorexia nervosa. INTERNATIONAL JOURNAL OF EATING DISORDERS. 2008;41(4):326-332.

2773. Vocks Silja, Stahn Catharina, Loenser Kerstin, Legenbauer Tanja Eating and Body Image Disturbances in Male-to-Female and Female-to-Male Transsexuals. ARCHIVES OF

SEXUAL BEHAVIOR. 2009;38(3):364-377.

2774. Angst Jules, Roessler Wulf, Ajdacic-Gross Vladeta, Angst Felix, Wittchen Hans, Lieb Rosalind, Beesdo-Baum Katja, Asselmann Eva, Merikangas Kathleen, Cui Lihong, Andrade Laura, Viana Maria, Lamers Femke, Penninx Brenda, Cardoso Taiane, Jansen Karen, Mattos Souza Luciano, Silva Ricardo, Kapczinski Flavio, Grobler Christoffel, Gholam-Rezaee Mehdi, Preisig Martin, Vandeleur Caroline Differences between unipolar mania and bipolar-I disorder: Evidence from nine epidemiological studies. BIPOLAR DISORDERS. 2019;21(5):437-448.

2775. Torresan Ricardo, Abreu Ramos-Cerqueira Ana, Mathis Maria, Diniz Juliana, Ferrao Ygor, Miguel Euripedes, Torres Albina Sex differences in the phenotypic expression of obsessive-compulsive disorder: an exploratory study from Brazil. COMPREHENSIVE PSYCHIATRY. 2009;50(1):63-69.

2776. Wiss David, Brewerton Timothy, Tomiyama A. Limitations of the protective measure theory in explaining the role of childhood sexual abuse in eating disorders, addictions, and obesity: an updated model with emphasis on biological embedding. EATING AND WEIGHT DISORDERS-STUDIES ON ANOREXIA BULIMIA AND OBESITY. 2022;27(4):1249-1267.

2777. Bodell Lindsay, Racine Sarah, Wildes Jennifer Examining Weight Suppression as a Predictor of Eating Disorder Symptom Trajectories in Anorexia Nervosa. INTERNATIONAL JOURNAL OF EATING DISORDERS. 2016;49(8):753-763.

2778. Stice Eric, Rohde Paul, Gau Jeff, Shaw Heather Effect of a Dissonance-Based Prevention Program on Risk for Eating Disorder Onset in the Context of Eating Disorder Risk Factors. PREVENTION SCIENCE. 2012;13(2):129-139.

2779. Kaslow Nadine, Broth Michelle, Smith Chaundrissa, Collins Marietta Family-Based Interventions for Child and Adolescent Disorders. JOURNAL OF MARITAL AND FAMILY THERAPY. 2012;38(1):82-100.

2780. Latzer Yael, Weinberger-Litman Sarah, Gerson Barbara, Rosch Anna, Mischel Rebecca, Hinden Talia, Kilstein Jeffrey, Silver Judith Negative Religious Coping Predicts Disordered Eating Pathology Among Orthodox Jewish Adolescent Girls. JOURNAL OF RELIGION \& HEALTH. 2015;54(5):1760-1771.

2781. Zhu Hong, Luo Xingwei, Cai Taisheng, He Jinbo, Lu Yao, Wu Siyao Life Event Stress and Binge Eating Among Adolescents: The Roles of Early Maladaptive Schemas and Impulsivity. STRESS AND HEALTH. 2016;32(4):395-401.

2782. D'Ambrosio Virginia, Albert Umberto, Bogetto Filippo, Maina Giuseppe Obsessive-compulsive disorder and cyclothymic temperament: An exploration of clinical features. JOURNAL OF AFFECTIVE DISORDERS. 2010;127(1-3):295-299.

2783. Chao Ariana, Wadden Thomas, Gorin Amy, Tronieri Jena, Pearl Rebecca, Bakizada Zayna, Yanovski Susan, Berkowitz Robert Binge Eating and Weight Loss Outcomes in Individuals with Type 2 Diabetes: 4-Year Results from the Look AHEAD Study. OBESITY. 2017;25(11):1830-1837.

2784. Vanderkruik Rachel, Gist Darcy, Dimidjian Sona Preventing Eating Disorders in Young Women: An RCT and Mixed-Methods Evaluation of the Peer-Delivered Body Project. JOURNAL OF CONSULTING AND CLINICAL PSYCHOLOGY. 2020;88(12):1105-1118.

2785. Chiavarino Francesca, Pruccoli Jacopo, Cecconi Ilaria, Vancini Nicolo, Cordelli Duccio, Parmeggiani Antonia Eating disorders in young patients with neurofibromatosis type 1. JOURNAL OF PAEDIATRICS AND CHILD HEALTH. 2023;59(5):723-728.

2786. Pesa J Psychosocial factors associated with dieting behaviors among female adolescents. JOURNAL OF SCHOOL HEALTH. 1999;69(5):196-201.

2787. Cavicchioli Marco, Ramella Pietro, Vassena Giulia, Simone Giulia, Prudenziati Francesca, Sirtori Federica, Movalli Mariagrazia, Maffei Cesare Mindful self-regulation of attention is a key protective factor for emotional dysregulation and addictive behaviors among individuals with alcohol use disorder. ADDICTIVE BEHAVIORS. 2020;105():.

2788. Pierson DM Case study of fulminant meningococcal septicemia diagnosed in a twenty-year-old woman with bulimia nervosa. HEART \& LUNG. 1997;26(6):492-500.

2789. Ousley Louise, Cordero Elizabeth, White Sabina Eating disorders and body image of undergraduate men. JOURNAL OF AMERICAN COLLEGE HEALTH. 2008;56(6):617-621.

2790. Masuda Akihiko, Hill Mary, Tully Erin, Garcia Sarah The role of disordered eating cognition and body image flexibility in disordered eating behavior in college men. JOURNAL

OF CONTEXTUAL BEHAVIORAL SCIENCE. 2015;4(1):12-20.

2791. Avila Jonathan, Park K., Golden Neville Eating disorders in adolescents with chronic gastrointestinal and endocrine diseases. LANCET CHILD \& ADOLESCENT HEALTH. 2019;3(3):181-189.

2792. Frare F, Perugi G, Ruffalo G, Toni C Obsessive-compulsive disorder and body dysmorphic disorder: a comparison of clinical features. EUROPEAN PSYCHIATRY. 2004;19(5):292-298.

2793. Striegel-Moore Ruth, Bulik Cynthia Risk factors for eating disorders. AMERICAN PSYCHOLOGIST. 2007;62(3):181-198.

2794. Hoskin Rhea, Holmberg Diane, Jenson Kay, Blair Karen Holy anorexia: Views of femininity as a potential mediator in the association between religiosity and disordered eating. WOMENS STUDIES INTERNATIONAL FORUM. 2020;79():.

2795. Celio AA, Winzelberg AJ, Wilfley DE, Eppstein-Herald D, Springer EA, Dev P, Taylor CB Reducing risk factors for eating disorders: Comparison of an Internet- and a classroom-delivered psychoeducational program. JOURNAL OF CONSULTING AND CLINICAL PSYCHOLOGY. 2000;68(4):650-657.

2796. Paulson Lauren, Rutledge Patricia Effects of perfectionism and exercise on disordered eating in college students. EATING BEHAVIORS. 2014;15(1):116-119.

2797. Mason Tyler, Smith Kathryn Delineating the role of binge eating in cancer research. EATING AND WEIGHT DISORDERS-STUDIES ON ANOREXIA BULIMIA AND OBESITY. 2021;26(7):2109-2116.

2798. Bravender T., Bryant-Waugh R., Herzog D., Katzman D., Kriepe R., Lask B., Le Grange D., Lock J., Loeb K., Marcus M., Madden S., Nicholls D., O'Toole J., Pinhas L., Rome E., Sokol-Burger M., Wallin U., Zucker N., WCEDCA Classification of Eating Disturbance in Children and Adolescents: Proposed Changes for the DSM-V. EUROPEAN EATING DISORDERS REVIEW. 2010;18(2):79-89.

2799. Choate Laura Negotiating Contradictory Cultural Pressures: A Treatment Model for Binge Eating in Adolescent Girls. WOMEN \& THERAPY. 2011;34(4):377-392.

2800. Rowe Sarah, Jordan Jenny, McIntosh Virginia, Carter Frances, Frampton Chris, Bulik Cynthia, Joyce Peter Complex personality disorder in bulimia nervosa. COMPREHENSIVE PSYCHIATRY. 2010;51(6):592-598.

2801. Brown Corbett, Shaibu Sheila, Maruapula Segametsi, Malete Leapetswe, Compher Charlene Perceptions and attitudes towards food choice in adolescents in Gaborone, Botswana. APPETITE. 2015;95():29-35.

2802. Watson Hunna, O'Brien Amy, Sadeh-Sharvit Shiri Children of Parents with Eating Disorders. CURRENT PSYCHIATRY REPORTS. 2018;20(11):.

2803. RIEG TS, MAESTRELLO AM, ARAVICH PF WEIGHT CYCLING ALTERS THE EFFECTS OF D-FENFLURAMINE ON SUSCEPTIBILITY TO ACTIVITY-BASED ANOREXIA. AMERICAN JOURNAL OF CLINICAL NUTRITION. 1994;60(4):494-500.

2804. Gonzalez-Juarez Carlos, Perez-Perez Esther, Martin Cabrera Beatriz, Mitja Pau Isabel, Pablo Rosa, Torre Escalera Paloma Detection of adolescents at risk of suffering eating disorders. ATENCION PRIMARIA. 2007;39(4):189-194.

2805. Hasler G, LaSalle-Ricci VH, Ronquillo JG, Crawley SA, Cochran LW, Kazuba D, Greenberg BD, Murphy DL Obsessive-compulsive disorder symptom dimensions show specific relationships to psychiatric comorbidity. PSYCHIATRY RESEARCH. 2005;135(2):121-132.

2806. Lichtenstein Mia, Hinze Cecilie, Emborg Bolette, Thomsen Freja, Hemmingsen Simone Compulsive exercise: links, risks and challenges faced. PSYCHOLOGY RESEARCH AND BEHAVIOR MANAGEMENT. 2017;10():85-95.

2807. Tamburrino MB, McGinnis RA Anorexia nervosa - A review. PANMINERVA MEDICA. 2002;44(4):301-311.

2808. Whittal ML Bulimia nervosa: A meta-analysis of psychosocial and pharmacological treatments. BEHAVIOR THERAPY. 1999;30(1):117-135.

2809. Penas-Lledo Eva, Bulik Cynthia, Lichtenstein Paul, Larsson Henrik, Baker Jessica Risk for self-reported anorexia or bulimia nervosa based on drive for thinness and negative affect clusters/dimensions during adolescence: A three-year prospective study of the TChAD cohort. INTERNATIONAL JOURNAL OF EATING DISORDERS. 2015;48(6):692-699.

2810. Cohrdes Caroline, Goebel Kristin, Schlack Robert, Hoelling Heike Symptoms of eating disorders in children and adolescents: frequencies and risk factors Results from KiGGS Wave 2 and trends. BUNDESGESUNDHEITSBLATT-GESUNDHEITSFORSCHUNG-GESUNDHEITSSCHUTZ. 2019;62(10, SI):1195-1204.

2811. Stice Eric, Marti C., Durant Shelley Risk factors for onset of eating disorders: Evidence of multiple risk pathways from an 8-year prospective study. BEHAVIOUR RESEARCH AND THERAPY. 2011;49(10):622-627.

2812. Yin Weiyao, Persson Martina, Sandin Sven Parental history of psychiatric disorders and risk of type 1 diabetes in the offspring. DIABETES \& METABOLISM. 2023;49(1):.

2813. Uriegas Nancy, Emerson Dawn, Smith Allison, Kelly Melani, Torres-McGehee Toni Examination of eating disorder risk among university marching band artists. JOURNAL OF EATING DISORDERS. 2021;9(1):.

2814. Stoving Rene, Andries Alin, Brixen Kim, Flyvbjerg Allan, Horder Kirsten, Frystyk Jan Leptin, ghrelin, and endocannabinoids: Potential therapeutic targets in anorexia nervosa. JOURNAL OF PSYCHIATRIC RESEARCH. 2009;43(7):671-679.

2815. Rohana Abdul, Aiba Naomi Childhood Obesity in Japan: A Growing Public Health Threat. INTERNATIONAL MEDICAL JOURNAL. 2012;19(2):146-149.

2816. Cimino Silvia, Cerniglia Luca, Porreca Alessio, Simonelli Alessandra, Ronconi Lucia, Ballarotto Giulia Mothers and Fathers with Binge Eating Disorder and Their 18-36 Months Old Children: A Longitudinal Study on Parent-Infant Interactions and Offspring's Emotional-Behavioral Profiles. FRONTIERS IN PSYCHOLOGY. 2016;7():.

2817. Katzenschlager R., Goerlich K., Eimeren T. Repetitive impulse-associated behavioral disorders in Parkinson's disease. NERVENARZT. 2012;83(12):1582+.

2818. Gehman Sarah, Ackerman Kathryn, Caksa Signe, Rudolph Sara, Hughes Julie, Garrahan Margaret, Tenforde Adam, Bouxsein Mary, Popp Kristin Restrictive Eating and Prior Low-Energy Fractures Are Associated With History of Multiple Bone Stress Injuries. INTERNATIONAL JOURNAL OF SPORT NUTRITION AND EXERCISE METABOLISM. 2022;32(5):325-333.

2819. Langbein Rachel, Martin Daniel, Allen-Collinson Jacquelyn, Crust Lee, Jackman Patricia ``I'd got self-destruction down to a fine art{''}: a qualitative exploration of relative energy deficiency in sport (RED-S) in endurance athletes. JOURNAL OF SPORTS SCIENCES. 2021;39(14):1555-1564.

2820. Menzel Jessie, Thompson J., Levine Michael Development and validation of the Physical Activity Body Experiences Questionnaire. BULLETIN OF THE MENNINGER CLINIC. 2019;83(1):53-83.

2821. Jose Menor-Rodriguez Maria, Cortes-Martin Jonathan, Rodriguez-Blanque Raquel, Isabel Tovar-Galvez Maria, Jose Aguilar-Cordero Maria, Carlos Sanchez-Garcia Juan Influence of an Educational Intervention on Eating Habits in School-Aged Children. CHILDREN-BASEL. 2022;9(4):.

2822. Srivastava Paakhi, Presseller Emily, Chen Joanna, Clark Kelsey, Hunt Rowan, Clancy Olivia, Manasse Stephanie, Juarascio Adrienne Weight status is associated with clinical characteristics among individuals with bulimia nervosa. EATING DISORDERS. 2022;():.

2823. Grabhorn R, Kopp W, Gitzinger I, Wietersheim J, Kaufhold J Differences between female and male patients with eating disorders results of the multicenter study on eating disorders (MZ-Ess). PSYCHOTHERAPIE PSYCHOSOMATIK MEDIZINISCHE PSYCHOLOGIE. 2003;53(1):15-22.

2824. Troop NA Eating disorders as coping strategies: A critique. EUROPEAN EATING DISORDERS REVIEW. 1998;6(4):229-237.

2825. Pearson Carolyn, Zapolski Tamika, Smith Gregory A Longitudinal Test of Impulsivity and Depression Pathways to Early Binge Eating Onset. INTERNATIONAL JOURNAL OF EATING DISORDERS. 2015;48(2):230-237.

2826. Baceviciene Migle, Jankauskiene Rasa, Balciuniene Vaiva The Role of Body Image, Disordered Eating and Lifestyle on the Quality of Life in Lithuanian University Students.

INTERNATIONAL JOURNAL OF ENVIRONMENTAL RESEARCH AND PUBLIC HEALTH. 2020;17(5):.

2827. Witt Ashley, Berkowitz Staci, Gillberg Christopher, Lowe Michael, Rastam Maria, Wentz Elisabet Weight Suppression and Body Mass Index Interact to Predict Long-Term Weight Outcomes in Adolescent-Onset Anorexia Nervosa. JOURNAL OF CONSULTING AND CLINICAL PSYCHOLOGY. 2014;82(6):1207-1211.

2828. Boswell James, Anderson Lisa, Anderson Drew Integration of Interoceptive Exposure in Eating Disorder Treatment. CLINICAL PSYCHOLOGY-SCIENCE AND PRACTICE. 2015;22(2):194-210.

2829. Ackard Diann, Eisenberg Marla, Neumark-Sztainer Dianne Long-term impact of adolescent dating violence on the behavioral and tsychological health of male and female youth. JOURNAL OF PEDIATRICS. 2007;151(5):476-481.

2830. Cao Wenjun, Hou Guodiang, Zhang Xin, San Hongxia, Zheng Jianzhong Potential risk factors related to the development of gastric polyps. IMMUNOPHARMACOLOGY AND IMMUNOTOXICOLOGY. 2018;40(4):338-343.

2831. Constant Aymery, Moirand Romain, Thibault Ronan, Val-Laillet David Meeting of Minds around Food Addiction: Insights from Addiction Medicine, Nutrition, Psychology, and Neurosciences. NUTRIENTS. 2020;12(11):.

2832. Cernelic-Bizjak Masa, Guine Raquel Predictors of binge eating: relevance of BMI, emotional eating and sensivity to environmental food cues. NUTRITION \& FOOD SCIENCE. 2022;52(1):171-180.

2833. Olajide Damilola, Eberth Barbara, Ludbrook Anne Analysis of Multiple Health Risky Behaviours and Associated Disease Outcomes Using Scottish Linked Hospitalisation Data. FRONTIERS IN PUBLIC HEALTH. 2022;10():.

2834. Wang Tao, Brede Markus, Ianni Antonella, Mentzakis Emmanouil Social interactions in online eating disorder communities: A network perspective. PLOS ONE. 2018;13(7):.

2835. Werneck Andre, Silva Danilo, Malta Deborah, Souza-Junior Paulo, Azevedo Luiz, Azevedo Barros Marilisa, Szwarcwald Celia Lifestyle behaviors changes during the COVID-

19 pandemic quarantine among 6,881 Brazilian adults with depression and 35,143 without depression. CIENCIA \& SAUDE COLETIVA. 2020;25(2):4151-4156.

2836. Grange Daniel, Loeb Katharine Early identification and treatment of eating disorders: prodrome to syndrome. EARLY INTERVENTION IN PSYCHIATRY. 2007;1(1):27-39.

2837. Senior R, Barnes J, Emberson JR, Golding J, Team ALSPAC Early experiences and their relationship to maternal eating disorder symptoms, both lifetime and during pregnancy. BRITISH JOURNAL OF PSYCHIATRY. 2005;187():268-273.

2838. Taylor Sharonda, Ditch Sarah, Hansen Shana Identifying and Preventing Eating Disorders in Adolescent Patients with Obesity. PEDIATRIC ANNALS. 2018;47(6):E232-E237.

2839. Diedrichs Phillippa, Atkinson Melissa, Steer Rebecca, Garbett Kirsty, Rumsey Nichola, Halliwell Emma Effectiveness of a brief school-based body image intervention `Dove Confident Me: Single Session' when delivered by teachers and researchers: Results from a cluster randomised controlled trial. BEHAVIOUR RESEARCH AND THERAPY. 2015;74():94-104.

2840. Amianto Federico, Abbate-Daga Giovanni, Morando Sara, Sobrero Cinzia, Fassino Secondo Personality development characteristics of women with anorexia nervosa, their healthy siblings and healthy controls: What prevents and what relates to psychopathology?. PSYCHIATRY RESEARCH. 2011;187(3):401-408.

2841. Leins Judith, Waldorf Manuel, Suchan Boris, Diers Martin, Herpertz Stephan, Paslakis Georgios, Steins-Loeber Sabine Exposure to the thin beauty ideal: Are there subliminal priming effects?. INTERNATIONAL JOURNAL OF EATING DISORDERS. 2021;54(4):506-515.

2842. Mathisen Therese, Sundgot-Borgen Christine, Anstensrud Beate, Sundgot-Borgen Jorunn Intervention in professional dance students to increase mental health- and nutrition literacy: A controlled trial with follow up. FRONTIERS IN SPORTS AND ACTIVE LIVING. 2022;4():.

2843. Larranaga Alejandra, Docet Maria, Garcia-Mayor Ricardo High prevalence of eating disorders not otherwise specified in northwestern Spain: population-based study. SOCIAL PSYCHIATRY AND PSYCHIATRIC EPIDEMIOLOGY. 2012;47(10):1669-1673.

2844. Kakamu Takeyasu, Hidaka Tomoo, Kumagai Tomohiro, Masuishi Yusuke, Kasuga Hideaki, Endo Shota, Sato Sei, Takeda Akiko, Koizumi Makoto, Fukushima Tetsuhito Unhealthy changes in eating habits cause acute onset hypertension in the normotensive community-dwelling elderly-3 years cohort study. MEDICINE. 2019;98(15):.

2845. Myszkowska-Ryciak Joanna, Harton Anna, Lange Ewa, Laskowski Waclaw, Gajewska Danuta Nutritional Behaviors of Polish Adolescents: Results of the Wise Nutrition-Healthy Generation Project. NUTRIENTS. 2019;11(7):.

2846. Billing-Bullen Gypsy, Nielsen Deirdre, Wham Carol, Kruger Rozanne Enablers and barriers to prevent weight-regain post bariatric surgery - A qualitative enquiry. EATING BEHAVIORS. 2022;47():.

2847. Palermo Madeline, Staples Cody, Rancourt Diana Examining the impact of weight bias on the association between exercise identity and maladaptive exercise behaviors. EATING BEHAVIORS. 2021;41():.

2848. Penalvo Jose, Fernandez-Friera Leticia, Lopez-Melgar Beatriz, Uzhova Irina, Oliva Belen, Miguel Fernandez-Alvira Juan, Laclaustra Martin, Pocock Stuart, Mocoroa Agustin, Mendiguren Jose, Sanz Gines, Guallar Eliseo, Bansilal Sameer, Vedanthan Rajesh, Jesus Jimenez-Borreguero Luis, Ibanez Borja, Ordovas Jose, Fernandez-Ortiz Antonio, Bueno Hector, Fuster Valentin Association Between a Social-Business Eating Pattern and Early Asymptomatic Atherosclerosis. JOURNAL OF THE AMERICAN COLLEGE OF CARDIOLOGY. 2016;68(8):805-814.

2849. Pollatos Olga, Georgiou Eleana, Kobel Susanne, Schreiber Anja, Dreyhaupt Jens, Steinacker Juergen Trait-Based Emotional Intelligence, Body Image Dissatisfaction, and HRQoL in Children. FRONTIERS IN PSYCHIATRY. 2020;10():.

2850. Eisenberg Marla, Berge Jerica, Fulkerson Jayne, Neumark-Sztainer Dianne Associations between hurtful weight-related comments by family and significant other and the development of disordered eating behaviors in young adults. JOURNAL OF BEHAVIORAL MEDICINE. 2012;35(5):500-508.

2851. Santiago Sarah, Park Grace, Huffman Kelly Consumption habits of pregnant women and implications for developmental biology: a survey of predominantly Hispanic women in California. NUTRITION JOURNAL. 2013;12():.

2852. Khedr E., El Fetoh N., El Bieh E., Ali A., Karim A. Altered cortical excitability in anorexia nervosa. NEUROPHYSIOLOGIE CLINIQUE-CLINICAL NEUROPHYSIOLOGY. 2014;44(3):291-299.

2853. Lei Gao, Shan Cui, Yu Han, Wei Dai, Yuan Su, Xin Zhang Does Periconceptional Fish Consumption by Parents Affect the Incidence of Autism Spectrum Disorder and Intelligence Deficiency? A Case-control Study in Tianjin, China. BIOMEDICAL AND ENVIRONMENTAL SCIENCES. 2016;29(12):885-892.

2854. Gupta Himanshu, Fatima Mirat-ul, Pandey Rukmani, Ram Kristipati Adult exposure of atrazine alone or in combination with carbohydrate diet hastens the onset/progression of type 2 diabetes in Drosophila. LIFE SCIENCES. 2023;316():.

2855. Kelly Allison, Vimalakanthan Kiruthiha, Carter Jacqueline Understanding the roles of self-esteem, self-compassion, and fear of self-compassion in eating disorder pathology: An examination of female students and eating disorder patients. EATING BEHAVIORS. 2014;15(3):388-391.

2856. Carlat DJ, Camargo CA, Herzog DB Eating disorders in males: A report on 135 patients. AMERICAN JOURNAL OF PSYCHIATRY. 1997;154(8):1127-1132.

2857. Choo Soo, Lee Hyun, Kim Chan, Yang Eun Severe hypernatremia in soft drink ketoacidosis and hyperglycemic hyperosmolar state at the onset of type 2 diabetes mellitus: a case series of three adolescents. CLINICAL PEDIATRIC ENDOCRINOLOGY. 2022;31(2):81-86.

2858. Prinz Philip, Hofmann Tobias, Ahnis Anne, Elbelt Ulf, Goebel-Stengel Miriam, Klapp Burghard, Rose Matthias, Stengel Andreas Plasma bile acids show a positive correlation with body mass index and are negatively associated with cognitive restraint of eating in obese patients. FRONTIERS IN NEUROSCIENCE. 2015;9():.

2859. Garcia-Burgos David, Wilhelm Peter, Voegele Claus, Munsch Simone Food Restriction in Anorexia Nervosa in the Light of Modern Learning Theory: A Narrative Review. BEHAVIORAL SCIENCES. 2023;13(2):.

2860. Talamayan Kathleen, Springer Andrew, Kelder Steven, Gorospe Emmanuel, Joye Karen Prevalence of overweight misperception and weight control behaviors among normal weight adolescents in the United States. THESCIENTIFICWORLDJOURNAL. 2006;6():365-

373.

2861. Godoy-Izquierdo Debora, Diaz Isabel Inhabiting the Body(ies) in Female Soccer Players: The Protective Role of Positive Body Image. FRONTIERS IN PSYCHOLOGY. 2021;12():.

2862. Blachno Magda, Brynska Anita, Tomaszewicz-Libudzic Celina, Jagielska Gabriela, Srebnicki Tomasz, Wolanczyk Tomasz The influence of obsessive compulsive symptoms on the course of anorexia nervosa. PSYCHIATRIA POLSKA. 2014;48(3):429-439.

2863. Kelly Megan, Dalrymple Kristy, Zimmerman Mark, Phillips Katharine A comparison study of body dysmorphic disorder versus social phobia. PSYCHIATRY RESEARCH. 2013;205(1-2):109-116.

2864. Sieben Angelien, Onzenoort Hein, Laarhoven Kees, Bredie Sebastian, Dulmen Sandra Identification of Cardiovascular Patient Groups at Risk for Poor Medication Adherence A Cluster Analysis. JOURNAL OF CARDIOVASCULAR NURSING. 2021;36(5):489-497.

2865. Vander Wal Jillon Unhealthy weight control behaviors among adolescents. JOURNAL OF HEALTH PSYCHOLOGY. 2012;17(1):110-120.

2866. THOMSEN PH OBSESSIVE-COMPULSIVE DISORDER IN CHILDREN AND ADOLESCENTS - A REVIEW OF THE LITERATURE. EUROPEAN CHILD \& ADOLESCENT PSYCHIATRY. 1994;3(3):138-158.

2867. Pinheiro Andrea, Raney T., Thornton Laura, Fichter Manfred, Berrettini Wade, Goldman David, Halmi Katherine, Kaplan Allan, Strober Michael, Treasure Janet, Woodside D., Kaye Walter, Bulik Cynthia Sexual Functioning in Women with Eating Disorders. INTERNATIONAL JOURNAL OF EATING DISORDERS. 2010;43(2):123-129.

2868. Smorthit Kelly, Sawbridge David, Fitzgerald Rhian Eating disorders and the orthodontist: Diagnosis, considerations and referral. JOURNAL OF ORTHODONTICS. 2021;48(3):313-322.

2869. Bardone-Cone Anna, Fitzsimmons-Craft Ellen, Harney Megan, Maldonado Christine, Lawson Melissa, Smith Roma, Robinson Paul The Inter-Relationships between Vegetarianism and Eating Disorders among Females. JOURNAL OF THE ACADEMY OF

NUTRITION AND DIETETICS. 2012;112(8):1247-1252.

2870. Yamaguchi N, Kobayashi J, Tachikawa H, Sato S, Hori M, Suzuki T, Shiraishi H Parental representation in eating disorder patients with suicide. JOURNAL OF PSYCHOSOMATIC RESEARCH. 2000;49(2):131-136.

2871. Bernacchi Dana Bulimia Nervosa: A Comprehensive Analysis of Treatment, Policy, and Social Work Ethics. SOCIAL WORK. 2017;62(2):174-180.

2872. Coutinho Walmir The first decade of sibutramine and orlistat: a reappraisal of their expanding roles in the treatment of obesity and associated conditions. ARQUIVOS BRASILEIROS DE ENDOCRINOLOGIA E METABOLOGIA. 2009;53(2):262-270.

2873. Schmidt U., Andiappan M., Grover M., Robinson S., Perkins S., Dugmore O., Landau S., Treasure J., Eisler I., Williams C. Randomised controlled trial of CD-ROM-based cognitive-behavioural self-care for bulimia nervosa. BRITISH JOURNAL OF PSYCHIATRY. 2008;193(6):493-500.

2874. Williams Fionnuala, Gibbs Susie, Addo Ama The assessment and management of pica in people with intellectual disability. BJPSYCH ADVANCES. 2022;28(6, SI):383-392.

2875. Norman Asa, Wright Julie, Patterson Emma Brief parental self-efficacy scales for promoting healthy eating and physical activity in children: a validation study. BMC PUBLIC HEALTH. 2021;21(1):.

2876. Fairburn CG, Cooper Z, Doll HA, Davies BA Identifying dieters who will develop an eating disorder: A prospective, population-based study. AMERICAN JOURNAL OF PSYCHIATRY. 2005;162(12):2249-2255.

2877. Ruuska Jaana, Koivisto Anna-Maija, Rantanen Paivi, Kaltiala-Heino Riittakerttu Psychosocial functioning needs attention in adolescent eating disorders. NORDIC JOURNAL OF PSYCHIATRY. 2007;61(6):452-458.

2878. MAIESE K, BOCCONE L NEUROPROTECTION BY PEPTIDE GROWTH-FACTORS AGAINST ANOXIA AND NITRIC-OXIDE TOXICITY REQUIRES MODULATION OF PROTEIN-KINASE-C. JOURNAL OF CEREBRAL BLOOD FLOW AND METABOLISM. 1995;15(3):440-449.

2879. Tierney S., Fox J., Butterfield C., Stringer E., Furber C. Treading the tightrope between motherhood and an eating disorder: A qualitative study. INTERNATIONAL JOURNAL OF NURSING STUDIES. 2011;48(10):1223-1233.

2880. Inguglia Cristiano, Costa Sebastiano, Iannello Nicolo, Liga Francesca Parental Monitoring and Youth's Binge Behaviors: The Role of Sensation Seeking and Life Satisfaction. CHILD CARE IN PRACTICE. 2021;27(2):120-138.

2881. Stice Eric, South Kelsey, Shaw Heather Future Directions in Etiologic, Prevention, and Treatment Research for Eating Disorders. JOURNAL OF CLINICAL CHILD AND ADOLESCENT PSYCHOLOGY. 2012;41(6):845-855.

2882. Fichter Manfred, Naab Silke, Voderholzer Ulrich, Quadflieg Norbert Mortality in males as compared to females treated for an eating disorder: a large prospective controlled study. EATING AND WEIGHT DISORDERS-STUDIES ON ANOREXIA BULIMIA AND OBESITY. 2021;26(5):1627-1637.

2883. Schumacher Leah, Arigo Danielle, Martin Lindsay UPWARD APPEARANCE COMPARISONS AND THE ONSET OF DISORDERED EATING SYMPTOMS DURING COLLEGE. ANNALS OF BEHAVIORAL MEDICINE. 2014;47(1):S21.

2884. Kostopoulou Myrsini, Varsou Eleftheria, Stalikas Anastassios Thought-shape fusion in anorexia and bulimia nervosa: a comparative experimental study. EATING AND WEIGHT DISORDERS-STUDIES ON ANOREXIA BULIMIA AND OBESITY. 2013;18(3):245-253.

2885. Mitchell JE, Fletcher L, Hanson K, Mussell MP, Seim H, Crosby R, Al-Banna M The relative efficacy of fluoxetine and manual-based self-help in the treatment of outpatients with bulimia nervosa. JOURNAL OF CLINICAL PSYCHOPHARMACOLOGY. 2001;21(3):298-304.

2886. Urdapilleta I, Mirabel-Sarron C, Meunier JM, Richard JF Study of the categorization process among patients with eating disorders: a new cognitive approach to psychopathology. ENCEPHALE-REVUE DE PSYCHIATRIE CLINIQUE BIOLOGIQUE ET THERAPEUTIQUE. 2005;31(1, 1):82-91.

2887. Pamies-Aubalat Lidia, Quiles Marcos Yolanda, Torregrosa Diez Maria Psychosocial profile related to disordered eating attitudes in Spanish adolescents. CURRENT

PSYCHOLOGY. 2022;():.

2888. Dang Hoang-Minh, Ho Ha, Weiss Bahr The `big four' health risk behaviors among Vietnamese adolescents: co-occurrence and socio-cultural risk factors. HEALTH PSYCHOLOGY AND BEHAVIORAL MEDICINE. 2022;10(1):379-398.

2889. Weintraub Daniel, Nirenberg Melissa Impulse Control and Related Disorders in Parkinson's Disease. NEURODEGENERATIVE DISEASES. 2013;11(2):63-71.

2890. Marino Joanna, Ertelt Troy, Lancaster Kathy, Steffen Kristine, Peterson Lisa, Zwaan Martina, Mitchell James The Emergence of Eating Pathology after Bariatric Surgery: A Rare Outcome with Important Clinical Implications. INTERNATIONAL JOURNAL OF EATING DISORDERS. 2012;45(2):179-184.

2891. Micali Nadia, Hagberg Katrina, Petersen Irene, Treasure Janet The incidence of eating disorders in the UK in 2000-2009: findings from the General Practice Research Database. BMJ OPEN. 2013;3(5):.

2892. Crisp A, Sedgwick P, Halek C, Joughin N, Humphrey H Why may teenage girls persist in smoking?. JOURNAL OF ADOLESCENCE. 1999;22(5):657-672.

2893. Kothari R., Solmi F., Treasure J., Micali N. The neuropsychological profile of children at high risk of developing an eating disorder. PSYCHOLOGICAL MEDICINE. 2013;43(7):1543-1554.

2894. Adambegan Mandana, Wagner Gudrun, Nader Ingo, Fernandez-Aranda Fernando, Treasure Janet, Karwautz Andreas Internalizing and Externalizing Behaviour Problems in Childhood Contribute to the Development of Anorexia and Bulimia Nervosa-A Study Comparing Sister Pairs. EUROPEAN EATING DISORDERS REVIEW. 2012;20(2):116-120.

2895. Tokumaru Toshiaki, Toyama Tadashi, Hara Akinori, Kitagawa Kiyoki, Yamamura Yuta, Nakagawa Shiori, Oshima Megumi, Miyagawa Taro, Sato Koichi, Ogura Hisayuki, Kitajima Shinji, Iwata Yasunori, Sakai Norihiko, Shimizu Miho, Furuichi Kengo, Hashiba Atsushi, Wada Takashi Association between Unhealthy Dietary Habits and Proteinuria Onset in a Japanese General Population: A Retrospective Cohort Study. NUTRIENTS. 2020;12(9):.

2896. Christiano B, Mizes JS Appraisal and coping deficits associated with eating disorders: Implications for treatment. COGNITIVE AND BEHAVIORAL PRACTICE. 1997;4(2):263-290.

2897. Tang Wymann, Ng Tricia, Wong Joseph, Ho Cyrus The Role of Serious Video Games in the Treatment of Disordered Eating Behaviors: Systematic Review. JOURNAL OF MEDICAL INTERNET RESEARCH. 2022;24(8):.

2898. Barnhill Anne, King Katherine, Kass Nancy, Faden Ruth The Value of Unhealthy Eating and the Ethics of Healthy Eating Policies. KENNEDY INSTITUTE OF ETHICS JOURNAL. 2014;24(3, SI):187-217.

2899. Hallward Laura, Nagata Jason, Rodgers Rachel, Ganson Kyle Examination of eating disorder psychopathology across sexual and gender identities among a Canadian sample. INTERNATIONAL JOURNAL OF EATING DISORDERS. 2023;56(3):604-615.

2900. Silen Yasmina, Sipila Pyry, Raevuori Anu, Mustelin Linda, Marttunen Mauri, Kaprio Jaakko, Keski-Rahkonen Anna Detection, treatment, and course of eating disorders in Finland: A population-based study of adolescent and young adult females and males. EUROPEAN EATING DISORDERS REVIEW. 2021;29(5):720-732.

2901. Felonis Christina, Juarascio Adrienne Hedonic hunger as a mechanism of action in outpatient cognitive behavioral therapy for bulimia nervosa. INTERNATIONAL JOURNAL OF EATING DISORDERS. 2020;53(9):1539-1543.

2902. Mellentin Angelina, Mejldal Anna, Guala Maria, Stoving Rene, Eriksen Lene, Stenager Elsebeth, Skot Lotte The Impact of Alcohol and Other Substance Use Disorders on Mortality in Patients With Eating Disorders: A Nationwide Register-Based Retrospective Cohort Study. AMERICAN JOURNAL OF PSYCHIATRY. 2022;179(1):46-57.

2903. Mussell MP, Binford RB, Fulkerson JA Eating disorders: Summary of risk factors, prevention programming, and prevention research. COUNSELING PSYCHOLOGIST. 2000;28(6):764-796.

2904. Schneider Lisa, Monaco Sara, Warren Michelle Elevated ghrelin level in women of normal weight with amenorrhea is related to disordered eating. FERTILITY AND STERILITY. 2008;90(1):121-128.

2905. Quintela Barbara, Carioca Antonio, Oliveira Juliana, Fraser Simon, Silva Junior Geraldo Dietary patterns and chronic kidney disease outcomes: A systematic review. NEPHROLOGY. 2021;26(7):603-612.

2906. Corno Giulia, Paquette Amelia, Monthuy-Blanc Johana, Ouellet Marilou, Bouchard Stephane The Relationship Between Women's Negative Body Image and Disordered Eating Behaviors During the COVID-19 Pandemic: A Cross-Sectional Study. FRONTIERS IN PSYCHOLOGY. 2022;13():.

2907. O'Dea JA, Abraham S Onset of disordered eating attitudes and behaviors in early adolescence: Interplay of pubertal status, gender, weight, and age. ADOLESCENCE. 1999;34(136):671-679.

2908. Musci Rashelle, Hart Shelley, Ialongo Nicholas Internalizing Antecedents and Consequences of Binge-Eating Behaviors in a Community-Based, Urban Sample of African American Females. PREVENTION SCIENCE. 2014;15(4):570-578.

2909. Wright A., Pritchard M. An examination of the relation of gender, mass media influence, and loneliness to disordered eating among college students. EATING AND WEIGHT DISORDERS-STUDIES ON ANOREXIA BULIMIA AND OBESITY. 2009;14(2-3):E144-E147.

2910. Lima Mariana, Nunes Fernanda, Custodio Isis, Carvalho Kamila, Canto Paula, Paiva Carlos, Crispim Cibele, Maia Yara Eating Earlier and More Frequently Is Associated With Better Diet Quality in Female Brazilian Breast Cancer Survivors Using Tamoxifen. JOURNAL OF THE ACADEMY OF NUTRITION AND DIETETICS. 2022;122(9):1688+.

2911. Bould H., Sovio U., Koupil I., Dalman C., Micali N., Lewis G., Magnusson C. Do eating disorders in parents predict eating disorders in children? Evidence from a Swedish cohort. ACTA PSYCHIATRICA SCANDINAVICA. 2015;132(1):51-59.

2912. Kenangil Guelay, Ozekmekci Sibel, Sohtaoglu Melis, Erginoz Ethem Compulsive Behaviors in Patients With Parkinson's Disease. NEUROLOGIST. 2010;16(3):192-195.

2913. Romano Kelly, Lipson Sarah, Beccia Ariel, Quatromoni Paula, Gordon Allegra, Murgueitio Jose Changes in the prevalence and sociodemographic correlates of eating disorder symptoms from 2013 to 2020 among a large national sample of US young adults: A repeated cross-sectional study. INTERNATIONAL JOURNAL OF EATING DISORDERS.

2022;55(6):776-789.

2914. Cassin Stephanie, Buchman Daniel, Leung Samantha, Kantarovich Karin, Hawa Aceel, Carter Adrian, Sockalingam Sanjeev Ethical, Stigma, and Policy Implications of Food Addiction: A Scoping Review. NUTRIENTS. 2019;11(4):.

2915. Wright Fiona, Bewick Bridgette, Barkham Michael, House Allan, Hill Andrew Co-occurrence of self-reported disordered eating and self-harm in UK university students. BRITISH JOURNAL OF CLINICAL PSYCHOLOGY. 2009;48():397-410.

2916. Fonseca Helena, Matos Margarida, Guerra Antonio, Pedro J. Are overweight adolescents at higher risk of engaging in unhealthy weight-control behaviours?. ACTA PAEDIATRICA. 2009;98(5):847-852.

2917. Manca Rosa, Bombillar Francisco, Glomski Chester, Pica Alessandra Obesity and immune system impairment: A global problem during the COVID-19 pandemic. INTERNATIONAL JOURNAL OF RISK \& SAFETY IN MEDICINE. 2022;33(2):193-208.

2918. Pines A. Lifestyle and diet in postmenopausal women. CLIMACTERIC. 2009;12(1):62-65.

2919. Hernandez-Rivero Isabel, Blechert Jens, Miccoli Laura, Naomi Eichin Katharina, Carmen Fernandez-Santaella M., Delgado-Rodriguez Rafael Emotional reactivity to binge food and erotic cues in women with bulimia nervosa symptoms. JOURNAL OF EATING DISORDERS. 2021;9(1):.

2920. Marcellini F., Giuli C., Papa R., Tirabassi G., Faloia E., Boscaro M., Polito A., Ciarapica D., Zaccaria M., Mocchegiani E. OBESITY AND BODY MASS INDEX (BMI) IN RELATION TO LIFE-STYLE AND PSYCHO-SOCIAL ASPECTS. ARCHIVES OF GERONTOLOGY AND GERIATRICS. 2009;49(1):195-206.

2921. Ayers John, Althouse Benjamin, Allem Jon-Patrick, Rosenquist J., Ford Daniel Seasonality in Seeking Mental Health Information on Google. AMERICAN JOURNAL OF PREVENTIVE MEDICINE. 2013;44(5):520-525.

2922. Masip Guiomar, Silventoinen Karri, Keski-Rahkonen Anna, Palviainen Teemu, Sipila Pyry, Kaprio Jaakko, Bogl Leonie The genetic architecture of the association between eating

behaviors and obesity: combining genetic twin modeling and polygenic risk scores. AMERICAN JOURNAL OF CLINICAL NUTRITION. 2020;112(4):956-966.

2923. Tondo Giacomo, De Marchi Fabiola, Terazzi Emanuela, Sacchetti Marta, Cantello Roberto Frontotemporal Dementia Presenting as Gambling Disorder: When a Psychiatric Condition Is the Clue to a Neurodegenerative Disease. COGNITIVE AND BEHAVIORAL NEUROLOGY. 2017;30(2):62-67.

2924. Imanda Aulia, Martini Santi, Artanti Kurnia Post Hypertension and Stroke: A Case Control Study. KESMAS-NATIONAL PUBLIC HEALTH JOURNAL. 2019;13(4):164-168.

2925. Sarra Sharon, Abar Caitlin Perceptions of control and disordered eating behaviors during college transitions. JOURNAL OF AMERICAN COLLEGE HEALTH. 2022;70(7):2091-2098.

2926. Arribas Pilar, Iranzo-Tatay Carmen, Rojo-Bofill Luis, Garcia-Blanco Ana, Conesa Llanos, Plumed Javier, Bofill-Moscardo Isabel, Livianos-Aldana Lorenzo, Rojo-Moreno Luis Changes in genetic and environmental influences on disordered eating between pre-menarche and post-menarche girls. A twin study. ACTAS ESPANOLAS DE PSIQUIATRIA. 2018;46(5):192-199.

2927. Allen Karina, Byrne Susan, Forbes David, Oddy Wendy Risk Factors for Full- and Partial-Syndrome Early Adolescent Eating Disorders: A Population-Based Pregnancy Cohort Study. JOURNAL OF THE AMERICAN ACADEMY OF CHILD AND ADOLESCENT PSYCHIATRY. 2009;48(8):800-809.

2928. Dittmer Nina, Jacobi Corinna, Voderholzer Ulrich Compulsive exercise in eating disorders: proposal for a definition and a clinical assessment. JOURNAL OF EATING DISORDERS. 2018;6():.

2929. Mastropietro Stefania, Favieri Francesca, Forte Giuseppe, Locuratolo Nicoletta, Mannarelli Daniela, Pauletti Caterina, Fattapposta Francesco, Casagrande Maria Behavioral Addictions Questionnaire (BAQ): Validation of a New Tool for the Screening of Multiple Addictive Behaviors in the Italian Population. INTERNATIONAL JOURNAL OF MENTAL HEALTH AND ADDICTION. 2022;():.

2930. Trompeter Nora, Bussey Kay, Forbes Miriam, Mitchison Deborah Emotion Dysregulation within the CBT-E Model of Eating Disorders: A Narrative Review. COGNITIVE

THERAPY AND RESEARCH. 2021;45(6):1021-1036.

2931. Taylor CB, Cameron RP, Newman MG, Junge J Issues related to combining risk factor reduction and clinical treatment for eating disorders in defined populations. JOURNAL OF BEHAVIORAL HEALTH SERVICES \& RESEARCH. 2002;29(1):81-90.

2932. Stewart Tiffany, Pollard Tarryn, Hildebrandt Tom, Wesley Nicole, Kilpela Lisa, Becker Carolyn The Female Athlete Body project study: 18-month outcomes in eating disorder symptoms and risk factors. INTERNATIONAL JOURNAL OF EATING DISORDERS. 2019;52(11):1291-1300.

2933. Bos Sandra, Soares Maria, Marques Mariana, Maia Berta, Pereira Ana, Nogueira Vasco, Valente Jose, Macedo Antonio Disordered eating behaviors and sleep disturbances. EATING BEHAVIORS. 2013;14(2):192-198.

2934. Zerwas Stephanie, Watson Hunna, Hofmeier Sara, Levine Michele, Hamer Robert, Crosby Ross, Runfola Cristin, Peat Christine, Shapiro Jennifer, Zimmer Benjamin, Moessner Markus, Kordy Hans, Marcus Marsha, Bulik Cynthia CBT4BN: A Randomized Controlled Trial of Online Chat and Face-to-Face Group Therapy for Bulimia Nervosa. PSYCHOTHERAPY AND PSYCHOSOMATICS. 2017;86(1):47-53.

2935. Abraham Suzanne, Boyd Catherine, Lal Maala, Luscombe Georgina, Taylor Alan Time since menarche, weight gain and body image awareness among adolescent girls: onset of eating disorders?. JOURNAL OF PSYCHOSOMATIC OBSTETRICS \& GYNECOLOGY. 2009;30(2):89-94.

2936. Escandon-Nagel Neli, Dada Gloria, Grau Antoni, Soriano Jose, Feixas Guillem EATING DISORDER PATIENTS EVOLUTION THREE YEARS AFTER INTAKE IN DAY HOSPITAL. REVISTA ARGENTINA DE CLINICA PSICOLOGICA. 2017;26(1):59-69.

2937. Mason Tyler, Heron Kristin Do depressive symptoms explain associations between binge eating symptoms and later psychosocial adjustment in young adulthood?. EATING BEHAVIORS. 2016;23():126-130.

2938. Braun DL, Sunday SR, Huang A, Halmi KA More males seek treatment for eating disorders. INTERNATIONAL JOURNAL OF EATING DISORDERS. 1999;25(4):415-424.

2939. Higgins Guy, Fletcher Paul, Shanahan William Lorcaserin: A review of its preclinical and clinical pharmacology and therapeutic potential. PHARMACOLOGY \& THERAPEUTICS. 2020;205():.

2940. Mond Jonathan, Marks Peta Beliefs of adolescent girls concerning the severity and prevalence of bulimia nervosa. AUSTRALIAN JOURNAL OF PSYCHOLOGY. 2007;59(2):87-93.

2941. HERPERTZDAHLMANN BM, WEWETZER C, REMSCHMIDT H THE PREDICTIVE VALUE OF DEPRESSION IN ANOREXIA-NERVOSA - RESULTS OF A 7-YEAR FOLLOW-UP-STUDY. ACTA PSYCHIATRICA SCANDINAVICA. 1995;91(2):114-119.

2942. Cella Stefania, Cipriano Annarosa, Aprea Cristina, Cotrufo Paolo Risk factors for binge eating severity among adolescent girls and boys. A structural equation modeling approach. APPETITE. 2022;169():.

2943. Aspen V., Stein R., Cooperberg J., Manwaring J., Barch D., Wilfley D. Selective processing of body image words in women at-risk for developing an eating disorder: A preliminary study. EATING AND WEIGHT DISORDERS-STUDIES ON ANOREXIA BULIMIA AND OBESITY. 2011;16(3):E199-E203.

2944. Escriva-Martinez Tamara, Miragall Marta, Herrero Rocio, Rodriguez-Arias Marta, Banos Rosa Eating behaviors, eating styles and body mass index during COVID-19 confinement in a college sample: a predictive model. JOURNAL OF EATING DISORDERS. 2022;10(1):.

2945. Levinson Cheri, Brosof Leigh, Ma Jackie, Fewell Laura, Lenze Eric Fear of food prospectively predicts drive for thinness in an eating disorder sample recently discharged from intensive treatment. EATING BEHAVIORS. 2017;27():45-51.

2946. Ziobrowski Hannah, Sonneville Kendrin, Eddy Kamryn, Crosby Ross, Micali Nadia, Horton Nicholas, Field Alison Maternal Eating Disorders and Eating Disorder Treatment Among Girls in the Growing Up Today Study. JOURNAL OF ADOLESCENT HEALTH. 2019;65(4):469-475.

2947. Friederich Hans-Christoph What will the Future of Psychobiological Research in Eating Disorders Look Like?. PSYCHOTHERAPIE PSYCHOSOMATIK MEDIZINISCHE PSYCHOLOGIE. 2015;65(1):11-13.

2948. Tong Lian, Ye Yan, Yan Qiong The moderating roles of bedtime activities and anxiety/depression in the relationship between attention-deficit/hyperactivity disorder symptoms and sleep problems in children. BMC PSYCHIATRY. 2018;18():.

2949. Fitzsimmons-Craft Ellen, Bardone-Cone Anna, Bulik Cynthia, Wonderlich Stephen, Crosby Ross, Engel Scott Examining an elaborated sociocultural model of disordered eating among college women: The roles of social comparison and body surveillance. BODY IMAGE. 2014;11(4):488-500.

2950. Baker Jessica, Munn-Chernoff Melissa, Lichtenstein Paul, Larsson Henrik, Maes Hermine, Kendler Kenneth Shared Familial Risk Between Bulimic Symptoms and Alcohol Involvement During Adolescence. JOURNAL OF ABNORMAL PSYCHOLOGY. 2017;126(5):506-518.

2951. Chang Cindy, Putukian Margot, Aerni Giselle, Diamond Alex, Hong Gene, Ingram Yvette, Reardon Claudia, Wolanin Andrew Mental health issues and psychological factors in athletes: detection, management, effect on performance and prevention: American Medical Society for Sports Medicine Position Statement-Executive Summary. BRITISH JOURNAL OF SPORTS MEDICINE. 2020;54(4):216-220.

2952. Darcy Alison, Fitzpatrick Kathleen, Manasse Stephanie, Datta Nandini, Klabunde Megan, Colborn Danielle, Aspen Vandana, Stiles-Shields Colleen, Labuschagne Zandre, Le Grange Daniel, Lock James Central coherence in adolescents with bulimia nervosa spectrum eating disorders. INTERNATIONAL JOURNAL OF EATING DISORDERS. 2015;48(5):487-493.

2953. Chamay-Weber C, Narring F, Michaud PA Partial eating disorders among adolescents: A review. JOURNAL OF ADOLESCENT HEALTH. 2005;37(5):417-427.

2954. Raj Kristin, Keane-Miller Casey, Golden Neville Hypomagnesemia in Adolescents With Eating Disorders Hospitalized for Medical Instability. NUTRITION IN CLINICAL PRACTICE. 2012;27(5):689-694.

2955. Pinhas-Hamiel O, Standiford D, Hamiel D, Dolan LM, Cohen R, Zeitler PS The type 2 family - A setting for development and treatment of adolescent type 2 diabetes mellitus. ARCHIVES OF PEDIATRICS \& ADOLESCENT MEDICINE. 1999;153(10):1063-1067.

2956. Fernandez-Aranda F, Crespo JM, Jimenez-Murcia S, Krug I, Vallejo-Ruiloba J Blindness and bulimia nervosa: A description of a case report and its treatment. INTERNATIONAL

JOURNAL OF EATING DISORDERS. 2006;39(3):263-265.

2957. Franzago Marica, Orecchini Elena, Porreca Annamaria, Mondanelli Giada, Orabona Ciriana, Dalla Ragione Laura, Di Nicola Marta, Stuppia Liborio, Vitacolonna Ester, Beccari Tommaso, Ceccarini Maria SLC6A4 DNA Methylation Levels and Serum Kynurenine/Tryptophan Ratio in Eating Disorders: A Possible Link with Psychopathological Traits?. NUTRIENTS. 2023;15(2):.

2958. Stice Eric, Onipede Z., Marti C. A meta-analytic review of trials that tested whether eating disorder prevention programs prevent eating disorder onset. CLINICAL PSYCHOLOGY REVIEW. 2021;87():.

2959. Tirlea Loredana, Truby Helen, Haines Terry Pragmatic, Randomized Controlled Trials of the Girls on the Go! Program to Improve Self-Esteem in Girls. AMERICAN JOURNAL OF HEALTH PROMOTION. 2016;30(4):231-241.

2960. Stice E, Trost A, Chase A Healthy weight control and dissonance-based eating disorder prevention programs: results from a controlled trial. INTERNATIONAL JOURNAL OF EATING DISORDERS. 2003;33(1):10-21.

2961. Lochner C, Hemmings SMJ, Kinnear CJ, Niehaus DJH, Nel DG, Corfield VA, Moolman-Smook JC, Seedat S, Stein DJ Cluster analysis of obsessive-compulsive spectrum disorders in patients with obsessive-compulsive disorder: clinical and genetic correlates. COMPREHENSIVE PSYCHIATRY. 2005;46(1):14-19.

2962. Gratacos Monica, Gonzalez Juan, Mercader Josep, Cid Rafael, Urretavizcaya Mikel, Estivill Xavier Brain-derived neurotrophic factor Val66Met and psychiatric disorders: Meta-analysis of case-control studies confirm association to substance-related disorders, eating disorders, and schizophrenia. BIOLOGICAL PSYCHIATRY. 2007;61(7):911-922.

2963. Javier Sarah, Belgrave Faye An Examination of Influences on Body Dissatisfaction Among Asian American College Females: Do Family, Media, or Peers Play a Role?. JOURNAL OF AMERICAN COLLEGE HEALTH. 2015;63(8):579-583.

2964. Calugi Simona, Franchini Cecilia, Pivari Silvia, Conti Maddalena, El Ghoch Marwan, Dalle Grave Riccardo Anorexia nervosa and childhood sexual abuse: Treatment outcomes of intensive enhanced cognitive behavioural therapy. PSYCHIATRY RESEARCH.

2018;262():477-481.

2965. Fitzsimmons-Craft Ellen Social psychological theories of disordered eating in college women: Review and integration. CLINICAL PSYCHOLOGY REVIEW. 2011;31(7):1224-1237.

2966. Bahreynian Maryam, Mozafarian Nafiseh, Motlagh Mohammad, Qorbani Mostafa, Heshmat Ramin, Kelishadi Roya Association between parental feeding practices and later body mass index in children and adolescents: The Weight disorder survey of the CASPIAN-IV Study. MEDITERRANEAN JOURNAL OF NUTRITION AND METABOLISM. 2019;12(1):119-130.

2967. Hill Erin, Ruark Robert An examination of the role of social comparison orientation and social norms in drunkorexia engagement. ADDICTIVE BEHAVIORS. 2022;124():.

2968. Thomsen PH Obsessions: the impact and treatment of obsessive-compulsive disorder in children and adolescents. JOURNAL OF PSYCHOPHARMACOLOGY. 2000;14(2, 1):S31-S37.

2969. Toman E Body mass index and its impact on the therapeutic alliance in the work with eating disorder patients. EUROPEAN EATING DISORDERS REVIEW. 2002;10(3):168-178.

2970. Mustelin Linda, Latvala Antti, Raevuori Anu, Rose Richard, Kaprio Jaakko, Keski-Rahkonen Anna Risky drinking behaviors among women with eating disordersA longitudinal community-based study. INTERNATIONAL JOURNAL OF EATING DISORDERS. 2016;49(6):563-571.

2971. Barberis Nadia, Gugliandolo Maria, Costa Sebastiano, Cannavo Marco Healthy and binge eating behaviours: the motivational processes underlying peer pressure. PSYCHOLOGY HEALTH \& MEDICINE. 2022;27(5):1144-1153.

2972. Papanek PE The female athlete triad: An emerging role for physical therapy. JOURNAL OF ORTHOPAEDIC \& SPORTS PHYSICAL THERAPY. 2003;33(10):594-614.

2973. Ion Raluca, Popescu Cristian INCOME INFLUENCE ON DIET AND HEALTH. QUALITY-ACCESS TO SUCCESS. 2018;19(1):254-259.

2974. Stice Eric, Rohde Paul, Shaw Heather, Gau Jeff Clinician-Led, Peer-Led, and Internet-Delivered Dissonance-Based Eating Disorder Prevention Programs: Acute Effectiveness of

These Delivery Modalities. JOURNAL OF CONSULTING AND CLINICAL PSYCHOLOGY. 2017;85(9):883-895.

2975. Ackard DM, Peterson CB Association between puberty and disordered eating, body image, and other psychological variables. INTERNATIONAL JOURNAL OF EATING DISORDERS. 2001;29(2):187-194.

2976. Guldan Georgia Asian Children's Obesogenic DietsTime to Change This Part of the Energy Balance Equation?. RESEARCH IN SPORTS MEDICINE. 2010;18(1):5-15.

2977. Akers Laura, Rohde Paul, Stice Eric, Butryn Meghan, Shaw Heather Cost-effectiveness of achieving clinical improvement with a dissonance-based eating disorder prevention program. EATING DISORDERS. 2017;25(3):263-272.

2978. Patton George, Coffey Carolyn, Carlin John, Sanci Lena, Sawyer Susan Prognosis of adolescent partial syndromes of eating disorder. BRITISH JOURNAL OF PSYCHIATRY. 2008;192(4):294-299.

2979. Kritsotakis George, Psarrou Maria, Vassilaki Maria, Androulaki Zacharenia, Philalithis Anastas Gender differences in the prevalence and clustering of multiple health risk behaviours in young adults. JOURNAL OF ADVANCED NURSING. 2016;72(9):2098-2113.

2980. Nestsiarovich Anastasiya, Kerner Bent, Mazurie Aurelien, Cannon Daniel, Hurwitz Nathaniel, Zhu Yiliang, Nelson Stuart, Oprea Tudor, Crisanti Annette, Tohen Mauricio, Perkins Douglas, Lambert Christophe Diabetes mellitus risk for 102 drugs and drug combinations used in patients with bipolar disorder. PSYCHONEUROENDOCRINOLOGY. 2020;112():.

2981. Rayment Dane, Asfaha Eden, Babiker Amir, Jaffa Tony Hyperthyroidism during refeeding in anorexia nervosa. INTERNATIONAL JOURNAL OF EATING DISORDERS. 2012;45(3):460-462.

2982. Telleria-Aramburu Nerea, Arroyo-Izaga Marta Risk factors of overweight/obesity-related lifestyles in university students: Results from the EHU12/24 study. BRITISH JOURNAL OF NUTRITION. 2022;127(6):914-926.

2983. Vigna Luisella, Morelli Federica, Agnelli Gianna, Napolitano Filomena, Ratto Daniela, Occhinegro Alessandra, Di Iorio Carmine, Savino Elena, Girometta Carolina, Brandalise Federico, Rossi Paola Hericium erinaceus Improves Mood and Sleep Disorders in Patients Affected by Overweight or Obesity: Could Circulating Pro-BDNF and BDNF Be Potential Biomarkers?. EVIDENCE-BASED COMPLEMENTARY AND ALTERNATIVE MEDICINE. 2019;2019():.

2984. Muscogiuri Giovanna, Barrea Luigi, Aprano Sara, Framondi Lydia, Di Matteo Rossana, Laudisio Daniela, Pugliese Gabriella, Savastano Silvia, Colao Annamaria, Project Opera Chronotype and Adherence to the Mediterranean Diet in Obesity: Results from the Opera Prevention Project. NUTRIENTS. 2020;12(5):.

2985. Wade T, Martin NG, Tiggemann M Genetic and environmental risk factors for the weight and shape concerns characteristic of bulimia nervosa. PSYCHOLOGICAL MEDICINE. 1998;28(4):761-771.

2986. Pace Cecilia, Muzi Stefania, Parolin Laura, Milesi Alberto, Tognasso Giacomo, Santona Alessandra Binge eating attitudes in community adolescent sample and relationships with interview-assessed attachment representations in girls: a multi-center study from North Italy. EATING AND WEIGHT DISORDERS-STUDIES ON ANOREXIA BULIMIA AND OBESITY. 2022;27(2):495-504.

2987. Yager Zali, O'Dea Jennifer Body image, dieting and disordered eating and activity practices among teacher trainees: implications for school-based health education and obesity prevention programs. HEALTH EDUCATION RESEARCH. 2009;24(3):472-482.

2988. Mitchell JE, Agras WS, Wilson GT, Halmi K, Kraemer H, Crow S A trial of a relapse prevention strategy in women with bulimia nervosa who respond to cognitive-behavior therapy. INTERNATIONAL JOURNAL OF EATING DISORDERS. 2004;35(4):549-555.

2989. Wong Siew, Manore Melinda, Pilolla Kari, Skoog Ingrid, Hill Darcie, Hand Taryn WAVE Project: Sport Nutrition Education Resources. JOURNAL OF YOUTH DEVELOPMENT. 2018;13(3, SI):275-283.

2990. Tabler Jennifer, Schmitz Rachel, Geist Claudia, Utz Rebecca, Smith Ken Reproductive Outcomes Among Women with Eating Disorders or Disordered Eating Behavior: Does Methodological Approach Shape Research Findings?. JOURNAL OF WOMENS HEALTH. 2018;27(11):1389-1399.

2991. Haase Anne, Mountford Victoria, Waller Glenn Associations Between Body Checking and Disordered Eating Behaviors in Nonclinical Women. INTERNATIONAL JOURNAL OF EATING DISORDERS. 2011;44(5):465-468.

2992. Vandenbrink D., Pauze E., Potvin Kent M. Strategies used by the Canadian food and beverage industry to influence food and nutrition policies. INTERNATIONAL JOURNAL OF BEHAVIORAL NUTRITION AND PHYSICAL ACTIVITY. 2020;17(1):.

2993. Landt Margarita, Furth Eric, Meulenbelt Ingrid, Bartels Meike, Hottenga Jouke, Slagboom P., Boomsma Dorret Association Study of the Estrogen Receptor I Gene (ESR1) in Anorexia Nervosa and Eating Disorders: No Replication Found. INTERNATIONAL JOURNAL OF EATING DISORDERS. 2014;47(2):211-214.

2994. Balantekin Katherine, Birch Leann, Savage Jennifer Family, friend, and media factors are associated with patterns of weight-control behavior among adolescent girls. EATING AND WEIGHT DISORDERS-STUDIES ON ANOREXIA BULIMIA AND OBESITY. 2018;23(2):215-223.

2995. Cecilia-Costa R., Volkening L., Laffel L. Factors associated with disordered eating behaviours in adolescents with Type 1 diabetes. DIABETIC MEDICINE. 2019;36(8):1020-1027.

2996. Tate Eleanor, Spruijt-Metz Donna, Pickering Trevor, Pentz Mary Two facets of stress and indirect effects on child diet through emotion-driven eating. EATING BEHAVIORS. 2015;18():84-90.

2997. Lanuza Fabian, Morales Gladys, Hidalgo-Rasmussen Carlos, Balboa-Castillo Teresa, Ortiz Manuel, Belmar Carlos, Munoz Sergio Association between eating habits and quality of life among Chilean university students. JOURNAL OF AMERICAN COLLEGE HEALTH. 2022;70(1):280-286.

2998. Duda Pawel, Knysz Brygida, Gasiorowski Jacek, Szetela Bartosz, Piotrowska Ewa, Bronkowska Monika Assessment of dietary habits and lifestyle among people with HIV. ADVANCES IN CLINICAL AND EXPERIMENTAL MEDICINE. 2020;29(12):1459-1467.

2999. Wiegand S., Bau A., Babitsch B. Dietary interventions and social care for treating obesity in children. BUNDESGESUNDHEITSBLATT-GESUNDHEITSFORSCHUNG-

GESUNDHEITSSCHUTZ. 2011;54(5):533-540.

3000. KENNEDY SH, KATZ R, NEITZERT CS, RALEVSKI E, MENDLOWITZ S EXPOSURE WITH RESPONSE PREVENTION TREATMENT OF ANOREXIA-NERVOSA BULIMIC SUBTYPE AND BULIMIA-NERVOSA. BEHAVIOUR RESEARCH AND THERAPY. 1995;33(6):685-689.

3001. Rodgers Rachel, Chabrol Henri, Paxton Susan An exploration of the tripartite influence model of body dissatisfaction and disordered eating among Australian and French college women. BODY IMAGE. 2011;8(3):208-215.

3002. Al Sabbah Haleama, Vereecken Carine, Abdeen Ziad, Kelly Colette, Ojala Kristiina, Nemeth Agnes, Ahluwalia Namanjeet, Maes Lea Weight Control Behaviors among Overweight, Normal Weight and Underweight Adolescents in Palestine: Findings from the National Study of Palestinian Schoolchildren (HBSC-WBG2004). INTERNATIONAL JOURNAL OF EATING DISORDERS. 2010;43(4):326-336.

3003. Bradford Jennifer, Petrie Trent Sociocultural Factors and the Development of Disordered Eating: A Longitudinal Analysis of Competing Hypotheses. JOURNAL OF COUNSELING PSYCHOLOGY. 2008;55(2):246-262.

3004. Momen Natalie, Plana-Ripoll Oleguer, Yilmaz Zeynep, Thornton Laura, McGrath John, Bulik Cynthia, Petersen Liselotte Comorbidity between eating disorders and psychiatric disorders. INTERNATIONAL JOURNAL OF EATING DISORDERS. 2022;55(4):505-517.

3005. Sehm Marie, Warschburger Petra The Specificity of Psychological Factors Associated with Binge Eating in Adolescent Boys and Girls. JOURNAL OF ABNORMAL CHILD PSYCHOLOGY. 2015;43(8):1563-1571.

3006. Cecil Charlotte, Barker Edward, Walton Esther Prenatal diet and childhood ADHD: exploring the potential role of IGF2 methylation. EPIGENOMICS. 2016;8(12):1573-1576.

3007. Morris Jane, Anderson Stephen An update on eating disorders. BJPSYCH ADVANCES. 2021;27(1):9-19.

3008. Milos GF, Spindler AM, Buddeberg C, Crameri A Axes I and II comorbidity and treatment experiences in eating disorder subjects. PSYCHOTHERAPY AND

PSYCHOSOMATICS. 2003;72(5):276-285.

3009. Ursoniu Sorin, Putnoky Salomeia, Vlaicu Brigitha Body weight perception among high school students and its influence on weight management behaviors in normal weight students: a cross-sectional study. WIENER KLINISCHE WOCHENSCHRIFT. 2011;123(11-12):327-333.

3010. Pursey Kirrilly, Hay Phillipa, Bussey Kay, Trompeter Nora, Lonergan Alexandra, Pike Kathleen, Mond Jonathon, Mitchison Deborah Diabetes and disordered eating behaviours in a community-based sample of Australian adolescents. JOURNAL OF EATING DISORDERS. 2020;8(1):.

3011. Frankovich Jennifer, Thienemann Margo, Pearlstein Jennifer, Crable Amber, Brown Kayla, Chang Kiki Multidisciplinary Clinic Dedicated to Treating Youth with Pediatric Acute-Onset Neuropsychiatric Syndrome: Presenting Characteristics of the First 47 Consecutive Patients. JOURNAL OF CHILD AND ADOLESCENT PSYCHOPHARMACOLOGY. 2015;25(1, SI):38-47.

3012. Zullig K, Ubbes VA, Pyle J, Valois RF Self-reported weight perceptions, dieting behavior, and breakfast eating among high school adolescents. JOURNAL OF SCHOOL HEALTH. 2006;76(3):87-92.

3013. Lamerz A, Kuepper-Nybelen J, Bruning N, Wehle C, Trost-Brinkhues G, Brenner H, Hebebrand J, Herpertz-Dahlmann B Prevalence of obesity, binge eating, and night eating in a cross-sectional field survey of 6-year-old children and their parents in a German urban population. JOURNAL OF CHILD PSYCHOLOGY AND PSYCHIATRY. 2005;46(4):385-393.

3014. MAIESE K PROTEIN-KINASE-C MODULATES THE PROTECTIVE ABILITY OF PEPTIDE GROWTH-FACTORS DURING ANOXIA. JOURNAL OF THE AUTONOMIC NERVOUS SYSTEM. 1994;49(S):S187-S193.

3015. Kiguli Juliet, Alvesson Helle, Mayega Roy, Kasujja Francis, Muyingo Anthony, Kirunda Barbara, Kiracho Elizabeth, Nalwadda Christine, Naggayi Gloria, Peterson Stefan, Olmen Josefien, Daivadanam Meena Dietary patterns and practices in rural eastern Uganda: Implications for prevention and management of type 2 diabetes. APPETITE. 2019;143():.

3016. Ahamed Sayed, Barek Md, Roy Uthpall, Kouser Md, Reza Md, Mannan Afifa, Alam Md, Uddin S. A review on association and correlation of genetic variants with eating disorders

and obesity. FUTURE JOURNAL OF PHARMACEUTICAL SCIENCES. 2021;7(1):.

3017. Moore Malcolm Diverse Influences of Dietary Factors on Cancer in Asia. ASIAN PACIFIC JOURNAL OF CANCER PREVENTION. 2009;10(6):981-986.

3018. Convertino Alexandra, Brady John, Albright Christopher, Gonzales Manuel, Blashill Aaron The role of sexual minority stress and community involvement on disordered eating, dysmorphic concerns and appearance- and performance-enhancing drug misuse. BODY IMAGE. 2021;36():53-63.

3019. Candeias Vanessa, Armstrong Timothy, Xuereb Godfrey Diet and Physical Activity in Schools: Perspectives from the Implementation of the WHO Global Strategy on Diet, Physical Activity and Health. CANADIAN JOURNAL OF PUBLIC HEALTH-REVUE CANADIENNE DE SANTE PUBLIQUE. 2010;101(2):S28-S30.

3020. Akey Jessica, Rintamaki Lance, Kane Tera Health Belief Model deterrents of social support seeking among people coping with eating disorders. JOURNAL OF AFFECTIVE DISORDERS. 2013;145(2):246-252.

3021. Silva Jessica, Weber Maria Risk of nutrition female students developing eating disorders. RBONE-REVISTA BRASILEIRA DE OBESIDADE NUTRICAO E EMAGRECIMENTO. 2021;15(98, 1):1248-1255.

3022. Grisotto Giorgia, Langton Christine, Li Yanping, Bertone-Johnson Elizabeth, Baden Megu, Franco Oscar, Hu Frank, Muka Taulant, Eliassen A. Association of plant-based diet and early onset of natural menopause. MENOPAUSE-THE JOURNAL OF THE NORTH AMERICAN MENOPAUSE SOCIETY. 2022;29(7):861-867.

3023. Vestergaard P, Emborg C, Stoving RK, Hagen C, Mosekilde L, Brixen K Fractures in patients with anorexia nervosa, bulimia nervosa, and other eating disorders - A nationwide register study. INTERNATIONAL JOURNAL OF EATING DISORDERS. 2002;32(3):301-308.

3024. Tagay Sefik, Mewes Ricarda, Braehler Elmar, Senf Wolfgang Sense of Coherence in Female Patients with Bulimia Nervosa: a Protective Factor of Mental Health?. PSYCHIATRISCHE PRAXIS. 2009;36(1):30-34.

3025. Van Malderen Eva, Kemps Eva, Verbeken Sandra, Goossens Lien Food for mood: Experimentally induced negative affect triggers loss of control over eating in adolescents with low inhibitory control. INTERNATIONAL JOURNAL OF EATING DISORDERS. 2021;54(3):388-398.

3026. Mond JM, Hay PJ, Rodgers B, Owen C, Beumont PJV Beliefs of the public concerning the helpfulness of interventions for bulimia nervosa. INTERNATIONAL JOURNAL OF EATING DISORDERS. 2004;36(1):62-68.

3027. McFarlane Traci, Olmsted Marion, Trottier Kathryn Timing and Prediction of Relapse in a Transdiagnostic Eating Disorder Sample. INTERNATIONAL JOURNAL OF EATING DISORDERS. 2008;41(7):587-593.

3028. Cheng Zhen, Perko Victoria, Fuller-Marashi Leada, Gau Jeff, Stice Eric Ethnic differences in eating disorder prevalence, risk factors, and predictive effects of risk factors among young women. EATING BEHAVIORS. 2019;32():23-30.

3029. Bejerot Susanne, Hylen Ulrika, Glans Martin, Hesselmark Eva, Humble Mats Joint Hypermobility in Paediatric Acute-Onset Neuropsychiatric Syndrome-A Preliminary Case-Control Study. FRONTIERS IN PSYCHIATRY. 2021;12():.

3030. Rodriguez-Lopez Alvaro, Rodriguez-Ortiz Erika, Romero-Gonzalez Borja Non-suicidal self-injury in patients with eating disorders: nuclear aspects. COLOMBIA MEDICA. 2021;52(1):.

3031. Wonderlich SA, Connolly KM, Stice E Impulsivity as a risk factor for eating disorder behavior: Assessment implications with adolescents. INTERNATIONAL JOURNAL OF EATING DISORDERS. 2004;36(2):172-182.

3032. Gosliner Wendi, Shah Heena Participant voices: examining issue, program and policy priorities of SNAP-Ed eligible adults in California. RENEWABLE AGRICULTURE AND FOOD SYSTEMS. 2020;35(4, SI):407-415.

3033. Spangler DL Testing the cognitive model of eating disorders: The role of dysfunctional beliefs about appearance. BEHAVIOR THERAPY. 2002;33(1):87-105.

3034. O'Dor Sarah, Zagaroli J., Belisle R., Hamel M., Downer O., Homayoun S., Williams K. The COVID-19 pandemic and children with PANS/PANDAS: an evaluation of symptom severity, telehealth, and vaccination hesitancy. CHILD PSYCHIATRY \& HUMAN DEVELOPMENT. 2022;():.

3035. Cortes-Garcia Laura, Rodriguez-Cano Ruben, Soest Tilmann Prospective associations between loneliness and disordered eating from early adolescence to adulthood. INTERNATIONAL JOURNAL OF EATING DISORDERS. 2022;55(12):1678-1689.

3036. Bridle-Fitzpatrick Susan Food deserts or food swamps?: A mixed-methods study of local food environments in a Mexican city. SOCIAL SCIENCE \& MEDICINE. 2015;142():202-213.

3037. Federico Amianto, Spalatro Angela, Giorgio Ilari, Enrica Marzola, Daga Giovanni, Secondo Fassino Personality and psychopathology differences between bariatric surgery candidates, subjects with obesity not seeking surgery management, and healthy subjects. EATING AND WEIGHT DISORDERS-STUDIES ON ANOREXIA BULIMIA AND OBESITY. 2019;24(4):623-631.

3038. Aceijas Carmen, Waldhausl Sabrina, Lambert Nicky, Cassar Simon, Bello-Corassa Rafael Determinants of health-related lifestyles among university students. PERSPECTIVES IN PUBLIC HEALTH. 2017;137(4):227-236.

3039. Stice Eric, Rohde Paul, Butryn Meghan, Shaw Heather, Marti C. Effectiveness trial of a selective dissonance-based eating disorder prevention program with female college students: Effects at 2-and 3-year follow-up. BEHAVIOUR RESEARCH AND THERAPY. 2015;71():20-26.

3040. Hutchinson Delyse, Rapee Ronald Do friends share similar body image and eating problems? The role of social networks and peer influences in early adolescence. BEHAVIOUR RESEARCH AND THERAPY. 2007;45(7):1557-1577.

3041. Lewinsohn PM, Striegel-Moore RH, Seeley JR Epidemiology and natural course of eating disorders in young women from adolescence to young adulthood. JOURNAL OF THE AMERICAN ACADEMY OF CHILD AND ADOLESCENT PSYCHIATRY. 2000;39(10):1284-1292.

3042. O'Brien Amy, Anderson Rebecca, Mazzucchelli Trevor, Egan Sarah A protocol for unguided internet self-help cognitive behaviour therapy for perfectionism in adolescents at-

risk of eating disorders. INTERNET INTERVENTIONS-THE APPLICATION OF INFORMATION TECHNOLOGY IN MENTAL AND BEHAVIOURAL HEALTH. 2022;29():.

3043. Figee Martijn, Pattij Tommy, Willuhn Ingo, Luigjes Judy, Brink Wim, Goudriaan Anneke, Potenza Marc, Robbins Trevor, Denys Damiaan Compulsivity in obsessive-compulsive disorder and addictions. EUROPEAN NEUROPSYCHOPHARMACOLOGY. 2016;26(5, SI):856-868.

3044. Jesus Saucedo-Molina Teresita, Unikel Santoncini Claudia Disordered eating, internalization of the body thin-ideal and body mass index in high school and college students from a private institution in Hidalgo, Mexico. SALUD MENTAL. 2010;33(1):11-19.

3045. Gordon Eliza, Ariel-Donges Aviva, Bauman Viviana, Merlo Lisa What Is the Evidence for ``Food Addiction?{''} A Systematic Review. NUTRIENTS. 2018;10(4):.

3046. Torres-McGehee Toni, Emerson Dawn, Pritchett Kelly, Moore Erin, Smith Allison, Uriegas Nancy Energy Availability With or Without Eating Disorder Risk in Collegiate Female Athletes and Performing Artists. JOURNAL OF ATHLETIC TRAINING. 2021;56(9):993-1002.

3047. Tsukamoto Masayo, Tanaka Atsushi, Arai Motoe, Ishii Naoki, Ohta Daisuke, Horiki Noriyuki, Fujita Yoshiyuki Hepatocellular injuries observed in patients with an eating disorder prior to nutritional treatment. INTERNAL MEDICINE. 2008;47(16):1447-1450.

3048. DeBate Rita, Severson Herbert, Cragun Deborah, Gau Jeff, Merrell Laura, Bleck Jennifer, Christiansen Steve, Koerber Anne, Tomar Scott, Brown Kelli, Tedesco Lisa, Hendricson William Evaluation of a theory-driven e-learning intervention for future oral healthcare providers on secondary prevention of disordered eating behaviors. HEALTH EDUCATION RESEARCH. 2013;28(3):472-487.

3049. Lee S, Chan YYL, Hsu LKG The intermediate-term outcome of Chinese patients with anorexia nervosa in Hong Kong. AMERICAN JOURNAL OF PSYCHIATRY. 2003;160(5):967-972.

3050. Adamo Kristi, Brett Kendra Parental Perceptions and Childhood Dietary Quality. MATERNAL AND CHILD HEALTH JOURNAL. 2014;18(4):978-995.

3051. Katcher Julia, Suminski Richard, Pacanowski Carly Impact of an Intuitive Eating Intervention on Disordered Eating Risk Factors in Female-Identifying Undergraduates: A Randomized Waitlist-Controlled Trial. INTERNATIONAL JOURNAL OF ENVIRONMENTAL RESEARCH AND PUBLIC HEALTH. 2022;19(19):.

3052. Pretorius N., Arcelus J., Beecham J., Dawson H., Doherty F., Eisler I., Gallagher C., Gowers S., Isaacs G., Johnson-Sabine E., Jones A., Newell C., Morris J., Richards L., Ringwood S., Rowlands L., Simic M., Treasure J., Waller G., Williams C., Yi I., Yoshioka M., Schmidt U. Cognitive-behavioural therapy for adolescents with bulimic symptomatology: The acceptability and effectiveness of internet-based delivery. BEHAVIOUR RESEARCH AND THERAPY. 2009;47(9):729-736.

3053. Bhargava A, Hays J Behavioral variables and education are predictors of dietary change in the women's health trial: Feasibility Study in Minority Populations. PREVENTIVE MEDICINE. 2004;38(4):442-451.

3054. Gregorowski Claire, Seedat Soraya, Jordaan Gerhard A clinical approach to the assessment and management of co-morbid eating disorders and substance use disorders. BMC PSYCHIATRY. 2013;13():.

3055. McCormack Lynne, Lewis Vivienne, Wells Jonathan Early Life Loss and Trauma: Eating Disorder Onset in a Middle- Aged Male- A Case Study. AMERICAN JOURNAL OF MENS HEALTH. 2014;8(2):121-136.

3056. Lopez-Guimera Gemma, Levine Michael, Sanchez-Carracedo David, Fauquet Jordi Influence of Mass Media on Body Image and Eating Disordered Attitudes and Behaviors in Females: A Review of Effects and Processes. MEDIA PSYCHOLOGY. 2010;13(4):387-416.

3057. Dakanalis Antonios, Colmegna Fabrizia, Zanetti Maria, Di Giacomo Ester, Riva Giuseppe, Clerici Massimo Evaluation of the DSM-5 Severity Specifier for Bulimia Nervosa in Treatment-Seeking Youth. CHILD PSYCHIATRY \& HUMAN DEVELOPMENT. 2018;49(1):137-145.

3058. Moon Hasom, Chang Hyein Eating Expectancy as a Moderator of Sociocultural Influence on Bulimic Symptoms among High-Risk Female College Students. EMERGING ADULTHOOD. 2023;11(3):546-556.

3059. DeBoer Lindsey, Medina Johnna, Davis Michelle, Presnell Katherine, Powers Mark, Smits Jasper Associations Between Fear of Negative Evaluation and Eating Pathology During Intervention and 12-Month Follow-up. COGNITIVE THERAPY AND RESEARCH. 2013;37(5):941-952.

3060. McIntyre Roger, Cha Danielle, Jerrell Jeanette, Soczynska Joanna, Woldeyohannes Hanna, Taylor Valerie, Kaidanovich-Beilin Oksana, Alsuwaidan Mohammad, Ahmed Ameena Obesity and Mental Illness: Implications for Cognitive Functioning. ADVANCES IN THERAPY. 2013;30(6):577-588.

3061. Palmeroni Nina, Luyckx Koen, Verschueren Margaux, Claes Laurence Body Dissatisfaction as a Mediator between Identity Formation and Eating Disorder Symptomatology in Adolescents and Emerging Adults. PSYCHOLOGICA BELGICA. 2020;60(1):328-346.

3062. Dour Halina, Theran Sally The interaction between the superhero ideal and maladaptive perfectionism as predictors of unhealthy eating attitudes and body esteem. BODY IMAGE. 2011;8(1):93-96.

3063. Thunfors Peter, Collins Bradley, Hanlon Alexandra Health behavior interests of adolescents with unhealthy diet and exercise: implications for weight management. HEALTH EDUCATION RESEARCH. 2009;24(4):634-645.

3064. SHARP CW, CLARK SA, DUNAN JR, BLACKWOOD DHR, SHAPIRO CM CLINICAL PRESENTATION OF ANOREXIA-NERVOSA IN MALES - 24 NEW CASES. INTERNATIONAL JOURNAL OF EATING DISORDERS. 1994;15(2):125-134.

3065. Yalug Irem, Tural Umit, Atasoy Nuray, Konuk Numan, Erdogan Ayten, Oezten Eylem, Tufan Ali, Aker Tamer Co-morbidity of panic disorder with or without agoraphobia. NEUROLOGY PSYCHIATRY AND BRAIN RESEARCH. 2007;14(2):59-64.

3066. Xu Xiaoyue, Shi Zumin, Liu Gang, Chang Dennis, Inglis Sally, Hall John, Schutte Aletta, Byles Julie, Parker Deborah The Joint Effects of Diet and Dietary Supplements in Relation to Obesity and Cardiovascular Disease over a 10-Year Follow-Up: A Longitudinal Study of 69,990 Participants in Australia. NUTRIENTS. 2021;13(3):.

3067. Liu Xiaohua, Kelsoe John, Greenwood Tiffany, BiGS Bipolar A genome-wide association study of bipolar disorder with comorbid eating disorder replicates the SOX2-OT

region. JOURNAL OF AFFECTIVE DISORDERS. 2016;189():141-149.

3068. Puccio Francis, Fuller-Tyszkiewicz Matthew, Youssef George, Mitchell Sarah, Byrne Michelle, Allen Nick, Krug Isabel Longitudinal Bi-directional Effects of Disordered Eating, Depression and Anxiety. EUROPEAN EATING DISORDERS REVIEW. 2017;25(5):351-358.

3069. Muratori F, Viglione V, Maestro S, Picchi L Internalizing and externalizing conditions in adolescent anorexia. PSYCHOPATHOLOGY. 2004;37(2):92-97.

3070. Okano G, Mu Z, Lin ZY, Sato Y, Holmes R, Suzuki M, Nakai Y Low prevalence of disordered eating patterns and menstrual irregularities in Chinese female athletes. JAPANESE JOURNAL OF PHYSICAL FITNESS AND SPORTS MEDICINE. 1998;47(3):271-278.

3071. PARRYJONES B MERYCISM OR RUMINATION DISORDER - A HISTORICAL INVESTIGATION AND CURRENT ASSESSMENT. BRITISH JOURNAL OF PSYCHIATRY. 1994;165():303-314.

3072. Bobo William, Na Peter, Geske Jennifer, McElroy Susan, Frye Mark, Biernacka Joanna The relative influence of individual risk factors for attempted suicide in patients with bipolar I versus bipolar II disorder. JOURNAL OF AFFECTIVE DISORDERS. 2018;225():489-494.

3073. Watkins Beth, Cooper Peter, Lask Bryan History of Eating Disorder in Mothers of Children with Early Onset Eating Disorder or Disturbance. EUROPEAN EATING DISORDERS REVIEW. 2012;20(2):121-125.

3074. Pucarin-Cvetkovic Jasna, Sekerija Mario, Holcer Natasa Five-Year Cumulative Incidence of Unhealthy Diet in Adult Croatian Population: the CroHort Study. COLLEGIUM ANTROPOLOGICUM. 2012;36(1):95-98.

3075. Melamed Isaac, Kobayashi Roger, O'Connor Maeve, Kobayashi Ai, Schechterman Andrew, Heffron Melinda, Canterberry Sharon, Miranda Holly, Rashid Nazia Evaluation of Intravenous Immunoglobulin in Pediatric Acute-Onset Neuropsychiatric Syndrome. JOURNAL OF CHILD AND ADOLESCENT PSYCHOPHARMACOLOGY. 2021;31(2):118-128.

3076. Cooper Myra, Todd Gillian, Turner Hannah, Weiis Adrian Cognitive therapy for bulimia nervosa: An A-B replication series. CLINICAL PSYCHOLOGY \& PSYCHOTHERAPY.

2007;14(5):402-411.

3077. Nickel Marius, Simek Marietta, Lojewski Niluefer, Muehlbacher Moritz, Fartacek Reinhold, Kettler Christian, Bachler Egon, Egger Christoph, Rother Nadine, Buschmann Wiebke, Gil Francisco, Kaplan Patrick, Mitterlehner Ferdinand, Anvar Javaid, Rother Wolfhardt, Loew Thomas, Nickel Cerstin Familial and socilopsychopathological risk factors for suicide attempt in bulilmic and in depressed women: Prospective study. INTERNATIONAL JOURNAL OF EATING DISORDERS. 2006;39(5):410-417.

3078. Carlin Jesse, McKee Sarah, Hill-Smith Tiffany, Grissom Nicola, George Robert, Lucki Irwin, Reyes Teresa REMOVAL OF HIGH-FAT DIET AFTER CHRONIC EXPOSURE DRIVES BINGE BEHAVIOR AND DOPAMINERGIC DYSREGULATION IN FEMALE MICE. NEUROSCIENCE. 2016;326():170-179.

3079. Val-Laillet D., Aarts E., Weber B., Ferrari M., Quaresima V., Stoeckel L., Alonso-Alonso M., Audette M., Malbert C., Stice E. Neuroimaging and neuromodulation approaches to study eating behavior and prevent and treat eating disorders and obesity. NEUROIMAGE-CLINICAL. 2015;8():1-31.

3080. Stokes PE, Holtz A Fluoxetine tenth anniversary update: The progress continues. CLINICAL THERAPEUTICS. 1997;19(5):1135-1250.

3081. Bozzatello Paola, Rocca Paola, Baldassarri Lorenzo, Bosia Marco, Bellino Silvio The Role of Trauma in Early Onset Borderline Personality Disorder: A Biopsychosocial Perspective. FRONTIERS IN PSYCHIATRY. 2021;12():.

3082. Powell-Jones Alycia, Simpson Susan Drunkorexia: An investigation of symptomatology and early maladaptive schemas within a female, young adult Australian population. AUSTRALIAN PSYCHOLOGIST. 2020;55(5):559-571.

3083. Walitza S, Schulze U, Warnke A Differences between female patients with anorexia nervosa and bulimia nervosa in respect to psychological and psychosocial features. ZEITSCHRIFT FUR KINDER-UND JUGENDPSYCHIATRIE UND PSYCHOTHERAPIE. 2001;29(2):117-125.

3084. Agranat-Meged Anat, Ghanadri Yoad, Eisenber Iris, Ben Neriah Ziva, Kieselstein-Gross Eva, Mitrani-Rosenbaum Stella Attention Deficit Hyperactivity Disorder in Obese Melanocortin-4-Receptor (MC4R) Deficient Subjects: A Newly Described Expression of

MC4R Deficiency. AMERICAN JOURNAL OF MEDICAL GENETICS PART B-NEUROPSYCHIATRIC GENETICS. 2008;147B(8, SI):1547-1553.

3085. Laporta-Herrero Isabel, Jauregui-Lobera Ignacio, Barajas-Iglesias Belen, Angel Santed-German Miguel Body dissatisfaction in adolescents with eating disorders. EATING AND WEIGHT DISORDERS-STUDIES ON ANOREXIA BULIMIA AND OBESITY. 2018;23(3):339-347.

3086. Hsu Yu-Yun, Chen Bai-Hsium, Huang Mei-Chih, Lin Shio, Lin Mei-Feng Disturbed eating behaviors in Taiwanese adolescents with type 1 diabetes mellitus: a comparative study. PEDIATRIC DIABETES. 2009;10(1):74-81.

3087. Everett-Murphy K., De Villiers A., Ketterer E., Steyn K. Using formative research to develop a nutrition education resource aimed at assisting low-income households in South Africa adopt a healthier diet. HEALTH EDUCATION RESEARCH. 2015;30(6):882-896.

3088. Lopez Tabbetha, Arlinghaus Katherine, Johnston Craig Developing Adaptive Learning Environments to Support Long-Term Health Promotion. AMERICAN JOURNAL OF LIFESTYLE MEDICINE. 2019;13(1):30-32.

3089. Roth Binia, Munsch Simone, Meyer Andrea, Metzke Christa, Isler Emanuel, Steinhausen Hans-Christoph, Schneider Silvia The mental status of overweight children. ZEITSCHRIFT FUR KINDER-UND JUGENDPSYCHIATRIE UND PSYCHOTHERAPIE. 2008;36(3):163-176.

3090. Antonini Angelo, Barone Paolo, Bonuccelli Ubaldo, Annoni Karin, Asgharnejad Mahnaz, Stanzione Paolo ICARUS study: prevalence and clinical features of impulse control disorders in Parkinson's disease. JOURNAL OF NEUROLOGY NEUROSURGERY AND PSYCHIATRY. 2017;88(4):317-324.

3091. Shu Chloe, Watson Hunna, Anderson Rebecca, Wade Tracey, Kane Robert, Egan Sarah A randomized controlled trial of unguided internet cognitive behaviour therapy for perfectionism in adolescents: Impact on risk for eating disorders. BEHAVIOUR RESEARCH AND THERAPY. 2019;120():.

3092. Laghi Fiorenzo, Liga Francesca, Pompili Sara Adolescents who binge eat and drink: The role of emotion regulation. JOURNAL OF ADDICTIVE DISEASES. 2018;37(1-2):77-86.

3093. Brunner Eric, Mosdol Annhild, Witte Daniel, Martikainen Pekka, Stafford Mai, Shipley Martin, Marmot Michael Dietary patterns and 15-y risks of major coronary events, diabetes, and mortality. AMERICAN JOURNAL OF CLINICAL NUTRITION. 2008;87(5):1414-1421.

3094. Timmerman Gayle Restaurant eating in nonpurge binge-eating women. WESTERN JOURNAL OF NURSING RESEARCH. 2006;28(7):811-824.

3095. Herpertz-Dahlmann B, Muller B Early diagnosis of eating disorders in pubertal athletes. MONATSSCHRIFT KINDERHEILKUNDE. 2000;148(5):462-468.

3096. Riess H Integrative time-limited group therapy for bulimia nervosa. INTERNATIONAL JOURNAL OF GROUP PSYCHOTHERAPY. 2002;52(1):1-26.

3097. Gander Manuela, Sevecke Kathrin, Buchheim Anna Eating disorders in adolescence: attachment issues from a developmental perspective. FRONTIERS IN PSYCHOLOGY. 2015;6():.

3098. Boyd Hope, Kass Andrea, Accurso Erin, Goldschmidt Andrea, Wildes Jennifer, Le Grange Daniel Relationship between desired weight and eating disorder pathology in youth. INTERNATIONAL JOURNAL OF EATING DISORDERS. 2017;50(8):963-969.

3099. Juton Charlotte, Berruezo Paula, Torres Silvia, Castaner Olga, Segun Genis, Fito Montserrat, Homs Clara, Gomez Santiago, Schroder Helmut Association between Meal Frequency and Weight Status in Spanish Children: A Prospective Cohort Study. NUTRIENTS. 2023;15(4):.

3100. Antonini A., Chaudhuri K., Boroojerdi B., Asgharnejad M., Bauer L., Grieger F., Weintraub D. Impulse control disorder related behaviours during long-term rotigotine treatment: a post hoc analysis. EUROPEAN JOURNAL OF NEUROLOGY. 2016;23(10):1556-1565.

3101. Gomez Castillo Maria, Lopez Pina Jose, Torres Ortuno Ana, Lopez Duran Alicia, Ricarte Trives Jorge Parental eating disorders symptoms in different clinical diagnoses. PSICOTHEMA. 2018;30(4):382-387.

3102. Stice E, Chase A, Stormer S, Appel A A randomized trial of a dissonance-based eating disorder prevention program. INTERNATIONAL JOURNAL OF EATING DISORDERS.

2001;29(3):247-262.

3103. Wang Li, Nichols Lauren, Austin S. The Economic Effect of Planet Health on Preventing Bulimia Nervosa. ARCHIVES OF PEDIATRICS \& ADOLESCENT MEDICINE. 2011;165(8):756-762.

3104. Steinhausen Hans-Christoph, Jakobsen Helle, Helenius Dorte, Munk-Jorgensen Povl, Strober Michael A Nation-Wide Study of the Family Aggregation and Risk Factors in Anorexia Nervosa over Three Generations. INTERNATIONAL JOURNAL OF EATING DISORDERS. 2015;48(1):1-8.

3105. Fernanda Zeron-Rugerio Maria, Cambras Trinitat, Izquierdo-Pulido Maria Social Jet Lag Associates Negatively with the Adherence to the Mediterranean Diet and Body Mass Index among Young Adults. NUTRIENTS. 2019;11(8):.

3106. Si Yuqi, Wang Lihui, Zhao Min Anti-saccade as a Tool to Evaluate Neurocognitive Impairment in Alcohol Use Disorder. FRONTIERS IN PSYCHIATRY. 2022;13():.

3107. McElroy Susan, Hudson James, Mitchell James, Wilfley Denise, Ferreira-Cornwell M., Gao Joseph, Wang Jiannong, Whitaker Timothy, Jonas Jeffrey, Gasior Maria Efficacy and Safety of Lisdexamfetamine for Treatment of Adults With Moderate to Severe Binge-Eating Disorder A Randomized Clinical Trial. JAMA PSYCHIATRY. 2015;72(3):235-246.

3108. Bas Murat, Bozan Nuray, Cigerim Nevin DIETING, DIETARY RESTRAINT, AND BINGE EATING DISORDER AMONG OVERWEIGHT ADOLESCENTS IN TURKEY. ADOLESCENCE. 2008;43(171):635-648.

3109. Yokose Chio, McCormick Natalie, Lu Na, Joshi Amit, Curhan Gary, Choi Hyon Adherence to 2020 to 2025 Dietary Guidelines for Americans and the Risk of New-Onset Female Gout. JAMA INTERNAL MEDICINE. 2022;182(3):254-264.

3110. Chen Yi-Ting, Su Kuan-Pin, Chang Jane Atypical major depressive episode as initial presentation of intracranial germinoma in a male adolescent. NEUROPSYCHIATRIC DISEASE AND TREATMENT. 2017;13():35-40.

3111. Carter FA, McIntosh VVW, Joyce PR, Sullivan PF, Bulik CM Role of exposure with response prevention in cognitive-behavioral therapy for bulimia nervosa: Three-year

follow-up results. INTERNATIONAL JOURNAL OF EATING DISORDERS. 2003;33(2):127-135.

3112. Aljuraiban Ghadeer, Gibson Rachel, Griep Linda, Okuda Nagako, Steffen Lyn, Van Horn Linda, Chan Queenie Perspective: The Application of A Priori Diet Quality Scores to Cardiovascular Disease Risk-A Critical Evaluation of Current Scoring Systems. ADVANCES IN NUTRITION. 2020;11(1):10-24.

3113. Stone Kayla, Dimitropoulos Gina, MacMaster Frank Food for Thought: A Dissonance Between Healthcare Utilization Costs and Research Funding for Eating Disorders in Canada. JOURNAL OF THE CANADIAN ACADEMY OF CHILD AND ADOLESCENT PSYCHIATRY. 2021;30(3):197-203.

3114. Van Diest Ashley, Perez Marisol Exploring the integration of thin-ideal internalization and self-objectification in the prevention of eating disorders. BODY IMAGE. 2013;10(1):16-25.

3115. Brewster David, Nowell Sian, Clark David Risk of oesophageal cancer among patients previously hospitalised with eating disorder. CANCER EPIDEMIOLOGY. 2015;39(3):313-320.

3116. Zwaan M, Burgard MA, Schenck CH, Mitchell JE Night time eating: A review of the literature. EUROPEAN EATING DISORDERS REVIEW. 2003;11(1):7-24.

3117. Brown Catherine, Kola-Palmer Susanna, Dhingra Katie Gender differences and correlates of extreme dieting behaviours in US adolescents. JOURNAL OF HEALTH PSYCHOLOGY. 2015;20(5, SI):569-579.

3118. Anderson Emma, Wei Ruobin, Liu Binkai, Plummer Rachel, Kelahan Heather, Tamez Martha, Marrero Abrania, Bhupathiraju Shilpa, Mattei Josiemer Improving Healthy Food Choices in Low-Income Settings in the United States Using Behavioral Economic-Based Adaptations to Choice Architecture. FRONTIERS IN NUTRITION. 2021;8():.

3119. Demissie Zewditu, Lowry Richard, Eaton Danice, Nihiser Allison Trends in Weight Management Goals and Behaviors Among 9th-12th Grade Students: United States, 1999-2009. MATERNAL AND CHILD HEALTH JOURNAL. 2015;19(1):74-83.

3120. Sharma Ashish, Goyal Vinay, Behari Madhuri, Srivastva Achal, Shukla Garima, Vibha Deepti Impulse control disorders and related behaviours (ICD-RBs) in Parkinson's disease patients: Assessment using ``Questionnaire for impulsive-compulsive disorders in Parkinson's disease{''} (QUIP). ANNALS OF INDIAN ACADEMY OF NEUROLOGY. 2015;18(1):49-59.

3121. Koch Susanne, Larsen Janne, Plessen Kerstin, Thornton Laura, Bulik Cynthia, Petersen Liselotte Associations between parental socioeconomic-, family-, and sibling status and risk of eating disorders in offspring in a Danish national female cohort. INTERNATIONAL JOURNAL OF EATING DISORDERS. 2022;55(8):1130-1142.

3122. Domingo-Rodriguez Laura, Azua Inigo, Dominguez Eduardo, Senabre Eric, Serra Irene, Kummer Sami, Navandar Mohit, Baddenhausen Sarah, Hofmann Clementine, Andero Raul, Gerber Susanne, Navarrete Marta, Dierssen Mara, Lutz Beat, Martin-Garcia Elena, Maldonado Rafael A specific prelimbic-nucleus accumbens pathway controls resilience versus vulnerability to food addiction. NATURE COMMUNICATIONS. 2020;11(1):.

3123. Sollid Charlotte, Clausen Loa, Maimburg Rikke The first 20 weeks of pregnancy is a high-risk period for eating disorder relapse. INTERNATIONAL JOURNAL OF EATING DISORDERS. 2021;54(12):2132-2142.

3124. Ooi Cara, Kennedy James, Levitan Robert A Putative Model of Overeating and Obesity Based on Brain-Derived Neurotrophic Factor: Direct and Indirect Effects. BEHAVIORAL NEUROSCIENCE. 2012;126(4):505-514.

3125. Fitzgerald Elizabeth, Wick Madeline, Keel Pamela Enduring value of perfectionism and maturity fears for predicting eating disorder maintenance over 10-, 20-, and 30-year follow-up. INTERNATIONAL JOURNAL OF EATING DISORDERS. 2021;54(3):346-353.

3126. Bruno Veronica, Ruiz-Lopez Marta, Terroba-Chambi Cinthia, Freitas Maria, Rajalingam Rajasumi, Chang Anna, Fox Susan, Lang Anthony Rapid Eye Movement Sleep Behavior Disorder in Parkinson's Disease: A Survey-Based Study. CANADIAN JOURNAL OF NEUROLOGICAL SCIENCES. 2022;():.

3127. Hilger Jennifer, Loerbroks Adrian, Diehi Katharina Eating behaviour of university students in Germany: Dietary intake, barriers to healthy eating and changes in eating behaviour since the time of matriculation. APPETITE. 2017;109():100-107.

3128. Seidel Anke, Presnell Katherine, Rosenfield David Mediators in the dissonance eating disorder prevention program. BEHAVIOUR RESEARCH AND THERAPY. 2009;47(8):645-653.

3129. Mendelsohn Felicia, Warren Michelle Anorexia, Bulimia, and the Female Athlete Triad: Evaluation and Management. ENDOCRINOLOGY AND METABOLISM CLINICS OF NORTH AMERICA. 2010;39(1):155+.

3130. Ceravolo Roberto, Frosini Daniela, Rossi Carlo, Bonuccelli Ubaldo Impulse control disorders in Parkinson's disease: definition, epidemiology, risk factors, neurobiology and management. PARKINSONISM \& RELATED DISORDERS. 2009;15(4):S111-S115.

3131. Call Christine, D'Adamo Laura, Butryn Meghan, Stice Eric Examining weight suppression as a predictor and moderator of intervention outcomes in an eating disorder and obesity prevention trial: A replication and extension study. BEHAVIOUR RESEARCH AND THERAPY. 2021;141():.

3132. Gratchev V. Affective pathology in the premanifesting period of anorexia nervosa in adolescents. ZHURNAL NEVROLOGII I PSIKHIATRII IMENI S S KORSAKOVA. 2013;113(5, 2):69-74.

3133. Brietzke Elisa, Moreira Camila, Toniolo Ricardo, Lafer Beny Clinical correlates of eating disorder comorbidity in women with bipolar disorder type I. JOURNAL OF AFFECTIVE DISORDERS. 2011;130(1-2):162-165.

3134. Fontenelle LF, Mendlowicz MV, Soares ID, Versiani M Patients with obsessive-compulsive disorder and hoarding symptoms: A distinctive clinical subtype?. COMPREHENSIVE PSYCHIATRY. 2004;45(5):375-383.

3135. Eraslan Defne, Ozturk Ozgur, Bor Serhat Eating Disorder Symptoms Improved by Antireflux Surgery: A Case Report with a Six-Year Follow Up. ISRAEL JOURNAL OF PSYCHIATRY AND RELATED SCIENCES. 2009;46(3):231-235.

3136. Schwartz SA, Weissberg-Benchell J, Perlmuter LC Personal control and disordered eating in female adolescents with type 1 diabetes. DIABETES CARE. 2002;25(11):1987-1991.

3137. Sevelko Katrin, Bischof Gallus, Bischof Anja, Besser Bettina, John Ulrich, Meyer Christian, Rumpf Hans-Juergen The role of self-esteem in Internet addiction within the context of comorbid mental disorders: Findings from a general population-based sample. JOURNAL OF BEHAVIORAL ADDICTIONS. 2018;7(4):976-984.

3138. Abd El-Azeem Azza, Abu-Zaid Hany, Desouky Dalia Eating Disorders Among Female Students of Taif University, Saudi Arabia. ARCHIVES OF IRANIAN MEDICINE. 2018;21(3):111-117.

3139. Fiskum Charlotte, Riiber Ashild, Eik-Nes Trine Prevention of Unhealthy Weight, Disordered Eating, and Poor Body Image in Children. Perspectives From Norwegian Parents and Healthcare Professionals. FRONTIERS IN PSYCHIATRY. 2022;13():.

3140. Llado Gina, Gonzalez-Soltero Rocio, Jose Blanco Maria Anorexia and bulimia nervosa: virtual diffusion of the disease as a lifestyle. NUTRICION HOSPITALARIA. 2017;34(3):693-701.

3141. Ruuska J, Kaltiala-Heino R, Rantanen P, Koivisto AM Psychopathological distress predicts suicidal ideation and self-harm in Adolescent Eating Disorder outpatients. EUROPEAN CHILD \& ADOLESCENT PSYCHIATRY. 2005;14(5):276-281.

3142. Rome ES, Ammerman S, Rosen DS, Keller RJ, Lock J, Mammel KA, O'Toole J, Rees JM, Sanders MJ, Sawyer SM, Schneider M, Sigel E, Silber TJ Children and adolescents with eating disorders: The state of the art. PEDIATRICS. 2003;111(1):.

3143. Claus Nathalie, Miegel Franziska, Jelinek Lena, Landmann Sarah, Moritz Steffen, Kuelz Anne, Rubel Julian, Cludius Barbara Perfectionism as Possible Predictor for Treatment Success in Mindfulness-Based Cognitive Therapy and Metacognitive Training as Third-Wave Treatments for Obsessive-Compulsive Disorder. COGNITIVE THERAPY AND RESEARCH. 2023;47(3):439-453.

3144. Denisoff E, Endler NS Life experiences, coping, and weight preoccupation in young adult women. CANADIAN JOURNAL OF BEHAVIOURAL SCIENCE-REVUE CANADIENNE DES SCIENCES DU COMPORTEMENT. 2000;32(2):97-103.

3145. Forcano Laura, Fernandez-Aranda Fernando, Alvarez-Moya Eva, Bulik Cynthia, Granero Roser, Gratacos Monica, Jimenez-Murcia Susana, Krug Isabel, Mercader Josep, Riesco Nadine, Saus Ester, Jose Santamaria Juan, Estivill Xavier Suicide attempts in bulimia

nervosa: Personality and psychopathological correlates. EUROPEAN PSYCHIATRY. 2009;24(2):91-97.

3146. Conviser Jenny, Fisher Sheehan, Mitchell Kristin Oral care behavior after purging in a sample of women with bulimia nervosa. JOURNAL OF THE AMERICAN DENTAL ASSOCIATION. 2014;145(4):352-354.

3147. Ro O, Martinsen EW, Hoffart A, Rosenvinge J Two-year prospective study of personality disorders in adults with longstanding eating disorders. INTERNATIONAL JOURNAL OF EATING DISORDERS. 2005;37(2):112-118.

3148. Maestro S., Scardigli S., Brunori E., Calderoni S., Curzio O., Denoth F., Lorenzoni V., Molinaro S., Morales M., Muratori F. ANOREXIA NERVOSA AND HYPERACTIVITY IN ADOLESCENCE: PSYCHIATRIC AND INTERNISTIC FEATURES. MINERVA PEDIATRICA. 2014;66(4):237-248.

3149. PEKKARINEN T, KOSKELA K, HUIKURI K, MUSTAJOKI P LONG-TERM RESULTS OF GASTROPLASTY FOR MORBID-OBESITY - BINGE-EATING AS A PREDICTOR OF POOR OUTCOME. OBESITY SURGERY. 1994;4(3):248-255.

3150. Jimenez Cabanas M., Garcia Carpintero A., Perez Navarro V., Perez Moreno M. Could lockdown increase the incidence of eating disorders?. EUROPEAN PSYCHIATRY. 2021;64(1, SI):S223.

3151. Rijlaarsdam Jolien, Cecil Charlotte, Walton Esther, Mesirow Maurissa, Relton Caroline, Gaunt Tom, McArdle Wendy, Barker Edward Prenatal unhealthy diet, insulin-like growth factor 2 gene (IGF2) methylation, and attention deficit hyperactivity disorder symptoms in youth with early-onset conduct problems. JOURNAL OF CHILD PSYCHOLOGY AND PSYCHIATRY. 2017;58(1):19-27.

3152. Potterton Rachel, Austin Amelia, Allen Karina, Lawrence Vanessa, Schmidt Ulrike ``I'm not a teenager, I'm 22. Why can't I snap out of it?{''}: a qualitative exploration of seeking help for a first-episode eating disorder during emerging adulthood. JOURNAL OF EATING DISORDERS. 2020;8(1):.

3153. Key A, Lacey H Progress in eating disorder research. CURRENT OPINION IN PSYCHIATRY. 2002;15(2):143-148.

3154. Bodell Lindsay, Hames Jennifer, Holm-Denoma Jill, Smith April, Gordon Kathryn, Joiner Thomas Does the stress generation hypothesis apply to eating disorders?: An examination of stress generation in eating, depressive, and anxiety symptoms. JOURNAL OF AFFECTIVE DISORDERS. 2012;142(1-3):139-142.

3155. Stefanova Eliska, Baska Tibor, Boberova Zuzana, Husarova Daniela, Veselska Zuzana, Hudeckova Henrieta ``Voice of Children{''}: Qualitative analysis of children's interpretations regarding nutritional behaviour. ACTA GYMNICA. 2018;48(3):121-129.

3156. MINICHIELLO WE, OSULLIVAN RL, OSGOODHYNES D, BAER L TRICHOTILLOMANIA - CLINICAL ASPECTS AND TREATMENT STRATEGIES. HARVARD REVIEW OF PSYCHIATRY. 1994;1(6):336-344.

3157. Bleck Jennifer, DeBate Rita, Olivardia Roberto The Comorbidity of ADHD and Eating Disorders in a Nationally Representative Sample. JOURNAL OF BEHAVIORAL HEALTH SERVICES \& RESEARCH. 2015;42(4):437-451.

3158. Misra Madhusmita Long-term skeletal effects of eating disorders with onset in adolescence. . 2008;1135():212-218.

3159. Solomon Lynn, Merzianu Mihai, Sullivan Maureen, Rigual Nestor Necrotizing sialometaplasia associate with bulimia: case report and literature review. ORAL SURGERY ORAL MEDICINE ORAL PATHOLOGY ORAL RADIOLOGY AND ENDODONTOLOGY. 2007;103(2):E39-E42.

3160. Zahra J., Ford T., Jodrell D. Cross-sectional survey of daily junk food consumption, irregular eating, mental and physical health and parenting style of British secondary school children. CHILD CARE HEALTH AND DEVELOPMENT. 2014;40(4):481-491.

3161. Berkowitz Staci, Witt Ashley, Gillberg Christopher, Rastam Maria, Wentz Elisabet, Lowe Michael Childhood body mass index in adolescent-onset anorexia nervosa. INTERNATIONAL JOURNAL OF EATING DISORDERS. 2016;49(11):1002-1009.

3162. Bratland-Sanda Solfrid, Sundgot-Borgen Jorunn, Myklebust Grethe Injuries and musculoskeletal pain among Norwegian group fitness instructors. EUROPEAN JOURNAL OF SPORT SCIENCE. 2015;15(8):784-792.

3163. Sanci Lena, Coffey Carolyn, Epi Grad, Olsson Craig, Reid Sophie, Carlin John, Patton George Childhood sexual abuse and eating disorders in females. ARCHIVES OF PEDIATRICS \& ADOLESCENT MEDICINE. 2008;162(3):261-267.

3164. Hawkins Katherine, Linvill Darren Public Health Framing of News Regarding Childhood Obesity in the United States. HEALTH COMMUNICATION. 2010;25(8):709-717.

3165. Womble LG, Williamson DA, Martin CK, Zucker NL, Thaw JM, Netemeyer R, Lovejoy JC, Greenway FL Psychosocial variables associated with binge eating in obese males and females. INTERNATIONAL JOURNAL OF EATING DISORDERS. 2001;30(2):217-221.

3166. Marazziti D., Dell'Osso M. The role of oxytocin in neuropsychiatric disorders. CURRENT MEDICINAL CHEMISTRY. 2008;15(7):698-704.

3167. Serdar Kasey, Kelly Nichole, Palmberg Allison, Lydecker Janet, Thornton Laura, Tully Carrie, Mazzeo Suzanne Comparing Online and Face-to-Face Dissonance-Based Eating Disorder Prevention. EATING DISORDERS. 2014;22(3):244-260.

3168. Grant Jon, Potenza Marc Gender-related differences in individuals seeking treatment for kleptomania. CNS SPECTRUMS. 2008;13(3):235-245.

3169. Lundgren JD, Danoff-Burg S, Anderson DA Cognitive-behavioral therapy for bulimia nervosa: An empirical analysis of clinical significance. INTERNATIONAL JOURNAL OF EATING DISORDERS. 2004;35(3):262-274.

3170. Smitka Kvido, Papezova Hana, Vondra Karel, Hill Martin, Hainer Vojtech, Nedvidkova Jara The Role of ``Mixed'' Orexigenic and Anorexigenic Signals and Autoantibodies Reacting with Appetite-Regulating Neuropeptides and Peptides of the Adipose Tissue-Gut-Brain Axis: Relevance to Food Intake and Nutritional Status in Patients with Anorexia Nervosa and Bulimia Nervosa. INTERNATIONAL JOURNAL OF ENDOCRINOLOGY. 2013;2013():.

3171. Pena Francisco, Rosetti Marcos, Rodriguez-Delgado Andres, Villavicencio Lino, Palacio Juan, Montiel Cecilia, Mayer Pablo, Felix Fernando, Larraguibel Marcela, Viola Laura, Ortiz Silvia, Fernandez Sofia, Jaimes Aurora, Feria Miriam, Sosa Liz, Palacios-Cruz Lino, Ulloa Rosa Construct validity and parent-child agreement of the six new or modified disorders included in the Spanish version of the Kiddie Schedule for Affective Disorders and Schizophrenia present and Lifetime Version DSM-5 (K-SADS-PL-5). JOURNAL OF PSYCHIATRIC RESEARCH.

2018;101():28-33.

3172. Miotto Paola, Preti Antonio Eating disorders and suicide ideation: the mediating role of depression and aggressiveness. COMPREHENSIVE PSYCHIATRY. 2007;48(3):218-224.

3173. Stice Eric, Rohde Paul, Shaw Heather, Marti C. Efficacy Trial of a Selective Prevention Program Targeting Both Eating Disorders and Obesity Among Female College Students: 1- and 2-Year Follow-Up Effects. JOURNAL OF CONSULTING AND CLINICAL PSYCHOLOGY. 2013;81(1):183-189.

3174. SCARANO GM, KALODNERMARTIN CR A DESCRIPTION OF THE CONTINUUM OF EATING DISORDERS - IMPLICATIONS FOR INTERVENTION AND RESEARCH. JOURNAL OF COUNSELING AND DEVELOPMENT. 1994;72(4):356-361.

3175. Fantini M., Macedo L., Zibetti M., Sarchioto M., Vidal T., Pereira B., Marques A., Debilly B., Derost P., Ulla M., Vitello N., Cicolin A., Lopiano L., Durif F. Increased risk of impulse control symptoms in Parkinson's disease with REM sleep behaviour disorder. JOURNAL OF NEUROLOGY NEUROSURGERY AND PSYCHIATRY. 2015;86(2):174-179.

3176. Stice E, Shaw H, Nemeroff C Dual pathway model of bulimia nervosa: Longitudinal support for dietary restraint and affect-regulation mechanisms. JOURNAL OF SOCIAL AND CLINICAL PSYCHOLOGY. 1998;17(2):129-149.

3177. Stettler N, Tershakovec AM, Leonard MB Onset of adolescent eating disorders - Dieting may be an early sign, rather than a cause, of eating disorder. BRITISH MEDICAL JOURNAL. 1999;318(7200):1761.

3178. Laghi Fiorenzo, Bianchi Dora, Pompili Sara, Lonigro Antonia, Baiocco Roberto Binge eating and binge drinking behaviors: the role of family functioning. PSYCHOLOGY HEALTH \& MEDICINE. 2021;26(4):408-420.

3179. Cockell SJ, Zaitsoff SL, Geller J Maintaining change following eating disorder treatment. PROFESSIONAL PSYCHOLOGY-RESEARCH AND PRACTICE. 2004;35(5):527-534.

3180. Ignarro Louis, Balestrieri Maria, Napoli Claudio Nutrition, physical activity, and cardiovascular disease: An update. CARDIOVASCULAR RESEARCH. 2007;73(2):326-340.

3181. Tay Tuan, Bechade Catherine, D'Andrea Ivana, St-Pierre Marie-Kim, Henry Mathilde, Roumier Anne, Tremblay Marie-Eve Microglia Gone Rogue: Impacts on Psychiatric Disorders across the Lifespan. FRONTIERS IN MOLECULAR NEUROSCIENCE. 2018;10():.

3182. Morgan Emily, Graham Meredith, Folta Sara, Seguin Rebecca A qualitative study of factors related to cardiometabolic risk in rural men. BMC PUBLIC HEALTH. 2016;16():.

3183. Tolentino Laica, Iqbal Asif, Rahman Shafiqur, Lutfy Kabirullah The Role of Beta-Endorphin in Food Deprivation-Mediated Increases in Food Intake and Binge-Eating. BRAIN SCIENCES. 2023;13(2):.

3184. Modaberi Shaghayegh, Andel Steven, Saemi Esmaeel, Joubert Lanae, Taheri Morteza Differences between boulderers and top rope climbers in the relationship between anxiety and disordered eating. SPORT SCIENCES FOR HEALTH. 2022;():.

3185. Ong Say, Wickramaratne Priya, Tang Min, Weissman Myrna Early childhood sleep and eating problems as predictors of adolescent and adult mood and anxiety disorders. JOURNAL OF AFFECTIVE DISORDERS. 2006;96(1-2):1-8.

3186. Rowe Sarah, Jordan Jennifer, McIntosh Virginia, Carter Frances, Bulik Cynthia, Joyce Peter Impact of borderline personality disorder on bulimia nervosa. AUSTRALIAN AND NEW ZEALAND JOURNAL OF PSYCHIATRY. 2008;42(12):1021-1029.

3187. Rosen P., Frohm A., Kottorp A., Friden C., Heijne A. Too little sleep and an unhealthy diet could increase the risk of sustaining a new injury in adolescent elite athletes. SCANDINAVIAN JOURNAL OF MEDICINE \& SCIENCE IN SPORTS. 2017;27(11):1364-1371.

3188. Dabek Jozefa, Skorus Pawel, Lepich Tomasz, Bajor Grzegorz, Gasior Zbigniew KNOWLEDGE OF CORONARY ARTERIOSCLEROSIS RISK FACTORS AND THEIR OCCURRENCE AND THE LIFESTYLES OF THE FIRST-YEAR MEDICAL STUDENTS. HEALTH PROBLEMS OF CIVILIZATION. 2018;12(2):78-87.

3189. Dodd Dorian, Crosby Ross, Cao Li, Gordon Kathryn, Wonderlich Stephen Borderline personality disorder symptoms as mediational mechanisms linking childhood trauma and nonsuicidal self-injury among women with bulimia nervosa. INTERNATIONAL JOURNAL OF EATING DISORDERS. 2022;55(3):372-381.

3190. Adom Theodosia, De Villiers Anniza, Puoane Thandi, Kengne Andre A Scoping Review of Policies Related to the Prevention and Control of Overweight and Obesity in Africa. NUTRIENTS. 2021;13(11):.

3191. Dybdal Daniel, Tolstrup Janne, Sildorf Stine, Boisen Kirsten, Svensson Jannet, Skovgaard Anne, Teilmann Grete Increasing risk of psychiatric morbidity after childhood onset type 1 diabetes: a population-based cohort study. DIABETOLOGIA. 2018;61(4):831-838.

3192. Patton GC Onset of adolescent eating disorders - Dieting may be an early sign, rather than a cause, of eating disorder - Reply. BRITISH MEDICAL JOURNAL. 1999;318(7200):1761-1762.

3193. Nolen-Hoeksema Susan, Wisco Blair, Lyubomirsky Sonja Rethinking Rumination. PERSPECTIVES ON PSYCHOLOGICAL SCIENCE. 2008;3(5):400-424.

3194. Abramovitch Amitai, Anholt Gideon, Cooperman Allison, Balkom Anton, Giltay Erik, Penninx Brenda, Oppen Patricia Body mass index in obsessive-compulsive disorder. JOURNAL OF AFFECTIVE DISORDERS. 2019;245():145-151.

3195. Dunker Karin, Carvalho Pedro, Amaral Ana Eating disorders prevention programs in Latin American countries: A systematic review. INTERNATIONAL JOURNAL OF EATING DISORDERS. 2023;56(4, SI):691-707.

3196. Koppenburg C., Saxer F., Vach W., Luechtenberg D., Goesele A. Eating disorder risks and awareness among female elite cyclists: an anonymous survey. BMC SPORTS SCIENCE MEDICINE AND REHABILITATION. 2022;14(1):.

3197. Shank Richard, Maryanoff Bruce Molecular pharmacodynamics, clinical therapeutics, and pharmacokinetics of topiramate. CNS NEUROSCIENCE \& THERAPEUTICS. 2008;14(2):120-142.

3198. DeBate Rita, Severson Herbert, Cragun Deborah, Bleck Jennifer, Gau Jeff, Merrell Laura, Cantwell Carley, Christiansen Steve, Koerber Anne, Tomar Scott, Brown Kelli, Tedesco Lisa, Hendricson William, Taris Mark Randomized Trial of Two e-Learning Programs for Oral Health Students on Secondary Prevention of Eating Disorders. JOURNAL OF DENTAL EDUCATION. 2014;78(1):5-15.

3199. Daneman D, Olmsted M, Rydall A, Maharaj S, Rodin G Eating disorders in young women with type 1 diabetes - Prevalence, problems and prevention. HORMONE RESEARCH. 1998;50(1):79-86.

3200. Noria Sabrena, Shelby Rita, Atkins Katelyn, Nguyen Ninh, Gadde Kishore Weight Regain After Bariatric Surgery: Scope of the Problem, Causes, Prevention, and Treatment. CURRENT DIABETES REPORTS. 2023;23(3):31-42.

3201. Rosa Sepulveda Ana, Moreno Alba, Beltran Lucia UPDATE OF INTERVENTIONS AIMED AT THE FAMILY CONTEXT IN EATING DISORDERS: THE ROLE OF THE PARENTS. REVISTA DE PSICOTERAPIA. 2020;31(115):49-62.

3202. Melles Hanna, Spix Michelle, Jansen Anita Avoidance in Anorexia Nervosa: Towards a research agenda. PHYSIOLOGY \& BEHAVIOR. 2021;238():.

3203. Puckett Leah Renal and electrolyte complications in eating disorders: a comprehensive review. JOURNAL OF EATING DISORDERS. 2023;11(1):.

3204. Torstveit MK, Sundgot-Borgen J The female athlete triad exists in both elite athletes and controls. MEDICINE AND SCIENCE IN SPORTS AND EXERCISE. 2005;37(9):1449-1459.

3205. Cook Erica, Powell Faye, Ali Nasreen, Penn-Jones Catrin, Ochieng Bertha, Constantinou Georgina, Randhawa Gurch `They Are Kids, Let Them Eat': A Qualitative Investigation into the Parental Beliefs and Practices of Providing a Healthy Diet for Young Children among a Culturally Diverse and Deprived Population in the UK. INTERNATIONAL JOURNAL OF ENVIRONMENTAL RESEARCH AND PUBLIC HEALTH. 2021;18(24):.

3206. Miyawaki Dai, Goto Ayako, Harada Tomoko, Yamauchi Tsuneo, Iwakura Yoshihiro, Terakawa Hiroki, Hirai Kaoru, Miki Yusuke, Harima Yuji, Inoue Koki High prevalence of shoplifting in patients with eating disorders. EATING AND WEIGHT DISORDERS-STUDIES ON ANOREXIA BULIMIA AND OBESITY. 2018;23(6):761-768.

3207. Milliren Carly, Richmond Tracy, Hudgins Joel Emergency Department Visits and Hospitalizations for Eating Disorders During the COVID-19 Pandemic. PEDIATRICS. 2023;151(1):.

3208. Javaras Kristin, Runfola Cristin, Thornton Laura, Agerbo Esben, Birgegard Andreas, Norring Claes, Yao Shuyang, Rastam Maria, Larsson Henrik, Lichtenstein Paul, Bulik Cynthia Sex- and Age-Specific Incidence of Healthcare-Register-Recorded Eating Disorders in the Complete Swedish 1979-2001 Birth Cohort. INTERNATIONAL JOURNAL OF EATING DISORDERS. 2015;48(8):1070-1081.

3209. Weintraub Daniel, Claassen Daniel Impulse Control and Related Disorders in Parkinson's Disease. . 2017;133():679-717.

3210. Matsunaga H, Kiriike N, Iwasaki Y, Miyata A, Matsui T, Nagata T, Yamagami S, Kaye WH Multi-impulsivity among bulimic patients in Japan. INTERNATIONAL JOURNAL OF EATING DISORDERS. 2000;27(3):348-352.

3211. Bothe Tim, Walker Jochen, Kroeger Christoph Gender-related differences in health-care and economic costs for eating disorders: A comparative cost-development analysis for anorexia and bulimia nervosa based on anonymized claims data. INTERNATIONAL JOURNAL OF EATING DISORDERS. 2022;55(1):61-75.

3212. Lydsdottir Linda, Howard Louise, Olafsdottir Halldora, Thome Marga, Tyrfingsson Petur, Sigurdsson Jon The Mental Health Characteristics of Pregnant Women With Depressive Symptoms Identified by the Edinburgh Postnatal Depression Scale. JOURNAL OF CLINICAL PSYCHIATRY. 2014;75(4):393-398.

3213. Groth-Marnat G, Michel N Dissociation, comorbidity of dissociative disorders, and childhood abuse in a community sample of women with current and past bulimia. SOCIAL BEHAVIOR AND PERSONALITY. 2000;28(3):279-292.

3214. Murray Stuart Updates in the treatment of eating disorders in 2019: a year in review in Eating Disorders: The Journal of Treatment \& Prevention. EATING DISORDERS. 2020;28(1):21-31.

3215. Krafchek Jennifer, Kronborg Leonie Academic Emotions Experienced by Academically High-Achieving Females Who Developed Disordered Eating. ROEPER REVIEW-A JOURNAL ON GIFTED EDUCATION. 2019;41(4):258-272.

3216. Hyseni L., Atkinson M., Bromley H., Orton L., Lloyd-Williams F., McGill R., Capewell S. The effects of policy actions to improve population dietary patterns and prevent diet-related non-communicable diseases: scoping review. EUROPEAN JOURNAL OF CLINICAL

NUTRITION. 2017;71(6):694-711.

3217. Fox John, Dean Madeleine, Whittlesea Anna The Experience of Caring For or Living with an Individual with an Eating Disorder: A Meta-Synthesis of Qualitative Studies. CLINICAL PSYCHOLOGY \& PSYCHOTHERAPY. 2017;24(1):103-125.

3218. Johnson Jennifer Using Research-Supported Group Treatments. JOURNAL OF CLINICAL PSYCHOLOGY. 2008;64(11):1206-1224.

3219. D'Innocenzo Santa, Biagi Carlotta, Lanari Marcello Obesity and the Mediterranean Diet: A Review of Evidence of the Role and Sustainability of the Mediterranean Diet. NUTRIENTS. 2019;11(6):.

3220. Abraham SF Dieting, body weight, body image and self-esteem in young women: doctors' dilemmas. MEDICAL JOURNAL OF AUSTRALIA. 2003;178(12):607-611.

3221. Driessen C., Cameron A., Thornton L., Lai S., Barnett L. Effect of changes to the school food environment on eating behaviours and/or body weight in children: a systematic review. OBESITY REVIEWS. 2014;15(12):968-982.

3222. Perez-Gaspar M, Gual P, Irala-Estevez J, Martinez-Gonzalez MA, Lahortiga F, Cervera S Prevalence of eating disorders in a representative sample of female adolescents from Navarra (Spain). MEDICINA CLINICA. 2000;114(13):481-486.

3223. Navarro-Prado Silvia, Schmidt-RioValle Jacqueline, Montero-Alonso Miguel, Fernandez-Aparicio Angel, Gonzalez-Jimenez Emilio Unhealthy Lifestyle and Nutritional Habits Are Risk Factors for Cardiovascular Diseases Regardless of Professed Religion in University Students. INTERNATIONAL JOURNAL OF ENVIRONMENTAL RESEARCH AND PUBLIC HEALTH. 2018;15(12):.

3224. Kaufman Kenneth Anticonvulsants in sports: Ethical considerations. EPILEPSY \& BEHAVIOR. 2007;10(2):268-271.

3225. Leone Alessandro, Angel Martinez-Gonzalez Miguel, Lahortiga-Ramos Francisca, Molero Santos Patricio, Bertoli Simona, Battezzati Alberto, Bes-Rastrollo Maira Adherence to the Mediterranean dietary pattern and incidence of anorexia and bulimia nervosa in

women: The SUN cohort. NUTRITION. 2018;54():19-25.

3226. Shank Lisa, Neyland M., Lavender Jason, Schindler Rachel, Solomon Senait, Hennigan Kathrin, Leu William, Schvey Natasha, Sbrocco Tracy, Jorgensen Sarah, Stephens Mark, Olsen Cara, Haigney Mark, Klein David, Quinlan Jeffrey, Yanovski Jack, Tanofsky-Kraff Marian Sex differences in metabolic syndrome components in adolescent military dependents at high-risk for adult obesity. PEDIATRIC OBESITY. 2020;15(8):.

3227. Steinhausen HC The outcome of anorexia nervosa in the 20th century. AMERICAN JOURNAL OF PSYCHIATRY. 2002;159(8):1284-1293.

3228. Watson Hunna, Von Holle Ann, Knoph Cecilie, Hamer Robert, Torgersen Leila, Reichborn-Kjennerud Ted, Stoltenberg Camilla, Magnus Per, Bulik Cynthia Psychosocial factors associated with bulimia nervosa during pregnancy: An internal validation study. INTERNATIONAL JOURNAL OF EATING DISORDERS. 2015;48(6):654-662.

3229. Cha Susan, Ihongbe Timothy, Masho Saba Racial and Gender Differences in Dating Violence Victimization and Disordered Eating Among US High Schools. JOURNAL OF WOMENS HEALTH. 2016;25(8):791-800.

3230. Reichelt A., Westbrook R., Morris M. Integration of reward signalling and appetite regulating peptide systems in the control of food-cue responses. BRITISH JOURNAL OF PHARMACOLOGY. 2015;172(22):5225-5238.

3231. Jasinska Agnes, Yasuda Marie, Burant Charles, Gregor Nicolette, Khatri Sara, Sweet Matthew, Falk Emily Impulsivity and inhibitory control deficits are associated with unhealthy eating in young adults. APPETITE. 2012;59(3):738-747.

3232. Stice E, Ragan J A preliminary controlled evaluation of an eating disturbance psychoeducational intervention for college students. INTERNATIONAL JOURNAL OF EATING DISORDERS. 2002;31(2):159-171.

3233. Broadley M., Zaremba N., Andrew B., Ismail K., Treasure J., White M., Stadler M. 25 Years of psychological research investigating disordered eating in people with diabetes: what have we learnt?. DIABETIC MEDICINE. 2020;37(3):401-408.

3234. Can Hafize, Ceber Esin, Sogukpinar Neriman, Saydam Birsen, Otles Semih, Ozenturk Gulsun Eating Habits, Knowledge about Cancer Prevention and the HPLP Scale in Turkish Adolescents. ASIAN PACIFIC JOURNAL OF CANCER PREVENTION. 2008;9(4):569-574.

3235. Morris Anne, Elliott Elizabeth, Madden Sloane Early-onset eating disorders in Australian children: A national surveillance study showing increased incidence. INTERNATIONAL JOURNAL OF EATING DISORDERS. 2022;55(12):1838-1842.

3236. Raich RM, Claraso JT, Giral MM Structural analysis of the influential variables on the onset of eating behavior disturbances. BEHAVIORAL PSYCHOLOGY-PSICOLOGIA CONDUCTUAL. 1997;5(1):55-70.

3237. Brand-Gothelf Ayelet, Leor Shani, Apter Alan, Fennig Silvana The Impact of Comorbid Depressive and Anxiety Disorders on Severity of Anorexia Nervosa in Adolescent Girls. JOURNAL OF NERVOUS AND MENTAL DISEASE. 2014;202(10):759-762.

3238. Joyce Fiona, Watson Hunna, Egan Sarah, Kane Robert Mediators between perfectionism and eating disorder psychopathology in a community sample. EATING BEHAVIORS. 2012;13(4):361-365.

3239. Mehler PS, Gray MC, Schulte M Medical complications of anorexia nervosa. JOURNAL OF WOMENS HEALTH. 1997;6(5):533-541.

3240. Szczepanska Elzbieta, Bielaszka Agnieszka, Kiciak Agata, Wanat-Kantoch Gabriela, Staskiewicz Wiktoria, Bialek-Dratwa Agnieszka, Kardas Marek The Project ``Colourful Means Healthy{''} as an Educational Measure for the Prevention of Diet-Related Diseases: Investigating the Impact of Nutrition Education for School-Aged Children on Their Nutritional Knowledge. INTERNATIONAL JOURNAL OF ENVIRONMENTAL RESEARCH AND PUBLIC HEALTH. 2022;19(20):.

3241. Lampis J., Cataudella S., Busonera A., De Simone S., Tommasi M. The moderating effect of gender role on the relationships between gender and attitudes about body and eating in a sample of Italian adolescents. EATING AND WEIGHT DISORDERS-STUDIES ON ANOREXIA BULIMIA AND OBESITY. 2019;24(1):3-11.

3242. Bremser Jennifer, Gallup Gordon From One Extreme to the Other: Negative Evaluation Anxiety and Disordered Eating as Candidates for the Extreme Female Brain.

EVOLUTIONARY PSYCHOLOGY. 2012;10(3):457-486.

3243. Dore Riccardo, Valenza Marta, Wang Xiaofan, Rice Kenner, Sabino Valentina, Cottone Pietro The inverse agonist of CB1 receptor SR141716 blocks compulsive eating of palatable food. ADDICTION BIOLOGY. 2014;19(5):849-861.

3244. Ziobrowski Hannah, Buka Stephen, Austin S., Duncan Alexis, Simone Melissa, Sullivan Adam, Horton Nicholas, Field Alison Child and adolescent maltreatment patterns and risk of eating disorder behaviors developing in young adulthood. CHILD ABUSE \& NEGLECT. 2021;120():.

3245. Kotagal S, Krahn LE, Slocumb N A putative link between childhood narcolepsy and obesity. SLEEP MEDICINE. 2004;5(2):147-150.

3246. Valente Martina, Brenner Rachel, Cesuroglu Tomris, Bunders-Aelen Joske, Syurina Elena ``And it snowballed from there{''}: The development of orthorexia nervosa from the perspective of people who self-diagnose. APPETITE. 2020;155():.

3247. Hoelcke Mats, Marcus Claude, Gillberg Christopher, Fernell Elisabeth Paediatric obesity: a neurodevelopmental perspective. ACTA PAEDIATRICA. 2008;97(6):819-821.

3248. Patricia Nunez-Rivas Hilda, Holst-Schumacher Ileana, Campos-Saborio Natalia New Diet Quality Index for children and adolescents in Costa Rica. NUTRICION HOSPITALARIA. 2020;37(1):65-72.

3249. Perry CL, McGuire MT, Neumark-Sztainer D, Story M Characteristics of vegetarian adolescents in a multiethnic urban population. JOURNAL OF ADOLESCENT HEALTH. 2001;29(6):406-416.

3250. Gowers SG, Shore A Development of weight and shape concerns in the aetiology of eating disorders. BRITISH JOURNAL OF PSYCHIATRY. 2001;179():236-242.

3251. Friedel S, Horro FF, Wermter AK, Geller F, Dempfle A, Reichwald K, Smidt J, Bronner G, Konrad K, Herpetz-Dahlmann B, Warnke A, Hemminger U, Linder M, Kiefl H, Goldschmidt HP, Siegfried W, Remschmidt H, Hinney A, Hebebrand J Mutation screen of the brain derived neurotrophic factor gene (BDNF): Identification of several genetic variants and association studies in patients with obesity, eating disorders, and attention-deficit/hyperactivity

disorder. AMERICAN JOURNAL OF MEDICAL GENETICS PART B-NEUROPSYCHIATRIC GENETICS. 2005;132B(1):96-99.

3252. CORWIN J, CONNELLY S, PAZ S, SCHWARTZ M, WIRTH JA CHART REVIEW OF RATE OF WEIGHT-GAIN IN EATING DISORDER PATIENTS TREATED WITH TRICYCLIC ANTIDEPRESSANTS OR FLUOXETINE. PROGRESS IN NEURO-PSYCHOPHARMACOLOGY \& BIOLOGICAL PSYCHIATRY. 1995;19(2):223-228.

3253. Entin Anna, Kaufman-Shriqui Vered, Naggan Lechaim, Vardi Hillel, Shahar Danit Parental Feeding Practices in Relation to Low Diet Quality and Obesity among LSES Children. JOURNAL OF THE AMERICAN COLLEGE OF NUTRITION. 2014;33(4):306-314.

3254. Klemm S, Blanz B Long-term development and treatment of a feeding and an eating disorder in early childhood. KINDHEIT UND ENTWICKLUNG. 2003;12(4):258-261.

3255. Mason Susan, Flint Alan, Roberts Andrea, Agnew-Blais Jessica, Koenen Karestan, Rich-Edwards Janet Posttraumatic Stress Disorder Symptoms and Food Addiction in Women by Timing and Type of Trauma Exposure. JAMA PSYCHIATRY. 2014;71(11):1271-1278.

3256. Gu Lian, Zou Yunling, Huang Yue, Liu Qiang, Chen Han, Chen Jue The effect of group cognitive behavior therapy on Chinese patients with anorexia nervosa: an open label trial. JOURNAL OF EATING DISORDERS. 2021;9(1):.

3257. Sunday SR, Reeman IM, Eckert E, Halmi KA Ten-year outcome in adolescent onset anorexia nervosa. JOURNAL OF YOUTH AND ADOLESCENCE. 1996;25(4):533-544.

3258. Green Melinda, Willis Mary, Fernandez-Kong Kristen, Reyes Shuhan, Linkhart Ruby, Johnson Molly, Thorne Tyler, Kroska Emily, Woodward Halley, Lindberg Jessica Dissonance-Based Eating Disorder Program Reduces Cardiac Risk: A Preliminary Trial. HEALTH PSYCHOLOGY. 2017;36(4):346-355.

3259. Keel Pamela, Heatherton Todd Weight Suppression Predicts Maintenance and Onset of Bulimic Syndromes at 10-Year Follow-Up. JOURNAL OF ABNORMAL PSYCHOLOGY. 2010;119(2):268-275.

3260. Tsai Meng-Che, Gan Shu-Ting, Lee Chih-Ting, Liang Yi-Lin, Lee Lan-Ting, Lin Sheng-Hsiang National population-based data on the incidence, prevalence, and psychiatric

comorbidity of eating disorders in Taiwanese adolescents and young adults. INTERNATIONAL JOURNAL OF EATING DISORDERS. 2018;51(11):1277-1284.

3261. Magriplis Emmanuela, Farajian Paul, Panagiotakos Demosthenes, Risvas Grigoris, Zampelas Antonis The relationship between behavioral factors, weight status and a dietary pattern in primary school aged children: The GRECO study. CLINICAL NUTRITION. 2019;38(1):310-316.

3262. Degortes Daniela, Zanetti Tatiana, Tenconi Elena, Santonastaso Paolo, Favaro Angela Childhood Obsessive-compulsive Traits in Anorexia Nervosa Patients, Their Unaffected Sisters and Healthy Controls: A Retrospective Study. EUROPEAN EATING DISORDERS REVIEW. 2014;22(4):237-242.

3263. Thompson RA, Sherman RT Athletes, athletic performance, and eating disorders: Healthier alternatives. JOURNAL OF SOCIAL ISSUES. 1999;55(2):317-337.

3264. Cornelissen Piers, Tovee Martin Targeting body image in eating disorders. CURRENT OPINION IN PSYCHOLOGY. 2021;41():71-77.

3265. Paixao Carolina, Oliveira Sara, Ferreira Claudia A comprehensive model of disordered eating among aesthetic athletic girls: Exploring the role of body image-related cognitive fusion and perfectionistic self- presentation. CURRENT PSYCHOLOGY. 2021;40(11):5727-5734.

3266. Budkevich Roman, Putilov Arcady, Tinkova Elena, Budkevich Elena Chronobiological traits predict the restrained, uncontrolled, and emotional eating behaviors of female university students. CHRONOBIOLOGY INTERNATIONAL. 2021;38(7):1032-1041.

3267. Heuman Amy, Scholl Juliann, Wilkinson Kenton Rural Hispanic Populations at Risk in Developing Diabetes: Sociocultural and Familial Challenges in Promoting a Healthy Diet. HEALTH COMMUNICATION. 2013;28(3):260-274.

3268. Brunstrom JM, Mitchell GL, Baguley TS Potential early-life predictors of dietary behaviour in adulthood: a retrospective study. INTERNATIONAL JOURNAL OF OBESITY. 2005;29(5):463-474.

3269. Cruz Ana, Goncalves-Pinho Manuel, Santos Joao, Coutinho Francisco, Brandao Isabel, Freitas Alberto Eating disorders-Related hospitalizations in Portugal: A nationwide study from 2000 to 2014. INTERNATIONAL JOURNAL OF EATING DISORDERS. 2018;51(10):1201-1206.

3270. Rohde Paul, Stice Eric, Marti C. Development and Predictive Effects of Eating Disorder Risk Factors During Adolescence: Implications for Prevention Efforts. INTERNATIONAL JOURNAL OF EATING DISORDERS. 2015;48(2):187-198.

3271. Ochoa-Aviles Angelica, Verstraeten Roosmarijn, Huybregts Lieven, Andrade Susana, Van Camp John, Donoso Silvana, Liliana Ramirez Patricia, Lachat Carl, Maes Lea, Kolsteren Patrick A school-based intervention improved dietary intake outcomes and reduced waist circumference in adolescents: a cluster randomized controlled trial. NUTRITION JOURNAL. 2017;16():.

3272. Akbaraly Tasnime, Shipley Martin, Ferrie Jane, Virtanen Marianna, Lowe Gordon, Hamer Mark, Kivimaki Mika Long-term Adherence to Healthy Dietary Guidelines and Chronic Inflammation in the Prospective Whitehall II Study. AMERICAN JOURNAL OF MEDICINE. 2015;128(2):152-U162.

3273. Jagielska Gabriela, Kacperska Iwona Outcome, comorbidity and prognosis in anorexia nervosa. PSYCHIATRIA POLSKA. 2017;51(2):205-218.

3274. Sagiv Eran, Hadlaczky Gergo, Sheetrit Noga, Gur Eitan, Horesh Netta, Gvion Yari The Fear of Losing-Nonsuicidal Self-Injury as a Protective Mechanism in Eating Disorders. FRONTIERS IN PSYCHIATRY. 2019;10():.

3275. Matheson Brittany, Eichen Dawn A Review of Childhood Behavioral Problems and Disorders in the Development of Obesity: Attention Deficit/Hyperactivity Disorder, Autism Spectrum Disorder, and Beyond. CURRENT OBESITY REPORTS. 2018;7(1):19-26.

3276. Powers PS, Santana CA Childhood and adolescent anorexia nervosa. CHILD AND ADOLESCENT PSYCHIATRIC CLINICS OF NORTH AMERICA. 2002;11(2):219+.

3277. Skinner Hayley, Haines Jess, Austin S., Field Alison A Prospective Study of Overeating, Binge Eating, and Depressive Symptoms Among Adolescent and Young Adult Women. JOURNAL OF ADOLESCENT HEALTH. 2012;50(5):478-483.

3278. Anckarsater Henrik, Lundstrom Sebastian, Kollberg Linnea, Kerekes Nora, Palm Camilla, Carlstrom Eva, Langstrom Niklas, Magnusson Patrik, Halldner Linda, Bolte Sven, Gillberg Christopher, Gumpert Clara, Rastam Maria, Lichtenstein Paul The Child and Adolescent Twin Study in Sweden (CATSS). TWIN RESEARCH AND HUMAN GENETICS. 2011;14(6):495-508.

3279. Thamotharan Sneha, Hubbard Meagan, Fields Sherecce Delay discounting, but not disinhibition or inattention, partially mediates the effects of neuroticism on disordered eating in adolescents. EATING BEHAVIORS. 2015;18():91-96.

3280. Novotny Daniela, Matthews Eric, Powell Sara Disordered Eating Attitudes and Behaviors of Dietetics Students. TOPICS IN CLINICAL NUTRITION. 2021;36(4):299-310.

3281. Stein S, Chalhoub N, Hodes M Very early-onset bulimia nervosa: Report of two cases. INTERNATIONAL JOURNAL OF EATING DISORDERS. 1998;24(3):323-327.

3282. Fazzino Tera, Raheel Amani, Peppercorn Natalie, Forbush Kelsie, Kirby Taylor, Sher Kenneth, Befort Christie Motives for drinking alcohol and eating palatable foods: An evaluation of shared mechanisms and associations with drinking and binge eating. ADDICTIVE BEHAVIORS. 2018;85():113-119.

3283. Platen P The female athlete's triad: eating disorders, menstrual cycle disturbances, and osteoporosis. MEDIZINISCHE WELT. 1999;50(9):363-368.

3284. Benson JE, EngelbertFenton KA, Eisenman PA Nutritional aspects of amenorrhea in the female athlete triad. INTERNATIONAL JOURNAL OF SPORT NUTRITION. 1996;6(2):134-145.

3285. Murray Stuart, Strober Michael, Craske Michelle, Griffiths Scott, Levinson Cheri, Strigo Irina Fear as a translational mechanism in the psychopathology of anorexia nervosa. NEUROSCIENCE AND BIOBEHAVIORAL REVIEWS. 2018;95():383-395.

3286. VANFURTH EF, VANSTRIEN DC, VANSON MJM, VANENGELAND H THE VALIDITY OF THE 5-MINUTE SPEECH SAMPLE AS AN INDEX OF EXPRESSED EMOTION IN PARENTS OF EATING DISORDER PATIENTS. JOURNAL OF CHILD PSYCHOLOGY AND PSYCHIATRY AND ALLIED DISCIPLINES. 1993;34(7):1253-1260.

3287. Torreglosa Camila, Sarti Flavia, Bersch-Ferreira Angela, Weber Bernardete, Nakagawa Santos Renato, Chiavegatto Filho Alexandre Quality of diet and daily spending on food by adults with cardiovascular disease in Brazil. CADERNOS DE SAUDE PUBLICA. 2020;36(10):.

3288. Al-Sarraf Hameed, Malatiali Slava, Al-Awadi Mariam, Redzic Zoran Effects of erythropoietin on astrocytes and brain endothelial cells in primary culture during anoxia depend on simultaneous signaling by other cytokines and on duration of anoxia. NEUROCHEMISTRY INTERNATIONAL. 2018;113():34-45.

3289. Baik Inkyung, Lee Myoungsook, Jun Nu-Ri, Lee Jae-Yeon, Shin Chol A healthy dietary pattern consisting of a variety of food choices is inversely associated with the development of metabolic syndrome. NUTRITION RESEARCH AND PRACTICE. 2013;7(3):233-241.

3290. Sommerfeldt Bente, Skarderud Finn, Kvalem Ingela, Gulliksen Kjersti, Holte Arne Bodies out of control: Relapse and worsening of eating disorders in pregnancy. FRONTIERS IN PSYCHOLOGY. 2022;13():.

3291. Marconi Sara, Rizzo Giovanni, Capellari Sabina, Scaglione Cesa, Cortelli Pietro, Martinelli Paolo, Bonazza Sara Eating Disorder as a Psychiatric Onset of Juvenile Huntington's Disease. AMERICAN JOURNAL OF PSYCHIATRY. 2011;168(10):1120-1121.

3292. Weissman Ruth The Role of Sociocultural Factors in the Etiology of Eating Disorders. PSYCHIATRIC CLINICS OF NORTH AMERICA. 2019;42(1, 1):121+.

3293. Steinhausen HC, Boyadjieva S, Grigoroiu-Serbanescu M, Seidel R, Metzke CW A transcultural outcome study of adolescent eating disorders. ACTA PSYCHIATRICA SCANDINAVICA. 2000;101(1):60-66.

3294. Caponnetto Pasquale, Casu Mirko, Amato Miriam, Cocuzza Dario, Galofaro Valeria, La Morella Alessandra, Paladino Sara, Pulino Kamil, Raia Nicoletta, Recupero Flavia, Resina Cristian, Russo Samuele, Terranova Laura, Tiralongo Jennifer, Vella Maria The Effects of Physical Exercise on Mental Health: From Cognitive Improvements to Risk of Addiction. INTERNATIONAL JOURNAL OF ENVIRONMENTAL RESEARCH AND PUBLIC HEALTH. 2021;18(24):.

3295. Pearson J, Goldklang D, Striegel-Moore RH Prevention of eating disorders: Challenges and opportunities. INTERNATIONAL JOURNAL OF EATING DISORDERS. 2002;31(3):233-

239.

3296. Giusti V, Suter M, Heraief E, Gaillard RC, Burckhardt P Rising role of obesity surgery caused by increase of morbid obesity, failure of conventional treatments and unrealistic expectations: Trends from 1997 to 2001. OBESITY SURGERY. 2003;13(5):693-698.

3297. Simone Melissa, Lockhart Ginger Two distinct mediated pathways to disordered eating in response to weight stigmatization and their application to prevention programs. JOURNAL OF AMERICAN COLLEGE HEALTH. 2016;64(7):520-526.

3298. Pauli Dagmar, Aebi Marcel, Metzke Christa, Steinhausen Hans-Christoph Motivation to change, coping, and selfesteem in adolescent anorexia nervosa: a validation study of the Anorexia Nervosa Stages of Change Questionnaire (ANSOCQ). JOURNAL OF EATING DISORDERS. 2017;5():.

3299. Olie Jean-Pierre, Gourion David, Canceil Olivier, Loo Henri Physiological adolescence, pathological adolescence. BULLETIN DE L ACADEMIE NATIONALE DE MEDECINE. 2006;190(8):1643-1652.

3300. Sebastiani Giorgia, Andreu-Fernandez Vicente, Herranz Barbero Ana, Aldecoa-Bilbao Victoria, Miracle Xavier, Meler Barrabes Eva, Balada Ibanez Arantxa, Astals-Vizcaino Marta, Ferrero-Martinez Silvia, Dolores Gomez-Roig Maria, Garcia-Algar Oscar Eating Disorders During Gestation: Implications for Mother's Health, Fetal Outcomes, and Epigenetic Changes. FRONTIERS IN PEDIATRICS. 2020;8():.

3301. Svendsen Mette, Heggen Eli, Klemsdal Tor, Tonstad Serena Diet, eating behaviour and weight gain in men and women with overweight/obesity receiving varenicline for smoking cessation. CLINICAL OBESITY. 2021;11(3):.

3302. San Mauro Martin Ismael, Garicano Vilar Elena, Mendive Dubourdieu Paula, Paredes Barato Victor, Garagarza Cristina, Morales Hurtado Alexis, Rincon Barrado Mario, Bentancor Fabiana, Valente Ana, Romo Orozco Denisse Diet quality, assessed by the Healthy Eating Index-2010, and exercise associated factors of obesity: a cross-sectional study. REVISTA ESPANOLA DE NUTRICION HUMANA Y DIETETICA. 2021;25(2):189-198.

3303. Torre Alberto, Perez-Garcia Ana, Torre Elvira, Mateo Silleras Beatriz Is an integral nutritional approach to eating disorders feasible in primary care?. BRITISH JOURNAL OF

NUTRITION. 2006;96(1):S82-S85.

3304. Rodgers Rachel, Ziff Sara, Lowy Alice, Yu Kimberly, Austin S. Results of a strategic science study to inform policies targeting extreme thinness standards in the fashion industry. INTERNATIONAL JOURNAL OF EATING DISORDERS. 2017;50(3, SI):284-292.

3305. Pardos-Gascon Estela, Gomez Calmaestra Nicolas, Rodriguez Vacas Maria GRID TECHNIQUE AS AN INSTRUMENT FOR EVALUATION OF EATING DISORDERS: EXPLORATION OF COGNITIVE STRUCTURE AND INTERPERSONAL RELATIONSHIPS IN A SAMPLE OF 20 PATIENTS. REVISTA DE PSICOTERAPIA. 2020;31(115):129-146.

3306. Burkhart N, Roberts M, Alexander M, Dodds A Communicating effectively with patients suspected of having bulimia nervosa. JOURNAL OF THE AMERICAN DENTAL ASSOCIATION. 2005;136(8):1130-1137.

3307. Harmon Brook, Blake Christine, Armstead Cheryl, Hebert James Intersection of identities. Food, role, and the African-American pastor. APPETITE. 2013;67():44-52.

3308. Becker Carolyn From Efficacy to Global Impact: Lessons Learned About What Not to Do in Translating Our Research to Reach. BEHAVIOR THERAPY. 2017;48(5):718-730.

3309. Ackard Diann, Richter Sara, Frisch Maria, Mangham Deborah, Cronemeyer Catherine Eating disorder treatment among women forty and older: Increases in prevalence over time and comparisons to young adult patients. JOURNAL OF PSYCHOSOMATIC RESEARCH. 2013;74(2):175-178.

3310. Kindermann Sally, Moessner Markus, Ozer Fikret, Bauer Stephanie Associations between eating disorder related symptoms and participants' utilization of an individualized Internet-based prevention and early intervention program. INTERNATIONAL JOURNAL OF EATING DISORDERS. 2017;50(10):1215-1221.

3311. Karatzias Thanos, Chouliara Zoe, Power Kevin, Collin Paula, Yellowlees Alex, Grierson David General Psychopathology in Anorexia Nervosa: The Role of Psychosocial Factors. CLINICAL PSYCHOLOGY \& PSYCHOTHERAPY. 2010;17(6):519-527.

3312. Nicholls Dasha, Yi Irene Early intervention in eating disorders: a parent group approach. EARLY INTERVENTION IN PSYCHIATRY. 2012;6(4):357-367.

3313. Modrzejewska Justyna, Modrzejewska Adriana, Czepczor-Bernat Kamila, Matusik Pawel The role of body mass index, healthy eating-related apps and educational activities on eating motives and behaviours among women during the COVID-19 pandemic: A cross sectional study. PLOS ONE. 2022;17(3):.

3314. Coelho Jennifer, Suen Janet, Clark Beth, Marshall Sheila, Geller Josie, Lam Pei-Yoong Eating Disorder Diagnoses and Symptom Presentation in Transgender Youth: a Scoping Review. CURRENT PSYCHIATRY REPORTS. 2019;21(11):.

3315. Linville Deanna, Cobb Erin, Lenee-Bluhm Tracy, Lopez-Zeron Gabriela, Gau Jeff, Stice Eric Effectiveness of an eating disorder preventative intervention in primary care medical settings. BEHAVIOUR RESEARCH AND THERAPY. 2015;75():32-39.

3316. Yoon Cynthia, Hazzard Vivienne, Emery Rebecca, Mason Susan, Neumark-Sztainer Dianne Everyday discrimination as a predictor of maladaptive and adaptive eating: Findings from EAT 2018. APPETITE. 2022;170():.

3317. Boughton Roberta, Falenchuk Olesya Vulnerability and Comorbidity factors of female problem gambling. JOURNAL OF GAMBLING STUDIES. 2007;23(3):323-334.

3318. Griffen Trevor, Naumann Eva, Hildebrandt Tom Mirror exposure therapy for body image disturbances and eating disorders: A review. CLINICAL PSYCHOLOGY REVIEW. 2018;65():163-174.

3319. Mann T, NolenHoeksema S, Huang K, Burgard D, Wright A, Hanson K Are two interventions worse than none? Joint primary and secondary prevention of eating disorders in college females. HEALTH PSYCHOLOGY. 1997;16(3):215-225.

3320. Slyepchenko A., Frey B., Lafer B., Nierenberg A., Sachs G., Dias R. Increased illness burden in women with comorbid bipolar and premenstrual dysphoric disorder: data from 1 099 women from STEP-BD study. ACTA PSYCHIATRICA SCANDINAVICA. 2017;136(5):473-482.

3321. GRILO CM, WILFLEY DE, BROWNELL KD, RODIN J TEASING, BODY-IMAGE, AND SELF-ESTEEM IN A CLINICAL-SAMPLE OF OBESE WOMEN. ADDICTIVE BEHAVIORS. 1994;19(4):443-450.

3322. Pollina-Pocallet Merce, Artigues-Barbera Eva, Tort-Nasarre Gloria, Sol Joaquim, Azlor Laura, Foguet-Boreu Quinti, Ortega-Bravo Marta Self-Perception and Self-Acceptance Are Related to Unhealthy Weight Control Behaviors in Catalan Adolescents: A Cross-Sectional Study. INTERNATIONAL JOURNAL OF ENVIRONMENTAL RESEARCH AND PUBLIC HEALTH. 2021;18(9):.

3323. Chong Lin, Chin Yit, Gan Wan, Nasir Mohd Associations between socio-demographic characteristics and pubertal status with disordered eating among primary school children in Selangor, Malaysia. ASIA PACIFIC JOURNAL OF CLINICAL NUTRITION. 2017;26(2):326-333.

3324. Li Zhitao, Wang Lili, Guan Haixia, Han Cheng, Cui Peng, Liu Aihua, Li Yongze Burden of Eating Disorders in China, 1990-2019: An Updated Systematic Analysis of the Global Burden of Disease Study 2019. FRONTIERS IN PSYCHIATRY. 2021;12():.

3325. Forman Evan, Berry Michael, Butryn Meghan, Hagerman Charlotte, Huang Zhuoran, Juarascio Adrienne, LaFata Erica, Ontanon Santiago, Tilford J., Zhang Fengqing Using artificial intelligence to optimize delivery of weight loss treatment: Protocol for an efficacy and cost-effectiveness trial. CONTEMPORARY CLINICAL TRIALS. 2023;124():.

3326. Tata P, Fox J, Cooper J An investigation into the influence of gender and parenting styles on excessive exercise and disordered eating. EUROPEAN EATING DISORDERS REVIEW. 2001;9(3):194-206.

3327. Cifelli Christopher, Zachwieja Jeffrey Examining the nutritional needs of children and adolescents Dairy protein and body composition. AGRO FOOD INDUSTRY HI-TECH. 2012;23(5):52-54.

3328. Mehlenbeck R, Katzow M, Roesler T, Nassau J, High P Food refusal in school age children: Atypical eating disorder vs. early-onset anorexia. JOURNAL OF DEVELOPMENTAL AND BEHAVIORAL PEDIATRICS. 2005;26(6):460-461.

3329. Thompson-Brenner Heather, Boisseau Christina, Satir Dana Adolescent Eating Disorders: Treatment and Response in a Naturalistic Study. JOURNAL OF CLINICAL

PSYCHOLOGY. 2010;66(3):277-301.

3330. Feldhege Johannes, Moessner Markus, Stieler Christiane, Stipelen Jhana, Bauer Stephanie Knowledge and attitudes towards eating disorders among adolescents with Turkish migration background. Potential implications for eating disorder prevention?. PSYCHOTHERAPEUT. 2019;64(1):9-15.

3331. TobiaszAdamczyk B Health beliefs and health behaviours in subpopulation of stomach cancer families and in the control group. CANCER LETTERS. 1997;114(1-2):301-304.

3332. Long Clive, Fitzgerald Kirsty-Anne, Hollin Clive Treatment of chronic anorexia nervosa: a 4-year follow-up of adult patients treated in an acute inpatient setting. CLINICAL PSYCHOLOGY \& PSYCHOTHERAPY. 2012;19(1):1-13.

3333. Leon GR, Keel PK, Klump KL, Fulkerson JA The future of risk factor research in understanding the etiology of eating disorders. PSYCHOPHARMACOLOGY BULLETIN. 1997;33(3):405-411.

3334. Larsen Anna, Lilja Marie, Sturidsson Knut, Blatny Marek, Hrdlicka Michal, Stickley Andrew, Ruchkin Vladislav Bulimia symptoms in Czech youth: prevalence and association with internalizing problems. EATING AND WEIGHT DISORDERS-STUDIES ON ANOREXIA BULIMIA AND OBESITY. 2020;25(6):1543-1552.

3335. Collo Alessandro, Ferro Arianna, Belci Paola, Cerutti Franco, Rabbone Ivana, Ignaccolo Maria, Carletto Giulia, Vallini Camilla, Cadario Francesco, Savastio Silvia, Carrera Deborah, Gruden Gabriella, Siliquini Roberta, Traversi Deborah, Durazzo Marilena Nutritional behavior in Italian and immigrant children. MINERVA PEDIATRICA. 2019;71(6):481-487.

3336. Ebert David, Cuijpers Pim, Munoz Ricardo, Baumeister Harald Prevention of Mental Health Disorders Using Internet- and Mobile-Based Interventions: A Narrative Review and Recommendations for Future Research. FRONTIERS IN PSYCHIATRY. 2017;8():.

3337. Wilksch Simon, O'Shea Anne, Taylor C., Wilfley Denise, Jacobi Corinna, Wade Tracey Online prevention of disordered eating in at-risk young-adult women: a two-country pragmatic randomized controlled trial. PSYCHOLOGICAL MEDICINE. 2018;48(12):2034-2044.

3338. Ellis Jordan, Galloway Amy, Zickgraf Hana, Whited Matthew Picky eating and fruit and vegetable consumption in college students. EATING BEHAVIORS. 2018;30():5-8.

3339. Seneviratne Sumudu, Sachchithananthan Sanathanee, Gamage Pavithra, Peiris Renuka, Wickramasinghe Vithanage, Somasundaram Noel Effectiveness and acceptability of a novel school-based healthy eating program among primary school children in urban Sri Lanka. BMC PUBLIC HEALTH. 2021;21(1):.

3340. Wang Fenglei, Ugai Tomotaka, Haruki Koichiro, Wan Yi, Akimoto Naohiko, Arima Kota, Zhong Rong, Twombly Tyler, Wu Kana, Yin Kanhua, Chan Andrew, Giannakis Marios, Nowak Jonathan, Meyerhardt Jeffrey, Liang Liming, Song Mingyang, Smith-Warner Stephanie, Zhang Xuehong, Giovannucci Edward, Willett Walter, Ogino Shuji Healthy and unhealthy plant-based diets in relation to the incidence of colorectal cancer overall and by molecular subtypes. CLINICAL AND TRANSLATIONAL MEDICINE. 2022;12(8):.

3341. Damiano Stephanie, Paxton Susan, Wertheim Eleanor, McLean Sian, Gregg Karen Dietary Restraint of 5-Year-Old Girls: Associations with Internalization of the Thin Ideal and Maternal, Media, and Peer Influences. INTERNATIONAL JOURNAL OF EATING DISORDERS. 2015;48(8):1166-1169.

3342. Pedlow C., Niemeier Heather Sociotropic cognition and eating disordered attitudes and behavior in young adults. EATING BEHAVIORS. 2013;14(2):95-101.

3343. Wong Emily, Fleishman Aaron, Brem Amanda, Jones Daniel, Wee Christina High-Risk Alcohol Use and Disordered Eating Behavior Before and 1 Year After Sleeve Gastrectomy. OBESITY SURGERY. 2022;32(3):593-598.

3344. Pandit Rahul, Mercer Julian, Overduin Joost, Fleur Susanne, Adan Roger Dietary Factors Affect Food Reward and Motivation to Eat. OBESITY FACTS. 2012;5(2):221-242.

3345. Pallotto Isabella, Sockol Laura, Stutts Lauren General and sport-specific weight pressures as risk factors for body dissatisfaction and disordered eating among female collegiate athletes. BODY IMAGE. 2022;40():340-350.

3346. Pamies Aubalat Lidia, Quiles Marcos Yolanda Avoidance coping style and the risk of developing an eating disorder in adolescents. PSICOTHEMA. 2012;24(2):230-235.

3347. Burckhardt Rowan, Manicavasagar Vijaya, Shaw Frances, Fogarty Andrea, Batterham Philip, Dobinson Katie, Karpin Ilana Preventing mental health symptoms in adolescents using dialectical behaviour therapy skills group: a feasibility study. INTERNATIONAL JOURNAL OF ADOLESCENCE AND YOUTH. 2018;23(1):70-85.

3348. Nielsen GB, Lausch B, Thomsen PH Three cases of severe early-onset eating disorder: Are they cases of anorexia nervosa?. PSYCHOPATHOLOGY. 1997;30(1):49-52.

3349. Cuijpers Pim, Beekman Aartjan, Smit Filip, Deeg Dorly Predicting the onset of major depressive disorder and dysthymia in older adults with subthreshold depression: a community based study. INTERNATIONAL JOURNAL OF GERIATRIC PSYCHIATRY. 2006;21(9):811-818.

3350. Sadeh-Sharvit Shiri, Sacks Madeline, Runfola Cristin, Bulik Cynthia, Lock James Interventions to Empower Adults with Eating Disorders and Their Partners around the Transition to ParenthoodPalabras clave(sic)(sic)(sic). FAMILY PROCESS. 2020;59(4, SI):1407-1422.

3351. Nieto Claudia, Jauregui Alejandra, Contreras-Manzano Alejandra, Arillo-Santillan Edna, Barquera Simon, White Christine, Hammond David, Thrasher James Understanding and use of food labeling systems among Whites and Latinos in the United States and among Mexicans: Results from the International Food Policy Study, 2017. INTERNATIONAL JOURNAL OF BEHAVIORAL NUTRITION AND PHYSICAL ACTIVITY. 2019;16(1):.

3352. Pradeilles Rebecca, Marr Colette, Laar Amos, Holdsworth Michelle, Zotor Francis, Tandoh Akua, Klomegah Senam, Coleman Nathaniel, Bash Kristin, Green Mark, Griffiths Paula How ready are communities to implement actions to improve diets of adolescent girls and women in urban Ghana?. BMC PUBLIC HEALTH. 2019;19():.

3353. Kurz Susanne, Dyck Zoe, Dremmel Daniela, Munsch Simone, Hilbert Anja Variants of Early-Onset Restrictive Eating Disturbances in Middle Childhood. INTERNATIONAL JOURNAL OF EATING DISORDERS. 2016;49(1):102-106.

3354. Zeeni Nadine, Safieddine Hiba, Doumit Rita Eating Disorders in Lebanon: Directions for Public Health Action. COMMUNITY MENTAL HEALTH JOURNAL. 2017;53(1):117-125.

3355. Troisi Alfonso, Di Lorenzo Giorgio, Alcini Stefano, Nanni Roberta, Di Pasquale Claudia, Siracusano Alberto Body dissatisfaction in women with eating disorders: Relationship to

early separation anxiety and insecure attachment. PSYCHOSOMATIC MEDICINE. 2006;68(3):449-453.

3356. Alouani Saoussen, Alouani Sondess, Ben Haouala Amjed, Mhalla Ahmed, Marzougui Ameni, Gaha Lotfi, Amamou Badii Eating disorder and anxiety during Covid19 pandemic: the Maghrebian experience. PSYCHOLOGY HEALTH \& MEDICINE. 2022;():.

3357. Ogata R., Usami M., Iwadare Y., Ushijima H., Tanaka T., Watanabe K. Clinical characteristics of early onset eating disorder in Japanese boys: a retrospective observational study. EUROPEAN CHILD \& ADOLESCENT PSYCHIATRY. 2015;24(1):S137.

3358. Andres-Pepina Susana, Plana Maria, Flamarique Itziar, Romero Sonia, Borras Roger, Julia Laia, Garriz Miguel, Castro-Fornieles Josefina Long-term outcome and psychiatric comorbidity of adolescent-onset anorexia nervosa. CLINICAL CHILD PSYCHOLOGY AND PSYCHIATRY. 2020;25(1):33-44.

3359. Laghi Fiorenzo, Pompili Sara, Bianchi Dora, Lonigro Antonia, Baiocco Roberto Psychological characteristics and eating attitudes in adolescents with drunkorexia behavior: an exploratory study. EATING AND WEIGHT DISORDERS-STUDIES ON ANOREXIA BULIMIA AND OBESITY. 2020;25(3):709-718.

3360. Stice Eric, Rohde Paul, Butryn Meghan, Menke Katharine, Marti C. Randomized Controlled Pilot Trial of a Novel Dissonance-Based Group Treatment for Eating Disorders. BEHAVIOUR RESEARCH AND THERAPY. 2015;65():67-75.

3361. Wiklund Camilla, Kuja-Halkola Ralf, Thornton Laura, Hubel Christopher, Leppa Virpi, Bulik Cynthia Prolonged constipation and diarrhea in childhood and disordered eating in adolescence. JOURNAL OF PSYCHOSOMATIC RESEARCH. 2019;126():.

3362. Winzelberg AJ, Eppstein D, Eldredge KL, Wilfley D, Dasmahapatra R, Dev P, Taylor CB Effectiveness of an Internet-based program for reducing risk factors for eating disorders. JOURNAL OF CONSULTING AND CLINICAL PSYCHOLOGY. 2000;68(2):346-350.

3363. Duarte C., Pinto-Gouveia J., Stubbs R. The prospective associations between bullying experiences, body image shame and disordered eating in a sample of adolescent girls. PERSONALITY AND INDIVIDUAL DIFFERENCES. 2017;116():319-325.

3364. Campbell Iain, Mill Jonathan, Uher Rudolf, Schmidt Ulrike Eating disorders, gene-environment interactions and epigenetics. NEUROSCIENCE AND BIOBEHAVIORAL REVIEWS. 2011;35(3):784-793.

3365. Quintero Gutierrez Adrian, Gonzalez Rosendo Guillermina, Gutierrez Trujillo Jesus, Puga Diaz Ruben, Villanueva Sanchez Javier Prevalence of risk eating behaviors and metabolic syndrome in teenage school students of Morelos state. NUTRICION HOSPITALARIA. 2018;35(4):796-804.

3366. Harrer Mathias, Adam Sophia, Messner Eva-Maria, Baumeister Harald, Cuijpers Pim, Bruffaerts Ronny, Auerbach Randy, Kessler Ronald, Jacobi Corinna, Taylor Craig, Ebert David Prevention of eating disorders at universities: A systematic review and meta-analysis. INTERNATIONAL JOURNAL OF EATING DISORDERS. 2020;53(6):813-833.

3367. Higgins Ashley, Cahn Stacey Detection of anorexia nervosa in primary care. EATING DISORDERS. 2018;26(3):213-228.

3368. Smitka Kvido, Prochazkova Petra, Roubalova Radka, Dvorak Jiri, Papezova Hana, Hill Martin, Pokorny Jaroslav, Kittnar Otomar, Bilej Martin, Tlaskalova-Hogenova Helena Current Aspects of the Role of Autoantibodies Directed Against Appetite-Regulating Hormones and the Gut Microbiome in Eating Disorders. FRONTIERS IN ENDOCRINOLOGY. 2021;12():.

3369. Lochner C, Seedat S, Hemmings SMJ, Kinnear CJ, Corfield VA, Niehaus DJH, Moolman-Smook JC, Stein DJ Dissociative experiences in obsessive-compulsive disorder and trichotillomania: Clinical and genetic findings. COMPREHENSIVE PSYCHIATRY. 2004;45(5):384-391.

3370. Mar Javier, Larranaga Igor, Ibarrondo Oliver, Gonzalez-Pinto Ana, Hayas Carlota, Fullaondo Ane, Izco-Basurko Irantzu, Alonso Jordi, Zorrilla Inaki, Vilagut Gemma, Mateo-Abad Maider, Manuel Esteban, Consortium UPRIGHT Incidence of mental disorders in the general population aged 1-30 years disaggregated by gender and socioeconomic status. SOCIAL PSYCHIATRY AND PSYCHIATRIC EPIDEMIOLOGY. 2023;58(6):961-971.

3371. Pullins Christopher, Seele Pernessa, White Richard, Willis Floyd, Poole Kenneth, Albertie Monica, Chamie Chara, Allen Angela, Kelly Marion, Penheiter Sumedha, Buras Matthew, Brewer LaPrincess Health Behaviors and Preventive Healthcare Utilization Among African-American Attendees at a Faith-Based Public Health Conference: Healthy

Churches 2020. JOURNAL OF RELIGION \& HEALTH. 2018;57(6):2538-2551.

3372. Cusack Kaleb, Petrie Trent, Moore E. Self-compassion, body satisfaction, and disordered eating symptoms in male collegiate athletes: A longitudinal analysis. BODY IMAGE. 2022;43():134-142.

3373. Lv Jun, Liu Qingmin, Ren Yanjun, Gong Ting, Wang Shengfeng, Li Liming, CIH Community Socio-demographic association of multiple modifiable lifestyle risk factors and their clustering in a representative urban population of adults: a cross-sectional study in Hangzhou, China. INTERNATIONAL JOURNAL OF BEHAVIORAL NUTRITION AND PHYSICAL ACTIVITY. 2011;8():.

3374. CARTER FA, BULIK CM EXPOSURE TREATMENTS FOR BULIMIA-NERVOSA - PROCEDURE, EFFICACY, AND MECHANISMS. ADVANCES IN BEHAVIOUR RESEARCH AND THERAPY. 1994;16(2):77-129.

3375. Sadeghi Mahya, Vahid Farhad, Rahmani Diyako, Akbari Mohammad, Davoodi Sayed The Association between Dietary Patterns and Breast Cancer Pathobiological Factors Progesterone Receptor (PR) and Estrogen Receptors (ER): New Findings from Iranian Case-Control Study. NUTRITION AND CANCER-AN INTERNATIONAL JOURNAL. 2019;71(8):1290-1298.

3376. Park Haeryun, Tserendejid Zuunnast, Song Kyung-Hee, Lee Jounghee, Lee Youngmi Dietary Patterns and the Association with Dietary Quality Among Mongolian Immigrants in South Korea. JOURNAL OF IMMIGRANT AND MINORITY HEALTH. 2015;17(2):422-431.

3377. Theander S Anorexia nervosa with an early onset: Selection, gender, outcome, and results of a long-term follow-up study. JOURNAL OF YOUTH AND ADOLESCENCE. 1996;25(4):419-429.

3378. STACY AW, BENTLER PM, FLAY BR ATTITUDES AND HEALTH BEHAVIOR IN DIVERSE POPULATIONS - DRUNK DRIVING, ALCOHOL-USE, BINGE-EATING, MARIJUANA USE, AND CIGARETTE USE. HEALTH PSYCHOLOGY. 1994;13(1):73-85.

3379. Thiel Nicola, Tuschen-Caffier Brunna, Herbst Nirmal, Kuelz Anne, Nissen Christoph, Hertenstein Elisabeth, Gross Ellen, Voderholzer Ulrich The prediction of treatment outcomes by early maladaptive schemas and schema modes in obsessive-compulsive

disorder. BMC PSYCHIATRY. 2014;14():.

3380. Lilo Emily, Munoz Marlene, Cruz Theresa Perceptions of Healthy Eating Among Hispanic Parent-Child Dyads. HEALTH PROMOTION PRACTICE. 2019;20(2):231-238.

3381. Cottee-Lane D, Pistrang N, Bryant-Waugh R Childhood onset anorexia nervosa: The experience of parents. EUROPEAN EATING DISORDERS REVIEW. 2004;12(3):169-177.

3382. Krug Isabel, Fuller-Tyszkiewicz Matthew, Anderluh Marija, Bellodi Laura, Bagnoli Silvia, Collier David, Fernandez-Aranda Fernando, Karwautz Andreas, Mitchell Sarah, Nacmias Benedetta, Ricca Valdo, Sorbi Sandro, Tchanuria Kate, Wagner Gudrun, Treasure Janet, Micali Nadia A new social-family model for eating disorders: A European multicentre project using a case-control design. APPETITE. 2015;95():544-553.

3383. Stice E, Shaw H Eating disorder prevention programs: A meta-analytic review. PSYCHOLOGICAL BULLETIN. 2004;130(2):206-227.

3384. Lutter Chessa, Grummer-Strawn Laurence, Rogers Lisa Complementary feeding of infants and young children 6 to 23 months of age. NUTRITION REVIEWS. 2021;79(8):825-846.

3385. Arvind Teli, Karthika Anu, Motwani Yogesh, Prakash Krishna, Nair Shobha, Chaudhari Aditi, Mazumdar Kaustubh PSYCHIATRIC DISORDERS PRESENTING WITH GASTROINTESTINAL SYMPTOMS. INDIAN JOURNAL OF PSYCHIATRY. 2019;61(9, 3):S452.

3386. Hedman Linnea, Backman Helena, Stridsman Caroline, Lundback Magnus, Andersson Martin, Ronmark Eva Predictors of electronic cigarette use among Swedish teenagers: a population-based cohort study. BMJ OPEN. 2020;10(12):.

3387. Arthur-Cameselle Jessyca, Sossin Kayla, Quatromoni Paula A qualitative analysis of factors related to eating disorder onset in female collegiate athletes and non-athletes. EATING DISORDERS. 2017;25(3):199-215.

3388. Baile Jose, Gonzalez-Calderon Maria, Palomo Ruth, Rabito-Alcon Maria Psychological intervention of obesity: development and perspectives. REVISTA CLINICA CONTEMPORANEA. 2020;11(1):.

3389. Stice Eric, Becker Carolyn, Yokum Sonja Eating disorder prevention: Current evidence-base and future directions. INTERNATIONAL JOURNAL OF EATING DISORDERS. 2013;46(5, SI):478-485.

3390. Anderson Christina, Holody Kyle, Flynn Mark, Hussa-Farrell Robyn An exploratory evaluation of the feasibility, acceptability, and efficacy of the mental fitness disordered eating program in schools. EATING DISORDERS. 2017;25(3):230-245.

3391. Fairweather-Schmidt A., Wade Tracey Weight-related peer-teasing moderates genetic and environmental risk and disordered eating: twin study. BRITISH JOURNAL OF PSYCHIATRY. 2017;210(5):350+.

3392. Mogeni Brenda, Ouma Luke Dietary patterns, behaviours, and their associated factors among university students in coastal Kenya. COGENT FOOD \& AGRICULTURE. 2022;8(1):.

3393. Pasdar Yahya, Hamzeh Behrooz, Moradi Shima, Mohammadi Ehsan, Cheshmeh Sahar, Darbandi Mitra, Faramani Roya, Najafi Farid Healthy eating index 2015 and major dietary patterns in relation to incident hypertension; a prospective cohort study. BMC PUBLIC HEALTH. 2022;22(1):.

3394. Wade Tracey, Wilksch Simon, Lee Christina A Longitudinal Investigation of the Impact of Disordered Eating on Young Women's Quality of Life. HEALTH PSYCHOLOGY. 2012;31(3):352-359.

3395. Machado Barbara, Goncalves Sonia, Martins Carla, Hoek Hans, Machado Paulo Risk Factors and Antecedent Life Events in the Development of Anorexia Nervosa: A Portuguese Case-Control Study. EUROPEAN EATING DISORDERS REVIEW. 2014;22(4):243-251.

3396. Amodeo Simona, Mirarchi Luigi, Seidita Aurelio, Citarrella Roberto, Licata Anna, Soresi Maurizio, Iovanna Juan, Giannitrapani Lydia EVOO's Effects on Incretin Production: Is There a Rationale for a Combination in T2DM Therapy?. INTERNATIONAL JOURNAL OF MOLECULAR SCIENCES. 2022;23(17):.

3397. Vermeulen-Smit E., Ten Have M., Van Laar M., De Graaf R. Clustering of health risk behaviours and the relationship with mental disorders. JOURNAL OF AFFECTIVE DISORDERS. 2015;171():111-119.

3398. Barakat Sarah, McLean Sian, Bryant Emma, Le Anvi, Marks Peta, Touyz Stephen, Maguire Sarah, Res Natl Risk factors for eating disorders: findings from a rapid review. JOURNAL OF EATING DISORDERS. 2023;11(1):.

3399. Kasemsuk Chayut, Oyama Genko, Hattori Nobutaka Management of impulse control disorders with deep brain stimulation: A double-edged sword. JOURNAL OF THE NEUROLOGICAL SCIENCES. 2017;374(SI):63-68.

3400. Lacatusu Cristina-Mihaela, Grigorescu Elena-Daniela, Floria Mariana, Onofriescu Alina, Mihai Bogdan-Mircea The Mediterranean Diet: From an Environment-Driven Food Culture to an Emerging Medical Prescription. INTERNATIONAL JOURNAL OF ENVIRONMENTAL RESEARCH AND PUBLIC HEALTH. 2019;16(6):.

3401. Boddy Lynne, Knowles Zoe, Davies Ian, Warburton Genevieve, Mackintosh Kelly, Houghton Laura, Fairclough Stuart Using formative research to develop the healthy eating component of the CHANGE! school-based curriculum intervention. BMC PUBLIC HEALTH. 2012;12():.

3402. Guardia D., Metral M., Pigeyre M., Bauwens I., Cottencin O., Luyat M. Body distortions after massive weight loss: lack of updating of the body schema hypothesis. EATING AND WEIGHT DISORDERS-STUDIES ON ANOREXIA BULIMIA AND OBESITY. 2013;18(3):333-336.

3403. Atkinson Melissa, Wade Tracey Impact of Metacognitive Acceptance on Body Dissatisfaction and Negative Affect: Engagement and Efficacy. JOURNAL OF CONSULTING AND CLINICAL PSYCHOLOGY. 2012;80(3):416-425.

3404. Elmberg Sjoholm M., Eriksson G., Bii A., Asungu J., Koch L., Guidetti S. Living with consequences of stroke and risk factors for unhealthy diet- experiences among stroke survivors and caregivers in Nairobi, Kenya. BMC PUBLIC HEALTH. 2021;21(1):.

3405. Lee Megan, Madsen Julian, Williams Susan, Browne Matthew, Burke Karena Differential Effects of Intuitive and Disordered Eating on Physical and Psychological Outcomes for Women with Young Children. MATERNAL AND CHILD HEALTH JOURNAL. 2022;26(2):407-414.

3406. Karim Mohammad, Majumder Abdullah, Islam Khandaker, Alam Muhammad, Paul Makhan, Islam Mohammad, Chowdhury Kamrun, Islam Sheikh Risk factors and in-hospital

outcome of acute ST segment elevation myocardial infarction in young Bangladeshi adults. BMC CARDIOVASCULAR DISORDERS. 2015;15():.

3407. Hulley AJ, Hill AJ Eating disorders and health in elite women distance runners. INTERNATIONAL JOURNAL OF EATING DISORDERS. 2001;30(3):312-317.

3408. Melin A., Tornberg A., Skouby S., Moller S., Faber J., Sundgot-Borgen J., Sjodin A. Low-energy density and high fiber intake are dietary concerns in female endurance athletes. SCANDINAVIAN JOURNAL OF MEDICINE \& SCIENCE IN SPORTS. 2016;26(9):1060-1071.

3409. Godart N Clinical signs of bipolar vulnerability in adolescents. ENCEPHALE-REVUE DE PSYCHIATRIE CLINIQUE BIOLOGIQUE ET THERAPEUTIQUE. 2005;31(2, S):S3+.

3410. Taylor CB, Bryson SW, Altman TM, Abascal L, Celio A, Cunning D, Killen JD, Shisslak CM, Crago M, Ranger-Moore J, Cook P, Ruble A, Olmsted ME, Kraemer HC, Smolak L, Investigators McKnight Risk factors for the onset of eating disorders in adolescent girls: Results of the McKnight longitudinal risk factor study. AMERICAN JOURNAL OF PSYCHIATRY. 2003;160(2):248-254.

3411. Pavlova Barbara, Uher Rudolf, Papezova Hana It would not have happened to me at home qualitative exploration of sojourns abroad and eating disorders in young Czech women. EUROPEAN EATING DISORDERS REVIEW. 2008;16(3):207-214.

3412. Keshishian Ani, Tabri Nassim, Becker Kendra, Franko Debra, Herzog David, Thomas Jennifer, Eddy Kamryn Eating disorder recovery is associated with absence of major depressive disorder and substance use disorders at 22-year longitudinal follow-up. COMPREHENSIVE PSYCHIATRY. 2019;90():49-51.

3413. Skubisz Christine, Seeney Angelina, Pacanowski Carly Testing Theory-Based Expressive Writing Interventions to Reduce Disordered Eating Behaviors and Cognitions. AMERICAN JOURNAL OF LIFESTYLE MEDICINE. 2022;():.

3414. Tirlea Loredana, Truby Helen, Haines Terry Investigation of the effectiveness of the ``Girls on the Go!{''} program for building self-esteem in young women: trial protocol. SPRINGERPLUS. 2013;2():.

3415. DiGioacchino DeBate Rita, Plichta Stacey, Tedesco Lisa, Kerschbaum Wendy Integration of oral health care and mental health services: Dental hygienists' readiness and capacity for secondary prevention of eating disorders. JOURNAL OF BEHAVIORAL HEALTH SERVICES \& RESEARCH. 2006;33(1):113-125.

3416. Maele Karolien, De Geyter Charlotte, Vandenplas Yvan, Gies Inge, Devlieger Roland Eating Habits of Children Born after Maternal Bariatric Surgery. NUTRIENTS. 2020;12(9):.

3417. Hummel Alexandra, Smith April Ask and You Shall Receive: Desire and Receipt of Feedback via Facebook Predicts Disordered Eating Concerns. INTERNATIONAL JOURNAL OF EATING DISORDERS. 2015;48(4):436-442.

3418. Mazza Elisa, Ferro Y., Pujia R., Mare R., Maurotti S., Montalcini T., Pujia A. Mediterranean Diet In Healthy Aging. JOURNAL OF NUTRITION HEALTH \& AGING. 2021;25(9):1076-1083.

3419. Corepal Rekesh, Tully Mark, Kee Frank, Miller Sarah, Hunter Ruth Behavioural incentive interventions for health behaviour change in young people (5-18 years old): A systematic review and meta-analysis. PREVENTIVE MEDICINE. 2018;110():55-66.

3420. Wong Melody, Hay Phillipa Exploring associations between age of onset and quality of life of people with eating disorder behaviours and weight/shape overvaluation: a general population study. AUSTRALASIAN PSYCHIATRY. 2020;28(6):660-663.

3421. Thamotharan Sneha, Lange Krista, Ramos Ashley, Fields Sherecce Examining weight concern and delay discounting in adolescent females. EATING BEHAVIORS. 2016;21():228-231.

3422. Hazzard Vivienne, Mason Tyler, Smith Kathryn, Schaefer Lauren, Anderson Lisa, Dodd Dorian, Crosby Ross, Wonderlich Stephen Identifying transdiagnostically relevant risk and protective factors for internalizing psychopathology: An umbrella review of longitudinal meta-analyses. JOURNAL OF PSYCHIATRIC RESEARCH. 2023;158():231-244.

3423. Viborg Njordur, Wangby-Lundh Margit, Lundh Lars-Gunnar Reciprocal prospective associations between disordered eating and other psychological problems in a community sample of Swedish adolescent girls. EATING BEHAVIORS. 2014;15(1):159-163.

3424. Kang Harmeet, Rhodes Christopher, Rivers Emerald, Thornton Clifton, Rodney Tamar Prevalence of Mental Health Disorders Among Undergraduate University Students in the United States A Review. JOURNAL OF PSYCHOSOCIAL NURSING AND MENTAL HEALTH SERVICES. 2021;59(2):17+.

3425. Swenne Ingemar Influence of premorbid BMI on clinical characteristics at presentation of adolescent girls with eating disorders. BMC PSYCHIATRY. 2016;16():.

3426. Pimenta Adriano, Sanchez-Villegas Almudena, Bes-Rastrollo Maira, Lopez Celeste, Angel Martinez-Gonzalez Miguel Relationship between body image disturbance and incidence of depression: the SUN prospective cohort. BMC PUBLIC HEALTH. 2009;9():.

3427. Brown JB, Winzelberg AJ, Abascal LB, Taylor CB An evaluation of an Internet-delivered eating disorder prevention program for adolescents and their parents. JOURNAL OF ADOLESCENT HEALTH. 2004;35(4):290-296.

3428. FOMBONNE E ANOREXIA-NERVOSA - NO EVIDENCE OF AN INCREASE. BRITISH JOURNAL OF PSYCHIATRY. 1995;166():462-471.

3429. Stein KF, Corte C Reconceptualizing causative factors and intervention strategies in the eating disorders: A shift from body image to self-concept impairments. ARCHIVES OF PSYCHIATRIC NURSING. 2003;17(2):57-66.

3430. Chenkov Yavor, Hristova Darina A BRIEF LITERATURE OVERVIEW ON ORTHOREXIA NERVOSA - ONE NEW REPRESENTATIVE IN CLUSTER OF EATING DISORDERS. JOURNAL OF IMAB. 2021;27(1):3568-3571.

3431. Rousselet M., Guerineau B., Paruit M., Guinot M., Lise S., Destrube B., Ruffio-Thery S., Dominguez N., Brisseau-Gimenez S., Dubois V., Mora C., Trolonge S., Lambert S., Grall-Bronnec M., Pretagut S. Disordered eating in French high-level athletes: association with type of sport, doping behavior, and psychological features. EATING AND WEIGHT DISORDERS-STUDIES ON ANOREXIA BULIMIA AND OBESITY. 2017;22(1):61-68.

3432. Dang Amit, Garg Gaurav, Rataboli Padmanabh Zolpidem Induced Nocturnal Sleep-Related Eating Disorder (NSRED) in a Male Patient. INTERNATIONAL JOURNAL OF EATING DISORDERS. 2009;42(4):385-386.

3433. Griffiths RA, HadziPavlovic D, ChannonLittle L The short-term follow-up effects of hypnobehavioural and cognitive behavioural treatment for bulimia nervosa. EUROPEAN EATING DISORDERS REVIEW. 1996;4(1):12-31.

3434. Flaudias Valentin, Zerhouni Oulmann, Pereira Bruno, Cherpitel Cheryl, Boudesseul Jordane, Chazeron Ingrid, Romo Lucia, Guillaume Sebastien, Samalin Ludovic, Cabe Julien, Begue Laurent, Gerbaud Laurent, Rolland Benjamin, Llorca Pierre-Michel, Naassila Mickael, Brousse Georges The Early Impact of the COVID-19 Lockdown on Stress and Addictive Behaviors in an Alcohol-Consuming Student Population in France. FRONTIERS IN PSYCHIATRY. 2021;12():.

3435. Bisaga K, Whitaker A, Davies M, Chuang S, Feldman J, Walsh BT Eating disorder and depressive symptoms in urban high school girls from different ethnic backgrounds. JOURNAL OF DEVELOPMENTAL AND BEHAVIORAL PEDIATRICS. 2005;26(4):257-266.

3436. Mansson Mattias, Holte Jan, Landin-Wilhelmsen Kerstin, Dahlgren Eva, Johansson Anette, Landen Mikael Women with polycystic ovary syndrome are often depressed or anxious - A case control study. PSYCHONEUROENDOCRINOLOGY. 2008;33(8):1132-1138.

3437. Mitchell JE, Peterson CB, Myers T, Wonderlich S Combining pharmacotherapy and psychotherapy in the treatment of patients with eating disorders. PSYCHIATRIC CLINICS OF NORTH AMERICA. 2001;24(2):315+.

3438. Meierer Klara, Hudon Alexandre, Sznajder Marc, Leduc Marie-France, Taddeo Danielle, Jamoulle Olivier, Frappier Jean-Yves, Stheneur Chantal Anorexia nervosa in adolescents: evolution of weight history and impact of excess premorbid weight. EUROPEAN JOURNAL OF PEDIATRICS. 2019;178(2):213-219.

3439. Sancanuto Cintia, Jimenez-Rodriguez Diana, Javier Tebar Francisco, Jose Hernandez-Morante Juan Translation and validation of the Diabetes Eating Problem Survey to screen eating disorders in patients with type-1 diabetes mellitus. MEDICINA CLINICA. 2017;148(12):548-554.

3440. Fichter Manfred, Quadflieg Norbert, Nisslmueller Kerstin, Lindner Susanne, Osen Bernhard, Huber Thomas, Wuensch-Leiteritz Wally Does internet-based prevention reduce the risk of relapse for anorexia nervosa?. BEHAVIOUR RESEARCH AND THERAPY. 2012;50(3):180-190.

3441. Henderson Katherine, Obeid Nicole, Buchholz Annick, Schubert Nicholas, Flament Martine, Thai Helen, Goldfield Gary Coping in adolescents: A mediator between stress and disordered eating{*},{*}{*}. EATING BEHAVIORS. 2022;47():.

3442. Milano Walter, Milano Luca, Capasso Anna Eating Disorders in Athletes: From Risk Management to Therapy. ENDOCRINE METABOLIC \& IMMUNE DISORDERS-DRUG TARGETS. 2020;20(1):2-14.

3443. Torstveit Monica, Aagedal-Mortensen Kjersti, Stea Tonje More than Half of High School Students Report Disordered Eating: A Cross Sectional Study among Norwegian Boys and Girls. PLOS ONE. 2015;10(3):.

3444. Zhang Aimee, Fisher Aaron, Bailey Jakki, Kass Andrea, Wilfley Denise, Taylor C. The Self-Rating of the Effects of Alcohol Questionnaire Predicts Heavy Episodic Drinking In a High-Risk Eating Disorder Population. INTERNATIONAL JOURNAL OF EATING DISORDERS. 2015;48(3):333-336.

3445. Dalsgaard Soren, Thorsteinsson Erla, Trabjerg Betina, Schullehner Jorg, Plana-Ripoll Oleguer, Brikell Isabell, Wimberley Theresa, Thygesen Malene, Madsen Kathrine, Timmerman Allan, Schendel Diana, McGrath John, Mortensen Preben, Pedersen Carsten Incidence Rates and Cumulative Incidences of the Full Spectrum of Diagnosed Mental Disorders in Childhood and Adolescence. JAMA PSYCHIATRY. 2020;77(2):155-164.

3446. Huebel Christopher, Gaspar Helena, Coleman Jonathan, Hanscombe Ken, Purves Kirstin, Prokopenko Inga, Graff Mariaelisa, Ngwa Julius, Workalemahu Tsegaselassie, O'Reilly Paul, Bulik Cynthia, Breen Gerome, Consortium Psychiat, Insulin-related Meta-Anal, Consort German, Consortium Int Genetic correlations of psychiatric traits with body composition and glycemic traits are sex- and age-dependent. NATURE COMMUNICATIONS. 2019;10():.

3447. Marques Ana, Durif Franck, Fernagut Pierre-Olivier Impulse control disorders in Parkinson's disease. JOURNAL OF NEURAL TRANSMISSION. 2018;125(8, SI):1299-1312.

3448. Griffiths Scott, Mond Jonathan, Murray Stuart, Thornton Chris, Touyz Stephen Stigma resistance in eating disorders. SOCIAL PSYCHIATRY AND PSYCHIATRIC EPIDEMIOLOGY. 2015;50(2):279-287.

3449. Warne Naomi, Heron Jon, Mars Becky, Solmi Francesca, Biddle Lucy, Gunnell David, Hammerton Gemma, Moran Paul, Munafo Marcus, Penton-Voak Ian, Skinner Andy, Stewart Anne, Bould Helen Emotional dysregulation in childhood and disordered eating and self-harm in adolescence: prospective associations and mediating pathways. JOURNAL OF CHILD PSYCHOLOGY AND PSYCHIATRY. 2023;64(5):797-806.

3450. Sepulveda A., Carrobles J., Gandarillas A., Poveda J., Pastor V. Prevention program for disturbed eating and body dissatisfaction in a Spanish university population: A pilot study. BODY IMAGE. 2007;4(3):317-328.

3451. Stepankova Tereza, Papezova Hana Comorbid adult ADHD among ED patients. CESKOSLOVENSKA PSYCHOLOGIE. 2019;63(4):413-429.

3452. Kilpela Lisa, Blomquist Kerstin, Verzijl Christina, Wilfred Salome, Beyl Robbie, Becker Carolyn The body project 4 all: A pilot randomized controlled trial of a mixed-gender dissonance-based body image program. INTERNATIONAL JOURNAL OF EATING DISORDERS. 2016;49(6):591-602.

3453. Lloyd E., Frampton I., Verplanken B., Haase A. How extreme dieting becomes compulsive: A novel hypothesis for the role of anxiety in the development and maintenance of anorexia nervosa. MEDICAL HYPOTHESES. 2017;108():144-150.

3454. Solikhah, Lestari Asri Processed meat consumption increases risk of type 2 diabetes mellitus in adults aged 40 years and older. UNIVERSA MEDICINA. 2022;41(1):18-28.

3455. Zhou Zhiqing, Liew Jeffrey, Luo Wen Acculturation and Disordered Eating among Asian American College Students: The Role of Objectification through a Sociocultural Lens. INTERNATIONAL JOURNAL OF ENVIRONMENTAL RESEARCH AND PUBLIC HEALTH. 2022;19(21):.

3456. Mostafavi Farideh, Moradi Ghobad, Azadi Namamali, Esmaeilnasab Nader, Chamary Maryam The Association of Unhealthy Diet with Socioeconomic Inequality in Children: A Study in Kurdistan, West of Iran. HEALTH SCOPE. 2019;8(4):.

3457. O'Brien Katie, Whelan Denis, Sandler Dale, Hall Janet, Weinberg Clarice Predictors and long-term health outcomes of eating disorders. PLOS ONE. 2017;12(7):.

3458. Stewart T., Plasencia M., Han H., Jackson H., Becker C. Moderators and predictors of response to eating disorder risk factor reduction programs in collegiate female athletes. PSYCHOLOGY OF SPORT AND EXERCISE. 2014;15(6, SI):713-720.

3459. Yaylaci Ferhat, Kucuk Onder, Erkuran Handan L-Carnitine use as a trigger for the onset of Kleine-Levin syndrome: A case presentation. KLINIK PSIKIYATRI DERGISI-TURKISH JOURNAL OF CLINICAL PSYCHIATRY. 2022;25(2):223-228.

3460. Bresin Konrad, Mekawi Yara Unpacking the Construct of Dysregulated Behaviors Using Variable-Centered and Person-Centered Analytic Approaches. SUBSTANCE USE \& MISUSE. 2022;57(4):603-612.

3461. Spangler Diane, Allen Mark An fMRI investigation of emotional processing of body shape in bulimia nervosa. INTERNATIONAL JOURNAL OF EATING DISORDERS. 2012;45(1):17-25.

3462. Mendes Ana, Canavarro Maria, Ferreira Claudia How psychological inflexibility mediates the association between general feelings of shame with body image-related shame and eating psychopathology severity?. APPETITE. 2021;163():.

3463. Simsek Hatice, Doganay Sinem, Budak Refik, Ucku Reyhan Relationship of socioeconomic status with health behaviors and self-perceived health in the elderly: A community-based study, Turkey. GERIATRICS \& GERONTOLOGY INTERNATIONAL. 2014;14(4):960-968.

3464. Gketsios Ioannis, Tsiampalis Thomas, Kanellopoulou Aikaterini, Vassilakou Tonia, Notara Venetia, Antonogeorgos George, Rojas-Gil Andrea, Kornilaki Ekaterina, Lagiou Areti, Panagiotakos Demosthenes, Kosti Rena The Synergetic Effect of Soft Drinks and Sweet/Salty Snacks Consumption and the Moderating Role of Obesity on Preadolescents' Emotions and Behavior: A School-Based Epidemiological Study. LIFE-BASEL. 2023;13(3):.

3465. Liou Yiing, Hsu Ya-Wen, Ho Jow-Fei, Lin Che-Hung, Hsu Wen-Yen, Liou Tsan-Hon Prevalence and correlates of self-induced vomiting as weight-control strategy among adolescents in Taiwan. JOURNAL OF CLINICAL NURSING. 2012;21(1-2):11-20.

3466. Katz Bracha Gender and Disordered Eating of Adolescents in Israel. ISRAEL JOURNAL OF PSYCHIATRY AND RELATED SCIENCES. 2014;51(2):137-144.

3467. Steegers-Theunissen Regine, Wiegel Rosalieke, Jansen Pauline, Laven Joop, Sinclair Kevin Polycystic Ovary Syndrome: A Brain Disorder Characterized by Eating Problems Originating during Puberty and Adolescence. INTERNATIONAL JOURNAL OF MOLECULAR SCIENCES. 2020;21(21):.

3468. Simone Melissa, Hooper Laura, Eisenberg Marla, Neumark-Sztainer Dianne Unhealthy weight control behaviors and substance use among adolescent girls: The harms of weight stigma. SOCIAL SCIENCE \& MEDICINE. 2019;233():64-70.

3469. Hirsch Katherine, Blomquist Kerstin Community-Based Prevention Programs for Disordered Eating and Obesity: Updates and Current Limitations. CURRENT OBESITY REPORTS. 2020;9(2):81-97.

3470. Jackson Dylan, Vaughn Michael Obesogenic food consumption among young children: the role of maltreatment. PUBLIC HEALTH NUTRITION. 2019;22(10):1840-1849.

3471. Yu Zhijie, Parker Louise, Dummer Trevor Depressive symptoms, diet quality, physical activity, and body composition among populations in Nova Scotia, Canada: Report from the Atlantic Partnership for Tomorrow's Health. PREVENTIVE MEDICINE. 2014;61():106-113.

3472. Jalali-Farahani Sara, Chin Yit, Nasir Mohd, Amiri Parisa Disordered Eating and its Association with Overweight and Health-Related Quality of Life Among Adolescents in Selected High Schools of Tehran. CHILD PSYCHIATRY \& HUMAN DEVELOPMENT. 2015;46(3):485-492.

3473. Johnson Paul, Kenny Paul Dopamine D2 receptors in addiction-like reward dysfunction and compulsive eating in obese rats. NATURE NEUROSCIENCE. 2010;13(5):635-641.

3474. Maine M, Goldberg MH The role of third molar surgery in the exacerbation of eating disorders. JOURNAL OF ORAL AND MAXILLOFACIAL SURGERY. 2001;59(11):1297-1300.

3475. Evans Elizabeth, Tovee Martin, Boothroyd Lynda, Drewett Robert Body dissatisfaction and disordered eating attitudes in 7- to 11-year-old girls: Testing a sociocultural model. BODY IMAGE. 2013;10(1):8-15.

3476. Johnson F, Wardle J Dietary restraint, body dissatisfaction, and psychological distress: A prospective analysis. JOURNAL OF ABNORMAL PSYCHOLOGY. 2005;114(1):119-125.

3477. Goode Rachel, Kalarchian Melissa, Conroy Molly, Craighead Linda, Sereika Susan, Mattos Meghan, Zheng Yaguang, Mancino Juliet, Burke Lora Feasibility of an Appetite Awareness Intervention to Reduce Cardiovascular Disease Risk Factors and Binge Eating in African-American Women with Obesity. CIRCULATION. 2017;135(1):.

3478. Gomes Rui, Goncalves Sonia, Costa Joana Exercise, eating disordered behaviors and psychological well-being: a study with Portuguese adolescents. REVISTA LATINOAMERICANA DE PSICOLOGIA. 2015;47(1):66-74.

3479. Kinasz Kathryn, Accurso Erin, Kass Andrea, Le Grange Daniel Does Sex Matter in the Clinical Presentation of Eating Disorders in Youth?. JOURNAL OF ADOLESCENT HEALTH. 2016;58(4):410-416.

3480. Mohammadi Shooka, Su Tin, Papadaki Angeliki, Jalaludin Muhammad, Dahlui Maznah, Mohamed Mohd, Jago Russell, Toumpakari Zoi, Johnson Laura, Majid Hazreen Perceptions of eating practices and physical activity among Malaysian adolescents in secondary schools: a qualitative study with multi-stakeholders. PUBLIC HEALTH NUTRITION. 2021;24(8):2273-2285.

3481. Booij Linda, Casey Kevin, Antunes Juliana, Szyf Moshe, Joober Ridha, Israel Mimi, Steiger Howard DNA methylation in individuals with anorexia nervosa and in matched normal-eater controls: A genome-wide study. INTERNATIONAL JOURNAL OF EATING DISORDERS. 2015;48(7):874-882.

3482. Sun Shaojing, He Jinbo, Fan Xitao, Chen Yibei, Lu Xueke Chinese media coverage of eating disorders: Disorder representations and patient profiles. INTERNATIONAL JOURNAL OF EATING DISORDERS. 2020;53(1):113-122.

3483. Munn-Chernoff Melissa, Grant Julia, Bucholz Kathleen, Agrawal Arpana, Lynskey Michael, Madden Pamela, Heath Andrew, Duncan Alexis Bulimic Behaviors and Early Substance Use: Findings from a Cotwin-Control Study. ALCOHOLISM-CLINICAL AND EXPERIMENTAL RESEARCH. 2015;39(9):1740-1748.

3484. Mead E., Gittelsohn J., De Roose E., Sharma S. Important psychosocial factors to target in nutrition interventions to improve diet in Inuvialuit communities in the Canadian Arctic.

JOURNAL OF HUMAN NUTRITION AND DIETETICS. 2010;23(1):92-99.

3485. Sobreira Neto Manoel, Pena Pereira Marcio, Tavares Sobreira Emmanuelle, Nishiara Chagas Marcos, Rodrigues Guilherme, Franca Fernandes Regina, Tumas Vitor, Schenck Carlos, Eckeli Alan Sleep-Related Eating Disorder in Two Patients with Early-Onset Parkinson's Disease. EUROPEAN NEUROLOGY. 2011;66(2):106-109.

3486. Calado Maria, Lameiras Maria, Sepulveda Ana, Rodriguez Yolanda, Carrera Maria The Mass Media Exposure and Disordered Eating Behaviours in Spanish Secondary Students. EUROPEAN EATING DISORDERS REVIEW. 2010;18(5):417-427.

3487. Jaaskelainen Anne, Nevanpera Nina, Remes Jouko, Rahkonen Fanni, Jarvelin Marjo-Riitta, Laitinen Jaana Stress-related eating, obesity and associated behavioural traits in adolescents: a prospective population-based cohort study. BMC PUBLIC HEALTH. 2014;14():.

3488. Carter Jacqueline, Mercer-Lynn Kimberley, Norwood Sarah, Bewell-Weiss Carmen, Crosby Ross, Woodside D., Olmsted Marion A prospective study of predictors of relapse in anorexia nervosa: Implications for relapse prevention. PSYCHIATRY RESEARCH. 2012;200(2-3):518-523.

3489. Hutchison Erica, Haden Sara, Saunders Benjamin, Cain Nicole, Grundleger Alexandra Disordered Eating in Men and Women: Internalization of Sociocultural Body Image Norms and Emotion Dysregulation. AMERICAN JOURNAL OF HEALTH EDUCATION. 2020;51(3):151-160.

3490. Oliveira Claire, Tanner Bryan, Colton Patricia, Kurdyak Paul Understanding the scope of preventable acute care spending among patients with eating disorders. INTERNATIONAL JOURNAL OF EATING DISORDERS. 2023;56(6):1156-1187.

3491. Beneke Johannes, Koerner Michael, Zwaan Martina Erythema ab igne in a Patient with Bulimia Nervosa. PSYCHOTHERAPIE PSYCHOSOMATIK MEDIZINISCHE PSYCHOLOGIE. 2014;64(5):197-199.

3492. Beintner Ina, Jacobi Corinna Internet-based aftercare for women with bulimia nervosa following inpatient treatment: The role of adherence. INTERNET INTERVENTIONS-THE APPLICATION OF INFORMATION TECHNOLOGY IN MENTAL AND BEHAVIOURAL

HEALTH. 2019;15():67-75.

3493. Graaf R, Dorsselaer S, Have M, Schoemaker C, Vollebergh WAM Seasonal variations in mental disorders in the general population of a country with a maritime climate: Findings from the Netherlands Mental Health Survey and incidence study. AMERICAN JOURNAL OF EPIDEMIOLOGY. 2005;162(7):654-661.

3494. Gosling Rachael, Stanistreet Debbi, Swami Viren `If Michael Owen drinks it, why can't I?' - 9 and 10 year olds' perceptions of physical activity and healthy eating. HEALTH EDUCATION JOURNAL. 2008;67(3):167-181.

3495. Coombs Elizabeth, Brosnan Mark, Bryant-Waugh Rachel, Skevington Suzanne An investigation into the relationship between eating disorder psychopathology and autistic symptomatology in a non-clinical sample. BRITISH JOURNAL OF CLINICAL PSYCHOLOGY. 2011;50(3):326-338.

3496. Newton M, Boblin S, Brown B, Ciliska D `An engagement-distancing flux': Bringing a voice to experiences with romantic relationships for women with anorexia nervosa. EUROPEAN EATING DISORDERS REVIEW. 2005;13(5):317-329.

3497. Thangaraju Siva, Karpagalakshmi R., Arumuganathan S., Usaid S., Devi S., Sethumadhavan V A cross-sectional study on prevalence of eating disorder and body image disturbance among female undergraduate medical students. JOURNAL OF MENTAL HEALTH AND HUMAN BEHAVIOUR. 2020;25(1):53-56.

3498. Bernard Arnaud, Ancel Deborah, Neyrinck Audrey, Dastugue Aurelie, Bindels Laure, Delzenne Nathalie, Besnard Philippe A Preventive Prebiotic Supplementation Improves the Sweet Taste Perception in Diet-Induced Obese Mice. NUTRIENTS. 2019;11(3):.

3499. Gibson Derrick, Prochaska John, Yu Xiaoying, Kaul Sapna An examination between census tract unhealthy food availability and colorectal cancer incidence. CANCER EPIDEMIOLOGY. 2020;67():.

3500. Striegel-Moore R., DeBar L., Wilson G., Dickerson J., Rosselli F., Perrin N., Lynch F., Kraemer H. Health services use in eating disorders. PSYCHOLOGICAL MEDICINE. 2008;38(10):1465-1474.

3501. Hermont Ana, Pordeus Isabela, Paiva Saul, Nogueira Guimaraes Abreu Mauro, Auad Sheyla Eating Disorder Risk Behavior and Dental Implications among Adolescents. INTERNATIONAL JOURNAL OF EATING DISORDERS. 2013;46(7):677-683.

3502. Malinowska Anna Easy Diet Screener: A quick and easy tool for determining dietary patterns associated with lipid profile and body adiposity. JOURNAL OF HUMAN NUTRITION AND DIETETICS. 2022;35(3):590-604.

3503. Reas Deborah, Wisting Line, Stedal Kristin, Dahlgren Camilla Unhealthy eating and weight dissatisfaction in adolescents who never, occasionally, or regularly use smokeless tobacco (Swedish snus). INTERNATIONAL JOURNAL OF EATING DISORDERS. 2019;52(7):846-854.

3504. Barthels Friederike, Barrada Juan, Roncero Maria Orthorexia nervosa and healthy orthorexia as new eating styles. PLOS ONE. 2019;14(7):.

3505. Domingues Rita, Carmo Claudia Disordered eating behaviours and correlates in yoga practitioners: a systematic review. EATING AND WEIGHT DISORDERS-STUDIES ON ANOREXIA BULIMIA AND OBESITY. 2019;24(6):1015-1024.

3506. Unikel-Santoncini Claudia, Munoz-Espinosa Alicia, Leon-Vazquez Concepcion, Rivera-Marquez Jose, Parra-Carriedo Alicia, Vazquez-Velazquez Veronica, Rocha-Velis Ingrid, Morcelle Gladys, Diaz-Gutierrez Mary Sensitivity, specificity, and cut-off points in the Brief Questionnaire for Measuring Disordered Eating Behaviors in Mexican Women. EATING AND WEIGHT DISORDERS-STUDIES ON ANOREXIA BULIMIA AND OBESITY. 2021;26(7):2401-2405.

3507. Stok F., Ridder Denise, Vet Emely, Nureeva Liliya, Luszczynska Aleksandra, Wardle Jane, Gaspar Tania, Wit John Hungry for an intervention? Adolescents' ratings of acceptability of eating-related intervention strategies. BMC PUBLIC HEALTH. 2016;16():.

3508. Dufour Rachel, Novack Kaylee, Picard Louis, Chadi Nicholas, Booij Linda The use of technology in the treatment of youth with eating disorders: A scoping review. JOURNAL OF EATING DISORDERS. 2022;10(1):.

3509. Berloffa Stefano, Salvati Andrea, Pantalone Gloria, Falcioni Ludovica, Rizzi Micaela, Naldini Francesca, Masi Gabriele, Gagliano Antonella Steroid treatment response to post

SARS-CoV-2 PANS symptoms: Case series. FRONTIERS IN NEUROLOGY. 2023;14():.

3510. Casper RC, Jabine LN An eight-year follow-up: Outcome from adolescent compared to adult onset anorexia nervosa. JOURNAL OF YOUTH AND ADOLESCENCE. 1996;25(4):499-517.

3511. Ferguson Gail, Muzaffar Henna, Iturbide Maria, Chu Hui, Gardner Julie Feel American, Watch American, Eat American? Remote Acculturation, TV, and Nutrition Among Adolescent-Mother Dyads in Jamaica. CHILD DEVELOPMENT. 2018;89(4):1360-1377.

3512. Shi Liu-Bin, Huang Jian-Hua, Han Bao-San Hypoxia inducible factor-1 alpha mediates protective effects of ischemic preconditioning on ECV-304 endothelial cells. WORLD JOURNAL OF GASTROENTEROLOGY. 2007;13(16):2369-2373.

3513. Oliveira Amanda, Rezende Alexandre, Calabria Luciana Overweight and risk factors in university students during a graduation course. RBONE-REVISTA BRASILEIRA DE OBESIDADE NUTRICAO E EMAGRECIMENTO. 2020;14(85):207-215.

3514. Roberts Marion, Tchanturia Kate, Treasure Janet Is attention to detail a similarly strong candidate endophenotype for anorexia nervosa and bulimia nervosa?. WORLD JOURNAL OF BIOLOGICAL PSYCHIATRY. 2013;14(6):452-463.

3515. Shaw Jena, Herzog David, Clark Vicki, Berner Laura, Eddy Kamryn, Franko Debra, Lowe Michael Elevated pre-morbid weights in bulimic individuals are usually surpassed post-morbidly: Implications for perpetuation of the disorder. INTERNATIONAL JOURNAL OF EATING DISORDERS. 2012;45(4):512-523.

3516. Taylor C., Bryson Susan, Luce Kristine, Cunning Darby, Doyle Angela, Abascal Liana, Rockwell Roxanne, Dev Pavarti, Winzelberg Andrew, Wilfley Denise Prevention of eating disorders in at-risk college-age women. ARCHIVES OF GENERAL PSYCHIATRY. 2006;63(8):881-888.

3517. Nouri Fatemeh, Sadeghi Masoumeh, Mohammadifard Noushin, Roohafza Hamidreza, Feizi Awat, Sarrafzadegan Nizal Longitudinal association between an overall diet quality index and latent profiles of cardiovascular risk factors: results from a population based 13-year follow up cohort study. NUTRITION \& METABOLISM. 2021;18(1):.

3518. Sattler Frank, Eickmeyer Sarah, Eisenkolb Julia Body image disturbance in children and adolescents with anorexia nervosa and bulimia nervosa: a systematic review. EATING AND WEIGHT DISORDERS-STUDIES ON ANOREXIA BULIMIA AND OBESITY. 2020;25(4):857-865.

3519. Whitney Kristin, Holtzman Bryan, Cook Danielle, Bauer Stuart, Maffazioli Giovana, Parziale Allyson, Ackerman Kathryn Low energy availability and impact sport participation as risk factors for urinary incontinence in female athletes. JOURNAL OF PEDIATRIC UROLOGY. 2021;17(3):.

3520. Abbott Alexandra, Bird Mackenzie, Wild Emily, Brown Symone, Stewart Greg, Mulcahey Mary Part I: epidemiology and risk factors for stress fractures in female athletes. PHYSICIAN AND SPORTSMEDICINE. 2020;48(1):17-24.

3521. Richter Felicitas, Braehler Elmar, Strauss Bernhard, Berger Uwe Factoranalytic Structure of a Short Version of the Eating Attitudes Test (EAT-13) and Prevalences of Disordered Eating in a Representative German Sample. PSYCHOTHERAPIE PSYCHOSOMATIK MEDIZINISCHE PSYCHOLOGIE. 2014;64(12):465-471.

3522. Stojek Monika, Maples-Keller Jessica, Dixon Hayley, Umpierrez Guillermo, Gillespie Charles, Michopoulos Vasiliki Associations of childhood trauma with food addiction and insulin resistance in African-American women with diabetes mellitus. APPETITE. 2019;141():.

3523. Hutson Peter, Vickers S., Goddard S., Hallam M., Brammer R., Heal D. Preclinical Evidence to Demonstrate that Lisdexamfetamine Prevents Impulsivity in Binge-Eating. NEUROPSYCHOPHARMACOLOGY. 2015;40(1):S134-S135.

3524. Lang Brittany, Ahlich Erica, Verzijl Christina, Thompson J., Rancourt Diana The role of drive for thinness in the association between weight status misperception and disordered eating. EATING BEHAVIORS. 2019;35():.

3525. Vitiello B, Lederhendler I Research on eating disorders: Current status and future prospects. BIOLOGICAL PSYCHIATRY. 2000;47(9):777-786.

3526. Monge-Rojas Rafael, O'Neill June, Lee-Bravatti Michelle, Mattei Josiemer A Traditional Costa Rican Adolescents' Diet Score Is a Valid Tool to Capture Diet Quality and Identify Sociodemographic Groups With Suboptimal Diet. FRONTIERS IN PUBLIC HEALTH.

2021;9():.

3527. Kaap-Deeder Jolene, Smets Jos, Boone Liesbet The Impeding Role of Self-Critical Perfectionism on Therapeutic Alliance During Treatment and Eating Disorder Symptoms at Follow-up in Patients with an Eating Disorder. PSYCHOLOGICA BELGICA. 2016;56(2):101-110.

3528. Veilleux Jennifer, Pollert Garrett, Zielinski Melissa, Shaver Jennifer, Hill Morgan Behavioral Assessment of the Negative Emotion Aspect of Distress Tolerance: Tolerance to Emotional Images. ASSESSMENT. 2019;26(3):386-403.

3529. Anderson-Fye EP A{''}coca-cola{''} shape: Cultural change, body image, and eating disorders in San Andres, Belize. CULTURE MEDICINE AND PSYCHIATRY. 2004;28(4):561-595.

3530. Yao Shuyang, Kuja-Halkola Ralf, Thornton Laura, Norring Claes, Almqvist Catarina, D'Onofrio Brian, Lichtenstein Paul, Langstrom Niklas, Bulik Cynthia, Larsson Henrik Risk of being convicted of theft and other crimes in anorexia nervosa and bulimia nervosa: A prospective cohort study in a Swedish female population. INTERNATIONAL JOURNAL OF EATING DISORDERS. 2017;50(9):1095-1103.

3531. Gerrits Joanne, O'Hara Ross, Piko Bettina, Gibbons Frederick, Ridder Denise, Keresztes Noemi, Kamble Shanmukh, Wit John Self-control, diet concerns and eater prototypes influence fatty foods consumption of adolescents in three countries. HEALTH EDUCATION RESEARCH. 2010;25(6):1031-1041.

3532. Muehleck Julia, Richter Felicitas, Bell Luise, Wick Katharina, Strauss Bernhard, Berger Uwe Regional utilization of the healthcare system and treatment prevalence in eating disorders. Retrospective cohort study with insured females aged between 11 and 25 years. PSYCHOTHERAPEUT. 2018;63(4):315-321.

3533. Miller Alexia, Racine Sarah, Klonsky E. Symptoms of anorexia nervosa and bulimia nervosa have differential relationships to borderline personality disorder symptoms. EATING DISORDERS. 2021;29(2):161-174.

3534. Hussenoeder Felix, Pabst Alexander, Conrad Ines, Loebner Margrit, Engel Christoph, Zeynalova Samira, Reyes Nigar, Glaesmer Heide, Hinz Andreas, Witte Veronica, Schroeter Matthias, Wirkner Kerstin, Kirsten Toralf, Loeffler Markus, Villringer Arno, Riedel-Heller

Steffi Anxiety and Food Addiction in Men and Women: Results From the Longitudinal LIFE-Adult-Study. FRONTIERS IN PSYCHIATRY. 2022;13():.

3535. Matusek JA, Wendt SJ, Wiseman CV Dissonance thin-ideal and didactic healthy behavior eating disorder prevention programs: Results from a controlled trial. INTERNATIONAL JOURNAL OF EATING DISORDERS. 2004;36(4):376-388.

3536. Green M., Kroska A., Herrick A., Bryant B., Sage E., Miles L., Ravet M., Powers M., Whitegoat W., Linkhart R., King B. A preliminary trial of an online dissonance-based eating disorder intervention. EATING BEHAVIORS. 2018;31():88-98.

3537. Barchitta M., Maugeri A., Agrifoglio O., Favara G., La Mastra C., La Rosa M., San Lio R., Agodi A. Dietary patterns and school performance: evidence from a sample of adolescents in Sicily, Italy. ANNALI DI IGIENE MEDICINA PREVENTIVA E DI COMUNITA. 2019;31(2, 1):72-80.

3538. Neumark-Sztainer Dianne, Wall Melanie, Haines Jess, Story Mary, Eisenberg Marla Why does dieting predict weight gain in adolescents? Findings from project EAT-II: A 5-year longitudinal study. JOURNAL OF THE AMERICAN DIETETIC ASSOCIATION. 2007;107(3):448-455.

3539. Bagherniya Mohammad, Darani Firoozeh, Keshavarz Seyed, Movahhed Sara, Allipour-Birgani Ramesh, Sharma Manoj, Safarian Mohammad, Taghipour Ali The Impact of Adolescent Friendships on Unhealthy Eating Behaviors of Overweight and Obese Adolescents: A Qualitative Study. INTERNATIONAL JOURNAL OF PEDIATRICS-MASHHAD. 2018;6(10):8385-8398.

3540. Lampis Jessica, Agus Mirian, Cacciarru Barbara Quality of Family Relationships as Protective Factors of Eating Disorders: An Investigation Amongst Italian Teenagers. APPLIED RESEARCH IN QUALITY OF LIFE. 2014;9(2):309-324.

3541. Cortes-Garcia Laura, Hoffmann Svenja, Warschburger Petra, Senra Carmen Exploring the reciprocal relationships between adolescents' perceptions of parental and peer attachment and disordered eating: A multiwave cross-lagged panel analysis. INTERNATIONAL JOURNAL OF EATING DISORDERS. 2019;52(8):924-934.

3542. Roberto Christina How Psychological Insights Can Inform Food Policies to Address Unhealthy Eating Habits. AMERICAN PSYCHOLOGIST. 2020;75(2, SI):265-273.

3543. Dakanalis Antonios, Favagrossa Laura, Clerici Massimo, Prunas Antonio, Colmegna Fabrizia, Zanetti M., Riva Giuseppe Body Dissatisfaction and Eating Disorder Symptomatology: A Latent Structural Equation Modeling Analysis of Moderating Variables in 18-to-28-Year-Old Males. JOURNAL OF PSYCHOLOGY. 2015;149(1):85-112.

3544. Heianza Yoriko, Ma Wenjie, DiDonato Joseph, Sun Qi, Rimm Eric, Hu Frank, Rexrode Kathryn, Manson JoAnn, Qi Lu Long-Term Changes in Gut Microbial Metabolite Trimethylamine N-Oxide and Coronary Heart Disease Risk. JOURNAL OF THE AMERICAN COLLEGE OF CARDIOLOGY. 2020;75(7):763-772.

3545. Fararouei Mohammad, Iqbal Aqsa, Rezaian Shahab, Gheibi Zahra, Dianatinasab Aria, Shakarami Saba, Dianatinasab Mostafa Dietary Habits and Physical Activity are Associated With the Risk of Breast Cancer Among Young Iranian Women: A Case-control Study on 1010 Premenopausal Women. CLINICAL BREAST CANCER. 2019;19(1):E127-E134.

3546. Pereira Beatriz, Rosario Pedro, Nunez Jose, Rosendo Daniela, Roces Cristina, Magalhaes Paula Food Availability, Motivational-Related Factors, and Food Consumption: A Path Model Study with Children. INTERNATIONAL JOURNAL OF ENVIRONMENTAL RESEARCH AND PUBLIC HEALTH. 2021;18(24):.

3547. Ozcan Burcu, Yeslikaya Burcu Adverse Effect of Emotional Eating Developed During the COVID-19 Pandemic on Healthy Nutrition, a Vicious Circle: A cross-sectional descriptive study. REVISTA ESPANOLA DE NUTRICION HUMANA Y DIETETICA. 2021;25(2):.

3548. Huang Qiren, He Ming, Chen Heping, Shao Lijian, Liu Dan, Luo Yongming, Dai Yucheng Protective effects of sasanquasaponin on injury of endothelial cells induced by anoxia and reoxygenation in vitro. BASIC \& CLINICAL PHARMACOLOGY \& TOXICOLOGY. 2007;101(5):301-308.

3549. Lindner Danielle, Tantleff-Dunn Stacey The Development and Psychometric Evaluation of the Self-Objectification Beliefs and Behaviors Scale. PSYCHOLOGY OF WOMEN QUARTERLY. 2017;41(2):254-272.

3550. Tanofsky-Kraff Marian, Shomaker Lauren, Wilfley Denise, Young Jami, Sbrocco Tracy, Stephens Mark, Brady Sheila, Galescu Ovidiu, Demidowich Andrew, Olsen Cara, Kozlosky

Merel, Reynolds James, Yanovski Jack Excess Weight Gain Prevention in Adolescents: Three-Year Outcome Following a Randomized Controlled Trial. JOURNAL OF CONSULTING AND CLINICAL PSYCHOLOGY. 2017;85(3):218-227.

3551. Wolters Eric, Werf Ysbrand, Heuvel Odile Parkinson's disease-related disorders in the impulsive-compulsive spectrum. JOURNAL OF NEUROLOGY. 2008;255(5):48-56.

3552. Hu Yu-Pei, Wehrly Rebecca, Gorrindo Tristan, Hezel Dianne, Gironda Christina, Jenike Michael, Stewart S. Gender specific short stature in male adolescents with obsessive-compulsive disorder. JOURNAL OF OBSESSIVE-COMPULSIVE AND RELATED DISORDERS. 2013;2(1):30-36.

3553. Ohm R, Enzell K, Angmar-Mansson B Oral status of 81 subjects with eating disorders. EUROPEAN JOURNAL OF ORAL SCIENCES. 1999;107(3):157-163.

3554. Steinberg Dori, Tate Deborah, Bennett Gary, Ennett Susan, Samuel-Hodge Carmen, Ward Dianne Daily Self-Weighing and Adverse Psychological Outcomes A Randomized Controlled Trial. AMERICAN JOURNAL OF PREVENTIVE MEDICINE. 2014;46(1):24-29.

3555. Vliet Jolanda, Gustafsson Per, Nelson Nina Feeling `too fat' rather than being `too fat' increases unhealthy eating habits among adolescents - even in boys. FOOD \& NUTRITION RESEARCH. 2016;60():.

3556. Stewart DA, Carter JC, Drinkwater J, Hainsworth J, Fairburn CG Modification of eating attitudes and behavior in adolescent girls: A controlled study. INTERNATIONAL JOURNAL OF EATING DISORDERS. 2001;29(2):107-118.

3557. Feldhege Johannes, Bilic Sally, Ali Kathina, Fassnacht Daniel, Moessner Markus, Farrer Louise, Griffiths Kathleen, Bauer Stephanie Knowledge and Myths about Eating Disorders in a German Adolescent Sample: A Preliminary Investigation. INTERNATIONAL JOURNAL OF ENVIRONMENTAL RESEARCH AND PUBLIC HEALTH. 2022;19(11):.

3558. Kirsten Doret, Plessis Wynand Lived Experiences of Subclinical Eating Disorder: Female Students' Perceptions. JOURNAL OF PSYCHOLOGY IN AFRICA. 2008;18(4):561-572.

3559. Zimmerman Jacqueline, Fisher Martin Avoidant/Restrictive Food Intake Disorder (ARFID). CURRENT PROBLEMS IN PEDIATRIC AND ADOLESCENT HEALTH CARE.

2017;47(4):35-43.

3560. Baechle Christina, Castillo Katty, Strassburger Klaus, Stahl-Pehe Anna, Meissner Thomas, Holl Reinhard, Giani Guido, Rosenbauer Joachim, Surveillance German, Initiative DPV-Sci Is disordered eating behavior more prevalent in adolescents with early-onset type 1 diabetes than in their representative peers?. INTERNATIONAL JOURNAL OF EATING DISORDERS. 2014;47(4):342-352.

3561. Pooni Jyoti, Ninteman Aafke, Bryant-Waugh Rachel, Nicholls Dasha, Mandy William Investigating autism spectrum disorder and autistic traits in early onset eating disorder. INTERNATIONAL JOURNAL OF EATING DISORDERS. 2012;45(4):583-591.

3562. Mestre Tiago, Zurowski Mateusz, Fox Susan 5-Hydroxytryptamine 2A receptor antagonists as potential treatment for psychiatric disorders. EXPERT OPINION ON INVESTIGATIONAL DRUGS. 2013;22(4):411-421.

3563. Kiezebrink K., Campbell D., Mann E., Blundell J. Similarities and differences between excessive exercising anorexia nervosa patients compared with DSM-IV defined anorexia nervosa subtypes. EATING AND WEIGHT DISORDERS-STUDIES ON ANOREXIA BULIMIA AND OBESITY. 2009;14(4):E199-E204.

3564. Benjet Corina, Borges Guilherme, Mendez Enrique, Albor Yesica, Casanova Leticia, Orozco Ricardo, Curiel Teresa, Fleiz Clara, Elena Medina-Mora Maria Eight-year incidence of psychiatric disorders and service use from adolescence to early adulthood: longitudinal follow-up of the Mexican Adolescent Mental Health Survey. EUROPEAN CHILD \& ADOLESCENT PSYCHIATRY. 2016;25(2):163-173.

3565. Peterson Claire, Baker Jessica, Thornton Laura, Trace Sara, Mazzeo Suzanne, Neale Michael, Munn-Chernoff Melissa, Lichtenstein Paul, Pedersen Nancy, Bulik Cynthia Genetic and environmental components to self-induced vomiting. INTERNATIONAL JOURNAL OF EATING DISORDERS. 2016;49(4):421-427.

3566. Linardon Jake, Shatte Adrian, Tepper Hannah, Fuller-Tyszkiewicz Matthew A survey study of attitudes toward, and preferences for, e-therapy interventions for eating disorder psychopathology. INTERNATIONAL JOURNAL OF EATING DISORDERS. 2020;53(6):907-916.

3567. Wang Wenying, Chen Zhiyan, Ding Xinfang Cyberbullying victimization and disordered eating behaviors: The mediating roles of self-compassion and self-objectification. APPETITE. 2022;178():.

3568. Penney Tarra, Jones Nicholas, Adams Jean, Maguire Eva, Burgoine Thomas, Monsivais Pablo Utilization of Away-From-Home Food Establishments, Dietary Approaches to Stop Hypertension Dietary Pattern, and Obesity. AMERICAN JOURNAL OF PREVENTIVE MEDICINE. 2017;53(5):E155-E163.

3569. McAlpin Ngina, Elaiho Cordelia, Khan Farrah, Cruceta Cristina, Goytia Crispin, Vangeepuram Nita Use of Focus Groups to Inform a New Community-Based Youth Diabetes Prevention Program. INTERNATIONAL JOURNAL OF ENVIRONMENTAL RESEARCH AND PUBLIC HEALTH. 2022;19(15):.

3570. Flament Martine, Hill Erin, Buchholz Annick, Henderson Katherine, Tasca Giorgio, Goldfield Gary Internalization of the thin and muscular body ideal and disordered eating in adolescence: The mediation effects of body esteem. BODY IMAGE. 2012;9(1):68-75.

3571. Naab Silke, Fumi Markus, Schlegl Sandra, Voderholzer Ulrich Inpatient Treatment of Children and Adolescents With Anorexia nervosa and Bulimia Nervosa. KINDHEIT UND ENTWICKLUNG. 2019;28(4):230-241.

3572. Preston Kerry, Corwin Rebecca, Bader Julia, Crimmins Stephen Relatively enriched housing conditions delay binge onset but do not attenuate binge size. PHYSIOLOGY \& BEHAVIOR. 2018;184():196-204.

3573. Levinson Cheri, Vanzhula Irina, Smith Tosha, Stice Eric Group and longitudinal intra-individual networks of eating disorder symptoms in adolescents and young adults at-risk for an eating disorder. BEHAVIOUR RESEARCH AND THERAPY. 2020;135():.

3574. Tanaka H, Kiriike N, Nagata T, Riku K Outcome of severe anorexia nervosa patients receiving inpatient treatment in Japan: An 8-year follow-up study. PSYCHIATRY AND CLINICAL NEUROSCIENCES. 2001;55(4):389-396.

3575. Hao Ming, Fang Yifei, Yan Wenjing, Gu Junwang, Hao Yanbin, Wu Chunmei Relationship between body dissatisfaction, insufficient physical activity, and disordered eating behaviors among university students in southern China. BMC PUBLIC HEALTH.

2022;22(1):.

3576. Alfreeh Leenah, Alomar Suliman, Aljuraiban Ghadeer Association of diet quality with serum high-sensitivity C-reactive protein level and the adherence to the Saudi dietary guidelines among female college students. JOURNAL OF KING SAUD UNIVERSITY SCIENCE. 2022;34(2):.

3577. Castelnuovo Gianluca, Manzoni Gian, Cuzziol Paola, Cesa Gian, Tuzzi Cristina, Villa Valentina, Liuzzi Antonio, Petroni Maria, Molinari Enrico TECNOB: study design of a randomized controlled trial of a multidisciplinary telecare intervention for obese patients with type-2 diabetes. BMC PUBLIC HEALTH. 2010;10():.

3578. Davis C, Katzman DK, Kaptein S, Kirsh C, Brewer H, Kalmbach K, Olmsted MP, Woodside DB, Kaplan AS The prevalence of high-level exercise in the eating disorders: Etiological implications. COMPREHENSIVE PSYCHIATRY. 1997;38(6):321-326.

3579. Dudova Iva, Kocourkova Jana, Koutek Jiri Early-onset anorexia nervosa in girls with Asperger syndrome. NEUROPSYCHIATRIC DISEASE AND TREATMENT. 2015;11():1639-1643.

3580. Alger-Mayer S., Rosati C., Polimeni J., Malone M. Preoperative Binge Eating Status and Gastric Bypass Surgery: A Long-Term Outcome Study. OBESITY SURGERY. 2009;19(2):139-145.

3581. Vartanian Lenny, Smyth Joshua, Zawadzki Matthew, Heron Kristin, Coleman Sulamunn Early Adversity, Personal Resources, Body Dissatisfaction, and Disordered Eating. INTERNATIONAL JOURNAL OF EATING DISORDERS. 2014;47(6):620-629.

3582. Nagata Jason, Murray Stuart, Bibbins-Domingo Kirsten, Garber Andrea, Mitchison Deborah, Griffiths Scott Predictors of muscularity-oriented disordered eating behaviors in US young adults: A prospective cohort study. INTERNATIONAL JOURNAL OF EATING DISORDERS. 2019;52(12, SI):1380-1388.

3583. Cotrufo P., Cella S., Cremato F., Labella A. Eating disorder attitude and abnormal eating behaviours in a sample of 11-13-year-old school children: The role of pubertal body transformation. EATING AND WEIGHT DISORDERS-STUDIES ON ANOREXIA BULIMIA AND OBESITY. 2007;12(4):154-160.

3584. Kroeller Katja, Warschburger Petra Problematic Eating Behavior in Childhood: Do Maternal Feeding Patterns Play a Role?. PRAXIS DER KINDERPSYCHOLOGIE UND KINDERPSYCHIATRIE. 2011;60(4):253-269.

3585. Binford RB, Mussell MP, Crosby RD, Peterson CB, Crow SJ, Mitchell JE Coping strategies in bulimia nervosa treatment: Impact on outcome in group cognitive-behavioral therapy. JOURNAL OF CONSULTING AND CLINICAL PSYCHOLOGY. 2005;73(6):1089-1096.

3586. Bailer Josef, Schwarz Daniela, Witthoeft Michael, Stuebinger Cornelia, Rist Fred Prevalence of Mental Disorders Among College Students at a German University. PSYCHOTHERAPIE PSYCHOSOMATIK MEDIZINISCHE PSYCHOLOGIE. 2008;58(11):423-429.

3587. Satyal Medha, Basso Julia, Tegge Allison, Metpally Anvitha, Bickel Warren A Novel Model of Obesity Prediction: Neurobehaviors as Targets for Treatment. BEHAVIORAL NEUROSCIENCE. 2021;135(3):426-442.

3588. Reaves Danielle, Dickson Joanne, Halford Jason, Christiansen Paul, Hardman Charlotte A Qualitative Analysis of Problematic and Non-problematic Alcohol Use After Bariatric Surgery. OBESITY SURGERY. 2019;29(7):2200-2209.

3589. Svantorp-Tveiten Kethe, Ivarsson Andreas, Torstveit Monica, Sundgot-Borgen Christine, Mathisen Therese, Bratland-Sanda Solfrid, Rosenvinge Jan, Friborg Oddgeir, Pettersen Gunn, Sundgot-Borgen Jorunn The Healthy Body Image Intervention and Reduction in Eating Disorder Symptomatology and Muscle Building Supplement Use in High School Students: A Study of Mediating Factors. FRONTIERS IN PSYCHOLOGY. 2022;13():.

3590. Smith FM, Latchford G, Hall RM, Millner PA, Dickson RA Indications of disordered eating behaviour in adolescent patients with idiopathic scoliosis. JOURNAL OF BONE AND JOINT SURGERY-BRITISH VOLUME. 2002;84B(3):392-394.

3591. Bradley Sarah, Reardon Claudia Bipolar disorder and eating disorders in sport: a case of comorbidity and review of treatment principles in an elite athlete. PHYSICIAN AND SPORTSMEDICINE. 2022;50(1):84-91.

3592. Springmann Marie-Luise, Svaldi Jennifer, Kiegelmann Mechthild A qualitative study of gendered psychosocial processes in eating disorder development. INTERNATIONAL JOURNAL OF EATING DISORDERS. 2022;55(7):947-955.

3593. Cloitre M, Yonkers KA, Pearlstein T, Altemus M, Davidson KW, Pigott TA Women and anxiety disorders: Implications for diagnosis and treatment. CNS SPECTRUMS. 2004;9(9):1-16.

3594. Hopwood Christopher, Clarke Analesa, Perez Marisol Pathoplasticity of bulimic features and interpersonal problems. INTERNATIONAL JOURNAL OF EATING DISORDERS. 2007;40(7):652-658.

3595. Chew Chu, Kelly Siobhan, Baeg Amerie, Oh Jean, Rajasegaran Kumudhini, Davis Courtney First presentation of restrictive early onset eating disorders in Asian children. INTERNATIONAL JOURNAL OF EATING DISORDERS. 2021;54(1, SI):81-87.

3596. Markowitz J., Lowe M., Volkening L., Laffel L. Self-reported history of overweight and its relationship to disordered eating in adolescent girls with Type 1 diabetes. DIABETIC MEDICINE. 2009;26(11):1165-1171.

3597. Espinoza Paola, Penelo Eva, Mora Marisol, Francisco Rita, Gonzalez Marcela, Raich Rosa Bidirectional Relations Between Disordered Eating, Internalization of Beauty Ideals, and Self-Esteem: A Longitudinal Study With Adolescents. JOURNAL OF EARLY ADOLESCENCE. 2019;39(9):1244-1260.

3598. Cardoso Ana, Oliveira Sara, Ferreira Claudia Negative and positive affect and disordered eating: The adaptive role of intuitive eating and body image flexibility. CLINICAL PSYCHOLOGIST. 2020;24(2):176-185.

3599. Gonzalez Aranzazu, Kohn Michael, Clarke Simon Eating disorders in adolescents. AUSTRALIAN FAMILY PHYSICIAN. 2007;36(8):614-619.

3600. Viljakainen H., Valta H., Lipsanen-Nyman M., Saukkonen T., Kajantie E., Andersson S., Makitie O. Bone Characteristics and Their Determinants in Adolescents and Young Adults with Early-Onset Severe Obesity. CALCIFIED TISSUE INTERNATIONAL. 2015;97(4):364-375.

3601. Krist Alex, Davidson Karina, Mangione Carol, Barry Michael, Cabana Michael, Caughey Aaron, Donahue Katrina, Doubeni Chyke, Epling John, Kubik Martha, Landefeld Seth, Ogedegbe Gbenga, Pbert Lori, Silverstein Michael, Simon Melissa, Tseng Chien-Wen, Wong John, Force US Behavioral Counseling Interventions to Promote a Healthy Diet and Physical Activity for Cardiovascular Disease Prevention in Adults With Cardiovascular Risk Factors:

US Preventive Services Task Force Recommendation Statement. JAMA-JOURNAL OF THE AMERICAN MEDICAL ASSOCIATION. 2020;324(20):2069-2075.

3602. Kim SS Role of fluoxetine in anorexia nervosa. ANNALS OF PHARMACOTHERAPY. 2003;37(6):890-892.

3603. Plateau Carolyn, McDermott Hilary, Arcelus Jon, Meyer Caroline Identifying and preventing disordered eating among athletes: Perceptions of track and field coaches. PSYCHOLOGY OF SPORT AND EXERCISE. 2014;15(6, SI):721-728.

3604. Angst J, Gamma A, Benazzi F, Silverstein B, Ajdacic-Gross V, Eich D, Rossler W Atypical depressive syndromes in varying definitions. EUROPEAN ARCHIVES OF PSYCHIATRY AND CLINICAL NEUROSCIENCE. 2006;256(1):44-54.

3605. Sala Margarita, Linde Jennifer, Crosby Ross, Pacanowski Carly Affect and engagement in healthy and unhealthy weight control behaviors in college women: An ecological momentary assessment study. EATING BEHAVIORS. 2021;40():.

3606. Martyn-Nemeth Pamela, Penckofer Sue, Gulanick Meg, Velsor-Friedrich Barbara, Bryant Fred The Relationships Among Self-Esteem, Stress, Coping, Eating Behavior, and Depressive Mood in Adolescents. RESEARCH IN NURSING \& HEALTH. 2009;32(1):96-109.

3607. Roessner Anne, Juniak Izabela, Noort Betteke, Pfeiffer Ernst, Lehmkuhl Ulrike, Kappel Viola Cognitive Flexibility in Juvenile Anorexia Nervosain Relation to Comorbid Symptoms of Depression, Obsessive Compulsive Symptoms and Duration of Illness. ZEITSCHRIFT FUR KINDER-UND JUGENDPSYCHIATRIE UND PSYCHOTHERAPIE. 2017;45(5):371-380.

3608. Machairiotis Nikolaos, Vasilakaki Sofia, Minns Laura, Malakasis Anastasios Nutrients that modulate gestational diabetes mellitus: A systematic review of cohort studies Jan 2019-Jan 2020. INTERNATIONAL JOURNAL OF CLINICAL PRACTICE. 2021;75(8):.

3609. Conti Chiara, Di Nardo Maria, Lanzara Roberta, Guagnano Maria, Cardi Valentina, Porcelli Piero Improvement in binge eating and alexithymia predicts weight loss at 9-month follow-up of the lifestyle modification program. EATING AND WEIGHT DISORDERS-STUDIES ON ANOREXIA BULIMIA AND OBESITY. 2023;28(1):.

3610. Jessri Mahsa, Lou Wendy, L'Abbe Mary The 2015 Dietary Guidelines for Americans is associated with a more nutrient-dense diet and a lower risk of obesity. AMERICAN JOURNAL OF CLINICAL NUTRITION. 2016;104(5):1378-1392.

3611. Mond JM, Hay PJ, Rodgers B, Owen C, Beumont PJV Beliefs of women concerning causes and risk factors for bulimia nervosa. AUSTRALIAN AND NEW ZEALAND JOURNAL OF PSYCHIATRY. 2004;38(6):463-469.

3612. Iuso Salvatore, Bellomo Antonello, Pagano Tiziana, Carnevale Raffaella, Ventriglio Antonio, Petito Annamaria Sport Activity as Risk or Protective Factor in Feeding and Eating Disorder. BEHAVIORAL SCIENCES. 2019;9(12):.

3613. Brown Amy, McClelland Jessica, Boysen Elena, Mountford Victoria, Glennon Danielle, Schmidt Ulrike The FREED Project (first episode and rapid early intervention in eating disorders): service model, feasibility and acceptability. EARLY INTERVENTION IN PSYCHIATRY. 2018;12(2):250-257.

3614. Caso Gerarda, Vecchio Riccardo Factors influencing independent older adults (un)healthy food choices: A systematic review and research agenda. FOOD RESEARCH INTERNATIONAL. 2022;158():.

3615. Muzi Laura, Tieghi Laura, Rugo Michele, Lingiardi Vittorio Personality as a predictor of symptomatic change in a residential treatment setting for anorexia nervosa and bulimia nervosa. EATING AND WEIGHT DISORDERS-STUDIES ON ANOREXIA BULIMIA AND OBESITY. 2021;26(4):1195-1209.

3616. Gattu Shilpa, Rashid Rashid, Khachemoune Amor Self-induced Skin Lesions: A Review of Dermatitis Artefacta. CUTIS. 2009;84(5):247-251.

3617. Samadi Mehnoosh, Moradi Shima, Moradinazar Mehdi, Mostafai Roghayeh, Pasdar Yahya Dietary pattern in relation to the risk of Alzheimer's disease: a systematic review. NEUROLOGICAL SCIENCES. 2019;40(10):2031-2043.

3618. Kaikkonen Jari, Mikkila Vera, Raitakari Olli Role of Childhood Food Patterns on Adult Cardiovascular Disease Risk. CURRENT ATHEROSCLEROSIS REPORTS. 2014;16(10):.

3619. Johns Gemma, Taylor Bridget, John Ann, Tan Jacinta Current eating disorder healthcare services - the perspectives and experiences of individuals with eating disorders, their families and health professionals: systematic review and thematic synthesis. BJPSYCH OPEN. 2019;5(4):.

3620. Stice Eric, Rohde Paul, Butryn Meghan, Desjardins Christopher, Shaw Heather Enhancing Efficacy of a Brief Obesity and Eating Disorder Prevention Program: Long-Term Results from an Experimental Therapeutics Trial. NUTRIENTS. 2023;15(4):.

3621. Arellano J., Torres M., Rivera C., Moncada L., Jimenez-Capdeville M. Abnormal eating attitudes in Mexican female students: A study of prevalence and sociodemographic-clinical associated factors. EATING AND WEIGHT DISORDERS-STUDIES ON ANOREXIA BULIMIA AND OBESITY. 2009;14(2-3):E42-E49.

3622. Segal Leonie, Opie Rachelle A nutrition strategy to reduce the burden of diet related disease: access to dietician services must complement population health approaches. FRONTIERS IN PHARMACOLOGY. 2015;6():.

3623. Wentz E, Lacey JH, Waller G, Rastam M, Turk J, Gillberg C Childhood onset neuropsychiatric disorders in adult eating disorder patients - A pilot study. EUROPEAN CHILD \& ADOLESCENT PSYCHIATRY. 2005;14(8):431-437.

3624. Roehrig M, Thompson JK, Brannick M, Berg P Dissonance-based eating disorder prevention program: A preliminary dismantling investigation. INTERNATIONAL JOURNAL OF EATING DISORDERS. 2006;39(1):1-10.

3625. Matsushita S, Suzuki K, Murayama M, Nishiguchi N, Hishimoto A, Takeda A, Shirakawa O, Higuchi S Serotonin transporter regulatory region polymorphism is associated with anorexia nervosa. AMERICAN JOURNAL OF MEDICAL GENETICS PART B-NEUROPSYCHIATRIC GENETICS. 2004;128B(1):114-117.

3626. Zarychta Karolina, Luszczynska Aleksandra, Scholz Urte The association between automatic thoughts about eating, the actual-ideal weight discrepancies, and eating disorders symptoms: a longitudinal study in late adolescence. EATING AND WEIGHT DISORDERS-STUDIES ON ANOREXIA BULIMIA AND OBESITY. 2014;19(2):199-207.

3627. Baraskewich Jessica, Climie Emma The relation between symptoms of ADHD and symptoms of eating disorders in university students. JOURNAL OF GENERAL PSYCHOLOGY.

2022;149(3):405-419.

3628. Ali Amira, Kunugi Hiroshi Apitherapy for Age-Related Skeletal Muscle Dysfunction (Sarcopenia): A Review on the Effects of Royal Jelly, Propolis, and Bee Pollen. FOODS. 2020;9(10):.

3629. Tam Carmen, Ng Cherry, Yu Chak, Young Betty Disordered eating attitudes and behaviours among adolescents in Hong Kong: Prevalence and correlates. JOURNAL OF PAEDIATRICS AND CHILD HEALTH. 2007;43(12):811-817.

3630. Baceviciene Migle, Jankauskiene Rasa Associations between Body Appreciation and Disordered Eating in a Large Sample of Adolescents. NUTRIENTS. 2020;12(3):.

3631. Ferreiro Fatima, Seoane Gloria, Senra Carmen Gender-related Risk and Protective Factors for Depressive Symptoms and Disordered Eating in Adolescence: A 4-year Longitudinal Study. JOURNAL OF YOUTH AND ADOLESCENCE. 2012;41(5):607-622.

3632. Reininghaus Eva, Lackner Nina, Fellendorf Frederike, Bengesser Susanne, Birner Armin, Reininghaus Bernd, Unterweger Renate, Platzer Martina, Wallner-Liebmann Sandra, Zelzer Sieglinde, Mangge Harald, Fuchs Dietmar, Kapfhammer Hans-Peter, McIntyre Roger Weight cycling in bipolar disorder. JOURNAL OF AFFECTIVE DISORDERS. 2015;171():33-38.

3633. Frieiro Padin Paula, Gonzalez-Rodriguez Ruben, Verde-Diego Carmen, Vazquez-Perez Raquel Social Media and Eating Disorder Psychopathology: A Systematic Review. CYBERPSYCHOLOGY-JOURNAL OF PSYCHOSOCIAL RESEARCH ON CYBERSPACE. 2021;15(3):.

3634. Hsu Ti, Forestell Catherine Mindfulness, mood, and food: The mediating role of positive affect. APPETITE. 2021;158():.

3635. Pinto Catarina, Ferreira Claudia, Mendes Ana, Trindade Ines Social safeness and disordered eating: Exploring underlying mechanisms of body appreciation and inflexible eating. EATING AND WEIGHT DISORDERS-STUDIES ON ANOREXIA BULIMIA AND OBESITY. 2017;22(2):303-309.

3636. Barbiero Sandra, Pellanda Lucia, Cesa Claudia, Campagnolo Paula, Beltrami Flavia, Abrantes Caroline Overweight, obesity and other risk factors for IHD in Brazilian

schoolchildren. PUBLIC HEALTH NUTRITION. 2009;12(5):710-715.

3637. Gan Wan, Nasir Mohd, Zalilah Mohd, Hazizi Abu Psychological distress as a mediator in the relationships between biopsychosocial factors and disordered eating among Malaysian university students. APPETITE. 2012;59(3):679-687.

3638. Buksh Shazna, Wit John, Hay Phillipa Sociocultural Influences Contribute to Overeating and Unhealthy Eating: Creating and Maintaining an Obesogenic Social Environment in Indigenous Communities in Urban Fiji. NUTRIENTS. 2022;14(14):.

3639. Haynes Ashleigh, Bayly Megan, Dixon Helen, McAleese Alison, Martin Jane, Chen Yan, Wakefield Melanie Obesity prevention and related public health advertising versus competing commercial advertising expenditure in Australia. HEALTH PROMOTION INTERNATIONAL. 2022;37(6):.

3640. Tanofsky-Kraff Marian, Wilfley Denise, Young Jami, Mufson Laura, Yanovski Susan, Glasofer Deborah, Salaita Christine Preventing excessive weight gain in adolescents: Interpersonal psychotherapy for binge eating in obesity (vol 15, pg 1345, 2007). OBESITY. 2007;15(10):2520.

3641. MacKay S. Legislative solutions to unhealthy eating and obesity in Australia. PUBLIC HEALTH. 2011;125(12):896-904.

3642. Chen Joanna, Piers Amani, Lesser Elin, Lowe Michael The effect of weight suppression on eating behavior: Does the intentionality of weight loss matter?. APPETITE. 2022;174():.

3643. Somasundaram Pooja, Burgess Alexandra The Role of Division III Sports Participation in the Relationship Between Perfectionism and Disordered Eating Symptomology. JOURNAL OF CLINICAL SPORT PSYCHOLOGY. 2018;12(1):57-74.

3644. Calderoni Sara, Muratori Filippo, Leggero Chiara, Narzisi Antonio, Apicella Fabio, Balottin Umberto, Carigi Tiziana, Maestro Sandra, Fabbro Franco, Urgesi Cosimo Neuropsychological functioning in children and adolescents with restrictive-type anorexia nervosa: An in-depth investigation with NEPSY-II. JOURNAL OF CLINICAL AND EXPERIMENTAL NEUROPSYCHOLOGY. 2013;35(2):167-179.

3645. Mitchell Sharon, Klein Jessalyn, Maduramente Althea Assessing the Impact of an Eating Disorders Treatment Team Approach With College Students. EATING DISORDERS. 2015;23(1):45-59.

3646. Souza Ana, Almeida Alexandre, Noll Priscilla, Noll Matias Unhealthy life habits associated with self-induced vomiting and laxative misuse in Brazilian adolescents. SCIENTIFIC REPORTS. 2021;11(1):.

3647. Del Corno Manuela, Donninelli Gloria, Conti Lucia, Gessani Sandra Linking Diet to Colorectal Cancer: The Emerging Role of MicroRNA in the Communication between Plant and Animal Kingdoms. FRONTIERS IN MICROBIOLOGY. 2017;8():.

3648. Pennesi Jamie-Lee, Wade Tracey A systematic review of the existing models of disordered eating: Do they inform the development of effective interventions?. CLINICAL PSYCHOLOGY REVIEW. 2016;43():175-192.

3649. Kong S Day treatment programme for patients with eating disorders: randomized controlled trial. JOURNAL OF ADVANCED NURSING. 2005;51(1):5-14.

3650. Dinsmore BD, Stormshak EA Family functioning and eating attitudes and behaviors in at-risk early adolescent girls: The mediating role intra-personal competencies. CURRENT PSYCHOLOGY. 2003;22(2):100-116.

3651. Mata Jutta, Gotlib Ian 5-HTTLPR Moderates the Relation between Changes in Depressive and Bulimic Symptoms in Adolescent Girls: A Longitudinal Study. INTERNATIONAL JOURNAL OF EATING DISORDERS. 2011;44(5):383-388.

3652. Kong Angela, Beresford Shirley, Imayama Ikuyo, Duggan Catherine, Alfano Catherine, Foster-Schubert Karen, Neuhouser Marian, Johnson Donna, Wang Ching-Yun, Xiao Liren, Bain Carolyn, McTiernan Anne Adoption of diet-related self-monitoring behaviors varies by race/ethnicity, education, and baseline binge eating score among overweight-to-obese postmenopausal women in a 12-month dietary weight loss intervention. NUTRITION RESEARCH. 2012;32(4):260-265.

3653. Leong Sook, Gray Andrew, Haszard Jillian, Horwath Caroline Weight-Control Methods, 3-Year Weight Change, and Eating Behaviors: A Prospective Nationwide Study of Middle-Aged New Zealand Women. JOURNAL OF THE ACADEMY OF NUTRITION AND DIETETICS.

2016;116(8):1276-1284.

3654. Stasik-O'Brien Sara, Schmidt Jeremy The role of disgust in body image disturbance: Incremental predictive power of self-disgust. BODY IMAGE. 2018;27():128-137.

3655. Lakhan Shaheen, Vieira Karen Nutritional therapies for mental disorders. NUTRITION JOURNAL. 2008;7():.

3656. Silva Janaina, Sarubbi Junior Vicente, Nascimento Viviane, Bertoli Ciro, Gallo Paulo, Leone Claudio Mothers' conceptions about excess weight in infancy and the nutritional status of their children. CLINICS. 2016;71(9):500-505.

3657. Franko DL, Mintz LB, Villapiano M, Green TC, Mainelli D, Folensbee L, Butler SF, Davidson MM, Hamilton E, Little D, Kearns M, Budman SH Food, mood, and attitude: Reducing risk for eating disorders in college women. HEALTH PSYCHOLOGY. 2005;24(6):567-578.

3658. Nierenberg AA, Phillips KA, Petersen TJ, Kelly KE, Alpert JE, Worthington JJ, Tedlow JR, Rosenbaum JF, Fava M Body dysmorphic disorder in outpatients with major depression. JOURNAL OF AFFECTIVE DISORDERS. 2002;69(1-3):141-148.

3659. Scime Melinda, Cook-Cottone Catherine Primary prevention of eating disorders: A constructivist integration of mind and body strategies. INTERNATIONAL JOURNAL OF EATING DISORDERS. 2008;41(2):134-142.

3660. Olvera Norma, McCarley Kendall, Matthews-Ewald Molly, Fisher Felicia, Jones Martinque, Flynn Erika Pathways for Disordered Eating Behaviors in Minority Girls: The Role of Adiposity, Peer Weight-Related Teasing, and Desire to Be Thinner. JOURNAL OF EARLY ADOLESCENCE. 2017;37(3):367-386.

3661. Irving LM Media exposure and disordered eating: Introduction to the special section. JOURNAL OF SOCIAL AND CLINICAL PSYCHOLOGY. 2001;20(3):259-269.

3662. Katzman Debra, Madden Sloane, Nicholls Dasha, Mawjee Karizma, Norris Mark From questions to answers: Examining the role of pediatric surveillance units in eating disorder research. INTERNATIONAL JOURNAL OF EATING DISORDERS. 2017;50(3, SI):259-265.

3663. Norwood Sarah, Bowker Anne, Buchholz Annick, Henderson Katherine, Goldfield Gary, Flament Martine Self-silencing and anger regulation as predictors of disordered eating among adolescent females. EATING BEHAVIORS. 2011;12(2):112-118.

3664. Spanos Alexia, Burt S., Klump Kelly Do Weight and Shape Concerns Exhibit Genetic Effects? Investigating Discrepant Findings. INTERNATIONAL JOURNAL OF EATING DISORDERS. 2010;43(1):29-34.

3665. Hafstad Stine, Bauer Jonas, Harris Anette, Pallesen Stale The prevalence of orthorexia in exercising populations: a systematic review and meta-analysis. JOURNAL OF EATING DISORDERS. 2023;11(1):.

3666. Yau Yvonne, Potenza Marc Gambling Disorder and Other Behavioral Addictions: Recognition and Treatment. HARVARD REVIEW OF PSYCHIATRY. 2015;23(2):134-146.

3667. Inagaki T, Horiguchi J, Tsubouchi K, Miyaoka T, Uegaki J, Seno H Late onset anorexia nervosa: Two case reports. INTERNATIONAL JOURNAL OF PSYCHIATRY IN MEDICINE. 2002;32(1):91-95.

3668. Kiani-Sheikhabadi Maryam, Bergi Marjan, Mohebbi-Dehnavi Zahra The relationship between perfectionism and body image with eating disorder in pregnancy. JOURNAL OF EDUCATION AND HEALTH PROMOTION. 2019;8(1):.

3669. Smith Christina, Pisetsky Emily, Wonderlich Stephen, Crosby Ross, Mitchell James, Joiner Thomas, Bardone-Cone Anna, Le Grange Daniel, Klein Marjorie, Crow Scott, Peterson Carol Is childhood trauma associated with lifetime suicide attempts in women with bulimia nervosa?. EATING AND WEIGHT DISORDERS-STUDIES ON ANOREXIA BULIMIA AND OBESITY. 2016;21(2):199-204.

3670. Lampignano Luisa, Donghia Rossella, Sila Annamaria, Bortone Ilaria, Tatoli Rossella, De Nucci Sara, Castellana Fabio, Zupo Roberta, Tirelli Sarah, Giannoccaro Viviana, Guerra Vito, Panza Francesco, Lozupone Madia, Mastronardi Mauro, De Pergola Giovanni, Giannelli Gianluigi, Sardone Rodolfo Mediterranean Diet and Fatty Liver Risk in a Population of Overweight Older Italians: A Propensity Score-Matched Case-Cohort Study. NUTRIENTS. 2022;14(2):.

3671. Jacobi Corinna, Huetter Kristian, Voelker Ulrike, Moebius Katharina, Richter Robert, Trockel Mickey, Bell Megan, Lock James, Taylor C. Efficacy of a Parent-Based, Indicated

Prevention for Anorexia Nervosa: Randomized Controlled Trial. JOURNAL OF MEDICAL INTERNET RESEARCH. 2018;20(12):.

3672. Gubbels Jessica, Kremers Stef, Stafleu Annette, Dagnelie Pieter, Goldbohm R., Vries Nanne, Thijs Carel Diet-related restrictive parenting practices. Impact on dietary intake of 2-year-old children and interactions with child characteristics. APPETITE. 2009;52(2):423-429.

3673. Chen Ximei, Luo Yi-jun, Chen Hong Body Image Victimization Experiences and Disordered Eating Behaviors among Chinese Female Adolescents: The Role of Body Dissatisfaction and Depression. SEX ROLES. 2020;83(7-8):442-452.

3674. Alcaraz-Ibanez Manuel, Paterna Adrian, Griffiths Mark, Demetrovics Zsolt, Sicilia Alvaro Gender-related differences in self-reported problematic exercise symptoms: A systematic review and meta-analysis. PSYCHOLOGY OF SPORT AND EXERCISE. 2022;63():.

3675. Li Wei-hua, Tang Li-rong, Wang Miao, Wang Jia-ni, Guo Ting, He Qiong, He Yu-yang, Lv Zi-ling, Chen Qian, Wang Zheng, Li Xiao-hong, Zhang Peng, Li Zhan-jiang, Wang Zhen-chang Altered gray matter volume and functional connectivity in medial orbitofrontal cortex of bulimia nervosa patients: A combined VBM and FC study. FRONTIERS IN PSYCHIATRY. 2022;13():.

3676. Lee Tien-Jui, Kinzig Kimberly Reprint of ``Repeated adolescent activity-based anorexia influences central estrogen signaling and adulthood anxiety-like behaviors in rats. PHYSIOLOGY \& BEHAVIOR. 2017;178(SI):179-186.

3677. Vander Wal Jillon The relationship between body mass index and unhealthy weight control behaviors among adolescents: The role of family and peer social support. ECONOMICS \& HUMAN BIOLOGY. 2012;10(4, SI):395-404.

3678. Lowinger K, Griffiths RA, Beumont PJV, Scicluna H, Touyz SW Fluid restriction in anorexia nervosa: A neglected symptom or new phenomenon?. INTERNATIONAL JOURNAL OF EATING DISORDERS. 1999;26(4):392-396.

3679. Dryer Rachel, Tyson Graham, Kiernan Michael Bulimia Nervosa: Professional and Lay People's Beliefs About the Causes. AUSTRALIAN PSYCHOLOGIST. 2013;48(5):338-344.

3680. Boutelle KN The use of exposure with response prevention in a male anorexic. JOURNAL OF BEHAVIOR THERAPY AND EXPERIMENTAL PSYCHIATRY. 1998;29(1):79-84.

3681. Luisa Alvarez-Male Maria, Bautista Castano Inmaculada, Serra Majem Lluis PREVALENCE OF EATING DISORDERS IN ADOLESCENTS FROM GRAN CANARIA. NUTRICION HOSPITALARIA. 2015;31(5):2283-2288.

3682. Farrell C, Shafran R, Lee M, Fairburn CG Testing a brief cognitive-behavioural intervention to improve extreme shape concern: A case series. BEHAVIOURAL AND COGNITIVE PSYCHOTHERAPY. 2005;33(2):189-200.

3683. Noakes Timothy Hiding unhealthy heart outcomes in a low-fat diet trial: the Women's Health Initiative Randomized Controlled Dietary Modification Trial finds that postmenopausal women with established coronary heart disease were at increased risk of an adverse outcome if they consumed a low-fat `heart-healthy' diet. OPEN HEART. 2021;8(2):.

3684. Wellington Nadine, Shanmuganathan Meera, Souza Russell, Zulyniak Michael, Azab Sandi, Bloomfield Jonathon, Mell Alicia, Ly Ritchie, Desai Dipika, Anand Sonia, Britz-McKibbin Philip Metabolic Trajectories Following Contrasting Prudent and Western Diets from Food Provisions: Identifying Robust Biomarkers of Short-Term Changes in Habitual Diet. NUTRIENTS. 2019;11(10):.

3685. Calugi Simona, Dalle Grave Riccardo, Marchesini Giulio Longstanding underweight eating disorder: Associated features and treatment outcome. PSYCHOTHERAPY RESEARCH. 2013;23(3, SI):315-323.

3686. Twamley EW, Davis MC The sociocultural model of eating disturbance in young women: The effects of personal attributes and family environment. JOURNAL OF SOCIAL AND CLINICAL PSYCHOLOGY. 1999;18(4):467-489.

3687. Oliveira Leandro, Souza Francis, Silveira Maria Food consumption and weight status in third cycle basic education students and their parents: a cross-sectional study. REVISTA ESPANOLA DE NUTRICION HUMANA Y DIETETICA. 2022;26(4):324-337.

3688. Boynton-Jarrett Renee, Fargnoli Jessica, Suglia Shakira, Zuckerman Barry, Wright Rosalind Association Between Maternal Intimate Partner Violence and Incident Obesity in Preschool-Aged Children Results From the Fragile Families and Child Well-being Study.

ARCHIVES OF PEDIATRICS \& ADOLESCENT MEDICINE. 2010;164(6):540-546.

3689. COOPER PJ, STEERE J A COMPARISON OF 2 PSYCHOLOGICAL TREATMENTS FOR BULIMIA-NERVOSA - IMPLICATIONS FOR MODELS OF MAINTENANCE. BEHAVIOUR RESEARCH AND THERAPY. 1995;33(8):875-885.

3690. Hernandez-Lopez Monica, Quinones-Jimenez Lourdes, Blanco-Romero Alberto, Rodriguez-Valverde Miguel Testing the discrepancy between actual and ideal body image with the Implicit Relational Assessment Procedure (IRAP). JOURNAL OF EATING DISORDERS. 2021;9(1):.

3691. Brandt Stephanie, Schnurbein Julia, Lennerz Belinda, Kohlsdorf Katja, Vollbach Heike, Denzer Christian, Bode Harald, Hebebrand Johannes, Wabitsch Martin Methylphenidate in children with monogenic obesity due to LEPR or MC4R deficiency improves feeling of satiety and reduces BMI-SDS-A case series. PEDIATRIC OBESITY. 2020;15(1):.

3692. Ferreiro Fatima, Wichstrom Lars, Seoane Gloria, Senra Carmen Reciprocal Associations Between Depressive Symptoms and Disordered Eating Among Adolescent Girls and Boys: A Multiwave, Prospective Study. JOURNAL OF ABNORMAL CHILD PSYCHOLOGY. 2014;42(5):803-812.

3693. Delahanty L, Meigs JB, Hayden D, Williamson DA, Nathan DM, Grp DPP Psychological and behavioral correlates of baseline BMI in the Diabetes Prevention Program (DPP). DIABETES CARE. 2002;25(11):1992-1998.

3694. Favieri Francesca, Forte Giuseppe, Casagrande Maria The Executive Functions in Overweight and Obesity: A Systematic Review of Neuropsychological Cross-Sectional and Longitudinal Studies. FRONTIERS IN PSYCHOLOGY. 2019;10():.

3695. Costarelli V., Demerzi M., Stamou D. Disordered eating attitudes in relation to body image and emotional intelligence in young women. JOURNAL OF HUMAN NUTRITION AND DIETETICS. 2009;22(3):239-245.

3696. Heruc Gabriella, Hurst Kim, Casey Anjanette, Fleming Kate, Freeman Jeremy, Fursland Anthea, Hart Susan, Jeffrey Shane, Knight Rachel, Roberton Michelle, Roberts Marion, Shelton Beth, Stiles Garalynne, Sutherland Fiona, Thornton Chris, Wallis Andrew, Wade Tracey ANZAED eating disorder treatment principles and general clinical practice and

training standards. JOURNAL OF EATING DISORDERS. 2020;8(1):.

3697. Thaipisuttikul Papan, Ittasakul Pichai, Waleeprakhon Punjaporn, Wisajun Pattarabhorn, Jullagate Sudawan Psychiatric comorbidities in patients with major depressive disorder. NEUROPSYCHIATRIC DISEASE AND TREATMENT. 2014;10():2097-2103.

3698. Engbers Luuk, Poppel Mireille, Paw Marijke, Mechelen Willem The effects of a controlled worksite environmental intervention on determinants of dietary behavior and self-reported fruit, vegetable and fat intake. BMC PUBLIC HEALTH. 2006;6():.

3699. Tanofsky-Kraff Marian, Sbrocco Tracy, Theim Kelly, Cohen L., Mackey Eleanor, Stice Eric, Henderson Jennifer, McCreight Sarah, Bryant Edny, Stephens Mark Obesity and the US Military Family. OBESITY. 2013;21(11):2205-2220.

3700. McBride Caitlin, Costello Nancy, Ambwani Suman, Wilhite Brcannc, Austin S. Digital Manipulation of Images of Models' Appearance in Advertising: Strategies for Action Through Law and Corporate Social Responsibility Incentives to Protect Public Health. AMERICAN JOURNAL OF LAW \& MEDICINE. 2019;45(1):7-31.

3701. Agras W., Lock James, Brandt Harry, Bryson Susan, Dodge Elizabeth, Halmi Katherine, Jo Booil, Johnson Craig, Kaye Walter, Wilfley Denise, Woodside Blake Comparison of 2 Family Therapies for Adolescent Anorexia Nervosa A Randomized Parallel Trial. JAMA PSYCHIATRY. 2014;71(11):1279-1286.

3702. Lloyd E., Haase Anne, Foster Charlie, Verplanken Bas A systematic review of studies probing longitudinal associations between anxiety and anorexia nervosa. PSYCHIATRY RESEARCH. 2019;276():175-185.

3703. Miyamoto Justin, Berkowitz Zahava, Jones Sherry, Saraiya Mona Indoor Tanning Device Use Among Male High School Students in the United States. JOURNAL OF ADOLESCENT HEALTH. 2012;50(3):308-310.

3704. Byrne Meghan, LeMay-Russell Sarah, Tanofsky-Kraff Marian Loss-of-Control Eating and Obesity Among Children and Adolescents. CURRENT OBESITY REPORTS. 2019;8(1):33-42.

3705. Rumball JS, Lebrun CM Preparticipation physical examination - Selected issues for the female athlete. CLINICAL JOURNAL OF SPORT MEDICINE. 2004;14(3):153-160.

3706. Lyu Wei, Wolinsky Fredric The Onset of ADL Difficulties and Changes in Health-Related Quality of Life. HEALTH AND QUALITY OF LIFE OUTCOMES. 2017;15():.

3707. Girges Christine, Vijiaratnam Nirosen, Wirth Thomas, Tjoakarfa Clarissa, Idaszak Jacqueline, Seneviratne Udaya Seizures triggered by eating - A rare form of reflex epilepsy: A systematic review. SEIZURE-EUROPEAN JOURNAL OF EPILEPSY. 2020;83():21-31.

3708. ROSE UM, BINDELS RJM, JANSEN JWCM, VANOS CH EFFECTS OF CA2+ CHANNEL BLOCKERS, LOW CA2+ MEDIUM AND GLYCINE ON CELL CA2+ AND INJURY IN ANOXIC RABBIT PROXIMAL TUBULES. KIDNEY INTERNATIONAL. 1994;46(1):223-229.

3709. Plimier Colleen, Hewawitharana Sridharshi, Webb Karen, Au Lauren, Neumark-Sztainer Dianne, Ritchie Lorrene Community-level obesity prevention is not associated with dieting behaviours and weight dissatisfaction in children: The Healthy Communities Study. PEDIATRIC OBESITY. 2020;15(4):.

3710. Ahlich Erica, Verzijl Christina, Simon Julia, Schlauch Robert, Rancourt Diana Support for a two-dimensional model of food craving using self-report questionnaire and cue-reactivity methodologies. INTERNATIONAL JOURNAL OF EATING DISORDERS. 2020;53(9):1439-1449.

3711. Stok F., Vet Emely, Wit John, Renner Britta, Ridder Denise Communicating eating-related rules. Suggestions are more effective than restrictions. APPETITE. 2015;86():45-53.

3712. Mas Marine, Brindisi Marie-Claude, Chambaron Stephanie Socio-economic, psychological and environmental factors of obesity: Towards a better understanding to open new perspectives for action. CAHIERS DE NUTRITION ET DE DIETETIQUE. 2021;56(4):208-219.

3713. Chopra Sakshi, Ranjan Piyush, Malhotra Anita, Verma Aditi, Kumari Archana, Sharma K., Sarkar Siddharth, Vikram Naval Perceived risk factors for weight gain, barriers, and facilitators related to weight loss experienced by perimenopausal women: focus group discussion and thematic analysis. MENOPAUSE-THE JOURNAL OF THE NORTH AMERICAN MENOPAUSE SOCIETY. 2022;29(2):219-224.

3714. Conti Chiara, Di Francesco Giulia, Lanzara Roberta, Severo Melania, Fumagalli Luna, Guagnano Maria, Porcelli Piero Alexithymia and binge eating in obese outpatients who are starting a weight-loss program: A structural equation analysis. EUROPEAN EATING DISORDERS REVIEW. 2019;27(6):628-640.

3715. Frydrych AM, Davies GR, McDermott B Eating disorders and oral health: A review of the literature. AUSTRALIAN DENTAL JOURNAL. 2005;50(1):6-15.

3716. Varela Carmen, Andres Ana, Saldana Carmina The behavioral pathway model to overweight and obesity: coping strategies, eating behaviors and body mass index. EATING AND WEIGHT DISORDERS-STUDIES ON ANOREXIA BULIMIA AND OBESITY. 2020;25(5):1277-1283.

3717. Pisetsky Emily, Wonderlich Stephen, Crosby Ross, Peterson Carol, Mitchell James, Engel Scott, Joiner Thomas, Bardone-Cone Anna, Le Grange Daniel, Klein Marjorie, Crow Scott Depression and Personality Traits Associated With Emotion Dysregulation: Correlates of Suicide Attempts in Women with Bulimia Nervosa. EUROPEAN EATING DISORDERS REVIEW. 2015;23(6, SI):537-544.

3718. Bodell Lindsay, Smith April, Holm-Denoma Jill, Gordon Kathryn, Joiner Thomas The impact of perceived social support and negative life events on bulimic symptoms. EATING BEHAVIORS. 2011;12(1):44-48.

3719. Kurz Susanne, Dyck Zoe, Dremmel Daniela, Munsch Simone, Hilbert Anja Early-onset restrictive eating disturbances in primary school boys and girls. EUROPEAN CHILD \& ADOLESCENT PSYCHIATRY. 2015;24(7):779-785.

3720. Athanasoulia-Kaspar Anastasia, Popp Kathrin, Stalla Gunter Neuropsychiatric and metabolic aspects of dopaminergic therapy: perspectives from an endocrinologist and a psychiatrist. ENDOCRINE CONNECTIONS. 2018;7(2):R88-R94.

3721. Sihvola Elina, Keski-Rahkonen Anna, Dick Danielle, Hoek Hans, Raevuori Anu, Rose Richard, Pulkkinen Lea, Marttunen Mauri, Kaprio Jaakko Prospective associations of early-onset Axis I disorders with developing eating disorders. COMPREHENSIVE PSYCHIATRY. 2009;50(1):20-25.

3722. Levinson Cheri, Rodebaugh Thomas Clarifying the prospective relationships between social anxiety and eating disorder symptoms and underlying vulnerabilities. APPETITE.

2016;107():38-46.

3723. Jankauskiene Rasa, Baceviciene Migle, Trinkuniene Laima Examining Body Appreciation and Disordered Eating In Adolescents of Different Sports Practice: Cross-Sectional Study. INTERNATIONAL JOURNAL OF ENVIRONMENTAL RESEARCH AND PUBLIC HEALTH. 2020;17(11):.

3724. Verschueren Margaux, Claes Laurence, Palmeroni Nina, Raemen Leni, Buelens Tinne, Moons Philip, Luyckx Koen Identity Functioning and Eating Disorder Symptomatology: The Role of Cognitive Emotion Regulation Strategies. FRONTIERS IN PSYCHOLOGY. 2021;12():.

3725. Voica Simina, Kling Johanna, Frisen Ann, Piran Niva Disordered eating through the lens of positive psychology: The role of embodiment, self-esteem and identity coherence. BODY IMAGE. 2021;39():103-113.

3726. Sanchez-Carracedo David, Fauquet Jordi, Lopez-Guimera Gemma, Leiva David, Punti Joaquim, Trepat Esther, Pamias Montserrat, Palao Diego The MABIC project: An effectiveness trial for reducing risk factors for eating disorders. BEHAVIOUR RESEARCH AND THERAPY. 2016;77():23-33.

3727. Sala L., Mirabel-Sarron C., Pham-Scottez A., Blanchet A., Rouillon F., Gorwood P. Body dissatisfaction is improved but the ideal silhouette is unchanged during weight recovery in anorexia nervosa female inpatients. EATING AND WEIGHT DISORDERS-STUDIES ON ANOREXIA BULIMIA AND OBESITY. 2012;17(2):E109-E115.

3728. Kotadia Hiral, Maheshwari Ankita Anorexia nervosa in a preadolescent male. JOURNAL OF INDIAN ASSOCIATION FOR CHILD AND ADOLESCENT MENTAL HEALTH. 2020;16(4):164-169.

3729. Moessner Markus, Bauer Stephanie Maximizing the public health impact of eating disorder services: A simulation study. INTERNATIONAL JOURNAL OF EATING DISORDERS. 2017;50(12):1378-1384.

3730. Thompson-Brenner H, Glass S, Westen D A multidimensional meta-analysis of psychotherapy for bulimia nervosa. CLINICAL PSYCHOLOGY-SCIENCE AND PRACTICE. 2003;10(3):269-287.

3731. Villaecija Joaquin, Luque Barbara, Martinez Sandra, Castillo-Mayen Rosario, Cuadrado Esther, Dominguez-Escribano Marta, Tabernero Carmen Perceived social support and healthy eating self efficacy on the well-being of children and adolescents. REVISTA IBEROAMERICANA DE PSICOLOGIA Y SALUD. 2022;13(1):56-72.

3732. Sehm Marie, Warschburger Petra The dual-pathway model of binge eating: Is there a need for modification?. APPETITE. 2017;114():137-145.

3733. Guzman-Rodriguez Sergio, Chavez-Reyes Jesus, Vazquez-Leon Priscila, Soriano-Ursua Marvin, Rosalez Melvin, Allende Gonzalo, Marichal-Cancino Bruno 1-Boc-Piperidine-4-Carboxaldehyde Prevents Binge-Eating Behaviour and Anxiety in Rats. PHARMACOLOGY. 2021;106(5-6):305-315.

3734. Heberlein A., Bleich S., Kornhuber J., Hillemacher T. Pharmacological treatment options for prevention of alcohol relapse. FORTSCHRITTE DER NEUROLOGIE PSYCHIATRIE. 2008;76(7):421-428.

3735. Austin S., Liu Selena, Tefft Nathan Could a tax on unhealthy products sold for weight loss reduce consumer use? A novel estimation of potential taxation effects. PREVENTIVE MEDICINE. 2018;114():39-46.

3736. Shearer Annie, Russon Jody, Herres Joanna, Atte Tita, Kodish Tamar, Diamond Guy The relationship between disordered eating and sexuality amongst adolescents and young adults. EATING BEHAVIORS. 2015;19():115-119.

3737. Figueiredo Rejane, Simola-Strom Sabina, Isomaa Rasmus, Weiderpass Elisabete Body dissatisfaction and disordered eating symptoms in Finnish preadolescents. EATING DISORDERS. 2019;27(1):34-51.

3738. Zeigler-Hill Virgil, Noser Amy Will I Ever Think I'm Thin Enough? A Moderated Mediation Study of Women's Contingent Self-Esteem, Body Image Discrepancies, and Disordered Eating. PSYCHOLOGY OF WOMEN QUARTERLY. 2015;39(1):109-118.

3739. Berge Jerica, Loth Katie, Hanson Carrie, Croll-Lampert Jillian, Neumark-Sztainer Dianne Family life cycle transitions and the onset of eating disorders: a retrospective grounded theory approach. JOURNAL OF CLINICAL NURSING. 2012;21(9-10):1355-1363.

3740. Skot Lotte, Mejldal Anna, Guala Maria, Stoving Rene, Ascone Leonie, Stenager Elsebeth, Lichtenstein Mia, Mellentin Angelina Eating disorders and subsequent risk of substance use disorders involving illicit drugs: a Danish nationwide register-based cohort study. SOCIAL PSYCHIATRY AND PSYCHIATRIC EPIDEMIOLOGY. 2022;57(4):695-708.

3741. Cella Stefania, Iannaccone Mara, Cotrufo Paolo Does body shame mediate the relationship between parental bonding, self-esteem, maladaptive perfectionism, body mass index and eating disorders? A structural equation model. EATING AND WEIGHT DISORDERS-STUDIES ON ANOREXIA BULIMIA AND OBESITY. 2020;25(3):667-678.

3742. Cortes-Garcia L., McLaren V., Vanwoerden S., Sharp C. Attachment, mentalizing, and eating disorder symptoms in adolescent psychiatric inpatients and healthy controls: a test of a mediational model. EATING AND WEIGHT DISORDERS-STUDIES ON ANOREXIA BULIMIA AND OBESITY. 2021;26(4):1159-1168.

3743. Eisler I, Dare C, Russell GFM, Szmukler G, leGrange D, Dodge E Family and individual therapy in anorexia nervosa - A 5-year follow-up. ARCHIVES OF GENERAL PSYCHIATRY. 1997;54(11):1025-1030.

3744. Jia Jenny, Levy Douglas, McCurley Jessica, Anderson Emma, Gelsomin Emily, Porneala Bianca, Thorndike Anne Health Literacy, Numeracy, and Health Promotion: A Secondary Analysis of the Choosewell 365 Workplace Trial. AMERICAN JOURNAL OF PREVENTIVE MEDICINE. 2022;63(1):93-101.

3745. Bonomi Amy, Nemeth Julianna, Altenburger Lauren, Anderson Melissa, Snyder Anastasia, Dotto Irma Fiction or Not? Fifty Shades is Associated with Health Risks in Adolescent and Young Adult Females. JOURNAL OF WOMENS HEALTH. 2014;23(9):720+.

3746. Kaufman Francine Consequences of weight gain associated with insulin therapy in adolescents. ENDOCRINOLOGIST. 2006;16(3):155-162.

3747. Olson KayLoni, Neiberg Rebecca, Tate Deborah, Garcia Katelyn, Gorin Amy, Lewis Cora, Unick Jessica, Wing Rena Weight and Shape Concern Impacts Weight Gain Prevention in the SNAP Trial: Implications for Tailoring Intervention Delivery. OBESITY. 2018;26(8):1270-1276.

3748. McNulty KY, Adams CH, Anderson JM, Affenito SG Development and validation of a screening tool to identify eating disorders in female athletes. JOURNAL OF THE AMERICAN

DIETETIC ASSOCIATION. 2001;101(8):886-892.

3749. McCarvill Rachael, Weaver Kathryn Primary care of female adolescents with type 1 diabetes mellitus and disordered eating. JOURNAL OF ADVANCED NURSING. 2014;70(9):2005-2018.

3750. Ventura Vera, Cavaliere Alessia, Ianno Beatrice \#Socialfood: Virtuous or vicious? A systematic review. TRENDS IN FOOD SCIENCE \& TECHNOLOGY. 2021;110():674-686.

3751. Nguyen Tuyen, Trat Tiffany, Tieu Ngoc, Vu Linda, Sokal-Gutierrez Karen Key Informants' Perspectives on Childhood Obesity in Vietnam: A Qualitative Study. MATERNAL AND CHILD HEALTH JOURNAL. 2022;26(9):1811-1819.

3752. Chan Joshua, Caesar Michelle, Mann Amandeep, Koh-Bell Alex, Richardson Michael, Johnson Caitlin, Kapp Daniel, Chan John The Role of Diet Compared to Physical Activity on Women's Cancer Mortality: Results From the Third National Health and Nutrition Examination Survey. FRONTIERS IN PUBLIC HEALTH. 2022;10():.

3753. Sim Leslie, Peterson Carol The peril and promise of sensitivity in eating disorders. INTERNATIONAL JOURNAL OF EATING DISORDERS. 2021;54(11):2046-2056.

3754. Lilford Philippa, Hughes Julian Epidemiology and mental illness in old age. BJPSYCH ADVANCES. 2020;26(2):92-103.

3755. Lebow Jocelyn, Chuy Jeffrey, Cedermark Kyle, Cook Katlyn, Sim Leslie The Development or Exacerbation of Eating Disorder Symptoms After Topiramate Initiation. PEDIATRICS. 2015;135(5):E1312-E1316.

3756. Weintraub Daniel Dopamine and Impulse Control Disorders in Parkinson's Disease. ANNALS OF NEUROLOGY. 2008;64(6, S):S93-S100.

3757. GRIFFITHS RA, HADZIPAVLOVIC D, CHANNONLITTLE L A CONTROLLED EVALUATION OF HYPNOBEHAVIOURAL TREATMENT FOR BULIMIA-NERVOSA - IMMEDIATE PRE-POST-TREATMENT EFFECTS. EUROPEAN EATING DISORDERS REVIEW. 1994;2(4):202-220.

3758. Frank E, Wright EH, Serdula MK, Elon LK, Baldwin G Personal and professional nutrition-related practices of US female physicians. AMERICAN JOURNAL OF CLINICAL NUTRITION. 2002;75(2):326-332.

3759. Wisting Line, Bang Lasse, Skrivarhaug Torild, Dahl-Jorgensen Knut, Ro Oyvind Adolescents with Type 1 Diabetes - The Impact of Gender, Age, and Health-Related Functioning on Eating Disorder Psychopathology. PLOS ONE. 2015;10(11):.

3760. Carr Jacquelyn, Kleiman Susan, Bulik Cynthia, Bulik-Sullivan Emily, Carroll Ian Can attention to the intestinal microbiota improve understanding and treatment of anorexia nervosa?. EXPERT REVIEW OF GASTROENTEROLOGY \& HEPATOLOGY. 2016;10(5):565-569.

3761. Stice Eric, Rohde Paul, Shaw Heather, Gau Jeff Clinician-Led, Peer-Led, and Internet-Delivered Dissonance-Based Eating Disorder Prevention Programs: Effectiveness of These Delivery Modalities Through 4-Year Follow-Up. JOURNAL OF CONSULTING AND CLINICAL PSYCHOLOGY. 2020;88(5):481-494.

3762. Bardone-Cone Anna, Hunt Rowan, Watson Hunna An Overview of Conceptualizations of Eating Disorder Recovery, Recent Findings, and Future Directions. CURRENT PSYCHIATRY REPORTS. 2018;20(9):.

3763. Stice Eric, Ng Janet, Shaw Heather Risk factors and prodromal eating pathology. JOURNAL OF CHILD PSYCHOLOGY AND PSYCHIATRY. 2010;51(4):518-525.

3764. Kaap-Deeder Jolene, Vansteenkiste Maarten, Soenens Bart, Verstuyf Joke, Boone Liesbet, Smets Jos Fostering Self-Endorsed Motivation to Change in Patients with an Eating Disorder: The Role of Perceived Autonomy Support and Psychological Need Satisfaction. INTERNATIONAL JOURNAL OF EATING DISORDERS. 2014;47(6):585-600.

3765. Aydin Volkan, Vizdiklar Caner, Akici Ahmet, Akman Mehmet, Yavuz Dilek, Altikardes Zehra, Kucukguzel S., Topcu Mumine, Aysevinc Berrin, Fak Ali Evaluation of health-related knowledge, attitudes, and behaviors of undergraduate students by cardiovascular risk factors. PRIMARY HEALTH CARE RESEARCH AND DEVELOPMENT. 2021;22():.

3766. Vollert Bianka, Bloh Paula, Eiterich Nadine, Beintner Ina, Huetter Kristian, Taylor Craig, Jacobi Corinna Recruiting participants to an Internet-based eating disorder prevention trial: Impact of the recruitment strategy on symptom severity and program

utilization. INTERNATIONAL JOURNAL OF EATING DISORDERS. 2020;53(5):476-484.

3767. Balciuniene Vaiva, Jankauskiene Rasa, Baceviciene Migle Effect of an education and mindfulness-based physical activity intervention for the promotion of positive body image in Lithuanian female students. EATING AND WEIGHT DISORDERS-STUDIES ON ANOREXIA BULIMIA AND OBESITY. 2022;27(2):563-577.

3768. Maia Emanuella, Mendes Larissa, Pimenta Adriano, Levy Renata, Claro Rafael Cluster of risk and protective factors for obesity among Brazilian adolescents. INTERNATIONAL JOURNAL OF PUBLIC HEALTH. 2018;63(4):481-490.

3769. Toledo-Hinarejos M., Leon-Zarceno E., Canton-Chirivella E. THE ROLE OF SPORT IN DEVELOPMENTOF EATING DISORDERS: A REVIEW OF THE MOST RECENT LITERATURE (2015-2020). JOURNAL OF SPORT AND HEALTH RESEARCH. 2023;15(1):1-16.

3770. Perkins SJ, Keville S, Schmidt U, Chalder T Eating disorders and irritable bowel syndrome: is there a link?. JOURNAL OF PSYCHOSOMATIC RESEARCH. 2005;59(2):57-64.

3771. McCrone S, Dennis K, Tomoyasu N, Carroll J A profile of early versus late onset of obesity in postmenopausal women. JOURNAL OF WOMENS HEALTH \& GENDER-BASED MEDICINE. 2000;9(9):1007-1013.

3772. McClelland Jessica, Robinson Lauren, Potterton Rachel, Mountford Victoria, Schmidt Ulrike Symptom trajectories into eating disorders: A systematic review of longitudinal, nonclinical studies in children/adolescents. EUROPEAN PSYCHIATRY. 2020;63(1):.

3773. Dakanalis Antonios, Timko C., Favagrossa Laura, Riva Giuseppe, Zanetti M., Clerici Massimo Why Do Only a Minority of Men Report Severe Levels of Eating Disorder Symptomatology, When so Many Report Substantial Body Dissatisfaction? Examination of Exacerbating Factors. EATING DISORDERS. 2014;22(4):292-305.

3774. Pinna Federica, Sardu Claudia, Orru Walter, Velluzzi Fernanda, Loviselli Andrea, Contu Paolo, Carpiniello Bernardo Psychopathology, psychosocial factors and obesity. RIVISTA DI PSICHIATRIA. 2016;51(1):30-36.

3775. McVey GL, Pepler D, Davis R, Flett GL, Abdolell M Risk and protective factors associated with disordered eating during early adolescence. JOURNAL OF EARLY

ADOLESCENCE. 2002;22(1):75-95.

3776. Welch Elisabeth, Birgegard Andreas, Parling Thomas, Ghaderi Ata Eating disorder examination questionnaire and clinical impairment assessment questionnaire: General population and clinical norms for young adult women in Sweden. BEHAVIOUR RESEARCH AND THERAPY. 2011;49(2):85-91.

3777. Micali Nadia, De Stavola Bianca, Ploubidis George, Simonoff Emily, Treasure Janet The effects of maternal eating disorders on offspring childhood and early adolescent psychiatric disorders. INTERNATIONAL JOURNAL OF EATING DISORDERS. 2014;47(4):385-393.

3778. Kraak Vivica, Story Mary, Wartella Ellen, Ginter Jaya Industry Progress to Market a Healthful Diet to American Children and Adolescents. AMERICAN JOURNAL OF PREVENTIVE MEDICINE. 2011;41(3):322-333.

3779. Rahe Corinna, Unrath Michael, Berger Klaus Dietary patterns and the risk of depression in adults: a systematic review of observational studies. EUROPEAN JOURNAL OF NUTRITION. 2014;53(4):997-1013.

3780. Lewis-Smith Helena, Bray Isabelle, Salmon Debra, Slater Amy Prospective Pathways to Depressive Symptoms and Disordered Eating in Adolescence:A 7-Year Longitudinal Cohort Study. JOURNAL OF YOUTH AND ADOLESCENCE. 2020;49(10):2060-2074.

3781. Stice E, Presnell K, Shaw H, Rohde P Psychological and behavioral risk factors for obesity onset in adolescent girls: A prospective study. JOURNAL OF CONSULTING AND CLINICAL PSYCHOLOGY. 2005;73(2):195-202.

3782. Favaro Angela, Caregaro Loyenza, Tenconi Elena, Bosello Romina, Santonastaso Paolo Time Trends in Age at Onset of Anorexia Nervosa and Bulimia Nervosa. JOURNAL OF CLINICAL PSYCHIATRY. 2009;70(12):1715-1721.

3783. Melbye Elisabeth, Hansen Havard Promotion and Prevention Focused Feeding Strategies: Exploring the Effects on Healthy and Unhealthy Child Eating. BIOMED RESEARCH INTERNATIONAL. 2015;2015():.

3784. Doan Stacey, Venkatesh Shruthi, Mendiola Isabel, Smiley Patricia, Schmolze Daniel Stressed out and fed up: The effect of stress on maternal feeding behaviors and the

moderating role of executive function. APPETITE. 2022;168():.

3785. Yee Andrew, Lwin May, Ho Shirley The influence of parental practices on child promotive and preventive food consumption behaviors: a systematic review and meta-analysis. INTERNATIONAL JOURNAL OF BEHAVIORAL NUTRITION AND PHYSICAL ACTIVITY. 2017;14():.

3786. Neale Josephine, Hudson Lee Anorexia nervosa in adolescents. BRITISH JOURNAL OF HOSPITAL MEDICINE. 2020;81(6):.

3787. Filaire E., Larue J., Rouveix M. Eating Behaviours in Relation to Emotional Intelligence. INTERNATIONAL JOURNAL OF SPORTS MEDICINE. 2011;32(4):309-315.

3788. Marta-Simoes Joana, Tylka Tracy, Ferreira Claudia Adolescent girls' body appreciation: influences of compassion and social safeness, and association with disordered eating. EATING AND WEIGHT DISORDERS-STUDIES ON ANOREXIA BULIMIA AND OBESITY. 2022;27(4):1359-1366.

3789. Schneider Catharina, Rollitz Laura, Voracek Martin, Hennig-Fast Kristina Biological, Psychological, and Sociocultural Factors Contributing to the Drive for Muscularity in Weight-Training Men. FRONTIERS IN PSYCHOLOGY. 2016;7():.

3790. Kim Hyunju, Lee Kyueun, Rebholz Casey, Kim Jihye Association between unhealthy plant-based diets and the metabolic syndrome in adult men and women: a population-based study in South Korea. BRITISH JOURNAL OF NUTRITION. 2021;125(5):577-590.

3791. Casado-Aranda Luis-Alberto, Laan Nynke, Sanchez-Fernandez Juan Neural activity in self-related brain regions in response to tailored nutritional messages predicts dietary change. APPETITE. 2022;170():.

3792. Ciao Anna, Loth Katie, Neumark-Sztainer Dianne Preventing Eating Disorder Pathology: Common and Unique Features of Successful Eating Disorders Prevention Programs. CURRENT PSYCHIATRY REPORTS. 2014;16(7):.

3793. Ronto Rimante, Wu Jason, Singh Gitanjali The global nutrition transition: trends, disease burdens and policy interventions. PUBLIC HEALTH NUTRITION. 2018;21(12):2267-

2270.

3794. Verrotti A., Loiacono G., Di Sabatino F., Zaccara G. The adverse event profile of zonisamide: a meta-analysis. ACTA NEUROLOGICA SCANDINAVICA. 2013;128(5):297-304.

3795. Oliveira Sara, Trindade Ines, Ferreira Claudia The buffer effect of body compassion on the association between shame and body and eating difficulties. APPETITE. 2018;125():118-123.

3796. Rodgers Rachel, Wertheim Eleanor, Damiano Stephanie, Paxton Susan Maternal influences on body image and eating concerns among 7-and 8-year-old boys and girls: Cross-sectional and prospective relations. INTERNATIONAL JOURNAL OF EATING DISORDERS. 2020;53(1):79-84.

3797. Simone Melissa, Askew Autumn, Lust Katherine, Eisenberg Marla, Pisetsky Emily Disparities in self-reported eating disorders and academic impairment in sexual and gender minority college students relative to their heterosexual and cisgender peers. INTERNATIONAL JOURNAL OF EATING DISORDERS. 2020;53(4):513-524.

3798. Troop Nicholas Social Rank, Rank-Related Life Events and Eating Pathology. EUROPEAN EATING DISORDERS REVIEW. 2016;24(1):75-77.

3799. Wiederman MW, Pryor T Body dissatisfaction and sexuality among women with bulimia nervosa. INTERNATIONAL JOURNAL OF EATING DISORDERS. 1997;21(4):361-365.

3800. Cecchini Andrea, Biscetti Federico, Rando Maria, Nardella Elisabetta, Pecorini Giovanni, Eraso Luis, Dimuzio Paul, Gasbarrini Antonio, Massetti Massimo, Flex Andrea Dietary Risk Factors and Eating Behaviors in Peripheral Arterial Disease (PAD). INTERNATIONAL JOURNAL OF MOLECULAR SCIENCES. 2022;23(18):.

3801. Auchincloss Amy, Riolo Rick, Brown Daniel, Cook Jeremy, Roux Ana An Agent-Based Model of Income Inequalities in Diet in the Context of Residential Segregation. AMERICAN JOURNAL OF PREVENTIVE MEDICINE. 2011;40(3):303-311.

3802. Huang Christina, Reisch Lucia, Gwozdz Wencke, Molnar Denes, Konstabel Kenn, Michels Nathalie, Tornaritis Michalis, Eiben Gabriele, Siani Alfonso, Fernandez-Alvira Juan, Ahrens Wolfgang, Pigeot Iris, Lissner Lauren, Consortium IDEFICS Pester power and its

consequences: do European children's food purchasing requests relate to diet and weight outcomes?. PUBLIC HEALTH NUTRITION. 2016;19(13):2393-2403.

3803. Musaad Salma, Donovan Sharon, Fiese Barbara, Team STRONG Parental perception of child weight in the first two years-of-life: a potential link between infant feeding and preschoolers' diet. APPETITE. 2015;91():90-100.

3804. Simpson Susan, Azam Fatima, Brown SiennaMarisa, Hronis Anastasia, Brockman Robert The impact of personality disorders and personality traits on psychotherapy treatment outcome of eating disorders: A systematic review. PERSONALITY AND MENTAL HEALTH. 2022;16(3):217-234.

3805. Kilicaslan Fethiye, Bakirci Busra Early Onset Atypical Anorexia Nervosa Case Associated with Social Media in Pandemic Conditions. PSYCHIATRY AND BEHAVIORAL SCIENCES. 2021;11(4):288-290.

3806. Eisenberg Marla, Puhl Rebecca, Areba Eunice, Neumark-Sztainer Dianne Family weight teasing, ethnicity and acculturation: Associations with well-being among Latinx, Hmong, and Somali Adolescents. JOURNAL OF PSYCHOSOMATIC RESEARCH. 2019;122():88-93.

3807. Pinus Uri, Canetti Laura, Bonne Omer, Bachar Eytan Selflessness as a predictor of remission from an eating disorder: 1-4 year outcomes from an adolescent day-care unit. EATING AND WEIGHT DISORDERS-STUDIES ON ANOREXIA BULIMIA AND OBESITY. 2019;24(4):777-786.

3808. Kothari Radha, Rosinska Magda, Treasure Janet, Micali Nadia The Early Cognitive Development of Children at High Risk of Developing an Eating Disorder. EUROPEAN EATING DISORDERS REVIEW. 2014;22(2):152-156.

3809. Telleus Gry, Lauritsen Marlene, Rodrigo-Domingo Maria Prevalence of Various Traumatic Events Including Sexual Trauma in a Clinical Sample of Patients With an Eating Disorder. FRONTIERS IN PSYCHOLOGY. 2021;12():.

3810. Cunningham-Sabo Leslie, Bauer Mark, Pareo Shirley, Phillips-Benally Shirleen, Roanhorse Julia, Garcia Linda Qualitative Investigation of Factors Contributing to Effective Nutrition Education for Navajo Families. MATERNAL AND CHILD HEALTH JOURNAL.

2008;12(1):S68-S75.

3811. Hobart JA, Smucker DR The female athlete triad. AMERICAN FAMILY PHYSICIAN. 2000;61(11):3357-3364.

3812. Wang Chen How does incidental curiosity affect consumers' unhealthy eating?. JOURNAL OF CONSUMER MARKETING. 2019;36(6):784-793.

3813. Hailey David, Roine Risto, Ohinmaa Arto The Effectiveness of Telemental Health Applications: A Review. CANADIAN JOURNAL OF PSYCHIATRY-REVUE CANADIENNE DE PSYCHIATRIE. 2008;53(11):769-778.

3814. Blasczyk-Schiep Sybilla, Adamczewska Kaja, Sokola Kaja Subclinical eating disorder symptoms and positive vs. negative affect in high school students: the mediating role of self-regulation. CURRENT ISSUES IN PERSONALITY PSYCHOLOGY. 2019;7(2):120-131.

3815. Poynter Brittany, Hunter Jon, Coverdale John, Kempinsky Cheryl Hard to swallow: a systematic review of deliberate foreign body ingestion. GENERAL HOSPITAL PSYCHIATRY. 2011;33(5):518-524.

3816. Poracova Janka, Uher Ivan, Vaskova Hedviga, Kimakova Tatiana, Konecna Maria, Blascakova Marta, Sedlak Vincent Effectiveness of Adherence to a Mediterranean Diet in the Management of Overweight Women: The Prospective Interventional Cohort Study. INTERNATIONAL JOURNAL OF ENVIRONMENTAL RESEARCH AND PUBLIC HEALTH. 2022;19(23):.

3817. Scavone Cristina, Stelitano Barbara, Rafaniello Concetta, Rossi Francesco, Sportiello Liberata, Capuano Annalisa Drugs-Induced Pathological Gambling: An Analysis of Italian Spontaneous Reporting System. JOURNAL OF GAMBLING STUDIES. 2020;36(1):85-96.

3818. Valle M., Martin-Payo R., Cuesta-Briand B., Lana A. Impact of two nurse-led interventions targeting diet among breast cancer survivors: Results from a randomized controlled trial. EUROPEAN JOURNAL OF CANCER CARE. 2018;27(4):.

3819. Anderson Lisa, Berg Hannah, Brown Tiffany, Menzel Jessie, Reilly Erin The Role of Disgust in Eating Disorders. CURRENT PSYCHIATRY REPORTS. 2021;23(2):.

3820. Kurian M., Schmitt-Mechelke T., Korff C., Delavelle J., Landis T., Seeck M. ``Gourmand syndrome{''} in a child with pharmacoresistant epilepsy. EPILEPSY \& BEHAVIOR. 2008;13(2):413-415.

3821. Linnaranta Outi, Bourguignon Clement, Crescenzi Olivia, Sibthorpe Duncan, Buyukkurt Asli, Steiger Howard, Storch Kai-Florian Late and Instable Sleep Phasing is Associated With Irregular Eating Patterns in Eating Disorders. ANNALS OF BEHAVIORAL MEDICINE. 2020;54(9):680-690.

3822. Aardoom Jiska, Dingemans Alexandra, Fokkema Marjolein, Spinhoven Philip, Van Furth Eric Moderators of change in an Internet-based intervention for eating disorders with different levels of therapist support: What works for whom?. BEHAVIOUR RESEARCH AND THERAPY. 2017;89():66-74.

3823. Pietrabissa Giada, Rossi Alessandro, Gaudenzi Michela, Bertuzzi Venessa, Tagliagambe Andrea, Volpi Clarissa, Manzoni Gian, Cattivelli Roberto, Mannarini Stefania, Castelnuovo Gianluca, Simpson Susan Drunkorexia: empirical investigation and analysis of the characteristics of the phenomenon in an Italian sample of adolescents and young adults. PSYCHOLOGY SOCIETY \& EDUCATION. 2018;10(3):285-299.

3824. Keski-Rahkonen A., Hoek H., Linna M., Raevuori A., Sihvola E., Bulik C., Rissanen A., Kaprio J. Incidence and outcomes of bulimia nervosa: a nationwide population-based study. PSYCHOLOGICAL MEDICINE. 2009;39(5):823-831.

3825. Geist R, Heinmaa M, Stephens D, Davis R, Katzman DK Comparison of family therapy and family group psychoeducation in adolescents with anorexia nervosa. CANADIAN JOURNAL OF PSYCHIATRY-REVUE CANADIENNE DE PSYCHIATRIE. 2000;45(2):173-178.

3826. Walcott DD, Pratt HD, Patel DR Adolescents and eating disorders: Gender, racial, ethnic, sociocultural, and socioeconomic issues. JOURNAL OF ADOLESCENT RESEARCH. 2003;18(3):223-243.

3827. Baechle Christina, Lange Karin, Stahl-Pehe Anna, Castillo Katty, Scheuing Nicole, Holl Reinhard, Giani Guido, Rosenbauer Joachim Symptoms of Eating Disorders and Depression in Emerging Adults with Early-Onset, Long-Duration Type 1 Diabetes and Their Association with Metabolic Control. PLOS ONE. 2015;10(6):.

3828. Argyrides Marios, Kkeli Natalie Predictive Factors of Disordered Eating and Body Image Satisfaction in Cyprus. INTERNATIONAL JOURNAL OF EATING DISORDERS. 2015;48(4):431-435.

3829. Bea Paszthy, Maria Torzsok-Sonnevend The challenging heterogenity of anorexia nervosa Neurobiological risk factors and possible endophenotypes. ORVOSI HETILAP. 2014;155(4):124-131.

3830. Rychkova Lyubov, Pogodina Anna, Ayurova Zhanna, Berdina Olga Risk Factors for Obesity in Adolescents Living in Rural Areas of Buryatia: A Case-Control Study. INTERNATIONAL JOURNAL OF BIOMEDICINE. 2019;9(2):190-195.

3831. Tozzi F, Sullivan PF, Fear JL, McKenzie J, Bulik CM Causes and recovery in anorexia nervosa: The patient's perspective. INTERNATIONAL JOURNAL OF EATING DISORDERS. 2003;33(2):143-154.

3832. Czepiel Diana, Koopman Hendricus Does physical appearance perfectionism predict disordered dieting?. CURRENT PSYCHOLOGY. 2023;42(1):24-34.

3833. Topino Eleonora, Gori Alessio, Cacioppo Marco Alexithymia, Dissociation, and Family Functioning in a Sample of Online Gamblers: A Moderated Mediation Study. INTERNATIONAL JOURNAL OF ENVIRONMENTAL RESEARCH AND PUBLIC HEALTH. 2021;18(24):.

3834. Bufferd Sara, Levinson Cheri, Olino Thomas, Dougherty Lea, Dyson Margaret, Carlson Gabrielle, Klein Daniel Temperament and psychopathology in early childhood predict body dissatisfaction and eating disorder symptoms in adolescence. BEHAVIOUR RESEARCH AND THERAPY. 2022;151():.

3835. Maras Danijela, Obeid Nicole, Flament Martine, Buchholz Annick, Henderson Katherine, Gick Mary, Goldfield Gary Attachment Style and Obesity: Disordered Eating Behaviors as a Mediator in a Community Sample of Canadian Youth. JOURNAL OF DEVELOPMENTAL AND BEHAVIORAL PEDIATRICS. 2016;37(9):762-770.

3836. Stoving Rene, Andries Alin, Brixen Kim, Bilenberg Niels, Lichtenstein Mia, Horder Kirsten Purging behavior in anorexia nervosa and eating disorder not otherwise specified: A retrospective cohort study. PSYCHIATRY RESEARCH. 2012;198(2):253-258.

3837. Guo Mengxi, Lou Yiling, Zhang Ning Consideration of future consequences and self-control mediate the impact of time perspectives on self-rated health and engagement in healthy lifestyles among young adults. CURRENT PSYCHOLOGY. 2022;():.

3838. Iannaccone Mara, D'Olimpio Francesca, Cella Stefania, Cotrufo Paolo Self-esteem, body shame and eating disorder risk in obese and normal weight adolescents: A mediation model. EATING BEHAVIORS. 2016;21():80-83.

3839. Alvarenga Marle, Lourenco Barbara, Philippi Sonia, Scagliusi Fernanda Disordered eating among Brazilian female college students. CADERNOS DE SAUDE PUBLICA. 2013;29(5):879-888.

3840. Mazzeo S., Mitchell K., Bulik C., Aggen S., Kendler K., Neale M. A twin study of specific bulimia nervosa symptoms. PSYCHOLOGICAL MEDICINE. 2010;40(7):1203-1213.

3841. Lopez-Gil Jose, Garcia-Hermoso Antonio, Smith Lee, Firth Joseph, Trott Mike, Mesas Arthur, Jimenez-Lopez Estela, Gutierrez-Espinoza Hector, Tarraga-Lopez Pedro, Victoria-Montesinos Desiree Global Proportion of Disordered Eating in Children and Adolescents A Systematic Review and Meta-analysis. JAMA PEDIATRICS. 2023;177(4):363-372.

3842. Saboo Banshi, Misra Anoop, Kalra Sanjay, Mohan V., Aravind S., Joshi Shashank, Chowdhury Subhankar, Sahay Rakesh, Kesavadev Jothydev, John Mathew, Kapoor Nitin, Das Sambit, Krishnan Dharini, Salis Sheryl, Fiber Decode Role and importance of high fiber in diabetes management in India. DIABETES \& METABOLIC SYNDROME-CLINICAL RESEARCH \& REVIEWS. 2022;16(5):.

3843. Ames Gretchen, Koball Afton, Clark Matthew Behavioral Interventions to Attenuate Driven Overeating and Weight Regain After Bariatric Surgery. FRONTIERS IN ENDOCRINOLOGY. 2022;13():.

3844. Sullivan Grace, Gervais Sarah, Brock Rebecca, Stoltenberg Scott Social Responsiveness and Objectification: The Moderating Roles of Serotonin Transporter and Serotonin Receptor 2A Genotypes in an Objectification Theory Model of Disordered Eating. SEX ROLES. 2020;82(9-10):584-599.

3845. Hermont Ana, Oliveira Patricia, Martins Carolina, Paiva Saul, Pordeus Isabela, Auad Sheyla Tooth Erosion and Eating Disorders: A Systematic Review and Meta-Analysis. PLOS

ONE. 2014;9(11):.

3846. Campbell Karen, Crawford David, Salmon Jo, Carver Alison, Garnett Sarah, Baur Louise Associations between the home food environment and obesity-promoting eating behaviors in adolescence. OBESITY. 2007;15(3):719-730.

3847. Pacitti Francesca, Maraone Annalisa, Zazzara Francesca, Biondi Massimo, Caredda Maria Stress and Night Eating Syndrome: a comparison study between a sample of psychiatric outpatients and healthy subjects. RIVISTA DI PSICHIATRIA. 2011;46(3):195-202.

3848. Konings Gerdy, Drukker Marjan, Mulkens Sandra, Severeijns Ruud, Os Jim, Ponds Rudolf Postsurgical Compliance and Eating Behavior 5 Years After Surgery. BARIATRIC SURGICAL PRACTICE AND PATIENT CARE. 2020;15(3):148-154.

3849. Moreno Gonzalez Miriam, Ortiz Viveros Godeleva Eating Disorder and its Relationship with Body Image and Self-Esteem in Adolescents. TERAPIA PSICOLOGICA. 2009;27(2):181-189.

3850. Yeo Michele, Hughes Elizabeth Eating disorders Early identification in general practice. AUSTRALIAN FAMILY PHYSICIAN. 2011;40(3):108-111.

3851. Pamuk Omer, Makaraci Yucel, Ozer Omer, Soslu Recep Examination of Eating Attitudes of Universities' Sports Department Students In Terms of Individual and Team Sports. INTERNATIONAL JOURNAL OF APPLIED EXERCISE PHYSIOLOGY. 2020;9(12):43-49.

3852. Vignau Jean Anorexia nervosa, an addiction to starving cues. CORRESPONDANCES EN METABOLISMES HORMONES DIABETES ET NUTRITION. 2016;20(8):228-233.

3853. Butwicka Agnieszka, Olen Ola, Larsson Henrik, Halfvarson Jonas, Almqvist Catarina, Lichtenstein Paul, Serlachius Eva, Frisen Louise, Ludvigsson Jonas Association of Childhood-Onset Inflammatory Bowel Disease With Risk of Psychiatric Disorders and Suicide Attempt. JAMA PEDIATRICS. 2019;173(10):969-978.

3854. Ghosh Debabrata, Petrecca Abigail, Khuhro Abdul Sleep-Related Eating Disorder (SRED): Paradoxical Effect of Clonazepam. JOURNAL OF CLINICAL SLEEP MEDICINE.

2018;14(7):1261-1263.

3855. Dyson Pamela, Anthony Denis, Fenton Brenda, Stevens Denise, Champagne Beatriz, Li Li-Ming, Lv Jun, Hernandez Jorge, Thankappan K., Matthews David, Collaboration CIH Successful Up-Scaled Population Interventions to Reduce Risk Factors for Non-Communicable Disease in Adults: Results from the International Community Interventions for Health (CIH) Project in China, India and Mexico. PLOS ONE. 2015;10(4):.

3856. Daly Suzanne Secure Body Attachment and the Prevention of Eating Disorders: A Case Application. SMITH COLLEGE STUDIES IN SOCIAL WORK. 2015;85(3):311-329.

3857. Huurne Elke, Postel Marloes, Haan Hein, Drossaert Constance, DeJong Cor Web-Based Treatment Program Using Intensive Therapeutic Contact for Patients With Eating Disorders: Before-After Study. JOURNAL OF MEDICAL INTERNET RESEARCH. 2013;15(2):.

3858. Saffari Mohsen, Fan Chia-Wei, Chang Yen-Ling, Huang Po-Ching, Tung Serene, Poon Wai, Lin Chien-Ching, Yang Wen-Chi, Lin Chung-Ying, Potenza Marc Yale Food Addiction Scale 2.0 (YFAS 2.0) and modified YFAS 2.0 (mYFAS 2.0): Rasch analysis and differential item functioning. JOURNAL OF EATING DISORDERS. 2022;10(1):.

3859. Grilo Carlos, Udo Tomoko Examining the significance of age of onset in persons with lifetime anorexia nervosa: Comparing child, adolescent, and emerging adult onsets in nationally representative US study. INTERNATIONAL JOURNAL OF EATING DISORDERS. 2021;54(9):1632-1640.

3860. Yan Fangfang, Zhang Meimei, Meng Yan, Li Huijuan, Yu Lie, Fu Xiaojie, Tang Youcai, Jiang Chao Erythropoietin improves hypoxic-ischemic encephalopathy in neonatal rats after short-term anoxia by enhancing angiogenesis. BRAIN RESEARCH. 2016;1651():104-113.

3861. Fluegge Keith Commentary: `Unhealthy diet,' nutrient status, and ADHD symptoms: a confounding role for environmental nitrous oxide exposure - reflections on Rijlaarsdam etal. (2016). JOURNAL OF CHILD PSYCHOLOGY AND PSYCHIATRY. 2017;58(1):28-29.

3862. Nicholls D Reading the signs of starvation. ACTA PAEDIATRICA. 2005;94(10):1356-1358.

3863. Jenkins Zoe, Chait Lior, Cistullo Leonardo, Castle David A comparison of eating disorder symptomatology, psychological distress and psychosocial function between early, typical and later onset anorexia nervosa. JOURNAL OF EATING DISORDERS. 2020;8(1):.

3864. Leplow Bernd, Renftle Daniela, Thomas Mareike, Michaelis Katja, Solbrig Susanne, Maetzler Walter, Berg Daniela, Liepelt-Scarfone Inga Characteristics of behavioural addiction in Parkinson's disease patients with self-reported impulse control disorder and controls matched for levodopa equivalent dose: a matched case-control study. JOURNAL OF NEURAL TRANSMISSION. 2023;130(2):125-133.

3865. Franko DL, Orosan-Weine P The prevention of eating disorders: Empirical, methodological, and conceptual considerations. CLINICAL PSYCHOLOGY-SCIENCE AND PRACTICE. 1998;5(4):459-477.

3866. Andonian Caroline, Langer Fabian, Beclunann Juergen, Bischoff Gert, Ewert Peter, Freilinger Sebastian, Kaemmerer Harald, Oberhoffer Renate, Pieper Lars, Neidenbach Rhoia Overweight and obesity: an emerging problem in patients with congenital heart disease. CARDIOVASCULAR DIAGNOSIS AND THERAPY. 2019;9(2):S360-S368.

3867. Holm-Denoma Jill, Richey J., Joiner Thomas The Latent Structure of Dietary Restraint, Body Dissatisfaction, and Drive for Thinness: A Series of Taxometric Analyses. PSYCHOLOGICAL ASSESSMENT. 2010;22(4):788-797.

3868. SANDERSPHILLIPS K CORRELATES OF HEALTHY EATING HABITS IN LOW-INCOME BLACK-WOMEN AND LATINAS. PREVENTIVE MEDICINE. 1994;23(6):781-787.

3869. McNicholas F. Childhood onset exercise addiction or atypical anorexia nervosa during Covid-19: case report. JOURNAL OF EATING DISORDERS. 2021;9(1):.

3870. Ernst Verena, Buerger Arne, Hammerle Florian Prevalence and severity of eating disorders: A comparison of DSM-IV and DSM-5 among German adolescents. INTERNATIONAL JOURNAL OF EATING DISORDERS. 2017;50(11):1255-1263.

3871. Murga Camila, Cabezas Ruth, Mora Carolina, Campos Susana, Nunez Daniel Examining associations between symptoms of eating disorders and symptoms of anxiety, depression, suicidal ideation, and perceived family functioning in university students: A brief report. INTERNATIONAL JOURNAL OF EATING DISORDERS. 2023;56(4, SI):783-789.

3872. Pinkasavage Emilie, Arigo Danielle, Schumacher Leah Social comparison, negative body image, and disordered eating behavior: The moderating role of coping style. EATING BEHAVIORS. 2015;16():72-77.

3873. Doubeni Chyke, Major Jacqueline, Laiyemo Adeyinka, Schootman Mario, Zauber Ann, Hollenbeck Albert, Sinha Rashmi, Allison Jeroan Contribution of Behavioral Risk Factors and Obesity to Socioeconomic Differences in Colorectal Cancer Incidence. JNCI-JOURNAL OF THE NATIONAL CANCER INSTITUTE. 2012;104(18):1353-1362.

3874. Lynn R., Viner R., Nicholls D. Ascertainment of early onset eating disorders: a pilot for developing a national child psychiatric surveillance system. CHILD AND ADOLESCENT MENTAL HEALTH. 2012;17(2):109-112.

3875. Ghaderi A, Scott B The Big Five and eating disorders: A prospective study in the general population. EUROPEAN JOURNAL OF PERSONALITY. 2000;14(4):311-323.

3876. Lampard Amy, Maclehose Richard, Eisenberg Marla, Larson Nicole, Davison Kirsten, Neumark-Sztainer Dianne Adolescents who engage exclusively in healthy weight control behaviors: Who are they?. INTERNATIONAL JOURNAL OF BEHAVIORAL NUTRITION AND PHYSICAL ACTIVITY. 2016;13():.

3877. Sanchez-Guarnido Antonio, Pino-Osuna Maria, Herruzo-Cabrera Francisco Personality prototype as a risk factor for eating disorders. REVISTA BRASILEIRA DE PSIQUIATRIA. 2015;37(4):325-330.

3878. Breton Edith, Dufour Rachel, Cote Sylvana, Dubois Lise, Vitaro Frank, Boivin Michel, Tremblay Richard, Booij Linda Developmental trajectories of eating disorder symptoms: A longitudinal study from early adolescence to young adulthood. JOURNAL OF EATING DISORDERS. 2022;10(1):.

3879. Keski-Rahkonen A, Bulik CM, Neale BM, Rose RJ, Rissanen A, Kaprio J Body dissatisfaction and drive for thinness in young adult twins. INTERNATIONAL JOURNAL OF EATING DISORDERS. 2005;37(3):188-199.

3880. Wright Jan, Halse Christine, Levy Gary Preteen Boys, Body Image, and Eating Disorders. MEN AND MASCULINITIES. 2016;19(1):3-21.

3881. Wu Ya-Ke, Berry Diane Impact of weight stigma on physiological and psychological health outcomes for overweight and obese adults: A systematic review. JOURNAL OF ADVANCED NURSING. 2018;74(5):1030-1042.

3882. Rosling Agneta, Ros Helena, Swenne Ingemar One-year outcome and incidence of anorexia nervosa and restrictive eating disorders among adolescent girls treated as out-patients in a family-based setting. UPSALA JOURNAL OF MEDICAL SCIENCES. 2016;121(1):50-59.

3883. Lewis-Smith Helena, Diedrichs Phillippa, Rumsey Nichola, Harcourt Diana A Systematic Review of Interventions on Body Image and Disordered Eating Outcomes among Women in Midlife. INTERNATIONAL JOURNAL OF EATING DISORDERS. 2016;49(1):5-18.

3884. Gillberg I., Billstedt Eva, Wentz Elisabet, Anckarsater Henrik, Rastam Maria, Gillberg Christopher Attention, executive functions, and mentalizing in anorexia nervosa eighteen years after onset of eating disorder. JOURNAL OF CLINICAL AND EXPERIMENTAL NEUROPSYCHOLOGY. 2010;32(4):358-365.

3885. PETRIE TA, STOEVER S THE INCIDENCE OF BULIMIA-NERVOSA AND PATHOGENIC WEIGHT CONTROL BEHAVIORS IN FEMALE COLLEGIATE GYMNASTS. RESEARCH QUARTERLY FOR EXERCISE AND SPORT. 1993;64(2):238-241.

3886. Garcia-Burgos David, Wilhelm Peter, Voegele Claus, Munsch Simone Food avoidance versus food aversion in restrictive eating disorders. ZEITSCHRIFT FUR PSYCHIATRIE PSYCHOLOGIE UND PSYCHOTHERAPIE. 2019;67(1):30-38.

3887. Carrard Isabelle, Rothen Stephane, Rodgers Rachel Body image and disordered eating in older women: A Tripartite Sociocultural model. EATING BEHAVIORS. 2020;38():.

3888. Galmiche Marie, Godefroy Clemence, Achamrah Najate, Grigioni Sebastien, Colange Guillaume, Folope Vanessa, Petit Andre, Rapp Clement, Coeffier Moise, Dechelotte Pierre, Tavolacci Marie-Pierre Mental health and health behaviours among patients with eating disorders: a case-control study in France. JOURNAL OF EATING DISORDERS. 2022;10(1):.

3889. Accurso Erin, Sim Leslie, Muhlheim Lauren, Lebow Jocelyn Parents know best: Caregiver perspectives on eating disorder recovery. INTERNATIONAL JOURNAL OF EATING DISORDERS. 2020;53(8):1252-1260.

3890. Siddiqui Sana, Mateen Somaiya, Ahmad Rizwan, Moin Shagufta A brief insight into the etiology, genetics, and immunology of polycystic ovarian syndrome (PCOS). JOURNAL OF ASSISTED REPRODUCTION AND GENETICS. 2022;39(11):2439-2473.

3891. Farmer Cristan, Thienemann Margo, Leibold Collin, Kamalani Gabrielle, Sauls Bethany, Frankovich Jennifer Psychometric Evaluation of the Caregiver Burden Inventory in Children and Adolescents With PANS. JOURNAL OF PEDIATRIC PSYCHOLOGY. 2018;43(7):749-757.

3892. Chen Jue, Wang Zhen, Guo Boliang, Arcelus Jon, Zhang Haiyin, Jia Xiuzhen, Xu Yong, Qiu Jianyin, Xiao Zeping, Yang Min Negative Affect Mediates Effects of Psychological Stress on Disordered Eating in Young Chinese Women. PLOS ONE. 2012;7(10):.

3893. Wood Ashley, Dygdon Judith, Conger Anthony Eating disorders and sense of self: A learning theory conceptualization. EATING BEHAVIORS. 2015;17():45-48.

3894. Ayenigbara Israel The Accumulation of Visceral Fat and Preventive Measures among the Elderly. CARDIOVASCULAR INNOVATIONS AND APPLICATIONS. 2020;4(4):279-285.

3895. Niessen S., Kohlenberg-Mueller K., Hofmann W. EATING DISORDER CHARACTERISTICS AMONG GERMAN MALE AND FEMALE TRIATHLETES. ANNALS OF NUTRITION AND METABOLISM. 2013;63(1):867.

3896. Strobel Christian, Kolbeck Daniela, Mayer Isabelle, Perras Manuela, Wunderer Eva Muscle Dysmorphia: The compulsive Pursuit of a muscular Body Characteristics, Prevalence, Causes, Treatment, Prevention, and nutritional Implications. ERNAHRUNGS UMSCHAU. 2020;67(12):M702-M709.

3897. Jannasch Franziska, Kroeger Janine, Schulze Matthias Dietary Patterns and Type 2 Diabetes: A Systematic Literature Review and Meta-Analysis of Prospective Studies. JOURNAL OF NUTRITION. 2017;147(6):1174-1182.

3898. Nguyen Mathew, Shapiro Michael, Welch Stephen A case of severe adolescent obsessive-compulsive disorder treated with inpatient hospitalization, risperidone and sertraline. JOURNAL OF BEHAVIORAL ADDICTIONS. 2012;1(2):78-82.

3899. Richter Felicitas, Strauss Bernhard, Braehler Elmar, Adametz Luise, Berger Uwe Screening disordered eating in a representative sample of the German population: Usefulness and psychometric properties of the German SCOFF questionnaire. EATING BEHAVIORS. 2017;25(SI):81-88.

3900. McClelland L, Crisp A Anorexia nervosa and social class. INTERNATIONAL JOURNAL OF EATING DISORDERS. 2001;29(2):150-156.

3901. Cella Stefania, Iannaccone Mara, Cotrufo Paolo How perceived parental bonding affects self-concept and drive for thinness: A community-based study. EATING BEHAVIORS. 2014;15(1):110-115.

3902. Pearson Adria, Follette Victoria, Hayes Steven A Pilot Study of Acceptance and Commitment Therapy as a Workshop Intervention for Body Dissatisfaction and Disordered Eating Attitudes. COGNITIVE AND BEHAVIORAL PRACTICE. 2012;19(1):181-197.

3903. Donnadieu-Rigole Helene, Olive Laetitia, Nalpas Bertrand, Duny Yohan, Nocca David, Perney Pascal Prevalence of Psychoactive Substance Consumption in People With Obesity. SUBSTANCE USE \& MISUSE. 2016;51(12):1649-1654.

3904. Cottone Pietro, Sabino Valentina, Roberto Marisa, Bajo Michal, Pockros Lara, Frihauf Jennifer, Fekete Eva, Steardo Luca, Rice Kenner, Grigoriadis Dimitri, Conti Bruno, Koob George, Zorrilla Eric CRF system recruitment mediates dark side of compulsive eating. PROCEEDINGS OF THE NATIONAL ACADEMY OF SCIENCES OF THE UNITED STATES OF AMERICA. 2009;106(47):20016-20020.

3905. Yang Chi-Ju, Chiu Ching-Tang, Yeh Yu-Chang, Chao Anne Successful management of delirium with dexmedetomidine in a patient with haloperidol-induced neuroleptic malignant syndrome: A case report. WORLD JOURNAL OF CLINICAL CASES. 2022;10(2):625-630.

3906. Del Giacco Stefano, Cappai Alessandra, Gambula Luisanna, Cabras Stefano, Perra Silvia, Manconi Paolo, Carpiniello Bernardo, Pinna Federica The asthma-anxiety connection. RESPIRATORY MEDICINE. 2016;120():44-53.

3907. STEINHAUSEN HC, SEIDEL R SHORT-TERM AND INTERMEDIATE-TERM OUTCOME IN ADOLESCENT EATING DISORDERS. ACTA PSYCHIATRICA SCANDINAVICA.

1993;88(3):169-173.

3908. Mendelsohn Bryce, Malone James, Townsend R., Gitlin Jonathan Proteomic analysis of anoxia tolerance in the developing zebrafish embryo. COMPARATIVE BIOCHEMISTRY AND PHYSIOLOGY D-GENOMICS \& PROTEOMICS. 2009;4(1):21-31.

3909. Hyman PE, Bursch B, Sood M, Schwankovsky L, Cocjin J, Zeltzer LK Visceral pain-associated disability syndrome: A descriptive analysis. JOURNAL OF PEDIATRIC GASTROENTEROLOGY AND NUTRITION. 2002;35(5):663-668.

3910. Stewart Shannon, Toohey Ashley, Celebre Angela, Poss Jeff Abuse, Mental State, and Health Factors Pre and during the COVID-19 Pandemic: A Comparison among Clinically Referred Adolescents in Ontario, Canada. INTERNATIONAL JOURNAL OF ENVIRONMENTAL RESEARCH AND PUBLIC HEALTH. 2021;18(19):.

3911. Edney Sarah, Park Su, Tan Linda, Chua Xin, Dickens Borame, Rebello Salome, Petrunoff Nick, Muller Andre, Tan Cheun, Mueller-Riemenschneider Falk, Dam Rob Advancing understanding of dietary and movement behaviours in an Asian population through real-time monitoring: Protocol of the Continuous Observations of Behavioural Risk Factors in Asia study (COBRA). DIGITAL HEALTH. 2022;8():.

3912. Mukherjee Koninika, Hussain Dilwar The role of peer victimization and emotion dysregulation in social anxiety and disordered eating comorbidity in young adults. CURRENT PSYCHOLOGY. 2022;41(9):6424-6438.

3913. Latzer Itay, Lerner-Geva Liat, Stein Daniel, Weiss Batia, Pinhas-Hamiel Orit Disordered eating behaviors in adolescents with celiac disease. EATING AND WEIGHT DISORDERS-STUDIES ON ANOREXIA BULIMIA AND OBESITY. 2020;25(2):365-371.

3914. Matsumura Yuichiro, Yamamoto Ryohei, Shinzawa Maki, Matsushita Taisuke, Yoshimura Ryuichi, Otsuki Naoko, Mizui Masayuki, Matsui Isao, Kaimori Junya, Sakaguchi Yusuke, Ishibashi Chisaki, Ide Seiko, Nakanishi Kaori, Nishida Makoto, Kudo Takashi, Yamauchi-Takihara Keiko, Nagatomo Izumi, Moriyama Toshiki Skipping Breakfast and Incidence of Frequent Alcohol Drinking in University Students in Japan: A Retrospective Cohort Study. NUTRIENTS. 2022;14(13):.

3915. Reas DL, Schoemaker C, Zipfel S, Williamson DA Prognostic value of duration of illness and early intervention in bulimia nervosa: A systematic review of the outcome literature.

INTERNATIONAL JOURNAL OF EATING DISORDERS. 2001;30(1):1-10.

3916. Brahm Nancy, McElwain Did, Brown Robert Potential aripiprazole-mediated extrapyramidal symptoms in an adult with developmental disabilities. AMERICAN JOURNAL OF HEALTH-SYSTEM PHARMACY. 2007;64(8):827-829.

3917. HOEK HW, BARTELDS AIM, BOSVELD JJF, VANDERGRAAF Y, LIMPENS VEL, MAIWALD M, SPAAIJ CJK IMPACT OF URBANIZATION ON DETECTION RATES OF EATING DISORDERS. AMERICAN JOURNAL OF PSYCHIATRY. 1995;152(9):1272-1278.

3918. Muehlenkamp Jennifer, Peat Christine, Claes Laurence, Smits Dirk Self-Injury and Disordered Eating: Expressing Emotion Dysregulation Through the Body. SUICIDE AND LIFE-THREATENING BEHAVIOR. 2012;42(4):416-425.

3919. Francisco Rita, Espinoza Paola, Gonzalez Marcela, Penelo Eva, Mora Marisol, Roses Rocio, Raich Rosa Body dissatisfaction and disordered eating among Portuguese and Spanish adolescents: The role of individual characteristics and internalisation of sociocultural ideals. JOURNAL OF ADOLESCENCE. 2015;41():7-16.

3920. Hetherington MM Eating disorders: Diagnosis, etiology, and prevention. NUTRITION. 2000;16(7-8):547-551.

3921. Bonci Christine, Bonci Leslie, Granger Lorita, Johnson Craig, Malina Robert, Milne Leslie, Ryan Randa, Vanderbunt Erin National athletic trainers' association position statement: Preventing, detecting, and managing disordered eating in athletes. JOURNAL OF ATHLETIC TRAINING. 2008;43(1):80-108.

3922. Watanabe Yasuhiro, Yamaguchi Takashi, Tanaka Sho, Sasaki Akira, Naitoh Takeshi, Matsubara Hisahiro, Yokote Koutaro, Okazumi Shinichi, Ugi Satoshi, Yamamoto Hiroshi, Ohta Masayuki, Ishigaki Yasushi, Kasama Kazunori, Seki Yosuke, Tsujino Motoyoshi, Shirai Kohji, Miyazaki Yasuhiro, Masaki Takayuki, Nagayama Daiji, Saiki Atushito, Tatsuno Ichiro Characteristics of Childhood Onset and Post-Puberty Onset Obesity and Weight Regain after Laparoscopic Sleeve Gastrectomy in Japanese Subjects: A Subgroup Analysis of J-SMART. OBESITY FACTS. 2022;15(4):498-507.

3923. Griffiths RA, ChannonLittle L Psychological treatments and bulimia nervosa: An update. AUSTRALIAN PSYCHOLOGIST. 1996;31(2):79-96.

3924. Herrera Pineda Isoled, Garces Maggi Bertha, Monserrate Juan, Suarez Lindao Bolivar SELF-CARE IN TYPE 2 DIABETES MELLITUS: INTERPRETATION OF THE SEDENTARISM VARIABLE. COMUNIDAD Y SALUD. 2021;19(1):42-51.

3925. Czepczor-Bernat Kamila, Modrzejewska Adriana, Modrzejewska Justyna, Majzner Rafal Comparison of Food-Based and Music-Based Regulatory Strategies for (Un)Healthy Eating, Depression, Anxiety and Stress. NUTRIENTS. 2022;14(1):.

3926. McCabe Marita, Fuller-Tyszkiewicz Matthew, Mellor David, Maiano Christophe Body image, disordered eating, higher weight, and their associated factors: Can we use the same scales to measure constructs across different countries?. BODY IMAGE. 2020;35():316-319.

3927. Barker ET, Galambos NL Body dissatisfaction of adolescent girls and boys: Risk and resource factors. JOURNAL OF EARLY ADOLESCENCE. 2003;23(2):141-165.

3928. Wang Yufeng, Zong Ligeng, Wang Xiaolei TGF-beta improves myocardial function and prevents apoptosis induced by anoxia-reoxygenation, through the reduction of endoplasmic reticulum stress. CANADIAN JOURNAL OF PHYSIOLOGY AND PHARMACOLOGY. 2016;94(1):9-17.

3929. Byrne Meghan, Eichen Dawn, Fitzsimmons-Craft Ellen, Taylor C., Wilfley Denise Perfectionism, emotion dysregulation, and affective disturbance in relation to clinical impairment in college-age women at high risk for or with eating disorders. EATING BEHAVIORS. 2016;23():131-136.

3930. Jenkins P., Lebow J., Rienecke R. Weight suppression as a predictor variable in the treatment of eating disorders: A systematic review. JOURNAL OF PSYCHIATRIC AND MENTAL HEALTH NURSING. 2018;25(5-6):297-306.

3931. MITRANY E, LUBIN F, CHETRIT A, MODAN B EATING DISORDERS AMONG JEWISH FEMALE ADOLESCENTS IN ISRAEL - A 5-YEAR STUDY. JOURNAL OF ADOLESCENT HEALTH. 1995;16(6):454-457.

3932. Pop Lavinia-Maria, Iorga Magdalena, Muraru Iulia-Diana FACTORS INFLUENCING DIETARY HABITS AMONG MEDICAL STUDENTS AND DOCTORS. A THEORETICAL APPROACH. MEDICAL-SURGICAL JOURNAL-REVISTA MEDICO-CHIRURGICALA. 2019;123(4):717-727.

3933. Angst Jules, Gamma Alex, Roessler Wulf, Ajdacic Vladeta, Klein Daniel Long-term depression versus episodic major depression: Results from the prospective Zurich study of a community sample. JOURNAL OF AFFECTIVE DISORDERS. 2009;115(1-2):112-121.

3934. Gregory John Prevention of Obesity and Metabolic Syndrome in Children. FRONTIERS IN ENDOCRINOLOGY. 2019;10():.

3935. Lindenberg Katajun, Kordy Hans Efficacy of an Internet-Delivered Tiered Strategy for Eating Disorder Prevention in High School Students. KINDHEIT UND ENTWICKLUNG. 2015;24(1):55-63.

3936. Tommasi Marco, Toro Francesca, Salvia Alessandra, Saggino Aristide Connections between Children's Eating Habits, Mental Health, and Parental Stress. JOURNAL OF OBESITY. 2022;2022():.

3937. Rodin G, Olmsted MP, Rydall AC, Maharaj SI, Colton PA, Jones JM, Biancucci LA, Daneman D Eating disorders in young women with type 1 diabetes mellitus. JOURNAL OF PSYCHOSOMATIC RESEARCH. 2002;53(4):943-949.

3938. Zamantakis Alithia, Lackey Dresden Dying to be (A)Gendered: An Exploratory Content Analysis of Trans/Nonbinary People's Experiences with Eating Disorders. SOCIOLOGICAL INQUIRY. 2022;92(1):870-893.

3939. Berksoy Emel, Ozyurt Gonca, Anil Murat, Uzum Ozlem, Appak Yeliz Can pediatricians recognize eating disorders? A case study of early-onset anorexia nervosa in a male child. NUTRICION HOSPITALARIA. 2018;35(2):499-502.

3940. Cherubini Valentino, Skrami Edlira, Iannilli Antonio, Cesaretti Alessandra, Paparusso Anna, Alessandrelli Maria, Carle Flavia, Ferrito Lucia, Gesuita Rosaria Disordered eating behaviors in adolescents with type 1 diabetes: A cross-sectional population-based study in Italy. INTERNATIONAL JOURNAL OF EATING DISORDERS. 2018;51(8):890-898.

3941. Morris K, Burke IT, Boothman C, Lloyd JR, Mortimer RJG Technetium solubility during the onset of progressive anoxia. GEOCHIMICA ET COSMOCHIMICA ACTA. 2004;68(11, S):A501.

3942. Barreto Margarida, Ferreira Claudia, Marta-Simoes Joana, Mendes Ana Exploring the paths between self-compassionate attributes and actions, body compassion and disordered eating. EATING AND WEIGHT DISORDERS-STUDIES ON ANOREXIA BULIMIA AND OBESITY. 2020;25(2):291-297.

3943. Okamoto Yuri, Miyake Yoshie, Nagasawa Ichie, Yoshihara Masaharu Cohort survey of college students' eating attitudes: interventions for depressive symptoms and stress coping were key factors for preventing bulimia in a subthreshold group. BIOPSYCHOSOCIAL MEDICINE. 2018;12():.

3944. Messouak Ouafae, Boujraf Said, Chaouki Sana, Belahsen Mohammed Delayed onset dystonia secondary to neonatal anoxia. NEUROSCIENCES. 2008;13(2):184-185.

3945. Berger Uwe, Joseph Andrea, Sowa Melanie, Strauss Bernhard The Barbie-matrix: Effectiveness of a school based German program for the primary prevention of anorexia nervosa developed for girls up to the age of 12. PSYCHOTHERAPIE PSYCHOSOMATIK MEDIZINISCHE PSYCHOLOGIE. 2007;57(6):248-255.

3946. Hou Fangli, Xu Shaojun, Zhao Yuqiu, Lu Qingyun, Zhang Shichen, Zu Ping, Sun Ying, Su Puyu, Tao Fangbiao Effects of emotional symptoms and life stress on eating behaviors among adolescents. APPETITE. 2013;68():63-68.

3947. Hwalla Nahla, Jaafar Zeinab, Sawaya Sally Dietary Management of Type 2 Diabetes in the MENA Region: A Review of the Evidence. NUTRIENTS. 2021;13(4):.

3948. Fernandes Tito, Garrine Carmen, Ferrao Jorge, Bell Victoria, Varzakas Theodoros Mushroom Nutrition as Preventative Healthcare in Sub-Saharan Africa. APPLIED SCIENCES-BASEL. 2021;11(9):.

3949. Holland Anthony, Aman Lucie, Whittington Joyce Defining Mental and Behavioural Disorders in Genetically Determined Neurodevelopmental Syndromes with Particular Reference to Prader-Willi Syndrome. GENES. 2019;10(12):.

3950. Goldschmidt A., Wall M., Choo T-H, Larson N., Neumark-Sztainer D. Mediators involved in the relation between depressive symptoms and weight status in female adolescents and young adults. INTERNATIONAL JOURNAL OF OBESITY. 2015;39(6):1027-1029.

3951. Allison Stephen, Wade Tracey, Warin Megan, Long Randall, Bastiampillai Tarun, Looi Jeffrey Tertiary eating disorder services: is it time to integrate specialty care across the life span?. AUSTRALASIAN PSYCHIATRY. 2021;29(5):516-518.

3952. Musaiger Abdulrahman, Al-Mannai Mariam, Tayyem Reema, Al-Lalla Osama, Ali Essa, Kalam Faiza, Benhamed Mofida, Saghir Sabri, Halahleh Ismail, Djoudii Zahra, Chirane Manel Risk of disordered eating attitudes among adolescents in seven Arab countries by gender and obesity: A cross-cultural study. APPETITE. 2013;60():162-167.

3953. Marchiori David, Papies Esther A brief mindfulness intervention reduces unhealthy eating when hungry, but not the portion size effect. APPETITE. 2014;75():40-45.

3954. Tavolacci Marie-Pierre, Ladner Joel, Dechelotte Pierre COVID-19 Pandemic and Eating Disorders among University Students. NUTRIENTS. 2021;13(12):.

3955. Sundgot-Borgen J, Bahr R, Falch JA, Schneider LS Normal bone mass in bulimic women. JOURNAL OF CLINICAL ENDOCRINOLOGY \& METABOLISM. 1998;83(9):3144-3149.

3956. Warner Christopher, Warner Carolynn, Matuszak Theresa, Rachal James, Flynn Julianne, Grieger Thomas Disordered eating in entry-level military personnel. MILITARY MEDICINE. 2007;172(2):147-151.

3957. Busetto L., Mazza M., Salvalaio S., De Stefano F., Marangon M., Calo E., Sampietro S., Enzi G. Obesity treatment in elderly outpatients: Predictors of efficacy and drop-out. EATING AND WEIGHT DISORDERS-STUDIES ON ANOREXIA BULIMIA AND OBESITY. 2009;14(2-3):E56-E65.

3958. Yoon Cynthia, Jacobs David, Duprez Daniel, Dutton Gareth, Lewis Cora, Neumark-Sztainer Dianne, Steffen Lyn, West Delia, Mason Susan Questionnaire-based problematic relationship to eating and food is associated with 25 year body mass index trajectories during midlife: The Coronary Artery Risk Development In Young Adults (CARDIA) Study. INTERNATIONAL JOURNAL OF EATING DISORDERS. 2018;51(1):10-17.

3959. Koller Katherine, Thompson Katherine, Miller Alexandra, Walsh Emily, Bardone-Cone Anna Body appreciation and intuitive eating in eating disorder recovery. INTERNATIONAL JOURNAL OF EATING DISORDERS. 2020;53(8):1261-1269.

3960. Badaly Daryaneh Peer similarity and influence for weight-related outcomes in adolescence: A meta-analytic review. CLINICAL PSYCHOLOGY REVIEW. 2013;33(8):1218-1236.

3961. Bray Isabelle, Slater Amy, Lewis-Smith Helena, Bird Emma, Sabey Abigail Promoting positive body image and tackling overweight/obesity in children and adolescents: A combined health psychology and public health approach. PREVENTIVE MEDICINE. 2018;116():219-221.

3962. Glashouwer Klaske, Veer Roosmarijn, Adipatria Fayanadya, Jong Peter, Vocks Silja The role of body image disturbance in the onset, maintenance, and relapse of anorexia nervosa: A systematic review. CLINICAL PSYCHOLOGY REVIEW. 2019;74():.

3963. Knatz Stephanie, Braden Abby, Boutelle Kerri Parent Coaching Model for Adolescents With Emotional Eating. EATING DISORDERS. 2015;23(4, SI):377-386.

3964. Hernandez J., Gomez F., Stadheim J., Perez M., Bekele B., Yu K., Henning T. Hourglass Body Shape Ideal Scale and disordered eating. BODY IMAGE. 2021;38():85-94.

3965. Austin SB Prevention research in eating disorders: theory and new directions. PSYCHOLOGICAL MEDICINE. 2000;30(6):1249-1262.

3966. Dessauvagie Anja, Dang Hoang-Minh, Nguyen Thi, Groen Gunter Mental Health of University Students in Southeastern Asia: A Systematic Review. ASIA-PACIFIC JOURNAL OF PUBLIC HEALTH. 2022;34(2-3):172-181.

3967. Vervoort L., Naets T., De Guchtenaere A., Tanghe A., Braet C. Using confidence interval-based estimation of relevance to explore bottom-up and top-down determinants of problematic eating behavior in children and adolescents with obesity from a dual pathway perspective. APPETITE. 2020;150():.

3968. Lloyd E., Haase Anne, Verplanken Bas Anxiety and the development and maintenance of anorexia nervosa: protocol for a systematic review. SYSTEMATIC REVIEWS. 2018;7():.

3969. Echeverria Guadalupe, McGee Emma, Urquiaga Ines, Jimenez Paulina, D'Acuna Sonia, Villarroel Luis, Velasco Nicolas, Leighton Federico, Rigotti Attilio Inverse Associations between a Locally Validated Mediterranean Diet Index, Overweight/Obesity, and Metabolic

Syndrome in Chilean Adults. NUTRIENTS. 2017;9(8):.

3970. Du Yanyan, Zhu Hong, Li Dongye, Wang Lele, Zhang Lin, Luo Yuanyuan, Pan Defeng, Huang Manman Lentiviral-mediated overexpression of Akt1 reduces anoxia-reoxygenation injury in cardiomyocytes. CELL BIOLOGY INTERNATIONAL. 2014;38(4):488-496.

3971. Favaro A, Zanetti T, Huon G, Santonastaso P Engaging teachers in an eating disorder preventive intervention. INTERNATIONAL JOURNAL OF EATING DISORDERS. 2005;38(1):73-77.

3972. Ricca V, Mannucci E, Calabro A, Di Bernardo M, Cabras PL, Rotella CM Anorexia nervosa and celiac disease: Two case reports. INTERNATIONAL JOURNAL OF EATING DISORDERS. 2000;27(1):119-122.

3973. Steinhausen Hans-Christoph, Jensen Christina Time trends in lifetime incidence rates of first-time diagnosed anorexia nervosa and bulimia nervosa across 16 years in a danish nationwide psychiatric registry study. INTERNATIONAL JOURNAL OF EATING DISORDERS. 2015;48(7):845-850.

3974. Ghaderi A, Scott B Prevalence, incidence and prospective risk factors for eating disorders. ACTA PSYCHIATRICA SCANDINAVICA. 2001;104(2):122-130.

3975. Skoog Annelie, Arias-Esquivel Victor The effect of induced anoxia and reoxygenation on benthic fluxes of organic carbon, phosphate, iron, and manganese. SCIENCE OF THE TOTAL ENVIRONMENT. 2009;407(23):6085-6092.

3976. Moe EL, Elliot DL, Goldberg L, DeFrancesco CA, Durham MB, Hix-Small H Curriculum fop preventing disordered eating and body-shaping drug use.. JOURNAL OF INVESTIGATIVE MEDICINE. 2005;53(1, S):S144.

3977. Kenardy J, Brown WJ, Vogt E Dieting and health in young Australian women. EUROPEAN EATING DISORDERS REVIEW. 2001;9(4):242-254.

3978. Milano Walter, Ambrosio Paola, Carizzone Francesca, De Biasio Valeria, Foia Maria, Saetta Biancamaria, Milano Maria, Capasso Anna Menstrual Disorders Related to Eating Disorders. ENDOCRINE METABOLIC \& IMMUNE DISORDERS-DRUG TARGETS.

2022;22(5):471-480.

3979. Quattropani Maria, Geraci Alessandra, Lenzo Vittorio, Sardella Alberto, Schimmenti Adriano Failures in Reflective Functioning, Dissociative Experiences, and Eating Disorder: a Study On a Sample of Italian Adolescents. JOURNAL OF CHILD \& ADOLESCENT TRAUMA. 2022;15(2):365-374.

3980. Rosenbaum Diane, White Kamila, Artime Tiffany Coping with childhood maltreatment: Avoidance and eating disorder symptoms. JOURNAL OF HEALTH PSYCHOLOGY. 2021;26(14):2832-2840.

3981. Kuoppamaki M, Bhatia KP, Quinn N Progressive delayed-onset dystonia after cerebral anoxic insult in adults. MOVEMENT DISORDERS. 2002;17(6):1345-1349.

3982. Mutterperl JA, Sanderson CA Mind over matter: Internalization of the thinness norm as a moderator of responsiveness to norm misperception education in college women. HEALTH PSYCHOLOGY. 2002;21(5):519-523.

3983. Loucas Christina, Fairburn Christopher, Whittington Craig, Pennant Mary, Stockton Sarah, Kendall Tim E-therapy in the treatment and prevention of eating disorders: A systematic review and meta-analysis. BEHAVIOUR RESEARCH AND THERAPY. 2014;63():122-131.

3984. Minnick Alyssa, Cachelin Fary, Gil-Rivas Virginia Examining predictors of binge eating behaviors among racially and ethnically diverse college men. JOURNAL OF AMERICAN COLLEGE HEALTH. 2022;():.

3985. Morris Margaret, Beilharz Jessica, Maniam Jayanthi, Reichelt Amy, Westbrook R. Why is obesity such a problem in the 21st century? The intersection of palatable food, cues and reward pathways, stress, and cognition. NEUROSCIENCE AND BIOBEHAVIORAL REVIEWS. 2015;58():36-45.

3986. Mehler PS Diagnosis and care of patients with anorexia nervosa in primary care settings. ANNALS OF INTERNAL MEDICINE. 2001;134(11):1048-1059.

3987. De Nucci Sara, Zupo Roberta, Castellana Fabio, Sila Annamaria, Triggiani Vincenzo, Lisco Giuseppe, De Pergola Giovanni, Sardone Rodolfo Public Health Response to the SARS-

CoV-2 Pandemic: Concern about Ultra-Processed Food Consumption. FOODS. 2022;11(7):.

3988. Marzola Enrica, Porliod Alain, Panero Matteo, De-Bacco Carlotta, Abbate-Daga Giovanni Affective temperaments and eating psychopathology in anorexia nervosa: Which role for anxious and depressive traits?. JOURNAL OF AFFECTIVE DISORDERS. 2020;266():374-380.

3989. Reynolds-May Margaret, Kenna Heather, Marsh Wendy, Stemmle Pascale, Wang Po, Ketter Terence, Rasgon Natalie Evaluation of reproductive function in women treated for bipolar disorder compared to healthy controls. BIPOLAR DISORDERS. 2014;16(1, SI):37-47.

3990. Raza Syed, Sabir Sobia, Ali Kamran, Ali Choudhary, Riaz Amir, Hussain Irshad, Hussain Shujaat, Tareen Mujeeb, Mubeen Khalid, Shahzad Riyaz, Sarwar Shahbaz Metabesity: expert panel recommendation for taking up the challenge by a multidisciplinary approach. JOURNAL OF THE PAKISTAN MEDICAL ASSOCIATION. 2020;70(8):1418-1424.

3991. Carter JC, Stewart DA, Dunn VJ, Fairburn CG Primary prevention of eating disorders: Might it do more harm than good?. INTERNATIONAL JOURNAL OF EATING DISORDERS. 1997;22(2):167-172.

3992. Boswell Rebecca, Sun Wendy, Suzuki Shosuke, Kober Hedy Training in cognitive strategies reduces eating and improves food choice. PROCEEDINGS OF THE NATIONAL ACADEMY OF SCIENCES OF THE UNITED STATES OF AMERICA. 2018;115(48):E11238-E11247.

3993. Ridgeway Laura, Katzman Debra, McNicholas Fiona Behaviour, Belief and Impairment (BBI): a diagnostic procedure for eating disorders in primary care. IRISH JOURNAL OF MEDICAL SCIENCE. 2022;():.

3994. Mun Hyukjin, So Eun Changes in Physical Activity, Healthy Diet, and Sleeping Time during the COVID-19 Pandemic in South Korea. NUTRIENTS. 2022;14(5):.

3995. Graaf R, Bijl RV, Smit F, Ravelli A, Vollebergh WAM Psychiatric and sociodemographic predictors of attrition in a longitudinal study - The Netherlands Mental Health Survey and Incidence Study (NEMESIS). AMERICAN JOURNAL OF EPIDEMIOLOGY. 2000;152(11):1039-1047.

3996. Vincenzi Brenda, O'Toole Julie, Lask Bryan PANDAS and Anorexia Nervosa-A Spotters' Guide: Suggestions for Medical Assessment. EUROPEAN EATING DISORDERS REVIEW. 2010;18(2):116-123.

3997. Lee Ju-Yeon, Kim Sung-Wan, Kim Jae-Min, Shin Il-Seon, Yoon Jin-Sang Two Cases of Eating Disorders in Adolescents with Dental Braces Fitted Prior to the Onset of Anorexia Nervosa. PSYCHIATRY INVESTIGATION. 2015;12(3):411-414.

3998. Loftus J., Scott J., Vorspan F., Icick R., Henry C., Gard S., Kahn J., Leboyer M., Bellivier F., Etain B. Psychiatric comorbidities in bipolar disorders: An examination of the prevalence and chronology of onset according to sex and bipolar subtype. JOURNAL OF AFFECTIVE DISORDERS. 2020;267():258-263.

3999. Knez R, Munjas R, Petrovecki M, Paucic-Kirincic E, Persic M Disordered eating attitudes among elementary school population. JOURNAL OF ADOLESCENT HEALTH. 2006;38(5):628-630.

4000. Requena-Ocana Nerea, Flores-Lopez Maria, San Martin Alicia, Garcia-Marchena Nuria, Pedraz Maria, Jesus Ruiz Juan, Serrano Antonia, Suarez Juan, Javier Pavon Francisco, Fonseca Fernando, Araos Pedro Influence of gender and education on cocaine users in an outpatient cohort in Spain. SCIENTIFIC REPORTS. 2021;11(1):.

4001. Viborg Njordur, Wangby-Lundh Margit, Lundh Lars-Gunnar, Wallin Ulf, Johnsson Per Disordered eating in a Swedish community sample of adolescent girls: subgroups, stability, and associations with body esteem, deliberate self-harm and other difficulties. JOURNAL OF EATING DISORDERS. 2018;6():.

4002. Neubauer Karolin, Weigel Angelika, Daubmann Anne, Wendt Hanna, Rossi Maddalena, Loewe Bernd, Gumz Antje Paths to First Treatment and Duration of Untreated Illness in Anorexia Nervosa: Are There Differences According to Age of Onset?. EUROPEAN EATING DISORDERS REVIEW. 2014;22(4):292-298.

4003. Neal Timothy, Diamond Alex, Goldman Scott, Liedtka Karl, Mathis Kembra, Morse Eric, Putukian Margot, Quandt Eric, Ritter Stacey, Sullivan John, Welzant Victor Interassociation Recommendations for Developing a Plan to Recognize and Refer Student-Athletes With Psychological Concerns at the Secondary School Level: A Consensus Statement. JOURNAL OF ATHLETIC TRAINING. 2015;50(3):231-249.

4004. Stunkard A., Lu X-Y Rapid changes in night eating: Considering mechanisms. EATING AND WEIGHT DISORDERS-STUDIES ON ANOREXIA BULIMIA AND OBESITY. 2010;15(1-2):E2-E8.

4005. Friedman S, Vila G, Timsit J, Boitard C, Mouren-Simeoni MC Eating disorders and insulin-dependent diabetes mellitus (IDDM): relationships with glycaemic control and somatic complications. ACTA PSYCHIATRICA SCANDINAVICA. 1998;97(3):206-212.

4006. Al Banna Md, Dewan Md, Tariq Mohammad, Sayeed Abu, Kundu Satyajit, Disu Tasnim, Akter Sumaiya, Sahrin Sumaia, Khan Md Prevalence and determinants of eating disorder risk among Bangladeshi public university students: A cross-sectional study. HEALTH PSYCHOLOGY RESEARCH. 2021;9(1):.

4007. Herbert Cornelia, Kuebler Andrea, Voegele Claus Risk for Eating Disorders Modulates Startle-Responses to Body Words. PLOS ONE. 2013;8(1):.

4008. Thornborrow T., Evans E., Tovee M., Boothroyd L. Sociocultural drivers of body image and eating disorder risk in rural Nicaraguan women. JOURNAL OF EATING DISORDERS. 2022;10(1):.

4009. Cardi Valentina, Mallorqui-Bague Nuria, Albano Gaia, Monteleone Alessio, Fernandez-Aranda Fernando, Treasure Janet Social Difficulties As Risk and Maintaining Factors in Anorexia Nervosa: A Mixed-Method Investigation. FRONTIERS IN PSYCHIATRY. 2018;9():.

4010. BRYANTWAUGH R, LASK B ANNOTATION - EATING DISORDERS IN CHILDREN. JOURNAL OF CHILD PSYCHOLOGY AND PSYCHIATRY AND ALLIED DISCIPLINES. 1995;36(2):191-202.

4011. GOODMAN A DIAGNOSIS AND TREATMENT OF SEXUAL ADDICTION. JOURNAL OF SEX \& MARITAL THERAPY. 1993;19(3):225-251.

4012. McComb JJR, Clopton JR Explanatory variance in bulimia nervosa. WOMEN \& HEALTH. 2002;36(4):115-123.

4013. Javier Sarah, Moore Melanie, Belgrave Faye Racial comparisons in perceptions of maternal and peer attitudes, body dissatisfaction, and eating disorders among African

American and White women. WOMEN \& HEALTH. 2016;56(6):615-633.

4014. McElroy Susan, Winstanley Erin, Mori Nicole, Martens Brian, McCoy Jessica, Moeller Dianna, Guerdjikova Anna, Keck Paul A Randomized, Placebo-Controlled Study of Zonisamide to Prevent Olanzapine-Associated Weight Gain. JOURNAL OF CLINICAL PSYCHOPHARMACOLOGY. 2012;32(2):165-172.

4015. Bauer Stephanie, Okon Eberhard, Meermann Rolf, Kordy Hans Technology-Enhanced Maintenance of Treatment Gains in Eating Disorders: Efficacy of an Intervention Delivered via Text Messaging. JOURNAL OF CONSULTING AND CLINICAL PSYCHOLOGY. 2012;80(4):700-706.

4016. Mason Tyler, Barrington-Trimis Jessica, Leventhal Adam Eating to Cope With the COVID-19 Pandemic and Body Weight Change in Young Adults. JOURNAL OF ADOLESCENT HEALTH. 2021;68(2):277-283.

4017. Castejon Maria, Los Fayos Enrique, Berenguei Rosendo Analysis of relationships between personality factors and the risk of Eating Disorders. REVISTA LATINOAMERICANA DE PSICOLOGIA. 2023;55():46-54.

4018. Modan-Moses Dalit, Levy-Shraga Yael, Pinhas-Hamiel Orit, Kochavi Brigitte, Enoch-Levy Adi, Vered Iris, Stein Daniel High prevalence of vitamin D deficiency and insufficiency in adolescent inpatients diagnosed with eating disorders. INTERNATIONAL JOURNAL OF EATING DISORDERS. 2015;48(6):607-614.

4019. Wales Jackie, Brewin Nicola, Cashmore Rebecca, Haycraft Emma, Baggott Jonathan, Cooper Amy, Arcelus Jon Predictors of Positive Treatment Outcome in People With Anorexia Nervosa Treated in a Specialized Inpatient Unit: The Role of Early Response to Treatment. EUROPEAN EATING DISORDERS REVIEW. 2016;24(5):417-424.

4020. Concina Federica, Pani Paola, Carletti Claudia, Bravo Giulia, Knowles Alessandra, Parpinel Maria, Ronfani Luca, Barbone Fabio Dietary Intake of the Italian PHIME Infant Cohort: How We Are Getting Diet Wrong from as Early as Infancy. NUTRIENTS. 2021;13(12):.

4021. Piazzi Giulia, Prossomariti Anna, Baldassarre Maurizio, Montagna Claudio, Vitaglione Paola, Fogliano Vincenzo, Biagi Elena, Candela Marco, Brigidi Patrizia, Balbi Tiziana, Munarini Alessandra, Belluzzi Andrea, Pariali Milena, Bazzoli Franco, Ricciardiello Luigi A

Mediterranean Diet Mix Has Chemopreventive Effects in a Murine Model of Colorectal Cancer Modulating Apoptosis and the Gut Microbiota. FRONTIERS IN ONCOLOGY. 2019;9():.

4022. Castellanos-Ryan Natalie, Briere Frederic, O'Leary-Barrett Maeve, Banaschewski Tobias, Bokde Arun, Bromberg Uli, Buechel Christian, Flor Herta, Frouin Vincent, Gallinat Juergen, Garavan Hugh, Martinot Jean-Luc, Nees Frauke, Paus Tomas, Pausova Zdenka, Rietschel Marcella, Smolka Michael, Robbins Trevor, Whelan Robert, Schumann Gunter, Conrod Patricia, Consortium IMAGEN The Structure of Psychopathology in Adolescence and Its Common Personality and Cognitive Correlates. JOURNAL OF ABNORMAL PSYCHOLOGY. 2016;125(8):1039-1052.

4023. Castillo Irais, Solano Santos, Sepulveda Ana A controlled study of an integrated prevention program for improving disordered eating and body image among Mexican university students: A 3-month follow-up. EUROPEAN EATING DISORDERS REVIEW. 2019;27(5):541-556.

4024. Fulkerson JA, McGuire MT, Neumark-Sztainer D, Story M, French SA, Perry CL Weight-related attitudes and behaviors of adolescent boys and girls who are encouraged to diet by their mothers. INTERNATIONAL JOURNAL OF OBESITY. 2002;26(12):1579-1587.

4025. Zhang Xinxin, Xu Min, Cai Shuilin, Chen Bei, Lin Hetong, Liu Zhiyu Effects of astaxanthin on microRNA expression in a rat cardiomyocyte anoxia-reoxygenation model. FRONTIERS IN PHARMACOLOGY. 2023;14():.

4026. Lange C., Fjertorp Hanna, Holmer Riitta, Wijk Elin, Wallin Ulf Long-term follow-up study of low-weight avoidant restrictive food intake disorder compared with childhood-onset anorexia nervosa: Psychiatric and occupational outcome in 56 patients. INTERNATIONAL JOURNAL OF EATING DISORDERS. 2019;52(4, SI):435-438.

4027. Shobana S., Bai R., Sudha V, Unnikrishnan R., Pradeepa R., Anjana R., Mohan V Nutrition and Its Link with Diabetes in Asian Indians: Challenges and Solutions. PROCEEDINGS OF THE INDIAN NATIONAL SCIENCE ACADEMY. 2018;84(4):955-963.

4028. Chavez Hernandez Ivonne, Jesus Saucedo-Molina Teresita, Pena Irecta Amanda, Unikel Santoncini Claudia EATING DISORDERS ASSOCIATED RISK FACTORS: TRENDS FROM 2007 TO 2010. REVISTA DE INVESTIGACION CLINICA-CLINICAL AND TRANSLATIONAL INVESTIGATION. 2015;67(1):54-63.

4029. Skovgaard Anne, Bakermans-Kranenburg Marian, Pontoppidan Maiken, Tjornhoj-Thomsen Tine, Madsen Katrine, Voss Ida, Wehner Stine, Pedersen Trine, Finseth Lotte, Taylor Rodney, Tolstrup Janne, Ammitzboll Janni The Infant Health Study - Promoting mental health and healthy weight through sensitive parenting to infants with cognitive, emotional, and regulatory vulnerabilities: protocol for a stepped-wedge cluster-randomized trial and a process evaluation within municipality settings. BMC PUBLIC HEALTH. 2022;22(1):.

4030. Dubern Beatrice, Bisbis Selma, Talbaoui Habiba, Le Beyec Johanne, Tounian Patrick, Lacorte Jean-Marc, Clement Karine Homozygous null mutation of the melanocortin-4 receptor and severe early-onset obesity. JOURNAL OF PEDIATRICS. 2007;150(6):613-617.

4031. Michels Nathalie, De Witte Fien, Di Bisceglie Eline, Seynhaeve Maya, Vandebuerie Tori Green nature effect on stress response and stress eating in the lab: Color versus environmental content. ENVIRONMENTAL RESEARCH. 2021;193():.

4032. McCurley Jessica, Levy Douglas, Rimm Eric, Gelsomin Emily, Anderson Emma, Sanford Jenny, Thorndike Anne Association of Worksite Food Purchases and Employees' Overall Dietary Quality and Health. AMERICAN JOURNAL OF PREVENTIVE MEDICINE. 2019;57(1):87-94.

4033. Booth Charlotte, Spronk Desiree, Grol Maud, Fox Elaine Uncontrolled eating in adolescents: The role of impulsivity and automatic approach bias for food. APPETITE. 2018;120():636-643.

4034. Liu Yixin, Cao Yuping The Effect of Exercise Motivation on Eating Disorders in Bodybuilders in Social Networks: The Mediating Role of State Anxiety. COMPUTATIONAL AND MATHEMATICAL METHODS IN MEDICINE. 2022;2022():.

4035. Sevilla Guillermo, Barcelo Guido Olga, Cruz Maria, Fernandez Ascension, Alejo Lidia, Ramirez Goercke Maria, Perez-Ruiz Margarita Remotely Supervised Exercise during the COVID-19 Pandemic versus in-Person-Supervised Exercise in Achieving Long-Term Adherence to a Healthy Lifestyle. INTERNATIONAL JOURNAL OF ENVIRONMENTAL RESEARCH AND PUBLIC HEALTH. 2021;18(22):.

4036. Bulik Cynthia If we build it, will they come? Commentary on ``Preventing eating disorders and disordered eating in high-risk families{''}. INTERNATIONAL JOURNAL OF EATING DISORDERS. 2023;56(3):535-537.

4037. Hirschtritt Matthew, Lee Paul, Pauls David, Dion Yves, Grados Marco, Illmann Cornelia, King Robert, Sandor Paul, McMahon William, Lyon Gholson, Cath Danielle, Kurlan Roger, Robertson Mary, Osiecki Lisa, Scharf Jeremiah, Mathews Carol, Consor Tourette Lifetime Prevalence, Age of Risk, and Genetic Relationships of Comorbid Psychiatric Disorders in Tourette Syndrome. JAMA PSYCHIATRY. 2015;72(4):325-333.

4038. Vanderkruik Rachel, Ellison Kalin, Kanamori Margaux, Freeman Marlene, Cohen Lee, Stice Eric ADAPTING A BODY IMAGE AND DISORDERED EATING PREVENTION PROGRAM FOR THE PERINATAL PERIOD. ANNALS OF BEHAVIORAL MEDICINE. 2022;56(SUPP 1, 1, SI):S105.

4039. Gonzales Victoria, Lodeiro Carlos, Macias Amanda, Francis Denease, Gutierrez Fatima, Pathak Indu Incidence of Refeeding Syndrome in Pediatric Inpatients at the US-Mexico Border. SOUTHERN MEDICAL JOURNAL. 2021;114(6):351-355.

4040. Bamber D, Cockerill IM, Carroll D The pathological status of exercise dependence. BRITISH JOURNAL OF SPORTS MEDICINE. 2000;34(2):125-132.

4041. Mougharbel Fatima, Valois Darcie, Lamb Megan, Buchholz Annick, Obeid Nicole, Flament Martine, Goldfield Gary Mediating role of disordered eating in the relationship between screen time and BMI in adolescents: longitudinal findings from the Research on Eating and Adolescent Lifestyles (REAL) study. PUBLIC HEALTH NUTRITION. 2020;23(18):3336-3345.

4042. Milligan RAK, Burke V, Beilin LJ, Richards J, Dunbar D, Spencer M, Balde E, Gracey MP Health-related behaviours and psycho-social characteristics of 18 year-old Australians. SOCIAL SCIENCE \& MEDICINE. 1997;45(10):1549-1562.

4043. Hudson JI, Chase EA, Pope HG Eye movement desensitization and reprocessing in eating disorders: Caution against premature acceptance. INTERNATIONAL JOURNAL OF EATING DISORDERS. 1998;23(1):1-5.

4044. Youssef G, Plancherel B, Laget J, Corcos M, Flament MF, Halfon O Personality trait risk factors for attempted suicide among young women with eating disorders. EUROPEAN PSYCHIATRY. 2004;19(3):131-139.

4045. Fukunishi I, Kitaoka T, Shirai T, Watanabe S Two male patients with bulimia nervosa having onset during hemodialysis therapy. PSYCHOSOMATICS. 1997;38(5):511-512.

4046. Hofseth Lorne, Hebert James, Chanda Anindya, Chen Hexin, Love Bryan, Pena Maria, Murphy E., Sajish Mathew, Sheth Amit, Buckhaults Phillip, Berger Franklin Early-onset colorectal cancer: initial clues and current views. NATURE REVIEWS GASTROENTEROLOGY \& HEPATOLOGY. 2020;17(6):352-364.

4047. Sharp William, Stubbs Kathryn Avoidant/restrictive food intake disorder: A diagnosis at the intersection of feeding and eating disorders necessitating subtype differentiation. INTERNATIONAL JOURNAL OF EATING DISORDERS. 2019;52(4, SI):398-401.

4048. Oltmans Emmeline, Confectioner Kaizeen, Jonkers Ruud, Kerkhoffs Gino, Moen Maarten, Verhagen Evert, Wylleman Paul, Gouttebarge Vincent A 12-month prospective cohort study on symptoms of mental health disorders among Dutch former elite athletes. PHYSICIAN AND SPORTSMEDICINE. 2022;50(2):123-131.

4049. Keski-Rahkonen Anna, Hoek Hans, Susser Ezra, Linna Milla, Sihvola Elina, Raevuori Anu, Bulik Cynthia, Kaprio Jaakko, Rissanen Aila Epidemiology and course of anorexia nervosa in the community. AMERICAN JOURNAL OF PSYCHIATRY. 2007;164(8):1259-1265.

4050. Moreno Ruge Angelica, Londono-Perez Constanza Family and Personal Predictors of Eating Disorders in Young People. ANALES DE PSICOLOGIA. 2017;33(2):235-242.

4051. Selby Edward, Coniglio Kathryn Positive Emotion and Motivational Dynamics in Anorexia Nervosa: A Positive Emotion Amplification Model (PE-AMP). PSYCHOLOGICAL REVIEW. 2020;127(5):853-890.

4052. Almasi-Dooghaee Mostafa, Vahedi Taravat, Vahedi Nooshin, Zamani Babak Sour aversion in frontotemporal dementia: a case report and review on physiologic-anatomic mechanisms. NEUROCASE. 2021;27(2):178-180.

4053. Cornelius Jason, Tippmann-Peikert Maja, Slocumb Nancy, Frerichs Courtney, Silber Michael Impulse Control Disorders with the use of Dopaminergic Agents in Restless Legs Syndrome: a Case-Control Study. SLEEP. 2010;33(1):81-87.

4054. Buckingham-Howes Stacy, Armstrong Bridget, Pejsa-Reitz Megan, Wang Yan, Witherspoon Dawn, Hager Erin, Black Maureen BMI and disordered eating in urban, African American, adolescent girls: The mediating role of body dissatisfaction. EATING BEHAVIORS. 2018;29():59-63.

4055. Couturier Jennifer, Sy Alice, Johnson Natasha, Findlay Sheri Bone Mineral Density in Adolescents With Eating Disorders Exposed to Selective Serotonin Reuptake Inhibitors. EATING DISORDERS. 2013;21(3):238-248.

4056. Dahlgren Camilla, Wisting Line Transitioning from DSM-IV to DSM-5: A systematic review of eating disorder prevalence assessment. INTERNATIONAL JOURNAL OF EATING DISORDERS. 2016;49(11):975-997.

4057. Richards Katie, Flynn Michaela, Austin Amelia, Lang Katie, Allen Karina, Bassi Ranjeet, Brady Gabrielle, Brown Amy, Connan Frances, Franklin-Smith Mary, Glennon Danielle, Grant Nina, Jones William, Kali Kuda, Koskina Antonia, Mahony Kate, Mountford Victoria, Nunes Nicole, Schelhase Monique, Serpell Lucy, Schmidt Ulrike Assessing implementation fidelity in the First Episode Rapid Early Intervention for Eating Disorders service model. BJPSYCH OPEN. 2021;7(3):.

4058. Slater Amy, Tiggemann Marika Time Since Menarche and Sport Participation as Predictors of Self-Objectification: A Longitudinal Study of Adolescent Girls. SEX ROLES. 2012;67(9-10):571-581.

4059. Rodriguez-Perez Robinson, Correa-Matos Nancy, Valdes-Valderrama Angelica, Alexis Rodriguez-Cruz Luis, Rodriguez Maria A Qualitative Study of Puerto Rican Parent and Child Perceptions Regarding Eating Patterns. JOURNAL OF NUTRITION EDUCATION AND BEHAVIOR. 2019;51(5):608-615.

4060. Van Tine Meredith, McNicholas Fiona, Safer Debra, Agras W. Follow-up of selective eaters from childhood to adulthood. EATING BEHAVIORS. 2017;26():61-65.

4061. De Panfilis Chlara, Torre Mariateresa, Cero Sara, Salvatore Paola, Dall'Aglio Elisabetta, Marchesi Carlo, Cabrino Chiara, Aprile Sonja, Maggini Carlo Personality and attrition from behavioral weight-loss treatment for obesity. GENERAL HOSPITAL PSYCHIATRY. 2008;30(6):515-520.

4062. Manuel Ballesteros Arribas Juan, Dal-Re Saavedra Marian, Perez-Farinos Napoleon, Villar Villalba Carmen The Spanish strategy for nutrition, physical activity and the prevention of obesity (NAOS strategy). REVISTA ESPANOLA DE SALUD PUBLICA. 2007;81(5):443-449.

4063. Pas Marinus, Koopmans Sietse-Jan, Kruijt Leo, Boeren Sjef, Smits Mari Changes in Plasma Protein Expression Indicative of Early Diet-induced Metabolic Disease in Male Pigs (Sus scrofa). COMPARATIVE MEDICINE. 2018;68(4):286-293.

4064. Al-sheyab Nihaya, Gharaibeh Tamer, Kheirallah Khalid Relationship between Peer Pressure and Risk of Eating Disorders among Adolescents in Jordan. JOURNAL OF OBESITY. 2018;2018():.

4065. Varnado-Sullivan Paula, Parr Francoise, O'Grady Megan, Savoy Sarah Educators' views of eating disorder prevention programs. EATING AND WEIGHT DISORDERS-STUDIES ON ANOREXIA BULIMIA AND OBESITY. 2013;18(2):143-150.

4066. Mascitelli L., Pezzetta F. Unhealthy lifestyle, statins and the secondary prevention of cardiovascular disease. INTERNAL MEDICINE JOURNAL. 2007;37(6):427-428.

4067. Schmidt U, Tiller J, Blanchard M, Andrews B, Treasure J Is there a specific trauma precipitating anorexia nervosa?. PSYCHOLOGICAL MEDICINE. 1997;27(3):523-530.

4068. Quittkat Hannah, Dusing Rainer, Holtmann Friederike-Johanna, Buhlmann Ulrike, Svaldi Jennifer, Vocks Silja Perceived Impact of Covid-19 Across Different Mental Disorders: A Study on Disorder-Specific Symptoms, Psychosocial Stress and Behavior. FRONTIERS IN PSYCHOLOGY. 2020;11():.

4069. Goss Kenneth, Allan Steven Shame, Pride and Eating Disorders. CLINICAL PSYCHOLOGY \& PSYCHOTHERAPY. 2009;16(4, SI):303-316.

4070. Valk Eline, Akker Erica, Savas Mesut, Kleinendorst Lotte, Visser Jenny, Van Haelst Mieke, Sharma A., Rossum Elisabeth A comprehensive diagnostic approach to detect underlying causes of obesity in adults. OBESITY REVIEWS. 2019;20(6):795-804.

4071. Dadaeva Valida, Aleksandrov Aleksander, Orlova Aleksandra, Drapkina Oxana Sleep and Obesity: Mechanisms of Association. RATIONAL PHARMACOTHERAPY IN CARDIOLOGY.

2020;16(4):564-570.

4072. Lopeza Sergio, Bermudez Beatriz, Paz Sergio, Jaramillo Sara, Abia Rocio, Muriana Francisco Virgin Olive Oil and Hypertension. CURRENT VASCULAR PHARMACOLOGY. 2016;14(4):323-329.

4073. Wang Youfa, Yan Alice, Shi Xinyu, Wang Huijun, Wang Zhiyong, Gittelsohn Joel, Xu Fei Child and parental perspectives on diet and physical activity decisions: implications for childhood obesity prevention in China. ASIA PACIFIC JOURNAL OF CLINICAL NUTRITION. 2017;26(5):888-898.

4074. Horstkotte E. Too Fat, Too Thin? - Bodily Self-Perception and Eating Habits of Teenagers in Bremen. GESUNDHEITSWESEN. 2011;73(2):73-77.

4075. Wadden TA, Brownell KD, Foster GD Obesity: Responding to the global epidemic. JOURNAL OF CONSULTING AND CLINICAL PSYCHOLOGY. 2002;70(3):510-525.

4076. Melisse Bernou, Blankers Matthijs, Beurs Edwin, Furth Eric Correlates of eating disorder pathology in Saudi Arabia: BMI and body dissatisfaction. JOURNAL OF EATING DISORDERS. 2022;10(1):.

4077. Weigel Angelika, Gumz Antje, Uhlenbusch Natalie, Wegscheider Karl, Romer Georg, Loewe Bernd Preventing eating disorders with an interactive gender-adapted intervention program in schools: Study protocol of a randomized controlled trial. BMC PSYCHIATRY. 2015;15():.

4078. Sigurdh Jeanette, Allard Per, Spigset Olav, Hagglof Bruno Platelet serotonin transporter and 5-HT2A receptor binding in adolescents with eating disorders. INTERNATIONAL JOURNAL OF NEUROSCIENCE. 2013;123(5):333-338.

4079. Noort Betteke, Lohmar Sylvie, Pfeiffer Ernst, Lehmkuhl Ulrike, Winter Sibylle, Kappel Viola Clinical characteristics of early onset anorexia nervosa. EUROPEAN EATING DISORDERS REVIEW. 2018;26(5):519-525.

4080. Moccia Lorenzo, Conte Eliana, Ambrosecchia Marianna, Janiri Delfina, Di Pietro Salvatore, De Martin Valentina, Di Nicola Marco, Rinaldi Lucio, Sani Gabriele, Gallese Vittorio, Janiri Luigi Anomalous self-experience, body image disturbance, and eating

disorder symptomatology in first-onset anorexia nervosa. EATING AND WEIGHT DISORDERS-STUDIES ON ANOREXIA BULIMIA AND OBESITY. 2022;27(1):101-108.

4081. Berman Margit, Morton Stephanie, Hegel Mark Health at Every Size and Acceptance and Commitment Therapy for Obese, Depressed Women: Treatment Development and Clinical Application. CLINICAL SOCIAL WORK JOURNAL. 2016;44(3):265-278.

4082. Castro-Fornieles Josefina, Serna Elena, Calvo Anna, Pariente Jose, Andres-Perpina Susana, Plana Maria, Romero Sonia, Flamarique Itziar, Garriz Miguel, Bargallo Nuria Cortical thickness 20 years after diagnosis of anorexia nervosa during adolescence. EUROPEAN ARCHIVES OF PSYCHIATRY AND CLINICAL NEUROSCIENCE. 2021;271(6):1133-1139.

4083. Gibbings Nicole, Kurdyak Paul, Colton Patricia, Shah Baiju Diabetic Ketoacidosis and Mortality in People With Type 1 Diabetes and Eating Disorders. DIABETES CARE. 2021;44(8):1783-1787.

4084. Panagiotakos D., Georgousopoulou E., Pitsavos C., Chrysohoou C., Skoumas I., Pitaraki E., Georgiopoulos G., Ntertimani M., Christou A., Stefanadis C., Grp ATTICA Exploring the path of Mediterranean diet on 10-year incidence of cardiovascular disease: The ATTICA study (2002-2012). NUTRITION METABOLISM AND CARDIOVASCULAR DISEASES. 2015;25(3):327-335.

4085. Ma Joyce An exploratory study of the impact of an adolescent's eating disorder on Chinese parents' well-being, marital life and perceived family functioning in Shenzhen, China: implications for social work practice. CHILD \& FAMILY SOCIAL WORK. 2011;16(1):33-42.

4086. Won Ying, Christensen Kara, Forbush Kelsie Habitual adaptive emotion regulation moderates the association between maladaptive emotion regulation and eating disorder symptoms, but not clinical impairment. EATING AND WEIGHT DISORDERS-STUDIES ON ANOREXIA BULIMIA AND OBESITY. 2022;27(7):2629-2639.

4087. Barrack Michelle, Van Loan Marta Proper nutrition can prevent negative health outcomes in young female athletes. CALIFORNIA AGRICULTURE. 2011;65(3):124-129.

4088. Troncone Alda, Chianese Antonietta, Cascella Crescenzo, Zanfardino Angela, Piscopo Alessia, Rollato Serena, Iafusco Dario Eating Problems in Youths with Type 1 Diabetes During and After Lockdown in Italy: An 8-Month Follow-Up Study. JOURNAL OF CLINICAL

PSYCHOLOGY IN MEDICAL SETTINGS. 2023;30(1):227-237.

4089. Ataollahi Maryam, Sedighi Sedigheh, Masoumi Seyyedeh Nutritional and Unhealthy Behaviors in Women With and Without Breast Cancer. IRANIAN RED CRESCENT MEDICAL JOURNAL. 2014;16(9):.

4090. Bunio Lindsey, Battles Jennifer, Loverich Tamara The nuances of emotion regulation difficulties and mindfulness in food addiction. ADDICTION RESEARCH \& THEORY. 2021;29(1):11-17.

4091. Sebastiao Joana, Sampaio Daniel, Barbosa Maria Prevalence and Risk Factors for Eating Disorders in Adolescents Aged Between 12 to 18 Years Old in Manteigas, Portugal. PSILOGOS. 2018;16(2):31-45.

4092. Lee Hansongyi, Kim Jieun, Lim Hyunjung Coexistence of metabolic syndrome and osteopenia associated with social inequalities and unhealthy lifestyle among postmenopausal women in South Korea: the 2008 to 2011 Korea National Health and Nutritional Examination Survey (KNHANES). MENOPAUSE-THE JOURNAL OF THE NORTH AMERICAN MENOPAUSE SOCIETY. 2020;27(6):668-678.

4093. Micali N., De Stavola B., Ploubidis G., Simonoff E., Treasure J., Field A. Adolescent eating disorder behaviours and cognitions: gender-specific effects of child, maternal and family risk factors. BRITISH JOURNAL OF PSYCHIATRY. 2015;207(4):320-327.

4094. Haliczer Lauren, Harnedy Lauren, Oakley Marykate, Dixon-Gordon Katherine Clarifying the Role of Multiple Self-Damaging Behaviors in the Association Between Emotion Dysregulation and Suicide Risk Among College Students. JOURNAL OF PRIMARY PREVENTION. 2021;42(5):473-492.

4095. Austin Amelia, Flynn Michaela, Shearer James, Long Mike, Allen Karina, Mountford Victoria, Glennon Danielle, Grant Nina, Brown Amy, Franklin-Smith Mary, Schelhase Monique, Jones William, Brady Gabrielle, Nunes Nicole, Connan Frances, Mahony Kate, Serpell Lucy, Schmidt Ulrike The First Episode Rapid Early Intervention for Eating Disorders - Upscaled study: Clinical outcomes. EARLY INTERVENTION IN PSYCHIATRY. 2022;16(1):97-105.

4096. Pomerleau CS, Brouwer RJN, Jones LT Weight concerns in women smokers during pregnancy and postpartum. ADDICTIVE BEHAVIORS. 2000;25(5):759-767.

4097. Melo Jayanne, Dourado Bruna, Menezes Risia, Longo-Silva Giovana, Silveira Jonas Early onset of overweight among children from low-income families: The role of exclusive breastfeeding and maternal intake of ultra-processed food. PEDIATRIC OBESITY. 2021;16(12):.

4098. Valentin M., Radon L., Duclos J., Curt F., Godart N. Bipolar disorders and anorexia nervosa: A clinical study. ENCEPHALE-REVUE DE PSYCHIATRIE CLINIQUE BIOLOGIQUE ET THERAPEUTIQUE. 2019;45(1):27-33.

4099. Shimul Anwar, Cheah Isaac, Lou Andrew Regulatory focus and junk food avoidance: The influence of health consciousness, perceived risk and message framing. APPETITE. 2021;166():.

4100. Schwartz Marlene, Thomas Jennifer, Bohan Kristin, Vartanian Lenny Intended and unintended effects of an eating disorder educational program: Impact of presenter identity. INTERNATIONAL JOURNAL OF EATING DISORDERS. 2007;40(2):187-192.

4101. Lopez L., Portela M., Soler C. Nutrient intake in women with pagophagia and other forms of pica during the pregnancy. NUTRICION HOSPITALARIA. 2007;22(6):641-647.

4102. Gonzalez-Aragon Pineda Alvaro, Garcia-Perez Alvaro, Francisco Gomez-Clavel Jose Caries experience in adolescents 13-14 years with and without erosive tooth wear: a case-control study. JOURNAL OF CLINICAL PEDIATRIC DENTISTRY. 2022;46(5):31-37.

4103. De Bourdeaudhuij I., Van Cauwenberghe E., Spittaels H., Oppert J., Rostami C., Brug J., Van Lenthe F., Lobstein T., Maes L. School-based interventions promoting both physical activity and healthy eating in Europe: a systematic review within the HOPE project. OBESITY REVIEWS. 2011;12(3):205-216.

4104. Beilharz Francesca, Phillipou Andrea, Castle David, Jenkins Zoe, Cistullo Leonardo, Rossell Susan Dysmorphic concern in anorexia nervosa: Implications for recovery. PSYCHIATRY RESEARCH. 2019;273():657-661.

4105. MCCREA C AN INVESTIGATION OF THE USEFULNESS OF VIDEOFEEDBACK IN THE TREATMENT OF OBESITY. CLINICAL PSYCHOLOGY \& PSYCHOTHERAPY. 1995;2(3):192-198.

4106. Naab Silke, Schlegl Sandra, Korte Alexander, Heuser Joerg, Fumi Markus, Fichter Manfred, Cuntz Ulrich, Voderholzer Ulrich Effectiveness of a multimodal inpatient treatment for adolescents with anorexia nervosa in comparison with adults: an analysis of a specialized inpatient setting. EATING AND WEIGHT DISORDERS-STUDIES ON ANOREXIA BULIMIA AND OBESITY. 2013;18(2):167-173.

4107. Abebe Dawit, Lien Lars, Soest Tilmann The development of bulimic symptoms from adolescence to young adulthood in females and males: A population-based longitudinal cohort study. INTERNATIONAL JOURNAL OF EATING DISORDERS. 2012;45(6):737-745.

4108. Tabuchi K, Takahashi K, Ito Z, Hara A, Wada T, Kusakari J Effect of 7-nitroindazole upon cochlear dysfunction induced by transient local anoxia. ANNALS OF OTOLOGY RHINOLOGY AND LARYNGOLOGY. 2000;109(8, 1):715-719.

4109. Bentz Mette, Guldberg Johanne, Vangkilde Signe, Pedersen Tine, Plessen Kerstin, Jepsen Jens Heightened Olfactory Sensitivity in Young Females with Recent-Onset Anorexia Nervosa and Recovered Individuals. PLOS ONE. 2017;12(1):.

4110. Sousa Taciana, Santos Luana, Pinto Costa Hellena, Carvalho Renata, Pereira Cardoso Factors Associated with the Consumption of Food Markers of Unhealthy Diet Among School Children in Situations of High Health Vulnerability. JOURNAL OF TROPICAL PEDIATRICS. 2019;65(6):576-582.

4111. Artaud Fanny, Lee Pei-Chen, Mangone Graziella, Vidailhet Marie, Corvol Jean-Christophe, Elbaz Alexis Longitudinal association between dopamine agonists and weight in Parkinson's disease. PARKINSONISM \& RELATED DISORDERS. 2020;80():158-164.

4112. Thein-Nissenbaum Jill Long term consequences of the female athlete triad. MATURITAS. 2013;75(2):107-112.

4113. Lieberman Melissa, Houser Melissa, Voyer Anne-Pier, Grady Shelley, Katzman Debra Children with avoidant/restrictive food intake disorder and anorexia nervosa in a tertiary care pediatric eating disorder program: A comparative study. INTERNATIONAL JOURNAL

OF EATING DISORDERS. 2019;52(3):239-245.

4114. AMANO S, OHASHI M, ISHIKO T, HAZAMA F HUMORAL-FACTORS DERIVED FROM GLIAL-CELLS PROTECT CULTURED NEUROBLASTOMA-CELLS AGAINST GLUTAMATE TOXICITY. BRAIN RESEARCH. 1994;645(1-2):347-350.

4115. Riley Elizabeth, Davis Heather, Combs Jessica, Jordan Carol, Smith Gregory Nonsuicidal Self-injury as a Risk Factor for Purging Onset: Negatively Reinforced Behaviours that Reduce Emotional Distress. EUROPEAN EATING DISORDERS REVIEW. 2016;24(1):78-82.

4116. Falk LW, Sobal J, Bisogni CA, Connors M, Devine CM Managing healthy eating: Definitions, classifications, and strategies. HEALTH EDUCATION \& BEHAVIOR. 2001;28(4):425-439.

4117. Tabuchi K, Tsuji S, Wada T, Ito Z, Hara A, Kusakari J Effect of ketamine, dextromethorphan, and MK-801 on cochlear dysfunction induced by transient ischemia. ANNALS OF OTOLOGY RHINOLOGY AND LARYNGOLOGY. 2002;111(1):44-49.

4118. Wang Ruining, Ye Baojuan, Wang Peiyi, Tang Chunyan, Yang Qiang Coronavirus stress and overeating: the role of anxiety and COVID-19 burnout. JOURNAL OF EATING DISORDERS. 2022;10(1):.

4119. Wertheim EH Prevention of eating disordered behaviours and body image concerns: How should we approach research in the field?. AUSTRALIAN JOURNAL OF PSYCHOLOGY. 2001;53(S):180-181.

4120. Kouvari Matina, Panagiotakos Demosthenes, Yannakoulia Mary, Georgousopoulou Ekavi, Critselis Elena, Chrysohoou Christina, Tousoulis Dimitrios, Pitsavos Christos, Skoumas Y., Katinioti N., Papadimitriou L., Masoura C., Vellas S., Lentzas Y., Kambaxis M., Paliou K., Metaxa V, Skourlis N., Papanikolaou C., Kalogeropoulou A., Pitaraki E., Laskaris A., Hatzigeorgiou M., Grekas A., Kokkou E., Vassiliadou C., Dedousis G., Toutouza-Giotsa M., Tselika C., Poulopouloou S., Toutouza M., Investigators ATTICA Transition from metabolically benign to metabolically unhealthy obesity and 10-year cardiovascular disease incidence: The ATTICA cohort study. METABOLISM-CLINICAL AND EXPERIMENTAL. 2019;93():18-24.

4121. Dukhi Natisha, Sartorius Benn, Taylor Myra A behavioural change intervention study for the prevention of childhood obesity in South Africa: protocol for a randomized controlled trial. BMC PUBLIC HEALTH. 2020;20(1):.

4122. Gagnon Cynthia, Aime Annie, Belanger Claude Predictors of Comorbid Eating Disorders and Diabetes in People with Type 1 and Type 2 Diabetes. CANADIAN JOURNAL OF DIABETES. 2017;41(1):52-57.

4123. Favieri Francesca, Marini Andrea, Casagrande Maria Emotional Regulation and Overeating Behaviors in Children and Adolescents: A Systematic Review. BEHAVIORAL SCIENCES. 2021;11(1):.

4124. Parnarouskis Lindsey, Gearhardt Ashley, Mason Ashley, Adler Nancy, Laraia Barbara, Epel Elissa, Leung Cindy Association of Food Insecurity and Food Addiction Symptoms: A Secondary Analysis of Two Samples of Low-Income Female Adults. JOURNAL OF THE ACADEMY OF NUTRITION AND DIETETICS. 2022;122(10):1885-1892.

4125. Conley Terri, Garza Megan Gender and Sequelae of Child Versus Adult Onset of Sexual Victimization: Body Mass, Binge Eating, and Promiscuity. JOURNAL OF APPLIED SOCIAL PSYCHOLOGY. 2011;41(11):2551-2572.

4126. Hill AJ Does dieting make you fat?. BRITISH JOURNAL OF NUTRITION. 2004;92(1):S15-S18.

4127. Vanderkruik Rachel, Ellison Kalin, Kanamori Margaux, Freeman Marlene, Cohen Lee, Stice Eric Body dissatisfaction and disordered eating in the perinatal period: an underrecognized high-risk timeframe and the opportunity to intervene. ARCHIVES OF WOMENS MENTAL HEALTH. 2022;25(4):739-751.

4128. Centis Elena, Marzocchi Rebecca, Di Domizio Silvia, Ciaravella Maria, Marchesini Giulio The Effect of Lifestyle Changes in Non-Alcoholic Fatty Liver Disease. DIGESTIVE DISEASES. 2010;28(1):267-273.

4129. Goncalves Sonia, Ribeiro Amadeu, Felix Silvia, Gomes Antonio Does weight change relate to psychological variables and eating behaviours in combat sports?. EATING AND WEIGHT DISORDERS-STUDIES ON ANOREXIA BULIMIA AND OBESITY. 2021;26(3):921-930.

4130. Ellard Kristen, Fairholme Christopher, Boisseau Christina, Farchione Todd, Barlow David Unified Protocol for the Transdiagnostic Treatment of Emotional Disorders: Protocol Development and Initial Outcome Data. COGNITIVE AND BEHAVIORAL PRACTICE. 2010;17(1):88-101.

4131. Broen Martijn, Duits Annelien, Visser-Vandewalle Veerle, Temel Yasin, Winogrodzka Ania Impulse control and related disorders in Parkinson's disease patients treated with bilateral subthalamic nucleus stimulation: A review. PARKINSONISM \& RELATED DISORDERS. 2011;17(6):413-417.

4132. Heinicke Brooke, Paxton Susan, McLean Sian, Wertheim Eleanor Internet-delivered targeted group intervention for body dissatisfaction and disordered eating in adolescent girls: A randomized controlled trial. JOURNAL OF ABNORMAL CHILD PSYCHOLOGY. 2007;35(3):379-391.

4133. Sirmans Susan, Pate Kristen Epidemiology, diagnosis, and management of polycystic ovary syndrome. CLINICAL EPIDEMIOLOGY. 2014;6():1-13.

4134. Nakazawa Masato, Tang Akaysha Adult aggression during an initial social encounter: effects of neonatal anoxia and relation to juvenile open-field activity. NEUROSCIENCE LETTERS. 2006;408(2):119-123.

4135. March J., Suess A., Prieto M., Escudero M., Nebot M., Cabeza E., Pallicer A. Dietary behavior disorders: Opinions and expectancies on prevention and treatment strategies from the perspective of the several social actors. NUTRICION HOSPITALARIA. 2006;21(1):4-12.

4136. Charlton Olivia, Dickison Philippa, Smith Saxon, Roger Simon Nail clubbing in laxative abuse: case report and review of the literature. JOURNAL OF EATING DISORDERS. 2019;7():.

4137. Frerichs Leah, Intolubbe-Chmil Loren, Brittin Jeri, Teitelbaum Kiersten, Trowbridge Matthew, Huang Terry Children's Discourse of Liked, Healthy, and Unhealthy Foods. JOURNAL OF THE ACADEMY OF NUTRITION AND DIETETICS. 2016;116(8):1323-1331.

4138. Angkurawaranon C., Jiraporncharoen W., Chenthanakij B., Doyle P., Nitsch D. Urbanization and non-communicable disease in Southeast Asia: a review of current evidence. PUBLIC HEALTH. 2014;128(10):886-895.

4139. Carter Frances, Boden Joseph, Jordan Jennifer, McIntosh Virginia, Bulik Cynthia, Joyce Peter Weight suppression predicts total weight gain and rate of weight gain in outpatients with anorexia nervosa. INTERNATIONAL JOURNAL OF EATING DISORDERS. 2015;48(7):912-918.

4140. Murciano Manuel, Biancone Davide, De Luca Francesca, Piras Marafon Denise, Guido Cristiana, Spalice Alberto Breastfeeding in Pediatric Acute-Onset Neuropsychiatric Syndrome: An Italian Observational Study. FRONTIERS IN PEDIATRICS. 2021;9():.

4141. Becker AE, Franko DL, Nussbaum K, Herzog DB Secondary prevention for eating disorders: The impact of education, screening, and referral in a college-based screening program. INTERNATIONAL JOURNAL OF EATING DISORDERS. 2004;36(2):157-162.

4142. Leenaerts N., Ceccarini J., Sunaert S., Vrieze E. Striatal cerebral blood flow changes in patients with recent-onset bulimia nervosa and alcohol use disorder. EUROPEAN NEUROPSYCHOPHARMACOLOGY. 2021;53(1):S7-S8.

4143. Andrews Kyle, Silk Kami, Eneli Ihuoma Parents as Health Promoters: A Theory of Planned Behavior Perspective on the Prevention of Childhood Obesity. JOURNAL OF HEALTH COMMUNICATION. 2010;15(1):95-107.

4144. Lask B, Bryant-Waugh R, Wright F, Campbell M, Willoughby K, Waller G Family physician consultation patterns indicate high risk for early-onset anorexia nervosa. INTERNATIONAL JOURNAL OF EATING DISORDERS. 2005;38(3):269-272.

4145. Le Grange Daniel, O'Connor Meredith, Hughes Elizabeth, Macdonald Jacqui, Little Keriann, Olsson Craig Developmental Antecedents of Abnormal Eating Attitudes and Behaviors in Adolescence. INTERNATIONAL JOURNAL OF EATING DISORDERS. 2014;47(7, SI):813-824.

4146. Goncalves Sonia, Machado Barbara, Martins Carla, Machado Paulo Eating and Weight/Shape Criticism as a Specific Life-Event Related to Bulimia Nervosa: A Case Control Study. JOURNAL OF PSYCHOLOGY. 2014;148(1):61-72.

4147. Levine Michael, Smolak Linda The role of protective factors in the prevention of negative body image and disordered eating. EATING DISORDERS. 2016;24(1, SI):39-46.

4148. Markowitz Jessica, Butler Deborah, Volkening Lisa, Antisdel Jeanne, Anderson Barbara, Laffel Lori Brief Screening Tool for Disordered Eating in Diabetes Internal consistency and external validity in a contemporary sample of pediatric patients with type 1 diabetes. DIABETES CARE. 2010;33(3):495-500.

4149. Sander Johanna, Moessner Markus, Bauer Stephanie Depression, Anxiety and Eating Disorder-Related Impairment: Moderators in Female Adolescents and Young Adults. INTERNATIONAL JOURNAL OF ENVIRONMENTAL RESEARCH AND PUBLIC HEALTH. 2021;18(5):.

4150. Ahn Jaeun, Lee Jung-Hyun, Jung Young-Chul Predictors of Suicide Attempts in Individuals with Eating Disorders. SUICIDE AND LIFE-THREATENING BEHAVIOR. 2019;49(3):789-797.

4151. Ganson Kyle, Cunningham Mitchell, Pila Eva, Rodgers Rachel, Murray Stuart, Nagata Jason ``Bulking and cutting{''} among a national sample of Canadian adolescents and young adults. EATING AND WEIGHT DISORDERS-STUDIES ON ANOREXIA BULIMIA AND OBESITY. 2022;27(8):3759-3765.

4152. Akillioglu Tugce, Bas Murat, Kose Gizem Restrained, emotional eating and depression can be a risk factor for metabolic syndrome. NUTRICION HOSPITALARIA. 2022;39(6):1264-1271.

4153. Stice E, Cameron RP, Killen JD, Hayward C, Taylor CB Naturalistic weight-reduction efforts prospectively predict growth in relative weight and onset of obesity among female adolescents. JOURNAL OF CONSULTING AND CLINICAL PSYCHOLOGY. 1999;67(6):967-974.

4154. Ferrari Manuela Understanding the feasibility of integrating the eating disorders and obesity fields: the beyond obesity and disordered eating in youth (BODY) Study. EATING AND WEIGHT DISORDERS-STUDIES ON ANOREXIA BULIMIA AND OBESITY. 2015;20(2):257-269.

4155. Lin EHB, Katon W, Von Korff M, Rutter C, Simon GE, Oliver M, Ciechanowski P, Ludman EJ, Bush T, Young B Relationship of depression and diabetes self-care, medication adherence, and preventive care. DIABETES CARE. 2004;27(9):2154-2160.

4156. Jacka Felice, Kremer Peter, Berk Michael, Silva-Sanigorski Andrea, Moodie Marjorie, Leslie Eva, Pasco Julie, Swinburn Boyd A Prospective Study of Diet Quality and Mental

Health in Adolescents. PLOS ONE. 2011;6(9):.

4157. Zorbas Christina, Lee Amanda, Peeters Anna, Lewis Meron, Landrigan Timothy, Backholer Kathryn Streamlined data-gathering techniques to estimate the price and affordability of healthy and unhealthy diets under different pricing scenarios. PUBLIC HEALTH NUTRITION. 2021;24(1):1-11.

4158. Raith Anna-Marie, Haemmerling Marie, Klein Sabrina, Peitz Diana, Knaevelsrud Christine, Zagorscak Pavle Promotion of self-esteem in the universal prevention of eating disorders. Pilot study of an internet-based intervention in a sample of students. PSYCHOTHERAPEUT. 2021;66(4, SI):275-281.

4159. Kawa I, Carter JD, Joyce PR, Doughty CJ, Frampton CM, Wells JE, Walsh AES, Olds RJ Gender differences in bipolar disorder: age of onset, course, comorbidity, and symptom presentation. BIPOLAR DISORDERS. 2005;7(2):119-125.

4160. Kern David, Auchincloss Amy, Stehr Mark, Roux Ana, Moore Kari, Kanter Genevieve, Robinson Lucy Neighborhood price of healthier food relative to unhealthy food and its association with type 2 diabetes and insulin resistance: The multi-ethnic study of atherosclerosis. PREVENTIVE MEDICINE. 2018;106():122-129.

4161. Gonzalez Marcela, Penelo Eva, Gutierrez Teresa, Raich Rosa Disordered Eating Prevention Programme in Schools: A 30-Month Follow-up. EUROPEAN EATING DISORDERS REVIEW. 2011;19(4):349-356.

4162. Monni Alessandra, Scalas L. Health Risk Behaviour Inventory Validation and its Association with Self-regulatory Dispositions. JOURNAL OF CLINICAL PSYCHOLOGY IN MEDICAL SETTINGS. 2022;29(4):861-874.

4163. Cooney Megan, Lieberman Melissa, Guimond Tim, Katzman Debra Clinical and psychological features of children and adolescents diagnosed with avoidant/restrictive food intake disorder in a pediatric tertiary care eating disorder program: a descriptive study. JOURNAL OF EATING DISORDERS. 2018;6():.

4164. Decsamer Suhong, Piaseu Noppawan, Maneesriwongul Wantana, Orathai Pisamai, Schepp Karen Development and Psychometric Testing of the Thai-Nutrition Literacy Assessment Tool for Adolescents. PACIFIC RIM INTERNATIONAL JOURNAL OF NURSING

RESEARCH. 2020;24(1):5-19.

4165. Kenny Bridget, Orellana Liliana, Fuller-Tyszkiewicz Matthew, Moodie Marj, Brown Vicki, Williams Joanne Depression and eating disorders in early adolescence: A network analysis approach. INTERNATIONAL JOURNAL OF EATING DISORDERS. 2021;54(12):2143-2154.

4166. Rodrigo J, Maiquez L, Garcia M, Mendoza R, Rubio A, Martinez A, Martin JC Parent-child relationships and life-styles of adolescents. PSICOTHEMA. 2004;16(2):203-210.

4167. Wong Fiona, Stevens Denise, O'Connor-Duffany Kathleen, Siegel Karen, Gao Yue Community Health Environment Scan Survey (CHESS): a novel tool that captures the impact of the built environment on lifestyle factors. GLOBAL HEALTH ACTION. 2011;4(SI):.

4168. Saleem Sheikh, Bhattacharya Sudip, Deshpande Nalini Non-communicable diseases, type 2 diabetes, and influence of front of package nutrition labels on consumer's behaviour: Reformulations and future scope. DIABETES \& METABOLIC SYNDROME-CLINICAL RESEARCH \& REVIEWS. 2022;16(2):.

4169. Keller Kristin, Rodriguez Lopez Santiago, Carmenate Moreno Margarita Association between meal intake behaviour and abdominal obesity in Spanish adults. APPETITE. 2015;92():1-6.

4170. Bryant-Waugh Rachel Avoidant restrictive food intake disorder: An illustrative case example. INTERNATIONAL JOURNAL OF EATING DISORDERS. 2013;46(5, SI):420-423.

4171. Utschig Angela, Presnell Katherine, Madeley Mary, Smits Jasper An investigation of the relationship between fear of negative evaluation and bulimic psychopathology. EATING BEHAVIORS. 2010;11(4):231-238.

4172. Tamura Akira, Kawamoto Daiki, Minami Koichi, Yasuda Shingo, Tsujimoto Hiroshi, Tsuda Yuko, Mizumoto Kazuhiro, Suzuki Hiroyuki Candida guilliermondii-induced chorioretinitis in a patient with eating disorder. JOURNAL OF INFECTION AND CHEMOTHERAPY. 2021;27(4):642-646.

4173. Guenther Julia, Hoffmann Julia, Spies Monika, Meyer Dorothy, Kunath Julia, Stecher Lynne, Rosenfeld Eva, Kick Luzia, Rauh Kathrin, Hauner Hans Associations between the

Prenatal Diet and Neonatal Outcomes-A Secondary Analysis of the Cluster-Randomised GeliS Trial. NUTRIENTS. 2019;11(8):.

4174. Nilsson Karin, Engstrom Ingemar, Hagglof Bruno Family Climate and Recovery in Adolescent Onset Eating Disorders: A Prospective Study. EUROPEAN EATING DISORDERS REVIEW. 2012;20(1):e96-e102.

4175. Currie A, Morse ED Eating disorders in athletes: Managing the risks. CLINICS IN SPORTS MEDICINE. 2005;24(4):871+.

4176. Marucci Simonetta, Almerighi Guido, Cerutti Nadia, Corbo Filomena, Zupo Roberta, De Iaco Giulia, Lisco Giuseppe, Triggiani Vincenzo, De Pergola Giovanni Eating Disorders in the Time of the COVID-19 Pandemic: A Perspective. ENDOCRINE METABOLIC \& IMMUNE DISORDERS-DRUG TARGETS. 2023;23(2):123-128.

4177. Huhmann Kimberly Menses Requires Energy: A Review of How Disordered Eating, Excessive Exercise, and High Stress Lead to Menstrual Irregularities. CLINICAL THERAPEUTICS. 2020;42(3):401-407.

4178. Jordao Lidia, Malta Deborah, Freire Maria Clustering patterns of oral and general health-risk behaviours in Brazilian adolescents: Findings from a national survey. COMMUNITY DENTISTRY AND ORAL EPIDEMIOLOGY. 2018;46(2):194-202.

4179. Gupta Aakriti, Storey Kenneth Regulation of antioxidant systems in response to anoxia and reoxygenation in Rana sylvatica. COMPARATIVE BIOCHEMISTRY AND PHYSIOLOGY B-BIOCHEMISTRY \& MOLECULAR BIOLOGY. 2020;243():.

4180. Holmes Jennifer, St Laurent Christine, Spencer Rebecca Unhealthy Diet Is Associated With Poor Sleep in Preschool-Aged Children. JOURNAL OF GENETIC PSYCHOLOGY. 2021;182(5, SI):289-303.

4181. Hasking Penelope, Boyes Mark, Greves Stuart Self-efficacy and emotionally dysregulated behaviour: An exploratory test of the role of emotion regulatory and behaviour-specific beliefs. PSYCHIATRY RESEARCH. 2018;270():335-340.

4182. Currenti Walter, Godos Justyna, Castellano Sabrina, Caruso Giuseppe, Ferri Raffaele, Caraci Filippo, Grosso Giuseppe, Galvano Fabio Time-restricted feeding is associated with

mental health in elderly Italian adults. CHRONOBIOLOGY INTERNATIONAL. 2021;38(10):1507-1516.

4183. Congdon Peter, Amugsi Dickson Editorial: The obesity epidemic: Causes, context, prevention. FRONTIERS IN PUBLIC HEALTH. 2022;10():.

4184. Giel Katrin, Hermann-Werner Anne, Mayer Jochen, Diehl Katharina, Schneider Sven, Thiel Ansgar, Zipfel Stephan, Grp GOAL Eating disorder pathology in elite adolescent athletes. INTERNATIONAL JOURNAL OF EATING DISORDERS. 2016;49(6):553-562.

4185. Bruni Vincenzina, Dei Metella, Peruzzi Elena, Seravalli Viola The anorectic and obese adolescent. BEST PRACTICE \& RESEARCH CLINICAL OBSTETRICS \& GYNAECOLOGY. 2010;24(2):243-258.

4186. Winkler Laura, Andersen Marianne, Horder Kirsten, Schumann Thorsten, Stoving Rene Slow-Growing Craniopharyngioma Masquarading as Early-Onset Eating Disorder: Two Cases. INTERNATIONAL JOURNAL OF EATING DISORDERS. 2009;42(5):475-478.

4187. Eichenberg Christiane, Ott Ralf Internet-based psychological intervention. Review of empirical results of disorder-specific programs. PSYCHOTHERAPEUT. 2012;57(1):58-69.

4188. Groth Susan, Morrison-Beedy Dianne Smoking, Substance Use, and Mental Health Correlates in Urban Adolescent Girls. JOURNAL OF COMMUNITY HEALTH. 2011;36(4):552-558.

4189. Williams NI Lessons from experimental disruptions of the menstrual cycle in humans and monkeys. MEDICINE AND SCIENCE IN SPORTS AND EXERCISE. 2003;35(9):1564-1572.

4190. Deas Suzanne, Power Kevin, Collin Paula, Yellowlees Alex, Grierson David The Relationship between Disordered Eating, Perceived Parenting, and Perfectionistic Schemas. COGNITIVE THERAPY AND RESEARCH. 2011;35(5):414-424.

4191. Carels Robert, Miller J., Selensky Jennifer, Hlavka Reid, Solar Chelsey, Rossi James, Ellis Jordan Using an acceptance-based behavioral approach as a supplement to obesity treatment: A stepped-care approach. JOURNAL OF CONTEXTUAL BEHAVIORAL SCIENCE. 2019;12():98-105.

4192. Gonzalez Lupita, Lammert Amy, Phelan Suzanne, Ventura Alison Associations between parenting stress, parent feeding practices, and perceptions of child eating behaviors during the COVID-19 pandemic. APPETITE. 2022;177():.

4193. Ganson Kyle, Cunningham Mitchell, Murray Stuart, Nagata Jason Use of appearance- and performance-enhancing drugs and substances is associated with eating disorder symptomatology among US college students. EATING AND WEIGHT DISORDERS-STUDIES ON ANOREXIA BULIMIA AND OBESITY. 2022;27(6):2245-2250.

4194. Izydorczyk Bernadetta, Sitnik-Warchulska Katarzyna, Lizinczyk Sebastian, Lipowska Malgorzata Socio-Cultural Standards Promoted by the Mass Media as Predictors of Restrictive and Bulimic Behavior. FRONTIERS IN PSYCHIATRY. 2020;11():.

4195. Barolia Rubina, Clark Alexander, Higginbottom Gina Protocol for a qualitative study on promoting dietary change and positive food choices for poor people with low income who experience cardiovascular disease in Pakistan. BMJ OPEN. 2013;3(12):.

4196. Nilsson Karin, Sundbom Elisabet, Hagglof Bruno A longitudinal study of perfectionism in adolescent onset anorexia nervosa-restricting type. EUROPEAN EATING DISORDERS REVIEW. 2008;16(5):386-394.

4197. Fontenelle Leonardo, Oostermeijer Sanne, Harrison Ben, Pantelis Christos, Yuecel Murat Obsessive-Compulsive Disorder, Impulse Control Disorders and Drug Addiction Common Features and Potential Treatments. DRUGS. 2011;71(7):827-840.

4198. Lang Undine, Beglinger Christoph, Schweinfurth Nina, Walter Marc, Borgwardt Stefan Nutritional Aspects of Depression. CELLULAR PHYSIOLOGY AND BIOCHEMISTRY. 2015;37(3):1029-1043.

4199. Khalsa Sahib, Portnoff Larissa, McCurdy-McKinnon Danyale, Feusner Jamie What happens after treatment? A systematic review of relapse, remission, and recovery in anorexia nervosa. JOURNAL OF EATING DISORDERS. 2017;5():.

4200. McAulay Claire, Dawson Lisa, Mond Jonathan, Outhred Tim, Touyz Stephen ``The Food Matches the Mood{''}: Experiences of Eating Disorders in Bipolar Disorder. QUALITATIVE HEALTH RESEARCH. 2021;31(1):100-112.

4201. Arnold Sabine, Wiese Antonia, Zaid Sarah, Correll Christoph, Jaite Charlotte Lifetime prevalence and clinical correlates of nonsuicidal self-injury in youth inpatients with eating disorders: a retrospective chart review. CHILD AND ADOLESCENT PSYCHIATRY AND MENTAL HEALTH. 2022;16(1):.

4202. Juli Maria, Juli Luigi BODY IDENTITY SEARCH: THE SUSPENDED BODY. PSYCHIATRIA DANUBINA. 2020;32(1):83-87.

4203. Sonneville Kendrin, Thurston Idia, Milliren Carly, Gooding Holly, Richmond Tracy Weight misperception among young adults with overweight/obesity associated with disordered eating behaviors. INTERNATIONAL JOURNAL OF EATING DISORDERS. 2016;49(10):937-946.

4204. Lam Siu-Ping, Fong Samson, Yu Mandy, Li Shirley, Wing Yun-Kwok Sleepwalking in psychiatric patients: comparison of childhood and adult onset. AUSTRALIAN AND NEW ZEALAND JOURNAL OF PSYCHIATRY. 2009;43(5):426-430.

4205. Giraudeau Nicolas, Camman Paul, Pourreyron Laurence, Inquimbert Camille, Lefebvre Patrick The contribution of teledentistry in detecting tooth erosion in patients with eating disorders. DIGITAL HEALTH. 2021;7():.

4206. Azorin Jean-Michel, Belzeaux Raoul, Adida Marc Age-at-onset and comorbidity may separate depressive disorder subtypes along a descending gradient of bipolar propensity. BEHAVIOURAL BRAIN RESEARCH. 2015;282():185-193.

4207. Lucarini Emanuela, Attademo Luigi, Moretti Patrizia, Spollon Giulio, Elisei Sandro, Quartesan Roberto, Tortorella Alfonso PERSONALITY DISORDERS FEATURES IN A SAMPLE OF WOMEN WITH PERINATAL DEPRESSION IN PERUGIA, ITALY. PSYCHIATRIA DANUBINA. 2017;29(3):S323-S332.

4208. Harden K., Mendle J., Kretsch N. Environmental and genetic pathways between early pubertal timing and dieting in adolescence: distinguishing between objective and subjective timing. PSYCHOLOGICAL MEDICINE. 2012;42(1):183-193.

4209. Akhrif Atae, Romanos Marcel, Domschke Katharina, Schmitt-Boehrer Angelika, Neufang Susanne Fractal Analysis of BOLD Time Series in a Network Associated With Waiting Impulsivity. FRONTIERS IN PHYSIOLOGY. 2018;9():.

4210. Fu Ruijia, Liang Pei, Lu Gang, Gu Jinbao, Wang Dayong Prevalence of sparganum infection in wild frogs in Hainan province of China involves a risk for sparganosis. AMERICAN JOURNAL OF TRANSLATIONAL RESEARCH. 2022;14(3):1826-1837.

4211. Doumit R., Kharma J., Sanchez-Ruiz M., Zeeni N. Predictors of Disordered Eating in Young Males. COMMUNITY MENTAL HEALTH JOURNAL. 2018;54(2):236-244.

4212. Peterson Kathleen, Paulson Sharon, Williams Kristen Relations of eating disorder symptomology with perceptions of pressures from mother, peers, and media in adolescent girls and boys. SEX ROLES. 2007;57(9-10):629-639.

4213. Pursey Kirrilly, Burrows Tracy, Barker Daniel, Hart Melissa, Paxton Susan Disordered eating, body image concerns and weight control behaviours in children: A systematic review and meta-analysis of universal-selective prevention interventions. JOURNAL OF EATING DISORDERS. 2021;9(SUPPL 1, 1, SI):.

4214. Son Gabrielle, Hoeken Daphne, Bartelds Aad, Furth Eric, Hoek Hans Time trends in the incidence of eating disorders: A primary care study in the Netherlands. INTERNATIONAL JOURNAL OF EATING DISORDERS. 2006;39(7):565-569.

4215. Brion Agnes, Flamand Mathilde, Oudiette Delphine, Voillery Dorothee, Golmard Jean-Louis, Arnulf Isabelle Sleep-related eating disorder versus sleepwalking: A controlled study. SLEEP MEDICINE. 2012;13(8):1094-1101.

4216. Wadolowska Lidia, Hamulka Jadwiga, Kowalkowska Joanna, Ulewicz Natalia, Hoffmann Monika, Gornicka Magdalena, Bronkowska Monika, Leszczynska Teresa, Glibowski Pawel, Korzeniowska-Ginter Renata Changes in Sedentary and Active Lifestyle, Diet Quality and Body Composition Nine Months after an Education Program in Polish Students Aged 11-12 Years: Report from the ABC of Healthy Eating Study. NUTRIENTS. 2019;11(2):.

4217. Alaini Reham, Rajikan Roslee, Elias Siti Diet optimization using linear programming to develop low cost cancer prevention food plan for selected adults in Kuala Lumpur, Malaysia. BMC PUBLIC HEALTH. 2019;19(4):.

4218. Lee Amanda, Kane Sarah, Ramsey Rebecca, Good Elizabeth, Dick Mathew Testing the price and affordability of healthy and current (unhealthy) diets and the potential impacts of

policy change in Australia. BMC PUBLIC HEALTH. 2016;16():.

4219. Vuillier L., Carter Z., Teixeira A., Moseley R. Alexithymia may explain the relationship between autistic traits and eating disorder psychopathology. MOLECULAR AUTISM. 2020;11(1):.

4220. Bouattour Wiem, Aribi Lobna, Mseddi Neila, Charfeddine Fadwa, Amami Othmen Food addiction: Prevalence and associated factors. A cross-sectional study on a non-clinical sample in Sfax-Tunisia. ANNALES MEDICO-PSYCHOLOGIQUES. 2021;179(8):700-706.

4221. Hay Phillipa, Claudino Angelica, Touyz Stephen, Abd Elbaky Ghada Individual psychological therapy in the outpatient treatment of adults with anorexia nervosa. COCHRANE DATABASE OF SYSTEMATIC REVIEWS. 2015;(7):.

4222. Perena Garcia Francisco Body and subjectivity: About anorexia nervosa. REVISTA ESPANOLA DE SALUD PUBLICA. 2007;81(5):529-542.

4223. Flament Martine, Henderson Katherine, Buchholz Annick, Obeid Nicole, Nguyen Hien, Birmingham Meagan, Goldfield Gary Weight Status and DSM-5 Diagnoses of Eating Disorders in Adolescents From the Community. JOURNAL OF THE AMERICAN ACADEMY OF CHILD AND ADOLESCENT PSYCHIATRY. 2015;54(5):403-411.

4224. Linders Louisa, Patrikiou Lefkothea, Soiza-Reilly Mariano, Schut Evelien, Schaffelaar Bram, Boger Leonard, Wolterink-Donselaar Inge, Luijendijk Mieneke, Adan Roger, Meye Frank Stress-driven potentiation of lateral hypothalamic synapses onto ventral tegmental area dopamine neurons causes increased consumption of palatable food. NATURE COMMUNICATIONS. 2022;13(1):.

4225. Bonnet F, Irving K, Terra JL, Nony P, Berthezene F, Moulin P Depressive symptoms are associated with unhealthy lifestyles in hypertensive patients with the metabolic syndrome. JOURNAL OF HYPERTENSION. 2005;23(3):611-617.

4226. PARK RJ, LAWRIE SM, FREEMAN CP POSTVIRAL ONSET OF ANOREXIA-NERVOSA. BRITISH JOURNAL OF PSYCHIATRY. 1995;166():386-389.

4227. Klump KL, Kaye WH, Strober M The evolving genetic foundations of eating disorders. PSYCHIATRIC CLINICS OF NORTH AMERICA. 2001;24(2):215+.

4228. Bhattacharya Romit, Zekavat Seyedeh, Uddin Md, Pirruccello James, Niroula Abhishek, Gibson Christopher, Griffin Gabriel, Libby Peter, Ebert Benjamin, Bick Alexander, Natarajan Pradeep Association of Diet Quality With Prevalence of Clonal Hematopoiesis and Adverse Cardiovascular Events. JAMA CARDIOLOGY. 2021;6(9):1069-1077.

4229. StriegelMoore RH Risk factors for eating disorders. . 1997;817():98-109.

4230. Spieker Elena, Sbrocco Tracy, Theim Kelly, Maurer Douglas, Johnson Dawn, Bryant Edny, Bakalar Jennifer, Schvey Natasha, Ress Rachel, Seehusen Dean, Klein David, Stice Eric, Yanovski Jack, Chan Linda, Gentry Shari, Ellsworth Carol, Hill Joanne, Tanofsky-Kraff Marian, Stephens Mark Preventing Obesity in the Military Community (POMC): The Development of a Clinical Trials Research Network. INTERNATIONAL JOURNAL OF ENVIRONMENTAL RESEARCH AND PUBLIC HEALTH. 2015;12(2):1174-1195.

4231. Mancini Karen Body Image, Eating Attitudes and Breastfeeding Intention: Implications for Mental Health and Maternal Child Nurses. ISSUES IN MENTAL HEALTH NURSING. 2017;38(9):750-755.

4232. Schoeffel Hannah, Hiemisch Andreas, Kiess Wieland, Hilbert Anja, Schmidt Ricarda Characteristics of avoidant/restrictive food intake disorder in a general paediatric inpatient sample. EUROPEAN EATING DISORDERS REVIEW. 2021;29(1):60-73.

4233. Schlapfer Leslie, Fujimoto Akane, Gettis Margaret Impact of caloric prescriptions and degree of malnutrition on incidence of refeeding syndrome and clinical outcomes in patients with eating disorders: A retrospective review. NUTRITION IN CLINICAL PRACTICE. 2022;37(2):459-469.

4234. Bonnet F, Irving K, Terra JL, Nony P, Berthezene F, Moulin P Anxiety and depression are associated with unhealthy lifestyle in patients at risk of cardiovascular disease. ATHEROSCLEROSIS. 2005;178(2):339-344.

4235. Berkol Tonguc, Islam Serkan, Kirli Ebru, Pinarbasi Rasim, Ozyildirim Ilker Suicide attempts and clinical features of bipolar patients. SAUDI MEDICAL JOURNAL. 2016;37(6):662-667.

4236. Li Mian, Xu Yu, Wan Qin, Shen Feixia, Xu Min, Zhao Zhiyun, Lu Jieli, Gao Zhengnan, Chen Gang, Wang Tiange, Xu Yiping, Zhao Jiajun, Chen Lulu, Shi Lixin, Hu Ruying, Ye Zhen, Tang Xulei, Su Qing, Qin Guijun, Wang Guixia, Luo Zuojie, Qin Yingfen, Huo Yanan, Li Qiang, Zhang Yinfei, Chen Yuhong, Liu Chao, Mu Yiming, Wang Youmin, Wu Shengli, Yang Tao, Chen Li, Yu Xuefeng, Yan Li, Deng Huacong, Ning Guang, Bi Yufang, Wang Weiqing Individual and Combined Associations of Modifiable Lifestyle and Metabolic Health Status With New-Onset Diabetes and Major Cardiovascular Events: The China Cardiometabolic Disease and Cancer Cohort (4C) Study. DIABETES CARE. 2020;43(8):1929-1936.

4237. DeBate R., Severson H., Cragun D., Shaw T., Cantwell C., Gallentine A., Christiansen S., Koerber A., Tomar S., Hendricson W., Brown K., Tedesco L. INCREASING THE CAPACITY OF ORAL HEALTH STUDENTS TO DELIVER SECONDARY PREVENTION OF DISORDERED EATING BEHAVIORS: EVALUATION OF A THEORY-BASED ONLINE TRAINING PROGRAM. ANNALS OF BEHAVIORAL MEDICINE. 2012;43(1):S183.

4238. Berends Tamara, Boonstra Nynke, Elburg Annemarie Relapse in anorexia nervosa: a systematic review and meta-analysis. CURRENT OPINION IN PSYCHIATRY. 2018;31(6):445-455.

4239. Zack Sanno, Saekow Jenine, Kelly Megan, Radke Anneliese Mindfulness Based Interventions for Youth. JOURNAL OF RATIONAL-EMOTIVE AND COGNITIVE-BEHAVIOR THERAPY. 2014;32(1):44-56.

4240. Kalk Nicola, Lingford-Hughes Anne The clinical pharmacology of acamprosate. BRITISH JOURNAL OF CLINICAL PHARMACOLOGY. 2014;77(2, SI):315-323.

4241. Rapee Ronald, Oar Ella, Johnco Carly, Forbes Miriam, Fardouly Jasmine, Magson Natasha, Richardson Cele Adolescent development and risk for the onset of social-emotional disorders: A review and conceptual model. BEHAVIOUR RESEARCH AND THERAPY. 2019;123():.

4242. Bender Nicole Contribution of ethology to evolutionary medicine. ETHOLOGY. 2021;127(10):821-826.

4243. Cockell SJ, Geller J, Linden W The development of a decisional balance scale for anorexia nervosa. EUROPEAN EATING DISORDERS REVIEW. 2002;10(5):359-375.

4244. Thiria Etienne, Pellegrini Christine, Kase Bezawit, DeVivo Katherine, Steck Susan Health behavior and anxiety changes during the COVID-19 pandemic among students, faculty, and staff at a US university. JOURNAL OF AMERICAN COLLEGE HEALTH. 2022;():.

4245. Kiechle Marion, Grill Sabine Lifestyle, nutrition, sport, and their importance in prevention of hereditary cancer in women. GYNAKOLOGE. 2020;53(11, SI):756-760.

4246. Roman G., Teodorescu G. INCREASED PREVALENCE OF CARDIOVASCULAR RISK FACTORS IN NEWLY DIAGNOSED TYPE 2 DIABETES PATIENTS - A RETROSPECTIVE STUDY. ACTA ENDOCRINOLOGICA-BUCHAREST. 2021;17(3):.

4247. Metcalf Christina, Dimidjian Sona Extensions and Mechanisms of Mindfulness-based Cognitive Therapy: A Review of the Evidence. AUSTRALIAN PSYCHOLOGIST. 2014;49(5):271-279.

4248. Herpertz-Dahlmann Beate, Seitz Jochen, Konrad Kerstin Aetiology of anorexia nervosa: from a ``psychosomatic family model{''} to a neuropsychiatric disorder?. EUROPEAN ARCHIVES OF PSYCHIATRY AND CLINICAL NEUROSCIENCE. 2011;261(2):177-181.

4249. Kelly-Weeder Susan, Phillips Kathryn, Leonard Kelly, Veroneau Margaret Binge eating and weight loss behaviors of overweight and obese college students. JOURNAL OF THE AMERICAN ASSOCIATION OF NURSE PRACTITIONERS. 2014;26(8):445-451.

4250. Kiechle Marion Nutrition and physical activity-Importance for prevention, prognosis and treatment of gynecological tumors. GYNAKOLOGE. 2021;54(1):14-18.

4251. Lim Shen-Yang, Tan Zi, Ngam Pei, Lor Tong, Mohamed Hafsa, Schee Jie, Tan Aik, Goh Jun, Ooi Eugene, Soh Patrick Impulsive-compulsive behaviors are common in Asian Parkinson's disease patients: Assessment using the QUIP. PARKINSONISM \& RELATED DISORDERS. 2011;17(10):761-764.

4252. Sapozhnikova I., Tarlovskaya E., Vedenskaya T. Eating behavior in patients with diabetes mellitus. TERAPEVTICHESKII ARKHIV. 2012;84(12):71-75.

4253. Sharpe Helen, Schober Ilka, Treasure Janet, Schmidt Ulrike The role of high-quality friendships in female adolescents' eating pathology and body dissatisfaction. EATING AND

WEIGHT DISORDERS-STUDIES ON ANOREXIA BULIMIA AND OBESITY. 2014;19(2):159-168.

4254. Melisse Bernou, Beurs Edwin, Furth Eric Eating disorders in the Arab world: a literature review. JOURNAL OF EATING DISORDERS. 2020;8(1):.

4255. Azrin Nathan, Kellen Michael, Brooks Jeannie, Ehle Chris, Vinas Veronica Relationship Between Rate of Eating and Degree of Satiation. CHILD \& FAMILY BEHAVIOR THERAPY. 2008;30(4):355-364.

4256. Maghsoudi Zahra, Azadbakht Leila How dietary patterns could have a role in prevention, progression, or management of diabetes mellitus? Review on the current evidence. JOURNAL OF RESEARCH IN MEDICAL SCIENCES. 2012;17(7):694-709.

4257. Stachteas P., Stachteas C. Contemporary public health challenges: Physical activity and nutrition in the COVID-19 pandemic era. ARCHIVES OF HELLENIC MEDICINE. 2022;39(5):601-608.

4258. Ozsoy Serpil, Ozer Emel A Diabetes Risk Screening In Northern Cyprus: What We Learned With FINDRISC. PROGRESS IN NUTRITION. 2022;24(2):.

4259. Rayner Kathryn, Schniering Carolyn, Rapee Ronald, Hutchinson Delyse A Longitudinal Investigation of Perceived Friend Influence on Adolescent Girls' Body Dissatisfaction and Disordered Eating. JOURNAL OF CLINICAL CHILD AND ADOLESCENT PSYCHOLOGY. 2013;42(5):643-656.

4260. Dyck Zoe, Bellwald Laura, Kurz Susanne, Dremmel Daniela, Munsch Simone, Hilbert Anja Eating disorders in childhood and adolescence. ZEITSCHRIFT FUR GESUNDHEITSPSYCHOLOGIE. 2013;21(2):91-100.

4261. Curry John, Aubuchon-Endsley Nicki, Brancu Mira, Runnals Jennifer, Fairbank John, Vet VA, Registry VA Lifetime major depression and comorbid disorders among current-era women veterans. JOURNAL OF AFFECTIVE DISORDERS. 2014;152():434-440.

4262. Enten Roni, Golan Moria Parenting styles and weight-related symptoms and behaviors with recommendations for practice. NUTRITION REVIEWS. 2008;66(2):65-75.

4263. Austin S., Kim Juhee, Wiecha Jean, Troped Philip, Feldman Henry, Peterson Karen School-based overweight preventive intervention lowers incidence of disordered weight-control behaviors in early adolescent girls. ARCHIVES OF PEDIATRICS \& ADOLESCENT MEDICINE. 2007;161(9):865-869.

4264. Bicvic Antonela, Hammer Helly, Sarikaya Hakan, Heldner Mirjam Healthy diet in primary and secondary prevention of stroke. THERAPEUTISCHE UMSCHAU. 2021;78(6):259-268.

4265. Wentz Elisabet, Gillberg I., Anckarsater Henrik, Gillberg Christopher, Rastam Maria Somatic problems and self-injurious behaviour 18 years after teenage-onset anorexia nervosa. EUROPEAN CHILD \& ADOLESCENT PSYCHIATRY. 2012;21(8):421-432.

4266. Adametz Luise, Richter Felicitas, Strauss Bernhard, Walther Mario, Wick Katharina, Berger Uwe Long-term effectiveness of a school-based primary prevention program for anorexia nervosa: A 7-to 8-year follow-up. EATING BEHAVIORS. 2017;25(SI):42-50.

4267. Thompson Ron, Sherman Roberta Reflections on athletes and eating disorders. PSYCHOLOGY OF SPORT AND EXERCISE. 2014;15(6, SI):729-734.

4268. Jones Michelle, Crowther Janis, Ciesla Jeffrey A naturalistic study of fat talk and its behavioral and affective consequences. BODY IMAGE. 2014;11(4):337-345.

4269. Hart Laura, Cornell Chelsea, Damiano Stephanie, Paxton Susan Parents and Prevention: A Systematic Review of Interventions Involving Parents that Aim to Prevent Body Dissatisfaction or Eating Disorders. INTERNATIONAL JOURNAL OF EATING DISORDERS. 2015;48(2):157-169.

4270. Howard Lindsay, Romano Kelly, Heron Kristin Prospective changes in disordered eating and body dissatisfaction across women's first year of college: The relative contributions of sociocultural and college adjustment risk factors. EATING BEHAVIORS. 2020;36():.

4271. Valls Marjorie, Bonvin Patrick, Chabrol Henri Association Between Muscularity Dissatisfaction and Body Dissatisfaction Among Normal-Weight French Men. JOURNAL OF MENS HEALTH. 2013;10(4):139-145.

4272. Jansen Pauline, Roza Sabine, Jaddoe Vincent, Mackenbach Joreintje, Raat Hein, Hofman Albert, Verhulst Frank, Tiemeier Henning Children's eating behavior, feeding practices of parents and weight problems in early childhood: results from the population-based Generation R Study. INTERNATIONAL JOURNAL OF BEHAVIORAL NUTRITION AND PHYSICAL ACTIVITY. 2012;9():.

4273. Liu D., He H., Li G., Chen J., Yin D., Liao Z., Tang L., Huang Q., Lai Z., He M. Mechanisms of chloride in cardiomyocyte anoxia-reoxygenation injury: the involvement of oxidative stress and NF-kappaB activation. MOLECULAR AND CELLULAR BIOCHEMISTRY. 2011;355(1-2):201-209.

4274. Chang Vicky, Cotterchio Michelle, De Prithwish, Tinmouth Jill Risk factors for early-onset colorectal cancer: a population-based case-control study in Ontario, Canada. CANCER CAUSES \& CONTROL. 2021;32(10):1063-1083.

4275. Fisher Laurie, Miles Isa, Austin Bryn, Camargo Carlos, Colditz Graham Predictors of initiation of alcohol use among US adolescents - Findings from a prospective cohort study. ARCHIVES OF PEDIATRICS \& ADOLESCENT MEDICINE. 2007;161(10):959-966.

4276. Lukas Linda, Buhl Christina, Schulte-Koerne Gerd, Sfaerlea Anca Family, friends, and feelings: the role of relationships to parents and peers and alexithymia in adolescents with anorexia nervosa. JOURNAL OF EATING DISORDERS. 2022;10(1):.

4277. Virmani Tuhin, Greene Paul, Pearson Toni Delayed onset of progressive chorea after acute basal ganglia injury. MOVEMENT DISORDERS. 2013;28(5):585-587.

4278. Nickelson Jen, Bryant Carol, McDermott Robert, Buhi Eric, DeBate Rita A Modified Obesity Proneness Model Predicts Adolescent Weight Concerns and Inability to Self-Regulate Eating. JOURNAL OF SCHOOL HEALTH. 2012;82(12):560-571.

4279. Kato-Noguchi H Hypoxic acclimation to anoxia in Avena roots. PLANT GROWTH REGULATION. 2002;38(1):1-5.

4280. O'Mara Madieson, Greene Danyelle, Watson Hunna, Shafran Roz, Kenworthy Isabel, Cresswell Camilla, Egan Sarah The efficacy of randomised controlled trials of guided and unguided self-help interventions for the prevention and treatment of eating disorders in young people: A systematic review and preliminary meta-analysis. JOURNAL OF BEHAVIOR

THERAPY AND EXPERIMENTAL PSYCHIATRY. 2023;78():.

4281. Guerrero MNV The body, cult or tyranny?. PSICOTHEMA. 1998;10(1):111-125.

4282. Shaw Heather, Rohde Paul, Desjardins Christopher, Stice Eric Sexual orientation correlates with baseline characteristics but shows no moderating effects of dissonance-based eating disorder prevention programs for women. BODY IMAGE. 2020;32():94-102.

4283. Story M, French SA, NeumarkSztainer D, Downes B, Resnick MD, Blum RW Psychosocial and behavioral correlates of dieting and purging in Native American adolescents. PEDIATRICS. 1997;99(4):art. no.-e8.

4284. Jones Christina, O'Donnell Nicola, John Mary, Cooke Debbie, Stewart Rose, Hale Lucy, Skene Simon, Kanumakala Shankar, Harrington Megan, Satherley Rose-Marie PaRent InterventiOn to pRevent dIsordered eating in children with TYpe 1 diabetes (PRIORITY): Study protocol for a feasibility randomised controlled trial. DIABETIC MEDICINE. 2022;39(4):.

4285. Gaudio Santino, Dakanalis Antonios Personality and eating and weight disorders: an open research challenge. EATING AND WEIGHT DISORDERS-STUDIES ON ANOREXIA BULIMIA AND OBESITY. 2018;23(2):143-147.

4286. Stice Eric, Desjardins Christopher, Shaw Heather, Rohde Paul Moderators of two dual eating disorder and obesity prevention programs. BEHAVIOUR RESEARCH AND THERAPY. 2019;118():77-86.

4287. Rich Alexandra, Haynos Ann, Anderson Drew, Ehrlich Lauren, Anderson Lisa The role of rumination and positive beliefs about rumination in eating pathology. EATING AND WEIGHT DISORDERS-STUDIES ON ANOREXIA BULIMIA AND OBESITY. 2022;27(3):979-988.

4288. Riddle Megan, McKenna Morgan, Yoon Yone, Pattwell Siobhan, Santos Patricia, Casey B., Glatt Charles Caloric Restriction Enhances Fear Extinction Learning in Mice. NEUROPSYCHOPHARMACOLOGY. 2013;38(6):930-937.

4289. Aouad Phillip, Hambleton Ashlea, Marks Peta, Maloney Danielle, Calvert Shannon, Caldwell Belinda, McLean Sian, Shelton Beth, Cowan Katherine, Feneley John, Pepin

Genevieve, Paxton Susan, Williams Michelle, Meddick Thy, Squire Sarah, Hickie Ian, Lambkin Frances, Touyz Stephen, Maguire Sarah Setting the top 10 eating disorder research and translation priorities for Australia. AUSTRALIAN AND NEW ZEALAND JOURNAL OF PSYCHIATRY. 2022;():.

4290. Montgremier M., Moro M-R, Chen J., Lachal J. Female patients with eating disorders and their parents experience in China: A qualitative study. ENCEPHALE-REVUE DE PSYCHIATRIE CLINIQUE BIOLOGIQUE ET THERAPEUTIQUE. 2022;48(1):43-51.

4291. Leenaerts N., Vaessen T., Sunaert S., Ceccarini J., Vrieze E. Linking stress to impulsivity in recent-onset bulimia nervosa and alcohol use disorder: preliminary results from an ecological momentary assessments study. EUROPEAN NEUROPSYCHOPHARMACOLOGY. 2020;40(1):S90.

4292. Komada Yoko, Takaesu Yoshikazu, Matsui Kentaro, Nakamura Masaki, Nishida Shingo, Kanno Meri, Usui Akira, Inoue Yuichi Comparison of clinical features between primary and drug-induced sleep-related eating disorder. NEUROPSYCHIATRIC DISEASE AND TREATMENT. 2016;12():1275-1280.

4293. Raemen Leni, Luyckx Koen, Palmeroni Nina, Verschueren Margaux, Gandhi Amarendra, Grobler Adelene, Claes Laurence Trauma and self-harming behaviors in high school students: The mediating role of identity formation. JOURNAL OF ADOLESCENCE. 2021;92():20-29.

4294. Alima Nural, Yuksel Aysun, Pehlivan Leyla, Karakaya Rahime, Besler Zehra Eating Disorder Risk and Factors Associated with Obesity Prejudice Among University Students: A cross-sectional descriptive study. REVISTA ESPANOLA DE NUTRICION HUMANA Y DIETETICA. 2022;26(2):104-113.

4295. Masi Laura, Gignac Martin ADHD and comorbid disorders in pedopsychiatry: Psychiatric problems, medical problems, learning disorders and developmental coordination disorder. ANNALES MEDICO-PSYCHOLOGIQUES. 2017;175(5):422-429.

4296. Yousman Lori ``Usually People Just Accept Media And Don't Talk About It{''} The Perceived Value And Enjoyment Of Critical Media Literacy In Eating Disorder Treatment. MEDIA LITERACY AND ACADEMIC RESEARCH. 2021;4(2):42-57.

4297. Farrell Nicholas, Brosof Leigh, Vanzhula Irina, Christian Caroline, Bowie Owen, Levinson Cheri Exploring Mechanisms of Action in Exposure-Based Cognitive Behavioral Therapy for Eating Disorders: The Role of Eating-Related Fears and Body-Related Safety Behaviors. BEHAVIOR THERAPY. 2019;50(6, SI):1125-1135.

4298. Ravaldi Claudia, Vannacci Alfredo, Bolognesi Enrica, Mancini Stefania, Faravelli Carlo, Ricca Valdo Gender role, eating disorder symptoms, and body image concern in ballet dancers. JOURNAL OF PSYCHOSOMATIC RESEARCH. 2006;61(4):529-535.

4299. Antunez Juan, Navarro Jose, Adan Ana Circadian typology and problems in mental health. ANALES DE PSICOLOGIA. 2014;30(3):971-984.

4300. Olive Lisa, Rice Simon, Gao Caroline, Pilkington Vita, Walton Courtney, Butterworth Matt, Abbott Lyndel, Cross Gemma, Clements Matti, Purcell Rosemary Risk and protective factors for mental ill-health in elite para- and non-para athletes. FRONTIERS IN PSYCHOLOGY. 2022;13():.

4301. Lindekilde Nanna, Scheuer Stine, Rutters Femke, Knudsen Lenette, Lasgaard Mathias, Rubin Katrine, Henriksen Jan, Kivimaki Mika, Andersen Gregers, Pouwer Frans Prevalence of type 2 diabetes in psychiatric disorders: an umbrella review with meta-analysis of 245 observational studies from 32 systematic reviews. DIABETOLOGIA. 2022;65(3):440-456.

4302. Teufel Martin, Wild Beate, Giel KatrinE., Friederich Hans-Christoph, Resmark Gaby, Zwaan Martina, Herpertz Stephan, Loewe Bernd, Tagay Sefik, Wietersheim Joern, Zeeck Almut, Burgmer Markus, Dinkel Andreas, Ziser Katrin, Zehnpfennig Dominique, Zipfel Stephan, Herzog Wolfgang, Junne Florian Family, partnership, education and occupation in patients with anorexia nervosa. Secondary analysis of the ANTOP sample. PSYCHOTHERAPEUT. 2017;62(3):212-221.

4303. Hoglund K, Normen L A high exercise load is linked to pathological weight control behavior and eating disorders in female fitness instructors. SCANDINAVIAN JOURNAL OF MEDICINE \& SCIENCE IN SPORTS. 2002;12(5):261-275.

4304. Rohrbach Pieter, Dingemans Alexandra, Spinhoven Philip, Van Ginkel Joost, Fokkema Marjolein, Wilderjans Tom, Bauer Stephanie, Van Furth Eric Effectiveness of an online self-help program, expert-patient support, and their combination for eating disorders: Results from a randomized controlled trial. INTERNATIONAL JOURNAL OF EATING DISORDERS. 2022;55(10):1361-1373.

4305. Sala Margarita, Reyes-Rodriguez Mae, Bulik Cynthia, Bardone-Cone Anna Race, Ethnicity, and Eating Disorder Recognition by Peers. EATING DISORDERS. 2013;21(5):423-436.

4306. Melioli Tiffany, Bauer Stephanie, Franko Debra, Moessner Markus, Ozer Fikret, Chabrol Henri, Rodgers Rachel Reducing Eating Disorder Symptoms and Risk Factors Using the Internet: A Meta-Analytic Review. INTERNATIONAL JOURNAL OF EATING DISORDERS. 2016;49(1):19-31.

4307. BAKSHI VP, KELLEY AE FEEDING INDUCED BY OPIOID STIMULATION OF THE VENTRAL STRIATUM - ROLE OF OPIATE RECEPTOR SUBTYPES. JOURNAL OF PHARMACOLOGY AND EXPERIMENTAL THERAPEUTICS. 1993;265(3):1253-1260.

4308. Wang Jingfeng, Zhou Jingmin, Wang Yanyan, Yang Chunjie, Fu Mingqiang, Zhang Jingjing, Han Xueting, Li Zhiming, Hu Kai, Ge Junbo Qiliqiangxin protects against anoxic injury in cardiac microvascular endothelial cells via NRG-1/ErbB-PI3K/Akt/mTOR pathway. JOURNAL OF CELLULAR AND MOLECULAR MEDICINE. 2017;21(9):1905-1914.

4309. Herrmann Tracy, Preib Emily, French Madeline, Beckstrom Julie, Nazarenko Elena, Lackner Ryan, Marchand William, Yabko Brandon Veterans' experiences with mindfulness-based eating: A mixed methods study on MB-SAVOR. COMPLEMENTARY THERAPIES IN CLINICAL PRACTICE. 2022;47():.

4310. Elizathe Luciana, Custodio Jesica, Murawski Brenda, Rutsztein Guillermina ATTITUDES TOWARD OVERWEIGHT AND THEIR ASSOCIATION WITH RISK OF EATING DISORDER IN CHILDREN. AN EXPLORATORY STUDY. REVISTA ARGENTINA DE CLINICA PSICOLOGICA. 2017;26(3):324-331.

4311. Rogers Laura, Magill-Evans Joyce, Rempel Gwen Mothers' Challenges in Feeding their Children with Autism Spectrum Disorder-Managing More Than Just Picky Eating. JOURNAL OF DEVELOPMENTAL AND PHYSICAL DISABILITIES. 2012;24(1):19-33.

4312. McLaughlin Katie, Hilt Lori, Nolen-Hoeksema Susan Racial/Ethnic differences in internalizing and externalizing symptoms in adolescents. JOURNAL OF ABNORMAL CHILD PSYCHOLOGY. 2007;35(5):801-816.

4313. Colton P., Olmsted M., Daneman D., Rydall A., Rodin G. Natural history and predictors of disturbed eating behaviour in girls with Type 1 diabetes. DIABETIC MEDICINE.

2007;24(4):424-429.

4314. Skinner Kayla, Rojas Sasha, Veilleux Jennifer Connecting Eating Pathology with Risk for Engaging in Suicidal Behavior: The Mediating Role of Experiential Avoidance. SUICIDE AND LIFE-THREATENING BEHAVIOR. 2017;47(1):3-13.

4315. Prost-Lehmann Christelle, Shankland Rebecca, Franca Lionel, Laurent Annie, Flaudias Valentin Symptomatology long-term evolution after hospitalization for anorexia nervosa: Drive for thinness to explain effects of body dissatisfaction on type of outcome. PSYCHIATRY RESEARCH. 2018;266():212-217.

4316. Panjwani Anita, Bailey Regan, Kelleher Bridgette COVID-19 and Food-Related Outcomes in Children with Autism Spectrum Disorder: Disparities by Income and Food Security Status. CURRENT DEVELOPMENTS IN NUTRITION. 2021;5(9):.

4317. Juli Maria PERCEPTION OF BODY IMAGE IN EARLY ADOLESCENCE. AN INVESTIGATION IN SECONDARY SCHOOLS. PSYCHIATRIA DANUBINA. 2017;29(3):S409-S415.

4318. Hellings Bridie, Bowles Terry Understanding and managing eating disorders in the school setting. AUSTRALIAN JOURNAL OF GUIDANCE AND COUNSELLING. 2007;17(1):60-67.

4319. Tavolacci Marie-Pierre, Dechelotte Pierre, Ladner Joel Eating Disorders among College Students in France: Characteristics, Help-and Care-Seeking. INTERNATIONAL JOURNAL OF ENVIRONMENTAL RESEARCH AND PUBLIC HEALTH. 2020;17(16):.

4320. Reay Megan, Holliday Joanna, Stewart John, Adams Joanna Creating a care pathway for patients with longstanding, complex eating disorders. JOURNAL OF EATING DISORDERS. 2022;10(1):.

4321. Wilson J., Blizzard L., Gall S., Magnussen C., Oddy W., Dwyer T., Sanderson K., Venn A., Smith K. An eating pattern characterised by skipped or delayed breakfast is associated with mood disorders among an Australian adult cohort. PSYCHOLOGICAL MEDICINE. 2020;50(16):2711-2721.

4322. ROONEY B, MCCLELLAND L, CRISP AH, SEDGWICK PM THE INCIDENCE AND PREVALENCE OF ANOREXIA-NERVOSA IN 3 SUBURBAN HEALTH DISTRICTS IN SOUTH-WEST LONDON, UK. INTERNATIONAL JOURNAL OF EATING DISORDERS. 1995;18(4):299-307.

4323. Richardson Candice, Paslakis Georgios Men's experiences of eating disorder treatment: A qualitative systematic review of men-only studies. JOURNAL OF PSYCHIATRIC AND MENTAL HEALTH NURSING. 2021;28(2):237-250.

4324. Mitchison Deborah, Dawson Lisa, Hand Lucy, Mond Jonathan, Hay Phillipa Quality of life as a vulnerability and recovery factor in eating disorders: a community-based study. BMC PSYCHIATRY. 2016;16():.

4325. Meier Karien, Hoeken Daphne, Hoek Hans Review of the unprecedented impact of the COVID-19 pandemic on the occurrence of eating disorders. CURRENT OPINION IN PSYCHIATRY. 2022;35(6):353-361.

4326. KILLEN JD, TAYLOR CB, HAMMER LD, LITT I, WILSON DM, RICH T, HAYWARD C, SIMMONDS B, KRAEMER H, VARADY A AN ATTEMPT TO MODIFY UNHEALTHFUL EATING ATTITUDES AND WEIGHT REGULATION PRACTICES OF YOUNG ADOLESCENT GIRLS. INTERNATIONAL JOURNAL OF EATING DISORDERS. 1993;13(4):369-384.

4327. Becker Kendra, Keshishian Ani, Liebman Rachel, Coniglio Kathryn, Wang Shirley, Franko Debra, Eddy Kamryn, Thomas Jennifer Impact of expanded diagnostic criteria for avoidant/restrictive food intake disorder on clinical comparisons with anorexia nervosa. INTERNATIONAL JOURNAL OF EATING DISORDERS. 2019;52(3):230-238.

4328. De Caro Elide, Di Blas Lisa A prospective study on the reciprocal influence between personality and attitudes, behaviors, and psychological characteristics salient in eating disorders in a sample of non-clinical adolescents. EATING DISORDERS. 2016;24(5):453-468.

4329. Sokkary Nancy, Oelschlager Anne-Marie, Hornberger Laurie, Care Comm Gynecologic Care for Adolescents and Young Women With Eating Disorders. OBSTETRICS AND GYNECOLOGY. 2018;131(6):E205-E213.

4330. Teng Kathryn Premenopausal osteoporosis, an overlooked consequence of anorexia nervosa. CLEVELAND CLINIC JOURNAL OF MEDICINE. 2011;78(1):50-58.

4331. Haderlein Taona The role of thin-idealization in associations between body dissatisfaction, dieting, and eating pathology: A moderated mediation analysis. CURRENT PSYCHOLOGY. 2020;39(2):550-555.

4332. Carbone Manuel, Della Rocca Filippo NEUROPSYCHIATRIC MANIFESTATIONS OF FAHR'S DISEASE, DIAGNOSTIC AND THERAPEUTIC CHALLENGE: A CASE REPORT AND A LITERATURE REVIEW. CLINICAL NEUROPSYCHIATRY. 2022;19(2):121-131.

4333. Gil Joan, Lopez-Casasnovas Guillem, Mora Toni Taxation of unhealthy consumption of food and drinks: An updated literature review. HACIENDA PUBLICA ESPANOLA-REVIEW OF PUBLIC ECONOMICS. 2013;(207):119-140.

4334. Mead Erin, Gittelsohn Joel, Roache Cindy, Corriveau Andre, Sharma Sangita A Community-Based, Environmental Chronic Disease Prevention Intervention to Improve Healthy Eating Psychosocial Factors and Behaviors in Indigenous Populations in the Canadian Arctic. HEALTH EDUCATION \& BEHAVIOR. 2013;40(5):592-602.

4335. Marmorstein Naomi, Ranson Kristin, Iacono William, Malone Stephen Prospective associations between depressive symptoms and eating disorder symptoms among adolescent girls. INTERNATIONAL JOURNAL OF EATING DISORDERS. 2008;41(2):118-123.

4336. Fredericson Michael, Kussman Andrea, Misra Madhusmita, Barrack Michelle, De Souza Mary, Kraus Emily, Koltun Kristen, Williams Nancy, Joy Elizabeth, Nattiv Aurelia The Male Athlete Triad-A Consensus Statement From the Female and Male Athlete Triad Coalition Part II: Diagnosis, Treatment, and Return-To-Play. CLINICAL JOURNAL OF SPORT MEDICINE. 2021;31(4):349-366.

4337. De Giuseppe Rachele, Di Napoli Ilaria, Porri Debora, Cena Hellas Pediatric Obesity and Eating Disorders Symptoms: The Role of the Multidisciplinary Treatment. A Systematic Review. FRONTIERS IN PEDIATRICS. 2019;7():.

4338. Haderlein Taona, Tomiyama A. Effects of internet-delivered eating disorder prevention on reward-based eating drive: A randomized controlled trial. EATING BEHAVIORS. 2021;43():.

4339. Hahn Samantha, Sonneville Kendrin, Kaciroti Niko, Eisenberg Daniel, Bauer Katherine Relationships between patterns of technology-based weight-related self-monitoring and eating disorder behaviors among first year university students. EATING BEHAVIORS. 2021;42():.

4340. Martin Josune, Padierna Angel, Aguirre Urko, Gonzalez Nerea, Munoz Pedro, Quintana Jose Predictors of quality of life and caregiver burden among maternal and paternal caregivers of patients with eating disorders. PSYCHIATRY RESEARCH. 2013;210(3):1107-1115.

4341. Grange Daniel, Lock James, Loeb Katharine, Nicholls Dasha Academy for Eating Disorders Position Paper: The Role of the Family in Eating Disorders. INTERNATIONAL JOURNAL OF EATING DISORDERS. 2010;43(1):1-5.

4342. LaMarre Andrea, Levine Michael, Holmes Su, Malson Helen An open invitation to productive conversations about feminism and the spectrum of eating disorders (part 1): basic principles of feminist approaches. JOURNAL OF EATING DISORDERS. 2022;10(1):.

4343. Michishita Ryoma, Matsuda Takuro, Kawakami Shotaro, Tanaka Satoshi, Kiyonaga Akira, Tanaka Hiroaki, Morito Natsumi, Higaki Yasuki The association between changes in lifestyle behaviors and the incidence of chronic kidney disease (CKD) in middle-aged and older men. JOURNAL OF EPIDEMIOLOGY. 2017;27(8):389-397.

4344. Hahn Samantha, Hazzard Vivienne, Loth Katie, Larson Nicole, Klein Laura, Neumark-Sztainer Dianne Using apps to self-monitor diet and physical activity is linked to greater use of disordered eating behaviors among emerging adults. PREVENTIVE MEDICINE. 2022;155():.

4345. Spyreli Eleni, McKinley Michelle, Woodside Jayne, Kelly Colette A qualitative exploration of the impact of COVID-19 on food decisions of economically disadvantaged families in Northern Ireland. BMC PUBLIC HEALTH. 2021;21(1):.

4346. Duhaney Tara, Campbell Norm, Niebylski Mark, Kaczorowski Janusz, Tsuyuki Ross, Willis Kevin, Mang Eric, Arango Manuel, Morris Dorothy, Ashley Lisa Death by Diet: The Role of Food Pricing Interventions as a Public Policy Response and Health Advocacy Opportunity. CANADIAN JOURNAL OF CARDIOLOGY. 2015;31(2):.

4347. Guller Leila, Zapolski Tamika, Smith Gregory Personality Measured in Elementary School Predicts Middle School Addictive Behavior Involvement. JOURNAL OF PSYCHOPATHOLOGY AND BEHAVIORAL ASSESSMENT. 2015;37(3):523-532.

4348. Javed Asma, Tebben Peter, Fischer Philip, Lteif Aida Female Athlete Triad and Its Components: Toward Improved Screening and Management. MAYO CLINIC PROCEEDINGS. 2013;88(9):996-1009.

4349. Aulinas Anna, Marengi Dean, Galbiati Francesca, Asanza Elisa, Slattery Meghan, Mancuso Christopher, Wons Olivia, Micali Nadia, Bern Elana, Eddy Kamryn, Thomas Jennifer, Misra Madhusmita, Lawson Elizabeth Medical comorbidities and endocrine dysfunction in low-weight females with avoidant/restrictive food intake disorder compared to anorexia nervosa and healthy controls. INTERNATIONAL JOURNAL OF EATING DISORDERS. 2020;53(4):631-636.

4350. Kambanis P., Bottera Angeline, Mancuso Christopher, Spoor Samantha, Anderson Lisa, Burke Natasha, Eddy Kamryn, Forbush Kelsie, Keith Jill, Lavender Jason, Mensinger Janell, Mujica Christin, Nagata Jason, Perez Marisol, De Young Kyle Eating Disorder Examination-Questionnaire and Clinical Impairment Assessment norms for intersectional identities using an MTurk sample. INTERNATIONAL JOURNAL OF EATING DISORDERS. 2022;55(12):1690-1707.

4351. Gibson Edward, Androutsos Odysseas, Moreno Luis, Flores-Barrantes Paloma, Socha Piotr, Iotova Violeta, Cardon Greet, De Bourdeaudhuij Ilse, Koletzko Berthold, Skripkauskaite Simona, Manios Yannis, Grp Toybox-Study Influences of Parental Snacking-Related Attitudes, Behaviours and Nutritional Knowledge on Young Children's Healthy and Unhealthy Snacking: The ToyBox Study. NUTRIENTS. 2020;12(2):.

4352. Agaoglu Esra, Erdogan Hilal, Acer Ersoy, Atay Emrah, Metintas Selma, Saracoglu Zeynep Prevalence of early-onset androgenetic alopecia and its relationship with lifestyle and dietary habits. ITALIAN JOURNAL OF DERMATOLOGY AND VENEREOLOGY. 2021;156(6):675-680.

4353. Hoybye Charlotte, Tauber Maithe Approach to the Patient With Prader-Willi Syndrome. JOURNAL OF CLINICAL ENDOCRINOLOGY \& METABOLISM. 2022;107(6):1698-1705.

4354. Wertheim EH, Koerner J, Paxton SJ Longitudinal predictors of restrictive eating and bulimic tendencies in three different age groups of adolescent girls. JOURNAL OF YOUTH

AND ADOLESCENCE. 2001;30(1):69-81.

4355. Tabuchi K, Kusakari J, Ito Z, Takahashi K, Wada T, Hara A Effect of nitric oxide synthase inhibitor on cochlear dysfunction induced by transient local anoxia. ACTA OTO-LARYNGOLOGICA. 1999;119(2):179-184.

4356. Bjorkenstam Emma, Cheng Siwei, Burstrom Bo, Pebley Anne, Bjorkenstam Charlotte, Kosidou Kyriaki Association between income trajectories in childhood and psychiatric disorder: a Swedish population-based study. JOURNAL OF EPIDEMIOLOGY AND COMMUNITY HEALTH. 2017;71(7):648-654.

4357. Wilksch Simon, O'Shea Anne, Wade Tracey Media Smart-Targeted: Diagnostic outcomes from a two-country pragmatic online eating disorder risk reduction trial for young adults. INTERNATIONAL JOURNAL OF EATING DISORDERS. 2018;51(3):270-274.

4358. Doumit Rita, Zeeni Nadine, Ruiz Maria, Khazen Georges Anxiety as a Moderator of the Relationship Between Body Image and Restrained Eating. PERSPECTIVES IN PSYCHIATRIC CARE. 2016;52(4):254-264.

4359. Sundgot-Borgen Jorunn, Torstveit Monica The female football player, disordered eating, menstrual function and bone health. BRITISH JOURNAL OF SPORTS MEDICINE. 2007;41(1):I68-I72.

4360. Kakuma Tetsuya, Yoshida Yuichi, Okamoto Mitsuhiro, Shibata Hirotaka, Tsutsumi Takashi, Kudo Yoshikuni Effects of Self-Awareness of Eating Behaviors and Differences in Daily Habits Among Japanese University Students on Changes in Weight and Metabolism. JOURNAL OF ENDOCRINOLOGY AND METABOLISM. 2020;10(5):131-139.

4361. Hunt Rowan, Levinson Cheri, Peiper Nicholas Leveraging general risk surveillance to reduce suicide in early adolescents: Associations between suicidality, disordered eating, and other developmental risk factors. SUICIDE AND LIFE-THREATENING BEHAVIOR. 2021;51(2):247-254.

4362. Sahlan Reza, Akoury Liya, Habashy Jessica, Culbert Kristen, Warren Cortney Sociocultural correlates of eating pathology in college women from US and Iran. FRONTIERS IN PSYCHOLOGY. 2022;13():.

4363. Napoli A., Framarino M., Colatrella A., Merola G., Trappolini M., Toscano V., Talucci V., Iafusco D. Eating disorders and diabetic ketoacidosis in a pregnant woman with type 1 diabetes: A case report. EATING AND WEIGHT DISORDERS-STUDIES ON ANOREXIA BULIMIA AND OBESITY. 2011;16(2):E146-E149.

4364. Nicholls Ben, Ang Chee, Kanjo Eiman, Siriaraya Panote, Bafti Saber, Yeo Woon-Hong, Tsanas Athanasios An EMG-based Eating Behaviour Monitoring system with haptic feedback to promote mindful eating. COMPUTERS IN BIOLOGY AND MEDICINE. 2022;149():.

4365. Chen Gui, He Jinbo, Zhang Bin, Fan Xitao Revisiting the relationship between body dissatisfaction and eating disorder symptoms in Chinese adolescents: the mediating roles of regulatory emotional self-efficacy and depression symptoms. EATING AND WEIGHT DISORDERS-STUDIES ON ANOREXIA BULIMIA AND OBESITY. 2021;26(1):239-247.

4366. Carrard Isabelle, Della Torre Sophie, Levine Michael Promoting a positive body image in young people. SANTE PUBLIQUE. 2019;31(4):507-515.

4367. Mansouri Vahid, Mansourian Marjan, Qorbani Mostafa, Riahi Roya, Karimi Rahele, Motlagh Mohammad, Heshmat Ramin, Kelishadi Roya Path Analysis on Determinants of Childhood Obesity and Associated Risk Factors of Cardiovascular, Renal, and Hepatic Diseases: The CASPIAN-V Study. JOURNAL OF CHILD SCIENCE. 2020;10(1):E148-E156.

4368. Striegel-Moore RH, McMahon RP, Biro FM, Schreiber G, Crawford PB, Voorhees C Exploring the relationship between timing of menarche and eating disorder symptoms in black and white adolescent girls. INTERNATIONAL JOURNAL OF EATING DISORDERS. 2001;30(4):421-433.

4369. Triffleman Elisa, Pole Nnamdi Future Directions in Studies of Trauma Among Ethnoracial and Sexual Minority Samples: Commentary. JOURNAL OF CONSULTING AND CLINICAL PSYCHOLOGY. 2010;78(4):490-497.

4370. Anastasiadou Dimitra, Parks Melissa, Brugnera Agostino, Sepulveda Ana, Graell Montserrat Psychiatric comorbidity and maternal distress among adolescent eating disorder patients: A comparison with substance use disorder patients. EATING BEHAVIORS. 2017;24():74-80.

4371. Baker Amy, Maiorino Elizabeth Assessments of emotional abuse and neglect with the CTQ: Issues and estimates. CHILDREN AND YOUTH SERVICES REVIEW. 2010;32(5):740-748.

4372. Diaz Rosa, Goti Javier, Garcia Montse, Gual Antoni, Serrano Lourdes, Gonzalez Laura, Calvo Rosa, Castro-Fornieles Josefina Patterns of substance use in adolescents attending a mental health department. EUROPEAN CHILD \& ADOLESCENT PSYCHIATRY. 2011;20(6):279-289.

4373. Bruin A., Oudejans Raoul Athletes' Body Talk: The Role of Contextual Body Image in Eating Disorders as Seen Through the Eyes of Elite Women Athletes. JOURNAL OF CLINICAL SPORT PSYCHOLOGY. 2018;12(4, SI):675-698.

4374. Bailey Rachel, Wang Tianjiao, Liu Jiawei, Clayton Russell, Kwon Kyeongwon, Diwanji Vaibhav, Karimkhanashtiyani Farzaneh Social Facilitation in Fear Appeals Creates Positive Affect but Inhibits Healthy Eating Intentions. FRONTIERS IN PSYCHOLOGY. 2022;13():.

4375. Dondzilo Laura, Rieger Elizabeth, Palermo Romina, Byrne Susan, Bell Jason The mediating role of rumination in the relation between attentional bias towards thin female bodies and eating disorder symptomatology. PLOS ONE. 2017;12(5):.

4376. Oved Irit, Vaiman Inbal, Hod Keren, Mardy-Tilbor Limor, Torban Yakov, Dagan Shiri Poor Health Behaviors Prior to Laparoscopic Sleeve Gastrectomy Surgery. OBESITY SURGERY. 2017;27(2):469-475.

4377. Mueller Sina, Stice Eric Moderators of the intervention effects for a dissonance-based eating disorder prevention program; results from an amalgam of three randomized trials. BEHAVIOUR RESEARCH AND THERAPY. 2013;51(3):128-133.

4378. Juli Maria THE SUFFERING BODY: MANIPULATION AND DISCOMFORT IN EATING DISORDERS. PSYCHIATRIA DANUBINA. 2018;30(7):521-526.

4379. Moludi Jalal, Moradinazar Mehdi, Hamzeh Behrooz, Najafi Farid, Soleimani Davood, Pasdar Yahya Depression Relationship with Dietary Patterns and Dietary In fl ammatory Index in Women: Result from Ravansar Cohort Study. NEUROPSYCHIATRIC DISEASE AND TREATMENT. 2020;16():1595-1603.

4380. Speranza M, Corcos M, Godart N, Jeammet P, Flament M Current and lifetime prevalence of obsessive compulsive disorders in eating disorders. ENCEPHALE-REVUE DE PSYCHIATRIE CLINIQUE BIOLOGIQUE ET THERAPEUTIQUE. 2001;27(6):541-550.

4381. Haltom Cris, Halverson Tate Relationship between college lifestyle variables, eating disorder education, and eating disorder risk. JOURNAL OF AMERICAN COLLEGE HEALTH. 2022;():.

4382. Penniment Kylie, Egan Sarah Perfectionism and learning experiences in dance class as risk factors for eating disorders in dancers. EUROPEAN EATING DISORDERS REVIEW. 2012;20(1):13-22.

4383. Prather Heidi, Hunt Devyani, McKeon Kathryn, Simpson Scott, Meyer E., Yemm Ted, Brophy Robert Are Elite Female Soccer Athletes at Risk for Disordered Eating Attitudes, Menstrual Dysfunction, and Stress Fractures?. PM\&R. 2016;8(3):208-213.

4384. Berger Uwe, Hentrich Isabel, Wick Katharina, Bormann Bianca, Brix Christina, Sowa Melanie, Schwartze Dominique, Strauss Bernhard Psychometric Quality of the ``Eating Attitudes Test{''} (German Version EAT-26D) for Measuring Disordered Eating in Pre-Adolescents and Proposal for a 13-Item Short Version. PSYCHOTHERAPIE PSYCHOSOMATIK MEDIZINISCHE PSYCHOLOGIE. 2012;62(6):223-226.

4385. Andersen Karl, Gudnason Vilmundur Health policy interventions: The pathway to public health. LAEKNABLADID. 2013;99(3):129-134.

4386. Limone Pierpaolo, Messina Giovanni, Toto Giusi Serious games and eating behaviors: A systematic review of the last 5 years (2018-2022). FRONTIERS IN NUTRITION. 2022;9():.

4387. Sira Natalia, Pawlak Roman Prevalence of overweight and obesity, and dieting attitudes among Caucasian and African American college students in Eastern North carolina: A cross-sectional survey. NUTRITION RESEARCH AND PRACTICE. 2010;4(1):36-42.

4388. Schneider Lisa, Warren Michelle Functional hypothalamic amenorrhea is associated with elevated ghrelin and disordered eating. FERTILITY AND STERILITY. 2006;86(6):1744-1749.

4389. Van Son Gabrielle, Van Hoeken Daphne, Bartelds Aad, Van Furth Eric, Hoek Hans Urbanisation and the incidence of eating disorders. BRITISH JOURNAL OF PSYCHIATRY. 2006;189():562-563.

4390. Kopp W, Jacoby GE The impact of child sexual abuse (CSA) on the result of psychotherapy. An investigation of eating-disordered female patients two years after inpatient therapy. PSYCHOTHERAPIE PSYCHOSOMATIK MEDIZINISCHE PSYCHOLOGIE. 1996;46(3-4):131-138.

4391. Wimmer-Puchinger B., Strobich M. Eating disorders: a serious and underestimated public health issue, eating disorder prevention campaign, city of Vienna/Austria. EUROPEAN JOURNAL OF PUBLIC HEALTH. 2006;16(1):67-68.

4392. Wang Tiange, Xu Min, Bi Yufang, Ning Guang Interplay between diet and genetic susceptibility in obesity and related traits. FRONTIERS OF MEDICINE. 2018;12(6):601-607.

4393. Hoque Kazi, Kamaluddin Megat, Razak Ahmad, Wahid Afiq Building healthy eating habits in childhood: a study of the attitudes, knowledge and dietary habits of schoolchildren in Malaysia. PEERJ. 2016;4():.

4394. Michaud Andreanne, Vainik Uku, Garcia-Garcia Isabel, Dagher Alain Overlapping Neural Endophenotypes in Addiction and Obesity. FRONTIERS IN ENDOCRINOLOGY. 2017;8():.

4395. Javaras K., Rickert M., Thornton L., Peat C., Baker J., Birgegard A., Norring C., Landen M., Almqvist C., Larsson H., Lichtenstein P., Bulik C., D'Onofrio B. Paternal age at childbirth and eating disorders in offspring. PSYCHOLOGICAL MEDICINE. 2017;47(3):576-584.

4396. DelBello M, Grcevich S Phenomenology and epidemiology of childhood psychiatric disorders that may necessitate treatment with atypical antipsychotics. JOURNAL OF CLINICAL PSYCHIATRY. 2004;65(6):12-19.

4397. Elberling H, Skovgaard AM Children aged 0-3 years referred to child psychiatric department - a descriptive epidemiological study. UGESKRIFT FOR LAEGER. 2002;164(48):5658-5661.

4398. Bargiacchi A. Brain imaging in early onset anorexia. ARCHIVES DE PEDIATRIE. 2014;21(5):548-551.

4399. Wisting Line, Siegwarth Cecilie, Skrivarhau Torild, Dahl-Jorgensen Knut, Ro Oyvind The impact of psychological aspects, age, and BMI on eating disorder psychopathology among adult males and females with type 1 diabetes. HEALTH PSYCHOLOGY OPEN. 2020;7(2):.

4400. Brown Tiffany, Forney K., Klein Kelly, Grillot Charlotte, Keel Pamela A 30-Year Longitudinal Study of Body Weight, Dieting, and Eating Pathology Across Women and Men From Late Adolescence to Later Midlife. JOURNAL OF ABNORMAL PSYCHOLOGY. 2020;129(4):376-386.

4401. Dakanalis Antonios, Clerici Massimo, Bartoli Francesco, Caslini Manuela, Crocamo Cristina, Riva Giuseppe, Carra Giuseppe Risk and maintenance factors for young women's DSM-5 eating disorders. ARCHIVES OF WOMENS MENTAL HEALTH. 2017;20(6):721-731.

4402. Fitzsimmons-Craft Ellen, Balantekin Katherine, Eichen Dawn, Graham Andrea, Monterubio Grace, Sadeh-Sharvit Shiri, Goel Neha, Flatt Rachael, Saffran Kristina, Karam Anna, Firebaugh Marie-Laure, Trockel Mickey, Taylor C., Wilfley Denise Screening and offering online programs for eating disorders: Reach, pathology, and differences across eating disorder status groups at 28 US universities. INTERNATIONAL JOURNAL OF EATING DISORDERS. 2019;52(10, SI):1125-1136.

4403. Ganson Kyle, Rodgers Rachel, Lipson Sarah, Cadet Tamara, Putnam Michelle Sexual Assault Victimization and Eating Disorders Among College-enrolled Men. JOURNAL OF INTERPERSONAL VIOLENCE. 2022;37(7-8):NP5143-NP5166.

4404. Nagy-Penzes Gabriella, Vincze Ferenc, Biro Eva A School Intervention's Impact on Adolescents' Health-Related Knowledge and Behavior. FRONTIERS IN PUBLIC HEALTH. 2022;10():.

4405. Levinson Cheri, Christian Caroline, Vanzhula Irina Manipulating the theoretical framing of exposure therapy for eating disorders impacts clinicians' treatment preferences. EATING AND WEIGHT DISORDERS-STUDIES ON ANOREXIA BULIMIA AND OBESITY. 2020;25(5):1205-1212.

4406. Yao Liangshuang, Niu Gengfeng, Sun Xiaojun Body Image Comparisons on Social Networking Sites and Chinese Female College Students' Restrained Eating: The Roles of Body Shame, Body Appreciation, and Body Mass Index. SEX ROLES. 2021;84(7-8):465-476.

4407. Podina Ioana, Fodor Liviu, Cosmoiu Ana, Boian Rares An evidence-based gamified mHealth intervention for overweight young adults with maladaptive eating habits: study protocol for a randomized controlled trial. TRIALS. 2017;18():.

4408. Kelly Sarah, Martin Steven, Kuhn Isla, Cowan Andy, Brayne Carol, Lafortune Louise Barriers and Facilitators to the Uptake and Maintenance of Healthy Behaviours by People at Mid-Life: A Rapid Systematic Review. PLOS ONE. 2016;11(1):.

4409. Kwan Mun, Haynos Ann, Blomquist Kerstin, Roberto Christina Warning labels on fashion images: Short- and longer-term effects on body dissatisfaction, eating disorder symptoms, and eating behavior. INTERNATIONAL JOURNAL OF EATING DISORDERS. 2018;51(10):1153-1161.

4410. Will MJ, Franzblau EB, Kelley AE The amygdala is critical for opioid-mediated binge eating of fat. NEUROREPORT. 2004;15(12):1857-1860.

4411. Schaefer Lauren, Steinglass Joanna Reward Learning Through the Lens of RDoC: a Review of Theory, Assessment, and Empirical Findings in the Eating Disorders. CURRENT PSYCHIATRY REPORTS. 2021;23(1):.

4412. Graham Andrea, Fitzsimmons-Craft Ellen, Sadeh-Sharvit Shiri, Balantekin Katherine, Eichen Dawn, Firebaugh Marie-Laure, Goel Neha, Monterubio Grace, Karam Anna, Flatt Rachael, Jo Booil, Jacobi Corinna, Wilfley Denise, Taylor C., Trockel Mickey Moderators and Mediators of a Digital Cognitive Behavior Therapy-Guided Self-Help Intervention for Eating Disorders: Informing Future Design Efforts. JOURNAL OF CONSULTING AND CLINICAL PSYCHOLOGY. 2023;91(5):280-284.

4413. Schtscherbyna Annie, Soares Eliane, Oliveira Fatima, Ribeiro Beatriz Female athlete triad in elite swimmers of the city of Rio de Janeiro, Brazil. NUTRITION. 2009;25(6):634-639.

4414. Bowen Deborah, Quintiliani Lisa, Bhosrekar Sarah, Goodman Rachel, Smith Eugenia Changing the housing environment to reduce obesity in public housing residents: a cluster

randomized trial. BMC PUBLIC HEALTH. 2018;18():.

4415. Stewart TM, Williamson DA, White MA Rigid vs. flexible dieting: association with eating disorder symptoms in nonobese women. APPETITE. 2002;38(1):39-44.

4416. Mascola Anthony, Bryson Susan, Agras W. Picky eating during childhood: A longitudinal study to age 11 years. EATING BEHAVIORS. 2010;11(4):253-257.

4417. Pearson Natalie, Biddle Stuart, Griffiths Paula, Johnston Julie, Haycraft Emma Clustering and correlates of screen-time and eating behaviours among young children. BMC PUBLIC HEALTH. 2018;18():.

4418. Murray Helen, Calabrese Samantha Identification and Management of Eating Disorders (including ARFID) in GI Patients. GASTROENTEROLOGY CLINICS OF NORTH AMERICA. 2022;51(4):765-783.

4419. Lemamsha H., Papadopoulos C., Randhawa G. Understanding the risk and protective factors associated with obesity amongst Libyan adults - a qualitative study. BMC PUBLIC HEALTH. 2018;18():.

4420. Viguria Iranzu, Angel Alvarez-Mon Miguel, Llavero-Valero Maria, Barco Angel, Ortuno Felipe, Alvarez-Mon Melchor Eating Disorder Awareness Campaigns: Thematic and Quantitative Analysis Using Twitter. JOURNAL OF MEDICAL INTERNET RESEARCH. 2020;22(7):.

4421. Bellastella Giuseppe, Scappaticcio Lorenzo, Esposito Katherine, Giugliano Dario, Maiorino Maria Metabolic syndrome and cancer: ``The common soil hypothesis{''}. DIABETES RESEARCH AND CLINICAL PRACTICE. 2018;143():389-397.

4422. Sandgren Sebastian, Haycraft Emma, Plateau Carolyn Nature and efficacy of interventions addressing eating psychopathology in athletes: A systematic review of randomised and nonrandomised trials. EUROPEAN EATING DISORDERS REVIEW. 2020;28(2):105-121.

4423. Striley Catherine, Kelso-Chichetto Natalie, Cottler Linda Nonmedical Prescription Stimulant Use Among Girls 10-18 Years of Age: Associations With Other Risky Behavior.

JOURNAL OF ADOLESCENT HEALTH. 2017;60(3):328-332.

4424. Suka Machi, Yamauchi Takashi, Yanagisawa Hiroyuki Lifestyle Changes and Weight Gain A 2-Year Follow-up Study of Japanese Workers. JOURNAL OF OCCUPATIONAL AND ENVIRONMENTAL MEDICINE. 2020;62(7):E318-E327.

4425. Keshishian Ani, Christian Caroline, Williams Brenna, Spoor Samantha, Peiper Nicholas, Levinson Cheri A Network Analysis Investigation of Disordered Eating Across Demographic and Developmental Subpopulations Using a National Epidemiological Sample of High School Students. BEHAVIOR THERAPY. 2022;53(3):535-545.

4426. Babin A., Chechetkina E., Koltunov I. Psychosomatic aspects of obesity as a risk factor of metabolic syndrome. CARDIOVASCULAR THERAPY AND PREVENTION. 2010;9(7):71-78.

4427. Blechert Jens, Naumann Eva, Schmitz Julian, Herbert Beate, Tuschen-Caffier Brunna Startling Sweet Temptations: Hedonic Chocolate Deprivation Modulates Experience, Eating Behavior, and Eyeblink Startle. PLOS ONE. 2014;9(1):.

4428. Uragami Ryoko, Kojima Yayoi, Sawamiya Yoko Relation Between Thin-Ideal Internalization and Drive for Thinness in Male and Female Adolescents. JAPANESE JOURNAL OF EDUCATIONAL PSYCHOLOGY. 2013;61(2):146-157.

4429. Swenne I Changes in body weight and body mass index (BMI) in teenage girls prior to the onset and diagnosis of an eating disorder. ACTA PAEDIATRICA. 2001;90(6):677-681.

4430. Rosenblatt Daniel, Summerell Patrick, Ng Alyssa, Dixon Helen, Murawski Carsten, Wakefield Melanie, Bode Stefan Food product health warnings promote dietary self-control through reductions in neural signals indexing food cue reactivity. NEUROIMAGE-CLINICAL. 2018;18():702-712.

4431. Sanchez-Cerezo Javier, Nagularaj Lidushi, Gledhill Julia, Nicholls Dasha What do we know about the epidemiology of avoidant/restrictive food intake disorder in children and adolescents? A systematic review of the literature. EUROPEAN EATING DISORDERS REVIEW. 2023;31(2):226-246.

4432. Hyam Lucy, Richards Katie, Allen Karina, Schmidt Ulrike The impact of the COVID-19 pandemic on referral numbers, diagnostic mix, and symptom severity in Eating Disorder

Early Intervention Services in England. INTERNATIONAL JOURNAL OF EATING DISORDERS. 2023;56(1, SI):269-275.

4433. PAGSBERG AK, WANG AR EPIDEMIOLOGY OF ANOREXIA-NERVOSA AND BULIMIA-NERVOSA IN BORNHOLM COUNTY, DENMARK, 1970-1989. ACTA PSYCHIATRICA SCANDINAVICA. 1994;90(4):259-265.

4434. Galmiche Marie, Achamrah Najate, Dechelotte Pierre, Ribet David, Breton Jonathan Role of microbiota-gut-brain axis dysfunctions induced by infections in the onset of anorexia nervosa. NUTRITION REVIEWS. 2022;80(3):381-391.

4435. Mento Carmela, Silvestri Maria, Muscatello Maria, Rizzo Amelia, Celebre Laura, Pratico Martina, Zoccali Rocco, Bruno Antonio Psychological Impact of Pro-Anorexia and Pro-Eating Disorder Websites on Adolescent Females: A Systematic Review. INTERNATIONAL JOURNAL OF ENVIRONMENTAL RESEARCH AND PUBLIC HEALTH. 2021;18(4):.

4436. Orji Rita, Mandryk Regan Developing culturally relevant design guidelines for encouraging healthy eating behavior. INTERNATIONAL JOURNAL OF HUMAN-COMPUTER STUDIES. 2014;72(2):207-223.

4437. Moreno Encinas Alba, Moraleda Merino Jessica, Graell-Berna Montserrat, Villa-Asensi Jose, Alvarez Tamara, Lacruz-Gascon Tatiana, Sepulveda Garcia Ana Internalization and exteriorization model to explain the beginning of the psychopathology of eating disorders in adolescence. BEHAVIORAL PSYCHOLOGY-PSICOLOGIA CONDUCTUAL. 2021;29(1):51-72.

4438. Convertino Alexandra, Helm Jonathan, Pennesi Jamie-Lee, Gonzales Manuel, Blashill Aaron Integrating minority stress theory and the tripartite influence model: A model of eating disordered behavior in sexual minority young adults. APPETITE. 2021;163():.

4439. Imaizumi T, Kocsis JD, Waxman SG Resistance to anoxic injury in the dorsal columns of adult rat spinal cord following demyelination. BRAIN RESEARCH. 1998;779(1-2):292-296.

4440. Christensen Kara, Short Nicole The case for investigating a bidirectional association between insomnia symptoms and eating disorder pathology. INTERNATIONAL JOURNAL OF EATING DISORDERS. 2021;54(5):701-707.

4441. Pauuvale Alvina, Vickers Mark, Pamaka Soana, Apelu Dorothy, Fehoko Anaseini, Ofanoa Malakai, Bay Jacquie Exploring the Retail Food Environment Surrounding Two Secondary Schools with Predominantly Pacific Populations in Tonga and New Zealand to Enable the Development of Mapping Methods Appropriate for Testing in a Classroom. INTERNATIONAL JOURNAL OF ENVIRONMENTAL RESEARCH AND PUBLIC HEALTH. 2022;19(23):.

4442. Allgaier Katharina, Schneider Priska, Buck Simone, Reusch Pauline, Hagmann Daniela, Barth Gottfried, Renner Tobias Child and Adolescent Psychiatric Emergencies During the Second Wave of the SARS-CoV2-19 Pandemic: Findings from the Tubingen University Hospital. ZEITSCHRIFT FUR KINDER-UND JUGENDPSYCHIATRIE UND PSYCHOTHERAPIE. 2022;50(4):275-285.

4443. Wimmer-Puchinger Beate Eating disorders: A serious and underestimated public health issue: The eating disorder prevention campaign of Vienna womens health program. JOURNAL OF PSYCHOSOMATIC OBSTETRICS AND GYNECOLOGY. 2007;28(1):70.

4444. Simopoulos Artemis, Bourne Peter, Faergeman Ole BELLAGIO REPORT ON HEALTHY AGRICULTURE, HEALTHY NUTRITION, HEALTHY PEOPLE. NUTRICION HOSPITALARIA. 2013;28(6):1761-1769.

4445. Leme Ana, Haines Jess, Tang Lisa, Dunker Karin, Philippi Sonia, Fisberg Mauro, Ferrari Gerson, Fisberg Regina Impact of Strategies for Preventing Obesity and Risk Factors for Eating Disorders among Adolescents: A Systematic Review. NUTRIENTS. 2020;12(10):.

4446. Musaiger Abdulrahman, Nabag Fatima, Al-Mannai Mariam Obesity, Dietary Habits, and Sedentary Behaviors Among Adolescents in Sudan: Alarming Risk Factors for Chronic Diseases in a Poor Country. FOOD AND NUTRITION BULLETIN. 2016;37(1):65-72.

4447. Saucedo-Molina Teresita, Villarreal Castillo Martin, Oliva Macias Luz, Unikel Santoncini Claudia, Guzman Saldana Rebeca Disordered eating behaviours and sedentary lifestyle prevention among young Mexicans: A pilot study. HEALTH EDUCATION JOURNAL. 2018;77(8):872-883.

4448. Calvert Sian, Dempsey Robert, Povey Rachel, Clark-Carter David An in-school social norms approach intervention for reducing unhealthy snacking behaviours amongst 11-12-year-olds. BRITISH JOURNAL OF HEALTH PSYCHOLOGY. 2022;27(3):891-914.

4449. Lee Mi, Amorin-Woods Lyndon, Cascioli Vincenzo, Adams Jon The use of nutritional guidance within chiropractic patient management: a survey of 333 chiropractors from the ACORN practice-based research network. CHIROPRACTIC \& MANUAL THERAPIES. 2018;26():.

4450. Buhler Susan, Raine Kim, Arango Manuel, Pellerin Suzie, Neary Neil Building a Strategy for Obesity Prevention One Piece at a Time: The Case of Sugar-Sweetened Beverage Taxation. CANADIAN JOURNAL OF DIABETES. 2013;37(2):97-102.

4451. Simopoulos Artemis, Bourne Peter, Faergeman Ole Bellagio Report on Healthy Agriculture, Healthy Nutrition, Healthy People. NUTRIENTS. 2013;5(2):411-423.

4452. Ward Kara, Happel Kyle An eating disorder leading to wet beriberi heart failure in a 30-year-old woman. AMERICAN JOURNAL OF EMERGENCY MEDICINE. 2013;31(2):.

4453. Effertz Tobias Marketing of unhealthy food products to children. MONATSSCHRIFT KINDERHEILKUNDE. 2022;170(2, SI):133-138.

4454. Khalid Shakeela, Arshad Muhammad, Mahmood Shahid, Siddique Farzana, Roobab Ume, Ranjha Muhammad, Lorenzo Jose Extraction and Quantification of Moringa oleifera Leaf Powder Extracts by HPLC and FTIR. FOOD ANALYTICAL METHODS. 2023;():.

4455. Attia E, Schroeder L Pharmacologic treatment of anorexia nervosa: Where do we go from here?. INTERNATIONAL JOURNAL OF EATING DISORDERS. 2005;37(S):S60-S63.

4456. Wentz Elisabet, Mellstroem Dan, Gillberg I., Gillberg Christopher, Rastant Maria Brief report: Decreased bone mineral density as a long-term complication of teenage-onset anorexia nervosa. EUROPEAN EATING DISORDERS REVIEW. 2007;15(4):290-295.

4457. Ybarra J, Stefano M, Kammer A, Tonnac N, Lehmann T, Golay A Interest of pronostic score for optimal clinical management of obese patients. DIABETES \& METABOLISM. 2003;29(4, 1):418-423.

4458. Mathis Bryan, Tanaka Kiyoji, Hiramatsu Yuji Metabolically Healthy Obesity: Are Interventions Useful?. CURRENT OBESITY REPORTS. 2023;12(1):36-60.

4459. Verpeut Jessica, Bello Nicholas Drug safety evaluation of naltrexone/bupropion for the treatment of obesity. EXPERT OPINION ON DRUG SAFETY. 2014;13(6):831-841.

4460. Hawkes Corinna, Smith Trenton, Jewell Jo, Wardle Jane, Hammond Ross, Friel Sharon, Thow Anne, Kain Juliana Smart food policies for obesity prevention. LANCET. 2015;385(9985):2410-2421.

4461. Lohr C., Fladung A. Stress, emotion regulation and risk of eating disorder in adolescence - results of a survey among schoolchildren. NERVENHEILKUNDE. 2012;31(6):461-466.

4462. Micali Nadia, Daniel Rhian, Ploubidis George, De Stavola Bianca Maternal Prepregnancy Weight Status and Adolescent Eating Disorder Behaviors A Longitudinal Study of Risk Pathways. EPIDEMIOLOGY. 2018;29(4):579-589.

4463. HALL P, DRISCOLL R ANOREXIA IN THE ELDERLY - AN ANNOTATION. INTERNATIONAL JOURNAL OF EATING DISORDERS. 1993;14(4):497-499.

4464. Atlantis E., Baker M. Obesity effects on depression: systematic review of epidemiological studies. INTERNATIONAL JOURNAL OF OBESITY. 2008;32(6):881-891.

4465. Doba K., Nandrino J. Is there a family typology of addictive behaviors? Critical review of the literature in the families of adolescents with an eating disorder or with a substance-dependence. PSYCHOLOGIE FRANCAISE. 2010;55(4):355-371.

4466. Thomas Jennifer, Judge Abigail, Brownell Kelly, Vartanian Lenny Evaluating the effects of eating disorder memoirs on readers' eating attitudes and behaviors. INTERNATIONAL JOURNAL OF EATING DISORDERS. 2006;39(5):418-425.

4467. Abed Riadh, Mehta Sunil, Figueredo Aurelio, Aldridge Sarah, Balson Hannah, Meyer Caroline, Palmer Robert Eating Disorders and Intrasexual Competition: Testing an Evolutionary Hypothesis among Young Women. SCIENTIFIC WORLD JOURNAL. 2012;():.

4468. Berger Uwe, Wick Katharina, Hoelling Heike, Schlack Robert, Bormann Bianca, Brix Christina, Sowa Melanie, Schwartze Dominique, Strauss Bernhard Screening of Disordered Eating in 12-Year-Old Girls and Boys: Psychometric Analysis of the German Versions of SCOFF and EAT-26. PSYCHOTHERAPIE PSYCHOSOMATIK MEDIZINISCHE PSYCHOLOGIE.

2011;61(7):311-318.

4469. Henriksson Hanna, Alexandrou Christina, Henriksson Pontus, Henstrom Maria, Bendtsen Marcus, Thomas Kristin, Mussener Ulrika, Nilsen Per, Lof Marie MINISTOP 2.0: a smartphone app integrated in primary child health care to promote healthy diet and physical activity behaviours and prevent obesity in preschool-aged children: protocol for a hybrid design effectiveness-implementation study. BMC PUBLIC HEALTH. 2020;20(1):.

4470. Andersen Susanne, Lindgreen Pil, Rokkedal Kristian, Clausen Loa Grasping the weight cut-off for anorexia nervosa in children and adolescents. INTERNATIONAL JOURNAL OF EATING DISORDERS. 2018;51(12):1346-1351.

4471. Baltic Ryan, Weier Rory, Katz Mira, Kennedy Stephenie, Lengerich Eugene, Lesko Samuel, Reese David, Roberto Karen, Schoenberg Nancy, Young Gregory, Dignan Mark, Paskett Electra Study design, intervention, and baseline characteristics of a group randomized trial involving a faith-based healthy eating and physical activity intervention (Walk by Faith) to reduce weight and cancer risk among overweight and obese Appalachian adults. CONTEMPORARY CLINICAL TRIALS. 2015;44():1-10.

4472. Tapajoz Fernanda, Soneira Sebastian, Allegri Ricardo Cognitive functioning as an endophenotype of anorexia nervosa. A study in first-degree relatives unaffected by the disease. INTERDISCIPLINARIA. 2022;39(1):113-126.

4473. Versini Audrey, Ramoz Nicolas, Le Strat Yann, Scherag Susann, Ehrlich Stefan, Boni Claudette, Hinney Anke, Hebebrand Johannes, Romo Lucia, Guelfi Julien-Daniel, Gorwood Philip Estrogen Receptor 1 Gene (ESR1) is Associated with Restrictive Anorexia Nervosa. NEUROPSYCHOPHARMACOLOGY. 2010;35(8):1818-1825.

4474. Shankar-Krishnan Nithya, Fornieles Deu Albert, Sanchez-Carracedo David Associations Between Food Insecurity And Psychological Wellbeing, Body Image, Disordered Eating And Dietary Habits: Evidence From Spanish Adolescents. CHILD INDICATORS RESEARCH. 2021;14(1):163-183.

4475. Rinella Sergio, Massimino Simona, Giunta Alessandra, Perciavalle Valentina, Coco Marinella EATING BEHAVIOR AND PERSONOLOGICAL FEATURES OF AN ITALIAN SAMPLE OF UNIVERSITY STUDENTS: A PILOT STUDY. JOURNAL OF PSYCHOLOGICAL AND EDUCATIONAL RESEARCH. 2018;26(2):33-47.

4476. Jensen Henning, Keogh-Brown Marcus, Shankar Bhavani, Aekplakorn Wichai, Basu Sanjay, Cuevas Soledad, Dangour Alan, Gheewala Shabbir, Green Rosemary, Joy Edward, Rojroongwasinkul Nipa, Thaiprasert Nalitra, Smith Richard International trade, dietary change, and cardiovascular disease health outcomes: Import tariff reform using an integrated macroeconomic, environmental and health modelling framework for Thailand. SSM-POPULATION HEALTH. 2019;9():.

4477. Holm Janni, Brixen Kim, Andries Alin, Horder Kirsten, Stoving Rene Reflections on involuntary treatment in the prevention of fatal anorexia nervosa: A review of five cases. INTERNATIONAL JOURNAL OF EATING DISORDERS. 2012;45(1):93-100.

4478. Yue Y., Hur J., Cao Y., Tabung F., Wang M., Wu K., Song M., Zhang X., Liu Y., Meyerhardt J., Ng K., Smith-Warner S., Willett W., Giovannucci E. Prospective evaluation of dietary and lifestyle pattern indices with risk of colorectal cancer in a cohort of younger women. ANNALS OF ONCOLOGY. 2021;32(6):778-786.

4479. Mulchandani Megha, Shetty Namrata, Conrad Agatha, Muir Petra, Mah Beth Treatment of eating disorders in older people: a systematic review. SYSTEMATIC REVIEWS. 2021;10(1):.

4480. Becker Carolyn, Smith Lisa, Ciao Anna Peer-facilitated eating disorder prevention: A randomized effectiveness trial of cognitive dissonance and media advocacy. JOURNAL OF COUNSELING PSYCHOLOGY. 2006;53(4):550-555.

4481. Talbot Catherine, Gavin Jeffrey, Steen Tommy, Morey Yvette A content analysis of thinspiration, fitspiration, and bonespiration imagery on social media. JOURNAL OF EATING DISORDERS. 2017;5():.

4482. Repke Hannah, Gulley Lauren, Rice Alexander, Gallagher-Teske Julia, Markos Bethelhem, Sanchez Natalia, Bristol Madison, Haynes Hannah, Lavender Jason, Neyland Mary, Shank Lisa, Emerick Jill, Gutierrez-Colina Ana, Arnold Thomas, Thomas Victoria, Haigney Mark, Shomaker Lauren, Tanofsky-Kraff Marian Addressing Anxiety and Stress for Healthier Eating in Teens (ASSET): A Pilot Randomized Controlled Trial Protocol for Reducing Anxiety, Disinhibited Eating, Excess Weight Gain, and Cardiometabolic Risk in Adolescent Girls. NUTRIENTS. 2022;14(20):.

4483. Li Rong, Zong Zhi-Ying, Gu Xi-Xi, Wang Dan-Ni, Dong Chen, Sun Chi, Zhao Rui, Gu Zhi-Feng, Gao Jian-Lin Higher dietary diversity as a protective factor against depression among older adults in China: a cross-sectional study. ANNALS OF PALLIATIVE MEDICINE.

2022;11(4):1278+.

4484. Sousa Janekeyla, Lima Laurineide, Sousa Fernandes Catiane, Santos Gleyson Physical activity and eating habits of adolescent students: national school health survey (PeNSE), 2015. RBNE-REVISTA BRASILEIRA DE NUTRICAO ESPORTIVA. 2019;13(77):87-93.

4485. Pinhas Leora, Nicholls Dasha, Crosby Ross, Morris Anne, Lynn Richard, Madden Sloane Classification of childhood onset eating disorders: A latent class analysis. INTERNATIONAL JOURNAL OF EATING DISORDERS. 2017;50(6):657-664.

4486. Yannakoulia M, Sitara M, Matalas AL Reported eating behavior and attitudes improvement after a nutrition intervention program in a group of young female dancers. INTERNATIONAL JOURNAL OF SPORT NUTRITION AND EXERCISE METABOLISM. 2002;12(1):24-32.

4487. Masdor Noor, Nawi Azmawati, Hod Rozita, Wong Zhiqin, Makpol Suzana, Chin Siok-Fong The Link between Food Environment and Colorectal Cancer: A Systematic Review. NUTRIENTS. 2022;14(19):.

4488. Melin Anna, Torstveit Monica, Burke Louise, Marks Saul, Sundgot-Borgen Jorunn Disordered Eating and Eating Disorders in Aquatic Sports. INTERNATIONAL JOURNAL OF SPORT NUTRITION AND EXERCISE METABOLISM. 2014;24(4):450-459.

4489. Volker Ulrike, Jacobi Corinna, Taylor C. Adaptation and evaluation of an internet-based prevention program for eating disorders in a sample of women with subclinical eating disorder syndromes. INTERNATIONAL JOURNAL OF PSYCHOLOGY. 2008;43(3-4):419.

4490. TOBIN DL TREATMENT OF EARLY TRAUMA AND DISSOCIATION IN EATING DISORDERS OF LATE-ONSET. EUROPEAN EATING DISORDERS REVIEW. 1995;3(3):160-173.

4491. Linardon Jake, Shatte Adrian, Messer Mariel, Firth Joseph, Fuller-Tyszkiewicz Matthew E-Mental Health Interventions for the Treatment and Prevention of Eating Disorders: An Updated Systematic Review and Meta-Analysis. JOURNAL OF CONSULTING AND CLINICAL PSYCHOLOGY. 2020;88(11):994-1007.

4492. Rohde Paul, Auslander Beth, Shaw Heather, Raineri Kate, Gau Jeff, Stice Eric Dissonance-based Prevention of Eating Disorder Risk Factors in Middle School Girls: Results from Two Pilot Trials. INTERNATIONAL JOURNAL OF EATING DISORDERS. 2014;47(5):483-494.

4493. Baranowski MJ, Hetherington MM Testing the efficacy of an eating disorder prevention program. INTERNATIONAL JOURNAL OF EATING DISORDERS. 2001;29(2):119-124.

4494. Rohrbach Pieter, Dingemans Alexandra, Furth Eric, Spinhoven Philip, Ginkel Joost, Bauer Stephanie, Akker-Van Marle M. Cost-effectiveness of three internet-based interventions for eating disorders: A randomized controlled trial. INTERNATIONAL JOURNAL OF EATING DISORDERS. 2022;55(8):1143-1155.

4495. Swenne Ingemar Poor Catch-up Growth in Late Adolescent Boys with Eating Disorders, Weight Loss and Stunting of Growth. EUROPEAN EATING DISORDERS REVIEW. 2013;21(5):395-398.

4496. Goukens Caroline, Klesse Anne Internal and external forces that prevent (vs. Facilitate) healthy eating: Review and outlook within consumer Psychology. CURRENT OPINION IN PSYCHOLOGY. 2022;46():.

4497. Favaro Angela, Tenconi Elena, Bosello Romina, Degortes Daniela, Santonastaso Paolo Perinatal complications in unaffected sisters of anorexia nervosa patients: testing a covariation model between genetic and environmental factors. EUROPEAN ARCHIVES OF PSYCHIATRY AND CLINICAL NEUROSCIENCE. 2011;261(6):391-396.

4498. Troncone Alda, Cascella Crescenzo, Chianese Antonietta, Galiero Ilaria, Zanfardino Angela, Confetto Santino, Perrone Laura, Iafusco Dario Changes in body image and onset of disordered eating behaviors in youth with type 1 diabetes over a five-year longitudinal follow-up. JOURNAL OF PSYCHOSOMATIC RESEARCH. 2018;109():44-50.

4499. Braet C, Van Winckel M Long-term follow-up of a cognitive behavioral treatment program for obese children. BEHAVIOR THERAPY. 2000;31(1):55-74.

4500. Sullivan PF, Bulik CM, Carter FA, Joyce PR Correlates of severity in bulimia nervosa. INTERNATIONAL JOURNAL OF EATING DISORDERS. 1996;20(3):239-251.

4501. Ceravolo Roberto, Frosini Daniela, Rossi Carlo, Bonuccelli Ubaldo Spectrum of addictions in Parkinson's disease: from dopamine dysregulation syndrome to impulse control disorders. JOURNAL OF NEUROLOGY. 2010;257(2):S276-S283.

4502. Jones Steve, Raykos Bronwyn, McEvoy Peter, Ieraci Jonica, Fursland Anthea, Byrne Susan, Waller Glenn The Development and Validation of a Measure of Eating Disorder-Specific Interpersonal Problems: The Interpersonal Relationships in Eating Disorders (IR-ED) Scale. PSYCHOLOGICAL ASSESSMENT. 2019;31(3):389-403.

4503. Rocco PL, Ciano RP, Balestrieri M Psychoeducation in the prevention of eating disorders: An experimental approach in adolescent schoolgirls. BRITISH JOURNAL OF MEDICAL PSYCHOLOGY. 2001;74(3):351-358.

4504. Nielsen S, Moller-Madsen S, Isager T, Jorgensen J, Pagsberg K, Theander S Standardized mortality in eating disorders - A quantitative summary of previously published and new evidence. JOURNAL OF PSYCHOSOMATIC RESEARCH. 1998;44(3-4):413-434.

4505. Liechty Janet Body Image Distortion and Three Types of Weight Loss Behaviors Among Nonoverweight Girls in the United States. JOURNAL OF ADOLESCENT HEALTH. 2010;47(2):176-182.

4506. He Jinbo, Huang Fang, Yan Jinjin, Wu Wen, Cai Zhihui, Fan Xitao Prevalence, demographic correlates, and association with psychological distress of night eating syndrome among Chinese college students. PSYCHOLOGY HEALTH \& MEDICINE. 2018;23(5):578-584.

4507. Potocka Adrianna, Moscicka Agnieszka OCCUPATIONAL STRESS, COPING STYLES AND EATING HABITS AMONG POLISH EMPLOYEES. MEDYCYNA PRACY. 2011;62(4):377-388.

4508. Vivarini Prudence, Jenkins Zoe, Castle David, Gwee Karen Borderline personality disorder symptoms in individuals with eating disorder: Association with severity, psychological distress, and psychosocial function. PERSONALITY AND MENTAL HEALTH. 2023;17(2):109-116.

4509. Shan Hongyun, Li Fei, Zhang Jun, Wang Hui, Li Jiong Feeding and Eating Disorder and Risk of Subsequent Neurodevelopmental Disorders: A Population-Based Cohort Study.

FRONTIERS IN PEDIATRICS. 2021;9():.

4510. Srithiphaphirom Phinyaphat, Robertson R. Rapid cold hardening delays the onset of anoxia-induced coma via an octopaminergic pathway in Locusta migratoria. JOURNAL OF INSECT PHYSIOLOGY. 2022;137():.

4511. Derricks Veronica, Earl Allison, Carmichael Alicia, Jayaratne Toby Psychological Pathways Through Which Social Norms and Social Identity Influence Eating Behavior: Testing a Conceptual Model. INTERNATIONAL JOURNAL OF BEHAVIORAL MEDICINE. 2023;30(1):7-18.

4512. Tith Rasmi, Paradis Gilles, Potter Brian, Low Nancy, Healy-Profitos Jessica, He Siyi, Auger Nathalie Association of Bulimia Nervosa With Long-term Risk of Cardiovascular Disease and Mortality Among Women. JAMA PSYCHIATRY. 2020;77(1):44-51.

4513. Chareonrungrueangchai Kridsada, Wongkawinwoot Keerati, Anothaisintawee Thunyarat, Reutrakul Sirimon Dietary Factors and Risks of Cardiovascular Diseases: An Umbrella Review. NUTRIENTS. 2020;12(4):.

4514. Hartmann Andrea, Greenberg Jennifer, Wilhelm Sabine The relationship between anorexia nervosa and body dysmorphic disorder. CLINICAL PSYCHOLOGY REVIEW. 2013;33(5):675-685.

4515. Dustira Anna, Wignall Paul, Joachimski Michael, Blomeier Dierk, Hartkopf-Froeder Christoph, Bond David Gradual onset of anoxia across the Permian-Triassic Boundary in Svalbard, Norway. PALAEOGEOGRAPHY PALAEOCLIMATOLOGY PALAEOECOLOGY. 2013;374():303-313.

4516. Leonard T, Mirabel-Sarron C, Foulon C, Melchior JC, Rigaud D, Apfelbaum M, Samuel-Lajeunesse B A short-term cognitive behavioural therapy for bulimia nervosa including brief hospitalisation. EUROPEAN PSYCHIATRY. 1997;12(8):405-411.

4517. Waesserle Ulrike, Ermer Uwe, Habisch Barbara, Seeliger Stephan Anorexia nervosa: does the incidence increase during the coronavirus pandemic?. PADIATRIE UND PADOLOGIE. 2022;57(5):247-253.

4518. Mahmoud Sara, El Moshy Sara, Rady Dina, Radwan Israa, Abbass Marwa, Al Jawaldeh Ayoub The effect of unhealthy dietary habits on the incidence of dental caries and overweight/obesity among Egyptian school children (A cross-sectional study). FRONTIERS IN PUBLIC HEALTH. 2022;10():.

4519. Loddo Giuseppe, Zanardi Marilena, Caletti Maria, Mignani Francesco, Petroni Maria, Chiaro Giacomo, Marchesini Giulio, Provini Federica Searching food during the night: the role of video-polysomnography in the characterization of the night eating syndrome. SLEEP MEDICINE. 2019;64():85-91.

4520. Shaw Heather, Stice Eric, Becker Carolyn Preventing Eating Disorders. CHILD AND ADOLESCENT PSYCHIATRIC CLINICS OF NORTH AMERICA. 2009;18(1):199+.

4521. SMOLAK L, LEVINE MP, GRALEN S THE IMPACT OF PUBERTY AND DATING ON EATING PROBLEMS AMONG MIDDLE SCHOOL GIRLS. JOURNAL OF YOUTH AND ADOLESCENCE. 1993;22(4):355-368.

4522. Tavolacci Marie, Ladner Joel, Grigioni Sebastien, Richard Laure, Villet Herve, Dechelotte Pierre Prevalence and association of perceived stress, substance use and behavioral addictions: a cross-sectional study among university students in France, 2009-2011. BMC PUBLIC HEALTH. 2013;13():.

4523. Francisco Rita, Narciso Isabel, Alarcao Madalena Parental Influences on Elite Aesthetic Athletes' Body Image Dissatisfaction and Disordered Eating. JOURNAL OF CHILD AND FAMILY STUDIES. 2013;22(8):1082-1091.

4524. Gulhan Pinar, Ataoglu Ozlem, Balbay Ege, YAsli Nevra, Annakkaya Ali The Evaluation of Eating Attitudes in Patients with Sarcoidosis. KONURALP TIP DERGISI. 2020;12(1):112-117.

4525. Brown Kayla, Farmer Cristan, Freeman G., Spartz Ellen, Farhadian Bahare, Thienemann Margo, Frankovich Jennifer Effect of Early and Prophylactic Nonsteroidal Anti-Inflammatory Drugs on Flare Duration in Pediatric Acute-Onset Neuropsychiatric Syndrome: An Observational Study of Patients Followed by an Academic Community-Based Pediatric Acute-Onset Neuropsychiatric Syndrome Clinic. JOURNAL OF CHILD AND ADOLESCENT PSYCHOPHARMACOLOGY. 2017;27(7, SI):619-628.

4526. Balzafiore Danielle, Rasgon Natalie, Yuen Laura, Shah Saloni, Kim Hyun, Goffin Kathryn, Miller Shefali, Wang Po, Ketter Terence Lifetime eating disorder comorbidity associated with delayed depressive recovery in bipolar disorder. INTERNATIONAL JOURNAL OF BIPOLAR DISORDERS. 2017;5():.

4527. Waesserle Ulrike, Ermer Uwe, Habisch Barbara, Seeliger Stephan Anorexia nervosa: does the incidence increase during the coronavirus pandemic?. MONATSSCHRIFT KINDERHEILKUNDE. 2022;170(5, SI):430-434.

4528. Tuncer Gulsum, Duman Zekiye Emotional eating experiences of individuals with severe mental disorders: A qualitative study. PERSPECTIVES IN PSYCHIATRIC CARE. 2022;58(4):2723-2732.

4529. Chang Yevvon, Wu Pai-Lu, Chiou Wen-Bin Thoughts of social distancing experiences affect food intake and hypothetical binge eating: Implications for people in home quarantine during COVID-19. SOCIAL SCIENCE \& MEDICINE. 2021;284():.

4530. Chu Jonathan, Ganson Kyle, Vittinghoff Eric, Mitchison Deborah, Hay Phillipa, Tabler Jennifer, Rodgers Rachel, Murray Stuart, Nagata Jason Weight Goals, Disordered Eating Behaviors, and BMI Trajectories in US Young Adults. JOURNAL OF GENERAL INTERNAL MEDICINE. 2021;36(9):2622-2630.

4531. Chote Brittany, McKelvie-Sebileau Pippa, Swinburn Boyd, Tipene-Leach David, D'Souza Erica Culture of Healthy Eating and Food Environments, Policies, and Practices in Regional New Zealand Schools. INTERNATIONAL JOURNAL OF ENVIRONMENTAL RESEARCH AND PUBLIC HEALTH. 2022;19(11):.

4532. Aljulifi Mohammed Prevalence and reasons of increased type 2 diabetes in Gulf Cooperation Council Countries. SAUDI MEDICAL JOURNAL. 2021;42(5):481-490.

4533. Schneider N, Mouithys-Mickalad AL, Lejeune JP, Deby-Dupont GP, Hoebeke M, Serteyn DA Synoviocytes, not chondrocytes, release free radicals after cycles of anoxia/re-oxygenation. BIOCHEMICAL AND BIOPHYSICAL RESEARCH COMMUNICATIONS. 2005;334(2):669-673.

4534. Zapata F Damaris, Granfeldt M Gislaine, Munoz R Sara, Celis B Magdalena, Vicente P Benjamin, Saez C Katia, Peterman R Fanny, Gaete R Daniel, Leonario R Marcell, Mosso C Constanza Risk of eating disorders in Chilean adolescents of different types educational

establishments. ARCHIVOS LATINOAMERICANOS DE NUTRICION. 2018;68(3):217-223.

4535. FOX GG, MCCALLAN NR, RATCLIFFE RG MANIPULATING CYTOPLASMIC PH UNDER ANOXIA - A CRITICAL TEST OF THE ROLE OF PH IN THE SWITCH FROM AEROBIC TO ANAEROBIC METABOLISM. PLANTA. 1995;195(3):324-330.

4536. Basterfield Annie, Dimitropoulos Gina, Bills Donna, Cullen Olivia, Freeman Victoria ``I would love to have online support but I don't trust it{''}: Positive and negative views of technology from the perspective of those with eating disorders in Canada. HEALTH \& SOCIAL CARE IN THE COMMUNITY. 2018;26(4):604-612.

4537. Zhang Li, Liu Yuan, Sun Ying, Zhang Xin Combined Physical Exercise and Diet: Regulation of Gut Microbiota to Prevent and Treat of Metabolic Disease: A Review. NUTRIENTS. 2022;14(22):.

4538. Low KG, Charanasomboon S, Brown C, Hiltunen G, Long K, Reinhalter K, Jones H Internalization of the thin ideal, weight and body image concerns. SOCIAL BEHAVIOR AND PERSONALITY. 2003;31(1):81-89.

4539. Tuiten A, Laan E, Panhuysen G, Everaerd W, DeHaan E, Koppeschaar H, Vroon P Discrepancies between genital responses and subjective sexual function during testosterone substitution in women with hypothalamic amenorrhea. PSYCHOSOMATIC MEDICINE. 1996;58(3):234-241.

4540. Bijlenga Denise, Heijden Kristiaan, Breuk Minda, Someren Eus, Lie Maria, Boonstra A., Swaab Hanna, Kooij J. Associations Between Sleep Characteristics, Seasonal Depressive Symptoms, Lifestyle, and ADHD Symptoms in Adults. JOURNAL OF ATTENTION DISORDERS. 2013;17(3):261-275.

4541. Mohoric Tamara, Pokrajac-Bulian Alessandra, Anic Petra, Kukic Miljana, Mohovic Patrizia Emotion regulation, perfectionism, and eating disorder symptoms in adolescents: the mediating role of cognitive eating patterns. CURRENT PSYCHOLOGY. 2022;():.

4542. Wang Lixin, Xue Yan, Ma Hao, Shi Haiyan, Wang Ling, Cui Xiaozheng Prazosin protects myocardial cells against anoxia-reoxygenation injury via the extracellular signal-regulated kinase signaling pathway. MOLECULAR MEDICINE REPORTS. 2018;17(2):2145-2152.

4543. Leon-Vazquez Concepcion, Villalobos-Hernandez Aremis, Alberto Rivera-Marquez Jose, Unikel-Santoncini Claudia Effect of parental criticism on disordered eating behaviors in male and female university students in Mexico City. EATING AND WEIGHT DISORDERS-STUDIES ON ANOREXIA BULIMIA AND OBESITY. 2019;24(5):853-860.

4544. Gotovac Sandra, LaMarre Andrea, Lafreniere Kathryn Words with weight: The construction of obesity in eating disorders research. HEALTH. 2020;24(2):113-131.

4545. Vitale Elsa, Galatola Vito, Mea Rocco, Di Dio Francesca, Canonico Anna The Linkage ``Body Mass Index-Insomnia Levels-Eating Disorder Flexibility{''} in Italian Nurses During the Covid-19 Outbreak: A Psychoendocrinological Employment Disease. ENDOCRINE METABOLIC \& IMMUNE DISORDERS-DRUG TARGETS. 2022;22(5):490-501.

4546. Stice Eric, Rohde Paul, Gau Jeff, Shaw Heather An Effectiveness Trial of a Dissonance-Based Eating Disorder Prevention Program for High-Risk Adolescent Girls. JOURNAL OF CONSULTING AND CLINICAL PSYCHOLOGY. 2009;77(5):825-834.

4547. Pitsavos CE, Toutouzas PK Cardiovascular risk factor profile in Greece: Results from the CARD102000 and ATTICA epidemiological studies. CURRENT MEDICAL RESEARCH AND OPINION. 2002;18(5):277-283.

4548. Stice E, Orjada K, Tristan J Trial of a psychoeducational eating disturbance intervention for college women: A replication and extension. INTERNATIONAL JOURNAL OF EATING DISORDERS. 2006;39(3):233-239.

4549. Bahadoor Rubaab, Alexandre Jean-Marc, Fournet Lucie, Gelle Thibaut, Serre Fuschia, Auriacombe Marc Inventory and Analysis of Controlled Trials of Mobile Phone Applications Targeting Substance Use Disorders: A Systematic Review. FRONTIERS IN PSYCHIATRY. 2021;12():.

4550. Martin-Piedra Laura, Alcala-Diaz Juan, Gutierrez-Mariscal Francisco, Larriva Antonio, Romero-Cabrera Juan, Torres-Pena Jose, Caballero-Villarraso Javier, Luque Raul, Perez-Martinez Pablo, Lopez-Miranda Jose, Delgado-Lista Javier Evolution of Metabolic Phenotypes of Obesity in Coronary Patients after 5 Years of Dietary Intervention: From the CORDIOPREV Study. NUTRIENTS. 2021;13(11):.

4551. Muniz Ribas Baraq EFFECTIVENESS OF COGNITIVE BEHAVIORAL THERAPY IN THE TREATMENT OF ANOREXIA NERVOSA. REVISTA ROL DE ENFERMERIA. 2019;42(3):56-67.

4552. Lebherz Corinna, Lehrke Michael Prevention of cardiovascular disease in patients with type 2 diabetes. DEUTSCHE MEDIZINISCHE WOCHENSCHRIFT. 2015;140(9):645-U16.

4553. Mayer-Brown Sarah, Lawless Casey, Fedele David, Dumont-Driscoll Marilyn, Janicke David The effects of media, self-esteem, and BMI on youth's unhealthy weight control behaviors. EATING BEHAVIORS. 2016;21():59-65.

4554. Christian Caroline, Brosof Leigh, Vanzhula Irina, Williams Brenna, Ram Shruti, Levinson Cheri Implementation of a dissonance-based, eating disorder prevention program in Southern, all-female high schools. BODY IMAGE. 2019;30():26-34.

4555. Dixon MH, Hill SA, Jackson MB, Ratcliffe RG, Sweetlove LJ Physiological and metabolic adaptations of Potamogeton pectinatus L. tubers support rapid elongation of stem tissue in the absence of oxygen. PLANT AND CELL PHYSIOLOGY. 2006;47(1):128-140.

4556. Maffei Cesare, Cavicchioli Marco, Movalli Mariagrazia, Cavallaro Roberto, Fossati Andrea Dialectical Behavior Therapy Skills Training in Alcohol Dependence Treatment: Findings Based on an Open Trial. SUBSTANCE USE \& MISUSE. 2018;53(14):2368-2385.

4557. Zickgraf Hana, Elkins Anjeli Sensory sensitivity mediates the relationship between anxiety and picky eating in children/adolescents ages 8-17, and in college undergraduates: A replication and age-upward extension. APPETITE. 2018;128():333-339.

4558. Grunewald William, Fogelberg Sammi, Ferguson Walton, Hines Sarah, Fortenberry Bailey, Smith April Longitudinal relationships between specific domains of interoception and muscle dysmorphia symptoms. EATING BEHAVIORS. 2023;48():.

4559. Drevytska T., Gonchar E., Okhai I., Lynnyk O., Mankovska I., Klionsky D., Dosenko V. The protective effect of Hif3a RNA interference and HIF-prolyl hydroxylase inhibition on cardiomyocytes under anoxia-reoxygenation. LIFE SCIENCES. 2018;202():131-139.

4560. Robinson Isabella, Stoyel Hannah, Robinson Paul ``If she had broken her leg she would not have waited in agony for 9 months{''}: Caregiver's experiences of eating disorder

treatment. EUROPEAN EATING DISORDERS REVIEW. 2020;28(6):750-765.

4561. Pearson Carolyn, Guller Leila, McPherson Laura, Lejuez Carl, Smith Gregory Validation of an existing measure of eating disorder risk for use with early adolescents. EATING BEHAVIORS. 2013;14(2):113-118.

4562. Waaddegaard Mette, Davidsen Michael, Kjoller Mette Obesity and prevalence of risk behaviour for eating disorders among young Danish women. SCANDINAVIAN JOURNAL OF PUBLIC HEALTH. 2009;37(7):736-743.

4563. Ranby Krista, Aiken Leona, MacKinnon David, Elliot Diane, Moe Esther, McGinnis Wendy, Goldberg Linn A Mediation Analysis of the ATHENA Intervention for Female Athletes: Prevention of Athletic-Enhancing Substance Use and Unhealthy Weight Loss Behaviors. JOURNAL OF PEDIATRIC PSYCHOLOGY. 2009;34(10):1069-1083.

4564. Rodgers Rachel, Sonneville Kendrin Research for leveraging food policy in universal eating disorder prevention. INTERNATIONAL JOURNAL OF EATING DISORDERS. 2018;51(6):503-506.

4565. Manduchi K., Presti G., Miselli G., Rabitti E. BIAAQ and eating disorder prevention. PSYCHOLOGY \& HEALTH. 2009;24(1, SI):256.

4566. Prnjak Katarina, Hay Phillipa, Mond Jonathan, Bussey Kay, Trompeter Nora, Lonergan Alexandra, Mitchison Deborah The Distinct Role of Body Image Aspects in Predicting Eating Disorder Onset in Adolescents After One Year. JOURNAL OF ABNORMAL PSYCHOLOGY. 2021;130(3):236-247.

4567. Wang Monica, Walls Courtney, Peterson Karen, Richmond Tracy, Spadano-Gasbarro Jennifer, Greaney Mary, Blood Emily, Mezgebu Solomon, McCormick Marie, Subramanian S., Austin S. Dietary and Physical Activity Factors Related to Eating Disorder Symptoms Among Middle School Youth. JOURNAL OF SCHOOL HEALTH. 2013;83(1):14-20.

4568. D'Souza CM, Forman SF, Austin SB Follow-up evaluation of a high school eating disorders screening program: knowledge, awareness and self-referral. JOURNAL OF ADOLESCENT HEALTH. 2005;36(3):208-213.

4569. Ho Li-Ju, Sheu Wayne, Lo Su-Huey, Yeh Yen-Po, Hwu Chii-Min, Huang Chien-Ning, Hsieh Chang-Hsun, Kuo Feng-Chih Unhealthy lifestyle associated with increased risk of macro- and micro-vascular comorbidities in patients with long-duration type 2 diabetes: results from the Taiwan Diabetes Registry. DIABETOLOGY \& METABOLIC SYNDROME. 2023;15(1):.

4570. Reel Justine, SooHoo Sonya, Summerhays Julia, Gill Diane Age before beauty: an exploration of body image in African-American and Caucasian adult women. JOURNAL OF GENDER STUDIES. 2008;17(4):321-330.

4571. Guo Chunyu, Xue Yanni, Xia Zhengmei, Cui Yingying, Hu Jie, Huang Xuexue, Wan Yuhui, Fang Jun, Zhang Shichen Association Between the Patterns of Five Unhealthy Behaviors and Suicidal Behaviors Among Adolescents in Six Provinces of China. FRONTIERS IN PSYCHIATRY. 2022;13():.

4572. Skeer Margie, Ballard Erica Are Family Meals as Good for Youth as We Think They Are? A Review of the Literature on Family Meals as They Pertain to Adolescent Risk Prevention. JOURNAL OF YOUTH AND ADOLESCENCE. 2013;42(7):943-963.

4573. Romano Kelly, Lipson Sarah Weight Misperception and Thin-Ideal Overvaluation Relative to the Positive Functioning and Eating Disorder Pathology of Transgender and Nonbinary Young Adults. PSYCHOLOGY OF SEXUAL ORIENTATION AND GENDER DIVERSITY. 2022;9(4):446-453.

4574. Ghamri Ranya, Alahmari Asma, Alghamdi Lama, Barashid Mada, Alamoudi Sarah Prevalence and predictors of eating disorders:A cross-sectional survey of medical students at King Abdul-Aziz University, Jeddah. PAKISTAN JOURNAL OF MEDICAL SCIENCES. 2022;38(6):1633-1638.

4575. Rossi G., Balottin U., Rossi M., Chiappedi M., Fazzi E., Lanzi G. Pharmacological treatment of anorexia nervosa: A retrospective study in preadolescents and adolescents. CLINICAL PEDIATRICS. 2007;46(9):806-811.

4576. Austin SB, Field AE, Wiecha J, Peterson KE, Gortmaker SL The impact of a school-based obesity prevention trial on disordered weight-control behaviors in early adolescent girls. ARCHIVES OF PEDIATRICS \& ADOLESCENT MEDICINE. 2005;159(3):225-230.

4577. Pasinetti Giulio, Eberstein Jacqueline Metabolic syndrome and the role of dietary lifestyles in Alzheimer's disease. JOURNAL OF NEUROCHEMISTRY. 2008;106(4):1503-1514.

4578. Yaghoubpour Kamran, Tasdighi Erfan, Abdi Hengameh, Barzin Maryam, Mahdavi Maryam, Valizadeh Majid, Azizi Fereidoun, Hosseinpanah Farhad Association of obesity phenotypes in adolescents and incidence of early adulthood type 2 diabetes mellitus: Tehran lipid and glucose study. PEDIATRIC DIABETES. 2021;22(7):937-945.

4579. Neuman Nicklas, Eli Karin, Nowicka Paulina Childhood memories of food and eating in lower-income families in the United States: a qualitative study. BMC PUBLIC HEALTH. 2021;21(1):.

4580. Lay B, Schmidt MH Relapses in the course of anorexia nervosa. ZEITSCHRIFT FUR KINDER-UND JUGENDPSYCHIATRIE UND PSYCHOTHERAPIE. 1999;27(3):207-219.

4581. Gorrell Sasha, Trainor Claire, Le Grange Daniel The impact of urbanization on risk for eating disorders. CURRENT OPINION IN PSYCHIATRY. 2019;32(3):242-247.

4582. Teresa Alcantara-Garces Maria, Monserrat Rodriguez-Ramirez Alejandra, Cristina Garcia-Ulloa Ana, Hernandez-Jimenez Sergio Comorbidity Between Recent Diagnosis of Type 2 Diabetes and Non-Psychotic Psychiatric Disorders: Metabolic Characteristics and Clinical Correlates. NEUROPSYCHIATRIC DISEASE AND TREATMENT. 2022;18():1151-1163.

4583. Harmanci Hatice, Akdeniz Seher, Ahci Zeynep Prevalence of Eating Disorders: Its Relationship with Alexithymia and Mental Complaints. CYPRUS TURKISH JOURNAL OF PSYCHIATRY AND PSYCHOLOGY. 2021;3(1):30-36.

4584. Herzberg Philipp, Wildfang Swetlana Eating disorder symptoms and personality: Implications for clinical assessment from a network perspective. ZEITSCHRIFT FUR PSYCHIATRIE PSYCHOLOGIE UND PSYCHOTHERAPIE. 2018;66(3, SI):187-194.

4585. Preti Antonio, Tondo Leonardo, Sisti Davide, Rocchi Marco, Girolamo Giovanni, Grp PROGRES Correlates and antecedents of hospital admission for attempted suicide: a nationwide survey in Italy. EUROPEAN ARCHIVES OF PSYCHIATRY AND CLINICAL NEUROSCIENCE. 2010;260(3):181-190.

4586. Grasby Stephen, Beauchamp Benoit Latest Permian to Early Triassic basin-to-shelf anoxia in the Sverdrup Basin, Arctic Canada. CHEMICAL GEOLOGY. 2009;264(1-4):232-246.

4587. Beraudi Alina, Bruno Valeria, Battaglia Giuseppe, Biagioni Francesca, Rampello Liborio, Nicoletti Ferdinando, Poli Alessandro Pharmacological activation of mGlu2/3 metabotropic glutamate receptors protects retinal neurons against anoxic damage in the goldfish Carassius auratus. EXPERIMENTAL EYE RESEARCH. 2007;84(3):544-552.

4588. Cuijpers Pim, Donker Tara, Weissman Myrna, Ravitz Paula, Cristea Ioana Interpersonal Psychotherapy for Mental Health Problems: A Comprehensive Meta-Analysis. AMERICAN JOURNAL OF PSYCHIATRY. 2016;173(7):680-687.

4589. Silvani Juliana, Schmidt Maria, Zajdenverg Lenita, Galliano Leony, Antunes Nunes Maria Impact of binge eating during pregnancy on gestational weight gain and postpartum weight retention among women with gestational diabetes mellitus: LINDA-Brasil. INTERNATIONAL JOURNAL OF EATING DISORDERS. 2020;53(11):1818-1825.

4590. Schmidt-Hantke Juliane, Vollert Bianka, Hagner Franziska, Beintner Ina, Hutter Kristian, Nitsch Martina, Jacobi Corinna, Waldherr Karin Stakeholders' perspectives on online interventions to improve mental health in eating disorder patients and carers in Germany. EUROPEAN JOURNAL OF PUBLIC HEALTH. 2021;31(1):80-87.

4591. Weafer Jessica, Crane Natania, Gorka Stephanie, Phan K., Wit Harriet Neural correlates of inhibition and reward are negatively associated. NEUROIMAGE. 2019;196():188-194.

4592. Stice Eric, Rohde Paul, Gau Jeff, Shaw Heather Implementation Factors That Predict Larger Effects From a Peer Educator Delivered Eating Disorder Prevention Program at Universities. JOURNAL OF CONSULTING AND CLINICAL PSYCHOLOGY. 2023;():.

4593. Guerro-Prado D, Romero JMB, Moreno AC The epidemiology of eating disorders and the influence of mass media: a literature review. ACTAS ESPANOLAS DE PSIQUIATRIA. 2001;29(6):403-410.

4594. Raabe K. Girl-specific prevention of Eating Disorder. PSYCHOTHERAPEUT. 2008;53(1):71-72.

4595. Lieb R, Zimmermann P, Friis RH, Hofler M, Tholen S, Wittchen HU The natural course of DSM-IV somatoform disorders and syndromes among adolescents and young adults: a prospective-longitudinal community study. EUROPEAN PSYCHIATRY. 2002;17(6):321-331.

4596. Kusuma Dian, Kusumawardani Nunik, Ahsan Abdillah, Sebayang Susy, Amir Vilda, Ng Nawi On the verge of a chronic disease epidemic: comprehensive policies and actions are needed in Indonesia. INTERNATIONAL HEALTH. 2019;11(6):422-424.

4597. Maganto Carmen, Garaigordobil Maite, Kortabarria Lorea Eating Problems in Adolescents and Youths: Explanatory Variables. SPANISH JOURNAL OF PSYCHOLOGY. 2016;19():.

4598. Abiri Behnaz, Koohi Fatemeh, Ebadinejad Amir, Valizadeh Majid, Hosseinpanah Farhad Transition from metabolically healthy to unhealthy overweight/obesity and risk of cardiovascular disease incidence: A systematic review and meta-analysis. NUTRITION METABOLISM AND CARDIOVASCULAR DISEASES. 2022;32(9):2041-2051.

4599. Tegethoff Marion, Stalujanis Esther, Belardi Angelo, Meinlschmidt Gunther Chronology of Onset of Mental Disorders and Physical Diseases in Mental-Physical Comorbidity - A National Representative Survey of Adolescents. PLOS ONE. 2016;11(10):.

4600. Feng Xiaoqi, Girosi Federico, Mcrae Ian People with multiple unhealthy lifestyles are less likely to consult primary healthcare. BMC FAMILY PRACTICE. 2014;15():.

4601. Astur Robert, Palmisano Alexandra, Hudd Ellie, Carew Andrew, Deaton Bonnie, Kuhney Franchesca, Niezrecki Rachel, Santos Melissa Pavlovian conditioning to food reward as a function of eating disorder risk. BEHAVIOURAL BRAIN RESEARCH. 2015;291():277-282.

4602. Voltas Nuria, Arija Victoria, Aparicio Estefania, Canals Josefa Longitudinal study of psychopathological, anthropometric and sociodemographic factors related to the level of Mediterranean diet adherence in a community sample of Spanish adolescents. PUBLIC HEALTH NUTRITION. 2016;19(10):1812-1822.

4603. Grossman Stephanie, Campagna Bianca, Brochu Hadley, Odermatt Meline, Annunziato Rachel Improving body image and sexual health behaviors among college women. JOURNAL OF AMERICAN COLLEGE HEALTH. 2018;66(8):826-830.

4604. Gonzalez Sandoval Claudia, Diaz Burke Yolanda, Patricia Mendizabal-Ruiz Adriana, Medina Diaz Eunice, Alejandro Morales Jose PREVALENCE OF OBESITY AND ALTERED LIPID PROFILE IN UNIVERSITY STUDENTS. NUTRICION HOSPITALARIA. 2014;29(2):315-321.

4605. Stice Eric, Rohde Paul, Gau Jeff, Butryn Meghan, Shaw Heather, Cloud Kasie, D'Adamo Laura Enhancing Efficacy of a Dissonance-Based Obesity and Eating Disorder Prevention Program: Experimental Therapeutics. JOURNAL OF CONSULTING AND CLINICAL PSYCHOLOGY. 2021;89(10):793-804.

4606. Kumar Amudha, Chidambaram Vignesh, Mehta Jawahar Vegetarianism, microbiota, and cardiovascular health: looking back, and forward. EUROPEAN JOURNAL OF PREVENTIVE CARDIOLOGY. 2022;29(14):1895-1910.

4607. Azorin Jean-Michel, Belzeaux Raoul, Kaladjian Arthur, Adida Marc, Hantouche Elie, Lancrenon Sylvie, Fakra Eric Risks associated with gender differences in bipolar I disorder. JOURNAL OF AFFECTIVE DISORDERS. 2013;151(3):1033-1040.

4608. Damour Lisa, Cordiano Tori, Anderson-Fye Eileen My Sister's Keeper: Identifying Eating Pathology Through Peer Networks. EATING DISORDERS. 2015;23(1):76-88.

4609. Walsh Erin, Jacka Felice, Butterworth Peter, Anstey Kaarin, Cherbuin Nicolas Midlife susceptibility to the effects of poor diet on diabetes risk. EUROPEAN JOURNAL OF CLINICAL NUTRITION. 2021;75(1):85-90.

4610. Stice Eric, Onipede Z., Shaw Heather, Rohde Paul, Gau Jeff Effectiveness of the Body Project Eating Disorder Prevention Program for Different Racial and Ethnic Groups and an Evaluation of the Potential Benefits of Ethnic Matching. JOURNAL OF CONSULTING AND CLINICAL PSYCHOLOGY. 2021;89(12):1007-1019.

4611. Nip Angel, Reboussin Beth, Dabelea Dana, Bellatorre Anna, Mayer-Davis Elizabeth, Kahkoska Anna, Lawrence Jean, Peterson Claire, Dolan Lawrence, Pihoker Catherine Disordered Eating Behaviors in Youth and Young Adults With Type 1 or Type 2 Diabetes Receiving Insulin Therapy: The SEARCH for Diabetes in Youth Study. DIABETES CARE. 2019;42(5):859-866.

4612. O'Hara Sarah, Smith Katherine Presentation of eating disorders in the news media: What are the implications for patient diagnosis and treatment?. PATIENT EDUCATION AND

COUNSELING. 2007;68(1):43-51.

4613. Muros Jose, avila-Alche Angela, Knox Emily, Zabala Mikel Likelihood of suffering from an eating disorder in a sample of Spanish cyclists and triathletes. JOURNAL OF EATING DISORDERS. 2020;8(1):.

4614. Wang Yoko, Shivappa Nitin, Hebert James, Page Amanda, Gill Tiffany, Melaku Yohannes Association between Dietary Inflammatory Index, Dietary Patterns, Plant-Based Dietary Index and the Risk of Obesity. NUTRIENTS. 2021;13(5):.

4615. Neumark-Sztainer Dianne, Friend Sarah, Flattum Colleen, Hannan Peter, Story Mary, Bauer Katherine, Feldman Shira, Petrich Christine New Moves-Preventing Weight-Related Problems in Adolescent Girls A Group-Randomized Study. AMERICAN JOURNAL OF PREVENTIVE MEDICINE. 2010;39(5):421-432.

4616. Thompson Katherine, Bardone-Cone Anna 2019-nCOV distress and depressive, anxiety and OCD-type, and eating disorder symptoms among postpartum and control women. ARCHIVES OF WOMENS MENTAL HEALTH. 2021;24(4):671-680.

4617. Raynal Patrick, Soccodato Marine, Fages Morgane, Sejourne Natalene A comparative study of orthorexia between premenopausal, perimenopausal, and postmenopausal women. EATING AND WEIGHT DISORDERS-STUDIES ON ANOREXIA BULIMIA AND OBESITY. 2022;27(7):2523-2531.

4618. Troncone Alda, Chianese Antonietta, Zanfardino Angela, Cascella Crescenzo, Piscopo Alessia, Borriello Anna, Rollato Serena, Casaburo Francesca, Testa Veronica, Iafusco Dario Disordered eating behaviors in youths with type 1 diabetes during COVID-19 lockdown: an exploratory study. JOURNAL OF EATING DISORDERS. 2020;8(1):.

4619. Amirzargar Nasibeh, Heidari-Soureshjani Saeid, Yang Qian, Abbaszadeh Saber, Khaksarian Mojtaba Neuroprotective Effects of Medicinal Plants in Cerebral Hypoxia and Anoxia: A Systematic Review. NATURAL PRODUCTS JOURNAL. 2020;10(5):550-565.

4620. BryantWaugh RJ, Cooper PJ, Taylor CL, Lask BD The use of the eating disorder examination with children: A pilot study. INTERNATIONAL JOURNAL OF EATING DISORDERS. 1996;19(4):391-397.

4621. Hoque Romy, Chesson Andrew Zolpidem-Induced Sleepwalking, Sleep Related Eating Disorder, and Sleep-Driving: Fluorine-18-Flourodeoxyglucose Positron Emission Tomograph Analysis, and a Literature Review of Other Unexpected Clinical Effects Zolpidem. JOURNAL OF CLINICAL SLEEP MEDICINE. 2009;5(5):471-476.

4622. Giel Katrin, Zipfel Stephan, Schweizer Roland, Braun Regina, Ranke Michael, Binder Gerhard, Ehehalt Stefan, Grp DISKUS Eating Disorder Pathology in Adolescents Participating in a Lifestyle Intervention for Obesity: Associations with Weight Change, General Psychopathology and Health-Related Quality of Life. OBESITY FACTS. 2013;6(4):307-316.

4623. Tamura Akira, Minami Koichi, Tsuda Yuko, Mizumoto Kazuhiro, Suzuki Hiroyuki Adolescent eating disorder with catheter-related bloodstream infection. PEDIATRICS INTERNATIONAL. 2021;63(6):678-684.

4624. Ravensbergen Eva, Waterlander Wilma, Kroeze Willemieke, Steenhuis Ingrid Healthy or Unhealthy on Sale? A cross-sectional study on the proportion of healthy and unhealthy foods promoted through flyer advertising by supermarkets in the Netherlands. BMC PUBLIC HEALTH. 2015;15():.

4625. Hawrysh Peter, Buck Leslie Anoxia-mediated calcium release through the mitochondrial permeability transition pore silences NMDA receptor currents in turtle neurons. JOURNAL OF EXPERIMENTAL BIOLOGY. 2013;216(23):4375-4387.

4626. Vaughan JL, King KA, Cottrell RR Collegiate athletic trainers' confidence in helping female athletes with eating disorders. JOURNAL OF ATHLETIC TRAINING. 2004;39(1):71-76.

4627. Pompeiano Antonio, Guglielminetti Lorenzo Carbohydrate metabolism in germinating caryopses of Oryza sativa L. exposed to prolonged anoxia. JOURNAL OF PLANT RESEARCH. 2016;129(5):833-840.

4628. Okada-Iwabu Miki, Iwabu Masato, Ueki Kohjiro, Yamauchi Toshimasa, Kadowaki Takashi Perspective of Small-Molecule AdipoR Agonist for Type 2 Diabetes and Short Life in Obesity. DIABETES \& METABOLISM JOURNAL. 2015;39(5):363-372.

4629. Eeden Annelies, Oldehinkel Albertine, Hoeken Daphne, Hoek Hans Risk factors in preadolescent boys and girls for the development of eating pathology in young adulthood.

INTERNATIONAL JOURNAL OF EATING DISORDERS. 2021;54(7):1147-1159.

4630. Reas Deborah, Ro Oyvind Time trends in healthcare-detected incidence of anorexia nervosa and bulimia nervosa in the Norwegian National Patient Register (2010-2016). INTERNATIONAL JOURNAL OF EATING DISORDERS. 2018;51(10):1144-1152.

4631. Harrison GG, Kagawa-Singer M, Foerster SB, Lee H, Kim LP, Nguyen TU, Fernandez-Ami A, Quinn V, Bal DG Seizing the moment - California's opportunity to prevent nutrition-related health disparities in low-income Asian American populations. CANCER. 2005;104(12, S):2962-2968.

4632. Ottevaere Charlene, Huybrechts Inge, Benser Jasmin, De Bourdeaudhuij Ilse, Cuenca-Garcia Magdalena, Dallongeville Jean, Zaccaria Maria, Gottrand Frederic, Kersting Mathilde, Rey-Lopez Juan, Manios Yannis, Molnar Denes, Moreno Luis, Smpokos Emmanouel, Widhalm Kurt, De Henauw Stefaan, Grp HELENA Clustering patterns of physical activity, sedentary and dietary behavior among European adolescents: The HELENA study. BMC PUBLIC HEALTH. 2011;11():.

4633. Barlow Sarah Expert committee recommendations regarding the prevention, assessment, and treatment of child and adolescent overweight and obesity: Summary report. PEDIATRICS. 2007;120(S):S164-S192.

4634. Saul Jennifer, Rodgers Rachel Adolescent Eating Disorder Risk and the Online World. CHILD AND ADOLESCENT PSYCHIATRIC CLINICS OF NORTH AMERICA. 2018;27(2):221+.

4635. Coleman Sophie, Caswell Noreen Diabetes and eating disorders: an exploration of `Diabulimia'. BMC PSYCHOLOGY. 2020;8(1):.

4636. Fabiano Valentina, Albani Elena, Cammi Giulia, Zuccotti Gian Nutrition in developmental age: few rules to stay healthy. MINERVA PEDIATRICA. 2020;72(3):182-195.

4637. Morais Celia, Bacurau Pinheiro Liana, Vieira Cunha Lima Severina, Lyra Clelia, Sena Evangelista Karine, Lima Kenio, Campos Pedrosa Lucia Dietary patterns of young adolescents in urban areas of Northeast Brazil. NUTRICION HOSPITALARIA. 2013;28(6):1977-1984.

4638. Bratland-Sanda Solfrid, Sundgot-Borgen Jorunn Eating disorders in athletes: Overview of prevalence, risk factors and recommendations for prevention and treatment. EUROPEAN JOURNAL OF SPORT SCIENCE. 2013;13(5):499-508.

4639. Ucar Sema, Dizdarer Ceyhun, Darcan Sukran, Korkmaz Serap, Ergudenler Yesim, Asilsoy Suna, Bilgili Gokmen, Ozcan Tugrul, Simsek Damla, Coker Mahmut Prevalence of obesity and overweight among children in Izmir, Turkey: effects of nutritional and socio-economic factors. OBESITY AND METABOLISM-MILAN. 2009;5(3-4):99-106.

4640. Hanachi Mouna, Dicembre Marika, Rives-Lange Claire, Ropers Jacques, Bemer Pauline, Zazzo Jean-Fabien, Poupon Joel, Dauvergne Agnes, Melchior Jean-Claude Micronutrients Deficiencies in 374 Severely Malnourished Anorexia Nervosa Inpatients. NUTRIENTS. 2019;11(4):.

4641. Yao Zhen, Xie Xiaoxia, Bai Ruoxue, Li Lan, Zhang Xu, Li Shaowei, Ma Yanna, Hui Zhenliang, Chen Jun The impact of eating behaviors during COVID-19 in health-care workers: A conditional process analysis of eating, affective disorders, and PTSD. HELIYON. 2022;8(10):.

4642. White Emily, Mooney Jan, Warren Cortney Ethnicity, eating pathology, drive for muscularity, and muscle dysmorphia in college men: a descriptive study. EATING DISORDERS. 2019;27(2, SI):137-151.

4643. Martinis Olgica, Coklo Miran, Aladrovic Jasna, Belavic Anja, Missoni Sasa ANTHROPOMETRIC MEASUREMENTS, DIETARY HABITS, SERUM LIPID AND GLUCOSE LEVELS IN RELATION TO HIGH BLOOD PRESSURE AMONG ADOLESCENT BOYS AND GIRLS IN CROATIA. ACTA CLINICA CROATICA. 2020;59(4):672-685.

4644. Fukutomi Akira, Connan Frances, Winston Anthony, Ghosh Pia Men in eating disorder units: a service evaluation survey regarding mixed gender accommodation rules in an eating disorder setting. BJPSYCH BULLETIN. 2018;42(6):258-263.

4645. Behar A Rosa, Manzo G Rodrigo, Casanova Z Dunny Lack of assertiveness in patients with eating disorders. REVISTA MEDICA DE CHILE. 2006;134(3):312-319.

4646. Schaumberg Katherine, Anderson Drew Dietary restraint and weight loss as risk factors for eating pathology. EATING BEHAVIORS. 2016;23():97-103.

4647. Priesterroth Lilli, Grammes Jennifer, Strohm Edda, Kubiak Thomas Disordered eating behaviours and eating disorders in adults with type 1 diabetes (DEBBI): rational and design of an observational longitudinal online study. BMJ OPEN. 2022;12(9):.

4648. Schoen Eva, Clougher Kelly, Wiese Joanna Developing an Eating Disorder Peer Advocate Program on Campus: A Report on the Eating Disorder Awareness and Advocacy Program (EDAAP). JOURNAL OF COLLEGE STUDENT PSYCHOTHERAPY. 2020;34(3):211-227.

4649. Ramon Munoz-Rodriguez Jose, Rodriguez-Cano Teresa, Polo Filomena, Saenz-Mateos Luis, Agarrado Andrea, Segura Esperanza, Casas Gloria, Martin-Fernandez Jesus, Beato-Fernandez Luis, Salas Elisabet, Gonzalez-Martin Carmen, Alguacil Luis The Neuroendocrine and Metabolic Outcomes of Bariatric Surgery Depend on Presurgical Control over Eating. NEUROENDOCRINOLOGY. 2020;110(1-2):63-69.

4650. Reiterer Melissa, Milton Sarah Induction of foxo3a protects turtle neurons against oxidative stress. COMPARATIVE BIOCHEMISTRY AND PHYSIOLOGY A-MOLECULAR \& INTEGRATIVE PHYSIOLOGY. 2020;243():.

4651. Bravini Elisabetta, Azzolina Danila, Janin Denise, Vercelli Stefano, Panella Massimiliano, Rinaldi Carmela Health-related lifestyles among Italian university students: A cross-sectional study. EPIDEMIOLOGIA \& PREVENZIONE. 2022;46(1-2):68-76.

4652. Siegfried Z, Berry EM, Hao SZ, Avraham Y Animal models in the investigation of anorexia. PHYSIOLOGY \& BEHAVIOR. 2003;79(1):39-45.

4653. Eisenhauer Christine, Brito Fabiana, Yoder Aaron, Kupzyk Kevin, Pullen Carol, Salinas Katherine, Miller Jessica, Hageman Patricia Mobile technology intervention for weight loss in rural men: protocol for a pilot pragmatic randomised controlled trial. BMJ OPEN. 2020;10(4):.

4654. Goodman Anna, Heshmati Amy, Koupil Ilona Family History of Education Predicts Eating Disorders across Multiple Generations among 2 Million Swedish Males and Females. PLOS ONE. 2014;9(8):.

4655. Michels N., Amenyah S. Body size ideals and dissatisfaction in Ghanaian adolescents: role of media, lifestyle and well-being. PUBLIC HEALTH. 2017;146():65-74.

4656. Forbush Kelsie, Hagan Kelsey, Kite Benjamin, Chapa Danielle, Bohrer Brittany, Gould Sara Understanding eating disorders within internalizing psychopathology: A novel transdiagnostic, hierarchical-dimensional model. COMPREHENSIVE PSYCHIATRY. 2017;79(SI):40-52.

4657. Amadou Amina, Torres-Mejia Gabriela, Hainaut Pierre, Romieu Isabelle Breast cancer in Latin America: global burden, patterns, and risk factors. SALUD PUBLICA DE MEXICO. 2014;56(5):547-554.

4658. Pamies Lidia, Quiles Yolanda Perfectionism and risk factors for the development of eating disorders in Spanish adolescents of both genders. ANALES DE PSICOLOGIA. 2014;30(2):620-626.

4659. Barral Carmen, Daigre Constanza, Bachiller Diana, Calvo Natalia, Ros-Cucurull Elena, Gancedo Beatriz, Grau-Lopez Lara, Ferrer Marc, Casas Miguel, Roncero Carlos Severity factors associated with borderline personality disorder among misusers in an outpatient sample in Spain. JOURNAL OF ADDICTIVE DISEASES. 2017;36(2):93-96.

4660. Pitol-Palin Leticia, Souza Batista Fabio, Silva Gomes-Ferreira Pedro, Mulinari-Santos Gabriel, Ervolino Edilson, Souza Francisley, Matsushita Doris, Okamoto Roberta Different Stages of Alveolar Bone Repair Process Are Compromised in the Type 2 Diabetes Condition: An Experimental Study in Rats. BIOLOGY-BASEL. 2020;9(12):.

4661. Conceicao Eva, Machado Paulo, Vaz Ana, Pinto-Bastos Ana, Ramalho Sofia, Silva Catia, Arrojado Filipa APOLO-Bari, an internet-based program for longitudinal support of bariatric surgery patients: study protocol for a randomized controlled trial. TRIALS. 2016;17():.

4662. Madigan C., Daley A., Kabir E., Aveyard P., Brown W. Cluster analysis of behavioural weight management strategies and associations with weight change in young women: a longitudinal analysis. INTERNATIONAL JOURNAL OF OBESITY. 2015;39(11):1601-1606.

4663. Koletzko Berthold, Fishbein Mark, Lee Way, Moreno Luis, Mouane Nezha, Mouzaki Marialena, Verduci Elvira Prevention of Childhood Obesity: A Position Paper of the Global Federation of International Societies of Paediatric Gastroenterology, Hepatology and Nutrition (FISPGHAN). JOURNAL OF PEDIATRIC GASTROENTEROLOGY AND NUTRITION. 2020;70(5):702-710.

4664. Chithambo Taona, Huey Stanley Internet-delivered eating disorder prevention: A randomized controlled trial of dissonance-based and cognitive-behavioral interventions. INTERNATIONAL JOURNAL OF EATING DISORDERS. 2017;50(10):1142-1151.

4665. Riggi Emilia, Baccini Michela, Camussi Elisa, Gallo Federica, Anatrone Caterina, Pezzana Andrea, Senore Carlo, Giordano Livia, Segnan Nereo Promoting healthy lifestyle habits among participants in cancer screening programs: Results of the randomized controlled Sti.Vi study. JOURNAL OF PUBLIC HEALTH RESEARCH. 2022;11(3):.

4666. Goncalves Sonia, Freitas Flavia, Freitas-Rosa Marta, Machado Barbara Dysfunctional eating behaviour, psychological well-being and adaptation to pregnancy: A study with women in the third trimester of pregnancy. JOURNAL OF HEALTH PSYCHOLOGY. 2015;20(5, SI):535-542.

4667. Riedel Bettina, Zuschin Martin, Stachowitsch Michael Tolerance of benthic macrofauna to hypoxia and anoxia in shallow coastal seas: a realistic scenario. MARINE ECOLOGY PROGRESS SERIES. 2012;458():39+.

4668. Linardon Jake, Susanto Luvena, Tepper Hannah, Fuller-Tyszkiewicz Matthew Self-compassion as a moderator of the relationships between shape and weight overvaluation and eating disorder psychopathology, psychosocial impairment, and psychological distress. BODY IMAGE. 2020;33():183-189.

4669. Kim Kye-Ha, Yang Kyoung-Mi The relationship between eating disorders and parent-adolescent communication in middle school students in rural areas. JOURNAL OF KOREAN ACADEMY OF NURSING. 2008;38(1):55-63.

4670. Papas Mia, Trabulsi Jillian, Axe Michelle, Rimmer James Predictors of Obesity in a US Sample of High School Adolescents With and Without Disabilities. JOURNAL OF SCHOOL HEALTH. 2016;86(11):803-812.

4671. Cavazos-Rehg Patricia, Min Caroline, Fitzsimmons-Craft Ellen, Savoy Bria, Kaiser Nina, Riordan Raven, Krauss Melissa, Costello Shaina, Wilfley Denise Parental consent: A potential barrier for underage teens' participation in an mHealth mental health intervention. INTERNET INTERVENTIONS-THE APPLICATION OF INFORMATION TECHNOLOGY IN MENTAL AND BEHAVIOURAL HEALTH. 2020;21():.

4672. Breithaupt Lauren, Eickman Laura, Byrne Catherine, Fischer Sarah Enhancing empowerment in eating disorder prevention: Another examination of the REbeL peer education model. EATING BEHAVIORS. 2017;25(SI):38-41.

4673. Ciorba Irina, Farcus Oana, Giger Roland, Nisa Lluis Facial self-mutilation: an analysis of published cases. POSTGRADUATE MEDICAL JOURNAL. 2014;90(1062):191-200.

4674. Russell Catherine, Taki Sarah, Azadi Leva, Campbell Karen, Laws Rachel, Elliott Rosalind, Denney-Wilson Elizabeth A qualitative study of the infant feeding beliefs and behaviours of mothers with low educational attainment. BMC PEDIATRICS. 2016;16():.

4675. San Giovanni Christine, Sweeney Brooke, Skelton Joseph, Kelsey Megan, Kelly Aaron Aversion to Off-label Prescribing in Clinical Pediatric Weight Management: The Quintessential Double Standard. JOURNAL OF CLINICAL ENDOCRINOLOGY \& METABOLISM. 2021;106(7):2103-2113.

4676. Nancy S., Rahman K., Kumar S., Sofia S., Robins M. Reasons and solutions for unhealthy food consumption and physical inactivity among school-going adolescents: A sequential mixed-methods study in Puducherry, South India. JOURNAL OF FAMILY MEDICINE AND PRIMARY CARE. 2022;11(11):6970-6977.

4677. Mont Meghan, Carlson C., Geisbuhler Timothy Resting Ca2+ influx does not contribute to anoxia-induced cell death in adult rat cardiac myocytes. CANADIAN JOURNAL OF PHYSIOLOGY AND PHARMACOLOGY. 2009;87(5):360-370.

4678. Hall Wendy Conference on `Obesity and the brain'. PROCEEDINGS OF THE NUTRITION SOCIETY. 2022;():.

4679. Basker Mona, Mathai Sarah, Korula Sophy, Mammen Priya Eating Disorders among Adolescents in a Tertiary Care Centre in India. INDIAN JOURNAL OF PEDIATRICS. 2013;80(3):211-214.

4680. Al Shanbari Nasser, Alharthi Abdulrahman, Bakry Salah, Alsalmi Safaa, Saleh Raghad, Kambiji Gufran, Saleh Jomanah, Alsulami Ethar, Shatla Mokhtar Screening Eating Disorders Among Female High School Students in Makkah City: A Cross- Sectional Survey. CUREUS JOURNAL OF MEDICAL SCIENCE. 2023;15(2):.

4681. Mensinger Janell, Cox Shelbi, Henretty Jennifer Treatment Outcomes and Trajectories of Change in Patients Attributing Their Eating Disorder Onset to Anti-obesity Messaging. PSYCHOSOMATIC MEDICINE. 2021;83(7):777-786.

4682. Swenne I, Thurfjell B Clinical onset and diagnosis of eating disorders in premenarcheal girls is preceded by inadequate weight gain and growth retardation. ACTA PAEDIATRICA. 2003;92(10):1133-1137.

4683. Daniel Sunil, Soleymani Taraneh, Garvey W. A complications-based clinical staging of obesity to guide treatment modality and intensity. CURRENT OPINION IN ENDOCRINOLOGY DIABETES AND OBESITY. 2013;20(5):377-388.

4684. Burke FJT, Bell TJ, Ismail N, Hartley P Bulimia: Implications for the practising dentist. BRITISH DENTAL JOURNAL. 1996;180(11):421-426.

4685. Steinberg Dori, Perry Taylor, Freestone David, Hellner Megan, Baker Jessica, Bohon Cara Evaluating differences in setting expected body weight for children and adolescents in eating disorder treatment. INTERNATIONAL JOURNAL OF EATING DISORDERS. 2023;56(3):595-603.

4686. Austin S., Spadano-Gasbarro Jennifer, Greaney Mary, Richmond Tracy, Feldman Henry, Osganian Stavroula, Hunt Anne, Mezgebu Solomon, Peterson Karen Disordered Weight Control Behaviors in Early Adolescent Boys and Girls of Color: An Under-Recognized Factor in the Epidemic of Childhood Overweight. JOURNAL OF ADOLESCENT HEALTH. 2011;48(1):109-112.

4687. Norton Lyza, Parkinson Joy, Harris Neil, Darcy Morgan, Hart Laura Parental food communication and child eating behaviours: A systematic literature review. HEALTH PROMOTION JOURNAL OF AUSTRALIA. 2023;34(2):366-378.

4688. Johnson Shannon, Edwards Katie, Gidycz Christine Interpersonal Weight-Related Pressure and Disordered Eating in College Women: A Test of an Expanded Tripartite Influence Model. SEX ROLES. 2015;72(1-2):15-24.

4689. Feig Emily, Piers Amani, Kral Tanja, Lowe Michael Eating in the absence of hunger is related to loss-of-control eating, hedonic hunger, and short-term weight gain in normal-weight women. APPETITE. 2018;123():317-324.

4690. Minkwitz Juliane, Scheipl Fabian, Cartwright Lydia, Campbell Iain, Chittka Tobias, Thormann Julia, Hegerl Ulrich, Sander Christian, Himmerich Hubertus Why some obese people become depressed whilst others do not: exploring links between cognitive reactivity, depression and obesity. PSYCHOLOGY HEALTH \& MEDICINE. 2019;24(3):362-373.

4691. Warriach Zain, Patel Sruti, Khan Fatima, Ferrer Gerardo Association of Depression With Cardiovascular Diseases. CUREUS JOURNAL OF MEDICAL SCIENCE. 2022;14(6):.

4692. Unikel-Santoncini Claudia, Bojorquez-Chapela Ietza, Hernandez-Serrato Maria, Villalobos-Hernandez Aremis Disordered eating behaviors and psychological correlates. Data from the Ensanut 2018-19. SALUD PUBLICA DE MEXICO. 2022;64(5):471-477.

4693. Reyes Miranda, Simpson Lauren, Sullivan Tami, Contractor Ateka, Weiss Nicole Intimate Partner Violence and Mental Health Outcomes Among Hispanic Women in the United States: A Scoping Review. TRAUMA VIOLENCE \& ABUSE. 2023;24(2):809-827.

4694. Mazzocchi Mario, Cagnone Silvia, Bech-Larsen Tino, Niedzwiedzka Barbara, Saba Anna, Shankar Bhavani, Verbeke Wim, Traill W. What is the public appetite for healthy eating policies? Evidence from a cross-European survey. HEALTH ECONOMICS POLICY AND LAW. 2015;10(3):267-292.

4695. Green M., Willis M., Fernandez-Kong K., Reyes S., Linkhart R., Johnson M., Thorne T., Lindberg J., Kroska E., Woodward H. A Controlled Randomized Preliminary Trial of a Modified Dissonance-Based Eating Disorder Intervention Program. JOURNAL OF CLINICAL PSYCHOLOGY. 2017;73(12):1612-1628.

4696. Kouvari Matina, Panagiotakos Demosthenes, Naumovski Nenad, Chrysohoou Christina, Georgousopoulou Ekavi, Yannakoulia Mary, Tousoulis Dimitrios, Pitsavos Christos, Investigators ATTICA Dietary anti-inflammatory index, metabolic syndrome and transition in metabolic status; a gender-specific analysis of ATTICA prospective study. DIABETES RESEARCH AND CLINICAL PRACTICE. 2020;161(SI):.

4697. Yoshimura Ai, Kusama Yoshiki, Omura Yuka, Shibata Mariko, Maihara Toshiro A Case of Eating Disorder Diagnosed As Orthorexia Nervosa. CUREUS JOURNAL OF MEDICAL SCIENCE. 2023;15(1):.

4698. Pacanowski Carly, Diers Lisa, Crosby Ross, Neumark-Sztainer Dianne Yoga in the treatment of eating disorders within a residential program: A randomized controlled trial.

EATING DISORDERS. 2017;25(1):37-51.

4699. Klem ML, Wing RR, SimkinSilverman L, Kuller LH The psychological consequences of weight gain prevention in healthy, premenopausal women. INTERNATIONAL JOURNAL OF EATING DISORDERS. 1997;21(2):167-174.

4700. Roehrig M, Thompson JK, Herbozo SM, Himes S, Shroff H, King H Dissonance-based eating disorder prevention program: A dismantling investigation. INTERNATIONAL JOURNAL OF EATING DISORDERS. 2004;35(4):476-477.

4701. Stice Eric, Marti C., Rohde Paul, Shaw Heather Testing Mediators Hypothesized to Account for the Effects of a Dissonance-Based Eating Disorder Prevention Program Over Longer Term Follow-Up. JOURNAL OF CONSULTING AND CLINICAL PSYCHOLOGY. 2011;79(3):398-405.

4702. Shanmugam Vaithehy, Jowett Sophia, Meyer Caroline Eating psychopathology as a risk factor for depressive symptoms in a sample of British athletes. JOURNAL OF SPORTS SCIENCES. 2014;32(17):1587-1595.

4703. Berczik Krisztina, Szabo Attila, Griffiths Mark, Kurimay Tamas, Kun Bernadette, Urban Robert, Demetrovics Zsolt Exercise Addiction: Symptoms, Diagnosis, Epidemiology, and Etiology. SUBSTANCE USE \& MISUSE. 2012;47(4):403-417.

4704. Wang Yong-yi, Liu Sha, Lian Feng, Yang Wen-gang, Xue Song Toll-like receptor 7/8 agonist resiquimod induces late preconditioning in neonatal cardiac myocytes. ACTA PHARMACOLOGICA SINICA. 2011;32(5):565-572.

4705. McCarroll Rebecca, Eyles Helen, Ni Mhurchu Cliona Effectiveness of mobile health (mHealth) interventions for promoting healthy eating in adults: A systematic review. PREVENTIVE MEDICINE. 2017;105():156-168.

4706. ROSE UM, COUWENBERG P, JANSEN JWCM, BINDELS RJM, VANOS CH EFFECTS OF SUBSTRATE-FREE ANOXIA AND VERATRIDINE ON INTRACELLULAR CALCIUM-CONCENTRATION IN ISOLATED RAT VENTRICULAR CARDIOMYOCYTES. PFLUGERS ARCHIV-EUROPEAN JOURNAL OF PHYSIOLOGY. 1994;428(2):142-149.

4707. Ajetunmobi Omotomilola, Taylor Mark, Stockton Diane, Wood Rachael Early death in those previously hospitalised for mental healthcare in Scotland: a nationwide cohort study, 1986-2010. BMJ OPEN. 2013;3(7):.

4708. Franklin Barry, Myers Jonathan, Kokkinos Peter Importance of Lifestyle Modification on Cardiovascular Risk Reduction COUNSELING STRATEGIES TO MAXIMIZE PATIENT OUTCOMES. JOURNAL OF CARDIOPULMONARY REHABILITATION AND PREVENTION. 2020;40(3):138-143.

4709. Dellava Jocilyn, Kendler Kenneth, Neale Michael GENERALIZED ANXIETY DISORDER AND ANOREXIA NERVOSA: EVIDENCE OF SHARED GENETIC VARIATION. DEPRESSION AND ANXIETY. 2011;28(8):728-733.

4710. Razzak Hira, Harbi Alya, Shelpai Wael, Qawas Ahmad Epidemiology of Diabetes Mellitus in the United Arab Emirates. CURRENT DIABETES REVIEWS. 2018;14(6):542-549.

4711. Asselmann E., Wittchen H., Lieb R., Beesdo-Baum K. Sociodemographic, clinical, and functional long-term outcomes in adolescents and young adults with mental disorders. ACTA PSYCHIATRICA SCANDINAVICA. 2018;137(1):6-17.

4712. Aspy Cheryl, Mold James, Thompson David, Blondell Richard, Landers Patti, Reilly Kathryn, Wright-Eakers Linda Integrating Screening and Interventions for Unhealthy Behaviors into Primary Care Practices. AMERICAN JOURNAL OF PREVENTIVE MEDICINE. 2008;35(5, S):S373-S380.

4713. Watkins B, Lask B Eating disorders in school-aged children. CHILD AND ADOLESCENT PSYCHIATRIC CLINICS OF NORTH AMERICA. 2002;11(2):185+.

4714. Bramorska Aleksandra, Zarzycka Wanda, Podolecka Wiktoria, Kuc Katarzyna, Brzezicka Aneta Age-Related Cognitive Decline May Be Moderated by Frequency of Specific Food Products Consumption. NUTRIENTS. 2021;13(8):.

4715. Tseng Mei-Chih, Fang David, Chang Chin-Hao, Lee Ming-Been Identifying high-school dance students who will develop an eating disorder: A 1-year prospective study. PSYCHIATRY RESEARCH. 2013;209(3):611-618.

4716. Herpertz-Dahlmann Beate, Dahmen Brigitte Children in Need-Diagnostics, Epidemiology, Treatment and Outcome of Early Onset Anorexia Nervosa. NUTRIENTS. 2019;11(8):.

4717. Yilmaz Zeynep, Kaplan Allan, Zai Clement, Levitan Robert, Kennedy James COMT Val158Met variant and functional haplotypes associated with childhood ADHD history in women with bulimia nervosa. PROGRESS IN NEURO-PSYCHOPHARMACOLOGY \& BIOLOGICAL PSYCHIATRY. 2011;35(4):948-952.

4718. Browne Mark, Wells J., Scott Kate, McGee Magnus, Res New Lifetime prevalence and projected lifetime risk of DSM-IV disorders in Te Rau Hinengaro: The New Zealand Mental Health Survey. AUSTRALIAN AND NEW ZEALAND JOURNAL OF PSYCHIATRY. 2006;40(10):865-874.

4719. Tavolacci M., Delay J., Grigioni S., Dechelotte P., Ladner J. Changes and specificities in health behaviors among healthcare students over an 8-year period. PLOS ONE. 2018;13(3):.

4720. Aparicio-Soto Marina, Sanchez-Hidalgo Marina, Angeles Rosillo Ma, Luisa Castejon Ma, Alarcon-de-la-Lastra Catalina Extra virgin olive oil: a key functional food for prevention of immune-inflammatory diseases. FOOD \& FUNCTION. 2016;7(11):4492-4505.

4721. Radin Rachel, Mason Ashley, Laudenslager Mark, Epel Elissa Maternal caregivers have confluence of altered cortisol, high reward-driven eating, and worse metabolic health. PLOS ONE. 2019;14(5):.

4722. Schwitzer Alan Diagnosing, Conceptualizing, and Treating Eating Disorders Not Otherwise Specified: A Comprehensive Practice Model. JOURNAL OF COUNSELING AND DEVELOPMENT. 2012;90(3):281-289.

4723. McComb JR, Cherry J, Romell M The relationship between eating disorder attitudes and the risk of cardiovascular disease. FAMILY \& COMMUNITY HEALTH. 2003;26(2):124-129.

4724. Karlsson Gunilla, Clinton David, Nevonen Lauri Prediction of weight increase in anorexia nervosa. NORDIC JOURNAL OF PSYCHIATRY. 2013;67(6):424-432.

4725. Beato-Fernandez Luis, Rodriguez-Cano Teresa, Pelayo-Delgado Esther, Calaf Myralys Are there gender-specific pathways from early adolescence psychological distress symptoms toward the development of substance use and abnormal eating behavior?. CHILD PSYCHIATRY \& HUMAN DEVELOPMENT. 2007;37(3):193-203.

4726. Paxton S., Damiano S. The Development of Body Image and Weight Bias in Childhood. . 2017;52():269-298.

4727. Sheldon C, Church J Reduced contribution from Na+/H+ exchange to acid extrusion during anoxia in adult rat hippocampal CA1 neurons. JOURNAL OF NEUROCHEMISTRY. 2004;88(3):594-603.

4728. Ilicic Jasmina, Brennan Stacey Shake it off and eat less: anxiety-inducing product packaging design influences food product interaction and eating. EUROPEAN JOURNAL OF MARKETING. 2022;56(2):562-583.

4729. Gonzales Manuel, Blashill Aaron Ethnic/racial and gender differences in body image disorders among a diverse sample of sexual minority US adults. BODY IMAGE. 2021;36():64-73.

4730. Melis Nicolas, Rubera Isabelle, Giraud Sebastien, Cougnon Marc, Duranton Christophe, Poet Mallorie, Jarretou Gisele, Thuillier Raphael, Counillon Laurent, Hauet Thierry, Pellerin Luc, Tauc Michel, Pisani Didier Renal Ischemia Tolerance Mediated by eIF5A Hypusination Inhibition Is Regulated by a Specific Modulation of the Endoplasmic Reticulum Stress. CELLS. 2023;12(3):.

4731. Voelker Dana, Visek Amanda, Learner Jordyn, DiBiasio Miranda Toward understanding of coaches' role in athletes' eating pathology: A systematic review and ecological application to advance research. PSYCHOLOGY OF SPORT AND EXERCISE. 2022;58():.

4732. Levitan Robert, Wendland Barbara Novel ``Thrifty{''} Models of Increased Eating Behaviour. CURRENT PSYCHIATRY REPORTS. 2013;15(11):.

4733. Marinaci Tiziana, Carpinelli Luna, Savarese Giulia What does anorexia nervosa mean? Qualitative study of the representation of the eating disorder, the role of the family and treatment by maternal caregivers. BJPSYCH OPEN. 2021;7(3):.

4734. Althunibat Osama, Saghir Sultan, Aladaileh Saleem, Alrawadeh Atika, Al-Areefi Mahmoud, Alghonmeen Reham, Alkhawaldeh Alayn, Obaidat Heba Distribution and association of weight-loss diet programs with body mass index and health status among students at Al-Hussein Bin Talal University, Jordan. ELECTRONIC JOURNAL OF GENERAL MEDICINE. 2022;19(6):.

4735. Cook-Cottone Catherine, Serwacki Michelle, Guyker Wendy, Sodano Sandro, Nickerson Amanda, Keddie-Olkas Emily, Anderson Laura The Role of Anxiety on the Experience of Peer Victimization and Eating Disorder Risk. SCHOOL MENTAL HEALTH. 2016;8(3):354-367.

4736. Fukao Atsushi, Takamatsu Junta, Arishima Takeshi, Tanaka Mika, Kawai Toshio, Okamoto Yasuki, Miyauchi Akira, Imagawa Akihisa Graves' disease and mental disorders. JOURNAL OF CLINICAL AND TRANSLATIONAL ENDOCRINOLOGY. 2020;19():.

4737. Vartapetian B., Polyakova L., Stepanova A., Dolgikh Yu Physiological role of nitrate under anaerobic stress in Saccharum officinarum callus cells tolerant and sensitive to anoxia. RUSSIAN JOURNAL OF PLANT PHYSIOLOGY. 2012;59(6):741-747.

4738. Bergman Elina, Vepsalainen Henna, Erkkola Maijaliisa, Laaksonen Marika, Kautiainen Hannu, Penttinen Markus, Rautava Paivi, Korhonen Paivi Healthy and Unhealthy Food Consumption in Relation to Quality of Life among Finnish Female Municipal Employees: A Cross-Sectional Study. NUTRIENTS. 2022;14(17):.

4739. Polidori Maria Preventive Benefits of Natural Nutrition and Lifestyle Counseling against Alzheimer's Disease Onset. JOURNAL OF ALZHEIMERS DISEASE. 2014;42(4):S475-S482.

4740. Schaefer Christoph, Keysser Gernot Lifestyle Factors and Their Influence on Rheumatoid Arthritis: A Narrative Review. JOURNAL OF CLINICAL MEDICINE. 2022;11(23):.

4741. Bjorklund Oda, Belsky Jay, Wichstrom Lars, Steinsbekk Silje Predictors of Eating Behavior in Middle Childhood: A Hybrid Fixed Effects Model. DEVELOPMENTAL PSYCHOLOGY. 2018;54(6):1099-1110.

4742. Algahtani Fahad Healthy Lifestyle among Ha'il University Students, Saudi Arabia. INTERNATIONAL JOURNAL OF PHARMACEUTICAL RESEARCH AND ALLIED SCIENCES.

2020;9(1):160-167.

4743. Groos Elisabeth, Chaumereuil Charlotte, Flamand Mathilde, Brion Agnes, Bourdin Hubert, Slimani Vanessa, Lecendreux Michel, Arnulf Isabelle Emerging psychiatric disorders in Kleine-Levin syndrome. JOURNAL OF SLEEP RESEARCH. 2018;27(5):.

4744. Cooley E., Toray T., Valdez N., Tee M. Risk factors for maladaptive eating patterns in college women. EATING AND WEIGHT DISORDERS-STUDIES ON ANOREXIA BULIMIA AND OBESITY. 2007;12(3):132-139.

4745. Tayyem Reema, Al-Awwad Narmeen, Allehdan Sabika, Ajeen Rawan, Al-Jaberi Tareq, Rayyan Yaser, Bawadi Hiba, Hushki Ahmad Mediterranean Dietary Pattern is Associated with Lower Odds of Gastric Cancer: A Case-Control. CANCER MANAGEMENT AND RESEARCH. 2022;14():2017-2029.

4746. Steinberg Sharon, Jotkowitz Alan THE ETHICS OF PUBLIC HEALTH LAWS, AND THE SPECIAL CASE OF THE NEW ``MODEL LAW{''}. PERSPECTIVES IN BIOLOGY AND MEDICINE. 2016;59(2):206-212.

4747. Conviser Jenny, Tierney Amanda, Nickols Riley Essentials for Best Practice: Treatment Approaches for Athletes With Eating Disorders. JOURNAL OF CLINICAL SPORT PSYCHOLOGY. 2018;12(4, SI):495-507.

4748. Cook-Cottone Catherine, Cox Anne, Neumark-Sztainer Dianne, Tylka Tracy Future directions for research on yoga and positive embodiment. EATING DISORDERS. 2020;28(4, SI):542-547.

4749. Xie Hejian, Li Jinchen, Zhu Xuanmeng, Li Jing, Yin Jinghua, Ma Tianqi, Luo Yi, He Lingfang, Bai Yongping, Zhang Guogang, Cheng Xunjie, Li Chuanchang Association between healthy lifestyle and the occurrence of cardiometabolic multimorbidity in hypertensive patients: a prospective cohort study of UK Biobank. CARDIOVASCULAR DIABETOLOGY. 2022;21(1):.

4750. Vornanen Matti, Haverinen Jaakko Glycogen dynamics of crucian carp (Carassius carassius) in prolonged anoxia. JOURNAL OF COMPARATIVE PHYSIOLOGY B-BIOCHEMICAL SYSTEMIC AND ENVIRONMENTAL PHYSIOLOGY. 2016;186(8):999-1007.

4751. Muscogiuri Giovanna, Barrea Luigi, Aprano Sara, Framondi Lydia, Di Matteo Rossana, Laudisio Daniela, Pugliese Gabriella, Savastano Silvia, Colao Annamaria, Project OPERA Sleep Quality in Obesity: Does Adherence to the Mediterranean Diet Matter?. NUTRIENTS. 2020;12(5):.

4752. Muratore Alexandra, Attia Evelyn Current Therapeutic Approaches to Anorexia Nervosa: State of the Art. CLINICAL THERAPEUTICS. 2021;43(1):85-94.

4753. Stice Eric, Rohde Paul, Durant Shelley, Shaw Heather, Wade Emily Effectiveness of peer-led dissonance-based eating disorder prevention groups: Results from two randomized pilot trials. BEHAVIOUR RESEARCH AND THERAPY. 2013;51(4-5):197-206.

4754. Anderson Hanna, Johengen Thomas, Godwin Casey, Purcell Heidi, Alsip Peter, Ruberg Steve, Mason Lacey Continuous In Situ Nutrient Analyzers Pinpoint the Onset and Rate of Internal P Loading under Anoxia in Lake Erie's Central Basin. ACS ES\&T WATER. 2021;1(4):774-781.

4755. Tajima Miki, Lee Jung, Watanabe Etsuko, Park Jong, Tsuchiya Rumiko, Fukahori Atsuko, Mori Katsumi, Kawakubo Kiyoshi Association Between Changes in 12 Lifestyle Behaviors and the Development of Metabolic Syndrome During 1 Year Among Workers in the Tokyo Metropolitan Area. CIRCULATION JOURNAL. 2014;78(5):1152-1159.

4756. Luk Jeremy, Miller Jacob, Lipsky Leah, Gilman Stephen, Haynie Denise, Simons-Morton Bruce A longitudinal investigation of perceived weight status as a mediator of sexual orientation disparities in maladaptive eating behaviors. EATING BEHAVIORS. 2019;33():85-90.

4757. Gulley Lauren, Shomaker Lauren Depression in Youth-Onset Type 2 Diabetes. CURRENT DIABETES REPORTS. 2020;20(10):.

4758. Pokrajac-Bulian A., Zivcic-Becirevic I., Calugi S., Dalle Grave R. School prevention program for eating disorders in Croatia: A controlled study with six months of follow-up. EATING AND WEIGHT DISORDERS-STUDIES ON ANOREXIA BULIMIA AND OBESITY. 2006;11(4):171-178.

4759. Di Raimondo Domenico, Buscemi Silvio, Musiari Gaia, Rizzo Giuliana, Pirera Edoardo, Corleo Davide, Pinto Antonio, Tuttolomondo Antonino Ketogenic Diet, Physical Activity, and

Hypertension-A Narrative Review. NUTRIENTS. 2021;13(8):.

4760. Mahmoud Ali, Grigoriou Nicholas Modelling parents' unhealthy food choices for their children: the moderating role of child food allergy and implications for health policy. JOURNAL OF FAMILY STUDIES. 2022;28(1):89-107.

4761. GLEASON NA A NEW APPROACH TO DISORDERED EATING - USING AN ELECTRONIC BULLETIN BOARD TO CONFRONT SOCIAL PRESSURE ON BODY-IMAGE. JOURNAL OF AMERICAN COLLEGE HEALTH. 1995;44(2):78-80.

4762. Yazdani Sahr, Bloomberg Zachary, Klauber Rachel, Meresh Edwin Avoidant restrictive food intake disorder emerging during COVID-19 pandemic resulting in superior mesenteric artery syndrome. EATING AND WEIGHT DISORDERS-STUDIES ON ANOREXIA BULIMIA AND OBESITY. 2022;27(7):2943-2945.

4763. Buck L, Espanol M, Litt L, Bickler P Reversible decreases in ATP and PCr concentrations in anoxic turtle brain. COMPARATIVE BIOCHEMISTRY AND PHYSIOLOGY A-MOLECULAR AND INTEGRATIVE PHYSIOLOGY. 1998;120(4):633-639.

4764. Wilksch Simon How can we improve dissemination of universal eating disorder risk reduction programs?. EATING BEHAVIORS. 2017;25(SI):58-61.

4765. Tudor-Locke C, McColl RS Factors related to variation in premenopausal bone mineral status: A health promotion approach. OSTEOPOROSIS INTERNATIONAL. 2000;11(1):1-24.

4766. Correa Natasha, Rajaraman Divya, Swaminathan Sumathi, Vaz Mario, Jayachitra K., Lear Scott, Punthakee Zubin Perceptions of healthy eating amongst Indian adolescents in India and Canada. APPETITE. 2017;116():471-479.

4767. Valle Cipatli, Garcia Fernando, Gomez Maria, Parker Paola, Ayuzo Brenda, Kanan Gina Weight stigma in Mexico and front-of-package labeling. A systemic review. SALUD MENTAL. 2022;45(2):81-87.

4768. Carson Traci, Tournat Troy, Sonneville Kendrin, Zernicke Ronald, Karvonen-Gutierrez Carrie Cultural and environmental associations with body image, diet and well-being in NCAA DI female distance runners: a qualitative analysis. BRITISH JOURNAL OF SPORTS

MEDICINE. 2021;55(8):.

4769. Baechle Christina, Hoyer Annika, Stahl-Pehe Anna, Castillo Katty, Toennies Thaddaeus, Lindner Lena, Reinauer Christina, Holl Reinhard, Kuss Oliver, Rosenbauer Joachim Course of Disordered Eating Behavior in Young People With Early-Onset Type I Diabetes: Prevalence, Symptoms, and Transition Probabilities. JOURNAL OF ADOLESCENT HEALTH. 2019;65(5):681-689.

4770. Custodio CM, Basford JR Delayed postanoxic encephalopathy: A case report and literature review. ARCHIVES OF PHYSICAL MEDICINE AND REHABILITATION. 2004;85(3):502-505.

4771. You Yanli, Jiang Yueming, Duan Xuewu, Su Xingguo, Song Lili, Liu Hai, Sun Jian, Yang Hengming Browning inhibition and quality maintenance of fresh-cut Chinese water chestnut by anoxia treatment. JOURNAL OF FOOD PROCESSING AND PRESERVATION. 2007;31(5):595-606.

4772. Martins JM, Trinca A, Afonso A, Carreiras F, Falcao J, Nunes JS, Vale S, Costa JC Psychoneuroendocrine characteristics of common obesity clinical subtypes. INTERNATIONAL JOURNAL OF OBESITY. 2001;25(1):24-32.

4773. Epel Elissa, Tomiyama A., Mason Ashley, Laraia Barbara, Hartman William, Ready Karen, Acree Michael, Adam Tanja, St Jeor Sachiko, Kessler David The Reward-Based Eating Drive Scale: A Self-Report Index of Reward-Based Eating. PLOS ONE. 2014;9(6):.

4774. Li Yanping, Ley Sylvia, VanderWeele Tyler, Curhan Gary, Rich-Edwards Janet, Willett Walter, Forman John, Hu Frank, Qi Lu Joint association between birth weight at term and later life adherence to a healthy lifestyle with risk of hypertension: a prospective cohort study. BMC MEDICINE. 2015;13():.

4775. Stice Eric, Shaw Heather, Marti C. A meta-analytic review of eating disorder prevention programs: Encouraging findings. ANNUAL REVIEW OF CLINICAL PSYCHOLOGY. 2007;3():207-231.

4776. Cohn Leigh, Murray Stuart, Walen Andrew, Wooldridge Tom Including the excluded: Males and gender minorities in eating disorder prevention. EATING DISORDERS. 2016;24(1, SI):114-120.

4777. Ladwig Robert, Hanson Paul, Dugan Hilary, Carey Cayelan, Zhang Yu, Shu Lele, Duffy Christopher, Cobourn Kelly Lake thermal structure drives interannual variability in summer anoxia dynamics in a eutrophic lake over 37 years. HYDROLOGY AND EARTH SYSTEM SCIENCES. 2021;25(2):1009-1032.

4778. Ramos-Martins Constanca, Oliveira Sara, Ferreira Claudia Can body appreciation buffer the association between external and internal shame experiences with eating psychopathology?. CLINICAL PSYCHOLOGIST. 2022;26(1):53-62.

4779. Butryn Meghan, Rohde Paul, Marti C., Stice Eric Do participant, facilitator, or group factors moderate effectiveness of the Body Project? Implications for dissemination. BEHAVIOUR RESEARCH AND THERAPY. 2014;61():142-149.

4780. Oldershaw Anna, Lavender Tony, Schmidt Ulrike Are socio-emotional and neurocognitive functioning predictors of therapeutic outcomes for adults with anorexia nervosa?. EUROPEAN EATING DISORDERS REVIEW. 2018;26(4):346-359.

4781. Estrem Hayley, Pados Britt, Park Jinhee, Knafl Kathleen, Thoyre Suzanne Feeding problems in infancy and early childhood: evolutionary concept analysis. JOURNAL OF ADVANCED NURSING. 2017;73(1):56-70.

4782. Bodell Lindsay, Brown Tiffany, Keel Pamela Weight Suppression Predicts Bulimic Symptoms at 20-Year Follow-Up: The Mediating Role of Drive for Thinness. JOURNAL OF ABNORMAL PSYCHOLOGY. 2017;126(1):32-37.

4783. Joy E, Clark N, Ireland ML, Martire J, Nattiv A, Varechok S Team management of the female athlete triad .2. Optimal treatment and prevention tactics - Roundtable. PHYSICIAN AND SPORTSMEDICINE. 1997;25(4):55-\&.

4784. Garcia-Cruz Eduardo, Luque Pilar, Alcaraz Antonio Can healthy life styles form a part of the treatment of the testosterone deficiency syndrome? A case report. REVISTA INTERNACIONAL DE ANDROLOGIA. 2012;10(1):33-36.

4785. Calcaterra Valeria, Mazzoni Chiara, Ballardini Donatella, Tomba Elena, Zuccotti Gian, Mameli Chiara, De Giuseppe Rachele, Cena Hellas Disturbed Eating Behaviors in Youth with Type 1 Diabetes: An Exploratory Study about Challenges in Diagnosis. DIAGNOSTICS. 2020;10(12):.

4786. Cai S., Zhu H., Li Q., Ma X., Yao K., Zhang S., Zheng S. Gender disparities in dietary status and its risk factors in underserved populations. PUBLIC HEALTH. 2012;126(4):324-331.

4787. Jankauskiene Rasa, Baceviciene Migle Body Image and Disturbed Eating Attitudes and Behaviors in Sport-Involved Adolescents: The Role of Gender and Sport Characteristics. NUTRIENTS. 2019;11(12):.

4788. Rampelli Simone, Guenther Kathrin, Turroni Silvia, Wolters Maike, Veidebaum Toomas, Kourides Yiannis, Molnar Denes, Lissner Lauren, Benitez-Paez Alfonso, Sanz Yolanda, Fraterman Arno, Michels Nathalie, Brigidi Patrizia, Candela Marco, Ahrens Wolfgang Pre-obese children's dysbiotic gut microbiome and unhealthy diets may predict the development of obesity. COMMUNICATIONS BIOLOGY. 2018;1():.

4789. Mitchell Karen, Masheb Robin, Smith Brian, Kehle-Forbes Shannon, Hardin Sabrina, Vogt Dawne Eating Disorder Measures in a Sample of Military Veterans: A Focus on Gender, Age, and Race/Ethnicity. PSYCHOLOGICAL ASSESSMENT. 2021;33(12):1226-1238.

4790. Rainer B, Rathner G EAT norms for German-speaking adolescent girls: A population-based study. ZEITSCHRIFT FUR KLINISCHE PSYCHOLOGIE PSYCHIATRIE UND PSYCHOTHERAPIE. 1997;45(1):16-35.

4791. Carter Jacqueline, McFarlane Traci, Bewell Carmen, Olmsted Marion, Woodside D., Kaplan Allan, Crosby Ross Maintenance Treatment for Anorexia Nervosa: A Comparison of Cognitive Behavior Therapy and Treatment as Usual. INTERNATIONAL JOURNAL OF EATING DISORDERS. 2009;42(3):202-207.

4792. Cimino Silvia, Cerniglia Luca, Paciello Marinella, Sinesi Stefania A Six-year Prospective Study on Children of Mothers with Eating Disorders: The Role of Paternal Psychological Profiles. EUROPEAN EATING DISORDERS REVIEW. 2013;21(3):238-246.

4793. Mehler Philip Clinical guidance on osteoporosis and eating disorders: the NEDA continuing education series. EATING DISORDERS. 2019;27(5):471-481.

4794. Puder J., Munsch S. Psychological correlates of childhood obesity. INTERNATIONAL JOURNAL OF OBESITY. 2010;34(2):S37-S43.

4795. Baskin Rachel, Hill Briony, Jacka Felice, O'Neil Adrienne, Skouteris Helen Antenatal dietary patterns and depressive symptoms during pregnancy and early post-partum. MATERNAL AND CHILD NUTRITION. 2017;13(1):.

4796. Malinauskiene Vilija, Malinauskas Romualdas Unhealthy food in relation to posttraumatic stress symptoms among adolescents. APPETITE. 2014;74():86-91.

4797. Steck EL, Abrams LM, Phelps L Positive psychology in the prevention of eating disorders. PSYCHOLOGY IN THE SCHOOLS. 2004;41(1):111-117.

4798. Behrens Gundula, Gredner Thomas, Stock Christian, Leitzmann Michael, Brenner Hermann, Mons Ute Cancers Due to Excess Weight, Low Physical Activity, and Unhealthy Diet Estimation of the Attributable Cancer Burden in Germany. DEUTSCHES ARZTEBLATT INTERNATIONAL. 2018;115(35-36):578+.

4799. Mansson Josefin, Parling Thomas, Swenne Ingemar Favorable Effects of Clearly Defined Interventions by Parents at the Start of Treatment of Adolescents with Restrictive Eating Disorders. INTERNATIONAL JOURNAL OF EATING DISORDERS. 2016;49(1):92-97.

4800. Pasupathy Dharmintra, Wood Angela, Pell Jill, Fleming Michael, Smith C. Rates of and Factors Associated With Delivery-Related Perinatal Death Among Term Infants in Scotland COMMENT. OBSTETRICAL \& GYNECOLOGICAL SURVEY. 2010;65(1):2-4.

4801. Mattioli Anna, Ballerini Puviani Matteo, Nasi Milena, Farinetti Alberto COVID-19 pandemic: the effects of quarantine on cardiovascular risk. EUROPEAN JOURNAL OF CLINICAL NUTRITION. 2020;74(6):852-855.

4802. Wilksch Simon, Durbridge Mitchell, Wade Tracey A preliminary controlled comparison of programs designed to reduce risk of eating disorders targeting perfectionism and media literacy. JOURNAL OF THE AMERICAN ACADEMY OF CHILD AND ADOLESCENT PSYCHIATRY. 2008;47(8):939-947.

4803. Jimenez-Bonilla J., Carril J., Pisano R., Marraco I., Martinez-Rodriguez I., Esteban A., Quattrociocchi H. Assessment of cerebral blood flow in patients with eating disorders in the acute clinical phase using Tc99m-HMPAO SPECT. REVISTA ESPANOLA DE MEDICINA NUCLEAR. 2008;27(5):350-354.

4804. Guarda Angela Treatment of anorexia nervosa: Insights and obstacles. PHYSIOLOGY \& BEHAVIOR. 2008;94(1, SI):113-120.

4805. Haerens L., De Bourdeaudhuij I., Barba G., Eiben G., Fernandez J., Hebestreit A., Kovacs E., Lasn H., Regber S., Shiakou M., De Henauw S., Consortium IDEFICS Developing the IDEFICS community-based intervention program to enhance eating behaviors in 2-to 8-year-old children: findings from focus groups with children and parents. HEALTH EDUCATION RESEARCH. 2009;24(3):381-393.

4806. Levine Michael, Murnen Sarah ``EVERYBODY KNOWS THAT MASS MEDIA ARE/ARE NOT {[}pick one] A CAUSE OF EATING DISORDERS{''}: A CRITICAL REVIEW OF EVIDENCE FOR A CAUSAL LINK BETWEEN MEDIA, NEGATIVE BODY IMAGE, AND DISORDERED EATING IN FEMALES. JOURNAL OF SOCIAL AND CLINICAL PSYCHOLOGY. 2009;28(1):9-42.

4807. Coopey Emily, Johnson George ``The male elephant in the room{''}: a qualitative evidence synthesis exploring male experiences of eating disorders. JOURNAL OF EATING DISORDERS. 2022;10(1):.

4808. Monzani Dario, Pancani Luca, Rusconi Patrice, Pravettoni Gabriella Perceived Onset Time of Medical Conditions: The Interplay Between Subjective Fear and Risk in Four Lifestyle Domains. PSYCHOLOGICAL REPORTS. 2022;125(6):2981-3005.

4809. Grunwald Martin, Wesemann Dorette Individual use of on line-consulting for persons affected with eating disorders and their relatives - Evaluation of an online consulting service. EUROPEAN EATING DISORDERS REVIEW. 2006;14(4):218-225.

4810. Morin Etienne, Michaud-Letourneau Isabelle, Couturier Yves, de Mathieu A whole-food, plant-based nutrition program: Evaluation of cardiovascular outcomes and exploration of food choices determinants. NUTRITION. 2019;66():54-61.

4811. Sepulveda Ana, Moreno-Encinas Alba, Martinez-Huertas Jose, Anastasiadou Dimitra, Nova Esther, Marcos Ascension, Gomez-Martinez Sonia, Villa-Asensi Jose, Mollejo Encarna, Graell Montserrat Toward a Biological, Psychological and Familial Approach of Eating Disorders at Onset: Case-Control ANOBAS Study. FRONTIERS IN PSYCHOLOGY. 2021;12():.

4812. Peyrot Mark, Skovlund Soren, Landgraf Ruediger Epidemiology and correlates of weight worry in the multinational Diabetes Attitudes, Wishes and Needs study. CURRENT

MEDICAL RESEARCH AND OPINION. 2009;25(8):1985-1993.

4813. Hebebrand J, Ballauff A, Hinney A, Herpertz S, Kopp W, Wewetzer C, Ziegler A, Blum WF, Remschmidt H Body weight regulation in anorexia nervosa under special consideration of leptin secretion. NERVENARZT. 1999;70(1):31-40.

4814. Thompson JK, Berg P, Roehrig M, Guarda AS, Heinberg LJ The Sociocultural Attitudes Towards Appearance Scale-3 (SATAQ-3): Development and validation. INTERNATIONAL JOURNAL OF EATING DISORDERS. 2004;35(3):293-304.

4815. Roberts EL, Chih CP The influence of age on pH regulation in hippocampal slices before, during, and after anoxia. JOURNAL OF CEREBRAL BLOOD FLOW AND METABOLISM. 1997;17(5):560-566.

4816. Dipasquale Salvatore, Pariante Carmine, Dazzan Paola, Aguglia Eugenio, McGuire Philip, Mondelli Valeria The dietary pattern of patients with schizophrenia: A systematic review. JOURNAL OF PSYCHIATRIC RESEARCH. 2013;47(2):197-207.

4817. Compton Michael, Daumit Gail, Druss Benjamin Cigarette smoking and overweight/obesity among individuals with serious mental illnesses: A preventive perspective. HARVARD REVIEW OF PSYCHIATRY. 2006;14(4):212-222.

4818. Dale Rachel, O'Rourke Teresa, Humer Elke, Jesser Andrea, Plener Paul, Pieh Christoph Mental Health of Apprentices during the COVID-19 Pandemic in Austria and the Effect of Gender, Migration Background, and Work Situation. INTERNATIONAL JOURNAL OF ENVIRONMENTAL RESEARCH AND PUBLIC HEALTH. 2021;18(17):.

4819. Latagliata Emanuele, Patrono Enrico, Puglisi-Allegra Stefano, Ventura Rossella Food seeking in spite of harmful consequences is under prefrontal cortical noradrenergic control. BMC NEUROSCIENCE. 2010;11():.

4820. Warren Cortney, Schafer Kerri, Crowley Mary, Olivardia Roberto Demographic and Work-Related Correlates of Job Burnout in Professional Eating Disorder Treatment Providers. PSYCHOTHERAPY. 2013;50(4):553-564.

4821. Kong Ji-Sook, Min Kyoung-Bok, Min Jin-Young Temporary Workers' Skipping of Meals and Eating Alone in South Korea: The Korean National Health and Nutrition Examination

Survey for 2013-2016. INTERNATIONAL JOURNAL OF ENVIRONMENTAL RESEARCH AND PUBLIC HEALTH. 2019;16(13):.

4822. Bryan Christopher, Yeager David, Hinojosa Cintia, Chabot Aimee, Bergen Holly, Kawamura Mari, Steubing Fred Harnessing adolescent values to motivate healthier eating. PROCEEDINGS OF THE NATIONAL ACADEMY OF SCIENCES OF THE UNITED STATES OF AMERICA. 2016;113(39):10830-10835.

4823. Buchholz Laura, Crowther Janis, Olds R., Smith Kathryn, Ridolfi Danielle Are restrained eaters accurate monitors of their intoxication? Results from a field experiment. ADDICTIVE BEHAVIORS. 2013;38(4):1966-1969.

4824. Stice Eric, Marti C., Shaw Heather, Rohde Paul Meta-analytic review of dissonance-based eating disorder prevention programs: Intervention, participant, and facilitator features that predict larger effects. CLINICAL PSYCHOLOGY REVIEW. 2019;70():91-107.

4825. Goodman LR, Warren MP The female athlete and menstrual function. CURRENT OPINION IN OBSTETRICS \& GYNECOLOGY. 2005;17(5):466-470.

4826. Chang Wei-wei, Nie Miao, Kang Yao-wen, He Lian-ping, Jin Yue-long, Yao Ying-shui Subclinical eating disorders in female medical students in Anhui, China: a cross-sectional study. NUTRICION HOSPITALARIA. 2015;31(4):1771-1777.

4827. Kim Jae-Sung, Lemasters John Opioid receptor-independent protection of ischemic rat hepatocytes by morphine. BIOCHEMICAL AND BIOPHYSICAL RESEARCH COMMUNICATIONS. 2006;351(4):958-964.

4828. Liu Jieyu, Teng Ziwei, Chen Zirong, Wei Zirou, Zou Tianxiang, Qin Yue, Yuan Hui, Liu Minghui, Chen Jindong, Tang Hui, Xiang Hui, Wu Haishan, Wu Renrong, Huang Jing Exploring the associations between behavioral health risk factors, abnormal eating attitudes and socio-demographic factors among Chinese youth: Survey of 7,984 vocational high school students in Hunan in 2020. FRONTIERS IN PSYCHIATRY. 2022;13():.

4829. Lunghar Janeline, Banu A. Dietary approaches in management of noncommunicable diseases: A review. INTERNATIONAL JOURNAL OF NONCOMMUNICABLE DISEASES. 2021;6(4):159-165.

4830. Yuan Lingzhi, Shen Peijun, Zheng Shaopeng, Wu Dongwen, Li Xinmeng, Cai Ting, Yao Yao, Song Yunhe, Wang Fen Analysis of living habit risk factors for esophageal cancer in central China: A bi-center case-control study. FRONTIERS IN ONCOLOGY. 2023;13():.

4831. Kuhl R, Schutze G Eating disorders during childhood and adolescence. NERVENHEILKUNDE. 2001;20(5):255-259.

4832. Roos Eira, Lahti Jouni, Rahkonen Ossi Lifestyle and cancer-a joint pairwise association of lifestyle habits with subsequent cancer diagnosis. EUROPEAN JOURNAL OF PUBLIC HEALTH. 2019;29(2):340-345.

4833. Unikel-Santoncini Claudia, Leon-Vazquez Concepcion, Rivera-Marquez Jose, Bojorquez-Chapela Ietza, Mendez-Rios Enrique Dissonance-based Program for Eating Disorders Prevention in Mexican University Students. PSYCHOSOCIAL INTERVENTION. 2019;28(1):29-35.

4834. Neumark-Sztainer Dianne Preventing Obesity and Eating Disorders in Adolescents: What Can Health Care Providers Do?. JOURNAL OF ADOLESCENT HEALTH. 2009;44(3):206-213.

4835. Murray S Eating disorders and criticism of cultural ideals. EUROPEAN EATING DISORDERS REVIEW. 1999;7(3):204-212.

4836. Becker Carolyn, Ciao Anna, Smith Lisa Moving from efficacy to effectiveness in eating disorders prevention: The sorority body image program. COGNITIVE AND BEHAVIORAL PRACTICE. 2008;15(1):18-27.

4837. Runfola Cristin The Body Project: A Dissonance-Based Eating Disorder Prevention Intervention, Updated Edition-Programs ThatWork. EATING DISORDERS. 2014;22(3):275-277.

4838. Robertson R., Van Dusen Rachel Motor patterning, ion regulation and spreading depolarization during CNS shutdown induced by experimental anoxia in Locusta migratoria. COMPARATIVE BIOCHEMISTRY AND PHYSIOLOGY A-MOLECULAR \& INTEGRATIVE PHYSIOLOGY. 2021;260():.

4839. Cybulski Lukasz, Ashcroft Darren, Carr Matthew, Garg Shruti, Chew-Graham Carolyn, Kapur Nav, Webb Roger Temporal trends in annual incidence rates for psychiatric disorders and self-harm among children and adolescents in the UK, 2003-2018. BMC PSYCHIATRY. 2021;21(1):.

4840. Rogers Naomi, Dinges David, Allison Kelly, Maislin Greg, Martino Nicole, O'Reardon John, Stunkard Albert Assessment of sleep in women with night eating syndrome. SLEEP. 2006;29(6):814-819.

4841. Luce KH, Winzelberg AJ, Zabinski MF, Osborne MI Internet-delivered psychological interventions for body image dissatisfaction and disordered eating. PSYCHOTHERAPY. 2003;40(1-2, SI):148-154.

4842. Collantoni Enrico, Elena Tenconi, Marco Solmi, Paolo Meneguzzo, Enrica Marzola, Federico D'Agata, Stefano Gotti, Giovanni Abbate, Renzo Manara, Angela Favaro Hippocampal volumes in anorexia nervosa at different stages of the disorder. EUROPEAN EATING DISORDERS REVIEW. 2021;29(1):112-122.

4843. KATCHMAN AN, HERSHKOWITZ N EARLY ANOXIA-INDUCED VESICULAR GLUTAMATE RELEASE RESULTS FROM MOBILIZATION OF CALCIUM FROM INTRACELLULAR STORES. JOURNAL OF NEUROPHYSIOLOGY. 1993;70(1):1-7.

4844. Knoll Susanne, Foecker Manuel, Hebebrand Johannes Clinical Problems Encountered in the Treatment of Adolescents with Anorexia Nervosa. ZEITSCHRIFT FUR KINDER-UND JUGENDPSYCHIATRIE UND PSYCHOTHERAPIE. 2013;41(6):433-446.

4845. Jung Sukyoung, Park Sohyun Positive association of unhealthy plant-based diets with the incidence of abdominal obesity in Korea: a comparison of baseline, most recent, and cumulative average diets. EPIDEMIOLOGY AND HEALTH. 2022;44():.

4846. Tillman Kathleen, Sell Darcie, Yates Lindsay, Mueller Nichole Effectiveness of one-time psychoeducational programming for students with high levels of eating concerns. EATING BEHAVIORS. 2015;19():133-138.

4847. Robert Margaux, Shankland Rebecca, Andreeva Valentina, Deschasaux-Tanguy Melanie, Kesse-Guyot Emmanuelle, Bellicha Alice, Leys Christophe, Hercberg Serge, Touvier Mathilde, Peneau Sandrine Resilience Is Associated with Less Eating Disorder Symptoms in the NutriNet-Sante Cohort Study. INTERNATIONAL JOURNAL OF ENVIRONMENTAL

RESEARCH AND PUBLIC HEALTH. 2022;19(3):.

4848. Walsh Cara, Mitchell Lee, Hrozanova Maria, Kotoulas Serafeim-Chrysovalantis, Derry Christopher, Morrison Ian, Riha Renata NREM Sleep Parasomnias Commencing in Childhood: Trauma and Atopy as Perpetuating Factors. CLOCKS \& SLEEP. 2022;4(4):549-560.

4849. Tay Chee, Chin Yit, Lee Shoo, Khouw Ilse, Poh Bee, Grp SEANUTS Association of Eating Behavior With Nutritional Status and Body Composition in Primary School-Aged Children. ASIA-PACIFIC JOURNAL OF PUBLIC HEALTH. 2016;28(5):47S-58S.

4850. Menzel Jessie, Schaefer Lauren, Burke Natasha, Mayhew Laura, Brannick Michael, Thompson J. Appearance-related teasing, body dissatisfaction, and disordered eating: A meta-analysis. BODY IMAGE. 2010;7(4):261-270.

4851. Bertuccio P., Rosato V., Andreano A., Ferraroni M., Decarli A., Edefonti V., La Vecchia C. Dietary patterns and gastric cancer risk: a systematic review and meta-analysis. ANNALS OF ONCOLOGY. 2013;24(6):1450-1458.

4852. Amella EJ Feeding and hydration issues for older adults with dementia. NURSING CLINICS OF NORTH AMERICA. 2004;39(3):607+.

4853. Watson Hunna, Hoiles Kimberley, Egan Sarah, Limburg Karina Normative Data for Female Adolescents with Eating Disorders on the Multidimensional Anxiety Scale for Children. INTERNATIONAL JOURNAL OF EATING DISORDERS. 2014;47(5):471-474.

4854. LeRoy Matthew, Gill Benjamin Evidence for the development of local anoxia during the Cambrian SPICE event in eastern North America. GEOBIOLOGY. 2019;17(4):381-400.

4855. Liu Yuting, Wang Haochen, Bai Bingqing, Liu Fengyao, Chen Yilin, Wang Yu, Liang Yanting, Shi Xiaohe, Yu Xueju, Wu Chao, Guo Lan, Ma Huan, Geng Qingshan Trends in Unhealthy Lifestyle Factors among Adults with Stroke in the United States between 1999 and 2018. JOURNAL OF CLINICAL MEDICINE. 2023;12(3):.

4856. Westerberg-Jacobson Josefin, Edlund Birgitta, Ghaderi Ata A 5-Year Longitudinal Study of the Relationship between the Wish to Be Thinner, Lifestyle Behaviours and Disturbed Eating in 9-20-Year Old Girls. EUROPEAN EATING DISORDERS REVIEW.

2010;18(3):207-219.

4857. Kilincel Oguzhan, Ay Rukiye Nighttime eating syndrome and its relationship with impulsivity in major depressive disorder. ANNALS OF CLINICAL AND ANALYTICAL MEDICINE. 2020;11(5):448-452.

4858. Mensi Martina, Rogantini Chiara, Nacinovich Renata, Riva Anna, Provenzi Livio, Chiappedi Matteo, Balottin Umberto, Borgatti Renato Clinical features of adolescents diagnosed with eating disorders and at risk for psychosis. EUROPEAN PSYCHIATRY. 2020;63(1):.

4859. Praveen Pradeep, Roy Ambuj, Prabhakaran Dorairaj Cardiovascular Disease Risk Factors: A Childhood Perspective. INDIAN JOURNAL OF PEDIATRICS. 2013;80(1):S3-S12.

4860. Hajivandi Leila, Noroozi Mahnaz, Mostafavi Firoozeh, Ekramzadeh Maryam A comprehensive interventional program for promoting eating behaviors in adolescent girls with polycystic ovarian syndrome (PCOS): protocol for a mixed methods study. REPRODUCTIVE HEALTH. 2018;15():.

4861. SCHIPPERS GM, COX WM PROBLEM PERCEPTION AND ADDICTIVE BEHAVIORS AMONG DUTCH AND AMERICAN-COLLEGE STUDENTS. DRUGS-EDUCATION PREVENTION AND POLICY. 1994;1(1):27-35.

4862. Morgan Helen, Winkel Abigail, Nguyen Anh, Carson Sandra, Ogburn Tony, Woodland Mark Obstetrics and Gynecology Residents' Perspectives on Wellness Findings From a National Survey. OBSTETRICS AND GYNECOLOGY. 2019;133(3):552-557.

4863. Correia Horvath Jaqueline, Castro Mariana, Kops Natalia, Malinoski Natasha, Friedman Rogerio Obesity coexists with malnutrition? adequacy of food consumption by severely obese patients to dietary reference intake recommendations. NUTRICION HOSPITALARIA. 2014;29(2):292-299.

4864. Farhadnejad Hossein, Darand Mina, Teymoori Farshad, Asghari Golaleh, Mirmiran Parvin, Azizi Fereidoun The association of Dietary Approach to Stop Hypertension (DASH) diet with metabolic healthy and metabolic unhealthy obesity phenotypes. SCIENTIFIC REPORTS. 2019;9():.

4865. Rubio-Tomas Teresa, Rueda-Robles Ascension, Plaza-Diaz Julio, alvarez-Mercado Ana Nutrition and cellular senescence in obesity-related disorders. JOURNAL OF NUTRITIONAL BIOCHEMISTRY. 2022;99():.

4866. Shriver Lenka, Dollar Jessica, Lawless Meg, Calkins Susan, Keane Susan, Shanahan Lilly, Wideman Laurie Longitudinal Associations between Emotion Regulation and Adiposity in Late Adolescence: Indirect Effects through Eating Behaviors. NUTRIENTS. 2019;11(3):.

4867. McDonald Andrea, Dawkins-Moultin Lenna, McWhinney Sharon Rural parents' beliefs about healthy eating. HEALTH EDUCATION JOURNAL. 2018;77(6, SI):705-719.

4868. Oganov R., Maslennikova G. Demographic situation and cardiovascular disease in Russia: problem scope and possible solutions. CARDIOVASCULAR THERAPY AND PREVENTION. 2007;6(8):7-14.

4869. Ralenkotter L, Dales C, Delcamp TJ, Hadley RW Cytosolic {[}Ca2+], {[}Na+], and pH in guinea pig ventricular myocytes exposed to anoxia and reoxygenation. AMERICAN JOURNAL OF PHYSIOLOGY-HEART AND CIRCULATORY PHYSIOLOGY. 1997;272(6):H2679-H2685.

4870. Lewis Hannah, Cini Erica Co-producing eating disorder prevention research with South Asian experts-by-experience: A case study. EUROPEAN EATING DISORDERS REVIEW. 2022;30(6):837.

4871. Jiang Shuai, Liu Hui, Li Chunbao Dietary Regulation of Oxidative Stress in Chronic Metabolic Diseases. FOODS. 2021;10(8):.

4872. Voelker U., Jacobi C., Taylor C. Adaptation and evaluation of an Internet-based prevention program for eating disorders in a sample of women with subclinical eating disorder symptoms: A pilot study. EATING AND WEIGHT DISORDERS-STUDIES ON ANOREXIA BULIMIA AND OBESITY. 2011;16(4):E270-E273.

4873. Ayyildiz Feride, Sahin Gulsah Effect of social media addiction on eating behavior, body weight and life satisfaction during pandemic period. BRITISH FOOD JOURNAL. 2022;124(9):2980-2992.

4874. Cruwys T., Haslam S., Fox N., McMahon H. GROUP INTERVENTIONS FACILITATE NORMATIVE CHANGE: INVESTIGATING THE MECHANISM OF ACTION IN EATING DISORDER PREVENTION GROUPS. INTERNATIONAL JOURNAL OF BEHAVIORAL MEDICINE. 2016;23(1):S78.

4875. Browne Marlene, Ward Pamela, Pickett Sarah, Cameron Erin Exploring the Experiences of Trainees in an Eating Disorder Prevention Program in Newfoundland and Labrador. INTERNATIONAL JOURNAL OF QUALITATIVE METHODS. 2018;17(1):6-7.

4876. Imayama Ikuyo, Alfano Catherine, Mason Caitlin, Wang Chiachi, Duggan Catherine, Campbell Kristin, Kong Angela, Foster-Schubert Karen, Blackburn George, Wang Ching-Yun, McTiernan Anne Weight and metabolic effects of dietary weight loss and exercise interventions in postmenopausal antidepressant medication users and non-users: A randomized controlled trial. PREVENTIVE MEDICINE. 2013;57(5):525-532.

4877. Thornley Louise, Signal Louise, Thomson George Does industry regulation of food advertising protect child rights?. CRITICAL PUBLIC HEALTH. 2010;20(1):25-33.

4878. Gowers S., Clark A., Roberts C., Byford S., Barrett S., Griffiths A., Edwards V., Bryan C., Smethurst N., Rowlands L., Roots P. A randomised controlled multicentre trial of treatments for adloescent anorexia nervosa including assessment of cost-effectiveness and patient acceptability - the TOuCAN trial. HEALTH TECHNOLOGY ASSESSMENT. 2010;14(15):1+.

4879. Brown Tiffany, Forney K., Pinner Dennis, Keel Pamela A randomized controlled trial of The Body Project: More Than Muscles for men with body dissatisfaction. INTERNATIONAL JOURNAL OF EATING DISORDERS. 2017;50(8):873-883.

4880. Westwood Heather, Tchanturia Kate Autism Spectrum Disorder in Anorexia Nervosa: An Updated Literature Review. CURRENT PSYCHIATRY REPORTS. 2017;19(7):.

4881. Leddy Meaghan, Lawrence Hal, Schulkin Jay Obstetrician-Gynecologists and Women's Mental Health: Findings of the Collaborative Ambulatory Research Network 2005-2009. OBSTETRICAL \& GYNECOLOGICAL SURVEY. 2011;66(5):316-323.

4882. Kennedy Grace, Forney Katherine, Pinner Dennis, Martinez Kimberly, Buchman-Schmitt Jennifer, Keel Pamela Reducing anticipated non-suicidal self-injury by improving body esteem in individuals with weight suppression: A proof of concept study.

INTERNATIONAL JOURNAL OF EATING DISORDERS. 2019;52(2):206-210.

4883. Sanborn CF, Horea M, Siemers BJ, Dieringer KI Disordered eating and the female athlete triad. CLINICS IN SPORTS MEDICINE. 2000;19(2):199+.

4884. Zeiler Michael, Philipp Julia, Truttmann Stefanie, Waldherr Karin, Wagner Gudrun, Karwautz Andreas Psychopathological Symptoms and Well-Being in Overweight and Underweight Adolescents: A Network Analysis. NUTRIENTS. 2021;13(11):.

4885. Lindenberg K., Bauer S., Moessner M., Kordy H. Individually tailored internet-based eating disorder prevention: A program for high-school and college students. PSYCHOLOGY \& HEALTH. 2009;24(1, SI):27.

4886. Roberts EL, He J, Chih CP The influence of glucose on intracellular and extracellular pH in rat hippocampal slices during and after anoxia. BRAIN RESEARCH. 1998;783(1):44-50.

4887. Nakaishi Lindsay, Sugden Steven, Merlo Gia Primary Care at the Intersection of Lifestyle Interventions and Unhealthy Substance Use. AMERICAN JOURNAL OF LIFESTYLE MEDICINE. 2022;():.

4888. Tseng Mei-Chih, Tu Chao-Ying, Hsieh Shu-Feng, Chang Chin-Hao Rates and trends in healthcare-detected incidence of anorexia nervosa and bulimia nervosa: A national health insurance claim data study in Taiwan, 2002-2013. INTERNATIONAL JOURNAL OF EATING DISORDERS. 2020;53(3):331-338.

4889. Stice E, Presnell K, Groesz L, Shaw H Effects of a weight maintenance diet on bulimic symptoms in adolescent girls: An experimental test of the dietary restraint theory. HEALTH PSYCHOLOGY. 2005;24(4):402-412.

4890. Esposito Katherine, Ciardiello Fortunato, Giugliano Dario Unhealthy diets: a common soil for the association of metabolic syndrome and cancer. ENDOCRINE. 2014;46(1):39-42.

4891. Gupta Aakriti, Breedon Sarah, Storey Kenneth Activation of p53 in anoxic freshwater crayfish, Faxonius virilis. JOURNAL OF EXPERIMENTAL BIOLOGY. 2022;225(12):.

4892. Button Eric, Aldridge Sarah, Palmer Robert Males Assessed by a Specialized Adult Eating Disorders Service: Patterns Over Time and Comparisons with Females. INTERNATIONAL JOURNAL OF EATING DISORDERS. 2008;41(8):758-761.

4893. McVey Gail Building partnerships with prevention experts targeting other mental health problems. EATING DISORDERS. 2016;24(1, SI):63-70.

4894. Vishwanath Arun Negative Public Perceptions of Juvenile Diabetics: Applying Attribution Theory to Understand the Public's Stigmatizing Views. HEALTH COMMUNICATION. 2014;29(5):516-526.

4895. Mizumoto Yuki, Sasaki Yoshinori, Sunakawa Hikaru, Tanese Shuichi, Shinohara Rena, Kurokouchi Toshinari, Sugimoto Kaori, Seto Manao, Ishida Masahiro, Itagaki Kotoe, Yoshida Yukino, Namekata Saori, Takahashi Momoka, Harada Ikuhiro, Sasaki Shoko, Saito Kiyoshi, Toguchi Yusuke, Hakosima Yuki, Usami Masahide Current situation and clinical burden of pediatricians for children with eating disorders during the COVID-19 pandemic. GLOBAL HEALTH \& MEDICINE. 2023;():.

4896. Fujiwara Tomoko, Sato Natsuyo, Awaji Hiroyo, Sakamoto Hiroko, Nakata Rieko Skipping breakfast adversely affects menstrual disorders in young college students. INTERNATIONAL JOURNAL OF FOOD SCIENCES AND NUTRITION. 2009;60(6):23-31.

4897. Gullon Patricia, Astray Gonzalo, Gullon Beatriz, Franco Daniel, Bastianello Campagnol Paulo, Lorenzo Jose Inclusion of seaweeds as healthy approach to formulate new low-salt meat products. CURRENT OPINION IN FOOD SCIENCE. 2021;40():20-25.

4898. Wang Monica, Walls Courtney, Peterson Karen, Richmond Tracy, Spadano-Gasbarro Jennifer, Greaney Mary, Blood Emily, Mezbebu Solomon, Ott Mary PROTECTIVE AND RISK FACTORS FOR EATING DISORDER SYMPTOMS AMONG MIDDLE SCHOOL YOUTH: IMPLICATIONS FOR PREVENTION PROGRAMS. JOURNAL OF ADOLESCENT HEALTH. 2011;48(2, 1):S52.

4899. Hujoel Philippe, Lingstrom Peter Nutrition, dental caries and periodontal disease: a narrative review. JOURNAL OF CLINICAL PERIODONTOLOGY. 2017;44(18, SI):S79-S84.

4900. Demeshkant Nataliia, Potyrala Katarzyna, Czrewiec Karolina A cognitive-behavioral eating disorder prevention program for children: A pilot study in Polish primary schools.

JOURNAL OF BEHAVIORAL ADDICTIONS. 2019;8(1):148.

4901. Axelsson K., Woessner M., Litsne H., Wheeler M., Flehr A., King A., Kalen M., Vandenput L., Lorentzon M. Eating disorders are associated with increased risk of fall injury and fracture in Swedish men and women. OSTEOPOROSIS INTERNATIONAL. 2022;33(6):1347-1355.

4902. Celiberto Larissa, Graef Franziska, Healey Genelle, Bosman Else, Jacobson Kevan, Sly Laura, Vallance Bruce Inflammatory bowel disease and immunonutrition: novel therapeutic approaches through modulation of diet and the gut microbiome. IMMUNOLOGY. 2018;155(1):36-52.

4903. Cruzat Mandich Claudia, Haemmerli Delucchi Constance LISTENING TO EATING DISORDER PATIENTS ABOUT THEIR TREATMENT. REVISTA ARGENTINA DE CLINICA PSICOLOGICA. 2009;18(2):135-141.

4904. Foster Katherine, Hicks Brian, Iacono William, McGue Matthew Alcohol Use Disorder in Women: Risks and Consequences of an Adolescent Onset and Persistent Course. PSYCHOLOGY OF ADDICTIVE BEHAVIORS. 2014;28(2):322-335.

4905. Ruiz-Lazaro Pedro, Calvo D., Villas E. ZARIMA program: secondary prevention of eating disorder and overweight in north of Spain. EUROPEAN CHILD \& ADOLESCENT PSYCHIATRY. 2015;24(1):S84-S85.

4906. Faulconbridge Lucy, Wadden Thomas, Rubin Richard, Wing Rena, Walkup Michael, Fabricatore Anthony, Coday Mace, Van Dorsten Brent, Mount David, Ewing Linda, Grp Look One-Year Changes in Symptoms of Depression and Weight in Overweight/Obese Individuals With Type 2 Diabetes in the Look AHEAD Study. OBESITY. 2012;20(4):783-793.

4907. Jang Saeheon, Jung Sungwon, Pae Chiun, Portland Kimberly, Nelson J., Patkar Ashwin Predictors of relapse in patients with major depressive disorder in a 52-week, fixed dose, double blind, randomized trial of selegiline transdermal system (STS). JOURNAL OF AFFECTIVE DISORDERS. 2013;151(3):854-859.

4908. Fuemmeler Bernard, Sheng Yaou, Schechter Julia, Do Elizabeth, Zucker Nancy, Majors Alesha, Maguire Rachel, Murphy Susan, Hoyo Cathrine, Kollins Scott Associations between attention deficit hyperactivity disorder symptoms and eating behaviors in early childhood.

PEDIATRIC OBESITY. 2020;15(7):.

4909. Gyamfi Daniel, Obirikorang Christian, Acheampong Emmanuel, Asamoah Evans, Sampong Bernard, Batu Emmanuella, Anto Enoch Weight management among school-aged children and adolescents: a quantitative assessment in a Ghanaian municipality. BMC PEDIATRICS. 2019;19(1):.

4910. Peng Xiang, Menhas Rashid, Dai Jianhui, Younas Muhammad The COVID-19 Pandemic and Overall Wellbeing: Mediating Role of Virtual Reality Fitness for Physical-Psychological Health and Physical Activity. PSYCHOLOGY RESEARCH AND BEHAVIOR MANAGEMENT. 2022;15():1741-1756.

4911. Sanigorski A., Bell A., Kremer P., Cuttler R., Swinburn B. Reducing unhealthy weight gain in children through community capacity-building: results of a quasi-experimental intervention program, Be Active Eat Well. INTERNATIONAL JOURNAL OF OBESITY. 2008;32(7):1060-1067.

4912. Watkins B, Willoughby K, Waller G, Serpell L, Lask B Pattern of birth in anorexia nervosa I: Early-onset cases in the United Kingdom. INTERNATIONAL JOURNAL OF EATING DISORDERS. 2002;32(1):11-17.

4913. Peters EM Nutritional aspects in ultra-endurance exercise. CURRENT OPINION IN CLINICAL NUTRITION AND METABOLIC CARE. 2003;6(4):427-434.

4914. ALVIN P, ZOGHEIB J, REY C, LOSAY J SEVERE COMPLICATIONS OF EATING DISORDERS IN ADOLESCENTS - 99 HOSPITALIZED-PATIENTS. ARCHIVES FRANCAISES DE PEDIATRIE. 1993;50(9):755-762.

4915. Ghandour Reem, Kogan Michael, Blumberg Stephen, Perry Deborah Prevalence and Correlates of Internalizing Mental Health Symptoms Among CSHCN. PEDIATRICS. 2010;125(2):E269-E277.

4916. Duncan Scott, McPhee Julia, Schluter Philip, Zinn Caryn, Smith Richard, Schofield Grant Efficacy of a compulsory homework programme for increasing physical activity and healthy eating in children: the healthy homework pilot study. INTERNATIONAL JOURNAL OF BEHAVIORAL NUTRITION AND PHYSICAL ACTIVITY. 2011;8():.

4917. Hartman-Munick Sydney, Lin Jessica, Milliren Carly, Braverman Paula, Brigham Kathryn, Fisher Martin, Golden Neville, Jary Jessica, Lemly Diana, Matthews Abigail, Ornstein Rollyn, Roche Alexandra, Rome Ellen, Rosen Elaine, Sharma Yamini, Shook Jennifer, Taylor Jaime, Thew Margaret, Vo Megen, Voss Michaela, Woods Elizabeth, Forman Sara, Richmond Tracy Association of the COVID-19 Pandemic With Adolescent and Young Adult Eating Disorder Care Volume. JAMA PEDIATRICS. 2022;176(12):1225-1232.

4918. Nagai K, Hosaka H, Kubo S, Nakabayashi T, Amagasaki Y, Nakamura N Vitamin A toxicity secondary to excessive intake of yellow-green vegetables, liver and laver. JOURNAL OF HEPATOLOGY. 1999;31(1):142-148.

4919. Smink F., Hoeken D., Donker G., Susser E., Oldehinkel A., Hoek H. Three decades of eating disorders in Dutch primary care: decreasing incidence of bulimia nervosa but not of anorexia nervosa. PSYCHOLOGICAL MEDICINE. 2016;46(6):1189-1196.

4920. Heredia Natalia, Lee MinJae, Mitchell-Bennett Lisa, Reininger Belinda Tu Salud !Si Cuenta! Your Health Matters! A Community-wide Campaign in a Hispanic Border Community in Texas. JOURNAL OF NUTRITION EDUCATION AND BEHAVIOR. 2017;49(10):801+.

4921. Li Ang, Li Xiang, Zhou Tao, Ma Hao, Heianza Yoriko, Williamson Donald, Smith Steven, Bray George, Sacks Frank, Qi Lu Sleep Disturbance and Changes in Energy Intake and Body Composition During Weight Loss in the POUNDS Lost Trial. DIABETES. 2022;71(5):934-944.

4922. Ponsford Jennie, Alway Yvette, Gould Kate Epidemiology and Natural History of Psychiatric Disorders After TBI. JOURNAL OF NEUROPSYCHIATRY AND CLINICAL NEUROSCIENCES. 2018;30(4):262-270.

4923. Marttila-Tornio Kaisa, Mannikko Niko, Ruotsalainen Heidi, Miettunen Jouko, Kaariainen Maria Lower parental socioeconomic status in childhood and adolescence predicts unhealthy health behaviour patterns in adolescence in Northern Finland. SCANDINAVIAN JOURNAL OF CARING SCIENCES. 2021;35(3):742-752.

4924. Shields Clarissa, Hultstrand Kara, West Caroline, Gunstad John, Sato Amy Disinhibited Eating and Executive Functioning in Children and Adolescents: A Systematic Review and Meta-Analysis. INTERNATIONAL JOURNAL OF ENVIRONMENTAL RESEARCH AND PUBLIC HEALTH. 2022;19(20):.

4925. Nechita Diana-Mirela, David Daniel The longitudinal links between shame, eating disorders and social anxiety symptoms: a cross-lagged panel analysis. CURRENT PSYCHOLOGY. 2022;():.

4926. Juli Maria CHILDHOOD OBESITY: GLOBAL ISSUES. PSYCHIATRIA DANUBINA. 2016;28(1):S87-S91.

4927. Dias Medici Saldiva Silvia, Venancio Sonia, Santana Andreia, Silva Castro Ana, Loureiro Escuder Maria, Justo Giugliani Elsa The consumption of unhealthy foods by Brazilian children is influenced by their mother's educational level. NUTRITION JOURNAL. 2014;13():.

4928. Stanford JN, McCabe MP Evaluation of a body image prevention programme for adolescent boys. EUROPEAN EATING DISORDERS REVIEW. 2005;13(5):360-370.

4929. Laplante Francois, Brake Wayne, Chehab Sara, Sullivan Ron Sex differences in the effects of perinatal anoxia on dopamine function in rats. NEUROSCIENCE LETTERS. 2012;506(1):89-93.

4930. Stice Eric, Shaw Heather, Becker Carolyn, Rohde Paul Dissonance-based interventions for the prevention of eating disorders: Using persuasion principles to promote health. PREVENTION SCIENCE. 2008;9(2):114-128.

4931. Musetti Alessandro, Gagliardini Giulia, Lenzo Vittorio, Cella Stefania From Childhood Emotional Maltreatment to Disordered Eating: A Path Analysis. PSYCHOANALYTIC PSYCHOLOGY. 2023;40(2):90-98.

4932. Bhattarai Sanju, Aryal Anu, Pyakurel Manita, Bajracharya Swornim, Baral Phanindra, Citrin David, Cox Helen, Dhimal Meghnath, Fitzpatrick Annette, Jha Anjani, Jha Niharika, Karmacharya Biraj, Koju Rajendra, Maharjan Rashmi, Oli Natalia, Pyakurel Prajjwal, Sapkota Bhim, Shrestha Rajeev, Shrestha Soniya, Spiegelman Donna, Vaidya Abhinav, Shrestha Archana Cardiovascular disease trends in Nepal - An analysis of global burden of disease data 2017. IJC HEART \& VASCULATURE. 2020;30():.

4933. Clemente-Suarez Vicente, Martinez-Gonzalez Marina, Benitez-Agudelo Juan, Navarro-Jimenez Eduardo, Beltran-Velasco Ana, Ruisoto Pablo, Diaz Arroyo Esperanza, Laborde-Cardenas Carmen, Tornero-Aguilera Jose The Impact of the COVID-19 Pandemic on Mental Disorders. A Critical Review. INTERNATIONAL JOURNAL OF ENVIRONMENTAL RESEARCH

AND PUBLIC HEALTH. 2021;18(19):.

4934. Farooq Muhammad, Moore Philip, Bhatt Archit, Aburashed Rany, Kassab Mounzer Therapeutic role of zonisamide in neuropsychiatric disorders. MINI-REVIEWS IN MEDICINAL CHEMISTRY. 2008;8(10):968-975.

4935. Forney K., Holland Lauren, Keel Pamela Influence of peer context on the relationship between body dissatisfaction and eating pathology in women and men. INTERNATIONAL JOURNAL OF EATING DISORDERS. 2012;45(8):982-989.

4936. Lazaro Iolanda, Bobi Joaquim, Cofan Montserrat, Kapravelou Garyfallia, Amor Antonio, Surra Joaquin, Gomez-Guerrero Carmen, Ortega Emilio, Osada Jesus, Dantas Ana, Sala-Vila Aleix Walnut inclusion in a palm oil-based atherogenic diet promotes traits predicting stable atheroma plaque in Apoe-deficient mice. FRONTIERS IN NUTRITION. 2023;10():.

4937. Brown Tiffany, Keel Pamela A randomized controlled trial of a peer co-led dissonance-based eating disorder prevention program for gay men. BEHAVIOUR RESEARCH AND THERAPY. 2015;74():1-10.

4938. Wiklund Camilla, Kuja-Halkola Ralf, Thornton Laura, Balter Katarina, Welch Elisabeth, Bulik Cynthia Childhood body mass index and development of eating disorder traits across adolescence. EUROPEAN EATING DISORDERS REVIEW. 2018;26(5):462-471.

4939. Raja N., Osman Nermin, Alqethami Abdullah, Abd El-Fatah Nesrin The relationship between the high-risk disordered eating and social network navigation among Saudi college females during the COVID pandemic. FRONTIERS IN PUBLIC HEALTH. 2022;10():.

4940. Stice Eric, Rohde Paul, Durant Shelley, Shaw Heather A Preliminary Trial of a Prototype Internet Dissonance-Based Eating Disorder Prevention Program for Young Women With Body Image Concerns. JOURNAL OF CONSULTING AND CLINICAL PSYCHOLOGY. 2012;80(5):907-916.

4941. Stice Eric, Bohon Cara, Shaw Heather, Desjardins Christopher Efficacy of Virtual Delivery of a Dissonance-Based Eating Disorder Prevention Program and Evaluation of a Donation Model to Support Sustained Implementation. JOURNAL OF CONSULTING AND CLINICAL PSYCHOLOGY. 2023;91(3):139-149.

4942. Kamarli Altun Hulya, Keser Ilkay, Bozkurt Selen Comparison of Eating Attitudes and the Susceptibility to Orthorexia Nervosa of Students in Health-Related Fields and Those in Other Fields. IRANIAN JOURNAL OF PUBLIC HEALTH. 2020;49(3):495-502.

4943. Pamies-Aubalat Lidia, Quiles Marcos Yolanda, Bernabe Castano Miguel Study of risk of eating disorders in a representative sample of adolescents. MEDICINA CLINICA. 2011;136(4):139-143.

4944. Lawrence Sally, Boyle Maria, Craypo Lisa, Samuels Sarah The Food and Beverage Vending Environment in Health Care Facilities Participating in the Healthy Eating, Active Communities Program. PEDIATRICS. 2009;123(S):S287-S292.

4945. Rohde Paul, Stice Eric, Shaw Heather, Gau Jeff, Ohls Olivia Age effects in eating disorder baseline risk factors and prevention intervention effects. INTERNATIONAL JOURNAL OF EATING DISORDERS. 2017;50(11):1273-1280.

4946. Bardone-Cone Anna, Brownstone Lisa, Higgins M., Harney Megan, Fitzsimmons-Craft Ellen PREDICTING DIFFICULTIES CONTROLLING OVEREATING AND DRINKING WHEN EXPERIENCING NEGATIVE AFFECT IN UNDERGRADUATE WOMEN. JOURNAL OF SOCIAL AND CLINICAL PSYCHOLOGY. 2012;31(10):1051-1073.

4947. BONIECE IR, WAGNER JA NGF PROTECTS PC12 CELLS AGAINST ISCHEMIA BY A MECHANISM THAT REQUIRES THE N-KINASE. JOURNAL OF NEUROSCIENCE RESEARCH. 1995;40(1):1-9.

4948. Jun Taejin, Huan Jiancheng, Eaga Linda, Oldenburg Diane Influence of school-based nutrition education program on healthy eating literacy and healthy food choice among primary school children. INTERNATIONAL JOURNAL OF HEALTH PROMOTION AND EDUCATION. 2019;57(2):67-81.

4949. Lewis Hannah, Foye Una From prevention to peer support: A systematic review exploring the involvement of lived experience in eating disorder interventions. EUROPEAN EATING DISORDERS REVIEW. 2022;30(6):837.

4950. Gomez-Candela Carmen, Palma Milla Samara, Mijan-de-la-Torre Alberto, Rodriguez Ortega Pilar, Matia Martin Pilar, Loria Kohen Viviana, Portillo Rocio, Virgili Casas Ma, Martinez Olmos Miguel, Mories Alvarez Maria, Castro Alija Maria, Martin-Palmero Angela Consensus on the evaluation and nutritional treatment of eating disorders: anorexia

nervosa. NUTRICION HOSPITALARIA. 2018;35(1):11-48.

4951. Saulle R., Boggi R., Abetti P., Napoli M., Zannini S., Ravelli G., Ruggieri A., La Torre G. Can the Local Health Unit staff serve as role model for positive health behaviours? Results from an observational study in Italy. ANNALI DI IGIENE MEDICINA PREVENTIVA E DI COMUNITA. 2018;30(1):3-13.

4952. Breithaupt Lauren, Eickman Laura, Byrne Catherine, Fischer Sarah REbeL peer education: A model of a voluntary, after-school program for eating disorder prevention. EATING BEHAVIORS. 2019;32():111-116.

4953. Ivy Catherine, Robertson Cayleih, Bernier Nicholas Acute embryonic anoxia exposure favours the development of a dominant and aggressive phenotype in adult zebrafish. PROCEEDINGS OF THE ROYAL SOCIETY B-BIOLOGICAL SCIENCES. 2017;284(1846):.

4954. Opler Mark, Sodhi Dimple, Zaveri Deval, Madhusoodanan Subramoniam Primary psychiatric prevention in children and adolescents. ANNALS OF CLINICAL PSYCHIATRY. 2010;22(4):220-234.

4955. Reguero Marina, Cedron Marta, Wagner Sonia, Reglero Guillermo, Quintela Jose, Molina Ana Precision Nutrition to Activate Thermogenesis as a Complementary Approach to Target Obesity and Associated-Metabolic-Disorders. CANCERS. 2021;13(4):.

4956. Sherrington Anna, Oakes Steve, Hunter-Jones Philippa Advertising healthy eating to young consumers: insights from English and Swedish adolescents. JOURNAL OF MARKETING MANAGEMENT. 2021;37(15-16):1624-1655.

4957. Cadwallader Jean, Godart Nathalie, Chastang Julie, Falissard Bruno, Huas Caroline Detecting eating disorder patients in a general practice setting: a systematic review of heterogeneous data on clinical outcomes and care trajectories. EATING AND WEIGHT DISORDERS-STUDIES ON ANOREXIA BULIMIA AND OBESITY. 2016;21(3):365-381.

4958. Escarfulleri Shaline, Ellickson-Larew Stephanie, Fein-Schaffer Dana, Mitchell Karen, Wolf Erika Emotion regulation and the association between PTSD, diet, and exercise: a longitudinal evaluation among US military veterans. EUROPEAN JOURNAL OF PSYCHOTRAUMATOLOGY. 2021;12(1):.

4959. Call Christine, D'Adamo Laura, Butryn Meghan, Stice Eric THE IMPACT OF WEIGHT SUPPRESSION AND DIETARY RESTRAINT ON OUTCOMES IN AN EATING DISORDER AND OBESITY PREVENTION TRIAL. ANNALS OF BEHAVIORAL MEDICINE. 2020;54(1):S619.

4960. Awofisoye Oyindamola, Olalekan Olaleye, Anumenechi Ndubuisi, Onwukpa Frankilin Boerhaave's syndrome after pentazocine-induced vomiting in a 21-year-old male with asthma: a case report. PAN AFRICAN MEDICAL JOURNAL. 2021;38():.

4961. Liu Yi-Hsuan, Gao Xiang, Na Muzi, Kris-Etherton Penny, Mitchell Diane, Jensen Gordon Dietary Pattern, Diet Quality, and Dementia: A Systematic Review and Meta-Analysis of Prospective Cohort Studies. JOURNAL OF ALZHEIMERS DISEASE. 2020;78(1):151-168.

4962. Posma Joram, Garcia-Perez Isabel, Frost Gary, Aljuraiban Ghadeer, Chan Queenie, Van Horn Linda, Daviglus Martha, Stamler Jeremiah, Holmes Elaine, Elliott Paul, Nicholson Jeremy Nutriome-metabolome relationships provide insights into dietary intake and metabolism. NATURE FOOD. 2020;1(7):.

4963. Bair Carrie, Kelly Nichole, Serdar Kasey, Mazzeo Suzanne Does the Internet function like magazines? An exploration of image-focused media, eating pathology, and body dissatisfaction. EATING BEHAVIORS. 2012;13(4):398-401.

4964. Matthews Abigail, Peterson Claire Intensive Family-Based Therapy During an Acute Medical Admission for Anorexia Nervosa: A Case Report. CLINICAL CASE STUDIES. 2016;15(4):313-325.

4965. Pantano Kathleen Coaching Concerns in Physically Active Girls and Young Women-Part II: Practical Application of Research. STRENGTH AND CONDITIONING JOURNAL. 2010;32(2):68-72.

4966. Goswami Nandu, Trozic Irhad, Fredriksen Maren, Fredriksen Per The effect of physical activity intervention and nutritional habits on anthropometric measures in elementary school children: the health oriented pedagogical project (HOPP). INTERNATIONAL JOURNAL OF OBESITY. 2021;45(8):1677-1686.

4967. Cortes-Garcia Laura, Takkouche Bahi, Seoane Gloria, Senra Carmen Mediators linking insecure attachment to eating symptoms: A systematic review and meta-analysis. PLOS ONE. 2019;14(3):.

4968. Findling RL, Steiner H, Weller EB Use of antipsychotics in children and adolescents. JOURNAL OF CLINICAL PSYCHIATRY. 2005;66(7):29-40.

4969. Abiri Behnaz, Valizadeh Majid, Nasreddine Lara, Hosseinpanah Farhad Dietary determinants of healthy/unhealthy metabolic phenotype in individuals with normal weight or overweight/obesity: a systematic review. CRITICAL REVIEWS IN FOOD SCIENCE AND NUTRITION. 2022;():.

4970. Bullis Jacqueline, Boettcher Hannah, Sauer-Zavala Shannon, Farchione Todd, Barlow David What is an emotional disorder? A transdiagnostic mechanistic definition with implications for assessment, treatment, and prevention. CLINICAL PSYCHOLOGY-SCIENCE AND PRACTICE. 2019;26(2):.

4971. Hildebrandt Tom, Harty Seth, Langenbucher James Fitness Supplements as a Gateway Substance for Anabolic-Androgenic Steroid Use. PSYCHOLOGY OF ADDICTIVE BEHAVIORS. 2012;26(4):955-962.

4972. WARSHAW MG, MASSION AO, PETERSON LG, PRATT LA, KELLER MB SUICIDAL-BEHAVIOR IN PATIENTS WITH PANIC DISORDER - RETROSPECTIVE AND PROSPECTIVE DATA. JOURNAL OF AFFECTIVE DISORDERS. 1995;34(3):235-247.

4973. Buelens Tinne, Luyckx Koen, Verschueren Margaux, Schoevaerts Katrien, Dierckx Eva, Depestele Lies, Claes Laurence Temperament and Character Traits of Female Eating Disorder Patients with(out) Non-Suicidal Self-Injury. JOURNAL OF CLINICAL MEDICINE. 2020;9(4):.

4974. Aspen Vandana, Martijn Carolien, Alleva Jessica, Nagel Jessica, Perret Cassie, Purvis Clare, Saekow Jenine, Lock James, Taylor C. Decreasing body dissatisfaction using a brief conditioning intervention. BEHAVIOUR RESEARCH AND THERAPY. 2015;69():93-99.

4975. Rivera-Ingraham Georgina, Rocchetta Iara, Meyer Stefanie, Abele Doris Oxygen radical formation in anoxic transgression and anoxia-reoxygenation: Foe or phantom? Experiments with a hypoxia tolerant bivalve. MARINE ENVIRONMENTAL RESEARCH. 2013;92():110-119.

4976. Bilsky Sarah, Olson Emily, Luber Maxwell, Petell Jennifer, Friedman Hannah An initial examination of the associations between appearance-related safety behaviors, socioemotional, and body dysmorphia symptoms during adolescence. JOURNAL OF

ADOLESCENCE. 2022;94(7):939-954.

4977. Breithaupt Lauren, Eickman Laura, Byrne Catherine, Fischer Sarah REbeL Peer Education: A model of a voluntary, after-school program for eating disorder prevention. EATING BEHAVIORS. 2017;25(SI):32-37.

4978. McMillan Whitney, Stice Eric, Rohde Paul High- and Low-Level Dissonance-Based Eating Disorder Prevention Programs With Young Women With Body Image Concerns: An Experimental Trial. JOURNAL OF CONSULTING AND CLINICAL PSYCHOLOGY. 2011;79(1):129-134.

4979. Jarosch Marlene, Gebhardt Christine, Fano Silvia, Huchzermeyer Christine, Haq Rizwan, Behrens Christoph, Heinemann Uwe Early adenosine release contributes to hypoxia-induced disruption of stimulus-induced sharp wave-ripple complexes in rat hippocampal area CA3. EUROPEAN JOURNAL OF NEUROSCIENCE. 2015;42(2):1808-1817.

4980. Lowe MR, Davis W, Lucks D, Annunziato R, Butryn M Weight suppression predicts weight gain during inpatient treatment of bulimia nervosa. PHYSIOLOGY \& BEHAVIOR. 2006;87(3):487-492.

4981. LINDSTROM P, BRISMAR T, SIMA AAF IMPAIRED RECOVERY IN DIABETIC RAT NERVE FOLLOWING ANOXIC CONDUCTION BLOCK. DIABETES RESEARCH AND CLINICAL PRACTICE. 1994;25(3):177-181.

4982. Kothari Anita, Gore Dana, MacDonald Marjorie, Bursey Gayle, Allan Diane, Scarr Jennifer, Team Renewal Chronic disease prevention policy in British Columbia and Ontario in light of public health renewal: a comparative policy analysis. BMC PUBLIC HEALTH. 2013;13():.

4983. Nakamizo Akira, Yoshimoto Koji, Amano Toshiyuki, Mizoguchi Masahiro, Sasaki Tomio Crocodile tears syndrome after vestibular schwannoma surgery. JOURNAL OF NEUROSURGERY. 2012;116(5):1121-1125.

4984. Gutierrez Teresa, Espinoza Paola, Penelo Eva, Mora Marisol, Gonzalez Marcela, Roses Rocio, Raich Rosa Association of biological, psychological and lifestyle risk factors for eating disturbances in adolescents. JOURNAL OF HEALTH PSYCHOLOGY. 2015;20(6, SI):839-849.

4985. Pearson Natalie, Griffiths Paula, Biddle Stuart, Johnston Julie, McGeorge Sonia, Haycraft Emma Clustering and correlates of screen-time and eating behaviours among young adolescents. BMC PUBLIC HEALTH. 2017;17():.

4986. Jacka Felice, Sacks Gary, Berk Michael, Allender Steven Food policies for physical and mental health. BMC PSYCHIATRY. 2014;14():.

4987. Hayden EP, Klein DN Outcome of dysthymic disorder at 5-year follow-up: The effect of familial psychopathology, early adversity, personality, comorbidity, and chronic stress. AMERICAN JOURNAL OF PSYCHIATRY. 2001;158(11):1864-1870.

4988. Tondo L., Pinna M., Serra G., De Chiara L., Baldessarini R. Age at menarche predicts age at onset of major affective and anxiety disorders. EUROPEAN PSYCHIATRY. 2017;39():80-85.

4989. Vanderkruik Rachel, Conte Isabella, Dimidjian Sona Fat talk frequency in high school women: Changes associated with participation in the Body Project. BODY IMAGE. 2020;34():196-200.

4990. Triyaniarta Adinda, Martini Santi, Artanti Kurnia, Widati Sri, Nastiti Rizma Determinants of Type 2 Diabetes Mellitus among Passive Smokers. KESMAS-NATIONAL PUBLIC HEALTH JOURNAL. 2022;17(3):191-197.

4991. Varnado-Sullivan Paula, Horton Rachael Acceptability of programs for the prevention of eating Disorders. JOURNAL OF CLINICAL PSYCHOLOGY. 2006;62(6):687-703.

4992. Francisco Rita, Narciso Isabel, Alarcao Madalena Specific predictors of disordered eating among elite and non-elite gymnasts and ballet dancers. INTERNATIONAL JOURNAL OF SPORT PSYCHOLOGY. 2012;43(6):479-502.

4993. McCaig Duncan, Elliott Mark, Prnjak Katarina, Walasek Lukasz, Meyer Caroline Engagement with MyFitnessPal in eating disorders: Qualitative insights from online forums. INTERNATIONAL JOURNAL OF EATING DISORDERS. 2020;53(3):404-411.

4994. Steinhausen HC, Boyadjieva S The outcome of adolescent anorexia nervosa: Findings from Berlin and Sofia. JOURNAL OF YOUTH AND ADOLESCENCE. 1996;25(4):473-481.

4995. RICHARDT G, BRENN T, SEYFARTH M, HAASS M, SCHOMIG E, SCHOMIG A DUAL EFFECT OF NICOTINE ON CARDIAC NORADRENALINE RELEASE DURING METABOLIC BLOCKADE. BASIC RESEARCH IN CARDIOLOGY. 1994;89(6):524-534.

4996. Green Emalee, Venta Amanda Lack of implementation of eating disorder education and prevention programs in high schools: Data from incoming college freshmen. EATING DISORDERS. 2018;26(5):430-447.

4997. Juarez Ismael, Gratton Alan, Flores Gonzalo Ontogeny of altered dendritic morphology in the rat prefrontal cortex, hippocampus, and nucleus Accumbens following Cesarean delivery and birth anoxia. JOURNAL OF COMPARATIVE NEUROLOGY. 2008;507(5):1734-1747.

4998. Oumrait Nuria, Daivadanam Meena, Absetz Pilvikki, Guwatudde David, Berggreen-Clausen Aravinda, Alvesson Helle, De Man Jeroen, Annerstedt Kristi Can Self-Determination Explain Dietary Patterns Among Adults at Risk of or with Type 2 Diabetes? A Cross-Sectional Study in Socio-Economically Disadvantaged Areas in Stockholm. NUTRIENTS. 2020;12(3):.

4999. Wijnant Kathleen, Klosowska Joanna, Braet Caroline, Verbeken Sandra, De Henauw Stefaan, Vanhaecke Lynn, Michels Nathalie Stress Responsiveness and Emotional Eating Depend on Youngsters' Chronic Stress Level and Overweight. NUTRIENTS. 2021;13(10):.

5000. Oliver Tracy, Shenkman Rebecca, Mensinger Janell, Moore Caroline, Diewald Lisa A Study of United States Registered Dietitian Nutritionists during COVID-19: From Impact to Adaptation. NUTRIENTS. 2022;14(4):.

5001. Marzo-Castillejo Merce, Vela-Vallespin Carmen, Bellas-Beceiro Begona, Bartolome-Moreno Cruz, Gines-Diaz Yolanda, Melus-Palazon Elena PAPPS Cancer Expert Group. Cancer Prevention Recommendations. 2020 PAPPS update. ATENCION PRIMARIA. 2020;52(2):44-69.

5002. Tappauf M., Sudi K., Scheer P. Anorexia athletica and the female athlete triad in adolescents. MONATSSCHRIFT KINDERHEILKUNDE. 2007;155(9):815+.

5003. Lavan H, Johnson JG The association between axis I and II psychiatric symptoms and high-risk sexual behavior during adolescence. JOURNAL OF PERSONALITY DISORDERS.

2002;16(1):73-94.

5004. Lewis Hannah Implementing eating disorder prevention policy amidst Covid-19: Challenges and proposals for the future of mental health support teams. EUROPEAN EATING DISORDERS REVIEW. 2022;30(6):836-837.

5005. Haedt-Matt Alissa, Zalta Alyson, Forbush Kelsie, Keel Pamela Experimental evidence that changes in mood cause changes in body dissatisfaction among undergraduate women. BODY IMAGE. 2012;9(2):216-220.

5006. Daly Michael, Costigan Erin Trends in eating disorder risk among US college students, 2013-2021. PSYCHIATRY RESEARCH. 2022;317():.

5007. Atkinson Melissa Universal Eating Disorder Prevention: Results from a Cluster Randomised Controlled study of Teacher-led Secondary School Body Image Programmes. EUROPEAN EATING DISORDERS REVIEW. 2021;29(6):E14.

5008. Ostachowska-Gasior Agnieszka, Kolarzyk Emilia, Majewska Renata, Gasior Anna, Kwiatkowski Jacek, Zaleska Izabela Diet and Physical Activity as Determinants of Lifestyle Chosen by Women from Southern Poland. INTERNATIONAL JOURNAL OF ENVIRONMENTAL RESEARCH AND PUBLIC HEALTH. 2018;15(10):.

5009. Bruin A. Athletes with eating disorder symptomatology, a specific population with specific needs. CURRENT OPINION IN PSYCHOLOGY. 2017;16():148-153.

5010. Marza-Florensa Anna, Gutierrez Laura, Gulayin Pablo, Vaartjes Ilonca, Grobbee Diederick, Klipstein-Grobusch Kerstin, Irazola Vilma Risk factor clustering in men and women with CHD in the Southern Cone of Latin America. INTERNATIONAL JOURNAL OF CARDIOLOGY CARDIOVASCULAR RISK AND PREVENTION. 2023;16():.

5011. Oliveira Edilaine, Souza Barboza Stephanie We Do Not Want to Just Eat: a Social Marketing Approach to Healthy Eating. TEORIA E PRATICA EM ADMINISTRACAO-TPA. 2020;10(1):84-94.

5012. Gokani Nikhil, Garde Amandine, Philpott Matthew, Ireland Robin, Owens Rebecca, Boyland Emma UK Nutrition Research Partnership `Hot Topic' workshop report: A `game changer' for dietary health - addressing the implications of sport sponsorship by food

businesses through an innovative interdisciplinary collaboration. NUTRITION BULLETIN. 2022;47(1):115-122.

5013. Boggiss Anna, Consedine Nathan, Jefferies Craig, Bluth Karen, Hofman Paul, Serlachius Anna Protocol for a feasibility study: a brief self-compassion intervention for adolescents with type 1 diabetes and disordered eating. BMJ OPEN. 2020;10(2):.

5014. Sollid J, Kjernsli A, De Angelis PM, Rohr AK, Nilsson GE Cell proliferation and gill morphology in anoxic crucian carp. AMERICAN JOURNAL OF PHYSIOLOGY-REGULATORY INTEGRATIVE AND COMPARATIVE PHYSIOLOGY. 2005;289(4):R1196-R1201.

5015. McVey GL, Davis R, Kaplan AS, Katzman DK, Pinhas L, Geist R, Heinmaa M, Forsyth G A community-based training program for eating disorders and its contribution to a provincial network of specialized services. INTERNATIONAL JOURNAL OF EATING DISORDERS. 2005;37(S):S35-S40.

5016. Prasad Kavitha, Rajan Roshni, Basker Mona, Mammen Priya, Reshmi Y. Clinical Profile of Adolescent Onset Anorexia Nervosa at a Tertiary Care Center. INDIAN PEDIATRICS. 2021;58(8):726-728.

5017. McAllister C., Whittington J., Holland A. Development of the eating behaviour in Prader-Willi Syndrome: advances in our understanding. INTERNATIONAL JOURNAL OF OBESITY. 2011;35(2):188-197.

5018. Mateos-Padorno C., Scoffier S., Polifrone M., Martinez-Patino M., Martinez-Vidal A., Zagalaz Sanchez M. Analysis of eating disorders among 12-17 year-old adolescents in the island of Gran Canaria. EATING AND WEIGHT DISORDERS-STUDIES ON ANOREXIA BULIMIA AND OBESITY. 2010;15(3):E190-E194.

5019. Favaro A, Tenconi E, Santonastaso P Perinatal factors and the risk of developing anorexia nervosa and bulimia nervosa. ARCHIVES OF GENERAL PSYCHIATRY. 2006;63(1):82-88.

5020. Clark Olivia, Lee Matthew, Jingree Muksha, O'Dwyer Erin, Yue Yiyang, Marrero Abrania, Tamez Martha, Bhupathiraju Shilpa, Mattei Josiemer Weight Stigma and Social Media: Evidence and Public Health Solutions. FRONTIERS IN NUTRITION. 2021;8():.

5021. Coffin Michael, Knysh Kyle, Theriault Emma, Pater Christina, Courtenay Simon, Heuvel Michael Are floating algal mats a refuge from hypoxia for estuarine invertebrates?. PEERJ. 2017;5():.

5022. Fan Jian-Gao, Cao Hai-Xia Role of diet and nutritional management in non-alcoholic fatty liver disease. JOURNAL OF GASTROENTEROLOGY AND HEPATOLOGY. 2013;28(4, SI):81-87.

5023. Trepal Heather, Boie Ioana, Kress Victoria A Relational Cultural Approach to Working With Clients With Eating Disorders. JOURNAL OF COUNSELING AND DEVELOPMENT. 2012;90(3):346-356.

5024. Datta Nandini, Bidopia Tatyana, Datta Samir, Mittal Gaurie, Alphin Franca, Marsh Elizabeth, Fitzsimons Gavan, Strauman Timothy, Zucker Nancy Meal skipping and cognition along a spectrum of restrictive eating. EATING BEHAVIORS. 2020;39():.

5025. Laccetti Roberta, Pota Andrea, Stranges Saverio, Falconi Claudio, Memoli Bruno, Bardaro Leopoldo, Guida Bruna Evidence on the prevalence and geographic distribution of major cardiovascular risk factors in Italy. PUBLIC HEALTH NUTRITION. 2013;16(2):305-315.

5026. Sisson Susan, Shay Christina, Broyles Stephanie, Leyva Misti Television-Viewing Time and Dietary Quality Among US Children and Adults. AMERICAN JOURNAL OF PREVENTIVE MEDICINE. 2012;43(2):196-200.

5027. HARWOOD P, NEWTON T DENTAL ASPECTS OF BULIMIA-NERVOSA - IMPLICATIONS FOR THE HEALTH-CARE TEAM. EUROPEAN EATING DISORDERS REVIEW. 1995;3(2):93-102.

5028. Penttinen OP, Kukkonen JVK Metabolic response of Lumbriculus variegatus to respiratory uncoupler in cold and anoxic water. ENVIRONMENTAL TOXICOLOGY AND CHEMISTRY. 2000;19(8):2073-2075.

5029. Taylor Katherine, Jones Megan, Kass Andrea, Bailey Jakki, Genkin Brooke, Rizk Marianne, Shorter Alivia, Redman Morgan, Romer Paige, Williams Joanne, Trockel Mickey, Wilfley Denise, Taylor C. STAYING FIT: A PILOT STUDY OF A SCHOOL-BASED ONLINE UNIVERSAL AND TARGETED HEALTHY WEIGHT REGULATION/EATING DISORDER

PREVENTION PROGRAM. ANNALS OF BEHAVIORAL MEDICINE. 2012;43(1):S28.

5030. Robinette Timothy, Nicholatos Justin, Francisco Adam, Brooks Kayla, Diao Rachel, Sorbi Sandro, Ricca Valdo, Nacmias Benedetta, Brieno-Enriquez Miguel, Libert Sergiy SIRT1 accelerates the progression of activity-based anorexia. NATURE COMMUNICATIONS. 2020;11(1):.

5031. Dumitrascu Mihai, Sandru Florica, Carsote Mara, Petca Razvan, Gheorghisan-galateanu Ancuta, Petca Aida, Valea Ana Anorexia nervosa: COVID-19 pandemic period (Review). EXPERIMENTAL AND THERAPEUTIC MEDICINE. 2021;22(2):.

5032. Zhang Yang, Zhang Tao, Yang Wenbo, Chen Hongze, Geng Xinglong, Li Guanqun, Chen Hua, Wang Yongwei, Li Le, Sun Bei Beneficial Diets and Pancreatic Cancer: Molecular Mechanisms and Clinical Practice. FRONTIERS IN ONCOLOGY. 2021;11():.

5033. Zucoloto Moises, Antoniolli Lucimara, De Siqueira Dalmo, Costa Czermainski Ana ALTERNATIVE FOR REDUCING PHYSIOLOGICAL DISORDERS IN `BARTLETT' PEARS. REVISTA BRASILEIRA DE FRUTICULTURA. 2016;38(2):.

5034. Shafran Roz Promises, progress, and pathos: Commentary on ``treatment and prevention{''} papers by stice \& becker, hay, and mitchell, roenig \& steffan. INTERNATIONAL JOURNAL OF EATING DISORDERS. 2013;46(5, SI):486-488.

5035. Zanna Valeria, Criscuolo Michela, Mereu Alberta, Cinelli Giulia, Marchetto Chiara, Pasqualetti Patrizio, Tozzi Alberto, Castiglioni Maria, Chianello Ilenia, Vicari Stefano Restrictive eating disorders in children and adolescents: a comparison between clinical and psychopathological profiles. EATING AND WEIGHT DISORDERS-STUDIES ON ANOREXIA BULIMIA AND OBESITY. 2021;26(5):1491-1501.

5036. Pickens Charles, Cifani Carlo, Navarre Brittany, Eichenbaum Hila, Theberge Florence, Baumann Michael, Calu Donna, Shaham Yavin Effect of fenfluramine on reinstatement of food seeking in female and male rats: implications for the predictive validity of the reinstatement model. PSYCHOPHARMACOLOGY. 2012;221(2):341-353.

5037. Naja Farah, Itani Leila, Nasrallah Mona, Chami Hassan, Tamim Hani, Nasreddine Lara A healthy lifestyle pattern is associated with a metabolically healthy phenotype in overweight and obese adults: a cross-sectional study. EUROPEAN JOURNAL OF NUTRITION.

2020;59(5):2145-2158.

5038. Lobera-Salvatierra Esmeralda, Fanlo-Arnal Sonia, Mongio-Pardo Belen, Otin-Guarga Nieves, Vizcaino-Bricio Blanca, Garcia-Moyano Loreto Analysis of the risk of suffering eating disorders in adolescents. REVISTA ROL DE ENFERMERIA. 2019;42(6):436-444.

5039. Treasure Janet, Zipfel Stephan, Micali Nadia, Wade Tracey, Stice Eric, Claudino Angelica, Schmidt Ulrike, Frank Guido, Bulik Cynthia, Wentz Elisabet Anorexia nervosa. NATURE REVIEWS DISEASE PRIMERS. 2015;1():.

5040. Soussignan Robert, Schaal Benoist, Rigaud Daniel, Royet Jean-Pierre, Jiang Tao Hedonic reactivity to visual and olfactory cues: Rapid facial electromyographic reactions are altered in anorexia nervosa. BIOLOGICAL PSYCHOLOGY. 2011;86(3):265-272.

5041. Kluckow Hannah, Telfer James, Abraham Suzanne Should We Screen for Misophonia in Patients with Eating Disorders? A Report of Three Cases. INTERNATIONAL JOURNAL OF EATING DISORDERS. 2014;47(5):558-561.

5042. Lepe-Salazar Francisco, Salgado-Torres Sarita Multiple Composite Scenarios: A Game-Based Methodology for the Prevention of Mental Disorders. ENTERTAINMENT COMPUTING. 2023;44():.

5043. Kerr Jessica, Gillespie Alanna, Gasser Constantine, Mensah Fiona, Burgner David, Wake Melissa Childhood dietary trajectories and adolescent cardiovascular phenotypes: Australian community-based longitudinal study. PUBLIC HEALTH NUTRITION. 2018;21(14):2642-2653.

5044. Rodrigue Christopher, Iceta Sylvain, Begin Catherine Food Addiction and Cognitive Functioning: What Happens in Adolescents?. NUTRIENTS. 2020;12(12):.

5045. Martin-Lopez Rocio, Hernandez-Barrera Valentin, Lopez De Andres Ana, Carrasco Garrido Pilar, Gil De Miguel Angel, Jimenez Garcia Rodrigo Breast and cervical cancer screening in Spain and predictors of adherence. EUROPEAN JOURNAL OF CANCER PREVENTION. 2010;19(3):239-245.

5046. Williamson DA, Thaw JM, Varnado-Sullivan PJ Cost-effectiveness analysis of a hospital-based cognitive-behavioral treatment program for eating disorders. BEHAVIOR

THERAPY. 2001;32(3):459-477.

5047. Rastam Maria, Taljemark Jakob, Tajnia Armin, Lundstrom Sebastian, Gustafsson Peik, Lichtenstein Paul, Gillberg Christopher, Anckarsater Henrik, Kerekes Nora Eating Problems and Overlap with ADHD and Autism Spectrum Disorders in a Nationwide Twin Study of 9- and 12-Year-Old Children. SCIENTIFIC WORLD JOURNAL. 2013;():.

5048. Oliveira Sara, Pires Claudia, Ferreira Claudia Does the recall of caregiver eating messages exacerbate the pathogenic impact of shame on eating and weight-related difficulties?. EATING AND WEIGHT DISORDERS-STUDIES ON ANOREXIA BULIMIA AND OBESITY. 2020;25(2):471-480.

5049. Trinh Nhung, Semark Birgitte, Munk-Olsen Trine, Liu Xiaoqin, Ro Oyvind, Bulik Cynthia, Torgersen Leila, Lupattelli Angela, Petersen Liselotte Psychiatric visits during the postpartum year in women with eating disorders who continue or discontinue antidepressant treatment in pregnancy. INTERNATIONAL JOURNAL OF EATING DISORDERS. 2023;56(3):582-594.

5050. Forero-Bogota Monica, Ojeda-Pardo Monica, Garcia-Hermoso Antonio, Correa-Bautista Jorge, Gonzalez-Jimenez Emilio, Schmidt-Rio Valle Jacqueline, Navarro-Perez Carmen, Gracia-Marco Luis, Vlachopoulos Dimitris, Martinez-Torres Javier, Ramirez-Velez Robinson Body Composition, Nutritional Profile and Muscular Fitness Affect Bone Health in a Sample of Schoolchildren from Colombia: The Fuprecol Study. NUTRIENTS. 2017;9(2):.

5051. Liveri Kyriaki, Dagla Maria, Sarantaki Antigoni, Orovou Eirini, Antoniou Evangelia Abuse of Girls During Childhood and Its Impacts on the Health of Their Adult Lives: A Systematic Review. CUREUS JOURNAL OF MEDICAL SCIENCE. 2023;15(2):.

5052. Forrester-Knauss Christine, Perren Sonja, Alsaker Francoise Does body mass index in childhood predict restraint eating in early adolescence?. APPETITE. 2012;59(3):921-926.

5053. Langmesser Lisa, Verscheure Susan Are Eating Disorder Prevention Programs Effective?. JOURNAL OF ATHLETIC TRAINING. 2009;44(3):304-305.

5054. Bulzacka E., Lavault S., Pelissolo A., Isnard C. Mindful neuropsychology: Mindfulness-based cognitive remediadon. ENCEPHALE-REVUE DE PSYCHIATRIE CLINIQUE BIOLOGIQUE ET THERAPEUTIQUE. 2018;44(1):75-82.

5055. Cardel Michelle, Newsome Faith, Pearl Rebecca, Ross Kathryn, Dillard Julia, Miller Darci, Hayes Jacqueline, Wilfley Denise, Keel Pamela, Dhurandhar Emily, Balantekin Katherine Patient-Centered Care for Obesity: How Health Care Providers Can Treat Obesity While Actively Addressing Weight Stigma and Eating Disorder Risk. JOURNAL OF THE ACADEMY OF NUTRITION AND DIETETICS. 2022;122(6):1089-1098.

5056. Kwag Kyung, Han Soo, Cho Ji-Yeoun, Ko Myeong, Park Eun, Kim Youl-Ri A school-based eating disorder prevention program (Me, You \& Us) for young adolescents in Korea: A 3-year follow-up study. ASIA-PACIFIC PSYCHIATRY. 2021;13(1, SI):.

5057. Ghadiri Marzieh, Cheshmazar Elhameh, Shateri Zainab, Gerami Shirin, Nouri Mehran, Gargari Bahram Healthy plant-based diet index as a determinant of bone mineral density in osteoporotic postmenopausal women: A case-control study. FRONTIERS IN NUTRITION. 2023;9():.

5058. Rondanelli Mariangela, Opizzi Annalisa, Solerte Sebastiano, Trotti Rosita, Klersy Catherine, Cazzola Roberta Administration of a dietary supplement (N-oleyl-phosphatidylethanolamine and epigallocatechin-3-gallate formula) enhances compliance with diet in healthy overweight subjects: a randomized controlled trial. BRITISH JOURNAL OF NUTRITION. 2009;101(3):457-464.

5059. Kotler LA, Walsh BT Eating disorders in children and adolescents: pharmacological therapies. EUROPEAN CHILD \& ADOLESCENT PSYCHIATRY. 2000;9(1):108-116.

5060. Arouca Aline, Moreno Luis, Gonzalez-Gil Esther, Marcos Ascension, Widhalm Kurt, Molnar Denes, Manios Yannis, Gottrand Frederic, Kafatos Anthony, Kersting Mathilde, Sjostrom Michael, Amaro-Gahete Francisco, Ferrari Marika, Huybrechts Inge, Gonzalez-Gross Marcela, De Henauw Stefaan, Michels Nathalie Diet as moderator in the association of adiposity with inflammatory biomarkers among adolescents in the HELENA study. EUROPEAN JOURNAL OF NUTRITION. 2019;58(5):1947-1960.

5061. Chandler Genevieve, Roberts Susan, Chiodo Lisa Resilience Intervention for Young Adults With Adverse Childhood Experiences. JOURNAL OF THE AMERICAN PSYCHIATRIC NURSES ASSOCIATION. 2015;21(6):406-416.

5062. Forester Glen, Schaefer Lauren, Dodd Dorian, Johnson Jeffrey The potential application of event-related potentials to enhance research on reward processes in eating disorders. INTERNATIONAL JOURNAL OF EATING DISORDERS. 2022;55(11):1484-1495.

5063. Santaularia Jeanie, Johnson Monica, Hart Laurie, Haskett Lori, Welsh Ericka, Faseru Babalola Relationships between sexual violence and chronic disease: a cross-sectional study. BMC PUBLIC HEALTH. 2014;14():.

5064. Del-Ponte Bianca, Quinte Gabriela, Cruz Suelen, Grellert Merlen, Santos Ina Dietary patterns and attention deficit/hyperactivity disorder (ADHD): A systematic review and meta-analysis. JOURNAL OF AFFECTIVE DISORDERS. 2019;252():160-173.

5065. Smith Chery, Klosterbuer Abby, Levine Allen Military experience strongly influences post-service eating behavior and BMI status in American veterans. APPETITE. 2009;52(2):280-289.

5066. Lambrinou Christina-Paulina, Stralen Maartje, Androutsos Odysseas, Cardon Greet, De Craemer Marieke, Iotova Violeta, Socha Piotr, Koletzko Berthold, Moreno Luis, Manios Yannis, Grp ToyBox-Study Mediators of the effectiveness of a kindergarten-based, family-involved intervention on pre-schoolers' snacking behaviour: the ToyBox-study. PUBLIC HEALTH NUTRITION. 2019;22(1):157-163.

5067. Altunbasak Sakir, Incecik Faruk, Herguner Ozlem, Burgut H. Prognosis of patients with seizures occurring in the first 2 years. JOURNAL OF CHILD NEUROLOGY. 2007;22(3):307-313.

5068. Needham Lisa, Dwyer John, Randall-Simpson Janis, Heeney Elizabeth Supporting healthy eating among preschoolers: Challenges for child care staff. CANADIAN JOURNAL OF DIETETIC PRACTICE AND RESEARCH. 2007;68(2):107-110.

5069. Wells Kimberley, Jeacocke Nikki, Appaneal Renee, Smith Hilary, Vlahovich Nicole, Burke Louise, Hughes David The Australian Institute of Sport (AIS) and National Eating Disorders Collaboration (NEDC) position statement on disordered eating in high performance sport. BRITISH JOURNAL OF SPORTS MEDICINE. 2020;54(21):1247+.

5070. Coniglio Kathryn, Farris Samantha Treatment of Comorbid Pica and Generalized Anxiety Disorder: A Case Study. COGNITIVE AND BEHAVIORAL PRACTICE. 2021;28(3):410-421.

5071. Radix Anne, Rinck Mike, Becker Eni, Legenbauer Tanja The Mediating Effect of Specific Social Anxiety Facets on Body Checking and Avoidance. FRONTIERS IN

PSYCHOLOGY. 2019;9():.

5072. Murray Stuart Updates in the treatment of eating disorders in 2018: A year in review in eating disorders: The Journal of Treatment \& Prevention. EATING DISORDERS. 2019;27(1):6-17.

5073. Koushiou Maria, Kapatais Alexandros, Iasonidou Eleni, Adonis Marios, Ferreira Nuno The moderating role of body image inflexibility in the relation between weight concerns and symptoms of eating disorders in Cypriot University students. MEDITERRANEAN JOURNAL OF CLINICAL PSYCHOLOGY. 2021;9(2):.

5074. Abraham S, Lovell N Research and clinical assessment of eating and exercise behaviour. HOSPITAL MEDICINE. 1999;60(7):481-485.

5075. Delahanty Linda, Peyrot Mark, Shrader Peter, Williamson Donald, Meigs James, Nathan David, Grp DPP Pretreatment, Psychological, and Behavioral Predictors of Weight Outcomes Among Lifestyle Intervention Participants in the Diabetes Prevention Program (DPP). DIABETES CARE. 2013;36(1):34-40.

5076. Pandey Rahul, Heeger Sebastian, Lehner Christian Rapid effects of acute anoxia on spindle kinetochore interactions activate the mitotic spindle checkpoint. JOURNAL OF CELL SCIENCE. 2007;120(16):2807-2818.

5077. Ahlich Erica, Rancourt Diana Boredom proneness, interoception, and emotional eating. APPETITE. 2022;178():.

5078. Kipman A, Bruins-Slot L, Boni C, Hanoun N, Ades J, Blot P, Hamon M, Mouren-Simeoni MC, Gorwood P 5-HT2A gene promoter polymorphism as a modifying rather than a vulnerability factor in anorexia nervosa. EUROPEAN PSYCHIATRY. 2002;17(4):227-229.

5079. Janas-Kozik Malgorzata, Zejda Jan, Stochel Martyna, Brozek Grzegorz, Janas Adam, Jelonek Ireneusz Orthorexia - a new diagnosis?. PSYCHIATRIA POLSKA. 2012;46(3):441-450.

5080. Sanchez-Johnsen LAP, Fitzgibbon ML, Martinovich Z, Stolley MR, Dyer AR, Van Horn L Ethnic differences in correlates of obesity between Latin-American and black women.

OBESITY RESEARCH. 2004;12(4):652-660.

5081. Tantillo Mary, Starr Taylor, Kreipe Richard The recruitment and acceptability of a project ECHO (R) eating disorders clinic: a pilot study of telementoring for primary medical and behavioral health care practitioners. EATING DISORDERS. 2020;28(3):230-255.

5082. Mensinger Janell, Granche Janeway, Cox Shelbi, Henretty Jennifer Sexual and gender minority individuals report higher rates of abuse and more severe eating disorder symptoms than cisgender heterosexual individuals at admission to eating disorder treatment. INTERNATIONAL JOURNAL OF EATING DISORDERS. 2020;53(4):541-554.

5083. Hawrysh Peter, Miles Ashley, Buck Leslie Phosphorylation of the mitochondrial ATP-sensitive potassium channel occurs independently of PKC epsilon in turtle brain. COMPARATIVE BIOCHEMISTRY AND PHYSIOLOGY B-BIOCHEMISTRY \& MOLECULAR BIOLOGY. 2016;200():44-53.

5084. Martin JR, Bos M, Jenck F, Moreau JL, Mutel V, Sleight AJ, Wichmann J, Andrews JS, Berendsen HHG, Broekkamp CLE, Ruigt GSF, Kohler C, Delft AML 5-HT2C receptor agonists: Pharmacological characteristics and therapeutic potential. JOURNAL OF PHARMACOLOGY AND EXPERIMENTAL THERAPEUTICS. 1998;286(2):913-924.

5085. Garcia Dantas Ana, Amado Alonso Diana, Antonio Sanchez-Miguel Pedro, Rio Sanchez Carmen Factors Dancers Associate with their Body Dissatisfaction. BODY IMAGE. 2018;25():40-47.

5086. Michels Nathalie Poor Mental Health Is Related to Excess Weight via Lifestyle: A Cross-Sectional Gender- and Age-Dependent Mediation Analysis. NUTRIENTS. 2021;13(2):.

5087. Geisbuesch C., Buehren K. Eating disorders with diabetes mellitus. MONATSSCHRIFT KINDERHEILKUNDE. 2015;163(7):696-700.

5088. Kim Dennis, Yu Joanna, Mui Ryan, Niibori Rieko, Bin Taufique Hamza, Aslam Rukhsana, Semple John, Cordes Sabine The tyrosine kinase receptor Tyro3 enhances lifespan and neuropeptide Y (Npy) neuron survival in the mouse anorexia (anx) mutation. DISEASE MODELS \& MECHANISMS. 2017;10(5):581-595.

5089. Gonzalez-Chica David, Licinio Julio, Musker Michael, Wong Mali, Bowden Jacqueline, Hay Phillipa, Chittleborough Catherine, Stocks Nigel Bullying and sexual abuse and their association with harmful behaviours, antidepressant use and health-related quality of life in adulthood: a population-based study in South Australia. BMC PUBLIC HEALTH. 2019;19():.

5090. Akasheva Dariga, Drapkina Oxana Mediterranean Diet: Origin History, Main Components, Evidence of Benefits and Feasibility to Adapt to the Russian Reality. RATIONAL PHARMACOTHERAPY IN CARDIOLOGY. 2020;16(2):307-316.

5091. Francis Lori, Ventura Alison, Marini Michele, Birch Leann Parent overweight predicts daughters' increase in BMI and disinhibited overeating from 5 to 13 years. OBESITY. 2007;15(6):1544-1553.

5092. Wilfley Denise, Vannucci Anna, White Emily Early Intervention of Eating- and Weight-Related Problems. JOURNAL OF CLINICAL PSYCHOLOGY IN MEDICAL SETTINGS. 2010;17(4):285-300.

5093. Rageliene Tija, Aschemann-Witzel Jessica, Gronhoj Alice Efficacy of a smartphone application-based intervention for encouraging children's healthy eating in Denmark. HEALTH PROMOTION INTERNATIONAL. 2022;37(1):.

5094. Zeeni Nadine, Doumit Rita, Abi Kharma Joelle, Sanchez-Ruiz Maria-Jose Media, Technology Use, and Attitudes: Associations With Physical and Mental Well-Being in Youth With Implications for Evidence-Based Practice. WORLDVIEWS ON EVIDENCE-BASED NURSING. 2018;15(4):304-312.

5095. Chen Ying, Kawachi Ichiro, Berkman Lisa, Trudel-Fitzgerald Claudia, Kubzansky Laura A Prospective Study of Marital Quality and Body Weight in Midlife. HEALTH PSYCHOLOGY. 2018;37(3):247-256.

5096. Huq E, Hodges TK An anaerobically inducible early (aie) gene family from rice. PLANT MOLECULAR BIOLOGY. 1999;40(4):591-601.

5097. Li Shuang, Halen Cor, Baaren Rick, Mueller Barbara Self-Persuasion Increases Healthy Eating Intention Depending on Cultural Background. INTERNATIONAL JOURNAL OF ENVIRONMENTAL RESEARCH AND PUBLIC HEALTH. 2020;17(10):.

5098. Manolis Antonis, Manolis Theodora, Manolis Antonis, Melita Helen Diet and Sudden Death: How to Reduce the Risk. CURRENT VASCULAR PHARMACOLOGY. 2022;20(5):383-408.

5099. Ornstein Rollyn, Essayli Jamal, Nicely Terri, Masciulli Emily, Lane-Loney Susan Treatment of avoidant/restrictive food intake disorder in a cohort of young patients in a partial hospitalization program for eating disorders. INTERNATIONAL JOURNAL OF EATING DISORDERS. 2017;50(9):1067-1074.

5100. Gomez Del Barrio Andres, Ruiz Guerrero Francisco, Benito Gonzalez Pilar, Perez Fernandez Marta, Sanchez Blanco Lucia, Losa Mugica Edurne, Calcedo Giraldo Gabriel, Gonzalez Gomez Jana A retrospective investigation of the prodromal stages of eating disorders and use of health services in young patients the year prior to the diagnosis. EARLY INTERVENTION IN PSYCHIATRY. 2022;16(2):162-167.

5101. Mazur Artur, Zachurzok Agnieszka, Baran Joanna, Deren Katarzyna, Luszczki Edyta, Weres Aneta, Wyszynska Justyna, Dylczyk Justyna, Szczudlik Ewa, Drozdz Dorota, Metelska Paulina, Brzezinski Michal, Koziol-Kozakowska Agnieszka, Matusik Pawel, Socha Piotr, Olszanecka-Gilianowicz Magdalena, Jackowska Teresa, Walczak Mieczyslaw, Peregud-Pogorzelski Jaroslaw, Tomiak Elzbieta, Wojci Malgorzata Childhood Obesity: Position Statement of Polish Society of Pediatrics, Polish Society for Pediatric Obesity, Polish Society of Pediatric Endocrinology and Diabetes, the College of Family Physicians in Poland and Polish Association for Study on Obesity. NUTRIENTS. 2022;14(18):.

5102. Biemelt S, Keetman U, Albrecht G Re-aeration following hypoxia or anoxia leads to activation of the antioxidative defense system in roots of wheat seedlings. PLANT PHYSIOLOGY. 1998;116(2):651-658.

5103. Dakanalis Antonios, Mentzelou Maria, Papadopoulou Souzana, Papandreou Dimitrios, Spanoudaki Maria, Vasios Georgios, Pavlidou Eleni, Mantzorou Maria, Giaginis Constantinos The Association of Emotional Eating with Overweight/Obesity, Depression, Anxiety/Stress, and Dietary Patterns: A Review of the Current Clinical Evidence. NUTRIENTS. 2023;15(5):.

5104. Francisco Rita, Alarcao Madalena, Narciso Isabel Aesthetic Sports as High-Risk Contexts for Eating Disorders - Young Elite Dancers and Gymnasts Perspectives. SPANISH JOURNAL OF PSYCHOLOGY. 2012;15(1):265-274.

5105. Pedersen Carsten, Mors Ole, Bertelsen Aksel, Waltoft Berit, Agerbo Esben, McGrath John, Mortensen Preben, Eaton William A Comprehensive Nationwide Study of the

Incidence Rate and Lifetime Risk for Treated Mental Disorders. JAMA PSYCHIATRY. 2014;71(5):573-581.

5106. Panagiotakos Demosthenes, Pitsavos Christos, Chrysohoou Christina, Vlismas Konstantinos, Skoumas Yannis, Palliou Konstantina, Stefanadis Christodoulos The effect of clinical characteristics and dietary habits on the relationship between education status and 5-year incidence of cardiovascular disease: the ATTICA study. EUROPEAN JOURNAL OF NUTRITION. 2008;47(5):258-265.

5107. Scharff Adela, Ortiz Shelby, Forrest Lauren, Smith April Comparing the clinical presentation of eating disorder patients with and without trauma history and/or comorbid PTSD. EATING DISORDERS. 2021;29(1):88-102.

5108. Richards Margaret, Banez Gerard, Dohil Ranjan, Stein Martin Chronic constipation, atypical eating pattern, weight loss, and anxiety in a 19-year old youth. JOURNAL OF DEVELOPMENTAL AND BEHAVIORAL PEDIATRICS. 2006;27(4):338-340.

5109. Phillips EL, Pratt HD Eating disorders in college. PEDIATRIC CLINICS OF NORTH AMERICA. 2005;52(1):85+.

5110. O'Dea JA, Abraham S Improving the body image, eating attitudes, and behaviors of young male and female adolescents: A new educational approach that focuses on self-esteem. INTERNATIONAL JOURNAL OF EATING DISORDERS. 2000;28(1):43-57.

5111. Stepanov Yu, Zavhorodnia N., Zavhorodnia O. FEATURES OF EATING BEHAVIOR IN CHILDREN WITH DIFFERENT FORMS OF NON-ALCOHOLIC FATTY LIVER DISEASE. MEDICAL PERSPECTIVES-MEDICNI PERSPEKTIVI. 2021;26(3):26-32.

5112. Becker Carolyn, Stice Eric From Efficacy to Effectiveness to Broad Implementation: Evolution of the Body Project. JOURNAL OF CONSULTING AND CLINICAL PSYCHOLOGY. 2017;85(8):767-782.

5113. Halliwell Emma, Diedrichs Phillippa Testing a Dissonance Body Image Intervention Among Young Girls. HEALTH PSYCHOLOGY. 2014;33(2):201-204.

5114. Moitra Panchali, Madan Jagmeet, Shaikh Nida Eating habits and sleep patterns of adolescents with depression symptoms in Mumbai, India. MATERNAL AND CHILD

NUTRITION. 2020;16(3, SI):.

5115. Farrell Clare, Shafran Roz, Lee Michelle Empirically evaluated treatments for body image disturbance: A review. EUROPEAN EATING DISORDERS REVIEW. 2006;14(5):289-300.

5116. Abdullatif Mona, AlAbady Kadhim, Altheeb Ayesha, Rishmawi Fidaa, Jaradat Hana, Farooq Sobya Prevalence of Overweight, Obesity, and Dietary Behaviors among Adolescents in Dubai Schools: A Complex Design Survey 2019. DUBAI MEDICAL JOURNAL. 2022;5(1):1-9.

5117. Ding Wengang, Guo Yueping, Cui Xiaoguang, Zhang Bing, Li Dongmei, Li Wenzhi Morphine-induced delayed pre-conditioning against anoxia/reoxygenation injury in pulmonary artery endothelial cells: The role of mitochondrial K-ATP channels. MOLECULAR MEDICINE REPORTS. 2016;13(1, B):1047-1053.

5118. Belizario Gabriel, Borges Junior Renato, Salvini Rogerio, Lafer Beny, Dias Rodrigo Predominant polarity classification and associated clinical variables in bipolar disorder: A machine learning approach. JOURNAL OF AFFECTIVE DISORDERS. 2019;245():279-282.

5119. Willett Walter Overview and perspective in human nutrition. ASIA PACIFIC JOURNAL OF CLINICAL NUTRITION. 2008;17(1):1-4.

5120. Liu Kiki, Chen Julie, Ng Michelle, Yeung Maegan, Bedford Laura, Lam Cindy How Does the Family Influence Adolescent Eating Habits in Terms of Knowledge, Attitudes and Practices? A Global Systematic Review of Qualitative Studies. NUTRIENTS. 2021;13(11):.

5121. Simon Yves Epidemiology and psychosocial risk factors in anorexia nervosa. NUTRITION CLINIQUE ET METABOLISME. 2007;21(4):137-142.

5122. Cook Won, Kerr William, Karriker-Jaffe Katherine, Li Libo, Lui Camillia, Greenfield Thomas Racial/Ethnic Variations in Clustered Risk Behaviors in the US. AMERICAN JOURNAL OF PREVENTIVE MEDICINE. 2020;58(1):E21-E29.

5123. Halvorsen Inger, Platou Dagmar, Hoiseth Arne Bone Mass Eight Years After Treatment for Adolescent-Onset Anorexia Nervosa. EUROPEAN EATING DISORDERS

REVIEW. 2012;20(5):386-392.

5124. Trojanowski Paige, Frietchen Rachel, Harvie Blair, Mehlenbeck Robyn, Fischer Sarah Internet-delivered eating disorders prevention program for adolescent girls with type 1 diabetes: Acceptable and feasible. PEDIATRIC DIABETES. 2022;23(7):1122-1132.

5125. Corning Alexandra, Heibel Haley Re-thinking eating disorder prevention: The case for prioritizing the promotion of healthy identity development. EATING DISORDERS. 2016;24(1, SI):106-113.

5126. Fichter Manfred, Quadflieg Norbert, Lindner Susanne Internet-based relapse prevention in anorexia nervosa - Long-term course in an intent-to-treat sample. ZEITSCHRIFT FUR PSYCHIATRIE PSYCHOLOGIE UND PSYCHOTHERAPIE. 2014;62(1):35-42.

5127. Nakhoul Tracy, Mina Anthony, Soufia Michel, Obeid Sahar, Hallit Souheil Restrained eating in Lebanese adolescents: scale validation and correlates. BMC PEDIATRICS. 2021;21(1):.

5128. Hofmann Stefan, Asnaani Anu, Vonk Imke, Sawyer Alice, Fang Angela The Efficacy of Cognitive Behavioral Therapy: A Review of Meta-analyses. COGNITIVE THERAPY AND RESEARCH. 2012;36(5):427-440.

5129. Hilger-Kolb Jennifer, Diehl Katharina `Oh God, I Have to Eat Something, But Where Can I Get Something Quickly?'-A Qualitative Interview Study on Barriers to Healthy Eating among University Students in Germany. NUTRIENTS. 2019;11(10):.

5130. Wilksch Simon Where Did Universal Eating Disorder Prevention Go?. EATING DISORDERS. 2014;22(2):184-192.

5131. Beck Amy, Iturralde Esti, Haya-Fisher Julissa, Kim Sarah, Keeton Victoria, Fernandez Alicia Barriers and facilitators to healthy eating among low-income Latino adolescents. APPETITE. 2019;138():215-222.

5132. Coccaro Emil Psychiatric comorbidity in Intermittent Explosive Disorder. JOURNAL OF PSYCHIATRIC RESEARCH. 2019;118():38-43.

5133. Awad Abdelmoneim, Al-Nafisi Hala Public knowledge of cardiovascular disease and its risk factors in Kuwait: a cross-sectional survey. BMC PUBLIC HEALTH. 2014;14():.

5134. Shaw Heather, Stice Eric The implementation of evidence-based eating disorder prevention programs. EATING DISORDERS. 2016;24(1, SI):71-78.

5135. Fischer Catherine, Luaute Jacques, Morlet Dominique Event-related potentials (MMN and novelty P3) in permanent vegetative or minimally conscious states. CLINICAL NEUROPHYSIOLOGY. 2010;121(7):1032-1042.

5136. Wolfenden Luke, Barnes Courtney, Jones Jannah, Finch Meghan, Wyse Rebecca, Kingsland Melanie, Tzelepis Flora, Grady Alice, Hodder Rebecca, Booth Debbie, Yoong Sze Strategies to improve the implementation of healthy eating, physical activity and obesity prevention policies, practices or programmes within childcare services. COCHRANE DATABASE OF SYSTEMATIC REVIEWS. 2020;(2):.

5137. Mercer Adrienne Obesity, battle of the bulge-policy behind change: Whose responsibility is it and who pays?. HEALTH EDUCATION JOURNAL. 2010;69(4):401-408.

5138. Snuggs Sarah, Houston-Price Carmel, Harvey Kate Healthy eating interventions delivered in the family home: A systematic review. APPETITE. 2019;140():114-133.

5139. Ferrari Alize, Santomauro Damian, Herrera Ana, Shadid Jamileh, Ashbaugh Charlie, Erskine Holly, Charlson Fiona, Degenhardt Louisa, Scott James, McGrath John, Allebeck Peter, Benjet Corina, Breitborde Nicholas, Brugha Traolach, Dai Xiaochen, Dandona Lalit, Dandona Rakhi, Fischer Florian, Haagsma Juanita, Maria Haro Josep, Kieling Christian, Knudsen Ann, Kumar G., Leung Janni, Majeed Azeem, Mitchell Philip, Moitra Modhurima, Mokdad Ali, Molokhia Mariam, Patten Scott, Patton George, Phillips Michael, Soriano Joan, Stein Dan, Stein Murray, Szoeke Cassandra, Naghavi Mohsen, Hay Simon, Murray Christopher, Vos Theo, Whiteford Harvey, Collabor GBD Global, regional, and national burden of 12 mental disorders in 204 countries and territories, 1990-2019: a systematic analysis for the Global Burden of Disease Study 2019. LANCET PSYCHIATRY. 2022;9(2):137-150.

5140. Kato-Noguchi H Hypoxic induction of anoxia tolerance in rice coleoptiles. PLANT PRODUCTION SCIENCE. 2002;5(3):211-214.

5141. Corcoran Rachael, Trainor Gemma, Robinson Ben The minority or the misunderstood? A young man's journey with anorexia nervosa. JOURNAL OF PSYCHIATRIC AND MENTAL HEALTH NURSING. 2021;28(5):760-772.

5142. Dimitratos Sarah, Swartz Johnna, Laugero Kevin Pathways of parental influence on adolescent diet and obesity: a psychological stress-focused perspective. NUTRITION REVIEWS. 2022;80(7):1800-1810.

5143. Artinian NT, Schim SM, Vander Wal JS, Nies MA Eating patterns and cardiovascular disease risk in a Detroit Mexican American population. PUBLIC HEALTH NURSING. 2004;21(5):425-434.

5144. Roordink Eline, Steenhuis Ingrid, Kroeze Willemieke, Hoekstra Trynke, Jacobs Nele, Stralen Maartje Social Environmental Predictors of Lapse in Dietary Behavior: An Ecological Momentary Assessment Study Amongst Dutch Adults Trying to Lose Weight. ANNALS OF BEHAVIORAL MEDICINE. 2023;():.

5145. Weigel Angelika, Gumz Antje, Kaestner Denise, Romer Georg, Wegscheider Karl, Loewe Bernd Prevention and Treatment of Eating Disorders: The Health Care Network Anorexia and Bulimia nervosa. PSYCHIATRISCHE PRAXIS. 2015;42(1):S30-S34.

5146. Alcaraz-Ibanez Manuel, Paterna Adrian, Griffiths Mark, Sicilia Alvaro Examining the role of social physique anxiety on the relationship between physical appearance comparisons and disordered eating symptoms among Spanish emerging adults. SCANDINAVIAN JOURNAL OF PSYCHOLOGY. 2020;61(6):803-808.

5147. Isomaa Rasmus, Isomaa Anna-Lisa And then what happened? A 5-year follow-up of eating disorder patients. NORDIC JOURNAL OF PSYCHIATRY. 2014;68(8):567-572.

5148. Hoelling Heike, Schlack Robert Eating disorders in adolescents - Results of the German Health Interview and Examination Survey for Children and Adolescents (KiGGS). ERNAHRUNGS UMSCHAU. 2007;54(9):514+.

5149. Townsend Rebecca, Logan Danielle, O'Neill Roisin, Prinelli Federica, Woodside Jayne, McEvoy Claire Whole Dietary Patterns, Cognitive Decline and Cognitive Disorders: A Systematic Review of Prospective and Intervention Studies. NUTRIENTS. 2023;15(2):.

5150. Perelman Hayley, Schwartz Natalie, Yeoward-Dodson Jennifer, Quinones Isabel, Murray Matthew, Dougherty Elizabeth, Townsel Raven, Arthur-Cameselle Jessyca, Haedt-Matt Alissa Reducing eating disorder risk among male athletes: A randomized controlled trial investigating the male athlete body project. INTERNATIONAL JOURNAL OF EATING DISORDERS. 2022;55(2):193-206.

5151. Taylor Julia, Gibson Donna Crisis on campus: Eating disorder intervention from a developmental-ecological perspective. JOURNAL OF AMERICAN COLLEGE HEALTH. 2016;64(3):251-255.

5152. Schuele Stephan, Bermeo Adriana, Alexopoulos Andreas, Burgess Richard Anoxia-ischemia: A mechanism of seizure termination in ictal asystole. EPILEPSIA. 2010;51(1):170-173.

5153. Stepien Adam, Salacinska Daria, Staszewski Jacek, Durka-Kesy Marta, Dobrogowski Jan Paroxysmal extreme pain disorder in family with c.3892G > T (p.Val1298Phe) in the SCN9A gene mutation - case report. BMC NEUROLOGY. 2020;20(1):.

5154. Tylka Tracy Refinement of the tripartite influence model for men: Dual body image pathways to body change behaviors. BODY IMAGE. 2011;8(3):199-207.

5155. Wehrly Sarah, Bonilla Chantal, Perez Marisol, Liew Jeffrey Controlling parental feeding practices and child body composition in ethnically and economically diverse preschool children. APPETITE. 2014;73():163-171.

5156. Friedman SS Girls in the 90s: A gender-based model for eating disorder prevention. PATIENT EDUCATION AND COUNSELING. 1998;33(3):217-224.

5157. Kitajima Tasuku, Otani Ryoko, Inoue Takeshi, Matsushima Naho, Matsubara Naoki, Sakuta Ryoichi Sensory processing in children and adolescents shortly after the onset of anorexia nervosa: a pilot study. BIOPSYCHOSOCIAL MEDICINE. 2022;16(1):.

5158. Duenas Disotuar Yunior, Murray Hurtado Mercedes, Rubio Morell Belen, Murjani Bharwani Hima, Jimenez Sosa Alejandro EATING DISORDERS IN PEDIATRIC AGE: A BOOM PATHOLOGY. NUTRICION HOSPITALARIA. 2015;32(5):2091-2097.

5159. Mathevon T, Rougier C, Ducher E, Pic D, Garcier JM, Schmidt J Acute abdominal dilatation, a serious complication in the case of anorexia nervosa. PRESSE MEDICALE. 2004;33(9, 1):601-603.

5160. Wang Xiaofen, Gao Hailiang, Xu Huilan Cluster Analysis of Unhealthy Lifestyles among Elderly Adults with Prediabetes: A Cross-Sectional Study in Rural China. DIABETES THERAPY. 2019;10(5):1935-1948.

5161. Guarin Maritza Associated variables with bipolar disorder risk in a sample of Colombian women with eating disorders. EUROPEAN EATING DISORDERS REVIEW. 2006;14(4):242-247.

5162. Whitelaw Melissa, Gilbertson Heather, Lee Katherine, Sawyer Susan Restrictive Eating Disorders Among Adolescent Inpatients. PEDIATRICS. 2014;134(3):E758-E764.

5163. Fertleman C., Ferrie C., Aicardi J., Bednarek N., Eeg-Olofsson O., Elmslie F., Griesemer D., Goutieres F., Kirkpatrick M., Malmros I., Pollitzer M., Rossiter M., Roulet-Perez E., Schubert R., Smith V., Testard H., Wong V., Stephenson J. Paroxysmal extreme pain disorder (previously familial rectal pain syndrome). NEUROLOGY. 2007;69(6):586-595.

5164. Thompson Alexandra, Petrie Trent, Tackett Bailey, Balcom Kayla, Watkins C. Eating disorder diagnosis and the female athlete: A longitudinal analysis from college sport to retirement. JOURNAL OF SCIENCE AND MEDICINE IN SPORT. 2021;24(6):531-535.

5165. Toledo Priscila, Lotufo-Neto Francisco, Verdeli Helen, Goulart Alessandra, Marques Andrea, Solis Ana, Wang Yuan-Pang Interpersonal psychotherapy for treatment of obesity: A systematic review and meta-analysis. JOURNAL OF AFFECTIVE DISORDERS. 2023;320():319-329.

5166. Notaras M., Hill R., Buuse M. The BDNF gene Val66Met polymorphism as a modifier of psychiatric disorder susceptibility: progress and controversy. MOLECULAR PSYCHIATRY. 2015;20(8):916-930.

5167. Tizazu Woinshet, Laillou Arnaud, Hailu Bayuh, Chitekwe Stanley, Baye Kaleab Complementary feeding and food-group level inequality among Ethiopian children 6-23 months of age (2011-2019). MATERNAL AND CHILD NUTRITION. 2022;():.

5168. Shaw Heather, Rohde Paul, Stice Eric Participant feedback from peer-led, clinician-led, and internet-delivered eating disorder prevention interventions. INTERNATIONAL JOURNAL OF EATING DISORDERS. 2016;49(12):1087-1092.

5169. Yalcin S., Tezol Ozlem, Caylan Nilgun, Nergiz Meryem, Yildiz Deniz, Cicek Seyma, Oflu Ayse Evaluation of problematic screen exposure in pre-schoolers using a unique tool called ``seven-in-seven screen exposure questionnaire{''}: cross-sectional study. BMC PEDIATRICS. 2021;21(1):.

5170. Steinhausen Hans-Christoph, Villumsen Martin, Horder Kirsten, Winkler Laura, Bilenberg Niels, Stoving Rene Increased risk of somatic diseases following anorexia nervosa in a controlled nationwide cohort study. INTERNATIONAL JOURNAL OF EATING DISORDERS. 2022;55(6):754-762.

5171. Fitzsimmons-Craft Ellen, Chan William, Smith Arielle, Firebaugh Marie-Laure, Fowler Lauren, Topooco Naira, DePietro Bianca, Wilfley Denise, Taylor C., Jacobson Nicholas Effectiveness of a chatbot for eating disorders prevention: A randomized clinical trial. INTERNATIONAL JOURNAL OF EATING DISORDERS. 2022;55(3):343-353.

5172. Haran Maeve, Killeen David, Healy Mike, Brophy Peadar, Donohue Aoife, Whyte Imelda, Doody Brendan Prevalence and correlates of restrictive interventions in an Irish child and adolescent psychiatric unit: a 4-year retrospective study. IRISH JOURNAL OF MEDICAL SCIENCE. 2023;():.

5173. Yazawa A., Shiba K., Hikichi H., Okuzono S., Aida J., Kondo K., Sasaki S., Kawachi I. Post-Disaster Mental Health and Dietary Patterns among Older Survivors of an Earthquake and Tsunami. JOURNAL OF NUTRITION HEALTH \& AGING. 2023;():.

5174. Ralph-Nearman Christina, Filik Ruth Development and validation of new figural scales for female body dissatisfaction assessment on two dimensions: thin-ideal and muscularity-ideal. BMC PUBLIC HEALTH. 2020;20(1):.

5175. Becker Carolyn, Bull Stephanie, Schaumberg Katherine, Cauble Adele, Franco Amanda Effectiveness of peer-led eating disorders prevention: A replication trial. JOURNAL OF CONSULTING AND CLINICAL PSYCHOLOGY. 2008;76(2):347-354.

5176. Hailes Helen, Yu Rongqin, Danese Andrea, Fazel Seena Long-term outcomes of childhood sexual abuse: an umbrella review. LANCET PSYCHIATRY. 2019;6(10):830-839.

5177. Zarger Melissa, Rich Brendan Predictors of treatment utilization among adolescents with social anxiety disorder. CHILDREN AND YOUTH SERVICES REVIEW. 2016;71():191-198.

5178. Kuitunen-Paul Soeren, Roessner Veit, Basedow Lukas, Golub Yulia Beyond the tip of the iceberg: A narrative review to identify research gaps on comorbid psychiatric disorders in adolescents with methamphetamine use disorder or chronic methamphetamine use. SUBSTANCE ABUSE. 2021;42(1):13-32.

5179. Sinclair Rachael, Millar Lynne, Allender Steven, Snowdon Wendy, Waqa Gade, Jacka Felice, Moodie Marj, Petersen Solveig, Swinburn Boyd The Cross-Sectional Association between Diet Quality and Depressive Symptomology amongst Fijian Adolescents. PLOS ONE. 2016;11(8):.

5180. Wentz Elisabet, Gillberg I., Anckarsater Henrik, Gillberg Christopher, Rastam Maria Reproduction and Offspring Status 18 years After Teenage-Onset Anorexia Nervosa - A Controlled Community-Based Study. INTERNATIONAL JOURNAL OF EATING DISORDERS. 2009;42(6):483-491.

5181. Luce KH, Osborne MI, Winzelberg AJ, Das S, Abascal LB, Celio AA, Wilfley DE, Stevenson D, Dev P, Taylor CB Application of an algorithm-driven protocol to simultaneously provide universal and targeted prevention programs. INTERNATIONAL JOURNAL OF EATING DISORDERS. 2005;37(3):220-226.

5182. Raj ABM Behaviour of pigs exposed to mixtures of gases and the time required to stun and kill them: welfare implications. VETERINARY RECORD. 1999;144(7):165-168.

5183. Dignard Nicole, Jarry Josee The ?Little Red Riding Hood effect:? Fitspiration is just as bad as thinspiration for women?s body satisfaction. BODY IMAGE. 2021;36():201-213.

5184. Bartoli Francesco, Callovini Tommaso, Cavaleri Daniele, Cioni Riccardo, Bachi Bianca, Calabrese Angela, Moretti Federico, Canestro Aurelia, Morreale Marco, Nasti Christian, Palpella Dario, Piacenti Susanna, Nacinovich Renata, Riboldi Ilaria, Crocamo Cristina, Carra Giuseppe Clinical correlates of comorbid attention deficit hyperactivity disorder in adults suffering from bipolar disorder: A meta-analysis. AUSTRALIAN AND NEW ZEALAND

JOURNAL OF PSYCHIATRY. 2023;57(1):34-48.

5185. Krom Hilde, Mameren Joost, Remijn Lianne, Nennie Katinka, Dumont Eric, Gaag Ellen, Leeuwen Marianne, Mulkens Sandra, Schakelaar Chantal, Kindermann Angelika Impact of COVID-19 Pandemic on Young Children With Feeding and Eating Problems and Disorders and Their Families. JOURNAL OF PEDIATRIC GASTROENTEROLOGY AND NUTRITION. 2022;75(4):529-534.

5186. Komatsu Hiroko, Nagamitsu Shinichiro, Ozono Shuichi, Yamashita Yushiro, Ishibashi Masatoshi, Matsuishi Toyojiro Regional cerebral blood flow changes in early-onset anorexia nervosa before and after weight gain. BRAIN \& DEVELOPMENT. 2010;32(8):625-630.

5187. Francesconi Marta, Flouri Eirini, Harrison Amy Decision-making difficulties mediate the association between poor emotion regulation and eating disorder symptoms in adolescence. PSYCHOLOGICAL MEDICINE. 2022;():.

5188. Bulc Mateja, Svab Igor, Godycki-Cwirko Maciek Factors that affect readiness to change lifestyle: A 22-country survey from primary care. EUROPEAN JOURNAL OF GENERAL PRACTICE. 2015;21(1):33-38.

5189. Raffoul Amanda, Leatherdale Scott, Kirkpatrick Sharon Dieting predicts engagement in multiple risky behaviours among adolescent Canadian girls: a longitudinal analysis. CANADIAN JOURNAL OF PUBLIC HEALTH-REVUE CANADIENNE DE SANTE PUBLIQUE. 2018;109(1):61-69.

5190. Munsch Simone Eating disorders - new challenges. ZEITSCHRIFT FUR PSYCHIATRIE PSYCHOLOGIE UND PSYCHOTHERAPIE. 2019;67(1):5-8.

5191. Wolff Elisabeth, Gaudlitz Katharina, Lindenberger Brigitt-Leila, Plag Jens, Heinz Andreas, Stroehle Andreas Exercise and physical activity in mental disorders. EUROPEAN ARCHIVES OF PSYCHIATRY AND CLINICAL NEUROSCIENCE. 2011;261(2):186-191.

5192. Smith Jamie, Serier Kelsey, Belon Katherine, Sebastian Riley, Smith Jane Evaluation of the relationships between dietary restraint, emotional eating, and intuitive eating moderated by sex. APPETITE. 2020;155():.

5193. McCarthy Mark, Cluzel Elodie, Dressel Kerstin, Newton Rachel Food and health research in Europe: Structures, gaps and futures. FOOD POLICY. 2013;39():64-71.

5194. Dong Debo, Wang Yulin, Jackson Todd, Chen Shuaiyu, Wang Yu, Zhou Feng, Chen Hong Impulse control and restrained eating among young women: Evidence for compensatory cortical activation during a chocolate-specific delayed discounting task. APPETITE. 2016;105():477-486.

5195. KATCHMAN AN, VICINI S, HERSHKOWITZ N MECHANISM OF EARLY ANOXIA-INDUCED SUPPRESSION OF THE GABA(A)-MEDIATED INHIBITORY POSTSYNAPTIC CURRENT. JOURNAL OF NEUROPHYSIOLOGY. 1994;71(3):1128-1138.

5196. Stice Eric, Presnell Katherine, Gau Jeff, Shaw Heather Testing mediators of intervention effects in randomized controlled trials: An evaluation of two eating disorder prevention programs. JOURNAL OF CONSULTING AND CLINICAL PSYCHOLOGY. 2007;75(1):20-32.

5197. Cong Ji-Yan, Zhao Yue, Xu Qun-Yan, Zhong Chun-De, Xing Qiu-Ling Health-related quality of life among Tianjin Chinese patients with type 2 diabetes: A cross-sectional survey. NURSING \& HEALTH SCIENCES. 2012;14(4, SI):528-534.

5198. Stahel Priska, Sud Shawn, Lee So, Jackson Timothy, Urbach David, Okrainec Allan, Allard Johane, Bassett Anne, Paterson Andrew, Sockalingam Sanjeev, Dash Satya Phenotypic and genetic analysis of an adult cohort with extreme obesity. INTERNATIONAL JOURNAL OF OBESITY. 2019;43(10):2057-2065.

5199. BONIECE IR, WAGNER JA GROWTH-FACTORS PROTECT PC12 CELLS AGAINST ISCHEMIA BY A MECHANISM THAT IS INDEPENDENT OF PKA, PKC, AND PROTEIN-SYNTHESIS. JOURNAL OF NEUROSCIENCE. 1993;13(10):4220-4228.

5200. Lin Ting-Ti, Park Chang, Kapella Mary, Martyn-Nemeth Pamela, Tussing-Humphreys Lisa, Rospenda Kathleen, Zenk Shannon Shift work relationships with same- and subsequent-day empty calorie food and beverage consumption. SCANDINAVIAN JOURNAL OF WORK ENVIRONMENT \& HEALTH. 2020;46(6):579-588.

5201. Serras Alisha, Saules Karen, Cranford James, Eisenberg Daniel Self-Injury, Substance Use, and Associated Risk Factors in a Multi-Campus Probability Sample of College Students.

PSYCHOLOGY OF ADDICTIVE BEHAVIORS. 2010;24(1):119-128.

5202. Sotodate Genichiro, Matsumoto Atsushi, Konishi Yu, Toya Yukiko, Endo Mikiya, Oyama Kotaro Fetal intracranial hemorrhage due to maternal subclinical vitamin K deficiency associated with long-term eating disorder. JOURNAL OF OBSTETRICS AND GYNAECOLOGY RESEARCH. 2019;45(2):461-465.

5203. Kato-Noguchi H Sugar utilization and anoxia tolerance in rice roots acclimated by hypoxic pretreatment. JOURNAL OF PLANT PHYSIOLOGY. 2004;161(7):803-808.

5204. Findlay Sheri, Toews Heather, Grant Christina Use of Gastrostomy Tubes in Children and Adolescents With Eating Disorders and Related Illnesses. JOURNAL OF ADOLESCENT HEALTH. 2011;48(6):625-629.

5205. Sanz Yolanda, Romani-Perez Marina, Benitez-Paez Alfonso, Portune Kevin, Brigidi Patrizia, Rampelli Simone, Dinan Ted, Stanton Catherine, Delzenne Nathalie, Blachier Francois, Neyrinck Audrey, Beaumont Martin, Olivares Marta, Holzer Peter, Guenther Kathrin, Wolters Maike, Ahrens Wolfgang, Claus Sandrine, Campoy Cristina, Murphy Rinki, Sadler Christina, Fernandez Laura, Kamp Jan-Willem Towards microbiome-informed dietary recommendations for promoting metabolic and mental health: Opinion papers of the MyNewGut project. CLINICAL NUTRITION. 2018;37(6, A):2191-2197.

5206. Bobadilla-Soto Paulina, Bugueno-Sierra Sophia, Guerrero-Jimenez Valentina, Munoz-Duran Maria, Zuniga-Coleman Jennifer, Nazar Gabriela Affective state, emotional regulation and eating styles among adults in Chile. REVISTA CHILENA DE NUTRICION. 2022;49(2):193-200.

5207. Pearce Christopher, Cohen Anthony, Coe Angela, Burton Kevin Molybdenum isotope evidence for global ocean anoxia coupled with perturbations to the carbon cycle during the early Jurassic. GEOLOGY. 2008;36(3):231-234.

5208. ROMEO F ADOLESCENT BOYS AND ANOREXIA-NERVOSA. ADOLESCENCE. 1994;29(115):643-647.

5209. Naylor P-J, Scott J., Drummond J., Bridgewater L., Mckay H., Panagiotopoulos C. Implementing a whole school physical activity and healthy eating model in rural and remote First Nations schools: a process evaluation of Action Schools! BC. RURAL AND REMOTE

HEALTH. 2010;10(2):.

5210. Arnold Charlotte, Johnson Hayley, Mahon Ciara, Agius Mark THE EFFECTS OF EATING DISORDERS IN PREGNANCY ON MOTHER AND BABY: A REVIEW. PSYCHIATRIA DANUBINA. 2019;31(3):S615-S618.

5211. Elderen T, Dusseldorp E Lifestyle effects of group health education for patients with coronary heart disease. PSYCHOLOGY \& HEALTH. 2001;16(3):327-341.

5212. Chowdhury U, Gordon I, Lask B, Watkins B, Watt H, Christie D Early-onset anorexia nervosa: Is there evidence limbic system imbalance?. INTERNATIONAL JOURNAL OF EATING DISORDERS. 2003;33(4):388-396.

5213. Hoste Renee, Labuschagne Zandre, Le Grange Daniel Adolescent Bulimia Nervosa. CURRENT PSYCHIATRY REPORTS. 2012;14(4):391-397.

5214. Camp-Spivey Logan, Newman Susan, Stevens Robert, Nichols Michelle Describing South Carolina Public School Administrators' Perceptions and Experiences Related to School-Based Interventions and Strategies to Promote Healthy Physical Activity and Eating Behaviors: A Qualitative Study. JOURNAL OF SCHOOL HEALTH. 2022;92(6):581-593.

5215. Francesconi M., Flouri E., Harrison A. Change in decision-making skills and risk for eating disorders in adolescence: A population-based study. EUROPEAN PSYCHIATRY. 2020;63(1):.

5216. Hao W, Takano T, Guillemette J, Papillon J, Ren GH, Cybulsky AV Induction of apoptosis by the Ste20-like kinase SLK, a germinal center kinase that activates apoptosis signal-regulating kinase and p38. JOURNAL OF BIOLOGICAL CHEMISTRY. 2006;281(6):3075-3084.

5217. Gabka Jakub Effect of different anaesthesia treatments on the onset of oviposition by virgin queen honey bees. JOURNAL OF APICULTURAL RESEARCH. 2023;():.

5218. Tommasi Marco, Sergi Maria, Konstantinidou Fani, Franzago Marica, Pesce Mirko, La Fratta Irene, Grilli Alfredo, Stuppia Liborio, Picconi Laura, Saggino Aristide, Gatta Valentina Association of COMT, BDNF and 5-HTT functional polymorphisms with personality

characteristics. FRONTIERS IN BIOSCIENCE-LANDMARK. 2021;26(11):1064-1074.

5219. Cecilia Castro Maria, Gonzalo Villagarcia Hernan, Laura Massa Maria, Francini Flavio Alpha-lipoic acid and its protective role in fructose induced endocrine-metabolic disturbances. FOOD \& FUNCTION. 2019;10(1):16-25.

5220. Lee Kirsty, Guy Alexa, Dale Jeremy, Wolke Dieter Does psychological functioning mediate the relationship between bullying involvement and weight loss preoccupation in adolescents? A two-stage cross-sectional study. INTERNATIONAL JOURNAL OF BEHAVIORAL NUTRITION AND PHYSICAL ACTIVITY. 2017;14():.

5221. Yanover T., Sacco W. Eating beyond satiety and body mass index. EATING AND WEIGHT DISORDERS-STUDIES ON ANOREXIA BULIMIA AND OBESITY. 2008;13(3):119-128.

5222. Parada Humberto, Sun Xuezheng, Tse Chiu-Kit, Olshan Andrew, Troester Melissa Lifestyle Patterns and Survival Following Breast Cancer in the Carolina Breast Cancer Study. EPIDEMIOLOGY. 2019;30(1):83-92.

5223. Reynes Barbara, Garcia-Ruiz Estefania, Diaz-Rua Ruben, Palou Andreu, Oliver Paula Reversion to a control balanced diet is able to restore body weight and to recover altered metabolic parameters in adult rats long-term fed on a cafeteria diet. FOOD RESEARCH INTERNATIONAL. 2014;64():839-848.

5224. Souza Santos Sthefany, Maciel Julia, Fagundos Andhressa, Ferreira Barbosa Kiriaque Risk behaviors for eating disorders in adolescents at a public school. MUNDO DA SAUDE. 2020;44(1):229-238.

5225. Marmara Joshua, Hosking Warwick, Lyons Anthony Body Image Disturbances as Predictors of Reduced Mental Health Among Australian Gay Men: Being in a Relationship Does Not Serve as a Protective Factor. ARCHIVES OF SEXUAL BEHAVIOR. 2018;47(8):2467-2479.

5226. Kask Jan, Ekselius Lisa, Brandt Lena, Kollia Natasa, Ekbom Anders, Papadopoulos Fotios Mortality in Women With Anorexia Nervosa: The Role of Comorbid Psychiatric Disorders. PSYCHOSOMATIC MEDICINE. 2016;78(8):910-919.

5227. Raevuori Anu, Hoek Hans, Susser Ezra, Kaprio Jaakko, Rissanen Aila, Keski-Rahkonen Anna Epidemiology of Anorexia Nervosa in Men: A Nationwide Study of Finnish Twins. PLOS ONE. 2009;4(2):.

5228. Del-Ponte Bianca, Anselmi Luciana, Assuncao Maria, Tovo-Rodrigues Luciana, Munhoz Tiago, Matijasevich Alicia, Rohde Luis, Santos Ina Sugar consumption and attention-deficit/hyperactivity disorder (ADHD): A birth cohort study. JOURNAL OF AFFECTIVE DISORDERS. 2019;243():290-296.

5229. Schoen Eva, Brock Rebecca, Hannon Jennifer Gender bias, other specified and unspecified feeding and eating disorders, and college students: a vignette study. EATING DISORDERS. 2019;27(3):291-304.

5230. Rose Kelsey, Negrete Christina, Sellinger Gina, Chang Tammy, Sonneville Kendrin Adolescent and emerging adult perceptions of eating disorder severity and stigma. INTERNATIONAL JOURNAL OF EATING DISORDERS. 2022;55(10):1296-1304.

5231. Godart NT, Flament MF, Perdereau F, Jeammet P Social disability in Anorexia nervosa and bulimia nervosa. ENCEPHALE-REVUE DE PSYCHIATRIE CLINIQUE BIOLOGIQUE ET THERAPEUTIQUE. 2003;29(2):149-156.

5232. Scholtens Salome, Brunekreef Bert, Smit Henriette, Gast Gerrie-Cor, Hoekstra Maarten, De Jongste Johan, Postma Dirkje, Gerritsen Jorrit, Seidell Jaap, Wijga Alet Do Differences in Childhood Diet Explain the Reduced Overweight Risk in Breastfed Children?. OBESITY. 2008;16(11):2498-2503.

5233. Schweren Lizanne, Larsson Henrik, Vinke Petra, Li Lin, Kvalvik Liv, Arias-Vasquez Alejandro, Haavik Jan, Hartman Catharina Diet quality, stress and common mental health problems: A cohort study of 121,008 adults. CLINICAL NUTRITION. 2021;40(3):901-906.

5234. Schneier FR, Blanco C, Antia SX, Liebowitz MR The social anxiety spectrum. PSYCHIATRIC CLINICS OF NORTH AMERICA. 2002;25(4):757+.

5235. Goodman Marianne, Banthin David, Blair Nicholas, Mascitelli Kathryn, Wilsnack Jaime, Chen Jennifer, Messenger Julie, Perez-Rodriguez M., Triebwasser Joseph, Koenigsberg Harold, Goetz Raymond, Hazlett Erin, New Antonia A Randomized Trial of Dialectical Behavior Therapy in High-Risk Suicidal Veterans. JOURNAL OF CLINICAL PSYCHIATRY.

2016;77(12):E1591-E1600.

5236. Wright F., Rodgers R. Low dose naloxone attenuates the pruritic but not anorectic response to rimonabant in male rats. PSYCHOPHARMACOLOGY. 2013;226(2):415-431.

5237. Itani Osamu, Kaneita Yoshitaka, Doi Kazuto, Tokiya Mikiko, Jike Maki, Nakagome Sachi, Otsuka Yuichiro, Ohida Takashi Longitudinal Epidemiologic Study of Poor Mental Health Status in Japanese Adolescents: Incidence of Predictive Lifestyle Factors. JOURNAL OF CLINICAL PSYCHIATRY. 2018;79(4):.

5238. Stefanova Eliska, Bakalar Peter, Baska Tibor Eating-Disordered Behavior in Adolescents: Associations with Body Image, Body Composition and Physical Activity. INTERNATIONAL JOURNAL OF ENVIRONMENTAL RESEARCH AND PUBLIC HEALTH. 2020;17(18):.

5239. Diarra A, Sheldon C, Brett CL, Baimbridge KG, Church J Anoxia-evoked intracellular pH and Ca2+ concentration changes in cultured postnatal rat hippocampal neurons. NEUROSCIENCE. 1999;93(3):1003-1016.

5240. Alonso Jordi, Jonge Peter, Lim Carmen, Aguilar-Gaxiola Sergio, Bruffaerts Ronny, Caldas-de-Almeida Jose, Liu Zhaorui, O'Neill Siobhan, Stein Dan, Viana Maria, Al-Hamzawi Ali, Angermeyer Matthias, Borges Guilherme, Ciutan Marius, Girolamo Giovanni, Fiestas Fabian, Maria Haro Josep, Hu Chiyi, Kessler Ronald, Lepine Jean, Levinson Daphna, Nakamura Yosikazu, Posada-Villa Jose, Wojtyniak Bogdan, Scott Kate Association between mental disorders and subsequent adult onset asthma. JOURNAL OF PSYCHIATRIC RESEARCH. 2014;59():179-188.

5241. West Caroline, Darling Katherine, Ruzicka Elizabeth, Sato Amy Household income and loss of control eating in adolescence: Examining the role of food insecurity. APPETITE. 2021;165():.

5242. Salvy Sarah-Jeanne, Haye Kayla, Bowker Julie, Hermans Roel Influence of peers and friends on children's and adolescents' eating and activity behaviors. PHYSIOLOGY \& BEHAVIOR. 2012;106(3, SI):369-378.

5243. Ornek Bahar, Gundogmus Ibrahim The Effects of Smartphone and Internet Gaming Addiction on Eating Attitudes Among University Students. PSYCHIATRY INVESTIGATION.

2022;19(1):1-8.

5244. ROSE UM, BINDELS RJM, VIS A, JANSEN JWCM, VANOS CH THE EFFECT OF L-TYPE CA2+ CHANNEL BLOCKERS ON ANOXIA-INDUCED INCREASES IN INTRACELLULAR CA2+ CONCENTRATION IN RABBIT PROXIMAL TUBULE CELLS IN PRIMARY CULTURE. PFLUGERS ARCHIV-EUROPEAN JOURNAL OF PHYSIOLOGY. 1993;423(5-6):378-386.

5245. Camplain Ricky, Lininger Monica, Baldwin Julie, Trotter Robert Cardiovascular Risk Factors among Individuals Incarcerated in an Arizona County Jail. INTERNATIONAL JOURNAL OF ENVIRONMENTAL RESEARCH AND PUBLIC HEALTH. 2021;18(13):.

5246. Jones Megan, Kass Andrea, Trockel Mickey, Glass Alan, Wilfley Denise, Taylor C. A Population-Wide Screening and Tailored Intervention Platform for Eating Disorders on College Campuses: The Healthy Body Image Program. JOURNAL OF AMERICAN COLLEGE HEALTH. 2014;62(5):351-356.

5247. Braet Caroline, Beyers Wim, Goossens Lien, Verbeken Sandra, Moens Ellen Subtyping Children and Adolescents Who Are Overweight Based on Eating Pathology and Psychopathology. EUROPEAN EATING DISORDERS REVIEW. 2012;20(4):279-286.

5248. Schafer Katherine, Lieberman Amy, Sever Anna, Joiner Thomas Prevalence rates of anxiety, depressive, and eating pathology symptoms between the pre- and peri-COVID-19 eras: A meta-analysis. JOURNAL OF AFFECTIVE DISORDERS. 2022;298(A):364-372.

5249. Alderman Sarah, Riggs Claire, Bullingham Oliver, Gillis Todd, Warren Daniel Cold acclimation induces life stage-specific responses in the cardiac proteome of western painted turtles (Chrysemys picta bellii): implications for anoxia tolerance. JOURNAL OF EXPERIMENTAL BIOLOGY. 2021;224(14):.

5250. Alsharairi Naser Current Government Actions and Potential Policy Options for Reducing Obesity in Queensland Schools. CHILDREN-BASEL. 2018;5(2):.

5251. Spagnuolo Maria, Pallottini Valentina, Mazzoli Arianna, Iannotta Lucia, Tonini Claudia, Morone Barbara, Stahlman Marcus, Crescenzo Raffaella, Strazzullo Maria, Iossa Susanna, Cigliano Luisa A Short-Term Western Diet Impairs Cholesterol Homeostasis and Key Players of Beta Amyloid Metabolism in Brain of Middle Aged Rats. MOLECULAR NUTRITION \& FOOD RESEARCH. 2020;64(16):.

5252. Kim Yongjoo, Austin S., Subramanian S., Thomas Jennifer, Eddy Kamryn, Franko Debra, Rodgers Rachel, Kawachi Ichiro Risk factors for disordered weight control behaviors among Korean adolescents: Multilevel analysis of the Korea Youth Risk Behavior Survey. INTERNATIONAL JOURNAL OF EATING DISORDERS. 2018;51(2):124-138.

5253. Katchman AN, Hershkowitz N Adenosine A(1) antagonism increases specific synaptic forms of glutamate release during anoxia, revealing a unique source of excitation. HIPPOCAMPUS. 1996;6(3):213-224.

5254. Ohlmer Ricarda, Jacobi Corinna, Taylor Craig Preventing Symptom Progression in Women at Risk for AN: Results of a Pilot Study. EUROPEAN EATING DISORDERS REVIEW. 2013;21(4):323-329.

5255. Goggin DE, Colmer TD Intermittent anoxia induces oxidative stress in wheat seminal roots: assessment of the antioxidant defence system, lipid peroxidation and tissue solutes. FUNCTIONAL PLANT BIOLOGY. 2005;32(6):495-506.

5256. Baer Karl-Juergen, Markser Valentin Sport specificity of mental disorders: the issue of sport psychiatry. EUROPEAN ARCHIVES OF PSYCHIATRY AND CLINICAL NEUROSCIENCE. 2013;263(2):S205-S210.

5257. Patel Ashish, Karlis Vasiliki Diagnosis and Management of Pediatric Salivary Gland Infections. ORAL AND MAXILLOFACIAL SURGERY CLINICS OF NORTH AMERICA. 2009;21(3):345+.

5258. Volpe Umberto, Tortorella Alfonso, Manchia Mirko, Monteleone Alessio, Albert Umberto, Monteleone Palmiero Eating disorders: What age at onset?. PSYCHIATRY RESEARCH. 2016;238():225-227.

5259. Fonseca H, Ireland M, Resnick MD Familial correlates of extreme weight control behaviors among adolescents. INTERNATIONAL JOURNAL OF EATING DISORDERS. 2002;32(4):441-448.

5260. Pollice C, Kaye WH, Greeno CG, Weltzin TE Relationship of depression, anxiety, and obsessionality to state of illness in anorexia nervosa. INTERNATIONAL JOURNAL OF EATING DISORDERS. 1997;21(4):367-376.

5261. Lenz Dunker Karin, Alvarenga Marle, Teixeira Paula, Grigolon Ruth Effects of participation level and physical activity on eating behavior and disordered eating symptoms in the Brazilian version of the New Moves intervention: data from a cluster randomized controlled trial. SAO PAULO MEDICAL JOURNAL. 2021;139(3):269-278.

5262. Zabinski MF, Wilfley DE, Pung MA, Winzelberg AJ, Eldredge K, Taylor CB An interactive Internet-based intervention for women at risk of eating disorders: A pilot study. INTERNATIONAL JOURNAL OF EATING DISORDERS. 2001;30(2):129-137.

5263. Garcia-Grau Eugeni, Fuste Adela, Mas Natalia, Gomez Juana, Bados Arturo, Saldana Carmina Dimensionality of Three Versions of the Eating Disorder Inventory in Adolescent Girls. EUROPEAN EATING DISORDERS REVIEW. 2010;18(4):318-327.

5264. Ballanyi K., Ruangkittisakul A., Onimaru H. Opioids prolong and anoxia shortens delay between onset of preinspiratory (pFRG) and inspiratory (preBotC) network bursting in newborn rat brainstems. PFLUGERS ARCHIV-EUROPEAN JOURNAL OF PHYSIOLOGY. 2009;458(3):571-587.

5265. Bullock Anastasia, Goldbacher Edie Interoceptive awareness and emotional eating in college women: the role of appetite and emotional awareness. JOURNAL OF AMERICAN COLLEGE HEALTH. 2021;():.

5266. Yager Zali, O'Dea Jennifer A controlled intervention to promote a healthy body image, reduce eating disorder risk and prevent excessive exercise among trainee health education and physical education teachers. HEALTH EDUCATION RESEARCH. 2010;25(5):841-852.

5267. Findlay S., Pinzon J., Taddeo D., Katzman D., Soc Canadian, Comm Adolescent Family-based treatment of children and adolescents with anorexia nervosa: Guidelines for the community physician. PAEDIATRICS \& CHILD HEALTH. 2010;15(1):31-35.

5268. Tran L, Kucera P, DeRibaupierre Y, Rochat AC, Raddatz E Glucose is arrhythmogenic in the anoxic-reoxygenated embryonic chick heart. PEDIATRIC RESEARCH. 1996;39(5):766-773.

5269. Oster Emily Diabetes and Diet: Purchasing Behavior Change in Response to Health Information. AMERICAN ECONOMIC JOURNAL-APPLIED ECONOMICS. 2018;10(4):308-348.

5270. Falco Maria, Francisco Rita Diabetes, eating disorders and body image in young adults: an exploratory study about ``diabulimia{''}. EATING AND WEIGHT DISORDERS-STUDIES ON ANOREXIA BULIMIA AND OBESITY. 2017;22(4):675-682.

5271. Hasebe Kyoko, Kendig Michael, Kaakoush Nadeem, Tajaddini Aynaz, Westbrook R., Morris Margaret The influence of maternal unhealthy diet on maturation of offspring gut microbiota in rat. ANIMAL MICROBIOME. 2022;4(1):.

5272. Ahn Jaeun, Lee Jung-Hyun, Jung Young-Chul Identifying Predictors of Non-Suicidal Self-Injuries in Individuals with Eating Disorders. YONSEI MEDICAL JOURNAL. 2021;62(2):159-163.

5273. Zabinski MF, Celio AA, Jacobs MJ, Manwaring J, Wilfley DE Internet-based prevention of eating disorders. EUROPEAN EATING DISORDERS REVIEW. 2003;11(3):183-197.

5274. Odukoya Oluwakemi, Manortey Steve, Takemoto Michelle, Alder Steve, Okuyemi Kolawole Body, Soul and Spirit, an adaptation of two evidence-based interventions to promote physical activity and healthy eating among adults in churches in Lagos Nigeria: a three-arm cluster randomized controlled pilot trial. PILOT AND FEASIBILITY STUDIES. 2020;6(1):.

5275. Ostermann Thomas, Vogel Hannah, Boehm Katja, Cramer Holger Effects of yoga on eating disorders-A systematic review. COMPLEMENTARY THERAPIES IN MEDICINE. 2019;46():73-80.

5276. Linville Deanna, O'Neil Maya, Huebner Angela Positive Adult Support and Depression Symptoms in Adolescent Females: The Partially Mediating Role of Eating Disturbances. JOURNAL OF RESEARCH ON ADOLESCENCE. 2011;21(2):335-341.

5277. Athanasiu Lavinia, Mattingsdal Morten, Melle Ingrid, Inderhaug Elin, Lien Trude, Agartz Ingrid, Lorentzen Steinar, Morken Gunnar, Andreassen Ole, Djurovic Srdjan Intron 12 in NTRK3 is associated with bipolar disorder. PSYCHIATRY RESEARCH. 2011;185(3):358-362.

5278. Findlay S., Pinzon J., Taddeo D., Katzman D., Pediat Soc, Adolescent Com Family-based treatment of children and adolescents with anorexia nervosa: Guidelines for the community physician. PAEDIATRICS \& CHILD HEALTH. 2010;15(1):36-40.

5279. Tylka Tracy, Calogero Rachel, Danielsdottir Sigrun Intuitive eating is connected to self-reported weight stability in community women and men. EATING DISORDERS. 2020;28(3):256-264.

5280. Jiang Hui, Tian Shunlian, Zeng Yan, Shi Jing Nerve Growth Factor Inhibits Gd3+-sensitive Calcium Influx and Reduces Chemical Anoxic Neuronal Death. JOURNAL OF HUAZHONG UNIVERSITY OF SCIENCE AND TECHNOLOGY-MEDICAL SCIENCES. 2008;28(4):379-382.

5281. Stein Murray An epidemiologic perspective on social anxiety disorder. JOURNAL OF CLINICAL PSYCHIATRY. 2006;67(12):3-8.

5282. Bisset Matthew, Rinehart Nicole, Sciberras Emma Body dissatisfaction and weight control behaviour in children with ADHD: a population-based study. EUROPEAN CHILD \& ADOLESCENT PSYCHIATRY. 2019;28(11):1507-1516.

5283. Moeck Ella, Bridgland Victoria, Takarangi Melanie Food for thought: Commentary on Burnette et al. (2021) ``Concerns and recommendations for using Amazon MTurk for eating disorder research{''} COMMENT. INTERNATIONAL JOURNAL OF EATING DISORDERS. 2022;55(2):282-284.

5284. Krishnan KRR Psychiatric and medical comorbidities of bipolar disorder. PSYCHOSOMATIC MEDICINE. 2005;67(1):1-8.

5285. Arouca Aline, Santaliestra-Pasias Alba, Moreno Luis, Marcos Ascension, Widhalm Kurt, Molnars Denes, Manios Yannis, Gottrand Frederic, Kafatos Anthony, Kersting Mathilde, Sjostrom Michael, Gutierrez Sainzu Angel, Ferrari Marika, Huybrechts Inge, Gonzalez-Gross Marcela, Forsner Maria, De Henauw Stefaan, Michels Nathalie, Gilbert Chantal, Libersa Christian, Castello Sara, Sjostrom Michael, Molnar Denes, Dallongeville Jean, Hall Gunnar, Maes Lea, Scalfi Luca, Melendez Pilar, Casajus Jose, Fleta Jesus, Rodriguez Gerardo, Tomas Concepcion, Mesana Maria, Vicente-Rodriguez German, Villarroya Adoracion, Gil Carlos, Ara Ignacio, Alvira Juan, Bueno Gloria, Bueno Olga, Leon Juan, Garagorri Jesus, Labayen Idoia, Bel Silvia, Marco Luis, Mouratidou Theodora, Santaliestra-Pasias Alba, Iglesia Iris, Gonzalez-Gil Esther, De Miguel-Etayo Pilar, Julian Cristina, Miguel-Berges Mary, Iguacel Isabel, Ruperez Azahara, Warnberg Julia, Nova Esther, Gomez Sonia, Diaz Ligia, Romeo Javier, Veses Ana, Zapatera Belen, Pozo Tamara, Martinez David, Beghin Laurent, Iliescu Catalina, Von Berlepsch Juliana, Sichert-Hellert Wolfgang, Koeppen Ellen, Erhardt Eva, Csernus Katalin, Torok Katalin, Bokor Szilvia, Angster, Nagy Eniko, Kovacs Orsolya, Repasi Judit, Codrington Caroline, Plada Maria, Papadaki Angeliki, Sarri Katerina, Viskadourou Anna,

Hatzis Christos, Kiriakakis Michael, Tsibinos George, Vardavas Constantine, Sbokos Manolis, Protoyeraki Eva, Fasoulaki Maria, Stehle Peter, Pietrzik Klaus, Breidenassel Christina, Spinneker Andre, Al-Tahan Jasmin, Segoviano Miriam, Berchtold Anke, Bierschbach Christine, Blatzheim Erika, Schuch Adelheid, Pickert Petra, Castillo Manuel, Gutierrez Angel, Ortega Francisco, Ruiz Jonatan, Artero Enrique, Espana Vanesa, Jimenez-Pavon David, Chilton Palma, Sanchez-Munoz Cristobal, Cuenca Magdalena, Arcella Davide, Azzini Elena, Barrison Emma, Bevilacqua Noemi, Buonocore Pasquale, Catasta Giovina, Censi Laura, Ciarapica Donatella, D'Acapito Paola, Galfo Myriam, Le Donne Cinzia, Leclercq Catherine, Maiani Giuseppe, Mauro Beatrice, Mistura Lorenza, Pasquali Antonella, Piccinelli Raffaela, Polito Angela, Roccaldo Romana, Spada Raffaella, Sette Stefania, Zaccaria Maria, Vitaglione Paola, Montagnese Concetta, De Bourdeaudhuij Ilse, De Vriendt Tineke, Matthys Christophe, Vereecken Carine, Maeyer Mieke, Ottevaere Charlene, Phillipp Katharina, Dietrich Sabine, Boriss-Riedl Birgit, Grammatikaki Eva, Bouloubasi Zoi, Cook Tina, Eleutheriou Sofia, Consta Orsalia, Moschonis George, Katsaroli Ioanna, Kraniou George, Papoutsou Stab, Keke Despoina, Petraki Ioanna, Bellou Elena, Tanagra Sofia, Kallianoti Kostalenia, Argyropoulou Dionysia, Tsikrika Stamatoula, Karaiskos Christos, Meirhaeghe Aline, Hagstromer Maria, Wenntof Anita, Hallstrom Lena, Patterson Emma, Kwak Lydia, Rizzo Nico, Sanchez-Motero Jackie, Pico Elena, Navarro Maite, Viadel Blanca, Carreres Jose, Merino Gema, Sanjuan Rosa, Lorente Maria, Sanchez Maria, Thomas Sarah, Allchurch Elaine, Burgess Peter, Astrom Annika, Sverken Anna, Broberg Agneta, Masson Annick, Lehoux Claire, Brabant Pascal, Pate Philippe, Fontaine Laurence, Sebok Andras, Kuti Tunde, Hegyi Adrienn, Maldonado Cristina, Llorente Ana, Garcia Emilio, Fircks Holger, Hallberg Marianne, Messerer Maria, Larsson Mats, Fredriksson Helena, Adamsson Viola, Borjesson Ingmar, Fernandez Laura, Smillie Laura, Wills Josephine, Pedrero-Chamizo Raquel, Melendez Agustin, Valtuena Jara, Albers Ulrike, Benito Pedro, Lorente Juan, Canada David, Urzanqui Alejandro, Torres Rosa, Navarro Paloma, Grp HELENA Diet as a moderator in the association of sedentary behaviors with inflammatory biomarkers among adolescents in the HELENA study. EUROPEAN JOURNAL OF NUTRITION. 2019;58(5):2051-2065.

5286. Herwaldt BL, Arroyave KR, Roberts JM, Juranek DD A multiyear prospective study of the risk factors for and incidence of diarrheal illness in a cohort of peace corps volunteers in Guatemala. ANNALS OF INTERNAL MEDICINE. 2000;132(12):982-988.

5287. Roustaee Roshank, Hajifaraji Majid, Djazayery Abolghasem, Mehrabi Yadolah Major Dietary patterns among female adolescents with eating disorders: A factor analysis approach. PROGRESS IN NUTRITION. 2018;20(3):378-386.

5288. Lopez-Guimera Gemma, Fauquet Jordi, Portell Mariona, Sanchez-Carracedo David, Raich Rosa Dieting in Spanish adolescent girls. EUROPEAN EATING DISORDERS REVIEW. 2008;16(3):234-240.

5289. Civil Arslan Filiz, Tiryaki Ahmet, Saglam Aykut Demet, Ozkorumak Evrim, Caliskan Ilter Zeynep, Gunaydin Dilek The Prevalence of Night Eating Syndrome among Outpatient Overweight or Obese Individuals with Serious Mental Illness. TURK PSIKIYATRI DERGISI. 2015;26(4):242-248.

5290. Krumschnabel G, Schwarzbaum PJ, Biasi C, Dorigatti M, Wieser W Effects of energy limitation on Ca2+ and K+ homeostasis in anoxia-tolerant and anoxia-intolerant hepatocytes. AMERICAN JOURNAL OF PHYSIOLOGY-REGULATORY INTEGRATIVE AND COMPARATIVE PHYSIOLOGY. 1997;273(1):R307-R316.

5291. Sawamoto Ryoko, Nozaki Takehiro, Nishihara Tomoe, Furukawa Tomokazu, Hata Tomokazu, Komaki Gen, Sudo Nobuyuki Predictors of successful long-term weight loss maintenance: a two-year follow-up. BIOPSYCHOSOCIAL MEDICINE. 2017;11():.

5292. Lee Amanda, Kane Sarah, Lewis Meron, Good Elizabeth, Pollard Christina, Landrigan Timothy, Dick Mathew Healthy diets ASAP - Australian Standardised Affordability and Pricing methods protocol. NUTRITION JOURNAL. 2018;17():.

5293. Matias Thiago, Veber Lopes Marcus, Mello Gabrielli, Silva Kelly Clustering of obesogenic behaviors and association with body image among Brazilian adolescents in the national school-based health survey (PeNSE 2015). PREVENTIVE MEDICINE REPORTS. 2019;16():.

5294. Viggiano Alessandro, Viggiano Emanuela, Di Costanzo Anna, Viggiano Andrea, Andreozzi Eleonora, Romano Vincenzo, Rianna Ines, Vicidomini Claudia, Gargano Giuliana, Incarnato Lucia, Fevola Celeste, Volta Pietro, Tolomeo Caterina, Scianni Giuseppina, Santangelo Caterina, Battista Roberta, Monda Marcellino, Viggiano Adela, De Luca Bruno, Amaro Salvatore Kaledo, a board game for nutrition education of children and adolescents at school: cluster randomized controlled trial of healthy lifestyle promotion. EUROPEAN JOURNAL OF PEDIATRICS. 2015;174(2):217-228.

5295. Tovar Alison, Hennessy Erin, Must Aviva, Hughes Sheryl, Gute David, Sliwa Sarah, Boulos Rebecca, Vikre Emily, Kamins Christina, Tofuri Kerline, Pirie Alex, Economos Christina Feeding styles and evening family meals among recent immigrants. INTERNATIONAL JOURNAL OF BEHAVIORAL NUTRITION AND PHYSICAL ACTIVITY. 2013;10():.

5296. Trier T., Mohammadnia N., Snaterse M., Peters R., Jorstad H., Bax W., Mackenbach J. An appeal to our government for nationwide policies in the prevention of cardiovascular

disease. NETHERLANDS HEART JOURNAL. 2022;30(1):58-62.

5297. Wolfenden Luke, Jones Jannah, Williams Christopher, Finch Meghan, Wyse Rebecca, Kingsland Melanie, Tzelepis Flora, Wiggers John, Williams Amanda, Seward Kirsty, Small Tameka, Welch Vivian, Booth Debbie, Yoong Sze Strategies to improve the implementation of healthy eating, physical activity and obesity prevention policies, practices or programmes within childcare services. COCHRANE DATABASE OF SYSTEMATIC REVIEWS. 2016;(10):.

5298. Merikangas Alison, Almasy Laura Using the tools of genetic epidemiology to understand sex differences in neuropsychiatric disorders. GENES BRAIN AND BEHAVIOR. 2020;19(6):.

5299. Karanti Alina, Kardell Mathias, Joas Erik, Runeson Bo, Palsson Erik, Landen Mikael Characteristics of bipolar I and II disorder: A study of 8766 individuals. BIPOLAR DISORDERS. 2020;22(4):392-400.

5300. Fond G., Capdevielle D., Macgregor A., Attal J., Larue A., Brittner M., Ducasse D., Boulenger J. Toxoplasma gondii: A potential role in the genesis of psychiatric disorders. ENCEPHALE-REVUE DE PSYCHIATRIE CLINIQUE BIOLOGIQUE ET THERAPEUTIQUE. 2013;39(1):38-43.

5301. Kasiak Przemyslaw, Adamczyk Natalia, Jodczyk Alicja, Kapron Aleksandra, Lisowska Anna, Mamcarz Artur, Sliz Daniel COVID-19 Pandemic Consequences among Individuals with Eating Disorders on a Clinical Sample in Poland-A Cross-Sectional Study. INTERNATIONAL JOURNAL OF ENVIRONMENTAL RESEARCH AND PUBLIC HEALTH. 2022;19(14):.

5302. Semenov DG, Samoilov MO, Lazarevich EV DTNB inhibits calcium response of rat brain cortical slices to anoxia of various duration. BULLETIN OF EXPERIMENTAL BIOLOGY AND MEDICINE. 2001;131(6):526-528.

5303. Rawana Jennine, Morgan Ashley, Nguyen Hien, Craig Stephanie The Relation Between Eating- and Weight-Related Disturbances and Depression in Adolescence: A Review. CLINICAL CHILD AND FAMILY PSYCHOLOGY REVIEW. 2010;13(3):213-230.

5304. Peat Christine, Feltner Cynthia Addressing eating disorders in primary care: Understanding screening recommendations and opportunities to improve care.

INTERNATIONAL JOURNAL OF EATING DISORDERS. 2022;55(9, SI):1202-1207.

5305. Wu Yi, Harrison Amy ``Our daily life was mainly comprised of eating and sitting:{''} a qualitative analysis of adolescents' experiences of inpatient eating disorder treatment in China. JOURNAL OF EATING DISORDERS. 2019;7():.

5306. Tenthorey D, Ribaupierre Y, Kucera P, Raddatz E Effects of verapamil and ryanodine on activity of the embryonic chick heart during anoxia and reoxygenation. JOURNAL OF CARDIOVASCULAR PHARMACOLOGY. 1998;31(2):195-202.

5307. Cimmino Fabiano, Catapano Angela, Trinchese Giovanna, Cavaliere Gina, Culurciello Rosanna, Fogliano Chiara, Penna Eduardo, Lucci Valeria, Crispino Marianna, Avallone Bice, Pizzo Elio, Mollica Maria Dietary Micronutrient Management to Treat Mitochondrial Dysfunction in Diet-Induced Obese Mice. INTERNATIONAL JOURNAL OF MOLECULAR SCIENCES. 2021;22(6):.

5308. Afflelou S, Duclos M, Simon S What are the links between sport activities and eating disorders?. PRESSE MEDICALE. 2004;33(22):1601-1605.

5309. Mohamed Sara, Abdel-Rahim Emam, Aly Tahany, Naguib AbdelMoneim, Khattab Marwa Barley microgreen incorporation in diet-controlled diabetes and counteracted aflatoxicosis in rats. EXPERIMENTAL BIOLOGY AND MEDICINE. 2022;247(5):385-394.

5310. Ferreira Claudia, Palmeira Lara, Trindade Ines Turning eating psychopathology risk factors into action. The pervasive effect of body image-related cognitive fusion. APPETITE. 2014;80():137-142.

5311. Fuemmeler Bernard, Pendzich Margaret, Tercyak Kenneth Weight, Dietary Behavior, and Physical Activity in Childhood and Adolescence: Implications for Adult Cancer Risk. OBESITY FACTS. 2009;2(3):179-186.

5312. Kelly-Weeder Susan, Wolfe Barbara Extreme Weight Loss Behaviors in Racially Diverse Urban Adolescents. JOURNAL OF ADOLESCENT HEALTH. 2019;64(2):276-278.

5313. Adjei Akosua, Amevinya Gideon, Quarpong Wilhemina, Tandoh Akua, Aryeetey Richmond, Holdsworth Michelle, Agyemang Charles, Zotor Francis, Laar Matilda, Mensah Kobby, Addo Phyllis, Laryea Dennis, Asiki Gershim, Sellen Daniel, Vandevijvere Stefanie,

Laar Amos Availability of healthy and unhealthy foods in modern retail outlets located in selected districts of Greater Accra Region, Ghana. FRONTIERS IN PUBLIC HEALTH. 2022;10():.

5314. Eisler Ivan, Simic Mima, Russell Gerald, Dare Christopher A randomised controlled treatment trial of two forms of family therapy in adolescent anorexia nervosa: a five-year follow-up. JOURNAL OF CHILD PSYCHOLOGY AND PSYCHIATRY. 2007;48(6):552-560.

5315. Aritici Colak Gozde, Orku Ecem, Parmaksiz Ayhan, Bas Murat Reliability and Validation of the Children's Eating Attitudes Test among 10- to 14-Year-Old School Children in Turkey. NUTRICION CLINICA Y DIETETICA HOSPITALARIA. 2022;42(2):36-42.

5316. Cunkus Nesrin, Yigitoglu Gulay A nursing care for a male anorexia nervosa case: A case report. JOURNAL OF PSYCHIATRIC NURSING. 2019;10(1):82-87.

5317. Harada Tomoko, Yamauchi Tsuneo, Miyawaki Dai, Miyamoto Saori, Yoshida Hisako, Nishimoto Kazuya, Matsuzuka Takumi, Honda Mihoko, Inoue Koki Anorexia nervosa restricting type has increased in severity over three decades: Japanese clinical samples from 1988 to 2018. INTERNATIONAL JOURNAL OF EATING DISORDERS. 2021;54(1, SI):54-58.

5318. LaMarre Andrea, Levine Michael, Holmes Su, Malson Helen An open invitation to productive conversations about feminism and the spectrum of eating disorders (part 2): Potential contributions to the science of diagnosis, treatment, and prevention. JOURNAL OF EATING DISORDERS. 2022;10(1):.

5319. Phillips P The rising cost of health care: can demand be reduced through more effective health promotion?. JOURNAL OF EVALUATION IN CLINICAL PRACTICE. 2002;8(4):415-419.

5320. Burke Natasha, Shomaker Lauren, Brady Sheila, Reynolds James, Young Jami, Wilfley Denise, Sbrocco Tracy, Stephens Mark, Olsen Cara, Yanovski Jack, Tanofsky-Kraff Marian Impact of Age and Race on Outcomes of a Program to Prevent Excess Weight Gain and Disordered Eating in Adolescent Girls. NUTRIENTS. 2017;9(9):.

5321. Yan Chiu-Lan, Kao Li-Ting, Yeh Ming-kung, Chien Wu-Chien, Yeh Chin-Bin Healthcare utilisation for eating disorders among patients with depression: a cross-sectional study in Taiwan. BMJ OPEN. 2019;9(12):.

5322. RaleySusman KM, Barnes JR, Kaja J Effects of volatile anesthetics on hippocampal slice metabolism, response to anoxia with and without glucose. BRAIN RESEARCH. 1997;755(1):1-8.

5323. Haines Jess, Neumark-Sztainer Dianne Prevention of obesity and eating disorders: a consideration of shared risk factors. HEALTH EDUCATION RESEARCH. 2006;21(6):770-782.

5324. Karmous Inchirah, Doggui Radhouene, Khan Amira, Ben Amor Nadia, Khan Naim, Jamoussi Henda Is fat taste associated with diet quality? A cross-sectional study conducted among Tunisian adults. APPETITE. 2022;176():.

5325. Muller Andre, Maher Carol, Vandelanotte Corneel, Hingle Melanie, Middelweerd Anouk, Lopez Michael, DeSmet Ann, Short Camille, Nathan Nicole, Hutchesson Melinda, Poppe Louise, Woods Catherine, Williams Susan, Wark Petra Physical Activity, Sedentary Behavior, and Diet-Related eHealth and mHealth Research: Bibliometric Analysis. JOURNAL OF MEDICAL INTERNET RESEARCH. 2018;20(4):.

5326. Parker Elizabeth, Maister Terri, Stefoska-Needham Anita, Wearne Christine, Anderson Gail, Gomes Linette, Clarke Simon, Kohn Michael An audit of the changes in thiamine levels during higher caloric nutritional rehabilitation of adolescent patients hospitalised with a restrictive eating disorder. JOURNAL OF EATING DISORDERS. 2020;8(1):.

5327. Danise Silvia, Twitchett Richard, Little Crispin, Clemence Marie-Emilie The Impact of Global Warming and Anoxia on Marine Benthic Community Dynamics: an Example from the Toarcian (Early Jurassic). PLOS ONE. 2013;8(2):.

5328. Herpertz-Dahlmann Beate, Bonin Eva, Dahmen Brigitte Can you find the right support for children, adolescents and young adults with anorexia nervosa: Access to age-appropriate care systems in various healthcare systems. EUROPEAN EATING DISORDERS REVIEW. 2021;29(3, SI):316-328.

5329. Foye Una, Hazlett Diane, Irving Pauline `The body is a battleground for unwanted and unexpressed emotions': exploring eating disorders and the role of emotional intelligence. EATING DISORDERS. 2019;27(3):321-342.

5330. Wable Gauri, Barbarich-Marsteller Nicole, Chowdhury Tara, Sabaliauskas Nicole, Farb Claudia, Aoki Chiye Excitatory Synapses on Dendritic Shafts of the Caudal Basal Amygdala Exhibit Elevated Levels of GABA(A) Receptor alpha 4 Subunits Following the Induction of

Activity-Based Anorexia. SYNAPSE. 2014;68(1):1-15.

5331. Dighe Shruti, Zhao Jiwei, Steffen Lyn, Mares J., Meuer Stacy, Klein Barbara, Klein Ronald, Millen Amy Diet patterns and the incidence of age-related macular degeneration in the Atherosclerosis Risk in Communities (ARIC) study. BRITISH JOURNAL OF OPHTHALMOLOGY. 2020;104(8):1070-1076.

5332. Lopez-Guimera Gemma, Sanchez-Carracedo David, Fauquet Jordi, Portell Mariona, Raich Rosa Impact of a School-Based Disordered Eating Prevention Program in Adolescent Girls: General and Specific Effects Depending on Adherence to the Interactive Activities. SPANISH JOURNAL OF PSYCHOLOGY. 2011;14(1):293-303.

5333. Burychka Diana, Miragall Marta, Banos Rosa Towards a Comprehensive Understanding of Body Image: Integrating Positive Body Image, Embodiment and Self-Compassion. PSYCHOLOGICA BELGICA. 2021;61(1):248-261.

5334. Powell Elisabeth, Frankel Leslie, Hernandez Daphne The mediating role of child self-regulation of eating in the relationship between parental use of food as a reward and child emotional overeating. APPETITE. 2017;113():78-83.

5335. Simeunovic Ostojic Mladena, Maas Joyce, Bodde Nynke COVID-19, anorexia nervosa and obese patients with an eating disorder-some considerations for practitioners and researchers. JOURNAL OF EATING DISORDERS. 2021;9(1):.

5336. Koning Elena, McDonald Alexandra, Bambokian Alexander, Gomes Fabiano, Vorstman Jacob, Berk Michael, Fabe Jennifer, McIntyre Roger, Milev Roumen, Mansur Rodrigo, Brietzke Elisa The concept of ``metabolic jet lag{''} in the pathophysiology of bipolar disorder: implications for research and clinical care. CNS SPECTRUMS. 2022;():.

5337. Zanarini MC, Frankenburg FR, Ridolfi ME, Jager-Hyman S, Hermen J, Gunderson JG Reported childhood onset of self-mutilation among borderline patients. JOURNAL OF PERSONALITY DISORDERS. 2006;20(1):9-15.

5338. Fardouly Jasmine, Crosby Ross, Sukunesan Suku Potential benefits and limitations of machine learning in the field of eating disorders: current research and future directions. JOURNAL OF EATING DISORDERS. 2022;10(1):.

5339. Ashton Lee, Morgan Philip, Hutchesson Melinda, Rollo Megan, Collins Clare Feasibility and preliminary efficacy of the `HEYMAN' healthy lifestyle program for young men: a pilot randomised controlled trial. NUTRITION JOURNAL. 2017;16():.

5340. Richards Margaret, Banez Gerard, Dohil Ranjan, Stein Martin Chronic Constipation, Atypical Eating Pattern, Weight Loss, and Anxiety in a 19-Year Old Youth. JOURNAL OF DEVELOPMENTAL AND BEHAVIORAL PEDIATRICS. 2010;31(3, S):S83-S85.

5341. ROBERTS EL, CHIH CP AGE-RELATED ALTERATIONS IN ENERGY-METABOLISM CONTRIBUTE TO THE INCREASED VULNERABILITY OF THE AGING BRAIN TO ANOXIC DAMAGE. BRAIN RESEARCH. 1995;678(1-2):83-90.

5342. Harper Jessica, Palka Jayme, McAdams Carrie Interpersonal attribution bias and social evaluation in adolescent eating disorders. EUROPEAN EATING DISORDERS REVIEW. 2023;31(2):258-270.

5343. Wilson J., Blizzard L., Gall S., Magnussen C., Oddy W., Dwyer T., Venn A., Smith K. Youth diet quality and hazard of mood disorder in adolescence and adulthood among an Australian cohort. JOURNAL OF AFFECTIVE DISORDERS. 2020;276():511-518.

5344. Spreckelsen Paula, Wessel Ineke, Glashouwer Klaske, Jong Peter Averting Repulsion? Body-Directed Self-Disgust and Autobiographical Memory Retrieval. JOURNAL OF EXPERIMENTAL PSYCHOPATHOLOGY. 2022;13(1):.

5345. Arena Ross, Pronk Nicolaas, Laddu Deepika, Whitsel Laurie, Sallis James, Lavie Carl, Network HL Mapping One Million COVID-19 Deaths and Unhealthy Lifestyle Behaviors in the United States: Recognizing the Syndemic Pattern and Taking Action. AMERICAN JOURNAL OF MEDICINE. 2022;135(11):1288-1295.

5346. Oliveira Marcio, Conti Cristiane, Prado Gilmar Pharmacological treatment for Kleine-Levin syndrome. COCHRANE DATABASE OF SYSTEMATIC REVIEWS. 2016;(5):.

5347. Turk Fidan, Kellett Stephen, Waller Glenn Determining the potential links of self-compassion with eating pathology and body image among women and men: A cross-sectional mediational study. BODY IMAGE. 2021;37():28-37.

5348. Ruchkin Vladislav, Isaksson Johan, Schwab-Stone Mary, Stickley Andrew Prevalence and early risk factors for bulimia nervosa symptoms in inner-city youth: gender and ethnicity perspectives. JOURNAL OF EATING DISORDERS. 2021;9(1):.

5349. Baena Ruiz Raul, Salinas Hernandez Pedro Diet and cancer: Risk factors and epidemiological evidence. MATURITAS. 2014;77(3):202-208.

5350. Trapp Georgina, Allen Karina, Black Lucinda, Ambrosini Gina, Jacoby Peter, Byrne Susan, Martin Karen, Oddy Wendy A prospective investigation of dietary patterns and internalizing and externalizing mental health problems in adolescents. FOOD SCIENCE \& NUTRITION. 2016;4(6):888-896.

5351. Romano Kelly, Heron Kristin Regulatory parental feeding behaviors, emotion suppression, and emotional eating in the absence of hunger: Examining parent-adolescent dyadic associations. APPETITE. 2021;167():.

5352. Eichen Dawn, Strong David, Rhee Kyung, Rock Cheryl, Crow Scott, Epstein Leonard, Wilfley Denise, Boutelle Kerri Change in eating disorder symptoms following pediatric obesity treatment. INTERNATIONAL JOURNAL OF EATING DISORDERS. 2019;52(3):299-303.

5353. Zhang Lirong, Zhao Shaocong, Weng Wei, Lin Qiong, Song Minmin, Wu Shouren, Zheng Hua Frequent Sports Dance May Serve as a Protective Factor for Depression Among College Students: A Real-World Data Analysis in China. PSYCHOLOGY RESEARCH AND BEHAVIOR MANAGEMENT. 2021;14():405-422.

5354. Goffin Kathryn, Dell'Osso Bernardo, Miller Shefali, Wang Po, Holtzman Jessica, Hooshmand Farnaz, Ketter Terence Different characteristics associated with suicide attempts among bipolar I versus bipolar II disorder patients. JOURNAL OF PSYCHIATRIC RESEARCH. 2016;76():94-100.

5355. Castaneda-Marquez Ana, Diaz-Benitez CinthyaEstefhany, Bahena-Roman Margarita, Campuzano-Benitez Guadalupe, Galvan-Portillo Marcia, Campuzano-Rincon Julio, Lagunas-Martinez Alfredo, Bermudez-Morales Victor, Orbe-Orihuela Yaneth, Peralta-Romero Jesus, Cruz Miguel, Burguete-Garcia Ana Lactobacillus paracasei as a protective factor of obesity induced by an unhealthy diet in children. OBESITY RESEARCH \& CLINICAL PRACTICE. 2020;14(3):271-278.

5356. Farhangi Mahdieh, Jahangiry Leila, Asghari-Jafarabadi Mohammad, Najafi Mahdi Association between dietary patterns and metabolic syndrome in a sample of Tehranian adults. OBESITY RESEARCH \& CLINICAL PRACTICE. 2016;10(1):S64-S73.

5357. NOACK K, BRONK SF, KATO A, GORES GJ THE GREATER VULNERABILITY OF BILE-DUCT CELLS TO REOXYGENATION INJURY THAN TO ANOXIA - IMPLICATIONS FOR THE PATHOGENESIS OF BILIARY STRICTURES AFTER LIVER-TRANSPLANTATION. TRANSPLANTATION. 1993;56(3):495-500.

5358. Brown Tiffany, Murray Stuart, Anderson Leslie, Kaye Walter Early predictors of treatment outcome in a partial hospital program for adolescent anorexia nervosa. INTERNATIONAL JOURNAL OF EATING DISORDERS. 2020;53(9):1550-1555.

5359. Jaworski Mariusz, Panczyk Mariusz, Sliwczyriski Andrzej, Brzozowska Melania, Janaszek Katarzyna, Malkowski Piotr, Gotlib Joanna Eating Disorders in Males: An 8-Year Population-Based Observational Study. AMERICAN JOURNAL OF MENS HEALTH. 2019;13(4):.

5360. Iacobini Carla, Vitale Martina, Haxhi Jonida, Pesce Carlo, Pugliese Giuseppe, Menini Stefano Food-Related Carbonyl Stress in Cardiometabolic and Cancer Risk Linked to Unhealthy Modern Diet. NUTRIENTS. 2022;14(5):.

5361. Tsekoura E., Kostopoulou E., Fouzas S., Souris E., Gkentzi D., Jelastopulu E., Varvarigou A. The association between obesity and the risk for development of eating disorders - A large-scale epidemiological study. EUROPEAN REVIEW FOR MEDICAL AND PHARMACOLOGICAL SCIENCES. 2021;25(19):6051-6056.

5362. Ufer N, Bredenkamp J, Jacoby GE Induced ``Freudian slips{''} and bulimia. ZEITSCHRIFT FUR KLINISCHE PSYCHOLOGIE-FORSCHUNG UND PRAXIS. 2000;29(3):180-186.

5363. Cai XinYong, Yang Chunli, Shao Liang, Zhu HongMin, Wang YunXia, Huang Xiao, Wang Shu, Hong Lang Targeting NOX 4 by petunidin improves anoxia/reoxygenation-induced myocardium injury. EUROPEAN JOURNAL OF PHARMACOLOGY. 2020;888():.

5364. Neumark-Sztainer D, Sherwood NE, Coller T, Hannan PJ Primary prevention of disordered eating among preadolescent girls: Feasibility and short-term effect of a community-based intervention. JOURNAL OF THE AMERICAN DIETETIC ASSOCIATION.

2000;100(12):1466-1473.

5365. Gele Abdi, Torheim Liv, Pettersen Kjell, Kumar Bernadette Beyond Culture and Language: Access to Diabetes Preventive Health Services among Somali Women in Norway. JOURNAL OF DIABETES RESEARCH. 2015;2015():.

5366. Kim Yongjoo, Roberts Andrea, Rimm Eric, Chibnik Lori, Tworoger Shelley, Nishimi Kristen, Sumner Jennifer, Koenen Karestan, Kubzansky Laura Posttraumatic stress disorder and changes in diet quality over 20 years among US women. PSYCHOLOGICAL MEDICINE. 2021;51(2):310-319.

5367. Samson Lelia, Nanne Annemarie, Buijzen Moniek Remember the motivationally-relevant appeals? The influence of social and sensory appeals on memory for pronutritional messages promoting healthy foods. INTERNATIONAL JOURNAL OF ADVERTISING. 2021;40(4, SI):582-601.

5368. Ben-Dor DH, Laufer N, Apter A, Frisch A, Weizman A Heritability, genetics and association findings in anorexia nervosa. ISRAEL JOURNAL OF PSYCHIATRY AND RELATED SCIENCES. 2002;39(4):262-270.

5369. Shin DSH, Buck LT Effect of anoxia and pharmacological anoxia on whole-cell NMDA receptor currents in cortical neurons from the western painted turtle. PHYSIOLOGICAL AND BIOCHEMICAL ZOOLOGY. 2003;76(1):41-51.

5370. Posternak MA, Zimmerman M Lack of association between seasonality and psychopathology in psychiatric outpatients. PSYCHIATRY RESEARCH. 2002;112(3):187-194.

5371. Rausch RN, Crawshaw LI, Wallace HL Effects of hypoxia, anoxia, and endogenous ethanol on thermoregulation in goldfish, Carassius auratus. AMERICAN JOURNAL OF PHYSIOLOGY-REGULATORY INTEGRATIVE AND COMPARATIVE PHYSIOLOGY. 2000;278(3):R545-R555.

5372. Al-Jawaldeh Ayoub, Abbass Marwa Unhealthy Dietary Habits and Obesity: The Major Risk Factors Beyond Non-Communicable Diseases in the Eastern Mediterranean Region. FRONTIERS IN NUTRITION. 2022;9():.

5373. Natarajan Vaishnav, Sekar Tharani, Chockalingam Priya Prevalence of cardiovascular health risk behaviors in college-going women in a major metropolis in India. INDIAN HEART JOURNAL. 2020;72(5):451-453.

5374. Zorbas Christina, Reeve Erica, Naughton Shaan, Batis Carolina, Whelan Jillian, Waqa Gade, Bell Colin The Relationship Between Feasting Periods and Weight Gain: a Systematic Scoping Review. CURRENT OBESITY REPORTS. 2020;9(1):39-62.

5375. Kavitha P., Vivek P., Hegde A. Eating Disorders and their Implications on Oral Health - Role of Dentists. JOURNAL OF CLINICAL PEDIATRIC DENTISTRY. 2011;36(2):155-160.

5376. Grassi Davide, Desideri Giovambattista, Ferri Claudio When dumbness turns vantages into disadvantages From healthy ingredients to ``bad foods{''}. AGRO FOOD INDUSTRY HI-TECH. 2010;21(4):8-11.

5377. Dixon Helen, Scully Maree, Wakefield Melanie, Kelly Bridget, Pettigrew Simone, Chapman Kathy, Niederdeppe Jeff The impact of unhealthy food sponsorship vs. pro-health sponsorship models on young adults' food preferences: a randomised controlled trial. BMC PUBLIC HEALTH. 2018;18():.

5378. Teymoori Farshad, Mokhtari Ebrahim, Jahromi Mitra, Farhadnejad Hossein, Mirmiran Parvin, Vafa Mohammadreza, Azizi Fereidoun Dietary and lifestyle indices for hyperinsulinemia with the risk of obesity phenotypes: a prospective cohort study among Iranian adult population. BMC PUBLIC HEALTH. 2022;22(1):.

5379. Fortier ME, Joober R, Luheshi GN, Boksa P Maternal exposure to bacterial endotoxin during pregnancy enhances amphetamine-induced locomotion and startle responses in adult rat offspring. JOURNAL OF PSYCHIATRIC RESEARCH. 2004;38(3):335-345.

5380. Wilkinson Paul, Qiu Tianyou, Neufeld Sharon, Jones Peter, Goodyer Ian Sporadic and recurrent non-suicidal self-injury before age 14 and incident onset of psychiatric disorders by 17 years: prospective cohort study. BRITISH JOURNAL OF PSYCHIATRY. 2018;212(4):222-226.

5381. Borumandnia Nasrin, Majd Hamid, Doosti Hassan, Olazadeh Keyvan The trend analysis of neurological disorders as major causes of death and disability according to human development, 1990-2019. ENVIRONMENTAL SCIENCE AND POLLUTION RESEARCH.

2022;29(10):14348-14354.

5382. Kimia M. Lifestyle modification helps health care system in diabetes management. AFRICAN JOURNAL OF DIABETES MEDICINE. 2020;28(2):.

5383. Fairweather-Schmidt A., Lee Christina, Wade Tracey A Longitudinal Study of Midage Women With Indicators of Disordered Eating. DEVELOPMENTAL PSYCHOLOGY. 2015;51(5):722-729.

5384. Brasseler Maire, Schonecker Anne, Steindor Mathis, Della Marina Adela, Bruns Nora, Dogan Burcin, Felderhoff-Muser Ursula, Hebebrand Johannes, Dohna-Schwake Christian, Goretzki Sarah Development of restrictive eating disorders in children and adolescents with long-COVID-associated smell and taste dysfunction. FRONTIERS IN PEDIATRICS. 2022;10():.

5385. Mendis Shanthi Global progress in prevention of cardiovascular disease. CARDIOVASCULAR DIAGNOSIS AND THERAPY. 2017;7(1):S32-S38.

5386. Izydorczyk Bernadetta, Sitnik-Warchulska Katarzyna, Wajda Zbigniew, Lizinczyk Sebastian, Sciegienny Aleksandra Bonding With Parents, Body Image, and Sociocultural Attitudes Toward Appearance as Predictors of Eating Disorders Among Young Girls. FRONTIERS IN PSYCHIATRY. 2021;12():.

5387. Barrea Luigi, Savastano Silvia, Di Somma Carolina, Savanelli Maria, Nappi Francesca, Albanese Lidia, Orio Francesco, Colao Annamaria Low serum vitamin D-status, air pollution and obesity: A dangerous liaison. REVIEWS IN ENDOCRINE \& METABOLIC DISORDERS. 2017;18(2):207-214.

5388. Myers Taryn What about being a feminist is protective? An examination of constructs related to feminist beliefs as moderators of the relationship between media awareness and thin-ideal internalization. BODY IMAGE. 2022;41():248-261.

5389. Strumila Robertas, Lengvenyte Aiste, Olie Emilie, Seneque Maude, Dupuis-Maurin Kathlyne, Alacreu-Crespo Adrian, Maimoun Laurent, Lefebvre Patrick, Renard Eric, Courtet Philippe, Guillaume Sebastien Selenium deficiency is associated with disease severity, disrupted reward processing, and increased suicide risk in patients with Anorexia Nervosa. PSYCHONEUROENDOCRINOLOGY. 2022;140():.

5390. Lawson-Boyd Elsher PLEASURES, PERCEPTIONS AND PRACTICES: Eating at a Uruguayan Social and Sporting Club. LOCALE-THE AUSTRALIAN-PACIFIC JOURNAL OF REGIONAL FOOD STUDIES. 2018;(7):82-99.

5391. Namin Aidin, Ratchford Brian, Saint Clair Julian, Bui My, Hamilton Mitchell Dine-in or take-out: Modeling millennials' cooking motivation and choice. JOURNAL OF RETAILING AND CONSUMER SERVICES. 2020;53():.

5392. Fukutomi Akira, Austin Amelia, McClelland Jessica, Brown Amy, Glennon Danielle, Mountford Victoria, Grant Nina, Allen Karina, Schmidt Ulrike First episode rapid early intervention for eating disorders: A two-year follow-up. EARLY INTERVENTION IN PSYCHIATRY. 2020;14(1):137-141.

5393. Eanes Linda, Fuentes Lilia, Bautista Beatriz, Salazar David, Garza Doreen Bridging the Gaps Through Nurse-Led Nutrition Education to Underserved Children. HISPANIC HEALTH CARE INTERNATIONAL. 2019;17(2):66-72.

5394. Rogozinska Ewelina, Chamillard Monica, Hitman Graham, Khan Khalid, Thangaratinam Shakila Nutritional Manipulation for the Primary Prevention of Gestational Diabetes Mellitus: A Meta-Analysis of Randomised Studies. PLOS ONE. 2015;10(2):.

5395. Enriquez Erin, Duncan Glen, Schur Ellen Age at dieting onset, body mass index, and dieting practices. A twin study. APPETITE. 2013;71():301-306.

5396. Wei Yanxin, Minhad Khairun, Selamat Nur, Md Ali Sawal, Sobhan Bhuiyan Mohammad, Ooi Kelvin, Samdin Siti A review of chewing detection for automated dietary monitoring. JOURNAL OF THE CHINESE INSTITUTE OF ENGINEERS. 2022;45(4):331-341.

5397. Marek Ryan, Ben-Porath Yossef, Merrell Julie, Ashton Kathleen, Heinberg Leslie Predicting One and Three Month Postoperative Somatic Concerns, Psychological Distress, and Maladaptive Eating Behaviors in Bariatric Surgery Candidates with the Minnesota Multiphasic Personality Inventory-2 Restructured Form (MMPI-2-RF). OBESITY SURGERY. 2014;24(4):631-639.

5398. Czegledi Edit, Szabo Kornelia HUNGARIAN EXPERIENCES WITH THE BELIEFS ABOUT ATTRACTIVENESS SCALE. IDEGGYOGYASZATI SZEMLE-CLINICAL NEUROSCIENCE. 2016;69(3-4):98-105.

5399. Farhangi Mahdieh Night Eating Syndrome and Its Relationship with Emotional Eating, Sleep Quality and Nutritional Status Among Adolescents' Boys. COMMUNITY MENTAL HEALTH JOURNAL. 2019;55(8):1411-1418.

5400. Cvjetan Branko, Utter Jennifer, Robinson Elizabeth, Denny Simon The Social Environment of Schools and Adolescent Nutrition: Associations Between the School Nutrition Climate and Adolescents' Eating Behaviors and Body Mass Index. JOURNAL OF SCHOOL HEALTH. 2014;84(10):677-682.

5401. Bruno Antonio, Mattei Antonella, Arnone Federico, Barbieri Arianna, Basile Valerio, Cedro Clemente, Celebre Laura, Mento Carmela, Rizzo Amelia, Silvestri Maria, Muscatello Maria, Zoccali Rocco, Pandolfo Gianluca LIFETIME PSYCHIATRIC COMORBIDITY AND DIAGNOSTIC TRAJECTORIES IN AN ITALIAN PSYCHIATRIC SAMPLE. CLINICAL NEUROPSYCHIATRY. 2020;17(5):263-270.

5402. Paschall Olivia, Carmichael Sarah, Koenigshof Peter, Waters Johnny, Ta Phuong, Komatsu Toshifumi, Dombrowski Allison The Devonian-Carboniferous boundary in Vietnam: Sustained ocean anoxia with a volcanic trigger for the Hangenberg Crisis?. GLOBAL AND PLANETARY CHANGE. 2019;175():64-81.

5403. Weissman Ruth Shining a light on ideas worth researching in the field of eating disorders: Introduction to a Virtual Issue of the International Journal of Eating Disorders. INTERNATIONAL JOURNAL OF EATING DISORDERS. 2021;54(8):1325-1327.

5404. Lyons Emma, Nekkanti Akhila, Funderburk Beverly, Skowron Elizabeth Parent-Child Interaction Therapy Supports Healthy Eating Behavior in Child Welfare-Involved Children. INTERNATIONAL JOURNAL OF ENVIRONMENTAL RESEARCH AND PUBLIC HEALTH. 2022;19(17):.

5405. Tan Rong, Dong Huiwei, Chen Zhengshan, Jin Min, Yin Jing, Li Haibei, Shi Danyang, Shao Yifan, Wang Huaran, Chen Tianjiao, Yang Dong, Li Junwen Intestinal Microbiota Mediates High-Fructose and High-Fat Diets to Induce Chronic Intestinal Inflammation. FRONTIERS IN CELLULAR AND INFECTION MICROBIOLOGY. 2021;11():.

5406. Azzi Vanessa, Hallit Souheil, Malaeb Diana, Obeid Sahar, Brytek-Matera Anna Drunkorexia and Emotion Regulation and Emotion Regulation Difficulties: The Mediating Effect of Disordered Eating Attitudes. INTERNATIONAL JOURNAL OF ENVIRONMENTAL RESEARCH AND PUBLIC HEALTH. 2021;18(5):.

5407. Alameda Luis, Trotta Giulia, Quigley Harriet, Rodriguez Victoria, Gadelrab Romayne, Dwir Daniella, Dempster Emma, Wong Chloe, Forti Marta Can epigenetics shine a light on the biological pathways underlying major mental disorders?. PSYCHOLOGICAL MEDICINE. 2022;52(9):1645-1665.

5408. Arriola Kimberly, Hermstad April, Flemming Shauna, Honeycutt Sally, Carvalho Michelle, Cherry Sabrina, Davis Tamara, Frazier Sheritta, Escoffery Cam, Kegler Michelle Promoting Policy and Environmental Change in Faith-Based Organizations: Description and Findings From a Mini-Grants Program. AMERICAN JOURNAL OF HEALTH PROMOTION. 2017;31(3):192-199.

5409. Lee Amanda, Patay Dori, Herron Lisa-Maree, Harrison Ella, Lewis Meron Affordability of current, and healthy, more equitable, sustainable diets by area of socioeconomic disadvantage and remoteness in Queensland: insights into food choice. INTERNATIONAL JOURNAL FOR EQUITY IN HEALTH. 2021;20(1):.

5410. Christiansen Dorte, McCarthy Margaret, Seeman Mary Where Sex Meets Gender: How Sex and Gender Come Together to Cause Sex Differences in Mental Illness. FRONTIERS IN PSYCHIATRY. 2022;13():.

5411. Shigematsu S, Arita M Anoxia-induced activation of ATP-sensitive K+ channels in guinea pig ventricular cells and its modulation by glycolysis. CARDIOVASCULAR RESEARCH. 1997;35(2):273-282.

5412. Watt Jaclyn, Dickens Geoffrey Community-based mealtime management for adolescents with anorexia nervosa: A qualitative study of clinicians' perspectives and experiences. JOURNAL OF CHILD AND ADOLESCENT PSYCHIATRIC NURSING. 2018;31(1):30-38.

5413. Hawrysh Peter, Buck Leslie Mitochondrial matrix pH acidifies during anoxia and is maintained by the F1Fo-ATPase in anoxia-tolerant painted turtle cortical neurons. FEBS OPEN BIO. 2019;9(4):571-581.

5414. Krug I., Villarejo C., Jimenez-Murcia S., Perpina C., Vilarrasa N., Granero R., Cebolla A., Botella C., Bernabe M., Penelo E., Casella S., Islam M., Orekhova E., Casanueva F., Karwautz A., Menchon J., Treasure J., Fernandez-Aranda F. Eating-related Environmental Factors in Underweight Eating Disorders and Obesity: Are There Common Vulnerabilities During Childhood and Early Adolescence?. EUROPEAN EATING DISORDERS REVIEW.

2013;21(3):202-208.

5415. Robert-McComb Jacalyn, Loucks Anne THE FEMALE ATHLETE TRIAD: Key Points for Health and Fitness Specialists. ACSMS HEALTH \& FITNESS JOURNAL. 2014;18(3):12-17.

5416. Skar M., Kirstein E., Kapur A. Lessons learnt from school-based health promotion projects in low- and middle-income countries. CHILD CARE HEALTH AND DEVELOPMENT. 2015;41(6):1114-1123.

5417. Kwag Kyung, Han Soo, Cho Ji-Yeoun, Ko Myeong, Park Eun, Kim Youl-Ri A school-based eating disorder prevention program (Me, You \& Us) for young adolescents in Korea: A 3-year follow-up study. INTERNATIONAL JOURNAL OF EATING DISORDERS. 2021;54(2, SI):168-173.

5418. Jauregui-Loberau Ignacio, Jose Santiago Maria Impulsivity and eating behavior in males. NUTRICION HOSPITALARIA. 2017;34(1):165-170.

5419. Pineda Arturo, Pourshafeie Armin, Ioannidis Alexander, Leibold Collin, Chan Avis, Bustamante Carlos, Frankovich Jennifer, Wojcik Genevieve Discovering prescription patterns in pediatric acute-onset neuropsychiatric syndrome patients. JOURNAL OF BIOMEDICAL INFORMATICS. 2021;113():.

5420. Wilksch Simon Universal school-based eating disorder prevention: Benefits to both high- and low-risk participants on the core cognitive feature of eating disorders. CLINICAL PSYCHOLOGIST. 2010;14(2):62-69.

5421. Plessow Franziska, Singhal Vibha, Toth Alexander, Micali Nadia, Eddy Kamryn, Misra Madhusmita Estrogen administration improves the trajectory of eating disorder pathology in oligo-amenorrheic athletes: A randomized controlled trial. PSYCHONEUROENDOCRINOLOGY. 2019;102():273-280.

5422. Christian Caroline, Ngo Betty, Brosof Leigh, Levinson Cheri Social appearance anxiety moderates the relationship between thin-ideal internalization and eating disorder symptoms cross-sectionally and prospectively in adolescent girls. EATING AND WEIGHT DISORDERS-STUDIES ON ANOREXIA BULIMIA AND OBESITY. 2021;26(6):2065-2070.

5423. Dawson-Scully K., Bukvic D., Chakaborty-Chatterjee M., Ferreira R., Milton S., Sokolowski M. Controlling anoxic tolerance in adult Drosophila via the cGMP-PKG pathway. JOURNAL OF EXPERIMENTAL BIOLOGY. 2010;213(14):2410-2416.

5424. Thompson AM, Chad KE The relationship of social physique anxiety to risk for developing an eating disorder in young females. JOURNAL OF ADOLESCENT HEALTH. 2002;31(2):183-189.

5425. Weinger Katie, Lee Jarim Psychosocial and psychiatric challenges of diabetes mellitus. NURSING CLINICS OF NORTH AMERICA. 2006;41(4):667+.

5426. Ramos-Lopez Omar, Riezu-Boj Jose, Milagro Fermin, Cuervo Marta, Goni Leticia, Alfredo Martinez J. Genetic and nongenetic factors explaining metabolically healthy and unhealthy phenotypes in participants with excessive adiposity: relevance for personalized nutrition. THERAPEUTIC ADVANCES IN ENDOCRINOLOGY AND METABOLISM. 2019;10():.

5427. Tam Wing, Chung Tony Psychosomatic disorders in pregnancy. CURRENT OPINION IN OBSTETRICS \& GYNECOLOGY. 2007;19(2):126-132.

5428. MacCormack TJ, Driedzic WR Mitochondrial ATP-sensitive K(+) channels influence force development and anoxic contractility in a flatfish, yellowtail flounder Limanda ferruginea, but not Atlantic cod Gadus morhua heart. JOURNAL OF EXPERIMENTAL BIOLOGY. 2002;205(10):1411-1418.

5429. Groot Mary, Golden Sherita, Wagner Julie Psychological Conditions in Adults With Diabetes. AMERICAN PSYCHOLOGIST. 2016;71(7, SI):552-562.

5430. Mathews Louise, Moodie Marj, Simmons Annie, Swinburn Boyd The process evaluation of It's Your Move!, an Australian adolescent community-based obesity prevention project. BMC PUBLIC HEALTH. 2010;10():1-13.

5431. He Zhonghua, Li Mingde, Liu Chanjun, Ma Xiaoyue Common Predictive Factors of Social Media Addiction and Eating Disorder Symptoms in Female College Students: State Anxiety and the Mediating Role of Cognitive Flexibility/Sustained Attention. FRONTIERS IN PSYCHOLOGY. 2022;12():.

5432. Jauregui Lobera I., Romero Candau J., Bolanos Rios P., Montes Berriatua C., Diaz Jaramillo R., Montana Gonzalez Ma., Morales Millan Ma., Leon Lozano P., Martin L., Justo Villalobos I., Vargas Sanchez N. EATING BEHAVIOUR AND BODY IMAGE IN A SAMPLE OF ADOLESCENTS FROM SEVILLA. NUTRICION HOSPITALARIA. 2009;24(5):568-573.

5433. Rhind Charlotte, Salerno Laura, Hibbs Rebecca, Micali Nadia, Schmidt Ulrike, Gowers Simon, Macdonald Pamela, Goddard Elizabeth, Todd Gillian, Tchanturia Kate, Lo Coco Gianluca, Treasure Janet The Objective and Subjective Caregiving Burden and Caregiving Behaviours of Parents of Adolescents with Anorexia Nervosa. EUROPEAN EATING DISORDERS REVIEW. 2016;24(4):310-319.

5434. Herpertz-Dahlmann Beate, Dempfle Astrid, Egberts Karin, Kappel Viola, Konrad Kerstin, Vloet Jennifer, Buehren Katharina Outcome of childhood anorexia nervosaThe results of a five- to ten-year follow-up study. INTERNATIONAL JOURNAL OF EATING DISORDERS. 2018;51(4):295-304.

5435. Lighton John, Schilman Pablo Oxygen Reperfusion Damage in an Insect. PLOS ONE. 2007;2(12):.

5436. Peltzer Karl, Pengpid Supa Prevalence, risk awareness and health beliefs of behavioural risk factors for cardiovascular disease among university students in nine ASEAN countries. BMC PUBLIC HEALTH. 2018;18():.

5437. Scoffier Stephanie, Gernigon Christophe, d'Arripe-Longueville Fabienne Effects of achievement goals on self-regulation of eating attitudes among elite female athletes: An experimental study. PSYCHOLOGY OF SPORT AND EXERCISE. 2012;13(2):201-207.

5438. Zaccari Belle, Lovejoy Travis, O'Neil Maya Skills Training in Affective and Interpersonal Regulation Narrative Therapy Delivered via Synchronous Telehealth: A Case Study of a Rural Woman Veteran With Complex Posttraumatic Stress Disorder. CLINICAL CASE STUDIES. 2023;22(4):420-435.

5439. Kovacs Michelle, Correa John, Brandon Thomas Smoking as Alternative to Eating Among Restrained Eaters: Effect of Food Prime on Young Adult Female Smokers. HEALTH PSYCHOLOGY. 2014;33(10):1174-1184.

5440. Mendes Ana, Ferreira Claudia, Trindade Ines The central role of self-reassurance to explain body and eating attitudes. EATING AND WEIGHT DISORDERS-STUDIES ON

ANOREXIA BULIMIA AND OBESITY. 2019;24(5):861-868.

5441. Bode Ann, Dong Zigang, Wang Hongyang Cancer prevention and control: alarming challenges in China. NATIONAL SCIENCE REVIEW. 2016;3(1):117-127.

5442. Jauregui-Lobera Ignacio, Ezquerra-Cabrera Mercedes, Carbonero-Carreno Rocio, Ruiz-Prieto Inmaculada Weight Misperception, Self-Reported Physical Fitness, Dieting and Some Psychological Variables as Risk Factors for Eating Disorders. NUTRIENTS. 2013;5(11):4486-4502.

5443. Danilenko Konstantin, Plisov Igor, Hebert Marc, Kraeuchi Kurt, Wirz-Justice Anna Influence of timed nutrient diet on depression and light sensitivity in seasonal affective disorder. CHRONOBIOLOGY INTERNATIONAL. 2008;25(1):51-64.

5444. Garcia-Garcia Isabel, Neseliler Selin, Morys Filip, Dadar Mahsa, Yau Yvonne, Scala Stephanie, Zeighami Yashar, Sun Natalie, Collins D., Vainik Uku, Dagher Alain Relationship between impulsivity, uncontrolled eating and body mass index: a hierarchical model. INTERNATIONAL JOURNAL OF OBESITY. 2022;46(1):129-136.

5445. Kempel A, Friedrich M, Schluter KD, Forssmann WG, Kuhn M, Piper HM ANP protects against reoxygenation-induced hypercontracture in adult cardiomyocytes. AMERICAN JOURNAL OF PHYSIOLOGY-HEART AND CIRCULATORY PHYSIOLOGY. 1997;273(1):H244-H249.

5446. Halvorsen I, Heyerdahl S Girls with anorexia nervosa as young adults: Personality, self-esteem, and life satisfaction. INTERNATIONAL JOURNAL OF EATING DISORDERS. 2006;39(4):285-293.

5447. Roquette R., Painho M., Nunes B. Geographical patterns of the incidence and mortality of colorectal cancer in mainland Portugal municipalities (2007-2011). BMC CANCER. 2019;19():.

5448. Riva G, Alcaniz M, Anolli L, Bacchetta M, Banos R, Beltrame F, Botella C, Galimberti C, Gamberini L, Gaggioli A, Molinari E, Mantovani G, Nugues P, Optale G, Orsi G, Perpina C, Troiani R The VEPSY Updated project: Virtual reality in clinical psychology. CYBERPSYCHOLOGY \& BEHAVIOR. 2001;4(4):449-455.

5449. Peeters Anna Obesity and the future of food policies that promote healthy diets. NATURE REVIEWS ENDOCRINOLOGY. 2018;14(7):430-437.

5450. Ochiai H., Shirasawa T., Nanri H., Nishimura R., Hoshino H., Kokaze A. Influence of eating quickly and eating until full on anthropometric gains in girls: A population-based, longitudinal study. CHILD CARE HEALTH AND DEVELOPMENT. 2017;43(6):918-925.

5451. Kirti Kirti, Singh Shri Quantifying the burden of lipid anomalies among adolescents in India. BMC CARDIOVASCULAR DISORDERS. 2022;22(1):.

5452. Jost Adam, Bachan Aviv, Schootbrugge Bas, Lau Kimberly, Weaver Karrie, Maher Kate, Payne Jonathan Uranium isotope evidence for an expansion of marine anoxia during the end-Triassic extinction. GEOCHEMISTRY GEOPHYSICS GEOSYSTEMS. 2017;18(8):3093-3108.

5453. Ott J., Ullrich A., Mascarenhas M., Stevens G. Global cancer incidence and mortality caused by behavior and infection. JOURNAL OF PUBLIC HEALTH. 2011;33(2):223-233.

5454. George Jessica, Franko Debra Cultural Issues in Eating Pathology and Body Image Among Children and Adolescents. JOURNAL OF PEDIATRIC PSYCHOLOGY. 2010;35(3):231-242.

5455. Kerr-Correa Florence, Igami Thais, Hiroce Vivian, Tucci Adriana Patterns of alcohol use between genders: A cross-cultural evaluation. JOURNAL OF AFFECTIVE DISORDERS. 2007;102(1-3):265-275.

5456. Kolar David, Hammerle Florian, Jenetzky Ekkehart, Huss Michael Smartphone-Enhanced Low-Threshold Intervention for adolescents with Anorexia Nervosa (SELTIAN) waiting for outpatient psychotherapy: study protocol of a randomised controlled trial. BMJ OPEN. 2017;7(10):.

5457. Lim Soo Association between obesity and COVID-19. JOURNAL OF THE KOREAN MEDICAL ASSOCIATION. 2022;65(7):423-429.

5458. COLLINGS S, KING M 10-YEAR FOLLOW-UP OF 50 PATIENTS WITH BULIMIA-NERVOSA. BRITISH JOURNAL OF PSYCHIATRY. 1994;164():80-87.

5459. Schleider Jessica, Smith Arielle, Ahuvia Isaac Realizing the untapped promise of single-session interventions for eating disorders. INTERNATIONAL JOURNAL OF EATING DISORDERS. 2023;56(5):853-863.

5460. Zorjan Sasa, Schwab Daniela, Schienle Anne The effects of imaginary eating on visual food cue reactivity: An event-related potential study. APPETITE. 2020;153():.

5461. Santangelo Gabriella, Barone Paolo, Trojano Luigi, Vitale Carmine Pathological gambling in Parkinson's disease. A comprehensive review. PARKINSONISM \& RELATED DISORDERS. 2013;19(7):645-653.

5462. Griffiths RA, HaziPavlovic D, ChannonLittle L Are there differences in response to psychological treatment for recruited and nonrecruited bulimic patients?. EUROPEAN EATING DISORDERS REVIEW. 1997;5(2):131-140.

5463. Wang Rongxin, Wang Jing, Hu Shuiqing Study on the relationship of depression, anxiety, lifestyle and eating habits with the severity of reflux esophagitis. BMC GASTROENTEROLOGY. 2021;21(1):.

5464. Cohen Deborah, Knopman Debra Existing Regulatory Approaches to Reducing Exposures to Chemical- and Product-Based Risk and Their Applicability to Diet-Related Chronic Disease. RISK ANALYSIS. 2018;38(10):2041-2054.

5465. Kristoffersen Mhairi, Johnson Catherine, Atkinson Melissa Feasibility and acceptability of video-based microinterventions for eating disorder prevention among adolescents in secondary schools. INTERNATIONAL JOURNAL OF EATING DISORDERS. 2022;55(11):1496-1505.

5466. Geisbuhler TP, Schwager TL, Ervin HD 3-Isobutyl-1-methylxanthine (IBMX) sensitizes cardiac myocytes to anoxia. BIOCHEMICAL PHARMACOLOGY. 2002;63(11):2055-2062.

5467. Crucianelli Laura, Serpell Lucy, Paloyelis Yannis, Ricciardi Lucia, Robinson Paul, Jenkinson Paul, Fotopoulou Aikaterini The effect of intranasal oxytocin on the perception of affective touch and multisensory integration in anorexia nervosa: protocol for a double-blind placebo-controlled crossover study. BMJ OPEN. 2019;9(3):.

5468. Kwast KE, Hand SC Oxygen and pH regulation of protein synthesis in mitochondria from Artemia franciscana embryos. BIOCHEMICAL JOURNAL. 1996;313(1):207-213.

5469. Zhang Hong, Zhao Danyang, Wang Zhanqiang, Zheng Dongming Diazoxide preconditioning alleviates caspase-dependent and caspase-independent apoptosis induced by anoxia-reoxygenation of PC12 cells. JOURNAL OF BIOCHEMISTRY. 2010;148(4):413-421.

5470. Kagaruki Gibson, Mahande Michael, Kreppel Katharina, Mbata Doris, Kilale Andrew, Shayo Elizabeth, Mfinanga Sayoki, Bonfoh Bassirou Barriers to the implementation, uptake and scaling up of the healthy plate model among regular street food consumers: a qualitative inquiry in Dar-es-Salaam city, Tanzania. BMC NUTRITION. 2022;8(1):.

5471. Athanasiadis Dimitrios, Martin Anna, Kapsampelis Panagiotis, Monfared Sara, Stefanidis Dimitrios Factors associated with weight regain post-bariatric surgery: a systematic review. SURGICAL ENDOSCOPY AND OTHER INTERVENTIONAL TECHNIQUES. 2021;35(8):4069-4084.

5472. Johnson B., Zarnowiecki D., Hendrie G., Mauch C., Golley R. How to reduce parental provision of unhealthy foods to 3-to 8-year-old children in the home environment? A systematic review utilizing the Behaviour Change Wheel framework. OBESITY REVIEWS. 2018;19(10):1359-1370.

5473. Wright Breanne, Gletsu-Miller Nana Iron Nutrition following Bariatric Surgery. BARIATRIC SURGICAL PRACTICE AND PATIENT CARE. 2015;10(1):3-11.

5474. Li Yanping, Ley Sylvia, Tobias Deirdre, Chiuve Stephanie, VanderWeele Tyler, Rich-Edwards Janet, Curhan Gary, Willett Walter, Manson JoAnn, Hu Frank, Qi Lu Birth weight and later life adherence to unhealthy lifestyles in predicting type 2 diabetes: prospective cohort study. BMJ-BRITISH MEDICAL JOURNAL. 2015;351():.

5475. Laroche D., Godin O., Dansou Y., Belzeaux R., Aouizerate B., Burte T., Courtet P., Dubertret C., Haffen E., Llorca P., Olie E., Roux P., Polosan M., Schwan R., Leboyer M., Bellivier F., Marie-Claire C., Etain B., Collaborators FACE-BD Influence of childhood maltreatment on prevalence, onset, and persistence of psychiatric comorbidities and suicide attempts in bipolar disorders. EUROPEAN PSYCHIATRY. 2022;65(1):.

5476. Li Mingqi, Li Yan Popularity Status Insecurity as a Risk Factor for Adolescents' Maladaptive Weight-Related Cognitions and Behaviors: Examining a Moderated Mediation

Model. JOURNAL OF YOUTH AND ADOLESCENCE. 2023;():.

5477. Al-Jawaldeh Ayoub, Hammerich Asmus, Doggui Radhouene, Engesveen Kaia, Lang Krista, McColl Karen Implementation of WHO Recommended Policies and Interventions on Healthy Diet in the Countries of the Eastern Mediterranean Region: From Policy to Action. NUTRIENTS. 2020;12(12):.

5478. Pitcher Grant, Probyn Trevor Anoxia in southern Benguela during the autumn of 2009 and its linkage to a bloom of the dinoflagellate Ceratium balechii. HARMFUL ALGAE. 2011;11():23-32.

5479. Kalsi JS, Arya M, Minhas S, Ralph DJ Priapism: a medical emergency. HOSPITAL MEDICINE. 2002;63(4):224-225.

5480. Laudico Adriano, Mirasol-Lumague Maria, Mapua Cynthia, Uy Gemma, Toral Jean, Medina Victoria, Pukkala Eero Cancer Incidence and Survival in Metro Manila and Rizal Province, Philippines. JAPANESE JOURNAL OF CLINICAL ONCOLOGY. 2010;40(7):603-612.

5481. Gleitz J, Tosch C, Beile A, Peters T The protective action of tetrodotoxin and (+/-)-kavain on anaerobic glycolysis, ATP content and intracellular Na+ and Ca2+ of anoxic brain vesicles. NEUROPHARMACOLOGY. 1996;35(12):1743-1752.

5482. Giguet-Covex Charline, Arnaud Fabien, Poulenard Jerome, Enters Dirk, Reyss Jean-Louis, Millet Laurent, Lazzaroto Jerome, Vidal Olivier Sedimentological and geochemical records of past trophic state and hypolimnetic anoxia in large, hard-water Lake Bourget, French Alps. JOURNAL OF PALEOLIMNOLOGY. 2010;43(1):171-190.

5483. Georgiev Svetoslav, Stein Holly, Hannah Judith, Xu Guangping, Bingen Bernard, Weiss Hermann Timing, duration, and causes for Late Jurassic-Early Cretaceous anoxia in the Barents Sea. EARTH AND PLANETARY SCIENCE LETTERS. 2017;461():151-162.

5484. Allegre A, Silvestre J, Morard P, Kallerhoff J, Pinelli E Nitrate reductase regulation in tomato roots by exogenous nitrate: a possible role in tolerance to long-term root anoxia. JOURNAL OF EXPERIMENTAL BOTANY. 2004;55(408):2625-2634.

5485. Gibbs J, Morrell S, Valdez A, Setter TL, Greenway T Regulation of alcoholic fermentation in coleoptiles of two rice cultivars differing in tolerance to anoxia. JOURNAL

OF EXPERIMENTAL BOTANY. 2000;51(345):785-796.

5486. Anderson Hanna, Johengen Thomas, Miller Russ, Godwin Casey Accelerated sediment phosphorus release in Lake Erie's central basin during seasonal anoxia. LIMNOLOGY AND OCEANOGRAPHY. 2021;66(9):3582-3595.

5487. McCabe Marita, Ricciardelli Lina, Stanford Jacqueline, Holt Kate, Keegan Salley, Miller Louise Where is all the pressure coming from? Messages from mothers and teachers about preschool children's appearance, diet and exercise. EUROPEAN EATING DISORDERS REVIEW. 2007;15(3):221-230.

5488. Ladilov Y, Haffner S, Balser-Schafer C, Maxeiner H, Piper HM Cardioprotective effects of KB-R7943: a novel inhibitor of the reverse mode of Na+/Ca2+ exchanger. AMERICAN JOURNAL OF PHYSIOLOGY-HEART AND CIRCULATORY PHYSIOLOGY. 1999;276(6):H1868-H1876.

5489. Meller Camie, Podrabsky Jason Avoidance of Apoptosis in Embryonic Cells of the Annual Killifish Austrofundulus limnaeus Exposed to Anoxia. PLOS ONE. 2013;8(9):.

5490. Ayala Guadalupe, Ibarra Leticia, Horton Lucy, Arredondo Elva, Slymen Donald, Engelberg Moshe, Rock Cheryl, Hernandez Erika, Parada Humberto, Elder John Evidence Supporting a Promotora-Delivered Entertainment Education Intervention for Improving Mothers' Dietary Intake: The Entre Familia: Reflejos de Salud Study. JOURNAL OF HEALTH COMMUNICATION. 2015;20(2):165-176.

5491. Navet Rachel, Mouithys-Mickalad Ange, Douette Pierre, Sluse-Goffart Claudine, Jarmuszkiewicz Wieslawa, Sluse Francis Proton leak induced by reactive oxygen species produced during in vitro anoxia/reoxygenation in rat skeletal muscle mitochondria. JOURNAL OF BIOENERGETICS AND BIOMEMBRANES. 2006;38(1):23-32.

5492. Sadeh-Sharvit Shiri, Levy-Shiff Rachel, Feldman Talya, Ram Anca, Gur Eitan, Zubery Eynat, Steiner Evelyne, Latzer Yael, Lock James Child feeding perceptions among mothers with eating disorders. APPETITE. 2015;95():67-73.

5493. Schild L, Plumeyer F, Reinheckel T, Augustin W Micromolar calcium prevents isolated rat liver mitochondria from anoxia-reoxygenation injury. BIOCHEMISTRY AND MOLECULAR BIOLOGY INTERNATIONAL. 1997;43(1):35-45.

5494. Viglione Valentina, Muratori Filippo, Maestro Sandra, Brunori Elena, Picchi Lara Denial of symptoms and psychopathology in adolescent anorexia nervosa. PSYCHOPATHOLOGY. 2006;39(5):255-260.

5495. Friedmann MS, McDermut WH, Solomon DA, Ryan CE, Keitner GI, Miller IW Family functioning and mental illness: A comparison of psychiatric and nonclinical families. FAMILY PROCESS. 1997;36(4):357-367.

5496. Cooper M., Lin A., Alvares G., De Klerk N., Jones T., Davis E. Psychiatric disorders during early adulthood in those with childhood onset type 1 diabetes: Rates and clinical risk factors from population-based follow-up. PEDIATRIC DIABETES. 2017;18(7):599-606.

5497. Lichtenberg Mads, Line Laura, Schrameyer Verena, Jakobsen Tim, Rybtke Morten, Toyofuku Masanori, Nomura Nobuhiko, Kolpen Mette, Tolker-Nielsen Tim, Kuhl Michael, Bjarnsholt Thomas, Jensen Peter Nitric-oxide-driven oxygen release in anoxic Pseudomonas aeruginosa. ISCIENCE. 2021;24(12):.

5498. Gamede Mlindeli, Mabuza Lindokuhle, Ngubane Phikelelani, Khathi Andile Plant-Derived Oleanolic Acid (OA) Ameliorates Risk Factors of Cardiovascular Diseases in a Diet-Induced Pre-Diabetic Rat Model: Effects on Selected Cardiovascular Risk Factors. MOLECULES. 2019;24(2):.

5499. Howell Michael, Schenck Carlos Treatment of nocturnal eating disorders. CURRENT TREATMENT OPTIONS IN NEUROLOGY. 2009;11(5):333-339.

5500. Maugeri Andrea, Medina-Inojosa Jose, Kunzova Sarka, Agodi Antonella, Barchitta Martina, Sochor Ondrej, Lopez-Jimenez Francisco, Geda Yonas, Vinciguerra Manlio Sleep Duration and Excessive Daytime Sleepiness Are Associated with Obesity Independent of Diet and Physical Activity. NUTRIENTS. 2018;10(9):.

5501. Rentsch Maria, Ossum Carlo, Hoffmann Else, Pedersen Stine Roles of Na+/H+ exchange in regulation of p38 mitogen-activated protein kinase activity and cell death after chemical anoxia in NIH3T3 fibroblasts. PFLUGERS ARCHIV-EUROPEAN JOURNAL OF PHYSIOLOGY. 2007;454(4):649-662.

5502. Constant Aymery, Conserve Donaldson, Gallopel-Morvan Karine, Raude Jocelyn Socio-Cognitive Factors Associated With Lifestyle Changes in Response to the COVID-19 Epidemic in the General Population: Results From a Cross-Sectional Study in France. FRONTIERS IN

PSYCHOLOGY. 2020;11():.

5503. Baldur-Felskov B., Kjaer S., Albieri V., Steding-Jessen M., Kjaer T., Johansen C., Dalton S., Jensen A. Psychiatric disorders in women with fertility problems: results from a large Danish register-based cohort study. HUMAN REPRODUCTION. 2013;28(3):683-690.

5504. Poulose Shibu, Miller Marshall, Shukitt-Hale Barbara Role of Walnuts in Maintaining Brain Health with Age. JOURNAL OF NUTRITION. 2014;144(4):561S-566S.

5505. Thomas Justin, O'Hara Lily, Tahboub-Schulte Sabrina, Grey Ian, Chowdhury Nayeefa Holy anorexia: Eating disorders symptomatology and religiosity among Muslim women in the United Arab Emirates. PSYCHIATRY RESEARCH. 2018;260():495-499.

5506. Diedrichs Phillippa, Ranson Kristin, Thomas Jennifer Innovation in eating disorders research and practice: Expanding our community and perspectives at the 2018 International Conference on Eating Disorders: Editorial to accompany IJED Virtual Issue in honor of the 2018 International Conference on Eating Disorders.. INTERNATIONAL JOURNAL OF EATING DISORDERS. 2018;51(6):585-587.

5507. Carpita Barbara, Muti Dario, Cremone Ivan, Fagiolini Andrea, Dell'Osso Liliana Eating disorders and autism spectrum: links and risks. CNS SPECTRUMS. 2022;27(3):272-280.

5508. Wignall PB, Newton R, Brookfield ME Pyrite framboid evidence for oxygen-poor deposition during the Permian-Triassic crisis in Kashmir. PALAEOGEOGRAPHY PALAEOCLIMATOLOGY PALAEOECOLOGY. 2005;216(3-4):183-188.

5509. Alcaraz-Ibanez Manuel, Sicilia Alvaro, Diez-Fernandez David, Paterna Adrian Physical appearance comparisons and symptoms of disordered eating: The mediating role of social physique anxiety in Spanish adolescents. BODY IMAGE. 2020;32():145-149.

5510. Doreswamy Shriya, Bashir Anam, Guarecuco Jesus, Lahori Simmy, Baig Ayesha, Narra Lakshmi, Patel Pinal, Heindl Stacey Effects of Diet, Nutrition, and Exercise in Children With Autism and Autism Spectrum Disorder: A Literature Review. CUREUS. 2020;12(12):.

5511. Alvarenga Marle, Scagliusi Fernanda, Philippi Sonia Eating disorders risk behavior in Brazilian female university students. REVISTA DE PSIQUIATRIA CLINICA. 2011;38(1):3-7.

5512. Halvorsen Inger, Heyerdahl Sonja Treatment perception in adolescent onset anorexia nervosa: Retrospective views of patients and parents. INTERNATIONAL JOURNAL OF EATING DISORDERS. 2007;40(7):629-639.

5513. Munguia Lucero, Jimenez-Murcia Susana, Valenciano-Mendoza Eduardo, Granero Roser, Gaspar-Perez Anahi, Guzman-Saldana Rebeca, Sanchez-Gutierrez Manuel, Fazia Gilda, Galvez Laura, Gearhardt Ashley, Fernandez-Aranda Fernando Risk patterns in food addiction: a Mexican population approach. EATING AND WEIGHT DISORDERS-STUDIES ON ANOREXIA BULIMIA AND OBESITY. 2022;27(3):1077-1087.

5514. Coniglio Kathryn, Christensen Kara, Haynos Ann, Rienecke Renee, Selby Edward The posited effect of positive affect in anorexia nervosa: Advocating for a forgotten piece of a puzzling disease. INTERNATIONAL JOURNAL OF EATING DISORDERS. 2019;52(9):971-976.

5515. Abascal L, Brown JB, Winzelberg AJ, Dev P, Taylor CB Combining universal and targeted prevention for school-based eating disorder programs. INTERNATIONAL JOURNAL OF EATING DISORDERS. 2004;35(1):1-9.

5516. Stice Eric, Marti Nathan, Shaw Heather, O'Neil Kelly General and Program-Specific Moderators of Two Eating Disorder Prevention Programs. INTERNATIONAL JOURNAL OF EATING DISORDERS. 2008;41(7):611-617.

5517. Pilatti Angelina, Caneto Florencia, Camerano Echavarria Maria, Verde Maria, Pautassi Ricardo Drinking before going out: prepartying in Argentinean adolescents and its association with drinking norms and prepartying motives. INTERDISCIPLINARIA. 2021;38(1):23-40.

5518. Braet C, Tanghe A, De Bode P, Franckx H, Van Winckel M Inpatient treatment of obese children: a multicomponent programme without stringent calorie restriction. EUROPEAN JOURNAL OF PEDIATRICS. 2003;162(6):391-396.

5519. Tyul'kova EI, Semenov DG, Samoilov MO Role of calcium and phosphoinositide cell regulatory system in adaptation of neurons in olfactory cortex section to in vitro hypoxia. BULLETIN OF EXPERIMENTAL BIOLOGY AND MEDICINE. 1998;125(3):227-229.

5520. FRIEDMAN JE, HADDAD GG REMOVAL OF EXTRACELLULAR-SODIUM PREVENTS ANOXIA-INDUCED INJURY IN FRESHLY DISSOCIATED RAT CA1 HIPPOCAMPAL-NEURONS.

BRAIN RESEARCH. 1994;641(1):57-64.

5521. Ruitenbeek Peter, Quaedflieg Conny, Hernaus Dennis, Hartogsveld Bart, Smeets Tom Dopaminergic and noradrenergic modulation of stress-induced alterations in brain activation associated with goal-directed behaviour. JOURNAL OF PSYCHOPHARMACOLOGY. 2021;35(12):1449-1463.

5522. Fekih-Romdhane Feten, Cheour Majda A rare case report of teen-onset pica in a female patient with a clinical high risk for psychosis. EARLY INTERVENTION IN PSYCHIATRY. 2022;16(7):808-811.

5523. Young EC, DurantJones L Gradual onset of dysphagia: A study of patients with oculopharyngeal muscular dystrophy. DYSPHAGIA. 1997;12(4):196-201.

5524. He Yupeng, Tanaka Ayako, Kishi Taro, Li Yuanying, Matsunaga Masaaki, Tanihara Shinichi, Iwata Nakao, Ota Atsuhiko Recent findings on subjective well-being and physical, psychiatric, and social comorbidities in individuals with schizophrenia: A literature review. NEUROPSYCHOPHARMACOLOGY REPORTS. 2022;42(4):430-436.

5525. Kegler Michelle, Alcantara Iris, Haardoerfer Regine, Gazmararian Julie, Ballard Denise, Sabbs Darrell The Influence of Home Food Environments on Eating Behaviors of Overweight and Obese Women. JOURNAL OF NUTRITION EDUCATION AND BEHAVIOR. 2014;46(3):188-196.

5526. Austin S., Spadano-Gasbarro Jennifer, Greaney Mary, Blood Emily, Hunt Anne, Richmond Tracy, Wang Monica, Mezgebu Solomon, Osganian Stavroula, Peterson Karen Effect of the Planet Health Intervention on Eating Disorder Symptoms in Massachusetts Middle Schools, 2005-2008. PREVENTING CHRONIC DISEASE. 2012;9():.

5527. Riggs Abigail, Giuliano Traci Running in the family or swimming in the gene pool - Discriminating between family history and genetic risk in illness perceptions. JOURNAL OF HEALTH PSYCHOLOGY. 2007;12(6):883-894.

5528. Buerger Arne, Ernst Verena, Wolter Vanessa, Huss Michael, Kaess Michael, Hammerle Florian Treating eating disorders in the real world - MaiStep: A skill-based universal prevention for schools. PREVENTIVE MEDICINE. 2019;123():324-332.

5529. Maalouf Elise, Hallit Souheil, Salameh Pascale, Hosseini Hassan Eating Behaviors, Lifestyle, and Ischemic Stroke: A Lebanese Case-Control Study. INTERNATIONAL JOURNAL OF ENVIRONMENTAL RESEARCH AND PUBLIC HEALTH. 2023;20(2):.

5530. Rodrigues Tiago, Matafome Paulo, Sereno Jose, Almeida Jose, Castelhano Joao, Gamas Luis, Neves Christian, Goncalves Sonia, Carvalho Catarina, Arslanagic Amina, Wilcken Elinor, Fonseca Rita, Simoes Ilda, Conde Silvia, Castelo-Branco Miguel, Seica Raquel Methylglyoxal-induced glycation changes adipose tissue vascular architecture, flow and expansion, leading to insulin resistance. SCIENTIFIC REPORTS. 2017;7():.

5531. Verger Pierre, Lions Caroline, Ventelou Bruno Is depression associated with health risk-related behaviour clusters in adults?. EUROPEAN JOURNAL OF PUBLIC HEALTH. 2009;19(6):618-624.

5532. Chiba Mitsuro, Morita Norikazu Incorporation of Plant-Based Diet Surpasses Current Standards in Therapeutic Outcomes in Inflammatory Bowel Disease. METABOLITES. 2023;13(3):.

5533. Brown Amanda, Avena Nicole, Hoebel Bartley A high-fat diet prevents and reverses the development of activity-based anorexia in rats. INTERNATIONAL JOURNAL OF EATING DISORDERS. 2008;41(5):383-389.

5534. DeBate R., Cragun D., Gallentine A., Severson H., Shaw T., Cantwell C., Christiansen S., Koerber A., Hendricson W., Tomar S., Brown K., Tedesco L. Evaluate, assess, treat: development and evaluation of the EAT framework to increase effective communication regarding sensitive oral-systemic health issues. EUROPEAN JOURNAL OF DENTAL EDUCATION. 2012;16(4):232-238.

5535. Yang Li-Chen, Lan Yan, Hu Jing, Yang Yan-Hua, Zhang Qian, Huang Zhen-Wu, Piao Jian-Hua Relatively High Bone Mineral Density in Chinese Adolescent Dancers Despite Lower Energy Intake and Menstrual Disorder. BIOMEDICAL AND ENVIRONMENTAL SCIENCES. 2010;23(2):130-136.

5536. Isei Michael, Chinnappareddy Nirmala, Stevens Don, Kamunde Collins Anoxia-reoxygenation alters H2O2 efflux and sensitivity of redox centers to copper in heart mitochondria. COMPARATIVE BIOCHEMISTRY AND PHYSIOLOGY C-TOXICOLOGY \& PHARMACOLOGY. 2021;248():.

5537. Mirzaei Mohsen, Mirzaei Masoud, Sarsangi Ali, Bagheri Nasser Prevalence of modifiable cardiovascular risk factors in Yazd inner-city municipalities. BMC PUBLIC HEALTH. 2020;20(1):.

5538. Germain V, Raymond P, Ricard B Differential expression of two tomato lactate dehydrogenase genes in response to oxygen deficit. PLANT MOLECULAR BIOLOGY. 1997;35(6):711-721.

5539. Limbers Christine, Cohen L., Gray Bethany Eating disorders in adolescent and young adult males: prevalence, diagnosis, and treatment strategies. ADOLESCENT HEALTH MEDICINE AND THERAPEUTICS. 2018;9():111-116.

5540. Kulik A, Trapp S, Ballanyi K Ischemia but not anoxia evokes vesicular and Ca2+-independent glutamate release in the dorsal vagal complex in vitro. JOURNAL OF NEUROPHYSIOLOGY. 2000;83(5):2905-2915.

5541. DEVAUD C, MICHAUD PA, NARRING F ANOREXIA AND BULIMIA-NERVOSA - DO THEIR INCIDENCE AND PREVALENCE INCREASE - A REVIEW OF THE EPIDEMIOLOGY OF EATING DISORDERS. REVUE D EPIDEMIOLOGIE ET DE SANTE PUBLIQUE. 1995;43(4):347-360.

5542. Carson Traci, West Brady, Sonneville Kendrin, Zernicke Ronald, Clarke Philippa, Harlow Sioban, Karvonen-Gutierrez Carrie Identifying latent classes of Relative Energy Deficiency in Sport (RED-S) consequences in a sample of collegiate female cross country runners. BRITISH JOURNAL OF SPORTS MEDICINE. 2022;():.

5543. West RV The female athlete - The triad of disordered eating, amenorrhoea and osteoporosis. SPORTS MEDICINE. 1998;26(2):63-71.

5544. Pike KM, Walsh BT Ethnicity and eating disorders: Implications for incidence and treatment. PSYCHOPHARMACOLOGY BULLETIN. 1996;32(2):265-274.

5545. Pujia Roberta, Ferro Yvelise, Maurotti Samantha, Khoory Janin, Gazzaruso Carmine, Pujia Arturo, Montalcini Tiziana, Mazza Elisa The Effects of COVID-19 on the Eating Habits of Children and Adolescents in Italy: A Pilot Survey Study. NUTRIENTS. 2021;13(8):.

5546. Punjani Nahid, Flannigan Ryan, Oliffe John, McCreary Donald, Black Nick, Goldenberg S. Unhealthy Behaviors Among Canadian Men Are Predictors of Comorbidities: Implications for Clinical Practice. AMERICAN JOURNAL OF MENS HEALTH. 2018;12(6):2183-2193.

5547. Jennings Ashley, LeBlanc Hayley, Kisch Karli, Lancaster Steven, Allen Jill Blurred boundaries between Pro-Anorexia and Fitspiration media? Diverging cognitive and emotional effects. EATING DISORDERS. 2021;29(6):580-590.

5548. Belc Nastasia, Smeu Irina, Macri Adriana, Vallauri Dario, Flynn Katherine Reformulating foods to meet current scientific knowledge about salt, sugar and fats. TRENDS IN FOOD SCIENCE \& TECHNOLOGY. 2019;84(SI):25-28.

5549. Burnette C., Luzier Jessica, Weisenmuller Chantel, Boutte Rachel A systematic review of sociodemographic reporting and representation in eating disorder psychotherapy treatment trials in the United States. INTERNATIONAL JOURNAL OF EATING DISORDERS. 2022;55(4):423-454.

5550. Mohammadi Soheil, Dolatshahi Mahsa, Zare-Shahabadi Ameneh, Rahmani Farzaneh Untangling narcolepsy and diabetes: Pathomechanisms with eyes on therapeutic options. BRAIN RESEARCH. 2019;1718():212-222.

5551. Shafi Zarina, Arif Shireen, Nasir Alia Association of Long Duty Hours and Unhealthy Dietary Habits among Nurses at Private and Public Sector in Karachi, Pakistan. JOURNAL OF THE LIAQUAT UNIVERSITY OF MEDICAL AND HEALTH SCIENCES. 2020;19(1):55-61.

5552. Vazquez M., Jimenez E., Nieto J., Sanchez J., Garcia A., Torres J. Development of a Mobile Health Architecture to Prevent Childhood Obesity. IEEE LATIN AMERICA TRANSACTIONS. 2015;13(5):1520-1527.

5553. Ponziani Maria, Karamouzis Ioannis, Mele Chiara, Chasseur Luisa, Zavattaro Marco, Caputo Marina, Sama Maria, Busti Arianna, Pagano Loredana, Castello Luigi, Marzullo Paolo, Aimaretti Gianluca, Prodam Flavia Baseline glucose homeostasis predicts the new onset of diabetes during statin therapy: A retrospective study in real life. HORMONES-INTERNATIONAL JOURNAL OF ENDOCRINOLOGY AND METABOLISM. 2017;16(4):396-404.

5554. Hubin-Gayte Mylene, Squires Claire Study of the impact of pregnancy on eating behaviors and aspects of weight concern by using the SCOFF questionnaire. EVOLUTION

PSYCHIATRIQUE. 2012;77(2):201-212.

5555. Taylor Veronique, Moseley Isabelle, Sun Shufang, Smith Ryan, Roy Alexandra, Ludwig Vera, Brewer Judson Awareness drives changes in reward value which predict eating behavior change: Probing reinforcement learning using experience sampling from mobile mindfulness training for maladaptive eating. JOURNAL OF BEHAVIORAL ADDICTIONS. 2021;10(3):482-497.

5556. Campbell Jacob, Werkhoven Simon, Harrison Jon Metabolomics of anoxia tolerance in Drosophila melanogaster: evidence against substrate limitation and for roles of protective metabolites and paralytic hypometabolism. AMERICAN JOURNAL OF PHYSIOLOGY-REGULATORY INTEGRATIVE AND COMPARATIVE PHYSIOLOGY. 2019;317(3):R442-R450.

5557. Hollman Jay Nutrition in Science and Scripture. PERSPECTIVES ON SCIENCE AND CHRISTIAN FAITH. 2020;72(3):144-150.

5558. Strasser Lauren, Wilson Mitchell, Healy Sarah, Doja Asif Tremor Presenting in Infants and Children Aged <2 Years. JOURNAL OF CHILD NEUROLOGY. 2022;37(4):298-302.

5559. Oldfield Christopher, Moffatt Teri, O'Hara Kimberley, Xiang Bo, Dolinsky Vernon, Duhamel Todd Muscle-specific sirtuin 3 overexpression does not attenuate the pathological effects of high-fat/high-sucrose feeding but does enhance cardiac SERCA2a activity. PHYSIOLOGICAL REPORTS. 2021;9(16):.

5560. Joos A, Steinert T Comorbidity of schizophrenia and anorexia nervosa, bulimic type. A forensic case study. NERVENARZT. 1997;68(5):417-420.

5561. Kelishadi Roya, Motlagh Mohammad, Roomizadeh Peyman, Abtahi Seyed-Hossein, Qorbani Mostafa, Taslimi Mahnaz, Heshmat Ramin, Aminaee Tahereh, Ardalan Gelayol, Poursafa Parinaz, Karimi Mehrdad First Report on Path Analysis for Cardiometabolic Components in a Nationally Representative Sample of Pediatric Population in the Middle East and North Africa (MENA): The CASPIAN-III Study. ANNALS OF NUTRITION AND METABOLISM. 2013;62(3):257-265.

5562. Magson Natasha, Oar Ella, Fardouly Jasmine, Johnco Carly, Rapee Ronald The Preteen Perfectionist: An Evaluation of the Perfectionism Social Disconnection Model. CHILD PSYCHIATRY \& HUMAN DEVELOPMENT. 2019;50(6):960-974.

5563. Ouhtit Allal, Al-Sharbati Marvvan, Gupta Ishita, Al-Farsi Yahya Potato chips and childhood: What does the science say? An unrecognized threat?. NUTRITION. 2014;30(10):1110-1112.

5564. Larsson JO, Bergman LR, Earls F, Rydelius PA Behavioral profiles in 4-5 year-old children: Normal and pathological variants. CHILD PSYCHIATRY \& HUMAN DEVELOPMENT. 2004;35(2):143-162.

5565. Claydon Elizabeth, Davidov Danielle, DeFazio Caterina, Zullig Keith, Ward Rose, Smith Kathryn The Relationship Between Sexual Assault, Intimate Partner Violence, and Eating Disorder Symptomatology Among College Students. VIOLENCE AND VICTIMS. 2022;37(1):63-76.

5566. Berlin Kristoffer, Davies W., Lobato Debra, Silverman Alan A Biopsychosocial Model of Normative and Problematic Pediatric Feeding. CHILDRENS HEALTH CARE. 2009;38(4):263-282.

5567. Haghighatdoost Fahimeh, Riahi Roya, Safari Shahla, Heidari Zahra Dose-response association between dietary patterns and gestational diabetes mellitus risk: A systematic review and meta-analysis of observational studies. FOOD SCIENCE \& NUTRITION. 2023;11(1):57-92.

5568. Clague Caitlin, Prnjak Katarina, Mitchison Deborah ?I don't want them to judge me?: Separating out the role of fear of negative evaluation, neuroticism, and low self-esteem in eating disorders. EATING BEHAVIORS. 2023;49():.

5569. Buzalaf M., Magalhaes A., Rios D. Prevention of erosive tooth wear: targeting nutritional and patient-related risks factors. BRITISH DENTAL JOURNAL. 2018;224(5):.

5570. Nasui Bogdana, Ungur Rodica, Talaba Patricia, Varlas Valentin, Ciuciuc Nina, Silaghi Cristina, Silaghi Horatiu, Opre Dana, Pop Anca Is Alcohol Consumption Related to Lifestyle Factors in Romanian University Students?. INTERNATIONAL JOURNAL OF ENVIRONMENTAL RESEARCH AND PUBLIC HEALTH. 2021;18(4):.

5571. Cavazos-Rehg Patricia, Krauss Melissa, Costello Shaina, Kaiser Nina, Cahn Elizabeth, Fitzsimmons-Craft Ellen, Wilfley Denise ``I just want to be skinny.{''}: A content analysis of tweets expressing eating disorder symptoms. PLOS ONE. 2019;14(1):.

5572. Recio-Barbero Maria, Fuertes-Soriano Sara, Cabezas-Garduno Janire, Lopez-Atanes Mayte, Pena-Rotella Alvar, Saenz-Herrero Margarita Delayed Diagnosis of an Eating Disorder in a Male Patient With Superior Mesenteric Artery Syndrome: Results From a Case Study. FRONTIERS IN PSYCHIATRY. 2019;10():.

5573. Casasnovas Arielle, Huryk Kathryn, Levinson Devorah, Markowitz Sara, Friedman Shoshana, Stice Eric, Loeb Katharine Cognitive dissonance-based eating disorder prevention: pilot study of a cultural adaptation for the Orthodox Jewish community. EATING DISORDERS. 2021;29(2):192-204.

5574. Miguel-Berges Maria, Zachari Konstantina, Santaliestra-Pasias Alba, Mouratidou Theodora, Androutsos Odysseas, Iotova Violeta, Galcheva Sonya, De Craemer Marieke, Cardon Greet, Koletzko Berthold, Kulaga Zbigniew, Manios Yannis, Moreno Luis, Grp ToyBox-Study Clustering of energy balance-related behaviours and parental education in European preschool children: the ToyBox study. BRITISH JOURNAL OF NUTRITION. 2017;118(12):1089-1096.

5575. Nosarti Chiara, Reichenberg Abraham, Murray Robin, Cnattingius Sven, Lambe Mats, Yin Li, MacCabe James, Rifkin Larry, Hultman Christina Preterm Birth and Psychiatric Disorders in Young Adult Life. ARCHIVES OF GENERAL PSYCHIATRY. 2012;69(6):610-617.

5576. Poston Sara, Dickson Michael, Johnsrud Michael, Rupnow Marcia, Gdovin Joette, Bramley Thomas, Armstrong Robert Topiramate prescribing patterns among medicaid patients: Diagnosis, comorbidities, and dosing. CLINICAL THERAPEUTICS. 2007;29(3):504-518.

5577. Tadege M, Brandle R, Kuhlemeier C Anoxia tolerance in tobacco roots: effect of overexpression of pyruvate decarboxylase. PLANT JOURNAL. 1998;14(3):327-335.

5578. Litwin Rachel, Goldbacher Edie, Cardaciotto LeeAnn, Gambrel Laura Negative emotions and emotional eating: the mediating role of experiential avoidance. EATING AND WEIGHT DISORDERS-STUDIES ON ANOREXIA BULIMIA AND OBESITY. 2017;22(1):97-104.

5579. Malova Ekaterina, Dunleavy Victoria Men have eating disorders too: an analysis of online narratives posted by men with eating disorders on YouTube. EATING DISORDERS. 2022;30(4):437-452.

5580. Hill Alison, Zuckerman Katharine, Fombonne Eric Obesity and Autism. PEDIATRICS. 2015;136(6):1051-1061.

5581. Wilksch Simon, Wade Tracey Reduction of Shape and Weight Concern in Young Adolescents: A 30-Month Controlled Evaluation of a Media Literacy Program. JOURNAL OF THE AMERICAN ACADEMY OF CHILD AND ADOLESCENT PSYCHIATRY. 2009;48(6):652-661.

5582. Berg Patricia, Keery Helene, Eisenberg Marla, Neumark-Sztainer Dianne Maternal and Adolescent Report of Mothers' Weight-Related Concerns and Behaviors: Longitudinal Associations with Adolescent Body Dissatisfaction and Weight Control Practices. JOURNAL OF PEDIATRIC PSYCHOLOGY. 2010;35(10):1093-1102.

5583. Rivoal J, Thind S, Pradet A, Ricard B Differential induction of pyruvate decarboxylase subunits and transcripts in anoxic rice seedlings. PLANT PHYSIOLOGY. 1997;114(3):1021-1029.

5584. Wakasugi Minako, Kazama Junichiro, Narita Ichiei, Iseki Kunitoshi, Fujimoto Shouichi, Moriyama Toshiki, Yamagata Kunihiro, Konta Tsuneo, Tsuruya Kazuhiko, Asahi Koichi, Kondo Masahide, Kurahashi Issei, Ohashi Yasuo, Kimura Kenjiro, Watanabe Tsuyoshi Association between Overall Lifestyle Changes and the Incidence of Proteinuria: A Population-based, Cohort Study. INTERNAL MEDICINE. 2017;56(12):1475-1484.

5585. Jiang Zongzhi, Fu Yuxin, Wei Xiaojing, Wang Ziyi, Yu Xuefan Case report: A unusual case of delayed propionic acidemia complicated with subdural hematoma. FRONTIERS IN NEUROLOGY. 2022;13():.

5586. Sheppard Justine, Hochman Roberta, Baer Carolynn The Dysphagia Disorder Survey: Validation of an assessment for swallowing and feeding function in developmental disability. RESEARCH IN DEVELOPMENTAL DISABILITIES. 2014;35(5):929-942.

5587. McVey GL, Davis R, Tweed S, Shaw BF Evaluation of a school-based program designed to improve body image satisfaction, global self-esteem, and eating attitudes and behaviors: A replication study. INTERNATIONAL JOURNAL OF EATING DISORDERS. 2004;36(1):1-11.

5588. Marzola Enrica, Cavallo Fabio, Panero Matteo, Porliod Alain, Amodeo Laura, Abbate-Daga Giovanni The role of prenatal and perinatal factors in eating disorders: a systematic

review. ARCHIVES OF WOMENS MENTAL HEALTH. 2021;24(2):185-204.

5589. Lin Jessica, Chadi Nicholas, Shrier Lydia Mindfulness-based interventions for adolescent health. CURRENT OPINION IN PEDIATRICS. 2019;31(4):469-475.

5590. Santore MT, McClintock DS, Lee VY, Budinger GRS, Chandel NS Anoxia-induced apoptosis occurs through a mitochondria-dependent pathway in lung epithelial cells. AMERICAN JOURNAL OF PHYSIOLOGY-LUNG CELLULAR AND MOLECULAR PHYSIOLOGY. 2002;282(4):L727-L734.

5591. Sandoval Viviana, Femenias Antoni, Martinez-Garza Ursula, Sanz-Lamora Hector, Manuel Castagnini Juan, Quifer-Rada Paola, Maria Lamuela-Raventos Rosa, Marrero Pedro, Haro Diego, Relat Joana Lyophilized Maqui (Aristotelia chilensis) Berry Induces Browning in the Subcutaneous White Adipose Tissue and Ameliorates the Insulin Resistance in High Fat Diet-Induced Obese Mice. ANTIOXIDANTS. 2019;8(9):.

5592. Nielsen S, Emborg C, Molbak AG Mortality in concurrent type 1 diabetes and anorexia nervosa. DIABETES CARE. 2002;25(2):309-312.

5593. Pan Shangha, Liu Lianxin, Pan Huayang, Ma Yong, Wang Dawei, Kang Kai, Wang Jizhou, Sun Bei, Sun Xueying, Jiang Hongchi Protective effects of hydroxytyrosol on liver ischemia/reperfusion injury in mice. MOLECULAR NUTRITION \& FOOD RESEARCH. 2013;57(7):1218-1227.

5594. Villalobos Aremis, Unikel Claudia, Hernandez-Serrato Maria, Bojorquez Ietza Disordered eating in Mexican adolescents, 2006-2018. SALUD PUBLICA DE MEXICO. 2020;62(6):734-744.

5595. Jorge Marcos-Pardo Pablo, Abelleira-Lamela Tomas, Vaquero-Cristobal Raquel, Gonzalez-Galvez Noelia Changes in life satisfaction, depression, general health and sleep quality of Spanish older women during COVID-19 lockdown and their relationship with lifestyle: an observational follow-up study. BMJ OPEN. 2022;12(8):.

5596. Gan Zuo, Cheong Huey, Tu Yu-Kang, Kuo Po-Hsiu Association between Plant-Based Dietary Patterns and Risk of Cardiovascular Disease: A Systematic Review and Meta-Analysis of Prospective Cohort Studies. NUTRIENTS. 2021;13(11):.

5597. Ivarsson T, Rastam M, Wentz E, Gillberg IC, Gillberg C Depressive disorders in teenage-onset anorexia nervosa: A controlled longitudinal, partly community-based study. COMPREHENSIVE PSYCHIATRY. 2000;41(5):398-403.

5598. Torres-McGehee Toni, Leaver-Dunn Deidre, Green James, Bishop Phillip, Leeper James, Richardson Mark KNOWLEDGE OF EATING DISORDERS AMONG COLLEGIATE ADMINISTRATORS, COACHES, AND AUXILIARY DANCERS. PERCEPTUAL AND MOTOR SKILLS. 2011;112(3):951-958.

5599. Carethers John, Doubeni Chyke Causes of Socioeconomic Disparities in Colorectal Cancer and Intervention Framework and Strategies. GASTROENTEROLOGY. 2020;158(2, SI):354-367.

5600. Barras Colin, Twitchett Richard Response of the marine infauna to Triassic-Jurassic environmental change: Ichnological data from southern England. PALAEOGEOGRAPHY PALAEOCLIMATOLOGY PALAEOECOLOGY. 2007;244(1-4):223-241.

5601. Phillips KA, Menard W, Fay C Gender similarities and differences in 200 individuals with body dysmorphic disorder. COMPREHENSIVE PSYCHIATRY. 2006;47(2):77-87.

5602. Gozal D, Torres JE, Gozal YM, Nuckton TJ Characterization and developmental aspects of anoxia-induced gasping in the rat. BIOLOGY OF THE NEONATE. 1996;70(5):280-288.

5603. Shroff Hemal, Thompson J. Peer influences, body-image dissatisfaction, eating dysfunction and self-esteem in adolescent girls. JOURNAL OF HEALTH PSYCHOLOGY. 2006;11(4):533-551.

5604. Brewerton TD Bulimia in children and adolescents. CHILD AND ADOLESCENT PSYCHIATRIC CLINICS OF NORTH AMERICA. 2002;11(2):237+.

5605. Kacar Murat, Hocaoglu Cicek What is pica and rumination disorder? diagnosis and treatment approaches. KLINIK PSIKIYATRI DERGISI-TURKISH JOURNAL OF CLINICAL PSYCHIATRY. 2019;22(3):347-354.

5606. Wilkes Patricia, Allen Deborah Nutrition Care: Managing Symptoms From Cancer. JNP- THE JOURNAL FOR NURSE PRACTITIONERS. 2018;14(4):267+.

5607. Lloyd E., Haase Anne, Zerwas Stephanie, Micali Nadia Anxiety disorders predict fasting to control weight: A longitudinal large cohort study of adolescents. EUROPEAN EATING DISORDERS REVIEW. 2020;28(3):269-281.

5608. Mozaffarian Nafiseh, Heshmat Ramin, Ataie-Jafari Asal, Motlagh Mohammad, Ziaodini Hasan, Shafiee Gita, Taheri Majzoubeh, Mansourian Morteza, Qorbani Mostafa, Kelishadi Roya Association of sleep duration and snack consumption in children and adolescents: The CASPIAN-V study. FOOD SCIENCE \& NUTRITION. 2020;8(4):1888-1897.

5609. Mari-Sanchis Amelia, Burgos-Balmaseda Jose, Hidalgo-Borrajo Rebeca Eating disorders in sport. Update and proposal for an integrated approach. ENDOCRINOLOGIA DIABETES Y NUTRICION. 2022;69(2):131-143.

5610. Dosenko Victor, Nagibin Vasyl, Tumanovska Lesya, Moibenko Alexey Protective effect of autophagy in anoxia-reoxygenation of isolated cardiomyocyte?. AUTOPHAGY. 2006;2(4):305-306.

5611. Carpinelli Luna, Marinaci Tiziana, Savarese Giulia Caring for Daughters with Anorexia Nervosa: A Qualitative Study on Parents' Representation of the Problem and Management of the Disorder. HEALTHCARE. 2022;10(7):.

5612. Davis C, Blackmore E, Katzman DK, Fox J Female adolescents with anorexia nervosa and their parents: a case-control study of exercise attitudes and behaviours. PSYCHOLOGICAL MEDICINE. 2005;35(3):377-386.

5613. Saavedra Melina, Aziz Julieta, Cacchiarelli San Roman Nicolas Scurvy due to restrictive diet in a child with autism spectrum disorder. Case report. ARCHIVOS ARGENTINOS DE PEDIATRIA. 2018;116(5):E684-E687.

5614. Lu Jiahui, Cayabyab Ysa, Malik Shelly, Lwin May The Associations between Mobile Media use and Food Consumption in Parent-Child Dyads. JOURNAL OF CHILD AND FAMILY STUDIES. 2022;31(7):2005-2014.

5615. Lourenco Maria, Azevedo Alvaro, Brandao Isabel, Gomes Pedro Orofacial manifestations in outpatients with anorexia nervosa and bulimia nervosa focusing on the vomiting behavior. CLINICAL ORAL INVESTIGATIONS. 2018;22(5):1915-1922.

5616. Nova Esther, Martinez-Gomez David, Gomez-Martinez Sonia, Veses Ana, Calle Maria, Veiga Oscar, Marcos Ascension Influence of health behaviours on the incidence of infection and allergy in adolescents: the AFINOS cross-sectional study. BMC PUBLIC HEALTH. 2014;14():.

5617. Ghosh Subrata, Das Gouri, Majumdar Rakhi, Sarkar Sabyasachi Nontoxic water soluble nanocarbons prevent respiration of mosquito larvae, causing anoxia. JOURNAL OF VECTOR BORNE DISEASES. 2018;55(2):159-164.

5618. Booth David, Treharne Gareth, Kitas George, Kumar Sudhesh Avoidance of unhealthy fattening: A longstanding proposal. APPETITE. 2007;48(2):129-134.

5619. Vitzthum Karin, Endres Eva, Koch Franziska, Groneberg David, Quarcoo David, Wanke Eileen, Mache Stefanie Eating Behavior and Nutrition Knowledge among Musical Theatre Students. MEDICAL PROBLEMS OF PERFORMING ARTISTS. 2013;28(1):19-23.

5620. GLEITZ J, BEILE A, KHAN S, WILFFERT B, TEGTMEIER F ANAEROBIC GLYCOLYSIS AND POSTANOXIC RECOVERY OF RESPIRATION OF RAT CORTICAL SYNAPTOSOMES ARE REDUCED BY SYNAPTOSOMAL SODIUM LOAD. BRAIN RESEARCH. 1993;611(2):286-294.

5621. Magallares Alejandro Social risk factors related to eating disorders in women. REVISTA LATINOAMERICANA DE PSICOLOGIA. 2013;45(1):147-154.

5622. Penafiel Daniela, Termote Celine, Lachat Carl, Espinel Ramon, Kolsteren Patrick, Van Damme Patrick Barriers to Eating Traditional Foods Vary by Age Group in Ecuador With Biodiversity Loss as a Key Issue. JOURNAL OF NUTRITION EDUCATION AND BEHAVIOR. 2016;48(4):258+.

5623. Corazza Ilaria, Pennucci Francesca, De Rosis Sabina Promoting healthy eating habits among youth according to their preferences: Indications from a discrete choice experiment in Tuscany. HEALTH POLICY. 2021;125(7):947-955.

5624. Di Lodovico L., Vansteene C., Poupon D., Gorwood P., Duriez P., FFAB Food avoidance in anorexia nervosa: associated and predicting factors. EATING AND WEIGHT DISORDERS-STUDIES ON ANOREXIA BULIMIA AND OBESITY. 2023;28(1):.

5625. Treasure Janet, Corfield Freya, Cardi Valentina A Three-phase Model of the Social Emotional Functioning in Eating Disorders. EUROPEAN EATING DISORDERS REVIEW. 2012;20(6, SI):431-438.

5626. Du Yanjiao, Gao Yue, Zeng Bo, Fan Xiaolan, Yang Deying, Yang Mingyao Effects of anti-aging interventions on intestinal microbiota. GUT MICROBES. 2021;13(1):.

5627. Sanchez-Gutierrez Teresa, Barbeito Sara, Calvo Ana A Revision of Preventive Web-based Psychotherapies in Subjects at Risk of Mental Disorders. INTERNATIONAL JOURNAL OF INTERACTIVE MULTIMEDIA AND ARTIFICIAL INTELLIGENCE. 2017;4(5):50-54.

5628. Pallavicini Federica, Serino Silvia, Cipresso Pietro, Pedroli Elisa, Giglioli Irene, Chirico Alice, Manzoni Gian, Castelnuovo Gianluca, Molinari Enrico, Riva Giuseppe Testing Augmented Reality for Cue Exposure in Obese Patients: An Exploratory Study. CYBERPSYCHOLOGY BEHAVIOR AND SOCIAL NETWORKING. 2016;19(2, SI):107-114.

5629. Wick Katharina, Leipold-Haas Steffi, Dye Louise, Strauss Bernhard Attachment, satisfaction with one's own figure and eating behavior in 13-to 18-year-old female and male adolescents. KINDHEIT UND ENTWICKLUNG. 2012;21(4):219-226.

5630. ALPERT JE, MADDOCKS A, ROSENBAUM JF, FAVA M CHILDHOOD PSYCHOPATHOLOGY RETROSPECTIVELY ASSESSED AMONG ADULTS WITH EARLY-ONSET MAJOR DEPRESSION. JOURNAL OF AFFECTIVE DISORDERS. 1994;31(3):165-171.

5631. Bray Molly Implications of Gene-Behavior Interactions: Prevention and Intervention for Obesity. OBESITY. 2008;16(3):S72-S78.

5632. Esposito Katherine, Ciotola Miryam, Maiorino Maria, Giugliano Dario Lifestyle Approach for Type 2 Diabetes and Metabolic Syndrome. CURRENT ATHEROSCLEROSIS REPORTS. 2008;10(6):523-528.

5633. Temple NJ, Balay-Karperien AL Nutrition in cancer prevention: An integrated approach. JOURNAL OF THE AMERICAN COLLEGE OF NUTRITION. 2002;21(2):79-83.

5634. Stice Eric, Rohde Paul, Gau Jeff, Bearman Sarah, Shaw Heather An Experimental Test of Increasing Implementation Support for College Peer Educators Delivering an Evidence-Based Prevention Program. JOURNAL OF CONSULTING AND CLINICAL PSYCHOLOGY.

2023;91(4):208-220.

5635. Kato-Noguchi H Hypoxic induction of anoxia tolerance in Avena coleoptiles. JOURNAL OF PLANT PHYSIOLOGY. 2002;159(7):751-755.

5636. Bratlien Unni, Oie Merete, Haug Elisabeth, Moller Paul, Andreassen Ole, Lien Lars, Melle Ingrid Self-reported symptoms and health service use in adolescence in persons who later develop psychotic disorders: A prospective case-control study. EARLY INTERVENTION IN PSYCHIATRY. 2015;9(3):221-227.

5637. Janner Simone, Suter Valerie, Altermatt Hans, Reichart Peter, Bornstein Michael Bilateral necrotizing sialometaplasia of the hard palate in a patient with bulimia: A case report and review of the literature. QUINTESSENCE INTERNATIONAL. 2014;45(5):431-437.

5638. Kamimura Akiko, Tabler Jennifer, Nourian Maziar, Jess Allison, Stephens Tamara, Aguilera Guadalupe, Wright Lindsey, Ashby Jeanie Promoting Healthy Eating Attitudes Among Uninsured Primary Care Patients. JOURNAL OF COMMUNITY HEALTH. 2016;41(4):805-811.

5639. Pagoto S., Curtin C., Bandini L., Anderson S., Schneider K., Bodenlos J., Ma Y. Weight loss following a clinic-based weight loss program among adults with attention deficit/hyperactivity disorder symptoms. EATING AND WEIGHT DISORDERS-STUDIES ON ANOREXIA BULIMIA AND OBESITY. 2010;15(3):E166-E172.

5640. Zou Liye, Yang Peiying, Herold Fabian, Liu Weina, Szabo Attila, Taylor Alyx, Sun Jing, Ji Liu The Contribution of BMI, Body Image Inflexibility, and Generalized Anxiety to Symptoms of Eating Disorders and Exercise Dependence in Exercisers. INTERNATIONAL JOURNAL OF MENTAL HEALTH PROMOTION. 2022;24(6):811-823.

5641. Grill Sabine, Quante Anne, Kiechle Marion Physical activity and nutrition in the prevention of hereditary breast cancer. GYNAKOLOGE. 2019;52(7):525-528.

5642. Zoletic Emina, Durakovic-Belko Elvira BODY IMAGE DISTORTION, PERFECTIONISM AND EATING DISORDER SYMPTOMS IN RISK GROUP OF FEMALE BALLET DANCERS AND MODELS AND IN CONTROL GROUP OF FEMALE STUDENTS. PSYCHIATRIA DANUBINA. 2009;21(3):302-309.

5643. Ihle J., Artaud F., Bekadar S., Mangone G., Sambin S., Mariani L., Bertrand H., Rascol O., Durif F., Derkinderen P., Scherzer C., Elbaz A., Corvol J., Corvol Jean-Christophe, Elbaz Alexis, Vidailhet Marie, Brice Alexis, Artaud Fanny, Bourdain Frederic, Brandel Jean-Philippe, Derkinderen Pascal, Durif Franck, Levy Richard, Pico Fernando, Rascol Olivier, Bonnet Anne-Marie, Bonnet Cecilia, Brefel-Courbon Christine, Cormier-Dequaire Florence, Degos Bertrand, Debilly Berangere, Galitsky Monique, Grabli David, Hartmann Andreas, Klebe Stephan, Kraemmer Julia, Lacomblez Lucette, Leder Sara, Mangone Graziella, Mariani Louise-Laure, Marques Ana-Raquel, Mesnage Valerie, Muellner Julia, Ory-Magne Fabienne, Plante-Bordeneuve Violaine, Roze Emmanuel, Tir Melissa, You Hana, Benchetrit Eve, Socha Julie, Pineau Fanny, Vidal Tiphaine, Pomies Elsa, Bayet Virginie, Lesage Suzanne, Tahiri Khadija, Bertrand Helene, Mallet Alain, Villeret Coralie, Mazmanian Merry, Manseur Hakima, Hajji Mostafa, Le Toullec Benjamin, Brochard Vanessa, Roy Monica, Rieu Isabelle, Bernard Stephane, Faurie-Grepon Antoine, Comm DIGPD Parkinson's disease polygenic risk score is not associated with impulse control disorders: A longitudinal study. PARKINSONISM \& RELATED DISORDERS. 2020;75():30-33.

5644. Kannan Saranya, Srinivasan Divya, Raghupathy Prasanth, Bhaskaran Ravi Association between duration of obesity and severity of ovarian dysfunction in rat-cafeteria diet approach. JOURNAL OF NUTRITIONAL BIOCHEMISTRY. 2019;71():132-143.

5645. Popa-Wagner Aurel, Dumitrascu Dinu, Capitanescu Bogdan, Petcu Eugen, Surugiu Roxana, Fang Wen-Hui, Dumbrava Danut-Adrian Dietary habits, lifestyle factors and neurodegenerative diseases. NEURAL REGENERATION RESEARCH. 2020;15(3):394-400.

5646. Decamps G., Gana K., Hagger M., Bruchon-Schweitzer M., Boujut E. A study of the relationships between sport practice and health issues among college students: Effects of gender on eating disorders and substance use. PSYCHOLOGIE FRANCAISE. 2016;61(4):361-374.

5647. Wang Der-Shiun, Chung Chi-Hsiang, Chang Hsin-An, Kao Yu-Chen, Chu Der-Ming, Wang Chih-Chien, Chen Shyi-Jou, Tzeng Nian-Sheng, Chien Wu-Chien Association between child abuse exposure and the risk of psychiatric disorders: A nationwide cohort study in Taiwan. CHILD ABUSE \& NEGLECT. 2020;101():.

5648. Jacobi Corinna, Schmitz Gabriele, Agras William Interactions Between Disturbed Eating and Weight in Children and Their Mothers. JOURNAL OF DEVELOPMENTAL AND BEHAVIORAL PEDIATRICS. 2008;29(5):360-366.

5649. Micali Nadia, Ploubidis George, De Stavola Bianca, Simonoff Emily, Treasure Janet Frequency and Patterns of Eating Disorder Symptoms in Early Adolescence. JOURNAL OF ADOLESCENT HEALTH. 2014;54(5):574-581.

5650. Yi Juan, Zheng Yijun, Miao Changhong, Tang Jianguo, Zhu Biao Desflurane Preconditioning Induces Oscillation of NF-kappa B in Human Umbilical Vein Endothelial Cells. PLOS ONE. 2013;8(6):.

5651. KHAZIPOV R, CONGAR P, BENARI Y HIPPOCAMPAL CA1 LACUNOSUM-MOLECULARE INTERNEURONS - COMPARISON OF EFFECTS OF ANOXIA ON EXCITATORY AND INHIBITORY POSTSYNAPTIC CURRENTS. JOURNAL OF NEUROPHYSIOLOGY. 1995;74(5):2138-2149.

5652. CHIRKOVA TV, WALTER G, LEFFLER S, NOVITSKAYA LO CHLOROPLASTS AND MITOCHONDRIA IN THE LEAVES OF WHEAT AND RICE SEEDLINGS EXPOSED TO ANOXIA AND LONG-TERM DARKNESS - SOME CHARACTERISTICS OF ORGANELLE STATE. RUSSIAN JOURNAL OF PLANT PHYSIOLOGY. 1995;42(3):321-329.

5653. Fjermestad K., Ro A., Espeland K., Halvorsen M., Halvorsen I. ``Do I exist in this world, really, or is it just her?{''} Youths' perspectives of living with a sibling with anorexia nervosa. EATING DISORDERS. 2020;28(1):80-95.

5654. Gopinath Bamini, Liew Gerald, Flood Victoria, Joachim Nichole, Burlutsky George, Mitchell Paul Combined influence of poor health behaviours on the prevalence and 15-year incidence of age-related macular degeneration. SCIENTIFIC REPORTS. 2017;7():.

5655. Jafarzadeh Jaber, Payahoo Laleh, Yousefi Mohammad, Barzegar Ali The comprehensive mechanistic insight into the effects of vitamin D on dementia - a review. NUTRITION \& FOOD SCIENCE. 2022;52(4):698-721.

5656. NEUMARKER KJ, STEINHAUSEN HC, DUDECK U, NEUMARKER U, SEIDEL R, REITZLE M EATING DISORDERS IN EAST AND WEST-BERLIN ADOLESCENTS IN THE 1980S. PRAXIS DER KINDERPSYCHOLOGIE UND KINDERPSYCHIATRIE. 1994;43(2):60-68.

5657. Tomiyama A., Mann Traci, Comer Lisa Triggers of eating in everyday life. APPETITE. 2009;52(1):72-82.

5658. Abdurahman Ahmed, Azadbakhat Leila, Rasouli Mahkameh, Chamari Maryam, Qorbani Mostafa, Dorosty Ahmed Association of dietary inflammatory index with metabolic profile in metabolically healthy and unhealthy obese people. NUTRITION \& DIETETICS. 2019;76(2):192-198.

5659. Carter FA, McIntosh VVW, Frampton CM, Joyce PR, Bulik CM Predictors of childbirth following treatment for bulimia nervosa. INTERNATIONAL JOURNAL OF EATING DISORDERS. 2003;34(3):337-342.

5660. Kant R., Wong-Chung Agnes, Evans Elizabeth, Stanton Elaine, Boothroyd Lynda The Impact of a Dissonance-Based Eating Disorders Intervention on Implicit Attitudes to Thinness in Women of Diverse Sexual Orientations. FRONTIERS IN PSYCHOLOGY. 2019;10():.

5661. Nishimi Kristen, Neylan Thomas, Bertenthal Daniel, Seal Karen, O'Donovan Aoife Association of Psychiatric Disorders With Incidence of SARS-CoV-2 Breakthrough Infection Among Vaccinated Adults. JAMA NETWORK OPEN. 2022;5(4):.

5662. FRENCH SA, PERRY CL, LEON GR, FULKERSON JA WEIGHT CONCERNS, DIETING BEHAVIOR, AND SMOKING INITIATION AMONG ADOLESCENTS - A PROSPECTIVE-STUDY. AMERICAN JOURNAL OF PUBLIC HEALTH. 1994;84(11):1818-1820.

5663. Oliveira Galvao Patricia, Valente Juliana, Cogo-Moreira Hugo, Mari Jair, Sanchez Zila Bullying as a Risk Factor for Eating Disorder Behaviors Among Students: Secondary Analysis for a Cluster Randomized Controlled Trial. CHILD PSYCHIATRY \& HUMAN DEVELOPMENT. 2022;():.

5664. Cuzzocrea F., Larcan R., Lanzarone C. Gender differences, personality and eating behaviors in non-clinical adolescents. EATING AND WEIGHT DISORDERS-STUDIES ON ANOREXIA BULIMIA AND OBESITY. 2012;17(4):E282-E289.

5665. Zhang Feifei, Dahl Tais, Lenton Timothy, Luo Genming, Shen Shu-zhong, Algeo Thomas, Planavsky Noah, Liu Jiangsi, Cui Ying, Qie Wenkun, Romaniello Stephen, Anbar Ariel Extensive marine anoxia associated with the Late Devonian Hangenberg Crisis. EARTH AND PLANETARY SCIENCE LETTERS. 2020;533():.

5666. Wilksch Simon, Paxton Susan, Byrne Susan, Austin S., O'Shea Anne, Wade Tracey Outcomes of three universal eating disorder risk reduction programs by participants with

higher and lower baseline shape and weight concern. INTERNATIONAL JOURNAL OF EATING DISORDERS. 2017;50(1):66-75.

5667. Moyad Mark Preventing Lethal Prostate Cancer with Diet, Supplements, and Rx: Heart Healthy Continues to Be Prostate Healthy and ``First Do No Harm{''} Part I. CURRENT UROLOGY REPORTS. 2018;19(12):.

5668. Gorter RC, Eijkman MAJ, Hoogstraten J Burnout and health among Dutch dentists. EUROPEAN JOURNAL OF ORAL SCIENCES. 2000;108(4):261-267.

5669. Hsu Ti, Forestell Catherine Mindfulness, depression, and emotional eating: The moderating role of nonjudging of inner experience. APPETITE. 2021;160():.

5670. Stein Kate, Warne Naomi, Heron Jon, Zucker Nancy, Bould Helen Do children with recurrent abdominal pain grow up to become adolescents who control their weight by fasting? Results from a UK population-based cohort. INTERNATIONAL JOURNAL OF EATING DISORDERS. 2021;54(6, SI):915-924.

5671. Shqair Ayah, Pauli Lais, Pereira Costa Vanessa, Cenci Maximiliano, Goettems Marilia Screen time, dietary patterns and intake of potentially cariogenic food in children: A systematic review. JOURNAL OF DENTISTRY. 2019;86():17-26.

5672. Zeng Shengqiang, Wang Hong, Chen Zaihua, Cao Qianqiang, Hu Lin, Wu Yanqing Effects of geranylgeranylacetone upon cardiovascular diseases. CARDIOVASCULAR THERAPEUTICS. 2018;36(4):.

5673. Johnson Jeremy, Byrne Gerard, Pelecanos Anita The prevalence of subthreshold psychiatric symptoms and associations with alcohol and substance use disorders: from a nationally representative survey of 36,309 adults. BMC PSYCHIATRY. 2022;22(1):.

5674. Richirt Julien, Riedel Bettina, Mouret Aurelia, Schweizer Magali, Langlet Dewi, Seitaj Dorina, Meysman Filip, Slomp Caroline, Jorissen Frans Foraminiferal community response to seasonal anoxia in Lake Grevelingen (the Netherlands). BIOGEOSCIENCES. 2020;17(6):1415-1435.

5675. Manwaring Jamie, Bryson Susan, Goldschmidt Andrea, Winzelberg Andrew, Luce Kristine, Cunning Darby, Wilfley Denise, Taylor C. Do adherence variables predict outcome

in an online program for the prevention of eating disorders?. JOURNAL OF CONSULTING AND CLINICAL PSYCHOLOGY. 2008;76(2):341-346.

5676. Mattes Richard Energy intake and obesity: Ingestive frequency outweighs portion size. PHYSIOLOGY \& BEHAVIOR. 2014;134(SI):110-118.

5677. Kotiniemi LH, Ryhanen PT, Moilanen IK Behavioural changes following routine ENT operations in two-to-ten-year-old children. PAEDIATRIC ANAESTHESIA. 1996;6(1):45-49.

5678. Wright Paul, McKinley Christopher Services and Information for Sexually Compulsive Students on College Counseling Center Websites: Results from a National Sample. JOURNAL OF HEALTH COMMUNICATION. 2010;15(6):665-678.

5679. Clowez Sophie, Godaux Damien, Cardol Pierre, Wollman Francis-Andre, Rappaport Fabrice The Involvement of Hydrogen-producing and ATP-dependent NADPH-consuming Pathways in Setting the Redox Poise in the Chloroplast of Chlamydomonas reinhardtii in Anoxia. JOURNAL OF BIOLOGICAL CHEMISTRY. 2015;290(13):8666-8676.

5680. Kessler Ute, Rekkedal Guro, Ro Oyvind, Berentsen Birgitte, Steinsvik Elisabeth, Lied Gulen, Danielsen Yngvild Association between gastrointestinal complaints and psychopathology in patients with anorexia nervosa. INTERNATIONAL JOURNAL OF EATING DISORDERS. 2020;53(5):532-536.

5681. Cradock Kevin, Quinlan Leo, Finucane Francis, Gainforth Heather, Ginis Kathleen, Sanders Elizabeth, OLaighin Gearoid Design of a Planner-Based Intervention to Facilitate Diet Behaviour Change in Type 2 Diabetes. SENSORS. 2022;22(7):.

5682. Kirk Katherine, Martin Felicity, Mao Amy, Parker Richard, Maguire Sarah, Thornton Laura, Zhu Gu, McAloney Kerrie, Freeman Jeremy, Hay Phillipa, Madden Sloane, Morgan Christine, Russell Janice, Sawyer Susan, Hughes Elizabeth, Fairweather-Schmidt A., Fursland Anthea, McCormack Julie, Wagg Fiona, Jordan Jennifer, Kennedy Martin, Ward Warren, Wade Tracey, Bulik Cynthia, Martin Nicholas The Anorexia Nervosa Genetics Initiative: Study description and sample characteristics of the Australian and New Zealand arm. AUSTRALIAN AND NEW ZEALAND JOURNAL OF PSYCHIATRY. 2017;51(6, SI):583-594.

5683. Mohamadi Azam, Shiraseb Farideh, Mirzababaei Atieh, Hosseininasab Dorsa, Rasaei Niloufar, Clark Cain, Mirzaei Khadijeh Circulating Inflammatory Markers May Mediate the Relationship between Healthy Plant-Based Diet and Metabolic Phenotype Obesity in

Women: A Cross-Sectional Study. INTERNATIONAL JOURNAL OF CLINICAL PRACTICE. 2022;2022():.

5684. BROOKS SPJ, STOREY KB EFFECT OF ANOXIA ON ISOLATED TURTLE TISSUES - IS THE RESPONSE TO ANOXIA MEDIATED BY PROTEIN-KINASE 2ND MESSENGERS. BIOCHEMISTRY AND MOLECULAR BIOLOGY INTERNATIONAL. 1994;34(6):1253-1258.

5685. Telgkamp P, Ramirez JM Differential responses of respiratory nuclei to anoxia in rhythmic brain stem slices of mice. JOURNAL OF NEUROPHYSIOLOGY. 1999;82(5):2163-2170.

5686. Davison KK, Markey CN, Birch LL A longitudinal examination of patterns in girls' weight concerns and body dissatisfaction from ages 5 to 9 years. INTERNATIONAL JOURNAL OF EATING DISORDERS. 2003;33(3):320-332.

5687. NILSSON GE, ROSEN P, JOHANSSON D ANOXIC DEPRESSION OF SPONTANEOUS LOCOMOTOR-ACTIVITY IN CRUCIAN CARP QUANTIFIED BY A COMPUTERIZED IMAGING TECHNIQUE. JOURNAL OF EXPERIMENTAL BIOLOGY. 1993;180():153-162.

5688. Kurochkin I., Ivanina A., Eilers S., Downs C., May L., Sokolova I. Cadmium affects metabolic responses to prolonged anoxia and reoxygenation in eastern oysters (Crassostrea virginica). AMERICAN JOURNAL OF PHYSIOLOGY-REGULATORY INTEGRATIVE AND COMPARATIVE PHYSIOLOGY. 2009;297(5):R1262-R1272.

5689. Jayachandran Muthukumaran, Chung Stephen, Xu Baojun A critical review on diet-induced microbiota changes and cardiovascular diseases. CRITICAL REVIEWS IN FOOD SCIENCE AND NUTRITION. 2020;60(17):2914-2925.

5690. Zhang Huafang, Guo F., Tang M., Dai H., Sheng J., Chen L., Liu S., Wang J., Shi Y., Ye C., Hou G., Wu X., Jin X., Chen Kun Association between Skeletal Muscle Strength and Dysphagia among Chinese Community-Dwelling Elderly Adults. JOURNAL OF NUTRITION HEALTH \& AGING. 2020;24(6):642-649.

5691. Jones Julie, Pena Roberto, Korczak Renee, Braun Hans Role of Carbohydrates and Grains in Nutrition and Neurological Disorders: Headache, Attention Deficit Hyperactivity Disorder, and Depression. CEREAL FOODS WORLD. 2017;62(4):162-171.

5692. Yang Qin, Wang Kun, Tian Qianqian, Zhang Jian, Qi Linyu, Chen Tao Effect of Diet and Exercise-Induced Weight Loss among Metabolically Healthy and Metabolically Unhealthy Obese Children and Adolescents. INTERNATIONAL JOURNAL OF ENVIRONMENTAL RESEARCH AND PUBLIC HEALTH. 2022;19(10):.

5693. Babington Lynn, Patel Bavika Understanding child feeding practices of Vietnamese mothers. MCN-THE AMERICAN JOURNAL OF MATERNAL-CHILD NURSING. 2008;33(6):377+.

5694. Maury P, Sarre A, Terrand J, Rosa A, Kucera P, Kappenberger L, Raddatz E Ventricular but not atrial electro-mechanical delay of the embryonic heart is altered by anoxia-reoxygenation and improved by nitric oxide. MOLECULAR AND CELLULAR BIOCHEMISTRY. 2004;265(1-2):141-149.

5695. Liu Tao, Yang Xiulu, Wu Yanli, Chen Min, Yang Yu, Chen Yun, Wang Yiying, Zhou Jie, Xu Kelin, Wang Na, Fu Chaowei Unhealthy Dietary Patterns Increased Risks of Incident Obesity: A Prospective Cohort Study in Southwest China. DIABETES METABOLIC SYNDROME AND OBESITY-TARGETS AND THERAPY. 2022;15():3111-3120.

5696. Mattioli Anna, Sciomer Susanna, Maffei Silvia, Gallina Sabina Lifestyle and Stress Management in Women During COVID-19 Pandemic: Impact on Cardiovascular Risk Burden. AMERICAN JOURNAL OF LIFESTYLE MEDICINE. 2021;15(3):356-359.

5697. Runge Katharina, Zon Sander, Bultmann Ute, Henkens Kene Metabolic syndrome incidence in an aging workforce: Occupational differences and the role of health behaviors. SSM-POPULATION HEALTH. 2021;15():.

5698. Batenburg Sietske, De Vleeschouwer David, Sprovieri Mario, Hilgen Frederik, Gale Andrew, Singer Brad, Koeberl Christian, Coccioni Rodolfo, Claeys Philippe, Montanari Alessandro Orbital control on the timing of oceanic anoxia in the Late Cretaceous. CLIMATE OF THE PAST. 2016;12(10):1995-2009.

5699. Smith Robert, Olin Bernie Wellness: Pharmacy Education's Role and Responsibility. AMERICAN JOURNAL OF PHARMACEUTICAL EDUCATION. 2010;74(4):.

5700. Covi JA, Treleaven WD, Hand SC V-ATPase inhibition prevents recovery from anoxia in Artemia franciscana embryos: quiescence signaling through dissipation of proton

gradients. JOURNAL OF EXPERIMENTAL BIOLOGY. 2005;208(14):2799-2808.

5701. Fernandes Bezerra Diego, Alves Sampaio Lucas, Raposo Landim Liejy Diagnosis of vigorexia and muscular dysmorphia in university of the health area. NUTRICION CLINICA Y DIETETICA HOSPITALARIA. 2019;38(4):179-182.

5702. Toennies Thaddaus, Heidemann Christin, Paprott Rebecca, Seidel-Jacobs Esther, Scheidt-Nave Christa, Brinks Ralph, Hoyer Annika Estimating the impact of tax policy interventions on the projected number and prevalence of adults with type 2 diabetes in Germany between 2020 and 2040. BMJ OPEN DIABETES RESEARCH \& CARE. 2021;9(1):.

5703. Parodi M, Rebaudo R, Perasso L, Gandolfo C, Cupello A, Balestrino M Effects of exogenous creatine on population spike amplitude and on postanoxic hyperexcitability in brain slices. BRAIN RESEARCH. 2003;963(1-2):197-202.

5704. FRIEDMAN JE, HADDAD GG ANOXIA INDUCES AN INCREASE IN INTRACELLULAR SODIUM IN RAT CENTRAL NEURONS IN-VITRO. BRAIN RESEARCH. 1994;663(2):329-334.

5705. Saade Sylvia, Hallit Souheil, Haddad Chadia, Hallit Rabih, Akel Marwan, Honein Karl, Akiki Maria, Kheir Nelly, Obeid Sahar Factors associated with restrained eating and validation of the Arabic version of the restrained eating scale among an adult representative sample of the Lebanese population: a cross-sectional study. JOURNAL OF EATING DISORDERS. 2019;7():.

5706. Dalton William, Klesges Lisa, Sherrill-Mittleman Deborah, Stockton Michelle, Allen Suzanne, Klesges Robert Family Context as It Relates to Weight-Related Behaviors in Preadolescent African American Girls. AMERICAN JOURNAL OF HEALTH BEHAVIOR. 2011;35(3):269-279.

5707. Keyhani E, Keyhani J Hypoxia/anoxia as signaling for increased alcohol dehydrogenase activity in saffron (Crocus sativus L.) corm. . 2004;1030():449-457.

5708. Li Qian, Cheng Chenxia, Zhang Chunjian, Xue Junxiu, Zhang Yong, Wang Caihong, Dang Ruihong, Yang Shaolan Pb4CL2 Inducing Lignin Accumulation in Superficial Scald `Chili' (Pyrus bretschneideri) Pear Fruit. AGRONOMY-BASEL. 2022;12(11):.

5709. Godet Ambre, Fortier Alexandra, Bannier Elise, Coquery Nicolas, Val-Laillet David Interactions between emotions and eating behaviors: Main issues, neuroimaging contributions, and innovative preventive or corrective strategies. REVIEWS IN ENDOCRINE \& METABOLIC DISORDERS. 2022;23(4, SI):807-831.

5710. KWON HY, WEIN AJ, LEVIN RM EFFECT OF ANOXIA ON THE URETHRAL RESPONSE TO PHENYLEPHRINE. JOURNAL OF UROLOGY. 1995;154(4):1527-1531.

5711. Anastasiadou Dimitra, Lupianez-Villanueva Francisco, Fauli Clara, Cunillera Jordina, Serrano-Troncoso Eduardo Cost-effectiveness of the mobile application TCApp combined with face-to-face CBT treatment compared to face-to-face CBT treatment alone for patients with an eating disorder: study protocol of a multi-centre randomised controlled trial. BMC PSYCHIATRY. 2018;18():.

5712. May Jon, Andrade Jackie, Batey Helen, Berry Lisa-Marie, Kavanagh David Less food for thought. Impact of attentional instructions on intrusive thoughts about snack foods. APPETITE. 2010;55(2):279-287.

5713. Murray Helen, Riddle Megan, Rao Fatima, McCann Barbara, Staller Kyle, Heitkemper Margaret, Zia Jasmine Eating disorder symptoms, including avoidant/restrictive food intake disorder, in patients with disorders of gut-brain interaction. NEUROGASTROENTEROLOGY AND MOTILITY. 2022;34(8):.

5714. Snortheim Craig, Hanson Paul, McMahon Katherine, Read Jordan, Carey Cayelan, Dugan Hilary Meteorological drivers of hypolimnetic anoxia in a eutrophic, north temperate lake. ECOLOGICAL MODELLING. 2017;343():39-53.

5715. Graell Montserrat, Andres Patricia, Rosa Sepulveda Ana, Moreno Alba, Villasenor Angel, Faya Mar, Martinez-Cantarero Carmen, Gomez-Martinez Sonia, Marcos Ascension, Morande Gonzalo, Nova Esther The adolescent onset anorexia nervosa study (ANABEL) Design and baseline results. INTERNATIONAL JOURNAL OF METHODS IN PSYCHIATRIC RESEARCH. 2018;27(3):.

5716. Okada Rieko, Tsushita Kazuyo, Wakai Kenji, Kato Kiminori, Wada Takashi, Shinohara Yukito Healthy lifestyle reduces incidence of trace/positive proteinuria and rapid kidney function decline after 2 years: from the Japan Ningen Dock study. NEPHROLOGY DIALYSIS TRANSPLANTATION. 2021;36(6):1039-1048.

5717. Molot L., Watson S., Creed I., Trick C., McCabe S., Verschoor M., Sorichetti R., Powe C., Venkiteswaran J., Schiff S. A novel model for cyanobacteria bloom formation: the critical role of anoxia and ferrous iron. FRESHWATER BIOLOGY. 2014;59(6):1323-1340.

5718. Castaneda Gail, Colby Sarah, Olfert Melissa, Barnett Tracey, Zhou Wenjun, Leite Walter, Staub Daniel, Mathews Anne Examining gender and the longitudinal effect of weight conscious drinking dimensions on body mass index among a college freshman cohort. JOURNAL OF AMERICAN COLLEGE HEALTH. 2021;():.

5719. Wise Nancy Pregnant Adolescents, Beliefs About Healthy Eating, Factors that Influence Food Choices, and Nutrition Education Preferences. JOURNAL OF MIDWIFERY \& WOMENS HEALTH. 2015;60(4):410-418.

5720. Wang Hong-Tian, Zhang Jing, Ji Ling-Chao, You Shao-Hua, Bai Yin, Dai Wei, Wang Zhong-Yuan Frequency of tuberculosis among diabetic patients in the People's Republic of China. THERAPEUTICS AND CLINICAL RISK MANAGEMENT. 2014;10():45-49.

5721. Kurotori Isaku, Shioda Katsutoshi, Abe Takaaki, Kato Rika, Ishikawa Shizukiyo, Suda Shiro An Inpatient Observational Study: Characteristics And Outcomes Of Avoidant/Restrictive Food Intake Disorder (ARFID) In Children And Adolescents In Japan. NEUROPSYCHIATRIC DISEASE AND TREATMENT. 2019;15():3313-3321.

5722. Ma Jian, Qiao Yijuan, Zhao Pei, Li Wei, Katzmarzyk Peter, Chaput Jean-Philippe, Fogelholm Mikael, Kuriyan Rebecca, Lambert Estelle, Maher Carol, Maia Jose, Matsudo Victor, Olds Timothy, Onywera Vincent, Sarmiento Olga, Standage Martyn, Tremblay Mark, Tudor-Locke Catrine, Hu Gang, Res Grp Breastfeeding and childhood obesity: A 12-country study. MATERNAL AND CHILD NUTRITION. 2020;16(3):.

5723. Schneider Sven, Huy Christina, Schuessler Marc, Diehl Katharina, Schwarz Stefanie Optimising lifestyle interventions: identification of health behaviour patterns by cluster analysis in a German 50 survey. EUROPEAN JOURNAL OF PUBLIC HEALTH. 2009;19(3):271-277.

5724. Karekla Maria, Georgiou Natasa, Panayiotou Georgia, Sandoz Emily, Kurz A., Constantinou Marios Cognitive Restructuring vs. Defusion: Impact on craving, healthy and unhealthy food intake. EATING BEHAVIORS. 2020;37():.

5725. Elias Marina, Gomes Daniela, Paracampo Carla Associations between Orthorexia Nervosa, Body Self-Image, Nutritional Beliefs, and Behavioral Rigidity. NUTRIENTS. 2022;14(21):.

5726. Badr Hoda, Paxton Raheem, Ater Joann, Urbauer Diana, Demark-Wahnefried Wendy Health Behaviors and Weight Status of Childhood Cancer Survivors and Their Parents: Similarities and Opportunities for Joint Interventions. JOURNAL OF THE AMERICAN DIETETIC ASSOCIATION. 2011;111(12):1917-1923.

5727. Montoya-Pino Carolina, Weyer Stefan, Anbar Ariel, Pross Joerg, Oschmann Wolfgang, Schootbrugge Bas, Arz Helge Global enhancement of ocean anoxia during Oceanic Anoxic Event 2: A quantitative approach using U isotopes. GEOLOGY. 2010;38(4):315-318.

5728. Yamada Atsurou, Katsuki Fujika, Kondo Masaki, Sawada Hanayo, Watanabe Norio, Akechi Tatsuo Association between the social support for mothers of patients with eating disorders, maternal mental health, and patient symptomatic severity: A cross-sectional study. JOURNAL OF EATING DISORDERS. 2021;9(1):.

5729. Stockey Richard, Cole Devon, Planavsky Noah, Loydell David, Fryda Jiri, Sperling Erik Persistent global marine euxinia in the early Silurian. NATURE COMMUNICATIONS. 2020;11(1):.

5730. Al-attar Rasha, Storey Kenneth RAGE against the stress: Mitochondrial suppression in hypometabolic hearts. GENE. 2020;761():.

5731. Khan Huda, Lee Richard, Khan Zaheer The interaction of social influence and message framing on children's food choice. EUROPEAN JOURNAL OF MARKETING. 2022;56(11, SI):2959-2977.

5732. XU ZC, PULSINELLI WA RESPONSES OF CA1 PYRAMIDAL NEURONS IS RAT HIPPOCAMPUS TO TRANSIENT FOREBRAIN ISCHEMIA - AN IN-VIVO INTRACELLULAR-RECORDING STUDY. NEUROSCIENCE LETTERS. 1994;171(1-2):187-191.

5733. Forney K., Buchman-Schmitt Jennifer, Keel Pamela, Frank Guido The medical complications associated with purging. INTERNATIONAL JOURNAL OF EATING DISORDERS. 2016;49(3):249-259.

5734. Sarre Alexandre, Maury Philippe, Kucera Pavel, Kappenberger Lukas, Raddatz Eric Arrhythmogenesis in the developing heart during anoxia-reoxygenation and hypothermia-rewarming: An in vitro model. JOURNAL OF CARDIOVASCULAR ELECTROPHYSIOLOGY. 2006;17(12):1350-1359.

5735. Cronberg T, Rytter A, Wieloch T Chelation of intracellular calcium reduces cell death after hyperglycemic in vitro ischemia in murine hippocampal slice cultures. BRAIN RESEARCH. 2005;1049(1):120-127.

5736. Gandhi Amarendra, Luyckx Koen, Baetens Imke, Kiekens Glenn, Sleuwaegen Ellen, Berens Ann, Maitra Shubhada, Claes Laurence Age of onset of non-suicidal self-injury in Dutch-speaking adolescents and emerging adults: An event history analysis of pooled data. COMPREHENSIVE PSYCHIATRY. 2018;80():170-178.

5737. Lindstedt Katarina, Kjellin Lars, Gustafsson Sanna Adolescents with full or subthreshold anorexia nervosa in a naturalistic sample - characteristics and treatment outcome. JOURNAL OF EATING DISORDERS. 2017;5():.

5738. Furlane Alessandro, Corona Alberto, Boyle Sara, Sharma Radhashree, Rubino Rachel, Habel Jill, Gablenze Eva, Giovanniello Jacqueline, Beyaz Semir, Janowitze Tobias, Shea Stephen, Li Bo Neurotensin neurons in the extended amygdala control dietary choice and energy homeostasis. NATURE NEUROSCIENCE. 2022;25(11):1470+.

5739. Viricel J, Bossu C, Galusca B, Kadem M, Germain N, Nicolau A, Millot L, Vergely N, Lassandre S, Carrot G, Lang E, Estour B Retrospective study of anorexia nervosa: reduced mortality and stable recovery rates. PRESSE MEDICALE. 2005;34(20, 1):1505-1510.

5740. Rodgers Rachel The role of the ``Healthy Weight{''} discourse in body image and eating concerns: An extension of sociocultural theory. EATING BEHAVIORS. 2016;22():194-198.

5741. Alonso-Magdalena Paloma, Quesada Ivan, Nadal Angel Prenatal Exposure to BPA and Offspring Outcomes: The Diabesogenic Behavior of BPA. DOSE-RESPONSE. 2015;13(2):.

5742. Konstantellou Anna, Sternheim Lot, Hale Lucy, Simic Mima, Eisler Ivan The experience of intolerance of uncertainty for parents of young people with a restrictive eating disorder. EATING AND WEIGHT DISORDERS-STUDIES ON ANOREXIA BULIMIA AND

OBESITY. 2022;27(4):1339-1348.

5743. Giontella Alice, Bonafini Sara, Tagetti Angela, Bresadola Irene, Minuz Pietro, Gaudino Rossella, Cavarzere Paolo, Ramaroli Diego, Marcon Denise, Branz Lorella, Principe Lara, Antoniazzi Franco, Maffeis Claudio, Fava Cristiano Relation between Dietary Habits, Physical Activity, and Anthropometric and Vascular Parameters in Children Attending the Primary School in the Verona South District. NUTRIENTS. 2019;11(5):.

5744. Cleator J., Abbott J., Judd P., Sutton C., Wilding J. Night eating syndrome: implications for severe obesity. NUTRITION \& DIABETES. 2012;2():.

5745. Pisetsky Emily, Crow Scott, Peterson Carol An empirical test of the interpersonal theory of suicide in a heterogeneous eating disorder sample. INTERNATIONAL JOURNAL OF EATING DISORDERS. 2017;50(2):162-165.

5746. Field AE, Camargo CA, Taylor CB, Berkey CS, Colditz GA Relation of peer and media influences to the development of purging behaviors among preadolescent and adolescent girls. ARCHIVES OF PEDIATRICS \& ADOLESCENT MEDICINE. 1999;153(11):1184-1189.

5747. Hirtz Raphael, Zheng Yiran, Rajcsanyi Luisa, Libuda Lars, Antel Jochen, Peters Triinu, Hebebrand Johannes, Hinney Anke Genetic Analyses of Complex Phenotypes Through the Example of Anorexia Nervosa and Bodyweight Regulation. ZEITSCHRIFT FUR KINDER-UND JUGENDPSYCHIATRIE UND PSYCHOTHERAPIE. 2022;50(3):175-185.

5748. Dingle Sara, Bowe Steven, Bujtor Melissa, Milte Catherine, Daly Robin, Anstey Kaarin, Shaw Jonathan, Torres Susan Associations between data-driven lifestyle profiles and cognitive function in the AusDiab study. BMC PUBLIC HEALTH. 2022;22(1):.

5749. Augustine Ann Nonrapid Eye Movement Parasomnias. JOURNAL OF CLINICAL NEUROPHYSIOLOGY. 2023;40(3):224-229.

5750. Reel Justine, Galli Nick, Miyairi Maya, Voelker Dana, Greenleaf Christy Development and validation of the intuitive exercise scale. EATING BEHAVIORS. 2016;22():129-132.

5751. Collin Lisa, Bindra Jasmeet, Raju Monika, Gillberg Christopher, Minnis Helen Facial emotion recognition in child psychiatry: A systematic review. RESEARCH IN

DEVELOPMENTAL DISABILITIES. 2013;34(5):1505-1520.

5752. Bondolfi Constantin, Taffe Patrick, Augsburger Aurelie, Jaques Cecile, Malebranche Mary, Clair Carole, Bodenmann Patrick Impact of incarceration on cardiovascular disease risk factors: a systematic review and meta-regression on weight and BMI change. BMJ OPEN. 2020;10(10):.

5753. Mutwalli Hiba, Keeler Johanna, Bektas Sevgi, Dhopatkar Namrata, Treasure Janet, Himmerich Hubertus Eating cognitions, emotions and behaviour under treatment with second generation antipsychotics: A systematic review and meta-analysis. JOURNAL OF PSYCHIATRIC RESEARCH. 2023;160():137-162.

5754. Schenck CH, Boyd JL, Mahowald MW A parasomnia overlap disorder involving sleepwalking, sleep terrors, and REM sleep behavior disorder in 33 polysomnographically confirmed cases. SLEEP. 1997;20(11):972-981.

5755. Caraballo Juan, Borcherding Jennifer, Rector Michael, Hornick Emma, Stoltz David, Zabner Joseph, Comellas Alejandro Role of PON in Anoxia-Reoxygenation Injury: A Drosophila Melanogaster Transgenic Model. PLOS ONE. 2014;9(1):.

5756. Robinson Justin, Szydlowski Steven Obesity Prevention Strategies for Teenage Adults in Central and Eastern Europe. CLINICAL SOCIAL WORK AND HEALTH INTERVENTION. 2019;10(1):39-43.

5757. Chen J, Mandel LJ Role of water and electrolyte influxes in anoxic plasma membrane disruption. AMERICAN JOURNAL OF PHYSIOLOGY-CELL PHYSIOLOGY. 1997;273(4):C1341-C1348.

5758. Pradeepa Rajendra, Mohan Viswanathan Epidemiology of type 2 diabetes in India. INDIAN JOURNAL OF OPHTHALMOLOGY. 2021;69(11):2932-2938.

5759. Lopez-Vicchi Felicitas, De Winne Catalina, Ornstein Ana, Sorianello Eleonora, Toneatto Judith, Becu-Villalobos Damasia Severe Hyperprolactinemia Promotes Brown Adipose Tissue Whitening and Aggravates High Fat Diet Induced Metabolic Imbalance. FRONTIERS IN ENDOCRINOLOGY. 2022;13():.

5760. Schauer Antje, Barthel Peggy, Adams Volker, Linke Axel, Poitz David, Weinbrenner Christof Pharmacological Pre- and Postconditioning With Levosimendan Protect H9c2 Cardiomyoblasts From Anoxia/Reoxygenation-induced Cell Death via PI3K/Akt Signaling. JOURNAL OF CARDIOVASCULAR PHARMACOLOGY. 2021;77(3):378-385.

5761. Lares-Michel Mariana, Housni Fatima, Aguilera Cervantes Virginia, Carrillo Presentacion, Michel Nava Rosa, Llanes Canedo Claudia Eat Well to Fight Obesity horizontal ellipsis and Save Water: The Water Footprint of Different Diets and Caloric Intake and Its Relationship With Adiposity. FRONTIERS IN NUTRITION. 2021;8():.

5762. {[}Anonymous] The role of urban food policy in preventing diet-related non-communicable diseases in Cape Town and New York. PUBLIC HEALTH. 2015;129(4):327-335.

5763. Lim Sun, You Sukkyung Effects of Self-Esteem and Depression on Abnormal Eating Behavior among Korean Female College Students: Mediating Role of Body Dissatisfaction. JOURNAL OF CHILD AND FAMILY STUDIES. 2017;26(1):176-182.

5764. Meiltz A, Kucera P, Ribaupierre Y, Raddatz E Inhibition of bicarbonate transport protects embryonic heart against reoxygenation-induced dysfunction. JOURNAL OF MOLECULAR AND CELLULAR CARDIOLOGY. 1998;30(2):327-335.

5765. Campbell Rachel, Vansteenkiste Maarten, Soenens Bart, Vandenkerckhove Beatrijs, Mouratidis Athanasios Toward a Better Understanding of the Reciprocal Relations Between Adolescent Psychological Need Experiences and Sleep. PERSONALITY AND SOCIAL PSYCHOLOGY BULLETIN. 2021;47(3):377-394.

5766. Cockerham William, Bauldry Shawn, Sims Mario Obesity-Related Health Lifestyles of Late-Middle Age Black Americans: The Jackson Heart Study. AMERICAN JOURNAL OF PREVENTIVE MEDICINE. 2022;63(1, 1):S47-S55.

5767. Jepson Ruth, Harris Fiona, Platt Stephen, Tannahill Carol The effectiveness of interventions to change six health behaviours: a review of reviews. BMC PUBLIC HEALTH. 2010;10():.

5768. Milton SL, Lutz PL Adenosine and ATP-sensitive potassium channels modulate dopamine release in the anoxic turtle (Trachemys scripta) striatum. AMERICAN JOURNAL OF PHYSIOLOGY-REGULATORY INTEGRATIVE AND COMPARATIVE PHYSIOLOGY.

2005;289(1):R77-R83.

5769. Mohiyeddini Changiz Repressive coping among British college women: A potential protective factor against body image concerns, drive for thinness, and bulimia symptoms. BODY IMAGE. 2017;22():39-47.

5770. Dello Russo Marika, Russo Paola, Angel Rufian-Henares Jose, Hinojosa-Nogueira Daniel, Perez-Burillo Sergio, Cueva Silvia, Rohn Sascha, Fatouros Alexandra, Douros Konstantinos, Gonzalez-Vigil Veronica, Epstein David, Francino M., Siani Alfonso, Lauria Fabio The Stance4Health Project: Evaluating a Smart Personalised Nutrition Service for Gut Microbiota Modulation in Normal-and Overweight Adults and Children with Obesity, Gluten-Related Disorders or Allergy/Intolerance to Cow's Milk. FOODS. 2022;11(10):.

5771. Unikel Santoncini Claudia, Leon Vazquez Concepcion, Rivera Marquez Jose Disordered eating behaviors and psychological correlates among overweight and obese freshmen College students. SALUD MENTAL. 2016;39(3):141-148.

5772. Espinoza Paola, Penelo Eva, Raich Rosa Prevention programme for eating disturbances in adolescents. Is their effect on body image maintained at 30 months later?. BODY IMAGE. 2013;10(2):175-181.

5773. Hoffmann Mauricio, McDaid David, Salum Giovanni, Silva-Ribeiro Wagner, Ziebold Carolina, King Derek, Gadelha Ary, Miguel Euripedes, Mari Jair, Rohde Luis, Pan Pedro, Bressan Rodrigo, Mojtabai Ramin, Evans-Lacko Sara The impact of child psychiatric conditions on future educational outcomes among a community cohort in Brazil. EPIDEMIOLOGY AND PSYCHIATRIC SCIENCES. 2021;30():.

5774. Bilali Aggeliki, Galanis Petros, Velonakis Emmanuel, Katostaras Theofanis Factors Associated with Abnormal Eating Attitudes among Greek Adolescents. JOURNAL OF NUTRITION EDUCATION AND BEHAVIOR. 2010;42(5):292-298.

5775. Martini Maria, Barona-Martinez Manuela, Micali Nadia Eating disorders mothers and their children: a systematic review of the literature. ARCHIVES OF WOMENS MENTAL HEALTH. 2020;23(4):449-467.

5776. Mathew Thomas, Souza Delon, John Saji, Kumar Sharath Transient Ischemic Attack after Eating Spicy Foods in Children: Think of Moya Moya Disease. NEUROLOGY INDIA.

2021;69(4):1032-1033.

5777. Chen Yuling, Wu Fangqin, Wu Ying, Li Jia, Yue Peng, Deng Ying, Lamb Karen, Fong Simon, Liu Yisi, Zhang Yan Development of interventions for an intelligent and individualized mobile health care system to promote healthy diet and physical activity: using an intervention mapping framework. BMC PUBLIC HEALTH. 2019;19(1):.

5778. Reardon Claudia The Mental Health of Athletes: Recreational to Elite. CURRENT SPORTS MEDICINE REPORTS. 2021;20(12):631-637.

5779. Voelker Dana, Petrie Trent, Reel Justine, Gould Dan Frequency and Psychosocial Correlates of Eating Disorder Symptomatology in Male Figure Skaters. JOURNAL OF APPLIED SPORT PSYCHOLOGY. 2018;30(1):119-126.

5780. Rosewall Juliet, Gleaves David, Latner Janet An examination of risk factors that moderate the body dissatisfaction-eating pathology relationship among New Zealand adolescent girls. JOURNAL OF EATING DISORDERS. 2018;6():.

5781. Bosy-Westphal Anja, Mueller Manfred Diet and Nutrition in the Prevention of Non-Communicable Diseases (NCD). AKTUELLE ERNAHRUNGSMEDIZIN. 2021;46(04):246-254.

5782. Cardi Valentina, Leppanen Jenni, Mataix-Cols David, Campbell Iain, Treasure Janet A case series to investigate food-related fear learning and extinction using in vivo food exposure in anorexia nervosa: A clinical application of the inhibitory learning framework. EUROPEAN EATING DISORDERS REVIEW. 2019;27(2):173-181.

5783. Rasheed P Perception of body weight and self-reported eating and exercise behaviour among obese and non-obese women in Saudi Arabia. PUBLIC HEALTH. 1998;112(6):409-414.

5784. Li Bai, Adab Peymane, Cheng Kar The role of grandparents in childhood obesity in China - evidence from a mixed methods study. INTERNATIONAL JOURNAL OF BEHAVIORAL NUTRITION AND PHYSICAL ACTIVITY. 2015;12():.

5785. Chan Hei, Jaffe Jane, D'Souza Natasha, Lowe Joshua, Matthews-Rensch Kylie Goal energy intake for medically compromised patients with eating disorders: A systematic

review. NUTRITION \& DIETETICS. 2021;78(1):86-100.

5786. Mladinov Anton, Rebernjak Blaz, Mladinov Maja, Puljevic Mislav, Pasicek Ljerka, Puljevic Davor Behaviour after Cardiosurgery. COLLEGIUM ANTROPOLOGICUM. 2010;34(4):1373-1378.

5787. Hannich J., Haribowo A., Gentina Sebastien, Paillard Melanie, Gomez Ludovic, Pillot Bruno, Thibault Helene, Abegg Daniel, Guex Nicolas, Zumbuehl Andreas, Adibekian Alexander, Ovize Michel, Martinou Jean-Claude, Riezman Howard 1-Deoxydihydroceramide causes anoxic death by impairing chaperonin-mediated protein folding. NATURE METABOLISM. 2019;1(10):996+.

5788. Brown AM, Fern R, Jarvinen JPL, Kaila K, Ransom BR Changes in {[}Ca2+](0) during anoxia in CNS white matter. NEUROREPORT. 1998;9(9):1997-2000.

5789. Field AE, Camargo CA, Taylor CB, Berkey CS, Frazier AL, Gillman MW, Colditz GA Overweight, weight concerns, and bulimic behaviors among girls and boys. JOURNAL OF THE AMERICAN ACADEMY OF CHILD AND ADOLESCENT PSYCHIATRY. 1999;38(6):754-760.

5790. Canas Laura, Palma Carol, Molano Ana, Domene Lola, Carulla-Roig Marta, Cecilia-Costa Raquel, Dolz Montserrat, Serrano-Troncoso Eduardo Avoidant/restrictive food intake disorder: Psychopathological similarities and differences in comparison to anorexia nervosa and the general population. EUROPEAN EATING DISORDERS REVIEW. 2021;29(2):245-256.

5791. Fond G., Capdevielle D., Attal J., Boulenger J. Is fibromyalgia a psychiatric illness?. PSN-PSYCHIATRIE SCIENCES HUMAINES NEUROSCIENCES. 2011;9(4):190-197.

5792. Strahl Julia, Dringen Ralf, Schmidt Maike, Hardenberg Silvia, Abele Doris Metabolic and physiological responses in tissues of the long-lived bivalve Arctica islandica to oxygen deficiency. COMPARATIVE BIOCHEMISTRY AND PHYSIOLOGY A-MOLECULAR \& INTEGRATIVE PHYSIOLOGY. 2011;158(4):513-519.

5793. Porfirio Maria-Cristina, Giovinazzo Silvia, Cortese Samuele, Giana Grazia, Lo-Castro Adriana, Mouren Marie-Christine, Curatolo Paolo, Purper-Ouakil Diane Role of ADHD symptoms as a contributing factor to obesity in patients with MC4R. MEDICAL HYPOTHESES. 2015;84(1):4-7.

5794. POPE HG, MANGWETH B, NEGRAO AB, HUDSON JI, CORDAS TA CHILDHOOD SEXUAL ABUSE AND BULIMIA-NERVOSA - A COMPARISON OF AMERICAN, AUSTRIAN, AND BRAZILIAN WOMEN. AMERICAN JOURNAL OF PSYCHIATRY. 1994;151(5):732-737.

5795. Kumar Soumitra, Ray Saumitra, Roy Debabrata, Ganguly Kajal, Dutta Sibananda, Mahapatra Tanmay, Mahapatra Sanchita, Gupta Kinnori, Chakraborty Kaushik, Das Mrinal, Guha Santanu, Deb Pradip, Banerjee Amal Exercise and eating habits among urban adolescents: a cross-sectional study in Kolkata, India. BMC PUBLIC HEALTH. 2017;17():.

5796. Allega Olivia, Leng Xiamin, Vaccarino Anthony, Skelly Matthew, Lanzini Mariana, Hidalgo Maria, Soares Claudio, Kennedy Sidney, Frey Benicio Performance of the biological rhythms interview for assessment in neuropsychiatry: An item response theory and actigraphy analysis. JOURNAL OF AFFECTIVE DISORDERS. 2018;225():54-63.

5797. Kimura Hiroyuki, Tonoike Takashi, Muroya Tamio, Yoshida Keizo, Ozaki Norio Age of onset has limited association with body mass index at time of presentation for anorexia nervosa: Comparison of peak-onset and late-onset anorexia nervosa groups. PSYCHIATRY AND CLINICAL NEUROSCIENCES. 2007;61(6):646-650.

5798. Nassir Che, Ghazali Mazira, Hashim Sabarisah, Idris Nur, Yuen Lee, Hui Wong, Norman Haziq, Gau Chuang, Jayabalan Nanthini, Na Yuri, Feng Linqing, Ong Lin, Hamid Hafizah, Ahamed Haja, Mustapha Muzaimi Diets and Cellular-Derived Microparticles: Weighing a Plausible Link With Cerebral Small Vessel Disease. FRONTIERS IN CARDIOVASCULAR MEDICINE. 2021;8():.

5799. Ghamri Ranya, Alzahrani Nada, Alharthi Amal, Gadah Hana, Badoghaish Bayan, Alzahrani Azzah Cardiovascular risk factors among high-risk individuals attending the general practice at king Abdulaziz University hospital: a cross-sectional study. BMC CARDIOVASCULAR DISORDERS. 2019;19(1):.

5800. DeJong Hannah, Perkins Sarah, Grover Miriam, Schmidt Ulrike The Prevalence of Irritable Bowel Syndrome in Outpatients with Bulimia Nervosa. INTERNATIONAL JOURNAL OF EATING DISORDERS. 2011;44(7):661-664.

5801. HAGERMAN L, SZANIAWSKA A HEMOLYMPH NITROGEN-COMPOUNDS AND AMMONIA EFFLUX RATES UNDER ANOXIA IN THE BRACKISH-WATER ISOPOD SADURIA ENTOMON. MARINE ECOLOGY PROGRESS SERIES. 1994;103(3):285-289.

5802. Wadolowska Lidia, Hamulka Jadwiga, Kowalkowska Joanna, Kostecka Malgorzata, Wadolowska Katarzyna, Biezanowska-Kopec Renata, Czarniecka-Skubina Ewa, Kozirok Witold, Piotrowska Anna Prudent-Active and Fast-Food-Sedentary Dietary-Lifestyle Patterns: The Association with Adiposity, Nutrition Knowledge and Sociodemographic Factors in Polish TeenagersThe ABC of Healthy Eating Project. NUTRIENTS. 2018;10(12):.

5803. Li An, Qiu Bingjiang, Goettsch Marjolein, Chen Yuntao, Ge Shaohua, Xu Shulan, Tjakkes Geerten-Has Association between the quality of plant-based diets and periodontitis in the US general population. JOURNAL OF CLINICAL PERIODONTOLOGY. 2023;():.

5804. Zon Sander, Amick III Benjamin, Jong Trynke, Brouwer Sandra, Bultmann Ute Occupational distribution of metabolic syndrome prevalence and incidence differs by sex and is not explained by age and health behavior: results from 75 000 Dutch workers from 40 occupational groups. BMJ OPEN DIABETES RESEARCH \& CARE. 2020;8(1):.

5805. Reeve Erica, Thow Anne, Bell Colin, Engelhardt Katrin, Gamolo-Naliponguit Ella, Go John, Sacks Gary Implementation lessons for school food policies and marketing restrictions in the Philippines: a qualitative policy analysis. GLOBALIZATION AND HEALTH. 2018;14():.

5806. Cooper Karen, Quested Tom, Lanctuit Helene, Zimmermann Diane, Espinoza-Orias Namy, Roulin Anne Nutrition in the Bin: A Nutritional and Environmental Assessment of Food Wasted in the UK. FRONTIERS IN NUTRITION. 2018;5():.

5807. Pek-Scott M, Lutz PL ATP-sensitive K+ channel activation provides transient protection to the anoxic turtle brain. AMERICAN JOURNAL OF PHYSIOLOGY-REGULATORY INTEGRATIVE AND COMPARATIVE PHYSIOLOGY. 1998;275(6):R2023-R2027.

5808. Javier Sarah, Belgrave Faye ``I'm Not White, I Have to Be Pretty and Skinny{''}: A Qualitative Exploration of Body Image and Eating Disorders Among Asian American Women. ASIAN AMERICAN JOURNAL OF PSYCHOLOGY. 2019;10(2):141-153.

5809. Fiamingo Giuseppe, Esposto Roberta, Dal Fabbro Beatrice, Terzaghi Michele Kleine-Levin syndrome: report of a case with marked dysautonomic features. JOURNAL OF CLINICAL SLEEP MEDICINE. 2022;18(9):2313-2316.

5810. Mueller J., Sun Y., Yang F., Regelous M., Joachimski M. Manganous water column in the Tethys Ocean during the Permian-Triassic transition. GLOBAL AND PLANETARY CHANGE.

2023;222():.

5811. Bjornsdotter Malin, Davidovic Monika, Karjalainen Louise, Starck Goran, Olausson Hakan, Wentz Elisabet Grey matter correlates of autistic traits in women with anorexia nervosa. JOURNAL OF PSYCHIATRY \& NEUROSCIENCE. 2018;43(2):79-86.

5812. Moessner Markus, Bilic Sally, Bauer Stephanie The Importance of Social Exchange for Internet-based Eating Disorder Prevention. PRAXIS DER KINDERPSYCHOLOGIE UND KINDERPSYCHIATRIE. 2019;68(8):728-741.

5813. Rahimi-Ardabili Hania, Reynolds Rebecca, Vartanian Lenny, McLeod Leigh, Zwar Nicholas A Systematic Review of the Efficacy of Interventions that Aim to Increase Self-Compassion on Nutrition Habits, Eating Behaviours, Body Weight and Body Image. MINDFULNESS. 2018;9(2):388-400.

5814. Buchanan Kiera, Sheffield Jeanie, Tan Wee Predictors of diet failure: A multifactorial cognitive and behavioural model. JOURNAL OF HEALTH PSYCHOLOGY. 2019;24(7):857-869.

5815. Kiriike N, Nagata T, Matsunaga H, Tobitan W, Nishiura T Single and married patients with eating disorders. PSYCHIATRY AND CLINICAL NEUROSCIENCES. 1998;52(S):S306-S308.

5816. Santini Silvano, Porcu Cristiana, Tarantino Giovanni, Amicarelli Fernanda, Balsano Clara Oleuropein overrides liver damage in steatotic mice. JOURNAL OF FUNCTIONAL FOODS. 2020;65():.

5817. Khiali Sajad, Agabalazadeh Amin, Sahrai Hadi, Baghi Hossein, Banaeian Gholamreza, Entezari-Maleki Taher Effect of Caffeine Consumption on Cardiovascular Disease: An Updated Review. PHARMACEUTICAL MEDICINE. 2023;37(2):139-151.

5818. Warren MP, Ramos RH, Bronson EM Exercise-associated amenorrhea - Are altered leptin levels an early warning sign?. PHYSICIAN AND SPORTSMEDICINE. 2002;30(10):41-46.

5819. Du XJ, Woodcock EA, Little PJ, Esler MD, Dart AM Protection of neuronal uptake-1 inhibitors in ischemic and anoxic hearts by norepinephrine-dependent and -independent

mechanisms. JOURNAL OF CARDIOVASCULAR PHARMACOLOGY. 1998;32(4):621-628.

5820. Gamperl AK, Todgham AE, Parkhouse WS, Dill R, Farrell AP Recovery of trout myocardial function following anoxia: preconditioning in a non-mammalian model. AMERICAN JOURNAL OF PHYSIOLOGY-REGULATORY INTEGRATIVE AND COMPARATIVE PHYSIOLOGY. 2001;281(6):R1755-R1763.

5821. Marchand Erica, Stice Eric, Rohde Paul, Becker Carolyn Moving from efficacy to effectiveness trials in prevention research. BEHAVIOUR RESEARCH AND THERAPY. 2011;49(1):32-41.

5822. Petrie Trent, Greenleaf Christy, Reel Justine, Carter Jennifer An Examination of Psychosocial Correlates of Eating Disorders Among Female Collegiate Athletes. RESEARCH QUARTERLY FOR EXERCISE AND SPORT. 2009;80(3):621-632.

5823. Skelton Kara, Evans Retta A Qualitative Investigation of College Student Perceptions of Their Nutrition Environment: Recommendations for Improvement. AMERICAN JOURNAL OF HEALTH EDUCATION. 2020;51(1):50-58.

5824. Atkinson Melissa, Wade Tracey Enhancing dissemination in selective eating disorders prevention: An investigation of voluntary participation among female university students. BEHAVIOUR RESEARCH AND THERAPY. 2013;51(12):806-816.

5825. Swenne Ingemar, Parling Thomas, Ros Helena Family-based intervention in adolescent restrictive eating disorders: early treatment response and low weight suppression is associated with favourable one-year outcome. BMC PSYCHIATRY. 2017;17():.

5826. Berger Uwe Primary prevention of eating disorders. PSYCHOTHERAPEUT. 2006;51(3):187+.

5827. Nganabashaka Jean, Ntawuyirushintege Seleman, Niyibizi Jean, Umwali Ghislaine, Bavuma Charlotte, Byiringiro Jean, Rulisa Stephen, Burns Jacob, Rehfuess Eva, Young Taryn, Tumusiime David Population-Level Interventions Targeting Risk Factors for Hypertension and Diabetes in Rwanda: A Situational Analysis. FRONTIERS IN PUBLIC HEALTH. 2022;10():.

5828. Dodig-Curkovic Katarina, Curkovic Mario, Radic Josipa, Degmecic Dunja, Pozgain Ivan, Filakovic Pavo The Case Report of Treatment Strategy for Anorexia nervosa with Psychotic Elements in Adolescent. COLLEGIUM ANTROPOLOGICUM. 2010;34(3):1093-1099.

5829. Pesch Megan, Rizk Monika, Appugliese Danielle, Rosenblum Katherine, Miller Alison, Lumeng Julie Maternal concerns about children overeating among low-income children. EATING BEHAVIORS. 2016;21():220-227.

5830. Kiriike N, Nagata T, Matsunaga H, Tobitani W, Nishiura T Married patients with eating disorders in Japan. ACTA PSYCHIATRICA SCANDINAVICA. 1996;94(6):428-432.

5831. El-kholy Thanaa, Naglaa H., Hassan Rasha, Anter Sahar Nutrient Intakes Affecting the Nutritional Status of preschool Children by Nationality Compared with RDA in Jeddah KSA. LIFE SCIENCE JOURNAL-ACTA ZHENGZHOU UNIVERSITY OVERSEAS EDITION. 2012;9(4):3338-3346.

5832. Piepoli Massimo, Villani Giovanni Lifestyle modification in secondary prevention. EUROPEAN JOURNAL OF PREVENTIVE CARDIOLOGY. 2017;24(3):101-107.

5833. Abrahamian Heidemarie, Kautzky-Willer Alexandra, Riessland-Seifert Angelika, Fasching Peter, Ebenbichler Christoph, Hofmann Peter, Toplak Hermann Mental disorders and diabetes mellitus. WIENER KLINISCHE WOCHENSCHRIFT. 2016;128(2):S170-S178.

5834. Abrahamian Heidemarie, Kautzky-Willer Alexandra, Riessland-Seifert Angelika, Fasching Peter, Ebenbichler Christoph, Kautzky Alexander, Hofmann Peter, Toplak Hermann Mental disorders and diabetes mellitus (Update 2019). WIENER KLINISCHE WOCHENSCHRIFT. 2019;131(1):186-195.

5835. WATSON GB, LANTHORN TH PHENYTOIN DELAYS ISCHEMIC DEPOLARIZATION, BUT CANNOT BLOCK ITS LONG-TERM CONSEQUENCES, IN THE RAT HIPPOCAMPAL SLICE. NEUROPHARMACOLOGY. 1995;34(5):553-558.

5836. Fogel Naomi, Weissberg-Benchell Jill Preventing Poor Psychological and Health Outcomes in Pediatric Type 1 Diabetes. CURRENT DIABETES REPORTS. 2010;10(6):436-443.

5837. Zhang Cui, Lin Guosheng, Wan Weiguo, Li Xuyon, Zeng Bin, Yang Bo, Huang Congxin Resveratrol, a polyphenol phytoalexin, protects cardiomyocytes against anoxia/reoxygenation injury via the TLR4/NF-kappa B signaling pathway. INTERNATIONAL JOURNAL OF MOLECULAR MEDICINE. 2012;29(4):557-563.

5838. Yu JN, Cunningham JA, Thouin SR, Gurvich T, Liu D Hyperlipidemia. PRIMARY CARE. 2000;27(3):541+.

5839. Brunelle Joslyn, Shroff Emelyn, Perlman Harris, Strasser Andreas, Moraes Carlos, Flavell Richard, Danial Nika, Keith Brian, Thompson Craig, Chandel Navdeep Loss of Mcl-1 protein and inhibition of electron transport chain together induce anoxic cell death. MOLECULAR AND CELLULAR BIOLOGY. 2007;27(4):1222-1235.

5840. Lloyd-Williams Ffion, Mwatsama Modi, Ireland Robin, Capewell Simon Small changes in snacking behaviour: the potential impact on CVD mortality. PUBLIC HEALTH NUTRITION. 2009;12(6):871-876.

5841. Becker A Television, disordered eating, and young women in Fiji: Negotiating body image and identity during rapid social change. CULTURE MEDICINE AND PSYCHIATRY. 2004;28(4):533-559.

5842. Northam EA, Matthews LK, Anderson PJ, Cameron FJ, Werther GA Psychiatric morbidity and health outcome in Type 1 diabetes - perspectives from a prospective longitudinal study. DIABETIC MEDICINE. 2005;22(2):152-157.

5843. Rossi Francesca, Tortora Chiara, Paoletta Marco, Marrapodi Maria, Argenziano Maura, Di Paola Alessandra, Pota Elvira, Di Pinto Daniela, Di Martino Martina, Iolascon Giovanni Osteoporosis in Childhood Cancer Survivors: Physiopathology, Prevention, Therapy and Future Perspectives. CANCERS. 2022;14(18):.

5844. Erguney-Okumus F., Sertel-Berk H. The Psychometric Properties of the Eating Attitudes Test Short Form (EAT-26) in a College Sample. STUDIES IN PSYCHOLOGY-PSIKOLOJI CALISMALARI DERGISI. 2020;40(1):57-78.

5845. Bauer Anika, Schneider Silvia, Waldorf Manuel, Adolph Dirk, Vocks Silja Familial transmission of a body-related attentional bias - An eye-tracking study in a nonclinical sample of female adolescents and their mothers. PLOS ONE. 2017;12(11):.

5846. Rieger Elizabeth, Dolan Ashleigh, Thomas Brittany, Bell Jason The effect of interpersonal rejection on attentional biases regarding thin-ideal and non-thin images: The moderating role of body weight- and shape-based self-worth. BODY IMAGE. 2017;22():78-86.

5847. Carro Amelia, Maria Panisello Josefa Deciphering the Riddles in Nutrition and Cardiovascular Disease. EUROPEAN CARDIOLOGY REVIEW. 2019;14(3):141-150.

5848. Austin S., Richmond Tracy, Spadano-Gasbarro Jennifer, Greaney Mary, Blood Emily, Walls Courtney, Wang Monica, Mezgebu Solomon, Osganian Stavroula, Peterson Karen The Contribution of School Environmental Factors to Individual and School Variation in Disordered Weight Control Behaviors in a Statewide Sample of Middle Schools. EATING DISORDERS. 2013;21(2):91-108.

5849. Ferrer-Garcia Marta, Gutierrez-Maldonado Jose, Treasure Janet, Vilalta-Abella Ferran Craving for Food in Virtual Reality Scenarios in Non-Clinical Sample: Analysis of its Relationship with Body Mass Index and Eating Disorder Symptoms. EUROPEAN EATING DISORDERS REVIEW. 2015;23(5):371-378.

5850. De Young Kyle, Kambanis Paraskevi Practice makes perfect: Commentary on Burnette et al. (2021). INTERNATIONAL JOURNAL OF EATING DISORDERS. 2022;55(2):273-275.

5851. Wang Zhonghua, Yang Fan, Jiang Yanan, Wang Rui, Chen Xinrui, Lv Jinxin, Li Chunlei, Sun Xin, Li Jia, Wang Shu Role of pyroptosis in normal cardiac response to calorie restriction and starvation. BIOCHEMICAL AND BIOPHYSICAL RESEARCH COMMUNICATIONS. 2018;495(1):1122-1128.

5852. Lecendreux Michel, Silverstein Michael, Konofal Eric, Cortese Samuele, Faraone Stephen A 9-Year Follow-Up of Attention-Deficit/Hyperactivity Disorder in a Population Sample. JOURNAL OF CLINICAL PSYCHIATRY. 2019;80(3):.

5853. Goncalves Sonia, Machado Barbara, Martins Carla, Hoek Hans, Machado Paulo Retrospective Correlates for Bulimia Nervosa: A Matched Case-Control Study. EUROPEAN EATING DISORDERS REVIEW. 2016;24(3):197-205.

5854. Pettigrew Simone, Jongenelis Michelle, Quester Pascale, Chapman Kathy, Miller Caroline Factors associated with parents' attitudes to unhealthy foods and beverages.

JOURNAL OF PAEDIATRICS AND CHILD HEALTH. 2016;52(4):449-454.

5855. Pantano Kathleen Coaching Concerns in Physically Active Girls and Young Women-Part I: The Female Athlete Triad. STRENGTH AND CONDITIONING JOURNAL. 2009;31(6):38-43.

5856. Matusik Aneta, Grajek Mateusz, Szlacheta Patryk, Korzonek-Szlacheta Ilona Comparison of the Prevalence of Eating Disorders among Dietetics Students and Students of Other Fields of Study at Selected Universities (Silesia, Poland). NUTRIENTS. 2022;14(15):.

5857. Kim Mina, Yang Soo, Kim Hyang, Jo Anna, Jhon Min, Lee Ju-Yeon, Ryu Seung-Hyung, Kim Jae, Kweon Young, Kim Sung Effects of Dietary Habits on General and Abdominal Obesity in Community-dwelling Patients with Schizophrenia. CLINICAL PSYCHOPHARMACOLOGY AND NEUROSCIENCE. 2023;21(2):68-76.

5858. Lee S, Chan YYL, Kwok K, Hsu LKG Relationship between control and the intermediate term outcome of anorexia nervosa in Hong Kong. AUSTRALIAN AND NEW ZEALAND JOURNAL OF PSYCHIATRY. 2005;39(3):141-145.

5859. Pilar Calvo-Rivera Maria, Isabel Navarrete-Paez Maria, Bodoano Isabel, Gutierrez-Rojas Luis Comorbidity Between Anorexia Nervosa and Depressive Disorder: A Narrative Review. PSYCHIATRY INVESTIGATION. 2022;19(3):155-163.

5860. Takada S., Sampaio C., Allemandi W., Ito P., Takase L., Nogueira M. A modified rat model of neonatal anoxia: Development and evaluation by pulseoximetry, arterial gasometry and Fos immunoreactivity. JOURNAL OF NEUROSCIENCE METHODS. 2011;198(1):62-69.

5861. Nilsson Franciska, Madsen Jens, Jensen Andreas, Olsen Birthe, Johannesen Jesper High prevalence of disordered eating behavior in Danish children and adolescents with type 1 diabetes. PEDIATRIC DIABETES. 2020;21(6):1043-1049.

5862. Verstraeten Roosmarijn, Leroy Jef, Pieniak Zuzanna, Ochoa-Aviles Angelica, Holdsworth Michelle, Verbeke Wim, Maes Lea, Kolsteren Patrick Individual and Environmental Factors Influencing Adolescents' Dietary Behavior in Low- and Middle-Income Settings. PLOS ONE. 2016;11(7):.

5863. Draper H Anorexia nervosa and respecting a refusal of life-prolonging therapy: A limited justification. BIOETHICS. 2000;14(2):120-133.

5864. Fitzsimmons-Craft Ellen, Taylor C., Newman Michelle, Zainal Nur, Rojas-Ashe Elsa, Lipson Sarah, Firebaugh Marie-Laure, Ceglarek Peter, Topooco Naira, Jacobson Nicholas, Graham Andrea, Kim Hyungjin, Eisenberg Daniel, Wilfley Denise Harnessing mobile technology to reduce mental health disorders in college populations: A randomized controlled trial study protocol. CONTEMPORARY CLINICAL TRIALS. 2021;103():.

5865. Haddad GG, Sun YA, Wyman RJ, Xu T Genetic basis of tolerance to O-2 deprivation in Drosophila melanogaster. PROCEEDINGS OF THE NATIONAL ACADEMY OF SCIENCES OF THE UNITED STATES OF AMERICA. 1997;94(20):10809-10812.

5866. Gumz Antje, Weigel Angelika, Wegscheider Karl, Romer Georg, Loewe Bernd The psychenet public health intervention for anorexia nervosa: a pre-post-evaluation study in a female patient sample. PRIMARY HEALTH CARE RESEARCH AND DEVELOPMENT. 2018;19(1):42-52.

5867. Norton Maria, Dew Jeffrey, Smith Heeyoung, Fauth Elizabeth, Piercy Kathleen, Breitner John, Tschanz JoAnn, Wengreen Heidi, Welsh-Bohmer Kathleen, Investigators Cache Lifestyle Behavior Pattern Is Associated with Different levels of Risk for Incident Dementia and Alzheimer's Disease: The Cache County Study. JOURNAL OF THE AMERICAN GERIATRICS SOCIETY. 2012;60(3):405-412.

5868. Martinez-Gomez David, Veses Ana, Gomez-Martinez Sonia, Heredia Fatima, Castillo Ruth, Santaliestra-Pasias Alba, Elisa Calle Maria, Garcia-Fuentes Miguel, Luis Veiga Oscar, Marcos Ascension Television viewing time and risk of eating disorders in Spanish adolescents: AVENA and AFINOS studies. PEDIATRICS INTERNATIONAL. 2015;57(3):455-460.

5869. St John WM Rostral medullary respiratory neuronal activities of decerebrate cats in eupnea, apneusis and gasping. RESPIRATION PHYSIOLOGY. 1999;116(1):47-65.

5870. Godart N., Perdereau F., Rein Z., Berthoz S., Wallier J., Jeammet Ph., Flament M. Comorbidity studies of eating disorders and mood disorders. Critical review of the literature. JOURNAL OF AFFECTIVE DISORDERS. 2007;97(1-3):37-49.

5871. Colton Patricia, Olmsted Marion, Daneman Denis, Rodin Gary Depression, disturbed eating behavior, and metabolic control in teenage girls with type 1 diabetes. PEDIATRIC DIABETES. 2013;14(5):372-376.

5872. Kucukgoncu Suat, Bestepe Emrem Night Eating Syndrome in Major Depression and Anxiety Disorders. NOROPSIKIYATRI ARSIVI-ARCHIVES OF NEUROPSYCHIATRY. 2014;51(4):368-375.

5873. Zong Xin-Nan, Li Hui, Zhang Ya-Qin Family-related risk factors of obesity among preschool children: results from a series of national epidemiological surveys in China. BMC PUBLIC HEALTH. 2015;15():.

5874. Brotons Carlos, Bulc Mateja, Sammut Mario, Sheehan Mary, Silva Martins Carlos, Bjorkelund Cecilia, Drenthen Antonius, Duhot Didier, Goerpelioglui Sueleyman, Jurgova Eva, Keinanen-Kiukkanniemi Sirkka, Kotanyi Peter, Markou Valia, Moral Irene, Mortsiefer Achim, Pas Leo, Pichler Ingrid, Sghedoni Donatella, Tataradze Revaz, Thireos Eleftherios, Valius Leonas, Vuchak Jasna, Collins Claire, Cornelis Esther, Ciurana Ramon, Kloppe Pilar, Mierzecki Artur, Nadaraia Kakha, Godycki-Cwirko Maciek Attitudes toward preventive services and lifestyle: the views of primary care patients in Europe. The EUROPREVIEW patient study. FAMILY PRACTICE. 2012;29(1):i168-i176.

5875. McIntyre M, Berry DR, McNeil B Response of Penicillium chrysogenum to oxygen starvation in glucose- and nitrogen-limited chemostat cultures. ENZYME AND MICROBIAL TECHNOLOGY. 1999;25(3-5):447-454.

5876. Tayyem Reema, Qalqili Tamara, Ajeen Rawan, Rayyan Yaser Dietary Patterns and the Risk of Inflammatory Bowel Disease: Findings from a Case-Control Study. NUTRIENTS. 2021;13(6):.

5877. Erbacher Gabriele, Bertsch Tobias Lipoedema and Pain: What is the role of the psyche? Results of a pilot study with 150 patients with Lipoedema. PHLEBOLOGIE. 2020;49(05):305-316.

5878. Miao Yan, Ding Zuochuan, Zou Zhimiao, Yang Yang, Yang Min, Zhang Xiaoqian, Li Zeyang, Zhou Liang, Zhang Limin, Zhang Xue, Du Dunfeng, Jiang Fengchao, Zhou Ping Inhibition of MyD88 by a novel inhibitor reverses two-thirds of the infarct area in myocardial ischemia and reperfusion injury. AMERICAN JOURNAL OF TRANSLATIONAL RESEARCH. 2020;12(9):5151-5169.

5879. Vlaskamp Danique, Shaw Benjamin, Burgess Rosemary, Mei Davide, Montomoli Martino, Xie Han, Myers Candace, Bennett Mark, XiangWei Wenshu, Williams Danielle, Maas Saskia, Brooks Alice, Mancini Grazia, Laar Ingrid, Hagen Johanna, Ware Tyson, Webster Richard, Malone Stephen, Berkovic Samuel, Kalnins Renate, Sicca Federico, Korenke G., Ravenswaaij-Arts Conny, Hildebrand Michael, Mefford Heather, Jiang Yuwu, Guerrini Renzo, Scheffer Ingrid SYNGAP1 encephalopathy A distinctive generalized developmental and epileptic encephalopathy. NEUROLOGY. 2019;92(2):E96-E107.

5880. Yoo Yang, Yu Boas, Choi Eun-Hi A Comparison Study: The Risk Factors in the Lifestyles of Thyroid Cancer Patients and Healthy Adults of South Korea. CANCER NURSING. 2018;41(1):E48-E56.

5881. Simpson Courtney, Burnette C., Mazzeo Suzanne Integrating eating disorder and weight gain prevention: a pilot and feasibility trial of INSPIRE. EATING AND WEIGHT DISORDERS-STUDIES ON ANOREXIA BULIMIA AND OBESITY. 2020;25(3):761-775.

5882. Hakulinen Christian, Mok Pearl, Horsdal Henriette, Pedersen Carsten, Mortensen Preben, Agerbo Esben, Webb Roger Parental income as a marker for socioeconomic position during childhood and later risk of developing a secondary care-diagnosed mental disorder examined across the full diagnostic spectrum: a national cohort study. BMC MEDICINE. 2020;18(1):.

5883. Manole Mioara, Hickey Robert, Momoi Nobuo, Tobita Kimimasa, Tinney Joseph, Suciu Gabriel, Johnnides Michael, Clark Robert, Keller Bradley Preterminal gasping during hypoxic cardiac arrest increases cardiac function in immature rats. PEDIATRIC RESEARCH. 2006;60(2):174-179.

5884. Watanabe K., Iwahara C., Nakayama H., Iwabuchi K., Matsukawa T., Yokoyama K., Yamaguchi K., Kamiyama Y., Inada E. Sevoflurane suppresses tumour necrosis factor-alpha-induced inflammatory responses in small airway epithelial cells after anoxia/reoxygenation. BRITISH JOURNAL OF ANAESTHESIA. 2013;110(4):637-645.

5885. Kaltiala-Heino R, Rimpela M, Rissanen A, Rantanen P Early puberty and early sexual activity are associated with bulimic-type eating pathology in middle adolescence. JOURNAL OF ADOLESCENT HEALTH. 2001;28(4):346-352.

5886. Yavuz Ayten, Altinsoy Canan The relationship between chronotype, night eating behavior and fear of COVID-19 in academics. CHRONOBIOLOGY INTERNATIONAL.

2022;39(10):1359-1367.

5887. Turk JC, Prentice WE, Chappell S, Shields EW Collegiate coaches' knowledge of eating disorders. JOURNAL OF ATHLETIC TRAINING. 1999;34(1):19-24.

5888. Stice Eric, Bohon Cara, Marti C., Fischer Kathryn Subtyping Women With Bulimia Nervosa Along Dietary and Negative Affect Dimensions: Further Evidence of Reliability and Validity. JOURNAL OF CONSULTING AND CLINICAL PSYCHOLOGY. 2008;76(6):1022-1033.

5889. Goovaerts P. Geostatistics: a common link between medical geography, mathematical geology, and medical geology. JOURNAL OF THE SOUTHERN AFRICAN INSTITUTE OF MINING AND METALLURGY. 2014;114(8):605-612.

5890. Casagrande Breno, Estadella Debora Withdrawing from obesogenic diets: benefits and barriers in the short- and long-term in rodent models. AMERICAN JOURNAL OF PHYSIOLOGY-ENDOCRINOLOGY AND METABOLISM. 2020;319(3):E485-E493.

5891. Kimura Mitsuru, Miyakawa Tomohiro, Matsushita Sachio, So Mirai, Higuchi Susumu Gender Differences in the Effects of ADH1B and ALDH2 Polymorphisms on Alcoholism. ALCOHOLISM-CLINICAL AND EXPERIMENTAL RESEARCH. 2011;35(11):1923-1927.

5892. Bernagie Chiara, Danckaerts Marina, Wampers Martien, De Hert Marc Aripiprazole and Acute Extrapyramidal Symptoms in Children and Adolescents: A Meta-Analysis. CNS DRUGS. 2016;30(9):807-818.

5893. McLean Sian, Paxton Susan, Wertheim Eleanor Mediators of the relationship between media literacy and body dissatisfaction in early adolescent girls: Implications for prevention. BODY IMAGE. 2013;10(3):282-289.

5894. Neumark-Sztainer D, Sherwood NE, French SA, Jeffery RW Weight control behaviors among adult men and women: Cause for concern?. OBESITY RESEARCH. 1999;7(2):179-188.

5895. Li Quan, Yu Wei-Feng, Zhou Mai-Tao, Lu Xin, Yang Li-Qun, Zhu Ming, Song Jian-Gang, Lu Jun-Hua Isoflurane preserves energy balance in isolated hepatocytes during in vitro anoxia/reoxygenation. WORLD JOURNAL OF GASTROENTEROLOGY. 2005;11(25):3920-

3924.

5896. Dolcini-Catania Luciano, Byrne Michelle, Whittle Sarah, Schwartz Orli, Simmons Julian, Allen Nicholas Temperament and Symptom Pathways to the Development of Adolescent Depression. JOURNAL OF ABNORMAL CHILD PSYCHOLOGY. 2020;48(6):839-849.

5897. Liu Shan-Xin, Zhang Yu, Wang Yun-Fan, Li Xiao-Chun, Xiang Mei-Xiang, Bian Chang, Chen Peng Upregulation of heme oxygenase-1 expression by hydroxysafflor yellow A conferring protection from anoxia/reoxygenation-induced apoptosis in H9c2 cardiomyocytes. INTERNATIONAL JOURNAL OF CARDIOLOGY. 2012;160(2):95-101.

5898. Tandon Shashank, Keefe Kristen, Taha Sharif Mu opioid receptor signaling in the nucleus accumbens shell increases responsiveness of satiety-modulated lateral hypothalamus neurons. EUROPEAN JOURNAL OF NEUROSCIENCE. 2017;45(11):1418-1430.

5899. Bowman Chelsie, Young Seth, Kaljo Dimitri, Eriksson Mats, Them Theodore, Hints Olle, Martma Tartu, Owens Jeremy Linking the progressive expansion of reducing conditions to a stepwise mass extinction event in the late Silurian oceans. GEOLOGY. 2019;47(10):968-972.

5900. Melisse Bernou, Furth Eric, Beurs Edwin The Saudi-Arabic adaptation of the Body Shape Questionnaire (BSQ34): Psychometrics and norms of the full version and the short version (BSQ8C). FRONTIERS IN PSYCHOLOGY. 2022;13():.

5901. Toniolo Ricardo, Caetano Sheila, Silva Patricia, Lafer Beny Clinical significance of lifetime panic disorder in the course of bipolar disorder type I. COMPREHENSIVE PSYCHIATRY. 2009;50(1):9-12.

5902. Machado Barbara, Goncalves Sonia, Martins Carla, Brandao Isabel, Roma-Torres Antonio, Hoek Hans, Machado Paulo Anorexia nervosa versus bulimia nervosa: differences based on retrospective correlates in a case-control study. EATING AND WEIGHT DISORDERS-STUDIES ON ANOREXIA BULIMIA AND OBESITY. 2016;21(2):185-197.

5903. Epstein Leonard, Leddy John, Temple Jennifer, Faith Myles Food reinforcement and eating: A multilevel analysis. PSYCHOLOGICAL BULLETIN. 2007;133(5):884-906.

5904. Tso Leopoldo, Leis Luciana, Glina Claudia, Busso Cristiano, Romano Rodrigo, Busso Newton, Wonchockier Roberta, Glina Sidney Does the controlled ovarian stimulation increase the weight of women undergoing IVF treatment?. EUROPEAN JOURNAL OF OBSTETRICS \& GYNECOLOGY AND REPRODUCTIVE BIOLOGY. 2021;263():205-209.

5905. Vander Wal Jillon, Mitchell Elisha Psychological Complications of Pediatric Obesity. PEDIATRIC CLINICS OF NORTH AMERICA. 2011;58(6):1393+.

5906. Genuis S., Willows N., Jardine C., Nation Alexander Through the lens of our cameras: children's lived experience with food security in a Canadian Indigenous community. CHILD CARE HEALTH AND DEVELOPMENT. 2015;41(4):600-610.

5907. Bar Rachel, Cassin Stephanie, Dionne Michelle Eating disorder prevention initiatives for athletes: A review. EUROPEAN JOURNAL OF SPORT SCIENCE. 2016;16(3):325-335.

5908. Shin DSH, Wilkie MP, Pamenter ME, Buck LT Calcium and protein phosphatase receptor activity in 1/2A attenuate N-methyl-D-aspartate the anoxic turtle cortex. COMPARATIVE BIOCHEMISTRY AND PHYSIOLOGY A-MOLECULAR \& INTEGRATIVE PHYSIOLOGY. 2005;142(1):50-57.

5909. Garcia Melawhy, Gatdula Natalia, Bonilla Erika, Frank Gail, Bird Mara, Rascon Mayra, Rios-Ellis Britt Engaging Intergenerational Hispanics/Latinos to Examine Factors Influencing Childhood Obesity Using the PRECEDE-PROCEED Model. MATERNAL AND CHILD HEALTH JOURNAL. 2019;23(6):802-810.

5910. Borisenkov Mikhail, Tserne Tatyana, Popov Sergey, Bakutova Larisa, Pecherkina Anna, Dorogina Olga, Martinson Ekaterina, Vetosheva Valentina, Gubin Denis, Solovieva Svetlana, Turovinina Elena, Symaniuk Elvira Food preferences and YFAS/YFAS-C scores in schoolchildren and university students. EATING AND WEIGHT DISORDERS-STUDIES ON ANOREXIA BULIMIA AND OBESITY. 2021;26(7):2333-2343.

5911. Berlin KS, Sass DA, Davies WH, Haines AA Impact of diabetes disclosure on perceptions of eating and self-care behaviors. DIABETES EDUCATOR. 2002;28(5):809-816.

5912. Forouhi Nita Embracing complexity: making sense of diet, nutrition, obesity and type 2 diabetes. DIABETOLOGIA. 2023;66(5):786-799.

5913. Isaksson Martina, Ghaderi Ata, Ramklint Mia, Wolf-Arehult Martina Radically open dialectical behavior therapy for anorexia nervosa: A multiple baseline single-case experimental design study across 13 cases. JOURNAL OF BEHAVIOR THERAPY AND EXPERIMENTAL PSYCHIATRY. 2021;71():.

5914. Pineda Garcia Gisela, Gomez-Peresmitre Gilda Eating disorders prevention program: A pilot study based on cognitive dissonance theory. REVISTA MEXICANA DE PSICOLOGIA. 2006;23(1):87-95.

5915. Sharma Shreya, Srinivasaraghavan Rangan, Krishnamurthy Sriram Central Nervous System Symptoms Due to Transient Methemoglobinemia in a Child With G6PD Deficiency. JOURNAL OF PEDIATRIC HEMATOLOGY ONCOLOGY. 2017;39(1):E27-E28.

5916. Darby Anita, Hay Phillipa, Mond Jonathan, Quirk Frances, Buttner Petra, Kennedy Lee The Rising Prevalence of Comorbid Obesity and Eating Disorder Behaviors from 1995 to 2005. INTERNATIONAL JOURNAL OF EATING DISORDERS. 2009;42(2):104-108.

5917. Tian GF, Baker AJ Glycolysis prevents anoxia-induced synaptic transmission damage in rat hippocampal slices. JOURNAL OF NEUROPHYSIOLOGY. 2000;83(4):1830-1839.

5918. Anderson SJ, Griesemer BA, Johnson MD, Martin TJ, McLain LG, Rowland TW, Small E, LeBlanc C, Krein C, Young JC, Malina R, Reed FE, Washington RL, Bar-Or O, Loucks A, Tanner S, Newland H, Pediat Amer Medical concerns in the female athlete. PEDIATRICS. 2000;106(3):610-613.

5919. Martinis Irena, Vrca Andelko, Bevanda Milenko, Botic-Stefanec Sanja, Badak Jasna, Kuster Dinka, Suttil Tatjana, Lasic Mirna, Bolaric Kim, Bituh Martina NUTRITIONAL ASSESSMENT OF PATIENTS WITH PRIMARY PROGRESSIVE DEMENTIA AT THE TIME OF DIAGNOSIS. PSYCHIATRIA DANUBINA. 2021;33(13):S226-S235.

5920. Winkelman John Efficacy and tolerability of open-label topiramate in the treatment of sleep-related eating disorder: A retrospective case series. JOURNAL OF CLINICAL PSYCHIATRY. 2006;67(11):1729-1734.

5921. Dieteren Charlotte, Brouwer Werner, Exel Job How do combinations of unhealthy behaviors relate to attitudinal factors and subjective health among the adult population in the Netherlands?. BMC PUBLIC HEALTH. 2020;20(1):.

5922. Ramanathan Ramya, Mohan Janani, Ramesh Shanthi, Subramanian Sundari Knowledge Attitude and Practice among Mothers towards Childhood Obesity: A Cross-sectional Study. JOURNAL OF CLINICAL AND DIAGNOSTIC RESEARCH. 2022;16(7):SC5-SC9.

5923. Riviere Julie, Douilliez Celine Perfectionism, rumination, and gender are related to symptoms of eating disorders: A moderated mediation model. PERSONALITY AND INDIVIDUAL DIFFERENCES. 2017;116():63-68.

5924. Steegers Cathelijne, Dieleman Gwen, Moskalenko Valeria, Santos Susana, Hillegers Manon, White Tonya, Jansen Pauline The longitudinal relationship between set-shifting at 4 years of age and eating disorder related features at 9 years of age in the general pediatric population. INTERNATIONAL JOURNAL OF EATING DISORDERS. 2021;54(12):2180-2191.

5925. Dash Sarah, Clarke Gerard, Berk Michael, Jacka Felice The gut microbiome and diet in psychiatry: focus on depression. CURRENT OPINION IN PSYCHIATRY. 2015;28(1):1-6.

5926. Foster Hamish, Polz Peter, Mair Frances, Gill Jason, O'Donnell Catherine Understanding the influence of socioeconomic status on the association between combinations of lifestyle factors and adverse health outcomes: a systematic review protocol. BMJ OPEN. 2021;11(5):.

5927. Keser N., Cinar N., Dogu O., Gunduz H., Akdemir R., Altinkaynak S. Any difference in sociodemograpic variables and risk factors of patients hospitalised with cardiovascular diseasse (CVD)?. HEALTHMED. 2012;6(7):2325-2331.

5928. Sachinidis Alexandros, Doumas Michael, Imprialos Konstantinos, Stavropoulos Konstantinos, Katsimardou Alexandra, Athyros Vasilios Dysmetabolic Iron Overload in Metabolic Syndrome. CURRENT PHARMACEUTICAL DESIGN. 2020;26(10):1019-1024.

5929. Christensen Bodil, Schmidt Julie, Nielsen Mette, Taekker Louise, Holm Lotte, Lunn Susanne, Bredie Wender, Ritz Christian, Holst Jens, Hansen Torben, Hilbert Anja, Roux Carel, Hulme Oliver, Siebner Hartwig, Morville Tobias, Naver Lars, Floyd Andrea, Sjodin Anders Patient profiling for success after weight loss surgery (GO Bypass study): An interdisciplinary study protocol. CONTEMPORARY CLINICAL TRIALS COMMUNICATIONS. 2018;10():121-130.

5930. Akesdotter Cecilia, Kentta Goran, Eloranta Sandra, Franck Johan The prevalence of mental health problems in elite athletes. JOURNAL OF SCIENCE AND MEDICINE IN SPORT.

2020;23(4):329-335.

5931. Sand-Jensen Kaj, Moller Claus, Borum Jens High resistance of oligotrophic isoetid plants to oxic and anoxic dark exposure. FRESHWATER BIOLOGY. 2015;60(5):1044-1051.

5932. Ferragud Antonio, Velazquez-Sanchez Clara, Al Abdullatif Ali, Sabino Valentina, Cottone Pietro Withdrawal from Extended, Intermittent Access to A Highly Palatable Diet Impairs Hippocampal Memory Function and Neurogenesis: Effects of Memantine. NUTRIENTS. 2020;12(5):.

5933. Jacobi C, Dahme B, Rustenbach S Comparison of controlled psychotherapeutic and pharmacological trials for bulimia and anorexia nervosa. PSYCHOTHERAPIE PSYCHOSOMATIK MEDIZINISCHE PSYCHOLOGIE. 1997;47(9-10):346-364.

5934. Aparicio-Llopis Estefania, Canals Josefa, Arija Victoria Dietary Intake According to the Course of Symptoms of Eating Disorders in a School-based Follow-up Study of Adolescents. EUROPEAN EATING DISORDERS REVIEW. 2014;22(6):412-422.

5935. Agostino Holly, Erdstein Julius, Di Meglio Giuseppina Shifting Paradigms: Continuous Nasogastric Feeding With High Caloric Intakes in Anorexia Nervosa. JOURNAL OF ADOLESCENT HEALTH. 2013;53(5):590-594.

5936. Andreoli Cristiana, Vieira-Ribeiro Sarah, Almeida Fonseca Poliana, Bandeira Moreira Ana, Rocha Ribeiro Sonia, Morais Mauro, Castro Franceschini Sylvia Eating habits, lifestyle and intestinal constipation in children aged four to seven years. NUTRICION HOSPITALARIA. 2019;36(1):25-31.

5937. Yemelyanov V., Lastochkin V., Prikaziuk E., Chirkova T. Activities of Catalase and Peroxidase in Wheat and Rice Plants under Conditions of Anoxia and Post-Anoxic Aeration. RUSSIAN JOURNAL OF PLANT PHYSIOLOGY. 2022;69(6):.

5938. Berg FM Health risks associated with weight loss and obesity treatment programs. JOURNAL OF SOCIAL ISSUES. 1999;55(2):277-297.

5939. Pullmer Rachelle, Kerrigan Stephanie, Grilo Carlos, Lydecker Janet Factors Linking Perceived Discrimination and Weight Bias Internalization to Body Appreciation and Eating Pathology: A Moderated Mediation Analysis of Self-Compassion and Psychological Distress.

STIGMA AND HEALTH. 2021;6(4):494-501.

5940. Chavez-Leon Enrique, Ng Bernardo, Ontiveros-Uribe Martha Pharmacological treatment of borderline personality disorder. SALUD MENTAL. 2006;29(5):16-24.

5941. Su Te-Jen, Lee Feng-Chun, Sun Cheuk-Kwan, Ke Fu-Xiang, Wang Shih-Ming, Huang Ming-Chih The Application of Convolutional Neural Network Combined with Fuzzy Algorithm in Colorectal Endoscopy for Tumor Assessment. DISCRETE DYNAMICS IN NATURE AND SOCIETY. 2022;2022():.

5942. Bullivant Bianca, Mitchison Deborah, Skromanis Sarah, Mond Jonathan Elucidating individuals' beliefs about the severity of eating disorders and obesity: implications for public health programs. EATING AND WEIGHT DISORDERS-STUDIES ON ANOREXIA BULIMIA AND OBESITY. 2020;25(4):929-938.

5943. Korge P, Honda HM, Weiss JN Regulation of the mitochondrial permeability transition by matrix Ca2+ and voltage during anoxia/reoxygenation. AMERICAN JOURNAL OF PHYSIOLOGY-CELL PHYSIOLOGY. 2001;280(3):C517-C526.

5944. Bickler PE, Donohoe PH, Buck LT Hypoxia-induced silencing of NMDA receptors in turtle neurons. JOURNAL OF NEUROSCIENCE. 2000;20(10):3522-3528.

5945. Cooper Marita, Guarda Angela, Petterway Felicia, Schreyer Colleen Change in normative eating self-efficacy is associated with six-month weight restoration following inpatient treatment for anorexia nervosa. EATING BEHAVIORS. 2021;42():.

5946. Yon L., Doyen C., Asch M., Cook-Darzens S., Mouren M. Treatment of anorexia nervosa in young patients in a special care unit at Robert-Debre Hospital (Paris): Guidelines and practical methods. ARCHIVES DE PEDIATRIE. 2009;16(11):1491-1498.

5947. Zhao Zhihao, Shi Aimin, Wang Qiang, Zhou Jinrong High Oleic Acid Peanut Oil and Extra Virgin Olive Oil Supplementation Attenuate Metabolic Syndrome in Rats by Modulating the Gut Microbiota. NUTRIENTS. 2019;11(12):.

5948. Ludvigsson Jonas, Olen Ola, Larsson Henrik, Halfvarson Jonas, Almqvist Catarina, Lichtenstein Paul, Butwicka Agnieszka Association Between Inflammatory Bowel Disease and Psychiatric Morbidity and Suicide: A Swedish Nationwide Population-Based Cohort

Study With Sibling Comparisons. JOURNAL OF CROHNS \& COLITIS. 2021;15(11):1824-1836.

5949. Ciao Anna, Munson Bethany, Pringle Kevin, Roberts Savannah, Lalgee Indira, Lawley Kendall, Brewster Janae Inclusive Dissonance-Based Body Image Interventions for College Students: Two Randomized-Controlled Trials of the EVERYbody Project. JOURNAL OF CONSULTING AND CLINICAL PSYCHOLOGY. 2021;89(4):301-315.

5950. Ying Z, Yong W, Avigne WT, Koch KE Differential regulation of sugar-sensitive sucrose synthases by hypoxia and anoxia indicate complementary transcriptional and posttranscriptional responses. PLANT PHYSIOLOGY. 1998;116(4):1573-1583.

5951. Rui Tao, Tang Qizhu IL-33 Attenuates Anoxia/Reoxygenation-Induced Cardiomyocyte Apoptosis by Inhibition of PKC beta/JNK Pathway. PLOS ONE. 2013;8(2):.

5952. Scott-Sheldon Lori, Carey Kate, Carey Michael Health behavior and college students: Does Greek affiliation matter?. JOURNAL OF BEHAVIORAL MEDICINE. 2008;31(1):61-70.

5953. Lunn Trevor, Nowson Caryl, Worsley Anthony, Torres Susan Does personality affect dietary intake?. NUTRITION. 2014;30(4):403-409.

5954. Chatoor Irene, Webb Lauren, Kerzner Benny Anorexia nervosa and depression in a 5-year-old girl: Treatment with focused family play therapy and medication. INTERNATIONAL JOURNAL OF EATING DISORDERS. 2019;52(9):1065-1069.

5955. Ghenciulescu Ana, Park Rebecca, Burnet Philip The Gut Microbiome in Anorexia Nervosa: Friend or Foe?. FRONTIERS IN PSYCHIATRY. 2021;11():.

5956. Chua Joelle, Tam Wilson, Shorey Shefaly Research Review: Effectiveness of universal eating disorder prevention interventions in improving body image among children: a systematic review and meta-analysis. JOURNAL OF CHILD PSYCHOLOGY AND PSYCHIATRY. 2020;61(5):522-535.

5957. Temple Norman A Comparison of Strategies to Improve Population Diets: Government Policy versus Education and Advice. JOURNAL OF NUTRITION AND METABOLISM. 2020;2020():.

5958. Sainsbury Emma, Colagiuri Stephen, Magnusson Roger An audit of food and beverage advertising on the Sydney metropolitan train network: regulation and policy implications. BMC PUBLIC HEALTH. 2017;17():.

5959. Scutt Eleanor, Langdon-Daly Jasmin, Smithson Janet Experiences of eating difficulties in siblings of people with anorexia nervosa: a reflexive thematic analysis. JOURNAL OF EATING DISORDERS. 2022;10(1):.

5960. Power Thomas, Bindler Ruth, Goetz Summer, Daratha Kenneth Obesity Prevention in Early Adolescence: Student, Parent, and Teacher Views. JOURNAL OF SCHOOL HEALTH. 2010;80(1):13-19.

5961. Teufel-Shone Nicolette, Jiang Luohua, Beals Janette, Henderson William, Zhang Lijing, Acton Kelly, Roubideaux Yvette, Manson Spero Demographic characteristics and food choices of participants in the Special Diabetes Program for American Indians Diabetes Prevention Demonstration Project. ETHNICITY \& HEALTH. 2015;20(4):327-340.

5962. Varnado-Sullivan PJ, Zucker N The body logic program for adolescents - A treatment manual for the prevention of eating disorders. BEHAVIOR MODIFICATION. 2004;28(6):854-875.

5963. Skutnik Marta, Rychter Anna Differential response of antioxidant systems in leaves and roots of barley subjected to anoxia and post-anoxia. JOURNAL OF PLANT PHYSIOLOGY. 2009;166(9):926-937.

5964. Juli Maria THE ``IMPERFECT BEAUTY{''} IN EATING DISORDERS. PSYCHIATRIA DANUBINA. 2019;31(3):S447-S451.

5965. Parmar Deepika, Tabler Jennifer, Okumura Megumi, Nagata Jason Investigating Protective Factors Associated With Mental Health Outcomes in Sexual Minority Youth. JOURNAL OF ADOLESCENT HEALTH. 2022;70(3):470-477.

5966. Paris Jason, Franco Christine, Sodano Ruthlyn, Freidenberg Brian, Gordis Elana, Anderson Drew, Forsyth John, Wulfert Edelgard, Frye Cheryl Sex differences in salivary cortisol in response to acute stressors among healthy participants, in recreational or pathological gamblers, and in those with posttraumatic stress disorder. HORMONES AND BEHAVIOR. 2010;57(1, SI):35-45.

5967. Skinner Janelle, Jebeile Hiba, Burrows Tracy Food addiction and mental health in adolescents: a systematic review. LANCET CHILD \& ADOLESCENT HEALTH. 2021;5(10):751-766.

5968. Dailey Simon Does the salience of possible selves mediate the impact of approach and avoidance temperaments on women's weight-loss dieting?. PERSONALITY AND INDIVIDUAL DIFFERENCES. 2016;88():267-271.

5969. Herpertz-Dahlmann Beate, Elburg Annemarie, Castro-Fornieles Josefina, Schmidt Ulrike ESCAP Expert Paper: New developments in the diagnosis and treatment of adolescent anorexia nervosa-a European perspective. EUROPEAN CHILD \& ADOLESCENT PSYCHIATRY. 2015;24(10):1153-1167.

5970. Magalhaes Paula, Silva Catia, Pereira Beatriz, Figueiredo Gabriela, Guimaraes Ana, Pereira Armanda, Rosario Pedro An online-based intervention to promote healthy eating through self-regulation among children: study protocol for a randomized controlled trial. TRIALS. 2020;21(1):.

5971. Susin Nathalia, Boff Raquel, Brusius Ludwig Martha, Pandolfo Feoli Ana, Silva Andreia, Macagnan Fabricio, Oliveira Margareth Predictors of adherence in a prevention program for patients with metabolic syndrome. JOURNAL OF HEALTH PSYCHOLOGY. 2016;21(10):2156-2167.

5972. Christian Caroline, Levinson Cheri An integrated review of fear and avoidance learning in anxiety disorders and application to eating disorders. NEW IDEAS IN PSYCHOLOGY. 2022;67():.

5973. Castro Maria, Villagarcia Hernan, Roman Carolina, Maiztegui Barbara, Flores Luis, Schinella Guillermo, Massa Maria, Francini Flavio Chronological Appearance of Endocrine and Metabolic Dysfunctions Induced by an Unhealthy Diet in Rats. MEDICINA-LITHUANIA. 2022;58(1):.

5974. Sofija Ernesta, Harris Neil, Phung Dung, Sav Adem, Sebar Bernadette Does Flourishing Reduce Engagement in Unhealthy and Risky Lifestyle Behaviours in Emerging Adults?. INTERNATIONAL JOURNAL OF ENVIRONMENTAL RESEARCH AND PUBLIC HEALTH. 2020;17(24):.

5975. NeumarkSztainer D, Story M, Resnick MD, Blum RW Psychosocial concerns and weight control behaviors among overweight and nonoverweight Native American adolescents. JOURNAL OF THE AMERICAN DIETETIC ASSOCIATION. 1997;97(6):598-604.

5976. Ayran Gulsun, Suleyman Zeynep, Avci Umit, Arik Umut The effect of Internet addiction on eating attitude and body image in university students. JOURNAL OF CHILD AND ADOLESCENT PSYCHIATRIC NURSING. 2021;34(3):199-205.

5977. Kukreti Shikha, Yu Tsung, Chiu Po, Strong Carol Clustering of Modifiable Behavioral Risk Factors and Their Association with All-Cause Mortality in Taiwan's Adult Population: a Latent Class Analysis. INTERNATIONAL JOURNAL OF BEHAVIORAL MEDICINE. 2022;29(5):565-574.

5978. Matheson Brittany, Camacho Casandra, Peterson Carol, Rhee Kyung, Rydell Sarah, Zucker Nancy, Boutelle Kerri The relationship between parent feeding styles and general parenting with loss of control eating in treatment-seeking overweight and obese children. INTERNATIONAL JOURNAL OF EATING DISORDERS. 2015;48(7):1047-1055.

5979. Blondin Soline, Meilleur Dominique, Taddeo Danielle, Frappier Jean-Yves Caregiving experience and expressed emotion among parents of adolescents suffering from anorexia nervosa following illness onset. EATING DISORDERS. 2019;27(5):453-470.

5980. Levinson Cheri, Ralph-Nearman Christina, Brown Mackenzie, Gardner Taylor, Cardi Valentina, Treasure Janet, Purves Kirstin, Eley Thalia A pilot randomized control trial of online exposure for eating disorders and mechanisms of change delivered after discharge from intensive eating disorder care: A registered report. INTERNATIONAL JOURNAL OF EATING DISORDERS. 2021;54(11):2066-2074.

5981. Lande Marie, Rosenvinge Jan, Skeie Guri, Rylander Charlotta Prevalence and correlates of self-reported disordered eating: A cross-sectional study among 90 592 middle-aged Norwegian women. PLOS ONE. 2019;14(1):.

5982. Kovacs Viktoria, Messing Sven, Sandu Petru, Nardone Paola, Pizzi Enrica, Hassapidou Maria, Brukalo Katarzyna, Tecklenburg Ernestine, Abu-Omar Karim, Consortium JANPA Improving the food environment in kindergartens and schools: An overview of policies and policy opportunities in Europe. FOOD POLICY. 2020;96():.

5983. Breedon Sarah, Hadj-Moussa Hanane, Storey Kenneth Nrf2 activates antioxidant enzymes in the anoxia-tolerant red-eared slider turtle, Trachemys scripta elegans. JOURNAL OF EXPERIMENTAL ZOOLOGY PART A-ECOLOGICAL AND INTEGRATIVE PHYSIOLOGY. 2021;335(4):426-435.

5984. Kinderlehrer Daniel Anorexia Nervosa Caused by Polymicrobial Tick-Borne Infections: A Case Study. INTERNATIONAL MEDICAL CASE REPORTS JOURNAL. 2021;14():279-287.

5985. Carava Elena, Moretto Paola, Caon Ilaria, Parnigoni Arianna, Passi Alberto, Karousou Evgenia, Vigetti Davide, Canino Jessica, Canobbio Ilaria, Viola Manuela HA and HS Changes in Endothelial Inflammatory Activation. BIOMOLECULES. 2021;11(6):.

5986. Kalinowska Sylwia, Trzesniowska-Drukala Beata, Kloda Karolina, Safranow Krzysztof, Misiak Blazej, Cyran Agnieszka, Samochowiec Jerzy The Association between Lifestyle Choices and Schizophrenia Symptoms. JOURNAL OF CLINICAL MEDICINE. 2021;10(1):.

5987. Rodgers Rachel, Donovan Elizabeth, Cousineau Tara, McGowan Kayla, Yates Kayla, Cook Elizabeth, Lowy Alice, Franko Debra Ethnic and racial diversity in eating disorder prevention trials. EATING DISORDERS. 2019;27(2, SI):168-182.

5988. Mesirow Maurissa, Cecil Charlotte, Maughan Barbara, Barker Edward Associations between Prenatal and Early Childhood Fish and Processed Food Intake, Conduct Problems, and Co-Occurring Difficulties. JOURNAL OF ABNORMAL CHILD PSYCHOLOGY. 2017;45(5):1039-1049.

5989. Sasaki R, Hirota K, Roth SH, Yamazaki M Anoxic depolarization of rat hippocampal slices is prevented by thiopental but not by propofol or isoflurane. BRITISH JOURNAL OF ANAESTHESIA. 2005;94(4):486-491.

5990. Jia P., Li M., Xue H., Lu L., Xu F., Wang Y. School environment and policies, child eating behavior and overweight/obesity in urban China: the childhood obesity study in China megacities. INTERNATIONAL JOURNAL OF OBESITY. 2017;41(5):813-819.

5991. Lipsitz JD, Fyer AJ, Paterniti BA, Klein DF Emetophobia: Preliminary results of an internet survey. DEPRESSION AND ANXIETY. 2001;14(2):149-152.

5992. Vyver Ellie, Steinegger Cathleen, Katzman Debra Eating disorders and menstrual dysfunction in adolescents. . 2008;1135():253-264.

5993. Schwarz Anne-Sophie, Nielsen Bent, Nielsen Anette Lifestyle factors in somatic patients with and without potential alcohol problems. JOURNAL OF PUBLIC HEALTH-HEIDELBERG. 2018;26(4):453-459.

5994. Sabel Allison, Rosen Elissa, Mehler Philip Severe Anorexia Nervosa in Males: Clinical Presentations and Medical Treatment. EATING DISORDERS. 2014;22(3):209-220.

5995. Carter Francis, Bulik Cynthia Childhood obesity prevention programs: How do they affect eating pathology and other psychological measures?. PSYCHOSOMATIC MEDICINE. 2008;70(3):363-371.

5996. CHEN J, DOCTOR RB, MANDEL LJ CYTOSKELETAL DISSOCIATION OF EZRIN DURING RENAL ANOXIA - ROLE IN MICROVILLAR INJURY. AMERICAN JOURNAL OF PHYSIOLOGY. 1994;267(3, 1):C784-C795.

5997. Choi So, Lee Kyung, Kim Soon, Lee Eun, Lee Yoo, Kim Yu, Yi Dae, Kim Ju, Kang Ben, Jang Hyo-Jeong, Hong Suk, Choi You, Kim Hyun Cardiac Complications Associated with Eating Disorders in Children: A Multicenter Retrospective Study. PEDIATRIC GASTROENTEROLOGY HEPATOLOGY \& NUTRITION. 2022;25(5):432-440.

5998. Glabska Dominika, Kolota Aleksandra, Lachowicz Katarzyna, Skolmowska Dominika, Stachon Malgorzata, Guzek Dominika The Influence of Vitamin D Intake and Status on Mental Health in Children: A Systematic Review. NUTRIENTS. 2021;13(3):.

5999. Brar BK, Stephanou A, Liao ZH, O'Leary RM, Pennica D, Yellon DM, Latchman DS Cardiotrophin-1 can protect cardiac myocytes from injury when added both prior to simulated ischaemia and at reoxygenation. CARDIOVASCULAR RESEARCH. 2001;51(2):265-274.

6000. Agher Dahbia, Sedki Karima, Despres Sylvie, Albinet Jean-Pierre, Jaulent Marie-Christine, Tsopra Rosy Encouraging Behavior Changes and Preventing Cardiovascular Diseases Using the Prevent Connect Mobile Health App: Conception and Evaluation of App Quality. JOURNAL OF MEDICAL INTERNET RESEARCH. 2022;24(1):.

6001. Tikk Kaja, Sookthai Disorn, Monni Stefano, Gross Marie-Luise, Lichy Christoph, Kloss Manja, Kaaks Rudolf Primary Preventive Potential for Stroke by Avoidance of Major Lifestyle Risk Factors The European Prospective Investigation Into Cancer and Nutrition-Heidelberg Cohort. STROKE. 2014;45(7):2041-2046.

6002. Himmelstein Mary, Puhl Rebecca, Quinn Diane Intersectionality: An Understudied Framework for Addressing Weight Stigma. AMERICAN JOURNAL OF PREVENTIVE MEDICINE. 2017;53(4):421-431.

6003. Angulo Macarena, Garcia Nuria, Carvalho Rui, Aragon Ignacio, Avila Belen, Portillo Rocio Hiperfosfatemia durante la renutricion en pacientes con anorexia nerviosa grave. ENDOCRINOLOGIA DIABETES Y NUTRICION. 2022;69(9):715-722.

6004. Volino-Souza Monica, Oliveira Gustavo, Conte-Junior Carlos, Alvares Thiago Covid-19 Quarantine: Impact of Lifestyle Behaviors Changes on Endothelial Function and Possible Protective Effect of Beetroot Juice. FRONTIERS IN NUTRITION. 2020;7():.

6005. Carmen Hernandez Maria, Alcocer Javier, Oseguera Luis, Escobar Elva Profundal benthic invertebrates in an oligotrophic tropical lake: different strategies for coping with anoxia. JOURNAL OF LIMNOLOGY. 2014;73(2):387-399.

6006. Kvaloy Kirsti, Holmen Jostein, Hveem Kristian, Holmen Turid Genetic Effects on Longitudinal Changes from Healthy to Adverse Weight and Metabolic Status-The HUNT Study. PLOS ONE. 2015;10(10):.

6007. Kang Joseph, Ciecierski Christina, Malin Emily, Carroll Allison, Gidea Marian, Craft Lynette, Spring Bonnie, Hitsman Brian A latent class analysis of cancer risk behaviors among US college students. PREVENTIVE MEDICINE. 2014;64():121-125.

6008. McAulay Claire, Mond Jonathan, Touyz Stephen Early intervention for bipolar disorder in adolescents: A psychosocial perspective. EARLY INTERVENTION IN PSYCHIATRY. 2018;12(3):286-291.

6009. Safiri Saeid, Noori Maryam, Nejadghaderi Seyed, Shamekh Ali, Karamzad Nahid, Sullman Mark, Grieger Jessica, Collins Gary, Abdollahi Morteza, Kolahi Ali-Asghar The estimated burden of bulimia nervosa in the Middle East and North Africa region, 1990-2019. INTERNATIONAL JOURNAL OF EATING DISORDERS. 2022;():.

6010. Shingleton Rebecca, Eddy Kamryn, Keshaviah Aparna, Franko Debra, Swanson Sonja, Yu Jessica, Krishna Meera, Nock Matthew, Herzog David Binge/Purge Thoughts in Nonsuicidal Self-Injurious Adolescents: An Ecological Momentary Analysis. INTERNATIONAL JOURNAL OF EATING DISORDERS. 2013;46(7):684-689.

6011. Kilpela Lisa, Hill Kaitlin, Kelly Mackenzie, Elmquist Joanna, Ottoson Paige, Keith Demetra, Hildebrandt Thomas, Becker Carolyn Reducing eating disorder risk factors: A controlled investigation of a blended task-shifting/train-the-trainer approach to dissemination and implementation. BEHAVIOUR RESEARCH AND THERAPY. 2014;63():70-82.

6012. Zemlyanskaya Yana, Valente Martina, Syurina Elena Orthorexia nervosa and Instagram: exploring the Russian-speaking conversation around \#op tau ope kappa c. EATING AND WEIGHT DISORDERS-STUDIES ON ANOREXIA BULIMIA AND OBESITY. 2022;27(3):1011-1020.

6013. Rothstein TL Recovery from near death following cerebral anoxia: A case report demonstrating superiority of median somatosensory evoked potentials over EEG in predicting a favorable outcome after cardiopulmonary resuscitation. RESUSCITATION. 2004;60(3):335-341.

6014. Lehnert T., Riedel-Heller S., Koenig H. Taxing unhealthy foods. A measure to promote public health in Germany?. BUNDESGESUNDHEITSBLATT-GESUNDHEITSFORSCHUNG-GESUNDHEITSSCHUTZ. 2013;56(4):562-570.

6015. Holm Astrid, Laursen Mai-Britt, Koch Maria, Jensen Jorgen, Diderichsen Finn The health benefits of selective taxation as an economic instrument in relation to IHD and nutrition-related cancers. PUBLIC HEALTH NUTRITION. 2013;16(12):2124-2131.

6016. Kikutani Takeshi, Ichikawa Yoko, Kitazume Eri, Mizukoshi Arato, Tohara Takashi, Takahashi Noriaki, Tamura Fumiyo, Matsutani Manami, Onishi Junko, Makino Eiichiro COVID-19 Infection-Related Weight Loss Decreases Eating/Swallowing Function in Schizophrenic Patients. NUTRIENTS. 2021;13(4):.

6017. Podrabsky Jason, Menze Michael, Hand Steven Long-Term Survival of Anoxia Despite Rapid ATP Decline in Embryos of the Annual Killifish Austrofundulus limnaeus. JOURNAL OF EXPERIMENTAL ZOOLOGY PART A-ECOLOGICAL GENETICS AND PHYSIOLOGY. 2012;317A(8):524-532.

6018. Schneider S, Hauf C, Schiltenwolf M Back care programs for health promotion - representative user profiles and correlates of participation in Germany. PREVENTIVE MEDICINE. 2005;40(2):227-238.

6019. Quintana P, Alberi S, Hakkoum D, Muller D Glutamate receptor changes associated with transient anoxia/hypoglycaemia in hippocampal slice cultures. EUROPEAN JOURNAL OF NEUROSCIENCE. 2006;23(4):975-983.

6020. Steinhilber Kylie, Ray Sukanya, Harkins Debra, Sienkiewicz Megan Father-daughter relationship dynamics \& daughters' body image, eating patterns, and empowerment: An exploratory study. WOMEN \& HEALTH. 2020;60(10):1083-1094.

6021. Speijer RP, Schmitz B, vanderZwaan GJ Benthic foraminiferal extinction and repopulation in response to latest Paleocene Tethyan anoxia. GEOLOGY. 1997;25(8):683-686.

6022. Kramer-Kostecka Eydie, Fulkerson Jayne, Sherwood Nancy, Barr-Anderson Daheia, Larson Nicole, Neumark-Sztainer Dianne What Brings Young Adults to the Yoga Mat? Cross-Sectional Associations Between Motivational Profiles and Physical and Psychological Health Among Participants in the Project EAT-IV Survey. JOURNAL OF INTEGRATIVE AND COMPLEMENTARY MEDICINE. 2022;():.

6023. DeJong Erica, Surette Michael, Bowdish Dawn The Gut Microbiota and Unhealthy Aging: Disentangling Cause from Consequence. CELL HOST \& MICROBE. 2020;28(2):180-189.

6024. Pawlowska Beata, Masiak Marek Analysis of demographic data and family relationships in women with bulimia. PSYCHIATRIA POLSKA. 2007;41(3):365-376.

6025. Rothschild-Yakar Lily, Waniel Ariela, Stein Daniel Mentalizing in Self vs. Parent Representations and Working Models of Parents as Risk and Protective Factors From Distress and Eating Disorders. JOURNAL OF NERVOUS AND MENTAL DISEASE. 2013;201(6):510-518.

6026. Gilsbach Susanne, Teresa Plana Maria, Castro-Fornieles Josefina, Gatta Michela, Karlsson Gunilla, Flamarique Itziar, Raynaud Jean-Philippe, Riva Anna, Solberg Anne-Line, Elburg Annemarie, Wentz Elisabet, Nacinovich Renata, Herpertz-Dahlmann Beate Increase in admission rates and symptom severity of childhood and adolescent anorexia nervosa in

Europe during the COVID-19 pandemic: data from specialized eating disorder units in different European countries. CHILD AND ADOLESCENT PSYCHIATRY AND MENTAL HEALTH. 2022;16(1):.

6027. Glasofer Deborah, Albano Anne, Simpson H., Steinglass Joanna Overcoming Fear of Eating: A Case Study of a Novel Use of Exposure and Response Prevention. PSYCHOTHERAPY. 2016;53(2):223-231.

6028. Haveman-Nies A, Groot LCPGM, Staveren WA Dietary quality, lifestyle factors and healthy ageing in Europe: the SENECA study. AGE AND AGEING. 2003;32(4):427-434.

6029. Manzanares Nuria, Monseny Rosa, Ortega Laura, Montalvo Ltziar, Franch Joan, Gutierrez-Zotes Alfonso, Reynolds Rebecca, Walker Brian, Vilella Elisabet, Labad Javier Unhealthy lifestyle in early psychoses: The role of life stress and the hypothalamic-pituitary-adrenal axis. PSYCHONEUROENDOCRINOLOGY. 2014;39():1-10.

6030. Bostic JQ, Muriel AC, Hack S, Weinstein S, Herzog D Anorexia nervosa in a 7-year-old girl. JOURNAL OF DEVELOPMENTAL AND BEHAVIORAL PEDIATRICS. 1997;18(5):331-333.

6031. Fernandez Gomez Elisabet, Angustias Sanchez-Ojeda Maria, Enrique Miron Carmen, Martin-Salvador Adelina Pre-post evaluation of a training activity on food and physical activity addressed to university students of the Melilla campus. SPORT TK-REVISTA EUROAMERICANA DE CIENCIAS DEL DEPORTE. 2019;8(2, 1):73-80.

6032. Trudel-Fitzgerald Claudia, Tworoger Shelley, Poole Elizabeth, Williams David, Kubzansky Laura Prospective Changes in Healthy Lifestyle Among Midlife Women When Psychological Symptoms Get in the Way. AMERICAN JOURNAL OF PREVENTIVE MEDICINE. 2016;51(3):327-335.

6033. Guimond Anne-Josee, Zevon Emily, Tucker-Seeley Reginald, Giovannucci Edward, Trudel-Fitzgerald Claudia, Kubzansky Laura Childhood socioeconomic status, healthy lifestyle, and colon cancer risk in a cohort of US women. PREVENTIVE MEDICINE. 2022;161():.

6034. Fassarella Marina, Blaak Ellen, Penders John, Nauta Arjen, Smidt Hauke, Zoetendal Erwin Gut microbiome stability and resilience: elucidating the response to perturbations in order to modulate gut health. GUT. 2021;70(3):595-605.

6035. Platt Jonathan, Colich Natalie, McLaughlin Katie, Gary Dahsan, Keyes Katherine Transdiagnostic psychiatric disorder risk associated with early age of menarche: A latent modeling approach. COMPREHENSIVE PSYCHIATRY. 2017;79(SI):70-79.

6036. Yager Zali, O'Dea Jennifer Prevention programs for body image and eating disorders on University campuses: a review of large, controlled interventions. HEALTH PROMOTION INTERNATIONAL. 2008;23(2):173-189.

6037. Kosti Rena, Tsiampalis Thomas, Kouvari Matina, Chrysohoou Christina, Georgousopoulou Ekavi, Skoumas John, Pitsavos Christos, Panagiotakos Demosthenes Dietary patterns and alcoholic beverage preference in relation to 10-year cardiovascular disease, hypertension, hypercholesterolemia and diabetes mellitus incidence in the ATTICA cohort study. OENO ONE. 2022;56(3):121-135.

6038. Samaiya Puneet, Narayan Gopeshwar, Kumar Ashok, Krishnamurthy Sairam 2,4 Dinitrophenol Attenuates Mitochondrial Dysfunction and Improves Neurobehavioral Outcomes Postanoxia in Neonatal Rats. NEUROTOXICITY RESEARCH. 2018;34(1):121-136.

6039. Al-Qawasmeh Rawan, Tayyem Reema Dietary and Lifestyle Risk Factors and Metabolic Syndrome: Literature Review. CURRENT RESEARCH IN NUTRITION AND FOOD SCIENCE. 2018;6(3):594-608.

6040. Zambon RA, Nandakumar M, Vakharia VN, Wu LP The Toll pathway is important for an antiviral response in Drosophila. PROCEEDINGS OF THE NATIONAL ACADEMY OF SCIENCES OF THE UNITED STATES OF AMERICA. 2005;102(20):7257-7262.

6041. Escolar-Llamazares Maria-Camino, Martinez-Martin Maria-Angeles, Medina-Gomez Maria-Begona, Gonzalez-Alonso Maria-Yolanda, Mercado-Val Elvira, Lara-Ortega Fernando Sociodemographic Variables and Body Mass Index Associated with the Risk of Eating Disorders in Spanish University Students. EUROPEAN JOURNAL OF INVESTIGATION IN HEALTH PSYCHOLOGY AND EDUCATION. 2023;13(3):595-612.

6042. Kletkiewicz Hanna, Nowakowska Anna, Siejka Agnieszka, Mila-Kierzenkowska Celestyna, Wozniak Alina, Caputa Michal, Rogalska Justyna Deferoxamine prevents cerebral glutathione and vitamin E depletions in asphyxiated neonatal rats: role of body temperature. INTERNATIONAL JOURNAL OF HYPERTHERMIA. 2016;32(2):211-220.

6043. Kazaz Ismet, Angin Ender, Kabaran Seray, Iyiguen Gozde, Kirmizigil Berkiye, Malkoc Mehtap Evaluation of the physical activity level, nutrition quality, and depression in patients with metabolic syndrome: Comparative study. MEDICINE. 2018;97(18):.

6044. Bjerregaard Peter, Larsen Christina Social determinants of dietary patterns, food basket costs and expenditure on alcohol and tobacco amongst Greenland Inuit. PUBLIC HEALTH NUTRITION. 2021;24(15):4975-4984.

6045. Turner Monique, Ford Lindsay, Somerville Victoria, Javellana Donna, Day Kelsey, Lapinski Maria The Use of Stigmatizing Messaging in Anti-Obesity Communications Campaigns: Quantification of Obesity Stigmatization. COMMUNICATION REPORTS. 2020;33(3):107-120.

6046. Adorni Roberta, Lonati Elena, Zanatta Francesco, Belingheri Michael, Rossetti Massimiliano, Colleoni Matteo, Riva Michele, Palestini Paola, Steca Patrizia Lifestyle Profiles and Their Sociodemographic Correlate in an Academic Community Sample. INTERNATIONAL JOURNAL OF ENVIRONMENTAL RESEARCH AND PUBLIC HEALTH. 2023;20(1):.

6047. Eisenberg Marla, Larson Nicole, Gollust Sarah, Neumark-Sztainer Dianne Snacking on Television: A Content Analysis of Adolescents' Favorite Shows. PREVENTING CHRONIC DISEASE. 2016;13():.

6048. Safiri Saeid, Noori Maryam, Nejadghaderi Seyed, Mousavi Seyed, Karamzad Nahid, Sullman Mark, Pirotta Stephanie, Collins Gary, Abdollahi Morteza, Kolahi Ali-Asghar Comparison of the burden of anorexia nervosa in the Middle East and North Africa region between 1990 and 2019. JOURNAL OF EATING DISORDERS. 2022;10(1):.

6049. Teder Marie, Morelius Evalotte, Nordwall Maria, Bolme Per, Ekberg Joakim, Wilhelm Elisabeth, Timpka Toomas Family-Based Behavioural Intervention Program for Obese Children: An Observational Study of Child and Parent Lifestyle Interpretations. PLOS ONE. 2013;8(8):.

6050. Lapointe Annie, Laramee Catherine, Belanger-Gravel Ariane, Buckeridge David, Desroches Sophie, Garriguet Didier, Gauvin Lise, Lemieux Simone, Plante Celine, Lamarche Benoit NutriQuebec: a unique web-based prospective cohort study to monitor the population's eating and other lifestyle behaviours in the province of Quebec. BMJ OPEN. 2020;10(10):.

6051. Springett RJ, Wylezinska M, Cady EB, Hollis V, Cope M, Delpy DT The oxygen dependency of cerebral oxidative metabolism in the newborn piglet studied with P-31 NMRS and NIRS. . 2003;530():555-563.

6052. Makri Stamatina, Rey Fabian, Gobet Erika, Gilli Adrian, Tinner Willy, Grosjean Martin Early human impact in a 15,000-year high-resolution hyperspectral imaging record of paleoproduction and anoxia from a varved lake in Switzerland. QUATERNARY SCIENCE REVIEWS. 2020;239():.

6053. Zhang Melvyn, Ho Roger, Cassin Stephanie, Hawa Raed, Sockalingam Sanjeev Online and smartphone based cognitive behavioral therapy for bariatric surgery patients: Initial pilot study. TECHNOLOGY AND HEALTH CARE. 2015;23(6):737-744.

6054. Bartlett Rick, Elrick Maya, Wheeley James, Polyak Victor, Desrochers Andre, Asmerom Yemane Abrupt global-ocean anoxia during the Late Ordovician-early Silurian detected using uranium isotopes of marine carbonates. PROCEEDINGS OF THE NATIONAL ACADEMY OF SCIENCES OF THE UNITED STATES OF AMERICA. 2018;115(23):5896-5901.

6055. Ortiz Cristina, Lopez-Cuadrado Teresa, Rodriguez-Blazquez Carmen, Simon Lorena, Perez-Vicente Raquel, Merlo Juan, Galan Inaki Physical and social environmental factors related to co-occurrence of unhealthy lifestyle behaviors. HEALTH \& PLACE. 2022;75():.

6056. Ahmed Haitham, Blaha Michael, Nasir Khurram, Jones Steven, Rivera Juan, Agatston Arthur, Blankstein Ron, Wong Nathan, Lakoski Susan, Budoff Matthew, Burke Gregory, Sibley Christopher, Ouyang Pamela, Blumenthal Roger Low-Risk Lifestyle, Coronary Calcium, Cardiovascular Events, and Mortality: Results From MESA. AMERICAN JOURNAL OF EPIDEMIOLOGY. 2013;178(1):12-21.

6057. Imaizumi T, Kocsis JD, Waxman SG The role of voltage-gated Ca2+ channels in anoxic injury of spinal cord white matter. BRAIN RESEARCH. 1999;817(1-2):84-92.

6058. Ceballos Daniel, Hernandez-Camba A., Ramos Laura Diet and microbiome in the beginning of the sequence of gut inflammation. WORLD JOURNAL OF CLINICAL CASES. 2021;9(36):11122-11147.

6059. Arnulf Isabelle, Rico Thomas, Mignot Emmanuel Diagnosis, disease course, and management of patients with Kleine-Levin syndrome. LANCET NEUROLOGY.

2012;11(10):918-928.

6060. Olofsson Malin, Oddli Hanne, Vrabel Kari, Hoffart Asle << In solitude is safeness >>: a patient perspective on eating disorders in the context of multiple childhood trauma. NORDIC PSYCHOLOGY. 2021;73(1):29-42.

6061. Justamente Ilze, Raudeniece Jelena, Ozolina-Moll Liga, Guadalupe-Grau Amelia, Reihmane Dace Comparative Analysis of the Effects of Daily Eating Habits and Physical Activity on Anthropometric Parameters in Elementary School Children in Latvia: Pach Study. NUTRIENTS. 2020;12(12):.

6062. Arthur MA, Dean WE Organic-matter production and preservation and evolution of anoxia in the Holocene Black Sea. PALEOCEANOGRAPHY. 1998;13(4):395-411.

6063. Mason Susan, Emery Rebecca, Friedman Jessica, Hanson Stephanie, Johnson Sydney, Neumark-Sztainer Dianne Associations of abuse and neglect with young adult health in a population-based longitudinal cohort: Findings from Project EAT. PREVENTIVE MEDICINE. 2022;164():.

6064. Ott CH, Haertlein C Social norms marketing: a prevention strategy to decrease high-risk drinking among college students. NURSING CLINICS OF NORTH AMERICA. 2002;37(2):351+.

6065. Jew KN, Moore RL Exercise training alters an anoxia-induced, glibenclamide-sensitive current in rat ventricular cardiocytes. JOURNAL OF APPLIED PHYSIOLOGY. 2002;92(4):1473-1479.

6066. Hijova Emilia, Geckova Andrea, Babinska Ingrid, Team HepaMeta DO EATING HABITS OF THE POPULATION LIVING IN ROMA SETTLEMENTS DIFFER FROM THOSE OF THE MAJORITY POPULATION IN SLOVAKIA?. CENTRAL EUROPEAN JOURNAL OF PUBLIC HEALTH. 2014;22(S):S65-S68.

6067. Faurholt-Jepsen Maria, Bauer Michael, Kessing Lars Smartphone-based objective monitoring in bipolar disorder: status and considerations. INTERNATIONAL JOURNAL OF BIPOLAR DISORDERS. 2018;6():.

6068. Bo Simona, Zoccali Rossana, Ponzo Valentina, Soldati Laura, De Carli Luca, Benso Andrea, Fea Elisabetta, Rainoldi Alberto, Durazzo Marilena, Fassino Secondo, Abbate-Daga Giovanni University courses, eating problems and muscle dysmorphia: are there any associations?. JOURNAL OF TRANSLATIONAL MEDICINE. 2014;12():.

6069. Laraia Barbara, Epel Elissa, Siega-Riz Anna Food insecurity with past experience of restrained eating is a recipe for increased gestational weight gain. APPETITE. 2013;65():178-184.

6070. Mas Natalia, Fuste Adela, Garcia-Grau Eugeni, Bados Arturo Coping styles and vulnerability to eating disorders in adolescent girls, by age. TERAPIA PSICOLOGICA. 2015;33(3):161-168.

6071. Hong Jisoo, Bales Diane, Wallinga Charlotte Using Family Backpacks as a Tool to Involve Families in Teaching Young Children About Healthy Eating. EARLY CHILDHOOD EDUCATION JOURNAL. 2018;46(2):209-221.

6072. Dukoff David, Hogg David, Hawrysh Peter, Buck Leslie Scavenging ROS dramatically increase NMDA receptor whole-cell currents in painted turtle cortical neurons. JOURNAL OF EXPERIMENTAL BIOLOGY. 2014;217(18):3346-3355.

6073. Wade Tracey, Wilksch Simon Internet eating disorder prevention. CURRENT OPINION IN PSYCHIATRY. 2018;31(6):456-461.

6074. Svendsen Vegard, Lokkerbol Joran, Danner Unna, Jansingh Alberte, Evers Silvia, Wijnen Ben Design and testing of a health economic Markov model for treatment of anorexia nervosa. EXPERT REVIEW OF PHARMACOECONOMICS \& OUTCOMES RESEARCH. 2022;22(8):1243-1251.

6075. Karczewska-Kupczewska Monika, Straczkowski Marek, Adamska Agnieszka, Nikolajuk Agnieszka, Otziomek Elzbieta, Gorska Maria, Kowalska Irina Insulin sensitivity, metabolic flexibility, and serum adiponectin concentration in women with anorexia nervosa. METABOLISM-CLINICAL AND EXPERIMENTAL. 2010;59(4):473-477.

6076. Oostindjer Marije, Amdam Gro, Egelandsdal Bjorg Getting Norway to eat healthier: What are the opportunities?. SCANDINAVIAN JOURNAL OF PUBLIC HEALTH. 2015;43(1):66-75.

6077. Swenne Ingemar Weight and growth requirements for menarche in teenage girls with eating disorders, weight loss and primary amenorrhea. HORMONE RESEARCH. 2008;69(3):146-151.

6078. Ventura Alison, Garst Barry Residential summer camp: a new venue for nutrition education and physical activity promotion. INTERNATIONAL JOURNAL OF BEHAVIORAL NUTRITION AND PHYSICAL ACTIVITY. 2013;10():.

6079. Jahrami Haitham, Saif Zahraa, Faris Mo'ez, Levine Michael The relationship between risk of eating disorders, age, gender and body mass index in medical students: a meta-regression. EATING AND WEIGHT DISORDERS-STUDIES ON ANOREXIA BULIMIA AND OBESITY. 2019;24(2):169-177.

6080. Johnson F., Pratt M., Wardle J. Dietary restraint and self-regulation in eating behavior. INTERNATIONAL JOURNAL OF OBESITY. 2012;36(5, SI):665-674.

6081. Brooks Cristy, Helson Catherine, McCormack Madalyn, Baur Louise, Gill Timothy, Green Julie, Billah Baki, Cronin Paula, Johar Anoop, Plaskett Jennifer, Nolan Michelle, Latanik Monika, Renzaho Andre Protocol for a randomised controlled trial of a family strengthening program to prevent unhealthy weight gain among 5 to 11-year-old children from at-risk families: the Strong Families Trial. BMC PUBLIC HEALTH. 2022;22(1):.

6082. Bastons-Compta A., Astals M., Andreu V., Navarro E., Garcia-Algar O. Postnatal nutritional treatment of neurocognitive deficits in fetal alcohol spectrum disorder. BIOCHEMISTRY AND CELL BIOLOGY. 2018;96(2, SI):213-221.

6083. Riedel B., Pados T., Pretterebner K., Schiemer L., Steckbauer A., Haselmair A., Zuschin M., Stachowitsch M. Effect of hypoxia and anoxia on invertebrate behaviour: ecological perspectives from species to community level. BIOGEOSCIENCES. 2014;11(6):1491-1518.

6084. Gibbons Christopher, Goebel-Fabbri Ann Microvascular Complications Associated With Rapid Improvements in Glycemic Control in Diabetes. CURRENT DIABETES REPORTS. 2017;17(7):.

6085. JONES DP MITOCHONDRIAL DYSFUNCTION DURING ANOXIA AND ACUTE CELL INJURY. BIOCHIMICA ET BIOPHYSICA ACTA-MOLECULAR BASIS OF DISEASE. 1995;1271(1):29-33.

6086. Demou Evangelia, MacLean Alice, Cheripelli Lismy, Hunt Kate, Gray Cindy Group-based healthy lifestyle workplace interventions for shift workers: a systematic review. SCANDINAVIAN JOURNAL OF WORK ENVIRONMENT \& HEALTH. 2018;44(6):568-584.

6087. Khambadkone Seva, Cordner Zachary, Dickerson Faith, Severance Emily, Prandovszky Emese, Pletnikov Mikhail, Xiao Jianchun, Li Ye, Boersma Gretha, Talbot C., Campbell Wayne, Wright Christian, Siple C., Moran Timothy, Tamashiro Kellie, Yolken Robert Nitrated meat products are associated with mania in humans and altered behavior and brain gene expression in rats. MOLECULAR PSYCHIATRY. 2020;25(3):560-571.

6088. Iyer Shruti, Shriraam Vanishree Prevalence of Eating Disorders and Its Associated Risk Factors in Students of a Medical College Hospital in South India. CUREUS JOURNAL OF MEDICAL SCIENCE. 2021;13(1):.

6089. Henry C, DemotesMainard J, Bourgeois M Kleine-Levin syndrome in adolescent's behavior disorders spectrum. ANNALES MEDICO-PSYCHOLOGIQUES. 1997;155(8):526-529.

6090. Kukihara Hiroko, Yamawaki Niwako, Uchiyama Kumi, Arai Shoichi, Horikawa Etsuo Trauma, depression, and resilience of earthquake/tsunami/nuclear disaster survivors of Hirono, Fukushima, Japan. PSYCHIATRY AND CLINICAL NEUROSCIENCES. 2014;68(7):524-533.

6091. Finzer P, Haffner J, Muller-Kuppers M Outcome and prognostic factors of anorexia nervosa: Follow-up study of 41 patients. PRAXIS DER KINDERPSYCHOLOGIE UND KINDERPSYCHIATRIE. 1998;47(5):302-313.

6092. Bedard Annabelle, Garcia-Aymerich Judith, Sanchez Margaux, Le Moual Nicole, Clavel-Chapelon Francoise, Boutron-Ruault Marie-Christine, Maccario Jean, Varraso Raphaelle Confirmatory Factor Analysis Compared with Principal Component Analysis to Derive Dietary Patterns: A Longitudinal Study in Adult Women. JOURNAL OF NUTRITION. 2015;145(7):1559-1568.

6093. Cruwys Tegan, Haslam S., Fox Nicole, McMahon Hayley ``That's not what we do{''}: Evidence that normative change is a mechanism of action in group interventions. BEHAVIOUR RESEARCH AND THERAPY. 2015;65():11-17.

6094. Peretti S., Mariano M., Mazzocchetti C., Mazza M., Pino M., Di Pianella A., Valenti M. Diet: the keystone of autism spectrum disorder?. NUTRITIONAL NEUROSCIENCE.

2019;22(12):825-839.

6095. Perez Marisol, Becker Carolyn, Ramirez Ana Transportability of an empirically supported dissonance-based prevention program for eating disorders. BODY IMAGE. 2010;7(3):179-186.

6096. Angelova Rosa, Utermohlen Virginia Culture-specific influences on body image and eating distress in a sample of urban Bulgarian women: The roles of faith and traditional fasting. EATING BEHAVIORS. 2013;14(3):386-389.

6097. Nagata T, Yamada H, Iketani T, Kiriike N Relationship between plasma concentrations of cytokines, ratio of CD4 and CD8, lymphocyte proliferative responses, and depressive and anxiety state in bulimia nervosa. JOURNAL OF PSYCHOSOMATIC RESEARCH. 2006;60(1):99-103.

6098. Laghi Fiorenzo, Pompili Sara, Bianchi Dora, Lonigro Antonia, Baiocco Roberto Exploring the association between psychological distress and drunkorexia behaviors in non-clinical adolescents: the moderating role of emotional dysregulation. EATING AND WEIGHT DISORDERS-STUDIES ON ANOREXIA BULIMIA AND OBESITY. 2021;26(3):797-806.

6099. Watson Hunna, Joyce Tara, French Elizabeth, Willan Vivienne, Kane Robert, Tanner-Smith Emily, McCormack Julie, Dawkins Hayley, Hoiles Kimberley, Egan Sarah Prevention of Eating Disorders: A Systematic Review of Randomized, Controlled Trials. INTERNATIONAL JOURNAL OF EATING DISORDERS. 2016;49(9):833-862.

6100. Flatt Rachael, Taylor Craig Adapting a Technology-Based Eating Disorder Model for Athletes. JOURNAL OF CLINICAL SPORT PSYCHOLOGY. 2018;12(4, SI):699-717.

6101. LEITENBERG H COGNITIVE-BEHAVIORAL TREATMENT OF BULIMIA-NERVOSA. BEHAVIOUR CHANGE. 1995;12(2):81-97.

6102. Eickman Laura, Betts Jessica, Pollack Lauren, Bozsik Frances, Beauchamp Marshall, Lundgren Jennifer Randomized controlled trial of REbeL: A peer education program to promote positive body image, healthy eating behavior, and empowerment in teens. EATING DISORDERS. 2018;26(2):127-142.

6103. Mora Fernando, Alvarez-Mon Miguel, Fernandez-Rojo Sonia, Ortega Miguel, Felix-Alcantara Miriam, Morales-Gil Isabel, Rodriguez-Quiroga Alberto, Alvarez-Mon Melchor, Quintero Javier Psychosocial Factors in Adolescence and Risk of Development of Eating Disorders. NUTRIENTS. 2022;14(7):.

6104. Gray Heewon, Pang Tiantian, Agazzi Heather, Shaffer-Hudkins Emily, Kim Eunsook, Miltenberger Raymond, Waters Karah, Jimenez Claudia, Harris Monise, Stern Marilyn A nutrition education intervention to improve eating behaviors of children with autism spectrum disorder: Study protocol for a pilot randomized controlled trial. CONTEMPORARY CLINICAL TRIALS. 2022;119():.

6105. Testa Rylan, Rider G., Haug Nancy, Balsam Kimberly Gender Confirming Medical Interventions and Eating Disorder Symptoms Among Transgender Individuals. HEALTH PSYCHOLOGY. 2017;36(10):927-936.

6106. Bertrand Valerie, Dhenin Caroline, Dechelotte Pierre, Faerber Mathieu Delayed avoidant restrictive food intake disorder diagnosis leading to Ogilvie's syndrome in an adolescent. EATING AND WEIGHT DISORDERS-STUDIES ON ANOREXIA BULIMIA AND OBESITY. 2022;27(5):1913-1918.

6107. Fuks Betty, Porto Campos Tatiana ANOREXIA: THE URGENCY OF A NEW CLINICAL PRACTICE. TEMPO PSICANALITICO. 2010;42(1):39-59.

6108. Chen Jian-feng, Fan Jian, Tian Xiao-wu, Tang Tian-si PROTECTIVE EFFECTS OF TWO CONSTITUENTS OF CHINESE HERBS ON SPINAL MOTOR NEURONS FROM EMBRYONIC RATS WITH HYPOXIA INJURY. AFRICAN JOURNAL OF TRADITIONAL COMPLEMENTARY AND ALTERNATIVE MEDICINES. 2012;9(2):234-241.

6109. Keel PK, Klump KL Are eating disorders culture-bound syndromes? Implications for conceptualizing their etiology. PSYCHOLOGICAL BULLETIN. 2003;129(5):747-769.

6110. Agostino Holly, Burstein Brett, Moubayed Dina, Taddeo Danielle, Grady Rosheen, Vyver Ellie, Dimitropoulos Gina, Dominic Anna, Coelho Jennifer Trends in the Incidence of New-Onset Anorexia Nervosa and Atypical Anorexia Nervosa Among Youth During the COVID-19 Pandemic in Canada. JAMA NETWORK OPEN. 2021;4(12):.

6111. Reader Shane, Lopez Richard, Denny Bryan Cognitive reappraisal of low-calorie food predicts real-world craving and consumption of high- and low-calorie foods in daily life.

APPETITE. 2018;131():44-52.

6112. Chang Mei-Wei, Nitzke Susan, Buist Diana, Cain Deborah, Horning Stefanie, Eghtedary Kobra I Am Pregnant and Want to Do Better But I Can't: Focus Groups with Low-Income Overweight and Obese Pregnant Women. MATERNAL AND CHILD HEALTH JOURNAL. 2015;19(5):1060-1070.

6113. Tripodi Alberto, Severi Sabrina, Midili Simona, Corradini Barbara ``Community projects{''} in Modena (Italy): promote regular physical activity and healthy nutrition habits since childhood. INTERNATIONAL JOURNAL OF PEDIATRIC OBESITY. 2011;6(2):54-56.

6114. Kletkiewicz Hanna, Nowakowska Anna, Siejka Agnieszka, Mila-Kierzenkowska Celestyna, Wozniak Alina, Caputa Michal, Rogalska Justyna Deferoxamine improves antioxidative protection in the brain of neonatal rats: The role of anoxia and body temperature. NEUROSCIENCE LETTERS. 2016;628():116-122.

6115. Gutuskey Lila, McCaughtry Nate, Shen Bo, Centeio Erin, Garn Alex The role and impact of student leadership on participants in a healthy eating and physical activity programme. HEALTH EDUCATION JOURNAL. 2016;75(1):27-37.

6116. Buck Leslie, Hogg D., Rodgers-Garlick C., Pamenter M. Oxygen Sensitive Synaptic Neurotransmission in Anoxia-Tolerant Turtle Cerebrocortex. . 2012;758():71-79.

6117. SAMUTA T, BECKER GL, POHORECKI R, ARMSTRONG K, LANDERS DF EFFECTS OF ISOFLURANE DOSE, DURATION OF ANOXIA, AND REOXYGENATION ON ISOFLURANES PRESERVATION OF ENERGY-BALANCE IN ANOXIC ISOLATED HEPATOCYTES. ANESTHESIA AND ANALGESIA. 1993;77(1):38-43.

6118. Kumar Bharat THE MIND-BODY CONNECTION: AN INTEGRATED APPROACH TO THE DIAGNOSIS OF COLONIC TRICHOBEZOAR. INTERNATIONAL JOURNAL OF PSYCHIATRY IN MEDICINE. 2011;41(3):263-270.

6119. Trude Angela, Kharmats Anna, Jock Brittany, Liu Debra, Lee Katherine, Martins Paula, Pardilla Marla, Swartz Jaqueline, Gittelsohn Joel Patterns of Food Consumption are Associated with Obesity, Self-Reported Diabetes and Cardiovascular Disease in Five American Indian Communities. ECOLOGY OF FOOD AND NUTRITION. 2015;54(5):437-454.

6120. Shawon Md, Jahan Esrat, Rouf Rashawan, Hossain Fariha Psychological distress and unhealthy dietary behaviours among adolescents aged 12-15 years in nine South-East Asian countries: a secondary analysis of the Global School-Based Health Survey data. BRITISH JOURNAL OF NUTRITION. 2023;129(7):1242-1251.

6121. Lando HA, Thai DT, Murray DM, Robinson LA, Jeffery RW, Sherwood NE, Hennrikus DJ Age of initiation, smoking patterns, and risk in a population of working adults. PREVENTIVE MEDICINE. 1999;29(6, 1):590-598.

6122. Solmi Marco, Campeol Mara, Gentili Federica, Favaro Angela, Cremonese Carla Clinical presentation and need for treatment of a cohort of subjects accessing to a mental illness prevention service. RESEARCH IN PSYCHOTHERAPY-PSYCHOPATHOLOGY PROCESS AND OUTCOME. 2020;23(1):24-32.

6123. Gumz Antje, Weigel Angelika, Daubmann Anne, Wegscheider Karl, Romer Georg, Loewe Bernd Efficacy of a prevention program for eating disorders in schools: a cluster-randomized controlled trial. BMC PSYCHIATRY. 2017;17():.

6124. Otis CL, Drinkwater B, Johnson M, Loucks A, Wilmore J The female athlete triad. MEDICINE \& SCIENCE IN SPORTS \& EXERCISE. 1997;29(5):R1-R9.

6125. Bergstrom Ingrid, Crisby Milita, Engstrom Anne-May, Holcke Mats, Fored Monika, Kruse Pia, Sandberg Ann-Marie Women with anorexia nervosa should not be treated with estrogen or birth control pills in a bone-sparing effect. ACTA OBSTETRICIA ET GYNECOLOGICA SCANDINAVICA. 2013;92(8):877-880.

6126. Rohde Paul, Shaw Heather, Butryn Meghan, Stice Eric Assessing program sustainability in an eating disorder prevention effectiveness trial delivered by college clinicians. BEHAVIOUR RESEARCH AND THERAPY. 2015;72():1-8.

6127. Fitzsimmons-Craft Ellen, Laboe Agatha, McGinnis Claire, Firebaugh Marie-Laure, Shah Jillian, Wallendorf Michael, Jacobi Corinna, Bardone-Cone Anna, Pike Kathleen, Taylor C., Wilfley Denise A pilot randomized controlled trial of a cognitive-behavioral therapy-guided self-help mobile app for the post-acute treatment of anorexia nervosa: A registered report. INTERNATIONAL JOURNAL OF EATING DISORDERS. 2023;56(3):654-661.

6128. Pachydakis Anastasios, Koutroumanis Pelopidas, Geyushi Bohaiza, Hanna Leila Primary hyperparathyroidism in pregnancy presenting as intractable hyperemesis

complicating psychogenic anorexia - A case report. JOURNAL OF REPRODUCTIVE MEDICINE. 2008;53(9):714-716.

6129. Ookawara R, Satoh S, Yoshioka T, Ishizawa K Expression of alpha-expansin and xyloglucan endotransglucosylase/hydrolase genes associated with shoot elongation enhanced by anoxia, ethylene and carbon dioxide in arrowhead (Sagittaria pygmaea Miq.) tubers. ANNALS OF BOTANY. 2005;96(4):693-702.

6130. Larson Nicole, Chen Ying, Wall Melanie, Winkler Megan, Goldschmit Andrea, Neumark-Sztainer Dianne Personal, behavioral, and environmental predictors of healthy weight maintenance during the transition to adulthood. PREVENTIVE MEDICINE. 2018;113():80-90.

6131. Lemamsha Hamdi, Randhawa Gurch, Papadopoulos Chris Investigating the Association between Unhealthy Dietary Habits and Obesity among Libyan Adults. INTERNATIONAL JOURNAL OF ENVIRONMENTAL RESEARCH AND PUBLIC HEALTH. 2022;19(3):.

6132. Saugo Elena, Lasalvia Antonio, Bonetto Chiara, Cristofalo Doriana, Poli Sara, Bissoli Sarah, Bertani Mariaelena, Lazzarotto Lorenza, Gardellin Francesco, Ceccato Enrico, Pavanati Michele, Tosato Sarah, Ruggeri Mirella, Grp GET Dietary habits and physical activity in first-episode psychosis patients treated in community services. Effect on early anthropometric and cardio-metabolic alterations. SCHIZOPHRENIA RESEARCH. 2020;216():374-381.

6133. Drabkin Anne, Rothman Micol, Wassenaar Elizabeth, Mascolo Margherita, Mehler Philip Assessment and clinical management of bone disease in adults with eating disorders: a review. JOURNAL OF EATING DISORDERS. 2017;5():.

6134. Nichols DL, Bonnick SL, Sanborn CF Bone health and osteoporosis. CLINICS IN SPORTS MEDICINE. 2000;19(2):233+.

6135. Cave AC, Adrian S, Apstein CS, Silverman HS A model of anoxic preconditioning in the isolated rat cardiac myocyte. Importance of adenosine and insulin. BASIC RESEARCH IN CARDIOLOGY. 1996;91(3):210-218.

6136. Halmi Katherine Perplexities and provocations of eating disorders. JOURNAL OF CHILD PSYCHOLOGY AND PSYCHIATRY. 2009;50(1-2):163-169.

6137. Hartmann A., Hilbert A. Child and adolescent obesity, psychosocial consequences and treatment strategies. BUNDESGESUNDHEITSBLATT-GESUNDHEITSFORSCHUNG-GESUNDHEITSSCHUTZ. 2013;56(4):532-538.

6138. Gu Yuchen, Wu Jianping The potential of antioxidative and anti-inflammatory peptides in reducing the risk of cardiovascular diseases. CURRENT OPINION IN FOOD SCIENCE. 2016;8():25-32.

6139. Bondue Rebecca, Bilgin Ayten, Warschburger Petra Justice sensitivity and rejection sensitivity as predictors and outcomes of eating disorder pathology: A 5-year longitudinal study. INTERNATIONAL JOURNAL OF EATING DISORDERS. 2020;53(6):926-936.

6140. Kilic Ozgur, Johnson Urban, Kerkhoffs Gino, Rosier Philippe, Gouttebarge Vincent Exposure to physical and psychosocial stressors in relation to symptoms of common mental disorders among European professional football referees: a prospective cohort study. BMJ OPEN SPORT \& EXERCISE MEDICINE. 2018;4(1):.

6141. Thompson AM, Chad KE The relationship of pubertal status to body image, social physique anxiety, preoccupation with weight and nutritional status in young females. CANADIAN JOURNAL OF PUBLIC HEALTH-REVUE CANADIENNE DE SANTE PUBLIQUE. 2000;91(3):207-211.

6142. WILCOX DT, KARAMANOUKIAN HL, GLICK PL TOOTHBRUSH INGESTION BY BULIMICS MAY REQUIRE LAPAROTOMY. JOURNAL OF PEDIATRIC SURGERY. 1994;29(12):1596.

6143. Dauleh Hajar, Soliman Ali, Haris Basma, Khalifa Amal, Al Khori Noor, Hussain Khalid Case Report: Hepatic Adenomatosis in a Patient With Prader-Willi Syndrome. FRONTIERS IN ENDOCRINOLOGY. 2022;13():.

6144. Koch Susanne, Larsen Janne, Mouridsen Svend, Bentz Mette, Petersen Liselotte, Bulik Cynthia, Mortensen Preben, Plessen Kerstin Autism spectrum disorder in individuals with anorexia nervosa and in their first- and second-degree relatives: Danish nationwide register-based cohort-study. BRITISH JOURNAL OF PSYCHIATRY. 2015;206(5):401-407.

6145. Schluter U, Crawford RMM Metabolic adaptation to prolonged anoxia in leaves of American cranberry (Vaccinium macrocarpon). PHYSIOLOGIA PLANTARUM. 2003;117(4):492-499.

6146. Amendola Simone Burden of mental health and substance use disorders among Italian young people aged 10-24 years: results from the Global Burden of Disease 2019 Study. SOCIAL PSYCHIATRY AND PSYCHIATRIC EPIDEMIOLOGY. 2022;57(4):683-694.

6147. Romanovskii DY, Tyul'kova EI, Samoilov MO Preconditioning hypobaric hypoxia prevents anoxia-induced inhibition of generation of focal potentials in slices of olfactory cortex from rat brain. BULLETIN OF EXPERIMENTAL BIOLOGY AND MEDICINE. 2001;132(6):1154-1156.

6148. Borges Caroline, Francescato Roberta, Hoefel Ana Factors associated with the presence of symptoms of anxiety and depression in academics of different courses of a university center in southern country. RBONE-REVISTA BRASILEIRA DE OBESIDADE NUTRICAO E EMAGRECIMENTO. 2020;14(84):17-28.

6149. Chamay C, Michaud PA The medical follow-up of adolescents with an eating disorder. MEDECINE ET HYGIENE. 2000;58(2327):2573-2576.

6150. Dinkler Lisa, Taylor Mark, Rastam Maria, Hadjikhani Nouchine, Bulik Cynthia, Lichtenstein Paul, Gillberg Christopher, Lundstrom Sebastian Anorexia nervosa and autism: a prospective twin cohort study. JOURNAL OF CHILD PSYCHOLOGY AND PSYCHIATRY. 2021;62(3):316-326.

6151. Sasaki Yoshiaki, Akaba Yuichi, Kajino Hiroki Refeeding syndrome in a 12-year-old girl with distal renal tubular acidosis. CLINICAL CASE REPORTS. 2020;8(12):2682-2684.

6152. Koukou Melina, Javed Fawad, Michelogiannakis Dimitrios Is There an Association Between Fixed Orthodontic Treatment and Initiation of Eating Disorders? A Review of Currently Available Evidence. FRONTIERS IN ORAL HEALTH. 2021;2():.

6153. White Ralph, Hackman Robert, Kugelmass Joel The dogmas of nutrition and cancer: time for a second (and maybe third) look. . 2010;1190():118-125.

6154. Chowdhury U, Lask B Clinical implications of brain imaging in eating disorders. PSYCHIATRIC CLINICS OF NORTH AMERICA. 2001;24(2):227+.

6155. Segu Helena, Jalsevac Florijan, Pinent Montserrat, Ardevol Anna, Terra Ximena, Teresa Blay Maria Intestinal Morphometric Changes Induced by a Western-Style Diet in Wistar Rats and GSPE Counter-Regulatory Effect. NUTRIENTS. 2022;14(13):.

6156. McHugh S., Todkari N., Moloney T., Leahy A. Stercoral perforation in a 17-year old. IRISH JOURNAL OF MEDICAL SCIENCE. 2011;180(2):581-582.

6157. BRADLEY SJ, HOOD J PSYCHIATRICALLY REFERRED ADOLESCENTS WITH PANIC ATTACKS - PRESENTING SYMPTOMS, STRESSORS, AND COMORBIDITY. JOURNAL OF THE AMERICAN ACADEMY OF CHILD AND ADOLESCENT PSYCHIATRY. 1993;32(4):826-829.

6158. Anwar Firoz, Asar Turky, Al-Abassi Fahad, Kumar Vikas, Alhayyani Sultan Natural sea salt in diet ameliorates better protection compared to table salt in the doxorubicin-induced cardiac remodeling. JOURNAL OF TAIBAH UNIVERSITY FOR SCIENCE. 2022;16(1):1213-1224.

6159. Pantano M, Dalle Grave R, Oliosi M, Bartocci C, Todisco P, Marchi S Family backgrounds and eating disorders. PSYCHOPATHOLOGY. 1997;30(3):163-169.

6160. Liu R, Pei JJ, Wang XC, Zhou XW, Tian Q, Winblad B, Wang JZ Acute anoxia induces tau dephosphorylation in rat brain slices and its possible underlying mechanisms. JOURNAL OF NEUROCHEMISTRY. 2005;94(5):1225-1234.

6161. Lushnikova Iryna, Orlovsky Maxim, Dosenko Victor, Maistrenko Anastasiia, Skibo Galina Brief anoxia preconditioning and HIF prolyl-hydroxylase inhibition enhances neuronal resistance in organotypic hippocampal slices on model of ischemic damage. BRAIN RESEARCH. 2011;1386():175-183.

6162. Kapfhammer HP, Rothenhausler HB, Dietrich E, Dobmeier P, Mayer C Artificial disorders - between deception and self-harm. Psychiatric consultation in a university clinic. NERVENARZT. 1998;69(5):401-409.

6163. Riquin Elise, Lamas Claire, Nicolas Isabelle, Lebigre Corinne, Curt Florence, Cohen Henri, Legendre Guillaume, Corcos Maurice, Godart Nathalie A key for perinatal depression

early diagnosis: The body dissatisfaction. JOURNAL OF AFFECTIVE DISORDERS. 2019;245():340-347.

6164. Sun Chao-Yue, Zheng Zuo-Liang, Chen Cun-Wu, Lu Bao-Wei, Liu Dong Targeting Gut Microbiota With Natural Polysaccharides: Effective Interventions Against High-Fat Diet-Induced Metabolic Diseases. FRONTIERS IN MICROBIOLOGY. 2022;13():.

6165. Morgan J, Lacey JH Anorexia nervosa and steroid withdrawal. INTERNATIONAL JOURNAL OF EATING DISORDERS. 1996;19(2):213-215.

6166. Garcia-Grau E, Fuste A, Miro A, Saldana C, Bados A Coping style and disturbed eating attitudes in adolescent girls. INTERNATIONAL JOURNAL OF EATING DISORDERS. 2002;32(1):116-120.

6167. Bundgaard Amanda, James Andrew, Gruszczyk Anja, Martin Jack, Murphy Michael, Fago Angela Metabolic adaptations during extreme anoxia in the turtle heart and their implications for ischemia-reperfusion injury. SCIENTIFIC REPORTS. 2019;9():.

6168. Schobben Martin, Foster William, Sleveland Arve, Zuchuat Valentin, Svensen Henrik, Planke Sverre, Bond David, Marcelis Fons, Newton Robert, Wignall Paul, Poulton Simon A nutrient control on marine anoxia during the end-Permian mass extinction. NATURE GEOSCIENCE. 2020;13(9):640+.

6169. Godoy-Izquierdo Debora, Navarron Estefania, Lopez-Mora Clara, Gonzalez-Hernandez Juan Exercise Addiction in the Sports Context: What Is Known and What Is Yet to Be Known. INTERNATIONAL JOURNAL OF MENTAL HEALTH AND ADDICTION. 2023;21(2):1057-1074.

6170. Vitale Michele, Doherty Sean Lifestyle and weight changes among immigrants in Canada. INTERNATIONAL JOURNAL OF MIGRATION HEALTH AND SOCIAL CARE. 2018;14(4):439-454.

6171. Natale Ruby, Messiah Sarah, Asfour Lila, Uhlhorn Susan, Englebert Nicole, Arheart Kristopher Obesity Prevention Program in Childcare Centers: Two-Year Follow-Up. AMERICAN JOURNAL OF HEALTH PROMOTION. 2017;31(6):502-510.

6172. Luyckx Koen, Gandhi Amarendra, Bijttebier Patricia, Claes Laurence Non-suicidal self-injury in female adolescents and psychiatric patients: A replication and extension of the role

of identity formation. PERSONALITY AND INDIVIDUAL DIFFERENCES. 2015;77():91-96.

6173. IKETANI T, KIRIIKE N, NAKANISHI S, NAKASUJI T EFFECTS OF WEIGHT-GAIN AND RESUMPTION OF MENSES ON REDUCED BONE-DENSITY IN PATIENTS WITH ANOREXIA-NERVOSA. BIOLOGICAL PSYCHIATRY. 1995;37(8):521-527.

6174. Hicks JW, Wang T Cardiovascular regulation during anoxia in the turtle: An in vivo study. PHYSIOLOGICAL ZOOLOGY. 1998;71(1):1-14.

6175. Marco Jose, Canabate Montserrat, Martinez Cristina, Banos Rosa, Guillen Veronica, Perez Sandra Meaning in Life Mediates Between Emotional Deregulation and Eating Disorders Psychopathology: A Research From the Meaning-Making Model of Eating Disorders. FRONTIERS IN PSYCHOLOGY. 2021;12():.

6176. Cowell Daniel Autoerotic Asphyxiation: Secret Pleasure-Lethal Outcome?. PEDIATRICS. 2009;124(5):1319-1324.

6177. Hooshmand Farnaz, Do Dennis, Shah Saloni, Gershon Anda, Park Dong, Yuen Laura, Dell'Osso Bernardo, Wang Po, Miller Shefali, Ketter Terence Antidepressants have complex associations with longitudinal depressive burden in bipolar disorder. JOURNAL OF AFFECTIVE DISORDERS. 2019;246():836-842.

6178. Zsuga Judit, Tajti Gabor, Papp Csaba, Juhasz Bela, Gesztelyi Rudolf FNDC5/irisin, a molecular target for boosting reward-related learning and motivation. MEDICAL HYPOTHESES. 2016;90():23-28.

6179. Cheah Jason, Muhammed Julieana, Tharmathurai Sangeetha, Hamzah Norhafizah, Rahmat Jamalia Optic Neuropathy in an Autistic Child With Vitamin A Deficiency: A Case Report and Literature Review. CUREUS JOURNAL OF MEDICAL SCIENCE. 2022;14(2):.

6180. Guarda Angela, Schreyer Colleen, Boersma Gretha, Tamashiro Kellie, Moran Timothy Anorexia nervosa as a motivated behavior: Relevance of anxiety, stress, fear and learning. PHYSIOLOGY \& BEHAVIOR. 2015;152(B, SI):466-472.

6181. Jiang Shu-Man, Jia Lin, Lei Xiao-Gai, Xu Ming, Wang Sheng-Bing, Liu Jing, Song Min, Li Wei-Dong Incidence and psychological-behavioral characteristics of refractory functional dyspepsia: A large, multi-center, prospective investigation from China. WORLD JOURNAL OF

GASTROENTEROLOGY. 2015;21(6):1932-1937.

6182. Gallimberti Luigi, Buja Alessandra, Chindamo Sonia, Rabensteiner Andrea, Terraneo Alberto, Marini Elena, Perez Luis, Baldo Vincenzo Problematic Use of Video Games and Substance Abuse in Early Adolescence: A Cross-sectional Study. AMERICAN JOURNAL OF HEALTH BEHAVIOR. 2016;40(5):594-603.

6183. Kask J., Ramklint M., Kolia N., Panagiotakos D., Ekbom A., Ekselius L., Papadopoulos F. Anorexia nervosa in males: excess mortality and psychiatric co-morbidity in 609 Swedish in-patients. PSYCHOLOGICAL MEDICINE. 2017;47(8):1489-1499.

6184. Pikija Slaven, Trkulja Vladimir, Malojcic Branko, Mutzenbach J., Sellner Johann A High Burden of Ischemic Stroke in Regions of Eastern/Central Europe is Largely Due to Modifiable Risk Factors. CURRENT NEUROVASCULAR RESEARCH. 2015;12(4):341-352.

6185. Chirico Alice, Malighetti Clelia, Serino Silvia, Cipresso Pietro, Pedroli Elisa, Tuena Cosimo, Muratore Manuel, Riva Giuseppe Towards an Advancement of Multisensory Integration Deficits in Anorexia Nervosa: Exploring Temporal Discrimination Processing of Visuo-Auditory Stimuli. ANNUAL REVIEW OF CYBERTHERAPY AND TELEMEDICINE. 2019;17():53-58.

6186. Wu Li-Tzy, Blazer Dan, Gersing Kenneth, Burchett Bruce, Swartz Marvin, Mannelli Paolo, Workgrp NIDA Comorbid substance use disorders with other Axis I and II mental disorders among treatment-seeking Asian Americans, Native Hawaiians/Pacific Islanders, and mixed-race people. JOURNAL OF PSYCHIATRIC RESEARCH. 2013;47(12):1940-1948.

6187. Barzega G, Maina G, Venturello S, Bogetto F Gender-related differences in the onset of panic disorder. ACTA PSYCHIATRICA SCANDINAVICA. 2001;103(3):189-195.

6188. Thombs DL, Rosenberg JM, Mahoney CA, Daniel EL Weight-loss expectancies, relative weight, and symptoms of bulimia in young women. JOURNAL OF COLLEGE STUDENT DEVELOPMENT. 1996;37(4):405-414.

6189. SCHREIBER W, KRIEG JC DIAGNOSIS AND TREATMENT OF ANOREXIA-NERVOSA AND BULIMIA-NERVOSA. NERVENHEILKUNDE. 1995;14(3):136-142.

6190. Ghaderi Ata A European perspective on ``clean eating{''}: Commentary on Negowetti et al. (2021). INTERNATIONAL JOURNAL OF EATING DISORDERS. 2022;55(1):49-51.

6191. Balestrin Mariana, Brasil Carla, Bellei Ericles, Kirsten Vanessa, Wagner Mario Program for Healthier School Cafeterias in Rio Grande do Sul, Brazil: Protocol for a Community-Based Randomized Trial. JMIR RESEARCH PROTOCOLS. 2021;10(1):.

6192. Silber TJ, Shaer C, Atkins D Eating disorders in adolescents and young women with spina bifida. INTERNATIONAL JOURNAL OF EATING DISORDERS. 1999;25(4):457-461.

6193. Kim Byoungjun, Troxel Wendy, Dubowitz Tamara, Hunter Gerald, Ghosh-Dastidar Bonnie, Chaix Basile, Rudolph Kara, Morrison Christopher, Branas Charles, Duncan Dustin Neighborhood Built Environment and Sleep Health: A Longitudinal Study in Low-Income and Predominantly African-American Neighborhoods. AMERICAN JOURNAL OF EPIDEMIOLOGY. 2023;():.

6194. Zhang Z., Joachimski M., Grasby S., Sun Y. Intensive ocean anoxia and large d(13)C(carb)perturbations during the Carnian Humid Episode (Late Triassic) in Southwest China. GLOBAL AND PLANETARY CHANGE. 2022;217():.

6195. Dai Xu, Yuan Zhiwei, Brayard Arnaud, Li Mingtao, Liu Xiaokang, Jia Enhao, Du Yong, Song Huyue, Song Haijun Calibrating the late Smithian (Early Triassic) crisis: New insights from the Nanpanjiang Basin, South China. GLOBAL AND PLANETARY CHANGE. 2021;201():.

6196. Campbell Marcelo NCD Prevention and International Investment Law in Latin America: Chile's Experience in Preventing Obesity and Unhealthy Diets. JOURNAL OF WORLD INVESTMENT \& TRADE. 2020;21(5, SI):781-808.

6197. Carpita Barbara, Cremone Ivan, Amatori Giulia, Cappelli Andrea, Salerni Antonio, Massimetti Gabriele, Borgioli Davide, Carmassi Claudia, Massai Rossano, Dell'Osso Liliana Investigating the relationship between orthorexia nervosa and autistic traits in a university population. CNS SPECTRUMS. 2022;27(5):613-620.

6198. Sivamaruthi Bhagavathi, Kesika Periyanaina, Chaiyasut Chaiyavat The Role of Probiotics in Colorectal Cancer Management. EVIDENCE-BASED COMPLEMENTARY AND ALTERNATIVE MEDICINE. 2020;2020():.

6199. Milton SL, Thompson JW, Lutz PL Mechanisms for maintaining extracellular glutamate levels in the anoxic turtle striatum. AMERICAN JOURNAL OF PHYSIOLOGY-REGULATORY INTEGRATIVE AND COMPARATIVE PHYSIOLOGY. 2002;282(5):R1317-R1323.

6200. Scarpellini E., Cafarotti S., Cesario A., Lococo F., Margaritora S., Gabrielli M., Tortora A., Gasbarrini A., Granone P. A case of vomiting in an anorexic achalasic patient. EUROPEAN REVIEW FOR MEDICAL AND PHARMACOLOGICAL SCIENCES. 2012;16(4):44-47.

6201. BarChana Micha, Levav Itzhak, Lipshitz Irena, Pugachova Inna, Kohn Robert, Weizman Abraham, Grinshpoon Alexander Enhanced cancer risk among patients with bipolar disorder. JOURNAL OF AFFECTIVE DISORDERS. 2008;108(1-2):43-48.

6202. Booth David, Nouwen Arie Satiety. No way to slim. APPETITE. 2010;55(3):718-721.

6203. Scott Stephanie, Parkinson Kathryn, Kaner Eileen, Robalino Shannon, Stead Martine, Power Christine, Fitzgerald Niamh, Wrieden Wendy, Adamson Ashley Non-pharmacological interventions designed to reduce health risks due to unhealthy eating behaviour and linked risky or excessive drinking in adults aged 18-25 years: a systematic review protocol. SYSTEMATIC REVIEWS. 2017;6():.

6204. Gall Kelly, Zutven Kim, Lindstrom Joanna, Bentley Caroline, Gratwick-Sarll Kassandra, Harrison Carmel, Lewis Vivienne, Mond Jonathan Obesity and emotional well-being in adolescents: Roles of body dissatisfaction, loss of control eating, and self-rated health. OBESITY. 2016;24(4):837-842.

6205. Cisternas Pedro, Lindsay Carolina, Salazar Paulina, Silva-Alvarez Carmen, Retamales Rocio, Serrano Felipe, Vio Carlos, Inestrosa Nibaldo The increased potassium intake improves cognitive performance and attenuates histopathological markers in a model of Alzheimer's disease. BIOCHIMICA ET BIOPHYSICA ACTA-MOLECULAR BASIS OF DISEASE. 2015;1852(12):2630-2644.

6206. Rogalska Justyna, Caputa Michal, Piatkowska Katarzyna, Nowakowska Anna Neonatal asphyxia and hyperthermia and cognitive deficits in adult rats: Role of iron. JOURNAL OF THERMAL BIOLOGY. 2009;34(8):391-400.

6207. Brath H., Globits S., Siostrzonek P., Speidl W., Weber T. Primary and secondary prevention in cardiovascular diseases. JOURNAL FUR KARDIOLOGIE. 2020;27(1-2):12-30.

6208. Schreyer Colleen, Vanzhula Irina, Guarda Angela Evaluating the impact of COVID-19 on severity at admission and response to inpatient treatment for adult and adolescent patients with eating disorders. INTERNATIONAL JOURNAL OF EATING DISORDERS. 2023;56(1, SI):182-191.

6209. Jia Lin, Jiang Shu-Man, Liu Jing Behavioral gastroenterology: An emerging system and new frontier of action. WORLD JOURNAL OF GASTROENTEROLOGY. 2017;23(33):6059-6064.

6210. Lasserre A., Strippoli M-P, Glaus J., Gholam-Rezaee M., Vandeleur C., Castelao E., Marques-Vidal P., Waeber G., Vollenweider P., Preisig M. Prospective associations of depression subtypes with cardio-metabolic risk factors in the general population. MOLECULAR PSYCHIATRY. 2017;22(7):1026-1034.

6211. Filippone Lisa, Shankland Rebecca, Hallez Quentin The relationships between social media exposure, food craving, cognitive impulsivity and cognitive restraint. JOURNAL OF EATING DISORDERS. 2022;10(1):.

6212. Mitchell Karen, Smith Brian, Masheb Robin, Vogt Dawne The impact of the COVID-19 pandemic on eating disorders in US military veterans. INTERNATIONAL JOURNAL OF EATING DISORDERS. 2023;56(1, SI):108-117.

6213. Madlala Samukelisiwe, Hill Jillian, Kunneke Ernesta, Kengne Andre, Peer Nasheeta, Faber Mieke Dietary Diversity and its Association with Nutritional Status, Cardiometabolic Risk Factors and Food Choices of Adults at Risk for Type 2 Diabetes Mellitus in Cape Town, South Africa. NUTRIENTS. 2022;14(15):.

6214. Wong C., Faiz D., Safraa Diana, Azim Raja, Zubaidah Siti Prevalence and Modifiable Risk Factors of Non-Communicable Diseases among Jakun Orang Asli at Tasik Chini, Pekan, Pahang.. INTERNATIONAL MEDICAL JOURNAL MALAYSIA. 2018;17(3):3-15.

6215. Docet Maria, Larranaga Alejandra, Fernandez Sastre Jose, Garcia-Mayor Ricardo High rate of attention deficit hyperactivity disorder in obese adults: a case-control study. OBESITY AND METABOLISM-MILAN. 2010;6(4):121-124.

6216. Guengoer Ekin, Celebi Cengiz, Akvardar Yildiz The Relationship of Food Addiction With Other Eating Pathologies and Impulsivity: A Case-Control Study. FRONTIERS IN PSYCHIATRY. 2021;12():.

6217. Dell'Osso Bernardo, Vismara Matteo, Dobrea Cristina, Cremaschi Laura, Grancini Benedetta, Arici Chiara, Benatti Beatrice, Buoli Massimiliano, Ketter Terence, Altamura A. Clinical characterization of Italian suicide attempters with bipolar disorder. CNS SPECTRUMS. 2018;23(4):271-277.

6218. Erdal Gulcin, Yaman Mustafa, Servi Esra, Ugur Halime, Kasapoglu Pinar, Cikot Murat, Isiksacan Nilgun Measurement of Advanced Glycation End Products Could Be Used as an Indicator of Unhealthy Nutrition for Colorectal Cancer Risk. NUTRITION AND CANCER-AN INTERNATIONAL JOURNAL. 2022;74(3):896-902.

6219. Zhu Shuzhen, Hu Jie, McCoy Thomas, Li Guangwu, Zhu Jianyong, Lei Meirong, Yuan Jie, Peng Jingxia, Kong Linglin Socioeconomic Status and the Prevalence of Type 2 Diabetes Among Adults in Northwest China. DIABETES EDUCATOR. 2015;41(5):599-608.

6220. Kouvari Matina, Tsiampalis Thomas, Kosti Rena, Naumovski Nenad, Chrysohoou Christina, Skoumas John, Pitsavos Christos, Panagiotakos Demosthenes, Mantzoros Christos Quality of plant-based diets is associated with liver steatosis, which predicts type 2 diabetes incidence ten years later: Results from the ATTICA prospective epidemiological study. CLINICAL NUTRITION. 2022;41(10):2094-2102.

6221. Espinosa Adriana, Kadic-Maglajlic Selma The Mediating Role of Health Consciousness in the Relation Between Emotional Intelligence and Health Behaviors. FRONTIERS IN PSYCHOLOGY. 2018;9():.

6222. Brown Joanna, Colson Gregory, La Serre Claire, Magnan Nicholas Summer Garden Programs Improve Children's Food Knowledge and Preferences: Evidence Using Stated and Revealed Preference Measures. HORTTECHNOLOGY. 2016;26(2):133-140.

6223. Marzola Enrica, Martini Matteo, Brustolin Annalisa, Abbate-Daga Giovanni Inpatients with severe-enduring anorexia nervosa: Understanding the ``enduringness{''} specifier. EUROPEAN PSYCHIATRY. 2021;64(1):.

6224. French SA, Story M, Neumark-Sztainer D, Fulkerson JA, Hannan P Fast food restaurant use among adolescents: associations with nutrient intake, food choices and behavioral and

psychosocial variables. INTERNATIONAL JOURNAL OF OBESITY. 2001;25(12):1823-1833.

6225. Aguiar-Bloemer Ana, Agliussi Rosina, Pereira Pinho Thiago, Furtado Erikson, Diez-Garcia Rosa Eating behavior of schizophrenic patients. REVISTA DE NUTRICAO-BRAZILIAN JOURNAL OF NUTRITION. 2018;31(1):13-24.

6226. Reavie ED, Neill KE, Little JL, Smol JP Cultural eutrophication trends in three southeastern Ontario lakes: A paleolimnological perspective. LAKE AND RESERVOIR MANAGEMENT. 2006;22(1):44-58.

6227. Cobiac Linda, Veerman Lennert, Vos Theo The Role of Cost-Effectiveness Analysis in Developing Nutrition Policy. . 2013;33():373-393.

6228. Turner Dennis, Degan Simone, Hoffmann Ulrike, Galeffi Francesca, Colton Carol CVN-AD Alzheimer's mice show premature reduction in neurovascular coupling in response to spreading depression and anoxia compared to aged controls. ALZHEIMERS \& DEMENTIA. 2021;17(7):1109-1120.

6229. PUKASUNDVALL M, HAGBERG H, ANDINE P CHANGES IN EXTRACELLULAR CALCIUM-CONCENTRATION IN THE IMMATURE RAT CEREBRAL-CORTEX DURING ANOXIA ARE NOT INFLUENCED BY MK-801. DEVELOPMENTAL BRAIN RESEARCH. 1994;77(1):146-150.

6230. Hall James Promoting healthy hearing over the lifespan. AUDITORY AND VESTIBULAR RESEARCH. 2021;30(2):74-94.

6231. vanDellen Michelle, Isherwood Jennifer, Delose Julie How do people define moderation?. APPETITE. 2016;101():156-162.

6232. Renteria Ivan, Garcia-Suarez Patricia, Moncada-Jimenez Jose, Machado-Parra Juan, Antunes Barbara, Lira Fabio, Jimenez-Maldonado Alberto Unhealthy Dieting During the COVID-19 Pandemic: An Opinion Regarding the Harmful Effects on Brain Health. FRONTIERS IN NUTRITION. 2022;9():.

6233. Madesh M, Ramachandran A, Balasubramanian KA Nitric oxide prevents anoxia-induced apoptosis in colonic HT29 cells. ARCHIVES OF BIOCHEMISTRY AND BIOPHYSICS.

1999;366(2):240-248.

6234. Chidekel AS, Friedman JE, Haddad GG Anoxia-induced neuronal injury: Role of Na+ entry and Na+-dependent transport. EXPERIMENTAL NEUROLOGY. 1997;146(2):403-413.

6235. {[}Anonymous] Prevention of anoxia reoxygenation injury with chimeric superoxide 2,3.. JOURNAL OF ENDOUROLOGY. 2004;18(1):A5.

6236. Palmieri Daniela, Aliakbarian Bahar, Casazza Alessandro, Ferrari Nicoletta, Spinella Giovanni, Pane Bianca, Cafueri Giuseppe, Perego Patrizia, Palombo Domenico Effects of polyphenol extract from olive pomace on anoxia-induced endothelial dysfunction. MICROVASCULAR RESEARCH. 2012;83(3):281-289.

6237. Mars Becky, Harold Gordon, Elam Kit, Sellers Ruth, Owen Michael, Craddock Nicholas, Thapar Ajay, Rice Frances, Collishaw Stephan, Thapar Anita Specific Parental Depression Symptoms as Risk Markers for New-Onset Depression in High-Risk Offspring. JOURNAL OF CLINICAL PSYCHIATRY. 2013;74(9):925-931.

6238. SCHLUTER KD, WEBER M, SCHRAVEN E, PIPER HM NO DONOR SIN-1 PROTECTS AGAINST REOXYGENATION-INDUCED CARDIOMYOCYTE INJURY BY A DUAL-ACTION. AMERICAN JOURNAL OF PHYSIOLOGY-HEART AND CIRCULATORY PHYSIOLOGY. 1994;267(4):H1461-H1466.

6239. Urano Ayako, Hotta Mari, Ohwada Rina, Araki Mariko Vitamin K deficiency evaluated by serum levels of undercarboxylated osteocalcin in patients with anorexia nervosa with bone loss. CLINICAL NUTRITION. 2015;34(3):443-448.

6240. Kourmouli Niki, Samakouri Maria, Mamatsiou Athina, Trypsianis Gregory, Livaditis Miltos, Veletza Stavroula Effect of BDNF Val66Met and serotonin transporter 5-HTTLPR polymorphisms on psychopathological characteristics in a sample of university students. PSYCHIATRIC GENETICS. 2013;23(5):188-197.

6241. Swenne I Weight requirements for return of menstruations in teenage girls with eating disorders, weight loss and secondary amenorrhoea. ACTA PAEDIATRICA. 2004;93(11):1449-1455.

6242. Gozal D, Torres JE Maturation of anoxia-induced gasping in the rat: Potential role for N-methyl-D-aspartate glutamate receptors. PEDIATRIC RESEARCH. 1997;42(6):872-877.

6243. Saadati Kiyana, Chaboksavar Fakhreddin, Ghoozlu Khadije, Shamsalinia Abbas, Kordbageri Mohammad, Ghadimi Reza, Porasgari Zeinab, Ghaffari Fatemeh Evaluation of psychometric properties of dietary habits, lifestyle, food frequency consumption, and nutritional beliefs (KomPAN) questionnaire in Iranian adults. FRONTIERS IN PUBLIC HEALTH. 2022;10():.

6244. Perez Rodriguez Lianet, Utrera Diaz Grissel, Rodriguez Martinez Laritza Lifestyles characterization in hypertensive patients of the doctor ` s office 7 area IV. Cienfuegos, 2021. MEDISUR-REVISTA DE CIENCIAS MEDICAS DE CIENFUEGOS. 2022;20(6):1124-1131.

6245. Mousavizadeh Zohreh, Hosseini-Esfahani Firoozeh, Javadi Amir, Daneshpour Maryam, Akbarzadeh Mahdi, Javadi Maryam, Mirmrian Parvin, Azizi Fereidoun The interaction between dietary patterns and melanocortin-4 receptor polymorphisms in relation to obesity phenotypes. OBESITY RESEARCH \& CLINICAL PRACTICE. 2020;14(3):249-256.

6246. Thorndike Anne, Gelsomin Emily, McCurley Jessica, Levy Douglas Calories Purchased by Hospital Employees After Implementation of a Cafeteria Traffic Light-Labeling and Choice Architecture Program. JAMA NETWORK OPEN. 2019;2(7):.

6247. Bammann Karin, Peplies Jenny, Pigeot Iris, Ahrens Wolfgang IDEFICS: a Multicenter European project on diet- and lifestyle-related disorders in children. MEDIZINISCHE KLINIK. 2007;102(3):230-235.

6248. Rynkiewicz Agnieszka, Janas-Kozik Malgorzata, Slopien Agnieszka Girls and women with autism. PSYCHIATRIA POLSKA. 2019;53(4):737-752.

6249. Guimaraes Aline, Machado Soraia, Cunha Frana Ana, Calado Isabela EATING DISORDERS AND DISSATISFACTION WITH BODY IMAGE IN BALLET DANCERS. REVISTA BRASILEIRA DE MEDICINA DO ESPORTE. 2014;20(4):267-271.

6250. Subic-Wrana C, Bruder S, Thomas W, Gaus E, Merkle W, Kohle K Distribution of alexithymia as a personality-trait in psychosomatically ill in-patients - Measured with TAS 20 and LEAS. PSYCHOTHERAPIE PSYCHOSOMATIK MEDIZINISCHE PSYCHOLOGIE. 2002;52(11):454-460.

6251. Olavarria Veronica, Campodonico Paola, Vollrath Valeska, Geldern Paula, Velasquez Carolina, Pavez Patricia, Valente Barbara, Donoso Pamela, Ginesta Alexandra, Cavada Gabriel, Mazzon Enrico, Navia Victor, Guzman Matias, Brinck Pablo, Lavados Pablo Effects of an Avocado-based Mediterranean Diet on Serum Lipids for Secondary Prevention after Ischemic Stroke Trial (ADD-SPISE) Study protocol. MEDICINE. 2021;100(24):.

6252. Cook-Cottone Catherine, Guyker Wendy The Development and Validation of the Mindful Self-Care Scale (MSCS): an Assessment of Practices that Support Positive Embodiment. MINDFULNESS. 2018;9(1):161-175.

6253. Gomez-Delgado Francisco, Katsiki Niki, Lopez-Miranda Jose, Perez-Martinez Pablo Dietary habits, lipoprotein metabolism and cardiovascular disease: From individual foods to dietary patterns. CRITICAL REVIEWS IN FOOD SCIENCE AND NUTRITION. 2021;61(10):1651-1669.

6254. Panagiotakos Demosthenes, Georgousopoulou Ekavi, Georgiopoulos Georgios, Pitsavos Christos, Chrysohoou Christina, Skoumas Ioannis, Ntertimani Maria, Laskaris Alexandros, Papadimitriou Lampros, Tousoulis Dimitrios, Stefanadis Christodoulos, Grp ATTICA Adherence to Mediterranean Diet Offers an Additive Protection Over the Use of Statin Therapy: Results from the ATTICA Study (2002-2012). CURRENT VASCULAR PHARMACOLOGY. 2015;13(6):778-787.

6255. Le Long, Barendregt Jan, Hay Phillipa, Mihalopoulos Cathrine Prevention of eating disorders: A systematic review and meta-analysis. CLINICAL PSYCHOLOGY REVIEW. 2017;53():46-58.

6256. Crisostomo Luis, Jarak Ivana, Rato Luis, Raposo Joao, Batterham Rachel, Oliveira Pedro, Alves Marco Inheritable testicular metabolic memory of high-fat diet causes transgenerational sperm defects in mice. SCIENTIFIC REPORTS. 2021;11(1):.

6257. Tremblay Simon, Tremblay Safae, Poirier Pierre From filters to fillers: an active inference approach to body image distortion in the selfie era. AI \& SOCIETY. 2021;36(1):33-48.

6258. Nitsch Martina, Dimopoulos Christina, Flaschberger Edith, Saffran Kristina, Kruger Jenna, Garlock Lindsay, Wilfley Denise, Taylor Craig, Jones Megan A Guided Online and Mobile Self-Help Program for Individuals With Eating Disorders: An Iterative Engagement and Usability Study. JOURNAL OF MEDICAL INTERNET RESEARCH. 2016;18(1):.

6259. Adjibade Moufidath, Assmann Karen, Julia Chantal, Galan Pilar, Hercberg Serge, Kesse-Guyot Emmanuelle Prospective association between adherence to the MIND diet and subjective memory complaints in the French NutriNet-Sante cohort. JOURNAL OF NEUROLOGY. 2019;266(4):942-952.

6260. Wang Yan, Sugar David Internal browning disorder and fruit quality in modified atmosphere packaged `Bartlett' pears during storage and transit. POSTHARVEST BIOLOGY AND TECHNOLOGY. 2013;83():72-82.

6261. Gehring Josephine, Touvier Mathilde, Baudry Julia, Julia Chantal, Buscail Camille, Srour Bernard, Hercberg Serge, Peneau Sandrine, Kesse-Guyot Emmanuelle, Alles Benjamin Consumption of Ultra-Processed Foods by Pesco-Vegetarians, Vegetarians, and Vegans: Associations with Duration and Age at Diet Initiation. JOURNAL OF NUTRITION. 2021;151(1):120-131.

6262. Pinar-Gutierrez Ana, Dios-Fuentes Elena, Remon-Ruiz Pablo, Del Can-Sanchez Diego, Vazquez-Morejon Antonio, Lopez-Narbona Marta, Guzman Javier, Venegas-Moreno Eva, Soto-Moreno Alfonso Description of characteristics and outcomes of a cohort of patients with severe and enduring eating disorders (SE-ED). JOURNAL OF EATING DISORDERS. 2021;9(1):.

6263. Venturello S, Barzega G, Maina G, Bogetto F Premorbid conditions and precipitating events in early-onset panic disorder. COMPREHENSIVE PSYCHIATRY. 2002;43(1):28-36.

6264. Wilksch Simon, Tiggemann Marika, Wade Tracey Impact of interactive school-based media literacy lessons for reducing internalization of media ideals in young adolescent girls and boys. INTERNATIONAL JOURNAL OF EATING DISORDERS. 2006;39(5):385-393.

6265. KENNEDY SH MELATONIN DISTURBANCES IN ANOREXIA-NERVOSA AND BULIMIA-NERVOSA. INTERNATIONAL JOURNAL OF EATING DISORDERS. 1994;16(3):257-265.

6266. Klymiuk Ashley, Sikes Benjamin Suppression of root-endogenous fungi in persistently inundated Typha roots. MYCOLOGIA. 2019;111(5):748-757.

6267. Aldhban Abdullah, Abukhodair Abdulaziz, Fakeih Elaf, ALdakhlan Hussain, Alhassan Mohammed, Heameed Roaa, Jumah Suad, Alaish Mohammed, Alqahtani Saad, Hisan Fatimah DIETARY MANAGEMENT OF TYPE 2 DIABETES. INDO AMERICAN JOURNAL OF

PHARMACEUTICAL SCIENCES. 2018;5(11):13571-13577.

6268. Couturier Jennifer, Ma Zechen, Rahman Liah, Webb Cheryl A mixed methods exploratory evaluation of burnout in frontline staff implementing dialectical behavior therapy on a pediatric eating disorders unit. JOURNAL OF EATING DISORDERS. 2021;9(1):.

6269. Eleftheriadis Theodoros, Pissas Georgios, Nikolaou Evdokia, Filippidis Georgios, Liakopoulos Vassilios, Stefanidis Ioannis Mistimed H2S upregulation, Nrf2 activation and antioxidant proteins levels in renal tubular epithelial cells subjected to anoxia and reoxygenation. BIOMEDICAL REPORTS. 2020;13(2):.

6270. Stice E, Nemeroff C, Shaw HE Test of the dual pathway model of bulimia nervosa: Evidence for dietary restraint and affect regulation mechanisms. JOURNAL OF SOCIAL AND CLINICAL PSYCHOLOGY. 1996;15(3):340-363.

6271. Watson Hunna, Goodman Erica, McLagan Nicole, Joyce Tara, French Elizabeth, Willan Vivienne, Egan Sarah Quality of randomized controlled trials in eating disorder prevention. INTERNATIONAL JOURNAL OF EATING DISORDERS. 2017;50(5):459-470.

6272. Vetter Sven, Elsaesser Albrecht, Tutdibi Osman, Lang Sabrina, Schoels Wolfgang, Pott Anja, Ackermann Cordula, Reinhard Constanze, Wieland Felix, Katus Hugo, Kuebler Wolfgang, Vogt Achim Brief antecedent anoxia preserves mitochondrial function after sustained undersupply: A subcellular correlate to ischemic preconditioning?. MOLECULAR AND CELLULAR BIOCHEMISTRY. 2006;285(1-2):191-196.

6273. BUCKLER KJ, VAUGHANJONES RD EFFECTS OF HYPOXIA ON MEMBRANE-POTENTIAL AND INTRACELLULAR CALCIUM IN RAT NEONATAL CAROTID-BODY TYPE-I CELLS. JOURNAL OF PHYSIOLOGY-LONDON. 1994;476(3):423-428.

6274. Mounayar Rana, Jreij Rana, Hachem Jennifer, Abboud Frida, Tueni Maya Breakfast Intake and Factors Associated with Adherence to the Mediterranean Diet among Lebanese High School Adolescents. JOURNAL OF NUTRITION AND METABOLISM. 2019;2019():.

6275. Marrocos Leite Fernanda, Mais Lais, Ricardo Camila, Andrade Giovanna, Guimaraes Julia, Claro Rafael, Fonseca Leitao Duran Ana, Bortoletto Martins Ana Nutritional quality of foods and non-alcoholic beverages advertised on Brazilian free-to-air television: a cross-sectional study. BMC PUBLIC HEALTH. 2020;20(1):.

6276. Tovar Alison, Boulos Rebecca, Sliwa Sarah, Must Aviva, Gute David, Metayer Nesly, Hyatt Raymond, Chui Kenneth, Pirie Alex, Luongo Christina, Economos Christina Baseline Socio-demographic Characteristics and Self-Reported Diet and Physical Activity Shifts Among Recent Immigrants Participating in the Randomized Controlled Lifestyle Intervention: ``Live Well{''}. JOURNAL OF IMMIGRANT AND MINORITY HEALTH. 2014;16(3):457-465.

6277. Kaye WH, Frank GK, Bailer UF, Henry SE, Meltzer CC, Price JC, Mathis CA, Wagner A Serotonin alterations, in anorexia and bulimia nervosa: New insights from imaging studies. PHYSIOLOGY \& BEHAVIOR. 2005;85(1, SI):73-81.

6278. Gorrell Sasha, Reilly Erin, Brosof Leigh, Le Grange Daniel Use of Telehealth in the Management of Adolescent Eating Disorders: Patient Perspectives and Future Directions Suggested from the COVID-19 Pandemic. ADOLESCENT HEALTH MEDICINE AND THERAPEUTICS. 2022;13():45-53.

6279. Neumann Alexander, Nolte Ilja, Pappa Irene, Ahluwalia Tarunveer, Pettersson Erik, Rodriguez Alina, Whitehouse Andrew, Beijsterveldt Catharina, Benyamin Beben, Hammerschlag Anke, Helmer Quinta, Karhunen Ville, Krapohl Eva, Lu Yi, Most Peter, Palviainen Teemu, St Pourcain Beate, Seppaelae Ilkka, Suarez Anna, Vilor-Tejedor Natalia, Tiesler Carla, Wang Carol, Wills Amanda, Zhou Ang, Alemany Silvia, Bisgaard Hans, Bonnelykke Klaus, Davies Gareth, Hakulinen Christian, Henders Anjali, Hyppoenen Elina, Stokholm Jakob, Bartels Meike, Hottenga Jouke-Jan, Heinrich Joachim, Hewitt John, Keltikangas-Jaervinen Liisa, Korhonen Tellervo, Kaprio Jaakko, Lahti Jari, Lahti-Pulkkinen Marius, Lehtimaeki Terho, Middeldorp Christel, Najman Jackob, Pennell Craig, Power Chris, Oldehinkel Albertine, Plomin Robert, Raeikkoenen Katri, Raitakari Olli, Rimfeld Kaili, Sass Laerke, Snieder Harold, Standl Marie, Sunyer Jordi, Williams Gail, Bakermans-Kranenburg Marian, Boomsma Dorret, IJzendoorn Marinus, Hartman Catharina, Tiemeier Henning A genome-wide association study of total child psychiatric problems scores. PLOS ONE. 2022;17(8):.

6280. Willmore WG, Storey KB Antioxidant systems and anoxia tolerance in a freshwater turtle Trachemys scripta elegans. MOLECULAR AND CELLULAR BIOCHEMISTRY. 1997;170(1-2):177-185.

6281. Dashti Hassan, Mogensen Kris Recommending Small, Frequent Meals in the Clinical Care of Adults: A Review of the Evidence and Important Considerations. NUTRITION IN CLINICAL PRACTICE. 2017;32(3):365-377.

6282. Dohnt Hayley, Tiggemann Marika Promoting positive body image in young girls: An evaluation of `Shapesville'. EUROPEAN EATING DISORDERS REVIEW. 2008;16(3):222-233.

6283. Selby Edward, Harnedy Lauren, Hiner Michelle, Kim Joanne Developmental and Momentary Dynamics in the Onset and Maintenance of Nonsuicidal Self-Injurious Behavior and Borderline Personality Disorder. CURRENT PSYCHIATRY REPORTS. 2022;24(12):897-909.

6284. Morylowska-Topolska Justyna, Zieminski Rafal, Molas Agnieszka, Gajewski Jacek, Flis Marta, Stelmach Ewa, Karakula-Juchnowicz Hanna Schizophrenia and anorexia nervosa - reciprocal relationships. A literature review. PSYCHIATRIA POLSKA. 2017;51(2):261-270.

6285. Zhang Chenshu, Brook Judith, Leukefeld Carl, De La Rosa Mario, Brook David Lack of Preventive Health Behaviors in the Early Forties: The Role of Earlier Trajectories of Cigarette Smoking From Adolescence to Adulthood. SUBSTANCE USE \& MISUSE. 2017;52(12):1527-1537.

6286. Powell Heidi, Greenberg Deborah Screening for unhealthy diet and exercise habits: The electronic health record and a healthier population. PREVENTIVE MEDICINE REPORTS. 2019;14():.

6287. Lawniczak M., Romestaing C., Roussel D., Maazouzi C., Renault D., Hervant F. Preventive antioxidant responses to extreme oxygen level fluctuation in a subterranean crustacean. COMPARATIVE BIOCHEMISTRY AND PHYSIOLOGY A-MOLECULAR \& INTEGRATIVE PHYSIOLOGY. 2013;165(2):299-303.

6288. Lampard Amy, MacLehose Richard, Eisenberg Marla, Neumark-Sztainer Dianne, Davison Kirsten Weight-Related Teasing in the School Environment: Associations with Psychosocial Health and Weight Control Practices Among Adolescent Boys and Girls. JOURNAL OF YOUTH AND ADOLESCENCE. 2014;43(10, SI):1770-1780.

6289. Mottarlini Francesca, Rizzi Beatrice, Targa Giorgia, Fumagalli Fabio, Caffino Lucia Long-lasting BDNF signaling alterations in the amygdala of adolescent female rats exposed to the activity-based anorexia model. FRONTIERS IN BEHAVIORAL NEUROSCIENCE. 2022;16():.

6290. Shoebridge P, Gowers SG Parental high concern and adolescent-onset anorexia nervosa - A case-control study to investigate direction of causality. BRITISH JOURNAL OF

PSYCHIATRY. 2000;176():132-137.

6291. Wilksch Simon School-based eating disorder prevention: a pilot effectiveness trial of teacher-delivered Media Smart. EARLY INTERVENTION IN PSYCHIATRY. 2015;9(1):21-28.

6292. Hasin D, Samet S, Nunes E, Meydan J, Matseoane K, Waxman R Diagnosis of comorbid psychiatric disorders in substance users assessed with the Psychiatric Research Interview for Substance and Mental Disorders for DSM-IV. AMERICAN JOURNAL OF PSYCHIATRY. 2006;163(4):689-696.

6293. Oudman Erik, Wijnia Jan, Oey Misha, Dam Mirjam, Postma Albert Preventing Wernicke's encephalopathy in anorexia nervosa: A systematic review. PSYCHIATRY AND CLINICAL NEUROSCIENCES. 2018;72(10):774-779.

6294. Levinson Cheri, Spoor Samantha, Keshishian Ani, Pruitt Alexandria Pilot outcomes from a multidisciplinary telehealth versus in-person intensive outpatient program for eating disorders during versus before the Covid-19 pandemic. INTERNATIONAL JOURNAL OF EATING DISORDERS. 2021;54(9):1672-1679.

6295. Bissonnette-Maheux Veronique, Provencher Veronique, Lapointe Annie, Dugrenier Marilyn, Dumas Audree-Anne, Pluye Pierre, Straus Sharon, Gagnon Marie-Pierre, Desroches Sophie Exploring Women's Beliefs and Perceptions About Healthy Eating Blogs: A Qualitative Study. JOURNAL OF MEDICAL INTERNET RESEARCH. 2015;17(4):.

6296. Dzhala V, Ben-Ari Y, Khazipov R Seizures accelerate anoxia-induced neuronal death in the neonatal rat hippocampus. ANNALS OF NEUROLOGY. 2000;48(4):632-640.

6297. Dowson J Associations of the severity of depressive disorders in women with psychogenic low weight. JOURNAL OF AFFECTIVE DISORDERS. 2004;78(3):279-284.

6298. Eleftheriadis Theodoros, Pissas Georgios, Antoniadi Georgia, Golfinopoulos Spyridon, Liakopoulos Vassilios, Stefanidis Ioannis Energy handling in renal tubular epithelial cells of the hamster, a native hibernator, under warm anoxia or reoxygenation. BIOMEDICAL REPORTS. 2018;9(6):503-510.

6299. Goldstein Rise, Chou S., Saha Tulshi, Smith Sharon, Jung Jeesun, Zhang Haitao, Pickering Roger, Ruan W., Huang Boji, Grant Bridget The Epidemiology of Antisocial

Behavioral Syndromes in Adulthood: Results From the National Epidemiologic Survey on Alcohol and Related Conditions-III. JOURNAL OF CLINICAL PSYCHIATRY. 2017;78(1):90-98.

6300. Aldhban Abdullah, Abukhodair Abdulaziz, Fakeih Elaf, ALdakhlan Hussain, Alhassan Mohammed, Heameed Roaa, Jumah Suad, Alaish Mohammed, Alqahtani Saad, Hisan Fatimah DIETARY MANAGEMENT OF TYPE 2 DIABETES. INDO AMERICAN JOURNAL OF PHARMACEUTICAL SCIENCES. 2018;5(12):16467-16473.

6301. Feng Yun, Lu Yingwei, Lin Xin, Gao Yanfeng, Zhao Qianyu, Li Wei, Wang Rui Endomorphins and morphine limit anoxia-reoxygenation-induced brain mitochondrial dysfunction in the mouse. LIFE SCIENCES. 2008;82(13-14):752-763.

6302. Jacobi C., Fittig E., Bryson S., Wilfley D., Kraemer H., Taylor C. Who is really at risk? Identifying risk factors for subthreshold and full syndrome eating disorders in a high-risk sample. PSYCHOLOGICAL MEDICINE. 2011;41(9):1939-1949.

6303. Rana Ammara, Safdar Ammarah, Kundi Junaid FREQUENCY OF RISK FACTORS OF STROKE IN PATIENTS PRESENTING TO PUBLIC SECTOR HOSPITALS OF LAHORE. INDO AMERICAN JOURNAL OF PHARMACEUTICAL SCIENCES. 2018;5(5):3884-3893.

6304. Norton Lyza, Hart Laura, Butel Francoise, Moloney Susan, O'Connor Narelle, Attenborough Vicki, Roberts Shelley Promoting Confident Body, Confident Child in community child health: A mixed-methods implementation study. HEALTH PROMOTION JOURNAL OF AUSTRALIA. 2022;33(1):297-305.

6305. Cuadrado-Rios Sofia, Fuad Huaman-Garaicoa, Karla Cruz-Moreira Anorexia and bulimia nervosa in the practice of the paediatric dentist. EUROPEAN EATING DISORDERS REVIEW. 2023;31(1):9-23.

6306. Gill Raveena, Tyndall Sean, Vora Darshini, Hasan Rashedul, Megna James, Leontieva Luba Diet Quality and Mental Health Amongst Acute Inpatient Psychiatric Patients. CUREUS. 2021;13(1):.

6307. Fiorillo A, Farina V, D'Amore R, Scippa L, Cortese P, DeChiara C Longitudinal assessment of cardiac status by echocardiographic evaluation of left ventricular diastolic function in thalassaemic children. ACTA PAEDIATRICA. 2000;89(4):436-441.

6308. ARORA AS, DEGROEN PC, CROALL DE, GORES GJ HEPATOCELLULAR-CARCINOMA CELLS RESIST NECROSIS DURING ANOXIA BY PREVENTING ACTIVATION OF CALPAINS. FASEB JOURNAL. 1995;9(3, 1):143.

6309. Nielsen Soren, Dobrescu Sandra, Dinkler Lisa, Gillberg Carina, Gillberg Christopher, Rastam Maria, Wentz Elisabet Effects of autism on 30-year outcome of anorexia nervosa. JOURNAL OF EATING DISORDERS. 2022;10(1):.

6310. Morris D., Trivedi M., Husain M., Fava M., Budhwar N., Wisniewski S., Miyahara S., Gollan J., Davis L., Daly E., Rush A. Indicators of pretreatment suicidal ideation in adults with major depressive disorder. ACTA PSYCHIATRICA SCANDINAVICA. 2010;121(6):480-484.

6311. Avdic Hanna, Butwicka Agnieszka, Nordenstrom Anna, Almqvist Catarina, Nordenskjold Agneta, Engberg Hedvig, Frisen Louise Neurodevelopmental and psychiatric disorders in females with Turner syndrome: a population-based study. JOURNAL OF NEURODEVELOPMENTAL DISORDERS. 2021;13(1):.

6312. Zheng J., Wu G., Hu G., Peng Y., Xiong X. Protective effects against and potential mechanisms underlying the effect of magnesium isoglycyrrhizinate in hypoxia-reoxygenation injury in rat liver cells. GENETICS AND MOLECULAR RESEARCH. 2015;14(4):15453-15461.

6313. Greenberg I, Perna F, Kaplan M, Sullivan MA Behavioral and psychological factors in the assessment and treatment of obesity surgery patients. OBESITY RESEARCH. 2005;13(2):244-249.

6314. Baradel Giorgia, Pratile Diletta, Orlandi Marika, Vecchio Arianna, Casini Erica, De Giorgis Valentina, Borgatti Renato, Mensi Martina, Clin Mondino Life Events in the Etiopathogenesis and Maintenance of Restrictive Eating Disorders in Adolescence. CHILDREN-BASEL. 2023;10(2):.

6315. Doolittle Heather, Norton Stephen, Bacon Linda, Ewing Holly, Amirbahman Aria The internal and watershed controls on hypolimnetic sediment phosphorus release in Lake Auburn, Maine, USA. LAKE AND RESERVOIR MANAGEMENT. 2018;34(3):258-269.

6316. Feldman Marissa, King Callie, Vitale Sarah, Denhardt Brenna, Stroup Susan, Reese Jasmine, Stromberg Sarah The impact of COVID-19 on adolescents with eating disorders: Increased need for medical stabilization and decreased access to care. INTERNATIONAL

JOURNAL OF EATING DISORDERS. 2023;56(1, SI):257-262.

6317. SUBBAIAH CC, BUSH DS, SACHS MM ELEVATION OF CYTOSOLIC CALCIUM PRECEDES ANOXIC GENE-EXPRESSION IN MAIZE SUSPENSION-CULTURED CELLS. PLANT CELL. 1994;6(12):1747-1762.

6318. Kim Jae-Sung, Nitta Takashi, Mohuczy Dagmara, O'Malley Kerri, Moldawer Lyle, Dunn William, Behrns Kevin Impaired autophagy: A mechanism of mitochondrial dysfunction in anoxic rat hepatocytes. HEPATOLOGY. 2008;47(5):1725-1736.

6319. Wilkie Michael, Pamenter Matthew, Alkabie Samir, Carapic Dejana, Shin Damian, Buck Leslie Evidence of anoxia-induced channel arrest in the brain of the goldfish (Carassius auratus). COMPARATIVE BIOCHEMISTRY AND PHYSIOLOGY C-TOXICOLOGY \& PHARMACOLOGY. 2008;148(4):355-362.

6320. PerezPinzon MA, Mumford PL, Rosenthal M, Sick TJ Anoxic preconditioning in hippocampal slices: Role of adenosine. NEUROSCIENCE. 1996;75(3):687-694.

6321. Krumschnabel G, Frischmann ME, Schwarzbaum PJ, Wieser W Loss of K+ homeostasis in trout hepatocytes during chemical anoxia: A screening study for potential causes and mechanisms. ARCHIVES OF BIOCHEMISTRY AND BIOPHYSICS. 1998;353(2):199-206.

6322. Meyer C, Blissett J, Oldfield C Sexual orientation and eating psychopathology: The role of masculinity and femininity. INTERNATIONAL JOURNAL OF EATING DISORDERS. 2001;29(3):314-318.

6323. Schuen JN, Bamford OS, Carroll JL The cardiorespiratory response to anoxia: normal development and the effect of nicotine. RESPIRATION PHYSIOLOGY. 1997;109(3):231-239.

6324. Lee An-Chin, Lee Yu-Ching, Chin Tzong-Shean Effects of low dissolved oxygen on the digging behaviour and metabolism of the hard clam (Meretrix lusoria). AQUACULTURE RESEARCH. 2012;43(1):1-13.

6325. GREIST JH THE DIAGNOSIS OF SOCIAL PHOBIA. JOURNAL OF CLINICAL PSYCHIATRY. 1995;56(5):5-12.

6326. Leonidas Carolina, Santos Manoel Significant Social Nets of Women with Eating Disorders. PSICOLOGIA-REFLEXAO E CRITICA. 2013;26(3):561-571.

6327. Day Alice, Yao Chu, Costello Samuel, Andrews Jane, Bryant Robert Food-related quality of life in adults with inflammatory bowel disease is associated with restrictive eating behaviour, disease activity and surgery: A prospective multicentre observational study. JOURNAL OF HUMAN NUTRITION AND DIETETICS. 2022;35(1):234-244.

6328. Peckmezian Tina, Paxton Susan A systematic review of outcomes following residential treatment for eating disorders. EUROPEAN EATING DISORDERS REVIEW. 2020;28(3):246-259.

6329. English L., Masterson T., Fearnbach S., Tanofsky-Kraff M., Fisher J., Wilson S., Rolls B., Keller K. Increased brain and behavioural susceptibility to portion size in children with loss of control eating. PEDIATRIC OBESITY. 2019;14(2, SI):.

6330. Feng Tingting, Abebe Dawit Eating behaviour disorders among adolescents in a middle school in Dongfanghong, China. JOURNAL OF EATING DISORDERS. 2017;5():.

6331. Uauy R, Solomons N Diet, nutrition, and the life-course approach to cancer prevention. JOURNAL OF NUTRITION. 2005;135(12, S):2934S-2945S.

6332. Connan Frances, Troop Nick, Landau Sabine, Campbell Iain, Treasure Janet Poor social comparison and the tendency to submissive behavior in anorexia nervosa. INTERNATIONAL JOURNAL OF EATING DISORDERS. 2007;40(8):733-739.

6333. Leon M., Diaz J., Ruiz E. A pilot study of the clinical and statistical significance of a program to reduce eating disorder risk factors in children. EATING AND WEIGHT DISORDERS-STUDIES ON ANOREXIA BULIMIA AND OBESITY. 2008;13(3):111-118.

6334. Kozlowski Jessica, Kits K., Stein Lisa Comparison of Nitrogen Oxide Metabolism among Diverse Ammonia-Oxidizing Bacteria. FRONTIERS IN MICROBIOLOGY. 2016;7():.

6335. Weilinger Nicholas, Tang Peter, Thompson Roger Anoxia-Induced NMDA Receptor Activation Opens Pannexin Channels via Src Family Kinases. JOURNAL OF NEUROSCIENCE. 2012;32(36):12579-12588.

6336. De Souza Mary, Nattiv Aurelia, Joy Elizabeth, Misra Madhusmita, Williams Nancy, Mallinson Rebecca, Gibbs Jenna, Olmsted Marion, Goolsby Marci, Matheson Gordon 2014 Female Athlete Triad Coalition Consensus Statement on Treatment and Return to Play of the Female Athlete Triad: 1st International Conference Held in San Francisco, CA, May 2012, and 2nd International Conference Held in Indianapolis, IN, May 2013. CLINICAL JOURNAL OF SPORT MEDICINE. 2014;24(2):96-119.

6337. Loosli AR, Ruud JS Meatless diets in female athletes: A red flag. PHYSICIAN AND SPORTSMEDICINE. 1998;26(11):45-55.

6338. Liu Yijun, Zeng Yuhan, Liu Yixin, Wang Xiaoya, Chen Yuhuan, Lepp Dion, Tsao Rong, Sadakiyo Tsuyoshi, Zhang Hua, Mine Yoshinori Regulatory Effect of Isomaltodextrin on a High-Fat Diet Mouse Model with LPS-Induced Low-Grade Chronic Inflammation. JOURNAL OF AGRICULTURAL AND FOOD CHEMISTRY. 2022;70(36):11258-11273.

6339. Perdomo Carolina, D'Ingianna Paola, Escalada Javier, Petta Salvatore, Romero-Gomez Manuel, Ampuero Javier Nonalcoholic fatty liver disease and the risk of metabolic comorbidities: how to manage in clinical practice. POLISH ARCHIVES OF INTERNAL MEDICINE-POLSKIE ARCHIWUM MEDYCYNY WEWNETRZNEJ. 2020;130(11):975-985.

6340. Iordachescu Andreea, Cirstoiu Monica, Zugravu Corina-Aurelia, Teodor Oana, Turcan Natalia, Ducu Ionita, Bohiltea Roxana Dietary behavior during pregnancy. EXPERIMENTAL AND THERAPEUTIC MEDICINE. 2020;20(3):2460-2464.

6341. Steinhausen Hans-Christoph What happens to children and adolescents with mental disorders? Findings from long-term outcome research. ZEITSCHRIFT FUR KINDER-UND JUGENDPSYCHIATRIE UND PSYCHOTHERAPIE. 2013;41(6):419-431.

6342. Georgel Philippe, Georgel Philippe Where Epigenetics Meets Food Intake: Their Interaction in the Development/Severity of Gout and Therapeutic Perspectives. FRONTIERS IN IMMUNOLOGY. 2021;12():.

6343. deZwaan M, Karwautz A, Strnad A Treatment of eating disorders - Review of findings from controlled psychotherapy and pharmacotherapy studies. PSYCHOTHERAPEUT. 1996;41(5):275-287.

6344. EAGLES JM, JOHNSTON MI, HUNTER D, LOBBAN M, MILLAR HR INCREASING INCIDENCE OF ANOREXIA-NERVOSA IN THE FEMALE-POPULATION OF NORTHEAST

SCOTLAND. AMERICAN JOURNAL OF PSYCHIATRY. 1995;152(9):1266-1271.

6345. Osa Maggie, Kelly Nichole Experiences of Discrimination Are Associated With Drive for Muscularity Among African American Men. PSYCHOLOGY OF MEN \& MASCULINITIES. 2021;22(2):365-374.

6346. {[}Anonymous] The female athlete triad. MEDICINE AND SCIENCE IN SPORTS AND EXERCISE. 2007;39(10):1867-1882.

6347. Doke Mayur, Avecilla Vincent, Felty Quentin Inhibitor of Differentiation-3 and Estrogenic Endocrine Disruptors: Implications for Susceptibility to Obesity and Metabolic Disorders. BIOMED RESEARCH INTERNATIONAL. 2018;2018():.

6348. Hu Junwei, Li Yi, Wang Zheng, Li Xin, Hou Tianbo, Ning Zibo, Huang Runnian, Ma Chunhua, Yuan Xiaoyue, Wang Difei Association of plant-based dietary patterns with the risk of osteoporosis in community-dwelling adults over 60 years: a cross-sectional study. OSTEOPOROSIS INTERNATIONAL. 2023;():.

6349. Bai Yang, Yang Yanli, Cui Boqun, Lin Duomao, Wang Zhaoqi, Ma Jun Temporal effect of melatonin posttreatment on anoxia/reoxygenation injury in H9c2 cells. CELL BIOLOGY INTERNATIONAL. 2022;46(4):637-648.

6350. ROSE UM, ABRAHAMSE SL, BINDELS RJM, VANOS CH CELLULAR ACIDIFICATION OCCURS DURING ANOXIA IN CULTURED, BUT NOT IN FRESHLY ISOLATED, RABBIT PROXIMAL TUBULAR CELLS. PFLUGERS ARCHIV-EUROPEAN JOURNAL OF PHYSIOLOGY. 1995;429(5):722-728.

6351. Hood A., Wallace M., Reed C., Hoffmann K., Freyer E. Enigmatic carbonates of the Ombombo Subgroup, Otavi Fold Belt, Namibia: A prelude to extreme Cryogenian anoxia?. SEDIMENTARY GEOLOGY. 2015;324():12-31.

6352. Calderon C., Forns M., Varea V. IMPLICATION OF THE ANXIETY AND DEPRESSION IN EATING DISORDERS OF YOUNG OBESE. NUTRICION HOSPITALARIA. 2010;25(4):641-647.

6353. Greco Giulia, Zeppa Sabrina, Agostini Deborah, Attisani Giuseppe, Stefanelli Claudio, Ferrini Fabio, Sestili Piero, Fimognari Carmela The Anti- and Pro-Tumorigenic Role of

Microbiota and Its Role in Anticancer Therapeutic Strategies. CANCERS. 2023;15(1):.

6354. CARACENI P, BORLE AB, GASBARRINI A, FAGIUOLI S, VANTHIEL DH ANOXIA-REOXYGENATION INJURY AND ITS PREVENTION IN PERFUSED RAT HEPATOCYTES. GASTROENTEROLOGY. 1994;106(4, S):A871.

6355. Krause A., Nowak Z., Srbu R., Bell H. Respiratory autoresuscitation following severe acute hypoxemia in anesthetized adult rats. RESPIRATORY PHYSIOLOGY \& NEUROBIOLOGY. 2016;232():43-53.

6356. Lopez-de-Andres Ana, Carrasco-Garrido Pilar, Hernandez-Barrera Valentin, Gil-de-Miguel Angel, Jimenez-Trujillo Isabel, Jimenez-Garcia Rodrigo Hospitalization trends in Spanish children and adolescents with eating disorders (1998-2007). APPETITE. 2010;55(1):147-151.

6357. Ji BT, Chow WH, Yang G, McLaughlin JK, Zheng W, Shu XO, Jin F, Gao RN, Gao YT, Fraumeni JF Dietary habits and stomach cancer in Shanghai, China. INTERNATIONAL JOURNAL OF CANCER. 1998;76(5):659-664.

6358. Sun Mei, Ma Ming Childhood gastrointestinal dysfunction and protection mechanism of intestinal mucosal barrier. WORLD JOURNAL OF PEDIATRICS. 2007;3(1):30-35.

6359. Josephs Keith, Whitwell Jennifer, Parisi Joseph, Lapid Maria Coprophagia in neurologic disorders. JOURNAL OF NEUROLOGY. 2016;263(5):1008-1014.

6360. Kesika Periyanaina, Sivamaruthi Bhagavathi, Chaiyasut Chaiyavat Health promoting effects of fermented foods against cancer: an updated concise review. FOOD SCIENCE AND TECHNOLOGY. 2022;42():.

6361. Swed-Tobia Rana, Haj Ahmad, Militianu Daniella, Eshach Orly, Ravid Sarit, Weiss Ram, Aviel Yonatan Highly Selective Eating in Autism Spectrum Disorder Leading to Scurvy: A Series of Three Patients. PEDIATRIC NEUROLOGY. 2019;94():61-63.

6362. Eleftheriadis Theodoros, Pissas Georgios, Liakopoulos Vassilios, Stefanidis Ioannis Factors that May Protect the Native Hibernator Syrian Hamster Renal Tubular Epithelial Cells from Ferroptosis Due to Warm Anoxia-Reoxygenation. BIOLOGY-BASEL. 2019;8(2):.

6363. Buck LT, Bickler PE Adenosine and anoxia reduce N-methyl-D-aspartate receptor open probability in turtle cerebrocortex. JOURNAL OF EXPERIMENTAL BIOLOGY. 1998;201(2):289-297.

6364. Cureau F., Sparrenberger K., Bloch K., Ekelund U., Schaan B. Associations of multiple unhealthy lifestyle behaviors with overweight/obesity and abdominal obesity among Brazilian adolescents: A country-wide survey. NUTRITION METABOLISM AND CARDIOVASCULAR DISEASES. 2018;28(7):765-774.

6365. Vandevijvere Stefanie, Swinburn Boyd, Obesity Int Towards global benchmarking of food environments and policies to reduce obesity and diet-related non-communicable diseases: design and methods for nation-wide surveys. BMJ OPEN. 2014;4(5):.

6366. Pirkkalainen Hertta, Dopfer Dorte, Soveri Timo, Kujala-Wirth Minna Comparison of ozonated water and acidified copper sulphate in prevention of digital dermatitis in dairy cows. ACTA VETERINARIA SCANDINAVICA. 2022;64(1):.

6367. Tonshin A., Lobysheva N., Yaguzhinsky L. The interrelation of specific changes in mitochondrial membranes permeability and internuclearsomal DNA fragmentation in heart tissue incubated under anoxia conditions. BIOLOGICHESKIE MEMBRANY. 2006;23(5):394-401.

6368. Mendes Clarisse, Miranda Luiza, Claro Rafael, Horta Paula Food marketing in supermarket circulars in Brazil: An obstacle to healthy eating. PREVENTIVE MEDICINE REPORTS. 2021;21():.

6369. Stice E, Tristan J Sociocultural pressures and body image disturbances: A comment on Cafri, Yamamiya, Brannick, and Thompson. CLINICAL PSYCHOLOGY-SCIENCE AND PRACTICE. 2005;12(4):443-446.

6370. Leocadi Michela, Canu Elisa, Cividini Camilla, Russo Tommaso, Cecchetti Giordano, Celico Claudia, Cardamone Rosalinda, Barcella Valeria, Magnani Giuseppe, Agosta Federica, Filippi Massimo Brain structural abnormalities and cognitive changes in a patient with 17q21.31 microduplication and early onset dementia: a case report. JOURNAL OF NEUROLOGY. 2023;270(2):1127-1134.

6371. Liao ZH, Brar BK, Cai Q, Stephanou A, O'Leary RM, Pennica D, Yellon DM, Latchman DS Cardiotrophin-1 (CT-1) can protect the adult heart from injury when added both prior to

ischaemia and at reperfusion. CARDIOVASCULAR RESEARCH. 2002;53(4):902-910.

6372. Roberts Karyn, Ariza Adolfo, Selvaraj Kavitha, Quadri Maheen, Mangarelli Caren, Neault Sarah, Davis Erica, Binns Helen Testing for rare genetic causes of obesity: findings and experiences from a pediatric weight management program. INTERNATIONAL JOURNAL OF OBESITY. 2022;46(8):1493-1501.

6373. Robinson Rachel, Lahti-Pulkkinen Marius, Schnitzlein Daniel, Voit Falk, Girchenko Polina, Wolke Dieter, Lemola Sakari, Kajantie Eero, Heinonen Kati, Raikkonen Katri Mental health outcomes of adults born very preterm or with very low birth weight: A systematic review. SEMINARS IN FETAL \& NEONATAL MEDICINE. 2020;25(3):.

6374. Abaj Faezeh, Koohdani Fariba, Rafiee Masoumeh, Alvandi Ehsan, Yekaninejad Mir, Mirzaei Khadijeh Interactions between Caveolin-1 (rs3807992) polymorphism and major dietary patterns on cardio-metabolic risk factors among obese and overweight women. BMC ENDOCRINE DISORDERS. 2021;21(1):.

6375. Niedziela M., Sippell W. Growth Impairment in a Boy with Late-Onset Congenital Adrenal Hyperplasia and Anorexia Nervosa. EXPERIMENTAL AND CLINICAL ENDOCRINOLOGY \& DIABETES. 2010;118(3):180-183.

6376. Areli Medina-Tepal Karla, Vazquez-Arevalo Rosalia, Maria Trujillo-ChiVacuan Eva, Zickgraf Hana, Manuel Mancilla-Diaz Juan Cross-cultural adaptation and validation of the Nine Item ARFID Screen (NIAS) in Mexican youths. INTERNATIONAL JOURNAL OF EATING DISORDERS. 2023;56(4, SI):721-726.

6377. Pratap Akshay, Saha Gauri, Bhattarai Bal, Yadav Rohit, Nepal Ajit, Bajracharya Amir, Kumar Anand, Adhikary Shailesh Tracheal agenesis type B: further evidence to a lethal congenital tracheal malformation. JOURNAL OF PEDIATRIC SURGERY. 2007;42(7):1284-1287.

6378. Idier L., Decamps G., Rascle N., Koleck M. Comparative study of attraction, incidence and intensity of male and female student's addictive behaviors. ANNALES MEDICO-PSYCHOLOGIQUES. 2011;169(8):517-522.

6379. Ivezaj Valentina, Saules Karen, Schuh Leslie New-Onset Substance Use Disorder After Gastric Bypass Surgery: Rates and Associated Characteristics. OBESITY SURGERY.

2014;24(11):1975-1980.

6380. Ortega Miguel, Fraile-Martinez Oscar, Garcia-Montero Cielo, Alvarez-Mon Miguel, Lahera Guillermo, Monserrat Jorge, Llavero-Valero Maria, Mora Fernando, Rodriguez-Jimenez Roberto, Fernandez-Rojo Sonia, Quintero Javier, Alvarez De Mon Melchor Nutrition, Epigenetics, and Major Depressive Disorder: Understanding the Connection. FRONTIERS IN NUTRITION. 2022;9():.

6381. Bear Tracey, Dalziel Julie, Coad Jane, Roy Nicole, Butts Christine, Gopal Pramod The Role of the Gut Microbiota in Dietary Interventions for Depression and Anxiety. ADVANCES IN NUTRITION. 2020;11(4):890-907.

6382. Iqbal Romaina, Anand Sonia, Ounpuu Stephanie, Islam Shofiqul, Zhang Xiaohe, Rangarajan Sumathy, Chifamba Jephat, Al-Hinai Ali, Keltai Matyas, Yusuf Salim, Investigat Behalf Dietary Patterns and the Risk of Acute Myocardial Infarction in 52 Countries Results of the INTERHEART Study. CIRCULATION. 2008;118(19):1929-1937.

6383. Soldatov A., Andreenko T., Sysoeva I., Sysoev A. Tissue specificity of metabolism in the bivalve mollusc Anadara inaequivalvis Br. under conditions of experimental anoxia. JOURNAL OF EVOLUTIONARY BIOCHEMISTRY AND PHYSIOLOGY. 2009;45(3):349-355.

6384. Yoshimasu Kouichi, Barbaresi William, Colligan Robert, Voigt Robert, Killian Jill, Weaver Amy, Katusic Slavica Childhood ADHD is strongly associated with a broad range of psychiatric disorders during adolescence: a population-based birth cohort study. JOURNAL OF CHILD PSYCHOLOGY AND PSYCHIATRY. 2012;53(10):1036-1043.

6385. DeLong Amy, Larson Nicole, Story Mary, Neumark-Sztainer Dianne, Weber-Main Anne, Ireland Marjorie FACTORS ASSOCIATED WITH OVERWEIGHT AMONG URBAN AMERICAN INDIAN ADOLESCENTS: FINDINGS FROM PROJECT EAT. ETHNICITY \& DISEASE. 2008;18(3):317-323.

6386. Zuba Anna, Warschburger Petra The role of weight teasing and weight bias internalization in psychological functioning: a prospective study among school-aged children. EUROPEAN CHILD \& ADOLESCENT PSYCHIATRY. 2017;26(10):1245-1255.

6387. KOMUNE S, NAKAGAWA T, HISASHI K, KIMITSUKI T, UEMURA T MECHANISM OF LACK OF DEVELOPMENT OF NEGATIVE ENDOCOCHLEAR POTENTIAL IN GUINEA-PIGS

WITH HAIR CELL LOSS. HEARING RESEARCH. 1993;70(2):197-204.

6388. Funes Diaz Francisco, Gaete Pinto Veronica Risk behaviors among adolescents with complex diseases. REVISTA MEDICA DE CHILE. 2016;144(6):734-742.

6389. Kucukerdonmez Ozge, Akder Rana, Seckiner Selda, Oksel Esra, Akpinar Serife, Koksal Eda Turkish version of the `Three-Factor Eating Questionnaire-51' for obese individuals: a validity and reliability study. PUBLIC HEALTH NUTRITION. 2021;24(11):3269-3275.

6390. Kroshus Emily, Fischer Anastasia, Nichols Jeanne Assessing the Awareness and Behaviors of US High School Nurses With Respect to the Female Athlete Triad. JOURNAL OF SCHOOL NURSING. 2015;31(4):272-279.

6391. Dooley Jennifer, Deshpande Sameer, Adair Carol Comparing adolescent-focused obesity prevention and reduction messages. JOURNAL OF BUSINESS RESEARCH. 2010;63(2, SI):154-160.

6392. Pignon Baptiste, Rolland Benjamin, Tebeka Sarah, Zouitina-Lietaert Nadia, Cottencin Olivier, Vaiva Guillaume Clinical criteria of involuntary psychiatric treatment: A literature review and a synthesis of recommendations. PRESSE MEDICALE. 2014;43(11):1195-1205.

6393. Brenner Ronald, Madhusoodanan Subramoniam, Puttichanda Sharath, Chandra Prakash Primary prevention in psychiatry-adult populations. ANNALS OF CLINICAL PSYCHIATRY. 2010;22(4):239-248.

6394. Vargas M, Osorio J, Jimenez D, Moraga F, Sepulveda M, Del Solar J, Hudson C, Cortes G, Leon A Acute mountain sickness at 3500 and 4250 m. A study of symptom Incidence and severity. REVISTA MEDICA DE CHILE. 2001;129(2):166-172.

6395. Panagiotakos Demosthenes, Georgousopoulou Ekavi, Notara Venetia, Pitaraki Evangelia, Kokkou Eleni, Chrysohoou Christina, Skoumas Yannis, Metaxa Vassiliki, Pitsavos Christos, Stefanadis Christodoulos, Grp ATTICA Education status determines 10-year (2002-2012) survival from cardiovascular disease in Athens metropolitan area: the ATTICA study, Greece. HEALTH \& SOCIAL CARE IN THE COMMUNITY. 2016;24(3):334-344.

6396. Liu Binbin, Zhang Xiaojun, Bakken Lars, Snipen Lars, Frostegard Asa Rapid Succession of Actively Transcribing Denitrifier Populations in Agricultural Soil During an Anoxic Spell.

FRONTIERS IN MICROBIOLOGY. 2019;9():.

6397. Pugliese Gabriella, Barrea Luigi, Laudisio Daniela, Aprano Sara, Castellucci Bianca, Framondi Lydia, Di Matteo Rossana, Savastano Silvia, Colao Annamaria, Muscogiuri Giovanna Mediterranean diet as tool to manage obesity in menopause: A narrative review. NUTRITION. 2020;79-80():.

6398. Kolahdooz Fariba, Pakseresht Mohammadreza, Mead Erin, Beck Lindsay, Corriveau Andre, Sharma Sangita Impact of the Healthy Foods North nutrition intervention program on Inuit and Inuvialuit food consumption and preparation methods in Canadian Arctic communities. NUTRITION JOURNAL. 2014;13():.

6399. Lu Lientra, Strauss Michael, Miller Stuart The Gradient Perfusion Model Part 3:An extraordinary case of decompression sickness. UNDERSEA AND HYPERBARIC MEDICINE. 2018;45(3):307-311.

6400. Bahat-Stroomza Merav, Barhum Yael, Levy Yossef, Karpov Olga, Bulvik Shlomo, Melamed Eldad, Offen Daniel Induction of Adult Human Bone Marrow Mesenchymal Stromal Cells into Functional Astrocyte-Like Cells: Potential for Restorative Treatment in Parkinson's Disease. JOURNAL OF MOLECULAR NEUROSCIENCE. 2009;39(1-2):199-210.

6401. Gunay-Aygun M, Schwartz S, Heeger S, O'Riordan MA, Cassidy SB The changing purpose of Prader-Willi syndrome clinical diagnostic criteria and proposed revised criteria. PEDIATRICS. 2001;108(5):art. no.-e92.

6402. Shreiner Andrew, Huffnagle Gary, Noverr Mairi The ``Microflora Hypothesis{''} of allergic disease. . 2008;635():113-134.

6403. Verduci E., Martelli A., Miniello V., Landi M., Mariani B., Brambilla M., Diaferio L., Peroni D. Nutrition in the first 1000 days and respiratory health: A descriptive review of the last five years' literature. ALLERGOLOGIA ET IMMUNOPATHOLOGIA. 2017;45(4):405-413.

6404. Shahsanai Armindokht, Bahreynian Maryam, Fallah Zahra, Hovsepian Silva, Kelishadi Roya Perceived barriers to healthy lifestyle from the parental perspective of overweight and obese students. JOURNAL OF EDUCATION AND HEALTH PROMOTION. 2019;8(1):.

6405. Becker Inga, Nieder Timo, Cerwenka Susanne, Briken Peer, Kreukels Baudewijntje, Cohen-Kettenis Peggy, Cuypere GrietDe, Haraldsen Ira, Richter-Appelt Hertha Body Image in Young Gender Dysphoric Adults: A European Multi-Center Study. ARCHIVES OF SEXUAL BEHAVIOR. 2016;45(3):559-574.

6406. Barrett Joanne, Slatter Georgina, Whitehouse Joanna, Nash Edward Perception, experience and relationship with food and eating in adults with cystic fibrosis. JOURNAL OF HUMAN NUTRITION AND DIETETICS. 2022;35(5):757-764.

6407. Youssef Lara, Hallit Rabih, Akel Marwan, Kheir Nelly, Obeid Sahar, Hallit Souheil Social media use disorder and alexithymia: Any association between the two? Results of a cross-sectional study among Lebanese adults. PERSPECTIVES IN PSYCHIATRIC CARE. 2021;57(1):20-26.

6408. Nolen Erin, Panisch Lisa The Relationship between Body Appreciation and Health Behaviors among Women and Adolescent Girls: A Scoping Review. HEALTH \& SOCIAL WORK. 2022;47(2):113-122.

6409. Bentz Mette, Jepsen Jens, Pedersen Tine, Bulik Cynthia, Pedersen Lennart, Pagsberg Anne, Plessen Kerstin Impairment of Social Function in Young Females With Recent-Onset Anorexia Nervosa and Recovered Individuals. JOURNAL OF ADOLESCENT HEALTH. 2017;60(1):23-32.

6410. Manios Yannis, Kourlaba Georgia, Grammatikaki Evangelia, Androutsos Odysseas, Moschonis George, Roma-Giannikou Eleytheria Development of a diet-lifestyle quality index for young children and its relation to obesity: the Preschoolers Diet-Lifestyle Index. PUBLIC HEALTH NUTRITION. 2010;13(12):2000-2009.

6411. Burkart Sarah, Parker Hannah, Weaver R., Beets Michael, Jones Alexis, Adams Elizabeth, Chaput Jean-Philippe, Armstrong Bridget Impact of the COVID-19 pandemic on elementary schoolers' physical activity, sleep, screen time and diet: A quasi-experimental interrupted time series study. PEDIATRIC OBESITY. 2022;17(1):.

6412. Atkinson Melissa, Wade Tracey Mindfulness-based prevention for eating disorders: A school-based cluster randomized controlled study. INTERNATIONAL JOURNAL OF EATING DISORDERS. 2015;48(7):1024-1037.

6413. Santos MA, Pacheco M Anguilla anguilla L stress biomarkers recovery in clean water and secondary-treated pulp mill effluent. ECOTOXICOLOGY AND ENVIRONMENTAL SAFETY. 1996;35(1):96-100.

6414. Mohammadi Mohammad, Pourdehghan Parandis, Mostafavi Seyed-Ali, Hooshyari Zahra, Ahmadi Nastaran, Khaleghi Ali Generalized anxiety disorder: Prevalence, predictors, and comorbidity in children and adolescents. JOURNAL OF ANXIETY DISORDERS. 2020;73():.

6415. Acikgoz Ayla, Dayi Ayfer, Binbay Tolga Prevalence of depression among female university students and associated factors. CUKUROVA MEDICAL JOURNAL. 2018;43(1):131-140.

6416. Nsamenang Sheri, Gutierrez Carline, Jones Jane, Jenkins Glenn, Tibelius Stephanie, DiGravio Anna, Chamas Basma, Ewusie Joycelyne, Geddie Hannah, Punthakee Zubin, Samaan M., Wahi Gita, Morrison Katherine The effects of the COVID-19 pandemic on the mental and physical health of children attending a paediatric weight management clinic. PAEDIATRICS \& CHILD HEALTH. 2022;27(2, SI):S158-S164.

6417. Chiba Fernando, Sumida Doris, Moimaz Suzely, Chaves Neto Antonio, Nakamune Ana, Garbin Artenio, Garbin Clea Periodontal condition, changes in salivary biochemical parameters, and oral health-related quality of life in patients with anorexia and bulimia nervosa. JOURNAL OF PERIODONTOLOGY. 2019;90(12):1423-1430.

6418. Christensen Kara, Forbush Kelsie, Elliott Brendon, Jarmolowicz David A single-case multiple baseline design for treating insomnia in eating disorders: The TIRED study. INTERNATIONAL JOURNAL OF EATING DISORDERS. 2021;54(4):652-659.

6419. Voorend Carlijn, Norris Shane, Griffiths Paula, Sedibe Modiehi, Westerman Marjan, Doak Colleen `We eat together; today she buys, tomorrow I will buy the food': adolescent best friends' food choices and dietary practices in Soweto, South Africa. PUBLIC HEALTH NUTRITION. 2013;16(3):559-567.

6420. Meister Benjamin, Hong Soon-Gook, Shin Junchul, Rath Meghan, Sayoc Jacqueline, Park Joon-Young Healthy versus Unhealthy Adipose Tissue Expansion: the Role of Exercise. JOURNAL OF OBESITY \& METABOLIC SYNDROME. 2022;31(1):37-50.

6421. CARACENI P, GASBARRINI A, NUSSLER A, DISILVIO M, BARTOLI F, BORLE AB, VANTHIEL DH HUMAN HEPATOCYTES ARE MORE RESISTANT THAN RAT HEPATOCYTES TO ANOXIA-REOXYGENATION INJURY. HEPATOLOGY. 1994;20(5):1247-1254.

6422. JONSON ACJ, NILSSON DE EFFECTS OF ENERGY DEPRIVATION ON THE FLY PUPIL MECHANISM - EVIDENCE FOR A RIGOR STATE. JOURNAL OF COMPARATIVE PHYSIOLOGY A-SENSORY NEURAL AND BEHAVIORAL PHYSIOLOGY. 1994;174(6):701-706.

6423. Chirkova TV, Novitskaya LO, Blokhina OB Lipid peroxidation and antioxidant systems under anoxia in plants differing in their tolerance to oxygen deficiency. RUSSIAN JOURNAL OF PLANT PHYSIOLOGY. 1998;45(1):55-62.

6424. REAVIS PA, ESPTEIN BA, PIOTROWICZ LM PARENTS GUIDE TO EATING DISORDERS - PREVENTION AND TREATMENT OF ANOREXIA AND BULIMIA - VALETTE,B. PSYCHIATRIC SERVICES. 1995;46(12):1299.

6425. Favaro Angela, Tenconi Elena, Degortes Daniela, Soave Manuela, Zanetti Tatiana, Nardi Maria, Caregaro Lorenza, Santonastaso Paolo Association between low height and eating disorders: Cause or effect?. INTERNATIONAL JOURNAL OF EATING DISORDERS. 2007;40(6):549-553.

6426. Murphy Mark, Dempsey Philip, Gillespie Ciara, Murphy Alexandra, McNicholas Michelle Increased incidence of acute calculous cholecystitis observed during COVID-19 social restrictions. IRISH JOURNAL OF MEDICAL SCIENCE. 2022;191(1):229-232.

6427. Castro J., Gea G., Quijano M., Aguado R., Froehner S., Naafs B., Pancost R. Complex and protracted environmental and ecological perturbations during OAE 1a-Evidence from an expanded pelagic section from south Spain (Western Tethys). GLOBAL AND PLANETARY CHANGE. 2019;183():.

6428. Chamberland Valerie, Rioux Pierre Not only students can express alcohol dehydrogenase: goldfish can too!. ADVANCES IN PHYSIOLOGY EDUCATION. 2010;34(4):222-227.

6429. Lehning EJ, Doshi R, Isaksson N, Stys PK, LoPachin RM Mechanisms of injury-induced calcium entry into peripheral nerve myelinated axons: Role of reverse sodium-calcium exchange. JOURNAL OF NEUROCHEMISTRY. 1996;66(2):493-500.

6430. Jimenez Amanda, Hollanda Ana, Palou Eva, Ortega Emilio, Andreu Alba, Molero Judit, Mestre Carla, Ibarzabal Ainitze, Obach Amadeu, Flores Lilliam, Canizares Silvia, Balibrea Jose, Vidal Josep, Escarrabill Joan, Moize Violeta Psychosocial, Lifestyle, and Body Weight Impact of COVID-19-Related Lockdown in a Sample of Participants with Current or Past History of Obesity in Spain. OBESITY SURGERY. 2021;31(5):2115-2124.

6431. Fava M, Abraham M, Alpert J, Nierenberg AA, Pava JA, Rosenbaum JF Gender differences in Axis I comorbidity among depressed outpatients. JOURNAL OF AFFECTIVE DISORDERS. 1996;38(2-3):129-133.

6432. Siipola Virpi, Lehtimaki Maria, Tallberg Petra The effects of anoxia on Si dynamics in sediments. JOURNAL OF SOILS AND SEDIMENTS. 2016;16(1):266-279.

6433. Stys PK, Jiang QB Calpain-dependent neurofilament breakdown in anoxic and ischemic rat central axons. NEUROSCIENCE LETTERS. 2002;328(2):150-154.

6434. Zhao Yu, Tian Xuefeng, Liu Gengfeng, Wang Kuijing, Xie Yuanyuan, Qiu Yuxuan Berberine protects myocardial cells against anoxia-reoxygenation injury via p38 MAPK-mediated NF-B signaling pathways. EXPERIMENTAL AND THERAPEUTIC MEDICINE. 2019;17(1):230-236.

6435. Kristensen E, Kristiansen KD, Jensen MH Temporal behavior of manganese and iron in a sandy coastal sediment exposed to water column anoxia. ESTUARIES. 2003;26(3):690-699.

6436. Willmore WG, Storey KB Glutathione systems and anoxia tolerance in turtles. AMERICAN JOURNAL OF PHYSIOLOGY-REGULATORY INTEGRATIVE AND COMPARATIVE PHYSIOLOGY. 1997;273(1):R219-R225.

6437. Schulz Daniela, Kremers Stef, Osch Liesbeth, Schneider Francine, Adrichem Mathieu, Vries Hein Testing a Dutch web-based tailored lifestyle programme among adults: a study protocol. BMC PUBLIC HEALTH. 2011;11():.

6438. Vancells Lujan Pau, Vinas Esmel Esther, Sacanella Meseguer Emilio Overview of Non-Alcoholic Fatty Liver Disease (NAFLD) and the Role of Sugary Food Consumption and Other Dietary Components in Its Development. NUTRIENTS. 2021;13(5):.

6439. Mensorio Marinna, Cebolla-Marti Ausias, Rodilla Enrique, Palomar Gonzalo, Francisco Lison Juan, Rotella Cristina, Fernandez-Aranda Fernando, Jimenez-Murcia Susana, Banos Rosa Analysis of the efficacy of an internet-based self-administered intervention ({''}Living Better{''}) to promote healthy habits in a population with obesity and hypertension: An exploratory randomized controlled trial. INTERNATIONAL JOURNAL OF MEDICAL INFORMATICS. 2019;124():13-23.

6440. Smith Robert, Olin Bernie, Madsen Joseph Spitting into the wind: The irony of treating chronic disease. JOURNAL OF THE AMERICAN PHARMACISTS ASSOCIATION. 2006;46(3):397-400.

6441. FERN R, WAXMAN SG, RANSOM BR MODULATION OF ANOXIC INJURY IN CNS WHITE-MATTER BY ADENOSINE AND INTERACTION BETWEEN ADENOSINE AND GABA. JOURNAL OF NEUROPHYSIOLOGY. 1994;72(6):2609-2616.

6442. Arora AS, deGroen PC, Croall DE, Emori Y, Gores GJ Hepatocellular carcinoma cells resist necrosis during anoxia by preventing phospholipase-mediated calpain activation. JOURNAL OF CELLULAR PHYSIOLOGY. 1996;167(3):434-442.

6443. Mereu Alberta, Fantoni Teresa, Caini Saverio, Monzali Francesca, Roselli Elena, Taddei Silvia, Lucarelli Stefano, Pisano Tiziana Suicidality in adolescents with onset of anorexia nervosa. EATING AND WEIGHT DISORDERS-STUDIES ON ANOREXIA BULIMIA AND OBESITY. 2022;27(7):2447-2457.

6444. Veses A., Martinez-Gomez D., Gomez-Martinez S., Vicente-Rodriguez G., Castillo R., Ortega F., Gonzalez-Gross M., Calle M., Veiga O., Marcos A., Grp AVENA, Grp AFINOS Physical fitness, overweight and the risk of eating disorders in adolescents. The AVENA and AFINOS studies. PEDIATRIC OBESITY. 2014;9(1):1-9.

6445. Bijl RV, Ravelli A, Zessen G Prevalence of psychiatric disorder in the general population: results of the Netherlands Mental Health Survey and Incidence Study (NEMESIS). SOCIAL PSYCHIATRY AND PSYCHIATRIC EPIDEMIOLOGY. 1998;33(12):587-595.

6446. Neade Tina, Uribarri Jaime Diet, inflammation, and chronic kidney disease: Getting to the heart of the matter. SEMINARS IN DIALYSIS. 2008;21(4):331-337.

6447. Edrein Espinosa-Curiel Ismael, Efren Pozas-Bogarin Edgar, Hernandez-Arvizu Maryleidi, Elena Navarro-Jimenez Maria, Emeth Delgado-Perez Edwin, Martinez-Miranda Juan, Perez-Espinosa Humberto HelperFriend, a Serious Game for Promoting Healthy Lifestyle Behaviors in Children: Design and Pilot Study. JMIR SERIOUS GAMES. 2022;10(2):.

6448. Ohlmann A, Giffhorn-Katz S, Becker I, Katz N, Immenschuh S Regulation of heme oxygenase-1 gene expression by anoxia and reoxygenation in primary rat hepatocyte cultures. EXPERIMENTAL BIOLOGY AND MEDICINE. 2003;228(5):584-589.

6449. Nelson Lance, Halpern-Felsher Bonnie, Nagata Jason, Carlson Jennifer Clinician practices assessing hypothalamic-pituitary-gonadal axis suppression in adolescents with an eating disorder. INTERNATIONAL JOURNAL OF EATING DISORDERS. 2021;54(12):2218-2222.

6450. Dosenko VE, Nagibin VS, Moybenko AA Phosphocreatine (neoton) prevents necrotic and apoptotic death of cardiomyocytes in modelling of anoxia-reoxygenation. JOURNAL OF MOLECULAR AND CELLULAR CARDIOLOGY. 2004;36(5):746.

6451. Foucart S, Grondin L, Couture R, Nadeau R Paradoxical action of desipramine on the modulatory effect of bradykinin on noradrenaline release in a model of metabolic anoxia in rat isolated atria. CANADIAN JOURNAL OF PHYSIOLOGY AND PHARMACOLOGY. 1997;75(6):646-651.

6452. Fennig Silvana, Klomek Anat, Shahar Ben, Sarel-Michnik Zohar, Hadas Arie Inpatient treatment has no impact on the core thoughts and perceptions in adolescents with anorexia nervosa. EARLY INTERVENTION IN PSYCHIATRY. 2017;11(3):200-207.

6453. CARTER AJ, MULLER RE, PSCHORN U, STRANSKY W PREINCUBATION WITH CREATINE ENHANCES LEVELS OF CREATINE-PHOSPHATE AND PREVENTS ANOXIC DAMAGE IN RAT HIPPOCAMPAL SLICES. JOURNAL OF NEUROCHEMISTRY. 1995;64(6):2691-2699.

6454. GASBARRINI A, BORLE AB, VANTHIEL DH CA2+ ANTAGONISTS DO NOT PROTECT ISOLATED-PERFUSED RAT HEPATOCYTES FROM ANOXIC INJURY. BIOCHIMICA ET BIOPHYSICA ACTA. 1993;1177(1):1-7.

6455. Wu Tao, Guo Anqi, Shu Qingyu, Qi Yangjian, Kong Ying, Sun Zhiping, Sun Shumin, Fu Zhengwei L-Carnitine intake prevents irregular feeding-induced obesity and lipid

metabolism disorder. GENE. 2015;554(2):148-154.

6456. Forrest Lauren, Bodell Lindsay, Witte Tracy, Goodwin Natalie, Bartlett Mary, Siegfried Nicole, Eddy Kamryn, Thomas Jennifer, Franko Debra, Smith April Associations between eating disorder symptoms and suicidal ideation through thwarted belongingness and perceived burdensomeness among eating disorder patients. JOURNAL OF AFFECTIVE DISORDERS. 2016;195():127-135.

6457. Pamenter Matthew, Shin Damian, Buck Leslie Adenosine A1 receptor activation mediates NMDA receptor activity in a pertussis toxin-sensitive manner during normoxia but not anoxia in turtle cortical neurons. BRAIN RESEARCH. 2008;1213():27-34.

6458. ROSE UM, HARTOG A, JANSEN JWCM, VANOS CH, BINDELS RJM ANOXIA-INDUCED INCREASES IN INTRACELLULAR CALCIUM-CONCENTRATION IN PRIMARY CULTURES OF RABBIT THICK ASCENDING LIMB OF HENLES LOOP. BIOCHIMICA ET BIOPHYSICA ACTA-MOLECULAR BASIS OF DISEASE. 1994;1226(3):291-299.

6459. Strandjord Sarah, Sieke Erin, Richmond Miranda, Khadilkar Arjun, Rome Ellen Medical stabilization of adolescents with nutritional insufficiency: a clinical care path. EATING AND WEIGHT DISORDERS-STUDIES ON ANOREXIA BULIMIA AND OBESITY. 2016;21(3):403-410.

6460. Chen Tong-Jong, Jeng Jing-Yueh, Lin Cheng-Wei, Wu Chin-Yen, Chen Yen-Chou Quercetin inhibition of ROS-dependent and -independent apoptosis in rat glioma C6 cells. TOXICOLOGY. 2006;223(1-2):113-126.

6461. Valladares Macarena, Campos Brianda, Zapata Camila, Duran Aguero Samuel, Maria Obregon Ana Association between chronotype and obesity in young people. NUTRICION HOSPITALARIA. 2016;33(6):1336-1339.

6462. Bonnaire C., Phan O. Negative perceptions of the risks associated with gaming in young adolescents: An exploratory study to help thinking about a prevention program. ARCHIVES DE PEDIATRIE. 2017;24(7):607-617.

6463. Laine Jessica, Huybrechts Inge, Gunter Marc, Ferrari Pietro, Weiderpass Elisabete, Tsilidis Kostas, Aune Dagfinn, Schulze Matthias, Bergmann Manuela, Temme Elisabeth, Boer Jolanda, Agnoli Claudia, Ericson Ulrika, Stubbendorff Anna, Ibsen Daniel, Dahm Christina, Deschasaux Melanie, Touvier Mathilde, Kesse-Guyot Emmanuelle, Perez Maria-Jose,

Barranco Miguel, Tong Tammy, Papier Keren, Knuppel Anika, Boutron-Ruault Marie-Christine, Mancini Francesca, Severi Gianluca, Srour Bernard, Kuhn Tilman, Masala Giovanna, Agudo Antonio, Skeie Guri, Rylander Charlotta, Sandanger Torkjel, Riboli Elio, Vineis Paolo Co-benefits from sustainable dietary shifts for population and environmental health: an assessment from a large European cohort study. LANCET PLANETARY HEALTH. 2021;5(11):E786-E796.

6464. Lemille J., Le Bras M., Fauconnier M., Grall-Bronnec M. Anorexia nervosa: Abnormalities in hematological and biochemical parameters. REVUE DE MEDECINE INTERNE. 2021;42(8):558-565.

6465. Xu PeiHan, Yao YaLi, Guo PengJun, Wang Ting, Yang BingWu, Zhang Zheng Curcumin protects rat heart mitochondria against anoxia-reoxygenation induced oxidative injury. CANADIAN JOURNAL OF PHYSIOLOGY AND PHARMACOLOGY. 2013;91(9):715-723.

6466. Yockey R., King Keith, Vidourek Rebecca Family factors and parental correlates to adolescent conduct disorder. JOURNAL OF FAMILY STUDIES. 2019;():.

6467. Caprara Greta Mediterranean-Type Dietary Pattern and Physical Activity: The Winning Combination to Counteract the Rising Burden of Non-Communicable Diseases (NCDs). NUTRIENTS. 2021;13(2):.

6468. Nagaratnam N, Lewis-Jones M, Scott D, Palazzi L Behavioral and psychiatric manifestations in dementia patients in a community: Caregiver burden and outcome. ALZHEIMER DISEASE \& ASSOCIATED DISORDERS. 1998;12(4):330-334.

6469. Owens Matthew, Watkins Ed, Bot Mariska, Brouwer Ingeborg, Roca Miquel, Kohls Elisabeth, Penninx Brenda, Grootheest Gerard, Hegerl Ulrich, Gili Margalida, Visser Marjolein, Investig MooDFOOD Habitual Behavior as a Mediator Between Food-Related Behavioral Activation and Change in Symptoms of Depression in the MooDFOOD Trial. CLINICAL PSYCHOLOGICAL SCIENCE. 2021;9(4):649-665.

6470. Alegria Katie, Fleszar-Pavlovic Sara, Hua Jacqueline, Loyola Maria, Reuschel Hope, Song Anna How Socioeconomic Status and Acculturation Relate to Dietary Behaviors Within Latino Populations. AMERICAN JOURNAL OF HEALTH PROMOTION. 2022;36(3):450-457.

6471. Schmidt Ulrike Bulimic symptoms and disorders in young people: risk factors, prevention and treatment. EUROPEAN CHILD \& ADOLESCENT PSYCHIATRY.

2015;24(1):S96.

6472. Voelker Dana, Petrie Trent, Huang Qiushi, Chandran Avinash Bodies in Motion: An empirical evaluation of a program to support positive body image in female collegiate athletes. BODY IMAGE. 2019;28():149-158.

6473. Fuchs Klaus, Haldimann Mirella, Grundmann Tobias, Fleisch Elgar Supporting food choices in the Internet of People: Automatic detection of diet-related activities and display of real-time interventions via mixed reality headsets. FUTURE GENERATION COMPUTER SYSTEMS-THE INTERNATIONAL JOURNAL OF ESCIENCE. 2020;113():343-362.

6474. Fararova Adela, Papezova Hana, Gricova Jana, Stepankova Tereza, Capek Vaclav, Reedtz Charlotte, Lauritzen Camilla, Doesum Karin ChildTalks plus : a study protocol of a pre-post controlled, paired design study on the use of preventive intervention for children of parents with a mental illness with focus on eating disorders. BMC PSYCHIATRY. 2022;22(1):.

6475. Saes Mirelle, Neves Rosalia, Machado Karla, Flores Thayna Socioeconomic inequalities in the food consumption of the elderly Brazilian population: National Health Survey, 2019. CIENCIA \& SAUDE COLETIVA. 2022;27(7):2621-2628.

6476. Hart Laura, Damiano Stephanie, Li-Wai-Suen Connie, Paxton Susan Confident body, confident child: Evaluation of a universal parenting resource promoting healthy body image and eating patterns in early childhood-6-and 12-month outcomes from a randomized controlled trial. INTERNATIONAL JOURNAL OF EATING DISORDERS. 2019;52(2):121-131.

6477. Weisman Alanna, Fazli Ghazal, Johns Ashley, Booth Gillian Evolving Trends in the Epidemiology, Risk Factors, and Prevention of Type 2 Diabetes: A Review. CANADIAN JOURNAL OF CARDIOLOGY. 2018;34(5):552-564.

6478. Wanka Heike, Lutze Philipp, Steer Doreen, Bracke Katharina, Golchert Janine, Peters Jorg Angiotensin dependent and angiotensin independent protective effects of renin-b in H9c2 cells after anoxia. SCIENTIFIC REPORTS. 2020;10(1):.

6479. Mulvaney-Day Norah, Womack Catherine Obesity, Identity and Community: Leveraging Social Networks for Behavior Change in Public Health. PUBLIC HEALTH ETHICS. 2009;2(3):250-260.

6480. Richard V, Tamion F, Beauchamp P, Devaux B, Lallemand F, Daveau M, Vaudry H, Lebreton JP, Thuillez C Preconditioning prevents neutrophil adhesion and expression of ICAM-1 after anoxia and reoxygenation in cultured endothelial cells. CIRCULATION. 1996;94(8, S):2848.

6481. TAMION F, RICHARD V, BEAUCHAMP P, DAVEAU M, LEBRETON JP, THUILLEZ C PRECONDITIONING PREVENTS THE EXPRESSION OF INTERLEUKIN-1-BETA INDUCED BY ANOXIA AND REOXYGENATION IN CULTURED ENDOTHELIAL-CELLS. CIRCULATION. 1995;92(8, S):3426.

6482. RUI T, YANG YZ, ZHOU TS, YANG XY, CHEN HZ PROTECTIVE EFFECT OF CAPTOPRIL ON CULTURED RAT MYOCARDIAL-CELLS WITH ANOXIA AND REOXYGENATION INJURY. ACTA PHARMACOLOGICA SINICA. 1994;15(4):375-378.

6483. Borisenkov Mikhail, Popov Sergey, Tserne Tatyana, Bakutova Larisa, Pecherkina Anna, Dorogina Olga, Martinson Ekaterina, Vetosheva Valentina, Gubin Denis, Solovieva Svetlana, Turovinina Elena, Symanyuk Elvira Food addiction and symptoms of depression among inhabitants of the European North of Russia: Associations with sleep characteristics and photoperiod. EUROPEAN EATING DISORDERS REVIEW. 2020;28(3):332-342.

6484. Han Li, Zhang Yuting, Yue Cui, Huang Yiqin, Wu Yumin, Chen Jie Preliminary Study on Risk Factors for Morbidity of Nonalcoholic Fatty Liver Disease in High-Income Male Population. JOURNAL OF HEALTHCARE ENGINEERING. 2022;2022():.

6485. Brukhin A., Onegina E. Affective disorders in patients with anorexia nervosa and bulimia nervosa. ZHURNAL NEVROLOGII I PSIKHIATRII IMENI S S KORSAKOVA. 2011;111(1):21-26.

6486. Carter FA, Bulik CM Cue reactivity and bulimia nervosa: Refining and standardising methodology. BEHAVIOUR CHANGE. 1996;13(2):98-111.

6487. Nerini A., Matera C., Stefanile C. Siblings' appearance-related commentary, body dissatisfaction, and risky eating behaviors in young women. EUROPEAN REVIEW OF APPLIED PSYCHOLOGY-REVUE EUROPEENNE DE PSYCHOLOGIE APPLIQUEE. 2016;66(6):269-276.

6488. Sirri Laura, Garotti Maria, Grandi Silvana, Tossani Eliana Adolescents' hypochondriacal fears and beliefs: Relationship with demographic features, psychological

distress, well-being and health-related behaviors. JOURNAL OF PSYCHOSOMATIC RESEARCH. 2015;79(4):259-264.

6489. Campbell Norm, Duhaney Tara, Arango Manuel, Ashley Lisa, Bacon Simon, Gelfer Mark, Kaczorowski Janusz, Mang Eric, Morris Dorothy, Nagpal Seema, Tsuyuki Ross, Willis Kevin Healthy Food Procurement Policy: An Important Intervention to Aid the Reduction in Chronic Noncommunicable Diseases. CANADIAN JOURNAL OF CARDIOLOGY. 2014;30(11):1456-1459.

6490. Neumann Felix, Jagemann Bettina, Makarova Nataliya, Borschel Christin, Aarabi Ghazal, Gutmann Friederike, Schnabel Renate, Zyriax Birgit-Christiane Mediterranean Diet and Atrial Fibrillation: Lessons Learned from the AFHRI Case-Control Study. NUTRIENTS. 2022;14(17):.

6491. Springett R, Wylezinska M, Cady EB, Cope M, Delpy DT Oxygen dependency of cerebral oxidative phosphorylation in newborn piglets. JOURNAL OF CEREBRAL BLOOD FLOW AND METABOLISM. 2000;20(2):280-289.

6492. Sergi Giuseppe, Bano Giulia, Pizzato Simona, Veronese Nicola, Manzato Enzo Taste loss in the elderly: Possible implications for dietary habits. CRITICAL REVIEWS IN FOOD SCIENCE AND NUTRITION. 2017;57(17):3684-3689.

6493. Morin Jean-Pascal, Rodriguez-Duran Luis, Guzman-Ramos Kioko, Perez-Cruz Claudia, Ferreira Guillaume, Diaz-Cintra Sofia, Pacheco-Lopez Gustavo Palatable Hyper-Caloric Foods Impact on Neuronal Plasticity. FRONTIERS IN BEHAVIORAL NEUROSCIENCE. 2017;11():.

6494. Biederman Joseph, Petty Carter, Monuteaux Michael, Fried Ronna, Byrne Deirdre, Mirto Tara, Spencer Thomas, Wilens Timothy, Faraone Stephen Adult Psychiatric Outcomes of Girls With Attention Deficit Hyperactivity Disorder: 11-Year Follow-Up in a Longitudinal Case-Control Study. AMERICAN JOURNAL OF PSYCHIATRY. 2010;167(4):409-417.

6495. Cuijpers P, Langendoen Y, Bijl RV Psychiatric disorders in adult children of problem drinkers: prevalence, first onset and comparison with other risk factors. ADDICTION. 1999;94(10):1489-1498.

6496. Fredsted A., Gissel H., Ortenblad N., Clausen T. Effects of beta(2)-agonists on force during and following anoxia in rat extensor digitorum longus muscle. JOURNAL OF APPLIED

PHYSIOLOGY. 2012;112(12):2057-2067.

6497. Roux H., Chapelon E., Godart N. Epidemiology of anorexia nervosa: A review. ENCEPHALE-REVUE DE PSYCHIATRIE CLINIQUE BIOLOGIQUE ET THERAPEUTIQUE. 2013;39(2):85-93.

6498. Key Adrienne, O'Brien Aileen, Gordon Isky, Christie Deborah, Lask Bryan Assessment of neurobiology in adults with anorexia nervosa. EUROPEAN EATING DISORDERS REVIEW. 2006;14(5):308-314.

6499. STICE E REVIEW OF THE EVIDENCE FOR A SOCIOCULTURAL MODEL OF BULIMIA-NERVOSA AND AN EXPLORATION OF THE MECHANISMS OF ACTION. CLINICAL PSYCHOLOGY REVIEW. 1994;14(7):633-661.

6500. Nilsen Vegard, Bakke Per, Gallefoss Frode Effects of lifestyle intervention in persons at risk for type 2 diabetes mellitus - results from a randomised, controlled trial. BMC PUBLIC HEALTH. 2011;11():.

6501. DILISA F, BLANK PS, COLONNA R, GAMBASSI G, SILVERMAN HS, STERN MD, HANSFORD RG MITOCHONDRIAL-MEMBRANE POTENTIAL IN SINGLE LIVING ADULT-RAT CARDIAC MYOCYTES EXPOSED TO ANOXIA OR METABOLIC INHIBITION. JOURNAL OF PHYSIOLOGY-LONDON. 1995;486(1):1-13.

6502. Grazioli Valentina, Rossaro Bruno, Parenti Paolo, Giacchini Roberto, Lencioni Valeria Hypoxia and anoxia effects on alcohol dehydrogenase activity and hemoglobin content in Chironomus riparius Meigen, 1804. JOURNAL OF LIMNOLOGY. 2016;75(2):347-354.

6503. Jia Zhenyi, Chen Qian, Qin Huanlong Ischemia-Induced Apoptosis of Intestinal Epithelial Cells Correlates with Altered Integrin Distribution and Disassembly of F-Actin Triggered by Calcium Overload. JOURNAL OF BIOMEDICINE AND BIOTECHNOLOGY. 2012;():.

6504. Katsarou A., Triposkiadis F., Skoularigis J., Papageorgiou C., Panagiotakos D. Evaluating the role of Mediterranean diet and eating behaviors on the likelihood of having a non-fatal acute coronary syndrome, under the context of stress perception: a case-control study. EUROPEAN JOURNAL OF CLINICAL NUTRITION. 2014;68(9):1016-1021.

6505. Austin S. A public health approach to eating disorders prevention: It's time for public health professionals to take a seat at the table. BMC PUBLIC HEALTH. 2012;12():.

6506. Kato-Noguchi H Abscisic acid and hypoxic induction of anoxia tolerance in roots of lettuce seedlings. JOURNAL OF EXPERIMENTAL BOTANY. 2000;51(352):1939-1944.

6507. Griffiths RA, Beumont PJV, Russell J, Touyz SW, Moore G The use of guardianship legislation for anorexia nervosa: a report of 15 cases. AUSTRALIAN AND NEW ZEALAND JOURNAL OF PSYCHIATRY. 1997;31(4):525-531.

6508. Dinparast Fahimeh, Sharifi Akbar, Moradi Sara, Alipour Maedeh, Alipour Beitullah The associations between dietary pattern of chronic obstructive pulmonary disease patients and depression: a cross-sectional study. BMC PULMONARY MEDICINE. 2021;21(1):.

6509. Lingiardi Vittorio, Giovanardi Guido, Fortunato Alexandro, Nassisi Valentina, Speranza Anna Personality and Attachment in Transsexual Adults. ARCHIVES OF SEXUAL BEHAVIOR. 2017;46(5):1313-1323.

6510. Pruneti Carlo, Guidotti Sara, Lento Rene, Renda Nicolo Dissociation between cognitive-behavioral and emotional-psychophysiological aspects in Eating Disorders and its pre-post treatment stability. JOURNAL OF PSYCHOPATHOLOGY. 2022;28(1):30-38.

6511. Al-attar Rasha, Zhang Yichi, Storey Kenneth Osmolyte regulation by TonEBP/NFAT5 during anoxia-recovery and dehydration-rehydration stresses in the freeze-tolerant wood frog (Rana sylvatica). PEERJ. 2017;5():.

6512. Villanueva Sandra, Suazo Cristian, Santapau Daniela, Perez Francisco, Quiroz Mariana, Carreno Juan, Illanes Sebastian, Lavandero Sergio, Michea Luis, Irarrazabal Carlos NFAT5 Is Activated by Hypoxia: Role in Ischemia and Reperfusion in the Rat Kidney. PLOS ONE. 2012;7(7):.

6513. Johnston Olwyn, Startup Helen, Lavender Anna, Godfrey Emma, Schmidt Ulrike Therapeutic Writing as an Intervention for Symptoms of Bulimia Nervosa: Effects and Mechanism of Change. INTERNATIONAL JOURNAL OF EATING DISORDERS. 2010;43(5):405-419.

6514. Nowacka-Chmielewska Marta, Liskiewicz Daniela, Grabowska Konstancja, Liskiewicz Arkadiusz, Marczak Lukasz, Wojakowska Anna, Pondel Natalia, Grabowski Mateusz, Barski Jaroslaw, Malecki Andrzej Effects of Simultaneous Exposure to a Western Diet and Wheel-Running Training on Brain Energy Metabolism in Female Rats. NUTRIENTS. 2021;13(12):.

6515. Hennchen Benjamin What is enough on a plate? Professionals' practices of providing an ``adequate portion{''} in the food service sector. FOOD AND FOODWAYS. 2021;29(4):355-377.

6516. Roy Brita, Stanojevich Joel, Stange Paul, Jiwani Nafisa, King Raymond, Koo Denise Development of the Community Health Improvement Navigator Database of Interventions. MMWR-MORBIDITY AND MORTALITY WEEKLY REPORT. 2016;65(2):.

6517. Sim Leslie, Lebow Jocelyn, Billings Marcie Eating Disorders in Adolescents With a History of Obesity. PEDIATRICS. 2013;132(4):E1026-E1030.

6518. Latner Janet, Mond Jonathan, Vallance Joanna, Gleaves David, Buckett Geoffrey Quality of Life Impairment and the Attitudinal and Behavioral Features of Eating Disorders. JOURNAL OF NERVOUS AND MENTAL DISEASE. 2013;201(7):592-597.

6519. Dambros M, Jongh R, Koeveringe GA, Bast A, Kerrebroeck PEV Galangin protects pig detrusor nerves from repetitive field stimulation and anoxia/glucopenia injury. UROLOGY. 2005;66(6):1327-1331.

6520. Momene Janire, Estevez Ana, Griffiths Mark, Macia Patricia, Herrero Marta, Olave Leticia, Iruarrizaga Itziar Childhood trauma and body dissatisfaction among young adult women: the mediating role of self-criticism. CURRENT PSYCHOLOGY. 2022;():.

6521. Rutsztein Guillermina, Scappatura Maria, Elizathe Luciana, Leonardelli Eduardo, Murawski Brenda, Lievendag Leonora, Sanday Julieta, Falivelli Maria, Bidacovich German, Keegan Eduardo Efficacy of an integrated program (PIA-2) to reduce the risk for problems related to eating, weight and body image in female adolescents from Argentina. INTERNATIONAL JOURNAL OF EATING DISORDERS. 2023;56(4, SI):758-769.

6522. BHATTACHARYYA ML, SARKER S, SETH K DEXTROSE-INDUCED, ADENOSINE-INDUCED AND MAGNESIUM-INDUCED PROTECTIVE ACTIONS DURING ANOXIA AND REPERFUSION IN CANINE PURKINJE TISSUE. CLINICAL AND EXPERIMENTAL

PHARMACOLOGY AND PHYSIOLOGY. 1994;21(8):631-637.

6523. Caporaso Nicola, Morisco Filomena, Camera Silvia, Graziani Giulia, Donnarumma Laura, Ritieni Alberto Dietary approach in the prevention and treatment of NAFLD. FRONTIERS IN BIOSCIENCE-LANDMARK. 2012;17():2259-2268.

6524. Audi L, Mantzoros CS, Vidal-Puig A, Vargas D, Gussinye M, Carrascosa A Leptin in relation to resumption of menses in women with anorexia nervosa. MOLECULAR PSYCHIATRY. 1998;3(6):544-547.

6525. Bjorkenstam Emma, Burstrom Bo, Vinnerljung Bo, Kosidou Kyriaki Childhood adversity and psychiatric disorder in young adulthood: An analysis of 107,704 Swedes. JOURNAL OF PSYCHIATRIC RESEARCH. 2016;77():67-75.

6526. Chung Alicia, Vieira Dorice, Donley Tiffany, Tan Nicholas, Jean-Louis Girardin, Gouley Kathleen, Seixas Azizi Adolescent Peer Influence on Eating Behaviors via Social Media: Scoping Review. JOURNAL OF MEDICAL INTERNET RESEARCH. 2021;23(6):.

6527. Iersel Lieke, Beijers Rosanne, Gosker Harry, Schols Annemie Nutrition as a modifiable factor in the onset and progression of pulmonary function impairment in COPD: a systematic review. NUTRITION REVIEWS. 2022;80(6):1434-1444.

6528. Gostoli Sara, Montecchiarini Maria, Urgese Alessia, Ferrara Francesco, Polifemo Anna, Ceroni Liza, Gasparri Asia, Rafanelli Chiara, Cennamo Vincenzo The clinical utility of a comprehensive psychosomatic assessment in the program for colorectal cancer prevention: a cross-sectional study. SCIENTIFIC REPORTS. 2021;11(1):.

6529. Riviere S, Gillette-Guyonnet S, Nourhashemi F, Vellas B Nutrition and Alzheimer's disease. NUTRITION REVIEWS. 1999;57(12):363-367.

6530. Khalsa Sahib, Hassanpour Mahlega, Strober Michael, Craske Michelle, Arevian Armen, Feusner Jamie Interoceptive Anxiety and Body Representation in Anorexia Nervosa. FRONTIERS IN PSYCHIATRY. 2018;9():.

6531. Folsom Aaron, Cushman Mary Exploring Opportunities for Primary Prevention of Unprovoked Venous Thromboembolism: Ready for Prime Time?. JOURNAL OF THE

AMERICAN HEART ASSOCIATION. 2020;9(23):.

6532. Carter Frances, McIntosh Virginia, Joyce Peter, Frampton Christopher, Bulik Cynthia Cue reactivity in bulimia nervosa: A useful self-report approach. INTERNATIONAL JOURNAL OF EATING DISORDERS. 2006;39(8):694-699.

6533. Duan Ming-Jie, Dekker Louise, Carrero Juan-Jesus, Navis Gerjan Lifestyle patterns and incident type 2 diabetes in the Dutch lifelines cohort study. PREVENTIVE MEDICINE REPORTS. 2022;30():.

6534. COHLE SD, JONES DH, PURI S LINGUAL TONSILLAR HYPERTROPHY CAUSING FAILED INTUBATION AND CEREBRAL ANOXIA. AMERICAN JOURNAL OF FORENSIC MEDICINE AND PATHOLOGY. 1993;14(2):158-161.

6535. Ajjarapu Aparna, Hinkle Stefanie, Li Mengying, Francis Ellen, Zhang Cuilin Dietary Patterns and Renal Health Outcomes in the General Population: A Review Focusing on Prospective Studies. NUTRIENTS. 2019;11(8):.

6536. Wang Liang, Knudsen Markus, Lo Chun-Han, Wang Kai, He Mingming, Polychronidis Georgios, Hang Dong, He Xiaosheng, Zhong Rong, Wu Kana, Chan Andrew, Ogino Shuji, Giovannucci Edward, Song Mingyang Adherence to a healthy lifestyle in relation to colorectal cancer incidence and all-cause mortality after endoscopic polypectomy: A prospective study in three US cohorts. INTERNATIONAL JOURNAL OF CANCER. 2022;151(9):1523-1534.

6537. Eisenberg Daniel, Nicklett Emily, Roeder Kathryn, Kirz Nina Eating Disorder Symptoms Among College Students: Prevalence, Persistence, Correlates, and Treatment-Seeking. JOURNAL OF AMERICAN COLLEGE HEALTH. 2011;59(8):700-707.

6538. Kamal Nurkhalida, Ilowefah Muna, Hilles Ayah, Anua Nurul, Awin Tahani, Alshwyeh Hussah, Aldosary Sahar, Jambocus Najla, Alosaimi Areej, Rahman Azizur, Mahmood Syed, Mediani Ahmed Genesis and Mechanism of Some Cancer Types and an Overview on the Role of Diet and Nutrition in Cancer Prevention. MOLECULES. 2022;27(6):.

6539. Gout E, Boisson AM, Aubert S, Douce R, Bligny R Origin of the cytoplasmic pH changes during anaerobic stress in higher plant cells. Carbon-13 and phosphorous-31 nuclear magnetic resonance studies. PLANT PHYSIOLOGY. 2001;125(2):912-925.

6540. Carter FA, McIntosh VVW, Joyce PR, Gendall KA, Frampton CMA, Bulik CM Patterns of weight change after treatment for bulimia nervosa. INTERNATIONAL JOURNAL OF EATING DISORDERS. 2004;36(1):12-21.

6541. Probst-Hensch Nicole, Tanner Marcel, Kessler Claudia, Burri Christian, Kuenzli Nino Prevention - a cost-effective way to fight the non-communicable disease epidemic An academic perspective of the United Nations High-Level NCD Meeting. SWISS MEDICAL WEEKLY. 2011;141():.

6542. Dokucu Mehmet, Cloninger C. Personality disorders and physical comorbidities: a complex relationship. CURRENT OPINION IN PSYCHIATRY. 2019;32(5):435-441.

6543. Mian Annemiek, Jansen Pauline, Nguyen Anh, Bowling April, Renders Carry, Voortman Trudy Children's Attention-Deficit/Hyperactivity Disorder Symptoms Predict Lower Diet Quality but Not Vice Versa: Results from Bidirectional Analyses in a Population-Based Cohort. JOURNAL OF NUTRITION. 2019;149(4):642-648.

6544. Saez-Francas N., Marti Andres G., Ramirez N., Fabregues O., Alvarez-Sabin J., Casas M., Hernandez-Vara J. Clinical and psychopathological factors associated with impulse control disorders in Parkinson's disease. NEUROLOGIA. 2016;31(4):231-238.

6545. O'Dea J Nutrition education to prevent eating problems - primum laedere noli ({''}first do no harm{''}). FOOD AUSTRALIA. 1998;50(9):U1-U3.

6546. Mora Marisol, Penelo Eva, Roses Rocio, Gonzalez Marcela, Espinoza Paola, Devi Josep, Raich Rosa Pilot assessment of two disordered eating prevention programs. Preliminary findings on maladaptive beliefs related to eating disorders. EATING BEHAVIORS. 2017;25(SI):51-57.

6547. Franklin Barry, Durstine J., Roberts Christian, Barnard R. Impact of diet and exercise on lipid management in the modern era. BEST PRACTICE \& RESEARCH CLINICAL ENDOCRINOLOGY \& METABOLISM. 2014;28(3):405-421.

6548. Yang Jae, Keohane Laura, Pan Xiong-Fei, Qu Ruiqi, Shu Xiao-Ou, Lipworth Loren, Braun Kyle, Steinwandel Mark, Dai Qi, Shrubsole Martha, Zheng Wei, Blot William, Yu Danxia Association of Healthy Lifestyles With Risk of Alzheimer Disease and Related Dementias in Low-Income Black and White Americans. NEUROLOGY. 2022;99(9):E944-

E953.

6549. Berendsen A., Kang J., Feskens E., Groot C., Grodstein F., Rest O. Association of long-term adherence to the mind diet with cognitive function and cognitive decline in American women. JOURNAL OF NUTRITION HEALTH \& AGING. 2018;22(2):222-229.

6550. Saltzman Matthew, Edwards Cole, Adrain Jonathan, Westrop Stephen Persistent oceanic anoxia and elevated extinction rates separate the Cambrian and Ordovician radiations. GEOLOGY. 2015;43(9):807-810.

6551. Gebski Jerzy, Jezewska-Zychowicz Marzena, Guzek Dominika, Swiatkowska Monika, Stangierska Dagmara, Plichta Marta The Associations between Dietary Patterns and Short Sleep Duration in Polish Adults (LifeStyle Study). INTERNATIONAL JOURNAL OF ENVIRONMENTAL RESEARCH AND PUBLIC HEALTH. 2018;15(11):.

6552. Godart NT, Perdereau F, Jeammet PH, Flament MF Comorbidity between eating disorders and anxiety disorders: results. ENCEPHALE-REVUE DE PSYCHIATRIE CLINIQUE BIOLOGIQUE ET THERAPEUTIQUE. 2005;31(2):152-161.

6553. Leary Mark, Pursey Kirrilly, Verdejo-Garcia Antonio, Smout Scarlett, McBride Nyanda, Osman Bridie, Champion Katrina, Gardner Lauren, Jebeile Hiba, Kelly Erin, Thornton Louise, Teesson Maree, Burrows Tracy Socio-Demographic, Self-Control, Bullying, Parenting, and Sleep as Proximal Factors Associated with Food Addiction among Adolescents. BEHAVIORAL SCIENCES. 2022;12(12):.

6554. Wienbergen H., Gielen S., Gysan D., Albus C., Landmesser U., Hambrecht R. The qualification ``special cardiovascular prevention{''} of the German Cardiac Society. KARDIOLOGE. 2019;13(6):346-351.

6555. Gizzi Giulia, Cataldi Samuela, Mazzeschi Claudia, Delvecchio Elisa, Ceccarini Maria, Codini Michela, Albi Elisabetta Hypercholesterolemia in Cancer and in Anorexia Nervosa: A Hypothesis for a Crosstalk. INTERNATIONAL JOURNAL OF MOLECULAR SCIENCES. 2022;23(13):.

6556. Stice E, Shaw HE Role of body dissatisfaction in the onset and maintenance of eating pathology - A synthesis of research findings. JOURNAL OF PSYCHOSOMATIC RESEARCH. 2002;53(5):985-993.

6557. Lock J Treating adolescents with eating disorders in the family context - Empirical and theoretical considerations. CHILD AND ADOLESCENT PSYCHIATRIC CLINICS OF NORTH AMERICA. 2002;11(2):331+.

6558. Barchitta Martina, Maugeri Andrea, Quattrocchi Annalisa, Agrifoglio Ottavia, Scalisi Aurora, Agodi Antonella The Association of Dietary Patterns with High-Risk Human Papillomavirus Infection and Cervical Cancer: A Cross-Sectional Study in Italy. NUTRIENTS. 2018;10(4):.

6559. Liu Zhigang, Sun Yali, Qiao Qinglian, Zhao Tong, Zhang Wentong, Ren Bo, Liu Qian, Liu Xuebo Sesamol ameliorates high-fat and high-fructose induced cognitive defects via improving insulin signaling disruption in the central nervous system. FOOD \& FUNCTION. 2017;8(2):710-719.

6560. Murray Helen, Juarascio Adrienne, Thomas Jennifer Augmenting Diaphragmatic Breathing With Behavioral Exposure: Single-Case Experimental Design for Rumination Disorder. COGNITIVE AND BEHAVIORAL PRACTICE. 2020;27(3):347-356.

6561. Popkin Barry, Kenan W. Preventing type 2 diabetes: Changing the food industry. BEST PRACTICE \& RESEARCH CLINICAL ENDOCRINOLOGY \& METABOLISM. 2016;30(3):373-383.

6562. Johnson Jonetta, Eaton Danice, Pederson Linda, Lowry Richard Associations of Trying to Lose Weight, Weight Control Behaviors, and Current Cigarette Use Among US High School Students. JOURNAL OF SCHOOL HEALTH. 2009;79(8):355-360.

6563. GILLBERG C, RASTAM M, GILLBERG IC ANOREXIA-NERVOSA - WHO SEES THE PATIENTS AND WHO DO THE PATIENTS SEE. ACTA PAEDIATRICA. 1994;83(9):967-971.

6564. Ra Jin-Suk, Kim Hyesun Combined Effects of Unhealthy Lifestyle Behaviors on Metabolic Syndrome among Postmenopausal Women. HEALTHCARE. 2021;9(7):.

6565. Ma SS, Chen PM Storage disorder and ripening behavior of `Doyenne du Comice' pears in relation to storage conditions. POSTHARVEST BIOLOGY AND TECHNOLOGY. 2003;28(2):281-294.

6566. Benamar Abdelilah, Rolletschek Hardy, Borisjuk Ljudmilla, Avelange-Macherel Marie-Helene, Curien Gilles, Mostefai H., Andriantsitohaina Ramaroson, Macherel David Nitrite-nitric oxide control of mitochondrial respiration at the frontier of anoxia. BIOCHIMICA ET BIOPHYSICA ACTA-BIOENERGETICS. 2008;1777(10):1268-1275.

6567. Gundel Louise, Pedersen Carsten, Munk-Olsen Trine, Dalsgaard Soren Longitudinal association between mental disorders in childhood and subsequent depression - A nationwide prospective cohort study. JOURNAL OF AFFECTIVE DISORDERS. 2018;227():56-64.

6568. Boyer Diana, Haddad Emily, Seeger Emily THE LAST GASP: TRACE FOSSILS TRACK DEOXYGENATION LEADING INTO THE FRASNIAN-FAMENNIAN EXTINCTION EVENT. PALAIOS. 2014;29(12):646-651.

6569. Zipfel Stephan, Giel Katrin, Bulik Cynthia, Hay Phillipa, Schmidt Ulrike Anorexia nervosa: aetiology, assessment, and treatment. LANCET PSYCHIATRY. 2015;2(12):1099-1111.

6570. Yamaguchi Miwa, Nomura Marika, Arai Yusuke, Vandevijvere Stefanie, Swinburn Boyd, Nishi Nobuo An assessment of implementation gaps and priority recommendations on food environment policies: the Healthy Food Environment Policy Index in Japan. PUBLIC HEALTH NUTRITION. 2022;25(6):1720-1732.

6571. Kadiri Adjei, Ansu-Mensah Monica, Bawontuo Vitalis, Kuupiel Desmond Mapping research evidence on implementation of the WHO'best buys' and other interventions for the prevention and control of non-communicable diseases in sub-Saharan Africa: a scoping review protocol. SYSTEMATIC REVIEWS. 2022;11(1):.

6572. Pentony M., Featherstone M., Sheikh Y., Stroiescu A., Bruell H., Gill I., Gorman K. Dystonia in children with acquired brain injury. EUROPEAN JOURNAL OF PAEDIATRIC NEUROLOGY. 2022;41():41-47.

6573. Avgoustiniatos Efstathios, Hering Bernhard, Rozak Phillip, Wilson John, Tempelman Linda, Papas Klearchos Commercially available gas-permeable cell culture bags may not prevent anoxia in cultured or shipped islets. XENOTRANSPLANTATION. 2007;14(5):420.

6574. Martinon Prescilla, Fraticelli Laurie, Giboreau Agnes, Dussart Claude, Bourgeois Denis, Carrouel Florence Nutrition as a Key Modifiable Factor for Periodontitis and Main

Chronic Diseases. JOURNAL OF CLINICAL MEDICINE. 2021;10(2):.

6575. Hopkins Ramona Does near drowning in ice water prevent anoxic induced brain injury?. JOURNAL OF THE INTERNATIONAL NEUROPSYCHOLOGICAL SOCIETY. 2008;14(4):656-659.

6576. Malkawi Ahmad, Meertens Ree, Kremers Stef, Borgh-Sleddens Ester, Picknell Gareth, Al Shehhi Mouza Correlates of Dietary Behaviors Among Young Emirati Males Completing Compulsory Military Service. MILITARY MEDICINE. 2022;():.

6577. Seitz Jochen, Walter Martin, Mainz Verena, Herpertz-Dahlmann Beate, Konrad Kerstin, Polier Georg Brain volume reduction predicts weight development in adolescent patients with anorexia nervosa. JOURNAL OF PSYCHIATRIC RESEARCH. 2015;68():228-237.

6578. Aggarwal Monica, Devries Stephen, Freeman Andrew, Ostfeld Robert, Gaggin Hanna, Taub Pam, Rzeszut Anne, Allen Kathleen, Conti Richard The Deficit of Nutrition Education of Physicians. AMERICAN JOURNAL OF MEDICINE. 2018;131(4):339-345.

6579. Proemse Bernadette, Grasby Stephen, Wieser M., Mayer B., Beauchamp B. Molybdenum isotopic evidence for oxic marine conditions during the latest Permian extinction. GEOLOGY. 2013;41(9):967-970.

6580. Goncharov A., Bolotov S., Puklakov V., Malashenkov D., Erina O., Lomov V. Vertical Water Structure and Reservoir Plankton in Spring. INLAND WATER BIOLOGY. 2022;15(4):437-445.

6581. Oberne Alison Self-induced Vomiting as a Function of Bulimia Nervosa Increases the Risk for Oral Health Issues. JOURNAL OF EVIDENCE-BASED DENTAL PRACTICE. 2014;14(4):195-196.

6582. Chambry J, Corcos M, Guilbaud O, Jeammet P Masculine anorexia nervosa: realities and perspectives. ANNALES DE MEDECINE INTERNE. 2002;153(3):IS61-IS67.

6583. Koziarska-Rosciszewska Malgorzata, Dobielska Maria, Ocetek Marzena, Iwan Patrycja, Malik Praveen, Jozwik Adam, Rysz Jacek Eating disorders in university students in Lodz, the role of a family physician. FAMILY MEDICINE AND PRIMARY CARE REVIEW.

2021;23(2):179-184.

6584. Corsica Joyce, Hood Megan Eating Disorders in an Obesogenic Environment. JOURNAL OF THE AMERICAN DIETETIC ASSOCIATION. 2011;111(7):996-1000.

6585. Zhang Hongya, Tang Xiao, Hu Dongmei, Li Guorong, Song Guirong Transition patterns of metabolism-weight phenotypes over time: A longitudinal study using the multistate Markov model in China. FRONTIERS IN PUBLIC HEALTH. 2022;10():.

6586. Zerbe KJ Anorexia nervosa and bulimia nervosa - When the pursuit of bodily `perfection' becomes a killer. POSTGRADUATE MEDICINE. 1996;99(1):161+.

6587. Kim Dae, Sagar Utpal, Adams Suzanne, Whellan David Lifestyle Risk Factors and Utilization of Preventive Services in Disabled Elderly Adults in the Community. JOURNAL OF COMMUNITY HEALTH. 2009;34(5):440-448.

6588. Schaefer Miriam, Klier Claudia, Papageorgiou Konstantinos, Friedrich Max, Amminger G. Early detection of psychotic disorders. NEUROPSYCHIATRIE. 2007;21(1):37-44.

6589. Takeuchi Hiroaki, Terada Mika, Kobayashi Kazuko, Uraguchi Masahide, Nomura Yoshiaki, Hanada Nobuhiro Influences of Masticatory Function Recovery Combined with Health Guidance on Body Composition and Metabolic Parameters. OPEN DENTISTRY JOURNAL. 2019;13():124-136.

6590. Mizgier M., Jarzabek-Bielecka G., Mruczyk K., Kedzia W. Comparison of dietary behaviour of a selected student population as regards their influence on fertility. CLINICAL AND EXPERIMENTAL OBSTETRICS \& GYNECOLOGY. 2019;46(3):450-457.

6591. DeBate Rita, Bleck Jennifer, Raven Jessica, Severson Herb Using Intervention Mapping to Develop an Oral Health e-Curriculum for Secondary Prevention of Eating Disorders. JOURNAL OF DENTAL EDUCATION. 2017;81(6):716-725.

6592. Yegen Berrak Lifestyle and Peptic Ulcer Disease. CURRENT PHARMACEUTICAL DESIGN. 2018;24(18):2034-2040.

6593. Pereira Tintina, Lock James, Oggins Jean Role of therapeutic alliance in family therapy for adolescent anorexia nervosa. INTERNATIONAL JOURNAL OF EATING DISORDERS.

2006;39(8):677-684.

6594. Gonzalez-Correa JA, Arrebola MM, Urena IM, Guerrero A, Munoz-Marin J, Ruiz-Villafranca D, Cuesta FS, De La Cruz JP Effects of triflusal on oxidative stress, prostaglandin production and nitric oxide pathway in a model of anoxia-reoxygenation in rat brain slices. BRAIN RESEARCH. 2004;1011(2):148-155.

6595. Toulany Alene, Katzman Debra, Kaufman Miriam, Hiraki Linda, Silverman Earl Chicken or the Egg: Anorexia Nervosa and Systemic Lupus Erythematosus in Children and Adolescents. PEDIATRICS. 2014;133(2):E447-E450.

6596. Michalak Johannes, Zhang Xiao, Jacobi Frank Vegetarian diet and mental disorders: results from a representative community survey. INTERNATIONAL JOURNAL OF BEHAVIORAL NUTRITION AND PHYSICAL ACTIVITY. 2012;9():.

6597. Vethakkan Shireene, Venugopal Yogeswari, Tan Alexander, Paramasivam Sharmila, Ratnasingam Jeyakantha, Razak Rohaya, Alias Azmi, Kassim Fauziah, Choong Karen HYPOTHALAMIC GERMINOMA MASQUERADING AS SUPERIOR MESENTERIC ARTERY (SMA) SYNDROME. ENDOCRINE PRACTICE. 2013;19(1):E29-E34.

6598. Santaliestra-Pasias Alba, Pablo Rey-Lopez Juan, Moreno Aznar Luis Obesity and sedentarism in children and adolescents: What should be bone?. NUTRICION HOSPITALARIA. 2013;28(5):99-104.

6599. Borle AB, Stanko RT Pyruvate reduces anoxic injury and free radical formation in perfused rat hepatocytes. AMERICAN JOURNAL OF PHYSIOLOGY-GASTROINTESTINAL AND LIVER PHYSIOLOGY. 1996;270(3):G535-G540.

6600. Khemayanto Hidayat, Bimin Shi Role of Mediterranean diet in prevention and management of type 2 diabetes. CHINESE MEDICAL JOURNAL. 2014;127(20):3651-3656.

6601. Novakovic Budimka, Jovicic Jelena, Milic Natasa, Jusupovic Fatima, Grujicic Maja, Djuric Dusan Nutrition care process in cancer. HEALTHMED. 2010;4(2):427-433.

6602. Gostoli Sara, Roncuzzi Renzo, Urbinati Stefano, Morisky Donald, Rafanelli Chiara Unhealthy behaviour modification, psychological distress, and 1-year survival in cardiac

rehabilitation. BRITISH JOURNAL OF HEALTH PSYCHOLOGY. 2016;21(4):894-916.

6603. Brown DW, Balluz LS, Giles WH, Beckles GL, Moriarty DG, Ford ES, Mokdad AH Diabetes mellitus and health-related quality of life among older adults Findings from the behavioral risk factor surveillance system (BRFSS). DIABETES RESEARCH AND CLINICAL PRACTICE. 2004;65(2):105-115.

6604. PUGLIESE A, DARGENIO P, ARSIERI R, RUSSO R, PALUMBO F NEONATAL-MORTALITY CAUSES IN ITALY. RIVISTA ITALIANA DI PEDIATRIA-ITALIAN JOURNAL OF PEDIATRICS. 1993;19(3):241-249.

6605. Gans Kim, Tovar Alison, Jiang Qianxia, Mello Jennifer, Dionne Laura, Kang Augustine, Mena Noereem, Palomo Vanessa, Risica Patricia Nutrition-Related Practices of Family Child Care Providers and Differences by Ethnicity. CHILDHOOD OBESITY. 2019;15(3):167-184.

6606. Cooper Theodore, DeBon Margaret, Haddock C., Esquivel Denise, Klesges Robert, Lando Harry, Talcott Wayne Demographics and risky lifestyle behaviors associated with willingness to risk sexually transmitted infection in air force recruits. AMERICAN JOURNAL OF HEALTH PROMOTION. 2008;22(3):164-167.

6607. Peersen Kari, Otterstad Jan, Sverre Elise, Perk Joep, Gullestad Lars, Moum Torbjorn, Dammen Toril, Munkhaugen John Medical and Psychosocial Factors Associated With Low Physical Activity and Increasing Exercise Level After a Coronary Event. JOURNAL OF CARDIOPULMONARY REHABILITATION AND PREVENTION. 2020;40(1):35-40.

6608. Godos J., Bella F., Torrisi A., Sciacca S., Galvano F., Grosso G. Dietary patterns and risk of colorectal adenoma: a systematic review and meta-analysis of observational studies. JOURNAL OF HUMAN NUTRITION AND DIETETICS. 2016;29(6):757-767.

6609. Jong Elske, Schokker Dieuwke, Visscher Tommy, Seidell Jacob, Renders Carry Behavioural and socio-demographic characteristics of Dutch neighbourhoods with high prevalence of childhood obesity. INTERNATIONAL JOURNAL OF PEDIATRIC OBESITY. 2011;6(3-4):298-305.

6610. Lim Soo, Kong Alice, Tuomilehto Jaakko Influence of COVID-19 pandemic and related quarantine procedures on metabolic risk. PRIMARY CARE DIABETES. 2021;15(5):745-750.

6611. Mundula Tiziana, Russo Edda, Curini Lavinia, Giudici Francesco, Piccioni Andrea, Franceschi Francesco, Amedei Amedeo Chronic Systemic Low-Grade Inflammation and Modern Lifestyle: The Dark Role of Gut Microbiota on Related Diseases with a Focus on COVID-19 Pandemic. CURRENT MEDICINAL CHEMISTRY. 2022;29(33):5370-5396.

6612. Holtrop Jodi, Dosh Steven, Torres Trissa, Thum Yeow The Community Health Educator Referral Liaison (CHERL) A Primary Care Practice Role for Promoting Healthy Behaviors. AMERICAN JOURNAL OF PREVENTIVE MEDICINE. 2008;35(5, S):S365-S372.

6613. Gumz Antje, Uhlenbusch Natalie, Weigel Angelika, Wegscheider Karl, Romer Georg, Loewe Bernd Decreasing the duration of untreated illness for individuals with anorexia nervosa: study protocol of the evaluation of a systemic public health intervention at community level. BMC PSYCHIATRY. 2014;14():.

6614. Haines Jess, Neumark-Sztainer Dianne, Perry Cheryl, Hannan Peter, Levine Michael VIK (Very Important Kids): a school-based program designed to reduce teasing and unhealthy weight-control behaviors. HEALTH EDUCATION RESEARCH. 2006;21(6):884-895.

6615. Mao Chengyu, Li Dongjiu, Zhou En, Gao Erhe, Zhang Tiantian, Sun Shufang, Gao Lin, Fan Yuqi, Wang Changqian Extracellular vesicles from anoxia preconditioned mesenchymal stem cells alleviate myocardial ischemia/reperfusion injury. AGING-US. 2021;13(4):6156-6170.

6616. Rogalska J, Caputa M, Wentowska K, Nowakowska A Stress-induced behaviour in juvenile rats: effects of neonatal asphyxia, body temperature and chelation of iron. BEHAVIOURAL BRAIN RESEARCH. 2004;154(2):321-329.

6617. Veras Renato Chronic disease management: mistaken approach in the elderly. REVISTA DE SAUDE PUBLICA. 2012;46(6):929-934.

6618. Oyekale Abayomi Effect of Obesity and Other Risk Factors on Hypertension among Women of Reproductive Age in Ghana: An Instrumental Variable Probit Model. INTERNATIONAL JOURNAL OF ENVIRONMENTAL RESEARCH AND PUBLIC HEALTH. 2019;16(23):.

6619. Tabibzadeh Pantea, Mewes Ricarda Thin mother, obese child? A review of early risk factors for obesity in offspring. CURRENT OPINION IN PSYCHIATRY. 2016;29(5):309-315.

6620. Rubbert Stefanie, Bisnauth Radha, Offen Liz Establishing a body awareness group for adults with learning disabilities. BRITISH JOURNAL OF LEARNING DISABILITIES. 2014;42(1):43-49.

6621. Shalitin Shlomit, Battelino Tadej, Moreno Luis Obesity, Metabolic Syndrome, and Nutrition. . 2017;116():16-51.

6622. Eagles JM, Easton EA, Nicoll KS, Johnston MI, Millar HR Changes in the presenting features of females with anorexia nervosa in northeast Scotland, 1965-1991. INTERNATIONAL JOURNAL OF EATING DISORDERS. 1999;26(3):289-294.

6623. Denova-Gutierrez Edgar, Mendez-Sanchez Lucia, Munoz-Aguirre Paloma, Tucker Katherine, Clark Patricia Dietary Patterns, Bone Mineral Density, and Risk of Fractures: A Systematic Review and Meta-Analysis. NUTRIENTS. 2018;10(12):.

6624. Taylor C., Bryson Susan, Doyle Angela, Luce Kristine, Cunning Darby, Abascal Liana, Rockwell Roxanne, Field Alison, Striegel-Moore Ruth, Winzelberg Andrew, Wilfley Denise The adverse effect of negative comments about weight and shape from family and siblings on women at high risk for eating disorders. PEDIATRICS. 2006;118(2):731-738.

6625. Stice Eric, Yokum Sonja, Waters Allison Dissonance-Based Eating Disorder Prevention Program Reduces Reward Region Response to Thin Models; How Actions Shape Valuation. PLOS ONE. 2015;10(12):.

6626. Konstantinov Vsevolod, Gritsenko Valentina, Reznik Alexander, Isralowitz Richard The Impact of COVID-19 on Health and Well-Being: Foreign Medical Students in Eastern Europe. SOCIAL SCIENCES-BASEL. 2022;11(9):.

6627. Li Ming, Dibley Michael, Sibbritt David, Yan Hong Dietary habits and overweight/obesity in adolescents in Xi'an City, China. ASIA PACIFIC JOURNAL OF CLINICAL NUTRITION. 2010;19(1):76-82.

6628. Molina Paulina, Galvez Patricia, Jose Stecher Maria, Vizcarra Marcela, Jose Coloma Maria, Schwingel Andiara Family influences on maternal feeding practices of preschool

children from vulnerable families in the Metropolitan Region of Chile. ATENCION PRIMARIA. 2021;53(9):.

6629. Al-Thani Mohammed, Al-Mutawa Kholood, Alyafei Salah, Ijaz Muhammad, Khalifa Shamseldin, Kokku Suresh, Mishra Amit, Poovelil Benjamin, Soussi Mounir, Toumi Amine, Dargham Soha, Awad Susanne, Abu-Raddad Laith Characterizing epidemiology of prediabetes, diabetes, and hypertension in Qataris: A cross-sectional study. PLOS ONE. 2021;16(10):.

6630. Spettigue Wendy, Obeid Nicole, Erbach Madison, Feder Stephen, Finner Natalie, Harrison Megan, Isserlin Leanna, Robinson Amy, Norris Mark The impact of COVID-19 on adolescents with eating disorders: a cohort study. JOURNAL OF EATING DISORDERS. 2021;9(1):.

6631. Hoelling H., Schlack R. Eating disorders in children and adolescents. First results of the German Health Interview and Examination Survey for Children and Adolescents (KiGGS). BUNDESGESUNDHEITSBLATT-GESUNDHEITSFORSCHUNG-GESUNDHEITSSCHUTZ. 2007;50(5-6):794-799.

6632. Plichta Marta, Jezewska-Zychowicz Marzena Eating behaviors, attitudes toward health and eating, and symptoms of orthorexia nervosa among students. APPETITE. 2019;137():114-123.

6633. Sarebanhassanabadi Mohammadtaghi, Kalantari Manijeh, Boffetta Paolo, Beiki Omid, Pakseresht Mohammadreza, Sarrafzadegan Nizal, Mirzaei Masoud, Kraemer Alexander, Seyedhosseini Seyedmostafa, Mali Shahriar, Namayandeh Seyedeh, Razavi Seyed, Alipour Mohammad, Emami Mahmood, Abad Mostafa, Hosseini Habib, Salehi-Abargouei Amin Dietary habits and the 10-year risk of overweight and obesity in urban adult population: A cohort study predicated on Yazd Healthy Heart Project. DIABETES \& METABOLIC SYNDROME-CLINICAL RESEARCH \& REVIEWS. 2020;14(5):1391-1397.

6634. Christenson Anne, Johansson Eva, Reynisdottir Signy, Torgerson Jarl, Hemmingsson Erik Women's Perceived Reasons for Their Excessive Postpartum Weight Retention: A Qualitative Interview Study. PLOS ONE. 2016;11(12):.

6635. Hunsaker Sanita, Garland Beth, Rofey Dana, Reiter-Purtill Jennifer, Mitchell James, Courcoulas Anita, Jenkins Todd, Zeller Meg A Multisite 2-Year Follow Up of Psychopathology Prevalence, Predictors, and Correlates Among Adolescents Who Did or Did Not Undergo

Weight Loss Surgery. JOURNAL OF ADOLESCENT HEALTH. 2018;63(2):142-150.

6636. Stys PK, Lopachin RM Mechanisms of calcium and sodium fluxes in anoxic myelinated central nervous system axons. NEUROSCIENCE. 1998;82(1):21-32.

6637. Simpson Courtney, Griffin Brandon, Mazzeo Suzanne Psychological and behavioral effects of obesity prevention campaigns. JOURNAL OF HEALTH PSYCHOLOGY. 2019;24(9):1268-1281.

6638. Tyler Margaret, Wehby George, Robbins James, Damiano Peter Separation Anxiety in Children Ages 4 Through 9 With Oral Clefts. CLEFT PALATE-CRANIOFACIAL JOURNAL. 2013;50(5):520-527.

6639. Schiefer Johannes Sleep and epilepsy. ZEITSCHRIFT FUR EPILEPTOLOGIE. 2018;31(1):12-21.

6640. Barquera Jose Double-blind controlled study with clonazapam and placebo in social anxiety disorder. SALUD MENTAL. 2008;31(4):299-306.

6641. Wells J., Haroun D., Williams J., Nicholls D., Darch T., Eaton S., Fewtrell M. Body composition in young female eating-disorder patients with severe weight loss and controls: evidence from the four-component model and evaluation of DXA. EUROPEAN JOURNAL OF CLINICAL NUTRITION. 2015;69(12):1330-1335.

6642. Hatime Zineb, El Kinany Khaoula, Huybrechts Inge, Gunter Marc, Khalis Mohamed, Deoula Meimouna, Boudouaya Hanae, Benslimane Abdelilah, Nejjari Chakib, Benider Abdellatif, El Rhazi Karima Extended healthy lifestyle index and colorectal cancer risk in the Moroccan population. EUROPEAN JOURNAL OF NUTRITION. 2021;60(2):1013-1022.

6643. Carter FA, Bulik CM, McIntosh VV, Joyce PR Changes in cue reactivity following treatment for bulimia nervosa. INTERNATIONAL JOURNAL OF EATING DISORDERS. 2001;29(3):336-344.

6644. Melchin Michael, Mitchell Charles, Holmden Chris, Storch Petr Environmental changes in the Late Ordovicianearly Silurian: Review and new insights from black shales and nitrogen isotopes. GEOLOGICAL SOCIETY OF AMERICA BULLETIN. 2013;125(11-12):1635-

1670.

6645. Colton Patricia, Olmsted Marion, Daneman Denis, Farquhar Jamie, Wong Harmonie, Muskat Stephanie, Rodin Gary Eating Disorders in Girls and Women With Type 1 Diabetes: A Longitudinal Study of Prevalence, Onset, Remission, and Recurrence. DIABETES CARE. 2015;38(7):1212-1217.

6646. Di Cagno A., Pistone E., Battaglia C., Borrione P., Giombini A., Pigozzi F. Relationship between participation in lean sports and body image distortion: a controlled study. MEDICINA DELLO SPORT. 2014;67(1):99-107.

6647. Jones Meredith, Westen Drew DIAGNOSIS AND SUBTYPES OF ADOLESCENT ANTISOCIAL PERSONALITY DISORDER. JOURNAL OF PERSONALITY DISORDERS. 2010;24(2):217-243.

6648. Schlegl Sandra, Neumayr Christina, Voderholzer Ulrich Therapist-guidedsmartphone-based aftercare for inpatients with severeanorexianervosa (SMART-AN): Study protocol of a randomized controlled trial. INTERNATIONAL JOURNAL OF EATING DISORDERS. 2020;53(10):1739-1745.

6649. Bak-Sosnowska Monika, Skrzypulec-Plinta Violetta Eating habits and physical activity of adolescents in Katowice - the teenagers' declarations vs. their parents' beliefs. JOURNAL OF CLINICAL NURSING. 2012;21(17-18):2461-2468.

6650. Pejovic-Milovancevic Milica, Grujicic Roberto, Stupar Sanja, Ninkovic Minja Overcoming traps and challenges in child and adolescent psychiatry. SRPSKI ARHIV ZA CELOKUPNO LEKARSTVO. 2021;149(3-4):236-241.

6651. Hermans Roel, Bruin Hanneke, Larsen Junilla, Mensink Frederike, Hoek Annet Adolescents' Responses to a School-Based Prevention Program Promoting Healthy Eating at School. FRONTIERS IN PUBLIC HEALTH. 2017;5():.

6652. Santos Constanca, Picoito Joao, Nunes Carla, Loureiro Isabel Early Individual and Family Predictors of Weight Trajectories From Early Childhood to Adolescence: Results From the Millennium Cohort Study. FRONTIERS IN PEDIATRICS. 2020;8():.

6653. Zhang Juan, Seo Dong-Chul, Kolbe Lloyd, Lee Albert, Middlestadt Susan, Zhao Wenhua, Huang Songyuan Comparison of Overweight, Weight Perception, and Weight-Related Practices Among High School Students in Three Large Chinese Cities and Two Large U.S. Cities. JOURNAL OF ADOLESCENT HEALTH. 2011;48(4):366-372.

6654. Rodgers-Garlick C., Hogg D., Buck L. OXYGEN-SENSITIVE REDUCTION IN CA(2+)-ACTIVATED K+(CHANNEL OPEN PROBABILITY IN TURTLE CEREBROCORTEX. NEUROSCIENCE. 2013;237():243-254.

6655. Kabisch Stefan, Wenschuh Soren, Buccellato Palina, Spranger Joachim, Pfeiffer Andreas Affordability of Different Isocaloric Healthy Diets in Germany-An Assessment of Food Prices for Seven Distinct Food Patterns. NUTRIENTS. 2021;13(9):.

6656. Andrade Luciene, Maia Costa Maria, Caetano Joselany, Soares Enedina, Beserra Eveline The problematic aspects of the family caregiver of people who suffered strokes. REVISTA DA ESCOLA DE ENFERMAGEM DA USP. 2009;43(1):35-40.

6657. Botrel A, Magne C, Kaiser WM Nitrate reduction, nitrite reduction and ammonium assimilation in barley roots in response to anoxia. PLANT PHYSIOLOGY AND BIOCHEMISTRY. 1996;34(5):645-652.

6658. De Cock Valerie, Lannuzel Annie, Verhaeghe Stephane, Roze Emmanuel, Ruberg Merle, Derenne Jean, Willer Jean, Vidailhet Marie, Arnulf Isabelle REM sleep behavior disorder in patients with guadeloupean parkinsonism, a tauopathy. SLEEP. 2007;30(8):1026-1032.

6659. Boudreault Veronique, Labossiere Sophie, Gauthier Veronique, Brassard Sophie, Couture Sophie, Dionne Frederick, Laurier Catherine, Durand-Bush Natalie Symptoms of mental illness among university student-athletes during the second wave of the COVID-19 pandemic lockdown in Canada. FRONTIERS IN SPORTS AND ACTIVE LIVING. 2022;4():.

6660. Bryan Danielle, Cardi Valentina, Willmott Daniel, Teehan Eimear, Rowlands Katie, Treasure Janet A systematic review of interventions to support transitions from intensive treatment for adults with anorexia nervosa and/or their carers. EUROPEAN EATING DISORDERS REVIEW. 2021;29(3, SI):355-370.

6661. Leibbrand R Obesity: Course and factors influencing the course. VERHALTENSTHERAPIE. 2002;12(4):327-333.

6662. Sanchez-Carracedo David, Carretero Cristina, Conesa Alfons Roundtable on the Prevention of Eating Disorders: The Catalan public policy initiative. EATING BEHAVIORS. 2017;25(SI):15-17.

6663. Manuelli Matteo, Blundell John, Biino Ginevra, Cena Hellas Body composition and resting energy expenditure in women with anorexia nervosa: Is hyperactivity a protecting factor?. CLINICAL NUTRITION ESPEN. 2019;29():160-164.

6664. Robinson Sarah, Perkins Sarah, Bauer Stephanie, Hammond Neil, Treasure Janet, Schmidt Ulrike Aftercare intervention through text messaging in the treatment of bulimia nervosa - Feasibility pilot. INTERNATIONAL JOURNAL OF EATING DISORDERS. 2006;39(8):633-638.

6665. Becker Carolyn, Stice Eric, Shaw Heather, Woda Susan Use of empirically supported interventions for psychopathology: Can the participatory approach move us beyond the research-to-practice gap?. BEHAVIOUR RESEARCH AND THERAPY. 2009;47(4):265-274.

6666. Folkvord Frans, Naderer Brigitte, Coates Anna, Boyland Emma Promoting Fruit and Vegetable Consumption for Childhood Obesity Prevention. NUTRIENTS. 2022;14(1):.

6667. Montana Blasco Mireia, Jimenez-Morales Monika Breakfast Food Advertising and Prevention of Obesity: Analysis of the Nutritional Value of the Products and Discursive Strategies Used in the Breakfast Ads from 2015 to 2019. NUTRIENTS. 2021;13(1):.

6668. Pimenta Adriano, Bes-Rastrollo Maira, Gea Alfredo, Sayon-Orea Carmen, Zazpe Itziar, Lopez-Iracheta Roberto, Martinez-Gonzalez Miguel Snacking between main meals is associated with a higher risk of metabolic syndrome in a Mediterranean cohort: the SUN Project (Seguimiento Universidad de Navarra). PUBLIC HEALTH NUTRITION. 2016;19(3):658-666.

6669. Merdad Adnan, Karim Sajjad, Schulten Hans-Juergen, Jayapal Manikandan, Dallol Ashraf, Buhmeida Abdelbaset, Al-Thubaity Fatima, Garil Mamdooh, Chaudhary Adeel, Abuzenadah Adel, Al-Qahtani Mohammed Transcriptomics profiling study of breast cancer from Kingdom of Saudi Arabia revealed altered expression of Adiponectin and Fatty Acid Binding Protein4: Is lipid metabolism associated with breast cancer?. BMC GENOMICS.

2015;16(1):.

6670. Fung ML, Haddad GG Anoxia-induced depolarization in CA1 hippocampal neurons: role of Na+-dependent mechanisms. BRAIN RESEARCH. 1997;762(1-2):97-102.

6671. Uusitalo Ulla, Arkkola Tuula, Ovaskainen Marja-Leena, Kronberg-Kippila Carina, Kenward Mike, Veijola Riitta, Simell Olli, Knip Mikael, Virtanen Suvi Unhealthy dietary patterns are associated with weight gain during pregnancy among Finnish women. PUBLIC HEALTH NUTRITION. 2009;12(12):2392-2399.

6672. Koster Annemarie, Penninx Brenda, Newman Anne, Visser Marjolein, Gool Coen, Harris Tamara, Eijk Jacques, Kempen Gertrudis, Brach Jennifer, Simonsick Eleanor, Houston Denise, Tylavsky Frances, Rubin Susan, Kritchevsky Stephen Lifestyle factors and incident mobility limitation in obese and non-obese older adults. OBESITY. 2007;15(12):3122-3132.

6673. Frank Christoph, Fallah Mahdi, Ji Jianguang, Sundquist Jan, Hemminki Kari The population impact of familial cancer, a major cause of cancer. INTERNATIONAL JOURNAL OF CANCER. 2014;134(8):1899-1906.

6674. Xiao-yun Shen Studies on Wool-Eating Ailment in Guizhou Semi-Fine Wool Sheep. AGRICULTURAL SCIENCES IN CHINA. 2011;10(10):1618-1623.

6675. McCabe Marita, Ricciardelli Lina, Ridge Damien ``Who thinks i need a perfect body?{''} Perceptions and internal dialogue among adolescents about their bodies. SEX ROLES. 2006;55(5-6):409-419.

6676. Klein DA, Walsh BT Translational approaches to understanding anorexia nervosa. INTERNATIONAL JOURNAL OF EATING DISORDERS. 2005;37(S):S10-S14.

6677. Surova Olga, Nagibin Vasyl, Tumanovskaya Lesya, Dosenko Victor, Moibenko Alexey Effect of a low dose of proteasome inhibitor on cell death and gene expression in neonatal rat cardiomyocyte cultures exposed to anoxia-reoxygenation. EXPERIMENTAL \& CLINICAL CARDIOLOGY. 2009;14(2):E57-E61.

6678. Wang Zuoyuan, You Li, Ren Yuan, Zhu Xiaoye, Mao Xiaoyi, Liang Xiaowan, Wang Tingting, Guo Yumeng, Liu Te, Xue Jun Finasteride Alleviates High Fat Associated Protein-Overload Nephropathy by Inhibiting Trimethylamine N-Oxide Synthesis and Regulating Gut

Microbiota. FRONTIERS IN PHYSIOLOGY. 2022;13():.

6679. Panter KE, Baker DC, Kechele PO Water hemlock (Cicuta douglasii) in sheep: pathologic description and prevention of lesions and death. JOURNAL OF VETERINARY DIAGNOSTIC INVESTIGATION. 1996;8(4):474-480.

6680. Chui Harold, Christensen Bruce, Zipursky Robert, Richards Blake, Hanratty M., Kabani Noor, Mikulis David, Katzman Debra Cognitive function and brain structure in females with a history of adolescent-onset anorexia nervosa. PEDIATRICS. 2008;122(2):E426-E437.

6681. Pamenter Matthew, Hogg David, Gu Xiang, Buck Leslie, Haddad Gabriel Painted turtle cortex is resistant to an in vitro mimic of the ischemic mammalian penumbra. JOURNAL OF CEREBRAL BLOOD FLOW AND METABOLISM. 2012;32(11):2033-2043.

6682. Shen Y., Liu M., Chen C., Lai X., Zhang M. EFFECT OF GLP-1 ANALOGUE LIRAGLUTIDE ON PREVENTION OF CARDIOMYOCYTES APOPTOSIS INDUCED BY ANOXIA/REOXYGENATION. DIABETES RESEARCH AND CLINICAL PRACTICE. 2014;106(1):S227-S228.

6683. Xu Xiaoyue, Inglis Sally, Parker Deborah Sex differences in dietary consumption and its association with frailty among middle-aged and older Australians: a 10-year longitudinal survey. BMC GERIATRICS. 2021;21(1):.

6684. Nappi Francesca, Barrea Luigi, Di Somma Carolina, Savanelli Maria, Muscogiuri Giovanna, Orio Francesco, Savastano Silvia Endocrine Aspects of Environmental ``Obesogen{''} Pollutants. INTERNATIONAL JOURNAL OF ENVIRONMENTAL RESEARCH AND PUBLIC HEALTH. 2016;13(8):.

6685. Rodriguez JO, Fernandez MM, Narbona ML, Rivas ES, Martinez JR Incidence of anorexia nervosa in a public children and adolescents mental health unit. ACTAS ESPANOLAS DE PSIQUIATRIA. 2002;30(1):14-18.

6686. Horsager Christina, Faerk Emil, Gearhardt Ashley, Lauritsen Marlene, Ostergaard Soren Food addiction comorbid to mental disorders in adolescents: a nationwide survey and register-based study. EATING AND WEIGHT DISORDERS-STUDIES ON ANOREXIA BULIMIA AND OBESITY. 2022;27(3):945-959.

6687. Gong Yan, Zeng Qiang, Yan Yi, Han Chaojing, Zheng Yansong Association between Lifestyle and Gastroesophageal Reflux Disease Questionnaire Scores: A Cross-Sectional Study of 37 442 Chinese Adults. GASTROENTEROLOGY RESEARCH AND PRACTICE. 2019;2019():.

6688. Kelishadi Roya Life-Cycle Approach for Prevention of Non Communicable Disease. . 2019;1121():1-6.

6689. Weissman Ruth Fifty volumes of scholarship on eating disorders. INTERNATIONAL JOURNAL OF EATING DISORDERS. 2017;50(1):3-8.

6690. Di Ciaula Agostino, Portincasa Piero The environment as a determinant of successful aging or frailty. MECHANISMS OF AGEING AND DEVELOPMENT. 2020;188():.

6691. Jegatheesan Prasanthi, De Bandt Jean-Pascal Fructose and NAFLD: The Multifaceted Aspects of Fructose Metabolism. NUTRIENTS. 2017;9(3):.

6692. Wang Yongyi, Chen Baofu, Shen Dafu, Xue Song Osteopontin protects against cardiac ischemia-reperfusion injury through late preconditioning. HEART AND VESSELS. 2009;24(2):116-123.

6693. Marshall Teresa Dietary assessment and counseling for dental erosion. JOURNAL OF THE AMERICAN DENTAL ASSOCIATION. 2018;149(2):148-162.

6694. Schulz HM, Bechtel A, Rainer T, Sachsenhofer RF, Struck U Paleoceanography of the western central Paratethys during early oligocene nannoplankton zone NP23 in the Austrian Molasse Basin. GEOLOGICA CARPATHICA. 2004;55(4):311-323.

6695. Hall Peter, Bickel Warren, Erickson Kirk, Wagner Dylan Neuroimaging, neuromodulation, and population health: the neuroscience of chronic disease prevention. ANNALS OF THE NEW YORK ACADEMY OF SCIENCES. 2018;1428(1, SI):240-256.

6696. Carmichael SL, Shaw GM, Schaffer DM, Laurent C, Selvin S Dieting behaviors and risk of neural tube defects. AMERICAN JOURNAL OF EPIDEMIOLOGY. 2003;158(12):1127-1131.

6697. Mitchell JE, Hoberman HN, Peterson CB, Mussell M, Pyle RL Research on the psychotherapy of bulimia nervosa: Half empty or half full. INTERNATIONAL JOURNAL OF

EATING DISORDERS. 1996;20(3):219-229.

6698. Ksouri Rihab Food components and diet habits: chief factors of cancer development. FOOD QUALITY AND SAFETY. 2019;3(4):227-231.

6699. NISHIZAWA K, INOUE O, SAITO Y, SUZUKI A PROTECTIVE EFFECTS OF KAMIKIHI-TO, A TRADITIONAL CHINESE MEDICINE, AGAINST CEREBRAL-ISCHEMIA, HYPOXIA AND ANOXIA IN MICE AND GERBILS. JAPANESE JOURNAL OF PHARMACOLOGY. 1994;64(3):171-177.

6700. Vasquez Matsuda Victor, Bustelo Tejada Martin, Motta-Teixeira Livia, Ikebara Juliane, Cardoso Debora, Machado-Nils Aline, Lee Vitor, Diccini Isabelle, Arruda Bruna, Martins Pamela, Morales Dias Natalia, Tessarotto Rafaella, Raeisossadati Reza, Bruno Martin, Takase Luiz, Kihara Alexandre, Nogueira Maria, Xavier Gilberto, Takada Silvia Impact of neonatal anoxia and hypothermic treatment on development and memory of rats. EXPERIMENTAL NEUROLOGY. 2021;340():.

6701. He Zhonghu, Liu Zhen, Liu Mengfei, Guo Chuanhai, Xu Ruiping, Li Fenglei, Liu Anxiang, Yang Haijun, Shen Lin, Wu Qi, Duan Liping, Li Xiang, Zhang Chaoting, Pan Yaqi, Cai Hong, Ke Yang Efficacy of endoscopic screening for esophageal cancer in China (ESECC): design and preliminary results of a population-based randomised controlled trial. GUT. 2019;68(2):198+.

6702. Rosen Elissa, Sabel Allison, Brinton John, Catanach Brittany, Gaudiani Jennifer, Mehler Philip Liver dysfunction in patients with severe anorexia nervosa. INTERNATIONAL JOURNAL OF EATING DISORDERS. 2016;49(2):153-160.

6703. Ailanen Liisa, Vahatalo Laura, Salomaki-Myftari Henriikka, Makela Satu, Orpana Wendy, Ruohonen Suvi, Savontaus Eriika Peripherally Administered Y-2-Receptor Antagonist BIIE0246 Prevents Diet-Induced Obesity in Mice With Excess Neuropeptide Y, but Enhances Obesity in Control Mice. FRONTIERS IN PHARMACOLOGY. 2018;9():.

6704. Hochgraf Anna, McHale Susan, Fosco Gregory Interparental conflict and gender moderate the prospective link between parents' perceptions of adolescents' weight and weight concerns. INTERNATIONAL JOURNAL OF EATING DISORDERS. 2019;52(8):904-913.

6705. Avgoustiniatos E., Hering B., Rozak P., Wilson J., Tempelman L., Balamurugan A., Welch D., Weegman B., Suszynski T., Papas K. Commercially available gas-permeable cell

culture bags may not prevent anoxia in cultured or shipped islets. TRANSPLANTATION PROCEEDINGS. 2008;40(2):395-400.

6706. Jorgensen NK, Petersen SF, Damgaard I, Schousboe A, Hoffmann EK Increases in {[}Ca2+](i) and changes in intracellular pH during chemical anoxia in mouse neocortical neurons in primary culture. JOURNAL OF NEUROSCIENCE RESEARCH. 1999;56(4):358-370.

6707. Wignall Paul, Bond David The end-Triassic and Early Jurassic mass extinction records in the British Isles. PROCEEDINGS OF THE GEOLOGISTS ASSOCIATION. 2008;119(1):73-84.

6708. Thorndike Anne, McCurley Jessica, Gelsomin Emily, Anderson Emma, Chang Yuchiao, Porneala Bianca, Johnson Charles, Rimm Eric, Levy Douglas Automated Behavioral Workplace Intervention to Prevent Weight Gain and Improve Diet The ChooseWell 365 Randomized Clinical Trial. JAMA NETWORK OPEN. 2021;4(6):.

6709. Lavigne B., Audebert-Merilhou E., Buisson G., Kochman F., Clement J., Olliac B. Interpersonal therapy (IPT) in child psychiatry and adolescent. ENCEPHALE-REVUE DE PSYCHIATRIE CLINIQUE BIOLOGIQUE ET THERAPEUTIQUE. 2016;42(6):535-539.

6710. Elliot DL, Moe EL, Goldberg L, DeFrancesco CA, Durham MB, Hix-Small H Definition and outcome of a curriculum to prevent disordered eating and body-shaping drug use. JOURNAL OF SCHOOL HEALTH. 2006;76(2):67-73.

6711. Rogalska J, Danielisova V, Caputa M Effect of neonatal body temperature on postanoxic, potentially neurotoxic iron accumulation in the rat brain. NEUROSCIENCE LETTERS. 2006;393(2-3):249-254.

6712. Smythe Jodie, Colebourn Claire, Prisco Lara, Petrinic Tatjana, Leeson Paul Cardiac abnormalities identified with echocardiography in anorexia nervosa: systematic review and meta-analysis. BRITISH JOURNAL OF PSYCHIATRY. 2021;219(3):477-486.

6713. Geisbuhler Timothy Compartmentalization of non-adenine nucleotides in anoxic cardiac myocytes. BASIC RESEARCH IN CARDIOLOGY. 2008;103(1):31-40.

6714. Huber Gavin, Priest Sydney, Geisbuhler Timothy Cardioprotective Effect of Hydroxysafflor Yellow A via the Cardiac Permeability Transition Pore. PLANTA MEDICA.

2018;84(8):507-518.

6715. Keane Deirdre, Kelly Stacey, Healy Niamh, McArdle Maeve, Holohan Kieran, Roche Helen Diet and Metabolic Syndrome: An Overview. CURRENT VASCULAR PHARMACOLOGY. 2013;11(6):842-857.

6716. Godaux Damien, Bailleul Benjamin, Berne Nicolas, Cardol Pierre Induction of Photosynthetic Carbon Fixation in Anoxia Relies on Hydrogenase Activity and Proton-Gradient Regulation-Like1-Mediated Cyclic Electron Flow in Chlamydomonas reinhardtii. PLANT PHYSIOLOGY. 2015;168(2):648+.

6717. Kovalchik S. Comparative study of rate of weight loss among premorbid and healthy restrictive eaters. EATING AND WEIGHT DISORDERS-STUDIES ON ANOREXIA BULIMIA AND OBESITY. 2008;13(4):176-182.

6718. Marilov V., Sologub M. Comparative effectiveness of mood stabilizers in the complex therapy of bulimia nervosa. ZHURNAL NEVROLOGII I PSIKHIATRII IMENI S S KORSAKOVA. 2010;110(1):59-61.

6719. Giel Katrin, Martus Peter, Schag Kathrin, Herpertz Stephan, Hofmann Tobias, Schneider Antonius, Teufel Martin, Voderholzer Ulrich, Wietersheim Jorn, Wild Beate, Zeeck Almut, Bethge Wolfgang, Schmidt Ulrike, Zipfel Stephan, Junne Florian Specialized post-inpatient psychotherapy for sustained recovery in anorexia nervosa via videoconference - study protocol of the randomized controlled SUSTAIN trial. JOURNAL OF EATING DISORDERS. 2021;9(1):.

6720. Corbett James, Kappagoda Manel Doing Good and Doing Well: Corporate Social Responsibility in Post Obamacare America. JOURNAL OF LAW MEDICINE \& ETHICS. 2013;41(1, SI):17-21.

6721. Zabinski MF, Calfas KJ, Gehrman CA, Wilfley DE, Sallis JF Effects of a physical activity intervention on body image in university seniors: Project GRAD. ANNALS OF BEHAVIORAL MEDICINE. 2001;23(4):247-252.

6722. Yang Chuang, Jiang Guopan, Xing Yue Protective Effect of Ginsenosides Rg1 on Ischemic Injury of Cardiomyocytes After Acute Myocardial Infarction. CARDIOVASCULAR TOXICOLOGY. 2022;22(10-11):910-915.

6723. Covi JA, Treleaven WD, Hand SC V-ATPase inhibition prevents recovery from anoxia in Artemia franciscana embryos: quiescence signaling through dissipation of proton gradients (vol 208, pg 2779, 2005). JOURNAL OF EXPERIMENTAL BIOLOGY. 2005;208(20):3997.

6724. Kwiatkowski Sebastian, Kajdy Anna, Stefanska Katarzyna, Bednarek-Jedrzejek Magdalena, Dzidek Sylwia, Tousty Piotr, Sokolowska Malgorzata, Kwiatkowska Ewa PPAR gamma-A Factor Linking Metabolically Unhealthy Obesity with Placental Pathologies. INTERNATIONAL JOURNAL OF MOLECULAR SCIENCES. 2021;22(23):.

6725. Yokose Chio, McCormick Natalie, Choi Hyon Dietary and Lifestyle-Centered Approach in Gout Care and Prevention. CURRENT RHEUMATOLOGY REPORTS. 2021;23(7):.

6726. Kraak V., Story M. Influence of food companies' brand mascots and entertainment companies' cartoon media characters on children's diet and health: a systematic review and research needs. OBESITY REVIEWS. 2015;16(2):107-126.

6727. Clarke J., Fletcher B., Lancashire E., Pallan M., Adab P. The views of stakeholders on the role of the primary school in preventing childhood obesity: a qualitative systematic review. OBESITY REVIEWS. 2013;14(12):975-988.

6728. Zheng Yan, Ley Sylvia, Hu Frank Global aetiology and epidemiology of type 2 diabetes mellitus and its complications. NATURE REVIEWS ENDOCRINOLOGY. 2018;14(2):88-98.

6729. Wang Gene-Jack, Volkow Nora, Thanos Panayotis, Fowler Joanna Imaging of Brain Dopamine Pathways Implications for Understanding Obesity. JOURNAL OF ADDICTION MEDICINE. 2009;3(1):8-18.

6730. Sola-Garcia Alejandro, Angeles Caliz-Molina Maria, Espadas Isabel, Petr Michael, Panadero-Moron Concepcion, Gonzalez-Moran Daniel, Eugenia Martin-Vazquez Maria, Jesus Narbona-Perez Alvaro, Lopez-Noriega Livia, Martinez-Corrales Guillermo, Lopez-Fernandez-Sobrino Raul, Carmona-Marin Lina, Martinez-Force Enrique, Yanes Oscar, Vinaixa Maria, Lopez-Lopez Daniel, Carlos Reyes Jose, Dopazo Joaquin, Martin Franz, Gauthier Benoit, Scheibye-Knudsen Morten, Capilla-Gonzalez Vivian, Martin-Montalvo Alejandro Metabolic reprogramming by Acly inhibition using SB-204990 alters glucoregulation and modulates molecular mechanisms associated with aging. COMMUNICATIONS BIOLOGY. 2023;6(1):.

6731. Werling Klara, Lango Aniko Effects of physical activity in nonalcoholic fatty liver disease. ORVOSI HETILAP. 2020;161(6):203-207.

6732. Turrini Aida Perspectives of Dietary Assessment in Human Health and Disease. NUTRIENTS. 2022;14(4):.

6733. Ozcan Cevher, Terzic Andre, Bienengraeber Martin Effective pharmacotherapy against oxidative injury: Alternative utility of an ATP-sensitive potassium channel opener. JOURNAL OF CARDIOVASCULAR PHARMACOLOGY. 2007;50(4):411-418.

6734. Wilksch Simon Toward a more comprehensive understanding and support of parents with a child experiencing an eating disorder. INTERNATIONAL JOURNAL OF EATING DISORDERS. 2023;():.

6735. Nallely Lopez-Contreras Iris, Vilchis-Gil Jenny, Klunder-Klunder Miguel, Villalpando-Carrion Salvador, Flores-Huerta Samuel Dietary habits and metabolic response improve in obese children whose mothers received an intervention to promote healthy eating: randomized clinical trial. BMC PUBLIC HEALTH. 2020;20(1):.

6736. Feldman Charles, Hartwell Heather, Brusca Joseph, Su Haiyan, Zhao Hang Nutrition information and its influence on menu choice within higher education establishments. BRITISH FOOD JOURNAL. 2015;117(4):1399-1410.

6737. Xu Tingting, Nerren Jannah Investigating Young Children's Perceptions of Body Size and Healthy Habits. EARLY CHILDHOOD EDUCATION JOURNAL. 2017;45(4):499-507.

6738. Chauhan Pallavi, Yadav Dhananjay, Arukha Ananta Dietary Nutrients and Prevention of Alzheimer's Disease. CNS \& NEUROLOGICAL DISORDERS-DRUG TARGETS. 2022;21(3):217-227.

6739. Martinez-Gonzalez Leticia, Fernandez-Villa Tania, Jose Molina Antonio, Delgado-Rodriguez Miguel, Martin Vicente Incidence of Anorexia Nervosa in Women: A Systematic Review and Meta-Analysis. INTERNATIONAL JOURNAL OF ENVIRONMENTAL RESEARCH AND PUBLIC HEALTH. 2020;17(11):.

6740. Almeida Mauricio, Brown Tiffany, Campos Priscila, Amaral Ana, Carvalho Pedro Dissonance-based eating disorder prevention delivered in-person after an online training: A

randomized controlled trial for Brazilian men with body dissatisfaction. INTERNATIONAL JOURNAL OF EATING DISORDERS. 2021;54(3):293-304.

6741. Ebenegger Vincent, Marques-Vidal Pedro-Manuel, Munsch Simone, Quartier Vincent, Nydegger Andreas, Barral Jerome, Hartmann Tim, Dubnov-Raz Gal, Kriemler Susi, Puder Jardena Relationship of Hyperactivity/Inattention With Adiposity and Lifestyle Characteristics in Preschool Children. JOURNAL OF CHILD NEUROLOGY. 2012;27(7):852-858.

6742. Afflelou S. Anorexia athletica's place in female sports athletes. ARCHIVES DE PEDIATRIE. 2009;16(1):88-92.

6743. Grammatikopoulou Maria, Lampropoulou Maria, Milapidou Maria, Goulis Dimitrios At the heart of the matter: Cardiovascular health challenges among incarcerated women. MATURITAS. 2021;149():16-25.

6744. Koch Susanne, Andersson Mikael, Hvelplund Carolina, Skovgaard Anne Mental disorders in referred 0-3-year-old children: a population-based study of incidence, comorbidity and perinatal risk factors. EUROPEAN CHILD \& ADOLESCENT PSYCHIATRY. 2021;30(8):1251-1262.

6745. Viner R., Cole T. Who changes body mass between adolescence and adulthood? Factors predicting change in BMI between 16 year and 30 years in the 1970 British Birth Cohort. INTERNATIONAL JOURNAL OF OBESITY. 2006;30(9):1368-1374.

6746. Yao Hanwei, Chen Xi, Yin Runsheng, Grasby Stephen, Weissert Helmut, Gu Xue, Wang Chengshan Mercury Evidence of Intense Volcanism Preceded Oceanic Anoxic Event 1d. GEOPHYSICAL RESEARCH LETTERS. 2021;48(5):.

6747. Rui T, Kvietys PR Anoxia/reoxygenation-induced pro-inflammatory phenotype in cardiac myocytes is prevented by Erythropoietin via a PI3-kinase/NF kappa B signaling pathway. FASEB JOURNAL. 2005;19(4, 1, S):A711.

6748. Doyle Angela, Goldschmidt Andrea, Huang Christina, Winzelberg Andrew, Taylor C., Wilfley Denise Reduction of overweight and eating disorder symptoms via the Internet in adolescents: A randomized controlled trial. JOURNAL OF ADOLESCENT HEALTH. 2008;43(2):172-179.

6749. Ali Lamiaa, Kader Nefissa, Mahgoub Nelly Dietary/Exercise Guiding Program for Improvement of Nursing Students Body Image, Self-Esteem and Overweight Prevention: A Randomized Controlled study. PAKISTAN JOURNAL OF MEDICAL \& HEALTH SCIENCES. 2021;15(1):388-392.

6750. Dowd W., Renshaw Gillian, Cech Joseph, Kueltz Dietmar Compensatory proteome adjustments imply tissue-specific structural and metabolic reorganization following episodic hypoxia or anoxia in the epaulette shark (Hemiscyllium ocellatum). PHYSIOLOGICAL GENOMICS. 2010;42(1):93-114.

6751. De Bosschere H, Bos M, Ducatelle R Apoptotic neurons in a cat with cerebellar abiotrophy. VLAAMS DIERGENEESKUNDIG TIJDSCHRIFT. 2000;69(5):341-344.

6752. TORREY EF, TAYLOR EH, BRACHA HS, BOWLER AE, MCNEIL TF, RAWLINGS RR, QUINN PO, BIGELOW LB, RICKLER K, SJOSTROM K, HIGGINS ES, GOTTESMAN II PRENATAL ORIGIN OF SCHIZOPHRENIA IN A SUBGROUP OF DISCORDANT MONOZYGOTIC TWINS. SCHIZOPHRENIA BULLETIN. 1994;20(3):423-432.

6753. Francisco GE, Hu MM, Boake C, Ivanhoe CB Efficacy of early use of intrathecal baclofen therapy for treating spastic hypertonia due to acquired brain injury. BRAIN INJURY. 2005;19(5):359-364.

6754. Brotons Carlos, Drenthen Antonius, Durrer Dominique, Moral Irene, Hlth European Beliefs and attitudes to lifestyle, nutrition and physical activity: the views of patients in Europe. FAMILY PRACTICE. 2012;29(1):i49-i55.

6755. Convertino Alexandra, Elbe Connor, Mendoza Rebecca, Calzo Jerel, Brown Tiffany, Siegel Jaclyn, Jun Hee-Jin, Corliss Heather, Blashill Aaron Internalization of muscularity and thinness ideals: Associations with body dissatisfaction, eating disorder symptoms, and muscle dysmorphic symptoms in at risk sexual minority men. INTERNATIONAL JOURNAL OF EATING DISORDERS. 2022;55(12):1765-1776.

6756. Tabuchi K, Ito Z, Tsuji S, Nakagawa A, Serizawa F, Hara A, Kusakari J Poly(adenosine diphosphate-ribose) synthetase inhibitor 3-aminobenzamide alleviates cochlear dysfunction induced by transient ischemia. ANNALS OF OTOLOGY RHINOLOGY AND LARYNGOLOGY. 2001;110(2):118-121.

6757. Nichols Hazel, Trentham-Dietz Amy, Newcomb Polly, Yanke Lucinda, Remington Patrick, Love Richard What causes cancer? Reports from sixth-grade girls. JOURNAL OF CANCER EDUCATION. 2006;21(3):142-146.

6758. Lydecker Janet, Grilo Carlos The Apple of Their Eye: Attitudinal and Behavioral Correlates of Parents' Perceptions of Child Obesity. OBESITY. 2016;24(5):1124-1131.

6759. Frank Guido, DeGuzman Marisa, Shott Megan, Laudenslager Mark, Rossi Brogan, Pryor Tamara Association of Brain Reward Learning Response With Harm Avoidance, Weight Gain, and Hypothalamic Effective Connectivity in Adolescent Anorexia Nervosa. JAMA PSYCHIATRY. 2018;75(10):1071-1080.

6760. Becker Carolyn, McDaniel Leda, Bull Stephanie, Powell Marc, McIntyre Kevin Can we reduce eating disorder risk factors in female college athletes? A randomized exploratory investigation of two peer-led interventions. BODY IMAGE. 2012;9(1):31-42.

6761. Ficker Harald, Luger Martin, Gassner Hubert From dimictic to monomictic: Empirical evidence of thermal regime transitions in three deep alpine lakes in Austria induced by climate change. FRESHWATER BIOLOGY. 2017;62(8):1335-1345.

6762. Feldhege Johannes, Moessner Markus, Wolf Markus, Bauer Stephanie Changes in Language Style and Topics in an Online Eating Disorder Community at the Beginning of the COVID-19 Pandemic: Observational Study. JOURNAL OF MEDICAL INTERNET RESEARCH. 2021;23(7):.

6763. Machado Paulo, Pinto-Bastos Ana, Ramos Rita, Rodrigues Tania, Louro Elsa, Goncalves Sonia, Brandao Isabel, Vaz Ana Impact of COVID-19 lockdown measures on a cohort of eating disorders patients. JOURNAL OF EATING DISORDERS. 2020;8(1):.

6764. Ireland Alana, Russell-Mayhew Shelly, Wulff Dan, Strong Tom `One-size-fits-none': a situational analysis of weight-related issues in schools. INTERNATIONAL JOURNAL OF QUALITATIVE STUDIES IN EDUCATION. 2023;36(6):1155-1174.

6765. Rashidpour Parvane, Poursharif Zahra, Ayatollahi Mina, Farzannejad Elham Bupropion-induced acute dystonia in a patient with bulimia nervosa: A case report. JOURNAL OF FAMILY MEDICINE AND PRIMARY CARE. 2021;10(2):1034-1036.

6766. Levine Michael Prevention of eating disorders: 2019 in review. EATING DISORDERS. 2020;28(1):6-20.

6767. Duan Ming-Jie, Dekker Louise, Carrero Juan-Jesus, Navis Gerjan Using Structural Equation Modeling to Untangle Pathways of Risk Factors Associated with Incident Type 2 Diabetes: the Lifelines Cohort Study. PREVENTION SCIENCE. 2022;23(7):1090-1100.

6768. Hill SA, Milam M, Manaligod JM Tracheal agenesis: diagnosis and management. INTERNATIONAL JOURNAL OF PEDIATRIC OTORHINOLARYNGOLOGY. 2001;59(1):63-68.

6769. Zeis B, Becher B, Goldmann T, Clark R, Vollmer E, Bolke B, Bredebusch I, Lamkemeyer T, Pinkhaus O, Pirow R, Paul RJ Differential haemoglobin gene expression in the crustacean Daphnia magna exposed to different oxygen partial pressures. BIOLOGICAL CHEMISTRY. 2003;384(8):1133-1145.

6770. Monje ML, Chatten-Brown J, Hye SE, Raley-Susman KM Free radicals are involved in the damage to protein synthesis after anoxia/aglycemia and NMDA exposure. BRAIN RESEARCH. 2000;857(1-2):172-182.

6771. Radden Jennifer Starving to death and the anorexic frame of mind. TRANSCULTURAL PSYCHIATRY. 2022;59(3):302-311.

6772. Mellentin A., Nielsen D., Skot L., Stoving R., Guala M., Nielsen A., Wesselhoeft R., Mejldal A. Risk of somatic diseases in patients with eating disorders: the role of comorbid substance use disorders. EPIDEMIOLOGY AND PSYCHIATRIC SCIENCES. 2022;31():.

6773. Buyukakilli Belgin, Atici Aytug, Buyukdereli Zekeriya, Tasdelen Bahar, Gunes Sevgi, Turhan Ali Protective Effects of Platelet-Activating Factor Antagonist ABT-491 on the Peripheral Nerves in Hypoxic Ischemia-Induced Neonatal Rat Model. TURKIYE KLINIKLERI TIP BILIMLERI DERGISI. 2011;31(5):1179-1185.

6774. Steinhausen Hans-Christoph, Grigoroiu-Serbanescu Maria, Boyadjieva Svetlana, Neumaerker Klaus-Juergen, Metzke Christa The Relevance of Body Weight in the Medium-Term to Long-Term Course of Adolescent Anorexia Nervosa. Findings from a Multisite Study. INTERNATIONAL JOURNAL OF EATING DISORDERS. 2009;42(1):19-25.

6775. Haruma Ken, Kinoshita Yoshikazu, Sakamoto Shigeru, Sanada Katsuyuki, Hiroi Shinzo, Miwa Hiroto Lifestyle Factors and Efficacy of Lifestyle Interventions in Gastroesophageal Reflux Disease Patients with Functional Dyspepsia: Primary Care Perspectives from the LEGEND Study. INTERNAL MEDICINE. 2015;54(7):695-701.

6776. Altwaijri Yasmin, Al-Subaie Abdullah, Al-Habeeb Abdulhameed, Bilal Lisa, Al-Desouki Majid, Aradati Maggie, King Andrew, Sampson Nancy, Kessler Ronald Lifetime prevalence and age-of-onset distributions of mental disorders in the Saudi National Mental Health Survey. INTERNATIONAL JOURNAL OF METHODS IN PSYCHIATRIC RESEARCH. 2020;29(3):.

6777. Jelsma Judith, Poppel Mireille, Smith Ben, Cinnadaio Nancy, Bauman Adrian, Tapsell Linda, Cheung N., Ploeg Hidde Changing psychosocial determinants of physical activity and diet in women with a history of gestational diabetes mellitus. DIABETES-METABOLISM RESEARCH AND REVIEWS. 2018;34(1):.

6778. Carter FA, Bulik CM, McIntosh VV, Joyce PR Changes on the stroop test following treatment: Relation to word type, treatment condition, and treatment outcome among women with bulimia nervosa. INTERNATIONAL JOURNAL OF EATING DISORDERS. 2000;28(4):349-355.

6779. Striegel-Moore RH, Cachelin FM Etiology of eating disorders in women. COUNSELING PSYCHOLOGIST. 2001;29(5):635-661.

6780. Dokken Betsy, Piermarini Charles, Asghar Mostafa, Teachey Mary, Gura Michael, Dameff Christian, Heller Brian, Hilwig Ronald, Kern Karl Glucagon-like peptide-1 (GLP-1) improves coronary microvascular endothelial function after cardiac arrest and resuscitation and prevents anoxia-induced coronary microvascular endothelial cell death. FASEB JOURNAL. 2011;25():.

6781. Barzilay Shira, Apter Alan Predictors of suicide in adolescents and adults with mood and common comorbid disorders. NEUROPSYCHIATRY. 2014;4(1):81-93.

6782. Hsu LKG, Rand W, Sullivan S, Liu DW, Mulliken B, McDonagh B, Kaye WH Cognitive therapy, nutritional therapy and their combination in the treatment of bulimia nervosa. PSYCHOLOGICAL MEDICINE. 2001;31(5):871-879.

6783. Williams Brenna, Levinson Cheri Negative beliefs about the self prospectively predict eating disorder severity among undergraduate women. EATING BEHAVIORS. 2020;37():.

6784. Vall Eva, Wade Tracey Predictors and moderators of outcomes and readmission for adolescent inpatients with anorexia nervosa: A pilot study. CLINICAL PSYCHOLOGIST. 2017;21(2, SI):143-152.

6785. Gyori Dora, Balazs Judit Nonsuicidal Self-Injury and Perfectionism: A Systematic Review. FRONTIERS IN PSYCHIATRY. 2021;12():.

6786. Zhou Jie, Ho Chi-Tang, Long Piaopiao, Meng Qilu, Zhang Liang, Wan Xiaochun Preventive Efficiency of Green Tea and Its Components on Nonalcoholic Fatty Liver Disease. JOURNAL OF AGRICULTURAL AND FOOD CHEMISTRY. 2019;67(19):5306-5317.

6787. CHILDRESS AC, BREWERTON TD, HODGES EL, JARRELL MP THE KIDS EATING DISORDERS SURVEY (KEDS) - A STUDY OF MIDDLE SCHOOL STUDENTS. JOURNAL OF THE AMERICAN ACADEMY OF CHILD AND ADOLESCENT PSYCHIATRY. 1993;32(4):843-850.

6788. Guo Hongliang, Shen Xiaoran, Xu Ye, He Youdi, Hu Wenli The effect of activin A on signal transduction pathways in PC12 cells subjected to oxygen and glucose deprivation. INTERNATIONAL JOURNAL OF MOLECULAR MEDICINE. 2014;33(1):135-141.

6789. Makri Stamatina, Lami Andrea, Tu Luyao, Tylmann Wojciech, Vogel Hendrik, Grosjean Martin Holocene phototrophic community and anoxia dynamics in meromictic Lake Jaczno (NE Poland) using high-resolution hyperspectral imaging and HPLC data. BIOGEOSCIENCES. 2021;18(5):1839-1856.

6790. Rewers Arleta Current Concepts and Controversies in Prevention and Treatment of Diabetic Ketoacidosis in Children. CURRENT DIABETES REPORTS. 2012;12(5):524-532.

6791. Lee KW, Lee HJ, Lee CY Vitamins, phytochemicals, diets and their implementation in cancer chemoprevention. CRITICAL REVIEWS IN FOOD SCIENCE AND NUTRITION. 2004;44(6):437-452.

6792. Hinney A, Bornscheuer A, Depenbusch M, Mierke B, Tolle A, Middeke K, Ziegler A, Roth H, Gerber G, Zamzow K, Ballauff A, Hamann A, Mayer H, Siegfried W, Lehmkuhl G, Poustka F, Schmidt MH, Hermann H, Herpertz-Dahlmann BM, Fichter M, Remschmidt H,

Hebebrand J No evidence for involvement of the leptin gene in anorexia nervosa, bulimia nervosa, underweight or early onset extreme obesity: identification of two novel mutations in the coding sequence and a novel polymorphism in the leptin gene linked upstream region. MOLECULAR PSYCHIATRY. 1998;3(6):539-543.

6793. Gordon I, Lask B, BryantWaugh R, Christie D, Timimi S Childhood-onset anorexia nervosa: Towards identifying a biological substrate. INTERNATIONAL JOURNAL OF EATING DISORDERS. 1997;22(2):159-165.

6794. Chang Zhigang, Li Yongqing, He Wei, Liu Baoling, Duan Xiuzhen, Halaweish Ihab, Bambakidis Ted, Pan Baihong, Liang Yingjian, Nikolian Vahagn, Georgoff Patrick, Alam Hasan Inhibition of histone deacetylase 6 restores intestinal tight junction in hemorrhagic shock. JOURNAL OF TRAUMA AND ACUTE CARE SURGERY. 2016;81(3):512-519.

6795. Cheatley Jane, Aldea Alexandra, Lerouge Alienor, Devaux Marion, Vuik Sabine, Cecchini Michele Tackling the cancer burden: the economic impact of primary prevention policies. MOLECULAR ONCOLOGY. 2021;15(3):779-789.

6796. Jahng Jeong, Kim Jae, Kim Hyoung, Kang Dong-Won, Lee Jong-Ho Chronic food restriction in young rats results in depression- and anxiety-like behaviors with decreased expression of serotonin reuptake transporter. BRAIN RESEARCH. 2007;1150():100-107.

6797. Dolbier Christyn, Haley Erin, Conder Lauren, Guiler William Adverse childhood experiences and adult psychopathological symptoms: The moderating role of dispositional mindfulness. JOURNAL OF CONTEXTUAL BEHAVIORAL SCIENCE. 2021;21():73-79.

6798. Wignall Paul, Bond David, Kuwahara Kiyoko, Kakuwa Yoshitaka, Newton Robert, Poulton Simon An 80 million year oceanic redox history from Permian to Jurassic pelagic sediments of the Mino-Tamba terrane, SW Japan, and the origin of four mass extinctions. GLOBAL AND PLANETARY CHANGE. 2010;71(1-2):109-123.

6799. Jhe Grace, Lin Jessica, Freizinger Melissa, Richmond Tracy Adolescents with anorexia nervosa or atypical anorexia nervosa with premorbid overweight/obesity: What should we do about their weight loss?. JOURNAL OF CHILD AND ADOLESCENT PSYCHIATRIC NURSING. 2023;36(1):55-58.

6800. Pitcher Grant, Probyn Trevor, Randt Andre, Lucas Andrew, Bernard Stewart, Evers-King Haley, Lamont Tarron, Hutchings Larry Dynamics of oxygen depletion in the nearshore

of a coastal embayment of the southern Benguela upwelling system. JOURNAL OF GEOPHYSICAL RESEARCH-OCEANS. 2014;119(4):2183-2200.

6801. Peng Li, Jian-hua Fu, Jing-kun Wang, Jun-guo Ren, Jian-xun Liu Extract of Paris polyphylia Simth Protects Cardiomyocytes from Anoxia-Reoxia Injury through Inhibition of Calcium Overload. CHINESE JOURNAL OF INTEGRATIVE MEDICINE. 2011;17(4):283-289.

6802. Quach Jackie, Porter Kyle, Leventhal Howard, Kelly Kimberly Health behaviors among Ashkenazi Jewish individuals receiving counseling for BRCA1 and BRCA2 mutations. FAMILIAL CANCER. 2009;8(3):241-250.

6803. Moubarac Jean-Claude, Bortoletto Martins Ana, Claro Rafael, Levy Renata, Cannon Geoffrey, Monteiro Carlos Consumption of ultra-processed foods and likely impact on human health. Evidence from Canada. PUBLIC HEALTH NUTRITION. 2013;16(12):2240-2248.

6804. Akcan Gizem, Uhras Seda, Comert Itir, Yukseloglu Hulya, Islek Dilek, Tutkun Erkut Investigation of the Relationship between Food Addiction and Anxiety, Depression and Attention Deficit / Hyperactivity in Obese and Non-Obese. PROGRESS IN NUTRITION. 2021;23(1):.

6805. Eiken Ola, Gronkvist Mikael Signs and Symptoms During Supra-Tolerance +G(z) Exposures, with Reference to G-Garment Failure. AVIATION SPACE AND ENVIRONMENTAL MEDICINE. 2013;84(3):196-205.

6806. Griffiths P, Bentley M Women of higher socio-economic status are more likely to be overweight in Karnataka, India. EUROPEAN JOURNAL OF CLINICAL NUTRITION. 2005;59(10):1217-1220.

6807. Montemayor Sofia, Mascaro Catalina, Ugarriza Lucia, Casares Miguel, Llompart Isabel, Abete Itziar, Angeles Zulet Maria, Alfredo Martinez J., Tur Josep, Bouzas Cristina Adherence to Mediterranean Diet and NAFLD in Patients with Metabolic Syndrome: The FLIPAN Study. NUTRIENTS. 2022;14(15):.

6808. Cimino Silvia, Cerniglia Luca, Almenara Carlos, Jezek Stanislav, Erriu Michela, Tambelli Renata Developmental trajectories of body mass index and emotional-behavioral functioning of underweight children: A longitudinal study. SCIENTIFIC REPORTS. 2016;6():.

6809. Kilpela Lisa, Schaumberg Katherine, Hopkins Lindsey, Becker Carolyn Mechanisms of action during a dissonance-based intervention through 14-month follow-up: The roles of body shame and body surveillance. BODY IMAGE. 2017;23():171-175.

6810. Wieczorek Rebecca, Fantle Matthew, Kump Lee, Ravizza Gregory Geochemical evidence for volcanic activity prior to and enhanced terrestrial weathering during the Paleocene Eocene Thermal Maximum. GEOCHIMICA ET COSMOCHIMICA ACTA. 2013;119():391-410.

6811. Mette Wolfgang Upper Permian and lowermost Triassic stratigraphy, facies and ostracods in NW Iran - implications for the P/T extinction event. STRATIGRAPHY. 2008;5(2):205-219.

6812. Ouwens M., Strien T., Leeuwe J., Staak C. The dual pathway model of overeating. Replication and extension with actual food consumption. APPETITE. 2009;52(1):234-237.

6813. Barrea Luigi, Marzullo Paolo, Muscogiuri Giovanna, Di Somma Carolina, Scacchi Massimo, Orio Francesco, Aimaretti Gianluca, Colao Annamaria, Savastano Silvia Source and amount of carbohydrate in the diet and inflammation in women with polycystic ovary syndrome. NUTRITION RESEARCH REVIEWS. 2018;31(2):291-301.

6814. MESSIHA FS FLUOXETINE - A SPECTRUM OF CLINICAL-APPLICATIONS AND POSTULATES OF UNDERLYING MECHANISMS. NEUROSCIENCE AND BIOBEHAVIORAL REVIEWS. 1993;17(4):385-396.

6815. MARRIOTT PF, GREENWOOD KM, ARMSTRONG SM SEASONALITY IN PANIC DISORDER. JOURNAL OF AFFECTIVE DISORDERS. 1994;31(2):75-80.

6816. Allen Karina, Byrne Susan, Blair Eve, Davis Elizabeth Why do some overweight children experience psychological problems? The role of weight and shape concern. INTERNATIONAL JOURNAL OF PEDIATRIC OBESITY. 2006;1(4):239-247.

6817. Park Yong-Moon, White Alexandra, Nichols Hazel, O'Brien Katie, Weinberg Clarice, Sandler Dale The association between metabolic health, obesity phenotype and the risk of breast cancer. INTERNATIONAL JOURNAL OF CANCER. 2017;140(12):2657-2666.

6818. Herbert Cornelia Oral health and mental health in healthy adults, a topic of primary prevention and health care, empirical results from two online studies. CURRENT PSYCHOLOGY. 2023;():.

6819. Carter FA, Bulik CM, McIntosh VV, Joyce PR Cue reactivity as a predictor of outcome with bulimia nervosa. INTERNATIONAL JOURNAL OF EATING DISORDERS. 2002;31(3):240-250.

6820. Schots Pauke, Jansen Kirsten, Mrazek Jakub, Pedersen Alice, Olsen Ragnar, Larsen Terje Obesity-induced alterations in the gut microbiome in female mice fed a high-fat diet are antagonized by dietary supplementation with a novel, wax ester-rich, marine oil. NUTRITION RESEARCH. 2020;83():94-107.

6821. Young GP Colorectal disorders: A dietary management perspective. ASIA PACIFIC JOURNAL OF CLINICAL NUTRITION. 2000;9(S):S76-S82.

6822. Twitchett RJ, Krystyn L, Baud A, Wheeley JR, Richoz S Rapid marine recovery after the end-Permian mass-extinction event in the absence of marine anoxia. GEOLOGY. 2004;32(9):805-808.

6823. Ndubuisi Nweke Noncommunicable Diseases Prevention In Low- and Middle-Income Countries: An Overview of Health in All Policies (HiAP). INQUIRY-THE JOURNAL OF HEALTH CARE ORGANIZATION PROVISION AND FINANCING. 2021;58():.

6824. Lake Bjorn, Coolidge Kyle, Norton Stephen, Amirbahman Aria Factors contributing to the internal loading of phosphorus from anoxic sediments in six Maine, USA, lakes. SCIENCE OF THE TOTAL ENVIRONMENT. 2007;373(2-3):534-541.

6825. White Michelle, Holliday Katelyn, Hoover Stephanie, Robinson-Ezekwe Nicole, Corbie-Smith Giselle, Williams Anissa, Bess Kiana, Frerichs Leah The significant places of African American adults and their perceived influence on cardiovascular disease risk behaviors. BMC PUBLIC HEALTH. 2021;21(1):.

6826. Cipriano Annarosa, Aprea Cristina, Bellone Ludovica, Cotrufo Paolo, Cella Stefania Non-Suicidal Self-Injury: A School-Based Peer Education Program for Adolescents During COVID-19 Pandemic. FRONTIERS IN PSYCHIATRY. 2022;12():.

6827. Reilly Erin, Bohrer Brittany, Sullivan Daniel, Essayli Jamal, Farrell Nicholas, Brown Tiffany, Gorrell Sasha, Anderson Lisa, Cooper Marita, Schreyer Colleen, Olesnycky Olenka, Peros Olivia, Schaumberg Katherine Registered report: Initial development and validation of the eating disorders safety behavior scale. INTERNATIONAL JOURNAL OF EATING DISORDERS. 2021;54(4):660-667.

6828. Jha A, Dasgupta S, Deshpande SB Effect of ischaemia \& aglycaemia on the synaptic transmission in neonatal rat spinal cord in vitro. INDIAN JOURNAL OF MEDICAL RESEARCH. 2003;118():172-177.

6829. Khasteganan Nazanin, Lycett Deborah, Furze Gill, Turner Andy Health, not weight loss, focused programmes versus conventional weight loss programmes for cardiovascular risk factors: a systematic review and meta-analysis. SYSTEMATIC REVIEWS. 2019;8(1):.

6830. Klein David, Paradise Scott, Reeder Rachel Amenorrhea: A Systematic Approach to Diagnosis and Management. AMERICAN FAMILY PHYSICIAN. 2019;100(1):39-48.

6831. Tan Sidhartha Fault and Blame, Insults to the Perinatal Brain may be Remote from Time of Birth. CLINICS IN PERINATOLOGY. 2014;41(1):105+.

6832. Goldstone A., Holland A., Butler J., Whittington J. Appetite hormones and the transition to hyperphagia in children with Prader-Willi syndrome. INTERNATIONAL JOURNAL OF OBESITY. 2012;36(12):1564-1570.

6833. Dahl Tais, Boyle Richard, Canfield Donald, Connelly James, Gill Benjamin, Lenton Timothy, Bizzarro Martin Uranium isotopes distinguish two geochemically distinct stages during the later Cambrian SPICE event. EARTH AND PLANETARY SCIENCE LETTERS. 2014;401():313-326.

6834. Levine Michael, Sadeh-Sharvit Shiri Preventing eating disorders and disordered eating in genetically vulnerable, high-risk families. INTERNATIONAL JOURNAL OF EATING DISORDERS. 2023;56(3):523-534.

6835. Kong Weng, Tsuyama Naohiro, Inoue Hiroko, Guo Yun, Mokuda Sho, Nobukiyo Asako, Nakatani Nobuhiro, Yamaide Fumiya, Nakano Taiji, Kohno Yoichi, Ikeda Kazutaka, Nakanishi Yumiko, Ohno Hiroshi, Arita Makoto, Shimojo Naoki, Kanno Masamoto Long-chain saturated fatty acids in breast milk are associated with the pathogenesis of atopic

dermatitis via induction of inflammatory ILC3s. SCIENTIFIC REPORTS. 2021;11(1):.

6836. DESSI F, BENARI Y, CHARRIAUTMARLANGUE C RILUZOLE PREVENTS ANOXIC INJURY IN CULTURED CEREBELLAR GRANULE NEURONS. EUROPEAN JOURNAL OF PHARMACOLOGY. 1993;250(2):325-328.

6837. Azorin Jean-Michel, Belzeaux Raoul, Fakra Eric, Kaladjian Arthur, Hantouche Elie, Lancrenon Sylvie, Adida Marc Gender differences in a cohort of major depressive patients: Further evidence for the male depression syndrome hypothesis. JOURNAL OF AFFECTIVE DISORDERS. 2014;167():85-92.

6838. Clarke Julia, Peyre Hugo, Alison Marianne, Bargiacchi Anne, Stordeur Coline, Boizeau Priscilla, Mamou Gregor, Crepon Sophie, Alberti Corinne, Leger Juliane, Delorme Richard Abnormal bone mineral density and content in girls with early-onset anorexia nervosa. JOURNAL OF EATING DISORDERS. 2021;9(1):.

6839. Lehrmann Daniel, Stepchinski Leanne, Wolf Hannah, Li Liangzi, Li Xiaowei, Minzoni Marcello, Yu Meiyi, Payne Jonathan The role of carbonate factories and sea water chemistry on basin-wide ramp to high-relief carbonate platform evolution: Triassic, Nanpanjiang Basin, South China. DEPOSITIONAL RECORD. 2022;8(2):386-418.

6840. Perez-Pinzon MA, Born JG Rapid preconditioning neuroprotection following anoxia in hippocampal slices: Role of the K-ATP(+) channel and protein kinase C. NEUROSCIENCE. 1999;89(2):453-459.

6841. Amarnath Sumathi, Selvamani Meganathan, Varadarajan Vijayakumar Prognosis Model for Gestational Diabetes Using Machine Learning Techniques. SENSORS AND MATERIALS. 2021;33(9, SI):3011-3025.

6842. Varnado-Sullivan PJ, Zucker N, Williamson DA, Reas D, Thaw J, Netemeyer SB Development and implementation of the body logic program for adolescents: A two-stage prevention program for eating disorders. COGNITIVE AND BEHAVIORAL PRACTICE. 2001;8(3):248-259.

6843. Alves Mariane, Retondario Anabelle, Bricarello Liliana, Fernandes Ricardo, Souza Amanda, Zeni Lucia, Trindade Erasmo, Vasconcelos Francisco Association between dietary patterns and overweight/obesity: a Brazilian national school-based research (ERICA 2013-

2014). JOURNAL OF PUBLIC HEALTH-HEIDELBERG. 2020;28(2):163-171.

6844. Bauer Kohen, Bottini Cinzia, Frei Robert, Asael Dan, Planavsky Noah, Francois Roger, McKenzie N., Erba Elisabetta, Crowe Sean Pulsed volcanism and rapid oceanic deoxygenation during Oceanic Anoxic Event 1a. GEOLOGY. 2021;49(12):1452-1456.

6845. Pereira Emile, Silva Karine, Farias Costa Priscila, Silva Lais, Magalhaes Nepomuceno Carina, Silva Helena, Belfort Erika, Cunha Carla, Santana Monica Restrained eating behaviour, anorexia nervosa and food consumption between children and adolescents: a scoping review. BRITISH JOURNAL OF NUTRITION. 2022;128(8):1565-1586.

6846. Protogerou Cleo, McHugh R., Johnson Blair How best to reduce unhealthy risk-taking behaviours? A meta-review of evidence syntheses of interventions using self-regulation principles. HEALTH PSYCHOLOGY REVIEW. 2020;14(1, SI):86-115.

6847. Bever KA, Perry PJ Dexfenfluramine hydrochloride: An anorexigenic agent. AMERICAN JOURNAL OF HEALTH-SYSTEM PHARMACY. 1997;54(18):2059-2072.

6848. Bai Yang, Yang Yanli, Gao Yafen, Lin Duomao, Wang Zhaoqi, Ma Jun Melatonin postconditioning ameliorates anoxia/reoxygenation injury by regulating mitophagy and mitochondrial dynamics in a SIRT3-dependent manner. EUROPEAN JOURNAL OF PHARMACOLOGY. 2021;904():.

6849. CROMPTON M, ANDREEVA L ON THE INVOLVEMENT OF A MITOCHONDRIAL PORE IN REPERFUSION INJURY. BASIC RESEARCH IN CARDIOLOGY. 1993;88(5):513-523.

6850. Rastam M, Bjure J, Vestergren E, Uvebrant P, Gillberg IC, Wentz E, Gillberg C Regional cerebral blood flow in weight-restored anorexia nervosa: a preliminary study. DEVELOPMENTAL MEDICINE AND CHILD NEUROLOGY. 2001;43(4):239-242.

6851. Thibault Isabelle, Pauze Robert, Bravo Gina, Lavoie Eric, Pesant Caroline, Di Meglio Giuseppina, Frappier Jean-Yves, Meilleur Dominique, Nadeau Pierre-Olivier, Stheneur Chantal, Taddeo Danielle What Are the Individual, Family, and Social Characteristics Differentiating Prepubertal from Pubertal Anorexia Nervosa?. CANADIAN JOURNAL OF PSYCHIATRY-REVUE CANADIENNE DE PSYCHIATRIE. 2017;62(12):837-844.

6852. Kaye WH, Bailer UF, Frank GK, Wagner A, Henry SE Brain imaging of Serotonin after recovery from anorexia and bulimia nervosa. PHYSIOLOGY \& BEHAVIOR. 2005;86(1-2):15-17.

6853. Gadoth N, Kesler A, Vainstein G, Peled R, Lavie P Clinical and polysomnographic characteristics of 34 patients with Kleine-Levin syndrome. JOURNAL OF SLEEP RESEARCH. 2001;10(4):337-341.

6854. Luckhoff H., Plessis S., Scheffler F., Phahladira L., Kilian S., Buckle C., Smit R., Chiliza B., Asmal L., Emsley R. Fronto-limbic white matter fractional anisotropy and body mass index in first-episode schizophrenia spectrum disorder patients compared to healthy controls. PSYCHIATRY RESEARCH-NEUROIMAGING. 2020;305():.

6855. Blixen Carol, Levin Jennifer, Cassidy Kristin, Perzynski Adam, Sajatovic Martha Coping strategies used by poorly adherent patients for self-managing bipolar disorder. PATIENT PREFERENCE AND ADHERENCE. 2016;10():1327-1335.

6856. Menshanov Petr, Bannova Anita, Dygalo Nikolay The Early-Life ``Programming{''} of Anxiety-Driven Behaviours in Adulthood as a Product of Predator-Driven Evolution. EVOLUTIONARY BIOLOGY. 2022;49(3):303-313.

6857. Virolainen E, Blokhina O, Fagerstedt K Ca2+-induced high amplitude swelling and cytochrome c release from wheat (Triticum aestivum L.) mitochondria under anoxic stress. ANNALS OF BOTANY. 2002;90(4):509-516.

6858. Malighetti Clelia, Sansoni Maria, Gaudio Santino, Matamala-Gomez Marta, Di Lernia Daniele, Serino Silvia, Riva Giuseppe From Virtual Reality to Regenerative Virtual Therapy: Some Insights from a Systematic Review Exploring Inner Body Perception in Anorexia and Bulimia Nervosa. JOURNAL OF CLINICAL MEDICINE. 2022;11(23):.

6859. OConnor EA, Friel S, Kelleher CC Fashion consciousness as a social influence on lifestyle behaviour in young Irish adults. HEALTH PROMOTION INTERNATIONAL. 1997;12(2):135-139.

6860. Eskla Kattri-Liis, Vellama Hans, Tarve Liisi, Eichelmann Hillar, Jagomae Toomas, Porosk Rando, Oja Vello, Ramma Heikko, Peet Nadezda, Laisk Agu, Volke Vallo, Vasar Eero, Luuk Hendrik Hypothermia Alleviates Reductive Stress, a Root Cause of Ischemia

Reperfusion Injury. INTERNATIONAL JOURNAL OF MOLECULAR SCIENCES. 2022;23(17):.

6861. Mahneva Olena, Caplan Stacee, Ivko Polina, Dawson-Scully Ken, Milton Sarah NO/cGMP/PKG activation protects Drosophila cells subjected to hypoxic stress. COMPARATIVE BIOCHEMISTRY AND PHYSIOLOGY C-TOXICOLOGY \& PHARMACOLOGY. 2019;223():106-114.

6862. Verdoux H Does prenatal exposure to diethylstilbestrol (DES) have psychiatric consequences?. ANNALES MEDICO-PSYCHOLOGIQUES. 2000;158(2):105-117.

6863. Mohanty B, Ong BL Contrasting effects of submergence in light and dark on pyruvate decarboxylase activity in roots of rice lines differing in submergence tolerance. ANNALS OF BOTANY. 2003;91(2, SI):291-300.

6864. Tavolacci Marie-Pierre, Vasiliu Anca, Romo Lucia, Kotbagi Gayatri, Kern Laurence, Ladner Joel Patterns of electronic cigarette use in current and ever users among college students in France: a cross-sectional study. BMJ OPEN. 2016;6(5):.

6865. Frucht Steven Embouchure Dystonia-Portrait of a Task-Specific Cranial Dystonia. MOVEMENT DISORDERS. 2009;24(12):1752-1762.

6866. Giel Katrin, Leehr Elisabeth, Becker Sandra, Startup Helen, Zipfel Stephan, Schmidt Ulrike Relapse Prevention in Anorexia Nervosa. PSYCHOTHERAPIE PSYCHOSOMATIK MEDIZINISCHE PSYCHOLOGIE. 2013;63(7):290-295.

6867. Ranjan Sobhana, Nasser Jennifer Nutritional Status of Individuals with Autism Spectrum Disorders: Do We Know Enough?. ADVANCES IN NUTRITION. 2015;6(4):397-407.

6868. Shinkwin R, Standen PJ Trends in anorexia nervosa in Ireland: A register study. EUROPEAN EATING DISORDERS REVIEW. 2001;9(4):263-276.

6869. Logue Danielle, Madigan Sharon, Delahunt Eamonn, Heinen Mirjam, Mc Donnell Sarah-Jane, Corish Clare Low Energy Availability in Athletes: A Review of Prevalence, Dietary Patterns, Physiological Health, and Sports Performance. SPORTS MEDICINE. 2018;48(1):73-96.

6870. Baek Ji, Park Dong, Choi Jungmi, Kim Ji, Choi Ji, Ha Kyooseob, Kwon Jun, Lee Dongsoo, Hong Kyung Differences between bipolar I and bipolar II disorders in clinical features, comorbidity, and family history. JOURNAL OF AFFECTIVE DISORDERS. 2011;131(1-3):59-67.

6871. Rosen DS, Neumark-Sztainer D Review of options for primary prevention of eating disturbances among adolescents. JOURNAL OF ADOLESCENT HEALTH. 1998;23(6):354-363.

6872. Vermeulen Esther, Knuppel Anika, Shipley Martin, Brouwer Ingeborg, Visser Marjolein, Akbaraly Tasnime, Brunner Eric, Nicolaou Mary High-Sugar, High-Saturated-Fat Dietary Patterns Are Not Associated with Depressive Symptoms in Middle-Aged Adults in a Prospective Study. JOURNAL OF NUTRITION. 2018;148(10):1598-1604.

6873. Springall Gabriella, Cheung Michael, Sawyer Susan, Yeo Michele Impact of the coronavirus pandemic on anorexia nervosa and atypical anorexia nervosa presentations to an Australian tertiary paediatric hospital. JOURNAL OF PAEDIATRICS AND CHILD HEALTH. 2022;58(3):491-496.

6874. Sintra Diana, Agante Luisa Edutainment in childhood obesity prevention: a complex topic. YOUNG CONSUMERS. 2020;21(3):289-304.

6875. Mauri Mauro, Calderone Alba, Fagiolini Andrea, Santini Ferruccio, Borri Chiara, Oppo Annalisa, Romano Anna, Rinaldi Silvia, Polini Margherita, Lippi Chita, Pinchera Aldo, Cassano Giovanni Psychiatric disorders and lifetime mood spectrum symptoms in early and late onset obesity. OBESITY AND METABOLISM-MILAN. 2008;4(2):99-105.

6876. Mehnert Anja, Braehler Elmar, Faller Hermann, Haerter Martin, Keller Monika, Schulz Holger, Wegscheider Karl, Weis Joachim, Boehncke Anna, Hund Bianca, Reuter Katrin, Richard Matthias, Sehner Susanne, Sommerfeldt Sabine, Szalai Carina, Wittchen Hans-Ulrich, Koch Uwe Four-Week Prevalence of Mental Disorders in Patients With Cancer Across Major Tumor Entities. JOURNAL OF CLINICAL ONCOLOGY. 2014;32(31):3540+.

6877. Cooper Abigail, Loeb Katharine, McGlinchey Eleanor Sleep and eating disorders: current research and future directions. CURRENT OPINION IN PSYCHOLOGY. 2020;34():89-94.

6878. Zheng Jifeng, Mao Zhiyao, Zhang Jianqin, Jiang Liqing, Wang Ningfu Paeonol Pretreatment Attenuates Anoxia-Reoxygenation Induced Injury in Cardiac Myocytes via a BRCA1 Dependent Pathway. CHEMICAL \& PHARMACEUTICAL BULLETIN. 2020;68(12):1163-1169.

6879. Agras WS, Walsh BT, Fairburn CG, Wilson GT, Kraemer HC A multicenter comparison of cognitive-behavioral therapy and interpersonal psychotherapy for bulimia nervosa. ARCHIVES OF GENERAL PSYCHIATRY. 2000;57(5):459-466.

6880. Bilgin Ayten, Baumann Nicole, Jaekel Julia, Breeman Linda, Bartmann Peter, Baeuml Josef, Avram Mihai, Sorg Christian, Wolke Dieter Early Crying, Sleeping, and Feeding Problems and Trajectories of Attention Problems From Childhood to Adulthood. CHILD DEVELOPMENT. 2020;91(1):E77-E91.

6881. Champion Katrina, Parmenter Belinda, McGowan Cyanna, Spring Bonnie, Wafford Q., Gardner Lauren, Thornton Louise, McBride Nyanda, Barrett Emma, Teesson Maree, Newton Nicola, Team Hlth4Life Effectiveness of school-based eHealth interventions to prevent multiple lifestyle risk behaviours among adolescents: a systematic review and meta-analysis. LANCET DIGITAL HEALTH. 2019;1(5):E206-E221.

6882. BOISSETPIORO MH, ESDAILE JM, FITZCHARLES MA SEXUAL AND PHYSICAL ABUSE IN WOMEN WITH FIBROMYALGIA SYNDROME. ARTHRITIS AND RHEUMATISM. 1995;38(2):235-241.

6883. Keery H, Boutelle K, Berg P, Thompson JK The impact of appearance-related teasing by family members. JOURNAL OF ADOLESCENT HEALTH. 2005;37(2):120-127.

6884. Choquette Emily, Rancourt Diana, Thompson J. From fad to FAD: A theoretical formulation and proposed name change for ``drunkorexia{''} to food and alcohol disturbance (FAD). INTERNATIONAL JOURNAL OF EATING DISORDERS. 2018;51(8):831-834.

6885. Romer Adrienne, Kang Min, Nikolova Yuliya, Gearhardt Ashley, Hariri Ahmad Dopamine genetic risk is related to food addiction and body mass through reduced reward-related ventral striatum activity. APPETITE. 2019;133():24-31.

6886. Smith Kathryn, Mason Tyler, Crosby Ross, Cao Li, Leonard Rachel, Wetterneck Chad, Smith Brad, Farrell Nicholas, Riemann Bradley, Wonderlich Stephen, Moessner Markus A

comparative network analysis of eating disorder psychopathology and co-occurring depression and anxiety symptoms before and after treatment. PSYCHOLOGICAL MEDICINE. 2019;49(2):314-324.

6887. Noterdaeme M, Amorosa H Early diagnosis of infantile autism. MONATSSCHRIFT KINDERHEILKUNDE. 2002;150(2):149+.

6888. Perez C., Ball S., Wagner A., Clare I., Holland A., Redley M. The incidence of healthcare use, ill health and mortality in adults with intellectual disabilities and mealtime support needs. JOURNAL OF INTELLECTUAL DISABILITY RESEARCH. 2015;59(7):638-652.

6889. Han Eugene, Kim Gyuri, Hong Namki, Lee Yong-ho, Kim Dong, Shin Hyun, Lee Byung-Wan, Kang Eun, Lee In-Kyu, Cha Bong-Soo Association between dietary acid load and the risk of cardiovascular disease: nationwide surveys (KNHANES 2008-2011). CARDIOVASCULAR DIABETOLOGY. 2016;15():.

6890. Mangione Carol, Barry Michael, Nicholson Wanda, Cabana Michael, Coker Tumaini, Davidson Karina, Davis Esa, Donahue Katrina, Jaen Carlos, Kubik Martha, Li Li, Ogedegbe Gbenga, Pbert Lori, Ruiz John, Stevermer James, Wong John, Force US Behavioral Counseling Interventions to Promote a Healthy Diet and Physical Activity for Cardiovascular Disease Prevention in Adults Without Cardiovascular Disease Risk Factors US Preventive Services Task Force Recommendation Statement. JAMA-JOURNAL OF THE AMERICAN MEDICAL ASSOCIATION. 2022;328(4):367-374.

6891. Capewell Simon, Lloyd-Williams Ffion The role of the food industry in health: lessons from tobacco?. BRITISH MEDICAL BULLETIN. 2018;125(1):131-143.

6892. Lacerda Lydia, McCarthy Joy, Mungly Shazia, Lynn Edward, Sack Michael, Opie Lionel, Lecour Sandrine TNF alpha protects cardiac mitochondria independently of its cell surface receptors. BASIC RESEARCH IN CARDIOLOGY. 2010;105(6):751-762.

6893. Gittelsohn J, Harris SB, Burris KL, Kakegamic L, Landman LT, Sharma A, Wolever TMS, Logan A, Barnie A, Zinman B Use of ethnographic methods for applied research on diabetes among the Ojibway-Cree in northern Ontario. HEALTH EDUCATION QUARTERLY. 1996;23(3):365-382.

6894. Plas A., Monteiro P., Pascall A. Cross-shelf biogeochemical characteristics of sediments in the central Benguela and their relationship to overlying water column hypoxia. AFRICAN

JOURNAL OF MARINE SCIENCE. 2007;29(1):37-47.

6895. Zonnevylle-Bender MJS, Goozen SHM, Cohen-Kettenis PT, Elburg A, Wildt M, Stevelmans E, Engeland H Emotional functioning in anorexia nervosa patients: Adolescents compared to adults. DEPRESSION AND ANXIETY. 2004;19(1):35-42.

6896. Gorbena Susana, Govillard Leila, Iraurgi Ioseba A taxonomy of groups at risk based on reported and desired body mass index and its relationships with health. PSYCHOLOGY HEALTH \& MEDICINE. 2021;26(1, SI):49-61.

6897. Seymons K, De Moor A, De Raeve H, Lambert J Dermatologic signs of biotin deficiency leading to the diagnosis of multiple carboxylase deficiency. PEDIATRIC DERMATOLOGY. 2004;21(3):231-235.

6898. Emley Elizabeth, Musher-Eizenman Dara Social movement involvement and healthy diet and activity behaviors among US adults. HEALTH PROMOTION INTERNATIONAL. 2019;34(3):490-500.

6899. Hage Trine, Nilsen Jan-Vegard, Karlsen Katrine, Lyslid Martine, Wennersberg Anne, Wisting Line ``I am not alone{''}. A qualitative feasibility study of eating disorders prevention groups for young females with type 1 diabetes. JOURNAL OF EATING DISORDERS. 2023;11(1):.

6900. Johnson Patricia, Webber Troy, Wu Monica, Lewin Adam, Murphy Tanya, Storch Eric When selective audiovisual stimuli become unbearable: a case series on pediatric misophonia. NEUROPSYCHIATRY. 2013;3(6):569-575.

6901. Hoch Anne, Stavrakos John, Schimke Jane Prevalence of female athlete triad characteristics in a club triathlon team. ARCHIVES OF PHYSICAL MEDICINE AND REHABILITATION. 2007;88(5):681-682.

6902. Chao Dongman, Donnelly David, Feng Yin, Bazzy-Asaad Alia, Xia Ying Cortical delta-opioid receptors potentiate K+ homeostasis during anoxia and oxygen-glucose deprivation. JOURNAL OF CEREBRAL BLOOD FLOW AND METABOLISM. 2007;27(2):356-368.

6903. Hower Heather, Reilly Erin, Wierenga Christina, Kaye Walter Last word: a call to view temperamental traits as dual vulnerabilities and strengths in anorexia nervosa. EATING

DISORDERS. 2021;29(2):151-160.

6904. Saccomani L, Savoini M, Cirrincione M, Vercellino F, Ravera G Long-term outcome of children and adolescents with anorexia nervosa: Study of comorbidity. JOURNAL OF PSYCHOSOMATIC RESEARCH. 1998;44(5):565-571.

6905. Jang Hwan-Hee, Noh Hwayoung, Kim Gichang, Cho Su-Yeon, Kim Hyeon-Jeong, Choe Jeong-Sook, Kim Jeongseon, Scalbert Augustin, Gunter Marc, Kwon Oran, Kim Hyesook Differences in dietary patterns related to metabolic health by gut microbial enterotypes of Korean adults. FRONTIERS IN NUTRITION. 2023;9():.

6906. Kang Dingding, Su Meng, Duan Yanwen, Huang Yong Eurotium cristatum, a potential probiotic fungus from Fuzhuan brick tea, alleviated obesity in mice by modulating gut microbiota. FOOD \& FUNCTION. 2019;10(8):5032-5045.

6907. Steca Patrizia, Monzani Dario, Greco Andrea, Franzelli Cristina, Magrin Maria, Miglioretti Massimo, Sarini Marcello, Scrignaro Marta, Vecchio Luca, Fattirolli Francesco, D'Addario Marco Stability and change of lifestyle profiles in cardiovascular patients after their first acute coronary event. PLOS ONE. 2017;12(8):.

6908. Range Helene, Colon Pierre, Godart Nathalie, Kapila Yvonne, Bouchard Philippe Eating disorders through the periodontal lens. PERIODONTOLOGY 2000. 2021;87(1):17-31.

6909. Young-Hyman Deborah Introduction to Special Issue: Self-Regulation of Appetite-It's Complicated. OBESITY. 2017;25(1, SI):S5-S7.

6910. Calugi S., Dalle Grave R., Compare A., Dall'Aglio E., Petroni M., Marchesini G., Grp QUOVADIS Weight loss and clinical characteristics of young adults patients seeking treatment at medical centers: Data from the QUOVADIS Study. EATING AND WEIGHT DISORDERS-STUDIES ON ANOREXIA BULIMIA AND OBESITY. 2012;17(4):E314-E319.

6911. Moyad Mark Preventing Lethal Prostate Cancer with Diet, Supplements, and Rx: Heart Healthy Continues to Be Prostate Healthy and ``First Do No Harm{''} Part II. CURRENT UROLOGY REPORTS. 2020;21(3):.

6912. Gmeiner Michaela, Warschburger Petra Simply too much: the extent to which weight bias internalization results in a higher risk of eating disorders and psychosocial problems.

EATING AND WEIGHT DISORDERS-STUDIES ON ANOREXIA BULIMIA AND OBESITY. 2022;27(1):317-324.

6913. Forbush Kelsie, Bohrer Brittany, Hagan Kelsey, Chapa Danielle, Perko Victoria, Richson Brianne, Christian Kylie, Christensen Kara, Wildes Jennifer Development and Initial Validation of the Eating Pathology Symptoms Inventory-Clinician-Rated Version (EPSI-CRV). PSYCHOLOGICAL ASSESSMENT. 2020;32(10):943-955.

6914. Stigger Felipe, Lovatel Gisele, Marques Marilia, Bertoldi Karine, Moyses Felipe, Elsner Viviane, Siqueira Ionara, Achaval Matilde, Marcuzzo Simone Inflammatory response and oxidative stress in developing rat brain and its consequences on motor behavior following maternal administration of LPS and perinatal anoxia. INTERNATIONAL JOURNAL OF DEVELOPMENTAL NEUROSCIENCE. 2013;31(8):820-827.

6915. Colic Natalie, Platt Jonathan, Keyes Katherine, Sumner Jennifer, Allen Nicholas, McLaughlin Katie Earlier age at menarche as a transdiagnostic mechanism linking childhood trauma with multiple forms of psychopathology in adolescent girls. PSYCHOLOGICAL MEDICINE. 2020;50(7):1090-1098.

6916. Palermi Stefano, Vecchiato Marco, Pennella Sonia, Marasca Anna, Spinelli Alessandro, De Luca Mariarosaria, De Martino Lorena, Fernando Fredrick, Sirico Felice, Biffi Alessandro The Impact of the COVID-19 Pandemic on Childhood Obesity and Lifestyle-A Report from Italy. PEDIATRIC REPORTS. 2022;14(4):410-418.

6917. Schramm Adrien, Carton-Leclercq Antoine, Diallo Shana, Navarro Vincent, Chavez Mario, Mahon Severine, Charpier Stephane Identifying neuronal correlates of dying and resuscitation in a model of reversible brain anoxia. PROGRESS IN NEUROBIOLOGY. 2020;185():.

6918. Golbidi Saeid, Daiber Andreas, Korac Bato, Li Huige, Essop M., Laher Ismail Health Benefits of Fasting and Caloric Restriction. CURRENT DIABETES REPORTS. 2017;17(12):.

6919. Panagiotakos Demosthenes, Notara Venetia, Kouvari Matina, Pitsavos Christos The Mediterranean and other Dietary Patterns in Secondary Cardiovascular Disease Prevention: A Review. CURRENT VASCULAR PHARMACOLOGY. 2016;14(5):442-451.

6920. Okely Anthony, Collins Clare, Morgan Philip, Jones Rachel, Warren Janet, Cliff Dylan, Burrows Tracy, Colyvas Kim, Steele Julie, Baur Louise Multi-Site Randomized Controlled

Trial of a Child-Centered Physical Activity Program, a Parent-Centered Dietary-Modification Program, or Both in Overweight Children: The HIKCUPS Study. JOURNAL OF PEDIATRICS. 2010;157(3):388-394.

6921. Head Maggie, Bruchmann Kathryn The effects of health frame and target relevance in appearance social comparisons. JOURNAL OF APPLIED SOCIAL PSYCHOLOGY. 2019;49(1):27-35.

6922. Baranauskas Marius, Kupciunaite Ingrida, Stukas Rimantas Potential Triggers for Risking the Development of Eating Disorders in Non-Clinical Higher-Education Students in Emerging Adulthood. NUTRIENTS. 2022;14(11):.

6923. Schwiebbe L., Rest J., Verhagen E., Visser R., Kist-van Holthe J., Hirasing R. Childhood Obesity in the Caribbean. WEST INDIAN MEDICAL JOURNAL. 2011;60(4):442-445.

6924. SUBBAIAH CC, ZHANG JK, SACHS MM INVOLVEMENT OF INTRACELLULAR CALCIUM IN ANAEROBIC GENE-EXPRESSION AND SURVIVAL OF MAIZE SEEDLINGS. PLANT PHYSIOLOGY. 1994;105(1):369-376.

6925. Calella Patrizia, Galle Francesca, Di Onofrio Valeria, Buono Pasqualina, Liguori Giorgio, Valerio Giuliana Gym Members Show Lower Nutrition Knowledge than Youth Engaged in Competitive Sports. JOURNAL OF THE AMERICAN COLLEGE OF NUTRITION. 2021;40(5):465-471.

6926. Trubswasser Ursula, Candel Jeroen, Genye Tirsit, Bossuyt Anne, Holdsworth Michelle, Baye Kaleab, Talsma Elise Benchmarking policy goals and actions for healthy food environments in Ethiopia to prevent malnutrition in all its forms using document analysis. BMJ OPEN. 2022;12(8):.

6927. Canellas-Dols Francesca, Delgado Carlos, Arango-Lopez Celso, Peraita-Adrados Rosa Narcolepsy-cataplexy and psychosis: a case study. REVISTA DE NEUROLOGIA. 2017;65(2):70-74.

6928. Rodriguez-Concepcion Manuel, Daros Jose-Antonio Transient expression systems to rewire plant carotenoid metabolism. CURRENT OPINION IN PLANT BIOLOGY. 2022;66():.

6929. Fuerstenwerth Hauke Ouabain-The Key to Cardioprotection?. AMERICAN JOURNAL OF THERAPEUTICS. 2014;21(5):395-402.

6930. Johnson Catherine, Wade Tracey Acceptability and Effectiveness of an 8-week Mindfulness Program in Early- and Mid-adolescent School Students: a Randomised Controlled Trial. MINDFULNESS. 2021;12(10):2473-2486.

6931. MATTES RD, COWART BJ DIETARY ASSESSMENT OF PATIENTS WITH CHEMOSENSORY DISORDERS. JOURNAL OF THE AMERICAN DIETETIC ASSOCIATION. 1994;94(1):50-56.

6932. Wu Batubayan, Zhou Ruo-Lin, Ou Qing-Jian, Chen Yu-Ming, Fang Yu-Jing, Zhang Cai-Xia Association of plant-based dietary patterns with the risk of colorectal cancer: a large-scale case-control study. FOOD \& FUNCTION. 2022;13(20):10790-10801.

6933. Wang Tiange, Zhao Zhiyun, Wang Guixia, Li Qiang, Xu Yu, Li Mian, Hu Ruying, Chen Gang, Su Qing, Mu Yiming, Tang Xulei, Yan Li, Qin Guijun, Wan Qin, Gao Zhengnan, Yu Xuefeng, Shen Feixia, Luo Zuojie, Qin Yingfen, Chen Li, Huo Yanan, Zeng Tianshu, Chen Lulu, Ye Zhen, Zhang Yinfei, Liu Chao, Wang Youmin, Wu Shengli, Yang Tao, Deng Huacong, Zhao Jiajun, Shi Lixin, Xu Yiping, Xu Min, Chen Yuhong, Wang Shuangyuan, Lu Jieli, Bi Yufang, Ning Guang, Wang Weiqing Age-related disparities in diabetes risk attributable to modifiable risk factor profiles in Chinese adults: a nationwide, population-based, cohort study. LANCET HEALTHY LONGEVITY. 2021;2(10):E618-E628.

6934. Morris Heather, Edwards Susan, Cutter-Mackenzie Amy, Rutherford Leonie, Williams-Smith Janet, Skouteris Helen Evaluating the impact of teacher-designed, wellbeing and sustainability play-based learning experiences on young children's knowledge connections: A randomised trial. AUSTRALASIAN JOURNAL OF EARLY CHILDHOOD. 2018;43(4):33-42.

6935. Quinones Isabel, Herbozo Sylvia, Haedt-Matt Alissa Body dissatisfaction among ethnic subgroups of Latin women: An examination of acculturative stress and ethnic identity. BODY IMAGE. 2022;41():272-283.

6936. Mitchell JE, Maki DD, Adson DE, Ruskin BS, Crow S The selectivity of inclusion and exclusion criteria in bulimia nervosa treatment studies. INTERNATIONAL JOURNAL OF EATING DISORDERS. 1997;22(3):243-252.

6937. Vartanian Lenny, Kernan Kristin, Wansink Brian Clutter, Chaos, and Overconsumption: The Role of Mind-Set in Stressful and Chaotic Food Environments. ENVIRONMENT AND BEHAVIOR. 2017;49(2):215-223.

6938. Falsin Giglio Larriany, Silva Magalhaes Pedro, Andreazza Ana, Walz Julio, Jakobson Lourenco, Rucci Paola, Rosa Adriane, Hidalgo Maria, Vieta Eduard, Kapczinski Flavio Development and use of a biological rhythm interview. JOURNAL OF AFFECTIVE DISORDERS. 2009;118(1-3):161-165.

6939. Lin Hsin-Ti, Enchautegui-Colon Yazmin, Huang Yu-Ren, Zimmerman Chelsea, DeMarzo Danielle, Tsai Anne Novel compound heterozygote variants: c.4193_4206delinsG (p. Leu1398Argfs{*}25), c.793C > A (p.Pro265Thr), in the CPS1 gene (NM_001875.4) causing late onset carbamoyl phosphate synthetase 1 deficiency-Lessons learned. MOLECULAR GENETICS AND METABOLISM REPORTS. 2022;33():.

6940. Bird Emma, Halliwell Emma, Diedrichs Phillippa, Harcourt Diana Happy Being Me in the UK: A controlled evaluation of a school-based body image intervention with pre-adolescent children. BODY IMAGE. 2013;10(3):326-334.

6941. Sotos-Prieto Mercedes, Baylin Ana, Campos Hannia, Qi Lu, Mattei Josiemer Lifestyle Cardiovascular Risk Score, Genetic Risk Score, and Myocardial Infarction in Hispanic/Latino Adults Living in Costa Rica. JOURNAL OF THE AMERICAN HEART ASSOCIATION. 2016;5(12):.

6942. Jahanfar S, Maleki H, Mosavi AR, Jahanfar M Leptin and its association with polycystic ovary syndrome: a twin study. GYNECOLOGICAL ENDOCRINOLOGY. 2004;18(6):327-334.

6943. Landini Linda, Dadson Prince, Gallo Fabrizio, Honka Miikka-Juhani, Cena Hellas Microbiota in anorexia nervosa: potential for treatment. NUTRITION RESEARCH REVIEWS. 2022;():.

6944. Garcia-Blanco A., Ramirez-Lopez A., Navarrete F., Garcia-Gutierrez M., Manzanares J., Martin-Garcia E., Maldonado R. Role of CB2 cannabinoid receptor in the development of food addiction in male mice. NEUROBIOLOGY OF DISEASE. 2023;179():.

6945. Kuek Angeline, Utpala Ranjani, Lee Huei The clinical profile of patients with anorexia nervosa in Singapore: a follow-up descriptive study. SINGAPORE MEDICAL JOURNAL.

2015;56(6):324-328.

6946. Lindvall Kristina, Jenkins Paul, Scribani Melissa, Emmelin Maria, Larsson Christel, Norberg Margareta, Weinehall Lars Comparisons of weight change, eating habits and physical activity between women in Northern Sweden and Rural New York State-results from a longitudinal study. NUTRITION JOURNAL. 2015;14():.

6947. Ruggiero Cara, Hohman Emily, Birch Leann, Paul Ian, Savage Jennifer INSIGHT responsive parenting intervention effects on child appetite and maternal feeding practices through age 3 years. APPETITE. 2021;159():.

6948. Li SX, Jiang QB, Stys PK Important role of reverse Na+-Ca2+ exchange in spinal cord white matter injury at physiological temperature. JOURNAL OF NEUROPHYSIOLOGY. 2000;84(2):1116-1119.

6949. WATSON GB, LOPEZ OT, CHARLES VD, LANTHORN TH ASSESSMENT OF LONG-TERM EFFECTS OF TRANSIENT ANOXIA ON METABOLIC-ACTIVITY OF RAT HIPPOCAMPAL SLICES USING TRIPHENYLTETRAZOLIUM CHLORIDE. JOURNAL OF NEUROSCIENCE METHODS. 1994;53(2):203-208.

6950. Shachar-Lavie Iris, Segal Hila, Oryan Zohar, Halifa-Kurtzman Irit, Bar-Eyal Adi, Hadas Arik, Tamar Tahar, Benaroya-Milshtein Noa, Fennig Silvana Atypical anorexia nervosa: Rethinking the association between target weight and rehospitalization. EATING BEHAVIORS. 2022;46():.

6951. O'Leary Daniel, Suri Gaurav, Gross James Reducing behavioural risk factors for cancer: An affect regulation perspective. PSYCHOLOGY \& HEALTH. 2018;33(1, SI):17-39.

6952. Silva-Sanigorski A., Elea D., Bell C., Kremer P., Carpenter L., Nichols M., Smith M., Sharp S., Boak R., Swinburn B. Obesity prevention in the family day care setting: impact of the Romp \& Chomp intervention on opportunities for children's physical activity and healthy eating. CHILD CARE HEALTH AND DEVELOPMENT. 2011;37(3):385-393.

6953. Wu Jiayuan, Lin Zhixiong, Liu Zhou, He Hairong, Bai Ling, Lyu Jun Secular trends in the incidence of eating disorders in China from 1990 to 2017: a joinpoint and age-period-cohort analysis. PSYCHOLOGICAL MEDICINE. 2022;52(5):946-956.

6954. Constantin Andres, Cabrera Oscar, Rios Belen, Barbosa Isabel, Ramirez Ariadna, Cina Margherita, Guzman Silvia A human rights-based approach to non-communicable diseases: mandating front-of-package warning labels. GLOBALIZATION AND HEALTH. 2021;17(1):.

6955. Htun Htet, Teshale Achamyeleh, Cumpston Miranda, Demos Lisa, Ryan Joanne, Owen Alice, Freak-Poli Rosanne Effectiveness of social prescribing for chronic disease prevention in adults: a systematic review and meta-analysis of randomised controlled trials. JOURNAL OF EPIDEMIOLOGY AND COMMUNITY HEALTH. 2023;77(4):265-276.

6956. Taksande Brijesh, Chopde Chandrabhan, Umekar Milind, Kotagale Nandkishor Agmatine attenuates hyperactivity and weight loss associated with activity-based anorexia in female rats. PHARMACOLOGY BIOCHEMISTRY AND BEHAVIOR. 2015;132():136-141.

6957. Casati Martina, Ferri Evelyn, Azzolino Domenico, Cesari Matteo, Arosio Beatrice Gut microbiota and physical frailty through the mediation of sarcopenia. EXPERIMENTAL GERONTOLOGY. 2019;124():.

6958. Taylor Craig, Graham Andrea, Fitzsimmons-Craft Ellen, Sadeh-Sharvit Shiri, Balantekin Katherine, Flatt Rachael, Goel Neha, Monterubio Grace, Topooco Naira, Karam Anna, Firebaugh Marie-Laure, Ruzek Josef, Funk Burkhardt, Oldenburg Brian, Wilfley Denise, Jacobi Corinna Optimizing eating disorder treatment outcomes for individuals identified via screening: An idea worth researching. INTERNATIONAL JOURNAL OF EATING DISORDERS. 2019;52(11):1224-1228.

6959. Latawitz Toni, Spielau Ulrike, Lipek Tobias, Gausche Ruth, Lueck Martina, Kiess Wieland, Grande Gesine, Igel Ulrike Neighbourhood deprivation and community nutrition environment: associations between the social and built environment and food availability. GESUNDHEITSWESEN. 2019;81(5):405-412.

6960. Moradi Sajjad, Khorrami-nezhad Leila, Ali-akbar Sima, Zare Fatemeh, Alipour Tanaz, Bozorg Azadeh, Yekaninejad Mir, Maghbooli Zhila, Mirzaei Khadijeh The associations between dietary patterns and bone health, according to the TGF-beta 1 T869 -> C polymorphism, in postmenopausal Iranian women. AGING CLINICAL AND EXPERIMENTAL RESEARCH. 2018;30(6):563-571.

6961. Molot Lewis, Schiff Sherry, Venkiteswaran Jason, Baulch Helen, Higgins Scott, Zastepa Arthur, Verschoor Mark, Walters Daniel Low sediment redox promotes cyanobacteria blooms across a trophic range: implications for management. LAKE AND RESERVOIR

MANAGEMENT. 2021;37(2):120-142.

6962. Singhal A., Sheng X., Drakos S., Stehlik J. Impact of Donor Cause of Death on Transplant Outcomes: UNOS Registry Analysis. TRANSPLANTATION PROCEEDINGS. 2009;41(9):3539-3544.

6963. Levine Michael Prevention of eating disorders: 2018 in review. EATING DISORDERS. 2019;27(1):18-33.

6964. Maske Calyn, Coiduras Isabel, Ondriezek Zeleen, Terrill Sarah, Williams Diana Intermittent High-Fat Diet Intake Reduces Sensitivity to Intragastric Nutrient Infusion and Exogenous Amylin in Female Rats. OBESITY. 2020;28(5):942-952.

6965. Gonzalez-Dominguez Raul, Castellano-Escuder Pol, Carmona Francisco, Lefevre-Arbogast Sophie, Low Dorrain, Du Preez Andrea, Ruigrok Silvie, Manach Claudine, Urpi-Sarda Mireia, Korosi Aniko, Lucassen Paul, Aigner Ludwig, Pallas Merce, Thuret Sandrine, Samieri Cecilia, Sanchez-Pla Alex, Andres-Lacueva Cristina Food and Microbiota Metabolites Associate with Cognitive Decline in Older Subjects: A 12-Year Prospective Study. MOLECULAR NUTRITION \& FOOD RESEARCH. 2021;65(23):.

6966. Tulloch Alastair, Murray Susan, Vaicekonyte Regina, Avena Nicole Neural Responses to Macronutrients: Hedonic and Homeostatic Mechanisms. GASTROENTEROLOGY. 2015;148(6):1205-1218.

6967. Dakanalis Antonios, Clerici Massimo, Stice Eric Prevention of eating disorders: current evidence-base for dissonance-based programmes and future directions. EATING AND WEIGHT DISORDERS-STUDIES ON ANOREXIA BULIMIA AND OBESITY. 2019;24(4):597-603.

6968. Nunes Sara, Alves Andre, Preguica Ines, Barbosa Adelaide, Vieira Pedro, Mendes Fernando, Martins Diana, Viana Sofia, Reis Flavio Crescent-Like Lesions as an Early Signature of Nephropathy in a Rat Model of Prediabetes Induced by a Hypercaloric Diet. NUTRIENTS. 2020;12(4):.

6969. Doorn Diana, Richardson Noel, Storey Aubrey, Osborne Aoife, Cunningham Caitriona, Blake Catherine, McNamara John Investigating the Dietary Habits of Male Irish Farmers to Prevent Mortality and Morbidity. SAFETY. 2021;7(3):.

6970. Byers JE Differential susceptibility to hypoxia aids estuarine invasion. MARINE ECOLOGY PROGRESS SERIES. 2000;203():123-132.

6971. Navarro Valeria, Vio Fernando Systematic review with analysis of bias and quality of interventions in the food environment of the workplace and their impact on the nutritional status of workers. NUTRICION HOSPITALARIA. 2022;39(5):1153-1165.

6972. Yamada Masako, Hapsari Elsi, Matsuo Hiroya Behaviors toward Noncommunicable Diseases Prevention and Their Relationship with Physical Health Status among Community-dwelling, Middle-aged and Older Women in Indonesia. INTERNATIONAL JOURNAL OF ENVIRONMENTAL RESEARCH AND PUBLIC HEALTH. 2020;17(7):.

6973. Lenthe Frank, Bourdeaudhuij Ilse, Klepp Knut-Inge, Lien Nanna, Moore Laurence, Faggiano Fabrizio, Kunst Anton, Mackenbach Johan Preventing socioeconomic inequalities in health behaviour in adolescents in Europe: Background, design and methods of project TEENAGE. BMC PUBLIC HEALTH. 2009;9():.

6974. Hassan MK, Joshi AV, Madhavan SS, Amonkar MM Obesity and health-related quality of life: a cross-sectional analysis of the US population. INTERNATIONAL JOURNAL OF OBESITY. 2003;27(10):1227-1232.

6975. Wade TD, Davidson S, O'Dea JA A preliminary controlled evaluation of a school-based media literacy program and self-esteem program for reducing eating disorder risk factors. INTERNATIONAL JOURNAL OF EATING DISORDERS. 2003;33(4):371-383.

6976. Pamenter Matthew, Hogg David, Buck Leslie Endogenous reductions in N-methyl-D-aspartate receptor activity inhibit nitric oxide production in the anoxic freshwater turtle cortex. FEBS LETTERS. 2008;582(12):1738-1742.

6977. Kurz T, Richardt G, Seyfarth M, Schomig A Nonexocytotic noradrenaline release induced by pharmacological agents or anoxia in human cardiac tissue. NAUNYN-SCHMIEDEBERGS ARCHIVES OF PHARMACOLOGY. 1996;354(1):7-16.

6978. Alshehri Khalid, Altuwaylie Talal, Alqhtani Ali, Albawab Albaraa, Almalki Abdulrahman Type 2 Diabetic Patients Adherence Towards Their Medications. CUREUS. 2020;12(2):.

6979. Wisting Line, Haugvik Severina, Wennersberg Anne, Hage Trine, Stice Eric, Olmsted Marion, Ghaderi Ata, Brunborg Cathrine, Skrivarhaug Torild, Dahl-Jorgensen Knut, Ro Oyvind Feasibility of a virtually delivered eating disorder prevention program for young females with type 1 diabetes. INTERNATIONAL JOURNAL OF EATING DISORDERS. 2021;54(9):1696-1706.

6980. Jones Megan, Voelker Ulrike, Lock James, Taylor C., Jacobi Corinna Family-based Early Intervention for Anorexia Nervosa. EUROPEAN EATING DISORDERS REVIEW. 2012;20(3):e137-e143.

6981. McAlpine Donald, Frisch Maria, Rome Ellen, Clark Matthew, Signore Carol, Lindroos Anna, Allison Kelly Bariatric Surgery: A Primer for Eating Disorder Professionals. EUROPEAN EATING DISORDERS REVIEW. 2010;18(4):304-317.

6982. Dorard G., Bungener C., Phan O., Edel Y., Corcos M., Berthoz S. Which psychiatric comorbidities in cannabis dependence during adolescence? Comparison of outpatients and controls. ENCEPHALE-REVUE DE PSYCHIATRIE CLINIQUE BIOLOGIQUE ET THERAPEUTIQUE. 2018;44(1):2-8.

6983. Moca Abel, Vaida Luminita, Negrutiu Bianca, Moca Rahela, Todor Bianca The Influence of Age on the Development of Dental Caries in Children. A Radiographic Study. JOURNAL OF CLINICAL MEDICINE. 2021;10(8):.

6984. Gordon Chloe, Rodgers Rachel, Slater Amy, McLean Sian, Jarman Hannah, Paxton Susan A cluster randomized controlled trial of the SoMe social media literacy body image and wellbeing program for adolescent boys and girls: Study protocol. BODY IMAGE. 2020;33():27-37.

6985. Moyad Mark Preventing Lethal Prostate Cancer with Diet, Supplements, and Rx: Heart Healthy Continues to Be Prostate Healthy and ``First Do No Harm{''} Part III. CURRENT UROLOGY REPORTS. 2020;21(5):.

6986. Shi Chunmei, Li Nan, Dong Jing, Wang Li, Li Xiling, Ji Chenbo, Wang Xingyun, Chi Xia, Guo Xirong, Tong Meiling, Zhang Min Association between maternal nonresponsive feeding practice and child's eating behavior and weight status: children aged 1 to 6 years. EUROPEAN JOURNAL OF PEDIATRICS. 2017;176(12):1603-1612.

6987. ZWEIER JL, BRODERICK R, KUPPUSAMY P, THOMPSONGORMAN S, LUTTY GA DETERMINATION OF THE MECHANISM OF FREE-RADICAL GENERATION IN HUMAN AORTIC ENDOTHELIAL-CELLS EXPOSED TO ANOXIA AND REOXYGENATION. JOURNAL OF BIOLOGICAL CHEMISTRY. 1994;269(39):24156-24162.

6988. Musaiger Abdulrahman, Al-Khalifa Fatima, Al-Mannai Mariam Obesity, unhealthy dietary habits and sedentary behaviors among university students in Sudan: growing risks for chronic diseases in a poor country. ENVIRONMENTAL HEALTH AND PREVENTIVE MEDICINE. 2016;21(4):224-230.

6989. Reis J., Roman G., Giroud M., Palmer V., Spencer P. Medical management, prevention and mitigation of environmental risks factors in Neurology. REVUE NEUROLOGIQUE. 2019;175(10):698-704.

6990. Eilat-Adar S., Mete M., Fretts A., Fabsitz R., Handeland V., Lee E., Loria C., Xu J., Yeh J., Howard B. Dietary patterns and their association with cardiovascular risk factors in a population undergoing lifestyle changes: The Strong Heart Study. NUTRITION METABOLISM AND CARDIOVASCULAR DISEASES. 2013;23(6):528-535.

6991. Beros Katarina, Brajkovic Lovorka, Kopilas Vanja Psychological resilience and depression in women with anorexia nervosa. MEDITERRANEAN JOURNAL OF CLINICAL PSYCHOLOGY. 2021;9(1):.

6992. Espejo Maria, Magabo Shirley, Rivera-Castro Angel, Faiz Mohammed, Ramirez Leandro, Robles Cristabel, Shabarek Tarek, Shariff Masood, Kanna Balavenkatesh Qualitative Study of Knowledge, Perception, and Behavior Related to Hypertension and Cardiovascular Disease Risk Reduction Among Hypertensive African-Americans in Urban Inner City of South Bronx, New York. JOURNAL OF RACIAL AND ETHNIC HEALTH DISPARITIES. 2019;6(1):197-206.

6993. Johnson Catherine, Burke Christine, Brinkman Sally, Wade Tracey Effectiveness of a school-based mindfulness program for transdiagnostic prevention in young adolescents. BEHAVIOUR RESEARCH AND THERAPY. 2016;81():1-11.

6994. Ferenczi Emily, Asaria Perviz, Hughes Alun, Chaturvedi Nishi, Francis Darrel Can a Statin Neutralize the Cardiovascular Risk of Unhealthy Dietary Choices?. AMERICAN JOURNAL OF CARDIOLOGY. 2010;106(4):587-592.

6995. Xue Lei, Pan Tiewen, Xu Zhifei, Zhao Xuewei, Zhong Lei, Wu Lihui, Wu Bin, Qin Xiong Multi-Factor Investigation of Early Postoperative Cardiac Arrhythmia for Elderly Patients with Esophageal or Cardiac Carcinoma. WORLD JOURNAL OF SURGERY. 2009;33(12):2615-2619.

6996. Weihrauch-Blueher Susann, Wiegand Susanna Risk Factors and Implications of Childhood Obesity. CURRENT OBESITY REPORTS. 2018;7(4):254-259.

6997. Mascherini Gabriele, Petri Cristian, Cala Piergiuseppe, Bini Vittorio, Galanti Giorgio Lifestyle and resulting body composition in young athletes. MINERVA PEDIATRICS. 2021;73(5):391-397.

6998. Wilksch Simon, Wade Tracey Depression as a moderator of benefit from Media Smart: A school-based eating disorder prevention program. BEHAVIOUR RESEARCH AND THERAPY. 2014;52():64-71.

6999. Damiano Stephanie, McLean Sian, Nguyen Lilly, Yager Zali, Paxton Susan Do we cause harm? Understanding the impact of research with young children about their body image. BODY IMAGE. 2020;34():59-66.

7000. Romero-Martinez Angel, Ruiz-Robledillo Nicolas, Moya-Albiol Luis Depressive Mood and Testosterone Related to Declarative Verbal Memory Decline in Middle-Aged Caregivers of Children with Eating Disorders. INTERNATIONAL JOURNAL OF ENVIRONMENTAL RESEARCH AND PUBLIC HEALTH. 2016;13(3):.

7001. Chaves Graciela, Britez Nidia, Maciel Victor, Klinkhof Andreas, Mereles Derliz Prevalence of cardiovascular risk factors in an urban ambulatory adult population: AsuRiesgo study, Paraguay. REVISTA PANAMERICANA DE SALUD PUBLICA-PAN AMERICAN JOURNAL OF PUBLIC HEALTH. 2015;38(2):136-143.

7002. Bindoff Ivan, Salas Kristy, Peterson Gregory, Ling Tristan, Lewis Ian, Wells Lindsay, Gee Peter, Ferguson Stuart Quittr: The Design of a Video Game to Support Smoking Cessation. JMIR SERIOUS GAMES. 2016;4(2):.

7003. Goffe Louis, Penn Linda, Adams Jean, Araujo-Soares Vera, Summerbell Carolyn, Abraham Charles, White Martin, Adamson Ashley, Lake Amelia The challenges of interventions to promote healthier food in independent takeaways in England: qualitative

study of intervention deliverers' views. BMC PUBLIC HEALTH. 2018;18():.

7004. Depner Christopher, Stothard Ellen, Wright Kenneth Metabolic Consequences of Sleep and Circadian Disorders. CURRENT DIABETES REPORTS. 2014;14(7):.

7005. Marzola Enrica, Panepinto Corine, Delsedime Nadia, Amianto Federico, Fassino Secondo, Abbate-Daga Giovanni A factor analysis of the meanings of anorexia nervosa: intrapsychic, relational, and avoidant dimensions and their clinical correlates. BMC PSYCHIATRY. 2016;16():.

7006. Weissman Ruth, Frank Guido, Klump Kelly, Thomas Jennifer, Wade Tracey, Waller Glenn The current status of cognitive behavioral therapy for eating disorders: Marking the 51st Annual Convention of the Association of Behavioral and Cognitive Therapies. INTERNATIONAL JOURNAL OF EATING DISORDERS. 2017;50(12):1444-1446.

7007. MAQUEDA IG ADRENOCEPTORS, ENDOTHELIAL FUNCTION, AND LIPID PROFILE - EFFECTS OF ATENOLOL, DOXAZOSIN, AND CARVEDILOL. CORONARY ARTERY DISEASE. 1994;5(11):909-918.

7008. Chaves Tharcila, Sanchez Zila, Ribeiro Luciana, Nappo Solange Crack cocaine craving: behaviors and coping strategies among current and former users. REVISTA DE SAUDE PUBLICA. 2011;45(6):.

7009. Foley Brian, Jones Ian, Maberly Stephen, Rippey Brian Long-term changes in oxygen depletion in a small temperate lake: effects of climate change and eutrophication. FRESHWATER BIOLOGY. 2012;57(2):278-289.

7010. Reiter Christina, Graves Leah Nutrition Therapy for Eating Disorders. NUTRITION IN CLINICAL PRACTICE. 2010;25(2):122-136.

7011. De Pasquale Concetta, Pistorio Maria, Tornatore Eleonora, De Berardis Domenico, Fornaro Michele The relationship between drive to thinness, conscientiousness and bulimic traits during adolescence: a comparison between younger and older cases in 608 healthy volunteers. ANNALS OF GENERAL PSYCHIATRY. 2013;12():.

7012. Giner-Bartolome Cristina, Mallorqui-Bague Nuria, Tolosa-Sola Iris, Steward Trevor, Jimenez-Murcia Susana, Granero Roser, Fernandez-Aranda Fernando Non-suicidal Self-

Injury in Eating Disordered Patients: Associations with Heart Rate Variability and State-Trait Anxiety. FRONTIERS IN PSYCHOLOGY. 2017;8():.

7013. Yeomans Martin Alcohol, appetite and energy balance: Is alcohol intake a risk factor for obesity?. PHYSIOLOGY \& BEHAVIOR. 2010;100(1, SI):82-89.

7014. Navas-Leon Sergio, Sanchez-Martin Milagrosa, Tajadura-Jimenez Ana, De Coster Lize, Borda-Mas Mercedes, Morales Luis Eye movements and eating disorders: protocol for an exploratory experimental study examining the relationship in young-adult women with subclinical symptomatology. JOURNAL OF EATING DISORDERS. 2022;10(1):.

7015. Bundgaard Amanda, James Andrew, Joyce William, Murphy Michael, Fago Angela Suppression of reactive oxygen species generation in heart mitochondria from anoxic turtles: the role of complex I S-nitrosation. JOURNAL OF EXPERIMENTAL BIOLOGY. 2018;221(8):.

7016. Muller M, Ballanyi K Dynamic recording of cell death in the in vitro dorsal vagal nucleus of rats in response to metabolic arrest. JOURNAL OF NEUROPHYSIOLOGY. 2003;89(1):551-561.

7017. Oellingrath Inger, De Bortoli Marit, Svendsen Martin, Fell Anne Lifestyle and work ability in a general working population in Norway: a cross-sectional study. BMJ OPEN. 2019;9(4):.

7018. Shaw Heather, Rohde Paul, Stice Eric Using participant feedback to improve two selective eating disorder and obesity prevention programs. EATING BEHAVIORS. 2018;30():93-97.

7019. Phillipou Andrea, McGorry Patrick, Killackey Eoin, Maguire Sarah Eating disorders in young people. AUSTRALASIAN PSYCHIATRY. 2023;():.

7020. Cediel Gustavo, Reyes Marcela, Corvalan Camila, Levy Renata, Uauy Ricardo, Monteiro Carlos Ultra-processed foods drive to unhealthy diets: evidence from Chile. PUBLIC HEALTH NUTRITION. 2021;24(7):1698-1707.

7021. Souza Vinicius, Silva Eduardo, Ribeiro Mario, Martins Wolney Hypertension in Patients with Cancer. ARQUIVOS BRASILEIROS DE CARDIOLOGIA. 2015;104(3):246-252.

7022. MAIESE K, BONIECE I, DEMEO D, WAGNER JA PEPTIDE GROWTH-FACTORS PROTECT AGAINST ISCHEMIA IN CULTURE BY PREVENTING NITRIC-OXIDE TOXICITY. JOURNAL OF NEUROSCIENCE. 1993;13(7):3034-3040.

7023. Britton A., Marmot M., Shipley M. Who benefits most from the cardioprotective properties of alcohol consumption - health freaks or couch potatoes?. JOURNAL OF EPIDEMIOLOGY AND COMMUNITY HEALTH. 2008;62(10):905-908.

7024. Brown Carrie, Mehler Philip Anorexia Nervosa Complicated by Diabetes Mellitus: The Case for Permissive Hyperglycemia. INTERNATIONAL JOURNAL OF EATING DISORDERS. 2014;47(6):671-674.

7025. Sinha D, Wang ZY, Price VR, Schwartz JH, Lieberthal W Chemical anoxia of tubular cells induces activation of c-Src and its translocation to the zonula adherens. AMERICAN JOURNAL OF PHYSIOLOGY-RENAL PHYSIOLOGY. 2003;284(3):F488-F497.

7026. Slowakiewicz Miroslaw, Tucker Maurice, Hindenberg Katja, Mawson Mike, Idiz Erdem, Pancost Richard Nearshore euxinia in the photic zone of an ancient sea: Part II - The bigger picture and implications for understanding ocean anoxia. PALAEOGEOGRAPHY PALAEOCLIMATOLOGY PALAEOECOLOGY. 2016;461():432-448.

7027. Gidron Yori, Deschepper Reginald, De Couck Marijke, Thayer Julian, Velkeniers Brigitte The Vagus Nerve Can Predict and Possibly Modulate Non-Communicable Chronic Diseases: Introducing a Neuroimmunological Paradigm to Public Health. JOURNAL OF CLINICAL MEDICINE. 2018;7(10):.

7028. Oakey-Frost Nicolas, Trachik Benjamin, Ganulin Michelle, LoPresti Mathew, Dretsch Michael, Tucker Raymond Indirect effects of soldier healthy eating and physical activity on suicidal ideation through psychological health symptoms in active-duty military. MILITARY PSYCHOLOGY. 2022;34(3, SI):305-314.

7029. Dickie Liza, Wilson Marc, McDowall John, Surgenor Lois What Components of Perfectionism Predict Drive for Thinness?. EATING DISORDERS. 2012;20(3):232-247.

7030. Rabin Carolyn, Pinto Bernardine Cancer-related beliefs and health behavior change among breast cancer survivors and their first-degree relatives. PSYCHO-ONCOLOGY. 2006;15(8):701-712.

7031. Marlinna Marlinna, Julia Madarina, Noormanto Noormanto A 14-year-old patient with Prader-Willi syndrome: a case report. PAEDIATRICA INDONESIANA. 2023;63(1):51-56.

7032. Vallivattathillam Parvathi, Iyyappan Suresh, Lengaigne Matthieu, Ethe Christian, Vialard Jerome, Levy Marina, Suresh Neetu, Aumont Olivier, Resplandy Laure, Naik Hema, Naqvi Wajih Positive Indian Ocean Dipole events prevent anoxia off the west coast of India. BIOGEOSCIENCES. 2017;14(6):1541-1559.

7033. Xie Xiaochun, Gai Xiaosong, Zhou Yong A meta-analysis of media literacy interventions for deviant behaviors. COMPUTERS \& EDUCATION. 2019;139():146-156.

7034. Popa Amorin, Vesa Cosmin, Uivarosan Diana, Jurca Claudia, Isvoranu Gheorghita, Socea Bogdan, Stanescu Ana, Iancu Mihaela, Scarneciu Ioan, Zaha Dana Cross Sectional Study Regarding the Association Between Sweetened Beverages Intake, Fast-food Products, Body Mass Index, Fasting Blood Glucose and Blood Pressure in the Young Adults from North-western Romania. REVISTA DE CHIMIE. 2019;70(1):156-160.

7035. Leon Hernandez Rodrigo, Gomez-Peresmitre Gilda, Platas Acevedo Silvia Risk eating behaviors and social skills in a sample of Mexican adolescents. SALUD MENTAL. 2008;31(6):447-452.

7036. Jeste DV Tardive dyskinesia rates with atypical antipsychotics in older adults. JOURNAL OF CLINICAL PSYCHIATRY. 2004;65(9):21-24.

7037. Kucharczuk Adam, Oliver Tracy, Dowdell Elizabeth Social media's influence on adolescents ` food choices: A mixed studies systematic literature review. APPETITE. 2022;168():.

7038. Duart-Castells Leticia, Cantacorps Lidia, Lopez-Arnau Raul, Montagud-Romero Sandra, Puster Brigitte, Mera Paula, Serra Dolors, Camarasa Jorge, Pubill David, Valverde Olga, Escubedo Elena Effects of High-Fat Diet and Maternal Binge-Like Alcohol Consumption and Their Influence on Cocaine Response in Female Mice Offspring. INTERNATIONAL JOURNAL OF NEUROPSYCHOPHARMACOLOGY. 2021;24(1):77-88.

7039. Franko DL, Bean J, Tamer R, Dohm FA, Schreiber G, Striegel-Moore RH, Kraemer HC, Crawford PB, Daniels SR Psychosocial and health consequences of adolescent depression in black and white young adult women. HEALTH PSYCHOLOGY. 2005;24(6):586-593.

7040. Rodriguez-Caro H., Williams S. Strategies to reduce non-communicable diseases in the offspring: negative and positive in utero programming. JOURNAL OF DEVELOPMENTAL ORIGINS OF HEALTH AND DISEASE. 2018;9(6):642-652.

7041. Ali Asad, Akhtar Juber, Ahmad Usama, Basheer Abdul, Jaiswal Neha, Jahan Afroz Armamentarium in Drug Delivery for Colorectal Cancer. CRITICAL REVIEWS IN THERAPEUTIC DRUG CARRIER SYSTEMS. 2023;40(1):1-48.

7042. Ruuska J, Kaltiala-Heino R, Koivisto AM, Rantanen P Puberty, sexual development and eating disorders in adolescent outpatients. EUROPEAN CHILD \& ADOLESCENT PSYCHIATRY. 2003;12(5):214-220.

7043. Kirk Sara, Olstad Dana, Mcisaac Jessie-Lee, Prowse Rachel, Caswell Susan, Hanning Rhona, Raine Kim, Masse Louise, Naylor P. Appetite for change? Facilitators and barriers to nutrition guideline implementation in Canadian recreational facilities. HEALTH PROMOTION INTERNATIONAL. 2021;36(6):1672-1682.

7044. Herpertz-Dahlmann Beate, Buehren Katharina, Remschmidt Helmut Growing Up Is Hard Mental Disorders in Adolescence. DEUTSCHES ARZTEBLATT INTERNATIONAL. 2013;110(25):432-U22.

7045. Rezaul I, Persaud R, Takei N, Treasure J Season of birth and eating disorders. INTERNATIONAL JOURNAL OF EATING DISORDERS. 1996;19(1):53-61.

7046. Brown Stephanie, Manning Katherine, Fletcher Paul, Holland Anthony In vivo neuroimaging evidence of hypothalamic alteration in Prader-Willi syndrome. BRAIN COMMUNICATIONS. 2022;4(5):.

7047. Paulus Frank, Ohmann Susanne, Moehler Eva, Plener Paul, Popow Christian Emotional Dysregulation in Children and Adolescents With Psychiatric Disorders. A Narrative Review. FRONTIERS IN PSYCHIATRY. 2021;12():.

7048. Vannucchi G., Medda P., Pallucchini A., Bertelli M., Angst J., Azorin J-M, Bowden C., Vieta E., Young A., Mosolov S., Perugi G., Grp BRIDGE-II-Mix The relationship between attention deficit hyperactivity disorder, bipolarity and mixed features in major depressive patients: Evidence from the BRIDGE-II-Mix Study. JOURNAL OF AFFECTIVE DISORDERS. 2019;246():346-354.

7049. Stice E, Hayward C, Cameron RP, Killen JD, Taylor CB Body-image and eating disturbances predict onset of depression among female adolescents: A longitudinal study. JOURNAL OF ABNORMAL PSYCHOLOGY. 2000;109(3):438-444.

7050. Alsubaie Ali Consumption and correlates of sweet foods, carbonated beverages, and energy drinks among primary school children in Saudi Arabia. SAUDI MEDICAL JOURNAL. 2017;38(10):1045-1050.

7051. Milos G, Spindler A, Ruegsegger P, Seifert B, Muhlebach S, Uebelhart D, Hauselmann HJ Cortical and trabecular bone density and structure in anorexia nervosa. OSTEOPOROSIS INTERNATIONAL. 2005;16(7):783-790.

7052. Zhuravlev Andrey, Wood Rachel Eve of biomineralization: Controls on skeletal mineralogy. GEOLOGY. 2008;36(12):923-926.

7053. Balottin U, Nicoli F, Pitillo G, Ginevra OF, Borgatti R, Lanzi G Migraine and tension headache in children under 6 years of age. EUROPEAN JOURNAL OF PAIN. 2004;8(4):307-314.

7054. Scappaticcio Lorenzo, Maiorino Maria, Bellastella Giuseppe, Giugliano Dario, Esposito Katherine Insights into the relationships between diabetes, prediabetes, and cancer. ENDOCRINE. 2017;56(2):231-239.

7055. Eriksson Emmi, Ramklint Mia, Wolf-Arehult Martina, Isaksson Martina The relationship between self-control and symptoms of anxiety and depression in patients with eating disorders: a cross-sectional study including exploratory longitudinal data. JOURNAL OF EATING DISORDERS. 2023;11(1):.

7056. Mroweh Mariam, Roth Gael, Decaens Thomas, Marche Patrice, Lerat Herve, Jilkova Zuzana Targeting Akt in Hepatocellular Carcinoma and Its Tumor Microenvironment. INTERNATIONAL JOURNAL OF MOLECULAR SCIENCES. 2021;22(4):.

7057. Ackard DM, Neumark-Sztainer D, Story M, Perry C Parent-child connectedness and behavioral and emotional health among adolescents. AMERICAN JOURNAL OF PREVENTIVE MEDICINE. 2006;30(1):59-66.

7058. Bentum Elisabeth, Hetzel Almut, Brumsack Hans-J., Forster Astrid, Reichart Gert-Jan, Damste Jaap Reconstruction of water column anoxia in the equatorial Atlantic during the Cenomanian-Turonian oceanic anoxic event using biomarker and trace metal proxies. PALAEOGEOGRAPHY PALAEOCLIMATOLOGY PALAEOECOLOGY. 2009;280(3-4):489-498.

7059. Izydorczyk Bernadetta, Sitnik-Warchulska Katarzyna Sociocultural Appearance Standards and Risk Factors for Eating Disorders in Adolescents and Women of Various Ages. FRONTIERS IN PSYCHOLOGY. 2018;9():.

7060. Wu YW, Shek DW, Garcia PA, Zhao S, Johnston SC Incidence and mortality of generalized convulsive status epilepticus in California. NEUROLOGY. 2002;58(7):1070-1076.

7061. Thomas Annie, Janusek Linda Obesity Prevention Behaviors in Asian Indian Adolescent Girls: A Pilot Study. JOURNAL OF PEDIATRIC NURSING-NURSING CARE OF CHILDREN \& FAMILIES. 2018;42():9-15.

7062. Tung Serene, Nasir Mohd, Chin Yit, Zalilah Mohd, Zubaidah Jamil, Yim Hip Psychological Factors and Cardiovascular Disease Risk Factors as Mediators of the Relationship between Overweight/Obesity and Cognitive Function among School Children in Kuala Lumpur, Malaysia. CHILDHOOD OBESITY. 2019;15(1):56-62.

7063. Beckmann Britta, Hofmann Peter, Maerz Christian, Schouten Stefan, Damste Jaap, Wagner Thomas Coniacian-Santonian deep ocean anoxia/euxinia inferred from molecular and inorganic markers: Results from the Demerara Rise (ODP Leg 207). ORGANIC GEOCHEMISTRY. 2008;39(8):1092-1096.

7064. Trost Stewart, Messner Lana, Fitzgerald Karen, Roths Barbara Nutrition and Physical Activity Policies and Practices in Family Child Care Homes. AMERICAN JOURNAL OF PREVENTIVE MEDICINE. 2009;37(6):537-540.

7065. Li Qian, Li Chuanyu, Mahtani Harry, Du Jian, Patel Aashka, Lancaster Jack Nitrosothiol Formation and Protection against Fenton Chemistry by Nitric Oxide-induced Dinitrosyliron Complex Formation from Anoxia-initiated Cellular Chelatable Iron Increase. JOURNAL OF

BIOLOGICAL CHEMISTRY. 2014;289(29):19917-19927.

7066. Waqa Gade, Moodie Marj, Schultz Jimaima, Swinburn Boyd Process evaluation of a community-based intervention program: Healthy Youth Healthy Communities, an adolescent obesity prevention project in Fiji. GLOBAL HEALTH PROMOTION. 2013;20(4):23-34.

7067. Kletkiewicz Hanna, Klimiuk Maciej, Wozniak Alina, Mila-Kierzenkowska Celestyna, Dokladny Karol, Rogalska Justyna How to Improve the Antioxidant Defense in Asphyxiated Newborns-Lessons from Animal Models. ANTIOXIDANTS. 2020;9(9):.

7068. Wang Mengyao, Brage Soren, Sharp Stephen, Luo Shan, Au Yeung Shiu, Kim Youngwon Associations of genetic susceptibility and healthy lifestyle with incidence of coronary heart disease and stroke in individuals with hypertension. EUROPEAN JOURNAL OF PREVENTIVE CARDIOLOGY. 2022;():.

7069. Yary Teymoor, Soleimannejad Kourosh, Abd Rahim Firdaus, Kandiah Mirnalini, Aazami Sanaz, Poor Seyedehozma, Wee Wong, Aazami Golnaz Contribution of diet and major depression to incidence of acute myocardial infarction (AMI). LIPIDS IN HEALTH AND DISEASE. 2010;9():.

7070. Rodgers Rachel, Simone Melissa, Franko Debra, Eisenberg Marla, Loth Katie, Neumark-Sztainer Dianne The longitudinal relationship between family and peer teasing in young adulthood and later unhealthy weight control behaviors: The mediating role of body image. INTERNATIONAL JOURNAL OF EATING DISORDERS. 2021;54(5):831-840.

7071. Brunault Paul, Salame Ephrem, Jaafari Nematollah, Courtois Robert, Reveillere Christian, Silvain Christine, Benyamina Amine, Blecha Lisa, Belin David, Ballon Nicolas Why do liver transplant patients so often become obese? The addiction transfer hypothesis. MEDICAL HYPOTHESES. 2015;85(1):68-75.

7072. Zhang Shiyao, Xu Mengyi, Shen Ziyue, Shang Changrui, Zhang Wenxiang, Chen Siyu, Liu Chang Green light exposure aggravates high-fat diet feeding-induced hepatic steatosis and pancreatic dysfunction in male mice. ECOTOXICOLOGY AND ENVIRONMENTAL SAFETY. 2021;225():.

7073. Mousavi Seyed, Shayanfar Mehdi, Rigi Somaye, Mohammad-Shirazi Minoo, Sharifi Giuve, Esmaillzadeh Ahmad Adherence to plant-based dietary patterns in relation to glioma:

a case-control study. SCIENTIFIC REPORTS. 2021;11(1):.

7074. Morrison Amy, Fleming Suzannah, Levy Miles A review of the pathophysiology of functional hypothalamic amenorrhoea in women subject to psychological stress, disordered eating, excessive exercise or a combination of these factors. CLINICAL ENDOCRINOLOGY. 2021;95(2):229-238.

7075. Gordon Allegra, Austin S., Schultz Jordan, Guss Carly, Calzo Jerel, Wang Monica Gender Expression, Peer Victimization, and Disordered Weight-Control Behaviors Among US High School Students. JOURNAL OF ADOLESCENT HEALTH. 2021;68(6):1148-1154.

7076. Marciello Francesca, Monteleone Alessio, Cascino Giammarco, Patriciello Giuseppi, Pellegrino Francesca, Fiorenza Gianmarco, Monteleone Palmiero Early traumatic experiences and eating disorders: a focus on the endogenous stress response system. JOURNAL OF PSYCHOPATHOLOGY. 2020;26(1, SI):77-84.

7077. Rodriguez-Tovar Francisco Ichnology of the Toarcian Oceanic Anoxic Event: An understimated tool to assess palaeoenvironmental interpretations. EARTH-SCIENCE REVIEWS. 2021;216():.

7078. Funtikova A., Baena-Diez J., Koebnick C., Gomez S., Covas M-I, Goday A., Schroeder H. Validity of a short diet-quality index to predict changes in anthropometric and cardiovascular risk factors: a simulation study. EUROPEAN JOURNAL OF CLINICAL NUTRITION. 2012;66(12):1369-1371.

7079. Hossein-Javaheri Nariman, Buck Leslie GABA receptor inhibition and severe hypoxia induce a paroxysmal depolarization shift in goldfish neurons. JOURNAL OF NEUROPHYSIOLOGY. 2021;125(2):321-330.

7080. Martinez-Mallen Esteban, Castro-Fornieles Josefina, Lazaro Luisa, Moreno Elena, Morer Astrid, Font Elena, Julien Joana, Vila Montserrat, Toro Josep Cue exposure in the treatment of resistant adolescent bulimia nervosa. INTERNATIONAL JOURNAL OF EATING DISORDERS. 2007;40(7):596-601.

7081. Hosseini Naeimeh, Talaei Mohammad, Dianatkhah Minoo, Sadeghi Masoumeh, Oveisgharan Shahram, Sarrafzadegan Nizal Determinants of Incident Metabolic Syndrome in a Middle Eastern Population: Isfahan Cohort Study. METABOLIC SYNDROME AND

RELATED DISORDERS. 2017;15(7):354-362.

7082. Yela Bernabe Jose, Gomez Martinez Ma, Cortes Rodriguez Maria, Salgado Ruiz Alfonso Effects of exposure to food images on physiological reactivity and emotional responses in women with bulimia nervosa. PSICOTHEMA. 2013;25(2):185-191.

7083. Mikolajczyk Rafael, Maxwell Annette, El Ansari Walid, Stock Christiane, Petkeviciene Janina, Guillen-Grima Francisco Relationship between perceived body weight and body mass index based on self- reported height and weight among university students: a cross-sectional study in seven European countries. BMC PUBLIC HEALTH. 2010;10():.

7084. Bauer Stephanie, Bilic Sally, Reetz Christina, Ozer Fikret, Becker Katja, Eschenbeck Heike, Kaess Michael, Rummel-Kluge Christine, Salize Hans-Joachim, Diestelkamp Silke, Moessner Markus, Thomasius Rainer, Bertsch Katja, Brunner Romuald, Feldhege Johannes, Gallinat Christina, Herpertz Sabine, Koenig Julian, Lustig Sophia, Parzer Peter, Resch Franz, Ritter Sabrina, Spinner Jens, Wille Kristina, Baldofski Sabrina, Kohls Elisabeth, Peter Lina-Jolien, Gille Vera, Hofmann Hanna, Lehner Laya, Voss Elke, Pfeiffer Jens, Samel Alisa, Consortium ProHEAD Efficacy and cost-effectiveness of Internet-based selective eating disorder prevention: study protocol for a randomized controlled trial within the ProHEAD Consortium. TRIALS. 2019;20():.

7085. Gebremariam Lemlem, Aoyama Atsuko, Kahsay Alemayehu, Hirakawa Yoshihisa, Chiang Chifa, Yatsuya Hiroshi, Matsuyama Akiko Perception and practice of `healthy' diet in relation to noncommunicable diseases among the urban and rural people in northern Ethiopia: a community-based qualitative study. NAGOYA JOURNAL OF MEDICAL SCIENCE. 2018;80(4):451-464.

7086. Pasupathy Dharmintra, Wood Angela, Pell Jill, Fleming Michael, Smith Gordon Rates of and Factors Associated With Delivery-Related Perinatal Death Among Term Infants in Scotland. JAMA-JOURNAL OF THE AMERICAN MEDICAL ASSOCIATION. 2009;302(6):660-668.

7087. Solomos T, Gross KC Effects of hypoxia on respiration and the onset of senescence in cut carnation flowers (Dianthus caryophyllus L). POSTHARVEST BIOLOGY AND TECHNOLOGY. 1997;10(2):145-153.

7088. Chapelon Emeline, Barry Caroline, Hubert Tamara, Com-Ruelle Laure, Duclos Jeanne, Mattar Lama, Falissard Bruno, Huas Caroline, Godart Nathalie Health in adulthood after severe anorexia nervosa in adolescence: a study of exposed and unexposed women. EATING

AND WEIGHT DISORDERS-STUDIES ON ANOREXIA BULIMIA AND OBESITY. 2021;26(5):1389-1397.

7089. Mammarella Ivan, Gavrylenko Galyna, Zdorovennova Galina, Ojala Anne, Erkkila Kukka-Maaria, Zdorovennov Roman, Stepanyuk Oleg, Palshin Nikolay, Terzhevik Arkady, Vesala Timo, Heiskanen Jouni Effects of similar weather patterns on the thermal stratification, mixing regimes and hypolimnetic oxygen depletion in two boreal lakes with different water transparency. BOREAL ENVIRONMENT RESEARCH. 2018;23():237-247.

7090. Lee Amanda, Lewis Meron Testing the Price of Healthy and Current Diets in Remote Aboriginal Communities to Improve Food Security: Development of the Aboriginal and Torres Strait Islander Healthy Diets ASAP (Australian Standardised Affordability and Pricing) Methods. INTERNATIONAL JOURNAL OF ENVIRONMENTAL RESEARCH AND PUBLIC HEALTH. 2018;15(12):.

7091. Reddy KS, Katan MB Diet, nutrition and the prevention of hypertension and cardiovascular diseases. PUBLIC HEALTH NUTRITION. 2004;7(1A, SI):167-186.

7092. Madden Sloane, Morris Anne, Zurynski Yvonne, Kohn Michael, Elliot Elizabeth Burden of eating disorders in 5-13-year-old children in Australia. MEDICAL JOURNAL OF AUSTRALIA. 2009;190(8):410-414.

7093. Alvisi F., Giani M., Ravaioli M., Giordano P. Role of sedimentary environment in the development of hypoxia and anoxia in the NW Adriatic shelf (Italy). ESTUARINE COASTAL AND SHELF SCIENCE. 2013;128():9-21.

7094. Regulska-Ilow Bozena, Ilow Rafal, Biernat Jadwiga, Grajeta Halina, Kowalski Przemyslaw The influence of bioflavonoids from the radix of Scutellaria baicalensis on liver function of laboratory rats fed with fresh and oxidized fats. ADVANCES IN CLINICAL AND EXPERIMENTAL MEDICINE. 2007;16(1):13-20.

7095. Pathirana Thanya, Stoneman Rebecca, Lamont Amanda, Harris Neil, Lee Patricia Impact evaluation of ``Have Fun Be Healthy{''} program: A community based health promotion intervention to prevent childhood obesity. HEALTH PROMOTION JOURNAL OF AUSTRALIA. 2018;29(1):100-104.

7096. Wanke EM, Petruschke A, Korsten-Reck U Eating disorders in athletes - an inventory. DEUTSCHE ZEITSCHRIFT FUR SPORTMEDIZIN. 2004;55(11):286+.

7097. Ghai HS, Buck LT Acute reduction in whole cell conductance in anoxic turtle brain. AMERICAN JOURNAL OF PHYSIOLOGY-REGULATORY INTEGRATIVE AND COMPARATIVE PHYSIOLOGY. 1999;277(3):R887-R893.

7098. Carducci Bianca, Oh Christina, Keats Emily, Roth Daniel, Bhutta Zulfiqar Effect of Food Environment Interventions on Anthropometric Outcomes in School-Aged Children and Adolescents in Low- and Middle-Income Countries: A Systematic Review and Meta-Analysis. CURRENT DEVELOPMENTS IN NUTRITION. 2020;4(7):.

7099. Kheirouri Sorayya, Alizadeh Mohammad Dietary Inflammatory Potential and the Risk of Incident Depression in Adults: A Systematic Review. ADVANCES IN NUTRITION. 2019;10(1):9-18.

7100. Berendsen Maxime, Boss Myrthe, Smits Marcel, Pot Gerda Chrono-Nutrition and Diet Quality in Adolescents with Delayed Sleep-Wake Phase Disorder. NUTRIENTS. 2020;12(2):.

7101. Liao Yi-Hung, Chen Chung-Yu, Chen Chiao-Nan, Wu Chia-Ying, Tsai Shiow-Chwen An Amino Acids Mixture Attenuates Glycemic Impairment but not Affects Adiposity Development in Rats Fed with AGEs-containing Diet. INTERNATIONAL JOURNAL OF MEDICAL SCIENCES. 2018;15(2):176-187.

7102. Carter FA, McIntosh VVW, Joyce PR, Gendall KA, Bulik CM Impact of pre-treatment weight on weight trajectory in women treated for bulimia nervosa. EUROPEAN EATING DISORDERS REVIEW. 2004;12(6):387-391.

7103. Yau Y., Potenza M. Stress and eating behaviors. MINERVA ENDOCRINOLOGICA. 2013;38(3):255-267.

7104. Helmond Niels, Sluijs Appy, Reichart Gert-Jan, Damste Jaap, Slomp Caroline, Brinkhuis Henk A perturbed hydrological cycle during Oceanic Anoxic Event 2. GEOLOGY. 2014;42(2):123-126.

7105. Popkin Barry The Challenge in Improving the Diets of Supplemental Nutrition Assistance Program Recipients: A Historical Commentary. AMERICAN JOURNAL OF

PREVENTIVE MEDICINE. 2017;52(2, 2):S106-S114.

7106. Berciano Silvia, Ordovas Jose Nutrition and Cardiovascular Health. REVISTA ESPANOLA DE CARDIOLOGIA. 2014;67(9):738-747.

7107. Parekh Niyati, Henriksson Pontus, Nystrom Christine, Silfvernagel Kristin, Ruiz Jonatan, Ortega Francisco, Pomeroy Jeremy, Lof Marie Associations of Parental Self-Efficacy With Diet, Physical Activity, Body Composition, and Cardiorespiratory Fitness in Swedish Preschoolers: Results From the MINISTOP Trial. HEALTH EDUCATION \& BEHAVIOR. 2018;45(2):238-246.

7108. Stapley Emily, Midgley Nick, Target Mary The Experience of Being the Parent of an Adolescent with a Diagnosis of Depression. JOURNAL OF CHILD AND FAMILY STUDIES. 2016;25(2):618-630.

7109. Aguirre-Portoles Cristina, Fernandez Lara, Molina Ana Precision Nutrition for Targeting Lipid Metabolism in Colorectal Cancer. NUTRIENTS. 2017;9(10):.

7110. Gurne Frida, Svensson Per-Arne, Bjorkman Ida, Liden Eva, Jakobsson Sofie Seeking lifestyle counselling at primary health care centres: a cross-sectional study in the Swedish population. BMC PRIMARY CARE. 2023;24(1):.

7111. LaRose Jessica, Neiberg Rebecca, Evans E., Tate Deborah, Espeland Mark, Gorin Amy, Perdue Letitia, Hatley Karen, Lewis Cora, Robichaud Erica, Wing Rena, Ferguson Erica, Almeida Ana, Annis Kristen, Busha Ryan, Cassell Isabella, Chen Eva, Coward Pamela, DaCruz Jose, Egan Caitlin, Fisher Michelle, Guerra Stephanie, Himes Susan, James Brittany, Kearns Marie, McHugh Angelica, O'Leary Kevin, Palmer Kathy, Ranslow-Robles Deborah, Samuels Amanda, Story Kathryn, Strohacker Kelly, Sylvia Zeely, Trautvetter Jennifer, Unick Jessica, Whitehead Kristen, Williams Samantha, Wunsch Carolyn, Yolken Annajane, Tate Deborah, Hatley Karen, Alick Candace, Barnes Shelia, Blackman Loneke, Bordogna Rachel, Cooper Kimberly, Crane Melissa, Cryer Victoria, Diamond Molly, Frank Jennifer, Kulik Noel, Lerner Hannah, McMullin Megan, Polzien Kristen, Quick Keneisha, Tompkins Brooke, Turner-McGrievey Brie, Valle Carmina, Zablonski Stephen, Zeigler Erin, Gorin Amy, Perdue Letitia, Bahnson Judy, Lang Wei, Bentley Cheryl, Davis Patty, Garcia Katelyn, Griffin Leah, Harvin Lea, Hontz Mary, King Mark, Lane Kathy, Robertson Julia, Marcovina Santica, Hurting Jessica, Gaur Vinod, Arteaga S., Loria Catherine, Gain Study Dietary outcomes within the study of novel approaches to weight gain prevention (SNAP) randomized controlled trial. INTERNATIONAL JOURNAL OF BEHAVIORAL NUTRITION AND PHYSICAL ACTIVITY.

2019;16():.

7112. Kris-Etherton Penny, Sapp Philip, Riley Terrance, Davis Kristin, Hart Tricia, Lawler Olivia The Dynamic Interplay of Healthy Lifestyle Behaviors for Cardiovascular Health. CURRENT ATHEROSCLEROSIS REPORTS. 2022;24(12):969-980.

7113. Cummings Kevin, Hewitt Julie, Li Aihua, Daubenspeck John, Nattie Eugene Postnatal loss of brainstem serotonin neurones compromises the ability of neonatal rats to survive episodic severe hypoxia. JOURNAL OF PHYSIOLOGY-LONDON. 2011;589(21):5247-5256.

7114. Jain Akhilesh, Agrawal Aditi, Tripathi Anil, Bansod Roovendra, Jain Garima, Yadav Kuldeep Trichobezoar without a clear manifestation of trichotillomania. JOURNAL OF FAMILY MEDICINE AND PRIMARY CARE. 2020;9(5):2566-2568.

7115. Harshbarger J., Ahlers-Schmidt C., Atif M., Allred E., Carroll M., Hauser R. School counselors' knowledge of eating disorders. EATING AND WEIGHT DISORDERS-STUDIES ON ANOREXIA BULIMIA AND OBESITY. 2011;16(2):E131-E136.

7116. Fujisawa Takashi, Yatsuga Chiho, Mabe Hiroyo, Yamada Eiji, Masuda Masato, Tomoda Akemi Anorexia Nervosa during Adolescence Is Associated with Decreased Gray Matter Volume in the Inferior Frontal Gyrus. PLOS ONE. 2015;10(6):.

7117. Adjibade Moufidath, Lemogne Cedric, Julia Chantal, Hercberg Serge, Galan Pilar, Assmann Karen, Kesse-Guyot Emmanuelle Prospective association between combined healthy lifestyles and risk of depressive symptoms in the French NutriNet-Sante cohort. JOURNAL OF AFFECTIVE DISORDERS. 2018;238():554-562.

7118. Bolton K., Kremer P., Gibbs L., Waters E., Swinburn B., Silva A. The outcomes of health-promoting communities: being active eating well initiative-a community-based obesity prevention intervention in Victoria, Australia. INTERNATIONAL JOURNAL OF OBESITY. 2017;41(7):1080-1090.

7119. Renton T., Yilmaz Z., Gaballah K. Evaluation of trigeminal nerve injuries in relation to third molar surgery in a prospective patient cohort. Recommendations for prevention. INTERNATIONAL JOURNAL OF ORAL AND MAXILLOFACIAL SURGERY. 2012;41(12):1509-1518.

7120. Winter Channa, Magilsen Karla, Alfen J., Penning Corine, Evenhuis Heleen Prevalence of Cardiovascular Risk Factors in Older People With Intellectual Disability. AJIDD-AMERICAN JOURNAL ON INTELLECTUAL AND DEVELOPMENTAL DISABILITIES. 2009;114(6):427-436.

7121. Baldwin Scott, Bauer Daniel, Stice Eric, Rohde Paul Evaluating Models for Partially Clustered Designs. PSYCHOLOGICAL METHODS. 2011;16(2):149-165.

7122. Maiztegui Barbara, Lisi Roman Carolina, Prime Gagliardino Juan, Emilio Flores Luis Impaired endocrine-metabolic homeostasis: underlying mechanism of its induction by unbalanced diet. CLINICAL SCIENCE. 2018;132(8):869-881.

7123. Barkus Emma, Badcock Johanna A Transdiagnostic Perspective on Social Anhedonia. FRONTIERS IN PSYCHIATRY. 2019;10():.

7124. Scott A, Khan KM, Duronio V IGF-I activates PKB and prevents anoxic apoptosis in Achilles tendon cells. JOURNAL OF ORTHOPAEDIC RESEARCH. 2005;23(5):1219-1225.

7125. Poncova R., Skrenkova J., Fanta M. Eating disorders in the ambulance of pediatric and adolescence gynecology. CESKA GYNEKOLOGIE-CZECH GYNAECOLOGY. 2021;86(1):46-53.

7126. Pandey Ankita, Chawla Sheetal, Guchhait Prasenjit Type-2 Diabetes: Current Understanding and Future Perspectives. IUBMB LIFE. 2015;67(7, SI):506-513.

7127. Avidan Alon Parasomnias and Movement Disorders of Sleep. SEMINARS IN NEUROLOGY. 2009;29(4):372-392.

7128. Badon Sylvia, Enquobahrie Daniel, Wartko Paige, Miller Raymond, Qiu Chunfang, Gelaye Bizu, Sorensen Tanya, Williams Michelle Healthy Lifestyle During Early Pregnancy and Risk of Gestational Diabetes Mellitus. AMERICAN JOURNAL OF EPIDEMIOLOGY. 2017;186(3):326-333.

7129. Strong KG, Huon GF Controlled motivation and the persistence of weight-loss dieting. EUROPEAN EATING DISORDERS REVIEW. 1999;7(2):136-146.

7130. Kruglov Alexey, Subbotina Ksenia, Saris Nils-Erik Redox-cycling compounds can cause the permeabilization of mitochondrial membranes by mechanisms other than ROS

production. FREE RADICAL BIOLOGY AND MEDICINE. 2008;44(4):646-656.

7131. Douglas Valerie, Kwan Mun, Gordon Kathryn The roles of weight stigma, emotion dysregulation, and eating pathology in suicide risk. BODY IMAGE. 2021;38():162-170.

7132. Joy Elizabeth, De Souza Mary, Nattiv Aurelia, Misra Madhusmita, Williams Nancy, Mallinson Rebecca, Gibbs Jenna, Olmsted Marion, Goolsby Marci, Matheson Gordon, Barrack Michelle, Burke Louise, Drinkwater Barbara, Lebrun Connie, Loucks Anne, Mountjoy Margo, Nichols Jeanne, Borgen Jorunn 2014 Female Athlete Triad Coalition Consensus Statement on Treatment and Return to Play of the Female Athlete Triad. CURRENT SPORTS MEDICINE REPORTS. 2014;13(4):219-232.

7133. Swinburn Boyd Obesity Prevention in Children and Adolescents. CHILD AND ADOLESCENT PSYCHIATRIC CLINICS OF NORTH AMERICA. 2009;18(1):209+.

7134. Dalley Simon, Vidal Jose, Buunk Abraham, Schmitt Silvia, Haugwitz Ann-Christin, Kinds Nicole, Vlasma Anita Disentangling relations between the desirability of the thin-ideal, body checking, and worry on college women's weight-loss dieting: A self-regulation perspective. EATING BEHAVIORS. 2019;34():.

7135. Shlisky Julie, Bloom David, Beaudreault Amy, Tucker Katherine, Keller Heather, Freund-Levi Yvonne, Fielding Roger, Cheng Feon, Jensen Gordon, Wu Dayong, Meydani Simin Nutritional Considerations for Healthy Aging and Reduction in Age-Related Chronic Disease. ADVANCES IN NUTRITION. 2017;8(1):17-26.

7136. wang Monica, Peterson Karen, McCormick Marie, Austin S. Environmental factors associated with disordered weight-control behaviours among youth: a systematic review. PUBLIC HEALTH NUTRITION. 2014;17(7):1654-1667.

7137. Mayne S., Auchincloss A., Michael Y. Impact of policy and built environment changes on obesity-related outcomes: a systematic review of naturally occurring experiments. OBESITY REVIEWS. 2015;16(5):362-375.

7138. Drobnjak Suzana, Ehlert Ulrike Shape and Weight Concerns in Middle-Aged Women and the Relationship to Negative Affect and Education. VERHALTENSTHERAPIE. 2012;22(3):165-171.

7139. Rojo Luis, Conesa Llanos, Bermudez Ovidto, Livianos Lorenzo Influence of stress in the onset of eating disorders: Data from a two-stage epidemiologic controlled study. PSYCHOSOMATIC MEDICINE. 2006;68(4):628-635.

7140. Saucedo-Molina Teresita, Hernandez Leyda, Bautista-Diaz Maria Risk to develop body thin-ideal internalization in Mexican female adolescents. INTERDISCIPLINARIA. 2021;38(3):155-168.

7141. Fleming Richard Safety of ephedra and related anorexic medications. EXPERT OPINION ON DRUG SAFETY. 2008;7(6):749-759.

7142. Leone JE, Sedory EJ, Gray KA Recognition and treatment of muscle dysmorphia and related body image disorders. JOURNAL OF ATHLETIC TRAINING. 2005;40(4):352-359.

7143. Thiels C Childhood and adolescence of sufferers of eating disorders. PSYCHOTHERAPEUT. 2004;49(1):21-26.

7144. Orloff Natalia, Hormes Julia Pickles and ice cream! Food cravings in pregnancy: hypotheses, preliminary evidence, and directions for future research. FRONTIERS IN PSYCHOLOGY. 2014;5():.

7145. Pesola GR, Avasarala J Bupropion seizure proportion among new-onset generalized seizures and drug related seizures presenting to an Emergency Department. JOURNAL OF EMERGENCY MEDICINE. 2002;22(3):235-239.

7146. Amerzadeh Mohammad, Takian Amirhossein, Pouraram Hamed, Sari Ali, Ostovar Afshin The health system barriers to a healthy diet in Iran. PLOS ONE. 2023;18(1):.

7147. LEITENBERG H, ROSEN JC, WOLF J, VARA LS, DETZER MJ, SREBNIK D COMPARISON OF COGNITIVE-BEHAVIOR THERAPY AND DESIPRAMINE IN THE TREATMENT OF BULIMIA-NERVOSA. BEHAVIOUR RESEARCH AND THERAPY. 1994;32(1):37-45.

7148. Gilbert Kirsten The neglected role of positive emotion in adolescent psychopathology. CLINICAL PSYCHOLOGY REVIEW. 2012;32(6):467-481.

7149. Hoback WW, Stanley DW, Higley LG, Barnhart MC Survival of immersion and anoxia by larval tiger beetles, Cicindela togata. AMERICAN MIDLAND NATURALIST.

1998;140(1):27-33.

7150. Cossu Giovanni, Rinaldi Roberta, Colosimo Carlo The rise and fall of impulse control behavior disorders. PARKINSONISM \& RELATED DISORDERS. 2018;46(1):S24-S29.

7151. Lowery Christopher, Leckie R., Sageman Bradley Micropaleontological evidence for redox changes in the OAE3 interval of the US Western Interior: Global vs. local processes. CRETACEOUS RESEARCH. 2017;69():34-48.

7152. Hasan Shamimul, Ahmed Sameer, Panigrahi Rajat, Chaudhary Priyadarshini, Vyas Vijeta, Saeed Shazina Oral cavity and eating disorders: An insight to holistic health. JOURNAL OF FAMILY MEDICINE AND PRIMARY CARE. 2020;9(8):3890-3897.

7153. Carbone Salvatore, Del Buono Marco, Ozemek Cemal, Lavie Carl Obesity, risk of diabetes and role of physical activity, exercise training and cardiorespiratory fitness. PROGRESS IN CARDIOVASCULAR DISEASES. 2019;62(4):327-333.

7154. Pisot Sasa, Simunic Bostjan, Gentile Ambra, Bianco Antonino, Lo Coco Gianluca, Pisot Rado, Drid Patrik, Milovanovic Ivana The differences of Slovenian and Italian daily practices experienced in the first wave of covid-19 pandemic. BMC PUBLIC HEALTH. 2022;22(1):.

7155. Melin Anna, Heikura Ida, Tenforde Adam, Mountjoy Margo Energy Availability in Athletics: Health, Performance, and Physique. INTERNATIONAL JOURNAL OF SPORT NUTRITION AND EXERCISE METABOLISM. 2019;29(2, SI):152-164.

7156. Wesemann Dorette, Grunwald Martin Online counselling for persons with eating disorders and their relatives. Results of a survey on the effects and benefits of the consulting service on the ab-server. PSYCHOTHERAPEUT. 2008;53(4):284-289.

7157. Mitchell JE, Halmi K, Wilson GT, Agras WS, Kraemer H, Crow S A randomized secondary treatment study of women with bulimia nervosa who fail to respond to CBT. INTERNATIONAL JOURNAL OF EATING DISORDERS. 2002;32(3):271-281.

7158. Peters SMA, Tijsen MJH, Bindels RJM, VanOs CH, Wetzels JFM Rise in cytosolic Ca2+ and collapse of mitochondrial potential in anoxic, but not hypoxic, rat proximal tubules. JOURNAL OF THE AMERICAN SOCIETY OF NEPHROLOGY. 1996;7(11):2348-2356.

7159. Durkin SJ, Paxton SJ, Wertheim EH How do adolescent girls evaluate body dissatisfaction prevention messages?. JOURNAL OF ADOLESCENT HEALTH. 2005;37(5):381-390.

7160. Fombonne E Is bulimia nervosa increasing in frequency?. INTERNATIONAL JOURNAL OF EATING DISORDERS. 1996;19(3):287-296.

7161. Neumark-Sztainer D Can we simultaneously work toward the prevention of obesity and eating disorders in children and adolescents?. INTERNATIONAL JOURNAL OF EATING DISORDERS. 2005;38(3):220-227.

7162. Todd Alwyn, Street Steven, Ziviani Jenny, Byrne Nuala, Hills Andrew Overweight and Obese Adolescent Girls: The Importance of Promoting Sensible Eating and Activity Behaviors from the Start of the Adolescent Period. INTERNATIONAL JOURNAL OF ENVIRONMENTAL RESEARCH AND PUBLIC HEALTH. 2015;12(2):2306-2329.

7163. Bhupathiraju Shilpa, Sawicki Caleigh, Goon Shatabdi, Gujral Unjali, Hu Frank, Kandula Namrulha, Kanaya Alka A healthy plant-based diet is favorably associated with cardiometabolic risk factors among participants of South Asian ancestry. AMERICAN JOURNAL OF CLINICAL NUTRITION. 2022;116(4):1078-1090.

7164. Butwicka Agnieszka, Lichtenstein Paul, Landen Mikael, Nordenvall Anna, Nordenstrom Anna, Nordenskjold Agneta, Frisen Louise Hypospadias and increased risk for neurodevelopmental disorders. JOURNAL OF CHILD PSYCHOLOGY AND PSYCHIATRY. 2015;56(2):155-161.

7165. Strunk Sarah, Bussel Jamie The Healthy Kids, Healthy Communities National Program. JOURNAL OF PUBLIC HEALTH MANAGEMENT AND PRACTICE. 2015;21(3, SI):S1-S3.

7166. Hwang Seon Comparison of Clinical Manifestations and Treatment-Seeking Behavior in Younger and Older Patients with First-time Acute Coronary Syndrome. JOURNAL OF KOREAN ACADEMY OF NURSING. 2009;39(6):888-898.

7167. Udell Tuesday, Mehta Kaye When two sides go to war: newspaper reporting of `television food advertising restrictions' as a solution to childhood obesity. HEALTH RISK \& SOCIETY. 2008;10(6):535-548.

7168. Airhihenbuwa Collins, Tseng Tung-Sung, Sutton Victor, Price LeShawndra Global Perspectives on Improving Chronic Disease Prevention and Management in Diverse Settings. PREVENTING CHRONIC DISEASE. 2021;18():.

7169. Belanger Melissa, Dugas Camille, Perron Julie, Ruchat Stephanie-May, Weisnagel S., Marc Isabelle, Tchernof Andre, Robitaille Julie Association between lifestyle habits and adiposity values among children exposed and unexposed to gestational diabetes mellitus in utero. DIABETES \& METABOLIC SYNDROME-CLINICAL RESEARCH \& REVIEWS. 2019;13(5):2947-2952.

7170. Subramanian Ram, Chandel Navdeep, Budinger G., Schumacker Paul Hypoxic conformance of metabolism in primary rat hepatocytes: A model of hepatic hibernation. HEPATOLOGY. 2007;45(2):455-464.

7171. Kong Fanchang, Zhang Yan, Chen Hong The construct validity of the Restraint Scale among mainland Chinese women. EATING BEHAVIORS. 2013;14(3):356-360.

7172. Bulik CM, Sullivan PF, Carter FA, McIntosh VV, Joyce PR The role of exposure with response prevention in the cognitive-behavioural therapy for bulimia nervosa. PSYCHOLOGICAL MEDICINE. 1998;28(3):611-623.

7173. Tozzi Federica, Nicolaidou Iolie, Galani Anastasia, Antoniades Athos eHealth Interventions for Anxiety Management Targeting Young Children and Adolescents: Exploratory Review. JMIR PEDIATRICS AND PARENTING. 2018;1(1):.

7174. Aparicio E., Canals J., Arija V., De Henauw S., Michels N. The role of emotion regulation in childhood obesity: implications for prevention and treatment. NUTRITION RESEARCH REVIEWS. 2016;29(1):17-29.

7175. Carrard Isabelle, Della Torre Sophie A study protocol for a preliminary randomised controlled trial assessing the acceptability and effectiveness of two eating disorders prevention interventions in Switzerland: The HEIDI BP-HW project. PLOS ONE. 2021;16(11):.

7176. Fern R, Ransom BR, Waxman SG White matter stroke: Auto protective mechanisms with therapeutic implications. CEREBROVASCULAR DISEASES. 1996;6(2):59-65.

7177. Sun Chang-Qing, Chang Yu-Bo, Cui Ling-Ling, Chen Jia-Jun, Sun Nan, Zhang Wei-Jie, Jia Xiao-Can, Tian Yuan, Dai Li-Ping A Population-based Case-control Study on Risk Factors for Gastric Cardia Cancer in Rural Areas of Linzhou. ASIAN PACIFIC JOURNAL OF CANCER PREVENTION. 2013;14(5):2897-2901.

7178. Zwiep Karin, Hennekam Rick, Donders Timme, Helmond Niels, Lange Gert, Sangiorgi Francesca Marine productivity, water column processes and seafloor anoxia in relation to Nile discharge during sapropels S1 and S3. QUATERNARY SCIENCE REVIEWS. 2018;200():178-190.

7179. Iparraguirre Jose Socioeconomic determinants of risk of harmful alcohol drinking among people aged 50 or over in England. BMJ OPEN. 2015;5(7):.

7180. Koyama S, Jin YH, Akaike N ATP-sensitive and Ca2+-activated K+ channel activities in the rat locus coeruleus neurons during metabolic inhibition. BRAIN RESEARCH. 1999;828(1-2):189-192.

7181. Leitner Maya, Burstein Brett, Agostino Holly Prophylactic Phosphate Supplementation for the Inpatient Treatment of Restrictive Eating Disorders. JOURNAL OF ADOLESCENT HEALTH. 2016;58(6):616-620.

7182. Alvarez Pedro, Alvarado Carmen, Mathieu Florence, Jimenez Liliana, Fuente Monica Diet supplementation for 5 weeks with polyphenol-rich cereals improves several functions and the redox state of mouse leucocytes. EUROPEAN JOURNAL OF NUTRITION. 2006;45(8):428-438.

7183. Feng Yun, Lu Ying-Wei, Xu Pei-Han, Long Yuan, Wu Wei-Min, Li Wei, Wang Rui Caffeic acid phenethyl ester and its related compounds limit the functional alterations of the isolated mouse brain and liver mitochondria submitted to in vitro anoxia-reoxygenation: Relationship to their antioxidant activities. BIOCHIMICA ET BIOPHYSICA ACTA-GENERAL SUBJECTS. 2008;1780(4):659-672.

7184. MATTIA D, NAGAO T, ROGAWSKI MA, AVOLI M POTASSIUM CHANNEL ACTIVATORS COUNTERACT ANOXIC HYPEREXCITABILITY BUT NOT 4-AMINOPYRIDINE-INDUCED EPILEPTIFORM ACTIVITY IN THE RAT HIPPOCAMPAL SLICE. NEUROPHARMACOLOGY. 1994;33(12):1515-1522.

7185. Doley Joanna, McLean Sian, Griffiths Scott, Yager Zali Designing Body Image and Eating Disorder Prevention Programs for Boys and Men: Theoretical, Practical, and Logistical Considerations From Boys, Parents, Teachers, and Experts. PSYCHOLOGY OF MEN \& MASCULINITIES. 2021;22(1):124-134.

7186. Tjarnstrom J, Holmdahl L, Falk P, Falkenberg M, Arnell P, Risberg B Effects of hyperbaric oxygen on expression of fibrinolytic factors of human endothelium in a simulated ischaemia/reperfusion situation. SCANDINAVIAN JOURNAL OF CLINICAL \& LABORATORY INVESTIGATION. 2001;61(7):539-545.

7187. Sanders Amber, Stogner John, Miller Bryan PERCEPTION VS. REALITY: AN INVESTIGATION OF THE MISPERCEPTIONS CONCERNING THE EXTENT OF PEER NOVEL DRUG USE. JOURNAL OF DRUG EDUCATION. 2013;43(2):97-120.

7188. Keyes Katherine, Platt Jonathan, Kaufman Alan, McLaughlin Katie Association of Fluid Intelligence and Psychiatric Disorders in a Population-Representative Sample of US Adolescents. JAMA PSYCHIATRY. 2017;74(2):179-188.

7189. Nicholls Dasha, Hudson Lee, Mahomed Fermeda Managing anorexia nervosa. ARCHIVES OF DISEASE IN CHILDHOOD. 2011;96(10):977-982.

7190. Wijngaart L., Sieben A., Vlugt M., Leeuw F., Bredie S. A Nurse-Led Multidisciplinary Intervention to Improve Cardiovascular Disease Profile of Patients. WESTERN JOURNAL OF NURSING RESEARCH. 2015;37(6):705-723.

7191. Lovasi Gina, Johnson Norman, Altekruse Sean, Hirsch Jana, Moore Kari, Brown Janene, Rundle Andrew, Quinn James, Neckerman Kathryn, Siscovick David Healthy food retail availability and cardiovascular mortality in the United States: a cohort study. BMJ OPEN. 2021;11(7):.

7192. Li Yong-Xiang, Montanez Isabel, Liu Zhonghui, Ma Lifeng Astronomical constraints on global carbon-cycle perturbation during Oceanic Anoxic Event 2 (OAE2). EARTH AND PLANETARY SCIENCE LETTERS. 2017;462():35-46.

7193. Smith S. THE INFLUENCE OF STRESS AT PUBERTY ON MOOD AND LEARNING: ROLE OF THE alpha(4)beta delta GABA(A) RECEPTOR. NEUROSCIENCE. 2013;249():192-213.

7194. Thefeld W, Bergmann KE, Burger M, Holling H, Mensink GBM, Thamm M Assessment of health behaviour of parents and children in the national health interview and examination survey for children and adolescents. GESUNDHEITSWESEN. 2002;64(1):S36-S42.

7195. Vaezghasemi Masoud, Ohman Ann, Ng Nawi, Hakimi Mohammad, Eriksson Malin Concerned and Conscious, but DefenselessThe intersection of gender and generation in child malnutrition in Indonesia: a qualitative grounded theory study The intersection of gender and generation in child malnutrition in Indonesia: a qualitative grounded theory study. GLOBAL HEALTH ACTION. 2020;13(1):.

7196. Gammelmark Carina, Jensen Signe, Plessen Kerstin, Skadhede Soren, Larsen Janne, Munk-Jorgensen Povl Incidence of eating disorders in Danish psychiatric secondary healthcare 1970-2008. AUSTRALIAN AND NEW ZEALAND JOURNAL OF PSYCHIATRY. 2015;49(8):724-730.

7197. Caputa M, Rogalska J, Wentowska K, Nowakowska A Perinatal asphyxia, hyperthermia and hyperferremia as factors inducing behavioural disturbances in adulthood: A rat model. BEHAVIOURAL BRAIN RESEARCH. 2005;163(2):246-256.

7198. Black Katherine, Baker Dane, Sims Stacy Nutritional Needs of the Female Athlete: Risk and Prevention of Low Energy Availability. STRENGTH AND CONDITIONING JOURNAL. 2020;42(4):77-81.

7199. Lara Maria, Budde Claudio, Porrini Lucia, Borsani Julia, Murray Ricardo, Andreo Carlos, Drincovich Maria Peach (Prunus Persica) Fruit Response to Anoxia: Reversible Ripening Delay and Biochemical Changes. PLANT AND CELL PHYSIOLOGY. 2011;52(2, SI):392-403.

7200. GASBARRINI A, CARACENI P, FARGHALI H, VANTHIEL DH, BORLE AB EFFECTS OF HIGH AND LOW PH ON CA-I(2+) AND ON CELL INJURY EVOKED BY ANOXIA IN PERFUSED RAT HEPATOCYTES. BIOCHIMICA ET BIOPHYSICA ACTA-MOLECULAR CELL RESEARCH. 1994;1220(3):277-285.

7201. Dave Nandini Premedication and induction of anaesthesia in paediatric patients. INDIAN JOURNAL OF ANAESTHESIA. 2019;63(9):713-720.

7202. Huster D, Reichenbach A, Reichelt W The glutathione content of retinal Muller (glial) cells: effect of pathological conditions. NEUROCHEMISTRY INTERNATIONAL. 2000;36(4-5):461-469.

7203. Matamala-Gomez Marta, Brivio Eleonora, Chirico Alice, Malighetti Clelia, Realdon Olivia, Serino Silvia, Dakanalis Antonio, Corno Giulia, Polli Nicoletta, Cacciatore Chiara, Riva Giuseppe, Mantovani Fabrizia Assessing User Experience of a virtual reality training in patients with anorexia nervosa: insigths from a pilot study. ANNUAL REVIEW OF CYBERTHERAPY AND TELEMEDICINE. 2020;18():171-175.

7204. Porter Katherine, O'Neill Cara, Drake Elise, Andrews Sara, Delaney Kathleen, Parker Samantha, Escolar Maria, Montgomery Stacey, Moon William, Worrall Carolyn, Peay Holly Caregivers' assessment of meaningful and relevant clinical outcome assessments for Sanfilippo syndrome. JOURNAL OF PATIENT-REPORTED OUTCOMES. 2022;6(1):.

7205. Katzman DK Medical complications in adolescents with anorexia nervosa: A review of the literature. INTERNATIONAL JOURNAL OF EATING DISORDERS. 2005;37(S):S52-S59.

7206. Denham Bryan, Hawkins Katherine, Jones Karyn, Billings Andrew Anabolic-androgenic steroid use as a complicating factor in the female athlete triad: Behavioral implications for sport psychology. JOURNAL OF APPLIED SPORT PSYCHOLOGY. 2007;19(4):457-470.

7207. Sgarbi G., Barbato S., Costanzini A., Solaini G., Baracca A. The role of the ATPase inhibitor factor 1 (IF1) in cancer cells adaptation to hypoxia and anoxia. BIOCHIMICA ET BIOPHYSICA ACTA-BIOENERGETICS. 2018;1859(2):99-109.

7208. Hsu KS, Huang CC Characterization of the anoxia-induced long-term synaptic potentiation in area CA1 of the rat hippocampus. BRITISH JOURNAL OF PHARMACOLOGY. 1997;122(4):671-681.

7209. Kolbe L, Kann L, Patterson B, Wechsler H, Osorio J, Collins J Enabling the nation's schools to help prevent heart disease, stroke, cancer, COPD, diabetes, and other serious health problems. PUBLIC HEALTH REPORTS. 2004;119(3):286-302.

7210. Ondo William, Satija Pankaj Task-Specific Writing Tremor: Clinical Phenotypes, Progression, Treatment Outcomes, and Proposed Nomenclature. INTERNATIONAL

JOURNAL OF NEUROSCIENCE. 2012;122(2):88-91.

7211. Laz Tabassum, Rahman Mahbubur, Berenson Abbey Association of Frequent Use of Food Labels with Weight Loss Behaviors among Low-Income Reproductive-Age Women. JOURNAL OF THE AMERICAN COLLEGE OF NUTRITION. 2015;34(1):73-79.

7212. Eisenberg Marla, Franz Rachel, Berge Jerica, Loth Katie, Neumark-Sztainer Dianne Significant Others' Weight-Related Comments and Their Associations With Weight-Control Behavior, Muscle-Enhancing Behavior, and Emotional Well-Being. FAMILIES SYSTEMS \& HEALTH. 2017;35(4):474-485.

7213. Togha Mansoureh, Nematgorgani Shiva, Khorsha Faezeh, Mirzaei Khadijeh, Mirzababaei Atieh, Ghorbani Zeinab, Yekaninejad Mir, Okhovat Ali The Relationship Between Major Dietary Patterns and Disease Severity Among Migraine Patients. ARCHIVES OF NEUROSCIENCE. 2021;8(2):.

7214. Deneen Karen, Gold Mark, Liu Yijun Food Addiction and Cues in Prader-Willi Syndrome. JOURNAL OF ADDICTION MEDICINE. 2009;3(1):19-25.

7215. Tchanturia K, Happe F, Godley J, Treasure J, Bara-Carril N, Schmidt U `Theory of mind' in anorexia nervosa. EUROPEAN EATING DISORDERS REVIEW. 2004;12(6):361-366.

7216. Al-Hashel Jasem, Ismail Ismail Late-Onset Pompe Disease Presenting with Isolated Tongue Involvement. CASE REPORTS IN NEUROLOGY. 2022;14(1):98-103.

7217. Martinsen Marianne, Sherman Roberta, Thompson Ron, Sundgot-Borgen Jorunn Coaches' Knowledge and Management of Eating Disorders: A Randomized Controlled Trial. MEDICINE AND SCIENCE IN SPORTS AND EXERCISE. 2015;47(5):1070-1078.

7218. Aksoy Ayse, Koken Ozlem, Ceylan Ahmet, Dedeoglu Ozge KMT2B-Related Dystonia: Challenges in Diagnosis and Treatment. MOLECULAR SYNDROMOLOGY. 2021;():.

7219. Rohde Jeanett, Bohman Benjamin, Berglind Daniel, Hansson Lena, Frederiksen Peder, Mortensen Erik, Heitmann Berit, Rasmussen Finn Cross-sectional associations between maternal self-efficacy and dietary intake and physical activity in four-year-old children of first-time Swedish mothers. APPETITE. 2018;125():131-138.

7220. Couch Danielle, Thomas Samantha, Lewis Sophie, Blood R., Holland Kate, Komesaroff Paul Obese people's perceptions of the thin ideal. SOCIAL SCIENCE \& MEDICINE. 2016;148():60-70.

7221. Cacavas Katherine, Mavoa Helen, Kremer Peter, Malakellis Mary, Fotu Kalesita, Swinburn Boyd, Silva-Sanigorski Andrea Tongan Adolescents' Eating Patterns: Opportunities for Intervention. ASIA-PACIFIC JOURNAL OF PUBLIC HEALTH. 2011;23(1, SI):24-33.

7222. Vieira E., Kumar S., Narayan Y. Smoking, no-exercise, overweight and low back disorder in welders and nurses. INTERNATIONAL JOURNAL OF INDUSTRIAL ERGONOMICS. 2008;38(2):143-149.

7223. Safdie Margarita, Jennings-Aburto Nancy, Levesque Lucie, Janssen Ian, Campirano-Nunez Fabricio, Lopez-Olmedo Nancy, Aburto Tania, Rivera Juan Impact of a school-based intervention program on obesity risk factors in Mexican children. SALUD PUBLICA DE MEXICO. 2013;55(3):S374-S387.

7224. Bedard-Haughn A. Managing excess water in Canadian prairie soils: A review. CANADIAN JOURNAL OF SOIL SCIENCE. 2009;89(2):157-168.

7225. Linde Jennifer, Wall Melanie, Haines Jess, Neumark-Sztainer Dianne Predictors of initiation and persistence of unhealthy weight control behaviours in adolescents. INTERNATIONAL JOURNAL OF BEHAVIORAL NUTRITION AND PHYSICAL ACTIVITY. 2009;6():.

7226. Ma JLC The diagnostic and therapeutic uses of family conflicts in a Chinese context: the case of anorexia nervosa. JOURNAL OF FAMILY THERAPY. 2005;27(1):24-42.

7227. Freeman Becky, Kelly Bridget, Vandevijvere Stefanie, Baur Louise Young adults: beloved by food and drink marketers and forgotten by public health?. HEALTH PROMOTION INTERNATIONAL. 2016;31(4):954-961.

7228. Lewandowski LM, Gebing TA, Anthony JL, OBrien WH Meta-analysis of cognitive-behavioral treatment studies for bulimia. CLINICAL PSYCHOLOGY REVIEW. 1997;17(7):703-718.

7229. Trivedi Madhukar, Sreedharan Sapna, Nair Shana, Anees C., Unnikrishnan J., Sarma P., Radhakrishnan Ashalatha Central Disorders of Hypersomnolence in Children and Adults: A Comparative Study from South India. ANNALS OF INDIAN ACADEMY OF NEUROLOGY. 2019;22(4):442-446.

7230. Tabung Fred, Steck Susan, Burch James, Chen Chin-Fu, Zhang Hongmei, Hurley Thomas, Cavicchia Philip, Alexander Melannie, Shivappa Nitin, Creek Kim, Lloyd Stephen, Hebert James A Healthy Lifestyle Index Is Associated With Reduced Risk of Colorectal Adenomatous Polyps Among Non-Users of Non-Steroidal Anti-Inflammatory Drugs. JOURNAL OF PRIMARY PREVENTION. 2015;36(1):21-31.

7231. Frolich Jacob, Palm Camilla, Stoving Rene To the limit of extreme malnutrition. NUTRITION. 2016;32(1):146-148.

7232. Ceballos Natalie, Czyzewska Maria Body Image in Hispanic/Latino vs. European American Adolescents: Implications for Treatment and Prevention of Obesity in Underserved Populations. JOURNAL OF HEALTH CARE FOR THE POOR AND UNDERSERVED. 2010;21(3):823-838.

7233. Staiano Amanda, Harrington Deirdre, Broyles Stephanie, Gupta Alok, Katzmarzyk Peter Television, Adiposity, and Cardiometabolic Risk in Children and Adolescents. AMERICAN JOURNAL OF PREVENTIVE MEDICINE. 2013;44(1):40-47.

7234. Yamamoto Satsuki, Yamashita Shun, Kakiuchi Toshihiko, Kurogi Kazuya, Nishi Tomoyo, Tago Masaki, Yamashita Shu-ichi Late-Onset Ornithine Transcarbamylase Deficiency Complicated with Extremely High Serum Ammonia Level: Prompt Induction of Hemodialysis as the Key to Successful Treatment. AMERICAN JOURNAL OF CASE REPORTS. 2022;23():.

7235. Ozaki Kazumi, Tajika Eiichi Biogeochemical effects of atmospheric oxygen concentration, phosphorus weathering, and sea-level stand on oceanic redox chemistry: Implications for greenhouse climates. EARTH AND PLANETARY SCIENCE LETTERS. 2013;373():129-139.

7236. Perugi Giulio, Angst Jules, Azorin Jean-Michel, Bowden Charles, Vieta Eduard, Young Allan, Grp BRIDGE Is comorbid borderline personality disorder in patients with major depressive episode and bipolarity a developmental subtype? Findings from the international BRIDGE study. JOURNAL OF AFFECTIVE DISORDERS. 2013;144(1-2):72-78.

7237. Bond David, Wignall Paul, Grasby Stephen The Capitanian (Guadalupian, Middle Permian) mass extinction in NW Pangea (Borup Fiord, Arctic Canada): A global crisis driven by volcanism and anoxia. GEOLOGICAL SOCIETY OF AMERICA BULLETIN. 2020;132(5-6):931-942.

7238. Kalkman HO The role of the phosphatidylinositide 3-kinase-protein kinase B pathway in schizophrenia. PHARMACOLOGY \& THERAPEUTICS. 2006;110(1):117-134.

7239. Muller M, Brockhaus J, Ballanyi K ATP-independent anoxic activation of ATP-sensitive K+ channels in dorsal vagal neurons of juvenile mice in situ. NEUROSCIENCE. 2002;109(2):313-328.

7240. Luning S, Wendt J, Belka Z, Kaufmann B Temporal-spatial reconstruction of the early Frasnian (Late Devonian) anoxia in NW Africa: new field data from the Ahnet Basin (Algeria). SEDIMENTARY GEOLOGY. 2004;163(3-4):237-264.

7241. Paulke Alexander, Wunder Cora, Toennes Stefan Sleep self-intoxication and sleep driving as rare zolpidem-induced complex behaviour. INTERNATIONAL JOURNAL OF LEGAL MEDICINE. 2015;129(1):85-88.

7242. Choo Jina, Kim Hye-Jin, Park Sooyeon Neighborhood Environments: Links to Health Behaviors and Obesity Status in Vulnerable Children. WESTERN JOURNAL OF NURSING RESEARCH. 2017;39(8, SI):1169-1191.

7243. Torres-McGehee Toni, Monsma Eva, Dompier Thomas, Washburn Stefanie Eating Disorder Risk and the Role of Clothing in Collegiate Cheerleaders' Body Images. JOURNAL OF ATHLETIC TRAINING. 2012;47(5):541-548.

7244. Thompson Carly, Paprzycki Peter, Demers April, Tagoe Ishmael, Kruse-Diehr Aaron, Glassman Tavis ``IAmSizeSexy{''}: A health communication body image study. JOURNAL OF AMERICAN COLLEGE HEALTH. 2022;70(6):1867-1873.

7245. Robson E., Costa S., Hamer M., Johnson W. Life course factors associated with metabolically healthy obesity: a protocol for the systematic review of longitudinal studies. SYSTEMATIC REVIEWS. 2018;7():.

7246. Merlo Emanuele, Frisone Fabio, Settineri Salvatore, Mento Carmela Depression signs, Teasing and Low Self-esteem in Female Obese Adolescents: a clinical evaluation. MEDITERRANEAN JOURNAL OF CLINICAL PSYCHOLOGY. 2018;6(1):.

7247. Hagan Kelsey, Christensen Kara, Forbush Kelsie A preliminary systematic review and meta-analysis of randomized-controlled trials of cognitive remediation therapy for anorexia nervosa. EATING BEHAVIORS. 2020;37():.

7248. Ferguson CP, La Via MC, Crossan PJ, Kaye WH Are serotonin selective reuptake inhibitors effective in underweight anorexia nervosa?. INTERNATIONAL JOURNAL OF EATING DISORDERS. 1999;25(1):11-17.

7249. Jokela J, Taskinen J, Mutikainen P, Kopp K Virulence of parasites in hosts under environmental stress: experiments with anoxia and starvation. OIKOS. 2005;108(1):156-164.

7250. Ehrenberg Stephen, Svana Tore, Swart Peter Uranium depletion across the Pennian-Triassic boundary in Middle East carbonates: Signature of oceanic anoxia. AAPG BULLETIN. 2008;92(6):691-707.

7251. Mouro Lucas, Rakocinski Michal, Marynowski Leszek, Pisarzowska Agnieszka, Musabelliu Sabiela, Zaton Michal, Carvalho Marcelo, Fernandes Antonio, Waichel Breno Benthic anoxia, intermittent photic zone euxinia and elevated productivity during deposition of the Lower Permian, post-glacial fossiliferous black shales of the Parana Basin, Brazil. GLOBAL AND PLANETARY CHANGE. 2017;158():155-172.

7252. Rieder Jessica, Moon Jee-Young, Joels Joanna, Shankar Viswanathan, Meissner Paul, Johnson-Knox Elicia, Frohlich Bailey, Davies Shelby, Wylie-Rosett Judy Trends in health behavior and weight outcomes following enhanced afterschool programming participation. BMC PUBLIC HEALTH. 2021;21(1):.

7253. Ham John, Iorio Daniela, Sovinsky Michelle Caught in the Bulimic Trap? Persistence and State Dependence of Bulimia Among Young Women. JOURNAL OF HUMAN RESOURCES. 2013;48(3):736-767.

7254. Crnica Vanja, Bolic Bojana, Dzakula Aleksandar, Vitale Ksenija, Pjevac Neda Counseling on Cardiovascular Behavioral Risk Factors within the Healthcare System: the

CroHort Study. COLLEGIUM ANTROPOLOGICUM. 2012;36(1):251-255.

7255. Bridet Lionel, Beitia Martin Juan, Cabriada Nuno Jose Acute liver damage and anorexia nervosa: A case report. TURKISH JOURNAL OF GASTROENTEROLOGY. 2014;25(2):205-208.

7256. Eleftheriadis Theodoros, Pissas Georgios, Golfinopoulos Spyridon, Efthymiadi Maria, Liakopoulos Vassilios, Stefanidis Ioannis Inhibition of Malate Dehydrogenase-2 Protects Renal Tubular Epithelial Cells from Anoxia-Reoxygenation-Induced Death or Senescence. BIOMOLECULES. 2022;12(10):.

7257. Pashevin Denis, Nagibin Vasyl, Tumanovska Lesya, Moibenko Alex, Dosenko Victor Proteasome Inhibition Diminishes the Formation of Neutrophil Extracellular Traps and Prevents the Death of Cardiomyocytes in Coculture with Activated Neutrophils during Anoxia-Reoxygenation. PATHOBIOLOGY. 2015;82(6):290-298.

7258. Lee RB, Urban JPG Functional replacement of oxygen by other oxidants in articular cartilage. ARTHRITIS AND RHEUMATISM. 2002;46(12):3190-3200.

7259. Wilksch Simon, Wade Tracey Examination of the Sociocultural Attitudes Towards Appearance Questionnaire-3 in a Mixed-Gender Young-Adolescent Sample. PSYCHOLOGICAL ASSESSMENT. 2012;24(2):352-364.

7260. Radunz Marcela, Ali Kathina, Wade Tracey Pathways to improve early intervention for eating disorders: Findings from a systematic review and meta-analysis. INTERNATIONAL JOURNAL OF EATING DISORDERS. 2022;():.

7261. Plaza-Diaz Julio, Molina-Montes Esther, Jose Soto-Mendez Maria, Madrigal Casandra, Hernandez-Ruiz Angela, Valero Teresa, Lara Villoslada Federico, Leis Rosaura, Victoria Emilio, Manuel Moreno Jose, Ortega Rosa, Dolores Ruiz-Lopez Maria, Varela-Moreiras Gregorio, Gil Angel Clustering of Dietary Patterns and Lifestyles Among Spanish Children in the EsNuPI Study. NUTRIENTS. 2020;12(9):.

7262. Ranta Klaus, Vaananen Juha, Frojd Sari, Isomaa Rasmus, Kaltiala-Heino Riittakerttu, Marttunen Mauri Social phobia, depression and eating disorders during middle adolescence: longitudinal associations and treatment seeking. NORDIC JOURNAL OF PSYCHIATRY. 2017;71(8):605-613.

7263. Heim Christine, Mletzko Tanja, Purselle David, Musselman Dominique, Nemeroff Charles The dexamethasone/corticotropin-releasing factor test in men with major depression: Role of childhood trauma. BIOLOGICAL PSYCHIATRY. 2008;63(4):398-405.

7264. Wright Charles, Patel Madhukar, Gao Xiang, Witt Maxwell, Sally Mitchell, Groat Tahnee, Crutchfield Megan, Neidlinger Nikole, Pilot Markeith, Malinoski Darren, ODRC The Impact of Therapeutic Hypothermia Used to Treat Anoxic Brain Injury After Cardiopulmonary Resuscitation on Organ Donation Outcomes. THERAPEUTIC HYPOTHERMIA AND TEMPERATURE MANAGEMENT. 2019;9(4):258-264.

7265. Coovadia H., Jugnundan Y., Ramkissoon A. Adolescence: The age of Proteus. SAMJ SOUTH AFRICAN MEDICAL JOURNAL. 2016;106(7):29-31.

7266. Mendis Shanthi The Contribution of the Framingham Heart Study to the Prevention of Cardiovascular Disease: A Global Perspective. PROGRESS IN CARDIOVASCULAR DISEASES. 2010;53(1):10-14.

7267. Lienard Y, Vamecq J The self-addictive hypothesis of pathological eating habits. PRESSE MEDICALE. 2004;33(18, S):33-40.

7268. Ikonomidis JS, Shirai T, Weisel RD, Derylo B, Rao VV, Whiteside CI, Mickle DAG, Li RK Preconditioning cultured human pediatric myocytes requires adenosine and protein kinase C. AMERICAN JOURNAL OF PHYSIOLOGY-HEART AND CIRCULATORY PHYSIOLOGY. 1997;272(3):H1220-H1230.

7269. Choque Delgado Grethel, Cunha Tamashiro Wirla, Marostica Junior Mario, Moreno Yara, Pastore Glaucia The putative effects of prebiotics as immunomodulatory agents. FOOD RESEARCH INTERNATIONAL. 2011;44(10):3167-3173.

7270. Serra Giulia, Koukopoulos Athanasios, De Chiara Lavinia, Napoletano Flavia, Koukopoulos Alexia, Curto Martina, Manfredi Giovanni, Faedda Gianni, Girardi Paolo, Baldessarini Ross Features preceding diagnosis of bipolar versus major depressive disorders. JOURNAL OF AFFECTIVE DISORDERS. 2015;173():134-142.

7271. SORRELL BK AIRSPACE STRUCTURE AND MATHEMATICAL-MODELING OF OXYGEN DIFFUSION, AERATION AND ANOXIA IN ELEOCHARIS-SPHACELATA R-BR ROOTS. AUSTRALIAN JOURNAL OF MARINE AND FRESHWATER RESEARCH. 1994;45(8):1529-

1541.

7272. Jonikas Jessica, Cook Judith, Swarbrick Margaret, Nemec Patricia, Steigman Pamela, Boss Katherine, Brice George The impact of the COVID-19 pandemic on the mental health and daily life of adults with behavioral health disorders. TRANSLATIONAL BEHAVIORAL MEDICINE. 2021;11(5):1162-1171.

7273. Menshanov Petr, Bannova Anita, Dygalo Nikolay Anoxia ameliorates the dexamethasone-induced neurobehavioral alterations in the neonatal male rat pups. HORMONES AND BEHAVIOR. 2017;87():122-128.

7274. Harvitt DM, Bonanno JA Re-evaluation of the oxygen diffusion model for predicting minimum contact lens Dk/t values needed to avoid corneal anoxia. OPTOMETRY AND VISION SCIENCE. 1999;76(10):712-719.

7275. Boctor Dana, Jutteau Wiem, Fenton Tanis, Shourounis Jasmine, Galante Gary, Eicher Isabelle, Goulet Olivier, Lambe Cecile The prevalence of feeding difficulties and potential risk factors in pediatric intestinal failure: Time to consider promoting oral feeds?. CLINICAL NUTRITION. 2021;40(10):5399-5406.

7276. Jiotsa Barbara, Naccache Benjamin, Duval Melanie, Rocher Bruno, Grall-Bronnec Marie Social Media Use and Body Image Disorders: Association between Frequency of Comparing One's Own Physical Appearance to That of People Being Followed on Social Media and Body Dissatisfaction and Drive for Thinness. INTERNATIONAL JOURNAL OF ENVIRONMENTAL RESEARCH AND PUBLIC HEALTH. 2021;18(6):.

7277. Yap Marie, Whittle Sarah, Yuecel Murat, Sheeber Lisa, Pantelis Christos, Simmons Julian, Allen Nicholas Interaction of Parenting Experiences and Brain Structure in the Prediction of Depressive Symptoms in Adolescents. ARCHIVES OF GENERAL PSYCHIATRY. 2008;65(12):1377-1385.

7278. Record KE, Piascik P The Behavior modification experience: Application, accountability and coaching. AMERICAN JOURNAL OF PHARMACEUTICAL EDUCATION. 2002;66(4):450-457.

7279. Bulik Cynthia Are we really paddling as fast as we can? reflections on why eating disorders treatment and research always seem to be one step behind: Commentary on hay, mitchell, and stice \& becker: Prevention and treatment. INTERNATIONAL JOURNAL OF

EATING DISORDERS. 2013;46(5, SI):489-491.

7280. KLIBANSKI A, BILLER BMK, SCHOENFELD DA, HERZOG DB, SAXE VC THE EFFECTS OF ESTROGEN ADMINISTRATION ON TRABECULAR BONE LOSS IN YOUNG-WOMEN WITH ANOREXIA-NERVOSA. JOURNAL OF CLINICAL ENDOCRINOLOGY \& METABOLISM. 1995;80(3):898-904.

7281. Shelef Leah, Kaminsky Dan, Carmon Meytal, Kedem Ron, Bonne Omer, Mann J., Fruchter Eyal Risk factors for suicide attempt among Israeli Defense Forces soldiers: A retrospective case-control study. JOURNAL OF AFFECTIVE DISORDERS. 2015;186():232-240.

7282. Chavez-Ugalde Yanaina, Jago Russell, Toumpakari Zoi, Egan Matt, Cummins Steven, White Martin, Hulls Paige, De Vocht Frank Conceptualizing the commercial determinants of dietary behaviors associated with obesity: A systematic review using principles from critical interpretative synthesis. OBESITY SCIENCE \& PRACTICE. 2021;7(4):473-486.

7283. Adams Derek, Hurtgen Matthew, Sageman Bradley Volcanic triggering of a biogeochemical cascade during Oceanic Anoxic Event 2. NATURE GEOSCIENCE. 2010;3(3):201-204.

7284. Sagud Marina, Jaksic Nenad, Vuksan-Cusa Bjanka, Loncar Mladen, Loncar Ivana, Peles Alma, Milicic Davor, Jakovljevic Miro CARDIOVASCULAR DISEASE RISK FACTORS IN PATIENTS WITH POSTTRAUMATIC STRESS DISORDER (PTSD): A NARRATIVE REVIEW. PSYCHIATRIA DANUBINA. 2017;29(4):421-430.

7285. Salvo Deborah, Ranjit Nalini, Nielsen Aida, Akhavan Nika, Berg Alexandra Characterizing Micro-scale Disparities in Childhood Obesity: Examining the Influence of Multilevel Factors on 4-Year Changes in BMI, Healthy Eating, and Physical Activity, Among a Cohort of Children Residing in Disadvantaged Urban Enclaves. FRONTIERS IN PUBLIC HEALTH. 2019;7():.

7286. Funtikova Anna, Gomez Santiago, Fito Montserrat, Elosua Roberto, Bentiez-Arciniega Alejandra, Schroeder Helmut Effect of Energy Under-Reporting on Secular Trends of Dietary Patterns in a Mediterranean Population. PLOS ONE. 2015;10(5):.

7287. Sakuta Shizuka, Hashimoto Mamoru, Ikeda Manabu, Koyama Asuka, Takasaki Akihiro, Hotta Maki, Fukuhara Ryuji, Ishikawa Tomohisa, Yuki Seiji, Miyagawa Yusuke, Hidaka

Yosuke, Kaneda Keiichiro, Takebayashi Minoru Clinical features of behavioral symptoms in patients with semantic dementia: Does semantic dementia cause autistic traits?. PLOS ONE. 2021;16(2):.

7288. Lopez-Suarez Alejandro Burden of cancer attributable to obesity, type 2 diabetes and associated risk factors. METABOLISM-CLINICAL AND EXPERIMENTAL. 2019;92():136-146.

7289. Lopez-Cepero Andrea, Frisard Christine, Mabry Guadalupe, Spruill Tanya, Mattei Josiemer, Austin S., Lemon Stephenie, Rosal Milagros Association between poor sleep quality and emotional eating in US Latinx adults and the mediating role of negative emotions. BEHAVIORAL SLEEP MEDICINE. 2023;21(2):162-171.

7290. Gotler Maya, Oren Liat, Spierer Shoshanna, Yarom Noam, Ashkenazi Malka The impact of COVID-19 lockdown on maintenance of children's dental health A questionnaire-based survey. JOURNAL OF THE AMERICAN DENTAL ASSOCIATION. 2022;153(5):440-449.

7291. Imam Mustapha, Ismail Maznah The Impact of Traditional Food and Lifestyle Behavior on Epigenetic Burden of Chronic Disease. GLOBAL CHALLENGES. 2017;1(8):.

7292. Strasser Barbara Physical activity in obesity and metabolic syndrome. . 2013;1281():141-159.

7293. McDonald JT, Kennedy S Is migration to Canada associated with unhealthy weight gain? Overweight and obesity among Canada's immigrants. SOCIAL SCIENCE \& MEDICINE. 2005;61(12):2469-2481.

7294. Hirota Tomoya, Paksarian Diana, He Jian-Ping, Inoue Sachiko, Stapp Emma, Van Meter Anna, Merikangas Kathleen Associations of Social Capital with Mental Disorder Prevalence, Severity, and Comorbidity among US Adolescents. JOURNAL OF CLINICAL CHILD AND ADOLESCENT PSYCHOLOGY. 2022;51(6):970-981.

7295. Vangeepuram Nita, Carmona Jane, Arniella Guedy, Horowitz Carol, Burnet Deborah Use of Focus Groups to Inform a Youth Diabetes Prevention Model. JOURNAL OF NUTRITION EDUCATION AND BEHAVIOR. 2015;47(6):532-U68.

7296. Davico Chiara, Amianto Federico, Gaiotti Federica, Lasorsa Claudia, Peloso Anna, Bosia Chiara, Vesco Serena, Arletti Luca, Reale Laura, Vitiello Benedetto Clinical and

personality characteristics of adolescents with anorexia nervosa with or without non-suicidal self-injurious behavior. COMPREHENSIVE PSYCHIATRY. 2019;94():.

7297. Monnet Claude The Cenomanian-Turonian boundary mass extinction (Late Cretaceous): New insights from ammonoid biodiversity patterns of Europe, Tunisia and the Western Interior (North America). PALAEOGEOGRAPHY PALAEOCLIMATOLOGY PALAEOECOLOGY. 2009;282(1-4):88-104.

7298. Lu Zhaofeng, Miao Zhuang, Zhu Jian, Zhu Gangyi ETS-domain containing protein (Elk1) suppression protects cortical neurons against oxygen-glucose deprivation injury. EXPERIMENTAL CELL RESEARCH. 2018;371(1):42-49.

7299. Kroke Anja, Guenther Anke Deficiency in affluence - Epidemiological data on the relation between overweight and nutrient deficiency. ERNAHRUNGS UMSCHAU. 2006;53(12):480+.

7300. Pickering T, Clemow L, Davidson K, Gerin W Behavioral cardiology - Has its time finally arrived?. MOUNT SINAI JOURNAL OF MEDICINE. 2003;70(2):101-112.

7301. Zuckermann H, Harren FJM, Reuss J, Parker DH Dynamics of acetaldehyde production during anoxia and post-anoxia in red bell pepper studied by photoacoustic techniques. PLANT PHYSIOLOGY. 1997;113(3):925-932.

7302. Chen Hao, Nie Tong, Zhang Penglu, Ma Jun, Shan Anshan Hesperidin attenuates hepatic lipid accumulation in mice fed high-fat diet and oleic acid induced HepG2 via AMPK activation. LIFE SCIENCES. 2022;296():.

7303. Barclay Richard, McElwain Jennifer, Sageman Bradley Carbon sequestration activated by a volcanic CO2 pulse during Ocean Anoxic Event 2. NATURE GEOSCIENCE. 2010;3(3):205-208.

7304. Altintas Ebru, Ozlem Kutuk Meryem, Tufan Ali, Gozukara Bag Harika Alexithymia is not a good predictor of suicidal ideation in patients with social anxiety disorder. ANADOLU PSIKIYATRI DERGISI-ANATOLIAN JOURNAL OF PSYCHIATRY. 2018;19(6):577-585.

7305. Schoot G., Anthonio R., Jessurun G. Acute myocardial infarction in adolescents: reappraisal of underlying mechanisms. NETHERLANDS HEART JOURNAL. 2020;28(6):301-

308.

7306. Sun Aijun, Zou Yunzeng, Wang Ping, Xu Danling, Gong Hui, Wang Shijun, Qin Yingjie, Zhang Peng, Chen Yunqin, Harada Mutsuo, Isse Toyoshi, Kawamoto Toshihiro, Fan Huizhi, Yang Pengyuan, Akazawa Hiroshi, Nagai Toshio, Takano Hiroyuki, Ping Peipei, Komuro Issei, Ge Junbo Mitochondrial Aldehyde Dehydrogenase 2 Plays Protective Roles in Heart Failure After Myocardial Infarction via Suppression of the Cytosolic JNK/p53 Pathway in Mice. JOURNAL OF THE AMERICAN HEART ASSOCIATION. 2014;3(5):.

7307. Schnepper R., Blechert J., Stok F. Reception of health messages: effects of stigmatization and forcefulness. JOURNAL OF PUBLIC HEALTH. 2022;44(2):387-393.

7308. Garcia Maldonado Gerardo, Saldivar Gonzalez Atenogenes, Llanes Castillo Arturo, Sanchez Juarez Indira DSM-V. Lights and shadows of an unpublished manual. Challenges and expectations for the future. SALUD MENTAL. 2011;34(4):367-378.

7309. Eleftheriadis Theodoros, Pissas Georgios, Antoniadi Georgia, Liakopoulos Vassilios, Stefanidis Ioannis Cell Death Patterns Due to Warm Ischemia or Reperfusion in Renal Tubular Epithelial Cells Originating from Human, Mouse, or the Native Hibernator Hamster. BIOLOGY-BASEL. 2018;7(4):.

7310. Bransfield Robert Neuropsychiatric Lyme Borreliosis: An Overview with a Focus on a Specialty Psychiatrist's Clinical Practice. HEALTHCARE. 2018;6(3):.

7311. Hahn NI, Woolsey MM When food becomes a cry for help. JOURNAL OF THE AMERICAN DIETETIC ASSOCIATION. 1998;98(4):395+.

7312. Illig Romana, Klieser Eckhard, Kiesslich Tobias, Neureiter Daniel GERD-Barrett-Adenocarcinoma: Do We Have Suitable Prognostic and Predictive Molecular Markers?. GASTROENTEROLOGY RESEARCH AND PRACTICE. 2013;2013():.

7313. Nyberg Gisela, Norman Asa, Sundblom Elinor, Zeebari Zangin, Elinder Liselotte Effectiveness of a universal parental support programme to promote health behaviours and prevent overweight and obesity in 6-year-old children in disadvantaged areas, the Healthy School Start Study II, a cluster-randomised controlled trial. INTERNATIONAL JOURNAL OF BEHAVIORAL NUTRITION AND PHYSICAL ACTIVITY. 2016;13():.

7314. Tu Luyao, Gilli Adrian, Lotter Andre, Vogel Hendrik, Moyle Madeleine, Boyle John, Grosjean Martin The nexus among long-term changes in lake primary productivity, deep-water anoxia, and internal phosphorus loading, explored through analysis of a 15,000-year varved sediment record. GLOBAL AND PLANETARY CHANGE. 2021;207():.

7315. Mooney Jennifer, Dominic Anna, Lewis Alyona, Chafe Roger Young adults with eating disorders perspectives on educational resources to support the transition into adult medicine: a thematic analysis. JOURNAL OF EATING DISORDERS. 2023;11(1):.

7316. Sano Hiroyoshi, Wada Takuya, Naraoka Hiroshi Late Permian to Early Triassic environmental changes in the Panthalassic Ocean: Record from the seamount-associated deep-marine siliceous rocks, central Japan. PALAEOGEOGRAPHY PALAEOCLIMATOLOGY PALAEOECOLOGY. 2012;363():1-10.

7317. Trivedi Gunjan, Saboo Banshi, Singh Ram, Maheshwari Anuj, Sharma Kamal, Verma Narsingh Can decreased heart rate variability be a marker of autonomic dysfunction, metabolic syndrome and diabetes?. JOURNAL OF DIABETOLOGY. 2019;10(2):48-56.

7318. Lipson Sarah, Jones J., Taylor C., Wilfley Denise, Eichen Dawn, Fitzsimmons-Craft Ellen, Eisenberg Daniel Understanding and promoting treatment-seeking for eating disorders and body image concerns on college campuses through online screening, prevention and intervention. EATING BEHAVIORS. 2017;25(SI):68-73.

7319. Valenti Luca, Pedica Federica, Colombo Massimo Distinctive features of hepatocellular carcinoma in non-alcoholic fatty liver disease. DIGESTIVE AND LIVER DISEASE. 2022;54(2):154-163.

7320. Xiong Shuyun, Ding Meizhu, Li Ping, Pan Shufen, Li Guanlan, He Wenfang A health education model based on knowledge, attitude, and practice used as adjunct therapy for metabolic syndrome complicated with acute pancreatitis: A case report. JOURNAL OF INTERNATIONAL MEDICAL RESEARCH. 2020;48(5):.

7321. Fisher Caroline, Skocic Sonja, Rutherford Kathleen, Hetrick Sarah Family therapy approaches for anorexia nervosa. COCHRANE DATABASE OF SYSTEMATIC REVIEWS. 2018;(10):.

7322. Corcos M Sexuality and eating disorders: True-false sexual toxin and endogenous pleasure.. EVOLUTION PSYCHIATRIQUE. 1999;64(3):543-565.

7323. Hanna Kathleen, Hansen Jed Habits and Routines during Transitions among Emerging Adults with Type 1 Diabetes. WESTERN JOURNAL OF NURSING RESEARCH. 2020;42(6):446-453.

7324. Adorni Roberta, Zanatta Francesco, D'Addario Marco, Atella Francesca, Costantino Elena, Iaderosa Caterina, Petarle Giulia, Steca Patrizia Health-Related Lifestyle Profiles in Healthy Adults: Associations with Sociodemographic Indicators, Dispositional Optimism, and Sense of Coherence. NUTRIENTS. 2021;13(11):.

7325. Wischik Dora, Magny-Normilus Cherlie, Whittemore Robin Risk Factors of Obesity in Veterans of Recent Conflicts: Need for Diabetes Prevention. CURRENT DIABETES REPORTS. 2019;19(9):.

7326. Broda Krzysztof, Marynowski Leszek, Rakocinski Michal, Zaton Michal Coincidence of photic zone euxinia and impoverishment of arthropods in the aftermath of the Frasnian-Famennian biotic crisis. SCIENTIFIC REPORTS. 2019;9():.

7327. Morales Gladys, Ruiz Fabiola, Bes-Rastrollo Maira, Schifferli Ingrid, Munoz Andrea, Celedon Natalia Plant-based diets and cardio-metabolic risk factors. What does the evidence say?. REVISTA CHILENA DE NUTRICION. 2021;48(3):425-436.

7328. Wu Baojin, Luo Genming, Joachimski Michael, Wignall Paul, Lei Lidan, Huang Junhua, Lai Xulong Carbon and nitrogen isotope evidence for widespread presence of anoxic intermediate waters before and during the Permian-Triassic mass extinction. GEOLOGICAL SOCIETY OF AMERICA BULLETIN. 2022;134(5-6):1397-1413.

7329. Uzhova Irina, Fuster Valentin, Fernandez-Ortiz Antonio, Ordovas Jose, Sanz Javier, Fernandez-Friera Leticia, Lopez-Melgar Beatriz, Mendiguren Jose, Ibanez Borja, Bueno Hector, Penalvo Jose The Importance of Breakfast in Atherosclerosis Disease. JOURNAL OF THE AMERICAN COLLEGE OF CARDIOLOGY. 2017;70(15):1833-1842.

7330. Gerritsen Sarah, Wall Clare, Morton Susan Child-care nutrition environments: results from a survey of policy and practice in New Zealand early childhood education services. PUBLIC HEALTH NUTRITION. 2016;19(9):1531-1542.

7331. Hegazi Moustafa, Sehlo Mohammad, Al-Jasir Albandari, El-Deek Basem Development and cognitive functions in Saudi pre-school children with feeding problems without underlying medical disorders. JOURNAL OF PAEDIATRICS AND CHILD HEALTH. 2015;51(9):906-912.

7332. Fisher Caroline, Skocic Sonja, Rutherford Kathleen, Hetrick Sarah Family therapy approaches for anorexia nervosa. COCHRANE DATABASE OF SYSTEMATIC REVIEWS. 2019;(5):.

7333. Savona Natalie, Macauley Talia, Aguiar Anaely, Banik Anna, Boberska Monika, Brock Jessica, Brown Andrew, Hayward Joshua, Holbaek Helene, Rito Ana, Mendes Sofia, Vaaheim Fredrik, Houten Marloes, Veltkamp Gerlieke, Allender Steven, Rutter Harry, Knai Cecile Identifying the views of adolescents in five European countries on the drivers of obesity using group model building. EUROPEAN JOURNAL OF PUBLIC HEALTH. 2021;31(2):391-396.

7334. John Deborah, Winfield Tammy, Etuk Lena, Hystad Perry, Langellotto Gail, Manore Melinda, Gunter Kathy Community-Engaged Attribute Mapping: Exploring Resources and Readiness to Change the Rural Context for Obesity Prevention. PROGRESS IN COMMUNITY HEALTH PARTNERSHIPS-RESEARCH EDUCATION AND ACTION. 2017;11(2):183-196.

7335. Kincaid Halle, Nagpal Ravinder, Yadav Hariom Diet-Microbiota-Brain Axis in Alzheimer's Disease. ANNALS OF NUTRITION AND METABOLISM. 2021;77(2):21-27.

7336. Lu Yangbo, Hao Fang, Yan Detian, Lu Yongchao Volcanism-induced late Boda warming in the Late Ordovician: Evidence from the Upper Yangtze Platform, South China. PALAEOGEOGRAPHY PALAEOCLIMATOLOGY PALAEOECOLOGY. 2021;578():.

7337. Bennett Brooke, Wagner Allison, Latner Janet Body Checking and Body Image Avoidance as Partial Mediators of the Relationship between Internalized Weight Bias and Body Dissatisfaction. INTERNATIONAL JOURNAL OF ENVIRONMENTAL RESEARCH AND PUBLIC HEALTH. 2022;19(16):.

7338. Cabaco Antonio, Urchaga Jose, Guevara Raquel, Moral-Garcia Jose Psychopathological Risk Factors Associated with Body Image, Body Dissatisfaction and Weight-Loss Dieting in School-Age Adolescents. CHILDREN-BASEL. 2021;8(2):.

7339. Liebl A. Solutions for unsuccessful insulin pump therapy. DIABETOLOGE. 2009;5(4):275+.

7340. Abbas Muhammad, Boby Naila, Lee Eon-Bee, Hong Joo-Heon, Park Seung-Chun Anti-Obesity Effects of Ecklonia cava Extract in High-Fat Diet-Induced Obese Rats. ANTIOXIDANTS. 2022;11(2):.

7341. Farrington Jill, Faskunger Johan, Mackiewicz Karolina Evaluation of risk factor reduction in a European City Network. HEALTH PROMOTION INTERNATIONAL. 2015;30(1):i86-i98.

7342. Kim S, Popkin BM, Siega-Riz AM, Haines PS, Arab L A cross-national comparison of lifestyle between China and the United States, using a comprehensive cross-national measurement tool of the healthfulness of lifestyles: the Lifestyle Index. PREVENTIVE MEDICINE. 2004;38(2):160-171.

7343. Wade Tracey, Wilksch Simon, Paxton Susan, Byrne Susan, Austin S. Do universal media literacy programs have an effect on weight and shape concern by influencing media internalization?. INTERNATIONAL JOURNAL OF EATING DISORDERS. 2017;50(7):731-738.

7344. CERCO CF, COLE T 3-DIMENSIONAL EUTROPHICATION MODEL OF CHESAPEAKE BAY. JOURNAL OF ENVIRONMENTAL ENGINEERING-ASCE. 1993;119(6):1006-1025.

7345. Puhl Rebecca, Himmelstein Mary, Pearl Rebecca Weight Stigma as a Psychosocial Contributor to Obesity. AMERICAN PSYCHOLOGIST. 2020;75(2, SI):274-289.

7346. Smith Ariane, Petrie Trent Reducing the Risk of Disordered Eating Among Female Athletes: A Test of Alternative Interventions. JOURNAL OF APPLIED SPORT PSYCHOLOGY. 2008;20(4):392-407.

7347. Kemp David, Izumi Kentaro Multiproxy geochemical analysis of a Panthalassic margin record of the early Toarcian oceanic anoxic event (Toyora area, Japan). PALAEOGEOGRAPHY PALAEOCLIMATOLOGY PALAEOECOLOGY. 2014;414():332-341.

7348. Buwembo A., Long H., Walker C-D PARTICIPATION OF ENDOCANNABINOIDS IN RAPID SUPPRESSION OF STRESS RESPONSES BY GLUCOCORTICOIDS IN NEONATES.

NEUROSCIENCE. 2013;249():154-161.

7349. Pacanowski C., Diers L., Crosby R., Mackenzie M., Neumark-Sztainer D. Yoga's impact on risk and protective factors for disordered eating: a pilot prevention trial. EATING DISORDERS. 2020;28(4, SI):513-541.

7350. Nikniaz Leila, Farhangi Mahdieh, Tabrizi Jafar, Nikniaz Zeinab Association of major dietary patterns and different metabolic phenotypes: a population-based study of northwestern Iran. BMC ENDOCRINE DISORDERS. 2019;19(1):.

7351. Dean Elizabeth, Skinner Margot, Yu Homer, Jones Alice, Gosselink Rik, Soderlund Anne Why COVID-19 strengthens the case to scale up assault on non- communicable diseases: role of health professionals including physical therapists in mitigating pandemic waves. AIMS PUBLIC HEALTH. 2021;8(2):369-375.

7352. Coniglio Maria, Commodari Elena, Lagana Pasqualina Predictive factors for eating disorders in a cohort of Sicilian female students. PROGRESS IN NUTRITION. 2020;22(3):.

7353. Lenz Dunker Karin, Claudino Angelica Preventing weight-related problems among adolescent girls: A cluster randomized trial comparing the Brazilian `New Moves' program versus observation. OBESITY RESEARCH \& CLINICAL PRACTICE. 2018;12(1):102-115.

7354. Zhiratkova Zh., Petrova T., Leontyeva A. Promotion of a Healthy Lifestyle among Students (a Sociological Analysis). REGIONOLOGIYA-REGIONOLOGY RUSSIAN JOURNAL OF REGIONAL STUDIES. 2018;26(4):784-796.

7355. Shab-Bidar Sakineh, Golzarand Mahdieh, Hajimohammadi Mina, Mansouri Sara A posteriori dietary patterns and metabolic syndrome in adults: a systematic review and meta-analysis of observational studies. PUBLIC HEALTH NUTRITION. 2018;21(9):1681-1692.

7356. Svensson Ove, Hallberg Lillemor Hunting for health, well-being, and quality of life. INTERNATIONAL JOURNAL OF QUALITATIVE STUDIES ON HEALTH AND WELL-BEING. 2011;6(2):.

7357. Jonsson Ulf, Alaie Iman, Wilteus Anna, Zander Eric, Marschik Peter, Coghill David, Bolte Sven Annual Research Review: Quality of life and childhood mental and behavioural

disorders - a critical review of the research. JOURNAL OF CHILD PSYCHOLOGY AND PSYCHIATRY. 2017;58(4, SI):439-469.

7358. Michels Nathalie, Sioen Isabelle, Boone Liesbet, Clays Els, Vanaelst Barbara, Huybrechts Inge, De Henauw Stefaan Cross-Lagged Associations Between Children's Stress and Adiposity: The Children's Body Composition and Stress Study. PSYCHOSOMATIC MEDICINE. 2015;77(1):50-58.

7359. Beaulieu Dominique, Godin Gaston Staying in school for lunch instead of eating in fast-food restaurants: results of a quasi-experimental study among high-school students. PUBLIC HEALTH NUTRITION. 2012;15(12):2310-2319.

7360. Momen N., Plana-Ripoll O., Agerbo E., Benros M., Borglum A., Christensen M., Dalsgaard S., Degenhardt L., Jonge P., Debost J., Fenger-Gron M., Gunn J., Iburg K., Kessing L., Kessler R., Laursen T., Lim C., Mors O., Mortensen P., Musliner K., Nordentoft M., Pedersen C., Petersen L., Ribe A., Roest A., Saha S., Schork A., Scott K., Sievert C., Sorensen H., Stedman T., Vestergaard M., Vilhjalmsson B., Werge T., Weye N., Whiteford H., Prior A., McGrath J. Association between Mental Disorders and Subsequent Medical Conditions. NEW ENGLAND JOURNAL OF MEDICINE. 2020;382(18):1721-1731.

7361. Ollberding Nicholas, Nigg Claudio, Geller Karly, Horwath Caroline, Motl Rob, Dishman Rod Food Outlet Accessibility and Fruit and Vegetable Consumption. AMERICAN JOURNAL OF HEALTH PROMOTION. 2012;26(6):366-370.

7362. Katzman Debra, Spettigue Wendy, Agostino Holly, Couturier Jennifer, Dominic Anna, Findlay Sheri, Lam Pei-Yoong, Lane Margo, Maguire Bryan, Mawjee Karizma, Parikh Supriya, Steinegger Cathleen, Vyver Ellie, Norris Mark Incidence and Age- and Sex-Specific Differences in the Clinical Presentation of Children and Adolescents With Avoidant Restrictive Food Intake Disorder. JAMA PEDIATRICS. 2021;175(12):.

7363. Burton E., Wilder Tanganyika, Beech Bettina, Bruce Marino Caregiver feeding practices and weight status among African American adolescents: The Jackson Heart KIDS Pilot Study. EATING BEHAVIORS. 2017;27():33-38.

7364. Li Fuzhong, Harmer Peter, Cardinal Bradley, Bosworth Mark, Johnson-Shelton Deb Obesity and the Built Environment: Does the Density of Neighborhood Fast-Food Outlets Matter?. AMERICAN JOURNAL OF HEALTH PROMOTION. 2009;23(3):203-209.

7365. Campbell Kenisha, Peebles Rebecka Eating Disorders in Children and Adolescents: State of the Art Review. PEDIATRICS. 2014;134(3):582-592.

7366. Barria Francisco, Perez Francisca, Brahm M. Regulatory disorders in early childhood: identification, prevention and treatment guidelines. ANDES PEDIATRICA. 2022;93(2):159-166.

7367. Taliaferro Lindsay, Muehlenkamp Jennifer Risk Factors Associated With Self-injurious Behavior Among a National Sample of Undergraduate College Students. JOURNAL OF AMERICAN COLLEGE HEALTH. 2015;63(1):40-48.

7368. Howse Eloise, Hankey Catherine, Bauman Adrian, Freeman Becky Are young adults' discussions of public health nutrition policies associated with common food industry discourses? A qualitative pilot study. AUSTRALIAN AND NEW ZEALAND JOURNAL OF PUBLIC HEALTH. 2021;45(2):171-180.

7369. Spires Mark, Berggreen-Clausen Aravinda, Kasujja Francis, Delobelle Peter, Puoane Thandi, Sanders David, Daivadanam Meena Snapshots of Urban and Rural Food Environments: EPOCH-Based Mapping in a High-, Middle-, and Low-Income Country from a Non-Communicable Disease Perspective. NUTRIENTS. 2020;12(2):.

7370. Shaw RM, Rudy Y Electrophysiologic effects of acute myocardial ischemia: a theoretical study of altered cell excitability and action potential duration. CARDIOVASCULAR RESEARCH. 1997;35(2):256-272.

7371. El Ansari Walid, Ssewanyana Derrick, Stock Christiane Behavioral Health Risk Profiles of Undergraduate University Students in England, Wales, and Northern Ireland: A Cluster Analysis. FRONTIERS IN PUBLIC HEALTH. 2018;6():.

7372. TREASURE J, TODD G, BROLLY M, TILLER J, NEHMED A, DENMAN F A PILOT-STUDY OF A RANDOMIZED TRIAL OF COGNITIVE ANALYTICAL THERAPY VS EDUCATIONAL BEHAVIORAL-THERAPY FOR ADULT ANOREXIA-NERVOSA. BEHAVIOUR RESEARCH AND THERAPY. 1995;33(4):363-367.

7373. Holm-Denoma Jill, Joiner Thomas, Vohs Kathleen, Heatherton Todd The ``Freshman Fifteen{''} (the ``Freshman Five{''} actually): Predictors and possible explanations. HEALTH PSYCHOLOGY. 2008;27(1, S):S3-S9.

7374. Silva Thales, Matozinhos Fernanda, Almeida Gratao Lucia, Rocha Luana, Vilela Luisa, Oliveira Tatiana, Cunha Cristiane, Mendes Larissa Coexistence of risk factors for cardiovascular diseases among Brazilian adolescents: Individual characteristics and school environment. PLOS ONE. 2021;16(7):.

7375. Wang WG, Fung ML, Darnall RA, StJohn WM Characterizations and comparisons of eupnoea and gasping in neonatal rats. JOURNAL OF PHYSIOLOGY-LONDON. 1996;490(1):277-292.

7376. Jessri Mahsa, Rashidkhani Bahram Dietary Patterns and Risk of Gallbladder Disease: A Hospital-based Case-Control Study in Adult Women. JOURNAL OF HEALTH POPULATION AND NUTRITION. 2015;33(1):39-49.

7377. Thorndike Anne, Riis Jason, Sonnenberg Lillian, Levy Douglas Traffic-Light Labels and Choice Architecture Promoting Healthy Food Choices. AMERICAN JOURNAL OF PREVENTIVE MEDICINE. 2014;46(2):143-149.

7378. Cardarelli Kathryn, DeWitt Emily, Gillespie Rachel, Norman-Burgdolf Heather, Jones Natalie, Mullins Janet ``We're, Like, the Most Unhealthy People in the Country{''}: Using an Equity Lens to Reduce Barriers to Healthy Food Access in Rural Appalachia. PREVENTING CHRONIC DISEASE. 2020;17():.

7379. Nurul-Fadhilah Abdullah, Teo Pey, Huybrechts Inge, Foo Leng Infrequent Breakfast Consumption Is Associated with Higher Body Adiposity and Abdominal Obesity in Malaysian School-Aged Adolescents. PLOS ONE. 2013;8(3):.

7380. Persson Johanna, Bohman Benjamin, Tynelius Per, Rasmussen Finn, Ghaderi Ata Prevention of Childhood Obesity in Child Health Services: Follow-Up of the PRIMROSE Trial. CHILDHOOD OBESITY. 2018;14(2):99-105.

7381. Gill Timothy, Baur Louise, Bauman Adrian, Steinbeck Kate, Storlien Leonard, Singh Maria, Brand-Miller Jennie, Colagiuri Stephen, Caterson Ian Childhood obesity in Australia remains a widespread health concern that warrants population-wide prevention programs. MEDICAL JOURNAL OF AUSTRALIA. 2009;190(3):146-148.

7382. Burlew Larry, Shurts W. Men and Body Image: Current Issues and Counseling Implications. JOURNAL OF COUNSELING AND DEVELOPMENT. 2013;91(4, SI):428-435.

7383. Gao Zhibing, Wu Fei, Lv Gaoyaxin, Zhuang Xiangling, Ma Guojie Development and Validity of a General Nutrition Knowledge Questionnaire (GNKQ) for Chinese Adults. NUTRIENTS. 2021;13(12):.

7384. Shi Qiang, Feng Ya-Ni, Fang Jun, Xu Ke Pretreatment with Glutamine Attenuates Anoxia/Reoxygenation Injury of Human Proximal Renal Tubular Epithelial Cells via Induction of Heme Oxygenase-1. PHARMACOLOGY. 2009;84(1):1-8.

7385. Golan Moria, Tzabari Dana, Mozeikov Maya The Impact of Delivering School-Based Wellness Programs for Emerging Adult Facilitators-A Quasi-Controlled Clinical Trial. INTERNATIONAL JOURNAL OF ENVIRONMENTAL RESEARCH AND PUBLIC HEALTH. 2022;19(7):.

7386. Tareen Ruqiya, Tareen Kinza Psychosocial aspects of diabetes management: dilemma of diabetes distress. TRANSLATIONAL PEDIATRICS. 2017;6(4):383-396.

7387. Luten Karla, Dijkstra Arie, Winter Andrea, Reijneveld Sijmen Developing a community-based intervention for Dutch older adults in a socioeconomically disadvantaged community. HEALTH PROMOTION INTERNATIONAL. 2019;34(3):567-580.

7388. Laiola Manolo, De Filippis Francesca, Vitaglione Paola, Ercolini Danilo A Mediterranean Diet Intervention Reduces the Levels of Salivary Periodontopathogenic Bacteria in Overweight and Obese Subjects. APPLIED AND ENVIRONMENTAL MICROBIOLOGY. 2020;86(12):.

7389. Kaestner D., Loewe B., Weigel A., Osen B., Voderholzer U., Gumz A. Factors influencing the length of hospital stay of patients with anorexia nervosa - results of a prospective multi-center study. BMC HEALTH SERVICES RESEARCH. 2018;18():.

7390. Mander Johannes, Teufel Martin, Keifenheim Katharina, Zipfel Stephan, Giel Katrin Stages of change, treatment outcome and therapeutic alliance in adult inpatients with chronic anorexia nervosa. BMC PSYCHIATRY. 2013;13():.

7391. Eng W, Heimberg RG, Coles ME, Schneier FR, Liebowitz MR An empirical approach to subtype identification in individuals with social phobia. PSYCHOLOGICAL MEDICINE. 2000;30(6):1345-1357.

7392. Catina A, Boyadjieva S, Bergner M Social context, gender identity and eating disorders in western and eastern Europe: Preliminary results of a comparative study. EUROPEAN EATING DISORDERS REVIEW. 1996;4(2):100-106.

7393. Ghafouri Khloud, Qadhi Alaa, Ghaith Mazen, Azhar Wedad, Azzeh Firas, Habibullah Mahmoud Eating disorders amongst adolescents in Makkah: Effects of stress and smoking. MEDICAL SCIENCE. 2021;25(110):767-775.

7394. Pogosova N., Yufereva Yu, Yusubova A., Ausheva A., Starodubova A., Allenov A., Karpova A., Eganyan R., Vygodin V. THE EFFECTIVENESS OF PREVENTIVE COUNSELING WITH THE USE OF REMOTE TECHNOLOGIES ON MEDICAL AWARENESS OF CARDIOVASCULAR RISK FACTORS IN PATIENTS WITH HIGH AND VERY HIGH CARDIOVASCULAR RISK. KARDIOLOGIYA. 2019;59(10, S):31-40.

7395. McCabe Marita, Ricciardelli Lina, Holt Kate Are there different sociocultural influences on body image and body change strategies for overweight adolescent boys and girls?. EATING BEHAVIORS. 2010;11(3):156-163.

7396. Skiba Meghan, Lopez-Pentecost Melissa, Werts Samantha, Ingram Maia, Vogel Rosi, Enriquez Tatiana, Garcia Lizzie, Thomson Cynthia Health Promotion Among Mexican-Origin Survivors of Breast Cancer and Caregivers Living in the United States-Mexico Border Region: Qualitative Analysis From the Vida Plena Study. JMIR CANCER. 2022;8(1):.

7397. Dunbar Michael, Tucker Joan, Ewing Brett, Pedersen Eric, Miles Jeremy, Shih Regina, D'Amico Elizabeth Frequency of E-cigarette Use, Health Status, and Risk and Protective Health Behaviors in Adolescents. JOURNAL OF ADDICTION MEDICINE. 2017;11(1):55-62.

7398. Wan Zhenzhen, Shan Zhilei, Geng Tingting, Lu Qi, Li Lin, Yin Jiawei, Liu Liegang, Pan An, Liu Gang Associations of Moderate Low-Carbohydrate Diets With Mortality Among Patients With Type 2 Diabetes: A Prospective Cohort Study. JOURNAL OF CLINICAL ENDOCRINOLOGY \& METABOLISM. 2022;107(7):E2702-E2709.

7399. Isralowitz Richard, Romem Porat Shai-li, Zolotov Yuval, Yehudai Mor, Dagan Adi, Reznik Alexander Gaming Disorder and Psycho-Emotional Wellbeing among Male University Students and Other Young Adults in Israel. INTERNATIONAL JOURNAL OF ENVIRONMENTAL RESEARCH AND PUBLIC HEALTH. 2022;19(23):.

7400. Lotfi Mostafa, Nouri Mehran, Jalil Abduladheem, Rezaianzadeh Abbas, Babajafari Siavash, Johari Masoumeh, Faghih Shiva Plant-based diets could ameliorate the risk factors of cardiovascular diseases in adults with chronic diseases. FOOD SCIENCE \& NUTRITION. 2023;11(3):1297-1308.

7401. LEONARD BE THE COMPARATIVE PHARMACOLOGY OF NEW ANTIDEPRESSANTS. JOURNAL OF CLINICAL PSYCHIATRY. 1993;54(S):3-15.

7402. Fonville Leon, Lao-Kaim Nick, Giampietro Vincent, Eynde Frederique, Davies Helen, Lounes Naima, Andrew Christopher, Dalton Jeffrey, Simmons Andrew, Williams Steven, Baron-Cohen Simon, Tchanturia Kate Evaluation of Enhanced Attention to Local Detail in Anorexia Nervosa Using the Embedded Figures Test; an fMRI Study. PLOS ONE. 2013;8(5):.

7403. Pascoe Michaela, Bailey Alan, Craike Melinda, Carter Tim, Patten Rhiannon, Stepto Nigel, Parker Alexandra Exercise interventions for mental disorders in young people: a scoping review. BMJ OPEN SPORT \& EXERCISE MEDICINE. 2020;6(1):.

7404. McLean Joanne, Lobetti Remo, Mooney Carmel, Thompson Peter, Schoeman Johan Prevalence of and risk factors for feline hyperthyroidism in South Africa. JOURNAL OF FELINE MEDICINE AND SURGERY. 2017;19(10):1103-1109.

7405. Chan Kin, Goldmark Jesse, Roth Mark Suspended Animation Extends Survival Limits of Caenorhabditis elegans and Saccharomyces cerevisiae at Low Temperature. MOLECULAR BIOLOGY OF THE CELL. 2010;21(13):2161-2171.

7406. Wu LY, Ding AS, Zhao T, Ma ZM, Wang FZ, Fan M Involvement of increased stability of mitochondrial membrane potential and overexpression of Bcl-2 in enhanced anoxic tolerance induced by hypoxic preconditioning in cultured hypothalamic neurons. BRAIN RESEARCH. 2004;999(2):149-154.

7407. Das Manasi, Webster Nicholas Obesity, cancer risk, and time-restricted eating. CANCER AND METASTASIS REVIEWS. 2022;41(3, SI):697-717.

7408. vantHof S, Nicolson M The rise and fall of a fact: The increase in anorexia nervosa. SOCIOLOGY OF HEALTH \& ILLNESS. 1996;18(5):581-608.

7409. Zhu Anna, Yuan Changzheng, Pretty Jules, Ji John Plant-based dietary patterns and cognitive function: A prospective cohort analysis of elderly individuals in China (2008-2018). BRAIN AND BEHAVIOR. 2022;12(8):.

7410. Mallaiah Janhavi, De Leon Reynaldo, Williams Olajide, Allegrante John Cardiovascular Disease and Stroke-Focused Competency Assessment Tools for Community Health Workers in the United States: A Scoping Review. HEALTH PROMOTION PRACTICE. 2022;():.

7411. Amini Roya, Rajabi Maryam, Azami Hiva, Soltanian Alireza The effect of self-management intervention program on the lifestyle of postmyocardial infarction patients. JOURNAL OF EDUCATION AND HEALTH PROMOTION. 2021;10(1):.

7412. Isono Naofumi, Santou Keiko, Ueda Norihide, Endou Takayuki Acute subdural haematoma accompanied by anorexia nervosa. BMJ CASE REPORTS. 2019;12(9):.

7413. De Lepeleere Sara, Verloigne Maite, Brown Helen, Cardon Greet, De Bourdeaudhuij Ilse Using the Intervention Mapping Protocol to develop an online video intervention for parents to prevent childhood obesity: Movie Models. GLOBAL HEALTH PROMOTION. 2018;25(2):56-66.

7414. Ashdown Helen, Joita Silvia, Luheshi Giamal, Boksa Patricia Acute Brain Cytokine Responses After Global Birth Hypoxia in the Rat. JOURNAL OF NEUROSCIENCE RESEARCH. 2008;86(15):3401-3409.

7415. Zhang Yuehong, Jin De, An Xuedong, Duan Liyun, Duan Yingying, Lian Fengmei Lychee Seed as a Potential Hypoglycemic Agent, and Exploration of its Underlying Mechanisms. FRONTIERS IN PHARMACOLOGY. 2021;12():.

7416. Opie R., Itsiopoulos C., Parletta N., Sanchez-Villegas A., Akbaraly T., Ruusunen A., Jacka F. Dietary recommendations for the prevention of depression. NUTRITIONAL NEUROSCIENCE. 2017;20(3):161-171.

7417. Champion Katrina, Newton Nicola, Spring Bonnie, Wafford Q., Parmenter Belinda, Teesson Maree A systematic review of school-based eHealth interventions targeting alcohol use, smoking, physical inactivity, diet, sedentary behaviour and sleep among adolescents: a review protocol. SYSTEMATIC REVIEWS. 2017;6():.

7418. Kashif Rao, D'Cunha Nathan, Mellor Duane, Alexopoulos Natalie, Sergi Domenico, Naumovski Nenad Prickly Pear Cacti (Opuntia spp.) Cladodes as a Functional Ingredient for Hyperglycemia Management: A Brief Narrative Review. MEDICINA-LITHUANIA. 2022;58(2):.

7419. Upton Penney, Taylor Charlotte, Upton Dominic The effects of the Food Dudes Programme on children's intake of unhealthy foods at lunchtime. PERSPECTIVES IN PUBLIC HEALTH. 2015;135(3):152-159.

7420. Pearson Natalie, Biddle Stuart Sedentary Behavior and Dietary Intake in Children, Adolescents, and Adults A Systematic Review. AMERICAN JOURNAL OF PREVENTIVE MEDICINE. 2011;41(2):178-188.

7421. Kip Elodie, Parr-Brownlie Louise Healthy lifestyles and wellbeing reduce neuroinflammation and prevent neurodegenerative and psychiatric disorders. FRONTIERS IN NEUROSCIENCE. 2023;17():.

7422. HAGERMAN L, VISMANN B ANAEROBIC METABOLISM IN THE SHRIMP CRANGON-CRANGON EXPOSED TO HYPOXIA, ANOXIA AND HYDROGEN-SULFIDE. MARINE BIOLOGY. 1995;123(2):235-240.

7423. Dachs GU, Coralli C, Hart SL, Tozer GM Gene delivery to hypoxic cells in vitro. BRITISH JOURNAL OF CANCER. 2000;83(5):662-667.

7424. Ozawa Y, Shimizu T, Shishiba Y Elevation of serum aminotransferase as a sign of multiorgan-disorders in severely emaciated anorexia nervosa. INTERNAL MEDICINE. 1998;37(1):32-39.

7425. Muggeridge David, Goszcz Katarzyna, Treweeke Andrew, Adamson Janet, Hickson Kirsty, Crabtree Daniel, Megson Ian Co-ingestion of Antioxidant Drinks With an Unhealthy Challenge Meal Fails to Prevent Post-prandial Endothelial Dysfunction: An Open-Label, Crossover Study in Older Overweight Volunteers. FRONTIERS IN PHYSIOLOGY. 2019;10():.

7426. Geissler Cathleen, Krause Christin, Neumann Anne-Marie, Britsemmer Jan, Taege Natalie, Grohs Martina, Kaehler Meike, Cascorbi Ingolf, Lewis Alfor, Seeley Randy, Oster Henrik, Kirchner Henriette Dietary induction of obesity and insulin resistance is associated with changes in Fgf21 DNA methylation in liver of mice. JOURNAL OF NUTRITIONAL

BIOCHEMISTRY. 2022;100():.

7427. Perez-Mitre GG Body image disturbances in a Mexican sample of preadolescent students. REVISTA MEXICANA DE PSICOLOGIA. 1997;14(1):31-40.

7428. Calu Donna, Chen Yu-Wei, Kawa Alex, Nair Sunila, Shaham Yavin The use of the reinstatement model to study relapse to palatable food seeking during dieting. NEUROPHARMACOLOGY. 2014;76(B, SI):395-406.

7429. Kim Youjeong, Sundar S. Visualizing ideal self vs. actual self through avatars: Impact on preventive health outcomes. COMPUTERS IN HUMAN BEHAVIOR. 2012;28(4):1356-1364.

7430. Kotwas Artur, Karakiewicz-Krawczyk Katarzyna, Zabielska Paulina, Jurczak Anna, Bazydlo Marta, Karakiewicz Beata The incidence of eating disorders among upper secondary school female students. PSYCHIATRIA POLSKA. 2020;54(2):253-263.

7431. Gete Dereje, Waller Michael, Mishra Gita Pre-pregnancy diet quality and its association with offspring behavioral problems. EUROPEAN JOURNAL OF NUTRITION. 2021;60(1):503-515.

7432. Ling Anna, Eberli Gregor, Swart Peter, Reolid Jesus, Stainbank Stephanie, Ruggeberg Andres, Betzler Christian Middle Miocene platform drowning in the Maldives associated with monsoon-related intensification of currents. PALAEOGEOGRAPHY PALAEOCLIMATOLOGY PALAEOECOLOGY. 2021;567():.

7433. Ezzeddin Neda, Zavoshy Rosa, Noroozi Mostafa, Jahanihashemi Hassan, Riseh Shaghayegh Prevalence and risk factors for pica during pregnancy in Tehran, Iran. EATING AND WEIGHT DISORDERS-STUDIES ON ANOREXIA BULIMIA AND OBESITY. 2015;20(4):457-463.

7434. Witkos Joanna, Blazejewski Grzegorz, Gierach Marcin The Low Energy Availability in Females Questionnaire (LEAF-Q) as a Useful Tool to Identify Female Triathletes at Risk for Menstrual Disorders Related to Low Energy Availability. NUTRIENTS. 2023;15(3):.

7435. MORTOLA JF, LAUGHLIN GA, YEN SSC MELATONIN RHYTHMS IN WOMEN WITH ANOREXIA-NERVOSA AND BULIMIA-NERVOSA. JOURNAL OF CLINICAL ENDOCRINOLOGY

\& METABOLISM. 1993;77(6):1540-1544.

7436. Kurihara Yosuke, Kaburagi Takashi, Kumagai Satoshi, Matsumoto Toshiyuki Development of Swallowing-Movement-Sensing Device and Swallowing-State-Estimation System. IEEE SENSORS JOURNAL. 2019;19(9):3532-3542.

7437. Danca Cristina, Costea Claudia, Costan Victor, Turliuc Mihaela, Sava Anca, Turliuc Serban, Cucu Andrei, Dragomir Raluca, Scripcariu Ioana, Dumitrescu Nicoleta, Carauleanu Alexandru Gut Microbiota and Serotonin - Biochemical Pathways in Age-Related Macular Disease. REVISTA DE CHIMIE. 2018;69(10):2823-2825.

7438. Koskina A., Schmidt U. Who am I without anorexia? Identity exploration in the treatment of early stage anorexia nervosa during emerging adulthood: a case study. COGNITIVE BEHAVIOUR THERAPIST. 2019;12():.

7439. Pelletier Jennifer, Lytle Leslie, Laska Melissa Stress, Health Risk Behaviors, and Weight Status Among Community College Students. HEALTH EDUCATION \& BEHAVIOR. 2016;43(2):139-144.

7440. Wurtman RJ, Wurtman JJ Serotoninergic mechanisms and obesity. JOURNAL OF NUTRITIONAL BIOCHEMISTRY. 1998;9(9):511-515.

7441. Sanuade Olutobi, Dodoo Francis, Koram Kwadwo, Aikins Ama Explanatory models of stroke in Ghana: perspectives of stroke survivors and their caregivers. ETHNICITY \& HEALTH. 2021;26(5):697-719.

7442. Chen Baihe, Lu Di, Fu Yujuan, Zhang Jingwen, Huang Xiaobo, Cao Shiping, Xu Dingli, Bin Jianping, Kitakaze Masafumi, Huang Qiaobing, Liao Yulin Olmesartan prevents cardiac rupture in mice with myocardial infarction by modulating growth differentiation factor 15 and p53. BRITISH JOURNAL OF PHARMACOLOGY. 2014;171(15):3741-3753.

7443. Haqq Andrea, Kebbe Maryam, Tan Qiming, Manco Melania, Salas Ximena The Complexity and Stigma of Pediatric Obesity. CHILDHOOD OBESITY. 2021;17(4):229-240.

7444. Rufini Stefano, Grossi Daniele, Luly Paolo, Tancredi Virginia, Frank Claudio, D'Arcangelo Giovanna Cholesterol depletion inhibits electrophysiological changes induced

by anoxia in CA1 region of rat hippocampal slices. BRAIN RESEARCH. 2009;1298():178-185.

7445. Ciarcia Carla, Falsaperla Paolo, Giacobbe Andrea, Mulone Giuseppe A mathematical model of anorexia and bulimia. MATHEMATICAL METHODS IN THE APPLIED SCIENCES. 2015;38(14):2937-2952.

7446. Gratacos Monica, Escaramis Georgia, Bustamante Mariona, Saus Ester, Agueera Zaida, Bayes Monica, Cellini Elena, Cid Rafael, Fernandez-Aranda Fernando, Forcano Laura, Gonzalez Juan, Gorwood Philip, Hebebrand Johannes, Hinney Anke, Mercader Josep, Nacmias Benedetta, Ramoz Nicolas, Ribases Marta, Ricca Valdo, Romo Lucia, Sorbi Sandro, Versini Audrey, Estivill Xavier Role of the neurotrophin network in eating disorders' subphenotypes: Body mass index and age at onset of the disease. JOURNAL OF PSYCHIATRIC RESEARCH. 2010;44(13):834-840.

7447. Beutel MW Hypolimnetic anoxia and sediment oxygen demand in California drinking water reservoirs. LAKE AND RESERVOIR MANAGEMENT. 2003;19(3):208-221.

7448. Kakoschke Naomi, Cox David, Ryan Jillian, Gwilt Ian, Davis Aaron, Jansons Paul, Courten Barbora, Brinkworth Grant Disrupting future discounting: a commentary on an underutilised psychological approach for improving adherence to diet and physical activity interventions. PUBLIC HEALTH NUTRITION. 2023;():.

7449. Sucato Vincenzo, Coppola Giuseppe, Manno Girolamo, Amata Francesco, Evola Salvatore, Novo Giuseppina, Galassi Alfredo, Corrado Egle Cardiovascular diseases in patients from South Asia and resident in Italy: risk factors, pathogenesis and drug treatment. GIORNALE ITALIANO DI CARDIOLOGIA. 2021;22(3):203-211.

7450. Lafaye AL, Desachy A, Roustan J, Lagrange P, Vignon P, Francois B Brugada syndrome: a rare cause of sudden death in the young adult with a ``normal{''} heart. PRESSE MEDICALE. 1999;28(10):527-530.

7451. Keski-Rahkonen Anna, Raevuori Anu, Bulik Cynthia, Hoek Hans, Rissanen Aila, Kaprio Jaakko Factors Associated with Recovery from Anorexia Nervosa: A Population-Based Study. INTERNATIONAL JOURNAL OF EATING DISORDERS. 2014;47(2):117-123.

7452. Vanoli M., Grassi M., Rizzolo A. Ripening behavior and physiological disorders of `Abate Fetel' pears treated at harvest with 1-MCP and stored at different temperatures and

atmospheres. POSTHARVEST BIOLOGY AND TECHNOLOGY. 2016;111():274-285.

7453. Hartley P Does health education promote eating disorders?. EUROPEAN EATING DISORDERS REVIEW. 1996;4(1):3-11.

7454. Walsh Brandon, Stary Creed, Howlett Richard, Kelley Kevin, Hogan Michael Glycolytic activation at the onset of contractions in isolated Xenopus laevis single myofibres. EXPERIMENTAL PHYSIOLOGY. 2008;93(9):1076-1084.

7455. Mitchell-Gieleghem A, Mittelstaedt ME, Bulik CM Eating disorders and childbearing: Concealment and consequences. BIRTH-ISSUES IN PERINATAL CARE. 2002;29(3):182-191.

7456. Isabel Alvarez-Mercado Ana, Navarro-Oliveros Miguel, Robles-Sanchez Candido, Plaza-Diaz Julio, Jose Saez-Lara Maria, Munoz-Quezada Sergio, Fontana Luis, Abadia-Molina Francisco Microbial Population Changes and Their Relationship with Human Health and Disease. MICROORGANISMS. 2019;7(3):.

7457. Ifland J., Preuss H., Marcus M., Rourk K., Taylor W., Burau K., Jacobs W., Kadish W., Manso G. Refined food addiction: A classic substance use disorder. MEDICAL HYPOTHESES. 2009;72(5):518-526.

7458. Mohan Ananyaa, Huybrechts Inge, Michels Nathalie Psychosocial stress and cancer risk: a narrative review. EUROPEAN JOURNAL OF CANCER PREVENTION. 2022;31(6):585-599.

7459. Woodside DB, Bulik CM, Thornton L, Klump KL, Tozzi F, Fichter MM, Halmi KA, Kaplan AS, Strober M, Devlin B, Bacanu SA, Ganjei K, Crow S, Mitchell J, Rotondo A, Mauri M, Cassano G, Keel P, Berrettini WH, Kaye WH Personality in men with eating disorders. JOURNAL OF PSYCHOSOMATIC RESEARCH. 2004;57(3):273-278.

7460. Wan Weiguo, Jiang Xuejun, Li Xiaoyan, Zhang Cui, Yi Xin Silencing of angiotensin-converting enzyme by RNA interference prevents H9c2 cardiomyocytes from apoptosis induced by anoxia/reoxygenation through regulation of the intracellular renin-angiotensin system. INTERNATIONAL JOURNAL OF MOLECULAR MEDICINE. 2013;32(6):1380-1386.

7461. Wang Lei, Zhao Xujie, Mao Shuai, Liu Shaonan, Guo Xinfeng, Guo Liheng, Du Tinghai, Yang Haiyu, Zhao Fuhai, Wu Keng, Cong Hongliang, Wu Yang, Yang Phillip, Chen Keji, Zhang

Minzhou Efficacy of Danlou Tablet in Patients with Non-ST Elevation Acute Coronary Syndrome Undergoing Percutaneous Coronary Intervention: Results from a Multicentre, Placebo-Controlled, Randomized Trial. EVIDENCE-BASED COMPLEMENTARY AND ALTERNATIVE MEDICINE. 2016;2016():.

7462. Hosseinzadeh Mina, Ziaei Jamal, Mandavi Nader, Aghajari Parvaneh, Vahidi Maryam, Fateh Alaviehe, Asghari Elnaz Risk Factors for Breast Cancer in Iranian Women: A Hospital-Based Case-Control Study in Tabriz, Iran. JOURNAL OF BREAST CANCER. 2014;17(3):236-243.

7463. Wang Monica, Peterson Karen, Richmond Tracy, Spadano-Gasbarro Jennifer, Greaney Mary, Mezgebu Solomon, McCormick Marie, Austin S. Family Physical Activity and Meal Practices Associated With Disordered Weight Control Behaviors in a Multiethnic Sample of Middle-School Youth. ACADEMIC PEDIATRICS. 2013;13(4):379-385.

7464. Nagata Daisuke, Kiyosue Arihiro, Takahashi Masao, Satonaka Hiroshi, Tanaka Kimie, Sata Masataka, Nagano Tetsuo, Nagai Ryozo, Hirata Yasunobu A new constitutively active mutant of AMP-activated protein kinase inhibits anoxia-induced apoptosis of vascular endothelial cell. HYPERTENSION RESEARCH. 2009;32(2):133-139.

7465. CREPEL V, HAMMOND C, KRNJEVIC K, CHINESTRA P, BENARI Y ANOXIA-INDUCED LTP OF ISOLATED NMDA RECEPTOR-MEDIATED SYNAPTIC RESPONSES. JOURNAL OF NEUROPHYSIOLOGY. 1993;69(5):1774-1778.

7466. Gu Honglei, Bao Xiucong, Xia Tiansheng Basic psychological need frustration and adolescent non-suicidal self-injury: Testing a moderated mediation model of depression and self-compassion. CURRENT PSYCHOLOGY. 2022;():.

7467. Tavakol Zahra, Ghannadi Shima, Tabesh Mastaneh, Halabchi Farzin, Noormohammadpour Pardis, Akbarpour Samaneh, Alizadeh Zahra, Nezhad Malihe, Reyhan Sahar Relationship between physical activity, healthy lifestyle and COVID-19 disease severity; a cross-sectional study. JOURNAL OF PUBLIC HEALTH-HEIDELBERG. 2023;31(2):267-275.

7468. Liles EG, Woods SC Anorexia nervosa as viable behaviour: extreme self-deprivation in historical context. HISTORY OF PSYCHIATRY. 1999;10(38, 2):205-225.

7469. Vosloo Andre, Laas Anel, Vosloo Dalene Differential responses of juvenile and adult South African abalone (Haliotis midae Linnaeus) to low and high oxygen levels. COMPARATIVE BIOCHEMISTRY AND PHYSIOLOGY A-MOLECULAR \& INTEGRATIVE PHYSIOLOGY. 2013;164(1):192-199.

7470. Meier Evelyn, Gray James Facebook Photo Activity Associated with Body Image Disturbance in Adolescent Girls. CYBERPSYCHOLOGY BEHAVIOR AND SOCIAL NETWORKING. 2014;17(4):199-206.

7471. Precone V., Beccari T., Stuppia L., Baglivo M., Paolacci S., Manara E., Miggiano G., Falsini B., Trifiro A., Zanlari A., Herbst K., Unfer V., Bertelli M., Project GENEOB Taste, olfactory and texture related genes and food choices: implications on health status. EUROPEAN REVIEW FOR MEDICAL AND PHARMACOLOGICAL SCIENCES. 2019;23(3):1305-1321.

7472. Morley Belinda, Martin Jane, Niven Philippa, Wakefield Melanie Public opinion on food-related obesity prevention policy initiatives. HEALTH PROMOTION JOURNAL OF AUSTRALIA. 2012;23(2):86-91.

7473. Tanaka T, Hakoda S, Takeyama N Reoxygenation-induced mitochondrial damage is caused by the Ca2+-dependent mitochondrial inner membrane permeability transition. FREE RADICAL BIOLOGY AND MEDICINE. 1998;25(1):26-32.

7474. Mander Johannes, Wittorf Andreas, Teufel Martin, Schlarb Angelika, Hautzinger Martin, Zipfel Stephan, Sammet Isa Patients With Depression, Somatoform Disorders, and Eating Disorders on the Stages of Change: Validation of a Short Version of the URICA. PSYCHOTHERAPY. 2012;49(4):519-527.

7475. Takagi Satoshi, Kono Yu, Nagase Masashi, Mochio Soichiro, Kato Fusao Facilitation of distinct inhibitory synaptic inputs by chemical anoxia in neurons in the oculomotor, facial and hypoglossal motor nuclei of the rat. EXPERIMENTAL NEUROLOGY. 2017;290():95-105.

7476. Acres Meghan, Heath Joseph, Morris James Anorexia nervosa, autoimmunity and the hygiene hypothesis. MEDICAL HYPOTHESES. 2012;78(6):772-775.

7477. Wu Cheng, Wang Ming, Shi Hui Cholesterol Promotes Colorectal Cancer Growth by Activating the PI3K/AKT Pathway. JOURNAL OF ONCOLOGY. 2022;2022():.

7478. Sotos-Prieto Mercedes, Struijk Ellen, Fung Teresa, Rimm Eric, Rodriguez-Artalejo Fernando, Willett Walter, Hu Frank, Lopez-Garcia Esther Association between a lifestyle-based healthy heart score and risk of frailty in older women: a cohort study. AGE AND AGEING. 2022;51(2):.

7479. Hendricks KM, Dong KR, Tang AM, Ding B, Spiegelman D, Woods MN, Wanke CA High-fiber diet in HIV-positive men is associated with lower risk of developing fat deposition. AMERICAN JOURNAL OF CLINICAL NUTRITION. 2003;78(4):790-795.

7480. Perugi G., Ceraudo G., Vannucchi G., Rizzato S., Toni C., Dell'Osso L. Attention Deficit/Hyperactivity Disorder symptoms in Italian bipolar adult patients: A preliminary report. JOURNAL OF AFFECTIVE DISORDERS. 2013;149(1-3):430-434.

7481. Cordero-Herrera Isabel, Kozyra Mikael, Zhuge Zhengbing, Haworth Sarah, Moretti Chiara, Peleli Maria, Caldeira-Dias Mayara, Jahandideh Arghavan, Huirong Han, Cruz Josiane, Kleschyov Andrei, Montenegro Marcelo, Ingelman-Sundberg Magnus, Weitzberg Eddie, Lundberg Jon, Carlstrom Mattias AMP-activated protein kinase activation and NADPH oxidase inhibition by inorganic nitrate and nitrite prevent liver steatosis. PROCEEDINGS OF THE NATIONAL ACADEMY OF SCIENCES OF THE UNITED STATES OF AMERICA. 2019;116(1):217-226.

7482. Anna Kiss, Reka Ando, Peter Fritz, Zoltan Lakner Current and future burden of obesity at the Hungarian Roma population I. ORVOSI HETILAP. 2019;160(28):1097-1104.

7483. Ciao Anna, Latner Janet, Brown Krista, Ebneter Daria, Becker Carolyn Effectiveness of a peer-delivered dissonance-based program in reducing eating disorder risk factors in high school girls. INTERNATIONAL JOURNAL OF EATING DISORDERS. 2015;48(6):779-784.

7484. Li Kefeng, Liu Anli, Zong Wenhao, Dai Lulu, Liu Yang, Luo Renping, Ge Shulin, Dong Guijun Moderate exercise ameliorates osteoarthritis by reducing lipopolysaccharides from gut microbiota in mice. SAUDI JOURNAL OF BIOLOGICAL SCIENCES. 2021;28(1):40-49.

7485. Mirzay-Razaz Jalaledin, Hassanghomi Majid, Ajami Marjan, Koochakpoor Glareh, Hosseini-Esfahani Firoozeh, Mirmiran Parvin Effective food hygiene principles and dietary intakes to reinforce the immune system for prevention of COVID-19: a systematic review. BMC NUTRITION. 2022;8(1):.

7486. Shanahan Fergus, Sinderen Douwe, O'Toole Paul, Stanton Catherine Feeding the microbiota: transducer of nutrient signals for the host. GUT. 2017;66(9):1709-1717.

7487. Thilsing Trine, Sonderlund Anders, Sondergaard Jens, Svensson Nanna, Christensen Jeanette, Thomsen Janus, Hvidt Niels, Larsen Lars Changes in Health-Risk Behavior, Body Mass Index, Mental Well-Being, and Risk Status Following Participation in a Stepwise Web-Based and Face-to-Face Intervention for Prevention of Lifestyle-Related Diseases: Nonrandomized Follow-Up Cohort Study. JMIR PUBLIC HEALTH AND SURVEILLANCE. 2020;6(3):53-64.

7488. Garcia-Anaya Maria, Caballero-Romo Alejandro, Gonzalez-Macias Laura Parent-Focused Psychotherapy for the Preventive Management of Chronicity in Anorexia Nervosa: A Case Series. INTERNATIONAL JOURNAL OF ENVIRONMENTAL RESEARCH AND PUBLIC HEALTH. 2022;19(15):.

7489. Dykes S, Smilgin-Humphreys S, Bass C Chronic idiopathic constipation: a psychological enquiry. EUROPEAN JOURNAL OF GASTROENTEROLOGY \& HEPATOLOGY. 2001;13(1):39-44.

7490. Mason RB, Pluta RM, Walbridge S, Wink DA, Oldfield EH, Boock RJ Production of reactive oxygen species after reperfusion in vitro and in vivo: protective effect of nitric oxide. JOURNAL OF NEUROSURGERY. 2000;93(1):99-107.

7491. DeVylder Jordan, Lukens Ellen Family history of schizophrenia as a risk factor for axis I psychiatric conditions. JOURNAL OF PSYCHIATRIC RESEARCH. 2013;47(2):181-187.

7492. Gonzalez-Blazquez Raquel, Alcala Martin, Miguel Cardenas-Rebollo Jose, Viana Marta, Steckelings Ulrike, Boisvert William, Unger Thomas, Fernandez-Alfonso Maria, Somoza Beatriz, Gil-Ortega Marta AT(2)R stimulation with C21 prevents arterial stiffening and endothelial dysfunction in the abdominal aorta from mice fed a high-fat diet. CLINICAL SCIENCE. 2021;135(24):2763-2780.

7493. Finley John, Jaacks Lindsay, Peters Christian, Ort Donald, Aimone Ashley, Conrad Zach, Raiten Daniel Perspective: Understanding the Intersection of Climate/Environmental Change, Health, Agriculture, and Improved Nutrition - A Case Study: Type 2 Diabetes. ADVANCES IN NUTRITION. 2019;10(5):731-738.

7494. Teng Yu-Xian, Xie Si, Guo Ping-Ping, Deng Zhu-Jian, Zhang Zi-Yi, Gao Wei, Zhang Wan-Guang, Zhong Jian-Hong Hepatocellular Carcinoma in Non-alcoholic Fatty Liver Disease: Current Progresses and Challenges. JOURNAL OF CLINICAL AND TRANSLATIONAL HEPATOLOGY. 2022;10(5):955-964.

7495. Penalvo Jose, Sotos-Prieto Mercedes, Santos-Beneit Gloria, Pocock Stuart, Redondo Juliana, Fuster Valentin The Program Sl! intervention for enhancing a healthy lifestyle in preschoolers: first results from a cluster randomized trial. BMC PUBLIC HEALTH. 2013;13():.

7496. White Mary, Shoemaker Meredith, Park Sohyun, Neff Linda, Carlson Susan, Brown David, Kanny Dafna Prevalence of Modifiable Cancer Risk Factors Among US Adults Aged 18-44 Years. AMERICAN JOURNAL OF PREVENTIVE MEDICINE. 2017;53(3, 1, SI):S14-S20.

7497. Avanzini Fausto, Marzona Irene, Baviera Marta, Barlera Simona, Milani Valentina, Caimi Vittorio, Longoni Paolo, Tombesi Massimo, Silletta Maria, Tognoni Gianni, Roncaglioni Maria, Collaborat Risk Improving cardiovascular prevention in general practice: Results of a comprehensive personalized strategy in subjects at high risk. EUROPEAN JOURNAL OF PREVENTIVE CARDIOLOGY. 2016;23(9):947-955.

7498. Estlin Annabel, Ahern Amy, Griffin Simon, Strelitz Jean Modification of cardiovascular disease risk by health behaviour change following type 2 diabetes diagnosis. DIABETIC MEDICINE. 2021;38(10):.

7499. Azorin J., Kaladjian A., Besnier N., Adida M., Hantouche E., Lancrenon S., Akiskal H. Suicidal behaviour in a French Cohort of major depressive patients: Characteristics of attempters and nonattempters. JOURNAL OF AFFECTIVE DISORDERS. 2010;123(1-3):87-94.

7500. Borriello Giuseppina, Lavatelli Lisa, Ruzzi Francesca, Panariello Adelaide, Percudani Mauro Case Report: COVID-19 Infection With Gastrointestinal Symptoms and Mood Disorder: Criticalities in Differential Diagnosis, Therapy and Management of Complications. FRONTIERS IN PSYCHIATRY. 2021;12():.

7501. Thwaite Tanya, Heidke Penny, Williams Susan, Vandelanotte Corneel, Rebar Amanda, Khalesi Saman Barriers to healthy lifestyle behaviors in Australian nursing students: A qualitative study. NURSING \& HEALTH SCIENCES. 2020;22(4):921-928.

7502. Zaragozano JF, Vidal AJ, Picazo MV, Castro GG, Leita IP, Lopez JLO Anorexia nervosa and cerebral atrophy in adolescents. MEDICINA CLINICA. 2005;124(15):571-572.

7503. Vinokurova Daria, Zakharov Andrey, Chernova Kseniya, Burkhanova-Zakirova Gulshat, Horst Viktor, Lemale Coline, Dreier Jens, Khazipov Roustem Depth-profile of impairments in endothelin-1-induced focal cortical ischemia. JOURNAL OF CEREBRAL BLOOD FLOW AND METABOLISM. 2022;42(10):1944-1960.

7504. Kruger Ruan, Monyeki Makama, Schutte Aletta, Smith Wayne, Mels Catharina, Kruger Herculina, Pienaar Anita, Gafane-Matemane Lebo, Breet Yolandi, Lammertyn Leandi, Mokwatsi Gontse, Kruger Ankebe, Deacon Elmari, Hanssen Henner The Exercise, Arterial Modulation and Nutrition in Youth South Africa Study (ExAMIN Youth SA). FRONTIERS IN PEDIATRICS. 2020;8():.

7505. Roberts Susan, Silver Rachel, Das Sai, Fielding Roger, Gilhooly Cheryl, Jacques Paul, Kelly Jennifer, Mason Joel, McKeown Nicola, Reardon Meaghan, Rowan Sheldon, Saltzman Edward, Shukitt-Hale Barbara, Smith Caren, Taylor Allen, Wu Dayong, Zhang Fang, Panetta Karen, Booth Sarah Healthy Aging-Nutrition Matters: Start Early and Screen Often. ADVANCES IN NUTRITION. 2021;12(4):1438-1448.

7506. Willems Ruben, Pil Lore, Lambrinou Christina-Paulina, Kivela Jemina, Wikstrom Katja, Gonzalez-Gil Esther, De Miguel-Etayo Pilar, Nanasi Anna, Semanova Csilla, Van Stappen Vicky, Cardon Greet, Tsochev Kaloyan, Iotova Violeta, Chakarova Nevena, Makrilakis Konstantinos, Dafoulas George, Timpel Patrick, Schwarz Peter, Manios Yannis, Annemans Lieven, Grp Feel4Diabetes Methodology of the health economic evaluation of the Feel4Diabetes-study. BMC ENDOCRINE DISORDERS. 2020;20(1, SI):.

7507. Noble Gary, Stead Martine, Jones Sandra, McDermott Laura, McVie Danielle The paradoxical food buying behaviour of parents - Insights from the UK and Australia. BRITISH FOOD JOURNAL. 2007;109(4-5):387-398.

7508. Stella Alvarez Luz, Estrada Alejandro, Diego Goez Juan, Carreno Cristina, Patricia Mancilla Lorena The effects of socioeconomic status and short stature on overweight, obesity and the risk of metabolic complications in adults. COLOMBIA MEDICA. 2013;44(3):146-154.

7509. Ortega Miguel, Fraile-Martinez Oscar, Garcia-Montero Cielo, Angel Alvarez-Mon Miguel, Lahera Guillermo, Monserrat Jorge, Llavero-Valero Maria, Gutierrez-Rojas Luis, Molina Rosa, Rodriguez-Jimenez Roberto, Quintero Javier, Alvarez De Mon Melchor

Biological Role of Nutrients, Food and Dietary Patterns in the Prevention and Clinical Management of Major Depressive Disorder. NUTRIENTS. 2022;14(15):.

7510. Amirbahman Aria, Lake Bjorn, Norton Stephen Seasonal phosphorus dynamics in the surficial sediment of two shallow temperate lakes: a solid-phase and pore-water study. HYDROBIOLOGIA. 2013;701(1):65-77.

7511. Shotwell M., Drake K., Sidorov V., Wikswo J. Mechanistic Analysis of Challenge-Response Experiments. BIOMETRICS. 2013;69(3):741-747.

7512. Cohrdes Caroline, Santos-Hovener Claudia, Kajikhina Katja, Holling Heike The role of weight- and appearance-related discrimination on eating disorder symptoms among adolescents and emerging adults. BMC PUBLIC HEALTH. 2021;21(1):.

7513. Hamnvik Ole-Petter, McMahon Graham Glycemic Targets for Patients with Type 2 Diabetes Mellitus. MOUNT SINAI JOURNAL OF MEDICINE. 2009;76(3):227-233.

7514. Larocque Sarah, Cooke Steven, Blouin-Demers Gabriel A breath of fresh air: avoiding anoxia and mortality of freshwater turtles in fyke nets by the use of floats. AQUATIC CONSERVATION-MARINE AND FRESHWATER ECOSYSTEMS. 2012;22(2):198-205.

7515. Antel Jochen, Tan Susanne, Grabler Marvin, Ludwig Christine, Lohkemper Dominik, Brandenburg Tim, Barth Nikolaus, Hinney Anke, Libuda Lars, Remy Miriam, Milos Gabriella, Hebebrand Johannes Rapid amelioration of anorexia nervosa in a male adolescent during metreleptin treatment including recovery from hypogonadotropic hypogonadism. EUROPEAN CHILD \& ADOLESCENT PSYCHIATRY. 2022;31(10):1573-1579.

7516. Zhang Yan-Bo, Pan Xiong-Fei, Chen Junxiang, Cao Anlan, Xia Lu, Zhang Yuge, Wang Jing, Li Huiqi, Liu Gang, Pan An Combined lifestyle factors, all-cause mortality and cardiovascular disease: a systematic review and meta-analysis of prospective cohort studies. JOURNAL OF EPIDEMIOLOGY AND COMMUNITY HEALTH. 2021;75(1):92-99.

7517. Estey Esther, Roff Chelsea, Kozlowski Michael, Rovig Stephanie, Guyker Wendy, Cook-Cottone Catherine Efficacy of Eat Breathe Thrive: A randomized controlled trial of a yoga-based program. BODY IMAGE. 2022;42():427-439.

7518. Pietrzyk Lukasz Food properties and dietary habits in colorectal cancer prevention and development. INTERNATIONAL JOURNAL OF FOOD PROPERTIES. 2017;20(10):2323-2343.

7519. Wang Wenxiu, Lv Jun, Yu Canqing, Guo Yu, Pei Pei, Zhuang Zhenhuang, Yang Ling, Millwood Iona, Walters Robin, Chen Yiping, Du Huaidong, Wu Xianping, Chen Junshi, Chen Zhengming, Clarke Robert, Huang Tao, Li Liming, Collaborati China Lifestyle factors and fetal and childhood origins of type 2 diabetes: a prospective study of Chinese and European adults. AMERICAN JOURNAL OF CLINICAL NUTRITION. 2022;115(3):749-758.

7520. Rengefors K, Anderson DM Environmental and endogenous regulation of cyst germination in two freshwater dinoflagellates. JOURNAL OF PHYCOLOGY. 1998;34(4):568-577.

7521. Nelson Melissa, Lust Katherine, Story Mary, Ehlinger Ed Credit card debt, stress and key health risk behaviors among college students. AMERICAN JOURNAL OF HEALTH PROMOTION. 2008;22(6):400-407.

7522. Dallagi Yosra, Rahali Dalila, Perrotte Morgane, Dkhili Houssem, Korsan Asma, El May Michele, El Fazaa Saloua, Ramassamy Charles, El Golli Narges Date seeds alleviate behavioural and neuronal complications of metabolic syndrome in rats. ARCHIVES OF PHYSIOLOGY AND BIOCHEMISTRY. 2023;129(3):582-596.

7523. Aslibekyan Stella, Campos Hannia, Loucks Eric, Linkletter Crystal, Ordovas Jose, Baylin Ana Development of a Cardiovascular Risk Score for Use in Low- and Middle-Income Countries. JOURNAL OF NUTRITION. 2011;141(7):1375-1380.

7524. Lee Albert, St Leger Lawrence, Cheng Frances, Team Hong The status of health-promoting schools in Hong Kong and implications for further development. HEALTH PROMOTION INTERNATIONAL. 2007;22(4):316-326.

7525. Bakaloudi Dimitra, Chrysoula Lydia, Leonida Ioannis, Kotzakioula Evangelia, Theodoridis Xenophon, Chourdakis Michail Impact of the level of adherence to the Mediterranean Diet on blood pressure: A systematic review and meta-analysis of observational studies. CLINICAL NUTRITION. 2021;40(12):5771-5780.

7526. Stice Eric, Shaw Heather Eating disorders: Insights from imaging and behavioral approaches to treatment. JOURNAL OF PSYCHOPHARMACOLOGY. 2017;31(11, SI):1485-

1495.

7527. Beauchamp P, Richard V, Tamion F, Lallemand F, Lebreton JP, Vaudry H, Daveau M, Thuillez C Protective effects of preconditioning in cultured rat endothelial cells - Effects on neutrophil adhesion and expression of ICAM-1 after anoxia and reoxygenation. CIRCULATION. 1999;100(5):541-546.

7528. Pedro Morera Luis, Noel Marchiori Georgina, Adrian Medrano Leonardo, Daniela Defago Maria Stress, Dietary Patterns and Cardiovascular Disease: A Mini-Review. FRONTIERS IN NEUROSCIENCE. 2019;13():.

7529. Norman Asa, Bohman Benjamin, Nyberg Gisela, Elinder Liselotte Psychometric Properties of a Scale to Assess Parental Self-Efficacy for Influencing Children's Dietary, Physical Activity, Sedentary, and Screen Time Behaviors in Disadvantaged Areas. HEALTH EDUCATION \& BEHAVIOR. 2018;45(1):132-140.

7530. Musaiger Abdulrahman, Hassan Abdelmonem, Obeid Omar The Paradox of Nutrition-Related Diseases in the Arab Countries: The Need for Action. INTERNATIONAL JOURNAL OF ENVIRONMENTAL RESEARCH AND PUBLIC HEALTH. 2011;8(9):3637-3671.

7531. Vanajan Anushiya, Stier-Jarmer Marita, Ivandic Ivana, Schuh Angela, Sabariego Carla Can Participants' Characteristics Predict Benefit from a Multimodal Burnout Prevention Program? Secondary Analysis of a Randomized Controlled Trial Conducted in Germany. BEHAVIORAL MEDICINE. 2020;46(2):120-129.

7532. Bingaman Amanda, Waggoner Christine, Andrews Sara, Pangonis Diana, Trad Marie, Giugliani Roberto, Giorgino Ruben, Jarnes Jeanine, Vakili Rojan, Ballard Victoria, Peay Holly GM1-gangliosidosis: The caregivers' assessments of symptom impact and most important symptoms to treat. AMERICAN JOURNAL OF MEDICAL GENETICS PART A. 2023;191(2):408-423.

7533. Yang Tian, Yang Xudan, Wang Luping, Mo Jun Agenesis of the dorsal pancreas presenting with diabetic ketoacidosis - a case report and literature review. BMC ENDOCRINE DISORDERS. 2019;19(1):.

7534. Castro-Vazquez Genaro Ethno-essentialisms of the self: A critique of the cultural scripting of obesity in Japan. SOCIOLOGY OF HEALTH \& ILLNESS. 2021;43(3):796-811.

7535. Rice Elise, Patel Minal, Serrano Katrina, Thai Chan, Blake Kelly, Vanderpool Robin Beliefs About Behavioral Determinants of Obesity in Appalachia, 2011-2014. PUBLIC HEALTH REPORTS. 2018;133(4):379-384.

7536. Kjeldsen Emilie, Thomassen Jesper, Rasmussen Katrine, Nordestgaard Borge, Tybjaerg-Hansen Anne, Frikke-Schmidt Ruth Impact of diet on ten-year absolute cardiovascular risk in a prospective cohort of 94 321 individuals: A tool for implementation of healthy diets. LANCET REGIONAL HEALTH-EUROPE. 2022;19():.

7537. Arduini Tiziano, Iorio Daniela, Patacchini Eleonora Weight, reference points, and the onset of eating disorders. JOURNAL OF HEALTH ECONOMICS. 2019;65():170-188.

7538. Arena Ross, Bond Samantha, Calvo Isabel, Lebowicz Leah, Ozemek Cemal, Severin Richard, Laddu Deepika, Faghy Mark, Lavie Carl, Carbone Salvatore, Network HL-PIVOT Shelter from the cytokine storm: Healthy living is a vital preventative strategy in the COVID-19 era. PROGRESS IN CARDIOVASCULAR DISEASES. 2022;73():56-60.

7539. Habibovic Mirela, Broers Eva, Piera-Jimenez Jordi, Wetzels Mart, Ayoola Idowu, Denollet Johan, Widdershoven Jos Enhancing Lifestyle Change in Cardiac Patients Through the Do CHANGE System ({''}Do Cardiac Health: Advanced New Generation Ecosystem{''}): Randomized Controlled Trial Protocol. JMIR RESEARCH PROTOCOLS. 2018;7(2):.

7540. Schiestl Emma, Rios Julia, Parnarouskis Lindsey, Cummings Jenna, Gearhardt Ashley A narrative review of highly processed food addiction across the lifespan. PROGRESS IN NEURO-PSYCHOPHARMACOLOGY \& BIOLOGICAL PSYCHIATRY. 2021;106():.

7541. Karwautz Andreas, Wagner Gudrun, Berger Gabriele, Sinnreich Ursula, Grylli Vasileia, Huber Wolf-Dietrich Eating pathology in adolescents with celiac disease. PSYCHOSOMATICS. 2008;49(5):399-406.

7542. Silva Wanderson, Teixeira Patricia, Maroco Joao, Ferreira Eric, Teodoro Micaela, Duarte Bonini Campos Juliana Relationship between Attention to Body Shape, Social Physique Anxiety, and Personal Characteristics of Brazilians: A Structural Equation Model. INTERNATIONAL JOURNAL OF ENVIRONMENTAL RESEARCH AND PUBLIC HEALTH. 2022;19(22):.

7543. Buck L., Pamenter M. Adaptive responses of vertebrate neurons to anoxia - Matching supply to demand. RESPIRATORY PHYSIOLOGY \& NEUROBIOLOGY. 2006;154(1-2):226-

240.

7544. Adjibade Moufidath, Mariotti Francois, Leroy Pascal, Souchon Isabelle, Saint-Eve Anne, Fagherazzi Guy, Soler Louis-Georges, Huneau Jean-Francois Impact of intra-category food substitutions on the risk of type 2 diabetes: a modelling study on the pizza category. BRITISH JOURNAL OF NUTRITION. 2022;127(8):1240-1249.

7545. Serretti Alessandro, Calati Raffaella, Mandelli Laura, De Ronchi Diana Serotonin transporter gene variants and behavior: A comprehensive review. CURRENT DRUG TARGETS. 2006;7(12):1659-1669.

7546. Amerzadeh Mohammad, Takian Amirhossein, Pouraram Hamed, Sari Ali, Ostovar Afshin Policy analysis of nutrition stewardship for prevention and control of Non-communicable diseases in Iran. BMC HEALTH SERVICES RESEARCH. 2023;23(1):.

7547. Singh S., Taillie Lindsey, Gupta Ashish, Bercholz Maxime, Popkin Barry, Murukutla Nandita Front-of-Package Labels on Unhealthy Packaged Foods in India: Evidence from a Randomized Field Experiment. NUTRIENTS. 2022;14(15):.

7548. Shaw HE, Stice E, Springer DW Perfectionism, body dissatisfaction, and self-esteem in predicting bulimic symptomatology: Lack of replication. INTERNATIONAL JOURNAL OF EATING DISORDERS. 2004;36(1):41-47.

7549. LEGRANGE D FAMILY-THERAPY OUTCOME IN ADOLESCENT ANOREXIA-NERVOSA. SOUTH AFRICAN JOURNAL OF PSYCHOLOGY. 1993;23(4):174-179.

7550. Jefferson Wendy, Zunker Christie, Feucht Jennifer, Fitzpatrick Stephanie, Greene Lori, Shewchuk Richard, Baskin Monica, Walton Norman, Phillips Beatrice, Ard Jamy Use of the Nominal Group Technique (NGT) to understand the perceptions of the healthiness of foods associated with African Americans. EVALUATION AND PROGRAM PLANNING. 2010;33(4):343-348.

7551. Cook Jessica, McCormick Emily, Mickiewicz Theresa, Davidson Arthur, Main Deborah Associations of Adolescent Weight Status and Meeting National Obesity-Related Recommendations. JOURNAL OF SCHOOL HEALTH. 2017;87(12):923-931.

7552. Ciao Anna, Duvall Ally, Pascual Summer, Lawley Kendall Expert peer facilitation of the EVERYbody Project: A randomized-controlled evaluation of a diversity-focused, dissonance-based, universal body image program for college students. INTERNATIONAL JOURNAL OF EATING DISORDERS. 2022;():.

7553. Springett R, Newman J, Cope M, Delpy DT Oxygen dependency and precision of cytochrome oxidase signal from full spectral NIRS of the piglet brain. AMERICAN JOURNAL OF PHYSIOLOGY-HEART AND CIRCULATORY PHYSIOLOGY. 2000;279(5):H2202-H2209.

7554. Rezende Leandro, Azeredo Catarina, Canella Daniela, Luiz Olinda, Levy Renata, Eluf-Neto Jose Coronary heart disease mortality, cardiovascular disease mortality and all-cause mortality attributable to dietary intake over 20 years in Brazil. INTERNATIONAL JOURNAL OF CARDIOLOGY. 2016;217():64-68.

7555. Khaidakov Magomed, Mercanti Federico, Wang Xianwei, Ding Zufeng, Dai Yao, Romeo Francesco, Sawamura Tatsuya, Mehta Jawahar Prevention of export of anoxia/reoxygenation injury from ischemic to nonischemic cardiomyocytes via inhibition of endocytosis. AMERICAN JOURNAL OF PHYSIOLOGY-HEART AND CIRCULATORY PHYSIOLOGY. 2014;306(12):H1700-H1707.

7556. Lelli Lorenzo, Castellini Giovanni, Cassioli Emanuele, Monteleone Alessio, Ricca Valdo Cortisol levels before and after cognitive behavioural therapy in patients with eating disorders reporting childhood abuse: A follow-up study. PSYCHIATRY RESEARCH. 2019;275():269-275.

7557. McCreedy Nicole, Shung-King Maylene, Weimann Amy, Tatah Lambed, Mapa-Tassou Clarisse, Muzenda Trish, Govia Ishtar, Were Vincent, Oni Tolu Reducing Sugar Intake in South Africa: Learnings from A Multilevel Policy Analysis on Diet and Noncommunicable Disease Prevention. INTERNATIONAL JOURNAL OF ENVIRONMENTAL RESEARCH AND PUBLIC HEALTH. 2022;19(18):.

7558. Zanobini Patrizio, Lorini Chiara, Lastrucci Vieri, Minardi Valentina, Possenti Valentina, Masocco Maria, Garofalo Giorgio, Mereu Giovanna, Bonaccorsi Guglielmo Health Literacy, Socio-Economic Determinants, and Healthy Behaviours: Results from a Large Representative Sample of Tuscany Region, Italy. INTERNATIONAL JOURNAL OF ENVIRONMENTAL RESEARCH AND PUBLIC HEALTH. 2021;18(23):.

7559. Blomquist Kerstin, Pate Sarah, Hock Amanda, Austin S. Evidence-based policy solutions to prevent eating disorders: Do disclaimer labels on fashion advertisements

mitigate negative impact on adult women?. BODY IMAGE. 2022;43():180-192.

7560. Hosseini Fatemeh, Shab-Bidar Sakineh, Ghanbari Mahtab, Majdi Maryam, Sheikhhossein Fatemeh, Imani Hossein Food Quality Score and Risk of Breast Cancer among Iranian Women: Findings from a Case Control Study. NUTRITION AND CANCER-AN INTERNATIONAL JOURNAL. 2022;74(5):1660-1669.

7561. Altunel Attila, Muduroglu-Kirmizibekmez Aynur, Altunel Ozlem, Sever Ali, Kara Ihsan Hypsarrhythmia paroxysm intensities that initiate and render physical and mental retardation irreversible in West syndrome. INTERNATIONAL JOURNAL OF NEUROSCIENCE. 2022;():.

7562. Kletkiewicz Hanna, Maliszewska Justyna, Jaworski Krzysztof, Jermacz Lukasz, Smolinski Dariusz, Rogalska Justyna Thermal conditions during neonatal anoxia affect the endogenous level of brain-derived neurotrophic factor. JOURNAL OF NEUROSCIENCE RESEARCH. 2019;97(10):1266-1277.

7563. Zhou XB, Zhai XL, Ashraf M Preconditioning of bovine endothelial cells - The protective effect is mediated by an adenosine A(2) receptor through a protein kinase C signaling pathway. CIRCULATION RESEARCH. 1996;78(1):73-81.

7564. Zhang Honggang, Lyu Tao, Bi Lei, Tempero Grant, Hamilton David, Pan Gang Combating hypoxia/anoxia at sediment-water interfaces: A preliminary study of oxygen nanobubble modified clay materials. SCIENCE OF THE TOTAL ENVIRONMENT. 2018;637():550-560.

7565. Shiau Mei-Huey, Lee Meng-Chih, Lin Fang-Ling, Hurng Baai-Shyun, Yeh Chih-Jung Cross-Sectional, Short-, Medium-, and Long-Term Effects of Dietary Pattern on Frailty in Taiwan. INTERNATIONAL JOURNAL OF ENVIRONMENTAL RESEARCH AND PUBLIC HEALTH. 2021;18(18):.

7566. Nikonenko I, Jourdain P, Muller D Presynaptic remodeling contributes to activity-dependent synaptogenesis. JOURNAL OF NEUROSCIENCE. 2003;23(24):8498-8505.

7567. Ladilov Y, Schafer C, Held A, Schafer M, Noll T, Piper HM Mechanism of Ca2+ overload in endothelial cells exposed to simulated ischemia. CARDIOVASCULAR RESEARCH. 2000;47(2):394-403.

7568. Hanaki Takehiko, Sakamoto Teruhisa, Yata Shinsaku, Murakami Yuki, Fujiwara Yoshiyuki Successful Interventional Radiology for Acute Median Arcuate Ligament Syndrome After Pancreaticoduodenectomy. CUREUS. 2021;13(2):.

7569. Hatta Naoko, Tada Yuki, Ishikawa-Takata Kazuko, Furusho Tadasu, Kanehara Rieko, Hata Toshiki, Hida Azumi, Kawano Yukari Energy Intake from Healthy Foods Is Associated with Motor Fitness in Addition to Physical Activity: A Cross-Sectional Study of First-Grade Schoolchildren in Japan. INTERNATIONAL JOURNAL OF ENVIRONMENTAL RESEARCH AND PUBLIC HEALTH. 2022;19(3):.

7570. Pomeranz Jennifer, Yang Y. The Affordable Care Act and State Coverage of Clinical Preventive Health Services for Working-Age Adults. JOURNAL OF PUBLIC HEALTH MANAGEMENT AND PRACTICE. 2015;21(1):87-95.

7571. Snowdon Wendy, Schultz Jimaima, Swinburn Boyd Problem and solution trees: a practical approach for identifying potential interventions to improve population nutrition. HEALTH PROMOTION INTERNATIONAL. 2008;23(4):345-353.

7572. Dev Dipti, McBride Brent, Speirs Katherine, Blitch Kimberly, Williams Natalie ``Great Job Cleaning Your Plate Today!{''} Determinants of Child-Care Providers' Use of Controlling Feeding Practices: An Exploratory Examination. JOURNAL OF THE ACADEMY OF NUTRITION AND DIETETICS. 2016;116(11):1803-1809.

7573. Slowakiewicz Miroslaw, Tucker Maurice, Perri Edoardo, Pancost Richard Nearshore euxinia in the photic zone of an ancient sea. PALAEOGEOGRAPHY PALAEOCLIMATOLOGY PALAEOECOLOGY. 2015;426():242-259.

7574. Sharma Sudesh, Page Rachel, Matheson Anna, Lambrick Danielle, Faulkner James, Mishra Shiva Non-communicable disease prevention in Nepal: systemic challenges and future directions. GLOBAL HEALTH PROMOTION. 2019;26(3):94-97.

7575. Domisch Timo, Qian Ji, Sondej Izabela, Martz Francoise, Lehto Tarja, Piirainen Sirpa, Finer Leena, Silvennoinen Raimo, Repo Tapani Here comes the flood! Stress effects of continuous and interval waterlogging periods during the growing season on Scots pine saplings. TREE PHYSIOLOGY. 2020;40(7):869-885.

7576. Pineda Elisa, Poelman Maartje, Aaspollu Anu, Bica Margarida, Bouzas Cristina, Carrano Elena, De Miguel-Etayo Pilar, Djojosoeparto Sanne, Blenkus Mojca, Graca Pedro,

Geffert Karin, Hebestreit Antje, Helldan Anni, Henjum Sigrun, Huseby Camilla, Gregorio Maria, Kamphuis Carlijn, Laatikainen Tiina, Lovhaug Anne, Leydon Clarissa, Luszczynska Aleksandra, Maki Paivi, Martinez J., Raulio Susanna, Romaniuk Piotr, Roos Gun, Salvador Clara, Sassi Franco, Silano Marco, Sotlar Ingrid, Specchia Maria, Arriaga Miguel, Terragni Laura, Torheim Liv, Tur Josep, Philipsborn Peter, Harrington Janas, Vandevijvere Stefanie Policy implementation and priorities to create healthy food environments using the Healthy Food Environment Policy Index (Food-EPI): A pooled level analysis across eleven European countries. LANCET REGIONAL HEALTH-EUROPE. 2022;23():.

7577. Potter BK, Pederson LL, Chan SSH, Aubut JAL, Koval JJ Does a relationship exist between body weight, concerns about weight, and smoking among adolescents? An integration of the literature with an emphasis on gender. NICOTINE \& TOBACCO RESEARCH. 2004;6(3):397-425.

7578. Jelby Mads, Ineson Jon, Thibault Nicolas, Bodin Stephane, Blok Carlette, Edvardsen Niklas, Clemmensen Tatjana, Buls Toms, Anderskouv Kresten Facies and depositional processes of Lower Cretaceous carbonates, Danish Central Graben. BULLETIN OF THE GEOLOGICAL SOCIETY OF DENMARK. 2022;71():51-74.

7579. WAXMAN SG, BLACK JA, RANSOM BR, STYS PK ANOXIC INJURY OF RAT OPTIC-NERVE - ULTRASTRUCTURAL EVIDENCE FOR COUPLING BETWEEN NA+ INFLUX AND CA2+-MEDIATED INJURY IN MYELINATED CNS AXONS. BRAIN RESEARCH. 1994;644(2):197-204.

7580. Levine Michele, Cheng Yu, Marcus Marsha, Emery Rebecca Psychiatric disorders and gestational weight gain among women who quit smoking during pregnancy. JOURNAL OF PSYCHOSOMATIC RESEARCH. 2015;78(5):504-508.

7581. Baltali M, Kiziltan HT, Korkmaz ME, Topcu S, Demirtas M, Metin M, Topcuoglu MS, Birand A, Muderrisoglu H Prevalence of modifiable cardiovascular risk factors remain high after coronary bypass graft surgery: a multicentre study among Turkish patients. JOURNAL OF CARDIOVASCULAR RISK. 2002;9(4):207-214.

7582. Campbell David Economic Rationality in Choosing between Short-Term Bad-Health Choices and Longer-Term Good-Health Choices. INTERNATIONAL JOURNAL OF ENVIRONMENTAL RESEARCH AND PUBLIC HEALTH. 2013;10(11):5971-5988.

7583. Boettcher Hannah, Brake C., Barlow David Origins and outlook of interoceptive exposure. JOURNAL OF BEHAVIOR THERAPY AND EXPERIMENTAL PSYCHIATRY.

2016;53(SI):41-51.

7584. Becker Carolyn, Perez Marisol, Kilpela Lisa, Diedrichs Phillippa, Trujillo Eva, Stice Eric Engaging stakeholder communities as body image intervention partners: The Body Project as a case example. EATING BEHAVIORS. 2017;25(SI):62-67.

7585. Peter L, Nighoghossian N, Jouvet A, Derex L, Hermier M, Philippeau F, Honnorat J, Trouillas P Delayed post-anoxic leukoencephalopathy.. REVUE NEUROLOGIQUE. 2004;160(11):1085-1088.

7586. Aoki Chiye, Sabaliauskas Nicole, Chowdhury Tara, Min Jung-Yun, Colacino Anna, Laurino Kevin, Barbarich-Marsteller Nicole Adolescent female rats exhibiting activity-based anorexia express elevated levels of GABAA receptor a4 and d subunits at the plasma membrane of hippocampal CA1 spines. SYNAPSE. 2012;66(5):391-407.

7587. Tressera-Rimbau A., Arranz S., Eder M., Vallverdu-Queralt A. Dietary Polyphenols in the Prevention of Stroke. OXIDATIVE MEDICINE AND CELLULAR LONGEVITY. 2017;2017():.

7588. Eilers H, Bickler PE Hypothermia and isoflurane similarly inhibit glutamate release evoked by chemical anoxia in rat cortical brain slices. ANESTHESIOLOGY. 1996;85(3):600-607.

7589. Le Long, Tan Eng, Perez Joahna, Chiotelis Oxana, Hay Phillipa, Ananthapavan Jaithri, Lee Yong, Mihalopoulos Cathrine Prevention of high body mass index and eating disorders: a systematic review and meta-analysis. EATING AND WEIGHT DISORDERS-STUDIES ON ANOREXIA BULIMIA AND OBESITY. 2022;27(8):2989-3003.

7590. Wittchen HU, Lachner G, Wunderlich U, Pfister H Test-retest reliability of the computerized DSM-IV version of the Munich Composite International Diagnostic Interview (M-CIDI). SOCIAL PSYCHIATRY AND PSYCHIATRIC EPIDEMIOLOGY. 1998;33(11):568-578.

7591. Voelker Dana, Petrie Trent, Fairhurst Katherine, Casanave Karly ``My Body Loves Me, So I Should Love It Back{''}: A Qualitative Evaluation of the Bodies in Motion Program With Female Collegiate Athletes. SPORT EXERCISE AND PERFORMANCE PSYCHOLOGY. 2021;10(1):43-58.

7592. Shin Jun-Kyu, Kim Jae-Sung Cytoprotection of rat hepatocytes by desipramine in a model of simulated ischemia/reperfusion. BIOCHEMISTRY AND BIOPHYSICS REPORTS. 2021;27():.

7593. Subic-Wrana C, Bruder S, Thomas W, Lane RD, Kohle K Emotional awareness deficits in inpatients of a psychosomatic ward: A comparison of two different measures of alexithymia. PSYCHOSOMATIC MEDICINE. 2005;67(3):483-489.

7594. Torre Ma, Vicente Herrero Ma, Lopez Gonzalez Angel, Capdevila Garcia Luisa Metabolic syndrome and type 2 diabetes. Risk estimation in apparently healthy workers. MEDICINA BALEAR. 2020;35(2):34-40.

7595. Al Jawaldeh Ayoub, Al-Jawaldeh Hanin Fat Intake Reduction Strategies among Children and Adults to Eliminate Obesity and Non-Communicable Diseases in the Eastern Mediterranean Region. CHILDREN-BASEL. 2018;5(7):.

7596. Baser Duygu, Cankurtaran Mustafa The Assessment of the Orthorexia Nervosa Tendencies among Postpartum Women. KONURALP TIP DERGISI. 2021;13(2):218-225.

7597. Nickel MK, Tritt K, Mitterlehner FO, Leiberich P, Nickel C, Lahmann C, Forthuber P, Rother WK, Loew TH Sexual abuse in childhood and youth as psychopathologically relevant life occurrence: Cross-sectional survey. CROATIAN MEDICAL JOURNAL. 2004;45(4):483-489.

7598. Matthews Abigail, Kramer Rachel, Mitan Laurie Eating disorder severity and psychological morbidity in adolescents with anorexia nervosa or atypical anorexia nervosa and premorbid overweight/obesity. EATING AND WEIGHT DISORDERS-STUDIES ON ANOREXIA BULIMIA AND OBESITY. 2022;27(1):233-242.

7599. Dziergowska Katarzyna, Labowska Magdalena, Gasior-Glogowska Marlena, Kmiecik Barbara, Detyna Jerzy Modern noninvasive methods for monitoring glucose levels in patients: a review. BIO-ALGORITHMS AND MED-SYSTEMS. 2019;15(4):.

7600. Grasby Stephen, Beauchamp Benoit, Bond David, Wignall Paul, Talavera Cristina, Galloway Jennifer, Piepjohn Karsten, Reinhardt Lutz, Blomeier Dierk Progressive environmental deterioration in northwestern Pangea leading to the latest Permian extinction. GEOLOGICAL SOCIETY OF AMERICA BULLETIN. 2015;127(9-10):1331-1347.

7601. Menyhart Akos, Varga Daniel, Toth Orsolya, Makra Peter, Bari Ferenc, Farkas Eszter Transient Hypoperfusion to Ischemic/Anoxic Spreading Depolarization is Related to Autoregulatory Failure in the Rat Cerebral Cortex. NEUROCRITICAL CARE. 2022;37(SUPPL 1, 1, SI):112-122.

7602. Priest Naomi, Armstrong Rebecca, Doyle Jodie, Waters Elizabeth Policy interventions implemented through sporting organisations for promoting healthy behaviour change. COCHRANE DATABASE OF SYSTEMATIC REVIEWS. 2008;(3):.

7603. Gupta MA, Gilchrest BA Psychosocial aspects of aging skin. DERMATOLOGIC CLINICS. 2005;23(4):643+.

7604. Polidori M., Pientka L. A brief update on dementia prevention. ZEITSCHRIFT FUR GERONTOLOGIE UND GERIATRIE. 2012;45(1):7-10.

7605. Li Zhou, Wang Junjie, Ji Yuzhu, Song Fangzhou Expression Characteristics and Clinical Correlations of BRD1 in Colorectal Cancer Samples. TECHNOLOGY IN CANCER RESEARCH \& TREATMENT. 2021;20():.

7606. Tyrer Peter NOSOLOGY IS USUALLY WRONG, BUT IS A TEST BED FOR SCIENCE: A COMMENTARY ON MARKON. JOURNAL OF PERSONALITY DISORDERS. 2013;27(5):590-593.

7607. BARANTIN L, AKOKA S, TRANQUART F, SALIBA E, POURCELOT L MAGNETIC-RESONANCE SPECTROSCOPY - METHODOLOGY AND APPLICATIONS TO THE STUDY OF BIRTH ASPHYXIA. NEUROPHYSIOLOGIE CLINIQUE-CLINICAL NEUROPHYSIOLOGY. 1995;25(3):115-129.

7608. Shaibu Sheila, Holsten Joanna, Stettler Nicolas, Maruapula Segametsi, Jackson Jose, Malete Leapetswe, Mokone George, Wrotniak Brian, Compher Charlene Adolescent Obesity Prevention in Botswana: Beliefs and Recommendations of School Personnel. JOURNAL OF SCHOOL NURSING. 2012;28(3):220-229.

7609. Coenen A., Lankhaar J., Lowe J., McKeegan D. Remote monitoring of electroencephalogram, electrocardiogram, and behavior during controlled atmosphere stunning in broilers: Implications for welfare. POULTRY SCIENCE. 2009;88(1):10-19.

7610. Arnulf Isabelle, Lin Ling, Gadoth Nathan, File Jennifer, Lecendreux Michel, Franco Patricia, Zeitzer Jamie, Lo Betty, Faraco Juliette, Mignot Emmanuel Kleine-Levin syndrome: A systematic study of 108 patients. ANNALS OF NEUROLOGY. 2008;63(4):482-493.

7611. Itriyeva Khalida The effects of obesity on the menstrual cycle. CURRENT PROBLEMS IN PEDIATRIC AND ADOLESCENT HEALTH CARE. 2022;52(8):.

7612. Foster Hatnish, Celis-Morales Carlos, Nicholl Barbara, Petermann-Rocha Fanny, Pell Jill, Gill Jason, O'Donnell Catherine, Mair Frances The effect of socioeconomic deprivation on the association between an extended measurement of unhealthy lifestyle factors and health outcomes: a prospective analysis of the UK Biobank cohort. LANCET PUBLIC HEALTH. 2018;3(12):E576-E585.

7613. Milton Alyssa, Hambleton Ashlea, Dowling Mitchell, Roberts Anna, Davenport Tracey, Hickie Ian Technology-Enabled Reform in a Nontraditional Mental Health Service for Eating Disorders: Participatory Design Study. JOURNAL OF MEDICAL INTERNET RESEARCH. 2021;23(2):.

7614. Yamaguchi S, Endo K, Kitajima T, Hori Y Extracellular sodium concentration has diverse effects on the hypoxia-induced increase in intracellular Ca2+ in rat hippocampal slices. NEUROSCIENCE LETTERS. 1997;232(3):163-166.

7615. Bernas T, Zarebski M, Cook RR, Dobrucki JW Minimizing photobleaching during confocal microscopy of fluorescent probes bound to chromatin: role of anoxia and photon flux. JOURNAL OF MICROSCOPY. 2004;215(3):281-296.

7616. Aggarwal Monica, Bozkurt Biykem, Panjrath Gurusher, Aggarwal Brooke, Ostfeld Robert, Barnard Neal, Gaggin Hanna, Freeman Andrew, Allen Kathleen, Madan Shivank, Massera Daniele, Litwin Sheldon, Nutr Amer, Cardio Lifestyle Lifestyle Modifications for Preventing and Treating Heart Failure. JOURNAL OF THE AMERICAN COLLEGE OF CARDIOLOGY. 2018;72(19):2391-2405.

7617. Teipel Stefan, Gustafson Deborah, Ossenkoppele Rik, Hansson Oskar, Babiloni Claudio, Wagner Michael, Riedel-Heller Steffi, Kilimann Ingo, Tang Yi Alzheimer Disease: Standard of Diagnosis, Treatment, Care, and Prevention. JOURNAL OF NUCLEAR MEDICINE. 2022;63(7):981-985.

7618. Nedergaard Anders, Henriksen Kim, Karsdal Morten, Christiansen Claus Musculoskeletal ageing and primary prevention. BEST PRACTICE \& RESEARCH CLINICAL OBSTETRICS \& GYNAECOLOGY. 2013;27(5):673-688.

7619. Su Xiujuan, Liang Hong, Yuan Wei, Olsen Jorn, Cnattingius Sven, Li Jiong Prenatal and early life stress and risk of eating disorders in adolescent girls and young women. EUROPEAN CHILD \& ADOLESCENT PSYCHIATRY. 2016;25(11):1245-1253.

7620. Bulik Cynthia, Marcus Marsha, Zerwas Stephanie, Levine Michele, Hofmeier Sara, Trace Sara, Hamer Robert, Zimmer Benjamin, Moessner Markus, Kordy Hans CBT4BN versus CBTF2F: Comparison of online versus face-to-face treatment for bulimia nervosa. CONTEMPORARY CLINICAL TRIALS. 2012;33(5):1056-1064.

7621. McKisack C, Waller G Factors influencing the outcome of group psychotherapy for bulimia nervosa. INTERNATIONAL JOURNAL OF EATING DISORDERS. 1997;22(1):1-13.

7622. Imayama Ikuyo, Alfano Catherine, Kong Angela, Foster-Schubert Karen, Bain Carolyn, Xiao Liren, Duggan Catherine, Wang Ching-Yun, Campbell Kristin, Blackburn George, McTiernan Anne Dietary weight loss and exercise interventions effects on quality of life in overweight/obese postmenopausal women: a randomized controlled trial. INTERNATIONAL JOURNAL OF BEHAVIORAL NUTRITION AND PHYSICAL ACTIVITY. 2011;8():.

7623. Tseng Mei-Chih, Chien Li-Nien, Tu Chao-Ying, Liu Hung-Yi Mortality in anorexia nervosa and bulimia nervosa: A population-based cohort study in Taiwan, 2002-2017. INTERNATIONAL JOURNAL OF EATING DISORDERS. 2023;56(6):1135-1144.

7624. Salmi Pauliina, Malin Ismo, Salonen Kalevi Pumping of epilimnetic water into hypolimnion improves oxygen but not necessarily nutrient conditions in a lake recovering from eutrophication. INLAND WATERS. 2014;4(4):425-434.

7625. Tabuchi K, Okubo H, Fujihira K, Tsuji S, Hara A, Kusakari J Protection of outer hair cells from reperfusion injury by an iron chelator and a nitric oxide synthase inhibitor in the guinea pig cochlea. NEUROSCIENCE LETTERS. 2001;307(1):29-32.

7626. Gillberg I., Rastam Maria, Wentz Elisabet, Gillberg Christopher Cognitive and executive functions in anorexia nervosa ten years after onset of eating disorder. JOURNAL

OF CLINICAL AND EXPERIMENTAL NEUROPSYCHOLOGY. 2007;29(2):170-178.

7627. Wang Fenglei, Baden Megu, Guasch-Ferre Marta, Wittenbecher Clemens, Li Jun, Li Yanping, Wan Yi, Bhupathiraju Shilpa, Tobias Deirdre, Clish Clary, Mucci Lorelei, Eliassen A., Costenbader Karen, Karlson Elizabeth, Ascherio Alberto, Rimm Eric, Manson JoAnn, Liang Liming, Hu Frank Plasma metabolite profiles related to plant-based diets and the risk of type 2 diabetes. DIABETOLOGIA. 2022;65(7):1119-1132.

7628. Ng Ryan, Sutradhar Rinku, Kornas Kathy, Wodchis Walter, Sarkar Joykrishna, Fransoo Randall, Rosella Laura Development and Validation of the Chronic Disease Population Risk Tool (CDPoRT) to Predict Incidence of Adult Chronic Disease. JAMA NETWORK OPEN. 2020;3(6):.

7629. Mehler Philip, MacKenzie Thomas Treatment of Osteopenia and Osteoporosis in Anorexia Nervosa: A Systematic Review of the Literature. INTERNATIONAL JOURNAL OF EATING DISORDERS. 2009;42(3):195-201.

7630. Ploetner Maria, Moldt Katja, In-Albon Tina, Schmitz Julian Impact of the COVID-19 pandemic on outpatient psychotherapy for children and adolescents. PSYCHOTHERAPIE. 2022;67(6, SI):469-477.

7631. Skypala Isabel, McKenzie Rebecca Nutritional Issues in Food Allergy. CLINICAL REVIEWS IN ALLERGY \& IMMUNOLOGY. 2019;57(2, SI):166-178.

7632. Du G, Mouithys-Mickalad A, Sluse FE Generation of superoxide anion by mitochondria and impairment of their functions during anoxia and reoxygenation in vitro. FREE RADICAL BIOLOGY AND MEDICINE. 1998;25(9):1066-1074.

7633. Finger Mary, Madden Lyndsay, Haq Ihtsham, McLouth Christopher, Siddiqui Mustafa Analysis of the prevalence and onset of dysphonia and dysphagia symptoms in movement disorders at an academic medical center. JOURNAL OF CLINICAL NEUROSCIENCE. 2019;64():111-115.

7634. Nielsen Soren, Anckarsater Henrik, Gillberg Carina, Gillberg Christopher, Rastam Maria, Wentz Elisabet Effects of autism spectrum disorders on outcome in teenage-onset anorexia nervosa evaluated by the Morgan-Russell outcome assessment schedule: a controlled community-based study. MOLECULAR AUTISM. 2015;6():.

7635. Soria Acosta Aline, Rodriguez Plasencia Adisnay, Cabrera Capote Mayrenis, Medina Naranjo Gloria PREVALENCE AND ETIOLOGY OF ACUTE APPENDICITIS IN THE IESS HOSPITAL OF LATACUNGA. REVISTA UNIVERSIDAD Y SOCIEDAD. 2021;13(6):543-547.

7636. Correa Vanessa, Paiva Karina, Besen Eduarda, Silveira Deivid, Gonzales Ana, Moreira Emanuelle, Ferreira Alexsandra, Odila Maestri Miguel Fernanda, Haas Patricia Impact of childhood obesity in brazil: systematic review. RBONE-REVISTA BRASILEIRA DE OBESIDADE NUTRICAO E EMAGRECIMENTO. 2020;14(85):177-183.

7637. King-Casas Brooks, Chiu Pearl Understanding Interpersonal Function in Psychiatric Illness Through Multiplayer Economic Games. BIOLOGICAL PSYCHIATRY. 2012;72(2):119-125.

7638. Liu Wu, Lu Da, Du Xia, Sun Jian, Ge Jun, Wang Ren, Wang Ru, Zou Jun, Xu Chang, Ren Jie, Wen Xin, Liu Yang, Cheng Shu, Tan Xiao, Pekkala Satu, Munukka Eveliina, Wiklund Petri, Chen Yan, Gu Qing, Xia Zheng, Liu Jun, Liu Wen, Chen Xue, Zhang Yi, Li Rui, Borra Ronald, Yao Jia, Chen Pei, Cheng Sulin Effect of aerobic exercise and low carbohydrate diet on pre-diabetic non-alcoholic fatty liver disease in postmenopausal women and middle aged men - the role of gut microbiota composition: study protocol for the AELC randomized controlled trial. BMC PUBLIC HEALTH. 2014;14():.

7639. Ashdown-Franks Garcia, Sabiston Catherine, Stubbs Brendon The evidence for physical activity in the management of major mental illnesses: a concise overview to inform busy clinicians' practice and guide policy. CURRENT OPINION IN PSYCHIATRY. 2019;32(5):375-380.

7640. Hirschberg R, Ding H Mechanisms of insulin-like growth factor-I-induced accelerated recovery in experimental ischemic acute renal failure. MINERAL AND ELECTROLYTE METABOLISM. 1998;24(4):211-219.

7641. Yucel Basak, Uzum Ayse, Ozbey Nese, Kamali Sevil, Yager Joel Anorexia nervosa and Raynaud's phenomenon: A case report. INTERNATIONAL JOURNAL OF EATING DISORDERS. 2007;40(8):762-765.

7642. Subbaiah CC, Bush DS, Sachs MM Mitochondrial contribution to the anoxic Ca2+ signal in maize suspension-cultured cells. PLANT PHYSIOLOGY. 1998;118(3):759-771.

7643. Warren MP, Shantha S The female athlete. BEST PRACTICE \& RESEARCH CLINICAL ENDOCRINOLOGY \& METABOLISM. 2000;14(1):37-53.

7644. Sahni Leila, Boom Julie, Mire Sarah, Berry Leandra, Dowell Lauren, Minard Charles, Cunningham Rachel, Goin-Kochel Robin Vaccine hesitancy and illness perceptions: comparing parents of children with autism spectrum disorder to other parent groups. CHILDRENS HEALTH CARE. 2020;49(4, SI):385-402.

7645. Perez Marisol, Ohrt Tara, Bruening Amanda The effects of different recruitment and incentive strategies for body acceptance programs on college women. EATING DISORDERS. 2016;24(5):383-392.

7646. Norton Lyza, Hart Laura, Butel Francoise, Roberts Shelley Child health nurse perceptions of using confident body, confident child in community health: a qualitative descriptive study. BMC NURSING. 2020;19(1):.

7647. Luo Mengyun, Ding Ding, Bauman Adrian, Negin Joel, Phongsavan Philayrath Social engagement pattern, health behaviors and subjective well-being of older adults: an international perspective using WHO-SAGE survey data. BMC PUBLIC HEALTH. 2020;20(1):.

7648. Zaccardelli Alessandra, Friedlander H., Ford Julia, Sparks Jeffrey Potential of Lifestyle Changes for Reducing the Risk of Developing Rheumatoid Arthritis: Is an Ounce of Prevention Worth a Pound of Cure?. CLINICAL THERAPEUTICS. 2019;41(7):1323-1345.

7649. Johnson James, Laub Donald, John Sujit The effect on health of alternate day calorie restriction: Eating less and more than needed on alternate days prolongs life. MEDICAL HYPOTHESES. 2006;67(2):209-211.

7650. Martinez G., Cook-Darzens S., Chaste P., Mouren M., Doyen C. Anorexia nervosa in the light of neurocognitive functioning: New theoretical and therapeutic perspectives. ENCEPHALE-REVUE DE PSYCHIATRIE CLINIQUE BIOLOGIQUE ET THERAPEUTIQUE. 2014;40(2):160-167.

7651. Papas Eric, Sweeney Deborah Interpreting the corneal response to oxygen: Is there a basis for re-evaluating data from gas-goggle studies?. EXPERIMENTAL EYE RESEARCH. 2016;151():222-226.

7652. Dio Bleichmar Emilce The Fortune of a Precocious Awareness. PSYCHOANALYTIC INQUIRY. 2015;35(2, SI):155-171.

7653. Pierrehumbert B, Nicole A, Muller-Nix C, Forcada-Guex M, Ansermet F Parental post-traumatic reactions after premature birth: implications for sleeping and eating problems in the infant. ARCHIVES OF DISEASE IN CHILDHOOD-FETAL AND NEONATAL EDITION. 2003;88(5):F400-F404.

7654. Aouad Phillip, Bryant Emma, Maloney Danielle, Marks Peta, Le Anvi, Russell Haley, Hay Phillipa, Miskovic-Wheatley Jane, Touyz Stephen, Maguire Sarah, Consortiu Natl Informing the development of Australia's National Eating Disorders Research and Translation Strategy: a rapid review methodology. JOURNAL OF EATING DISORDERS. 2022;10(1):.

7655. Li Baojing, Allebeck Peter, Burstom Bo, Danielsson Anna-Karin, Degenhardt Louisa, Eikemo Terje, Ferrari Alize, Knudsen Ann, Lundin Andreas, Manhica Helio, Newton John, Whiteford Harvey, Flodin Par, Sjoqvist Hugo, Agardh Emilie Educational level and the risk of mental disorders, substance use disorders and self-harm in different age-groups: A cohort study covering 1,6 million subjects in the Stockholm region. INTERNATIONAL JOURNAL OF METHODS IN PSYCHIATRIC RESEARCH. 2023;():.

7656. Jaser Sarah, Yates Heather, Dumser Susan, Whittemore Robin Risky Business Risk Behaviors in Adolescents With Type 1 Diabetes. DIABETES EDUCATOR. 2011;37(6):756-764.

7657. Cimino Silvia, Marzilli Eleonora, Babore Alessandra, Trumello Carmen, Cerniglia Luca DAT1 and Its Psychological Correlates in Children with Avoidant/Restrictive Food Intake Disorder: A Cross-Sectional Pilot Study. BEHAVIORAL SCIENCES. 2021;11(1):.

7658. LIN CY, LEE CS, LIN DY, HONG CF, JAN YY, LIN PY, CHEN PC, WU CS EMPHYSEMATOUS GASTRITIS SECONDARY TO ACUTE GASTRIC DILATATION. JOURNAL OF GASTROENTEROLOGY AND HEPATOLOGY. 1995;10(5):612-615.

7659. Freisling Heinz, Viallon Vivian, Lennon Hannah, Bagnardi Vincenzo, Ricci Cristian, Butterworth Adam, Sweeting Michael, Muller David, Romieu Isabelle, Bazelle Pauline, Kvaskoff Marina, Arveux Patrick, Severi Gianluca, Bamia Christina, Kuehn Tilman, Kaaks Rudolf, Bergmann Manuela, Boeing Heiner, Tjonneland Anne, Olsen Anja, Overvad Kim, Dahm Christina, Menendez Virginia, Agudo Antonio, Sanchez Maria-Jose, Amiano Pilar, Santiuste Carmen, Gurrea Aurelio, Tong Tammy, Schmidt Julie, Tzoulaki Ioanna, Tsilidis

Konstantinos, Ward Heather, Palli Domenico, Agnoli Claudia, Tumino Rosario, Ricceri Fulvio, Panico Salvatore, Picavet H., Bakker Marije, Monninkhof Evelyn, Nilsson Peter, Manjer Jonas, Rolandsson Olov, Thysell Elin, Weiderpass Elisabete, Jenab Mazda, Riboli Elio, Vineis Paolo, Danesh John, Wareham Nick, Gunter Marc, Ferrari Pietro Lifestyle factors and risk of multimorbidity of cancer and cardiometabolic diseases: a multinational cohort study. BMC MEDICINE. 2020;18(1):.

7660. Yamamiya Yuko, Omori Mika How prepartum appearance-related attitudes influence body image and weight-control behaviors of pregnant Japanese women across pregnancy: Latent growth curve modeling analyses. BODY IMAGE. 2023;44():53-63.

7661. Bianciardi Emanuela, Imperatori Claudio, Niolu Cinzia, Campanelli Michela, Franceschilli Marzia, Petagna Lorenzo, Zerbin Francesca, Siracusano Alberto, Gentileschi Paolo Bariatric Surgery Closure During COVID-19 Lockdown in Italy: The Perspective of Waiting List Candidates. FRONTIERS IN PUBLIC HEALTH. 2020;8():.

7662. Schaller T, Moor HC, Wehrli B Sedimentary profiles of Fe, Mn, V, Cr, As and Mo as indicators of benthic redox conditions in Baldeggersee. AQUATIC SCIENCES. 1997;59(4):345-361.

7663. Jacobi F, Wittchen HU, Holting C, Hofler M, Pfister H, Muller N, Lieb R Prevalence, co-morbidity and correlates of mental disorders in the general population: results from the German Health Interview and Examination Survey (GHS). PSYCHOLOGICAL MEDICINE. 2004;34(4):597-611.

7664. KOPP W FREQUENCY OF SEXUAL ABUSE AMONG WOMEN WITH EATING DISORDERS. PSYCHOTHERAPIE PSYCHOSOMATIK MEDIZINISCHE PSYCHOLOGIE. 1994;44(5):159-162.

7665. Austin David, Scharf Roger, Carroll Jason, Enochs Mark Suppression of hypolimnetic methylmercury accumulation by liquid calcium nitrate amendment: redox dynamics and fate of nitrate. LAKE AND RESERVOIR MANAGEMENT. 2016;32(1):61-73.

7666. Stice E A prospective test of the dual-pathway model of bulimic pathology: Mediating effects of dieting and negative affect. JOURNAL OF ABNORMAL PSYCHOLOGY. 2001;110(1):124-135.

7667. Wassmann H, Greiner C, Hulsmann S, Moskopp D, Speckmann EJ, Meyer J, Van Aken H Hypothermia as cerebroprotective measure. Experimental hypoxic exposure of brain slices and clinical application in critically reduced cerebral perfusion pressure. NEUROLOGICAL RESEARCH. 1998;20(1):S61-S65.

7668. Wang H., He H., Miao M., Yu Y., Liu H., Zhang J., Li F., Li J. Maternal migraine and the risk of psychiatric disorders in offspring: a population-based cohort study. EPIDEMIOLOGY AND PSYCHIATRIC SCIENCES. 2021;30():.

7669. Sartoris FJ, Portner HO Increased concentrations of haemolymph Mg2+ protect intracellular pH and ATP levels during temperature stress and anoxia in the common shrimp Crangon crangon. JOURNAL OF EXPERIMENTAL BIOLOGY. 1997;200(4):785-792.

7670. Turgeon Steven, Creaser Robert Cretaceous oceanic anoxic event 2 triggered by a massive magmatic episode. NATURE. 2008;454(7202):323-U29.

7671. Xie Xiufeng, Li Tianchang, Yuan Haifeng Protective effects of Ulinastatin on oxidative stress and inflammation of rat-derived cardiomyocytes H9c2. AMERICAN JOURNAL OF TRANSLATIONAL RESEARCH. 2019;11(11):7094-7103.

7672. Chudzicka-Strugala Izabela, Golebiewska Iwona, Banaszewska Beata, Brudecki Grzegorz, Zwozdziak Barbara The Role of Individually Selected Diets in Obese Women with PCOS-A Review. NUTRIENTS. 2022;14(21):.

7673. Laske C., Eschweiler G. Brain-derived neurotrophic factor. From nerve growth factor to modulator of brain plasticity in cognitive processes and psychiatric diseases. NERVENARZT. 2006;77(5):523+.

7674. Gallouedec G, Sangla S, Jedynak CP, Vidailhet M Dystonias: Secondary dystonias: clinical analysis and diagnostic approach. PRESSE MEDICALE. 1999;28(6):306-311.

7675. Ducat Lee, Rubenstein Arthur, Philipson Louis, Anderson Barbara A Review of the Mental Health Issues of Diabetes Conference. DIABETES CARE. 2015;38(2):333-338.

7676. Krizanac-Bengez L, Kapural M, Parkinson F, Cucullo L, Hossain M, Mayberg MR, Janigro D Effects of transient loss of shear stress on blood-brain barrier endothelium: role of

nitric oxide and IL-6. BRAIN RESEARCH. 2003;977(2):239-246.

7677. Godart NT, Curt F, Perdereau F, Lang F, Venisse JL, Halfon O, Bizouard P, Loas G, Corcos M, Jeammet P, Flament MF Is depressive disorder linked to anxiety disorder among anorexics and bulimics. ENCEPHALE-REVUE DE PSYCHIATRIE CLINIQUE BIOLOGIQUE ET THERAPEUTIQUE. 2005;31(4, 1):403-411.

7678. Malecki Jennifer, Rhodes Paul, Ussher Jane, Boydell Katherine The embodiment of childhood abuse and anorexia nervosa: A body mapping study. HEALTH CARE FOR WOMEN INTERNATIONAL. 2022;():.

7679. Roy R, Kluber HD, Conrad R Early initiation of methane production in anoxic rice soil despite the presence of oxidants. FEMS MICROBIOLOGY ECOLOGY. 1997;24(4):311-320.

7680. Buse Kent, Aftab Wafa, Akhter Sadika, Phuong Linh, Chemli Haroun, Dahal Minakshi, Feroz Anam, Hofiani Sayad, Pradhan Nousheen, Anwar Iqbal, Skhiri Hajer, El Ati Jalila, Giang Kim, Puri Mahesh, Noormal Bashir, Rabbani Fauziah, Hawkes Sarah The state of diet-related NCD policies in Afghanistan, Bangladesh, Nepal, Pakistan, Tunisia and Vietnam: a comparative assessment that introduces a `policy cube' approach. HEALTH POLICY AND PLANNING. 2020;35(5):503-521.

7681. Yager Zali, Diedrichs Phillippa, Ricciardelli Lina, Halliwell Emma What works in secondary schools? A systematic review of classroom-based body image programs. BODY IMAGE. 2013;10(3):271-281.

7682. Solien J, Haynes V, Giulivi C Differential requirements of calcium for oxoglutarate dehydrogenase and mitochondrial nitric-oxide synthase under hypoxia: Impact on the regulation of mitochondrial oxygen consumption. COMPARATIVE BIOCHEMISTRY AND PHYSIOLOGY A-MOLECULAR \& INTEGRATIVE PHYSIOLOGY. 2005;142(2):111-117.

7683. Jambaque I, Hertz-Pannier L, Mikaeloff Y, Martins S, Peudenier S, Dulac O, Chiron C Severe memory impairment in a child with bihippocampal injury after status epilepticus. DEVELOPMENTAL MEDICINE AND CHILD NEUROLOGY. 2006;48(3):223-226.

7684. Battacharyya M Coronary heart disease prevention in Kolkata, India. JOURNAL OF THE ROYAL SOCIETY FOR THE PROMOTION OF HEALTH. 2003;123(4):222-228.

7685. Pokrajac-Bulian A Dissatisfaction with body and bulimic symptoms in the student population. DRUSTVENA ISTRAZIVANJA. 1998;7(4-5):581-601.

7686. Them Theodore, Gill Benjamin, Caruthers Andrew, Gerhardt Angela, Grocke Darren, Lyons Timothy, Marroquin Selva, Nielsen Sune, Alexandre Joao, Owens Jeremy Thallium isotopes reveal protracted anoxia during the Toarcian (Early Jurassic) associated with volcanism, carbon burial, and mass extinction. PROCEEDINGS OF THE NATIONAL ACADEMY OF SCIENCES OF THE UNITED STATES OF AMERICA. 2018;115(26):6596-6601.

7687. Kaviarasi Sathyasivam, Yuba Eiji, Harada Atsushi, Krishnan Uma Emerging paradigms in nanotechnology for imaging and treatment of cerebral ischemia. JOURNAL OF CONTROLLED RELEASE. 2019;300():22-45.

7688. Tylka Tracy, Wood-Barcalow Nichole What is and what is not positive body image? Conceptual foundations and construct definition. BODY IMAGE. 2015;14():118-129.

7689. Eleftheriadis Theodoros, Pissas Georgios, Golfinopoulos Spyridon, Liakopoulos Vassilios, Stefanidis Ioannis Role of indoleamine 2,3-dioxygenase in ischemia-reperfusion injury of renal tubular epithelial cells. MOLECULAR MEDICINE REPORTS. 2021;23(6):.

7690. Hsu KS, Huang CC Protein kinase C inhibitors block generation of anoxia-induced long-term potentiation. NEUROREPORT. 1998;9(15):3525-3529.

7691. Hepp S., Mueller M. Sulfhydryl oxidation: A potential strategy to achieve neuroprotection during severe hypoxia?. NEUROSCIENCE. 2008;152(4):903-912.

7692. Giraldo-Gomez Victor, Petrizzo Maria, Erba Elisabetta, Bottini Cinzia Paleoceanographic inferences from benthic foraminifera across the early Aptian Ocean Anoxic Event 1a in the western Tethys. PALAEOGEOGRAPHY PALAEOCLIMATOLOGY PALAEOECOLOGY. 2022;588():.

7693. Karunasinghe Rashika, Lipski Janusz Oxygen and glucose deprivation (OGD)-induced spreading depression in the Substantia Nigra. BRAIN RESEARCH. 2013;1527():209-221.

7694. Van Hook Jennifer, Altman Claire Competitive Food Sales in Schools and Childhood Obesity: A Longitudinal Study. SOCIOLOGY OF EDUCATION. 2012;85(1):23-39.

7695. McVey Gail, Kirsh Gillian, Maker Dara, Walker Kathryn, Mullane Jennifer, Laliberte Michelle, Ellis-Claypool Janis, Vorderbrugge Judy, Burnett Alison, Cheung Lydia, Banks Laura Promoting positive body image among university students: A collaborative pilot study. BODY IMAGE. 2010;7(3):200-204.

7696. Hayward Joshua, Jacka Felice, Skouteris Helen, Millar Lynne, Strugnell Claudia, Swinburn Boyd, Allender Steven Lifestyle factors and adolescent depressive symptomatology: Associations and effect sizes of diet, physical activity and sedentary behaviour. AUSTRALIAN AND NEW ZEALAND JOURNAL OF PSYCHIATRY. 2016;50(11):1064-1073.

7697. Taleb Sara, Itani Leila Nutrition Literacy among Adolescents and Its Association with Eating Habits and BMI in Tripoli, Lebanon. DISEASES. 2021;9(2):.

7698. Johnson JG, Cohen P, Kotler L, Kasen S, Brook JS Psychiatric disorders associated with risk for the development of eating disorders during adolescence and early adulthood. JOURNAL OF CONSULTING AND CLINICAL PSYCHOLOGY. 2002;70(5):1119-1128.

7699. GRABER JA, BROOKSGUNN J, PAIKOFF RL, WARREN MP PREDICTION OF EATING PROBLEMS - AN 8-YEAR STUDY OF ADOLESCENT GIRLS. DEVELOPMENTAL PSYCHOLOGY. 1994;30(6):823-834.

7700. Kenny Tiff-Annie, Little Matthew, Lemieux Tad, Griffin P., Wesche Sonia, Ota Yoshitaka, Batal Malek, Chan Hing, Lemire Melanie The Retail Food Sector and Indigenous Peoples in High-Income Countries: A Systematic Scoping Review. INTERNATIONAL JOURNAL OF ENVIRONMENTAL RESEARCH AND PUBLIC HEALTH. 2020;17(23):.

7701. Emonds-Alt Barbara, Coosemans Nadine, Gerards Thomas, Remacle Claire, Cardol Pierre Isolation and characterization of mutants corresponding to the MENA, MENB, MENC and MENE enzymatic steps of 5 `-monohydroxyphylloquinone biosynthesis in Chlamydomonas reinhardtii. PLANT JOURNAL. 2017;89(1):141-154.

7702. Chong ZZ, Lin SH, Li FQ, Maiese K The sirtuin inhibitor nicotinamide enhances neuronal cell survival, during acute anoxic injury through AKT, BAD, PARP, and mitochondrial associated ``anti-apoptotic{''} pathways. CURRENT NEUROVASCULAR RESEARCH. 2005;2(4):271-285.

7703. Kramer Rachel, Cuccolo Kelly Yoga Practice in a College Sample: Associated Changes in Eating Disorder, Body Image, and Related Factors Over Time. EATING DISORDERS. 2020;28(4, SI):494-512.

7704. Rokicka Gabriela, Wisniewska Klaudia, Okreglicka Katarzyna Eating habits among women with insulin resistance (IR) on a vegetarian vs non-vegetarian diet. FAMILY MEDICINE AND PRIMARY CARE REVIEW. 2022;24(4):336-340.

7705. Patino Sofia, Rajamohan Srijith, Meaney Kathleen, Coupey Eloise, Serrano Elena, Hedrick Valisa, Gomes Fabio, Polys Nicholas, Kraak Vivica Development of a Responsible Policy Index to Improve Statutory and Self-Regulatory Policies that Protect Children's Diet and Health in the America's Region. INTERNATIONAL JOURNAL OF ENVIRONMENTAL RESEARCH AND PUBLIC HEALTH. 2020;17(2):.

7706. Garcia Frederico, Coquerel Quentin, Rego Jean-Claude, Cravezic Aurore, Bole-Feysot Christine, Kiive Evelyn, Dechelotte Pierre, Harro Jaanus, Fetissov Serguei Anti-neuropeptide Y plasma immunoglobulins in relation to mood and appetite in depressive disorder. PSYCHONEUROENDOCRINOLOGY. 2012;37(9):1457-1467.

7707. Osland Emma, Powlesland Hilary, Guthrie Taylor, Lewis Carrie-Anne, Memon Muhammed Micronutrient management following bariatric surgery: the role of the dietitian in the postoperative period. ANNALS OF TRANSLATIONAL MEDICINE. 2020;8(1):.

7708. Blom Victoria, Lonn Amanda, Ekblom Bjorn, Kallings Lena, Vaisanen Daniel, Hemmingsson Erik, Andersson Gunnar, Wallin Peter, Stenling Andreas, Ekblom Orjan, Lindwall Magnus, Eriksson Jane, Holmlund Tobias, Ekblom-Bak Elin Lifestyle Habits and Mental Health in Light of the Two COVID-19 Pandemic Waves in Sweden, 2020. INTERNATIONAL JOURNAL OF ENVIRONMENTAL RESEARCH AND PUBLIC HEALTH. 2021;18(6):.

7709. Stice Eric, Marti C., Cheng Zhen Effectiveness of a dissonance-based eating disorder prevention program for ethnic groups in two randomized controlled trials. BEHAVIOUR RESEARCH AND THERAPY. 2014;55():54-64.

7710. Lu Yangbo, Shen Jun, Wang Yuxuan, Lu Yongchao, Algeo Thomas, Jiang Shu, Yan Detian, Gou Qiyang Seawater sources of Hg enrichment in Ordovician-Silurian boundary strata, South China. PALAEOGEOGRAPHY PALAEOCLIMATOLOGY PALAEOECOLOGY. 2022;601():.

7711. Vetharaniam I., Thomson R., Devine C., Daly C. Modelling muscle energy-metabolism in anaerobic muscle. MEAT SCIENCE. 2010;85(1):134-148.

7712. Tong Zhihong, Xie Yongyan, He Ming, Ma Wen, Zhou Yue, Lai Songqing, Meng Yan, Liao Zhangping VDAC1 deacetylation is involved in the protective effects of resveratrol against mitochondria-mediated apoptosis in cardiomyocytes subjected to anoxia/reoxygenation injury. BIOMEDICINE \& PHARMACOTHERAPY. 2017;95():77-83.

7713. Rogalska J., Caputa M., Wentowska K., Nowakowska A. STRESS-INDUCED BEHAVIOUR IN ADULT AND OLD RATS: EFFECTS OF NEONATAL ASPHYXIA, BODY TEMPERATURE AND CHELATION OF IRON. JOURNAL OF PHYSIOLOGY AND PHARMACOLOGY. 2006;57(8):17-34.

7714. Zhang XC, Shan PY, Alam J, Fu XY, Lee PJ Carbon monoxide differentially modulates STAT1 and STAT3 and inhibits apoptosis via a phosphatidylinositol 3-kinase/Akt and p38 kinase-dependent STAT3 pathway during anoxia-reoxygenation injury. JOURNAL OF BIOLOGICAL CHEMISTRY. 2005;280(10):8714-8721.

7715. Fulkerson Jayne, Kubik Martha, Story Mary, Lytle Leslie, Arcan Chrisa Are There Nutritional and Other Benefits Associated with Family Meals Among At-Risk Youth?. JOURNAL OF ADOLESCENT HEALTH. 2009;45(4):389-395.

7716. Diaz-Mendez Cecilia, Otero-Estevez Sonia, Sanchez-Sanchez Sandra Are Spanish Surveys Ready to Detect the Social Factors of Obesity?. INTERNATIONAL JOURNAL OF ENVIRONMENTAL RESEARCH AND PUBLIC HEALTH. 2022;19(18):.

7717. Cislak A., Safron M., Pratt M., Gaspar T., Luszczynska A. Family-related predictors of body weight and weight-related behaviours among children and adolescents: a systematic umbrella review. CHILD CARE HEALTH AND DEVELOPMENT. 2012;38(3):321-331.

7718. Rodgers Rachel, Ziff Sara, Lowy Alice, Austin S. Stakeholder attitudes towards fashion policy in the US: strategic research for the protection of models and prevention of body image and eating concerns. CRITICAL PUBLIC HEALTH. 2022;32(3):413-421.

7719. Kahl Kai, Deuschle Michael, Stubbs Brendon, Schweiger Ulrich Visceral adipose tissue in patients with severe mental illness. HORMONE MOLECULAR BIOLOGY AND CLINICAL INVESTIGATION. 2018;33(1, SI):.

7720. Carroll ST, Riffenburgh RH, Roberts TA, Myhre EB Tattoos and body piercings as indicators of adolescent risk-taking behaviors. PEDIATRICS. 2002;109(6):1021-1027.

7721. DIFIORI JP MENSTRUAL DYSFUNCTION IN ATHLETES - HOW TO IDENTIFY AND TREAT PATIENTS AT RISK FOR SKELETAL INJURY. POSTGRADUATE MEDICINE. 1995;97(3):143-\&.

7722. Greif Rebecca, Becker Carolyn, Hildebrandt Tom Reducing Eating Disorder Risk Factors: A Pilot Effectiveness Trial of a Train-the-Trainer Approach to Dissemination and Implementation. INTERNATIONAL JOURNAL OF EATING DISORDERS. 2015;48(8):1122-1131.

7723. Kokura S, Wolf RE, Yoshikawa T, Granger DN, Aw TY T-lymphocyte-derived tumor necrosis factor exacerbates anoxia-reoxygenation-induced neutrophil-endothelial cell adhesion. CIRCULATION RESEARCH. 2000;86(2):205-213.

7724. Fontaine Guillaume, Cossette Sylvie A theory-based adaptive E-learning program aimed at increasing intentions to provide brief behavior change counseling: Randomized controlled trial. NURSE EDUCATION TODAY. 2021;107():.

7725. Berle P, Kogel M Incidence, maternal and infantile morbidity of emergency Caesarean section in a perinatal centre (An analysis 1990-1998). GEBURTSHILFE UND FRAUENHEILKUNDE. 1999;59(9):465-469.

7726. Franko Debra, Rodgers Rachel, Lovering Meghan, Fernandes Caroline, Alfieri Alyssa, Matsumoto Atsushi, Accomando Kristin, Thompson-Brenner Heather Time Trends in Cover Images and Article Content in Latina Magazine: Potential Implications for Body Dissatisfaction in Latina Women. JOURNAL OF LATINA-O PSYCHOLOGY. 2013;1(4):243-254.

7727. Bajerska Joanna, Chmurzynska Agata, Muzsik-Kazimierska Agata, Madry Edyta, Pieta Beata, Sobkowski Maciej, Walkowiak Jaroslaw Determinants favoring weight regain after weight-loss therapy among postmenopausal women. SCIENTIFIC REPORTS. 2020;10(1):.

7728. Leone James, Mullin Elizabeth, Maurer-Starks Suanne, Rovito Michael THE ADOLESCENT BODY IMAGE SATISFACTION SCALE FOR MALES: EXPLORATORY FACTOR ANALYSIS AND IMPLICATIONS FOR STRENGTH AND CONDITIONING PROFESSIONALS.

JOURNAL OF STRENGTH AND CONDITIONING RESEARCH. 2014;28(9):2657-2668.

7729. Sharifuddin Yusrizam, Chin Yao-Xian, Lim Phaik-Eem, Phang Siew-Moi Potential Bioactive Compounds from Seaweed for Diabetes Management. MARINE DRUGS. 2015;13(8):5447-5491.

7730. Trico Domenico, Biancalana Edoardo, Solini Anna Protein and amino acids in nonalcoholic fatty liver disease. CURRENT OPINION IN CLINICAL NUTRITION AND METABOLIC CARE. 2021;24(1):96-101.

7731. Maatoug Jihene, Harrabi Imed, Hmad Sonia, Belkacem Mylene, al'Absi Mustafa, Lando Harry, Ghannem Hassen Clustering of Risk Factors With Smoking Habits Among Adults, Sousse, Tunisia. PREVENTING CHRONIC DISEASE. 2013;10():.

7732. Maximova Katerina, Khan Mohammad, Dabravolskaj Julia, Maunula Laena, Ohinmaa Arto, Veugelers Paul Perceived changes in lifestyle behaviours and in mental health and wellbeing of elementary school children during the first COVID-19 lockdown in Canada. PUBLIC HEALTH. 2022;202():35-42.

7733. Naseri Parisa, Majd Hamid, Tabatabaei Seyyed Cognitive regulation of food intake associated with weight management and obesity prevention. ACTIVITAS NERVOSA SUPERIOR REDIVIVA. 2019;61(3-4):85-90.

7734. Ling Jiying, King Kristi, Speck Barbara, Kim Seongho, Wu Dongfeng Preliminary Assessment of a School-Based Healthy Lifestyle Intervention Among Rural Elementary School Children. JOURNAL OF SCHOOL HEALTH. 2014;84(4):247-255.

7735. Theanjumpol Parichat, Wongzeewasakun Kumpon, Muenmanee Nadthawat, Wongsaipun Sakunna, Krongchai Chanida, Changrue Viboon, Boonyakiat Danai, Kittiwachana Sila Non-destructive identification and estimation of granulation in `Sai Num Pung' tangerine fruit using near infrared spectroscopy and chemometrics. POSTHARVEST BIOLOGY AND TECHNOLOGY. 2019;153():13-20.

7736. Moyad Mark, Lowe Franklin Educating patients about lifestyle modifications for prostate health. AMERICAN JOURNAL OF MEDICINE. 2008;121(8, 2):S34-S42.

7737. Landheim AS, Bakken K, Vaglum P What characterizes substance abusers who commit suicide attempts? Factors related to Axis I disorders and patterns of substance use disorders - A study of treatment-seeking substance abusers in Norway. EUROPEAN ADDICTION RESEARCH. 2006;12(2):102-108.

7738. Sadeghi Masoumeh, Talaei Mohammad, Parvaresh Rizi Ehsan, Dianatkhah Minoo, Oveisgharan Shahram, Sarrafzadegan Nizal Determinants of incident prediabetes and type 2 diabetes in a 7-year cohort in a developing country: The Isfahan Cohort Study. JOURNAL OF DIABETES. 2015;7(5):633-641.

7739. Lablanche Sandrine, Cottet-Rousselle Cecile, Argaud Laurent, Laporte Camille, Lamarche Frederic, Richard Marie-Jeanne, Berney Thierry, Benhamou Pierre-Yves, Fontaine Eric Respective effects of oxygen and energy substrate deprivation on beta cell viability. BIOCHIMICA ET BIOPHYSICA ACTA-BIOENERGETICS. 2015;1847(6-7):629-639.

7740. Akaike N Time-dependent rundown of GABA response in mammalian CNS neuron during experimental anoxia. OBESITY RESEARCH. 1995;3(5):S769-S777.

7741. Khatoon Fahmida, Mahmood Madiha, Obeidat Sofian, Alsatti Munirah, Alanazi Reema, Abu Kabbos Razan, Balouch Zahid, Obelanga Shimaa Assessment of Perception, Awareness about Predictors of Colorectal Cancer of Hail Region Saudi Arabia population. JOURNAL OF PHARMACEUTICAL RESEARCH INTERNATIONAL. 2020;32(47):14-24.

7742. Nishida-Hikiji Eri, Okamoto Miki, Iwanaga Ryoichiro, Nakane Hideyuki, Tanaka Goro Mental health literacy regarding eating disorders in female Japanese university students. EATING DISORDERS. 2021;29(1):17-28.

7743. Xu Tao, Liu Junting, Zhu Guangjin, Han Shaomei Prevalence and Associated Lifestyle Factors of Suboptimal Health Status among Chinese Children Using a Multi-Level Model. INTERNATIONAL JOURNAL OF ENVIRONMENTAL RESEARCH AND PUBLIC HEALTH. 2020;17(5):.

7744. Wakasugi Minako, Kazama Junichiro, Narita Ichiei, Iseki Kunitoshi, Moriyama Toshiki, Yamagata Kunihiro, Fujimoto Shouichi, Tsuruya Kazuhiko, Asahi Koichi, Konta Tsuneo, Kimura Kenjiro, Kondo Masahide, Kurahashi Issei, Ohashi Yasuo, Watanabe Tsuyoshi Association between Combined Lifestyle Factors and Non-Restorative Sleep in Japan: A Cross-Sectional Study Based on a Japanese Health Database. PLOS ONE. 2014;9(9):.

7745. MUSLEH W, BRUCE A, MALFROY B, BAUDRY M EFFECTS OF EUK-8, A SYNTHETIC CATALYTIC SUPEROXIDE SCAVENGER, ON HYPOXIA-INDUCED AND ACIDOSIS-INDUCED DAMAGE IN HIPPOCAMPAL SLICES. NEUROPHARMACOLOGY. 1994;33(7):929-934.

7746. Norman Asa, Zeebari Zangin, Nyberg Gisela, Elinder Liselotte Parental support in promoting children's health behaviours and preventing overweight and obesity - a long-term follow-up of the cluster-randomised healthy school start study II trial. BMC PEDIATRICS. 2019;19():.

7747. Brewerton Timothy Mechanisms by which adverse childhood experiences, other traumas and PTSD influence the health and well-being of individuals with eating disorders throughout the life span. JOURNAL OF EATING DISORDERS. 2022;10(1):.

7748. Meyer K., Ridgwell A., Payne J. The influence of the biological pump on ocean chemistry: implications for long-term trends in marine redox chemistry, the global carbon cycle, and marine animal ecosystems. GEOBIOLOGY. 2016;14(3):207-219.

7749. Stulac Sara, Bair-Merritt Megan, Wachman Elisha, Augustyn Marilyn, Howard Carey, Madoor Namrata, Costello Eileen Children and families of the opioid epidemic: Under the radar. CURRENT PROBLEMS IN PEDIATRIC AND ADOLESCENT HEALTH CARE. 2019;49(8):.

7750. Kroeller Katja, Warschburger Petra Maternal feeding strategies and child's food intake: considering weight and demographic influences using structural equation modeling. INTERNATIONAL JOURNAL OF BEHAVIORAL NUTRITION AND PHYSICAL ACTIVITY. 2009;6():.

7751. Bardone AM, Vohs KD, Abramson LY, Heatherton TF, Joiner TE The confluence of perfectionism, body dissatisfaction, and low self-esteem predicts bulimic symptoms: Clinical implications. BEHAVIOR THERAPY. 2000;31(2):265-280.

7752. Kang EunKyo, Kim Soojeong, Yun Young Comparison of eating habits and inappropriate weight control efforts of secondary school students enrolled in a weight control program. NUTRITION RESEARCH AND PRACTICE. 2021;15(5):628-638.

7753. Yoshimoto Takahiko, Ochiai Hirotaka, Shirasawa Takako, Nagahama Satsue, Uehara Akihito, Muramatsu Jun, Kokaze Akatsuki Clustering of Lifestyle Factors and Its Association with Low Back Pain: A Cross-Sectional Study of Over 400,000 Japanese Adults. JOURNAL OF

PAIN RESEARCH. 2020;13():1411-1419.

7754. Cathelain Sarah, Brunault Paul, Ballon Nicolas, Reveillere Christian, Courtois Robert Food addiction: Definition, measurement and limits of the concept, associated factors, therapeutic and clinical implications. PRESSE MEDICALE. 2016;45(12, 1):1154-1163.

7755. Kim DongHee, Jang SooCheong Stress and food choices: Examining gender differences and the time horizon framing effect. INTERNATIONAL JOURNAL OF HOSPITALITY MANAGEMENT. 2017;67():134-142.

7756. Cassar M, Jones MG, Szatkowski M Reduced adenosine uptake accelerates ischaemic block of population spikes in hippocampal slices from streptozotocin-treated diabetic rats. EUROPEAN JOURNAL OF NEUROSCIENCE. 1998;10(1):239-245.

7757. Widjaja Gunawan, Sijabat Hotmaria Study of e-Health nutritional interventions on disease patients based on meta-analysis. FOOD SCIENCE AND TECHNOLOGY. 2022;42():.

7758. Sinclair Dawn, Savage Eileen, O'Brien Maria, O'Reilly Anthony, Mullaney Carmel, Killeen Marie, OReilly Orlaith, Field Catherine, Fitzpatrick Patricia, Murrin Celine, Connolly Deirdre, Patterson Aileen, Denieffe Suzanne, Elmusharaf Khalifa, Hickey Anne, Mellon Lisa, Flood Michelle, Sweeney Mary Developing a national undergraduate standardized curriculum for future healthcare professionals on ``Making Every Contact Count{''} for chronic disease prevention in the Republic of Ireland. JOURNAL OF INTERPROFESSIONAL CARE. 2020;34(4):561-565.

7759. Deter HC, Shellberg D, Kopp W, Friederich HC, Herzog W Predictability of a favorable outcome in anorexia nervosa. EUROPEAN PSYCHIATRY. 2005;20(2):165-172.

7760. Keerthana Sai, Priya Vishnu, Gayatri R. Awareness on Weight Loss with Alternative Medicines Among College Students. JOURNAL OF RESEARCH IN MEDICAL AND DENTAL SCIENCE. 2020;8(7):435-440.

7761. Emeis KC, Neumann T, Endler R, Struck U, Kunzendorf H, Christiansen C Geochemical records of sediments in the Eastern Gotland Basin - products of sediment dynamics in a not-so-stagnant anoxic basin?. APPLIED GEOCHEMISTRY. 1998;13(3):349-358.

7762. Visted Endre, Vollestad Jon, Nielsen Morten, Schanche Elisabeth Emotion Regulation in Current and Remitted Depression: A Systematic Review and Meta-Anayss. FRONTIERS IN PSYCHOLOGY. 2018;9():.

7763. Huang Gang, Yan Juan, Zou Jiahua, Hu Chuxiang, Huang Dongbei, Huang Qiang, Chen Peize, Zhang Feiyan, Gong Liping Fire needle therapy for blood stasis syndrome of plaque psoriasis A protocol for systematic review and meta-analysis. MEDICINE. 2021;100(13):.

7764. Duan Kui, Wu Yufang Meta-Analysis of the Relationship between the Prognosis of Acute Cerebral Infarction Intravenous Lysis and Cerebral Microbleeds Based on Intelligent Medical Care. CONTRAST MEDIA \& MOLECULAR IMAGING. 2022;2022():.

7765. Su Rong-cheng, Huang Li-hong, Li Jia, Zhou Bo, Zhao Jia-jun, Li Hui The Sichuan Mental Health Survey: Methodology. FRONTIERS IN PSYCHIATRY. 2021;12():.

7766. Serra Monica, Marongiu Fabio, Laconi Ezio Long-term moderate caloric restriction and social isolation synergize to induce anorexia-like behavior in rats. NUTRITION. 2021;86():.

7767. Howard Louise, Oram Sian, Galley Helen, Trevillion Kylee, Feder Gene Domestic Violence and Perinatal Mental Disorders: A Systematic Review and Meta-Analysis. PLOS MEDICINE. 2013;10(5):.

7768. Iahtisham-Ul-Haq, Khan Sipper, Awan Kanza, Iqbal Muhammad Sulforaphane as a potential remedy against cancer: Comprehensive mechanistic review. JOURNAL OF FOOD BIOCHEMISTRY. 2022;46(3, SI):.

7769. Dimitriou Lygeri, Weiler Richard, Lloyd-Smith Rebecca, Turner Antony, Heath Luke, James Nic, Reid Anna Bone mineral density, rib pain and other features of the female athlete triad in elite lightweight rowers. BMJ OPEN. 2014;4(2):.

7770. Chung Winston, Jiang Sheng-Fang, Paksarian Diana, Nikolaidis Aki, Castellanos F., Merikangas Kathleen, Milham Michael Trends in the Prevalence and Incidence of Attention-Deficit/Hyperactivity Disorder Among Adults and Children of Different Racial and Ethnic Groups. JAMA NETWORK OPEN. 2019;2(11):.

7771. Coutelle Romain, Boedec Morgane, Vermeulen Karlijn, Kummeling Joost, Koolen David, Kleefstra Tjitske, Fournier Camille, Colin Florent, Strehle Axelle, Genevieve David, Burger Pauline, Mandel Jean-Louis The impact of lockdown on young people with genetic neurodevelopmental disabilities: a study with the international participatory database GenIDA. BMC PSYCHIATRY. 2022;22(1):.

7772. Ram PC, Singh BB, Singh AK, Ram P, Singh PN, Singh HP, Boamfa I, Harren F, Santosa E, Jackson MB, Setter TL, Reuss J, Wade LJ, Singh VP, Singh RK Submergence tolerance in rainfed lowland rice: physiological basis and prospects for cultivar improvement through marker-aided breeding. FIELD CROPS RESEARCH. 2002;76(2-3, SI):131-152.

7773. Hinney A, Schneider J, Ziegler A, Lehmkuhl G, Poustka F, Schmidt MH, Mayer H, Siegfried W, Remschmidt H, Hebebrand J No evidence for involvement of polymorphisms of the dopamine D4 receptor gene in anorexia nervosa, underweight, and obesity. AMERICAN JOURNAL OF MEDICAL GENETICS. 1999;88(6):594-597.

7774. Zatti Alberto, Zarbo Cristina Embodied and exbodied mind in clinical psychology. A proposal for a psycho-social interpretation of mental disorders. FRONTIERS IN PSYCHOLOGY. 2015;6():.

7775. JUDD LL SOCIAL PHOBIA - A CLINICAL OVERVIEW. JOURNAL OF CLINICAL PSYCHIATRY. 1994;55(S):5-9.

7776. Martini Maria, Taborelli Emma, Easter Abigail, Bye Amanda, Eisler Ivan, Schmidt Ulrike, Micali Nadia Effect of maternal eating disorders on mother-infant quality of interaction, bonding and child temperament: A longitudinal study. EUROPEAN EATING DISORDERS REVIEW. 2023;31(2):335-348.

7777. Visweswaraiah Naveen, Nathan Kousalya Adolescent Obesity and Eating Disorders: Can Calorie Restriction have a Positive Impact. CURRENT NUTRITION \& FOOD SCIENCE. 2020;16(4):433-443.

7778. Hara J, Yanagisawa Y, Sakurai T Difference in obesity phenotype between orexin-knockout mice and orexin neuron-deficient mice with same genetic background and environmental conditions. NEUROSCIENCE LETTERS. 2005;380(3):239-242.

7779. Di Marzo Vincenzo The endocannabinoidome as a substrate for noneuphoric phytocannabinoid action and gut microbiome dysfunction in neuropsychiatric disorders.

DIALOGUES IN CLINICAL NEUROSCIENCE. 2020;22(3):259-269.

7780. Tenforde Adam, DeLuca Stephanie, Wu Alexander, Ackerman Kathryn, Lewis Margo, Rauh Mitchell, Heiderscheit Bryan, Krabak Brian, Kraus Emily, Roberts William, Troy Karen, Barrack Michelle Prevalence and factors associated with bone stress injury in middle school runners. PM\&R. 2022;14(9):1056-1067.

7781. Michas George, Karvelas George, Trikas Athanasios Cardiovascular disease in Greece; the latest evidence on risk factors. HELLENIC JOURNAL OF CARDIOLOGY. 2019;60(5):271-275.

7782. Hudson Nicholas Mitochondrial treason: a driver of pH decline rate in post-mortem muscle?. ANIMAL PRODUCTION SCIENCE. 2012;52(12):1107-1110.

7783. Righini NC, Narring F, Navarro C, Perret-Catipovic M, Ladame F, Jeannin A, Berchtold A, Michaud PA Antecedents, psychiatric characteristics and follow-up of adolescents hospitalized for suicide attempt or overwhelming suicidal ideation. SWISS MEDICAL WEEKLY. 2005;135(29-30):440-447.

7784. Wu Joe, Chen Ping, Li Ying, Ardell Chris, Der Tatyana, Shohet Ralph, Chen Minghua, Wright Gary HIF-1 alpha in heart: Protective mechanisms. AMERICAN JOURNAL OF PHYSIOLOGY-HEART AND CIRCULATORY PHYSIOLOGY. 2013;305(6):H821-H828.

7785. Kenny Bridget, Bowe Steven, Taylor C., Moodie Marj, Brown Vicki, Hoban Elizabeth, Williams Joanne Longitudinal relationships between sub-clinical depression, sub-clinical eating disorders and health-related quality of life in early adolescence. INTERNATIONAL JOURNAL OF EATING DISORDERS. 2023;56(6):1114-1124.

7786. Micali Nadia, Simonoff Emily, Stahl Daniel, Treasure Janet Maternal eating disorders and infant feeding difficulties: maternal and child mediators in a longitudinal general population study. JOURNAL OF CHILD PSYCHOLOGY AND PSYCHIATRY. 2011;52(7):800-807.

7787. Wen Xia-Hong, Li Yan, Han Dong, Sun Li, Ren Ping-Xiao, Ren Dan The relationship between cognitive function and arterial partial pressure O-2 in patients with COPD A meta-analysis. MEDICINE. 2018;97(4):.

7788. Zeng Ya-Wen, Yang Jia-Zheng, Pu Xiao-Ying, Du Juan, Yang Tao, Yang Shu-Ming, Zhu Wei-Hua Strategies of Functional Food for Cancer Prevention in Human Beings. ASIAN PACIFIC JOURNAL OF CANCER PREVENTION. 2013;14(3):1585-1592.

7789. Santo Karla Can Digital Health Solutions Fill in the Gap for Effective Guideline Implementation in Cardiovascular Disease Prevention: Hope or Hype?. CURRENT ATHEROSCLEROSIS REPORTS. 2022;24(9):747-754.

7790. HIMORI N, TANAKA Y, KURASAWA M, MISHIMA K, AKAIKE N, IMAI M, UENO K, MATSUKURA T, WATANABE H DEXTRORPHAN ATTENUATES THE BEHAVIORAL CONSEQUENCES OF ISCHEMIA AND THE BIOCHEMICAL CONSEQUENCES OF ANOXIA - POSSIBLE ROLE OF N-METHYL-D-ASPARTATE RECEPTOR ANTAGONISM AND ATP REPLENISHING ACTION IN ITS CEREBROPROTECTING PROFILE. PSYCHOPHARMACOLOGY. 1993;111(2):153-162.

7791. Situju Sulfayanti, Takimoto Hironori, Sato Suzuka, Yamauchi Hitoshi, Kanagawa Akihiro, Lawi Armin Food Constituent Estimation for Lifestyle Disease Prevention by Multi-Task CNN. APPLIED ARTIFICIAL INTELLIGENCE. 2019;33(8):732-746.

7792. Lascar R., Letranchant A., Hirot F., Godart N. What factors explain the length of hospitalization for anorexia nervosa: A systematic review. ENCEPHALE-REVUE DE PSYCHIATRIE CLINIQUE BIOLOGIQUE ET THERAPEUTIQUE. 2021;47(4):362-368.

7793. Yang Chunxue, Kong Alice, Cai Zongwei, Chung Arthur Persistent Organic Pollutants as Risk Factors for Obesity and Diabetes. CURRENT DIABETES REPORTS. 2017;17(12):.

7794. Jahrami Haitham, Sater Mai, Abdulla Ahmed, Faris Mo'ez, AlAnsari Ahmed Eating disorders risk among medical students: a global systematic review and meta-analysis. EATING AND WEIGHT DISORDERS-STUDIES ON ANOREXIA BULIMIA AND OBESITY. 2019;24(3):397-410.

7795. Alavi-Naeini Amirmansour, Bagheri Mahtab, Mirzaei Khadijeh, Maljaei Mohammad, Yekaninejad Mir, Yazdani Alireza Relationship between dietary patterns and mild cognitive impairment (MCI) in elderly women. PROGRESS IN NUTRITION. 2019;21(1):270-280.

7796. Lee Yong, Le Long, Stockings Emily, Hay Phillipa, Whiteford Harvey, Barendregt Jan, Mihalopoulos Cathrine Estimation of a Relative Risk Effect Size when Using Continuous Outcomes Data: An Application of Methods in the Prevention of Major Depression and

Eating Disorders. MEDICAL DECISION MAKING. 2018;38(7):866-880.

7797. Lee Gi, Park Eun, Kim Youl-Ri, Kwag Kyung, Park Jin, An So, Lee Ji, Sim Jeong, Treasure Janet Feasibility and acceptability of a prevention program for eating disorders (Me, You and Us) adapted for young adolescents in Korea. EATING AND WEIGHT DISORDERS-STUDIES ON ANOREXIA BULIMIA AND OBESITY. 2018;23(5):673-683.

7798. Ford Earl, Bergmann Manuela, Kroeger Janine, Schienkiewitz Anja, Weikert Cornelia, Boeing Heiner Healthy Living Is the Best Revenge Findings From the European Prospective Investigation Into Cancer and Nutrition-Potsdam Study. ARCHIVES OF INTERNAL MEDICINE. 2009;169(15):1355-1362.

7799. Ghaddar Rola, Chartrand Jessica, Benomar Anass, Jamoulle Olivier, Taddeo Danielle, Frappier Jean-Yves, Stheneur Chantal Excessive laboratory monitoring to prevent adolescent's refeeding syndrome: opportunities for enhancement. EATING AND WEIGHT DISORDERS-STUDIES ON ANOREXIA BULIMIA AND OBESITY. 2020;25(4):1021-1027.

7800. Kaoser Ridhwana, Jones Wayne, Dove Naomi, Tallon Corinne, Small Will, Vigo Daniel, Samji Hasina Using novel methodology to estimate the prevalence of mental disorders in British Columbia, Canada. SOCIAL PSYCHIATRY AND PSYCHIATRIC EPIDEMIOLOGY. 2023;58(1):153-162.

7801. Hayden-Wade HA, Stein RI, Ghaderi A, Saelens BE, Zabinski MF, Wilfley DE Prevalence, characteristics, and correlates of teasing experiences among overweight children vs. non-overweight peers. OBESITY RESEARCH. 2005;13(8):1381-1392.

7802. Ortiz Shelby, Espel-Huynh Hallie, Felonis Christina, Scharff Adela Qualitative perceptions of and preferences for the research process among patients with eating disorders. INTERNATIONAL JOURNAL OF EATING DISORDERS. 2020;53(1):41-51.

7803. Yang Wanwei, Cao Meng, Mao Xiaodong, Wei Xiao, Li Xingjia, Chen Guofang, Zhang Jiaming, Wang Zhiguo, Shi Jianfeng, Huang HouCai, Yao Xiaoming, Liu Chao Alternate-day fasting protects the livers of mice against high-fat diet-induced inflammation associated with the suppression of Toll-like receptor 4/nuclear factor kappa B signaling. NUTRITION RESEARCH. 2016;36(6):586-593.

7804. Lane RM A critical review of selective serotonin reuptake inhibitor-related sexual dysfunction; Incidence, possible aetiology and implications for management. JOURNAL OF

PSYCHOPHARMACOLOGY. 1997;11(1):72-82.

7805. Li Xiumin, Xue Yu, Pang Liang, ShangGuan Zhaoshui, Pan Yutian Lysimachia Capillipes Inhibit Adipogenesis via Angiogenesis Inhibition. DRUG RESEARCH. 2019;69(5):284-290.

7806. Bowyer Susan, Shvarts Vladimir, Moran John, Mason Karen, Barkley Gregory, Tepley Norman Slow Brain Activity (ISA/DC) Detected by MEG. JOURNAL OF CLINICAL NEUROPHYSIOLOGY. 2012;29(4):320-326.

7807. Greenfield Shelly, Rosa Carmen, Putnins Susan, Green Carla, Brooks Audrey, Calsyn Donald, Cohen Lisa, Erickson Sarah, Gordon Susan, Haynes Louise, Killeen Therese, Miele Gloria, Tross Susan, Winhusen Theresa Gender Research in the National Institute on Drug Abuse National Treatment Clinical Trials Network: A Summary of Findings. AMERICAN JOURNAL OF DRUG AND ALCOHOL ABUSE. 2011;37(5):301-312.

7808. Becker Inga, Auer Matthias, Barkmann Claus, Fuss Johannes, Moeller Birgit, Nieder Timo, Fahrenkrug Saskia, Hildebrandt Thomas, Richter-Appelt Hertha A Cross-Sectional Multicenter Study of Multidimensional Body Image in Adolescents and Adults with Gender Dysphoria Before and After Transition-Related Medical Interventions. ARCHIVES OF SEXUAL BEHAVIOR. 2018;47(8):2335-2347.

7809. Li Bingda, Chen Tianpeng, Hu Wenfeng, Wang Zhenhua, Wu Ji, Zhou Qing, Li Ping Poncirin ameliorates cardiac ischemia-reperfusion injury by activating PI3K/AKT/PGC-1 alpha signaling. EUROPEAN JOURNAL OF PHARMACOLOGY. 2022;917():.

7810. Matsumori Akira Targeting Inflammation in the Diagnosis, Management, and Prevention of Cardiovascular Diseases. GLOBAL HEART. 2022;17(1):.

7811. Dahl T., Connelly J., Kouchinsky A., Gill B., Mansson S., Bizzarro M. Reorganisation of Earth's biogeochemical cycles briefly oxygenated the oceans 520 Myr ago. GEOCHEMICAL PERSPECTIVES LETTERS. 2017;3(2):210-220.

7812. Robinson Stuart, Murphy Daniel, Vance Derek, Thomas Deborah Formation of ``Southern Component Water{''} in the Late Cretaceous: Evidence from Nd-isotopes. GEOLOGY. 2010;38(10):871-874.

7813. Pina Alessandra, Castelletti Silvia COVID-19 and Cardiovascular Disease: a Global Perspective. CURRENT CARDIOLOGY REPORTS. 2021;23(10):.

7814. Zhang Yunfang, Shi Junchao, Rassoulzadegan Minoo, Tuorto Francesca, Chen Qi Sperm RNA code programmes the metabolic health of offspring. NATURE REVIEWS ENDOCRINOLOGY. 2019;15(8):489-498.

7815. Pimenta Ferreira Claudia, Sforza Chiarella, Rusconi Francesca, Castelo Paula, Bommarito Silvana Masticatory behaviour and chewing difficulties in young adults with temporomandibular disorders. JOURNAL OF ORAL REHABILITATION. 2019;46(6):533-540.

7816. Iglesias Dario, Cremonini Eleonora, Hester Shelly, Wood Steven, Bartlett Mark, Fraga Cesar, Oteiza Patricia Cyanidin and delphinidin restore colon physiology in high fat diet-fed mice: Involvement of TLR-4 and redox-regulated signaling. FREE RADICAL BIOLOGY AND MEDICINE. 2022;188():71-82.

7817. Wilde Parke, Conrad Zach, Rehm Colin, Pomeranz Jennifer, Penalvo Jose, Cudhea Frederick, Pearson-Stuttard Jonathan, O'Flaherty Martin, Micha Renata, Mozaffarian Dariush Reductions in national cardiometabolic mortality achievable by food price changes according to Supplemental Nutrition Assistance Program (SNAP) eligibility and participation. JOURNAL OF EPIDEMIOLOGY AND COMMUNITY HEALTH. 2018;72(9):817-824.

7818. Hawley Nicola, Suss Rachel, Cash Haley, Aitaoto Nia, Samoa Raynald, Ayers Britni, McElfish Pearl Diabetes Prevention and Care Programs in the US-Affiliated Pacific Islands: Challenges, Innovation, and Recommendations for Effective Scale-Up. CURRENT DIABETES REPORTS. 2019;19(5):.

7819. Romani-Perez Marina, Bullich-Vilarrubias Clara, Lopez-Almela Inmaculada, Liebana-Garcia Rebeca, Olivares Marta, Sanz Yolanda The Microbiota and the Gut-Brain Axis in Controlling Food Intake and Energy Homeostasis. INTERNATIONAL JOURNAL OF MOLECULAR SCIENCES. 2021;22(11):.

7820. Jaca A., Durao S., Harbron J. Omega-3 fatty acids for the primary and secondary prevention of cardiovascular disease. SAMJ SOUTH AFRICAN MEDICAL JOURNAL. 2020;110(12):1158-1159.

7821. Wang Yanan, Liu Qing, Quan Helong, Kang Seong-Gook, Huang Kunlun, Tong Tao Nutraceuticals in the Prevention and Treatment of the Muscle Atrophy. NUTRIENTS. 2021;13(6):.

7822. Deepa M., Anjana R., Mohan V. Role of lifestyle factors in the epidemic of diabetes: lessons learnt from India. EUROPEAN JOURNAL OF CLINICAL NUTRITION. 2017;71(7):825-831.

7823. Maalin Nadia, Mohamed Sophie, Kramer Robin, Cornelissen Piers, Martin Daniel, Tovee Martin Beyond BMI for self-estimates of body size and shape: A new method for developing stimuli correctly calibrated for body composition. BEHAVIOR RESEARCH METHODS. 2021;53(3):1308-1321.

7824. Hay Phillipa, Touyz Stephen, Sud Rishi Treatment for severe and enduring anorexia nervosa: A review. AUSTRALIAN AND NEW ZEALAND JOURNAL OF PSYCHIATRY. 2012;46(12):1136-1144.

7825. Freiberger V., Halle M. Dyslipoproteinemia and physical activity. DIABETOLOGE. 2012;8(7):562-567.

7826. Roy Nelson, Stemple Joseph, Merrill Ray, Thomas Lisa Dysphagia in the elderly: Preliminary evidence of prevalence, risk factors, and socioemotional effects. ANNALS OF OTOLOGY RHINOLOGY AND LARYNGOLOGY. 2007;116(11):858-865.

7827. Hebebrand Johannes, Hildebrandt Tom, Schloegl Haiko, Seitz Jochen, Denecke Saskia, Vieira Diana, Gradl-Dietsch Gertraud, Peters Triinu, Antel Jochen, Lau David, Fulton Stephanie The role of hypoleptinemia in the psychological and behavioral adaptation to starvation: Implications for anorexia nervosa. NEUROSCIENCE AND BIOBEHAVIORAL REVIEWS. 2022;141():.

7828. Rahmati-Najarkolaei Fatemeh, Tavafian Sedigheh, Fesharaki Mohammad, Jafari Mohammad Factors Predicting Nutrition and Physical Activity Behaviors Due to Cardiovascular Disease in Tehran University Students: Application of Health Belief Model. IRANIAN RED CRESCENT MEDICAL JOURNAL. 2015;17(3):.

7829. Hosokawa Rie, Kawabe Kentaro, Nakachi Kiwamu, Soga Junya, Horiuchi Fumie, Ueno Shu-ichi Effects of social media on body dissatisfaction in junior high school girls in Japan.

EATING BEHAVIORS. 2023;48():.

7830. Vinereanu Dragos Risk factors for atherosclerotic disease: Present and future. HERZ. 2006;31(3):5-24.

7831. Pelaez-Fernandez Maria, Romero-Mesa Juana, Extremera Natalio From Deficits in Emotional Intelligence to Eating Disorder Symptoms: A Sequential Path Analysis Approach Through Self-Esteem and Anxiety. FRONTIERS IN PSYCHOLOGY. 2021;12():.

7832. Wang Lei, Cheng Weining, Meng Jia, Speakmon Mickey, Qiu Jiangping, Pillai Suresh, Zhu-Salzman Keyan Hypoxic environment protects cowpea bruchid (Callosobruchus maculatus) from electron beam irradiation damage. PEST MANAGEMENT SCIENCE. 2019;75(3):726-735.

7833. Hattori Hiroyuki, Moriyama Akihiro, Ohno Tomoki, Shibata Takahiro, Iwahashi Hitoshi, Mitsunaga Tohru Molecular networking-based lipid profiling and multi-omics approaches reveal new contributions of functional vanilloids to gut microbiota and lipometabolism changes. FOOD CHEMISTRY: MOLECULAR SCIENCES. 2022;5():.

7834. Haring Bernhard, Wissel Stephanie, Manson JoAnn Somatic Mutations and Clonal Hematopoiesis as Drivers of Age-Related Cardiovascular Risk. CURRENT CARDIOLOGY REPORTS. 2022;24(8):1049-1058.

7835. Avan Abolfazl, Hachinski Vladimir Stroke and dementia, leading causes of neurological disability and death, potential for prevention. ALZHEIMERS \& DEMENTIA. 2021;17(6):1072-1076.

7836. Westing A., Kupers L., Geleijnse J. Diet and Kidney Function: a Literature Review. CURRENT HYPERTENSION REPORTS. 2020;22(2):.

7837. Poskitt E. Childhood obesity in low- and middle-income countries. PAEDIATRICS AND INTERNATIONAL CHILD HEALTH. 2014;34(4):239-249.

7838. Becue-Bertaut Monica, Kern Josipa, Hernandez-Maldonado Maria-Luisa, Juresa Vesna, Vuletic Silvije Health-risk behaviour in Croatia. PUBLIC HEALTH. 2008;122(2):140-150.

7839. Mumford Jessica, Kohn Michael, Briody Julie, Miskovic-Wheatley Jane, Madden Sloane, Clarke Simon, Biggin Andrew, Schindeler Aaron, Munns Craig Long-term Outcomes of Adolescent Anorexia Nervosa on Bone. JOURNAL OF ADOLESCENT HEALTH. 2019;64(3):305-310.

7840. Pombo CM, Tsujita T, Kyriakis JM, Bonventre JV, Force T Activation of the Ste20-like oxidant stress response kinase-1 during the initial stages of chemical anoxia-induced necrotic cell death - Requirement for dual inputs of oxidant stress and increased cytosolic {[}Ca2+]. JOURNAL OF BIOLOGICAL CHEMISTRY. 1997;272(46):29372-29379.

7841. De Bortoli Marit, Oellingrath Inger, Fell Anne, Burdorf Alex, Robroek Suzan Influence of lifestyle risk factors on work ability and sick leave in a general working population in Norway: a 5-year longitudinal study. BMJ OPEN. 2021;11(2):.

7842. Kononova Yulia, Likhonosov Nikolai, Babenko Alina Metformin: Expanding the Scope of Application-Starting Earlier than Yesterday, Canceling Later. INTERNATIONAL JOURNAL OF MOLECULAR SCIENCES. 2022;23(4):.

7843. Kim Jae-Sung, Wang Jin-Hee, Lemasters John Mitochondrial permeability transition in rat hepatocytes after anoxia/reoxygenation: role of Ca2+-dependent mitochondrial formation of reactive oxygen species. AMERICAN JOURNAL OF PHYSIOLOGY-GASTROINTESTINAL AND LIVER PHYSIOLOGY. 2012;302(7):G723-G731.

7844. O'Donoghue B. Addressing physical health in mental illness: the urgent need to translate evidence-based interventions into routine clinical practice. IRISH JOURNAL OF PSYCHOLOGICAL MEDICINE. 2021;38(1, SI):1-5.

7845. Hong Yangsun, Kim Sunghak Influence of Presumed Media Influence for Health Prevention: How Mass Media Indirectly Promote Health Prevention Behaviors through Descriptive Norms. HEALTH COMMUNICATION. 2020;35(14):1800-1810.

7846. Trudel-Fitzgerald Claudia, Tworoger Shelley, Poole Elizabeth, Zhang Xuehong, Giovannucci Edward, Meyerhardt Jeffrey, Kubzansky Laura Psychological Symptoms and Subsequent Healthy Lifestyle After a Colorectal Cancer Diagnosis. HEALTH PSYCHOLOGY. 2018;37(3):207-217.

7847. Malik Aqsa, Buck Leslie Adenosinergic modulation of neuronal activity in the pond snail Lymnaea stagnalis. JOURNAL OF EXPERIMENTAL BIOLOGY. 2010;213(7):1126-1132.

7848. Baker Jessica, Schaumberg Katherine, Munn-Chernoff Melissa Genetics of Anorexia Nervosa. CURRENT PSYCHIATRY REPORTS. 2017;19(11):.

7849. Luyten Patrick, Campbell Chloe, Allison Elizabeth, Fonagy Peter The Mentalizing Approach to Psychopathology: State of the Art and Future Directions. . 2020;16():297-325.

7850. Bonazza Federica, Politi Giuliana, Leone Daniela, Vegni Elena, Borghi Lidia Psychological factors in functional hypothalamic amenorrhea: A systematic review and meta-analysis. FRONTIERS IN ENDOCRINOLOGY. 2023;14():.

7851. Matovu Joseph, Kabwama Stephen, Ssekamatte Tonny, Ssenkusu John, Wanyenze Rhoda COVID-19 Awareness, Adoption of COVID-19 Preventive Measures, and Effects of COVID-19 Lockdown Among Adolescent Boys and Young Men in Kampala, Uganda. JOURNAL OF COMMUNITY HEALTH. 2021;46(4):842-853.

7852. Luo Honghui, Song Jun, Toivonen Peter, Gong Yihui, Forney Charles, Palmer Leslie, Fillmore Sherry, Pang XueQun, Zhang ZhaoQi Proteomic changes in `Ambrosia' apple fruit during cold storage and in response to delayed cooling treatment. POSTHARVEST BIOLOGY AND TECHNOLOGY. 2018;137():66-76.

7853. Percival L., Marynowski L., Baudin F., Goderis S., De Vleeschouwer D., Rakocinski M., Narkiewicz K., Corradini C., Da Silva A., Claeys P. Combined Nitrogen-Isotope and Cyclostratigraphy Evidence for Temporal and Spatial Variability in Frasnian-Famennian Environmental Change. GEOCHEMISTRY GEOPHYSICS GEOSYSTEMS. 2022;23(5):.

7854. Pillai Varshinie, Buck Leslie, Lari Ebrahim Scavenging of reactive oxygen species mimics the anoxic response in goldfish pyramidal neurons. JOURNAL OF EXPERIMENTAL BIOLOGY. 2021;224(10):.

7855. Aitken PG, Tombaugh GC, Turner DA, Somjen GG Similar propagation of SD and hypoxic SD-like depolarization in rat hippocampus recorded optically and electrically. JOURNAL OF NEUROPHYSIOLOGY. 1998;80(3):1514-1521.

7856. Luo Yong, Wan Qing, Xu Min, Zhou Qing, Chen Xuepiao, Yin Dong, He Huan, He Ming Nutritional preconditioning induced by astragaloside IV on isolated hearts and cardiomyocytes against myocardial ischemia injury via improving Bcl-2-mediated mitochondrial function. CHEMICO-BIOLOGICAL INTERACTIONS. 2019;309():.

7857. Lattimore PJ, Thompson GM, Halford JCG Developmental onset of eating-related color-naming interference: The role of restraint and eating psychopathology. INTERNATIONAL JOURNAL OF EATING DISORDERS. 2000;28(1):27-32.

7858. Wu Li-Ying, Ma Zi-Min, Fan Xue-Lai, Zhao Tong, Liu Zhao-Hui, Huang Xin, Li Ming-Ming, Xiong Lei, Zhang Kuan, Zhu Ling-Ling, Fan Ming The anti-necrosis role of hypoxic preconditioning after acute anoxia is mediated by aldose reductase and sorbitol pathway in PC12 cells. CELL STRESS \& CHAPERONES. 2010;15(4):387-394.

7859. Northam EA, Todd S, Cameron FJ Interventions to promote optimal health outcomes in children with Type 1 diabetes - are they effective?. DIABETIC MEDICINE. 2006;23(2):113-121.

7860. Sivanantham Parthibane, Sahoo Jayaprakash, Lakshminarayanan Subitha, Bobby Zachariah, Kar Sitanshu Profile of risk factors for Non-Communicable Diseases (NCDs) in a highly urbanized district of India: Findings from Puducherry district-wide STEPS Survey, 2019-20. PLOS ONE. 2021;16(1):.

7861. Heneghan Helen, Heinberg Leslie, Windover Amy, Rogula Tomasz, Schauer Philip Weighing the evidence for an association between obesity and suicide risk. SURGERY FOR OBESITY AND RELATED DISEASES. 2012;8(1):98-107.

7862. Ruiz Guerrero Francisco, Gonzalez Gomez Jana, Benito Gonzalez Pilar, Garcia Garcia Jesus, Berja Miguel Ana, Calcedo Giraldo Gabriel, Teresa Garcia-Unzueta Maria, Gomez Del Barrio Andres Low levels of proinflammatory cytokines in a transdiagnostic sample of young male and female early onset eating disorders without any previous treatment: A case control study. PSYCHIATRY RESEARCH. 2022;310():.

7863. Moskowitz David, Seal David Revisiting Obesity and Condom Use in Men Who Have Sex with Men. ARCHIVES OF SEXUAL BEHAVIOR. 2010;39(3):761-765.

7864. Stolses Bergamo Francisco Priscila, Assumpcao Daniela, Arbex Borim Flavia, Senicato Caroline, Malta Deborah Prevalence and co-occurrence of modifiable risk factors in adults

and older people. REVISTA DE SAUDE PUBLICA. 2019;53():.

7865. Akbari-Sedigh Assa, Asghari Golaleh, Yuzbashian Emad, Dehghan Pooneh, Imani Hossein, Mirmiran Parvin Association of dietary pattern with carotid intima media thickness among children with overweight or obesity. DIABETOLOGY \& METABOLIC SYNDROME. 2019;11(1):.

7866. Catala-Lopez Ferran, Hutton Brian, Driver Jane, Ridao Manuel, Valderas Jose, Genova-Maleras Ricard, Fores-Martos Jaume, Alonso-Arroyo Adolfo, Macias Saint-Gerons Diego, Vieta Eduard, Valencia Alfonso, Tabares-Seisdedos Rafael Anorexia nervosa and cancer: a protocol for a systematic review and meta-analysis of observational studies. SYSTEMATIC REVIEWS. 2017;6():.

7867. Forys Weronika, Tokuhama-Espinosa Tracey The Athlete's Paradox: Adaptable Depression. SPORTS. 2022;10(7):.

7868. Dorobantu Maria, Tautu Oana-Florentina, Dimulescu Doina, Sinescu Crina, Gusbeth-Tatomir Paul, Arsenescu-Georgescu Catalina, Mitu Florin, Lighezan Daniel, Pop Calin, Babes Katalin, Giuca Alina, Branza Ileana, Udrescu Mihaela, Herdea Valeria, Darabont Roxana Perspectives on hypertension's prevalence, treatment and control in a high cardiovascular risk East European country: data from the SEPHAR III survey. JOURNAL OF HYPERTENSION. 2018;36(3):690-700.

7869. Melis Nicolas, Rubera Isabelle, Cougnon Marc, Giraud Sebastien, Mograbi Baharia, Belaid Amine, Pisani Didier, Huber Stephan, Lacas-Gervais Sandra, Fragaki Konstantina, Blondeau Nicolas, Vigne Paul, Frelin Christian, Hauet Thierry, Duranton Christophe, Tauc Michel Targeting eIF5A Hypusination Prevents Anoxic Cell Death through Mitochondrial Silencing and Improves Kidney Transplant Outcome. JOURNAL OF THE AMERICAN SOCIETY OF NEPHROLOGY. 2017;28(3):811-822.

7870. Wilson Debra Health Consequences of Childhood Sexual Abuse. PERSPECTIVES IN PSYCHIATRIC CARE. 2010;46(1):56-64.

7871. Wang Zhaoqi, Lin Duomao, Zhang Liang, Liu Wenjun, Tan Hongbao, Ma Jun Penehyclidine hydrochloride prevents anoxia/reoxygenation injury and induces H9c2 cardiomyocyte apoptosis via a mitochondrial pathway. EUROPEAN JOURNAL OF PHARMACOLOGY. 2017;797():115-123.

7872. Breukelen Frank, Krumschnabel Gerhard, Podrabsky Jason Vertebrate cell death in energy-limited conditions and how to avoid it: what we might learn from mammalian hibernators and other stress-tolerant vertebrates. APOPTOSIS. 2010;15(3, SI):386-399.

7873. Pamenter Matthew, Buck Leslie Neuronal membrane potential is mildly depolarized in the anoxic turtle cortex. COMPARATIVE BIOCHEMISTRY AND PHYSIOLOGY A-MOLECULAR \& INTEGRATIVE PHYSIOLOGY. 2008;150(4):410-414.

7874. Smart N., Marshall B., Daley M., Boulos E., Windus J., Baker N., Kwok N. Low-fat diets for acquired hypercholesterolaemia. COCHRANE DATABASE OF SYSTEMATIC REVIEWS. 2011;(2):.

7875. Costa Oliveira Matheus, Coutinho Coelho Maria, Viana Camara Sonia, Souza Suellen Prevalence of obesity in adolescents and young people. RBONE-REVISTA BRASILEIRA DE OBESIDADE NUTRICAO E EMAGRECIMENTO. 2020;14(88):811-820.

7876. Scicluna Todd, Woodland Ryan, Zhu Yafei, Grace Michael, Cook Perran Deep dynamic pools of phosphorus in the sediment of a temperate lagoon with recurring blooms of diazotrophic cyanobacteria. LIMNOLOGY AND OCEANOGRAPHY. 2015;60(6):2185-2196.

7877. Nehring I., Kewitz K., Kries R., Thyen U. Long-term effects of enteral feeding on growth and mental health in adolescents with anorexia nervosa-results of a retrospective German cohort study. EUROPEAN JOURNAL OF CLINICAL NUTRITION. 2014;68(2):171-177.

7878. LeCroy Madison, Nicastro Holly, Truesdale Kimberly, Matheson Donna, Ievers-Landis Carolyn, Pratt Charlotte, Jones Sarah, Sherwood Nancy, Burgess Laura, Robinson Thomas, Yang Song, Stevens June Dietary patterns and associations with BMI in low-income, ethnic minority youth in the USA according to baseline data from four randomised controlled trials. BRITISH JOURNAL OF NUTRITION. 2021;126(1):81-91.

7879. Ma Shufen, Noble Abigail, Butcher Derek, Trouwborst Robert, Luther George Removal of H2S via an iron catalytic cycle and iron sulfide precipitation in the water column of dead end tributaries. ESTUARINE COASTAL AND SHELF SCIENCE. 2006;70(3):461-472.

7880. Anderson Skye, Podrabsky Jason The effects of hypoxia and temperature on metabolic aspects of embryonic development in the annual killifish Austrofundulus limnaeus. JOURNAL OF COMPARATIVE PHYSIOLOGY B-BIOCHEMICAL SYSTEMIC AND

ENVIRONMENTAL PHYSIOLOGY. 2014;184(3):355-370.

7881. Parra-Fernandez Maria, Manzaneque-Canadillas Maria, Onieva-Zafra Maria, Fernandez-Martinez Elia, Fernandez-Munoz Juan, Prado-Laguna Maria, Brytek-Matera Anna Pathological Preoccupation with Healthy Eating (Orthorexia Nervosa) in a Spanish Sample with Vegetarian, Vegan, and Non-Vegetarian Dietary Patterns. NUTRIENTS. 2020;12(12):.

7882. Riedel B., Zuschin M., Haselmair A., Stachowitsch M. Oxygen depletion under glass: Behavioural responses of benthic macrofauna to induced anoxia in the Northern Adriatic. JOURNAL OF EXPERIMENTAL MARINE BIOLOGY AND ECOLOGY. 2008;367(1):17-27.

7883. Arnoldy Lizanne, Gauci Sarah, Young Lauren, Marx Wolfgang, Macpherson Helen, Pipingas Andrew, Civier Oren, White David The association of dietary and nutrient patterns on neurocognitive decline: A systematic review of MRI and PET studies. AGEING RESEARCH REVIEWS. 2023;87():.

7884. Lohse Tina, Rohrmann Sabine, Bopp Matthias, Faeh David Heavy Smoking Is More Strongly Associated with General Unhealthy Lifestyle than Obesity and Underweight. PLOS ONE. 2016;11(2):.

7885. Fardet Anthony, Druesne-Pecollo Nathalie, Touvier Mathilde, Latino-Martel Paule Do alcoholic beverages, obesity and other nutritional factors modify the risk of familial colorectal cancer? A systematic review. CRITICAL REVIEWS IN ONCOLOGY HEMATOLOGY. 2017;119():94-112.

7886. Watanabe Kumi, Okada Ayumi, Okabe Nobuyuki, Onishi Masaru, Morishima Tsuneo A One-Message Question in a Structured Interview: Investigating Psychological Needs of Children and Adolescents with Eating Disorders Directed toward Their Mothers. ACTA MEDICA OKAYAMA. 2011;65(3):185-192.

7887. Larranaga Alejandro, Fluiters Enrique, Docet Maria, Fernandez Sastre Jose, Garcia-Mayor Ricardo Comparative study of cognitive-behavioral psychotherapy and nutritional support in patients with different types of eating disorders. MEDICINA CLINICA. 2014;143(5):196-200.

7888. Gutvirtz Gil, Wainstock Tamar, Landau Daniella, Sheiner Eyal Maternal smoking during pregnancy and long-term neurological morbidity of the offspring. ADDICTIVE

BEHAVIORS. 2019;88():86-91.

7889. Meyer K., Kump L., Ridgwell A. Biogeochemical controls on photic-zone euxinia during the end-Permian mass extinction. GEOLOGY. 2008;36(9):747-750.

7890. Blumenthal Susan, Hoffnagle Elena, Leung Cindy, Lofink Hayley, Jensen Helen, Foerster Susan, Cheung Lilian, Nestle Marion, Willett Walter Strategies to improve the dietary quality of Supplemental Nutrition Assistance Program (SNAP) beneficiaries: an assessment of stakeholder opinions. PUBLIC HEALTH NUTRITION. 2014;17(12):2824-2833.

7891. Koreshe Eyza, Paxton Susan, Miskovic-Wheatley Jane, Bryant Emma, Le Anvi, Maloney Danielle, Touyz Stephen, Maguire Sarah, Consortium Natl Prevention and early intervention in eating disorders: findings from a rapid review. JOURNAL OF EATING DISORDERS. 2023;11(1):.

7892. Cruwys Tegan, Steffens Niklas, Haslam S., Haslam Catherine, Hornsey Matthew, McGarty Craig, Skorich Daniel Predictors of social identification in group therapy. PSYCHOTHERAPY RESEARCH. 2020;30(3):348-361.

7893. Shirk Daisy, Williams Sarah Psychiatric Manifestations of Ehlers-Danlos Syndrome in Adolescents: A Case Report and Literature Review. CURRENT PSYCHIATRY RESEARCH AND REVIEWS. 2020;16(4):288-291.

7894. Cunningham Mitchell, Szabo Marianna, Rodgers Rachel, Franko Debra, Eddy Kamryn, Thomas Jennifer, Murray Stuart, Griffiths Scott An investigation of distress tolerance and difficulties in emotion regulation in the drive for muscularity among women. BODY IMAGE. 2020;33():207-213.

7895. Antin Tamar, Hunt Geoffrey Food choice as a multidimensional experience. A qualitative study with young African American women. APPETITE. 2012;58(3):856-863.

7896. Ricanati EHW, Rome ES Eating disorders: Recognize early to prevent complications. CLEVELAND CLINIC JOURNAL OF MEDICINE. 2005;72(10):895+.

7897. Bao Naren, Tang Bing, Wang Junke Dexmedetomidine Preconditioning Protects Rats from Renal Ischemia-Reperfusion Injury Accompanied with Biphasic Changes of Nuclear

Factor-Kappa B Signaling. JOURNAL OF IMMUNOLOGY RESEARCH. 2020;2020():.

7898. Zorina Svetlana Black shales contamination and depositional paleoenvironment during the Early Aptian OAE 1a in the Eastern Russian Platform. BULLETIN OF GEOSCIENCES. 2022;97(1):123-140.

7899. Whatnall Megan, Skinner Janelle, Pursey Kirrilly, Brain Katherine, Collins Rebecca, Hutchesson Melinda, Burrows Tracy Efficacy of dietary interventions in individuals with substance use disorders for illicit substances or illicit use of pharmaceutical substances: A systematic review. JOURNAL OF HUMAN NUTRITION AND DIETETICS. 2021;34(6):981-993.

7900. Bhutta Muhammad, Hussain Shahbaz, Khan Shahid ANALYSIS OF EFFECT OF LOW CARBOHYDRATE DIET FOR DIABETES MELLITUS MANAGEMENT: A RESEARCH ANALYSIS. INDO AMERICAN JOURNAL OF PHARMACEUTICAL SCIENCES. 2018;5(9):8934-8938.

7901. Centis Elena, Petroni Maria, Ghirelli Veronica, Cioni Mattia, Navacchia Paola, Guberti Emilia, Marchesini Giulio Motivational Interviewing Adapted to Group Setting for the Treatment of Relapse in the Behavioral Therapy of Obesity. A Clinical Audit. NUTRIENTS. 2020;12(12):.

7902. Tessin Allyson, Hendy Ingrid, Sheldon Nathan, Sageman Bradley Redox-controlled preservation of organic matter during ``OAE 3{''} within the Western Interior Seaway. PALEOCEANOGRAPHY. 2015;30(6):702-717.

7903. Hoen T, Lankhaar J Controlled atmosphere stunning of poultry. POULTRY SCIENCE. 1999;78(2):287-289.

7904. Taghizadeh Shahnaz, Farhangi Mahdieh The effectiveness of pediatric obesity prevention policies: a comprehensive systematic review and dose-response meta-analysis of controlled clinical trials. JOURNAL OF TRANSLATIONAL MEDICINE. 2020;18(1):.

7905. Becker Bruce Case report: The physiology of a preventable tragedy-Near death in a hot tub. CLINICAL CASE REPORTS. 2021;9(10):.

7906. Benagiano Giuseppe, Bianchi Paola, Guo Sun-Wei Endometriosis in adolescent and young women. MINERVA OBSTETRICS AND GYNECOLOGY. 2021;73(5):523-535.

7907. Kenney Erica, Wintner Suzanne, Lee Rebekka, Austin S. Obesity Prevention Interventions in US Public Schools: Are Schools Using Programs That Promote Weight Stigma?. PREVENTING CHRONIC DISEASE. 2017;14():.

7908. Sunnetci Eda, Solmaz Volkan, Erbas Oytun Chronic Oxytocin treatment has long lasting therapeutic potential in a rat model of neonatal hypercapnic-hypoxia injury, through enhanced GABAergic signaling and by reducing hippocampal gliosis with its anti-inflammatory feature. PEPTIDES. 2021;135():.

7909. Koh Yen-Chun, Lin Yen-Cheng, Lee Pei-Sheng, Lu Ting-Jang, Lin Kai-Yi, Pan Min-Hsiung A multi-targeting strategy to ameliorate high-fat-diet- and fructose-induced (western diet-induced) non-alcoholic fatty liver disease (NAFLD) with supplementation of a mixture of legume ethanol extracts. FOOD \& FUNCTION. 2020;11(9):7545-7560.

7910. De Souza Mary, Nattiv Aurelia, Joy Elizabeth, Misra Madhusmita, Williams Nancy, Mallinson Rebecca, Gibbs Jenna, Olmsted Marion, Goolsby Marci, Matheson Gordon, Panel Expert 2014 Female Athlete Triad Coalition Consensus Statement on Treatment and Return to Play of the Female Athlete Triad: 1st International Conference held in San Francisco, California, May 2012 and 2nd International Conference held in Indianapolis, Indiana, May 2013. BRITISH JOURNAL OF SPORTS MEDICINE. 2014;48(4):.

7911. Masi Gabriele, Lupetti Ilaria, D'Acunto Giulia, Milone Annarita, Fabiani Deborah, Madonia Ursula, Berloffa Stefano, Lenzi Francesca, Mucci Maria A Comparison between Severe Suicidality and Nonsuicidal Self-Injury Behaviors in Bipolar Adolescents Referred to a Psychiatric Emergency Unit. BRAIN SCIENCES. 2021;11(6):.

7912. Antai-Otong D Treatment considerations for the patient with borderline personality disorder. NURSING CLINICS OF NORTH AMERICA. 2003;38(1):101+.

7913. Abu-Sawwa Renad, Dunbar Sandra, Quyyumi Arshed, Sattler Elisabeth Nutrition intervention in heart failure: should consumption of the DASH eating pattern be recommended to improve outcomes?. HEART FAILURE REVIEWS. 2019;24(4):565-573.

7914. Bell Sarah, Audrey Suzanne, Gunnell David, Cooper Ashley, Campbell Rona The relationship between physical activity, mental wellbeing and symptoms of mental health disorder in adolescents: a cohort study. INTERNATIONAL JOURNAL OF BEHAVIORAL NUTRITION AND PHYSICAL ACTIVITY. 2019;16(1):.

7915. San-Cristobal Rodrigo, Navas-Carretero Santiago, Celis-Morales Carlos, Brennan Lorraine, Walsh Marianne, Lovegrove Julie, Daniel Hannelore, Saris Wim, Traczyk Iwonna, Manios Yannis, Gibney Eileen, Gibney Michael, Mathers John, Martinez J. Analysis of Dietary Pattern Impact on Weight Status for Personalised Nutrition through On-Line Advice: The Food4Me Spanish Cohort. NUTRIENTS. 2015;7(11):9523-9537.

7916. Hjorth Peter, Medici Clara, Juel Anette, Madsen Nikolaj, Vandborg Kirsten, Munk-Jorgensen Povl Improving quality of life and physical health in patients with schizophrenia: A 30-month program carried out in a real-life setting. INTERNATIONAL JOURNAL OF SOCIAL PSYCHIATRY. 2017;63(4):287-296.

7917. Mountjoy Margo, Junge Astrid, Budgett Richard, Doerr Dominik, Leglise Michel, Miller Stuart, Moran Jane, Foster Jeremy Health promotion by International Olympic Sport Federations: priorities and barriers. BRITISH JOURNAL OF SPORTS MEDICINE. 2019;53(17):1117+.

7918. Krishnamoorthy Radika, Venkatraman Anuradha Polyphenols activate energy sensing network in insulin resistant models. CHEMICO-BIOLOGICAL INTERACTIONS. 2017;275():95-107.

7919. Gillbanks Lucy, Mountjoy Margo, Filbay Stephanie Lightweight rowers' perspectives of living with Relative Energy Deficiency in Sport (RED-S). PLOS ONE. 2022;17(3):.

7920. StriegelMoore RH, Wilfley DE, Caldwell MB, Needham ML, Brownell KD Weight-related attitudes and behaviors of women who diet to lose weight: A comparison of black dieters and white dieters. OBESITY RESEARCH. 1996;4(2):109-116.

7921. Leone Alessandro, Battezzati Alberto, De Amicis Ramona, De Carlo Giulia, Bertoli Simona Trends of Adherence to the Mediterranean Dietary Pattern in Northern Italy from 2010 to 2016. NUTRIENTS. 2017;9(7):.

7922. Novak Josh, Pratt Keeley, Hernandez Daphne, Berge Jerica Family systems and obesity: A review of key concepts and influences within and between family subsystems and a call for family-informed interventions. JOURNAL OF FAMILY THEORY \& REVIEW. 2023;15(1):38-56.

7923. Yang Chung, Chen Xiaoxin Research on esophageal cancer: With personal perspectives from studies in China and Kenya. INTERNATIONAL JOURNAL OF CANCER.

2021;149(2):264-276.

7924. King Kristel, Meader Nick, Wright Kath, Graham Hilary, Power Christine, Petticrew Mark, White Martin, Sowden Amanda Characteristics of Interventions Targeting Multiple Lifestyle Risk Behaviours in Adult Populations: A Systematic Scoping Review. PLOS ONE. 2015;10(1):.

7925. Loveless Meredith, Hewitt Geri, Care Comm Committee Opinion No. 702: Female Athlete Triad. OBSTETRICS AND GYNECOLOGY. 2017;129(6):E160-E167.

7926. Shin Cha-Nam, Keller Colleen, An Kyungeh, Sim Jeongha Cardiovascular Disease in Korean Americans A Systematic Review. JOURNAL OF CARDIOVASCULAR NURSING. 2018;33(1):82-93.

7927. Michaelsen Kim, Grummer-Strawn Laurence, Begin France Emerging issues in complementary feeding: Global aspects. MATERNAL AND CHILD NUTRITION. 2017;13(2, SI):.

7928. Nsamenang Sheri, Gutierrez Carline, Jones Jane, Jenkins Glenn, Tibelius Stephanie, DiGravio Anna, Chamas Basma, Ewusie Joycelyne, Geddie Hannah, Punthakee Zubin, Samaan M., Wahi Gita, Morrison Katherine Impact of SARS-CoV-2 pandemic on the mental and physical health of children enrolled in a paediatric weight management clinic. PAEDIATRICS \& CHILD HEALTH. 2022;27(SUPPL 1, 1, SI):S72-S77.

7929. Saguil Aaron, Stephens Mark Interventions to Prevent Childhood Obesity. AMERICAN FAMILY PHYSICIAN. 2012;86(1):30-32.

7930. Cereda Emanuele, Malavazos Alexis, Caccialanza Riccardo, Rondanelli Mariangela, Fatati Giuseppe, Barichella Michela Weight cycling is associated with body weight excess and abdominal fat accumulation: A cross-sectional study. CLINICAL NUTRITION. 2011;30(6):718-723.

7931. Dalton William, Wang Liang, Southerland Jodi, Schetzina Karen, Slawson Deborah Self-Reported Versus Actual Weight and Height Data Contribute to Different Weight Misperception Classifications. SOUTHERN MEDICAL JOURNAL. 2014;107(6):348-355.

7932. Conti Maria, Gnesi Marco, De Giuseppe Rachele, Giampieri Francesca, Monti Maria, Mshanga Naelijwa, Kinabo Joyce, Msuya John, Cena Hellas Validation of a Food Knowledge Questionnaire on Tanzanian Women of Childbearing Age. NUTRIENTS. 2022;14(3):.

7933. Huon GF, Braganza C, Brown LB, Ritchie JE, Roncolato WG Reflections on prevention in dieting-induced disorders. INTERNATIONAL JOURNAL OF EATING DISORDERS. 1998;23(4):455-458.

7934. Bergmeier Heidi, Hill Briony, Haycraft Emma, Blewitt Claire, Lim Siew, Meyer Caroline, Skouteris Helen Maternal body dissatisfaction in pregnancy, postpartum and early parenting: An overlooked factor implicated in maternal and childhood obesity risk. APPETITE. 2020;147():.

7935. Alleman Rick, Tsang Alvin, Ryan Terence, Patteson Daniel, McClung Joseph, Spangenburg Espen, Shaikh Saame, Neufer P., Brown David Exercise-induced protection against reperfusion arrhythmia involves stabilization of mitochondrial energetics. AMERICAN JOURNAL OF PHYSIOLOGY-HEART AND CIRCULATORY PHYSIOLOGY. 2016;310(10):H1360-H1370.

7936. Arts Jennifer, Fernandez Maria, Lofgren Ingrid Coronary Heart Disease Risk Factors in College Students. ADVANCES IN NUTRITION. 2014;5(2):177-187.

7937. Tavallaie Mojdeh, Voshtani Ramouna, Deng Xinxian, Qiao Yixue, Jiang Faqin, Collman James, Fu Lei Moderation of mitochondrial respiration mitigates metabolic syndrome of aging. PROCEEDINGS OF THE NATIONAL ACADEMY OF SCIENCES OF THE UNITED STATES OF AMERICA. 2020;117(18):9840-9850.

7938. Salas Ximena The ineffectiveness and unintended consequences of the public health war on obesity. CANADIAN JOURNAL OF PUBLIC HEALTH-REVUE CANADIENNE DE SANTE PUBLIQUE. 2015;106(2):E79-E81.

7939. Verma Amit, Singh Prithvi, Al-Saeed Fatimah, Ahmed Ahmed, Kumar Sunil, Kumar Ashok, Dev Kapil, Dohare Ravins Unravelling the role of telomere shortening with ageing and their potential association with diabetes, cancer, and related lifestyle factors. TISSUE \& CELL. 2022;79():.

7940. James-Martin Genevieve, Baird Danielle, Hendrie Gilly Strategies to Reduce Consumption of Unhealthy Foods and Beverages: Scenario Modeling to Estimate the Impact

on the Australian Population's Energy and Nutrient Intakes. JOURNAL OF THE ACADEMY OF NUTRITION AND DIETETICS. 2021;121(8):1463-1483.

7941. Benca Ruth, Duncan Marilyn, Frank Ellen, McClung Colleen, Nelson Randy, Vicentic Aleksandra Biological rhythms, higher brain function, and behavior: Gaps, opportunities, and challenges. BRAIN RESEARCH REVIEWS. 2009;62(1):57-70.

7942. Benite-Ribeiro Sandra, Lucas-Lima Kamila, Jones Jessica, Santos Julia Transcription of mtDNA and dyslipidemia are ameliorated by aerobic exercise in type 2 diabetes. MOLECULAR BIOLOGY REPORTS. 2020;47(9):7297-7303.

7943. Mujamammi Ahmed, Alluhaymid Yousef, Alshibani Mohammed, Alotaibi Fawzan, Alzahrani Khalid, Alotaibi Abdulmajeed, Almasabi Ahmed, Sabi Essa Awareness of cardiovascular disease associated risk factors among Saudis in Riyadh City. JOURNAL OF FAMILY MEDICINE AND PRIMARY CARE. 2020;9(6):3100-3105.

7944. Haff Darlene Racial/Ethnic Differences in Weight Perceptions and Weight Control Behaviors Among Adolescent Females. YOUTH \& SOCIETY. 2009;41(2):278-301.

7945. Damaso Ana, Piano Aline, Silveira Campos Raquel, Corgosinho Flavia, Siegfried Wolfgang, Caranti Danielle, Landi Masquio Deborah, Carnier June, Sanches Priscila, Silva Patricia, Oller Nascimento Claudia, Oyama Lila, Aguilera Dantas Alexandre, Mello Marco, Tufik Sergio, Tock Lian Multidisciplinary Approach to the Treatment of Obese Adolescents: Effects on Cardiovascular Risk Factors, Inflammatory Profile, and Neuroendocrine Regulation of Energy Balance. INTERNATIONAL JOURNAL OF ENDOCRINOLOGY. 2013;2013():.

7946. Ross Amy, Paxton Susan, Rodgers Rachel Y's Girl: Increasing body satisfaction among primary school girls. BODY IMAGE. 2013;10(4):614-618.

7947. Stavridou Androniki, Kapsali Evangelia, Panagouli Eleni, Thirios Athanasios, Polychronis Konstantinos, Bacopoulou Flora, Psaltopoulou Theodora, Tsolia Maria, Sergentanis Theodoros, Tsitsika Artemis Obesity in Children and Adolescents during COVID-19 Pandemic. CHILDREN-BASEL. 2021;8(2):.

7948. Morton Hallie, Basu Tanisha, Bose Chhanda, Reddy P. Impact of Chronic Conditions and Dementia in Rural West Texas: A Healthy Aging Study. JOURNAL OF ALZHEIMERS

DISEASE. 2022;87(1):33-49.

7949. Silva Nathalia, Confortim Heloisa Classic ballet practice increases eating disorders prevalence in women. RBNE-REVISTA BRASILEIRA DE NUTRICAO ESPORTIVA. 2022;16(96):46-52.

7950. Castro Rodolfo, Ribeiro-Alves Marcelo, Oliveira Catia, Romero Carmen, Perazzo Hugo, Simjanoski Mario, Kapciznki Flavio, Balanza-Martinez Vicent, De Boni Raquel What Are We Measuring When We Evaluate Digital Interventions for Improving Lifestyle? A Scoping Meta-Review. FRONTIERS IN PUBLIC HEALTH. 2022;9():.

7951. Yang Yi-De, Zeng Yuan, Li Jian, Zhou Jun-Hua, He Quan-Yuan, Zheng Chan-Juan, Reichetzeder Christoph, Kraemer Bernhard, Hocher Berthold Association of BMAL1 clock gene polymorphisms with fasting glucose in children. PEDIATRIC RESEARCH. 2023;():.

7952. Madrigal Jessica, Cedillo-Couvert Esteban, Ricardo Ana, Appel Lawrence, Anderson Cheryl, Deo Rajat, Hamm L., Cornish-Zirker Denise, Tan Thida, Sha Daohang, Hsu Jesse, Zenk Shannon, Saunders Milda, Persky Victoria, Lash James, Investigators CRIC Neighborhood Food Outlet Access and Dietary Intake among Adults with Chronic Kidney Disease: Results from the Chronic Renal Insufficiency Cohort Study. JOURNAL OF THE ACADEMY OF NUTRITION AND DIETETICS. 2020;120(7):1151+.

7953. Wang Song, Xiong Zhuang, Liu Yangyang, Leng Yan, Deng Houbo, Shen Dong, Meng Xiangtong, Liu Tiejun Efficacy and safety of acupuncture and moxibustion combined with the external application of traditional Chinese medicine in the treatment of primary liver cancer A protocol for systematic review and meta-analysis. MEDICINE. 2021;100(43):.

7954. Gutke Annelie, Sundfeldt Karin, De Baets Liesbet Lifestyle and Chronic Pain in the Pelvis: State of the Art and Future Directions. JOURNAL OF CLINICAL MEDICINE. 2021;10(22):.

7955. Zutphen Moniek, Boshuizen Hendriek, Kenkhuis Marlou-Floor, Wesselink Evertine, Geijsen Anne, Wilt Johannes, Halteren Henk, Bilgen Ernst, Keulen Eric, Janssen-Heijnen Maryska, Breukink Stephanie, Bours Martijn, Kok Dieuwertje, Winkels Renate, Weijenberg Matty, Kampman Ellen, Duijnhoven Franzel Lifestyle after colorectal cancer diagnosis in relation to recurrence and all-cause mortality. AMERICAN JOURNAL OF CLINICAL NUTRITION. 2021;113(6):1447-1457.

7956. Pfitzner J. The role of an ambient pressure oxygen source during one-lung ventilation for thoracoscopic surgery. ANAESTHESIA AND INTENSIVE CARE. 2016;44(1):20-27.

7957. Saab IN, Sachs MM A flooding-induced xyloglucan endo-transglycosylase homolog in maize is responsive to ethylene and associated with aerenchyma. PLANT PHYSIOLOGY. 1996;112(1):385-391.

7958. Lee Yejin, Lee Dongbin, Jung Hyewon, Cho Yunji, Baek Ji, Hong Kyung Heterogeneous early illness courses of Korean patients with bipolar disorders: replication of the staging model. BMC PSYCHIATRY. 2022;22(1):.

7959. Cohen Juliana, Kraak Vivica, Choumenkovitch Silvina, Hyatt Raymond, Economos Christina The CHANGE Study: A Healthy-Lifestyles Intervention to Improve Rural Children's Diet Quality. JOURNAL OF THE ACADEMY OF NUTRITION AND DIETETICS. 2014;114(1):48-53.

7960. Wang Yan, Wang Tao Application of Improved LightGBM Model in Blood Glucose Prediction. APPLIED SCIENCES-BASEL. 2020;10(9):.

7961. Park So, Cormier Eileen Influence of Siblings on Child Health Behaviors and Obesity: A Systematic Review. JOURNAL OF CHILD AND FAMILY STUDIES. 2018;27(7):2069-2081.

7962. Merritt Rowena, Groot Jacqueline, Almajali Lama, Patel Nitesh Using Community-Based Prevention Marketing to Generate Demand for Healthy Diets in Jordan. NUTRIENTS. 2021;13(9):.

7963. Ladilov YV, Balser-Schafer C, Haffner S, Maxeiner H, Piper HM Pretreatment with PKC activator protects cardiomyocytes against reoxygenation-induced hypercontracture independently of Ca2+ overload. CARDIOVASCULAR RESEARCH. 1999;43(2):408-416.

7964. Anstruther Se'era, Barbour-Tuck Erin, Vatanparast Hassan Socioeconomic settings and food consumption patterns of 2-5-year-old children in developed countries: a scoping review. FACETS. 2021;6():1495-1509.

7965. CHEVALIER B, CALLENSELAMRANI F, HEYMES C, SWYNGHEDAUW B MOLECULAR-BASIS OF REGRESSION OF CARDIAC-HYPERTROPHY. AMERICAN JOURNAL OF

CARDIOLOGY. 1994;73(10):C10-C17.

7966. Han MW, Park YC The development of anoxia in the artificial Lake Shihwa, Korea, as a consequence of intertidal reclamation. MARINE POLLUTION BULLETIN. 1999;38(12):1194-1199.

7967. Wu Xiuyun, Veugelers Paul, Ohinmaa Arto Health Behavior, Health-Related Quality of Life, and Mental Health Among Canadian Children: A Population-Based Cohort Study. FRONTIERS IN NUTRITION. 2021;8():.

7968. Weider Siri, Indredavik Marit, Lydersen Stian, Hestad Knut Neuropsychological Function in Patients with Anorexia Nervosa or Bulimia Nervosa. INTERNATIONAL JOURNAL OF EATING DISORDERS. 2015;48(4):397-405.

7969. Nam Soohyun, Redeker Nancy, Whittemore Robin Social networks and future direction for obesity research: A scoping review. NURSING OUTLOOK. 2015;63(3):299-317.

7970. Agam K, Campenhausen M, Levy S, Ben-Ami HC, Cook B, Kirschfeld K, Minke B Metabolic stress reversibly activates the Drosophila light-sensitive channels TRP and TRPL in vivo. JOURNAL OF NEUROSCIENCE. 2000;20(15):5748-5755.

7971. Le Long, Barendregt Jan, Hay Phillipa, Sawyer Susan, Paxton Susan, Mihalopoulos Cathrine The modelled cost-effectiveness of cognitive dissonance for the prevention of anorexia nervosa and bulimia nervosa in adolescent girls in Australia. INTERNATIONAL JOURNAL OF EATING DISORDERS. 2017;50(7):834-841.

7972. Zhang Yi, Wang Xueyan, Yang Haixia Effect of traditional Chinese medicine nursing on postoperative patients with gastric cancer and its impact on quality of life. AMERICAN JOURNAL OF TRANSLATIONAL RESEARCH. 2021;13(5):5589-5595.

7973. Walnik Lukas, Kueck Momme, Tegtbur Uwe, Fischer Volkhard, Kerling Arno Physical Fitness, Nutrition and Quality of Life in German Medical Students. NUTRIENTS. 2022;14(24):.

7974. Kutcher Alison, Pichette Priscilla, Macdonald Mary, Carnevale Franco Exploring the health and well-being of children and youth in Winneway, Quebec. INTERNATIONAL

JOURNAL OF INDIGENOUS HEALTH. 2019;14(2):115-132.

7975. Ewert Benjamin Promoting health in schools: Theoretical reflections on the settings approach versus nudge tactics. SOCIAL THEORY \& HEALTH. 2017;15(4):430-447.

7976. Mortsiefer A., Ludt S., Pentzek M., Wilm S., Brotons C. Patients' Needs and Expectations Concerning Prevention and Health Advice in Primary Care. Results from the EUROPREVIEW Survey. GESUNDHEITSWESEN. 2014;76(7):417-422.

7977. Swinburn B., Millar L., Utter J., Kremer P., Moodie M., Mavoa H., Snowdon W., McCabe M., Malakellis M., De Courten M., Waqa G., Fotu K., Roberts G., Scragg R. The Pacific Obesity Prevention in Communities project: project overview and methods. OBESITY REVIEWS. 2011;12(2, SI):3-11.

7978. Cole Adam, Laxer Rachel, Patte Karen, Leatherdale Scott Can We Reverse this Trend? Exploring Health and Risk Behaviours of Grade 12 Cohorts of Ontario Students from 2013-2019. INTERNATIONAL JOURNAL OF ENVIRONMENTAL RESEARCH AND PUBLIC HEALTH. 2021;18(6):.

7979. FRANK E, SPANIER C INTERPERSONAL PSYCHOTHERAPY FOR DEPRESSION - OVERVIEW, CLINICAL EFFICACY, AND FUTURE-DIRECTIONS. CLINICAL PSYCHOLOGY-SCIENCE AND PRACTICE. 1995;2(4):349-369.

7980. Booth David, Booth Phil Targeting cultural changes supportive of the healthiest lifestyle patterns. A biosocial evidence-base for prevention of obesity. APPETITE. 2011;56(1):210-221.

7981. Romo Lynsey An Examination of How People Who Have Lost Weight Communicatively Negotiate Interpersonal Challenges to Weight Management. HEALTH COMMUNICATION. 2018;33(4):469-477.

7982. Chen Tianpeng, Niu Li, Wang Liang, Zhou Qing, Zhao Xiaoyu, Lai Songqing, He Xinlan, He Huan, He Ming Ferulic acid protects renal tubular epithelial cells against anoxia/reoxygenadon injury mediated by AMPK alpha 1. FREE RADICAL RESEARCH. 2022;56(2):173-184.

7983. Robert F, Bert L, Stoppini L Blockade of NMDA-receptors or calcium-channels attenuates the ischaemia-evoked efflux of glutamate and phosphoethanolamine and depression of neuronal activity in rat organotypic hippocampal slice cultures. COMPTES RENDUS BIOLOGIES. 2002;325(4):495-504.

7984. Bohon Cara, Stice Eric, Burton Emily, Fudell Molly, Nolen-Hoeksema Susan A prospective test of cognitive vulnerability models of depression with adolescent girls. BEHAVIOR THERAPY. 2008;39(1):79-90.

7985. Lussi A., Jaeggi T. Erosion - diagnosis and risk factors. CLINICAL ORAL INVESTIGATIONS. 2008;12(1):S5-S13.

7986. Demircan Mehmet, Aksoy Tugrul, Ceran Canan, Kafkasli Ayse Tracheal agenesis and esophageal atresia with proximal and distal bronchoesophageal fistulas. JOURNAL OF PEDIATRIC SURGERY. 2008;43(8):.

7987. Szoeke Cassandra, Dang Christa, Lehert Philippe, Hickey Martha, Morris Meg, Dennerstein Lorraine, Campbell Stephen Unhealthy habits persist: The ongoing presence of modifiable risk factors for disease in women. PLOS ONE. 2017;12(4):.

7988. Wiseman Nicola, Harris Neil, Lee Patricia Lifestyle knowledge and preferences in preschool children: Evaluation of the Get up and Grow healthy lifestyle education programme. HEALTH EDUCATION JOURNAL. 2016;75(8):1012-1024.

7989. Huang Donna, Brien Amanda, Omari Lima, Culpin Angela, Smith Melody, Egli Victoria Bus Stops Near Schools Advertising Junk Food and Sugary Drinks. NUTRIENTS. 2020;12(4):.

7990. Bengmark Stig Pre-, Pro-, Synbiotics and Human Health. FOOD TECHNOLOGY AND BIOTECHNOLOGY. 2010;48(4, SI):464-475.

7991. Hawes T., Hines A., Viant M., Bale J., Worland M., Convey P. METABOLOMIC FINGERPRINT OF CRYO-STRESS IN A FREEZE TOLERANT INSECT. CRYOLETTERS. 2008;29(6):505-515.

7992. FARRIMOND P, STODDART DP, JENKYNS HC AN ORGANIC GEOCHEMICAL PROFILE OF THE TOARCIAN ANOXIC EVENT IN NORTHERN ITALY. CHEMICAL GEOLOGY.

1994;111(1-4):17-33.

7993. Pregnall AM Effects of aerobic versus anoxic conditions on glutamine synthetase activity in eelgrass (Zostera marina L.) roots: regulation of ammonium assimilation potential. JOURNAL OF EXPERIMENTAL MARINE BIOLOGY AND ECOLOGY. 2004;311(1):11-24.

7994. Ricciardi-Rigault M, Bird DF, Prairie YT Changes in sediment viral and bacterial abundances with hypolimnetic oxygen depletion in a shallow eutrophic Lac Brome (Quebec, Canada). CANADIAN JOURNAL OF FISHERIES AND AQUATIC SCIENCES. 2000;57(6):1284-1290.

7995. Ghozlan A, Munnich A MAOB: a modifier gene in phenylketonuria?. M S-MEDECINE SCIENCES. 2004;20(10):929-932.

7996. Artamonova Irina, Petrova Natalia, Lyubimova Natalia, Kolbina Natalia, Bryzzhin Alexander, Borodin Alexander, Levko Tatyana, Mamaeva Ekaterina, Pervunina Tatiana, Vasichkina Elena, Nikitina Irina, Zlotina Anna, Efimtsev Alexander, Kostik Mikhail Case Report: COVID-19-Associated ROHHAD-Like Syndrome. FRONTIERS IN PEDIATRICS. 2022;10():.

7997. McKenzie Craig, Tan Jian, Macia Laurence, Mackay Charles The nutrition-gut microbiome-physiology axis and allergic diseases. IMMUNOLOGICAL REVIEWS. 2017;278(1, SI):277-295.

7998. Wild Christopher The Role of Cancer Research in Noncommunicable Disease Control. JOURNAL OF THE NATIONAL CANCER INSTITUTE. 2012;104(14):1051-1058.

7999. Ziegler Victor, Ploschuk Edmundo, Weibel Antonio, Insausti Pedro Short-term responses to flooding stress of three Prunus rootstocks. SCIENTIA HORTICULTURAE. 2017;224():135-141.

8000. Wewetzer C, Mauer-Mucke K, Ballauff A, Remschmidt H, Hebebrand J Possible pathophysiological, diagnostic and therapeutic implications of recent findings related to leptin secretion in patients with anorexia nervosa. ZEITSCHRIFT FUR KINDER-UND JUGENDPSYCHIATRIE UND PSYCHOTHERAPIE. 1998;26(4):244-252.

8001. Khoubaeva Diana, Dimick Mikaela, Timmins Vanessa, Fiksenbaum Lisa, Mitchell Rachel, Schaffer Ayal, Sinyor Mark, Goldstein Benjamin Clinical correlates of suicidality and self-injurious behaviour among Canadian adolescents with bipolar disorder. EUROPEAN CHILD \& ADOLESCENT PSYCHIATRY. 2023;32(1):41-51.

8002. Tearne Jessica, Allen Karina, Herbison Carly, Lawrence David, Whitehouse Andrew, Sawyer Michael, Robinson Monique The association between prenatal environment and children's mental health trajectories from 2 to 14 years. EUROPEAN CHILD \& ADOLESCENT PSYCHIATRY. 2015;24(9):1015-1024.

8003. King Claire, Robinson J., Cameron Ross Flooding tolerance in four `Garrigue' landscape plants: Implications for their future use in the urban landscapes of north-west Europe?. LANDSCAPE AND URBAN PLANNING. 2012;107(2):100-110.

8004. Kujau Ariane, Heimhofer Ulrich, Ostertag-Henning Christian, Greselle Benjamin, Mutterlose Joerg No evidence for anoxia during the Valanginian carbon isotope event-An organic-geochemical study from the Vocontian Basin, SE France. GLOBAL AND PLANETARY CHANGE. 2012;92-93():92-104.

8005. Holubiec Mariana, Romero Juan, Suarez Juan, Portavella Manuel, Fernandez-Espejo Emilio, Blanco Eduardo, Galeano Pablo, Fonseca Fernando Palmitoylethanolamide prevents neuroinflammation, reduces astrogliosis and preserves recognition and spatial memory following induction of neonatal anoxia-ischemia. PSYCHOPHARMACOLOGY. 2018;235(10):2929-2945.

8006. Paajanen V, Vornanen M The induction of an ATP-sensitive K+ current in cardiac myocytes of air- and water-breathing vertebrates. PFLUGERS ARCHIV-EUROPEAN JOURNAL OF PHYSIOLOGY. 2002;444(6):760-770.

8007. Lunardi G., Parodi A., Perasso L., Pohvozcheva A., Scarrone S., Adriano E., Florio T., Gandolfo C., Cupello A., Burov S., Balestrino M. The creatine transporter mediates the uptake of creatine by brain tissue, but not the uptake of two creatine-derived compounds. NEUROSCIENCE. 2006;142(4):991-997.

8008. Cipriano Annarosa, Cella Stefania, Cotrufo Paolo Nonsuicidal Self-injury: A Systematic Review. FRONTIERS IN PSYCHOLOGY. 2017;8():.

8009. Coleman Karen, Shordon Maggie, Caparosa Susan, Pomichowski Magdalena, Dzewaltowski David The healthy options for nutrition environments in schools (Healthy ONES) group randomized trial: using implementation models to change nutrition policy and environments in low income schools. INTERNATIONAL JOURNAL OF BEHAVIORAL NUTRITION AND PHYSICAL ACTIVITY. 2012;9():.

8010. Sholeye Oluwafolahan, Animasahun Victor, Salako Albert, Oduwole Adebisi Snacking and sweetened beverage consumption among adolescents in Sagamu, Southwest Nigeria. NUTRITION \& FOOD SCIENCE. 2018;48(3):442-452.

8011. Gouttebarge Vincent, Andersen Thor, Cowie Charlotte, Goedhart Edwin, Jorstad Harald, Kemp Simon, Konigs Marsh, Maas Mario, Orhant Emmanuel, Rantanen Jussi, Salo Jari, Serratosa Luis, Stokes Keith, Tol Johannes, Verhagen Evert, Weber Alexis, Kerkhoffs Gino Monitoring the health of transitioning professional footballers: protocol of an observational prospective cohort study. BMJ OPEN SPORT \& EXERCISE MEDICINE. 2019;5(1):.

8012. Mellemkjaer Lene, Papadopoulos Fotios, Pukkala Eero, Ekbom Anders, Gissler Mika, Christensen Jane, Olsen Jorgen Cancer Incidence among Patients with Anorexia Nervosa from Sweden, Denmark and Finland. PLOS ONE. 2015;10(5):.

8013. Haerter Martin, Brandes Andreas, Hillebrandt Bernd, Lambert Martin psychenet - The Hamburg Network for Mental Health. PSYCHIATRISCHE PRAXIS. 2015;42(1):S4-S8.

8014. Puccio Francis, Kalathas Fiona, Fuller-Tyszkiewicz Matthew, Krug Isabel A revised examination of the dual pathway model for bulimic symptoms: The importance of social comparisons made on Facebook and sociotropy. COMPUTERS IN HUMAN BEHAVIOR. 2016;65():142-150.

8015. Anastasiadou Dimitra, Folkvord Frans, Serrano-Troncoso Eduardo, Lupianez-Villanueva Francisco Mobile Health Adoption in Mental Health: User Experience of a Mobile Health App for Patients With an Eating Disorder. JMIR MHEALTH AND UHEALTH. 2019;7(6):.

8016. Sadler Christina, Bonsmann Stefan, Friel Mary Coeliac disease: an overview. AGRO FOOD INDUSTRY HI-TECH. 2013;24(2):12-15.

8017. Norton Maria, Eleuteri Stefano, Cerolini Silvia, Ballesio Andrea, Conte Salvatore, Falaschi Paolo, Lucidi Fabio Is poor sleep associated with obesity in older adults? A narrative review of the literature. EATING AND WEIGHT DISORDERS-STUDIES ON ANOREXIA BULIMIA AND OBESITY. 2018;23(1):23-38.

8018. Zhang Y., Yong Y., He X., Cheng X., Yang Q., Hu X. Fraction V of bovine albumin improves the adherence and survival of adult rat cerebral cortex neurons in primary culture. AFRICAN JOURNAL OF BIOTECHNOLOGY. 2009;8(3):490-498.

8019. Carvalho Sandra, Coelho Catarina, Kluwe-Schiavon Bruno, Magalhaes Juliana, Leite Jorge The Acute Impact of the Early Stages of COVID-19 Pandemic in People with Pre-Existing Psychiatric Disorders: A Systematic Review. INTERNATIONAL JOURNAL OF ENVIRONMENTAL RESEARCH AND PUBLIC HEALTH. 2022;19(9):.

8020. Vanderkruik Rachel, Strife Samantha, Dimidjian Sona Lessons learned from training peer-leaders to conduct Body Project workshops. EATING DISORDERS. 2017;25(4):358-374.

8021. Cordero-Herrera Isabel, Guimaraes Drielle, Moretti Chiara, Zhuge Zhengbing, Han Huirong, Haworth Sarah, Gonzalez Arturo, Andersson Daniel, Weitzberg Eddie, Lundberg Jon, Carlstrom Mattias Head-to-head comparison of inorganic nitrate and metformin in a mouse model of cardiometabolic disease. NITRIC OXIDE-BIOLOGY AND CHEMISTRY. 2020;97():48-56.

8022. Han Yu, Zhang Meng, Duan Jiahui, Li Leyi, Du Jinge, Cheng Hui, Zhang Sheng, Zhai Yanhui, An Xinglan, Li Qi, Zhang Xueming, Li Ziyi, Tang Bo Maternal Prepregnancy 5-Hydroxytryptamine Exposure Affects the Early Development of the Fetus. FRONTIERS IN PHYSIOLOGY. 2022;13():.

8023. Boehm Ilka, Finke Beatrice, Tam Friederike, Fittig Eike, Scholz Michael, Gantchev Krassimir, Roessner Veit, Ehrlich Stefan Effects of perceptual body image distortion and early weight gain on long-term outcome of adolescent anorexia nervosa. EUROPEAN CHILD \& ADOLESCENT PSYCHIATRY. 2016;25(12):1319-1326.

8024. Karim Sajjad, Merdad Adnan, Schulten Hans-Juergen, Jayapal Manikandan, Dallol Ashraf, Buhmeida Abdelbaset, Al-Thubaity Fatima, Mirza Zeenat, Gari Mamdooh, Chaudhary Adeel, Abuzenadah Adel, Al-Qahtani Mohammed Low expression of leptin and its association with breast cancer: A transcriptomic study. ONCOLOGY REPORTS.

2016;36(1):43-48.

8025. Paolini B., Maltese P., Del Ciondolo I., Tavian D., Missaglia S., Ciuoli C., Zuntini M., Cecchin S., Bertelli M., Pompucci G. Prevalence of mutations in LEP, LEPR, and MC4R genes in individuals with severe obesity. GENETICS AND MOLECULAR RESEARCH. 2016;15(3):.

8026. Pamenter Matthew, Buck Leslie delta-Opioid receptor antagonism induces NMDA receptor-dependent excitotoxicity in anoxic turtle cortex. JOURNAL OF EXPERIMENTAL BIOLOGY. 2008;211(21):3512-3517.

8027. McLean Carmen, Asnaani Anu, Litz Brett, Hofmann Stefan Gender differences in anxiety disorders: Prevalence, course of illness, comorbidity and burden of illness. JOURNAL OF PSYCHIATRIC RESEARCH. 2011;45(8):1027-1035.

8028. Fraga-Ferreira Paula, Ader Magali, Caetano-Filho Sergio, Sansjofre Pierre, Paula-Santos Gustavo, Babinski Marly, Guacaneme Cristian, Bedoya-Rueda Carolina, Rojas Virginia, Reis Humberto, Kuchenbecker Matheus, Trindade Ricardo The Nitrogen Cycle in an Epeiric Sea in the Core of Gondwana Supercontinent: A Study on the Ediacaran-Cambrian Bambui Group, East-central Brazil. FRONTIERS IN EARTH SCIENCE. 2021;9():.

8029. Wiseman CV, Sunday SR, Becker AE Impact of the media on adolescent body image. CHILD AND ADOLESCENT PSYCHIATRIC CLINICS OF NORTH AMERICA. 2005;14(3):453+.

8030. Rossi Paolo, Faroni Jessica, Tassorelli Cristina, Nappi Giuseppe Advice alone versus structured detoxification programmes for complicated medication overuse headache (MOH): a prospective, randomized, open-label trial. JOURNAL OF HEADACHE AND PAIN. 2013;14():.

8031. Jones Megan, Lynch Katherine, Kass Andrea, Burrows Amanda, Williams Joanne, Wilfley Denise, Taylor C. Healthy Weight Regulation and Eating Disorder Prevention in High School Students: A Universal and Targeted Web-Based Intervention. JOURNAL OF MEDICAL INTERNET RESEARCH. 2014;16(2):.

8032. Zhang Xinyuan, Xu Jipo, Liu Yesong, Chen Shuohua, Wu Shouling, Gao Xiang Diet Quality is Associated with Prodromal Parkinson's Disease Features in Chinese Adults. MOVEMENT DISORDERS. 2022;37(12):2367-2375.

8033. Purcell Audrey, Clarke Mary, Maidment Ian Venous thromboembolism prophylaxis in mental health in-patient services: a qualitative study. INTERNATIONAL JOURNAL OF CLINICAL PHARMACY. 2018;40(3):543-549.

8034. Song C, Al-Mehdi AB, Fisher AB An immediate endothelial cell signaling response to lung ischemia. AMERICAN JOURNAL OF PHYSIOLOGY-LUNG CELLULAR AND MOLECULAR PHYSIOLOGY. 2001;281(4):L993-L1000.

8035. Coppell Kirsten, Stamm Rosemary, Sharp Kiri Diagnostic delays and treatment challenges in children with coeliac disease: The New Zealand Coeliac Health Survey. NEW ZEALAND MEDICAL JOURNAL. 2019;132(1505):29-37.

8036. Bauer Kohen, Zeebe Richard, Wortmann Ulrich Quantifying the volcanic emissions which triggered Oceanic Anoxic Event 1a and their effect on ocean acidification. SEDIMENTOLOGY. 2017;64(1, SI):204-214.

8037. Vila G, Cabrol S, Goulet O, Rigour C, MourenSimeoni MC A new form of anorexia in babies and young children: Post drip-fed food refusal.. EVOLUTION PSYCHIATRIQUE. 1995;60(4):771-781.

8038. Zucker Nancy, Bulik Cynthia On bells, saliva, and abdominal pain or discomfort: Early aversive visceral conditioning and vulnerability for anorexia nervosa. INTERNATIONAL JOURNAL OF EATING DISORDERS. 2020;53(4):508-512.

8039. Jansen Pauline, Tharner Anne, Ende Jan, Wake Melissa, Raat Hem, Hofman Albert, Verhulst Frank, Ijfzendoorn Marinus, Jaddoe Vincent, Tiemeier Henning Feeding practices and child weight: is the association bidirectional in preschool children?. AMERICAN JOURNAL OF CLINICAL NUTRITION. 2014;100(5):1329-1336.

8040. Park Subin, Cho Soo-Churl, Hong Yun-Chul, Oh Se-Young, Kim Jae-Won, Shin Min-Sup, Kim Boong-Nyun, Yoo Hee-Jeong, Cho In-Hee, Bhang Soo-Young Association between dietary behaviors and attention-deficit/hyperactivity disorder and learning disabilities in school-aged children. PSYCHIATRY RESEARCH. 2012;198(3):468-476.

8041. Crespo Hirata Rosario, Cerda Alvaro, Dalla Vecchia Genvigir Fabiana, Hiroyuki Hirata Mario Pharmacogenetic implications in the management of metabolic diseases in Brazilian populations. BRAZILIAN JOURNAL OF PHARMACEUTICAL SCIENCES. 2018;54(SI):.

8042. Blokhina O, Virolainen E, Fagerstedt KV Antioxidants, oxidative damage and oxygen deprivation stress: a review. ANNALS OF BOTANY. 2003;91(2, SI):179-194.

8043. Neumayr Christina, Voderholzer Ulrich, Tregarthen Jenna, Schlegl Sandra Improving aftercare with technology for anorexia nervosa after intensive inpatient treatment: A pilot randomized controlled trial with a therapist-guided smartphone app. INTERNATIONAL JOURNAL OF EATING DISORDERS. 2019;52(10, SI):1191-1201.

8044. Mota-Rojas D, Martinez-Burnes J, Trujillo-Ortega ME, Alonso-Spilsbury ML, Ramirez-Necoechea R, Lopez A Effect of oxytocin treatment in sows on umbilical cord morphology, meconium staining, and neonatal mortality of piglets. AMERICAN JOURNAL OF VETERINARY RESEARCH. 2002;63(11):1571-1574.

8045. Yang Joshua, Mamudu Hadii, Mackey Timothy Governing Noncommunicable Diseases Through Political Rationality and Technologies of Government: A Discourse Analysis. INTERNATIONAL JOURNAL OF ENVIRONMENTAL RESEARCH AND PUBLIC HEALTH. 2020;17(12):.

8046. Sirohi Sunil, Skripnikova Elena, Davis Jon Vertical Sleeve Gastrectomy Attenuates Hedonic Feeding Without Impacting Alcohol Drinking in Rats. OBESITY. 2019;27(4):603-611.

8047. Islam Md, Yunus Fakir, Kabir Enamul, Khanam Rasheda Evaluating Risk and Protective Factors for Suicidality and Self-Harm in Australian Adolescents With Traditional Bullying and Cyberbullying Victimizations. AMERICAN JOURNAL OF HEALTH PROMOTION. 2021;():.

8048. Pamenter Matthew Mitochondria: a multimodal hub of hypoxia tolerance. CANADIAN JOURNAL OF ZOOLOGY. 2014;92(7):569-589.

8049. Muscente A., Martindale Rowan, Schiffbauer James, Creighton Abby, Bogan Brooke TAPHONOMY OF THE LOWER JURASSIC KONSERVAT-LAGERSTATTE AT YA HA TINDA (ALBERTA, CANADA) AND ITS SIGNIFICANCE FOR EXCEPTIONAL FOSSIL PRESERVATION DURING OCEANIC ANOXIC EVENTS. PALAIOS. 2019;34(11):515-541.

8050. Tian Mengyuan, Xie Yongyan, Meng Yan, Ma Wen, Tong Zhihong, Yang Xiaomei, Lai Songqing, Zhou Yue, He Ming, Liao Zhangping Resveratrol protects cardiomyocytes against anoxia/reoxygenation via dephosphorylation of VDAC1 by Akt-GSK3 beta pathway.

EUROPEAN JOURNAL OF PHARMACOLOGY. 2019;843():80-87.

8051. Oke Shariwa, Tan Marcia Techniques for Advertising Healthy Food in School Settings to Increase Fruit and Vegetable Consumption. INQUIRY-THE JOURNAL OF HEALTH CARE ORGANIZATION PROVISION AND FINANCING. 2022;59():.

8052. Hardisty Dalton, Riedinger Natascha, Planavsky Noah, Asael Dan, Andren Thomas, Jorgensen Bo, Lyons Timothy A HOLOCENE HISTORY OF DYNAMIC WATER COLUMN REDOX CONDITIONS IN THE LANDSORT DEEP, BALTIC SEA. AMERICAN JOURNAL OF SCIENCE. 2016;316(8):713-745.

8053. Liu Yuan, Luo Di, Xu Bo The combination of molecular docking and network pharmacology reveals the molecular mechanism of Danggui Niantong decoction in treating gout. MEDICINE. 2022;101(47):.

8054. McGrath John, Saha Sukanta, Al-Hamzawi Ali, Andrade Laura, Benjet Corina, Bromet Evelyn, Browne Mark, Almeida Jose, Chiu Wai, Demyttenaere Koen, Fayyad John, Florescu Silvia, Girolamo Giovanni, Gureje Oye, Haro Josep, Have Margreet, Hu Chiyi, Kovess-Masfety Viviane, Lim Carmen, Navarro-Mateu Fernando, Sampson Nancy, Posada-Villa Jose, Kendler Kenneth, Kessler Ronald The Bidirectional Associations Between Psychotic Experiences and DSM-IV Mental Disorders. AMERICAN JOURNAL OF PSYCHIATRY. 2016;173(10):997-1006.

8055. Kardakis Therese, Jerden Lars, Nystrom Monica, Weinehall Lars, Johansson Helene Implementation of clinical practice guidelines on lifestyle interventions in Swedish primary healthcare - a two-year follow up. BMC HEALTH SERVICES RESEARCH. 2018;18():.

8056. Newby Sean, Owens Jeremy, Schoepfer Shane, Algeo Thomas Transient ocean oxygenation at end-Permian mass extinction onset shown by thallium isotopes. NATURE GEOSCIENCE. 2021;14(9):678+.

8057. Michels Catherine, Dorai Thambi, Chander Praveen, Choudhury Muhammad, Grasso Michael Hypoxic pre-conditioning in a rat renal ischemia model: an evaluation of the use of hydralazine. WORLD JOURNAL OF UROLOGY. 2009;27(6):817-823.

8058. Liang Kuei-Yu, Tseng Mei-Chih Impulsive behaviors in female patients with eating disorders in a university hospital in northern Taiwan. JOURNAL OF THE FORMOSAN MEDICAL ASSOCIATION. 2011;110(9):607-610.

8059. Serra Riccardo, Di Chiara Nicolantonio, Di Febo Riccardo, De Franco Crescenzo, Johan Vanderlinden, Elske Vrieze, Ronny Bruffaerts, Camillo Loriedo, Massimo Pasquini, Lorenzo Tarsitani The transition from restrictive anorexia nervosa to binging and purging: a systematic review and meta-analysis. EATING AND WEIGHT DISORDERS-STUDIES ON ANOREXIA BULIMIA AND OBESITY. 2022;27(3):857-865.

8060. Jorgensen Torben, Jacobsen Rikke, Toft Ulla, Aadahl Mette, Glumer Charlotte, Pisinger Charlotta Effect of screening and lifestyle counselling on incidence of ischaemic heart disease in general population: Inter99 randomised trial. BMJ-BRITISH MEDICAL JOURNAL. 2014;348():.

8061. Muzi Laura, Tieghi Laura, Rugo Michele, Lingiardi Vittorio Evaluating empirically valid and clinically meaningful change in intensive residential treatment for severe eating disorders at discharge and at a 6-month follow-up. EATING AND WEIGHT DISORDERS-STUDIES ON ANOREXIA BULIMIA AND OBESITY. 2020;25(6):1609-1620.

8062. Martz DM, Bazzini DG Eating disorders prevention programming may be failing: Evaluation of 2 one-shot programs. JOURNAL OF COLLEGE STUDENT DEVELOPMENT. 1999;40(1):32-42.

8063. Young GP, Le Leu RK Preventing cancer: dietary lifestyle or clinical intervention?. ASIA PACIFIC JOURNAL OF CLINICAL NUTRITION. 2002;11(S):S618-S631.

8064. Datar Ashlesha, Chung Paul Accuracy of Weight Perceptions in a Nationally Representative Cohort of US 8th Grade Adolescents. ACADEMIC PEDIATRICS. 2016;16(3):267-274.

8065. Neuman Manuela, Nanau Radu, Cohen Lawrence Nonmedicinal interventions in nonalcoholic fatty liver disease. CANADIAN JOURNAL OF GASTROENTEROLOGY AND HEPATOLOGY. 2015;29(5):241-252.

8066. Schloss Maximilian, Swirski Filip, Nahrendorf Matthias Modifiable Cardiovascular Risk, Hematopoiesis, and Innate Immunity. CIRCULATION RESEARCH. 2020;126(9):1242-1259.

8067. Amin Fakhra, Khan Mohd, Bano Bilqees Mammalian cystatin and protagonists in brain diseases. JOURNAL OF BIOMOLECULAR STRUCTURE \& DYNAMICS.

2020;38(7):2171-2196.

8068. Kim JS, Qian T, Lemasters JJ Mitochondrial permeability transition in the switch from necrotic to apoptotic cell death in ischemic rat hepatocytes. GASTROENTEROLOGY. 2003;124(2):494-503.

8069. Freud Amir, Sheiner Eyal, Wainstock Tamar, Landau Daniella, Walfisch Asnat Gender Affects Long-Term Neurological Outcome of Neonates. PEDIATRIC NEUROLOGY. 2017;74():68-73.

8070. Dabhadkar K., Bellam N. POLYPILL STRATEGY FOR PRIMARY PREVENTION OF CARDIOVASCULAR DISORDERS. DRUGS OF TODAY. 2013;49(5):317-324.

8071. Gupta Rajeev, Mohan Indu, Narula Jagat Trends in Coronary Heart Disease Epidemiology in India. ANNALS OF GLOBAL HEALTH. 2016;82(2):307-315.

8072. Mensah G., Mayosi B. The 2011 United Nations High-Level Meeting on Non-Communicable Diseases: The Africa agenda calls for a 5-by-5 approach. SAMJ SOUTH AFRICAN MEDICAL JOURNAL. 2013;103(2):77-79.

8073. Kumar Rajeev, Mirza Mohd, Naseef Punnoth, Kuruniyan Mohamed, Zakir Foziyah, Aggarwal Geeta Exploring the Potential of Natural Product-Based Nanomedicine for Maintaining Oral Health. MOLECULES. 2022;27(5):.

8074. Cevik Ayfer, Sahin Serap, Kocan Sema, Karaaslan Mehtap, Pekmezci Hilal, Kirbas Aynur, Ayaz Teslime Gender related differences in dietary behaviors, cardiometabolic risks, unhealthy lifestyle factors, and their effect on cardiovascular morbidity in primary care. CUKUROVA MEDICAL JOURNAL. 2022;47(2):535-547.

8075. Pomeranz Jennifer, Zellers Leslie, Bare Michael, Pertschuk Mark State Preemption of Food and Nutrition Policies and Litigation: Undermining Government's Role in Public Health. AMERICAN JOURNAL OF PREVENTIVE MEDICINE. 2019;56(1):47-57.

8076. Adesina Miracle, Oladele Ruth, Olufadewa Isaac, Onothoja Ogheneruona, Oladipo Damilola, Iyiola Opeyemi, Ekott Marvelene, Nwachukwu Pamela, Baru Ararso, Akinloye Seyi Addressing the high burden of noncommunicable diseases in Nigeria: a commentary.

JOURNAL OF HEALTH RESEARCH. 2021;35(5):457-462.

8077. Rosa A, Maury JP, Terrand J, Lyon X, Kucera P, Kappenberger L, Raddatz E Ectopic pacing at physiological rate improves postanoxic recovery of the developing heart. AMERICAN JOURNAL OF PHYSIOLOGY-HEART AND CIRCULATORY PHYSIOLOGY. 2003;284(6):H2384-H2392.

8078. Mastorci Francesca, Vassalle Cristina, Chatzianagnostou Kyriazoula, Marabotti Claudio, Siddiqui Khawer, Eba Ahmed, Mhamed Soueid, Bandopadhyay Arun, Nazzaro Marco, Passera Mirko, Pingitore Alessandro Undernutrition and Overnutrition Burden for Diseases in Developing Countries: The Role of Oxidative Stress Biomarkers to Assess Disease Risk and Interventional Strategies. ANTIOXIDANTS. 2017;6(2):.

8079. Ohrig E, Geiss HC, Haas GM, Schwandt P The Prevention Education Program (PEP) Nuremberg: design and baseline data of a family oriented intervention study. INTERNATIONAL JOURNAL OF OBESITY. 2001;25(1):S89-S92.

8080. Tauzin M., Felix A., Michot C., Dedieu C., Aouste L., Fortas F., Guillier C., Ngo J., Wachter P., Petermann L., Kermorvant-Duchemin E. About twins: Epidemiological, genetic, and obstetrical aspects, specific risks, and outcome. ARCHIVES DE PEDIATRIE. 2017;24(12):1299-1311.

8081. SANKARAN H, LARKIN EC, RAO GA INDUCTION OF MALNUTRITION IN CHRONIC-ALCOHOLISM - ROLE OF GASTRIC-EMPTYING. MEDICAL HYPOTHESES. 1994;42(2):124-128.

8082. Wang Fulong, Jia Jocelyn, Rodrigues Brian Autophagy, Metabolic Disease, and Pathogenesis of Heart Dysfunction. CANADIAN JOURNAL OF CARDIOLOGY. 2017;33(7):850-859.

8083. Sharma Hanjabam COVID-19: Lifestyle, CoVesity and Exercise-Time to Identify and Defeat the Real Culprits with Clinical Physiological Interventions. JOURNAL OF CLINICAL AND DIAGNOSTIC RESEARCH. 2022;16(11):CE1-CE11.

8084. Stewart Catherine, Goddard Elizabeth, Cakir Ziba, Hall Richard, Allen Gill Can more people be ``Happy Being Me{''}? Testing the delivery of a universal body satisfaction program by clinicians and school staff. EATING DISORDERS. 2022;30(2):182-209.

8085. Chen Rui, Xu Jiehua, She Yanling, Jiang Ting, Zhou Shanyao, Shi Huacai, Li Cheng Necrostatin-1 protects C2C12 myotubes from CoCl2-induced hypoxia. INTERNATIONAL JOURNAL OF MOLECULAR MEDICINE. 2018;41(5):2565-2572.

8086. Ambrosio-Palma Abdias, Alberto Avila-Funes Jose, Mimenza-Alvarado Alberto, Elizabeth Serralde-Zuniga Aurora, Zavala-Solares Monica, Aguilar-Navarro Sara Prevalence and Biological Correlates of Oropharyngeal Dysphagia in Outpatients of a Geriatric Evaluation Clinic: A Brief Report. GERONTOLOGY. 2022;68(6):682-685.

8087. Parashar Anupam, Willeboordse Maartje, Gupta Anmol, Schayck Onno Effect of brief interventions to promote behavior change on clinical outcomes of selected non-communicable diseases - The World Health Organization (WHO) package of essential non-communicable disease (PEN) interventions for primary health care settings- study protocol of a quasi-experimental study. CONTEMPORARY CLINICAL TRIALS. 2022;113():.

8088. Gajo Eileen, Oberwetter Jacob, Mathew Merin, Dam Moumita, Sanborn Timothy, Chehab Lynn Correlation of Sugar-Sweetened Beverage Consumption and School Free and Reduced Lunch Eligibility as a Measure of Socioeconomic Status. JOURNAL OF COMMUNITY HEALTH. 2019;44(2):307-312.

8089. Karling M., Hagglof B. Child behaviour after anaesthesia: association of socioeconomic factors and child behaviour checklist to the post-hospital behaviour questionnaire. ACTA PAEDIATRICA. 2007;96(3):418-423.

8090. Willumsen T, Ogaard B, Hansen F, Rolla G Effects from pretreatment of stannous fluoride versus sodium fluoride on enamel exposed to 0.1 M or 0.01 M hydrochloric acid. ACTA ODONTOLOGICA SCANDINAVICA. 2004;62(5):278-281.

8091. Lewis Hannah, Foye Una From prevention to peer support: a systematic review exploring the involvement of lived-experience in eating disorder interventions. MENTAL HEALTH REVIEW JOURNAL. 2022;27(1):1-17.

8092. Olivo Gaia, Gaudio Santino, Schioth Helgi Brain and Cognitive Development in Adolescents with Anorexia Nervosa: A Systematic Review of fMRI Studies. NUTRIENTS. 2019;11(8):.

8093. Flynn K, Fitzgibbon M Body image ideals of low-income African American mothers and their preadolescent daughters. JOURNAL OF YOUTH AND ADOLESCENCE.

1996;25(5):615-630.

8094. Matsumoto K., Sato S., Fujita T., Kougo T., Kobayashi T. Physiological characterization of the yellow-skinned `Koukou' apples (Malus x domestica Borkh.) retaining green color with low brix values, and approaches for decreasing their production. EUROPEAN JOURNAL OF HORTICULTURAL SCIENCE. 2021;86(4):441-449.

8095. Carey Robert, Muntner Paul, Bosworth Hayden, Whelton Paul Prevention and Control of Hypertension JACC Health Promotion Series. JOURNAL OF THE AMERICAN COLLEGE OF CARDIOLOGY. 2018;72(11):1278-1293.

8096. ElKhodor BF, Boksa P Long-term reciprocal changes in dopamine levels in prefrontal cortex versus nucleus accumbens in rats born by caesarean section compared to vaginal birth. EXPERIMENTAL NEUROLOGY. 1997;145(1):118-129.

8097. Eke Helen, Janssens Astrid, Downs Johnny, Lynn Richard, Ani Cornelius, Ford Tamsin How to measure the need for transition to adult services among young people with Attention Deficit Hyperactivity Disorder (ADHD): a comparison of surveillance versus case note review methods. BMC MEDICAL RESEARCH METHODOLOGY. 2019;19(1):.

8098. Bhattacharya Romit, Bick Alexander Clonal Hematopoiesis of Indeterminate Potential: an Expanding Genetic Cause of Cardiovascular Disease. CURRENT ATHEROSCLEROSIS REPORTS. 2021;23(11):.

8099. Idris Idayu, Azit Noor, Abdul Ghani Siti, Syed Nor Sharifah, Mohammed Nawi Azmawati A systematic review on noncommunicable diseases among working women. INDUSTRIAL HEALTH. 2021;59(3):146-160.

8100. Del Savio Lorenzo, Loi Michele, Stupka Elia Epigenetics and Future Generations. BIOETHICS. 2015;29(8):580-587.

8101. Wang Jie, Koh Hyoung-Won, Zhou Lu, Bae Ui-Jin, Lee Hwa-Suk, Bang In, Ka Sun-O, Oh Seon-Hee, Bae Eun, Park Byung-Hyun Sirtuin 2 Aggravates Postischemic Liver Injury by Deacetylating Mitogen-Activated Protein Kinase Phosphatase-1. HEPATOLOGY. 2017;65(1):225-236.

8102. Thompson Walter, Sallis Robert, Joy Elizabeth, Jaworski Carrie, Stuhr Robyn, Trilk Jennifer Exercise Is Medicine. AMERICAN JOURNAL OF LIFESTYLE MEDICINE. 2020;14(5):511-523.

8103. Ben-Tovim DI Eating disorders: outcome, prevention and treatment of eating disorders. CURRENT OPINION IN PSYCHIATRY. 2003;16(1):65-69.

8104. Antonio G, Chiara PA A natural diet versus modern Western diets? A new approach to prevent ``well-being syndromes{''}. DIGESTIVE DISEASES AND SCIENCES. 2005;50(1):1-6.

8105. Parletta Natalie, Aljeesh Yousef, Baune Bernhard Health Behaviors, Knowledge, Life Satisfaction, and Wellbeing in People with Mental Illness across Four Countries and Comparisons with Normative Sample. FRONTIERS IN PSYCHIATRY. 2016;7():.

8106. Francisco Galvan-Molina Jesus, Jimenez-Capdeville Maria, Maria Hernandez-Mata Jose, Ramon Arellano-Cano Jose Psychopathology screening in medical school students. GACETA MEDICA DE MEXICO. 2017;153(1):75-87.

8107. Wan Li-Hong, Zhang Xiao-Pei, Mo Miao-Miao, Xiong Xiao-Ni, Ou Cui-Ling, You Li-Ming, Chen Shao-Xian, Zhang Min Effectiveness of Goal-Setting Telephone Follow-Up on Health Behaviors of Patients with Ischemic Stroke: A Randomized Controlled Trial. JOURNAL OF STROKE \& CEREBROVASCULAR DISEASES. 2016;25(9):2259-2270.

8108. Guinter Mark, McLain Alexander, Merchant Anwar, Sandler Dale, Steck Susan An estrogen-related lifestyle score is associated with risk of postmenopausal breast cancer in the PLCO cohort. BREAST CANCER RESEARCH AND TREATMENT. 2018;170(3):613-622.

8109. Yang Aiping, Cao Shifeng, Yang Zhenfeng, Cai Yuting, Zheng Yonghua gamma-Aminobutyric acid treatment reduces chilling injury and activates the defence response of peach fruit. FOOD CHEMISTRY. 2011;129(4):1619-1622.

8110. VINOGRADOVA LV, KOROLEVA VI CORTICO-CAUDATE SPREADING DEPRESSION AND SEIZURE ACTIVITY-INDUCED BY SYSTEMIC DAILY PENTYLENETETRAZOL INJECTION IN RATS. ZHURNAL VYSSHEI NERVNOI DEYATELNOSTI IMENI I P PAVLOVA. 1993;43(4):683-694.

8111. Ward A, Ramsay R, Turnbull S, Steele M, Steele H, Treasure J Attachment in anorexia nervosa: A transgenerational perspective. BRITISH JOURNAL OF MEDICAL PSYCHOLOGY. 2001;74(4):497-505.

8112. Yang X., Casement M., Yokum S., Stice E. Negative affect amplifies the relation between appetitive-food-related neural responses and weight gain over three-year follow-up among adolescents. NEUROIMAGE-CLINICAL. 2019;24():.

8113. Lopez CA, Munoz A, Ballesteros BP Changing socio-verbal context in women at risk of developing alimentary problems: A relational frame approach. REVISTA LATINOAMERICANA DE PSICOLOGIA. 2005;37(2):359-378.

8114. Skoczek-Rubinska Aleksandra, Muzsik-Kazimierska Agata, Chmurzynska Agata, Jamka Malgorzata, Walkowiak Jaroslaw, Bajerska Joanna Inflammatory Potential of Diet Is Associated with Biomarkers Levels of Inflammation and Cognitive Function among Postmenopausal Women. NUTRIENTS. 2021;13(7):.

8115. Huang Xiaoyan, Lindholm Bengt, Stenvinkel Peter, Carrero Juan Dietary fat modification in patients with chronic kidney disease: n-3 fatty acids and beyond. JOURNAL OF NEPHROLOGY. 2013;26(6):960-974.

8116. Njume Collise, Donkor Osaana, Vasiljevic Todor, McAinch Andrew Consumer acceptability and antidiabetic properties of flakes and crackers developed from selected native Australian plant species. INTERNATIONAL JOURNAL OF FOOD SCIENCE AND TECHNOLOGY. 2021;56(9, SI):4484-4495.

8117. Cha EunSeok, Akazawa Margeaux, Kim Kevin, Dawkins Colleen, Lerner Hannah, Umpierrez Guillermo, Dunbar Sandra Lifestyle habits and obesity progression in overweight and obese American young adults: Lessons for promoting cardiometabolic health. NURSING \& HEALTH SCIENCES. 2015;17(4):467-475.

8118. Stenman L., Burcelin R., Lahtinen S. Establishing a causal link between gut microbes, body weight gain and glucose metabolism in humans - towards treatment with probiotics. BENEFICIAL MICROBES. 2016;7(1):11-22.

8119. Tumiel Ewa, Wichniak Adam, Jarema Marek, Lew-Starowicz Michal Nonpharmacological Interventions for the Treatment of Cardiometabolic Risk Factors in

People With Schizophrenia-A Systematic Review. FRONTIERS IN PSYCHIATRY. 2019;10():.

8120. Tobin Leah, Sears Christopher, Ranson Kristin Two Eating Disorder Preventive Interventions Reduce Attentional Biases in Body-Dissatisfied University Women: A Cluster Randomized Controlled Trial. JOURNAL OF CONSULTING AND CLINICAL PSYCHOLOGY. 2022;():.

8121. Lin Chia-Yen, Yeh Wei-Ju How Does Health-Related Advertising with a Regulatory Focus and Goal Framing Affect Attitudes toward Ads and Healthy Behavior Intentions?. INTERNATIONAL JOURNAL OF ENVIRONMENTAL RESEARCH AND PUBLIC HEALTH. 2017;14(12):.

8122. Batal Malek, Makvandi Ewa, Imbeault Pascal, Gagnon-Arpin Isabelle, Grenier Jean, Chomienne Marie-Helene, Bouchard Louise Comparison of Dietary Intake Between Francophones and Anglophones in Canada: Data From CCHS 2.2. CANADIAN JOURNAL OF PUBLIC HEALTH-REVUE CANADIENNE DE SANTE PUBLIQUE. 2013;104(6, 1):S31-S38.

8123. Hagmar Magnus, Hirschberg Angelica, Berglund Lukas, Berglund Bo Special attention to the weight-control strategies employed by Olympic athletes striving for leanness is required. CLINICAL JOURNAL OF SPORT MEDICINE. 2008;18(1):5-9.

8124. Coker Elise, Abraham Suzanne Body weight dissatisfaction: A comparison of women with and without eating disorders. EATING BEHAVIORS. 2014;15(3):453-459.

8125. Gulec Hayriye, Moessner Markus, Mezei Agnes, Kohls Elisabeth, Tury Ferenc, Bauer Stephanie Internet-Based Maintenance Treatment for Patients With Eating Disorders. PROFESSIONAL PSYCHOLOGY-RESEARCH AND PRACTICE. 2011;42(6, SI):479-486.

8126. Sithey Gyambo, Wen Li, Dzed Laigden, Li Mu Noncommunicable diseases risk factors in Bhutan: A secondary analysis of data from Bhutan's nationwide STEPS survey 2014. PLOS ONE. 2021;16(9):.

8127. Thomas FJ, Wiles CM Dysphagia and nutritional status in multiple sclerosis. JOURNAL OF NEUROLOGY. 1999;246(8):677-682.

8128. Thomas J., Roberto C., Brownell K. Eighty-five per cent of what? Discrepancies in the weight cut-off for anorexia nervosa substantially affect the prevalence of underweight.

PSYCHOLOGICAL MEDICINE. 2009;39(5):833-843.

8129. Shi Yanjun, Rehman Hasibur, Ramshesh Venkat, Schwartz Justin, Liu Qinlong, Krishnasamy Yasodha, Zhang Xun, Lemasters John, Smith Charles, Zhong Zhi Sphingosine kinase-2 inhibition improves mitochondrial function and survival after hepatic ischemia-reperfusion. JOURNAL OF HEPATOLOGY. 2012;56(1):137-145.

8130. Xie Xingbin, Einhorn Todd, Wang Yan Inhibition of Ethylene Biosynthesis and Associated Gene Expression by Aminoethoxyvinylglycine and 1-Methylcyclopropene and Their Consequences on Eating Quality and Internal Browning of `Starkrimson' Pears. JOURNAL OF THE AMERICAN SOCIETY FOR HORTICULTURAL SCIENCE. 2015;140(6):587-596.

8131. Menakaya Nnamdi, Menakaya Ifeoma Qualitative study exploring perceptions, attitudes and practices of adolescent university students in Lagos, Nigeria, towards a healthy lifestyle. AFRICAN JOURNAL OF PRIMARY HEALTH CARE \& FAMILY MEDICINE. 2022;14(1):.

8132. Rajaobelina Kalina, Dow Courtney, Romana Mancini Francesca, Dartois Laureen, Boutron-Ruault Marie-Christine, Balkau Beverley, Bonnet Fabrice, Fagherazzi Guy Population attributable fractions of the main type 2 diabetes mellitus risk factors in women: Findings from the French E3N cohort. JOURNAL OF DIABETES. 2019;11(3):242-253.

8133. Asiki Gershim, Wanjohi Milkah, Barnes Amy, Bash Kristin, Muthuri Stella, Amugsi Dickson, Doughman Danielle, Kimani Elizabeth, Vandevijvere Stefanie, Holdsworth Michelle Benchmarking food environment policies for the prevention of diet-related non-communicable diseases in Kenya: National expert panel's assessment and priority recommendations. PLOS ONE. 2020;15(8):.

8134. Nicolaou Patrisia, Merwin Rhonda, Karekla Maria Acceptability and feasibility of a gamified digital eating disorder early-intervention program (AcceptME) based on Acceptance and Commitment Therapy (ACT). JOURNAL OF CONTEXTUAL BEHAVIORAL SCIENCE. 2022;25():26-34.

8135. Naganska Ewa, Matyja Ewa Apoptotic neuronal changes enhanced by zinc chelator-TPEN in organotypic rat hippocampal cultures exposed to anoxia. FOLIA NEUROPATHOLOGICA. 2006;44(2):125-132.

8136. Mayega Roy, Etajak Samuel, Rutebemberwa Elizeus, Tomson Goran, Kiguli Juliet `Change means sacrificing a good life': perceptions about severity of type 2 diabetes and preventive lifestyles among people afflicted or at high risk of type 2 diabetes in Iganga Uganda. BMC PUBLIC HEALTH. 2014;14():.

8137. Hansson L., Lind T., Ohlund I., Wiklund U., Rydberg A. Increased abdominal fat mass and high fat consumption in young school children with congenital heart disease: results from a case-control study. JOURNAL OF HUMAN NUTRITION AND DIETETICS. 2020;33(4):566-573.

8138. Nagourney Emily, Goodman Dina, Lam Yukyan, Hurley Kristen, Henderson Janice, Surkan Pamela Obese women's perceptions of weight gain during pregnancy: a theory-based analysis. PUBLIC HEALTH NUTRITION. 2019;22(12):2228-2236.

8139. Song Peng-kun, Li Hong, Man Qing-qing, Jia Shan-shan, Li Li-xiang, Zhang Jian Trends in Determinants of Hypercholesterolemia among Chinese Adults between 2002 and 2012: Results from the National Nutrition Survey. NUTRIENTS. 2017;9(3):.

8140. Albishi Ahdab, Bardisi Widad HEALTHY LIFESTYLE AMONG GOVERNMENTAL PRIMARY HEALTH CARE CENTER WORKERS IN JEDDAH, 2018. INDO AMERICAN JOURNAL OF PHARMACEUTICAL SCIENCES. 2019;6(6):13320-13331.

8141. Hjorth Peter, Juel Anette, Hansen Mette, Madsen Nikolaj, Viuff Anne, Munk-Jorgensen Povl Reducing the Risk of Cardiovascular Diseases in Non-selected Outpatients With Schizophrenia: A 30-Month Program Conducted in a Real-life Setting. ARCHIVES OF PSYCHIATRIC NURSING. 2017;31(6):602-609.

8142. Bell V., Silva C., Guina J., Fernandes T. Mushrooms as future generation healthy foods. FRONTIERS IN NUTRITION. 2022;9():.

8143. Cole Judith, Smith Susan, Hart Nigel, Cupples Margaret Do practitioners and friends support patients with coronary heart disease in lifestyle change? a qualitative study. BMC FAMILY PRACTICE. 2013;14():.

8144. Figueiredo Preza Bertin Cilce, Rezende Magda, Sigulem Dirce, Morais Tania Hurdles at work: perceptions of hospital food handlers. HUMAN RESOURCES FOR HEALTH. 2009;7():.

8145. Wirnitzer Katharina, Tanous Derrick, Motevalli Mohamad, Goebel Georg, Wirnitzer Gerold, Drenowatz Clemens, Ruedl Gerhard, Cocca Armando, Kirschner Werner Study protocol of ``From Science 2 School{''}-prevalence of sports and physical exercise linked to omnivorous, vegetarian and vegan, diets among Austrian secondary schools. FRONTIERS IN SPORTS AND ACTIVE LIVING. 2022;4():.

8146. Arora Dev, Devi R., Priya A. Knowledge and Awareness about the Connection between Lifestyle and Dementia among Adolescents. JOURNAL OF PHARMACEUTICAL RESEARCH INTERNATIONAL. 2021;33(47B):313-321.

8147. Barash M. Causes of the Great Mass Extinction of Marine Organisms in the Late Devonian. OCEANOLOGY. 2016;56(6):863-875.

8148. Kaprelyants L., Yegorova A., Trufkati L., Pozhitkova L. FUNCTIONAL FOODS: PROSPECTS IN UKRAINE. JOURNAL OF FOOD SCIENCE AND TECHNOLOGY-UKRAINE. 2019;13(2):15-23.

8149. Chonaill Doireann, Huggins Michaela, McHugh Catherine, Keaver Laura Nutrition knowledge related to chronic disease of Irish medical and nursing students. INTERNATIONAL JOURNAL OF HEALTH PROMOTION AND EDUCATION. 2022;():.

8150. Veromaa Veera, Kautiainen Hannu, Juonala Markus, Rantanen Ansa, Korhonen Paivi Self-rated health as an indicator of ideal cardiovascular health among working-aged women. SCANDINAVIAN JOURNAL OF PRIMARY HEALTH CARE. 2017;35(4):322-328.

8151. Debruyn Adrian, Trudel Marc, Eyding Nicola, Harding Joel, McNally Heather, Mountain Robert, Orr Craig, Urban Diane, Verenitch Sergei, Mazumder Asit Ecosystemic effects of salmon farming increase mercury contamination in wild fish. ENVIRONMENTAL SCIENCE \& TECHNOLOGY. 2006;40(11):3489-3493.

8152. Guillien Alicia, Bedard Annabelle, Dumas Orianne, Allegre Julien, Arnault Nathalie, Bochaton Audrey, Druesne-Pecollo Nathalie, Dumay Dorothee, Fezeu Leopold, Hercberg Serge, Le Moual Nicole, Pilkington Hugo, Rican Stephane, Sit Guillaume, Edelenyi Fabien, Touvier Mathilde, Galan Pilar, Feuillet Thierry, Varraso Raphaelle, Siroux Valerie Exposome Profiles and Asthma among French Adults. AMERICAN JOURNAL OF RESPIRATORY AND CRITICAL CARE MEDICINE. 2022;206(10):1208-1219.

8153. Pedley Rebecca, Lovell Karina, Bee Penny, Bradshaw Tim, Gellatly Judith, Ward Kate, Woodham Adrine, Wearden Alison Collaborative, individualised lifestyle interventions are acceptable to people with first episode psychosis; a qualitative study. BMC PSYCHIATRY. 2018;18():.

8154. Mahfouz Eman, Sadek Refaat, Abdel-Latief Wafaa, Mosallem Fadia, Hassan Ebtesam THE ROLE OF DIETARY AND LIFESTYLE FACTORS IN THE DEVELOPMENT OF COLORECTAL CANCER: CASE CONTROL STUDY IN MINIA, EGYPT. CENTRAL EUROPEAN JOURNAL OF PUBLIC HEALTH. 2014;22(4):215-222.

8155. Parekh Sanjoti, Vandelanotte Corneel, King David, Boyle Frances Improving diet, physical activity and other lifestyle behaviours using computer-tailored advice in general practice: a randomised controlled trial. INTERNATIONAL JOURNAL OF BEHAVIORAL NUTRITION AND PHYSICAL ACTIVITY. 2012;9():.

8156. Torma Johanna, Lundqvist Robert, Eliasson Mats, Nilsson Lena, Oskarsson Viktor, Wennberg Maria Comparison of dietary trends between two counties with and without a cardiovascular prevention programme: a population-based cross-sectional study in northern Sweden. PUBLIC HEALTH NUTRITION. 2022;25(7):1835-1843.

8157. Aaby Anna, Friis Karina, Christensen Bo, Rowlands Gill, Maindal Helle Health literacy is associated with health behaviour and self-reported health: A large population-based study in individuals with cardiovascular disease. EUROPEAN JOURNAL OF PREVENTIVE CARDIOLOGY. 2017;24(17):1880-1888.

8158. Fichter M, Cebulla M Self-help organizations and groups in behavioral medicine: Overview and description. VERHALTENSTHERAPIE. 2001;11(2):144-165.

8159. Simpson William, Frey Benicio, Steiner Meir Mild Depressive Symptoms During the Third Trimester of Pregnancy Are Associated with Disruptions in Daily Rhythms but Not Subjective Sleep Quality. JOURNAL OF WOMENS HEALTH. 2016;25(6):594-598.

8160. Li Shirley, Lam Siu, Yu Mandy, Zhang Jihui, Wing Yun Nocturnal Sleep Disturbances as a Predictor of Suicide Attempts Among Psychiatric Outpatients: A Clinical, Epidemiologic, Prospective Study. JOURNAL OF CLINICAL PSYCHIATRY. 2010;71(11):1440-1446.

8161. Rousseau A, Knotter A, Barbe P, Raich RM, Chabrol H Validation of the french version of the Body Shape Questionnaire. ENCEPHALE-REVUE DE PSYCHIATRIE CLINIQUE

BIOLOGIQUE ET THERAPEUTIQUE. 2005;31(2):162+.

8162. Zilius M., Giordani G., Petkuviene J., Lubiene I., Ruginis T., Bartoli M. Phosphorus mobility under short-term anoxic conditions in two shallow eutrophic coastal systems (Curonian and Sacca di Goro lagoons). ESTUARINE COASTAL AND SHELF SCIENCE. 2015;164():134-146.

8163. Meller Robert, Simon Roger A critical review of mechanisms regulating remote preconditioning-induced brain protection. JOURNAL OF APPLIED PHYSIOLOGY. 2015;119(10):1135-1142.

8164. Balestrino M, Lensman M, Parodi M, Perasso L, Rebaudo R, Melani R, Polenov S, Cupello A Role of creatine and phosphocreatine in neuronal protection from anoxic and ischemic damage. AMINO ACIDS. 2002;23(1-3):221-229.

8165. Mendoza-Jimenez Maria-Jose, Hannemann Tessa-Virginia, Atzendorf Josefine Behavioral Risk Factors and Adherence to Preventive Measures: Evidence From the Early Stages of the COVID-19 Pandemic. FRONTIERS IN PUBLIC HEALTH. 2021;9():.

8166. Vreijling Sarah, Penninx Brenda, Bot Mariska, Watkins Ed, Owens Matthew, Kohls Elisabeth, Hegerl Ulrich, Roca Miquel, Gili Margalida, Brouwer Ingeborg, Visser Marjolein, Beekman Aartjan, Jansen Rick, Lamers Femke Effects of dietary interventions on depressive symptom profiles: results from the MooDFOOD depression prevention study. PSYCHOLOGICAL MEDICINE. 2022;52(15):3580-3589.

8167. Armstrong William, Beckett Peter, Colmer Timothy, Setter Timothy, Greenway Hank Tolerance of roots to low oxygen: `Anoxic' cores, the phytoglobin-nitric oxide cycle, and energy or oxygen sensing. JOURNAL OF PLANT PHYSIOLOGY. 2019;239():92-108.

8168. Underwood Mair Body as choice or body as compulsion: An experiential perspective on body-self relations and the boundary between normal and pathological. HEALTH SOCIOLOGY REVIEW. 2013;22(4):377-388.

8169. Wood Sophie, Marchant Amanda, Allsopp Mark, Wilkinson Kathleen, Bethel Jackie, Jones Hywel, John Ann Epidemiology of eating disorders in primary care in children and young people: a Clinical Practice Research Datalink study in England. BMJ OPEN. 2019;9(8):.

8170. Nalbant Kevser, Kalayci Bilge, Akdemir Devrim, Akgul Sinem, Kanbur Nuray Emotion regulation, emotion recognition, and empathy in adolescents with anorexia nervosa. EATING AND WEIGHT DISORDERS-STUDIES ON ANOREXIA BULIMIA AND OBESITY. 2019;24(5):825-834.

8171. Morrison-Beedy Dianne, Melnyk Bernadette Making a Case for Integrating Evidence-Based Sexual Risk Reduction and Mental Health Interventions for Adolescent Girls. ISSUES IN MENTAL HEALTH NURSING. 2019;40(11):932-941.

8172. Rommel N., Tack J., Arts J., Caenepeel P., Bisschops R., Sifrim D. Rumination or belching-regurgitation? Differential diagnosis using oesophageal impedance-manometry. NEUROGASTROENTEROLOGY AND MOTILITY. 2010;22(4):e97-e104.

8173. Kochar Tanureet, Dhingra Parminder, Khaliq Muhammad, Mcjunkin Brittain Eosinophilic esophagitis presenting with spontaneous esophageal rupture: a case report. JOURNAL OF MEDICAL CASE REPORTS. 2019;13(1):.

8174. Leick Christian, Larsen Lars, Larrabee Sonderlund Anders, Svensson Nanna, Sondergaard Jens, Thilsing Trine Non-participation in a targeted prevention program aimed at lifestyle-related diseases: a questionnaire-based assessment of patient-reported reasons. BMC PUBLIC HEALTH. 2022;22(1):.

8175. Hoey Hilary Management of obesity in children differs from that of adults. PROCEEDINGS OF THE NUTRITION SOCIETY. 2014;73(4):519-525.

8176. Axpe Inge, Goni Alfredo, Infante Guillermo EFFICACY OF A COGNITIVE PROGRAM FOR IMPROVING THE PHYSICAL SELF-CONCEPT OF UNIVERSITY STUDENTS. PERCEPTUAL AND MOTOR SKILLS. 2013;117(3):720-732.

8177. Barlow P., Thow A. Neoliberal discourse, actor power, and the politics of nutrition policy: A qualitative analysis of informal challenges to nutrition labelling regulations at the World Trade Organization, 2007-2019. SOCIAL SCIENCE \& MEDICINE. 2021;273():.

8178. Juma Pamela, Mohamed Shukri, Mwagomba Beatrice, Ndinda Catherine, Mapa-tassou Clarisse, Oluwasanu Mojisola, Oladepo Oladimeji, Abiona Opeyemi, Nkhata Misheck, Wisdom Jennifer, Mbanya Jean-Claude Non-communicable disease prevention policy process in five African countries authors. BMC PUBLIC HEALTH. 2018;18(1):.

8179. Akinosun Adewale, Polson Rob, Diaz-Skeete Yohanca, De Kock Johannes, Carragher Lucia, Leslie Stephen, Grindle Mark, Gorely Trish Digital Technology Interventions for Risk Factor Modification in Patients With Cardiovascular Disease: Systematic Review and Meta-analysis. JMIR MHEALTH AND UHEALTH. 2021;9(3):.

8180. Tsuruga Koji, Sugawara Norio, Sato Yasushi, Saito Manabu, Furukori Hanako, Nakagami Taku, Nakamura Kazuhiko, Takahashi Ippei, Nakaji Shigeyuki, Yasui-Furukori Norio Dietary patterns and schizophrenia: a comparison with healthy controls. Neuropsychiatric Disease and Treatment. 2015;11():1115-1120.

8181. Gorwood P, Ades J, Bellodi L, Cellini E, Collier DA, Di Bella D, Di Bernardo M, Estivill X, Fernandez-Aranda F, Gratacos M, Hebebrand J, Hinney A, Hu X, Karwautz A, Kipman A, Mouren-Simeoni MC, Nacmias B, Ribases M, Remschmidt H, Ricca V, Rotella CM, Sorbi S, Treasure J The 5-HT2A-1438G/A polymorphism in anorexia nervosa: a combined analysis of 316 trios from six European centres. MOLECULAR PSYCHIATRY. 2002;7(1):90-94.

8182. Arendt Florian, Peter Christina, Beck Julia Idealized Female Beauty, Social Comparisons, and Awareness Intervention Material Evidence for Preventive Effects in Young Women. JOURNAL OF MEDIA PSYCHOLOGY-THEORIES METHODS AND APPLICATIONS. 2017;29(4):188-197.

8183. Sahoo Jyotiranjan, Mohanty Sambedana, Kundu Arijit, Epari Venkatarao Medication Adherence Among Patients of Type II Diabetes Mellitus and Its Associated Risk Factors: A Cross-Sectional Study in a Tertiary Care Hospital of Eastern India. CUREUS JOURNAL OF MEDICAL SCIENCE. 2022;14(12):.

8184. Gesualdo Nicole, Yanovitzky Itzhak Advertising Susceptibility and Youth Preference for and Consumption of Sugar-Sweetened Beverages: Findings from a National Survey. JOURNAL OF NUTRITION EDUCATION AND BEHAVIOR. 2019;51(1):16-22.

8185. Mathew Shycil, Dsouza Jenifer, Saldanha Prakash Lifestyle Practices and Knowledge on Prevention and Control of Overweight and Obesity among Adolescents: A Cross-sectional Study. JOURNAL OF CLINICAL AND DIAGNOSTIC RESEARCH. 2021;15(9):LC01-LC5.

8186. Dinkelborg LM, Kinne RKH, Grieshaber MK Transport and metabolism of L-glutamate during oxygenation, anoxia, and reoxygenation of rat cardiac myocytes. AMERICAN JOURNAL OF PHYSIOLOGY-HEART AND CIRCULATORY PHYSIOLOGY. 1996;270(5):H1825-H1832.

8187. Grammer Anne, Byrne Meghan, Pearlman Arielle, Klein David, Schvey Natasha Overweight and obesity in sexual and gender minority adolescents: A systematic review. OBESITY REVIEWS. 2019;20(10):1350-1366.

8188. Diedrichs Phillippa, Lee Christina, Kelly Marguerite Seeing the beauty in everyday people: A qualitative study of young Australians' opinions on body image, the mass media and models. BODY IMAGE. 2011;8(3):259-266.

8189. Lundahl Alyssa, Kidwell Katherine, Nelson Timothy Parental Misperceptions of Children's Underweight Status: A Meta-analysis. ANNALS OF BEHAVIORAL MEDICINE. 2014;48(2):184-193.

8190. Hilibrand Miryl, Hammoud Sommer, Bishop Meghan, Woods Daniel, Fredrick Robert, Dodson Christopher Common injuries and ailments of the female athlete; pathophysiology, treatment and prevention. PHYSICIAN AND SPORTSMEDICINE. 2015;43(4):403-411.

8191. Lee Chia-Kuei, Liao Li-Ling Feasibility of Intervention Program to Prevent Adolescent Health-Compromising Behaviors. JOURNAL OF SCHOOL NURSING. 2021;():.

8192. Schulze Ulrike, Keller Ferdinand Weight phobia in patients with Anorexia nervosa - development of a questionnaire. ZEITSCHRIFT FUR KINDER-UND JUGENDPSYCHIATRIE UND PSYCHOTHERAPIE. 2009;37(3):195-202.

8193. Sorscher Adam How is your sleep: A neglected topic for health care screening. JOURNAL OF THE AMERICAN BOARD OF FAMILY MEDICINE. 2008;21(2):141-148.

8194. Sajwani Najla, Qawas Ahmed, Al Ali Nouf, Sajwani Fatma, Alrustamani Asma, Al Maamari Shamma, Al Mazrouei Shereena, Al Shehhi Budoor, Al Rand Hussain, Fikri Asma The effect of lockdowns and distant learning on the health-related behaviours of school students in the United Arab Emirates. BMC PRIMARY CARE. 2022;23(1):.

8195. Mussener Ulrika, Lof Marie, Bendtsen Preben, Bendtsen Marcus Using Mobile Devices to Deliver Lifestyle Interventions Targeting At-Risk High School Students: Protocol for a Participatory Design Study. JMIR RESEARCH PROTOCOLS. 2020;9(1):.

8196. Gersh Elon, Arghira Adriana, Richardson Laura, Katzman Katherine, Sucato Gina, McCarty Carolyn Comparison of Health Risks among Adolescents from School-based Health

Centers and Community-based Primary Care Settings. HEALTH BEHAVIOR AND POLICY REVIEW. 2019;6(1):71-78.

8197. Millar L., Kremer P., Silva-Sanigorski A., McCabe M., Mavoa H., Moodie M., Utter J., Bell C., Malakellis M., Mathews L., Roberts G., Robertson N., Swinburn B. Reduction in overweight and obesity from a 3-year community-based intervention in Australia: the `It's Your Move!' project. OBESITY REVIEWS. 2011;12(2, SI):20-28.

8198. SCHWARZ B, BISCHOF HP, KUNZE M COFFEE, TEA, AND LIFE-STYLE. PREVENTIVE MEDICINE. 1994;23(3):377-384.

8199. Antunez Lucia, Alcaire Florencia, Brunet Geronimo, Bove Isabel, Ares Gaston COVID-washing of ultra-processed products: the content of digital marketing on Facebook during the COVID-19 pandemic in Uruguay. PUBLIC HEALTH NUTRITION. 2021;24(5):1142-1152.

8200. Gamage A., Jayawardana P. Knowledge of non-communicable diseases and practices related to healthy lifestyles among adolescents, in state schools of a selected educational division in Sri Lanka. BMC PUBLIC HEALTH. 2017;18():.

8201. Shriver Lenka, Harrist Amanda, Page Melanie, Hubbs-Tait Laura, Moulton Michelle, Topham Glade Differences in body esteem by weight status, gender, and physical activity among young elementary school-aged children. BODY IMAGE. 2013;10(1):78-84.

8202. Vassiliou Vassilios, Tsampasian Vasiliki, Abreu Ana, Kurpas Donata, Cavarretta Elena, O'Flaherty Martin, Colombet Zoe, Siegrist Monika, De Smedt Delphine, Marques-Vidal Pedro Promotion of healthy nutrition in primary and secondary cardiovascular disease prevention: a clinical consensus statement from the European Association of Preventive Cardiology. EUROPEAN JOURNAL OF PREVENTIVE CARDIOLOGY. 2023;30(8, SI):696-706.

8203. Mitchell Brittany, Campos Adrian, Renteria Miguel, Parker Richard, Sullivan Lenore, McAloney Kerrie, Couvy-Duchesne Baptiste, Medland Sarah, Gillespie Nathan, Scott Jan, Zietsch Brendan, Lind Penelope, Martin Nicholas, Hickie Ian Twenty-Five and Up (25Up) Study: A New Wave of the Brisbane Longitudinal Twin Study. TWIN RESEARCH AND HUMAN GENETICS. 2019;22(3):154-163.

8204. Cole Amanda, Vidgen Helen, Cleland Phoebe Food provision in early childhood education and care services: Exploring how staff determine nutritional adequacy.

NUTRITION \& DIETETICS. 2017;74(1):105-110.

8205. Chen Wenhan, Kemp David, Newton Robert, He Tianchen, Huang Chunju, Cho Tenichi, Izumi Kentaro Major sulfur cycle perturbations in the Panthalassic Ocean across the Pliensbachian-Toarcian boundary and the Toarcian Oceanic Anoxic Event. GLOBAL AND PLANETARY CHANGE. 2022;215():.

8206. Kurpas Donata, Szwamel Katarzyna, Mroczek Bozena Importance of Social Relationships in Patients with Chronic Respiratory Diseases. . 2016;935():63-73.

8207. Kumar Sanjeev, Kasseckert Sascha, Kostin Sawa, Abdallah Yaser, Schafer Claudia, Kaminski Alexander, Reusch H., Piper Hans, Steinhoff Gustav, Ladilov Yury Ischemic acidosis causes apoptosis in coronary endothelial cells through activation of caspase-12. CARDIOVASCULAR RESEARCH. 2007;73(1):172-180.

8208. Berg AL, Spitzer JB, Towers HM, Bartosiewicz C, Diamond BE Newborn hearing screening in the NICU: Profile of failed auditory brainstem response/passed otoacoustic emission. PEDIATRICS. 2005;116(4):933-938.

8209. Laar Amos, Addo Phyllis, Aryeetey Richmond, Agyemang Charles, Zotor Francis, Asiki Gershim, Rampalli Krystal, Amevinya Gideon, Tandoh Akua, Nanema Silver, Adjei Akosua, Laar Matilda, Mensah Kobby, Laryea Dennis, Sellen Daniel, Vandevijvere Stefanie, Turner Christopher, Osei-Kwasi Hibbah, Spires Mark, Blake Christine, Rowland Dominic, Kadiyala Suneetha, Madzorera Isabel, Diouf Adama, Covic Namukolo, Dzudzor Isaac, Annan Reginald, Milani Peiman, Nortey John, Bricas Nicholas, Mphumuzi Sukati, Anchang Kenneth, Jafri Ali, Dhall Meenal, Lee Amanda, Mackay Sally, Oti Samuel, Hofman Karen, Frongillo Edward, Holdsworth Michelle Perspective: Food Environment Research Priorities for Africa-Lessons from the Africa Food Environment Research Network. ADVANCES IN NUTRITION. 2022;13(3):739-747.

8210. Pinillos-Patino Yisel, Herazo-Beltran Yaneth, Rodriguez-Cordero Orlando, Escorcia-Bermejo Amada, Martelo-Lopez Enrique, Armando Vidarte-Claros Jose, Vanegas Garcia Jose, Cortes Moreno Gabriela User Preferences Related to Multimedia Elements of a Mobile Application to Prevent Diabetes. HEALTHCARE INFORMATICS RESEARCH. 2020;26(4):295-302.

8211. Flament MF, Cohen D, Choquet M, Jeammet P, Ledoux S Phenomenology, psychosocial. correlates, and treatment seeking in major depression and dysthymia of adolescence. JOURNAL OF THE AMERICAN ACADEMY OF CHILD AND ADOLESCENT PSYCHIATRY.

2001;40(9):1070-1078.

8212. Tuschen-Caffier B Eating disorders and obesity in childhood and adolescence. KINDHEIT UND ENTWICKLUNG. 2005;14(4):201-208.

8213. Smithers Lisa, Haag Dandara, Agnew Benjamin, Lynch John, Sorell Matthew Food advertising on Australian television: Frequency, duration and monthly pattern of advertising from a commercial network (four channels) for the entire 2016. JOURNAL OF PAEDIATRICS AND CHILD HEALTH. 2018;54(9):962-967.

8214. Lopes Coura Amanda, Arruda Neta Adelia, Lima Rafaela, Bersch-Ferreira Angela, Weber Bernardete, Toledo Vianna Rodrigo Tracking of Dietary Patterns in the Secondary Prevention of Cardiovascular Disease after a Nutritional Intervention Program-A Randomized Clinical Trial. NUTRIENTS. 2022;14(22):.

8215. Howard Janna, Skinner Asheley, Ravanbakht Sophie, Brown Jane, Perrin Andrew, Steiner Michael, Perrin Eliana Obesogenic Behavior and Weight-Based Stigma in Popular Children's Movies, 2012 to 2015. PEDIATRICS. 2017;140(6):.

8216. Martini Matteo, Marzola Enrica, Musso Maria, Brustolin Annalisa, Abbate-Daga Giovanni Association of emotion recognition ability and interpersonal emotional competence in anorexia nervosa: A study with a multimodal dynamic task. INTERNATIONAL JOURNAL OF EATING DISORDERS. 2022;():.

8217. Butt Nazish, Khan Muhammad, Rai Lajpat, Channa Riaz, Khemani Hanisha, Abbasi Amanullah Perception of Non-Alcoholic Fatty Liver Disease: Real-Life Experience From Pakistan. CUREUS. 2021;13(6):.

8218. Wang Lu, Li Yuanyuan, Liu Yan, Zhang Huanwen, Qiao Tingting, Chu Lei, Luo Tao, Zhang Zewen, Dai Jianghong Association between Different Types of Plant-Based Diets and Dyslipidemia in Middle-Aged and Elderly Chinese Participants. NUTRIENTS. 2023;15(1):.

8219. Nevsimalova Sona Narcolepsy in childhood. SLEEP MEDICINE REVIEWS. 2009;13(2):169-180.

8220. Rose CR, Waxman SG, Ransom BR Effects of glucose deprivation, chemical hypoxia, and simulated ischemia on Na+ homeostasis in rat spinal cord astrocytes. JOURNAL OF

NEUROSCIENCE. 1998;18(10):3554-3562.

8221. Jimenez Candel M., Carpena Lucas Pedro, Ceballos-Santamaria Guillermo, Mondejar Jimenez Jose Design and validation of a questionnaire to study healthy habits among adolescents aged 12-14 years. ARCHIVOS ARGENTINOS DE PEDIATRIA. 2021;119(3):177-184.

8222. Araque-Padilla Rafael, Villegas-Navas Victoria, Montero-Simo Maria-Jose Non-Branded Food Placements in Children's Entertainment Programs: A Content Analysis. HEALTH COMMUNICATION. 2019;34(10):1222-1229.

8223. Yijun Luo, Gengfeng Mu, Hong Chen Early life environmental unpredictability and overeating: Based on life history theory. ACTA PSYCHOLOGICA SINICA. 2020;52(10):1224-1236.

8224. Zhang Lirong, Zhao Shaocong, Lin Qiong, Song Minmin, Wu Shouren, Zheng Hua Algorithms to Predict Anxiety and Depression Among University Students in China After Analyzing Lifestyles and Sport Habits. NEUROPSYCHIATRIC DISEASE AND TREATMENT. 2021;17():2011-2025.

8225. Cheema H., Arora Rajiv Prevalence and Clinical Profile of Patients with Polycystic Ovary Syndrome-A Hospital Based Study. JOURNAL OF EVOLUTION OF MEDICAL AND DENTAL SCIENCES-JEMDS. 2019;8(32):2573-2576.

8226. Mircica E, Clutton RE, Kyles KW, Blissitt KJ Problems associated with perioperative morphine in horses: a retrospective case analysis. VETERINARY ANAESTHESIA AND ANALGESIA. 2003;30(3):147-155.

8227. Bartoli Marco, Benelli Sara, Lauro Marta, Magri Monia, Vybernaite-Lubiene Irma, Petkuviene Jolita Variable Oxygen Levels Lead to Variable Stoichiometry of Benthic Nutrient Fluxes in a Hypertrophic Estuary. ESTUARIES AND COASTS. 2021;44(3):689-703.

8228. Lu Yapeng, Kan Huiwen, Wang Ying, Wang Dan, Wang Xueting, Gao Jing, Zhu Li Asiatic acid ameliorates hepatic ischemia/reperfusion injury in rats via mitochondria-targeted protective mechanism. TOXICOLOGY AND APPLIED PHARMACOLOGY. 2018;338():214-223.

8229. Le Long, Hay Phillipa, Wade Tracey, Touyz Stephen, Mihalopoulos Cathrine The cost-effectiveness of cognitive behavioral therapy for bulimia nervosa in the Australian context. INTERNATIONAL JOURNAL OF EATING DISORDERS. 2017;50(12):1367-1377.

8230. Anderson CB, Joyce PR, Carter FA, McIntosh VV, Bulik CM The effect of cognitive-behavioral therapy for bulimia nervosa on temperament and character as measured by the temperament and character inventory. COMPREHENSIVE PSYCHIATRY. 2002;43(3):182-188.

8231. Murashita Hidekazu, Tabuchi Keiji, Hoshino Tomofumi, Tsuji Shigeki, Hara Akira The effects of tempol, 3-aminobenzamide and nitric oxide synthase inhibitors on acoustic injury of the mouse cochlea. HEARING RESEARCH. 2006;214(1-2):1-6.

8232. Helfert Susanne, Warschburger Petra The face of appearance-related social pressure: gender, age and body mass variations in peer and parental pressure during adolescence. CHILD AND ADOLESCENT PSYCHIATRY AND MENTAL HEALTH. 2013;7():.

8233. KENDLER KS, NEALE MC, KESSLER RC, HEATH AC, EAVES LJ CLINICAL CHARACTERISTICS OF FAMILIAL GENERALIZED ANXIETY DISORDER. ANXIETY. 1994;1(4):186-191.

8234. Schootbrugge Bas, Bachan Aviv, Suan Guillaume, Richoz Sylvain, Payne Jonathan MICROBES, MUD AND METHANE: CAUSE AND CONSEQUENCE OF RECURRENT EARLY JURASSIC ANOXIA FOLLOWING THE END-TRIASSIC MASS EXTINCTION. PALAEONTOLOGY. 2013;56(4):685-709.

8235. Schulte Peter, Scheibner Christian, Speijer Robert Fluvial discharge and sea-level changes controlling black shale deposition during the Paleocene-Eocene Thermal Maximum in the Dababiya Quarry section, Egypt. CHEMICAL GEOLOGY. 2011;285(1-4):167-183.

8236. Telgkamp P, Cao YQQ, Basbaum AI, Ramirez JM Long-term deprivation of substance P in PPT-A mutant mice alters the anoxic response of the isolated respiratory network. JOURNAL OF NEUROPHYSIOLOGY. 2002;88(1):206-213.

8237. Troisi A, Massaroni P, Cuzzolaro M Early separation anxiety and adult attachment style in women with eating disorders. BRITISH JOURNAL OF CLINICAL PSYCHOLOGY. 2005;44(1):89-97.

8238. AlMarzooqi Mazna, Nagy M. Childhood Obesity Intervention Programs: A Systematic Review. LIFE SCIENCE JOURNAL-ACTA ZHENGZHOU UNIVERSITY OVERSEAS EDITION. 2011;8(4):45-60.

8239. Zhang Liyu, Chen Daizhao, Huang Taiyu, Yu Hao, Zhou Xiqiang, Wang Jianguo An abrupt oceanic change and frequent climate fluctuations across the Frasnian-Famennian transition of Late Devonian: Constraints from conodont Sr isotope. GEOLOGICAL JOURNAL. 2020;55(6):4479-4492.

8240. Gutierrez Dimitri, Enriquez E., Purca S., Quipuzcoa L., Marquina R., Flores G., Graco M. Oxygenation episodes on the continental shelf of central Peru: Remote forcing and benthic ecosystem response. PROGRESS IN OCEANOGRAPHY. 2008;79(2-4, SI):177-189.

8241. McKean T, Scherzer A, Park H Hypoxia and ischaemia in buffer-perfused toad hearts. JOURNAL OF EXPERIMENTAL BIOLOGY. 1997;200(19):2575-2581.

8242. Vasiliu Octavian The current state of research for psychobiotics use in the management of psychiatric disorders-A systematic literature review. FRONTIERS IN PSYCHIATRY. 2023;14():.

8243. Carmeille Mehdi, Bourillot Raphael, Pellenard Pierre, Dupias Victor, Schnyder Johann, Riquier Laurent, Mathieu Olivier, Brunet Marie-Francoise, Enay Raymond, Grossi Vincent, Gaborieau Cecile, Razin Philippe, Visscher Pieter Formation of microbial organic carbonates during the Late Jurassic from the Northern Tethys (Amu Darya Basin, Uzbekistan): Implications for Jurassic anoxic events. GLOBAL AND PLANETARY CHANGE. 2020;186():.

8244. Piazza Veronica, Ullmann Clemens, Aberhan Martin Ocean warming affected faunal dynamics of benthic invertebrate assemblages across the Toarcian Oceanic Anoxic Event in the Iberian Basin (Spain). PLOS ONE. 2020;15(12):.

8245. AbdelHamid KM, Tymianski M Mechanisms and effects of intracellular calcium buffering on neuronal survival in organotypic hippocampal cultures exposed to anoxia/aglycemia or to excitotoxins. JOURNAL OF NEUROSCIENCE. 1997;17(10):3538-3553.

8246. Lloyd E., Sallis Hannah, Verplanken Bas, Haase Anne, Munafo Marcus Understanding the nature of association between anxiety phenotypes and anorexia nervosa: a triangulation

approach. BMC PSYCHIATRY. 2020;20(1):.

8247. Popat Shreeya, Winslade William While You Were Sleepwalking: Science and Neurobiology of Sleep Disorders \& the Enigma of Legal Responsibility of Violence During Parasomnia. NEUROETHICS. 2015;8(2):203-214.

8248. Kokura S, Wolf RE, Yoshikawa T, Granger DN, Aw TY Postanoxic T lymphocyte-endothelial cell interactions induce tumor necrosis factor-alpha production and neutrophil adhesion - Role of very late antigen-4/vascular cell adhesion molecule-1. CIRCULATION RESEARCH. 2000;86(12):1237-1244.

8249. Kreutzer U, Jue T H-1-NMR signal of Arenicola marina myoglobin in vivo as an index of tissue oxygenation. EUROPEAN JOURNAL OF BIOCHEMISTRY. 1996;235(3):622-628.

8250. Schreiber Anja, Kesztyues Dorothea, Wirt Tamara, Erkelenz Nanette, Kobel Susanne, Steinacker Juergen, Res Komm Why do mothers encourage their children to control their weight? A cross-sectional study of possible contributing factors. BMC PUBLIC HEALTH. 2014;14():.

8251. Braun KR, Davidson KM, Henry M, Nielsen HC Severe pulmonary hemorrhage in the premature newborn infant: Analysis of presurfactant and surfactant eras. BIOLOGY OF THE NEONATE. 1999;75(1):18-30.

8252. MEERSON FZ, KOPYLOV YN, GOLUBEVA LY THE ROLE OF ITP-DAG REGULATORY CASCADE IN THE MECHANISM OF CARDIOPROTECTIVE EFFECT OF ADAPTATION TO STRESS. CANADIAN JOURNAL OF CARDIOLOGY. 1994;10(1):137-147.

8253. Perez Marisol, Van Diest Ashley, Smith Haylie, Sladek Michael Body Dissatisfaction and Its Correlates in 5-to 7-Year-Old Girls: A Social Learning Experiment. JOURNAL OF CLINICAL CHILD AND ADOLESCENT PSYCHOLOGY. 2018;47(5):757-769.

8254. Sun Y., Wignall P., Joachimski M., Bond D., Grasby S., Lai X., Wang L., Zhang Z., Sun S. Climate warming, euxinia and carbon isotope perturbations during the Carnian (Triassic) Crisis in South China. EARTH AND PLANETARY SCIENCE LETTERS. 2016;444():88-100.

8255. Cox Anne, Ullrich-French Sarah, Cook-Cottone Catherine, Tylka Tracy, Neumark-Sztainer Dianne Examining the effects of mindfulness-based yoga instruction on positive

embodiment and affective responses. EATING DISORDERS. 2020;28(4, SI):458-475.

8256. Huang Jiyi, Liu Zhantu, Xu Ping, Zhang Zeyu, Yin Dong, Liu Jichun, He Huan, He Ming Capsaicin prevents mitochondrial damage, protects cardiomyocytes subjected to anoxia/reoxygenation injury mediated by 14-3-3 eta/Bcl-2. EUROPEAN JOURNAL OF PHARMACOLOGY. 2018;819():43-50.

8257. McClelland Jessica, Hodsoll John, Brown Amy, Lang Katie, Boysen Elena, Flynn Michaela, Mountford Victoria, Glennon Danielle, Schmidt Ulrike A pilot evaluation of a novel First Episode and Rapid Early Intervention service for Eating Disorders (FREED). EUROPEAN EATING DISORDERS REVIEW. 2018;26(2):129-140.

8258. Hendrick V, Altshuler LL, Burt VK Course of psychiatric disorders across the menstrual cycle. HARVARD REVIEW OF PSYCHIATRY. 1996;4(4):200-207.

8259. Semenov DG, Samoilov MO, Lazarewicz JW Calcium transients in the model of rapidly induced anoxic tolerance in rat cortical slices: Involvement of NMDA receptors. NEUROSIGNALS. 2002;11(6):329-335.

8260. Landt Margarita, Furth Eric, Beijsterveldt Catharina, Bartels Meike, Willemsen Gonneke, Geus Eco, Ligthart Lannie, Boomsma Dorret Prevalence of dieting and fear of weight gain across ages: a community sample from adolescents to the elderly. INTERNATIONAL JOURNAL OF PUBLIC HEALTH. 2017;62(8):911-919.

8261. Schein Allison, Courtenay Simon, Kidd Karen, Campbell K., Heuvel Michael Food web structure within an estuary of the southern Gulf of St. Lawrence undergoing eutrophication. CANADIAN JOURNAL OF FISHERIES AND AQUATIC SCIENCES. 2013;70(12):1805-1812.

8262. Zhang Jianguo, Jiang Zaixing, Wang Siqi, Kong Xiangxin Phytoplankton as main organism in the Eocene organic-rich turbidites of Jiyang Depression, China: Implication for organic matter accumulation mechanism. ENERGY SOURCES PART A-RECOVERY UTILIZATION AND ENVIRONMENTAL EFFECTS. 2023;45(3):7835-7845.

8263. Ladilov Y, Maxeiner H, Wolf C, Schafer C, Meuter K, Piper HM Role of protein phosphatases in hypoxic preconditioning. AMERICAN JOURNAL OF PHYSIOLOGY-HEART AND CIRCULATORY PHYSIOLOGY. 2002;283(3):H1092-H1098.

8264. Hetzelt Katalin, Kraus Cornelia, Kusnik Stefan, Thiel Christian, Uebe Steffen, Ekici Arif, Trollmann Regina, Reis Andre, Zweier Christiane A case of severe autosomal recessive spinocerebellar ataxia type 18 with a novel nonsense variant in GRID2. EUROPEAN JOURNAL OF MEDICAL GENETICS. 2020;63(9):.

8265. Maerz C., Schnetger B., Brumsack H. Nutrient leakage from the North Pacific to the Bering Sea (IODP Site U1341) following the onset of Northern Hemispheric Glaciation?. PALEOCEANOGRAPHY. 2013;28(1):.

8266. Gonzalez-Macias Laura, Caballero-Romo Alejandro, Garcia-Anaya Maria Group family psychotherapy during relapse. Case report of a novel intervention for severe and enduring anorexia nervosa. SALUD MENTAL. 2021;44(1):31-37.

8267. Flamarique I, Vidal B., Plana M., Andres-Perpina S., Garriz M., Sanchez P., Pajuelo C., Mont L., Castro-Fornieles J. Long-term cardiac assessment in a sample of adolescent-onset anorexia nervosa. JOURNAL OF EATING DISORDERS. 2022;10(1):.

8268. Cook Brian, Hausenblas Heather, Crosby Ross, Cao Li, Wonderlich Stephen Exercise dependence as a mediator of the exercise and eating disorders relationship: A pilot study. EATING BEHAVIORS. 2015;16():9-12.

8269. Gittman Rachel, Keller Danielle Fiddler crabs facilitate Spartina alterniflora growth, mitigating periwinkle overgrazing of marsh habitat. ECOLOGY. 2013;94(12):2709-2718.

8270. Li Wen-Juan, Nie Shao-Ping, Chen Yi, Xie Ming-Yong, He Ming, Yu Qiang, Yan Yan Ganoderma atrum Polysaccharide Protects Cardiomyocytes Against Anoxia/Reoxygenation-Induced Oxidative Stress by Mitochondrial Pathway. JOURNAL OF CELLULAR BIOCHEMISTRY. 2010;110(1):191-200.

8271. Tasneem Ayesha Neurobiological Underpinnings of Anorexia Nervosa. UNIVERSITY OF TORONTO MEDICAL JOURNAL. 2018;95(2):41-44.

8272. Sarro S. Transient psychosis in anorexia nervosa: Review and case report. EATING AND WEIGHT DISORDERS-STUDIES ON ANOREXIA BULIMIA AND OBESITY. 2009;14(2-3):E139-E143.

8273. Athanasian Christian, Lazarevic Bojan, Kriegel Elana, Milanaik Ruth Alternative diets among adolescents: facts or fads?. CURRENT OPINION IN PEDIATRICS. 2021;33(2):252-259.

8274. Young John Anorexia nervosa and estrogen: Current status of the hypothesis. NEUROSCIENCE AND BIOBEHAVIORAL REVIEWS. 2010;34(8):1195-1200.

8275. Askenazy FL, Sorci K, Benoit M, Lestideau K, Myquel M, Lecrubier Y Anxiety and impulsivity levels identify relevant subtypes in adolescents with at-risk behavior. JOURNAL OF AFFECTIVE DISORDERS. 2003;74(3):219-227.

8276. Baker Sarah, Hesselbo Stephen, Lenton Timothy, Duarte Luis, Belcher Claire Charcoal evidence that rising atmospheric oxygen terminated Early Jurassic ocean anoxia. NATURE COMMUNICATIONS. 2017;8():.

8277. Campbell Amy, Aulisio Mark The stigma of ``mental{''} illness: End stage anorexia and treatment refusal. INTERNATIONAL JOURNAL OF EATING DISORDERS. 2012;45(5):627-634.

8278. Doley Joanna, Rodgers Rachel, Paxton Susan, McLean Sian Effectiveness of recruitment strategies for a social media literacy E-intervention for young adults with body dissatisfaction: cost, time, diversity, and completion. EATING DISORDERS. 2022;30(5):515-539.

8279. Sternheim L., Harrison A. The acceptability, feasibility and possible benefits of a group-based intervention targeting intolerance of uncertainty in adolescent inpatients with anorexia nervosa. COGENT PSYCHOLOGY. 2018;5(1):.

8280. Marquez Sara, Vega Ricardo EXERCISE ADDICTION: AN EMERGENT BEHAVIORAL DISORDER. NUTRICION HOSPITALARIA. 2015;31(6):2384-2391.

8281. Jiang Haishui, Joachimski Michael, Wignall Paul, Zhang Muhui, Lai Xulong A delayed end-Permian extinction in deep-water locations and its relationship to temperature trends (Bianyang, Guizhou Province, South China). PALAEOGEOGRAPHY PALAEOCLIMATOLOGY PALAEOECOLOGY. 2015;440():690-695.

8282. Barash M. Mass extinction of the marine biota at the Ordovician-Silurian transition due to environmental changes. OCEANOLOGY. 2014;54(6):780-787.

8283. Andreeva NA, Stel'mashuk EV, Isaev NK, Ostrovskaya RU, Gudasheva TA, Viktorov IV Neuroprotective properties of nootropic dipeptide GVS-111 in in vitro oxygen-glucose deprivation, glutamate toxicity and oxidative stress. BULLETIN OF EXPERIMENTAL BIOLOGY AND MEDICINE. 2000;130(10):969-972.

8284. Danthinne Elisa, Giorgianni Francesca, Rodgers Rachel Labels to prevent the detrimental effects of media on body image: A systematic review and meta-analysis. INTERNATIONAL JOURNAL OF EATING DISORDERS. 2020;53(5):377-391.

8285. Schroder Arjan, Diepen Rosanne, Mazaheri Ali, Petropoulos-Petalas Diamantis, Amesti Vicente, Vulink Nienke, Denys Damiaan Diminished Ni auditory evoked poten als to oddball stimuli in misophonia patients. FRONTIERS IN BEHAVIORAL NEUROSCIENCE. 2014;8():.

8286. Xia Yuntian, Xiao Jingyuan, Yu Yongfu, Tseng Wan-Ling, Lebowitz Eli, DeWan Andrew, Pedersen Lars, Olsen Jorn, Li Jiong, Liew Zeyan Rates of Neuropsychiatric Disorders and Gestational Age at Birth in a Danish Population COMMENT. OBSTETRICAL \& GYNECOLOGICAL SURVEY. 2021;76(12):719-721.

8287. Hedayatnia Mahshad, Asadi Zahra, Zare-Feyzabadi Reza, Yaghooti-Khorasani Mahdiyeh, Ghazizadeh Hamideh, Ghaffarian-Zirak Roshanak, Nosrati-Tirkani Abolfazl, Mohammadi-Bajgiran Maryam, Rohban Mohadese, Sadabadi Fatemeh, Rahimi Hamid-Reza, Ghalandari Marzieh, Ghaffari Mohammad-Seddigh, Yousefi Asa, Pouresmaeili Elnaz, Besharatlou Mohammad-Reza, Moohebati Mohsen, Ferns Gordon, Esmaily Habibollah, Ghayour-Mobarhan Majid Dyslipidemia and cardiovascular disease risk among the MASHAD study population. LIPIDS IN HEALTH AND DISEASE. 2020;19(1):.

8288. Stice E, Agras WS, Hammer LD Risk factors for the emergence of childhood eating disturbances: A five-year prospective study. INTERNATIONAL JOURNAL OF EATING DISORDERS. 1999;25(4):375-387.

8289. Tandoh Akua, Aryeetey Richmond, Agyemang Charles, Holdsworth Michelle, Asiki Gershim, Zotor Francis, Mensah Kobby, Laar Matilda, Laryea Dennis, Sellen Daniel, Vandevijvere Stefanie, Laar Amos The Africa Food Environment Research Network (FERN): from concept to practice. GLOBAL HEALTH PROMOTION. 2023;30(2):5-10.

8290. DeFronzo Ralph, Ferrannini Ele, Groop Leif, Henry Robert, Herman William, Holst Jens, Hu Frank, Kahn C., Raz Itamar, Shulman Gerald, Simonson Donald, Testa Marcia, Weiss Ram Type 2 diabetes mellitus. NATURE REVIEWS DISEASE PRIMERS. 2015;1():.

8291. Nawrocki W., Bailleul B., Cardol P., Rappaport F., Wollman F., Joliot P. Maximal cyclic electron flow rate is independent of PGRL1 in Chlamydomonas. BIOCHIMICA ET BIOPHYSICA ACTA-BIOENERGETICS. 2019;1860(5):425-432.

8292. Saito Ayaka, Kinoshita Chihiro, Kawai Megumi, Fukuoka Takuya, Sato Katsufumi, Sakamoto Kentaro Effects of a parasympathetic blocker on the heart rate of loggerhead sea turtles during voluntary diving. JOURNAL OF EXPERIMENTAL BIOLOGY. 2022;225(9):.

8293. Zimmerman AR, Canuel EA A geochemical record of eutrophication and anoxia in Chesapeake Bay sediments: anthropogenic influence on organic matter composition. MARINE CHEMISTRY. 2000;69(1-2):117-137.

8294. Yavin E, Billia DM Apoptotic death in cerebral hemisphere cells is density dependent and modulated by transient oxygen and glucose deprivation. JOURNAL OF NEUROSCIENCE RESEARCH. 1997;47(5):471-478.
[truncated: 553,202 more chars]
