# Supplementary material for: A Bibliometric Analysis of Scientific Publications on Eating Disorder Prevention in the Past Three Decades
Source: Nutrients. 2024 Apr 10;16(8):1111. doi: 10.3390/nu16081111 (PMC11054308; doi:10.3390/nu16081111)
Supplement: Supplementary file 1 [file nutrients-16-01111-s001.zip › File S3.pdf]

Supplementary File S3

Sources

Among the 10,546 automatically generated papers, 6.4% were published in the "International Journal of Eating Disorders," which also ranks as the top source for the manually selected 374 papers. In the top 10 journals from the automatic set, there are five different journals compared to the manual selection, which include "Nutrients," "Appetite," "International Journal of Environmental Research and Public Health," "PLOS ONE," and "BMC Public Health."

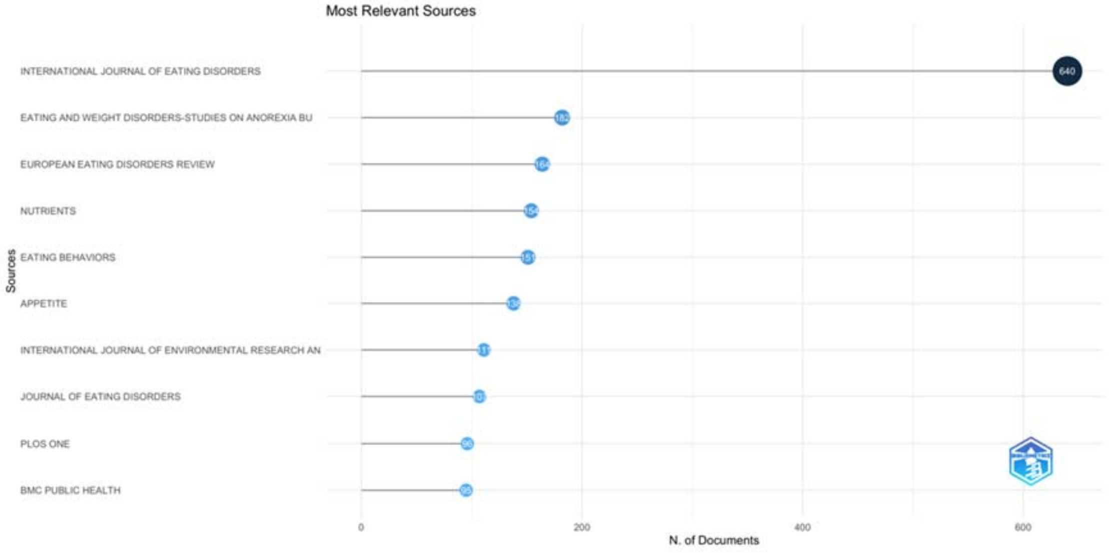

Supplementary Figure S1. Peer-reviewed journals, in their order of publishing research on prevention of ED (*n* = 10,546).

The graph shows a cumulative representation of publications over time from various journals related to eating disorders. There is a clear and steady increase in the number of publications across all journals from 1993 to 2023. Notably, the "International Journal of Eating Disorders" displays a significant and dominant upward trend, indicating a higher volume of publications over the years compared to the other journals. Other journals such as "European Eating Disorders Review," "Eating Behaviors," and "Eating and Weight Disorders-Studies on Anorexia Bulimia and Obesity" also show an upward trajectory, but their volume of publications is much lower than that of the "International Journal of Eating Disorders." "Nutrients" is another journal included on the graph, and while it shows growth, it does so at a pace that is less steep than the "International Journal of Eating Disorders."

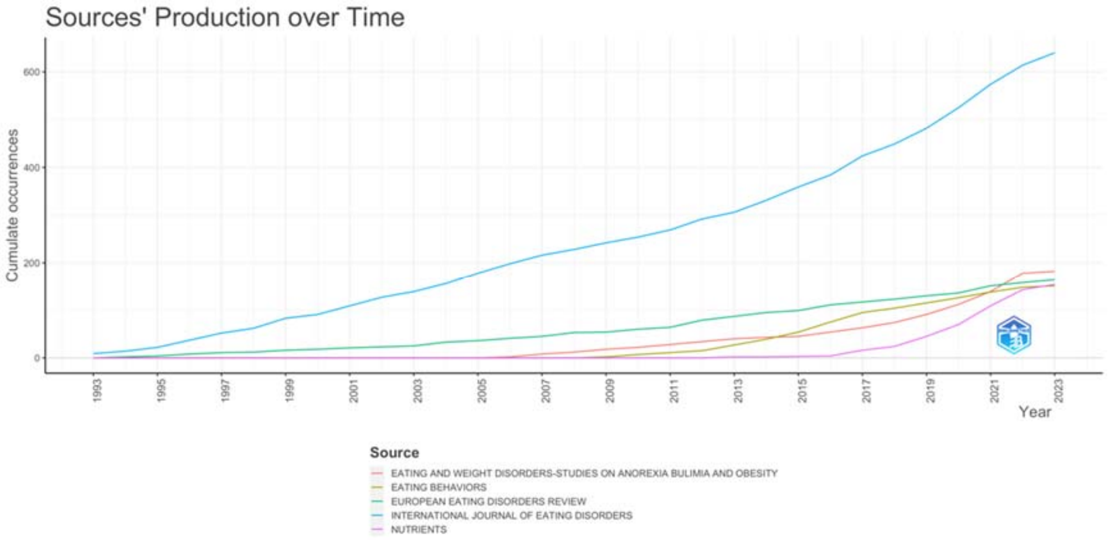

Supplementary Figure S2. The publications trend in journals over time (n = 10,546).

Authors

Eric Stice is highlighted as the most prolific author in the automated list, while Cynthia M. Bulik ranks second. However, Bulik's main contributions focus on the Eating Disorders Genetics Initiative and treatments, areas that fall outside our selection criteria.

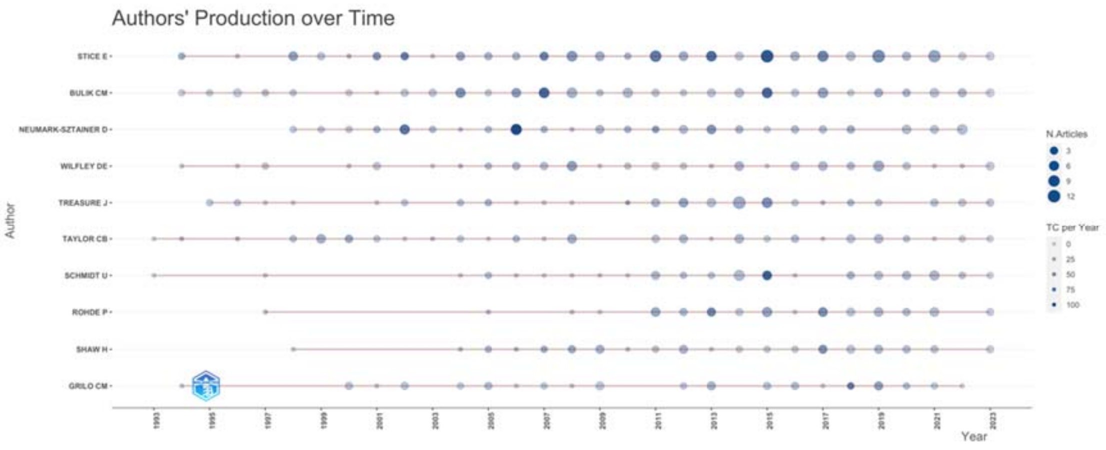

Supplementary Figure S3. The authors' production over time.

Collaboration Networks

Supplementary Figure S4 displays the collaborative networks and publication patterns among authors from the 10,546 papers in the automatic list. Four primary networks emerge: one centered on Eric Stice, another around Cynthia M. Bulik, a third around

Denise E. Wilfley, and a fourth cluster without a distinct central figure.

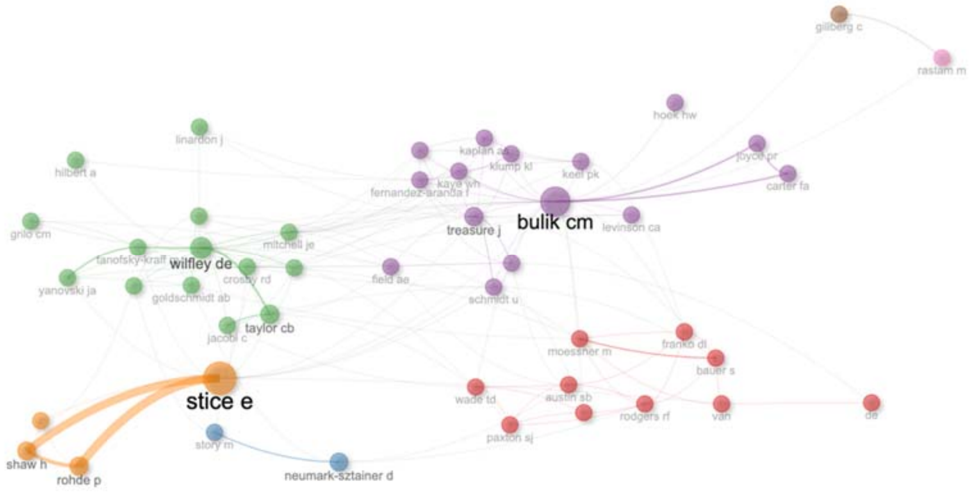

Supplementary Figure S4. The connections and publication orders among authors (*n* = 10,546).
